# Global terrestrial carbon fluxes of 1999–2019 estimated by upscaling eddy covariance data with a random forest

**DOI:** 10.1038/s41597-020-00653-5

**Published:** 2020-09-24

**Authors:** Jiye Zeng, Tsuneo Matsunaga, Zheng-Hong Tan, Nobuko Saigusa, Tomoko Shirai, Yanhong Tang, Shushi Peng, Yoko Fukuda

**Affiliations:** 1 National Institute for Environmental Studies, Tsukuba, Ibaraki 305-8506 Japan; 2 Department of Environmental Science, Hainan University, Haikou, 570228 China; 3 Department of Ecology, Peking University, Beijing, China; 4 College of Urban and Environmental Sciences, Peking University, Beijing, China

**Keywords:** Carbon cycle, Carbon cycle

## Abstract

The terrestrial biosphere is a key player in slowing the accumulation of carbon dioxide in the atmosphere. While quantification of carbon fluxes at global land scale is important for mitigation policy related to climate and carbon, measurements are only available at sites scarcely distributed in the world. This leads to using various methods to upscale site measurements to the whole terrestrial biosphere. This article reports a product obtained by using a Random Forest to upscale terrestrial net ecosystem exchange, gross primary production, and ecosystem respiration from FLUXNET 2015. Our product covers land from −60°S to 80°N with a spatial resolution of 0.1° × 0.1° every 10 days during the period 1999–2019. It was compared with four existing products. A distinguishable feature of our method is using three derived variables of leaf area index to represent plant functional type (PFT) so that measurements from different PFTs can be mixed better by the model. This product can be valuable for the carbon-cycle community to validate terrestrial biosphere models and cross check datasets.

## Abstract (summary)

**Table:** 

Measurement(s)	ecosystem-wide respiration • gross primary production • net ecosystem exchange • carbon flux • carbon dioxide
Technology Type(s)	Random forest
Factor Type(s)	year of data collection
Sample Characteristic - Environment	terrestrial biome • climate system
Sample Characteristic - Location	Earth (planet)

Machine-accessible metadata file describing the reported data: 10.6084/m9.figshare.12932882

## Background & Summary

Terrestrial ecosystems are a major forcing regulating climate by exchanges of energy and carbon with the atmosphere^1,2^. As a large and persistent carbon sink^3,4^, they have served to slow the accumulation of anthropogenic CO_2_ in the atmosphere^5^. Over the past 10 years (2008–2017), the terrestrial carbon sink was estimated to have removed about 30% of fossil CO_2_ emissions^6^. As direct estimate of the sink using observations is difficult due to high variability of the photosynthesis and biases in flux measurements^7^, the Global Carbon Project estimated the sink in early years as the residue of the fossil CO_2_ emissions minus CO_2_ accumulated in the atmosphere and removed by the oceans^8^, and recently as the assemble of process and inverse models^6^. Nevertheless, flux measurements by the eddy covariance technique^9^ have served as a benchmark for model validation^10–13^ and provided fundamental knowledge on global carbon cycling^14–16^.

Data-driven machine learning (DDML) methods have been used to combine flux tower measurements, remote sensing observations, and climate model data to upscale forest fluxes^17–28^. The key characteristic that distinguishes a DDML method from others is that the functional relationships are not assumed, but rather learned from patterns in the measurements. A DDML model is more objective than other types of models in that it does not subjectively impose conditions on valid ranges of model parameters in the optimization process. In the early 2000s, Papale and Valentini^17^ used observations of the EUROFLUX project to train a neural network simulator to estimate carbon fluxes of European forests at the continental scale. The same method was used later to examine the effect of spatial sampling on the extrapolation of the neural network^18^. Later, other DDML methods were introduced, including support vector machine^19–21^, model tree assemble^22–25^, and random forest^26–28^. Results of DDML have been used to evaluate terrestrial biosphere models^9–11^ and empirical data based on remote sensing^12^.

This study presents a data-driven global gridded product for terrestrial net ecosystem exchange, gross primary production, and ecosystem respiration obtained by using a Random Forest method to upscale FLUXNET-2015 to the land from −60°S to 80°N in the period 1999–2019. The spatial and temporal resolutions are in 0.1° × 0.1° and 10 days respectively. Although similar data-driven products using FLUXNET-2015 have been reported^27–29^, datasets obtained by a different approach are valuable considering uncertainty elements of data-driven methods, which include extrapolation to conditions unrepresented in the training data^17,18^, selection of predictor variables^26,30^, and product-specific biases of predictor variables^31^. The product can also be valuable for diagnosing large disparities existing among different types of models^32^.

## Methods

### Model setup

**Fig. 1:**
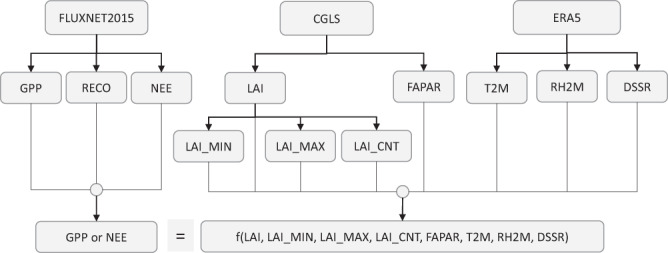
Datasets and workflow. GPP, RECO, and NEE are assumed to be the nonlinear function of LAI, LAI_MIN, LAI_MAX, LAI_CNT, FAPAR, T2M, RH2M, and DSSR. CGLS: Copernicus Global Land Service; ERA5: The fifth-generation ECMWF atmospheric reanalysis of the global climate; GPP: Gross primary production; RECO: Ecosystem respiration; NEE: Net ecosystem exchange; LAI: 10-day mean leaf area index; LAI_MIN: Minimum of LAI in a year; LAI_MAX: Maximum of LAI in a year; LAI_CNT: Count of LAIs in a year that are larger than the mean of LAI_MIN and LAI_MAX; FAPAR: Fraction of absorbed photosynthetically active radiation; T2M: Air temperature at 2 m above the surface; RH2M: Relative humidity at 2 m above the surface; DSSR: Downward shortwave solar radiation on the surface.

As illustrated in Fig. 1, the target variables of gross primary production (GPP), ecosystem respiration (RECO), and net ecosystem exchange (NEE) were modelled as the nonlinear function of leaf area index (LAI), fraction of absorbed photosynthetically active radiation (FAPAR), downward shortwave solar radiation on the surface (DSSR), air temperature (T2M), and relative humidity (RH2M) at 2 meters above the surface, and three variables derived from LAI to indicate plant functional type (PFT): the minimum (LAI_MIN) and maximum (LAI_MAX) of LAI in a year, and the number of LAI larger than the mean of LAI_MIN and LAI_MAX (LAI_COUNT) in a year. They directly reflect the spatial distributions of seasonality and leaf biomass. Replacing PFT by the three derived LAI variables is a new idea of this study.

The freeware Ranger^33^ that implements the regression algorithm of random forest^34^ (RF) was used to model the relationships between carbon fluxes and independent variables listed in Fig. 1. A RF includes many binary decision trees^35^, which are grown independently using a two-stage randomization procedure. The first step is to assign to each tree a subset of the training data randomly sampled with repetition; then each tree is recursively split into binary nodes until the number of data points in the terminal nodes is not larger than a specified number. In each split, the RF randomly selects a subset of predictor variables and searches them for splitting points that maximize node impurity^35^, which is equivalent to minimize the weighted variances of the target between parent and child nodes^36^. In making a prediction, a new set of predictors is examined with each tree in a trained RF, passing them through branches of nodes according to the splitting points until the journey ends up in a terminal node, and the mean of the target variable in the node is taken as an estimate. Then the mean estimate of all terminal nodes is used as the prediction.

### FLUXNET data

We extracted GPP, RECO, and NEE from the tier-1 sites of FLUXNET 2015^9^, specifically daily GPP from the GPP_NT_VUT_REF column, RECO from RECO_NT_VUT_REF, and NEE from NEE_VUT_REF. Daily NEE is the sum of hourly measurements of ecosystem exchange; RECO is the ecosystem respiration estimated by extending night-time hourly measurements (when photosynthesis stopped) to the whole day^37^, and GPP was calculated from NEE and RECO. The quality flags of these variables were used to exclude points with less than 90% of the measured and good-quality gap-fill data. We found, however, that this quality control measure was not sufficient to ensure the consistency between GPP-RECO and -NEE. Tramontana *et al*.^27^ used a robust regression method to select data, which would effectively filter out suspicious data points. We simply excluded those points which had an absolute difference between GPP-RECO and NEE larger than 0.1 gC m^−2^ d^−1^. About 7.5% of the data fell into this category.

The length of data record varied largely from site to site. To balance the presentation of sites in the RF, we used only the most recent data for up to three years. The daily fluxes were binned into 10-day means corresponding to the periods of remote sensing data, i.e., the first 10-day mean of a month included data from the first to the 10th days, the second 10-day mean from the 11th to the 20th days, and the third 10-day mean from the remaining days. The binned RECO and NEE were used to recalculate GPP. This process resulted in a total number of 16,939 records from 204 flux tower sites.

### Remote sensing data

The remote sensing data were derived from the Copernicus Global Land Service. LAI and FAPAR^38^ were available in 1 km spatial resolution for every 10 days from 1999 to present (https://land.copernicus.eu/global/themes/vegetation). We evaluated FAPAR by analysing the correlation between GPP and FAPAR*DSSR. The relationship was the basis of many light-use efficiency models^12,39–41^ for GPP. The results show that between GPP and FAPAR*DSSR, 62% of the sites have a R^2^ larger than 0.5 and 27% have a R^2^ larger than 0.7. Plots of LAI with flux observation for all sites show good correlations in terms of amplitude and seasonal pattern. These indicate that the extracted LAI and FAPAR are good predictors.

### Climate data

The predictor variables T2M, RH2M, and DSSR came from the fifth-generation ECMWF atmospheric reanalysis of the global climate (ERA5^42^). The spatial resolution of the hourly data on single levels is 0.25°x0.25°. Analysis and forecast data in every three hours were obtained for T2M and RH2M. Their daily means were calculated first and then used to calculate the 10-day means. Hourly accumulated DSSR data were used to calculate the daily accumulated DSSR, which was then used to calculate the 10-day mean. T2M and DSSR were checked by comparing them with the air temperature (TA) and photosynthetic photon flux density (PPFD) of FLUEXNET 2015. The R^2^ between T2M and TA is larger than 0.7 for 98% of the sites, and the percentage for R^2^ > 0.7 between DSSR and PPFD is 91%.

## Data Records

The product is available at 10.17595/20200227.001^43^. Data files in NetCDF format are named as VARIABLE.YEAR.ver.NUMBER.nc in which VAIARBLE can be GPP or NEE or RECO, YEAR is the year of fluxes, and the version NUMBER is usually the year the dataset was created or updated. The meta-information inside describes the method, software, and data sources.

**Fig. 2:**
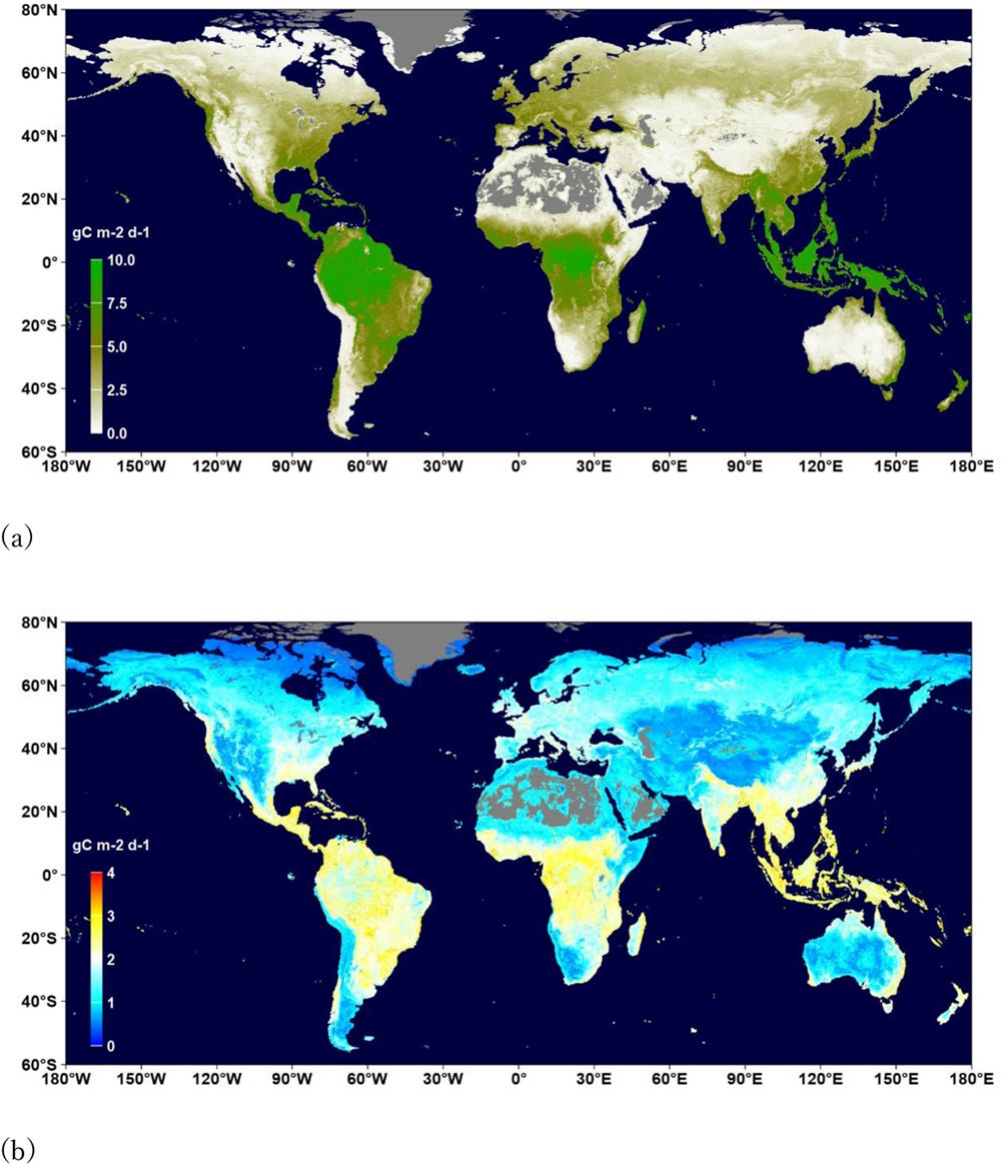
Distribution of the annual mean (**a**) and uncertainty (**b**) of GPP (gC m^−2^ d^−1^) in 2014. Uncertainty is the standard deviation of flux values in the terminal nodes of 500 trees used to make the prediction.

**Fig. 3:**
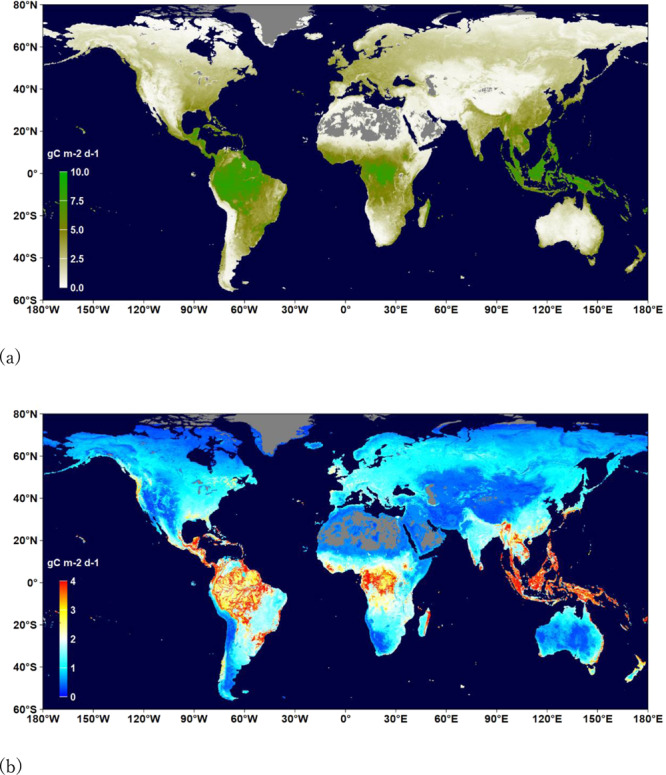
Distribution of the annual mean (**a**) and uncertainty (**b**) of RECO (gC m^−2^ d^−1^) in 2014. Uncertainty is the standard deviation of flux values in the terminal nodes of 500 trees used to make a prediction.

**Fig. 4:**
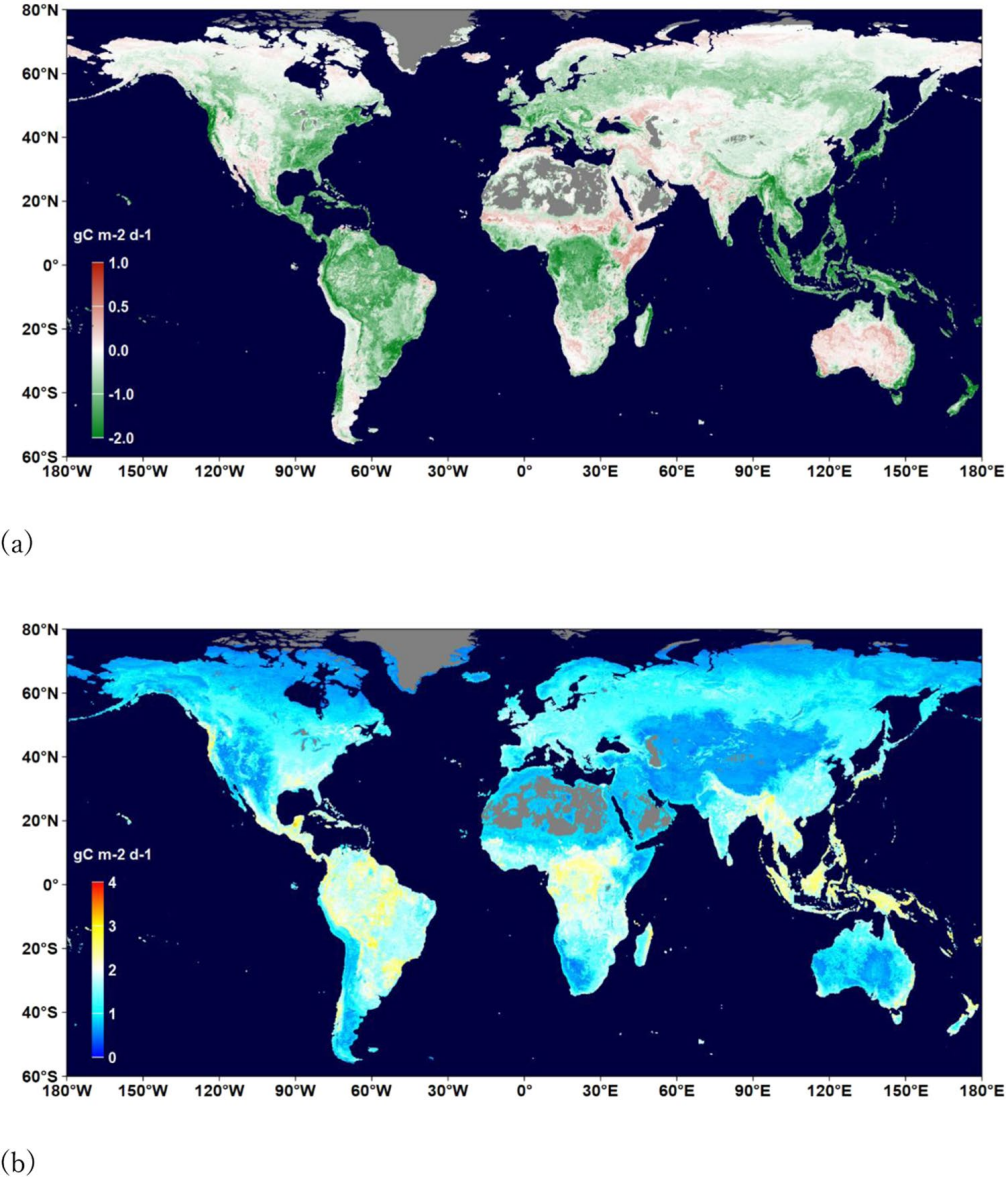
Distribution of the annual mean (**a**) and uncertainty (**b**) of NEE (gC m^−2^ d^−1^) in 2014. Uncertainty is the standard deviation of flux values in the terminal nodes of 500 trees used to make the prediction.

**Fig. 5:**
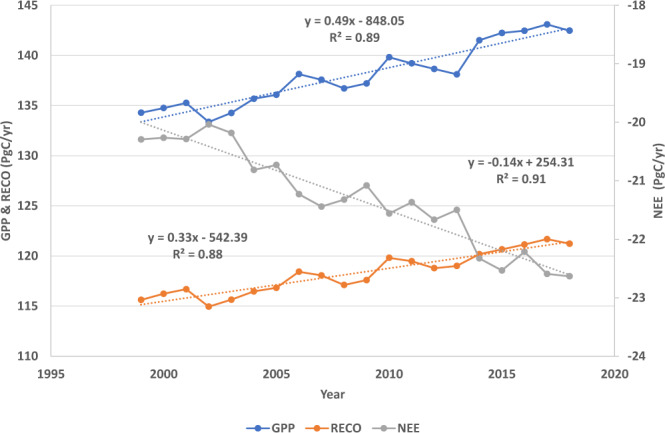
Trend of global annual GPP, RECO, and NEE (PgC yr^−1^). An annual mean is the global integral of fluxes in 0.1° × 0.1° grids.

Figures 2–4 show the distributions of the annual means and mean uncertainties of GPP, RECO, and NEE in 2014, respectively. An uncertainty is the standard deviation of flux values in the terminal nodes of 500 trees used to make a prediction. The spatial patterns are similar to other existing products^44,45^. The annual GPP increased from 134.3 PgC yr^−1^ in 1999 to 142.2 PgC yr^−1^ in 2019 with an increasing rate of 0.49 PgC yr^−1^ (Fig. 5). The estimate is slightly higher than those by Copernicus^41^ and Jung *et al*.^29^ (refer to as Jung-2019 hereafter), but lower than a recent estimate^46^. The RECO was estimated to be 115.6 PgC yr^−1^ in 1999 and 121.3 PgC yr^−1^ in 2019, which are higher than that of Jung-2019. The trend of RECO was 0.33 PgC yr^−1^. However, the estimated NEE in this study, which was −20.3 PgC yr^−1^ in 1999 and −22.8 PgC yr^−1^ in 2019, is lower than Jung-2019’s estimate. The trend of NEE is 0.14 PgC yr^−1^, which is smaller than some recent estimates^45,47^. Our GPP and RECO trends are larger than those of Li *et al*.^48^.

There is a 1.6 ± 0.3 PgC yr^−1^discrepancy between GPP-RECO and -NEE resulting from the node splitting scheme of RF. While GPP was recalculated as RECO-NEE and the values of predictor variables are the same in the training datasets, the trained tree structures can be significantly different from each other as the node splitting rule is based on the variances of the target in parent and child nodes. Therefore, the predicted GPP, RECO, and NEE for a new set of predictors would come from different records of the training datasets. Although the discrepancy is only about 1% of the annual GPP, it is large regarding the global terrestrial carbon sink^6^.

Our dataset is featured by using derived LAI variables (LAI_MAX, LAI_MIN, LAI_COUNT) to present PFT. While different PFTs are independent and have discrete spatial patterns (Figs. S1–S11 in Supplementary File 1, data extracted from the MCD12C1 MODIS/Terra + Aqua dataset^49^), derived LAI variables are related to leaf biomass and seasonality and show rather continuous spatial transitions (Fig. S12 in Supplementary File 1). Unbalanced sampling becomes worse when measurements are grouped into different PFTs. We therefore expect that using the derived LAI variables should improve the spatial interpolation of the random forest as measurements of different PFTs can be mixed more logically. However, evaluating the advantages or disadvantages requires a dedicated inter-comparison study, which is beyond the scope of this study.

## Technical Validation

We tested the performance of the RF with different numbers of trees (250–1000) and target data points (3–10) in the terminal nodes by a 10-fold cross-validation. The experiments indicated that raising or lowing these numbers did not change the goodness of fitting for GPP, RECO, and NEE. Therefore, we adopted the default configuration of Ranger, which has 500 trees and 5 target data points in the terminal nodes. With this configuration, the cross-validation obtained an R^2^ of 0.86 ± 0.01 between the modelled GPP and observations. With all the training data, we obtained R^2^ as 0.97 for GPP, 0.96 for RECO, and 0.94 for NEE.

**Fig. 6:**
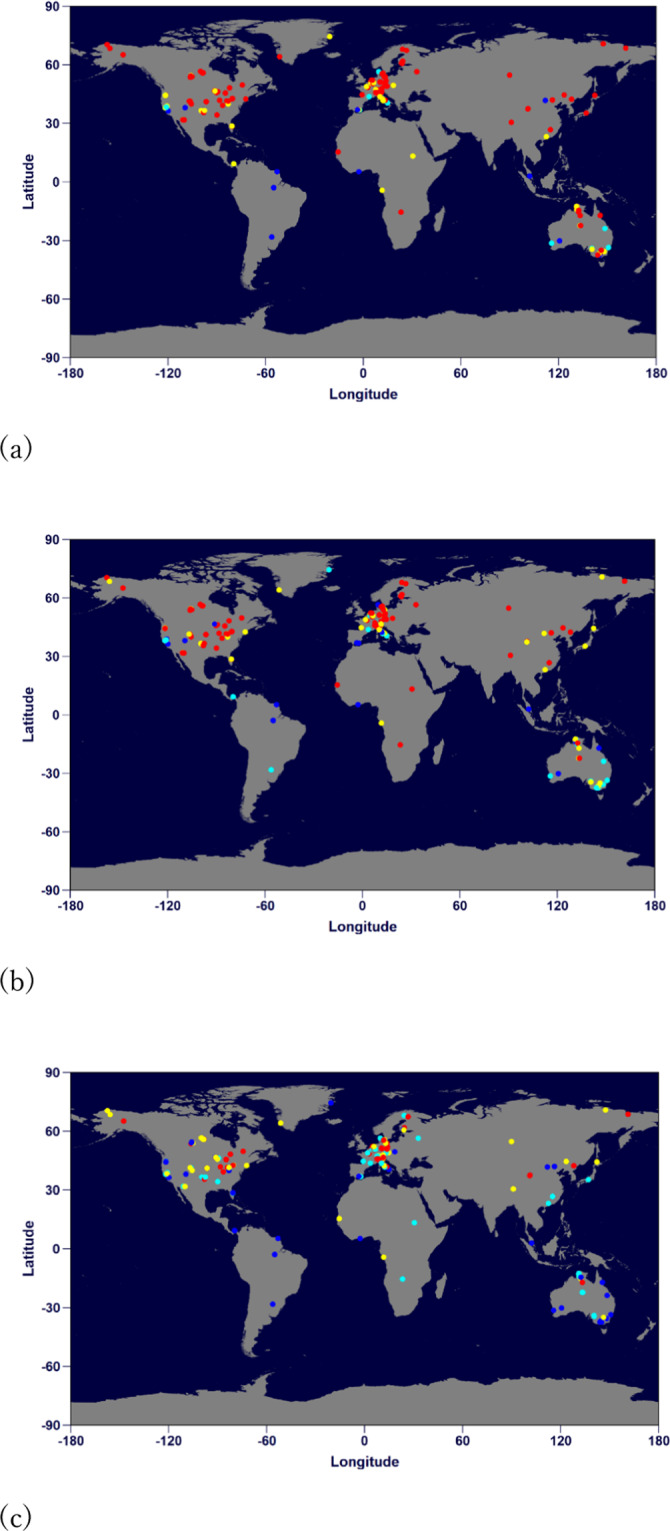
Leave-one-site-out validation for GPP (**a**), RECO (**b**) and NEE (**c**). Red: R^2^ ≥ 0.75; Yellow: 0.5 ≤ R^2^ < 0.75; Cyan: 0.25 ≤ R^2^ < 0.5; Blue: R^2^ < 0.25. For GPP, the percentage of sites is 62% (red), 24% (yellow), 8% (cyan), and 6% (blue). For NEE, the percentage is 23% (red), 27% (yellow), 27% (cyan), and 23% (blue).

The random data partition scheme of cross-validation may prevent RF from modelling a false relationship, but it gives little information on site-specific performances. To investigate these, we conducted leave-one-site-out validations. Sites were excluded one by one in the training data and the excluded sites were used for validation. The statistics of R^2^ are summarized in Table 1. The performance order is GPP > RECO > NEE, which indicates the order of uncertainties in their estimates. Figure 6 shows the spatial pattern of R^2^ for each site. Generally, RF performed better for forests that had large seasonal variations, as fluxes can be associated with the variations of predictors. In the areas where seasonal variation was small, site-specific uncertainty factors were more likely to blur the relationship between the target and predictors.

**Table 1:** Percentages of sites in ranges of R^2^ obtained by the leave-one-site-out validation.

Target	R^2^ > 0.75	0.5 < R^2^ < = 0.75	0.25 < R^2^ < = 0.5	R^2^ < 0.25
GPP	62%	24%	8%	6%
RECO	48%	30%	12%	10%
NEE	23%	27%	27%	23%

The total number of sites included in the training dataset is 204.

A concern on using short-term data to train RF to make long-term predictions is whether the bias would increase significantly with time due to the disturbance on ecosystems. We analysed the fitting of all sites’ data and summarised the results in Tables S1–S3. More statistical details are available in Supplementary File 2. Indeed, the p-value shows that the correlation of the bias with the year was significant for some sites.

**Fig. 7:**
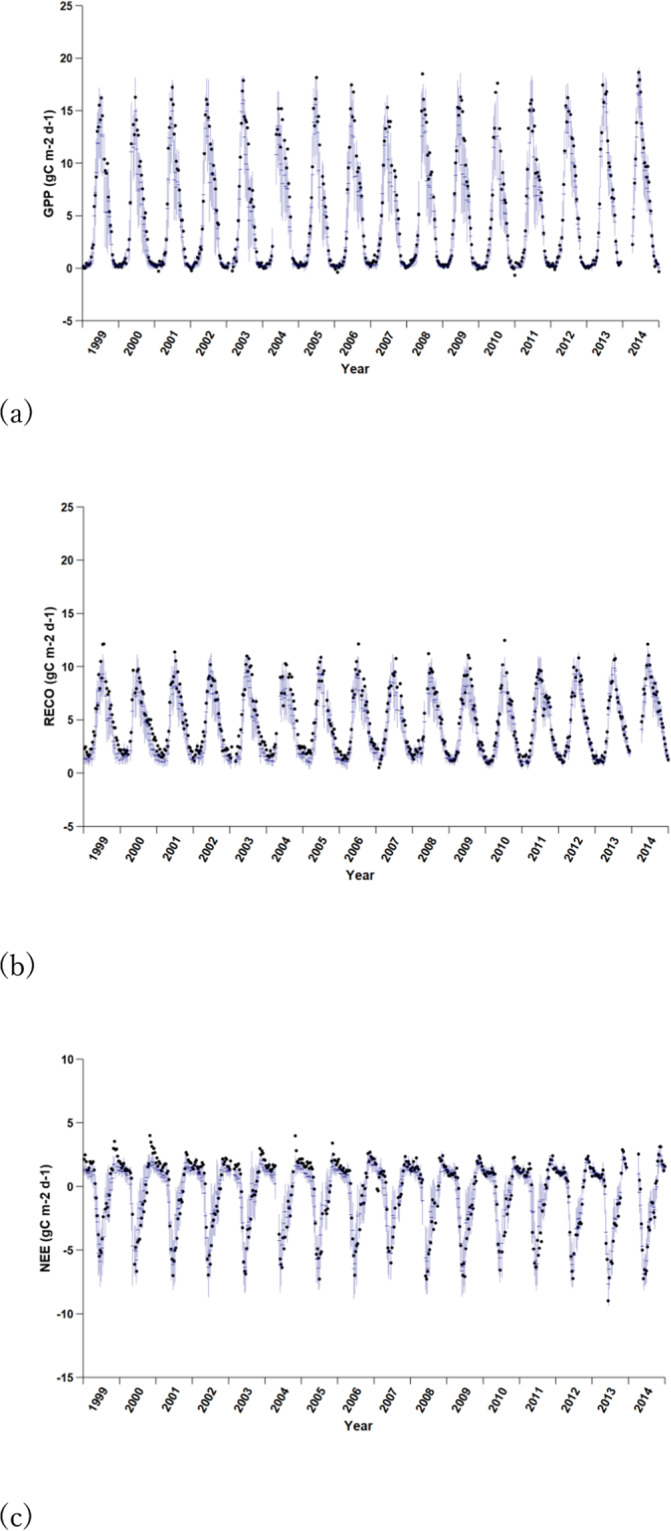
Fitting of GPP (**a**), RECO (**b**), and NEE (**c**) of site DK-SOR (55.4859°N, 11.6446°E). Dark dots represent measurements, horizontal blue bars represent the means (predictions) and vertical blue lines are the standard deviations of flux values in the terminal nodes of 500 trees.

Of all 204 sites, the site named DK-SOR (55.4859°N, 11.6446°E) is the only one that has data in all 16 years from 1999 (start year of this study) to 2014 (end year of FLUXNET2015) and its p-value is smaller than 0.05 for GPP, RECO, and NEE. We present its data fitting in Fig. 7. The trend is negligible considering the large variation of measurements. We provide plots and statistical details for all sites in Supplementary Files 3–5. They show that a significant bias trend was caused most likely by incomplete data or special events in some years.

**Fig. 8:**
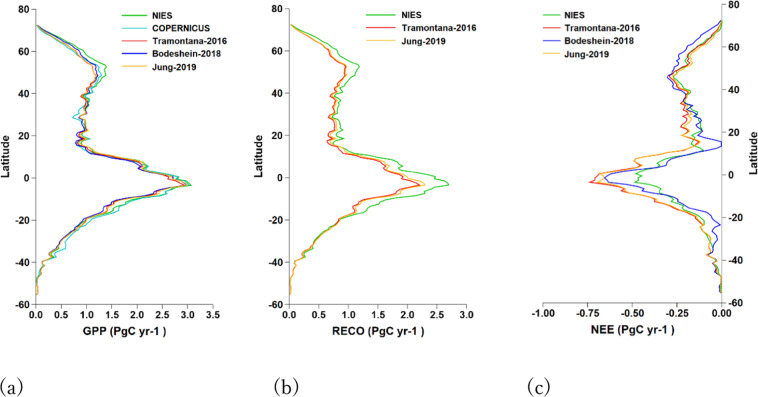
Inter-comparison of GPP (**a**), RECO (**b**) and NEE (**c**).

We compared this product (labelled as NIES) with those of Copernicus^41^, Tramontana *et al*.^27^, Bodesheim *et al*.^28^, and Jung *et al*.^29^ (Fig. 8). For cross-checking among products, we only compared data in the period 2001–2013 when all products were available. The temporal and spatial resolutions of the Copernicus GPP are 10 days and 1 km respectively. The Bodesheim-2018 GPP and RECO are in 0.5° by 0.5° grids. The annual fluxes were calculated from the half-hourly fluxes of 12 months. The spatial resolution of the annual products of Tramontana-2016 and Jung-2019 is also 0.5° by 0.5° degrees. Jung-2019 included GPP and RECO from both night-time and daytime partition methods. We used the datasets of night-time partition.

All products show similar latitudinal variations. The differences in GPP are the smallest, followed by those of RECO and NEE. Table 2 summarises the integrated fluxes. The GPP difference between NIES and Copernicus is the smallest, with about 1% larger in NIES GPP. The difference in NIES GPP is about 4%, 2%, and 3% when compared with the values of Tramontana-2016, Bodeshein-2018, and Jung-2019, respectively. While the NIES RECO is 8% larger than that of Tramontana-2016 and 6% larger than that of Bodeshein-2018, the NIES NEE is 13% smaller than that of Tramontana-2016, 4% smaller than that of Bodeshein-2018, and 10% smaller than that of Jung-2019.

**Table 2:** Inter-comparison of global fluxes per year.

NIES/Others	COPERNICUS	Tramontana-2016	Bodeshein-2018	Jung-2019
GPP	136.5/132.9	136.7/126.6	132.2/126.4	136.7/129.3
RECO		117.5/99.5		117.5/103.2
NEE		−21.0/−27.0	−20.2/−22.0	−21.0/−25.8

The integration includes grids in which the two products under comparison have data in 2001–2013.

## Bias and Uncertainty

Although the RF method usually produces unbiased fitting to measurements, it cannot avoid the problem of unbalanced sampling. As the RF makes predictions using values of the training samples, extrapolations to unsampled domains could lead to large biases and uncertainties. To investigate the problem, we compared the histograms of T2M and LAI in global grids with the histograms of T2M and LAI associated with the training samples. Photosynthesis is determined mainly by these two variables. In constructing the global histograms, the number of grid cells was weighted by the grid area so that the count would reflect the area correctly. The weighting was not used when counting samples as sites were considered representing the same area of their surroundings no matter where they were located.

**Fig. 9:**
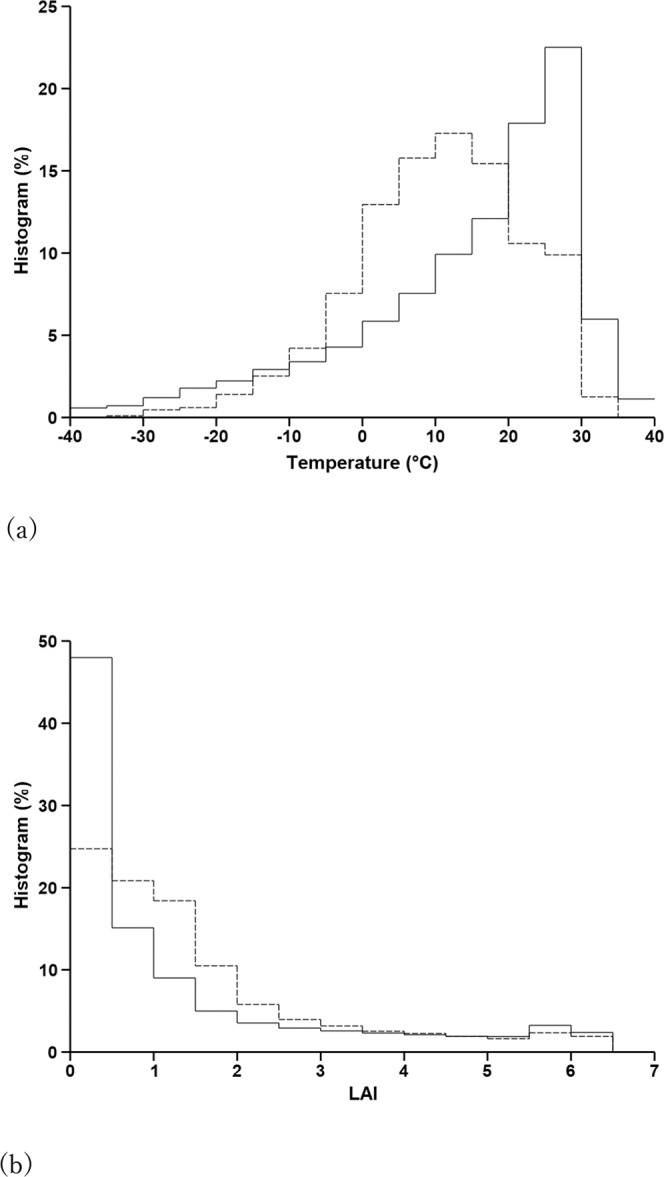
Histograms of T2M (**a**) and LAI (**b**) in 2014. Solid line: global grid statistics. Dashed line: flux site samples statistics.

Figure 9a shows that the sampling frequency of T2M was much smaller than that of the global grids in low- and high-temperature bins. This indicates that areas with a cold or hot climate are under-represented by the measurement sites; therefore, predictions for cold areas are likely to be biased toward warmer areas, although the exact impact is extremely difficult to diagnose unless the RF implementation is designed specifically for such a purpose. Similar biases exist in hot areas. Unbalanced sampling is also shown in the histogram of LAI (Fig. 9b), especially in the low LAI bins. This is partly related to air temperature as areas with a very cold climate tend to have a small LAI.

Unbalanced sampling might not be the main factor for the NEE overestimate as the modelled GPP agrees well with that of Copernicus, which was obtained by a different method. By analysing the variations of observed GPP, RECO, and NEE, we found that the main cause of the large NEE bias was that the mean NEE of any site in any season was relatively small in comparison with its variation, as shown in Table 3. For GPP and RECO, the mean flux was larger than the standard deviation (SD) and about three times as much as the model SD; but for the NEE, the mean flux was smaller than both the SD and the model SD. The bias of prediction was much smaller than all the mean fluxes, but the SD of the bias for NEE was nearly as large as the mean flux.

**Table 3:** Summary of flux, bias, and model. Unit is PgC per year.

	Flux	Bias	RF SD
GPP	3.24 ± 2.46	0.04 ± 0.69	1.07
RECO	2.61 ± 1.53	0.03 ± 0.50	0.83
NEE	−0.62 ± 1.42	−0.01 ± 0.58	0.90

Statistic details are listed in Tables S1 to S3.

Values in Table 3 are summaries of the statistics in Tables S1 to S3 with each site being considered as a unit no matter how many years of measurements it includes. Also, for each site, all years were considered equal in calculating the overall mean even if some years had a small number of data points. This could have led to the seasonality bias. However, if we only select the years with no missing data, many sites would have been excluded from the summary in Table 3, which would have increased the geographical bias.

## Supplementary information

Supplementary information

## Data Availability

We used the software by Wright and Ziegler^33^, available at https://github.com/imbs-hl/ranger. The code for data processing was written in ZeScript (https://www.zegraph.com/z-script/) and is available upon request.
