# Supplementary material for: Global terrestrial carbon fluxes of 1999–2019 estimated by upscaling eddy covariance data with a random forest
Source: Sci Data. 2020 Sep 24;7:313. doi: 10.1038/s41597-020-00653-5 (PMC7518252; doi:10.1038/s41597-020-00653-5)
Supplement: Supplementary file 1 — Supplementary information [file 41597_2020_653_MOESM1_ESM.zip › scidata/Supplementary File 1 (2).pdf]

## Plant Functional Type

This document contains maps of plant functional types extracted from MCD12C1 MODIS/Terra+Aqua and corresponding sites of FLUXNET 2015; and maps of derived variables of leaf area index extracted from Copernicus LAI data set.

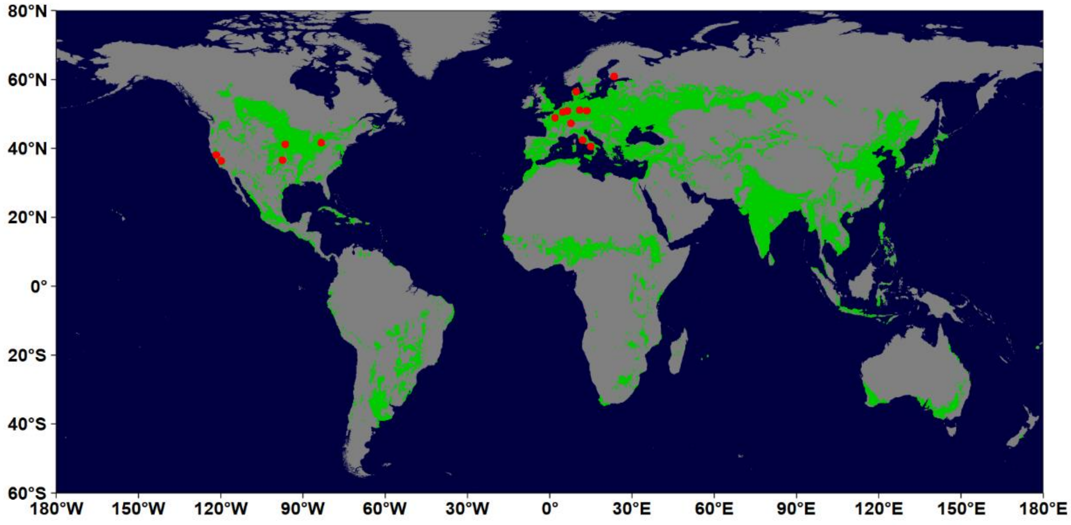


Figure S1. Croplands. Green: MCD12C1 MODIS/Terra+Aqua. Red: FLUXNET 2015.

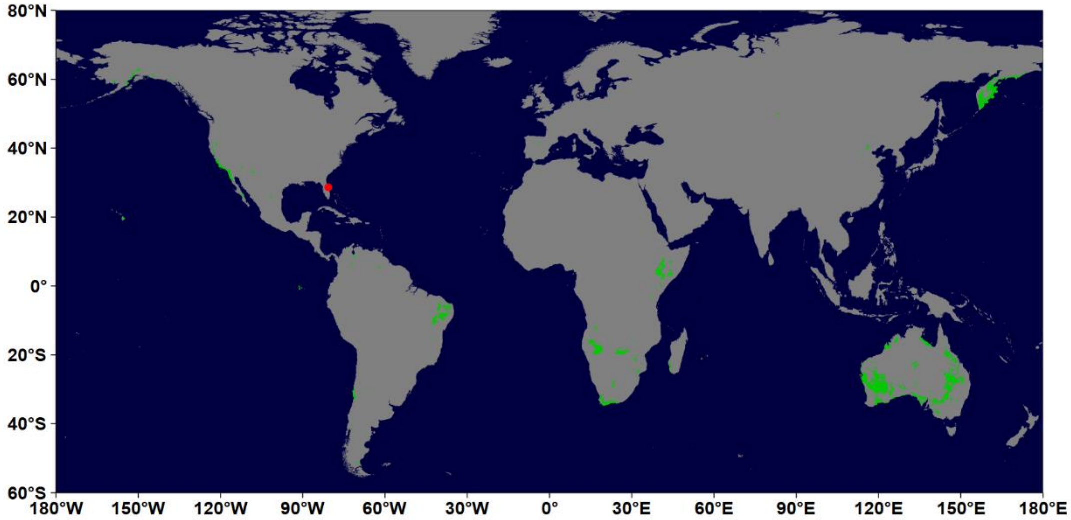


Figure S2. Closed Shrublands. Green: MCD12C1 MODIS/Terra+Aqua. Red: FLUXNET 2015.

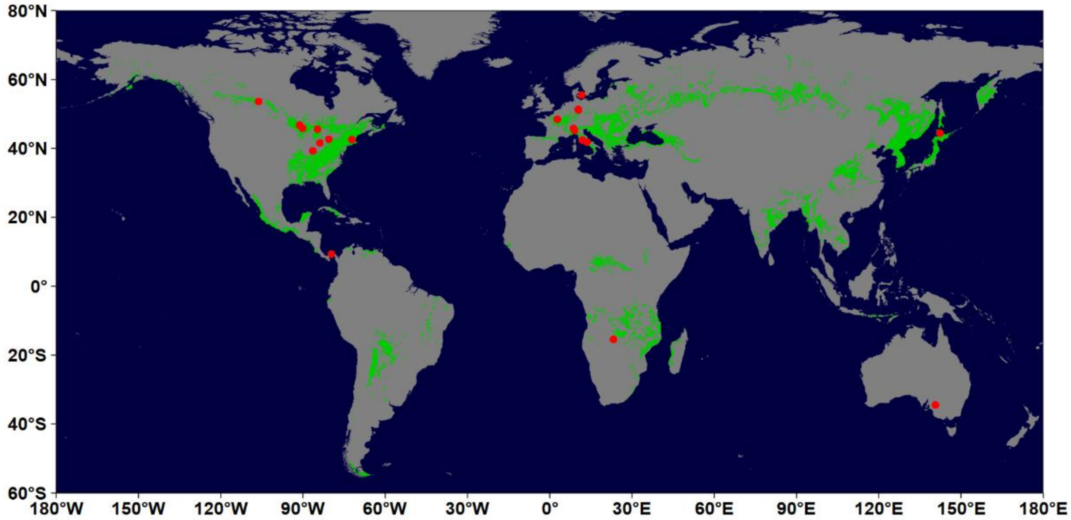


Figure S3. Deciduous Broadleaf Forests. Green: MCD12C1 MODIS/Terra+Aqua. Red: FLUXNET 2015.

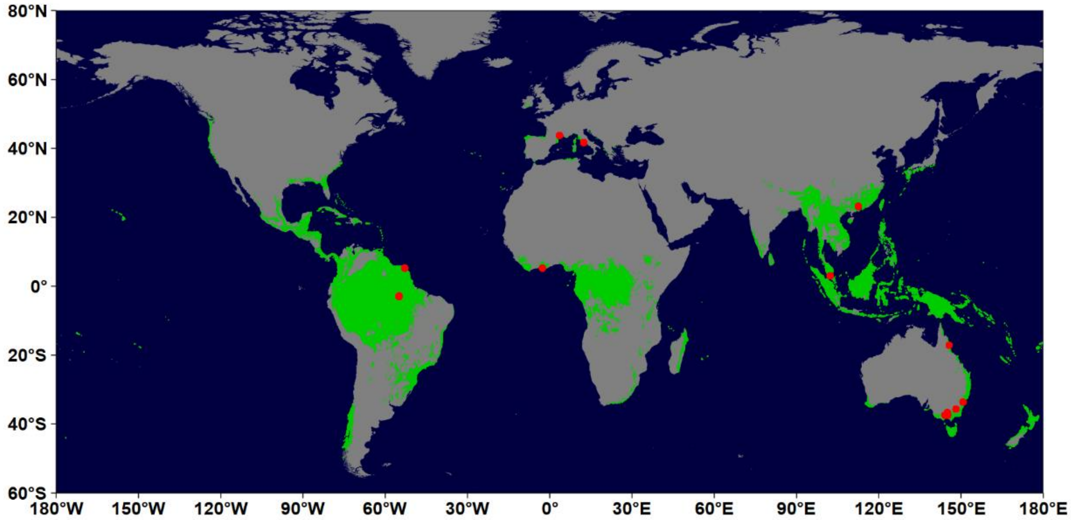


Figure S4. Evergreen Broadleaf Forests. Green: MCD12C1 MODIS/Terra+Aqua. Red: FLUXNET 2015.

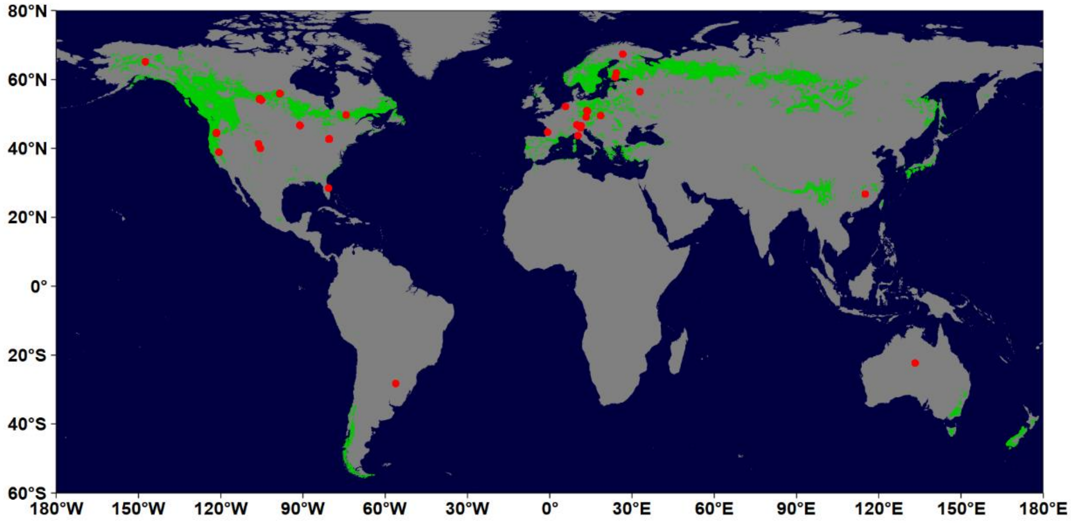


Figure S5. Evergreen Needleleaf Forests. Green: MCD12C1 MODIS/Terra+Aqua. Red: FLUXNET 2015.

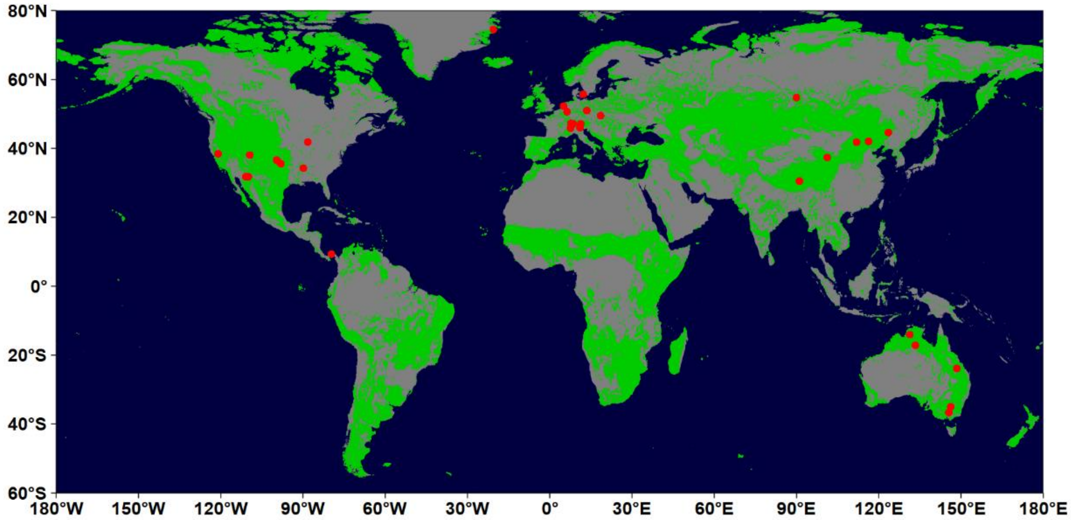


Figure S6. Grasslands. Green: MCD12C1 MODIS/Terra+Aqua. Red: FLUXNET 2015.

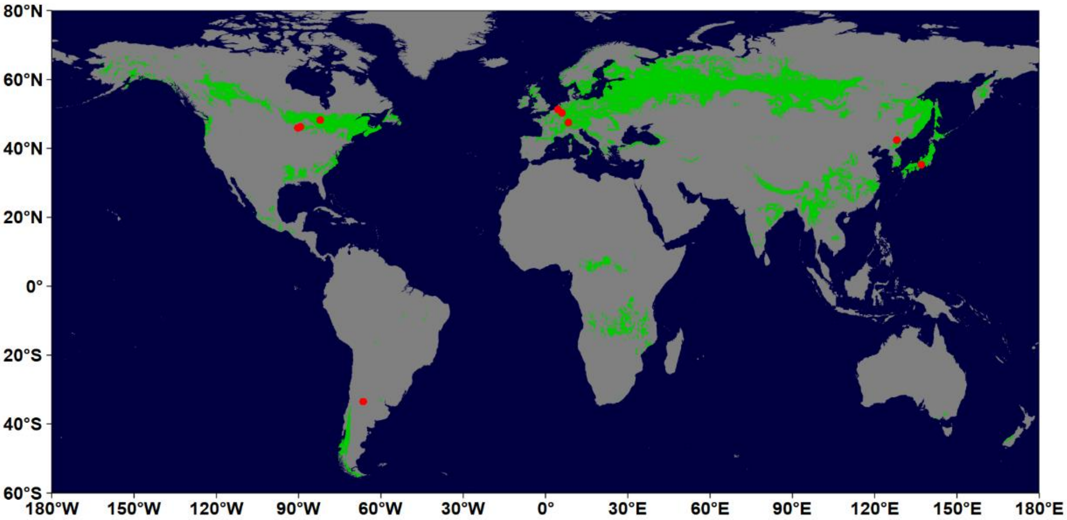


Figure S7. Mixed Forests. Green: MCD12C1 MODIS/Terra+Aqua. Red: FLUXNET 2015.

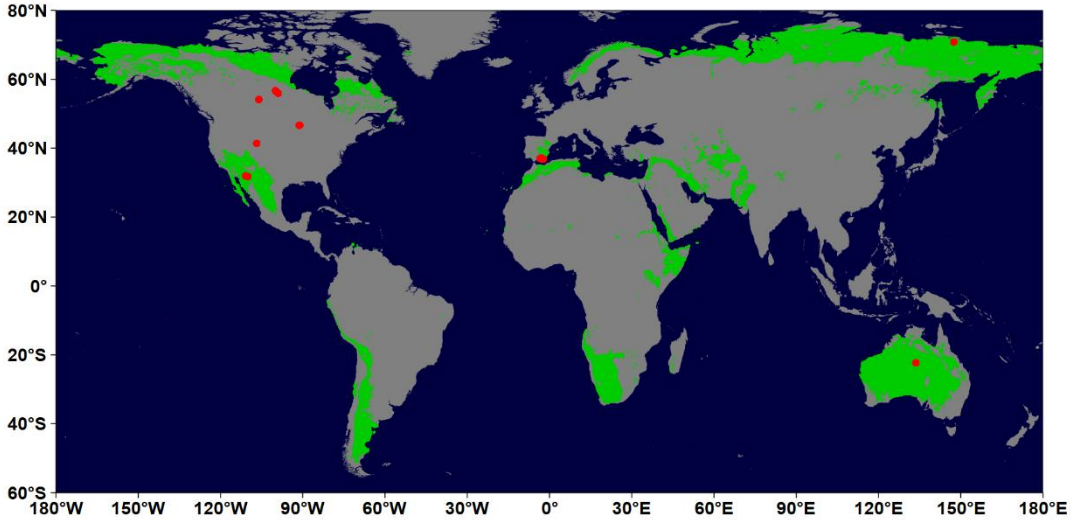


Figure S8. Open Shrublands. Green: MCD12C1 MODIS/Terra+Aqua. Red: FLUXNET 2015.

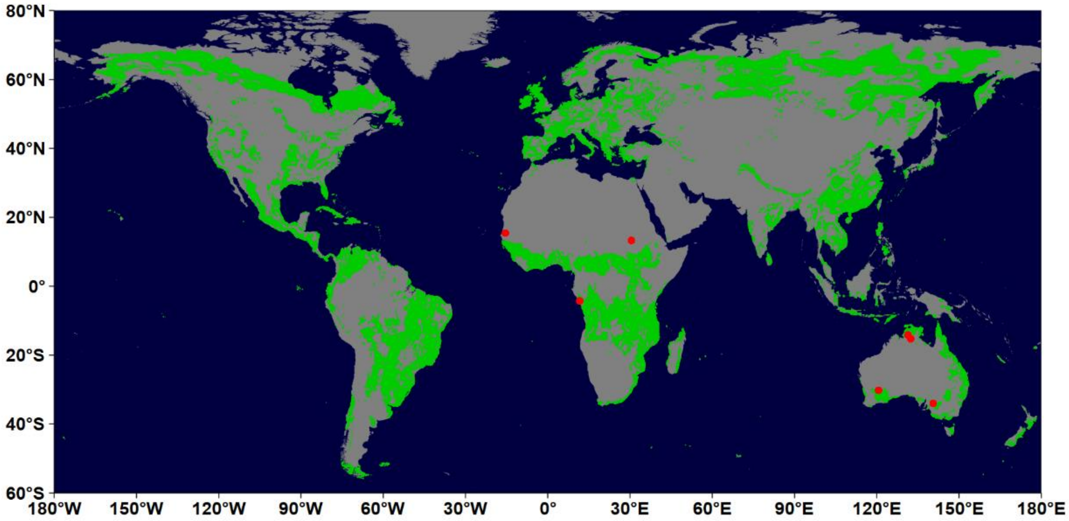


Figure S9. Savannas. Green: MCD12C1 MODIS/Terra+Aqua. Red: FLUXNET 2015.

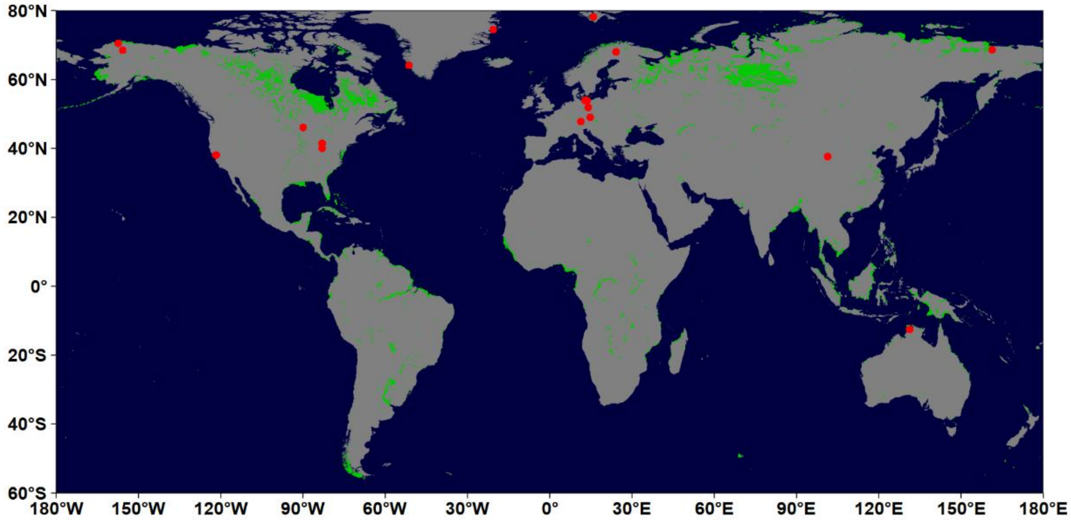


Figure S10. Permanent Wetlands. Green: MCD12C1 MODIS/Terra+Aqua. Red: FLUXNET 2015.

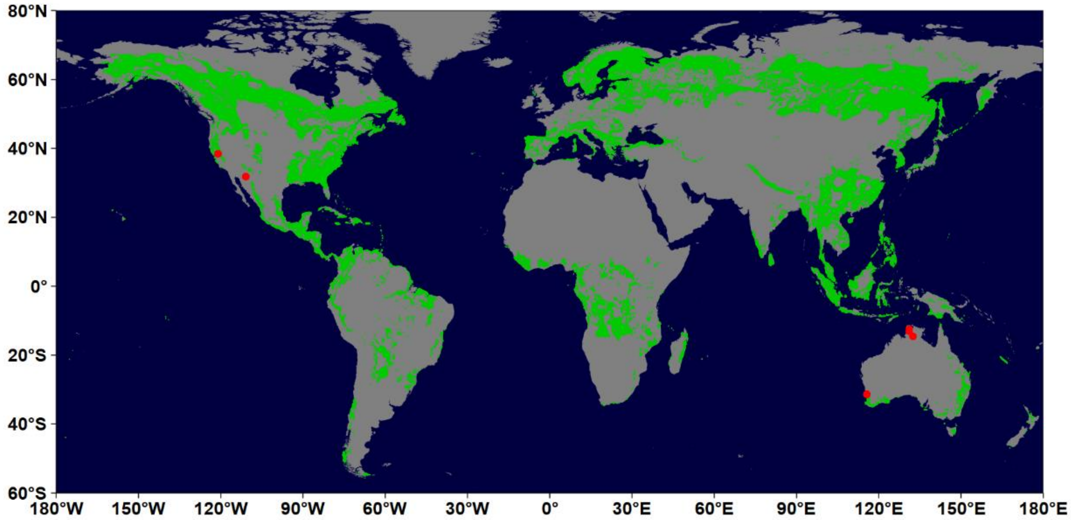


Figure S11. Woody Savannas. Green: MCD12C1 MODIS/Terra+Aqua. Red: FLUXNET 2015.

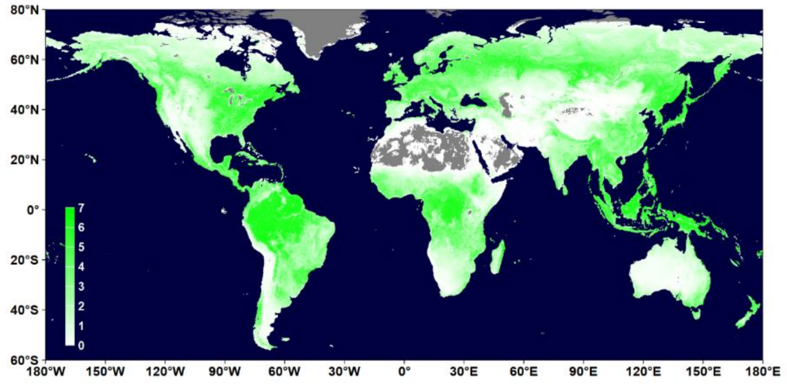


(a)

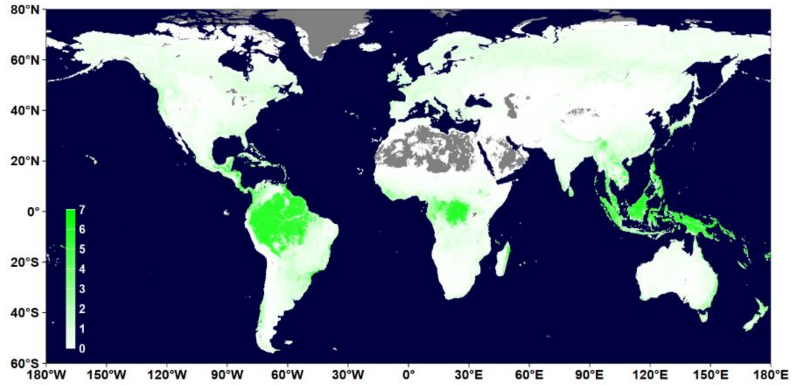


(b)

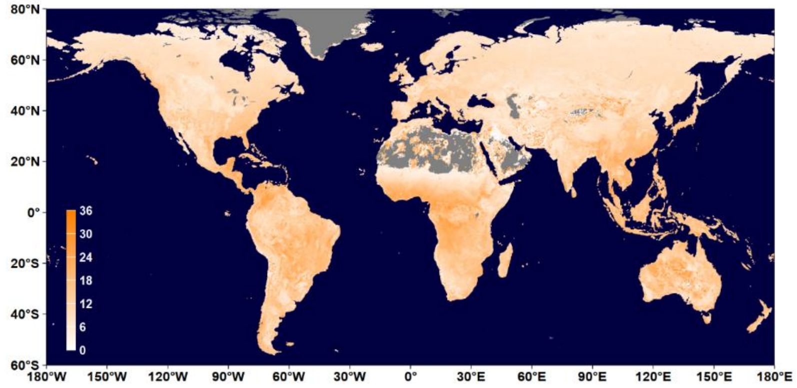


(c)

Figure S12. Distribution of derived LAI variables. (a): Maximum LAI in a year; (b): Minimum LAI in a year; (c): The number of LAI larger than the mean of LAI minimum and maximum.
