# Supplementary material for: Global terrestrial carbon fluxes of 1999–2019 estimated by upscaling eddy covariance data with a random forest
Source: Sci Data. 2020 Sep 24;7:313. doi: 10.1038/s41597-020-00653-5 (PMC7518252; doi:10.1038/s41597-020-00653-5)
Supplement: Supplementary file 1 — Supplementary information [file 41597_2020_653_MOESM1_ESM.zip › scidata/Supplementary File 2 (2).pdf]

Site Fitting Statistics.

This document contains site fitting statistics of GPP, RECO, and NEE. The p-value in Table S1 to S3 was calculated using a t-distribution

$$t = \frac{R\sqrt{n-2}}{\sqrt{1-R^2}} \qquad (1)$$

with n-2 degree of freedom; where n is the number of annual mean biases and R the correlation coefficient. The variation range of observed flux is the SD of 10-day means and the range of prediction bias is the SD of predictions minus observations. The model SD is calculated from flux values in the terminal nodes of 500 trees used to make predictions.

Table S1. Statistic summary of site GPP fitting.

Site ID	Latitude	Longitude	IGBP	Year	Observed Flux (gC m-2 d-1)	Prediction Bias (gC m-2 d-1)	ND	R	p-value	Model SD （gC m-2 d-1)
AR-SLu	-33.4648	-66.4598	MF	2009-2011	8.14±1.21	-0.68±0.32	3	-0.999	0.034	1.71
AR-Vir	-28.2395	-56.1886	ENF	2010-2012	11.00±2.24	-0.88±0.80	3	0.991	0.088	2.27
AT-Neu	47.1167	11.3175	GRA	2002-2012	4.98±5.27	-0.36±1.49	11	-0.080	0.816	1.73
AU-Ade	-13.0769	131.1178	WSA	2007-2009	5.90±2.20	-0.38±0.37	3	0.646	0.553	1.18
AU-ASM	-22.283	133.249	ENF	2010-2014	0.95±0.73	-0.12±0.35	5	0.893	0.042	0.45
AU-Cpr	-34.0021	140.5891	SAV	2010-2014	1.32±0.45	-0.07±0.21	5	0.868	0.057	0.60
AU-Cum	-33.6152	150.7236	EBF	2012-2014	3.35±0.64	0.07±0.28	3	0.738	0.471	0.91
AU-DaP	-14.0633	131.3181	GRA	2007-2013	3.56±3.80	-0.20±0.81	7	0.771	0.043	1.38
AU-DaS	-14.1593	131.3881	SAV	2008-2014	4.14±1.79	0.02±0.60	7	-0.392	0.384	1.06
AU-Dry	-15.2588	132.3706	SAV	2008-2014	3.11±1.35	0.15±0.36	7	-0.231	0.618	0.91
AU-Emr	-23.8587	148.4746	GRA	2011-2013	1.33±0.98	0.15±0.39	3	0.967	0.165	0.85
AU-Fog	-12.5452	131.3072	WET	2006-2008	2.34±1.36	0.55±0.47	3	-1.000	0.008	1.39
AU-Gin	-31.3764	115.7138	WSA	2011-2014	3.39±0.93	0.03±0.26	4	0.706	0.294	0.81
AU-GWW	-30.1913	120.6541	SAV	2013-2014	1.26±0.36	0.02±0.16	2			0.42
AU-How	-12.4943	131.1523	WSA	2001-2014	4.76±2.09	0.59±0.66	14	-0.923	0.000	1.41
AU-Lox	-34.4704	140.6551	DBF	2008-2009	7.67±3.96	-1.05±0.83	2			2.15
AU-RDF	-14.5636	132.4776	WSA	2011-2013	2.20±1.23	0.22±0.33	3	0.809	0.400	1.25
AU-Rig	-36.6499	145.5759	GRA	2011-2014	2.60±2.30	0.02±0.46	4	0.832	0.168	0.95
AU-Rob	-17.1175	145.6301	EBF	2014	5.11±1.15	0.86±0.42	1			1.97
AU-Stp	-17.1507	133.3502	GRA	2008-2014	1.01±1.33	0.09±0.44	7	0.123	0.794	0.68
AU-TTE	-22.287	133.64	OSH	2012-2014	0.00±0.56	0.01±0.17	3	0.711	0.497	0.30
AU-Tum	-35.6566	148.1517	EBF	2001-2014	9.16±3.51	-0.09±2.13	14	-0.088	0.765	2.61
AU-Wac	-37.4259	145.1878	EBF	2005-2008	6.38±1.54	0.10±0.77	4	0.707	0.293	1.34
AU-Whr	-36.6732	145.0294	EBF	2011-2014	3.88±0.85	-0.17±0.29	4	0.546	0.454	0.76
AU-Wom	-37.4222	144.0944	EBF	2010-2014	5.95±2.06	0.19±0.47	5	-0.162	0.795	1.24

AU-Ync	-34.9893	146.2907	GRA	2012-2014	0.34±0.62	0.07±0.17	3	0.984	0.113	0.45
BE-Bra	51.3076	4.5198	MF	1999-2014	3.33±2.82	0.45±0.79	15	-0.598	0.019	1.32
BE-Lon	50.5516	4.7461	CRO	2004-2014	3.74±5.18	-0.32±3.07	11	0.452	0.163	2.20
BE-Vie	50.305	5.9981	MF	1999-2014	3.72±3.30	0.05±0.76	16	-0.473	0.065	1.04
BR-Sa1	-2.8567	-54.9589	EBF	2002-2011	9.32±0.95	-0.13±0.53	9	0.053	0.892	0.77
BR-Sa3	-3.018	-54.9714	EBF	2000-2004	8.53±0.89	-0.09±0.46	5	-0.714	0.175	0.78
CA-Gro	48.2167	-82.1556	MF	2003-2014	2.33±2.67	0.09±0.70	12	0.601	0.039	1.04
CA-Man	55.8796	-98.4808	ENF	1999-2008	1.41±1.72	0.07±0.25	9	-0.617	0.077	0.57
CA-NS1	55.8792	-98.4839	ENF	2002-2005	2.17±2.35	-0.09±0.26	4	0.972	0.028	0.51
CA-NS2	55.9058	-98.5247	ENF	2001-2005	2.11±1.97	0.08±0.18	5	0.822	0.088	0.56
CA-NS3	55.9117	-98.3822	ENF	2001-2005	1.71±1.98	-0.07±0.42	5	-0.969	0.007	0.54
CA-NS4	55.9144	-98.3806	ENF	2002-2005	1.37±1.26	0.31±0.36	4	0.967	0.033	0.46
CA-NS5	55.8631	-98.485	ENF	2001-2005	2.09±2.48	0.12±0.28	5	-0.455	0.441	0.65
CA-NS6	55.9167	-98.9644	OSH	2001-2005	1.02±1.44	0.13±0.27	5	0.481	0.412	0.54
CA-NS7	56.6358	-99.9483	OSH	2002-2005	1.10±1.74	-0.01±0.26	4	0.943	0.057	0.44
CA-Oas	53.6289	-106.1978	DBF	1999-2010	2.57±4.06	0.12±0.66	12	-0.385	0.216	0.84
CA-Obs	53.9872	-105.1178	ENF	1999-2010	1.95±2.29	0.15±0.46	12	-0.501	0.097	0.88
CA-Qfo	49.6925	-74.3421	ENF	2003-2010	1.66±1.91	0.28±0.38	8	-0.742	0.035	0.77
CA-SF1	54.485	-105.8176	ENF	2003-2006	5.42±1.67	0.06±0.42	4	-0.950	0.050	1.18
CA-SF2	54.2539	-105.8775	ENF	2001-2005	6.23±2.85	-0.25±0.59	5	-0.374	0.535	1.46
CA-SF3	54.0916	-106.0053	OSH	2002-2006	2.48±1.13	0.09±0.29	5	0.433	0.467	0.89
CA-TP1	42.6609	-80.5595	ENF	2003-2014	1.93±1.97	0.90±1.30	12	-0.914	0.000	1.38
CA-TP2	42.7744	-80.4588	ENF	2003-2007	5.87±4.59	-0.73±1.01	5	-0.501	0.390	1.62
CA-TP3	42.7068	-80.3483	ENF	2003-2014	3.92±3.12	0.19±1.04	12	-0.678	0.015	1.29
CA-TP4	42.7102	-80.3574	ENF	2002-2014	3.21±3.41	0.37±0.94	13	-0.599	0.031	1.15
CA-TPD	42.6353	-80.5577	DBF	2012-2014	2.77±3.80	0.12±0.45	3	-0.995	0.065	0.76
CG-Tch	-4.2892	11.6564	SAV	2006-2009	2.85±2.50	0.00±0.51	4	-0.663	0.337	0.74
CH-Cha	47.2102	8.4104	GRA	2005-2014	5.52±4.16	-0.52±1.41	10	0.509	0.133	1.65

CH-Dav	46.8153	9.8559	ENF	1999-2014	3.13±2.44	0.10±0.83	16	-0.083	0.761	1.21
CH-Fru	47.1158	8.5378	GRA	2005-2014	4.56±4.65	-0.08±0.89	10	0.055	0.879	1.14
CH-Lae	47.4781	8.365	MF	2004-2014	4.52±3.14	0.16±0.77	11	-0.138	0.686	1.28
CH-Oe1	47.2858	7.7319	GRA	2002-2008	4.08±3.51	-0.65±1.10	7	0.951	0.001	1.25
CH-Oe2	47.2863	7.7343	CRO	2004-2014	3.17±3.78	0.16±1.76	11	-0.076	0.824	1.49
CN-Cha	42.4025	128.0958	MF	2003-2005	3.63±4.07	-0.25±0.40	3	0.997	0.051	0.95
CN-Cng	44.5934	123.5092	GRA	2007-2010	1.76±2.00	-0.04±0.39	4	-0.892	0.108	0.62
CN-Dan	30.4978	91.0664	GRA	2004-2005	0.90±0.91	-0.02±0.10	2			0.29
CN-Din	23.1733	112.5361	EBF	2003-2005	4.02±1.33	0.27±0.42	3	-0.374	0.756	1.11
CN-Du2	42.0467	116.2836	GRA	2007-2008	0.69±0.91	0.06±0.25	2			0.40
CN-Du3	42.0551	116.2809	GRA	2009-2010	0.06±0.47	0.25±0.28	2			0.39
CN-Ha2	37.6086	101.3269	WET	2003-2005	2.60±2.65	0.03±0.24	3	-0.850	0.353	0.69
CN-HaM	37.37	101.18	GRA	2002-2004	1.33±1.98	-0.04±0.33	3	0.734	0.476	0.50
CN-Qia	26.7414	115.0581	ENF	2003-2005	3.86±2.22	-0.04±0.42	3	0.082	0.948	1.12
CN-Sw2	41.7902	111.8971	GRA	2010-2012	0.62±0.44	-0.12±0.26	3	-0.479	0.682	0.47
CZ-BK1	49.5021	18.5369	ENF	2004-2014	5.09±3.49	0.03±1.17	11	-0.397	0.227	1.36
CZ-BK2	49.4944	18.5429	GRA	2006-2012	2.87±2.95	0.84±0.93	7	-0.774	0.041	1.32
CZ-wet	49.0247	14.7704	WET	2006-2014	2.52±3.20	0.33±0.87	9	0.092	0.813	1.21
DE-Akm	53.8662	13.6834	WET	2009-2014	3.64±3.62	-0.32±0.53	6	-0.044	0.935	1.13
DE-Geb	51.1001	10.9143	CRO	2001-2014	2.94±4.25	0.08±1.90	14	0.206	0.481	1.37
DE-Gri	50.95	13.5126	GRA	2004-2014	4.28±3.73	0.19±1.33	11	-0.472	0.143	1.36
DE-Hai	51.0792	10.453	DBF	2000-2012	4.24±4.88	-0.16±1.17	13	0.775	0.002	1.05
DE-Kli	50.8931	13.5224	CRO	2004-2014	3.32±4.05	-0.11±1.52	11	0.125	0.714	1.51
DE-Lkb	49.0996	13.3047	ENF	2009-2013	1.36±1.37	0.39±0.39	5	-0.537	0.351	1.01
DE-Lnf	51.3282	10.3678	DBF	2002-2012	4.28±4.90	-0.24±0.91	8	0.446	0.268	1.14
DE-Obe	50.7867	13.7213	ENF	2008-2014	3.88±3.84	0.02±0.74	7	-0.501	0.253	1.16
DE-RuR	50.6219	6.3041	GRA	2011-2014	4.94±3.50	0.02±0.61	4	0.611	0.389	1.04
DE-RuS	50.8659	6.4472	CRO	2011-2014	6.16±5.20	-0.53±1.41	4	0.299	0.701	2.21

DE-Seh	50.8706	6.4497	CRO	2007-2010	4.36±4.73	-0.63±1.79	4	0.776	0.224	1.75
DE-SfN	47.8064	11.3275	WET	2012-2014	2.06±1.64	0.47±0.52	3	-0.356	0.768	0.97
DE-Spw	51.8923	14.0337	WET	2010-2014	3.88±3.99	-0.04±0.53	5	-0.392	0.514	0.86
DE-Tha	50.9624	13.5652	ENF	1999-2014	4.17±3.76	-0.45±0.92	16	0.377	0.150	1.10
DE-Zrk	53.8759	12.889	WET	2013-2014	1.71±1.88	0.60±0.73	2			1.24
DK-Eng	55.6905	12.1918	GRA	2005-2008	2.33±1.94	0.37±0.97	4	0.526	0.474	1.28
DK-Fou	56.4842	9.5872	CRO	2005	3.66±2.92	0.43±1.23	1			1.58
DK-NuF	64.1308	-51.3861	WET	2008-2014	1.52±1.28	0.03±0.24	7	0.059	0.901	0.55
DK-Sor	55.4859	11.6446	DBF	1999-2014	5.41±5.64	-0.82±1.30	16	0.580	0.019	1.66
DK-ZaF	74.4814	-20.5545	WET	2008-2011	0.25±0.10	0.00±0.03	4	0.685	0.315	0.32
DK-ZaH	74.4733	-20.5503	GRA	2000-2014	0.69±0.53	0.02±0.23	15	0.351	0.200	0.27
ES-Amo	36.8336	-2.2523	OSH	2007-2012	-0.14±0.83	0.20±0.28	6	-0.670	0.146	0.48
ES-LgS	37.0979	-2.9658	OSH	2007-2009	1.30±1.08	0.21±0.23	3	-0.645	0.554	0.81
ES-LJu	36.9266	-2.7521	OSH	2004-2013	0.28±0.55	0.13±0.25	10	-0.517	0.126	0.56
ES-Ln2	36.9695	-3.4758	OSH	2009	-0.17±0.27	0.31±0.30	1			0.68
FI-Hyy	61.8474	24.2948	ENF	1999-2014	3.04±3.24	0.04±0.53	16	-0.460	0.073	0.98
FI-Jok	60.8986	23.5135	CRO	2000-2003	0.87±1.65	0.31±0.52	4	-0.854	0.146	0.82
FI-Let	60.6418	23.9595	ENF	2009-2012	2.37±3.10	-0.07±0.37	4	-0.760	0.240	0.64
FI-Lom	67.9972	24.2092	WET	2007-2009	1.06±1.94	0.13±0.28	3	-0.996	0.058	0.51
FI-Sod	67.3624	26.6386	ENF	2001-2014	1.58±2.06	-0.20±0.51	14	0.550	0.041	0.72
FR-Fon	48.4764	2.7801	DBF	2005-2014	4.73±4.88	-0.08±1.00	10	0.747	0.013	1.23
FR-Gri	48.8442	1.9519	CRO	2004-2014	4.14±4.81	-0.19±2.18	11	0.013	0.970	1.78
FR-LBr	44.7171	-0.7693	ENF	1999-2008	4.79±2.57	-0.04±0.91	10	-0.125	0.731	1.36
FR-Pue	43.7413	3.5957	EBF	2000-2014	3.24±1.59	-0.28±0.93	15	0.594	0.020	1.37
GF-Guy	5.2788	-52.9249	EBF	2004-2014	10.04±1.17	-0.20±1.01	11	0.110	0.747	1.19
GH-Ank	5.2685	-2.6942	EBF	2011-2014	6.88±1.48	0.05±0.64	3	0.727	0.482	1.55
IT-BCi	40.5238	14.9574	CRO	2004-2014	5.19±4.78	-0.05±3.24	11	-0.421	0.197	2.35
IT-CA1	42.3804	12.0266	DBF	2011-2014	3.22±2.65	0.04±0.60	4	0.672	0.328	1.11

IT-CA2	42.3772	12.026	CRO	2011-2014	2.46±2.45	0.23±0.91	4	0.368	0.632	1.51
IT-CA3	42.38	12.0222	DBF	2011-2014	2.36±1.45	0.11±0.40	4	-0.861	0.139	0.96
IT-Col	41.8494	13.5881	DBF	1999-2014	3.64±3.75	0.36±0.61	16	-0.366	0.164	1.18
IT-Cp2	41.7043	12.3573	EBF	2012-2014	5.46±1.70	0.23±0.58	3	-0.378	0.753	1.25
IT-Cpz	41.7053	12.3761	EBF	2000-2008	5.29±1.88	0.08±0.85	9	-0.194	0.616	1.44
IT-Isp	45.8126	8.6336	DBF	2013-2014	3.33±4.41	-0.38±0.75	2			1.14
IT-La2	45.9542	11.2853	ENF	2000-2002	3.89±3.45	-0.21±0.67	3	0.906	0.278	1.06
IT-Lav	45.9562	11.2813	ENF	2003-2014	6.10±3.67	-0.59±1.16	12	0.036	0.911	1.44
IT-MBo	46.0147	11.0458	GRA	2003-2013	3.87±4.26	-0.15±0.85	11	-0.237	0.482	1.30
IT-PT1	45.2009	9.061	DBF	2002-2004	5.34±4.60	-0.12±0.52	3	0.374	0.756	1.15
IT-Ren	46.5869	11.4337	ENF	1999-2013	2.78±2.96	-0.09±0.59	13	-0.412	0.162	0.90
IT-Ro1	42.4081	11.93	DBF	2000-2008	3.75±3.06	-0.01±0.89	9	0.180	0.643	1.64
IT-Ro2	42.3903	11.9209	DBF	2002-2012	3.60±3.83	0.13±1.47	10	0.226	0.531	1.69
IT-SR2	43.732	10.291	ENF	2013-2014	6.28±3.16	-0.11±0.60	2			1.24
IT-SRo	43.7279	10.2844	ENF	1999-2012	5.72±2.47	0.35±1.29	14	-0.646	0.013	1.75
IT-Tor	45.8444	7.5781	GRA	2008-2014	2.76±3.21	-0.10±0.58	7	0.549	0.202	1.07
JP-MBF	44.3869	142.3186	DBF	2004-2005	2.98±3.21	0.46±0.60	2			1.14
JP-SMF	35.2617	137.0788	MF	2002-2006	2.33±1.33	0.19±0.40	5	0.685	0.202	0.91
MY-PSO	2.973	102.3062	EBF	2003-2009	6.75±0.69	0.08±0.47	7	-0.227	0.624	0.57
NL-Hor	52.2404	5.0713	GRA	2004-2011	3.52±3.26	0.26±0.56	8	-0.349	0.397	1.04
NL-Loo	52.1666	5.7436	ENF	1999-2014	4.21±2.85	-0.53±0.69	16	0.592	0.016	1.16
NO-Adv	78.186	15.923	WET	2012-2013	0.77±0.81	-0.45±0.65	2			0.38
NO-Blv	78.9216	11.8311	SNO	2008-2009	0.05±0.11	0.23±0.13	2			0.38
PA-SPn	9.3181	-79.6346	DBF	2007-2009	4.99±1.48	0.08±0.59	3	-0.837	0.369	1.08
PA-SPs	9.3138	-79.6314	GRA	2007-2009	6.68±2.69	-0.01±0.74	3	0.346	0.775	1.14
RU-Che	68.613	161.3414	WET	2002-2005	1.17±1.51	0.09±0.19	4	0.965	0.035	0.48
RU-Cok	70.8291	147.4943	OSH	2003-2013	2.36±1.51	-0.01±0.29	11	0.376	0.254	0.77
RU-Fyo	56.4615	32.9221	ENF	1999-2014	3.78±3.85	-0.48±1.11	16	0.446	0.084	1.01

RU-Ha1	54.7252	90.0022	GRA	2002-2004	2.11±1.82	-0.05±0.21	3	-0.963	0.174	0.61
SD-Dem	13.2829	30.4783	SAV	2005-2009	2.86±1.73	-0.54±0.39	4	0.895	0.105	0.93
SN-Dhr	15.4028	-15.4322	SAV	2010-2013	5.61±3.03	-0.29±0.86	4	0.789	0.211	1.48
US-AR1	36.4267	-99.42	GRA	2009-2012	1.45±1.75	0.04±0.78	4	-0.314	0.686	0.94
US-AR2	36.6358	-99.5975	GRA	2009-2012	1.00±1.20	0.01±0.40	4	0.416	0.584	0.58
US-ARb	35.5497	-98.0402	GRA	2005-2006	2.73±3.60	0.08±0.67	2			1.14
US-ARc	35.5465	-98.04	GRA	2005-2006	3.53±3.31	0.12±0.41	2			1.08
US-ARM	36.6058	-97.4888	CRO	2003-2012	1.85±2.34	0.17±1.03	10	-0.579	0.080	1.18
US-Atq	70.4696	-157.4089	WET	2003-2008	0.65±1.11	-0.01±0.40	6	-0.241	0.645	0.37
US-Blo	38.8953	-120.6328	ENF	1999-2006	3.02±1.90	0.29±0.49	8	-0.492	0.215	1.21
US-Cop	38.09	-109.39	GRA	2001-2007	0.28±0.36	0.04±0.16	5	-0.465	0.430	0.28
US-CRT	41.6285	-83.3471	CRO	2011-2013	1.78±4.17	0.17±1.16	3	0.967	0.163	1.40
US-GBT	41.3658	-106.2397	ENF	2001-2003	1.48±1.25	0.02±0.37	3	0.955	0.191	0.66
US-GLE	41.3665	-106.2399	ENF	2005-2014	1.16±1.85	0.47±0.59	10	-0.495	0.145	0.96
US-Goo	34.2547	-89.8735	GRA	2002-2006	3.40±2.99	0.20±0.52	5	-0.503	0.388	1.12
US-Ha1	42.5378	-72.1715	DBF	1999-2012	2.39±3.41	0.37±0.69	14	-0.774	0.001	1.02
US-IB2	41.8406	-88.241	GRA	2004-2011	2.49±3.40	0.13±0.68	8	-0.223	0.596	0.90
US-Ivo	68.4865	-155.7503	WET	2004-2007	0.96±1.41	0.06±0.30	4	-0.969	0.031	0.51
US-KS1	28.4583	-80.6709	ENF	2002	5.30±1.26	0.21±0.28	1			1.04
US-KS2	28.6086	-80.6715	CSH	2003-2006	5.18±1.35	-0.03±0.52	4	0.945	0.055	1.05
US-Lin	36.3566	-119.8423	CRO	2009-2010	1.34±0.37	0.19±0.32	2			1.02
US-Los	46.0827	-89.9792	WET	2000-2014	2.11±2.15	0.51±0.53	11	-0.579	0.062	0.98
US-Me1	44.5794	-121.5	ENF	2004-2005	1.21±0.90	-0.11±0.16	2			0.43
US-Me2	44.4523	-121.5574	ENF	2002-2014	4.79±2.76	0.32±0.95	13	-0.677	0.011	1.48
US-Me3	44.3154	-121.6078	ENF	2004-2009	2.41±1.49	0.05±0.51	6	0.018	0.973	0.81
US-Me4	44.4992	-121.6224	ENF	1999-2000	4.32±1.61	0.02±0.58	2			1.32
US-Me5	44.4372	-121.5668	ENF	2000-2002	2.43±1.63	0.17±0.36	3	-0.389	0.745	0.92
US-Me6	44.3233	-121.6078	ENF	2010-2014	2.59±1.58	0.01±0.39	5	0.608	0.277	0.77

US-MMS	39.3232	-86.4131	DBF	1999-2014	3.45±4.68	0.29±1.20	16	0.015	0.956	1.16
US-Myb	38.0498	-121.7651	WET	2011-2014	5.05±3.38	0.15±0.93	4	-0.348	0.652	1.85
US-Ne1	41.1651	-96.4766	CRO	2001-2013	1.91±4.69	-0.28±1.49	13	0.738	0.004	0.98
US-Ne2	41.1649	-96.4701	CRO	2001-2013	3.06±5.16	-0.28±1.77	13	0.485	0.093	1.45
US-Ne3	41.1797	-96.4397	CRO	2001-2013	2.70±4.75	-0.44±1.90	13	0.521	0.068	1.33
US-NR1	40.0329	-105.5464	ENF	1999-2014	1.99±2.34	0.40±0.56	16	-0.272	0.309	1.29
US-Oho	41.5545	-83.8438	DBF	2004-2013	4.00±5.04	-0.14±0.89	10	0.719	0.019	1.05
US-ORv	40.0201	-83.0183	WET	2011	0.47±0.46	0.30±0.30	1			0.72
US-PFa	45.9459	-90.2723	MF	1999-2014	1.71±2.45	0.44±0.79	16	0.221	0.411	0.98
US-Prr	65.1237	-147.4876	ENF	2010-2014	0.64±1.03	0.09±0.19	5	0.635	0.250	0.35
US-SRC	31.9083	-110.8395	OSH	2008-2014	0.45±0.41	-0.00±0.17	7	-0.154	0.741	0.26
US-SRG	31.7894	-110.8277	GRA	2008-2014	1.19±1.54	-0.02±0.49	7	0.086	0.855	0.58
US-SRM	31.8214	-110.8661	WSA	2004-2014	0.70±1.00	0.03±0.29	11	0.130	0.702	0.44
US-Sta	41.3966	-106.8024	OSH	2005-2009	0.80±0.64	-0.04±0.18	5	-0.500	0.391	0.28
US-Syv	46.242	-89.3477	MF	2001-2014	3.47±3.67	0.32±0.71	10	-0.849	0.002	1.08
US-Ton	38.4316	-120.966	WSA	2001-2014	2.19±1.76	-0.13±0.80	14	-0.231	0.427	1.02
US-Tw1	38.1074	-121.6469	WET	2012-2014	8.75±3.53	-1.40±1.26	3	-0.953	0.196	2.28
US-Tw2	38.1047	-121.6433	CRO	2012-2013	3.34±3.47	0.10±0.65	2			1.36
US-Tw3	38.1159	-121.6467	CRO	2013-2014	8.97±4.75	-0.57±1.60	2			2.45
US-Tw4	38.103	-121.6414	WET	2013-2014	1.11±1.16	0.94±0.98	2			1.07
US-Twt	38.1087	-121.653	CRO	2009-2014	5.18±5.46	0.44±2.02	6	-0.765	0.077	2.36
US-UMB	45.5598	-84.7138	DBF	2000-2014	1.85±3.26	0.28±0.55	15	-0.881	0.000	0.64
US-UMd	45.5625	-84.6975	DBF	2007-2014	3.29±4.09	0.21±0.66	8	-0.761	0.028	0.87
US-Var	38.4133	-120.9507	GRA	2000-2014	1.82±2.59	-0.17±0.99	15	0.577	0.024	1.21
US-WCr	45.8059	-90.0799	DBF	1999-2014	2.12±3.54	0.24±0.76	13	-0.606	0.028	0.81
US-Whs	31.7438	-110.0522	OSH	2007-2014	0.36±0.73	0.03±0.19	8	-0.550	0.158	0.29
US-Wi0	46.6188	-91.0814	ENF	2002	4.62±3.57	0.18±0.47	1			1.09
US-Wi1	46.7305	-91.2329	DBF	2003	5.31±4.09	0.09±1.10	1			1.55

US-Wi2	46.6869	-91.1528	ENF	2003	2.78±2.30	-0.85±0.57	1			0.95
US-Wi3	46.6347	-91.0987	DBF	2002-2004	5.54±4.02	-0.03±0.47	2			1.12
US-Wi4	46.7393	-91.1663	ENF	2002-2005	7.21±2.27	-0.12±0.61	4	0.102	0.898	1.17
US-Wi5	46.6531	-91.0858	ENF	2004	4.25±3.20	0.46±0.58	1			1.30
US-Wi6	46.6249	-91.2982	OSH	2002	2.05±2.37	0.97±0.83	1			1.53
US-Wi7	46.6491	-91.0693	OSH	2005	3.19±2.61	1.24±0.76	1			2.12
US-Wi8	46.7223	-91.2524	DBF	2002	3.84±2.78	1.06±0.85	1			1.69
US-Wi9	46.6188	-91.0814	ENF	2004-2005	2.82±1.68	0.83±0.50	2			1.56
US-Wkg	31.7365	-109.9419	GRA	2004-2014	0.38±0.72	-0.02±0.29	11	-0.077	0.822	0.32
US-WPT	41.4646	-82.9962	WET	2011-2013	1.36±2.57	0.12±0.42	3	0.931	0.238	0.67
ZM-Mon	-15.4378	23.2528	DBF	2000-2009	4.76±2.38	0.09±0.36	4	-0.995	0.005	1.10

Table S2. Statistic summary of site RECO fitting.

Site ID	Latitude	Longitude	IGBP	Year	Observed Flux (gC m-2 d-1)	Prediction Bias (gC m-2 d-1)	ND	R	p-value	Model SD （gC m-2 d-1)
AR-SLu	-33.4648	-66.4598	MF	2009-2011	3.34±0.52	0.14±0.24	3	-0.894	0.296	0.92
AR-Vir	-28.2395	-56.1886	ENF	2010-2012	12.57±3.81	-1.15±2.05	3	-0.283	0.818	3.23
AT-Neu	47.1167	11.3175	GRA	2002-2012	5.98±4.40	-0.44±1.58	11	-0.205	0.545	1.95
AU-Ade	-13.0769	131.1178	WSA	2007-2009	4.57±1.43	-0.26±0.31	3	0.903	0.283	0.93
AU-ASM	-22.283	133.249	ENF	2010-2014	0.89±0.63	-0.08±0.30	5	0.917	0.028	0.36
AU-Cpr	-34.0021	140.5891	SAV	2010-2014	1.03±0.47	-0.01±0.23	5	0.797	0.107	0.51
AU-Cum	-33.6152	150.7236	EBF	2012-2014	2.71±0.85	0.17±0.27	3	-0.790	0.420	0.81
AU-DaP	-14.0633	131.3181	GRA	2007-2013	3.08±2.46	-0.13±0.78	7	0.770	0.043	1.00
AU-DaS	-14.1593	131.3881	SAV	2008-2014	3.45±1.67	-0.01±0.81	7	-0.165	0.723	1.10
AU-Dry	-15.2588	132.3706	SAV	2008-2014	2.44±1.05	0.10±0.45	7	0.582	0.170	0.81
AU-Emr	-23.8587	148.4746	GRA	2011-2013	1.39±0.43	0.10±0.20	3	0.986	0.107	0.52
AU-Fog	-12.5452	131.3072	WET	2006-2008	0.94±0.70	0.57±0.41	3	-0.874	0.323	1.24
AU-Gin	-31.3764	115.7138	WSA	2011-2014	2.59±0.58	-0.01±0.24	4	0.985	0.015	0.54
AU-GWW	-30.1913	120.6541	SAV	2013-2014	0.92±0.50	0.02±0.17	2			0.33
AU-How	-12.4943	131.1523	WSA	2001-2014	3.08±1.42	0.69±0.73	14	-0.829	0.000	1.05
AU-Lox	-34.4704	140.6551	DBF	2008-2009	4.35±1.72	-0.41±0.46	2			1.02
AU-RDF	-14.5636	132.4776	WSA	2011-2013	2.49±1.15	0.04±0.28	3	1.000	0.015	0.76
AU-Rig	-36.6499	145.5759	GRA	2011-2014	2.33±1.06	0.08±0.29	4	0.803	0.197	0.69
AU-Rob	-17.1175	145.6301	EBF	2014	3.01±1.75	0.59±1.08	1			1.97
AU-Stp	-17.1507	133.3502	GRA	2008-2014	0.92±0.67	0.25±0.33	7	-0.456	0.304	0.48
AU-TTE	-22.287	133.64	OSH	2012-2014	0.45±0.50	0.01±0.13	3	0.830	0.377	0.24
AU-Tum	-35.6566	148.1517	EBF	2001-2014	7.52±3.70	-0.58±2.31	14	0.287	0.321	2.70
AU-Wac	-37.4259	145.1878	EBF	2005-2008	1.68±1.46	0.19±0.72	4	0.856	0.144	1.50
AU-Whr	-36.6732	145.0294	EBF	2011-2014	2.53±0.76	0.09±0.30	4	-0.708	0.292	0.65
AU-Wom	-37.4222	144.0944	EBF	2010-2014	2.96±1.44	0.10±0.74	5	0.516	0.374	1.35

AU-Ync	-34.9893	146.2907	GRA	2012-2014	0.57±0.25	0.09±0.14	3	0.142	0.909	0.31
BE-Bra	51.3076	4.5198	MF	1999-2014	3.30±1.24	0.28±0.65	15	-0.242	0.385	1.18
BE-Lon	50.5516	4.7461	CRO	2004-2014	2.77±1.93	-0.04±0.98	11	0.084	0.805	1.20
BE-Vie	50.305	5.9981	MF	1999-2014	3.06±1.73	0.48±0.67	16	-0.706	0.002	0.96
BR-Sa1	-2.8567	-54.9589	EBF	2002-2011	9.18±1.14	-0.05±0.58	9	0.221	0.568	0.85
BR-Sa3	-3.018	-54.9714	EBF	2000-2004	8.12±1.37	-0.04±0.64	5	-0.808	0.098	0.97
CA-Gro	48.2167	-82.1556	MF	2003-2014	2.20±1.85	-0.21±0.58	12	0.534	0.073	0.80
CA-Man	55.8796	-98.4808	ENF	1999-2008	1.41±1.32	0.05±0.26	9	-0.281	0.463	0.50
CA-NS1	55.8792	-98.4839	ENF	2002-2005	1.79±1.41	-0.09±0.21	4	0.026	0.974	0.41
CA-NS2	55.9058	-98.5247	ENF	2001-2005	1.45±1.16	0.18±0.22	5	0.396	0.509	0.48
CA-NS3	55.9117	-98.3822	ENF	2001-2005	1.62±1.49	-0.08±0.39	5	-0.835	0.078	0.49
CA-NS4	55.9144	-98.3806	ENF	2002-2005	1.26±1.01	0.27±0.32	4	0.747	0.253	0.39
CA-NS5	55.8631	-98.485	ENF	2001-2005	1.84±1.89	0.07±0.28	5	-0.507	0.383	0.56
CA-NS6	55.9167	-98.9644	OSH	2001-2005	1.05±1.01	0.20±0.26	5	-0.375	0.534	0.48
CA-NS7	56.6358	-99.9483	OSH	2002-2005	1.35±1.24	-0.05±0.20	4	0.974	0.026	0.36
CA-Oas	53.6289	-106.1978	DBF	1999-2010	2.44±2.29	-0.14±0.51	12	-0.264	0.406	0.73
CA-Obs	53.9872	-105.1178	ENF	1999-2010	1.91±1.84	0.09±0.32	12	-0.582	0.047	0.68
CA-Qfo	49.6925	-74.3421	ENF	2003-2010	1.68±1.46	0.16±0.31	8	-0.820	0.013	0.62
CA-SF1	54.485	-105.8176	ENF	2003-2006	5.18±2.33	-0.25±0.69	4	-0.787	0.213	1.17
CA-SF2	54.2539	-105.8775	ENF	2001-2005	5.52±3.13	-0.46±0.73	5	0.366	0.545	1.36
CA-SF3	54.0916	-106.0053	OSH	2002-2006	2.39±1.00	0.09±0.25	5	-0.382	0.526	0.69
CA-TP1	42.6609	-80.5595	ENF	2003-2014	1.92±1.66	0.67±0.82	12	-0.946	0.000	0.89
CA-TP2	42.7744	-80.4588	ENF	2003-2007	3.84±3.29	-0.31±0.78	5	-0.556	0.330	1.03
CA-TP3	42.7068	-80.3483	ENF	2003-2014	2.69±2.34	0.46±0.68	12	-0.734	0.007	0.91
CA-TP4	42.7102	-80.3574	ENF	2002-2014	3.12±2.68	0.33±0.89	13	-0.649	0.017	0.97
CA-TPD	42.6353	-80.5577	DBF	2012-2014	2.64±2.19	-0.05±0.38	3	-0.911	0.271	0.67
CG-Tch	-4.2892	11.6564	SAV	2006-2009	2.62±1.72	0.03±0.34	4	-0.717	0.283	0.55
CH-Cha	47.2102	8.4104	GRA	2005-2014	5.61±3.72	-0.54±1.29	10	0.689	0.027	1.73

CH-Dav	46.8153	9.8559	ENF	1999-2014	2.33±1.71	-0.37±0.82	16	0.792	0.000	0.85
CH-Fru	47.1158	8.5378	GRA	2005-2014	3.93±3.08	-0.17±0.49	10	-0.190	0.598	0.94
CH-Lae	47.4781	8.365	MF	2004-2014	2.54±1.57	0.17±0.74	11	0.121	0.722	1.13
CH-Oe1	47.2858	7.7319	GRA	2002-2008	3.58±2.40	-0.45±0.57	7	0.894	0.007	0.88
CH-Oe2	47.2863	7.7343	CRO	2004-2014	3.29±2.19	-0.21±0.94	11	0.243	0.472	0.97
CN-Cha	42.4025	128.0958	MF	2003-2005	3.08±2.94	-0.18±0.45	3	0.895	0.294	0.74
CN-Cng	44.5934	123.5092	GRA	2007-2010	1.20±1.21	0.03±0.26	4	0.623	0.377	0.41
CN-Dan	30.4978	91.0664	GRA	2004-2005	0.62±0.47	-0.01±0.10	2			0.19
CN-Din	23.1733	112.5361	EBF	2003-2005	2.46±1.01	0.29±0.33	3	-0.979	0.132	1.11
CN-Du2	42.0467	116.2836	GRA	2007-2008	0.54±0.87	0.05±0.11	2			0.25
CN-Du3	42.0551	116.2809	GRA	2009-2010	0.17±0.35	0.12±0.13	2			0.21
CN-Ha2	37.6086	101.3269	WET	2003-2005	2.06±1.75	-0.01±0.20	3	-0.996	0.053	0.45
CN-HaM	37.37	101.18	GRA	2002-2004	1.06±1.14	-0.03±0.35	3	0.915	0.264	0.45
CN-Qia	26.7414	115.0581	ENF	2003-2005	2.66±1.52	0.14±0.22	3	0.659	0.542	0.76
CN-Sw2	41.7902	111.8971	GRA	2010-2012	0.29±0.45	-0.13±0.29	3	-0.855	0.347	0.13
CZ-BK1	49.5021	18.5369	ENF	2004-2014	2.64±1.37	0.14±0.61	11	-0.397	0.227	1.09
CZ-BK2	49.4944	18.5429	GRA	2006-2012	2.99±1.86	0.27±0.40	7	-0.709	0.074	0.85
CZ-wet	49.0247	14.7704	WET	2006-2014	2.63±2.14	0.18±0.55	9	-0.153	0.695	0.96
DE-Akm	53.8662	13.6834	WET	2009-2014	3.50±2.03	-0.16±0.52	6	-0.459	0.360	1.03
DE-Geb	51.1001	10.9143	CRO	2001-2014	2.41±2.02	0.18±0.75	14	0.270	0.351	1.00
DE-Gri	50.95	13.5126	GRA	2004-2014	3.96±2.79	0.13±0.86	11	-0.574	0.065	1.04
DE-Hai	51.0792	10.453	DBF	2000-2012	2.88±1.46	0.01±0.57	13	0.302	0.317	0.98
DE-Kli	50.8931	13.5224	CRO	2004-2014	3.02±2.13	-0.28±0.79	11	0.358	0.279	1.04
DE-Lkb	49.0996	13.3047	ENF	2009-2013	2.11±1.10	0.18±0.30	5	-0.206	0.740	0.73
DE-Lnf	51.3282	10.3678	DBF	2002-2012	2.91±1.67	-0.14±0.52	8	0.814	0.014	0.85
DE-Obe	50.7867	13.7213	ENF	2008-2014	3.35±2.23	0.02±0.72	7	-0.064	0.892	1.08
DE-RuR	50.6219	6.3041	GRA	2011-2014	4.65±2.65	-0.07±0.35	4	0.094	0.906	0.89
DE-RuS	50.8659	6.4472	CRO	2011-2014	3.36±1.83	-0.02±0.38	4	-0.176	0.824	0.87

DE-Seh	50.8706	6.4497	CRO	2007-2010	2.66±1.78	0.07±0.46	4	0.993	0.007	0.83
DE-SfN	47.8064	11.3275	WET	2012-2014	1.48±1.11	0.37±0.41	3	0.976	0.140	0.82
DE-Spw	51.8923	14.0337	WET	2010-2014	3.27±2.13	-0.23±0.52	5	0.626	0.258	0.75
DE-Tha	50.9624	13.5652	ENF	1999-2014	2.99±2.13	-0.09±0.55	16	-0.275	0.303	0.91
DE-Zrk	53.8759	12.889	WET	2013-2014	2.10±1.59	0.30±0.34	2			0.87
DK-Eng	55.6905	12.1918	GRA	2005-2008	2.03±1.30	0.23±0.36	4	0.925	0.075	0.81
DK-Fou	56.4842	9.5872	CRO	2005	2.77±2.19	0.40±0.90	1			1.26
DK-NuF	64.1308	-51.3861	WET	2008-2014	1.35±0.68	0.01±0.16	7	-0.545	0.206	0.40
DK-Sor	55.4859	11.6446	DBF	1999-2014	4.92±3.01	-0.70±0.72	16	0.808	0.000	1.17
DK-ZaF	74.4814	-20.5545	WET	2008-2011	0.75±0.18	-0.46±0.10	4	0.697	0.303	0.18
DK-ZaH	74.4733	-20.5503	GRA	2000-2014	0.61±0.24	0.01±0.12	15	0.392	0.149	0.17
ES-Amo	36.8336	-2.2523	OSH	2007-2012	0.48±0.35	-0.00±0.29	6	0.374	0.465	0.32
ES-LgS	37.0979	-2.9658	OSH	2007-2009	0.97±0.57	0.11±0.20	3	-0.726	0.483	0.52
ES-LJu	36.9266	-2.7521	OSH	2004-2013	0.30±0.25	0.12±0.19	10	-0.877	0.001	0.33
ES-Ln2	36.9695	-3.4758	OSH	2009	0.33±0.20	0.14±0.13	1			0.44
FI-Hyy	61.8474	24.2948	ENF	1999-2014	2.48±2.00	0.04±0.40	16	-0.036	0.896	0.80
FI-Jok	60.8986	23.5135	CRO	2000-2003	1.19±1.33	0.10±0.22	4	-0.830	0.170	0.48
FI-Let	60.6418	23.9595	ENF	2009-2012	2.91±2.71	-0.20±0.50	4	-0.864	0.136	0.76
FI-Lom	67.9972	24.2092	WET	2007-2009	1.03±1.16	0.08±0.17	3	-0.775	0.435	0.40
FI-Sod	67.3624	26.6386	ENF	2001-2014	1.86±1.73	-0.24±0.55	14	0.608	0.021	0.62
FR-Fon	48.4764	2.7801	DBF	2005-2014	3.20±1.97	0.20±0.75	10	-0.809	0.005	1.05
FR-Gri	48.8442	1.9519	CRO	2004-2014	2.86±1.98	0.20±0.78	11	-0.287	0.392	1.07
FR-LBr	44.7171	-0.7693	ENF	1999-2008	4.00±1.65	-0.03±0.70	10	0.033	0.928	1.23
FR-Pue	43.7413	3.5957	EBF	2000-2014	2.66±1.18	-0.28±0.66	15	0.676	0.006	1.13
GF-Guy	5.2788	-52.9249	EBF	2004-2014	9.53±1.47	-0.07±1.26	11	0.071	0.835	1.30
GH-Ank	5.2685	-2.6942	EBF	2011-2014	4.88±1.23	0.14±0.66	3	0.714	0.493	1.41
IT-BCi	40.5238	14.9574	CRO	2004-2014	4.38±2.53	0.34±1.45	11	-0.694	0.018	1.53
IT-CA1	42.3804	12.0266	DBF	2011-2014	2.58±1.33	0.01±0.29	4	0.688	0.312	0.65

IT-CA2	42.3772	12.026	CRO	2011-2014	2.14±1.02	0.20±0.32	4	0.176	0.824	0.82
IT-CA3	42.38	12.0222	DBF	2011-2014	1.81±0.74	0.23±0.22	4	0.529	0.471	0.68
IT-Col	41.8494	13.5881	DBF	1999-2014	1.98±0.99	0.60±0.55	16	-0.631	0.009	0.93
IT-Cp2	41.7043	12.3573	EBF	2012-2014	3.67±1.39	0.16±0.52	3	-0.772	0.439	1.12
IT-Cpz	41.7053	12.3761	EBF	2000-2008	3.85±1.46	-0.19±0.73	9	0.300	0.432	1.15
IT-Isp	45.8126	8.6336	DBF	2013-2014	3.85±1.78	-0.52±0.20	2			0.96
IT-La2	45.9542	11.2853	ENF	2000-2002	2.05±1.70	-0.05±0.41	3	0.309	0.800	0.77
IT-Lav	45.9562	11.2813	ENF	2003-2014	1.09±1.00	0.38±0.45	12	-0.216	0.500	1.12
IT-MBo	46.0147	11.0458	GRA	2003-2013	3.74±3.04	-0.36±0.74	11	0.005	0.988	1.11
IT-PT1	45.2009	9.061	DBF	2002-2004	3.39±1.91	0.06±0.42	3	-0.129	0.918	0.85
IT-Ren	46.5869	11.4337	ENF	1999-2013	1.56±1.10	0.17±0.39	13	-0.649	0.016	0.63
IT-Ro1	42.4081	11.93	DBF	2000-2008	3.37±1.47	-0.52±0.69	9	0.803	0.009	0.94
IT-Ro2	42.3903	11.9209	DBF	2002-2012	1.96±1.07	0.30±0.70	10	-0.072	0.843	1.04
IT-SR2	43.732	10.291	ENF	2013-2014	5.30±2.62	-0.05±0.61	2			1.21
IT-SRo	43.7279	10.2844	ENF	1999-2012	4.74±1.81	0.63±1.13	14	-0.649	0.012	1.66
IT-Tor	45.8444	7.5781	GRA	2008-2014	2.48±1.30	-0.10±0.49	7	0.520	0.231	0.72
JP-MBF	44.3869	142.3186	DBF	2004-2005	1.90±1.37	0.11±0.43	2			0.86
JP-SMF	35.2617	137.0788	MF	2002-2006	3.15±1.07	-0.12±0.43	5	0.223	0.718	0.81
MY-PSO	2.973	102.3062	EBF	2003-2009	4.02±0.82	0.07±0.56	7	-0.511	0.241	0.62
NL-Hor	52.2404	5.0713	GRA	2004-2011	3.10±2.14	0.15±0.55	8	-0.209	0.620	0.90
NL-Loo	52.1666	5.7436	ENF	1999-2014	3.12±1.78	-0.26±0.53	16	0.844	0.000	0.91
NO-Adv	78.186	15.923	WET	2012-2013	0.73±0.40	-0.42±0.29	2			0.23
NO-Blv	78.9216	11.8311	SNO	2008-2009	0.08±0.08	0.16±0.11	2			0.19
PA-SPn	9.3181	-79.6346	DBF	2007-2009	4.12±1.55	0.23±0.66	3	-0.305	0.803	1.14
PA-SPs	9.3138	-79.6314	GRA	2007-2009	7.46±2.02	-0.20±0.82	3	0.705	0.502	1.24
RU-Che	68.613	161.3414	WET	2002-2005	1.08±0.80	0.07±0.15	4	0.991	0.009	0.38
RU-Cok	70.8291	147.4943	OSH	2003-2013	1.49±0.62	0.06±0.19	11	0.122	0.722	0.50
RU-Fyo	56.4615	32.9221	ENF	1999-2014	4.20±3.57	-1.12±1.44	16	0.650	0.006	1.03

RU-Ha1	54.7252	90.0022	GRA	2002-2004	1.73±1.29	0.03±0.16	3	-0.722	0.486	0.46
SD-Dem	13.2829	30.4783	SAV	2005-2009	1.68±0.97	-0.10±0.20	4	0.912	0.088	0.39
SN-Dhr	15.4028	-15.4322	SAV	2010-2013	4.21±1.93	-0.18±0.29	4	0.823	0.178	0.88
US-AR1	36.4267	-99.42	GRA	2009-2012	1.70±1.25	-0.09±0.51	4	0.689	0.311	0.52
US-AR2	36.6358	-99.5975	GRA	2009-2012	1.00±0.74	0.03±0.21	4	0.747	0.253	0.38
US-ARb	35.5497	-98.0402	GRA	2005-2006	2.83±1.99	-0.03±0.37	2			0.76
US-ARc	35.5465	-98.04	GRA	2005-2006	3.32±1.95	0.01±0.38	2			0.77
US-ARM	36.6058	-97.4888	CRO	2003-2012	1.64±1.41	0.32±0.54	10	-0.764	0.010	0.77
US-Atq	70.4696	-157.4089	WET	2003-2008	0.56±0.61	0.00±0.19	6	-0.212	0.687	0.26
US-Blo	38.8953	-120.6328	ENF	1999-2006	2.47±1.31	0.10±0.51	8	-0.403	0.322	1.18
US-Cop	38.09	-109.39	GRA	2001-2007	0.19±0.16	0.03±0.08	5	-0.223	0.718	0.14
US-CRT	41.6285	-83.3471	CRO	2011-2013	1.90±1.86	0.13±0.44	3	0.969	0.159	0.74
US-GBT	41.3658	-106.2397	ENF	2001-2003	0.35±0.68	0.12±0.20	3	0.319	0.794	0.43
US-GLE	41.3665	-106.2399	ENF	2005-2014	1.50±1.24	0.06±0.45	10	-0.452	0.189	0.70
US-Goo	34.2547	-89.8735	GRA	2002-2006	3.03±2.39	0.16±0.42	5	-0.675	0.211	0.92
US-Ha1	42.5378	-72.1715	DBF	1999-2012	2.68±1.61	-0.11±0.58	14	-0.284	0.325	0.82
US-IB2	41.8406	-88.241	GRA	2004-2011	2.19±2.05	0.08±0.39	8	0.055	0.897	0.65
US-Ivo	68.4865	-155.7503	WET	2004-2007	0.87±0.87	0.03±0.25	4	-0.847	0.153	0.40
US-KS1	28.4583	-80.6709	ENF	2002	4.32±0.99	0.23±0.28	1			1.08
US-KS2	28.6086	-80.6715	CSH	2003-2006	4.34±1.42	0.02±0.57	4	0.961	0.039	1.00
US-Lin	36.3566	-119.8423	CRO	2009-2010	0.32±0.24	0.50±0.23	2			0.94
US-Los	46.0827	-89.9792	WET	2000-2014	2.28±1.43	0.11±0.29	11	-0.577	0.063	0.71
US-Me1	44.5794	-121.5	ENF	2004-2005	0.97±0.85	-0.05±0.22	2			0.38
US-Me2	44.4523	-121.5574	ENF	2002-2014	3.06±2.55	0.49±0.81	13	-0.645	0.017	1.44
US-Me3	44.3154	-121.6078	ENF	2004-2009	2.16±1.27	-0.10±0.42	6	0.697	0.124	0.65
US-Me4	44.4992	-121.6224	ENF	1999-2000	2.18±1.28	0.15±0.37	2			1.00
US-Me5	44.4372	-121.5668	ENF	2000-2002	1.89±1.16	0.07±0.27	3	-0.972	0.150	0.65
US-Me6	44.3233	-121.6078	ENF	2010-2014	1.83±1.28	0.02±0.31	5	-0.022	0.971	0.60

US-MMS	39.3232	-86.4131	DBF	1999-2014	3.00±2.33	0.03±0.79	16	-0.057	0.834	0.98
US-Myb	38.0498	-121.7651	WET	2011-2014	4.14±2.04	0.04±0.75	4	-0.356	0.644	1.45
US-Ne1	41.1651	-96.4766	CRO	2001-2013	2.36±2.44	-0.19±0.67	13	-0.070	0.820	0.62
US-Ne2	41.1649	-96.4701	CRO	2001-2013	2.63±2.45	0.13±0.78	13	0.408	0.166	0.82
US-Ne3	41.1797	-96.4397	CRO	2001-2013	2.29±2.25	-0.06±0.66	13	0.565	0.044	0.75
US-NR1	40.0329	-105.5464	ENF	1999-2014	1.60±1.45	0.10±0.31	16	0.100	0.714	0.74
US-Oho	41.5545	-83.8438	DBF	2004-2013	2.25±2.02	0.18±0.48	10	-0.612	0.060	0.79
US-ORv	40.0201	-83.0183	WET	2011	1.02±0.48	0.21±0.23	1			0.58
US-PFa	45.9459	-90.2723	MF	1999-2014	1.84±2.13	0.02±0.72	16	0.618	0.011	0.77
US-Prr	65.1237	-147.4876	ENF	2010-2014	0.56±0.68	0.13±0.18	5	0.682	0.205	0.34
US-SRC	31.9083	-110.8395	OSH	2008-2014	0.29±0.35	-0.01±0.11	7	0.594	0.160	0.17
US-SRG	31.7894	-110.8277	GRA	2008-2014	1.15±1.10	-0.10±0.36	7	0.658	0.108	0.40
US-SRM	31.8214	-110.8661	WSA	2004-2014	0.78±0.71	-0.03±0.30	11	0.574	0.065	0.29
US-Sta	41.3966	-106.8024	OSH	2005-2009	0.11±0.17	0.02±0.06	5	-0.027	0.965	0.11
US-Syv	46.242	-89.3477	MF	2001-2014	3.36±2.90	0.21±0.74	10	-0.764	0.010	1.01
US-Ton	38.4316	-120.966	WSA	2001-2014	1.86±1.21	-0.08±0.66	14	-0.464	0.094	0.74
US-Tw1	38.1074	-121.6469	WET	2012-2014	5.17±1.13	-0.06±0.68	3	-0.901	0.285	1.34
US-Tw2	38.1047	-121.6433	CRO	2012-2013	4.05±2.37	-0.15±0.55	2			1.10
US-Tw3	38.1159	-121.6467	CRO	2013-2014	7.16±1.82	-0.45±0.45	2			1.41
US-Tw4	38.103	-121.6414	WET	2013-2014	2.14±1.00	0.44±0.43	2			0.76
US-Twt	38.1087	-121.653	CRO	2009-2014	4.71±2.27	0.40±1.17	6	-0.889	0.018	1.65
US-UMB	45.5598	-84.7138	DBF	2000-2014	1.95±1.76	0.03±0.36	15	-0.405	0.134	0.51
US-UMd	45.5625	-84.6975	DBF	2007-2014	2.65±1.98	0.17±0.39	8	-0.859	0.006	0.61
US-Var	38.4133	-120.9507	GRA	2000-2014	1.93±1.75	-0.17±0.84	15	0.484	0.067	0.88
US-WCr	45.8059	-90.0799	DBF	1999-2014	1.89±1.75	0.00±0.72	13	0.274	0.366	0.71
US-Whs	31.7438	-110.0522	OSH	2007-2014	0.41±0.52	0.06±0.14	8	-0.778	0.023	0.21
US-Wi0	46.6188	-91.0814	ENF	2002	2.54±2.15	0.19±0.38	1			0.90
US-Wi1	46.7305	-91.2329	DBF	2003	6.35±3.09	-1.04±1.28	1			1.64

US-Wi2	46.6869	-91.1528	ENF	2003	0.45±0.64	0.36±0.31	1			0.59
US-Wi3	46.6347	-91.0987	DBF	2002-2004	1.69±1.17	0.52±0.48	2			1.08
US-Wi4	46.7393	-91.1663	ENF	2002-2005	2.78±1.12	0.07±0.38	4	0.069	0.931	0.90
US-Wi5	46.6531	-91.0858	ENF	2004	2.33±1.63	0.42±0.44	1			1.15
US-Wi6	46.6249	-91.2982	OSH	2002	1.02±0.89	1.01±0.66	1			1.45
US-Wi7	46.6491	-91.0693	OSH	2005	2.37±1.72	0.74±0.54	1			1.35
US-Wi8	46.7223	-91.2524	DBF	2002	4.40±2.58	-0.01±0.63	1			1.19
US-Wi9	46.6188	-91.0814	ENF	2004-2005	1.72±1.21	0.51±0.48	2			1.28
US-Wkg	31.7365	-109.9419	GRA	2004-2014	0.48±0.51	0.09±0.19	11	-0.633	0.037	0.25
US-WPT	41.4646	-82.9962	WET	2011-2013	2.04±1.91	0.01±0.38	3	0.960	0.180	0.55
ZM-Mon	-15.4378	23.2528	DBF	2000-2009	4.74±1.90	0.03±0.40	4	-0.973	0.027	0.97

Table S3. Statistic summary of site NEE fitting.

Site ID	Latitude	Longitude	IGBP	Year	Observed Flux (gC m-2 d-1)	Prediction Bias (gC m-2 d-1)	ND	R	p-value	Model SD （gC m-2 d-1)
AR-SLu	-33.4648	-66.4598	MF	2009-2011	-4.80±1.05	0.87±0.35	3	-0.851	0.351	1.48
AR-Vir	-28.2395	-56.1886	ENF	2010-2012	1.56±2.95	-0.99±1.59	3	-0.891	0.300	2.66
AT-Neu	47.1167	11.3175	GRA	2002-2012	1.01±2.06	-0.38±1.43	11	0.212	0.532	1.31
AU-Ade	-13.0769	131.1178	WSA	2007-2009	-1.33±0.99	0.13±0.27	3	0.935	0.231	0.84
AU-ASM	-22.283	133.249	ENF	2010-2014	-0.06±0.41	0.05±0.22	5	-0.702	0.187	0.34
AU-Cpr	-34.0021	140.5891	SAV	2010-2014	-0.29±0.35	0.06±0.19	5	-0.967	0.007	0.45
AU-Cum	-33.6152	150.7236	EBF	2012-2014	-0.65±0.74	0.08±0.34	3	-0.941	0.219	0.89
AU-DaP	-14.0633	131.3181	GRA	2007-2013	-0.48±1.79	0.11±0.68	7	-0.461	0.298	1.05
AU-DaS	-14.1593	131.3881	SAV	2008-2014	-0.69±1.04	-0.01±0.54	7	0.391	0.386	0.83
AU-Dry	-15.2588	132.3706	SAV	2008-2014	-0.67±0.77	-0.06±0.33	7	0.668	0.101	0.66
AU-Emr	-23.8587	148.4746	GRA	2011-2013	0.06±0.87	-0.04±0.34	3	-0.724	0.484	0.67
AU-Fog	-12.5452	131.3072	WET	2006-2008	-1.41±0.88	0.02±0.26	3	0.987	0.103	0.81
AU-Gin	-31.3764	115.7138	WSA	2011-2014	-0.80±0.59	-0.01±0.23	4	0.470	0.530	0.64
AU-GWW	-30.1913	120.6541	SAV	2013-2014	-0.34±0.29	0.02±0.14	2			0.31
AU-How	-12.4943	131.1523	WSA	2001-2014	-1.68±1.23	0.04±0.64	14	0.120	0.682	1.04
AU-Lox	-34.4704	140.6551	DBF	2008-2009	-3.31±2.52	0.58±0.71	2			1.45
AU-RDF	-14.5636	132.4776	WSA	2011-2013	0.29±0.70	-0.15±0.38	3	-0.467	0.690	0.84
AU-Rig	-36.6499	145.5759	GRA	2011-2014	-0.26±1.58	0.05±0.42	4	-0.358	0.642	0.86
AU-Rob	-17.1175	145.6301	EBF	2014	-2.10±1.11	0.07±0.70	1			1.19
AU-Stp	-17.1507	133.3502	GRA	2008-2014	-0.09±0.87	0.18±0.35	7	-0.866	0.012	0.52
AU-TTE	-22.287	133.64	OSH	2012-2014	0.45±0.32	-0.01±0.11	3	0.298	0.807	0.24
AU-Tum	-35.6566	148.1517	EBF	2001-2014	-1.63±1.95	-0.83±1.68	14	0.639	0.014	1.81
AU-Wac	-37.4259	145.1878	EBF	2005-2008	-4.71±1.97	0.19±1.08	4	0.722	0.278	1.51
AU-Whr	-36.6732	145.0294	EBF	2011-2014	-1.35±0.78	0.27±0.38	4	-0.637	0.363	0.71
AU-Wom	-37.4222	144.0944	EBF	2010-2014	-3.00±1.51	0.00±0.78	5	0.419	0.482	1.24

AU-Ync	-34.9893	146.2907	GRA	2012-2014	0.23±0.49	0.02±0.15	3	-0.940	0.222	0.37
BE-Bra	51.3076	4.5198	MF	1999-2014	-0.04±1.87	-0.17±0.64	15	0.608	0.016	1.19
BE-Lon	50.5516	4.7461	CRO	2004-2014	-0.97±3.58	0.34±2.38	11	-0.561	0.073	1.58
BE-Vie	50.305	5.9981	MF	1999-2014	-0.66±1.84	0.43±0.68	16	-0.597	0.015	1.02
BR-Sa1	-2.8567	-54.9589	EBF	2002-2011	-0.14±1.18	0.08±0.55	9	0.115	0.769	0.73
BR-Sa3	-3.018	-54.9714	EBF	2000-2004	-0.41±1.07	0.07±0.47	5	0.040	0.950	0.75
CA-Gro	48.2167	-82.1556	MF	2003-2014	-0.14±1.01	-0.16±0.43	12	0.025	0.938	0.89
CA-Man	55.8796	-98.4808	ENF	1999-2008	0.01±0.67	-0.05±0.22	9	0.758	0.018	0.47
CA-NS1	55.8792	-98.4839	ENF	2002-2005	-0.37±1.15	0.01±0.24	4	-0.768	0.232	0.43
CA-NS2	55.9058	-98.5247	ENF	2001-2005	-0.66±0.97	0.11±0.20	5	-0.966	0.007	0.49
CA-NS3	55.9117	-98.3822	ENF	2001-2005	-0.09±0.84	-0.02±0.29	5	0.025	0.968	0.41
CA-NS4	55.9144	-98.3806	ENF	2002-2005	-0.10±0.47	-0.04±0.22	4	-0.177	0.823	0.34
CA-NS5	55.8631	-98.485	ENF	2001-2005	-0.25±0.98	-0.04±0.33	5	0.148	0.812	0.55
CA-NS6	55.9167	-98.9644	OSH	2001-2005	0.03±0.66	0.06±0.20	5	-0.972	0.006	0.46
CA-NS7	56.6358	-99.9483	OSH	2002-2005	0.25±0.69	-0.06±0.18	4	0.193	0.807	0.36
CA-Oas	53.6289	-106.1978	DBF	1999-2010	-0.12±2.07	-0.23±0.64	12	0.163	0.612	0.87
CA-Obs	53.9872	-105.1178	ENF	1999-2010	-0.04±0.82	-0.04±0.40	12	-0.539	0.070	0.71
CA-Qfo	49.6925	-74.3421	ENF	2003-2010	0.02±0.63	-0.10±0.26	8	-0.149	0.725	0.62
CA-SF1	54.485	-105.8176	ENF	2003-2006	-0.24±1.47	-0.25±0.54	4	0.564	0.436	1.05
CA-SF2	54.2539	-105.8775	ENF	2001-2005	-0.71±1.44	-0.16±0.57	5	0.522	0.366	1.20
CA-SF3	54.0916	-106.0053	OSH	2002-2006	-0.10±0.51	-0.00±0.20	5	-0.955	0.012	0.72
CA-TP1	42.6609	-80.5595	ENF	2003-2014	-0.01±0.77	-0.25±0.68	12	0.589	0.044	1.00
CA-TP2	42.7744	-80.4588	ENF	2003-2007	-2.03±2.30	0.30±0.80	5	0.358	0.554	1.20
CA-TP3	42.7068	-80.3483	ENF	2003-2014	-1.22±1.34	0.25±0.83	12	-0.196	0.543	1.16
CA-TP4	42.7102	-80.3574	ENF	2002-2014	-0.09±1.14	-0.12±0.57	13	0.227	0.455	0.94
CA-TPD	42.6353	-80.5577	DBF	2012-2014	-0.13±1.97	-0.13±0.37	3	0.130	0.917	0.73
CG-Tch	-4.2892	11.6564	SAV	2006-2009	-0.23±0.89	0.02±0.24	4	0.223	0.777	0.40
CH-Cha	47.2102	8.4104	GRA	2005-2014	0.09±1.81	-0.22±1.06	10	0.274	0.443	1.26

CH-Dav	46.8153	9.8559	ENF	1999-2014	-0.80±1.40	-0.47±0.86	16	0.783	0.000	1.09
CH-Fru	47.1158	8.5378	GRA	2005-2014	-0.63±2.14	-0.11±0.74	10	-0.120	0.742	1.09
CH-Lae	47.4781	8.365	MF	2004-2014	-1.97±2.42	-0.01±0.75	11	0.266	0.429	1.30
CH-Oe1	47.2858	7.7319	GRA	2002-2008	-0.50±1.65	0.24±0.84	7	-0.913	0.004	0.89
CH-Oe2	47.2863	7.7343	CRO	2004-2014	0.12±2.55	-0.41±1.46	11	0.175	0.606	1.33
CN-Cha	42.4025	128.0958	MF	2003-2005	-0.54±1.39	0.04±0.32	3	-0.189	0.879	0.72
CN-Cng	44.5934	123.5092	GRA	2007-2010	-0.56±0.92	0.04±0.27	4	0.928	0.072	0.51
CN-Dan	30.4978	91.0664	GRA	2004-2005	-0.28±0.50	0.01±0.11	2			0.25
CN-Din	23.1733	112.5361	EBF	2003-2005	-1.56±1.04	0.06±0.38	3	-0.114	0.927	0.91
CN-Du2	42.0467	116.2836	GRA	2007-2008	-0.16±0.30	-0.02±0.21	2			0.32
CN-Du3	42.0551	116.2809	GRA	2009-2010	0.11±0.29	-0.13±0.19	2			0.29
CN-Ha2	37.6086	101.3269	WET	2003-2005	-0.53±1.04	-0.06±0.22	3	0.457	0.698	0.57
CN-HaM	37.37	101.18	GRA	2002-2004	-0.27±0.95	0.01±0.20	3	0.989	0.093	0.37
CN-Qia	26.7414	115.0581	ENF	2003-2005	-1.20±0.99	0.12±0.39	3	-0.425	0.721	0.89
CN-Sw2	41.7902	111.8971	GRA	2010-2012	-0.32±0.57	0.02±0.32	3	-0.444	0.707	0.51
CZ-BK1	49.5021	18.5369	ENF	2004-2014	-2.45±2.43	0.21±1.03	11	-0.099	0.773	1.42
CZ-BK2	49.4944	18.5429	GRA	2006-2012	0.12±1.45	-0.48±0.72	7	0.793	0.033	1.10
CZ-wet	49.0247	14.7704	WET	2006-2014	0.11±1.49	-0.03±0.71	9	-0.780	0.013	0.99
DE-Akm	53.8662	13.6834	WET	2009-2014	-0.15±2.06	0.18±0.47	6	-0.892	0.017	0.91
DE-Geb	51.1001	10.9143	CRO	2001-2014	-0.54±2.80	0.09±1.66	14	-0.008	0.979	1.16
DE-Gri	50.95	13.5126	GRA	2004-2014	-0.32±1.61	-0.04±1.10	11	-0.159	0.641	1.21
DE-Hai	51.0792	10.453	DBF	2000-2012	-1.35±3.75	0.21±1.23	13	-0.784	0.002	1.13
DE-Kli	50.8931	13.5224	CRO	2004-2014	-0.30±2.55	-0.13±1.26	11	0.271	0.420	1.20
DE-Lkb	49.0996	13.3047	ENF	2009-2013	0.75±0.68	-0.17±0.31	5	0.828	0.084	0.72
DE-Lnf	51.3282	10.3678	DBF	2002-2012	-1.36±3.52	0.13±0.96	8	-0.097	0.819	1.14
DE-Obe	50.7867	13.7213	ENF	2008-2014	-0.54±2.00	0.00±0.54	7	0.862	0.013	0.95
DE-RuR	50.6219	6.3041	GRA	2011-2014	-0.29±1.46	-0.10±0.54	4	-0.070	0.930	0.93
DE-RuS	50.8659	6.4472	CRO	2011-2014	-2.80±3.63	0.44±1.22	4	-0.450	0.550	1.85

DE-Seh	50.8706	6.4497	CRO	2007-2010	-1.70±3.32	0.65±1.62	4	-0.605	0.395	1.52
DE-SfN	47.8064	11.3275	WET	2012-2014	-0.58±0.72	-0.02±0.28	3	0.997	0.053	0.73
DE-Spw	51.8923	14.0337	WET	2010-2014	-0.61±2.07	-0.19±0.60	5	0.510	0.379	0.83
DE-Tha	50.9624	13.5652	ENF	1999-2014	-1.17±1.80	0.36±0.73	16	-0.676	0.004	1.04
DE-Zrk	53.8759	12.889	WET	2013-2014	0.40±0.53	-0.23±0.49	2			0.85
DK-Eng	55.6905	12.1918	GRA	2005-2008	-0.30±0.95	-0.08±0.64	4	0.675	0.325	1.00
DK-Fou	56.4842	9.5872	CRO	2005	-0.89±1.36	0.02±0.44	1			0.97
DK-NuF	64.1308	-51.3861	WET	2008-2014	-0.17±0.73	-0.01±0.18	7	-0.844	0.017	0.43
DK-Sor	55.4859	11.6446	DBF	1999-2014	-0.49±2.83	-0.10±0.83	16	0.702	0.002	1.16
DK-ZaF	74.4814	-20.5545	WET	2008-2011	0.50±0.11	-0.59±0.09	4	0.349	0.651	0.45
DK-ZaH	74.4733	-20.5503	GRA	2000-2014	-0.08±0.38	-0.02±0.18	15	-0.023	0.934	0.21
ES-Amo	36.8336	-2.2523	OSH	2007-2012	0.62±0.71	-0.18±0.29	6	0.526	0.284	0.41
ES-LgS	37.0979	-2.9658	OSH	2007-2009	-0.33±0.70	-0.04±0.20	3	0.501	0.666	0.59
ES-LJu	36.9266	-2.7521	OSH	2004-2013	0.02±0.45	0.01±0.24	10	-0.309	0.385	0.48
ES-Ln2	36.9695	-3.4758	OSH	2009	0.50±0.27	-0.18±0.27	1			0.54
FI-Hyy	61.8474	24.2948	ENF	1999-2014	-0.56±1.47	0.02±0.42	16	0.562	0.023	0.83
FI-Jok	60.8986	23.5135	CRO	2000-2003	0.32±0.67	-0.17±0.38	4	0.725	0.275	0.67
FI-Let	60.6418	23.9595	ENF	2009-2012	0.54±1.41	-0.14±0.36	4	0.058	0.942	0.66
FI-Lom	67.9972	24.2092	WET	2007-2009	-0.03±1.03	-0.03±0.24	3	0.404	0.735	0.47
FI-Sod	67.3624	26.6386	ENF	2001-2014	0.28±0.87	-0.06±0.43	14	0.045	0.879	0.60
FR-Fon	48.4764	2.7801	DBF	2005-2014	-1.53±3.29	0.24±1.00	10	-0.920	0.000	1.17
FR-Gri	48.8442	1.9519	CRO	2004-2014	-1.28±3.30	0.37±1.76	11	-0.163	0.631	1.49
FR-LBr	44.7171	-0.7693	ENF	1999-2008	-0.78±1.55	-0.04±0.72	10	0.293	0.411	1.16
FR-Pue	43.7413	3.5957	EBF	2000-2014	-0.58±1.06	0.02±0.71	15	-0.076	0.788	0.94
GF-Guy	5.2788	-52.9249	EBF	2004-2014	-0.52±0.97	0.10±0.83	11	-0.019	0.955	0.72
GH-Ank	5.2685	-2.6942	EBF	2011-2014	-2.01±0.50	0.09±0.33	3	-0.691	0.514	0.84
IT-BCi	40.5238	14.9574	CRO	2004-2014	-0.81±3.37	0.30±2.54	11	-0.408	0.213	1.87
IT-CA1	42.3804	12.0266	DBF	2011-2014	-0.63±1.54	-0.04±0.47	4	-0.679	0.321	0.89

IT-CA2	42.3772	12.026	CRO	2011-2014	-0.32±1.96	-0.05±0.75	4	-0.451	0.549	1.18
IT-CA3	42.38	12.0222	DBF	2011-2014	-0.56±1.09	0.10±0.40	4	0.915	0.085	0.80
IT-Col	41.8494	13.5881	DBF	1999-2014	-1.66±2.99	0.27±0.63	16	-0.690	0.003	1.13
IT-Cp2	41.7043	12.3573	EBF	2012-2014	-1.79±1.10	-0.00±0.45	3	-0.969	0.160	1.06
IT-Cpz	41.7053	12.3761	EBF	2000-2008	-1.45±1.18	-0.09±0.70	9	0.432	0.246	1.18
IT-Isp	45.8126	8.6336	DBF	2013-2014	0.52±2.70	-0.12±0.58	2			1.02
IT-La2	45.9542	11.2853	ENF	2000-2002	-1.83±1.99	0.06±0.47	3	-0.154	0.901	1.06
IT-Lav	45.9562	11.2813	ENF	2003-2014	-5.01±2.94	0.87±1.25	12	-0.048	0.882	1.80
IT-MBo	46.0147	11.0458	GRA	2003-2013	-0.13±1.78	-0.19±0.90	11	0.175	0.607	1.03
IT-PT1	45.2009	9.061	DBF	2002-2004	-1.95±3.10	0.17±0.49	3	-0.870	0.329	1.01
IT-Ren	46.5869	11.4337	ENF	1999-2013	-1.22±2.04	0.25±0.53	13	0.019	0.951	0.86
IT-Ro1	42.4081	11.93	DBF	2000-2008	-0.38±2.13	-0.48±0.87	9	0.669	0.049	1.34
IT-Ro2	42.3903	11.9209	DBF	2002-2012	-1.65±3.11	0.20±1.19	10	-0.316	0.373	1.43
IT-SR2	43.732	10.291	ENF	2013-2014	-0.97±1.44	0.01±0.47	2			0.95
IT-SRo	43.7279	10.2844	ENF	1999-2012	-0.99±1.47	0.26±0.95	14	-0.385	0.174	1.25
IT-Tor	45.8444	7.5781	GRA	2008-2014	-0.28±2.13	0.03±0.66	7	-0.582	0.171	0.93
JP-MBF	44.3869	142.3186	DBF	2004-2005	-1.08±2.25	-0.27±0.64	2			1.22
JP-SMF	35.2617	137.0788	MF	2002-2006	0.82±0.91	-0.26±0.47	5	-0.607	0.278	0.90
MY-PSO	2.973	102.3062	EBF	2003-2009	-2.73±0.70	0.02±0.49	7	-0.054	0.909	0.49
NL-Hor	52.2404	5.0713	GRA	2004-2011	-0.42±1.38	-0.03±0.46	8	0.108	0.798	0.87
NL-Loo	52.1666	5.7436	ENF	1999-2014	-1.08±1.35	0.33±0.61	16	0.327	0.216	1.03
NO-Adv	78.186	15.923	WET	2012-2013	-0.04±0.44	-0.01±0.41	2			0.46
NO-Blv	78.9216	11.8311	SNO	2008-2009	0.03±0.11	-0.17±0.14	2			0.48
PA-SPn	9.3181	-79.6346	DBF	2007-2009	-0.87±0.81	0.16±0.44	3	-0.448	0.704	0.90
PA-SPs	9.3138	-79.6314	GRA	2007-2009	0.78±1.41	-0.23±0.62	3	0.998	0.040	0.96
RU-Che	68.613	161.3414	WET	2002-2005	-0.09±0.86	-0.03±0.18	4	-0.148	0.852	0.43
RU-Cok	70.8291	147.4943	OSH	2003-2013	-0.86±1.04	0.06±0.26	11	-0.478	0.137	0.59
RU-Fyo	56.4615	32.9221	ENF	1999-2014	0.42±1.18	-0.65±0.96	16	0.767	0.001	0.96

RU-Ha1	54.7252	90.0022	GRA	2002-2004	-0.38±0.62	0.03±0.17	3	0.635	0.562	0.47
SD-Dem	13.2829	30.4783	SAV	2005-2009	-1.18±0.94	0.41±0.38	4	-0.885	0.115	0.74
SN-Dhr	15.4028	-15.4322	SAV	2010-2013	-1.40±1.54	0.02±0.62	4	-0.612	0.388	1.00
US-AR1	36.4267	-99.42	GRA	2009-2012	0.25±0.98	-0.13±0.48	4	0.912	0.088	0.72
US-AR2	36.6358	-99.5975	GRA	2009-2012	-0.01±0.72	-0.01±0.29	4	0.128	0.872	0.45
US-ARb	35.5497	-98.0402	GRA	2005-2006	0.10±1.87	-0.12±0.46	2			0.84
US-ARc	35.5465	-98.04	GRA	2005-2006	-0.21±1.75	-0.11±0.37	2			0.89
US-ARM	36.6058	-97.4888	CRO	2003-2012	-0.22±1.72	0.13±0.89	10	-0.215	0.551	1.11
US-Atq	70.4696	-157.4089	WET	2003-2008	-0.09±0.62	0.00±0.30	6	0.291	0.575	0.34
US-Blo	38.8953	-120.6328	ENF	1999-2006	-0.55±0.84	-0.13±0.34	8	-0.089	0.833	0.92
US-Cop	38.09	-109.39	GRA	2001-2007	-0.10±0.26	-0.01±0.11	5	0.588	0.297	0.23
US-CRT	41.6285	-83.3471	CRO	2011-2013	0.12±2.59	-0.05±0.82	3	0.849	0.354	1.09
US-GBT	41.3658	-106.2397	ENF	2001-2003	-1.13±0.92	0.10±0.39	3	-0.630	0.566	0.65
US-GLE	41.3665	-106.2399	ENF	2005-2014	0.34±0.99	-0.40±0.69	10	0.015	0.967	0.72
US-Goo	34.2547	-89.8735	GRA	2002-2006	-0.37±0.96	-0.02±0.37	5	-0.509	0.382	0.86
US-Ha1	42.5378	-72.1715	DBF	1999-2012	0.30±2.37	-0.37±0.79	14	0.446	0.110	1.08
US-IB2	41.8406	-88.241	GRA	2004-2011	-0.30±1.74	-0.07±0.61	8	0.242	0.564	0.81
US-Ivo	68.4865	-155.7503	WET	2004-2007	-0.09±0.78	-0.02±0.19	4	0.902	0.098	0.41
US-KS1	28.4583	-80.6709	ENF	2002	-0.98±0.64	0.00±0.25	1			0.73
US-KS2	28.6086	-80.6715	CSH	2003-2006	-0.83±0.86	0.05±0.46	4	-0.310	0.690	0.81
US-Lin	36.3566	-119.8423	CRO	2009-2010	-1.02±0.31	0.20±0.23	2			0.86
US-Los	46.0827	-89.9792	WET	2000-2014	0.16±1.04	-0.36±0.42	11	0.226	0.505	0.88
US-Me1	44.5794	-121.5	ENF	2004-2005	-0.24±0.23	0.05±0.09	2			0.37
US-Me2	44.4523	-121.5574	ENF	2002-2014	-1.74±1.52	0.18±0.84	13	-0.179	0.557	1.30
US-Me3	44.3154	-121.6078	ENF	2004-2009	-0.25±0.65	-0.15±0.32	6	0.794	0.059	0.65
US-Me4	44.4992	-121.6224	ENF	1999-2000	-2.14±1.49	0.19±0.67	2			1.21
US-Me5	44.4372	-121.5668	ENF	2000-2002	-0.54±0.76	-0.07±0.23	3	-0.879	0.316	0.69
US-Me6	44.3233	-121.6078	ENF	2010-2014	-0.77±0.90	0.01±0.33	5	-0.486	0.406	0.65

US-MMS	39.3232	-86.4131	DBF	1999-2014	-0.46±2.65	-0.13±0.89	16	-0.225	0.403	1.06
US-Myb	38.0498	-121.7651	WET	2011-2014	-0.91±2.13	-0.07±0.71	4	0.244	0.756	1.30
US-Ne1	41.1651	-96.4766	CRO	2001-2013	0.45±2.74	0.14±1.06	13	-0.725	0.005	0.89
US-Ne2	41.1649	-96.4701	CRO	2001-2013	-0.44±3.04	0.48±1.28	13	-0.508	0.077	1.21
US-Ne3	41.1797	-96.4397	CRO	2001-2013	-0.41±2.88	0.45±1.48	13	-0.511	0.074	1.11
US-NR1	40.0329	-105.5464	ENF	1999-2014	-0.39±1.12	-0.22±0.44	16	0.336	0.203	0.92
US-Oho	41.5545	-83.8438	DBF	2004-2013	-1.75±3.34	0.34±0.95	10	-0.778	0.008	1.07
US-ORv	40.0201	-83.0183	WET	2011	0.54±0.38	-0.09±0.24	1			0.62
US-PFa	45.9459	-90.2723	MF	1999-2014	0.14±0.64	-0.34±0.60	16	0.813	0.000	0.87
US-Prr	65.1237	-147.4876	ENF	2010-2014	-0.08±0.51	0.04±0.11	5	0.279	0.650	0.35
US-SRC	31.9083	-110.8395	OSH	2008-2014	-0.15±0.28	-0.00±0.15	7	0.498	0.255	0.25
US-SRG	31.7894	-110.8277	GRA	2008-2014	-0.04±0.78	-0.08±0.46	7	0.306	0.504	0.50
US-SRM	31.8214	-110.8661	WSA	2004-2014	0.08±0.53	-0.06±0.29	11	0.321	0.336	0.36
US-Sta	41.3966	-106.8024	OSH	2005-2009	-0.69±0.54	0.04±0.20	5	0.316	0.605	0.31
US-Syv	46.242	-89.3477	MF	2001-2014	-0.12±1.33	-0.32±0.63	10	0.344	0.331	1.08
US-Ton	38.4316	-120.966	WSA	2001-2014	-0.33±1.16	0.03±0.71	14	-0.194	0.507	0.84
US-Tw1	38.1074	-121.6469	WET	2012-2014	-3.58±2.99	1.26±0.97	3	-0.412	0.730	1.90
US-Tw2	38.1047	-121.6433	CRO	2012-2013	0.71±1.86	-0.26±0.60	2			1.35
US-Tw3	38.1159	-121.6467	CRO	2013-2014	-1.81±3.36	0.01±1.36	2			2.06
US-Tw4	38.103	-121.6414	WET	2013-2014	1.03±0.65	-0.50±0.76	2			0.95
US-Twt	38.1087	-121.653	CRO	2009-2014	-0.47±4.67	-0.10±2.13	6	-0.750	0.086	2.27
US-UMB	45.5598	-84.7138	DBF	2000-2014	0.10±1.72	-0.20±0.57	15	0.732	0.002	0.60
US-UMd	45.5625	-84.6975	DBF	2007-2014	-0.64±2.27	-0.00±0.56	8	0.037	0.930	0.77
US-Var	38.4133	-120.9507	GRA	2000-2014	0.10±1.27	0.01±0.63	15	-0.552	0.033	1.01
US-WCr	45.8059	-90.0799	DBF	1999-2014	-0.22±2.22	-0.20±0.75	13	0.654	0.015	0.85
US-Whs	31.7438	-110.0522	OSH	2007-2014	0.04±0.33	0.05±0.15	8	-0.155	0.715	0.25
US-Wi0	46.6188	-91.0814	ENF	2002	-2.08±1.73	-0.07±0.54	1			1.17
US-Wi1	46.7305	-91.2329	DBF	2003	1.04±1.92	-1.12±0.82	1			1.85

US-Wi2	46.6869	-91.1528	ENF	2003	-2.33±1.66	1.21±0.94	1			1.30
US-Wi3	46.6347	-91.0987	DBF	2002-2004	-3.84±3.11	0.60±0.47	2			1.29
US-Wi4	46.7393	-91.1663	ENF	2002-2005	-4.43±1.47	0.26±0.46	4	0.097	0.903	1.15
US-Wi5	46.6531	-91.0858	ENF	2004	-1.92±1.96	-0.07±0.44	1			1.21
US-Wi6	46.6249	-91.2982	OSH	2002	-1.03±1.85	0.08±0.33	1			0.96
US-Wi7	46.6491	-91.0693	OSH	2005	-0.82±1.16	-0.51±0.49	1			1.41
US-Wi8	46.7223	-91.2524	DBF	2002	0.56±0.88	-0.91±0.64	1			1.68
US-Wi9	46.6188	-91.0814	ENF	2004-2005	-1.10±0.78	-0.13±0.31	2			1.12
US-Wkg	31.7365	-109.9419	GRA	2004-2014	0.09±0.38	0.10±0.22	11	-0.576	0.064	0.28
US-WPT	41.4646	-82.9962	WET	2011-2013	0.69±0.96	-0.10±0.35	3	-0.899	0.288	0.62
ZM-Mon	-15.4378	23.2528	DBF	2000-2009	-0.02±0.74	-0.16±0.36	4	0.224	0.776	0.89

Table S4. Statistics of site GPP fitting.

Site ID	Latitude	Longitude	IGBP	Year	Observed Flux (gC m-2 d-1)	Prediction Bias (gC m-2 d-1)	ND	Model SD （gC m-2 d-1)
AR-SLu	-33.4648	-66.4598	MF	2009	8.79±0.00	-0.65±0.00	1	1.73
				2010	6.97±2.38	-0.67±0.59	32	1.75
				2011	8.67±1.25	-0.70±0.36	8	1.65
AR-Vir	-28.2395	-56.1886	ENF	2010	13.89±1.90	-2.21±0.92	7	3.02
				2011	10.40±1.98	-0.68±0.47	4	1.95
				2012	8.71±2.84	0.25±1.02	9	1.85
AT-Neu	47.1167	11.3175	GRA	2002	5.71±5.23	-0.45±1.57	35	2.16
				2003	5.73±5.54	-0.80±2.36	34	2.23
				2004	4.10±4.70	0.03±1.72	21	1.93
				2005	5.32±4.97	0.13±2.17	35	2.08
				2006	5.83±5.26	-0.07±1.86	25	2.11
				2007	6.22±5.29	-0.69±1.10	23	1.66
				2008	2.50±4.01	-0.27±0.87	19	1.01
				2009	5.82±7.73	-1.00±1.72	15	1.7
				2010	2.45±3.31	0.02±0.81	16	1.05
				2011	5.40±6.54	-0.62±1.28	13	1.54
				2012	5.66±5.43	-0.29±0.98	28	1.53
AU-Ade	-13.0769	131.1178	WSA	2007	7.06±1.77	-0.88±0.27	6	1.38
				2008	3.75±1.95	0.04±0.38	35	0.89
				2009	6.88±2.87	-0.28±0.45	14	1.25

AU-ASM	-22.283	133.249	ENF	2010	1.69±0.67	-0.35±0.42	11	0.7
				2011	1.56±1.70	-0.24±0.95	33	0.72
				2012	0.51±0.43	-0.01±0.14	35	0.29
				2013	0.21±0.16	0.03±0.07	34	0.19
				2014	0.78±0.71	-0.01±0.15	31	0.33
AU-Cpr	-34.0021	140.5891	SAV	2010	1.38±0.47	-0.39±0.26	15	0.64
				2011	2.09±0.62	-0.12±0.31	36	0.99
				2012	1.66±0.54	0.01±0.20	36	0.58
				2013	1.16±0.32	0.11±0.17	35	0.47
				2014	0.30±0.27	0.04±0.09	27	0.3
AU-Cum	-33.6152	150.7236	EBF	2012	3.67±0.51	-0.06±0.26	7	0.91
				2013	3.01±0.74	0.16±0.32	28	0.9
				2014	3.38±0.68	0.11±0.27	32	0.92
AU-DaP	-14.0633	131.3181	GRA	2007	4.72±3.39	-1.21±0.42	3	2.02
				2008	3.55±4.05	-0.26±1.48	32	1.5
				2009	2.23±3.34	-0.40±1.17	30	1.17
				2010	3.18±3.84	0.32±0.83	26	1.32
				2011	4.36±3.36	-0.26±0.54	33	1.26
				2012	2.61±3.95	0.26±0.55	19	1.11
				2013	4.25±4.68	0.15±0.65	25	1.28
AU-DaS	-14.1593	131.3881	SAV	2008	3.54±1.98	0.39±0.84	21	1.23
				2009	3.10±1.53	0.31±0.79	25	1.09
				2010	4.30±1.64	-0.33±0.66	23	1.27
				2011	4.36±1.99	-0.16±0.62	27	0.92
				2012	4.81±1.58	-0.18±0.31	16	0.93

				2013	4.81±2.46	0.01±0.57	35	1.01
				2014	4.05±1.31	0.09±0.44	30	0.96
AU-Dry	-15.2588	132.3706	SAV	2008	1.77±0.19	0.15±0.24	9	0.93
				2009	2.71±1.72	0.52±0.68	25	1.29
				2010	3.42±1.55	-0.05±0.34	27	0.91
				2011	3.53±1.19	0.14±0.34	16	0.88
				2012	3.49±1.90	-0.06±0.37	32	0.75
				2013	4.10±1.18	0.09±0.29	12	0.84
				2014	2.77±1.74	0.24±0.26	32	0.79
AU-Emr	-23.8587	148.4746	GRA	2011	1.45±0.74	0.02±0.35	16	0.69
				2012	1.51±1.26	0.18±0.48	34	0.99
				2013	1.03±0.93	0.24±0.36	34	0.86
AU-Fog	-12.5452	131.3072	WET	2006	3.08±1.45	0.80±0.51	16	1.77
				2007	1.93±1.34	0.55±0.68	23	1.24
				2008	2.03±1.28	0.31±0.21	15	1.15
AU-Gin	-31.3764	115.7138	WSA	2011	4.34±0.53	-0.18±0.23	7	1
				2012	3.35±0.98	0.09±0.23	29	0.79
				2013	2.70±1.05	0.14±0.29	30	0.7
				2014	3.18±1.17	0.06±0.28	35	0.77
AU-GWW	-30.1913	120.6541	SAV	2013	1.14±0.32	0.05±0.15	35	0.37
				2014	1.38±0.40	-0.01±0.17	31	0.47
AU-How	-12.4943	131.1523	WSA	2001	3.49±2.99	1.68±0.52	7	1.63
				2002	2.91±1.62	1.01±0.36	5	1.45

				2003	5.33±2.42	1.00±1.39	10	1.68
				2004	3.35±1.79	1.19±0.77	19	1.52
				2005	4.72±2.52	0.77±0.75	17	1.59
				2006	4.71±2.17	0.79±0.85	11	1.47
				2007	3.71±1.40	0.85±0.84	10	1.48
				2008	3.92±1.04	0.75±0.77	18	1.5
				2009	6.33±1.96	0.40±0.77	10	1.65
				2010	6.05±1.76	-0.17±0.21	11	1.12
				2011	5.51±2.49	-0.11±0.40	26	1.1
				2012	5.11±2.64	0.12±0.49	26	1.08
				2013	5.73±2.37	-0.03±0.53	25	1.17
				2014	5.77±2.11	-0.05±0.62	19	1.26
AU-Lox	-34.4704	140.6551	DBF	2008	8.74±3.47	-1.04±0.86	11	2.33
				2009	6.59±4.46	-1.06±0.80	15	1.98
AU-RDF	-14.5636	132.4776	WSA	2011	5.56±1.39	-0.30±0.32	7	1.5
				2012	0.83±1.92	0.53±0.49	14	1.24
				2013	0.22±0.37	0.44±0.18	6	1
AU-Rig	-36.6499	145.5759	GRA	2011	3.00±1.61	-0.08±0.59	32	1.08
				2012	2.55±1.96	-0.12±0.25	35	0.83
				2013	2.14±2.97	0.15±0.62	17	0.97
				2014	2.71±2.67	0.14±0.36	26	0.92
AU-Rob	-17.1175	145.6301	EBF	2014	5.11±1.15	0.86±0.42	7	1.97
AU-Stp	-17.1507	133.3502	GRA	2008	0.44±0.62	-0.40±0.42	13	0.38
				2009	0.40±0.97	0.61±0.75	27	0.81

				2010	1.05±1.65	-0.20±0.53	35	0.69
				2011	1.78±1.41	0.45±0.60	32	1.1
				2012	1.13±1.48	0.06±0.19	33	0.61
				2013	0.95±1.56	0.01±0.28	30	0.56
				2014	1.32±1.60	0.10±0.31	34	0.64
AU-TTE	-22.287	133.64	OSH	2012	-0.10±0.26	-0.04±0.14	17	0.25
				2013	-0.29±0.26	0.05±0.12	35	0.22
				2014	0.40±1.17	0.03±0.26	34	0.41
AU-Tum	-35.6566	148.1517	EBF	2001	8.94±3.59	-0.01±2.41	31	2.28
				2002	8.13±4.57	1.07±3.66	31	2.57
				2003	7.84±3.67	-0.57±3.85	34	3.09
				2004	8.91±2.83	-0.25±1.98	23	3.45
				2005	8.68±3.14	-0.74±2.07	34	3.05
				2006	8.82±2.92	0.23±1.69	35	3.07
				2007	9.16±3.51	-1.50±2.56	36	3
				2008	8.94±2.94	0.15±1.41	36	2.66
				2009	9.23±3.80	0.83±2.41	36	2.71
				2010	9.41±2.29	0.46±2.19	29	2.68
				2011	9.12±2.79	0.12±1.19	32	2.13
				2012	9.79±4.78	-0.36±1.78	22	1.89
				2013	10.45±4.53	-0.28±1.63	36	2.02
				2014	10.76±3.82	-0.43±1.02	36	1.96
AU-Wac	-37.4259	145.1878	EBF	2005	7.75±1.72	-0.24±0.81	8	1.38
				2006	5.98±1.80	0.28±0.68	25	1.29
				2007	6.35±1.67	0.07±0.99	31	1.47
				2008	5.46±0.97	0.28±0.60	20	1.21

AU-Whr	-36.6732	145.0294	EBF	2011	4.84±0.46	-0.60±0.47	3	0.9
				2012	3.48±0.91	0.05±0.21	36	0.68
				2013	3.23±1.03	0.03±0.23	36	0.66
				2014	3.96±0.99	-0.17±0.27	36	0.78
AU-Wom	-37.4222	144.0944	EBF	2010	6.09±2.11	0.20±0.54	26	1.52
				2011	6.46±2.21	0.17±0.66	29	1.29
				2012	6.31±2.04	0.13±0.41	31	1.07
				2013	4.48±1.75	0.41±0.29	31	1.13
				2014	6.41±2.17	0.01±0.45	30	1.19
AU-Ync	-34.9893	146.2907	GRA	2012	0.31±0.48	-0.03±0.26	5	0.38
				2013	0.95±1.24	0.09±0.17	28	0.55
				2014	-0.25±0.13	0.15±0.09	6	0.42
BE-Bra	51.3076	4.5198	MF	1999	3.70±2.80	0.38±0.70	18	1.54
				2000	2.49±2.26	0.25±0.58	26	1.18
				2001	3.54±2.62	1.11±0.89	26	1.64
				2002	2.80±2.17	1.00±0.81	26	1.42
				2004	2.56±2.25	0.93±0.91	24	1.39
				2005	2.93±2.82	1.27±0.83	31	1.49
				2006	3.26±2.92	0.38±1.32	30	1.57
				2007	3.91±3.02	0.40±0.81	29	1.53
				2008	3.21±2.78	0.95±1.50	27	1.42
				2009	3.82±3.35	0.29±0.65	29	1.49
				2010	3.12±2.60	0.37±0.95	31	1.22
				2011	3.56±3.11	-0.29±0.43	26	0.9
				2012	3.06±2.72	-0.20±0.40	23	0.89
				2013	4.46±3.73	-0.23±0.62	25	1.07

				2014	3.57±3.19	0.14±0.41	26	0.99
BE-Lon	50.5516	4.7461	CRO	2004	5.70±5.99	-1.46±3.04	23	2.7
				2005	4.57±5.96	-1.51±4.91	35	2.28
				2006	2.35±3.84	0.90±2.08	35	2.18
				2007	4.67±5.81	-1.15±4.76	36	2.42
				2008	4.64±5.87	-1.12±3.99	35	2.31
				2009	4.42±5.99	-1.07±4.01	33	2.25
				2010	1.04±2.31	1.98±1.68	32	2.16
				2011	3.71±5.88	-0.12±4.35	33	2.32
				2012	4.23±6.36	-0.40±2.39	35	2.13
				2013	4.27±6.04	-0.47±1.59	35	1.78
				2014	1.51±2.95	0.85±0.93	34	1.68
BE-Vie	50.305	5.9981	MF	1999	3.04±3.29	0.06±0.61	21	1.06
				2000	3.34±2.84	0.42±0.76	30	1.17
				2001	3.68±3.22	0.31±1.06	31	1.23
				2002	3.65±3.17	0.19±0.88	28	1.23
				2003	3.70±3.09	0.28±1.21	23	1.3
				2004	3.57±2.99	-0.17±1.08	22	1.19
				2005	4.32±3.54	0.08±0.72	31	1.26
				2006	3.51±2.82	0.38±0.80	29	1.2
				2007	4.07±3.10	0.29±0.99	31	1.18
				2008	3.71±3.28	-0.39±0.89	24	1.03
				2009	3.02±3.90	-0.49±1.04	19	0.8
				2010	4.38±4.33	-0.18±0.54	26	0.83
				2011	3.64±3.16	-0.01±0.35	22	0.8
				2012	4.74±3.66	-0.08±0.29	26	0.89
				2013	4.06±3.43	-0.01±0.35	19	0.77
				2014	3.08±3.05	0.06±0.53	16	0.72

BR-Sa1	-2.8567	-54.9589	EBF	2002	9.02±0.94	0.05±0.52	17	0.66
				2003	9.17±1.19	-0.04±0.67	16	0.72
				2004	8.96±0.81	0.16±0.63	13	0.74
				2005	9.55±0.88	-0.22±0.52	9	0.77
				2006	11.03±0.00	-1.15±0.00	1	0.81
				2008	9.26±0.84	-0.03±0.48	8	0.79
				2009	9.03±1.18	0.01±0.64	14	0.77
				2010	9.13±1.13	-0.04±0.47	12	0.78
				2011	8.73±1.58	0.08±0.80	16	0.91
BR-Sa3	-3.018	-54.9714	EBF	2000	8.25±1.01	-0.04±0.54	10	0.92
				2001	7.86±1.06	0.24±0.44	12	0.77
				2002	8.53±1.06	0.03±0.55	12	0.68
				2003	8.33±1.00	-0.05±0.42	18	0.68
				2004	9.67±0.32	-0.64±0.32	2	0.85
CA-Gro	48.2167	-82.1556	MF	2003	2.12±0.85	-0.12±0.53	4	0.97
				2004	2.94±3.49	-0.09±1.13	29	1.2
				2005	3.25±2.93	0.07±0.89	30	1.31
				2006	2.91±3.76	-0.23±0.87	33	1.16
				2007	3.03±3.63	0.07±0.87	33	1.23
				2008	3.22±3.78	-0.44±1.14	32	1.18
				2009	2.16±2.85	0.29±0.82	32	1.15
				2010	2.35±2.79	0.52±0.66	33	1.18
				2011	1.24±2.17	0.26±0.30	26	0.84
				2012	1.99±2.42	0.30±0.51	32	0.8
				2013	2.48±2.67	0.35±0.42	29	0.95
				2014	0.30±0.67	0.14±0.23	14	0.52
CA-Man	55.8796	-98.4808	ENF	1999	2.20±2.38	0.18±0.39	28	0.88
				2000	1.39±1.65	0.07±0.37	30	0.8

				2001	1.76±2.03	0.18±0.38	32	0.81
				2002	1.46±1.97	0.12±0.19	32	0.56
				2003	1.57±1.92	0.08±0.18	29	0.48
				2004	0.05±0.26	-0.04±0.14	5	0.15
				2006	1.32±1.76	-0.07±0.09	10	0.35
				2007	0.98±1.56	0.00±0.15	11	0.42
				2008	1.92±1.99	0.10±0.34	24	0.7
CA-NS1	55.8792	-98.4839	ENF	2002	3.26±2.44	-0.13±0.35	14	0.68
				2003	1.85±2.44	-0.12±0.26	27	0.44
				2004	1.53±2.28	-0.08±0.21	25	0.41
				2005	2.02±2.22	-0.03±0.21	22	0.51
CA-NS2	55.9058	-98.5247	ENF	2001	2.12±2.10	0.01±0.10	15	0.56
				2002	1.63±2.14	-0.00±0.20	31	0.47
				2003	1.94±2.00	0.07±0.25	24	0.46
				2004	1.35±1.92	0.05±0.22	29	0.44
				2005	3.50±1.67	0.29±0.16	11	0.85
CA-NS3	55.9117	-98.3822	ENF	2001	1.86±1.89	0.19±0.24	15	0.74
				2002	1.26±1.54	0.19±0.52	36	0.61
				2003	1.40±1.79	-0.06±0.38	30	0.37
				2004	1.35±2.01	-0.20±0.41	32	0.4
				2005	2.67±2.68	-0.47±0.53	26	0.59
CA-NS4	55.9144	-98.3806	ENF	2002	0.07±0.09	0.10±0.06	6	0.14
				2003	2.48±1.98	0.14±0.39	19	0.62
				2004	1.34±1.32	0.42±0.44	22	0.5
				2005	1.58±1.63	0.60±0.54	27	0.58
CA-NS5	55.8631	-98.485	ENF	2001	1.27±1.88	0.26±0.40	13	0.68
				2002	1.76±2.53	0.13±0.23	29	0.55
				2003	2.99±2.83	0.04±0.21	27	0.73

				2004	1.84±2.50	0.02±0.28	36	0.56
				2005	2.57±2.66	0.17±0.28	27	0.75
CA-NS6	55.9167	-98.9644	OSH	2001	0.11±0.21	0.00±0.08	7	0.26
				2002	1.19±1.77	0.22±0.50	34	0.72
				2003	1.17±1.67	0.15±0.32	35	0.6
				2004	0.91±1.52	0.07±0.15	32	0.42
				2005	1.70±2.01	0.22±0.32	26	0.72
CA-NS7	56.6358	-99.9483	OSH	2002	1.45±1.96	-0.11±0.34	15	0.48
				2003	0.79±1.73	-0.04±0.26	27	0.37
				2004	0.93±1.57	-0.02±0.17	34	0.38
				2005	1.22±1.68	0.14±0.26	24	0.52
CA-Oas	53.6289	-106.198	DBF	1999	2.55±4.12	0.27±0.64	34	0.9
				2000	2.62±4.06	-0.09±0.85	34	0.94
				2001	2.69±4.25	0.06±0.98	33	0.9
				2002	2.44±3.76	0.45±0.78	35	0.97
				2003	2.20±3.52	0.56±1.14	34	0.95
				2004	1.90±3.37	0.14±0.63	34	0.8
				2005	2.65±4.19	0.18±0.72	35	0.89
				2006	3.24±4.83	-0.17±0.73	33	0.84
				2007	2.53±3.84	0.10±0.53	34	1.01
				2008	2.58±4.14	-0.02±0.26	34	0.63
				2009	2.72±4.35	-0.02±0.28	34	0.59
				2010	2.69±4.28	0.03±0.41	34	0.64
CA-Obs	53.9872	-105.118	ENF	1999	2.83±2.29	0.24±0.60	22	1.15
				2000	1.86±2.29	0.13±0.35	32	0.97
				2001	1.16±1.76	0.17±0.49	25	0.81
				2002	1.58±2.24	0.23±0.73	32	0.94
				2003	1.43±1.91	0.26±0.64	27	1.02

				2004	1.49±2.03	0.00±0.41	29	0.79
				2005	1.76±2.22	0.24±0.43	31	0.89
				2006	2.09±2.44	0.34±0.55	30	0.99
				2007	2.21±2.39	0.14±0.66	35	0.99
				2008	2.00±2.49	0.04±0.17	33	0.61
				2009	2.36±2.73	-0.01±0.26	31	0.72
				2010	2.59±2.72	0.01±0.29	32	0.7
CA-Qfo	49.6925	-74.3421	ENF	2003	1.10±1.30	0.37±0.35	9	0.76
				2004	1.68±1.93	0.43±0.42	30	0.94
				2005	1.88±2.09	0.29±0.55	36	0.93
				2006	1.93±2.15	0.26±0.47	36	0.89
				2007	1.68±1.84	0.43±0.61	36	0.88
				2008	1.86±2.01	0.13±0.17	35	0.61
				2009	1.58±2.02	0.14±0.22	36	0.59
				2010	1.56±1.95	0.16±0.24	29	0.58
CA-SF1	54.485	-105.818	ENF	2003	5.59±1.60	0.40±0.33	7	1.2
				2004	4.28±1.76	0.12±0.62	16	1.13
				2005	5.23±1.82	0.08±0.31	16	1.14
				2006	6.60±1.50	-0.38±0.44	16	1.24
CA-SF2	54.2539	-105.878	ENF	2001	7.68±1.73	-0.04±0.49	8	1.54
				2002	6.20±3.84	-0.45±0.92	16	1.51
				2003	6.16±2.92	-0.22±0.57	11	1.49
				2004	4.39±2.82	-0.01±0.28	12	1.24
				2005	6.73±2.92	-0.54±0.70	15	1.53
CA-SF3	54.0916	-106.005	OSH	2002	2.01±0.48	0.14±0.31	3	0.85
				2003	2.62±1.29	-0.03±0.21	16	0.96
				2004	2.26±1.15	0.04±0.31	16	0.82
				2005	2.79±1.32	0.04±0.23	16	0.79

				2006	2.74±1.41	0.26±0.41	16	1.02
CA-TP1	42.6609	-80.5595	ENF	2003	0.74±1.28	2.19±2.08	14	1.62
				2004	0.89±1.20	2.11±2.04	15	1.57
				2005	0.81±0.81	2.79±2.67	10	1.79
				2006	2.20±1.67	1.57±1.58	6	2.19
				2007	0.37±0.99	0.54±0.73	11	0.73
				2008	1.89±1.56	1.37±1.56	10	1.54
				2009	2.64±2.10	0.85±1.49	28	1.72
				2010	2.64±2.56	0.00±0.81	29	1.48
				2011	3.22±2.87	-0.04±0.66	33	1.18
				2012	3.44±2.83	-0.19±0.76	31	1.21
				2013	2.62±3.03	-0.19±0.54	25	0.86
				2014	1.74±2.68	-0.22±0.62	19	0.65
CA-TP2	42.7744	-80.4588	ENF	2003	5.05±4.11	-0.62±0.68	19	1.41
				2004	5.45±4.89	-0.79±0.72	16	1.44
				2005	4.50±4.38	-0.19±1.52	14	1.66
				2006	4.69±6.16	-0.59±1.32	15	1.27
				2007	9.66±3.42	-1.44±0.81	11	2.29
CA-TP3	42.7068	-80.3483	ENF	2003	3.43±2.71	0.25±1.20	18	1.37
				2004	2.98±2.13	0.97±1.68	16	1.44
				2005	2.39±1.99	0.71±0.93	13	1.23
				2006	5.60±1.68	1.80±1.63	7	2.26
				2007	1.72±1.39	0.34±1.35	9	0.88
				2008	4.10±3.68	-0.07±0.83	36	1.38
				2009	4.37±3.91	-0.29±0.97	36	1.34
				2010	4.69±4.04	-0.42±1.08	35	1.35
				2011	3.75±3.49	-0.24±1.03	32	1.2
				2012	4.54±3.46	-0.16±0.59	35	1.08

				2013	4.80±4.62	-0.38±0.61	35	0.99
				2014	4.62±4.33	-0.22±0.55	33	0.94
CA-TP4	42.7102	-80.3574	ENF	2002	2.17±1.80	3.38±2.34	19	1.59
				2003	3.39±3.69	0.06±0.89	32	1.26
				2004	3.36±3.14	0.64±1.10	35	1.41
				2005	3.06±3.04	0.72±1.03	33	1.29
				2006	3.56±3.46	0.19±0.59	33	1.26
				2007	2.48±2.90	0.24±0.79	27	1.14
				2008	3.31±3.79	-0.27±0.94	29	1.15
				2009	3.04±3.94	-0.10±1.40	26	1.11
				2010	2.72±2.98	-0.04±1.08	24	1.05
				2011	3.93±3.99	-0.06±0.44	32	0.94
				2012	3.39±3.15	0.26±0.43	31	0.96
				2013	3.41±3.95	-0.05±0.60	23	0.89
				2014	3.85±4.50	-0.20±0.63	29	0.93
CA-TPD	42.6353	-80.5577	DBF	2012	2.97±3.36	0.30±0.71	28	0.99
				2013	2.23±3.20	0.14±0.31	23	0.65
				2014	3.12±4.86	-0.07±0.34	28	0.65
CG-Tch	-4.2892	11.6564	SAV	2006	1.76±2.15	0.18±0.54	16	0.65
				2007	2.83±2.52	-0.08±0.47	21	0.71
				2008	2.62±1.98	0.16±0.44	27	0.76
				2009	4.18±3.35	-0.25±0.60	28	0.85
CH-Cha	47.2102	8.4104	GRA	2005	4.39±4.12	-1.34±2.60	5	1.56
				2006	4.01±4.03	-0.21±1.67	12	1.67
				2007	5.57±5.14	-0.47±1.92	16	1.86
				2008	6.99±3.45	-0.75±1.04	10	1.84
				2009	7.93±5.26	-1.28±1.34	7	1.93
				2010	6.36±4.72	-0.50±1.76	12	1.83

				2011	5.10±3.47	-0.29±0.86	18	1.37
				2012	3.90±3.37	0.36±0.93	21	1.38
				2013	5.04±3.26	-0.45±0.91	20	1.43
				2014	5.95±4.72	-0.30±1.08	13	1.63
CH-Dav	46.8153	9.8559	ENF	1999	3.15±2.67	-0.12±0.67	28	1.25
				2000	2.69±2.67	0.13±0.79	26	1.21
				2001	3.38±2.69	0.09±0.94	22	1.51
				2002	2.86±2.62	-0.16±0.80	23	1.23
				2003	1.70±2.29	0.34±0.55	25	1.07
				2004	2.90±2.76	0.27±0.75	30	1.23
				2005	3.61±2.12	-0.14±0.90	10	1.29
				2006	3.08±2.05	0.57±1.12	28	1.36
				2007	3.08±2.12	0.58±1.12	24	1.34
				2008	2.95±2.39	0.04±0.99	28	1.28
				2009	3.11±2.65	0.12±1.18	28	1.23
				2010	3.22±2.37	-0.01±1.26	29	1.3
				2011	4.51±2.85	-0.09±0.55	23	1.1
				2012	3.27±2.36	0.09±0.57	29	1.02
				2013	2.95±2.13	0.10±0.48	27	0.93
				2014	3.70±2.38	-0.14±0.57	33	1.06
CH-Fru	47.1158	8.5378	GRA	2005	3.69±3.14	-0.23±1.60	12	1.12
				2006	4.24±4.32	0.24±0.66	19	1.12
				2007	5.09±5.21	-0.01±0.79	19	1.11
				2008	6.06±5.35	-0.36±0.78	10	1.27
				2009	3.14±4.95	0.06±0.87	10	1.11
				2010	4.16±3.73	-0.06±0.76	10	1.05
				2011	6.18±4.56	-0.56±0.52	10	1.21
				2012	4.49±4.58	0.07±0.87	11	1.09

				2013	4.14±5.76	-0.11±0.72	12	1.05
				2014	4.44±4.86	0.15±1.30	11	1.31
CH-Lae	47.4781	8.365	MF	2004	3.45±2.01	-0.05±0.79	2	1.72
				2005	6.23±2.57	1.68±1.21	7	1.83
				2006	3.56±2.89	-0.42±1.67	10	1.39
				2007	5.19±4.12	0.10±0.43	6	1.17
				2008	6.76±4.14	-0.32±0.40	10	1.07
				2009	4.01±3.68	0.01±0.47	18	1.04
				2010	4.68±3.16	0.17±0.60	5	1.32
				2011	3.31±2.82	-0.13±0.59	10	0.96
				2012	2.85±2.90	0.36±0.86	9	1.18
				2013	4.45±3.51	0.15±0.75	19	1.15
				2014	5.22±2.68	0.20±0.74	20	1.27
CH-Oe1	47.2858	7.7319	GRA	2002	5.35±3.56	-1.63±1.39	22	1.67
				2003	3.43±3.68	-1.30±2.14	17	1.22
				2004	6.07±4.21	-1.03±1.47	21	1.67
				2005	3.83±3.71	-0.26±0.60	20	1.04
				2006	3.87±3.63	-0.29±0.83	25	1.12
				2007	3.30±2.50	-0.02±0.60	23	1.01
				2008	2.71±3.29	-0.03±0.64	21	1.03
CH-Oe2	47.2863	7.7343	CRO	2004	3.94±5.38	0.08±1.81	30	1.72
				2005	3.80±3.84	-0.82±2.39	33	1.62
				2006	2.25±3.38	1.53±3.28	18	1.81
				2007	3.04±3.92	0.12±2.03	25	1.56
				2008	3.44±3.66	-0.35±1.81	30	1.66
				2009	3.13±3.57	-0.06±2.24	29	1.43
				2010	1.32±1.77	1.84±2.12	30	1.55
				2011	3.41±4.18	-0.27±0.96	24	1.17

				2012	2.94±3.61	0.06±1.09	28	1.31
				2013	3.19±3.85	0.06±0.70	30	1.21
				2014	4.39±4.43	-0.44±0.99	26	1.3
CN-Cha	42.4025	128.0958	MF	2003	4.14±4.83	-0.37±0.44	25	0.95
				2004	3.81±3.95	-0.24±0.40	27	1.02
				2005	2.93±3.42	-0.13±0.36	19	0.88
CN-Cng	44.5934	123.5092	GRA	2007	1.88±1.87	0.02±0.52	21	0.79
				2008	1.70±2.32	-0.02±0.37	36	0.56
				2009	1.56±1.87	-0.08±0.46	32	0.55
				2010	1.90±1.94	-0.06±0.22	26	0.6
CN-Dan	30.4978	91.0664	GRA	2004	0.98±0.94	-0.07±0.10	32	0.28
				2005	0.82±0.88	0.03±0.10	34	0.3
CN-Din	23.1733	112.5361	EBF	2003	3.80±1.23	0.40±0.46	18	1.16
				2004	4.60±1.39	0.11±0.39	21	1.1
				2005	3.65±1.37	0.29±0.40	23	1.06
CN-Du2	42.0467	116.2836	GRA	2007	0.41±0.48	0.09±0.18	33	0.35
				2008	0.98±1.35	0.03±0.32	32	0.46
CN-Du3	42.0551	116.2809	GRA	2009	0.28±0.69	0.33±0.40	14	0.52
				2010	-0.16±0.25	0.17±0.16	13	0.27
CN-Ha2	37.6086	101.3269	WET	2003	2.33±2.41	0.24±0.31	31	0.7
				2004	2.62±2.68	-0.08±0.19	32	0.68
				2005	2.84±2.87	-0.07±0.22	31	0.7
CN-HaM	37.37	101.18	GRA	2002	1.53±2.25	-0.11±0.29	33	0.5
				2003	1.61±2.36	-0.20±0.42	35	0.53
				2004	0.86±1.34	0.20±0.28	36	0.47
CN-Qia	26.7414	115.0581	ENF	2003	3.68±2.06	-0.01±0.50	15	1.15
				2004	3.90±2.17	-0.10±0.43	12	1.08
				2005	4.00±2.44	-0.00±0.34	17	1.11

CN-Sw2	41.7902	111.8971	GRA	2010	0.93±0.00	-0.12±0.00	1	0.3
				2011	-0.01±1.00	0.14±0.50	31	0.64
				2012	0.94±0.31	-0.37±0.29	2	0.48
CZ-BK1	49.5021	18.5369	ENF	2004	3.72±3.76	0.60±1.52	19	1.34
				2005	6.39±2.71	0.75±1.64	14	1.65
				2006	6.18±2.10	0.56±1.27	12	1.73
				2007	6.84±3.03	-0.63±1.51	18	1.82
				2008	5.46±4.15	-0.49±1.66	21	1.44
				2009	4.00±4.22	-0.06±1.33	28	1.32
				2010	4.85±3.63	-0.39±1.31	25	1.42
				2011	4.57±4.15	-0.22±0.93	30	1.09
				2012	4.17±3.36	0.22±0.47	28	1.04
				2013	4.56±3.51	-0.03±0.81	29	1.11
				2014	5.21±3.82	0.03±0.45	31	1.05
CZ-BK2	49.4944	18.5429	GRA	2006	3.24±2.47	2.70±1.15	12	1.83
				2007	4.22±3.08	1.07±1.32	13	1.83
				2008	3.47±3.39	0.66±1.11	17	1.45
				2009	2.42±3.26	0.38±0.98	28	1.12
				2010	2.74±3.10	0.48±0.75	27	1.1
				2011	3.08±3.71	0.10±0.61	28	1.04
				2012	0.93±1.60	0.49±0.61	21	0.89
CZ-wet	49.0247	14.7704	WET	2006	4.41±3.93	-0.15±1.06	18	1.95
				2007	1.56±2.56	0.42±1.00	26	1.01
				2008	2.90±3.59	0.46±0.90	35	1.48
				2009	2.94±3.16	0.60±1.01	34	1.45
				2010	2.89±3.69	0.55±1.48	33	1.5
				2011	1.64±2.45	0.41±0.49	27	0.87
				2012	2.58±3.49	0.07±0.46	27	0.9

				2013	1.59±3.10	0.21±0.70	24	0.72
				2014	2.16±2.81	0.37±0.71	22	0.97
DE-Akm	53.8662	13.6834	WET	2009	0.14±0.12	0.14±0.17	6	0.35
				2010	2.78±3.54	0.11±0.26	31	1.03
				2011	6.23±4.47	-1.09±0.83	13	1.58
				2012	5.20±4.78	-0.89±0.87	15	1.44
				2013	4.18±4.82	-0.69±0.65	29	1.15
				2014	3.34±3.99	0.48±0.42	22	1.26
DE-Geb	51.1001	10.9143	CRO	2001	3.25±4.98	-0.15±2.00	34	1.5
				2002	2.16±3.45	0.52±2.57	34	1.35
				2003	2.73±4.87	-0.28±1.39	35	1.27
				2004	3.54±4.78	-0.52±1.13	36	1.35
				2005	3.73±5.44	-0.38±2.73	34	1.42
				2006	3.82±5.17	-0.58±4.18	36	1.61
				2007	2.92±3.87	0.70±1.23	33	1.73
				2008	3.14±5.28	0.02±2.16	35	1.42
				2009	1.82±3.31	1.17±2.06	33	1.36
				2010	3.11±5.00	-0.28±1.78	36	1.41
				2011	1.77±1.82	1.15±3.15	35	1.47
				2012	2.43±2.50	-0.09±0.75	36	1.04
				2013	3.04±4.91	-0.02±0.79	36	1.06
				2014	3.74±4.11	-0.12±0.63	35	1.19
DE-Gri	50.95	13.5126	GRA	2004	3.39±3.09	0.72±1.38	34	1.58
				2005	4.14±4.02	-0.22±1.16	30	1.33
				2006	2.59±2.43	1.43±1.82	31	1.36
				2007	3.92±3.05	0.69±1.77	34	1.44
				2008	4.07±3.49	0.08±1.69	34	1.4
				2009	5.12±4.36	-0.43±1.45	35	1.5

				2010	4.53±4.03	-0.27±1.21	35	1.41
				2011	5.10±4.30	-0.12±1.43	33	1.5
				2012	4.56±4.17	0.18±0.94	36	1.17
				2013	3.87±3.72	0.10±0.82	35	1.04
				2014	5.78±4.38	-0.13±0.98	36	1.18
DE-Hai	51.0792	10.453	DBF	2000	4.55±5.54	-0.80±1.87	33	1.05
				2001	4.51±5.21	-0.40±1.37	35	1.16
				2002	4.77±5.28	-0.42±1.75	35	1.21
				2003	4.03±5.03	-0.04±1.42	34	1.06
				2004	4.02±4.58	-0.09±0.87	35	1.14
				2005	4.43±5.10	-0.32±1.30	35	1.21
				2006	3.99±4.68	-0.16±1.27	34	1.16
				2007	3.94±4.58	0.13±1.34	34	1
				2008	4.48±5.22	-0.24±1.39	36	1.1
				2009	4.42±4.95	-0.09±1.25	35	1.04
				2010	4.06±4.37	0.21±0.50	27	0.91
				2011	3.87±4.43	0.20±0.45	34	0.86
				2012	4.02±4.52	-0.01±0.41	35	0.79
DE-Kli	50.8931	13.5224	CRO	2004	4.75±4.12	-0.01±1.64	17	2.02
				2005	3.95±5.08	-0.88±2.47	32	1.53
				2006	3.36±4.86	-0.41±1.92	33	1.5
				2007	3.09±4.19	-0.20±2.58	35	1.53
				2008	2.26±2.74	0.75±0.93	24	1.78
				2009	4.01±4.76	0.29±1.86	23	1.93
				2010	3.54±4.14	0.04±1.51	33	1.62
				2011	4.15±5.01	-0.83±1.74	35	1.48
				2012	1.90±2.64	0.48±0.60	34	1.14
				2013	0.97±1.85	0.14±0.25	25	0.64

				2014	4.54±5.16	-0.54±1.22	36	1.43
DE-Lkb	49.0996	13.3047	ENF	2009	1.61±1.26	0.59±0.48	21	1.15
				2010	0.58±0.78	0.43±0.32	14	0.78
				2011	1.38±1.43	0.21±0.32	27	0.97
				2012	1.50±1.65	0.32±0.30	19	1.05
				2013	1.73±1.76	0.41±0.52	18	1.09
DE-Lnf	51.3282	10.3678	DBF	2002	6.26±5.19	-0.83±1.35	25	1.49
				2003	4.08±5.08	-0.49±1.25	33	1.23
				2004	3.43±4.07	0.13±0.55	33	1.27
				2005	4.68±5.31	-0.47±1.47	35	1.31
				2006	4.09±4.81	0.09±0.90	34	1.22
				2010	4.33±5.64	-0.20±0.75	32	0.95
				2011	3.06±3.93	0.16±0.37	28	0.79
				2012	4.29±5.21	-0.33±0.64	33	0.88
DE-Obe	50.7867	13.7213	ENF	2008	4.55±3.85	0.06±1.25	25	1.54
				2009	3.19±3.18	0.32±0.89	25	1.27
				2010	3.48±3.64	-0.15±0.78	28	1.21
				2011	3.66±3.64	0.15±0.40	26	0.98
				2012	3.87±4.26	-0.11±0.58	24	1
				2013	3.90±4.14	-0.05±0.67	31	1.06
				2014	4.53±4.12	-0.07±0.64	25	1.08
DE-RuR	50.6219	6.3041	GRA	2011	6.13±3.11	0.02±0.49	15	1.15
				2012	4.89±4.06	-0.09±0.63	27	1
				2013	4.33±3.62	0.04±0.60	34	0.97
				2014	4.39±3.20	0.09±0.70	31	1.04
DE-RuS	50.8659	6.4472	CRO	2011	9.44±4.45	-1.44±1.49	17	2.84
				2012	4.58±5.11	0.41±1.31	19	2.15
				2013	5.16±5.82	-0.55±1.19	33	1.74

				2014	5.43±5.43	-0.54±1.67	30	2.09
DE-Seh	50.8706	6.4497	CRO	2007	6.22±4.95	-2.23±2.90	15	1.92
				2008	4.01±5.48	-0.16±1.47	33	1.66
				2009	3.28±4.58	0.02±0.94	35	1.42
				2010	3.94±3.92	-0.14±1.85	28	1.99
DE-SfN	47.8064	11.3275	WET	2012	2.79±2.41	0.55±0.62	13	1.16
				2013	1.29±1.17	0.38±0.49	23	0.77
				2014	2.11±1.34	0.49±0.45	19	0.97
DE-Spw	51.8923	14.0337	WET	2010	6.11±4.23	0.39±1.22	6	1.5
				2011	3.37±4.08	-0.26±0.40	28	0.71
				2012	3.62±4.07	-0.27±0.38	29	0.74
				2013	2.45±3.28	0.01±0.22	27	0.55
				2014	3.83±4.31	-0.08±0.42	30	0.79
DE-Tha	50.9624	13.5652	ENF	1999	3.87±3.98	-0.84±1.20	24	1.13
				2000	4.83±3.91	-0.78±1.26	26	1.25
				2001	3.99±3.72	-0.22±0.71	29	1.19
				2002	4.48±3.54	-0.47±0.92	31	1.26
				2003	2.89±2.87	0.03±1.12	24	1.13
				2004	4.71±4.20	-0.99±1.22	30	1.34
				2005	3.90±4.15	-0.67±1.63	24	1.07
				2006	3.57±3.46	-0.22±0.85	27	1.13
				2007	4.30±3.54	-0.31±0.98	25	1.26
				2008	2.96±2.52	-0.50±0.91	19	1.03
				2009	4.36±4.53	-0.71±0.74	23	1.08
				2010	3.79±3.75	-0.69±1.16	22	1.11
				2011	5.25±4.10	-0.22±0.42	30	0.92
				2012	5.19±4.32	-0.26±0.51	29	0.99
				2013	3.34±3.75	-0.19±0.53	26	0.75

				2014	5.23±3.88	-0.20±0.50	29	1
DE-Zrk	53.8759	12.889	WET	2013	2.37±2.26	0.82±0.94	17	1.62
				2014	1.05±1.50	0.37±0.53	26	0.87
DK-Eng	55.6905	12.1918	GRA	2005	2.57±2.14	0.38±0.76	23	1.3
				2006	1.66±1.66	0.23±0.51	30	0.91
				2007	2.47±1.86	0.23±0.60	31	1.14
				2008	2.62±2.10	0.64±2.02	16	1.77
DK-Fou	56.4842	9.5872	CRO	2005	3.66±2.92	0.43±1.23	24	1.58
DK-NuF	64.1308	-51.3861	WET	2008	1.29±1.12	0.17±0.21	13	0.49
				2009	0.87±1.02	0.12±0.21	15	0.44
				2010	1.93±1.44	-0.20±0.30	15	0.54
				2011	0.84±0.79	-0.09±0.27	15	0.44
				2012	1.49±1.39	-0.01±0.20	12	0.53
				2013	1.71±1.56	-0.03±0.16	16	0.54
				2014	2.51±1.63	0.24±0.34	6	0.87
DK-Sor	55.4859	11.6446	DBF	1999	5.06±5.47	-0.80±1.29	35	1.74
				2000	5.06±5.27	-1.09±1.38	34	1.79
				2001	5.21±5.76	-0.95±1.28	36	1.67
				2002	4.97±5.45	-0.63±1.24	36	1.77
				2003	5.74±5.83	-1.04±1.53	33	1.87
				2004	5.40±5.80	-1.18±1.72	31	1.64
				2005	5.15±5.71	-0.83±1.27	34	1.72
				2006	5.10±5.69	-0.77±1.35	35	1.73
				2007	5.05±5.12	-0.95±1.45	33	1.68
				2008	5.31±5.92	-0.91±1.51	33	1.66
				2009	5.66±5.85	-0.90±1.64	36	1.85
				2010	4.51±5.36	-0.74±1.49	34	1.56
				2011	5.72±5.55	-0.96±1.71	35	1.61

				2012	5.31±5.65	-0.50±0.66	35	1.27
				2013	5.41±5.84	-0.38±0.51	33	1.24
				2014	7.91±6.03	-0.51±0.79	27	1.82
DK-ZaF	74.4814	-20.5545	WET	2008	0.89±0.00	-0.50±0.00	1	0.33
				2009	0.02±0.12	0.23±0.01	3	0.35
				2010	0.02±0.11	0.14±0.06	5	0.3
				2011	0.09±0.16	0.13±0.06	4	0.31
DK-ZaH	74.4733	-20.5503	GRA	2000	1.03±0.46	-0.20±0.21	9	0.28
				2001	0.73±0.46	0.05±0.23	9	0.28
				2002	1.11±0.48	-0.17±0.29	8	0.31
				2003	1.07±0.73	-0.03±0.32	9	0.29
				2004	0.60±0.50	0.25±0.27	9	0.28
				2005	1.19±0.61	-0.16±0.22	10	0.31
				2006	0.85±0.52	0.00±0.19	9	0.26
				2007	0.38±0.39	0.11±0.24	15	0.22
				2008	0.48±0.66	0.05±0.29	17	0.22
				2009	0.60±0.57	-0.07±0.16	16	0.22
				2010	0.55±0.57	-0.01±0.14	19	0.2
				2011	0.03±0.01	0.47±0.27	2	0.39
				2012	0.75±0.86	-0.12±0.24	14	0.24
				2013	0.50±0.47	0.04±0.17	18	0.23
				2014	0.46±0.59	0.11±0.14	15	0.35
ES-Amo	36.8336	-2.2523	OSH	2007	-0.51±0.94	0.35±0.34	14	0.5
				2008	0.07±0.87	0.47±0.54	9	0.62
				2009	-0.14±0.70	0.04±0.24	35	0.5
				2010	0.03±0.81	0.12±0.18	36	0.46
				2011	0.02±0.86	0.08±0.22	30	0.41
				2012	-0.31±0.78	0.15±0.16	35	0.39

ES-LgS	37.0979	-2.9658	OSH	2007	1.22±0.99	0.24±0.21	36	0.82
				2008	1.17±1.04	0.34±0.21	32	0.85
				2009	1.53±1.21	0.04±0.28	16	0.74
ES-LJu	36.9266	-2.7521	OSH	2004	0.14±0.57	0.14±0.27	16	0.54
				2005	-0.14±0.15	0.25±0.15	19	0.52
				2006	0.18±0.56	0.16±0.30	28	0.57
				2007	0.33±0.70	0.13±0.33	26	0.67
				2008	0.40±0.69	0.45±0.46	29	0.9
				2009	0.38±0.56	0.10±0.35	36	0.59
				2010	0.49±0.67	0.01±0.23	30	0.55
				2011	0.50±0.66	0.01±0.16	36	0.44
				2012	0.17±0.44	0.03±0.18	28	0.39
				2013	0.34±0.48	0.06±0.11	26	0.4
ES-Ln2	36.9695	-3.4758	OSH	2009	-0.17±0.27	0.31±0.30	19	0.68
FI-Hyy	61.8474	24.2948	ENF	1999	2.52±2.87	0.05±0.69	34	0.95
				2000	3.38±3.06	0.02±0.48	31	1.16
				2001	2.90±3.16	-0.07±0.46	35	1.03
				2002	2.98±3.19	0.18±0.60	34	1.04
				2003	2.62±3.02	0.27±0.58	35	0.98
				2004	2.94±2.98	0.35±0.56	35	1.07
				2005	3.61±3.06	0.12±0.80	28	1.17
				2006	2.91±3.16	0.22±0.69	33	1.08
				2007	3.13±3.28	0.02±0.54	36	1.06
				2008	2.92±3.07	0.07±0.55	35	0.99
				2009	3.37±3.74	-0.19±0.55	36	1
				2010	2.84±3.27	-0.16±0.59	31	1
				2011	3.35±3.66	-0.18±0.49	35	0.96
				2012	2.83±3.43	-0.03±0.32	34	0.66

				2013	3.05±3.65	-0.12±0.32	34	0.69
				2014	3.30±3.26	0.05±0.33	34	0.77
FI-Jok	60.8986	23.5135	CRO	2000	0.25±0.54	0.40±0.61	24	0.69
				2001	0.96±2.15	0.44±0.65	33	0.94
				2002	1.24±1.88	0.33±0.70	32	0.94
				2003	1.04±2.02	0.05±0.14	11	0.69
FI-Let	60.6418	23.9595	ENF	2009	0.82±1.96	0.15±0.21	8	0.38
				2010	2.91±3.30	-0.18±0.43	26	0.75
				2011	2.64±3.24	-0.06±0.44	21	0.7
				2012	3.11±3.88	-0.21±0.42	29	0.73
FI-Lom	67.9972	24.2092	WET	2007	1.07±1.88	0.21±0.30	30	0.56
				2008	0.93±1.82	0.14±0.24	33	0.47
				2009	1.18±2.13	0.04±0.31	32	0.51
FI-Sod	67.3624	26.6386	ENF	2001	2.15±2.64	-0.66±0.99	32	0.86
				2002	3.22±2.99	-1.13±1.16	22	1.14
				2003	1.30±1.69	-0.10±0.59	32	0.71
				2004	1.58±1.92	-0.18±0.49	35	0.81
				2005	1.35±1.87	-0.06±0.39	34	0.73
				2006	1.64±2.17	-0.17±0.51	36	0.83
				2007	1.21±1.74	0.12±0.54	32	0.73
				2008	1.30±1.78	-0.02±0.41	36	0.67
				2009	1.39±1.78	0.03±0.44	33	0.83
				2010	1.09±1.78	-0.12±0.44	28	0.6
				2011	1.57±2.07	-0.23±0.46	34	0.69
				2012	1.04±1.77	-0.05±0.22	31	0.43
				2013	1.67±2.39	-0.07±0.22	32	0.5
				2014	1.59±2.30	-0.09±0.35	34	0.53
FR-Fon	48.4764	2.7801	DBF	2005	5.93±4.59	-0.26±1.14	27	1.65

				2006	4.59±4.50	-0.06±0.87	33	1.37
				2007	5.10±4.99	-0.32±1.64	36	1.46
				2008	4.62±5.27	-0.19±1.41	34	1.21
				2009	5.08±5.51	-0.22±1.36	35	1.33
				2010	4.50±4.71	-0.04±0.98	33	1.33
				2011	5.17±5.00	-0.04±0.90	34	1.13
				2012	4.71±4.90	0.22±0.76	36	1.05
				2013	3.93±4.87	0.05±0.50	31	0.91
				2014	3.67±4.47	0.03±0.42	19	0.81
FR-Gri	48.8442	1.9519	CRO	2004	3.73±4.63	0.54±3.37	20	2.32
				2005	3.80±5.51	-0.62±4.98	31	1.87
				2006	3.26±4.38	-0.16±1.69	32	1.7
				2007	3.54±4.18	0.60±3.24	32	2.35
				2008	4.90±5.72	-1.69±4.09	21	1.95
				2009	3.77±5.37	-0.03±1.24	14	1.33
				2010	3.61±4.40	-0.34±0.86	18	1.17
				2011	5.22±5.66	-0.89±1.64	18	2.08
				2012	5.00±4.09	0.36±0.87	12	1.61
				2013	3.90±4.01	0.13±0.73	26	1.46
				2014	4.80±4.95	0.00±1.28	20	1.74
FR-LBr	44.7171	-0.7693	ENF	1999	4.02±1.77	0.17±0.64	15	1.42
				2000	6.29±3.75	-0.99±1.55	12	1.71
				2001	4.83±3.20	-0.35±1.52	23	1.56
				2002	3.21±1.79	1.49±1.14	24	1.55
				2003	4.10±1.83	0.26±1.73	10	1.72
				2004	6.09±3.06	-0.15±0.43	21	1.27
				2005	4.15±2.11	0.01±0.51	29	1.03
				2006	4.39±1.97	-0.17±0.52	29	1.09

				2007	5.16±3.32	-0.17±0.42	19	1.06
				2008	5.60±2.85	-0.51±0.66	15	1.23
FR-Pue	43.7413	3.5957	EBF	2000	2.53±1.33	-0.11±1.20	14	1.34
				2001	3.89±1.81	-0.99±0.99	34	1.34
				2002	3.83±1.76	-0.72±0.96	35	1.51
				2003	3.34±1.79	-0.41±1.39	34	1.45
				2004	4.03±1.87	-1.08±1.07	35	1.35
				2005	2.95±1.59	-0.22±1.09	35	1.26
				2006	2.56±1.10	0.19±1.08	33	1.35
				2007	3.99±1.89	-0.43±0.95	36	1.66
				2008	3.36±1.79	-0.10±0.88	36	1.59
				2009	3.47±1.67	-0.14±1.14	34	1.64
				2010	3.11±1.63	-0.20±0.95	33	1.42
				2011	3.51±1.85	-0.56±0.88	29	1.28
				2012	3.93±1.84	-0.24±0.64	30	1.22
				2013	2.27±1.20	0.35±0.47	35	1.07
				2014	1.81±0.66	0.48±0.25	35	1.07
GF-Guy	5.2788	-52.9249	EBF	2004	10.45±0.92	-0.47±0.88	23	1.27
				2005	9.83±1.30	0.27±1.40	22	1.25
				2006	9.84±1.08	-0.17±1.17	35	1.21
				2007	10.25±1.11	-0.31±1.22	34	1.15
				2008	9.55±0.93	-0.06±0.85	21	1.27
				2009	9.89±1.10	0.01±1.01	20	1.1
				2010	10.86±1.07	-1.02±1.20	36	1.45
				2011	10.44±1.23	-0.50±1.16	13	1.36
				2012	10.77±1.36	-0.48±0.74	35	0.97
				2013	9.77±1.31	0.09±0.76	35	0.97
				2014	8.79±1.44	0.44±0.76	36	1.12

GH-Ank	5.2685	-2.6942	EBF	2011	9.39±2.04	-1.26±0.71	8	1.75
				2012	5.46±0.85	0.75±0.62	9	1.43
				2014	5.80±1.55	0.65±0.61	2	1.46
IT-BCi	40.5238	14.9574	CRO	2004	6.20±6.35	0.04±3.27	21	3.33
				2005	5.03±5.57	0.04±4.32	35	2.75
				2006	4.89±6.33	0.54±4.95	33	2.69
				2007	4.77±4.98	0.18±4.11	34	2.57
				2008	5.04±5.44	-0.29±4.09	30	2.44
				2009	3.48±4.26	1.01±4.21	34	2.17
				2010	5.95±5.17	-0.90±4.74	33	2.37
				2011	5.70±6.59	-0.43±2.53	21	2.36
				2012	5.16±2.48	-0.07±1.26	28	1.88
				2013	5.16±2.59	-0.28±0.94	32	1.61
				2014	5.69±2.85	-0.41±1.26	31	1.74
IT-CA1	42.3804	12.0266	DBF	2011	4.43±3.34	-0.15±0.66	15	1.28
				2012	3.07±3.08	-0.13±0.83	26	1.11
				2013	1.62±1.52	0.36±0.52	17	0.83
				2014	3.75±2.68	0.10±0.41	28	1.24
IT-CA2	42.3772	12.026	CRO	2011	2.89±2.21	0.06±1.27	10	1.62
				2012	2.00±2.09	0.43±0.77	24	1.48
				2013	3.02±3.26	-0.13±0.77	24	1.3
				2014	1.94±2.25	0.55±0.84	7	1.65
IT-CA3	42.38	12.0222	DBF	2011	0.90±0.18	0.22±0.03	4	0.51
				2012	2.03±1.05	0.28±0.43	16	1.01
				2013	2.27±2.23	0.12±0.26	18	0.89
				2014	4.24±2.34	-0.17±0.90	5	1.43
IT-Col	41.8494	13.5881	DBF	1999	6.16±3.92	0.40±0.91	14	1.7
				2000	2.96±4.90	-0.00±1.11	9	1.18

				2001	2.00±3.15	0.61±0.91	26	1.24
				2002	1.65±2.40	0.76±0.51	8	1.48
				2003	7.95±0.00	2.09±0.00	1	1.6
				2004	3.33±3.50	0.08±0.74	14	1.34
				2005	3.83±4.66	0.18±0.68	20	1.19
				2006	2.13±3.02	0.46±0.60	10	1.05
				2007	3.50±4.13	0.14±0.78	27	1.35
				2008	3.20±4.04	0.43±0.84	26	1.27
				2009	3.83±5.13	-0.15±0.56	19	0.84
				2010	5.10±5.36	0.08±0.34	9	0.89
				2011	3.46±4.14	0.25±0.50	31	1.01
				2012	4.39±4.46	0.10±0.37	13	1.03
				2013	2.89±4.02	0.14±0.47	19	0.9
				2014	1.86±3.19	0.15±0.37	22	0.77
IT-Cp2	41.7043	12.3573	EBF	2012	4.36±1.63	0.46±0.75	18	1.31
				2013	5.49±1.85	-0.03±0.55	33	1.2
				2014	6.54±1.62	0.27±0.44	28	1.23
IT-Cpz	41.7053	12.3761	EBF	2000	5.90±1.58	0.44±1.28	27	1.79
				2001	5.40±1.77	0.10±0.87	24	1.72
				2002	6.10±1.64	0.01±0.95	21	1.75
				2003	5.28±1.94	-0.32±1.42	30	1.61
				2004	5.56±2.44	-0.16±0.78	25	1.52
				2005	4.04±2.03	0.43±0.65	26	1.09
				2006	4.53±1.44	0.28±0.51	31	1.08
				2007	4.72±1.78	0.09±0.55	23	1.16
				2008	6.13±2.33	-0.16±0.65	21	1.26
IT-Isp	45.8126	8.6336	DBF	2013	4.88±5.80	-0.70±1.05	4	1.47
				2014	1.78±3.02	-0.06±0.44	6	0.81

IT-La2	45.9542	11.2853	ENF	2000	4.58±4.12	-0.26±0.71	10	1.06
				2001	4.60±4.03	-0.24±0.64	17	1.05
				2002	2.47±2.19	-0.13±0.65	9	1.07
IT-Lav	45.9562	11.2813	ENF	2003	5.57±3.37	-0.02±1.53	31	1.7
				2004	5.68±3.76	-0.96±1.27	30	1.57
				2005	5.76±3.71	-0.29±1.22	24	1.53
				2006	5.93±3.95	-0.43±1.17	34	1.56
				2007	5.79±3.05	-0.30±1.04	32	1.52
				2008	6.13±3.56	-1.04±1.47	29	1.54
				2009	7.77±4.85	-1.78±1.77	32	1.54
				2010	6.40±4.03	-1.27±1.62	32	1.53
				2011	5.78±3.64	-0.31±0.87	28	1.3
				2012	5.60±3.71	-0.25±0.74	28	1.2
				2013	5.83±3.42	-0.20±0.58	34	1.1
				2014	6.99±2.97	-0.25±0.61	27	1.16
IT-MBo	46.0147	11.0458	GRA	2003	3.28±3.81	0.21±0.98	33	1.39
				2004	3.54±4.34	-0.09±0.97	36	1.35
				2005	3.94±4.10	-0.22±0.85	36	1.34
				2006	3.81±4.24	-0.38±1.05	35	1.38
				2007	4.01±3.99	-0.24±0.64	36	1.48
				2008	3.17±3.81	-0.28±1.58	33	1.24
				2009	4.04±4.72	0.10±1.27	35	1.49
				2010	4.08±4.29	-0.17±0.61	32	1.52
				2011	4.57±4.57	-0.21±0.38	35	1.08
				2012	4.36±4.49	-0.23±0.52	36	1.04
				2013	3.83±4.48	-0.11±0.48	33	0.99
IT-PT1	45.2009	9.061	DBF	2002	6.46±5.03	-0.29±0.62	27	1.32
				2003	3.90±3.83	0.07±0.48	35	1

				2004	5.67±4.93	-0.15±0.47	29	1.13
IT-Ren	46.5869	11.4337	ENF	1999	2.69±2.53	0.88±0.96	20	1.24
				2001	-0.28±0.00	0.34±0.00	1	0.34
				2002	3.82±3.21	-0.37±1.03	22	1.29
				2003	2.53±2.77	-0.04±0.74	19	1.12
				2005	2.51±3.15	-0.19±0.61	15	0.99
				2006	4.38±3.80	-0.90±1.09	10	1.19
				2007	2.67±3.19	0.14±0.37	16	0.77
				2008	2.33±2.79	-0.36±0.34	11	0.67
				2009	3.34±4.16	-0.27±0.60	20	0.81
				2010	3.81±3.53	-0.21±0.48	16	0.97
				2011	2.51±2.84	-0.06±0.54	12	0.72
				2012	2.37±3.04	-0.11±0.42	17	0.69
				2013	3.40±3.46	-0.08±0.52	17	0.87
IT-Ro1	42.4081	11.93	DBF	2000	3.23±1.56	0.49±1.09	21	1.94
				2001	4.74±3.38	-0.80±1.18	25	1.93
				2002	3.85±2.86	0.23±1.02	31	2.17
				2003	2.41±1.43	-0.08±0.81	25	1.63
				2004	4.50±4.16	-0.53±1.19	25	1.47
				2005	3.47±3.45	0.09±0.42	28	1.28
				2006	4.00±3.15	-0.05±0.56	31	1.31
				2007	3.44±3.60	0.45±0.77	11	1.46
				2008	4.08±3.93	0.08±1.01	23	1.58
IT-Ro2	42.3903	11.9209	DBF	2002	3.53±3.41	0.39±1.23	29	2.06
				2003	3.12±3.74	-0.39±1.92	28	1.77
				2004	3.77±4.52	-0.53±2.13	29	1.87
				2005	3.84±4.57	0.07±2.62	32	2
				2006	2.99±4.25	-0.12±2.40	27	1.8

				2007	1.93±2.63	1.12±1.33	24	1.57
				2008	4.26±4.01	0.32±1.01	20	1.54
				2010	3.95±3.89	0.30±0.76	34	1.47
				2011	6.13±4.26	-0.24±0.56	16	1.51
				2012	2.50±3.01	0.34±0.75	23	1.33
IT-SR2	43.732	10.291	ENF	2013	5.91±3.29	-0.17±0.67	31	1.2
				2014	6.65±3.03	-0.04±0.52	33	1.29
IT-SRo	43.7279	10.2844	ENF	1999	4.97±1.87	1.40±1.42	36	1.93
				2000	4.99±1.92	0.67±1.66	32	1.8
				2001	5.33±2.09	0.41±1.25	36	1.78
				2002	5.33±2.62	1.16±1.27	27	1.88
				2003	4.18±1.85	1.69±1.81	35	1.9
				2004	4.37±1.85	0.97±1.32	33	1.69
				2005	3.74±1.44	1.09±0.58	20	1.66
				2006	5.85±2.38	0.39±1.31	35	1.95
				2007	6.45±2.64	0.19±1.61	34	2.02
				2008	8.61±4.53	-1.60±2.43	21	2.07
				2009	7.43±3.67	-1.13±1.40	28	1.59
				2010	7.89±3.18	-0.60±0.70	23	1.51
				2011	5.57±2.11	0.48±0.63	32	1.48
				2012	5.44±2.42	-0.17±0.71	35	1.22
IT-Tor	45.8444	7.5781	GRA	2008	4.60±4.10	-0.75±0.74	10	1.56
				2009	2.65±3.25	-0.05±1.19	25	1.26
				2010	2.73±3.12	0.22±0.74	26	1.25
				2011	3.31±3.00	-0.23±0.34	26	0.98
				2012	2.13±3.09	0.03±0.35	29	0.76
				2013	1.88±2.85	0.10±0.41	21	0.92
				2014	2.00±3.05	-0.03±0.31	28	0.78

JP-MBF	44.3869	142.3186	DBF	2004	3.48±3.31	0.49±0.60	20	1.21
				2005	2.48±3.12	0.44±0.61	30	1.08
JP-SMF	35.2617	137.0788	MF	2002	2.17±0.12	-0.28±0.16	2	0.65
				2003	2.77±2.34	0.26±0.62	8	1.07
				2004	1.81±0.75	0.08±0.07	5	0.65
				2005	1.83±1.21	0.65±0.85	4	1.07
				2006	3.08±2.20	0.26±0.31	12	1.12
MY-PSO	2.973	102.3062	EBF	2003	6.38±0.83	0.42±0.70	19	0.59
				2004	6.60±0.83	0.10±0.48	14	0.62
				2005	6.49±0.87	0.11±0.48	17	0.63
				2006	7.24±0.55	-0.18±0.39	16	0.48
				2007	7.33±0.73	-0.24±0.51	19	0.48
				2008	6.99±0.56	0.01±0.44	15	0.56
				2009	6.24±0.43	0.35±0.27	15	0.61
NL-Hor	52.2404	5.0713	GRA	2004	3.39±3.05	0.26±0.74	14	1.07
				2005	3.16±2.84	0.97±0.73	27	1.25
				2006	1.58±1.64	0.23±0.18	9	0.81
				2007	3.60±3.73	0.23±1.00	30	1.24
				2008	3.43±3.53	0.04±0.40	30	0.87
				2009	5.48±4.38	-0.31±0.59	23	1.12
				2010	3.26±3.53	0.26±0.44	34	0.9
				2011	4.28±3.39	0.42±0.43	21	1.05
NL-Loo	52.1666	5.7436	ENF	1999	4.41±3.02	-0.56±0.87	25	1.3
				2000	4.59±3.17	-0.70±0.78	34	1.29
				2001	3.92±2.68	-0.83±0.71	31	1.19
				2002	3.95±2.72	-0.55±0.86	27	1.23
				2003	4.05±2.82	-0.73±0.86	31	1.34
				2004	4.64±3.18	-1.01±0.74	29	1.25

				2005	4.59±2.95	-0.98±0.94	31	1.22
				2006	3.71±2.65	-0.23±0.61	33	1.26
				2007	3.61±2.24	-0.21±0.59	25	1.12
				2008	4.23±2.76	-0.60±0.66	28	1.2
				2009	4.84±3.34	-0.86±0.80	8	1.35
				2010	3.79±2.90	-0.68±0.80	29	1.11
				2011	4.10±2.72	-0.51±0.81	31	1.06
				2012	4.33±3.13	-0.15±0.30	36	0.89
				2013	3.78±2.80	-0.07±0.34	29	0.87
				2014	4.76±2.55	0.12±0.37	28	0.96
NO-Adv	78.186	15.923	WET	2012	-0.13±0.00	0.20±0.00	1	0.3
				2013	1.67±1.62	-1.11±1.31	22	0.47
NO-Blv	78.9216	11.8311	SNO	2008	0.14±0.18	0.30±0.19	30	0.42
				2009	-0.03±0.05	0.17±0.07	7	0.34
PA-SPn	9.3181	-79.6346	DBF	2007	5.61±2.35	0.09±0.87	6	1.21
				2008	5.13±1.13	0.09±0.52	15	1.07
				2009	4.23±0.97	0.06±0.38	12	0.98
PA-SPs	9.3138	-79.6314	GRA	2007	7.30±2.28	0.01±0.86	18	1.13
				2008	6.42±3.18	-0.13±0.70	24	1.2
				2009	6.31±2.63	0.09±0.64	17	1.08
RU-Che	68.613	161.3414	WET	2002	1.93±2.06	-0.04±0.19	12	0.53
				2003	1.20±1.66	0.07±0.21	19	0.51
				2004	1.18±1.41	0.16±0.20	18	0.53
				2005	0.38±0.92	0.18±0.19	6	0.34
RU-Cok	70.8291	147.4943	OSH	2003	1.85±1.83	-0.05±0.24	14	0.59
				2004	2.91±1.16	-0.27±0.43	7	0.79
				2005	4.51±1.20	-0.53±0.46	6	1.11
				2006	2.03±1.30	0.05±0.26	4	0.6

				2007	2.58±1.42	0.21±0.13	7	0.77
				2008	1.84±1.74	0.02±0.30	13	0.7
				2009	1.84±1.99	-0.09±0.15	5	0.5
				2010	0.70±1.41	0.63±0.42	8	0.88
				2011	3.68±1.18	0.04±0.20	2	1.16
				2012	2.58±1.46	-0.16±0.14	7	0.75
				2013	1.39±1.92	0.07±0.41	9	0.66
RU-Fyo	56.4615	32.9221	ENF	1999	3.68±4.10	-0.81±1.33	34	1.03
				2000	4.00±3.93	-1.28±1.46	35	1.13
				2001	4.69±4.60	-1.33±1.48	32	1.08
				2002	1.90±3.00	0.25±1.44	26	0.74
				2003	2.89±3.24	-0.35±1.69	33	0.95
				2004	3.65±3.44	-0.18±0.92	33	1.07
				2005	4.19±4.46	-0.50±1.08	35	1.05
				2006	3.65±3.89	-0.40±0.82	34	1.1
				2007	3.40±3.60	-0.39±1.29	33	1.01
				2008	4.11±4.06	-0.69±1.08	32	1.07
				2009	4.65±4.53	-1.01±1.28	35	1.06
				2010	3.51±2.79	-0.00±1.68	24	1.17
				2011	4.75±4.08	-0.59±0.91	26	1.16
				2012	3.78±3.92	-0.11±0.53	35	0.85
				2013	3.74±3.99	-0.04±0.42	35	0.81
				2014	3.95±3.98	-0.19±0.40	35	0.82
RU-Ha1	54.7252	90.0022	GRA	2002	1.34±1.41	-0.01±0.21	18	0.46
				2003	2.59±1.75	-0.03±0.23	19	0.76
				2004	2.38±2.28	-0.09±0.18	23	0.63
SD-Dem	13.2829	30.4783	SAV	2005	5.36±1.46	-2.10±0.79	4	1.75
				2007	3.07±1.90	-0.06±0.24	14	0.91

				2008	1.67±1.96	-0.02±0.25	29	0.51
				2009	1.35±1.61	0.03±0.29	30	0.53
SN-Dhr	15.4028	-15.4322	SAV	2010	12.14±2.35	-1.55±1.33	6	2.44
				2011	5.31±3.78	0.20±1.31	3	1.63
				2012	2.46±2.94	-0.12±0.43	26	0.81
				2013	2.52±3.05	0.30±0.37	6	1.06
US-AR1	36.4267	-99.42	GRA	2009	1.99±2.36	-0.01±1.64	24	1.49
				2010	1.95±2.29	0.17±0.70	36	1.05
				2011	0.83±1.17	0.02±0.41	36	0.61
				2012	1.03±1.20	-0.03±0.39	24	0.59
US-AR2	36.6358	-99.5975	GRA	2009	1.45±1.14	0.05±0.45	22	0.7
				2010	1.40±2.13	-0.15±0.59	34	0.63
				2011	0.29±0.79	0.06±0.33	35	0.45
				2012	0.86±0.71	0.10±0.23	17	0.53
US-ARb	35.5497	-98.0402	GRA	2005	3.43±4.35	0.03±0.88	21	1.28
				2006	2.03±2.86	0.12±0.46	28	1
US-ARc	35.5465	-98.04	GRA	2005	4.49±3.80	0.09±0.46	29	1.14
				2006	2.56±2.83	0.15±0.36	29	1.02
US-ARM	36.6058	-97.4888	CRO	2003	1.33±1.99	0.65±0.87	34	1.23
				2004	2.16±2.23	0.33±1.10	30	1.41
				2005	1.03±1.35	0.78±1.28	31	1.33
				2006	1.56±2.25	-0.24±1.22	31	1.13
				2007	2.25±2.33	-0.03±1.52	28	1.44
				2008	3.20±4.44	0.18±2.19	20	1.58
				2009	2.00±2.05	-0.18±0.49	26	0.9
				2010	2.38±3.27	0.11±0.85	26	1.13
				2011	1.68±1.23	0.01±0.48	24	0.95
				2012	0.95±2.26	0.06±0.33	21	0.72

US-Atq	70.4696	-157.409	WET	2003	0.02±0.52	0.47±1.00	22	0.44
				2004	1.21±1.56	-0.45±0.62	27	0.46
				2005	0.64±1.20	-0.04±0.24	31	0.34
				2006	0.52±1.03	0.03±0.16	32	0.32
				2007	0.62±1.11	0.01±0.17	32	0.31
				2008	0.87±1.22	-0.09±0.19	24	0.35
US-Blo	38.8953	-120.633	ENF	1999	3.57±1.31	1.27±0.81	7	1.93
				2000	1.70±0.00	0.12±0.00	1	0.8
				2001	1.83±0.91	0.44±0.43	5	1.04
				2002	3.62±4.77	-0.36±1.21	3	1.26
				2003	2.36±0.77	0.51±0.28	3	1.11
				2004	3.43±2.57	0.06±0.43	5	1.12
				2005	4.77±2.78	0.04±0.66	17	1.4
				2006	2.89±2.09	0.22±0.12	2	1.07
US-Cop	38.09	-109.39	GRA	2001	0.30±0.26	0.18±0.26	24	0.42
				2002	0.14±0.17	0.02±0.08	27	0.19
				2003	0.35±0.51	-0.04±0.22	27	0.27
				2006	0.24±0.44	0.04±0.14	21	0.24
				2007	0.40±0.44	0.00±0.07	25	0.29
US-CRT	41.6285	-83.3471	CRO	2011	2.01±4.45	0.12±0.90	35	1.27
				2012	1.93±4.33	0.15±0.99	25	1.34
				2013	1.40±3.73	0.25±1.60	29	1.6
US-GBT	41.3658	-106.24	ENF	2001	0.98±0.40	-0.04±0.21	5	0.41
				2002	1.70±1.37	-0.00±0.42	26	0.68
				2003	1.74±1.98	0.11±0.48	23	0.89
US-GLE	41.3665	-106.24	ENF	2005	2.39±2.54	0.18±0.90	20	1.57
				2006	1.02±2.33	0.75±1.08	21	1.12
				2007	0.72±1.99	0.70±0.90	14	1.08

				2008	0.20±1.04	0.77±0.67	22	0.94
				2009	0.24±1.03	0.61±0.36	16	0.76
				2010	0.55±1.50	0.96±0.46	28	0.95
				2011	1.31±2.09	0.61±0.72	30	0.95
				2012	1.70±1.94	0.08±0.28	34	0.74
				2013	1.77±2.21	0.08±0.20	33	0.7
				2014	1.73±1.86	0.00±0.29	31	0.76
US-Goo	34.2547	-89.8735	GRA	2002	3.12±3.17	0.31±0.49	13	1.15
				2003	3.72±2.38	0.53±0.78	13	1.29
				2004	4.30±3.89	-0.09±0.54	28	1.18
				2005	2.30±2.39	0.10±0.23	22	0.84
				2006	3.55±3.11	0.15±0.54	31	1.14
US-Ha1	42.5378	-72.1715	DBF	1999	1.92±2.72	1.08±1.12	22	1.29
				2000	1.48±2.58	0.57±0.59	19	0.98
				2001	2.37±3.64	0.72±0.90	21	1.23
				2002	3.90±4.54	0.59±0.54	8	1.43
				2003	1.27±2.02	0.73±0.78	13	1.02
				2004	3.56±4.81	0.11±1.24	25	1.35
				2005	1.08±2.44	0.41±0.57	18	0.87
				2006	2.58±4.15	0.09±0.85	22	0.86
				2007	3.34±4.13	0.16±0.49	19	1.01
				2008	1.64±2.52	0.23±0.27	13	0.74
				2009	2.40±4.91	0.19±0.24	5	0.74
				2010	1.36±1.56	0.42±0.65	6	0.89
				2011	5.42±6.01	-0.30±1.20	10	1.33
				2012	1.11±1.67	0.20±0.24	17	0.56
US-IB2	41.8406	-88.241	GRA	2004	0.28±0.34	0.30±0.41	9	0.54
				2005	2.91±3.62	-0.07±0.68	30	1.18

				2006	2.55±4.26	0.15±1.53	29	1.01
				2007	2.74±3.74	0.51±1.15	29	1.21
				2008	2.82±3.95	-0.05±0.51	30	0.86
				2009	3.07±4.19	0.01±0.50	33	0.84
				2010	2.14±2.93	0.09±0.24	28	0.71
				2011	3.43±4.19	0.11±0.42	33	0.86
US-Ivo	68.4865	-155.75	WET	2004	0.43±1.14	0.21±0.25	32	0.48
				2005	0.84±1.32	0.10±0.20	30	0.52
				2006	0.97±1.36	-0.02±0.43	28	0.49
				2007	1.59±1.80	-0.04±0.31	22	0.56
US-KS1	28.4583	-80.6709	ENF	2002	5.30±1.26	0.21±0.28	21	1.04
US-KS2	28.6086	-80.6715	CSH	2003	6.04±1.62	-0.46±0.90	24	1.51
				2004	5.00±1.47	-0.01±0.41	36	0.9
				2005	4.97±1.26	0.10±0.38	36	0.9
				2006	4.69±1.04	0.25±0.38	36	0.88
US-Lin	36.3566	-119.842	CRO	2009	1.30±0.28	-0.22±0.16	5	0.51
				2010	1.37±0.46	0.61±0.47	21	1.53
US-Los	46.0827	-89.9792	WET	2000	0.13±0.24	0.60±0.49	5	0.78
				2001	0.34±0.87	0.43±0.48	24	0.72
				2002	1.20±2.22	0.86±1.13	31	0.93
				2003	0.78±1.81	0.74±0.80	26	0.87
				2004	1.46±2.44	0.69±0.72	30	1.08
				2005	1.33±2.50	0.35±0.38	31	0.73
				2006	1.12±2.22	0.25±0.27	29	0.63
				2007	4.63±2.53	0.59±0.15	5	1.43
				2008	5.05±2.97	0.40±0.47	8	1.48
				2010	5.18±2.76	0.54±0.60	7	1.48
				2014	2.04±3.06	0.18±0.34	31	0.68

US-Me1	44.5794	-121.5	ENF	2004	1.09±0.83	-0.04±0.14	16	0.43
				2005	1.33±0.96	-0.18±0.18	14	0.44
US-Me2	44.4523	-121.557	ENF	2002	3.87±2.76	1.02±1.12	24	1.73
				2003	4.90±2.89	0.79±1.60	10	1.82
				2004	4.99±2.45	-0.03±0.96	25	1.61
				2005	4.02±2.77	0.62±1.60	33	1.73
				2006	4.87±2.26	0.84±1.22	16	1.66
				2007	4.42±2.88	0.89±0.99	28	1.49
				2008	4.46±2.97	0.23±0.82	29	1.53
				2009	4.77±2.65	-0.24±0.88	33	1.49
				2010	4.42±2.75	0.02±0.49	28	1.1
				2011	6.40±2.93	0.02±0.64	18	1.27
				2012	7.53±2.66	-0.15±0.71	10	1.36
				2013	4.15±3.05	0.05±0.71	22	1.18
				2014	3.52±2.85	0.14±0.62	15	1.21
US-Me3	44.3154	-121.608	ENF	2004	2.50±1.65	-0.07±0.81	32	1.01
				2005	2.24±1.54	0.32±0.81	25	1.08
				2006	2.55±1.39	-0.17±0.48	26	0.7
				2007	2.44±1.37	0.11±0.33	27	0.7
				2008	2.30±1.41	0.00±0.30	35	0.67
				2009	2.44±1.58	0.08±0.32	26	0.69
US-Me4	44.4992	-121.622	ENF	1999	2.97±1.92	0.30±0.41	7	1.25
				2000	5.67±1.30	-0.26±0.75	17	1.4
US-Me5	44.4372	-121.567	ENF	2000	2.85±1.55	0.16±0.35	27	0.97
				2001	2.02±1.57	0.24±0.39	33	0.87
				2002	2.43±1.75	0.11±0.35	32	0.91
US-Me6	44.3233	-121.608	ENF	2010	4.38±1.87	-0.16±0.56	6	0.99
				2011	2.10±1.32	0.06±0.17	22	0.66

				2012	1.38±0.71	0.08±0.25	6	0.61
				2013	2.59±1.95	0.00±0.53	23	0.83
				2014	2.53±2.05	0.06±0.41	24	0.76
US-MMS	39.3232	-86.4131	DBF	1999	3.67±4.86	0.67±1.50	32	1.29
				2000	3.68±5.63	-0.20±1.96	25	1.12
				2001	2.68±4.18	0.47±1.18	26	1.14
				2002	3.15±4.40	0.29±1.02	29	1.16
				2003	3.43±4.82	-0.00±1.30	27	1.18
				2004	4.75±5.66	-0.24±1.55	31	1.26
				2005	3.71±4.78	0.43±1.45	31	1.3
				2006	3.51±4.83	0.28±1.31	30	1.19
				2007	2.41±3.53	0.90±1.53	26	1.24
				2008	3.04±4.30	0.41±0.86	29	1.25
				2009	3.88±5.44	0.07±1.63	32	1.21
				2010	2.59±3.92	0.79±1.32	28	1.35
				2011	3.33±4.51	0.11±1.00	29	1.13
				2012	3.58±4.43	0.30±0.60	29	1.01
				2013	3.93±4.58	0.28±0.55	34	0.87
				2014	3.93±5.08	0.02±0.46	33	0.83
US-Myb	38.0498	-121.765	WET	2011	2.38±1.80	1.02±1.25	19	2.13
				2012	7.84±5.71	-0.67±1.16	17	1.91
				2013	5.91±3.27	0.09±0.77	24	1.73
				2014	4.08±2.75	0.15±0.52	15	1.63
US-Ne1	41.1651	-96.4766	CRO	2001	4.01±6.15	-1.03±2.11	11	1.81
				2002	1.45±4.68	-0.74±3.25	23	0.87
				2003	1.49±4.41	-0.31±2.55	26	1.07
				2004	2.42±5.53	-0.31±1.26	23	1.22
				2005	1.22±3.47	-0.12±1.49	19	1.19

				2006	1.89±5.74	0.06±2.15	26	1.19
				2007	3.15±6.98	-0.42±2.35	27	1.51
				2008	1.54±4.45	-0.38±1.85	25	0.95
				2009	3.73±7.93	-0.28±0.82	26	0.93
				2010	2.14±5.70	-0.06±0.53	20	0.58
				2011	0.44±1.38	0.05±0.37	24	0.5
				2012	1.41±4.34	-0.09±0.47	20	0.61
				2013	-0.01±0.20	0.04±0.20	13	0.3
US-Ne2	41.1649	-96.4701	CRO	2001	12.86±7.30	-3.75±3.47	11	3.92
				2002	3.09±4.92	-0.09±3.01	18	2.02
				2003	3.05±7.22	-0.96±3.77	31	1.45
				2004	2.24±4.50	0.19±1.66	32	1.43
				2005	2.95±6.16	-0.65±1.98	32	1.41
				2006	2.24±4.68	0.90±1.39	34	1.66
				2007	2.24±5.15	0.17±1.69	30	1.48
				2008	1.17±2.95	0.93±1.96	29	1.21
				2009	1.95±5.32	-0.26±1.05	28	0.88
				2010	2.59±6.10	0.08±0.76	30	0.93
				2011	2.38±5.84	-0.08±0.61	29	0.81
				2012	3.11±6.56	-0.42±1.15	30	1.07
				2013	-0.02±0.33	0.28±0.48	15	0.53
US-Ne3	41.1797	-96.4397	CRO	2001	10.83±7.86	-5.44±5.05	13	2.97
				2002	1.06±2.96	0.31±1.54	31	1.22
				2003	2.99±6.56	-1.00±3.99	29	1.47
				2004	1.50±3.84	-0.14±1.45	29	1.1
				2005	3.30±6.29	-0.85±2.33	33	1.47
				2006	1.79±4.09	0.76±1.58	32	1.53
				2007	2.97±5.96	-0.29±2.52	33	1.58

				2008	1.63±3.65	0.82±1.93	32	1.3
				2009	3.04±6.80	-0.38±1.37	31	1.2
				2010	2.30±4.82	0.32±1.14	33	1.15
				2011	2.61±6.01	-0.22±0.72	32	0.94
				2012	1.03±2.77	0.28±0.59	26	0.95
				2013	0.01±0.17	0.16±0.43	15	0.47
US-NR1	40.0329	-105.546	ENF	1999	2.99±2.48	0.29±0.65	19	1.4
				2000	2.12±2.37	0.42±0.74	33	1.34
				2001	1.97±2.32	0.12±0.57	31	1.33
				2002	1.82±2.17	0.45±0.57	32	1.47
				2003	1.69±2.03	0.61±0.57	30	1.4
				2004	2.13±2.40	0.55±0.66	30	1.63
				2005	2.05±2.31	0.73±0.60	33	1.61
				2006	1.95±2.37	0.57±0.74	29	1.45
				2007	2.01±2.36	0.32±0.41	30	1.26
				2008	1.81±2.23	0.44±0.60	32	1.29
				2009	1.76±2.22	0.43±0.69	28	1.31
				2010	1.82±2.32	0.56±0.63	32	1.19
				2011	1.85±2.45	0.31±0.48	32	1.15
				2012	2.32±2.53	0.20±0.42	33	0.96
				2013	2.06±2.58	0.18±0.24	33	0.91
				2014	1.57±2.26	0.23±0.30	28	0.86
US-Oho	41.5545	-83.8438	DBF	2004	5.03±5.70	-0.61±1.25	29	1.26
				2005	3.52±4.24	-0.14±0.76	25	1.16
				2006	4.80±5.49	-0.19±0.92	34	1.21
				2007	4.31±5.53	-0.40±1.49	23	1.29
				2008	4.66±5.15	-0.02±1.26	23	1.32
				2009	4.41±5.62	-0.29±1.35	30	1.15

				2010	2.42±3.76	0.17±0.49	26	0.78
				2011	3.67±4.95	-0.07±0.35	30	0.72
				2012	4.00±5.12	0.07±0.40	31	0.79
				2013	3.24±4.89	0.03±0.65	27	0.8
US-ORv	40.0201	-83.0183	WET	2011	0.47±0.46	0.30±0.30	16	0.72
US-PFa	45.9459	-90.2723	MF	1999	2.75±3.53	0.07±0.77	31	1.25
				2000	2.31±2.97	0.36±0.47	33	1.17
				2001	1.85±2.59	0.18±0.46	29	1.02
				2002	1.81±3.31	-0.11±0.93	27	0.87
				2003	2.52±2.89	0.67±1.03	27	1.27
				2004	2.00±2.65	0.33±0.72	31	1.08
				2005	2.06±2.82	0.26±0.81	20	1.13
				2006	1.15±1.43	0.70±1.33	25	0.87
				2007	1.69±2.14	1.16±1.30	31	1.23
				2008	1.35±2.15	0.80±0.96	31	1
				2009	1.44±2.20	0.58±0.82	30	0.99
				2010	0.25±0.45	0.70±0.89	22	0.73
				2011	1.36±2.21	0.54±0.67	30	0.88
				2012	1.09±1.98	0.49±0.66	28	0.85
				2013	1.89±3.13	0.05±0.33	27	0.66
				2014	1.77±2.81	0.19±0.42	31	0.69
US-Prr	65.1237	-147.488	ENF	2010	-0.01±0.06	-0.02±0.04	8	0.1
				2011	0.81±1.58	0.11±0.22	18	0.38
				2012	0.90±1.46	0.14±0.27	12	0.43
				2013	0.07±0.19	0.06±0.15	19	0.21
				2014	1.42±1.85	0.15±0.27	24	0.63
US-SRC	31.9083	-110.84	OSH	2008	0.54±0.92	0.21±0.35	20	0.41
				2009	0.34±0.27	0.02±0.15	33	0.26

				2010	0.86±0.61	-0.30±0.34	33	0.35
				2011	0.16±0.29	0.04±0.08	34	0.2
				2012	0.43±0.31	-0.02±0.10	28	0.2
				2013	0.26±0.18	0.02±0.08	20	0.16
				2014	0.53±0.29	0.02±0.11	15	0.23
US-SRG	31.7894	-110.828	GRA	2008	1.89±2.43	0.05±0.78	28	0.9
				2009	0.54±1.03	-0.04±0.61	35	0.47
				2010	1.57±2.00	-0.08±0.61	36	0.8
				2011	0.84±1.10	0.01±0.59	36	0.54
				2012	1.45±1.64	-0.15±0.35	36	0.48
				2013	0.81±1.10	0.01±0.20	36	0.38
				2014	1.23±1.49	0.07±0.27	36	0.51
US-SRM	31.8214	-110.866	WSA	2004	0.49±0.62	-0.08±0.24	33	0.41
				2005	0.79±1.05	-0.11±0.31	34	0.51
				2006	0.61±1.17	0.13±0.29	35	0.52
				2007	0.59±0.90	0.17±0.29	30	0.46
				2008	0.81±1.54	0.05±0.41	31	0.54
				2009	0.34±0.53	0.03±0.31	36	0.41
				2010	0.96±1.16	0.01±0.51	36	0.6
				2011	0.76±1.29	0.11±0.34	35	0.45
				2012	0.95±0.94	-0.08±0.20	34	0.34
				2013	0.70±0.94	-0.01±0.16	29	0.3
				2014	0.68±0.82	0.08±0.14	28	0.32
US-Sta	41.3966	-106.802	OSH	2005	0.45±0.12	-0.05±0.06	5	0.15
				2006	0.24±0.12	0.06±0.11	2	0.14
				2007	1.07±0.80	-0.06±0.20	26	0.35
				2008	1.08±0.95	-0.04±0.21	33	0.37
				2009	1.18±1.19	-0.09±0.32	32	0.38

US-Syv	46.242	-89.3477	MF	2001	1.19±1.94	0.90±1.00	11	1
				2002	1.82±2.93	0.64±0.69	28	1
				2003	2.52±3.45	1.08±0.96	23	1.1
				2004	3.20±3.71	0.55±0.94	28	1.34
				2005	2.65±3.91	0.33±0.64	21	1.09
				2006	2.98±3.57	0.28±0.29	31	0.87
				2007	8.91±4.08	-0.20±1.10	4	1.67
				2012	4.44±4.16	-0.08±0.54	16	1.02
				2013	3.72±4.47	-0.27±0.46	19	0.9
				2014	3.32±4.51	-0.06±0.44	33	0.79
US-Ton	38.4316	-120.966	WSA	2001	1.15±0.76	0.28±0.65	18	0.94
				2002	2.13±1.80	-0.21±0.91	30	1
				2003	2.28±2.28	-0.10±0.82	26	1.24
				2004	2.33±1.86	-0.05±0.86	22	1.1
				2005	2.47±1.74	-0.10±1.66	18	1.2
				2006	2.30±1.93	-0.23±0.90	27	1.07
				2007	2.26±2.44	-0.12±1.14	26	1.15
				2008	2.05±1.30	-0.24±0.63	29	1.08
				2009	2.54±1.95	-0.32±1.01	32	1.13
				2010	2.89±1.69	-0.43±1.00	30	1.28
				2011	2.65±1.36	-0.26±0.42	23	0.87
				2012	2.34±1.87	-0.13±0.33	29	0.76
				2013	1.45±1.47	0.07±0.37	25	0.62
				2014	1.85±2.20	-0.01±0.53	27	0.78
US-Tw1	38.1074	-121.647	WET	2012	10.86±2.28	-0.86±0.89	6	1.93
				2013	7.54±5.05	-1.57±1.50	19	2.55
				2014	7.85±3.26	-1.78±1.39	16	2.38
US-Tw2	38.1047	-121.643	CRO	2012	5.00±5.62	0.23±0.75	22	1.69

				2013	1.69±1.33	-0.02±0.55	9	1.03
US-Tw3	38.1159	-121.647	CRO	2013	8.79±5.26	-0.56±1.83	14	2.43
				2014	9.15±4.24	-0.58±1.37	20	2.47
US-Tw4	38.103	-121.641	WET	2013	-0.11±0.48	0.04±0.32	2	0.3
				2014	2.34±1.85	1.84±1.65	23	1.85
US-Twt	38.1087	-121.653	CRO	2009	5.47±4.60	1.21±4.21	21	3.48
				2010	5.22±5.52	0.92±2.77	27	2.93
				2011	4.14±5.27	0.38±0.82	30	1.73
				2012	4.02±4.68	0.45±1.07	29	1.64
				2013	6.00±6.30	-0.66±1.80	25	2.19
				2014	6.24±6.37	0.31±1.44	22	2.19
US-UMB	45.5598	-84.7138	DBF	2000	2.17±3.41	0.72±1.01	27	0.94
				2001	1.54±2.60	0.78±1.12	27	0.85
				2002	2.08±3.54	0.45±0.75	28	0.73
				2003	1.20±2.54	0.37±0.65	25	0.56
				2004	1.55±3.32	0.30±0.47	24	0.59
				2005	2.42±4.11	0.37±0.66	17	0.75
				2006	1.66±3.34	0.23±0.59	19	0.62
				2007	2.02±3.60	0.26±0.72	25	0.68
				2008	2.89±4.17	0.07±0.58	30	0.82
				2009	0.73±1.83	0.22±0.43	21	0.51
				2010	1.60±2.65	0.12±0.36	26	0.67
				2011	1.34±2.89	-0.02±0.25	25	0.36
				2012	2.77±4.09	0.08±0.18	26	0.54
				2013	1.81±3.39	0.14±0.31	26	0.47
				2014	1.97±3.46	0.06±0.14	25	0.46
US-UMd	45.5625	-84.6975	DBF	2007	4.48±4.15	0.63±1.62	17	1.21
				2008	3.39±4.40	0.16±0.68	34	0.93

				2009	3.17±4.28	0.23±0.67	34	0.99
				2010	2.88±3.90	0.30±0.57	32	0.98
				2011	1.94±2.98	0.23±0.25	29	0.61
				2012	3.80±4.61	0.02±0.41	33	0.67
				2013	2.94±3.86	0.17±0.51	31	0.68
				2014	3.75±4.49	-0.03±0.55	27	0.93
US-Var	38.4133	-120.951	GRA	2000	4.02±2.18	-2.47±2.02	6	0.86
				2001	2.32±2.96	-0.37±1.42	34	1.47
				2002	1.69±2.79	0.04±1.19	36	1.24
				2003	2.31±3.33	-0.27±1.35	34	1.48
				2004	1.53±2.53	0.02±1.08	34	1.24
				2005	2.17±3.92	-0.24±1.12	27	1.44
				2006	1.76±2.39	0.04±0.90	33	1.31
				2007	1.96±3.13	-0.24±1.03	33	1.37
				2008	0.90±1.45	0.27±0.30	31	1.17
				2009	1.90±2.97	0.05±1.06	35	1.42
				2010	1.75±2.73	0.14±1.13	30	1.5
				2011	1.83±2.12	0.31±0.96	35	1.26
				2012	1.39±2.39	0.10±0.55	32	0.93
				2013	1.30±2.38	0.02±0.39	36	0.77
				2014	0.53±1.61	0.10±0.38	26	0.73
US-WCr	45.8059	-90.0799	DBF	1999	1.56±2.78	0.91±1.45	27	0.99
				2000	2.97±4.20	0.32±1.06	32	1.17
				2001	2.12±4.08	0.24±1.31	27	0.94
				2002	1.01±2.47	0.38±0.63	23	0.59
				2003	2.34±3.92	0.55±0.88	30	0.97
				2004	1.16±2.72	0.26±0.23	21	0.7
				2005	2.78±4.72	-0.07±1.05	31	0.99

				2006	2.85±4.58	0.17±0.78	26	1.03
				2010	0.18±0.23	-0.16±0.24	4	0.17
				2011	1.45±2.75	0.13±0.39	26	0.52
				2012	3.01±3.80	0.48±0.54	31	0.94
				2013	3.88±5.18	-0.04±0.78	32	0.92
				2014	2.21±4.52	-0.04±0.51	20	0.65
US-Whs	31.7438	-110.052	OSH	2007	0.43±0.80	0.20±0.24	18	0.38
				2008	0.41±1.04	0.03±0.26	36	0.32
				2009	0.18±0.44	-0.03±0.19	36	0.28
				2010	0.54±0.76	-0.03±0.34	36	0.41
				2011	0.23±0.66	0.03±0.21	36	0.27
				2012	0.18±0.53	0.04±0.09	36	0.2
				2013	0.34±0.75	0.02±0.09	36	0.2
				2014	0.59±0.84	-0.03±0.12	35	0.25
US-Wi0	46.6188	-91.0814	ENF	2002	4.62±3.57	0.18±0.47	20	1.09
US-Wi1	46.7305	-91.2329	DBF	2003	5.31±4.09	0.09±1.10	5	1.55
US-Wi2	46.6869	-91.1528	ENF	2003	2.78±2.30	-0.85±0.57	2	0.95
US-Wi3	46.6347	-91.0987	DBF	2002	6.57±3.80	-0.19±0.36	17	1.17
				2004	4.50±4.24	0.13±0.59	21	1.07
US-Wi4	46.7393	-91.1663	ENF	2002	6.48±2.45	-0.10±0.30	17	1.18
				2003	9.35±1.67	-0.27±0.87	4	1.1
				2004	5.81±2.85	0.06±0.48	20	1.15
				2005	7.20±2.11	-0.18±0.77	17	1.25
US-Wi5	46.6531	-91.0858	ENF	2004	4.25±3.20	0.46±0.58	22	1.3
US-Wi6	46.6249	-91.2982	OSH	2002	2.05±2.37	0.97±0.83	17	1.53
US-Wi7	46.6491	-91.0693	OSH	2005	3.19±2.61	1.24±0.76	13	2.12
US-Wi8	46.7223	-91.2524	DBF	2002	3.84±2.78	1.06±0.85	18	1.69
US-Wi9	46.6188	-91.0814	ENF	2004	2.07±1.71	0.22±0.27	7	0.89

				2005	3.57±1.65	1.44±0.73	6	2.22
US-Wkg	31.7365	-109.942	GRA	2004	0.48±0.65	-0.14±0.41	21	0.46
				2005	0.11±0.27	0.05±0.22	33	0.35
				2006	0.32±0.94	0.19±0.25	34	0.42
				2007	0.43±0.57	0.04±0.49	33	0.38
				2008	0.17±0.35	0.02±0.35	29	0.23
				2009	0.43±0.73	-0.22±0.44	36	0.33
				2010	0.63±1.12	-0.12±0.47	29	0.42
				2011	0.18±0.57	0.08±0.14	31	0.23
				2012	0.61±0.96	-0.12±0.20	33	0.24
				2013	0.34±0.64	0.01±0.13	34	0.21
				2014	0.52±1.08	0.03±0.13	29	0.28
US-WPT	41.4646	-82.9962	WET	2011	1.81±3.13	-0.12±0.35	27	0.62
				2012	1.08±2.21	0.21±0.51	28	0.71
				2013	1.18±2.37	0.27±0.39	31	0.67
ZM-Mon	-15.4378	23.2528	DBF	2000	5.01±0.00	0.55±0.00	1	1.15
				2007	4.91±3.24	0.01±0.55	8	1.16
				2008	4.20±2.66	-0.10±0.41	21	1.04
				2009	4.93±3.61	-0.10±0.50	6	1.07

Table S4. Statistics of site RECO fitting.

Site ID	Latitude	Longitude	IGBP	Year	Observed Flux (gC m-2 d-1)	Prediction Bias (gC m-2 d-1)	ND	Model SD （gC m-2 d-1)
AR-SLu	-33.4648	-66.4598	MF	2009	2.40±0.00	0.85±0.00	1	1.14
				2010	3.37±0.79	-0.18±0.31	32	0.76
				2011	4.25±0.77	-0.25±0.41	8	0.87
AR-Vir	-28.2395	-56.1886	ENF	2010	11.93±3.07	-0.70±1.38	7	3.02
				2011	12.89±3.62	-1.74±2.47	4	3.31
				2012	12.88±4.73	-1.00±2.28	9	3.36
AT-Neu	47.1167	11.3175	GRA	2002	6.59±4.92	-0.60±2.06	35	2.41
				2003	6.32±4.91	-0.84±2.84	34	2.47
				2004	4.92±4.16	0.16±1.66	21	2.16
				2005	6.00±4.14	0.05±1.96	35	2.37
				2006	6.63±4.02	-0.49±1.85	25	2.49
				2007	6.53±3.78	-0.31±1.18	23	1.76
				2008	4.39±3.37	-0.35±0.93	19	1.21
				2009	6.59±6.70	-1.00±2.05	15	1.83
				2010	4.08±2.94	-0.07±0.72	16	1.23
				2011	6.59±5.14	-0.79±1.18	13	1.75
				2012	7.16±4.30	-0.63±0.89	28	1.8
AU-Ade	-13.0769	131.1178	WSA	2007	6.22±0.83	-0.87±0.22	6	1.15
				2008	3.10±1.76	0.01±0.37	35	0.74
				2009	4.38±1.70	0.09±0.33	14	0.9
AU-ASM	-22.283	133.249	ENF	2010	1.49±0.37	-0.25±0.21	11	0.53
				2011	1.31±1.30	-0.18±0.84	33	0.54
				2012	0.65±0.59	-0.04±0.18	35	0.27
				2013	0.25±0.20	0.05±0.08	34	0.2
				2014	0.77±0.67	0.00±0.16	31	0.28

AU-Cpr	-34.0021	140.5891	SAV	2010	1.12±0.51	-0.18±0.31	15	0.56
				2011	1.48±0.69	-0.02±0.37	36	0.91
				2012	1.10±0.51	0.06±0.17	36	0.46
				2013	0.94±0.40	0.06±0.20	35	0.38
				2014	0.52±0.23	0.04±0.12	27	0.24
AU-Cum	-33.6152	150.7236	EBF	2012	2.53±0.56	0.32±0.25	7	0.77
				2013	1.95±0.80	0.41±0.24	28	0.84
				2014	3.64±1.20	-0.23±0.33	32	0.82
AU-DaP	-14.0633	131.3181	GRA	2007	4.10±2.59	-0.89±0.94	3	1.24
				2008	2.88±2.32	0.09±1.51	32	1.15
				2009	2.61±2.53	-0.40±1.01	30	0.98
				2010	3.32±2.28	-0.08±0.43	26	0.89
				2011	3.32±2.11	-0.12±0.58	33	0.94
				2012	2.40±2.53	0.22±0.40	19	0.87
				2013	2.91±2.84	0.29±0.57	25	0.93
AU-DaS	-14.1593	131.3881	SAV	2008	2.88±1.69	0.33±0.93	21	1.22
				2009	2.69±1.73	0.14±1.31	25	1.22
				2010	3.57±1.29	-0.20±0.78	23	1.29
				2011	4.22±2.24	-0.41±0.97	27	1.08
				2012	3.84±1.53	-0.18±0.49	16	0.94
				2013	3.85±1.87	0.01±0.67	35	0.99
				2014	3.07±1.33	0.23±0.51	30	1
AU-Dry	-15.2588	132.3706	SAV	2008	1.94±0.60	-0.02±0.57	9	0.88
				2009	2.36±1.41	0.13±0.84	25	1.09
				2010	2.68±1.04	0.03±0.32	27	0.78
				2011	2.44±1.02	0.19±0.37	16	0.74
				2012	2.61±1.51	-0.00±0.48	32	0.71
				2013	2.97±0.71	0.14±0.29	12	0.74

				2014	2.08±1.09	0.22±0.30	32	0.72
AU-Emr	-23.8587	148.4746	GRA	2011	1.37±0.35	0.05±0.18	16	0.43
				2012	1.53±0.47	0.09±0.21	34	0.57
				2013	1.27±0.48	0.16±0.21	34	0.57
AU-Fog	-12.5452	131.3072	WET	2006	1.37±0.86	0.64±0.37	16	1.45
				2007	0.73±0.55	0.64±0.57	23	1.18
				2008	0.72±0.71	0.43±0.31	15	1.07
AU-Gin	-31.3764	115.7138	WSA	2011	3.33±0.21	-0.21±0.21	7	0.55
				2012	2.80±0.60	-0.05±0.22	29	0.52
				2013	2.05±0.80	0.08±0.28	30	0.55
				2014	2.17±0.70	0.14±0.28	35	0.55
AU-GWW	-30.1913	120.6541	SAV	2013	0.81±0.43	0.05±0.14	35	0.3
				2014	1.02±0.58	-0.01±0.21	31	0.36
AU-How	-12.4943	131.1523	WSA	2001	3.25±1.67	0.64±0.45	7	1.29
				2002	1.32±0.60	1.62±0.64	5	1.03
				2003	2.60±1.88	1.83±2.27	10	1.29
				2004	2.21±1.09	1.15±1.19	19	1.15
				2005	3.04±1.36	0.83±0.68	17	1.2
				2006	2.63±1.24	1.05±0.55	11	1.15
				2007	2.23±1.17	0.89±0.77	10	1.16
				2008	2.45±0.94	0.86±0.81	18	1.13
				2009	3.97±1.69	0.66±0.83	10	1.1
				2010	4.01±1.34	-0.03±0.44	11	0.86
				2011	3.81±1.79	-0.01±0.37	26	0.79
				2012	3.50±1.58	0.11±0.30	26	0.76
				2013	3.98±1.59	0.05±0.38	25	0.83
				2014	4.11±1.91	-0.05±0.57	19	0.91
AU-Lox	-34.4704	140.6551	DBF	2008	4.89±1.37	-0.39±0.35	11	1.03

				2009	3.82±2.07	-0.43±0.56	15	1.01
AU-RDF	-14.5636	132.4776	WSA	2011	4.29±1.04	-0.21±0.24	7	0.87
				2012	2.10±1.94	0.04±0.45	14	0.79
				2013	1.09±0.45	0.27±0.16	6	0.63
AU-Rig	-36.6499	145.5759	GRA	2011	2.83±0.99	-0.07±0.42	32	0.75
				2012	2.22±0.89	0.06±0.23	35	0.61
				2013	1.94±1.31	0.22±0.28	17	0.73
				2014	2.35±1.07	0.13±0.23	26	0.68
AU-Rob	-17.1175	145.6301	EBF	2014	3.01±1.75	0.59±1.08	7	1.97
AU-Stp	-17.1507	133.3502	GRA	2008	0.48±0.48	0.15±0.31	13	0.35
				2009	0.37±0.43	0.71±0.52	27	0.54
				2010	1.01±0.86	0.12±0.35	35	0.54
				2011	1.28±0.71	0.52±0.58	32	0.73
				2012	1.04±0.64	0.10±0.14	33	0.41
				2013	1.04±0.79	0.05±0.20	30	0.38
				2014	1.18±0.80	0.09±0.22	34	0.43
AU-TTE	-22.287	133.64	OSH	2012	0.42±0.40	-0.03±0.12	17	0.22
				2013	0.28±0.31	0.03±0.12	35	0.2
				2014	0.66±0.80	0.03±0.16	34	0.3
AU-Tum	-35.6566	148.1517	EBF	2001	7.62±4.03	-0.90±2.78	31	2.43
				2002	7.04±5.20	-0.09±4.10	31	2.7
				2003	5.41±4.41	-0.08±3.84	34	2.96
				2004	7.77±3.61	-1.36±2.74	23	3.52
				2005	7.85±3.12	-1.99±2.32	34	3.15
				2006	6.99±2.37	-0.43±1.68	35	3.1
				2007	7.91±3.86	-2.35±2.75	36	3.08
				2008	7.05±3.20	-0.22±1.69	36	2.84
				2009	7.53±3.42	0.44±2.32	36	2.95

				2010	7.87±2.79	-0.10±2.16	29	2.65
				2011	7.45±3.51	-0.12±1.48	32	2.22
				2012	7.63±4.65	-0.12±1.74	22	1.92
				2013	8.05±3.73	-0.16±1.72	36	2.15
				2014	9.13±3.84	-0.65±1.07	36	2.14
AU-Wac	-37.4259	145.1878	EBF	2005	3.65±2.26	-0.46±1.14	8	1.9
				2006	1.23±1.16	0.30±0.50	25	1.31
				2007	1.10±1.25	0.46±0.70	31	1.55
				2008	0.72±1.17	0.46±0.55	20	1.25
AU-Whr	-36.6732	145.0294	EBF	2011	2.71±0.40	0.20±0.32	3	0.69
				2012	2.44±0.95	0.04±0.28	36	0.59
				2013	2.31±0.84	0.08±0.29	36	0.59
				2014	2.68±0.85	0.05±0.30	36	0.72
AU-Wom	-37.4222	144.0944	EBF	2010	3.66±1.63	-0.43±1.07	26	1.8
				2011	2.86±1.71	0.43±1.00	29	1.51
				2012	2.79±1.19	0.13±0.38	31	1.13
				2013	2.70±1.50	0.08±0.52	31	1.05
				2014	2.79±1.21	0.27±0.73	30	1.27
AU-Ync	-34.9893	146.2907	GRA	2012	0.49±0.20	0.05±0.16	5	0.25
				2013	0.80±0.40	0.16±0.20	28	0.46
				2014	0.42±0.14	0.07±0.07	6	0.23
BE-Bra	51.3076	4.5198	MF	1999	4.01±1.17	-0.33±0.41	18	1.31
				2000	3.50±1.10	-0.21±0.26	26	1.2
				2001	3.18±1.23	0.75±0.53	26	1.37
				2002	2.64±0.89	1.04±0.68	26	1.28
				2004	2.82±0.63	0.48±0.85	24	1.2
				2005	2.28±0.97	1.38±1.00	31	1.27
				2006	3.35±1.61	0.20±1.06	30	1.31

				2007	3.44±1.36	0.41±0.96	29	1.31
				2008	2.99±1.24	0.77±0.92	27	1.26
				2009	3.34±1.46	0.20±0.81	29	1.29
				2010	3.26±1.05	0.05±0.62	31	1.09
				2011	4.45±1.65	-0.58±0.54	26	1.01
				2012	3.88±1.24	-0.39±0.37	23	0.91
				2013	3.52±1.76	0.01±0.49	25	0.95
				2014	2.81±1.26	0.40±0.32	26	0.98
BE-Lon	50.5516	4.7461	CRO	2004	3.13±1.44	0.06±0.64	23	1.46
				2005	3.13±2.28	-0.51±1.33	35	1.26
				2006	2.06±1.57	0.61±0.72	35	1.29
				2007	3.37±2.40	-0.45±1.62	36	1.35
				2008	2.38±1.35	0.33±0.93	35	1.25
				2009	3.54±2.25	-0.89±1.30	33	1.18
				2010	1.96±1.10	0.56±0.81	32	1.27
				2011	2.93±2.05	-0.17±1.14	33	1.2
				2012	3.24±3.40	-0.26±1.34	35	1.12
				2013	2.97±2.49	-0.20±0.64	35	0.89
				2014	1.72±0.96	0.46±0.35	34	0.93
BE-Vie	50.305	5.9981	MF	1999	2.41±1.47	0.76±0.65	21	0.92
				2000	3.05±1.77	0.54±0.72	30	1.04
				2001	2.77±1.87	0.82±1.00	31	1.09
				2002	2.53±1.39	0.99±0.70	28	1.05
				2003	2.46±1.59	1.10±0.89	23	1.16
				2004	2.65±1.29	0.48±0.54	22	1.02
				2005	3.30±1.84	0.58±0.81	31	1.14
				2006	2.57±1.36	1.15±1.13	29	1.08
				2007	2.68±1.63	1.26±0.71	31	1.09

				2008	2.63±1.52	0.51±0.78	24	1
				2009	2.84±2.08	0.07±0.76	19	0.85
				2010	3.86±2.45	-0.15±0.30	26	0.8
				2011	4.03±2.32	-0.20±0.47	22	0.78
				2012	4.49±2.38	-0.30±0.34	26	0.87
				2013	3.76±1.66	-0.05±0.48	19	0.87
				2014	2.92±1.07	0.07±0.38	16	0.68
BR-Sa1	-2.8567	-54.9589	EBF	2002	9.67±1.37	-0.39±0.55	17	0.82
				2003	9.11±1.22	0.09±0.68	16	0.82
				2004	8.68±1.50	0.23±0.84	13	0.9
				2005	9.16±2.30	-0.04±1.05	9	0.95
				2006	10.74±0.00	-0.40±0.00	1	0.77
				2008	8.42±0.41	0.03±0.43	8	0.78
				2009	9.20±1.00	-0.14±0.50	14	0.81
				2010	9.39±1.16	-0.24±0.61	12	0.91
				2011	8.26±1.30	0.35±0.59	16	0.93
BR-Sa3	-3.018	-54.9714	EBF	2000	6.96±0.62	0.40±0.63	10	1.09
				2001	7.55±1.59	0.14±0.42	12	0.85
				2002	8.79±1.98	-0.36±0.57	12	0.89
				2003	8.01±1.41	-0.06±0.49	18	0.85
				2004	9.27±1.23	-0.30±1.08	2	1.17
CA-Gro	48.2167	-82.1556	MF	2003	1.96±0.99	0.09±0.56	4	0.9
				2004	2.48±2.15	-0.30±0.75	29	0.89
				2005	3.18±2.18	-0.71±0.60	30	1.05
				2006	2.96±2.90	-0.86±1.23	33	0.87
				2007	2.94±2.59	-0.52±0.85	33	0.94
				2008	3.03±2.77	-0.86±1.18	32	0.88
				2009	2.05±1.93	-0.06±0.42	32	0.92

				2010	1.92±1.71	0.24±0.53	33	0.89
				2011	1.28±1.31	0.13±0.18	26	0.58
				2012	1.89±1.64	0.08±0.27	32	0.6
				2013	2.15±1.77	0.15±0.27	29	0.72
				2014	0.54±0.26	0.11±0.16	14	0.33
CA-Man	55.8796	-98.4808	ENF	1999	2.10±1.97	0.08±0.40	28	0.82
				2000	1.41±1.25	-0.03±0.37	30	0.61
				2001	1.57±1.48	0.23±0.38	32	0.72
				2002	1.39±1.47	0.11±0.20	32	0.46
				2003	1.52±1.55	0.04±0.18	29	0.45
				2004	0.33±0.26	-0.04±0.16	5	0.18
				2006	1.51±1.42	-0.02±0.09	10	0.34
				2007	1.11±0.62	0.03±0.27	11	0.37
				2008	1.77±1.84	0.07±0.32	24	0.55
CA-NS1	55.8792	-98.4839	ENF	2002	2.66±1.64	-0.12±0.27	14	0.62
				2003	1.40±1.35	-0.05±0.15	27	0.32
				2004	1.33±1.10	-0.10±0.16	25	0.3
				2005	1.77±1.56	-0.10±0.24	22	0.41
CA-NS2	55.9058	-98.5247	ENF	2001	1.56±1.42	0.20±0.26	15	0.62
				2002	1.13±1.33	0.14±0.17	31	0.39
				2003	1.36±1.04	0.15±0.25	24	0.4
				2004	1.00±0.89	0.11±0.20	29	0.33
				2005	2.19±1.12	0.32±0.21	11	0.67
CA-NS3	55.9117	-98.3822	ENF	2001	1.98±1.69	0.04±0.18	15	0.78
				2002	1.04±0.89	0.27±0.46	36	0.54
				2003	1.17±0.97	0.02±0.38	30	0.28
				2004	1.31±1.33	-0.18±0.23	32	0.3
				2005	2.57±2.58	-0.56±0.71	26	0.55

CA-NS4	55.9144	-98.3806	ENF	2002	0.20±0.13	0.20±0.03	6	0.16
				2003	2.28±1.61	-0.06±0.43	19	0.5
				2004	1.20±1.00	0.33±0.21	22	0.37
				2005	1.38±1.30	0.61±0.60	27	0.54
CA-NS5	55.8631	-98.485	ENF	2001	1.48±1.69	0.09±0.31	13	0.64
				2002	1.44±1.72	0.22±0.22	29	0.52
				2003	2.46±2.14	0.02±0.25	27	0.62
				2004	1.66±1.94	-0.04±0.41	36	0.45
				2005	2.14±1.96	0.07±0.21	27	0.54
CA-NS6	55.9167	-98.9644	OSH	2001	0.50±0.40	0.21±0.14	7	0.37
				2002	1.11±1.18	0.32±0.51	34	0.56
				2003	1.15±1.12	0.19±0.27	35	0.5
				2004	1.01±0.98	0.07±0.14	32	0.37
				2005	1.47±1.39	0.23±0.25	26	0.61
CA-NS7	56.6358	-99.9483	OSH	2002	1.81±1.51	-0.18±0.28	15	0.41
				2003	1.10±1.25	-0.06±0.21	27	0.32
				2004	1.17±1.08	-0.04±0.17	34	0.33
				2005	1.31±1.12	0.07±0.14	24	0.4
CA-Oas	53.6289	-106.198	DBF	1999	2.56±2.56	-0.25±0.72	34	0.78
				2000	2.28±2.05	-0.16±0.47	34	0.81
				2001	2.19±2.15	0.06±0.65	33	0.8
				2002	2.17±2.15	0.10±0.50	35	0.88
				2003	2.17±2.13	0.13±0.56	34	0.8
				2004	2.09±1.92	-0.12±0.46	34	0.69
				2005	2.49±2.30	-0.14±0.63	35	0.81
				2006	2.96±2.73	-0.47±0.73	33	0.8
				2007	2.80±2.41	-0.47±0.58	34	0.78
				2008	2.57±2.40	-0.19±0.29	34	0.55

				2009	2.49±2.37	-0.10±0.27	34	0.54
				2010	2.55±2.26	-0.09±0.27	34	0.55
CA-Obs	53.9872	-105.118	ENF	1999	2.58±1.84	0.10±0.40	22	0.87
				2000	1.79±1.86	0.09±0.30	32	0.77
				2001	1.17±1.14	0.20±0.24	25	0.62
				2002	1.52±1.80	0.22±0.41	32	0.7
				2003	1.35±1.47	0.30±0.40	27	0.76
				2004	1.58±1.62	-0.00±0.28	29	0.63
				2005	1.77±1.83	0.18±0.37	31	0.69
				2006	2.00±1.97	0.28±0.34	30	0.78
				2007	2.19±2.10	-0.04±0.38	35	0.72
				2008	2.03±1.90	-0.07±0.21	33	0.5
				2009	2.40±2.25	-0.14±0.23	31	0.57
				2010	2.54±2.29	-0.05±0.27	32	0.57
CA-Qfo	49.6925	-74.3421	ENF	2003	1.30±0.90	0.42±0.53	9	0.74
				2004	1.63±1.43	0.30±0.37	30	0.72
				2005	1.92±1.61	0.14±0.39	36	0.74
				2006	1.92±1.64	0.07±0.37	36	0.7
				2007	1.70±1.40	0.15±0.42	36	0.68
				2008	1.89±1.59	0.06±0.17	35	0.5
				2009	1.58±1.58	0.10±0.12	36	0.47
				2010	1.51±1.50	0.05±0.14	29	0.4
CA-SF1	54.485	-105.818	ENF	2003	6.32±1.85	-0.17±0.75	7	1.3
				2004	3.55±2.04	0.01±0.61	16	0.98
				2005	4.84±2.89	-0.27±0.76	16	1.18
				2006	6.03±2.53	-0.55±0.65	16	1.21
CA-SF2	54.2539	-105.878	ENF	2001	8.71±1.78	-1.02±0.35	8	1.8
				2002	5.05±5.04	-0.45±1.39	16	1.44

				2003	4.56±2.83	-0.06±0.49	11	1.15
				2004	3.30±2.61	-0.05±0.41	12	0.94
				2005	5.96±3.38	-0.73±0.99	15	1.45
CA-SF3	54.0916	-106.005	OSH	2002	2.33±0.33	0.25±0.25	3	0.77
				2003	2.29±1.12	0.05±0.19	16	0.67
				2004	2.04±1.06	0.06±0.25	16	0.6
				2005	2.68±1.16	-0.08±0.23	16	0.63
				2006	2.59±1.32	0.16±0.31	16	0.76
CA-TP1	42.6609	-80.5595	ENF	2003	0.95±0.92	1.75±1.68	14	1.06
				2004	0.84±0.83	1.83±1.71	15	0.98
				2005	1.39±1.07	1.63±1.38	10	1.12
				2006	2.15±1.80	0.89±0.38	6	1.19
				2007	0.72±0.73	0.49±0.68	11	0.54
				2008	1.75±1.42	1.24±1.26	10	1.08
				2009	2.28±1.61	0.75±0.60	28	1.11
				2010	2.29±1.98	0.17±0.40	29	0.89
				2011	2.73±2.14	0.06±0.21	33	0.74
				2012	2.91±2.05	-0.07±0.34	31	0.75
				2013	2.77±2.72	-0.28±0.47	25	0.63
				2014	2.30±2.71	-0.38±0.74	19	0.6
CA-TP2	42.7744	-80.4588	ENF	2003	3.19±2.80	-0.18±0.34	19	0.86
				2004	3.62±3.87	-0.37±0.74	16	0.93
				2005	3.03±2.65	-0.05±0.86	14	1
				2006	3.72±4.80	-0.47±1.20	15	1.02
				2007	5.67±2.33	-0.46±0.75	11	1.34
CA-TP3	42.7068	-80.3483	ENF	2003	1.99±1.88	0.96±0.73	18	1.02
				2004	2.09±2.26	0.87±0.64	16	1
				2005	1.32±2.05	1.11±0.41	13	0.84

				2006	3.53±2.10	1.63±1.05	7	1.4
				2007	0.62±1.20	1.43±0.71	9	0.77
				2008	3.26±2.88	-0.14±0.84	36	0.99
				2009	3.23±2.76	-0.05±0.85	36	0.98
				2010	3.46±2.83	-0.16±0.90	35	0.98
				2011	2.95±2.53	-0.03±0.78	32	0.86
				2012	3.46±2.30	-0.05±0.34	35	0.69
				2013	3.46±2.88	-0.14±0.45	35	0.69
				2014	2.94±2.40	0.07±0.41	33	0.7
CA-TP4	42.7102	-80.3574	ENF	2002	2.05±1.13	3.08±2.11	19	1.4
				2003	3.08±2.86	0.17±0.70	32	1.05
				2004	3.25±2.39	0.53±0.80	35	1.15
				2005	2.84±2.39	0.78±0.94	33	1.02
				2006	3.31±2.74	0.31±0.76	33	1.05
				2007	2.64±2.11	0.18±0.70	27	0.93
				2008	3.31±3.25	-0.24±1.14	29	0.97
				2009	3.06±3.44	-0.11±1.66	26	0.92
				2010	2.96±2.46	-0.27±0.67	24	0.89
				2011	3.41±2.91	0.13±0.46	32	0.85
				2012	3.32±2.49	0.15±0.46	31	0.76
				2013	3.43±2.97	-0.11±0.47	23	0.76
				2014	3.89±3.69	-0.36±0.72	29	0.87
CA-TPD	42.6353	-80.5577	DBF	2012	2.57±1.71	0.13±0.51	28	0.75
				2013	2.53±2.19	-0.13±0.28	23	0.6
				2014	2.82±2.68	-0.16±0.34	28	0.64
CG-Tch	-4.2892	11.6564	SAV	2006	1.78±1.46	0.19±0.34	16	0.48
				2007	2.55±1.61	-0.03±0.42	21	0.55
				2008	2.53±1.43	0.14±0.28	27	0.55

				2009	3.60±2.37	-0.18±0.33	28	0.6
CH-Cha	47.2102	8.4104	GRA	2005	4.20±3.14	-0.85±1.59	5	1.56
				2006	3.89±2.66	-0.50±0.94	12	1.58
				2007	6.55±5.22	-0.70±1.96	16	1.96
				2008	7.37±3.30	-1.20±0.83	10	1.85
				2009	8.20±5.75	-1.19±1.71	7	2.05
				2010	6.74±4.58	-0.81±1.74	12	1.93
				2011	5.34±2.98	-0.45±1.04	18	1.51
				2012	4.58±3.40	0.18±0.75	21	1.53
				2013	3.94±2.29	0.07±1.03	20	1.51
				2014	5.30±3.88	0.04±1.30	13	1.83
CH-Dav	46.8153	9.8559	ENF	1999	3.28±1.97	-1.50±1.11	28	0.89
				2000	2.71±1.66	-0.89±0.98	26	0.88
				2001	3.13±2.06	-0.91±1.03	22	1.01
				2002	2.67±1.50	-1.00±0.74	23	0.86
				2003	2.81±1.72	-1.48±0.96	25	0.69
				2004	3.12±1.80	-1.18±0.71	30	0.86
				2005	2.32±1.46	0.12±0.85	10	1.04
				2006	2.03±1.58	0.16±0.90	28	0.93
				2007	1.85±1.79	0.35±0.89	24	0.93
				2008	1.76±1.54	0.09±0.82	28	0.87
				2009	1.92±1.68	-0.02±0.97	28	0.83
				2010	2.02±2.00	-0.03±1.19	29	0.91
				2011	2.48±1.74	0.01±0.53	23	0.81
				2012	2.00±1.78	0.05±0.37	29	0.66
				2013	1.44±1.31	0.24±0.46	27	0.69
				2014	1.76±1.77	0.12±0.54	33	0.79
CH-Fru	47.1158	8.5378	GRA	2005	3.58±2.34	-0.38±0.62	12	1.16

				2006	3.97±3.22	-0.09±0.56	19	0.97
				2007	4.17±3.53	-0.05±0.41	19	0.9
				2008	4.52±3.27	-0.14±0.29	10	0.91
				2009	2.99±3.32	-0.13±0.56	10	0.76
				2010	3.81±2.58	-0.11±0.42	10	0.86
				2011	3.76±2.62	0.01±0.46	10	0.99
				2012	4.16±2.87	-0.23±0.37	11	0.91
				2013	3.79±3.63	-0.31±0.42	12	0.8
				2014	4.57±3.48	-0.32±0.82	11	1.12
CH-Lae	47.4781	8.365	MF	2004	2.02±0.86	0.65±0.97	2	1.39
				2005	3.30±1.57	0.47±1.09	7	1.57
				2006	2.81±2.10	-0.33±1.19	10	1.23
				2007	2.66±1.75	0.09±0.51	6	1.07
				2008	3.85±1.47	-0.33±0.56	10	1.08
				2009	2.29±1.65	0.17±0.49	18	0.92
				2010	3.29±1.88	-0.13±0.50	5	0.89
				2011	2.27±1.83	-0.10±0.73	10	0.96
				2012	1.98±1.44	0.18±0.91	9	1.06
				2013	1.59±1.34	0.55±0.70	19	1.09
				2014	1.94±1.40	0.62±0.49	20	1.14
CH-Oe1	47.2858	7.7319	GRA	2002	4.15±2.51	-0.72±0.73	22	1.21
				2003	3.03±1.91	-0.97±0.87	17	0.87
				2004	4.63±2.64	-0.62±0.61	21	1.02
				2005	3.55±2.74	-0.30±0.47	20	0.77
				2006	3.54±2.90	-0.22±0.63	25	0.85
				2007	3.14±2.01	-0.16±0.41	23	0.76
				2008	2.98±2.11	-0.15±0.29	21	0.69
CH-Oe2	47.2863	7.7343	CRO	2004	2.85±2.12	0.26±0.95	30	1.07

				2005	3.66±2.01	-0.61±0.88	33	1.12
				2006	3.72±2.84	-0.56±1.69	18	1.22
				2007	3.47±2.28	-0.39±1.35	25	1.03
				2008	3.62±2.07	-0.59±1.02	30	1.06
				2009	2.86±2.04	0.13±1.24	29	0.94
				2010	3.11±2.34	-0.19±1.31	30	1.06
				2011	2.88±2.03	-0.04±0.49	24	0.76
				2012	3.16±1.89	0.02±0.38	28	0.8
				2013	3.37±2.37	-0.15±0.57	30	0.81
				2014	3.52±2.05	-0.20±0.46	26	0.85
CN-Cha	42.4025	128.0958	MF	2003	3.42±3.61	-0.26±0.50	25	0.72
				2004	3.29±3.09	-0.24±0.43	27	0.79
				2005	2.54±2.13	-0.03±0.41	19	0.72
CN-Cng	44.5934	123.5092	GRA	2007	1.48±1.16	-0.03±0.36	21	0.56
				2008	1.14±1.28	0.06±0.19	36	0.39
				2009	1.02±1.08	0.03±0.32	32	0.35
				2010	1.18±1.34	0.04±0.17	26	0.36
CN-Dan	30.4978	91.0664	GRA	2004	0.62±0.49	-0.01±0.12	32	0.19
				2005	0.62±0.46	-0.01±0.08	34	0.19
CN-Din	23.1733	112.5361	EBF	2003	2.42±0.92	0.36±0.33	18	1.18
				2004	2.49±1.21	0.31±0.40	21	1.12
				2005	2.47±0.89	0.20±0.27	23	1.03
CN-Du2	42.0467	116.2836	GRA	2007	0.34±0.54	0.08±0.12	33	0.22
				2008	0.74±1.20	0.02±0.11	32	0.28
CN-Du3	42.0551	116.2809	GRA	2009	0.32±0.62	0.20±0.22	14	0.31
				2010	0.03±0.08	0.04±0.03	13	0.11
CN-Ha2	37.6086	101.3269	WET	2003	1.97±1.69	0.08±0.20	31	0.47
				2004	1.94±1.62	-0.00±0.17	32	0.42

				2005	2.28±1.94	-0.11±0.23	31	0.47
CN-HaM	37.37	101.18	GRA	2002	1.53±1.59	-0.24±0.31	33	0.47
				2003	1.32±1.54	-0.18±0.34	35	0.42
				2004	0.33±0.28	0.32±0.40	36	0.45
CN-Qia	26.7414	115.0581	ENF	2003	2.48±1.64	0.12±0.24	15	0.7
				2004	2.66±1.34	0.08±0.21	12	0.74
				2005	2.85±1.57	0.20±0.21	17	0.82
CN-Sw2	41.7902	111.8971	GRA	2010	0.00±0.00	0.01±0.00	1	0.03
				2011	0.42±0.70	0.02±0.18	31	0.26
				2012	0.46±0.65	-0.42±0.68	2	0.1
CZ-BK1	49.5021	18.5369	ENF	2004	1.84±1.65	0.78±0.89	19	1.02
				2005	3.13±1.27	0.38±0.58	14	1.39
				2006	2.65±0.76	0.85±0.69	12	1.51
				2007	3.82±1.03	-0.41±0.58	18	1.47
				2008	3.01±1.73	-0.21±0.99	21	1.13
				2009	2.66±1.75	-0.24±0.79	28	1.1
				2010	2.65±1.38	-0.02±0.81	25	1.16
				2011	2.32±1.42	0.03±0.36	30	0.78
				2012	1.98±1.26	0.26±0.29	28	0.77
				2013	2.47±1.24	0.02±0.33	29	0.8
				2014	2.50±1.56	0.15±0.42	31	0.88
CZ-BK2	49.4944	18.5429	GRA	2006	3.15±1.34	1.20±0.76	12	1.21
				2007	4.00±1.73	0.25±0.56	13	1.31
				2008	3.23±1.93	0.24±0.37	17	0.92
				2009	2.92±2.37	-0.00±0.32	28	0.63
				2010	3.05±2.16	0.05±0.25	27	0.67
				2011	2.84±2.36	-0.00±0.29	28	0.65
				2012	1.74±1.13	0.13±0.28	21	0.56

CZ-wet	49.0247	14.7704	WET	2006	4.13±2.28	-0.40±1.09	18	1.43
				2007	1.75±1.73	0.53±0.43	26	0.87
				2008	2.71±2.20	0.34±0.41	35	1.1
				2009	2.62±2.24	0.60±0.83	34	1.22
				2010	2.43±1.99	0.52±0.62	33	1.1
				2011	2.04±1.75	0.23±0.27	27	0.7
				2012	3.16±2.57	-0.25±0.40	27	0.77
				2013	2.12±2.29	0.01±0.56	24	0.64
				2014	2.67±2.18	0.08±0.35	22	0.76
DE-Akm	53.8662	13.6834	WET	2009	0.94±0.24	0.88±0.44	6	0.8
				2010	2.53±2.35	0.27±0.39	31	0.92
				2011	5.09±2.02	-0.75±0.55	13	1.18
				2012	5.12±2.58	-0.92±0.60	15	1.24
				2013	4.16±2.76	-0.70±0.71	29	1.04
				2014	3.14±2.23	0.25±0.43	22	1
DE-Geb	51.1001	10.9143	CRO	2001	2.85±1.98	-0.16±0.56	34	1.11
				2002	2.28±1.85	0.27±0.57	34	1.07
				2003	1.85±2.07	0.34±0.65	35	0.98
				2004	2.90±2.57	-0.48±1.13	36	1
				2005	3.22±2.47	-0.55±0.91	34	0.98
				2006	2.22±1.80	0.51±1.00	36	1.08
				2007	2.58±2.18	0.45±0.91	33	1.22
				2008	2.55±2.55	0.03±1.45	35	1.07
				2009	1.72±1.62	0.87±0.45	33	0.98
				2010	2.33±2.25	0.16±0.84	36	1.04
				2011	1.80±1.29	0.84±0.81	35	1.07
				2012	2.14±1.54	0.12±0.34	36	0.76
				2013	2.27±2.11	0.16±0.37	36	0.72

				2014	2.97±2.03	-0.02±0.45	35	0.88
DE-Gri	50.95	13.5126	GRA	2004	2.71±2.22	1.03±1.53	34	1.21
				2005	3.89±3.11	-0.28±0.91	30	1.04
				2006	2.63±2.09	1.12±1.09	31	1.13
				2007	3.58±2.31	0.59±1.10	34	1.11
				2008	3.99±2.70	-0.07±0.85	34	1.06
				2009	4.63±3.26	-0.32±0.87	35	1.16
				2010	4.13±3.01	-0.24±0.69	35	1.09
				2011	4.64±3.08	-0.28±1.08	33	1.11
				2012	4.34±2.95	-0.03±0.41	36	0.82
				2013	3.69±2.65	0.13±0.32	35	0.74
				2014	5.31±3.30	-0.28±0.57	36	0.94
DE-Hai	51.0792	10.453	DBF	2000	3.19±1.68	-0.35±0.58	33	1.02
				2001	2.75±1.56	0.23±0.69	35	1.12
				2002	3.14±1.74	-0.05±0.81	35	1.15
				2003	2.77±1.50	0.08±0.71	34	1.04
				2004	3.06±1.49	-0.22±0.74	35	1.03
				2005	2.83±1.41	0.07±0.57	35	1.14
				2006	2.86±1.53	0.04±0.57	34	1.06
				2007	2.67±1.19	0.27±0.47	34	0.96
				2008	3.01±1.64	-0.09±0.87	36	1.05
				2009	2.97±1.53	-0.10±0.58	35	0.91
				2010	2.70±1.39	0.14±0.28	27	0.8
				2011	2.77±1.21	0.12±0.23	34	0.74
				2012	2.79±1.15	0.03±0.36	35	0.73
DE-Kli	50.8931	13.5224	CRO	2004	3.69±1.62	-0.15±0.46	17	1.28
				2005	3.63±2.96	-1.11±1.37	32	1.04
				2006	3.33±2.65	-0.80±1.37	33	1.07

				2007	2.93±2.26	-0.35±1.01	35	1.09
				2008	2.18±1.51	0.66±0.68	24	1.19
				2009	3.97±2.19	-0.53±0.90	23	1.34
				2010	3.26±2.29	-0.48±0.82	33	1.16
				2011	3.16±2.42	-0.45±0.99	35	1.01
				2012	1.95±1.57	0.28±0.37	34	0.78
				2013	1.52±1.67	0.08±0.25	25	0.58
				2014	3.59±2.35	-0.30±0.50	36	0.89
DE-Lkb	49.0996	13.3047	ENF	2009	2.46±1.09	0.29±0.18	21	0.8
				2010	1.62±0.84	0.21±0.38	14	0.64
				2011	2.16±1.28	0.05±0.31	27	0.66
				2012	2.22±1.15	0.10±0.22	19	0.7
				2013	2.08±1.13	0.28±0.41	18	0.83
DE-Lnf	51.3282	10.3678	DBF	2002	3.89±1.72	-0.53±0.73	25	1.08
				2003	2.87±1.79	-0.26±0.68	33	1
				2004	2.91±1.60	-0.26±0.71	33	0.98
				2005	3.01±1.75	-0.18±0.77	35	0.95
				2006	2.72±1.62	0.04±0.44	34	0.88
				2010	2.52±1.55	0.08±0.20	32	0.66
				2011	2.71±1.70	-0.02±0.35	28	0.64
				2012	2.68±1.64	-0.01±0.29	33	0.62
DE-Obe	50.7867	13.7213	ENF	2008	3.89±2.34	-0.01±1.10	25	1.37
				2009	3.04±1.82	0.13±1.03	25	1.24
				2010	3.28±2.45	-0.13±0.79	28	1.18
				2011	3.07±1.84	0.15±0.45	26	0.93
				2012	3.33±2.37	-0.08±0.49	24	0.86
				2013	3.20±2.37	0.08±0.61	31	0.94
				2014	3.61±2.41	-0.02±0.56	25	1.01

DE-RuR	50.6219	6.3041	GRA	2011	5.82±2.46	-0.17±0.48	15	1.07
				2012	4.28±2.76	0.04±0.23	27	0.84
				2013	4.07±2.69	-0.00±0.33	34	0.85
				2014	4.44±2.67	-0.13±0.34	31	0.81
DE-RuS	50.8659	6.4472	CRO	2011	4.10±1.25	-0.08±0.40	17	0.96
				2012	3.01±1.84	0.23±0.28	19	0.95
				2013	3.20±2.39	-0.20±0.38	33	0.77
				2014	3.13±1.84	-0.02±0.44	30	0.8
DE-Seh	50.8706	6.4497	CRO	2007	3.39±1.20	-0.20±0.55	15	0.83
				2008	3.02±2.52	-0.07±0.48	33	0.82
				2009	2.33±2.01	0.17±0.27	35	0.81
				2010	1.91±1.40	0.39±0.55	28	0.87
DE-SfN	47.8064	11.3275	WET	2012	2.21±1.68	0.27±0.46	13	0.88
				2013	1.02±0.94	0.34±0.35	23	0.73
				2014	1.20±0.71	0.50±0.40	19	0.86
DE-Spw	51.8923	14.0337	WET	2010	4.75±2.69	-0.63±1.20	6	1.21
				2011	2.73±1.65	-0.07±0.37	28	0.64
				2012	3.04±1.96	-0.19±0.29	29	0.64
				2013	2.56±2.06	-0.11±0.31	27	0.55
				2014	3.26±2.28	-0.16±0.46	30	0.71
DE-Tha	50.9624	13.5652	ENF	1999	2.60±2.02	-0.05±0.53	24	0.98
				2000	3.24±2.18	-0.14±0.59	26	1.02
				2001	2.56±1.95	0.37±0.46	29	0.98
				2002	2.90±1.93	0.23±0.64	31	1.03
				2003	2.33±1.85	0.12±0.39	24	0.95
				2004	3.45±2.50	-0.53±0.91	30	1.07
				2005	3.02±2.60	-0.33±1.02	24	0.94
				2006	2.77±2.18	-0.07±0.75	27	0.95

				2007	3.21±2.19	-0.25±0.55	25	0.97
				2008	2.26±1.35	0.05±0.52	19	0.87
				2009	3.17±2.35	-0.29±0.40	23	0.95
				2010	2.83±2.15	-0.18±0.61	22	0.97
				2011	3.81±2.27	-0.19±0.26	30	0.76
				2012	3.48±2.19	-0.06±0.42	29	0.76
				2013	2.66±2.13	-0.04±0.37	26	0.62
				2014	3.64±2.18	-0.14±0.35	29	0.79
DE-Zrk	53.8759	12.889	WET	2013	2.75±2.00	0.27±0.41	17	1
				2014	1.45±1.17	0.32±0.27	26	0.74
DK-Eng	55.6905	12.1918	GRA	2005	2.53±1.35	0.12±0.37	23	0.75
				2006	1.75±1.30	0.10±0.24	30	0.62
				2007	1.95±1.27	0.25±0.25	31	0.77
				2008	1.89±1.28	0.43±0.59	16	1.08
DK-Fou	56.4842	9.5872	CRO	2005	2.77±2.19	0.40±0.90	24	1.26
DK-NuF	64.1308	-51.3861	WET	2008	0.94±0.48	0.21±0.11	13	0.37
				2009	0.78±0.55	0.16±0.14	15	0.36
				2010	1.73±0.78	-0.15±0.22	15	0.41
				2011	1.12±0.60	-0.04±0.18	15	0.34
				2012	1.44±0.62	0.00±0.12	12	0.36
				2013	1.58±0.86	-0.10±0.12	16	0.41
				2014	1.86±0.85	0.02±0.24	6	0.53
DK-Sor	55.4859	11.6446	DBF	1999	5.21±3.08	-1.24±0.83	35	1.21
				2000	4.94±2.63	-0.93±0.64	34	1.3
				2001	4.86±3.03	-0.84±0.62	36	1.17
				2002	4.59±2.81	-0.51±0.67	36	1.22
				2003	5.36±3.09	-1.14±0.88	33	1.25
				2004	5.04±3.14	-1.12±1.01	31	1.16

				2005	4.82±2.89	-0.83±0.76	34	1.17
				2006	4.94±3.22	-0.82±0.83	35	1.23
				2007	4.72±3.00	-0.64±0.90	33	1.21
				2008	4.74±3.04	-0.63±0.68	33	1.23
				2009	4.79±3.23	-0.55±1.00	36	1.24
				2010	4.17±3.12	-0.56±0.82	34	1.14
				2011	4.92±2.92	-0.52±0.64	35	1.15
				2012	4.61±3.09	-0.29±0.42	35	0.91
				2013	4.37±2.91	-0.09±0.39	33	0.87
				2014	6.60±2.90	-0.47±0.43	27	1.27
DK-ZaF	74.4814	-20.5545	WET	2008	1.59±0.00	-1.02±0.00	1	0.3
				2009	0.42±0.16	-0.23±0.07	3	0.14
				2010	0.42±0.26	-0.26±0.13	5	0.13
				2011	0.58±0.30	-0.33±0.18	4	0.17
DK-ZaH	74.4733	-20.5503	GRA	2000	0.85±0.11	-0.13±0.10	9	0.15
				2001	0.79±0.26	-0.08±0.15	9	0.18
				2002	1.06±0.08	-0.27±0.05	8	0.18
				2003	0.95±0.28	-0.16±0.17	9	0.16
				2004	0.50±0.26	0.19±0.23	9	0.17
				2005	0.85±0.20	-0.09±0.09	10	0.17
				2006	0.60±0.15	0.09±0.05	9	0.15
				2007	0.20±0.20	0.27±0.22	15	0.17
				2008	0.43±0.36	0.05±0.11	17	0.14
				2009	0.54±0.32	-0.02±0.09	16	0.14
				2010	0.60±0.33	-0.06±0.06	19	0.13
				2011	0.11±0.00	0.35±0.23	2	0.25
				2012	0.67±0.49	-0.12±0.19	14	0.17
				2013	0.51±0.25	0.04±0.06	18	0.14

				2014	0.47±0.33	0.05±0.07	15	0.25
ES-Amo	36.8336	-2.2523	OSH	2007	0.40±0.54	0.06±0.47	14	0.35
				2008	0.96±0.68	-0.40±0.70	9	0.39
				2009	0.34±0.21	0.12±0.22	35	0.33
				2010	0.40±0.24	0.12±0.14	36	0.34
				2011	0.48±0.23	0.02±0.11	30	0.29
				2012	0.32±0.19	0.08±0.10	35	0.25
ES-LgS	37.0979	-2.9658	OSH	2007	0.89±0.52	0.13±0.17	36	0.51
				2008	0.92±0.59	0.16±0.20	32	0.53
				2009	1.10±0.62	0.03±0.23	16	0.51
ES-LJu	36.9266	-2.7521	OSH	2004	0.10±0.18	0.28±0.18	16	0.35
				2005	0.06±0.07	0.28±0.10	19	0.34
				2006	0.26±0.31	0.16±0.27	28	0.37
				2007	0.34±0.28	0.08±0.18	26	0.35
				2008	0.41±0.33	0.20±0.40	29	0.5
				2009	0.38±0.36	0.06±0.28	36	0.36
				2010	0.41±0.26	0.03±0.14	30	0.35
				2011	0.32±0.21	0.06±0.08	36	0.26
				2012	0.32±0.27	0.02±0.12	28	0.23
				2013	0.35±0.23	0.02±0.12	26	0.23
ES-Ln2	36.9695	-3.4758	OSH	2009	0.33±0.20	0.14±0.13	19	0.44
FI-Hyy	61.8474	24.2948	ENF	1999	2.27±2.01	-0.08±0.44	34	0.76
				2000	2.82±1.87	-0.05±0.35	31	0.98
				2001	2.38±1.89	0.02±0.31	35	0.86
				2002	2.46±2.03	0.01±0.40	34	0.82
				2003	2.34±1.98	0.04±0.45	35	0.75
				2004	2.34±1.90	0.24±0.34	35	0.83
				2005	2.70±2.01	0.22±0.52	28	0.92

				2006	2.45±1.88	0.15±0.53	33	0.91
				2007	2.55±2.03	0.06±0.37	36	0.85
				2008	2.31±1.79	0.17±0.49	35	0.83
				2009	2.61±2.28	-0.12±0.51	36	0.81
				2010	2.41±2.17	-0.05±0.43	31	0.88
				2011	2.65±2.08	0.01±0.31	35	0.84
				2012	2.33±2.12	-0.00±0.27	34	0.57
				2013	2.46±2.08	-0.04±0.30	34	0.58
				2014	2.55±1.91	0.07±0.33	34	0.66
FI-Jok	60.8986	23.5135	CRO	2000	0.84±0.69	0.17±0.29	24	0.45
				2001	1.49±1.70	0.09±0.26	33	0.5
				2002	1.40±1.66	0.12±0.25	32	0.56
				2003	1.02±1.29	0.03±0.09	11	0.4
FI-Let	60.6418	23.9595	ENF	2009	2.10±2.20	0.02±0.17	8	0.56
				2010	3.08±2.68	-0.27±0.47	26	0.84
				2011	3.07±2.79	-0.22±0.69	21	0.8
				2012	3.40±3.19	-0.35±0.68	29	0.84
FI-Lom	67.9972	24.2092	WET	2007	1.09±1.07	0.12±0.16	30	0.45
				2008	0.83±0.94	0.15±0.17	33	0.37
				2009	1.18±1.45	-0.02±0.18	32	0.39
FI-Sod	67.3624	26.6386	ENF	2001	2.36±2.57	-0.61±1.19	32	0.74
				2002	3.11±2.72	-1.01±1.34	22	0.86
				2003	1.72±1.09	-0.23±0.48	32	0.65
				2004	1.95±1.62	-0.30±0.35	35	0.67
				2005	1.86±1.61	-0.26±0.49	34	0.65
				2006	1.80±1.79	-0.19±0.53	36	0.68
				2007	1.53±1.58	0.07±0.56	32	0.64
				2008	1.50±1.36	0.02±0.42	36	0.62

				2009	1.61±1.59	-0.04±0.63	33	0.66
				2010	1.45±1.26	-0.23±0.38	28	0.54
				2011	1.90±1.66	-0.27±0.46	34	0.59
				2012	1.39±1.49	-0.05±0.22	31	0.4
				2013	1.92±1.98	-0.12±0.28	32	0.51
				2014	1.90±1.95	-0.14±0.38	34	0.5
FR-Fon	48.4764	2.7801	DBF	2005	3.72±1.74	0.27±0.78	27	1.29
				2006	2.97±1.63	0.49±0.70	33	1.15
				2007	3.19±1.61	0.32±0.85	36	1.24
				2008	3.03±1.98	0.28±0.76	34	1.08
				2009	3.39±2.50	0.10±1.21	35	1.14
				2010	2.94±1.96	0.26±1.07	33	1.12
				2011	3.41±1.89	0.08±0.69	34	0.97
				2012	3.29±2.22	0.16±0.57	36	0.93
				2013	2.90±1.99	0.11±0.40	31	0.8
				2014	3.20±2.21	-0.04±0.46	19	0.8
FR-Gri	48.8442	1.9519	CRO	2004	2.86±1.47	0.57±1.11	20	1.28
				2005	2.78±2.00	0.03±1.57	31	1.15
				2006	2.59±1.65	0.19±0.71	32	1.22
				2007	2.69±1.47	0.71±0.63	32	1.37
				2008	3.01±2.95	-0.13±1.98	21	1.17
				2009	2.33±1.83	0.32±0.39	14	0.84
				2010	2.49±1.80	-0.02±0.39	18	0.73
				2011	3.24±2.76	-0.09±0.83	18	1.09
				2012	3.18±1.86	0.34±0.33	12	1
				2013	3.07±2.18	0.07±0.32	26	0.9
				2014	3.19±1.82	0.23±0.28	20	1.02
FR-LBr	44.7171	-0.7693	ENF	1999	3.27±0.99	0.18±0.65	15	1.25

				2000	5.78±1.68	-0.88±0.74	12	1.73
				2001	3.70±1.48	-0.03±1.03	23	1.41
				2002	3.50±1.21	0.67±1.10	24	1.45
				2003	3.98±1.22	-0.09±0.96	10	1.48
				2004	4.46±2.14	0.14±0.43	21	1.08
				2005	3.75±1.68	-0.14±0.47	29	0.89
				2006	3.71±1.44	-0.08±0.38	29	0.87
				2007	3.07±2.13	0.39±0.51	19	1.05
				2008	4.82±2.57	-0.46±0.71	15	1.1
FR-Pue	43.7413	3.5957	EBF	2000	2.37±0.70	-0.10±1.04	14	1.24
				2001	3.18±1.47	-0.85±0.86	34	1.11
				2002	3.18±1.27	-0.67±0.71	35	1.27
				2003	3.04±1.33	-0.73±0.89	34	1.15
				2004	3.08±1.43	-0.76±0.77	35	1.1
				2005	2.78±1.36	-0.56±0.77	35	1.03
				2006	2.30±1.21	0.03±1.06	33	1.1
				2007	2.97±1.30	-0.20±0.58	36	1.35
				2008	2.58±1.33	-0.11±0.56	36	1.26
				2009	2.66±1.13	-0.11±0.54	34	1.28
				2010	2.65±1.13	-0.39±0.57	33	1.17
				2011	2.75±1.15	-0.33±0.36	29	1.06
				2012	3.27±1.28	-0.28±0.49	30	1
				2013	1.73±0.97	0.30±0.45	35	0.87
				2014	1.31±0.64	0.49±0.25	35	0.95
GF-Guy	5.2788	-52.9249	EBF	2004	10.15±1.28	-0.56±1.31	23	1.37
				2005	9.39±1.36	0.34±1.35	22	1.41
				2006	9.62±1.25	-0.28±1.38	35	1.28
				2007	9.56±1.50	0.03±1.55	34	1.29

				2008	8.65±1.28	0.46±1.03	21	1.35
				2009	8.87±1.33	0.61±1.27	20	1.18
				2010	10.63±1.89	-1.16±2.01	36	1.57
				2011	9.80±1.30	-0.27±1.14	13	1.42
				2012	10.17±1.68	-0.44±1.02	35	1.04
				2013	9.30±1.33	0.12±0.84	35	1.08
				2014	8.66±1.97	0.42±0.96	36	1.28
GH-Ank	5.2685	-2.6942	EBF	2011	6.83±1.56	-0.95±0.75	8	1.56
				2012	3.69±0.60	0.74±0.46	9	1.32
				2014	4.11±1.51	0.63±0.79	2	1.36
IT-BCi	40.5238	14.9574	CRO	2004	4.79±3.30	0.45±2.36	21	1.9
				2005	3.88±2.83	0.55±1.59	35	1.64
				2006	3.94±2.80	0.78±1.80	33	1.63
				2007	3.79±2.18	0.63±2.00	34	1.64
				2008	3.62±2.15	0.63±1.36	30	1.67
				2009	3.28±2.29	1.22±1.83	34	1.61
				2010	4.52±2.30	0.09±1.40	33	1.65
				2011	4.96±2.65	-0.11±0.87	21	1.27
				2012	4.84±1.80	0.13±0.60	28	1.27
				2013	5.04±2.66	-0.28±0.96	32	1.25
				2014	5.56±2.83	-0.31±1.24	31	1.34
IT-CA1	42.3804	12.0266	DBF	2011	3.01±1.48	-0.04±0.29	15	0.66
				2012	2.59±1.35	-0.07±0.32	26	0.66
				2013	1.77±1.09	0.11±0.29	17	0.56
				2014	2.96±1.38	0.04±0.26	28	0.7
IT-CA2	42.3772	12.026	CRO	2011	2.07±0.74	0.21±0.38	10	0.8
				2012	1.92±1.18	0.22±0.35	24	0.82
				2013	2.07±1.16	0.10±0.27	24	0.72

				2014	2.49±1.00	0.28±0.27	7	0.93
IT-CA3	42.38	12.0222	DBF	2011	1.35±0.42	0.12±0.07	4	0.4
				2012	1.67±0.77	0.32±0.31	16	0.76
				2013	1.77±1.08	0.21±0.26	18	0.65
				2014	2.43±0.69	0.28±0.24	5	0.9
IT-Col	41.8494	13.5881	DBF	1999	2.29±0.90	1.22±0.58	14	1.24
				2000	1.59±1.34	0.83±0.65	9	0.95
				2001	1.50±1.00	0.70±0.96	26	0.89
				2002	1.72±0.85	0.61±0.58	8	0.96
				2003	1.48±0.00	2.56±0.00	1	1.36
				2004	1.78±0.61	0.91±1.07	14	1.11
				2005	1.93±1.20	0.52±0.74	20	1.02
				2006	1.89±0.62	0.42±0.79	10	0.91
				2007	2.20±1.03	0.35±0.76	27	1.06
				2008	1.96±1.10	0.55±0.72	26	0.94
				2009	2.14±1.30	0.10±0.43	19	0.71
				2010	2.44±1.26	0.31±0.48	9	0.85
				2011	2.18±1.24	0.18±0.34	31	0.73
				2012	2.50±1.05	0.14±0.22	13	0.8
				2013	2.21±1.37	0.13±0.31	19	0.7
				2014	1.84±0.89	0.11±0.18	22	0.6
IT-Cp2	41.7043	12.3573	EBF	2012	2.67±1.45	0.48±0.57	18	1.11
				2013	3.89±1.39	-0.05±0.52	33	1.08
				2014	4.47±1.34	0.04±0.48	28	1.18
IT-Cpz	41.7053	12.3761	EBF	2000	4.21±0.79	0.08±0.65	27	1.36
				2001	3.98±1.18	-0.20±0.79	24	1.39
				2002	5.00±1.79	-0.88±1.14	21	1.36
				2003	3.90±1.73	-0.53±1.22	30	1.3

				2004	3.92±1.78	-0.42±0.80	25	1.25
				2005	2.51±1.19	0.37±0.45	26	0.84
				2006	3.46±1.66	0.02±0.54	31	0.93
				2007	3.66±1.57	-0.14±0.59	23	0.98
				2008	3.99±1.42	-0.03±0.44	21	0.96
IT-Isp	45.8126	8.6336	DBF	2013	4.30±2.60	-0.62±0.22	4	1.04
				2014	3.41±0.96	-0.43±0.19	6	0.87
IT-La2	45.9542	11.2853	ENF	2000	2.71±2.59	-0.11±0.65	10	1.02
				2001	2.15±1.95	0.03±0.31	17	0.77
				2002	1.29±0.57	-0.07±0.26	9	0.54
IT-Lav	45.9562	11.2813	ENF	2003	1.08±0.97	0.47±0.41	31	1.21
				2004	1.08±1.04	0.27±0.51	30	1.11
				2005	1.07±1.09	0.49±0.62	24	1.24
				2006	1.23±1.11	0.40±0.34	34	1.28
				2007	0.93±0.93	0.65±0.44	32	1.25
				2008	1.15±1.12	0.37±0.59	29	1.17
				2009	1.66±1.07	0.09±0.48	32	1.28
				2010	1.24±1.05	0.33±0.74	32	1.18
				2011	0.82±0.77	0.44±0.29	28	0.93
				2012	0.70±0.90	0.40±0.25	28	0.87
				2013	1.13±1.31	0.20±0.45	34	0.91
				2014	0.95±0.68	0.47±0.32	27	1.05
IT-MBo	46.0147	11.0458	GRA	2003	3.35±3.14	-0.29±0.83	33	1.14
				2004	3.46±2.97	-0.31±0.62	36	1.07
				2005	3.81±2.76	-0.49±0.80	36	1.16
				2006	3.55±2.65	-0.45±0.72	35	1.19
				2007	3.55±2.86	-0.25±0.95	36	1.22
				2008	3.23±2.85	-0.35±0.89	33	1.09

				2009	4.05±3.45	-0.46±1.02	35	1.21
				2010	3.82±3.00	-0.37±0.73	32	1.26
				2011	4.10±3.32	-0.21±0.58	35	0.99
				2012	4.33±3.32	-0.38±0.46	36	0.94
				2013	3.94±3.06	-0.39±0.49	33	0.9
IT-PT1	45.2009	9.061	DBF	2002	4.02±1.92	0.00±0.28	27	0.88
				2003	2.65±1.75	0.22±0.59	35	0.81
				2004	3.50±2.06	-0.03±0.40	29	0.86
IT-Ren	46.5869	11.4337	ENF	1999	1.92±1.55	0.28±0.43	20	0.85
				2001	0.34±0.00	0.37±0.00	1	0.37
				2002	1.95±1.48	0.22±0.61	22	0.84
				2003	0.84±0.93	0.75±0.69	19	0.72
				2005	1.33±1.23	0.27±0.39	15	0.67
				2006	1.95±0.97	0.08±0.75	10	0.82
				2007	1.44±1.18	0.25±0.26	16	0.56
				2008	1.58±1.28	-0.13±0.30	11	0.41
				2009	1.81±1.35	0.01±0.26	20	0.55
				2010	2.11±1.39	0.00±0.49	16	0.74
				2011	1.27±0.74	0.18±0.36	12	0.48
				2012	1.45±0.77	-0.03±0.20	17	0.43
				2013	2.25±1.42	-0.00±0.33	17	0.69
IT-Ro1	42.4081	11.93	DBF	2000	3.70±0.98	-0.76±0.82	21	1.11
				2001	3.94±1.96	-0.96±1.05	25	1.16
				2002	4.06±1.73	-1.07±0.94	31	1.15
				2003	3.53±1.49	-1.18±0.92	25	0.97
				2004	3.89±2.12	-0.79±1.09	25	0.98
				2005	2.62±1.22	-0.01±0.22	28	0.73
				2006	3.13±1.37	-0.12±0.27	31	0.72

				2007	2.18±0.87	0.34±0.42	11	0.79
				2008	3.27±1.49	-0.09±0.46	23	0.84
IT-Ro2	42.3903	11.9209	DBF	2002	2.52±1.02	-0.02±0.62	29	1.19
				2003	2.27±0.87	-0.20±0.63	28	1
				2004	2.07±1.40	0.19±1.02	29	1.14
				2005	1.57±1.17	0.87±0.86	32	1.2
				2006	1.55±1.03	0.55±0.89	27	1.08
				2007	0.87±0.83	1.05±1.00	24	0.91
				2008	1.93±1.32	0.37±0.68	20	0.97
				2010	1.26±0.67	0.62±0.53	34	1.06
				2011	3.08±1.12	-0.27±0.42	16	1.07
				2012	2.44±1.30	-0.16±0.38	23	0.76
IT-SR2	43.732	10.291	ENF	2013	4.65±2.46	-0.04±0.64	31	1.14
				2014	5.96±2.78	-0.05±0.59	33	1.28
IT-SRo	43.7279	10.2844	ENF	1999	3.97±0.71	1.50±1.94	36	1.81
				2000	3.92±1.02	1.28±1.29	32	1.72
				2001	4.30±1.21	0.84±0.84	36	1.71
				2002	4.34±1.56	1.80±1.21	27	1.86
				2003	3.14±1.12	1.74±1.08	35	1.74
				2004	3.08±1.20	1.87±1.14	33	1.64
				2005	2.56±1.10	1.43±0.65	20	1.6
				2006	4.26±1.80	1.19±1.22	35	1.82
				2007	5.03±1.60	0.80±1.13	34	1.89
				2008	8.38±3.93	-2.52±2.19	21	1.84
				2009	6.08±3.14	-0.59±0.94	28	1.51
				2010	6.64±2.86	-0.34±0.84	23	1.44
				2011	4.74±1.75	0.46±0.56	32	1.32
				2012	5.86±2.33	-0.56±0.82	35	1.33

IT-Tor	45.8444	7.5781	GRA	2008	3.44±1.64	-0.55±0.99	10	1.11
				2009	2.45±1.13	-0.21±0.73	25	0.8
				2010	2.30±1.06	0.24±0.63	26	0.87
				2011	2.57±1.41	-0.09±0.28	26	0.6
				2012	2.24±1.36	-0.04±0.30	29	0.5
				2013	2.02±0.98	0.05±0.23	21	0.58
				2014	2.32±1.49	-0.08±0.26	28	0.57
JP-MBF	44.3869	142.3186	DBF	2004	2.33±1.46	0.02±0.40	20	0.9
				2005	1.47±1.27	0.20±0.47	30	0.81
JP-SMF	35.2617	137.0788	MF	2002	3.01±0.97	-0.38±0.37	2	0.73
				2003	3.09±1.28	0.09±0.38	8	0.86
				2004	2.43±1.29	-0.01±0.51	5	0.64
				2005	3.67±1.02	-0.24±0.42	4	0.9
				2006	3.54±0.82	-0.08±0.48	12	0.93
MY-PSO	2.973	102.3062	EBF	2003	3.74±1.04	0.28±0.92	19	0.65
				2004	3.75±0.94	0.15±0.48	14	0.68
				2005	3.87±0.83	0.06±0.46	17	0.64
				2006	4.30±0.66	-0.06±0.49	16	0.55
				2007	4.24±0.74	0.06±0.54	19	0.58
				2008	4.50±0.77	-0.14±0.56	15	0.58
				2009	3.77±0.74	0.16±0.43	15	0.66
NL-Hor	52.2404	5.0713	GRA	2004	3.07±1.85	0.14±0.62	14	1.01
				2005	2.88±1.98	0.52±0.71	27	1.06
				2006	2.21±1.64	0.17±0.54	9	0.89
				2007	2.92±2.37	0.28±0.95	30	1.03
				2008	2.92±2.47	0.07±0.32	30	0.71
				2009	4.85±2.81	-0.49±0.61	23	1
				2010	2.84±2.18	0.14±0.20	34	0.66

				2011	3.11±1.85	0.41±0.43	21	0.85
NL-Loo	52.1666	5.7436	ENF	1999	3.86±1.84	-0.76±0.72	25	1.08
				2000	4.10±2.05	-1.08±0.60	34	0.99
				2001	3.63±1.94	-1.04±0.76	31	0.98
				2002	2.97±1.88	-0.13±0.58	27	0.99
				2003	2.89±1.71	-0.19±0.65	31	1.01
				2004	3.28±1.96	-0.34±0.62	29	1.04
				2005	3.28±1.75	-0.38±0.47	31	0.95
				2006	3.03±1.83	-0.23±0.74	33	0.98
				2007	2.99±1.48	-0.31±0.65	25	0.89
				2008	3.00±1.74	-0.08±0.47	28	0.93
				2009	2.94±2.04	0.04±0.59	8	0.98
				2010	2.48±1.83	-0.03±0.50	29	0.84
				2011	2.78±1.65	0.01±0.55	31	0.82
				2012	2.87±1.77	0.07±0.17	36	0.66
				2013	2.61±1.57	0.10±0.19	29	0.67
				2014	3.25±1.45	0.18±0.23	28	0.8
NO-Adv	78.186	15.923	WET	2012	0.20±0.00	-0.08±0.00	1	0.12
				2013	1.26±0.80	-0.76±0.57	22	0.33
NO-Blv	78.9216	11.8311	SNO	2008	0.12±0.09	0.24±0.16	30	0.26
				2009	0.04±0.06	0.08±0.06	7	0.12
PA-SPn	9.3181	-79.6346	DBF	2007	4.72±2.09	0.21±0.91	6	1.18
				2008	4.09±1.30	0.33±0.47	15	1.1
				2009	3.54±1.26	0.15±0.60	12	1.13
PA-SPs	9.3138	-79.6314	GRA	2007	7.93±1.66	-0.24±0.82	18	1.21
				2008	7.31±2.28	-0.31±0.69	24	1.21
				2009	7.14±2.14	-0.06±0.94	17	1.29
RU-Che	68.613	161.3414	WET	2002	1.48±0.91	-0.03±0.12	12	0.39

				2003	1.13±0.81	0.02±0.20	19	0.4
				2004	1.05±0.82	0.10±0.17	18	0.4
				2005	0.66±0.64	0.19±0.10	6	0.32
RU-Cok	70.8291	147.4943	OSH	2003	1.22±0.87	0.04±0.18	14	0.44
				2004	1.53±0.35	0.11±0.15	7	0.46
				2005	2.72±0.66	-0.27±0.34	6	0.66
				2006	1.18±0.82	0.15±0.10	4	0.36
				2007	2.02±0.58	0.11±0.06	7	0.58
				2008	1.22±0.94	0.10±0.18	13	0.5
				2009	1.23±1.04	-0.10±0.19	5	0.35
				2010	0.12±0.34	0.63±0.46	8	0.73
				2011	2.44±0.00	-0.17±0.10	2	0.58
				2012	1.67±0.26	-0.02±0.10	7	0.43
				2013	1.07±0.90	0.11±0.21	9	0.45
RU-Fyo	56.4615	32.9221	ENF	1999	4.67±4.32	-1.89±2.19	34	1.03
				2000	4.92±3.96	-2.28±2.20	35	1.06
				2001	5.72±4.94	-2.61±2.63	32	1.07
				2002	2.55±2.69	-0.31±1.69	26	0.81
				2003	3.43±3.11	-0.98±1.94	33	0.92
				2004	4.06±3.11	-1.09±0.96	33	1.03
				2005	4.43±3.96	-1.14±1.42	35	1.07
				2006	4.04±3.50	-1.07±1.54	34	1.08
				2007	3.48±3.11	-0.62±1.34	33	1.02
				2008	4.41±3.79	-1.32±1.71	32	1.14
				2009	4.83±3.67	-1.62±1.36	35	1.07
				2010	4.09±2.99	-0.86±1.10	24	1.2
				2011	4.91±3.82	-1.16±1.38	26	1.26
				2012	3.89±3.35	-0.32±0.71	35	0.91

				2013	4.04±3.41	-0.31±0.43	35	0.89
				2014	3.75±3.41	-0.28±0.49	35	0.86
RU-Ha1	54.7252	90.0022	GRA	2002	1.21±1.07	0.06±0.19	18	0.39
				2003	2.11±1.28	0.01±0.15	19	0.52
				2004	1.86±1.51	0.02±0.13	23	0.47
SD-Dem	13.2829	30.4783	SAV	2005	1.96±0.55	-0.32±0.31	4	0.49
				2007	2.30±1.00	-0.04±0.19	14	0.48
				2008	1.36±1.20	-0.02±0.14	29	0.29
				2009	1.12±1.15	-0.01±0.17	30	0.29
SN-Dhr	15.4028	-15.4322	SAV	2010	8.37±1.28	-0.95±0.17	6	1.46
				2011	4.16±2.19	0.09±0.38	3	0.8
				2012	2.12±2.16	-0.15±0.33	26	0.58
				2013	2.18±2.10	0.27±0.29	6	0.7
US-AR1	36.4267	-99.42	GRA	2009	2.44±1.81	-0.38±1.30	24	0.73
				2010	1.74±1.11	0.04±0.24	36	0.52
				2011	1.36±1.12	0.00±0.29	36	0.45
				2012	1.28±0.96	-0.02±0.21	24	0.4
US-AR2	36.6358	-99.5975	GRA	2009	1.46±0.87	-0.03±0.29	22	0.47
				2010	1.15±1.06	-0.04±0.25	34	0.39
				2011	0.42±0.36	0.11±0.17	35	0.3
				2012	0.95±0.67	0.06±0.13	17	0.35
US-ARb	35.5497	-98.0402	GRA	2005	3.27±2.38	-0.07±0.42	21	0.81
				2006	2.38±1.61	0.01±0.32	28	0.71
US-ARc	35.5465	-98.04	GRA	2005	3.91±2.27	0.00±0.44	29	0.82
				2006	2.73±1.63	0.01±0.31	29	0.72
US-ARM	36.6058	-97.4888	CRO	2003	1.28±0.83	0.70±0.74	34	0.87
				2004	1.85±1.58	0.41±0.61	30	0.93
				2005	1.13±1.10	0.92±0.87	31	0.9

				2006	1.36±1.33	0.23±0.57	31	0.79
				2007	1.80±1.51	0.39±0.67	28	0.9
				2008	2.24±2.39	0.22±0.74	20	0.83
				2009	1.86±1.33	-0.03±0.25	26	0.55
				2010	2.04±1.88	0.05±0.33	26	0.65
				2011	1.45±0.97	0.20±0.37	24	0.7
				2012	1.34±1.18	0.07±0.27	21	0.54
US-Atq	70.4696	-157.409	WET	2003	0.12±0.15	0.38±0.28	22	0.31
				2004	1.00±0.90	-0.36±0.38	27	0.32
				2005	0.51±0.67	-0.02±0.18	31	0.23
				2006	0.46±0.57	0.06±0.07	32	0.22
				2007	0.62±0.74	-0.03±0.12	32	0.21
				2008	0.65±0.60	-0.01±0.10	24	0.24
US-Blo	38.8953	-120.633	ENF	1999	0.88±1.11	1.56±0.83	7	2.26
				2000	1.41±0.00	0.16±0.00	1	0.61
				2001	2.29±0.64	-0.09±0.30	5	0.8
				2002	4.25±4.02	-0.78±1.49	3	1.62
				2003	2.42±0.20	-0.03±0.19	3	0.84
				2004	3.36±2.34	-0.32±0.69	5	1.24
				2005	3.59±1.75	-0.14±0.46	17	1.16
				2006	1.59±0.41	0.41±0.14	2	0.88
US-Cop	38.09	-109.39	GRA	2001	0.20±0.18	0.10±0.15	24	0.21
				2002	0.12±0.09	0.02±0.04	27	0.1
				2003	0.26±0.22	-0.03±0.08	27	0.12
				2006	0.14±0.13	0.03±0.05	21	0.1
				2007	0.21±0.16	0.04±0.06	25	0.16
US-CRT	41.6285	-83.3471	CRO	2011	2.21±2.29	0.02±0.38	35	0.7
				2012	1.93±1.87	0.09±0.29	25	0.7

				2013	1.57±1.43	0.28±0.66	29	0.82
US-GBT	41.3658	-106.24	ENF	2001	0.03±0.06	0.09±0.11	5	0.26
				2002	0.36±0.85	0.16±0.16	26	0.44
				2003	0.66±1.12	0.11±0.34	23	0.59
US-GLE	41.3665	-106.24	ENF	2005	1.23±1.53	0.86±0.58	20	1.06
				2006	1.18±1.21	0.39±0.47	21	0.8
				2007	1.72±1.20	-0.26±0.69	14	0.79
				2008	1.28±0.68	-0.17±0.48	22	0.64
				2009	1.28±0.52	-0.27±0.51	16	0.57
				2010	1.45±1.19	0.00±0.52	28	0.72
				2011	1.67±1.58	0.08±0.57	30	0.74
				2012	1.67±1.53	0.02±0.24	34	0.54
				2013	1.77±1.71	0.03±0.20	33	0.56
				2014	1.73±1.26	-0.06±0.25	31	0.56
US-Goo	34.2547	-89.8735	GRA	2002	3.07±2.52	0.23±0.57	13	0.99
				2003	2.60±2.12	0.59±0.32	13	1.01
				2004	3.65±2.88	-0.00±0.49	28	0.96
				2005	2.20±2.00	0.10±0.33	22	0.69
				2006	3.62±2.42	-0.10±0.41	31	0.93
US-Ha1	42.5378	-72.1715	DBF	1999	2.58±1.61	0.08±0.84	22	1
				2000	2.41±1.70	-0.26±0.65	19	0.83
				2001	2.47±1.14	0.17±0.89	21	0.97
				2002	3.61±1.97	-0.48±1.01	8	1.12
				2003	1.92±0.90	0.17±0.76	13	0.83
				2004	3.14±1.92	-0.27±0.67	25	1.02
				2005	1.57±0.94	0.19±0.34	18	0.62
				2006	2.53±1.49	0.02±0.32	22	0.67
				2007	2.79±1.91	0.05±0.56	19	0.83

				2008	1.65±0.98	0.20±0.25	13	0.57
				2009	2.27±1.22	-0.08±0.26	5	0.54
				2010	3.89±2.58	-0.75±0.66	6	0.97
				2011	4.39±2.86	-0.43±0.60	10	0.95
				2012	2.38±1.27	-0.12±0.28	17	0.55
US-IB2	41.8406	-88.241	GRA	2004	1.41±0.82	-0.07±0.24	9	0.61
				2005	2.23±1.98	0.14±0.56	30	0.79
				2006	2.43±2.81	-0.11±1.03	29	0.72
				2007	2.08±1.84	0.45±0.54	29	0.87
				2008	2.14±2.25	0.13±0.19	30	0.59
				2009	2.39±2.17	0.04±0.22	33	0.56
				2010	2.20±2.09	-0.05±0.19	28	0.46
				2011	2.67±2.46	0.09±0.16	33	0.55
US-Ivo	68.4865	-155.75	WET	2004	0.57±0.64	0.10±0.20	32	0.4
				2005	0.79±0.75	0.07±0.20	30	0.37
				2006	0.94±0.92	-0.05±0.43	28	0.39
				2007	1.18±1.16	-0.01±0.18	22	0.43
US-KS1	28.4583	-80.6709	ENF	2002	4.32±0.99	0.23±0.28	21	1.08
US-KS2	28.6086	-80.6715	CSH	2003	5.18±1.56	-0.29±1.24	24	1.49
				2004	4.40±1.55	-0.11±0.43	36	0.8
				2005	4.01±1.43	0.24±0.27	36	0.85
				2006	3.80±1.13	0.27±0.33	36	0.85
US-Lin	36.3566	-119.842	CRO	2009	0.11±0.11	0.34±0.16	5	0.62
				2010	0.53±0.38	0.66±0.30	21	1.27
US-Los	46.0827	-89.9792	WET	2000	1.09±0.80	0.08±0.22	5	0.67
				2001	0.92±0.76	0.12±0.28	24	0.58
				2002	1.37±1.75	0.38±0.53	31	0.66
				2003	1.10±1.38	0.31±0.33	26	0.67

				2004	1.50±1.65	0.31±0.35	30	0.73
				2005	1.63±1.95	0.10±0.21	31	0.52
				2006	1.38±1.66	0.11±0.24	29	0.5
				2007	4.50±0.81	0.00±0.31	5	1
				2008	4.43±1.58	-0.04±0.31	8	0.98
				2010	5.32±1.29	-0.25±0.27	7	0.97
				2014	1.83±2.10	0.05±0.16	31	0.51
US-Me1	44.5794	-121.5	ENF	2004	1.16±0.90	-0.09±0.19	16	0.39
				2005	0.77±0.80	-0.01±0.25	14	0.37
US-Me2	44.4523	-121.557	ENF	2002	2.92±2.00	0.40±0.98	24	1.59
				2003	2.53±2.37	1.75±0.78	10	1.86
				2004	2.96±1.95	0.31±0.73	25	1.54
				2005	2.76±2.30	0.39±1.24	33	1.51
				2006	2.92±2.11	1.43±1.10	16	1.63
				2007	2.24±2.33	1.18±0.70	28	1.47
				2008	2.53±2.66	0.40±0.81	29	1.43
				2009	2.27±2.88	0.65±0.95	33	1.32
				2010	2.56±2.74	0.14±0.59	28	1.09
				2011	3.04±2.81	0.61±0.54	18	1.36
				2012	4.96±3.47	-0.05±0.91	10	1.52
				2013	4.16±2.58	-0.53±0.63	22	1.15
				2014	3.90±2.89	-0.36±0.60	15	1.23
US-Me3	44.3154	-121.608	ENF	2004	2.28±1.19	-0.28±0.48	32	0.84
				2005	2.19±1.20	-0.04±0.65	25	0.85
				2006	2.37±1.13	-0.28±0.55	26	0.55
				2007	2.10±1.17	0.01±0.21	27	0.53
				2008	2.03±1.35	-0.06±0.31	35	0.57
				2009	1.98±1.56	0.06±0.31	26	0.54

US-Me4	44.4992	-121.622	ENF	1999	1.85±1.43	0.14±0.43	7	0.99
				2000	2.52±1.12	0.17±0.31	17	1.01
US-Me5	44.4372	-121.567	ENF	2000	1.94±1.02	0.16±0.27	27	0.7
				2001	1.71±1.19	0.10±0.29	33	0.62
				2002	2.02±1.27	-0.03±0.24	32	0.62
US-Me6	44.3233	-121.608	ENF	2010	3.39±1.50	-0.16±0.46	6	0.74
				2011	1.07±0.85	0.15±0.14	22	0.47
				2012	0.48±0.67	0.25±0.20	6	0.49
				2013	2.21±1.85	-0.11±0.43	23	0.67
				2014	1.98±1.52	-0.04±0.34	24	0.63
US-MMS	39.3232	-86.4131	DBF	1999	2.91±2.28	0.42±0.91	32	1.02
				2000	3.09±2.55	-0.17±0.93	25	0.9
				2001	2.72±2.07	0.09±0.73	26	0.97
				2002	2.88±2.26	0.02±0.73	29	1
				2003	3.19±2.76	-0.34±1.19	27	1.03
				2004	3.44±2.74	-0.12±1.13	31	1.02
				2005	3.00±2.55	0.11±1.14	31	1.07
				2006	3.03±2.29	-0.01±0.71	30	1.04
				2007	2.26±1.86	0.50±0.67	26	1.12
				2008	2.83±2.25	-0.02±0.57	29	1
				2009	3.40±2.84	-0.24±0.99	32	1
				2010	2.57±2.05	0.27±0.85	28	1.15
				2011	3.02±2.10	0.02±0.93	29	1.02
				2012	3.12±1.94	0.09±0.37	29	0.86
				2013	3.14±2.17	0.08±0.44	34	0.75
				2014	3.36±2.51	-0.17±0.34	33	0.67
US-Myb	38.0498	-121.765	WET	2011	1.98±1.92	0.73±0.83	19	1.64
				2012	6.03±3.83	-0.45±1.04	17	1.55

				2013	5.72±1.89	-0.31±0.54	24	1.34
				2014	2.85±0.53	0.19±0.57	15	1.25
US-Ne1	41.1651	-96.4766	CRO	2001	3.35±2.73	-0.23±0.85	11	1.05
				2002	1.93±2.36	-0.22±1.30	23	0.64
				2003	2.07±2.49	-0.17±1.22	26	0.65
				2004	2.43±2.69	-0.04±0.55	23	0.79
				2005	1.99±1.97	0.09±0.82	19	0.7
				2006	2.66±2.91	-0.44±0.93	26	0.73
				2007	3.00±3.05	-0.25±0.75	27	0.88
				2008	2.17±2.20	-0.24±0.69	25	0.63
				2009	2.89±3.34	-0.14±0.32	26	0.55
				2010	2.51±3.13	-0.23±0.46	20	0.42
				2011	2.34±1.93	-0.39±0.51	24	0.43
				2012	2.36±2.14	-0.23±0.29	20	0.4
				2013	1.02±0.74	-0.02±0.08	13	0.24
US-Ne2	41.1649	-96.4701	CRO	2001	6.64±2.84	-0.62±1.61	11	1.81
				2002	2.97±2.80	0.16±1.56	18	1.16
				2003	2.45±2.71	-0.04±0.93	31	0.78
				2004	2.57±2.57	0.16±1.02	32	0.92
				2005	2.48±2.58	0.09±0.65	32	0.83
				2006	2.54±2.92	0.33±1.01	34	0.95
				2007	2.32±2.59	0.28±0.66	30	0.86
				2008	1.67±1.75	0.65±0.81	29	0.74
				2009	1.86±2.35	0.29±0.42	28	0.58
				2010	2.58±2.91	0.09±0.42	30	0.57
				2011	2.50±2.47	0.07±0.45	29	0.53
				2012	2.57±2.54	0.04±0.37	30	0.6
				2013	1.01±0.78	0.22±0.22	15	0.35

US-Ne3	41.1797	-96.4397	CRO	2001	5.34±2.83	-1.20±1.24	13	1.47
				2002	1.73±1.79	0.12±0.85	31	0.69
				2003	2.25±2.29	-0.12±0.90	29	0.79
				2004	2.04±2.41	-0.21±1.14	29	0.71
				2005	2.44±2.41	-0.11±0.63	33	0.83
				2006	2.10±2.35	0.11±0.70	32	0.84
				2007	2.49±2.68	-0.03±0.61	33	0.86
				2008	1.99±2.21	0.26±0.65	32	0.74
				2009	2.17±2.83	0.08±0.59	31	0.73
				2010	2.54±2.89	-0.01±0.58	33	0.64
				2011	2.19±2.38	0.04±0.23	32	0.57
				2012	1.52±1.42	0.17±0.27	26	0.53
				2013	0.94±0.79	0.07±0.14	15	0.3
US-NR1	40.0329	-105.546	ENF	1999	2.04±1.38	0.09±0.24	19	0.86
				2000	1.61±1.38	0.15±0.39	33	0.77
				2001	1.47±1.36	0.00±0.39	31	0.73
				2002	1.52±1.45	0.13±0.40	32	0.82
				2003	1.49±1.36	0.12±0.37	30	0.78
				2004	1.72±1.52	0.02±0.56	30	0.96
				2005	1.76±1.52	0.17±0.38	33	0.95
				2006	1.65±1.38	0.07±0.35	29	0.83
				2007	1.69±1.59	0.00±0.30	30	0.7
				2008	1.47±1.42	0.10±0.25	32	0.7
				2009	1.43±1.30	0.14±0.21	28	0.73
				2010	1.50±1.49	0.29±0.30	32	0.77
				2011	1.44±1.41	0.17±0.19	32	0.64
				2012	1.86±1.69	0.01±0.24	33	0.57
				2013	1.69±1.75	0.07±0.19	33	0.56

				2014	1.29±1.15	0.11±0.16	28	0.51
US-Oho	41.5545	-83.8438	DBF	2004	2.28±2.12	0.54±0.81	29	0.96
				2005	1.92±1.78	0.40±0.46	25	0.88
				2006	2.77±2.23	0.06±0.59	34	0.88
				2007	2.54±2.20	0.10±0.68	23	0.95
				2008	2.44±2.14	0.26±0.56	23	0.93
				2009	2.48±2.09	-0.00±0.54	30	0.87
				2010	1.90±1.68	0.04±0.26	26	0.61
				2011	1.97±2.06	0.16±0.31	30	0.61
				2012	2.37±1.84	0.10±0.30	31	0.65
				2013	1.84±2.06	0.13±0.25	27	0.59
US-ORv	40.0201	-83.0183	WET	2011	1.02±0.48	0.21±0.23	16	0.58
US-PFa	45.9459	-90.2723	MF	1999	3.13±3.44	-0.73±1.46	31	0.95
				2000	2.57±2.60	-0.37±0.64	33	0.93
				2001	2.31±2.35	-0.51±0.74	29	0.85
				2002	2.22±3.11	-0.69±1.39	27	0.7
				2003	2.83±2.49	-0.30±0.67	27	1.01
				2004	2.31±2.30	-0.39±0.55	31	0.84
				2005	2.10±2.40	-0.13±0.53	20	0.79
				2006	1.24±1.34	0.49±0.67	25	0.77
				2007	1.40±1.43	0.90±1.06	31	0.95
				2008	1.22±1.55	0.63±0.82	31	0.8
				2009	1.30±1.89	0.49±0.94	30	0.82
				2010	0.47±0.48	0.48±0.45	22	0.55
				2011	1.34±1.59	0.34±0.47	30	0.68
				2012	1.20±1.92	0.30±0.58	28	0.64
				2013	1.99±2.74	-0.11±0.39	27	0.55
				2014	1.90±2.49	-0.04±0.21	31	0.54

US-Prr	65.1237	-147.488	ENF	2010	0.22±0.17	0.00±0.04	8	0.14
				2011	0.64±0.91	0.12±0.21	18	0.34
				2012	0.59±0.83	0.21±0.33	12	0.4
				2013	0.29±0.30	0.08±0.11	19	0.25
				2014	1.04±1.17	0.22±0.22	24	0.59
US-SRC	31.9083	-110.84	OSH	2008	0.56±0.76	-0.00±0.21	20	0.25
				2009	0.32±0.37	-0.06±0.13	33	0.18
				2010	0.50±0.51	-0.12±0.19	33	0.23
				2011	0.21±0.33	0.01±0.09	34	0.15
				2012	0.30±0.34	0.01±0.07	28	0.16
				2013	0.02±0.04	0.03±0.03	20	0.08
				2014	0.14±0.12	0.04±0.06	15	0.12
US-SRG	31.7894	-110.828	GRA	2008	1.68±1.51	-0.26±0.60	28	0.57
				2009	0.71±0.80	-0.03±0.45	35	0.36
				2010	1.34±1.44	-0.16±0.48	36	0.54
				2011	1.10±0.96	-0.21±0.37	36	0.41
				2012	1.18±1.01	-0.06±0.23	36	0.33
				2013	0.85±0.81	0.02±0.19	36	0.28
				2014	1.21±1.14	-0.02±0.21	36	0.34
US-SRM	31.8214	-110.866	WSA	2004	0.65±0.50	-0.06±0.29	33	0.28
				2005	0.84±0.85	-0.09±0.44	34	0.35
				2006	1.03±0.98	-0.27±0.55	35	0.35
				2007	0.86±0.67	-0.11±0.36	30	0.31
				2008	0.75±0.91	0.06±0.30	31	0.33
				2009	0.59±0.58	0.01±0.34	36	0.3
				2010	0.82±0.82	0.05±0.39	36	0.34
				2011	0.70±0.72	0.09±0.22	35	0.29
				2012	0.85±0.59	-0.02±0.14	34	0.23

				2013	0.75±0.60	0.00±0.12	29	0.21
				2014	0.75±0.62	0.02±0.13	28	0.23
US-Sta	41.3966	-106.802	OSH	2005	0.01±0.02	0.02±0.01	5	0.08
				2006	0.00±0.00	0.03±0.04	2	0.08
				2007	0.20±0.31	0.00±0.10	26	0.13
				2008	0.21±0.32	0.01±0.07	33	0.12
				2009	0.15±0.22	0.03±0.08	32	0.12
US-Syv	46.242	-89.3477	MF	2001	1.66±2.11	0.93±1.15	11	1.05
				2002	1.86±2.48	0.61±0.48	28	0.91
				2003	2.11±2.48	1.07±0.98	23	1.04
				2004	3.19±2.44	0.10±0.87	28	1.21
				2005	2.82±2.72	0.01±0.44	21	0.99
				2006	3.37±3.20	0.06±0.38	31	0.77
				2007	7.59±3.21	-0.17±1.05	4	1.37
				2012	4.24±3.02	-0.11±0.86	16	1.05
				2013	3.83±3.70	-0.42±0.56	19	0.92
				2014	2.90±3.63	-0.03±0.63	33	0.8
US-Ton	38.4316	-120.966	WSA	2001	1.24±0.52	0.19±0.56	18	0.69
				2002	1.64±0.99	0.12±0.60	30	0.77
				2003	1.89±1.50	0.01±0.78	26	0.91
				2004	1.87±1.15	0.07±0.49	22	0.79
				2005	1.94±1.44	-0.07±1.16	18	0.86
				2006	1.90±1.42	-0.07±0.81	27	0.8
				2007	2.11±1.54	-0.42±0.88	26	0.83
				2008	1.73±0.98	-0.05±0.84	29	0.78
				2009	2.26±1.57	-0.37±1.04	32	0.84
				2010	2.21±1.34	-0.19±0.83	30	0.93
				2011	2.14±1.11	-0.15±0.35	23	0.6

				2012	1.95±1.38	-0.10±0.39	29	0.59
				2013	1.38±0.85	-0.02±0.23	25	0.45
				2014	1.75±1.19	-0.06±0.33	27	0.57
US-Tw1	38.1074	-121.647	WET	2012	5.38±0.68	0.73±0.86	6	1.67
				2013	4.97±1.99	-0.40±0.65	19	1.16
				2014	5.16±0.73	-0.50±0.54	16	1.18
US-Tw2	38.1047	-121.643	CRO	2012	5.76±3.27	-0.58±0.77	22	1.41
				2013	2.34±1.46	0.28±0.32	9	0.79
US-Tw3	38.1159	-121.647	CRO	2013	7.23±2.08	-0.63±0.45	14	1.41
				2014	7.08±1.55	-0.28±0.45	20	1.41
US-Tw4	38.103	-121.641	WET	2013	1.46±0.59	0.01±0.24	2	0.35
				2014	2.81±1.40	0.86±0.62	23	1.17
US-Twt	38.1087	-121.653	CRO	2009	3.33±1.21	2.45±1.21	21	2.28
				2010	4.04±1.74	0.78±1.80	27	1.87
				2011	4.45±2.49	-0.03±0.72	30	1.27
				2012	3.80±2.30	0.36±1.15	29	1.4
				2013	5.84±3.22	-0.58±1.24	25	1.53
				2014	6.80±2.65	-0.60±0.88	22	1.53
US-UMB	45.5598	-84.7138	DBF	2000	1.99±1.79	0.29±0.38	27	0.69
				2001	1.78±1.69	0.27±0.26	27	0.62
				2002	2.08±2.10	0.01±0.34	28	0.57
				2003	1.54±1.41	0.18±0.18	25	0.48
				2004	2.06±1.94	-0.24±0.42	24	0.51
				2005	2.20±2.13	0.08±0.84	17	0.61
				2006	1.73±1.47	0.20±0.33	19	0.55
				2007	2.16±2.06	-0.17±0.73	25	0.55
				2008	2.53±2.11	-0.21±0.49	30	0.62
				2009	1.41±1.24	0.01±0.37	21	0.43

				2010	1.93±1.46	0.00±0.27	26	0.57
				2011	1.73±1.58	0.00±0.14	25	0.34
				2012	2.44±2.10	-0.03±0.23	26	0.45
				2013	1.73±1.60	0.10±0.28	26	0.35
				2014	2.00±1.69	-0.05±0.13	25	0.38
US-UMd	45.5625	-84.6975	DBF	2007	3.33±1.89	0.25±0.93	17	0.85
				2008	2.51±2.00	0.27±0.41	34	0.71
				2009	2.45±2.09	0.31±0.43	34	0.68
				2010	2.45±1.96	0.26±0.42	32	0.7
				2011	1.98±1.66	0.24±0.24	29	0.45
				2012	2.91±2.20	0.04±0.27	33	0.47
				2013	2.68±2.06	-0.02±0.15	31	0.43
				2014	2.90±2.02	-0.01±0.30	27	0.61
US-Var	38.4133	-120.951	GRA	2000	3.50±1.65	-1.50±1.81	6	0.68
				2001	2.04±1.83	-0.11±1.05	34	1.04
				2002	1.69±1.78	0.08±0.85	36	0.92
				2003	2.37±2.17	-0.41±1.06	34	1.1
				2004	1.62±1.69	0.09±0.90	34	0.86
				2005	2.19±2.24	-0.50±0.92	27	1.02
				2006	1.97±1.81	-0.14±0.84	33	0.96
				2007	1.76±1.86	-0.06±0.74	33	1.02
				2008	1.17±1.13	0.25±0.63	31	0.83
				2009	1.94±1.76	-0.04±0.74	35	1.02
				2010	1.97±1.88	-0.07±0.90	30	1.02
				2011	2.31±1.63	-0.21±0.97	35	0.9
				2012	1.90±1.81	-0.08±0.51	32	0.72
				2013	1.33±1.58	0.04±0.32	36	0.59
				2014	1.13±1.46	0.04±0.31	26	0.56

US-WCr	45.8059	-90.0799	DBF	1999	1.77±1.09	0.36±1.06	27	0.85
				2000	2.58±2.20	-0.20±0.83	32	0.9
				2001	2.67±3.52	-0.63±2.23	27	0.8
				2002	1.19±1.05	0.10±0.71	23	0.62
				2003	1.99±1.55	0.13±0.93	30	0.83
				2004	1.83±1.29	-0.45±0.63	21	0.65
				2005	2.48±2.21	-0.34±0.88	31	0.85
				2006	2.23±2.30	0.04±0.68	26	0.85
				2010	0.05±0.10	0.61±0.08	4	0.33
				2011	0.83±1.31	0.42±0.28	26	0.58
				2012	2.05±1.69	0.36±0.39	31	0.77
				2013	2.66±2.56	-0.10±0.52	32	0.7
				2014	2.29±1.95	-0.24±0.19	20	0.54
US-Whs	31.7438	-110.052	OSH	2007	0.53±0.64	0.12±0.21	18	0.3
				2008	0.31±0.59	0.17±0.16	36	0.22
				2009	0.31±0.41	0.02±0.17	36	0.19
				2010	0.48±0.51	0.05±0.16	36	0.27
				2011	0.33±0.50	0.04±0.14	36	0.19
				2012	0.33±0.41	0.03±0.08	36	0.14
				2013	0.45±0.59	-0.00±0.09	36	0.15
				2014	0.52±0.53	0.02±0.11	35	0.19
US-Wi0	46.6188	-91.0814	ENF	2002	2.54±2.15	0.19±0.38	20	0.9
US-Wi1	46.7305	-91.2329	DBF	2003	6.35±3.09	-1.04±1.28	5	1.64
US-Wi2	46.6869	-91.1528	ENF	2003	0.45±0.64	0.36±0.31	2	0.59
US-Wi3	46.6347	-91.0987	DBF	2002	2.05±1.15	0.49±0.35	17	1.1
				2004	1.34±1.19	0.55±0.60	21	1.06
US-Wi4	46.7393	-91.1663	ENF	2002	2.30±1.40	0.22±0.26	17	0.85
				2003	4.25±0.60	-0.39±0.37	4	0.74

				2004	1.62±1.20	0.44±0.33	20	0.96
				2005	2.96±1.26	0.01±0.58	17	1.04
US-Wi5	46.6531	-91.0858	ENF	2004	2.33±1.63	0.42±0.44	22	1.15
US-Wi6	46.6249	-91.2982	OSH	2002	1.02±0.89	1.01±0.66	17	1.45
US-Wi7	46.6491	-91.0693	OSH	2005	2.37±1.72	0.74±0.54	13	1.35
US-Wi8	46.7223	-91.2524	DBF	2002	4.40±2.58	-0.01±0.63	18	1.19
US-Wi9	46.6188	-91.0814	ENF	2004	1.61±1.21	0.11±0.43	7	0.86
				2005	1.83±1.22	0.91±0.52	6	1.69
US-Wkg	31.7365	-109.942	GRA	2004	0.51±0.41	0.05±0.23	21	0.32
				2005	0.29±0.25	0.15±0.15	33	0.27
				2006	0.30±0.52	0.33±0.27	34	0.32
				2007	0.47±0.43	0.14±0.29	33	0.29
				2008	0.30±0.39	0.14±0.18	29	0.19
				2009	0.43±0.44	0.08±0.21	36	0.26
				2010	0.55±0.66	0.08±0.22	29	0.29
				2011	0.48±0.47	0.03±0.17	31	0.19
				2012	0.73±0.69	-0.06±0.15	33	0.19
				2013	0.62±0.58	-0.03±0.10	34	0.17
				2014	0.57±0.71	0.04±0.11	29	0.22
US-WPT	41.4646	-82.9962	WET	2011	2.19±2.26	-0.07±0.43	27	0.55
				2012	1.89±1.66	0.04±0.43	28	0.56
				2013	2.04±1.80	0.07±0.29	31	0.53
ZM-Mon	-15.4378	23.2528	DBF	2000	4.86±0.00	0.63±0.00	1	1.15
				2007	4.96±2.05	-0.07±0.39	8	0.97
				2008	4.53±2.09	-0.29±0.48	21	0.91
				2009	4.62±3.47	-0.16±0.71	6	0.87

Table S4. Statistics of site NEE fitting.

Site ID	Latitude	Longitude	IGBP	Year	Observed Flux (gC m-2 d-1)	Prediction Bias (gC m-2 d-1)	ND	Model SD （gC m-2 d-1)
AR-SLu	-33.4648	-66.4598	MF	2009	-6.40±0.00	1.71±0.00	1	1.91
				2010	-3.60±1.85	0.42±0.53	32	1.25
				2011	-4.42±1.29	0.47±0.53	8	1.29
AR-Vir	-28.2395	-56.1886	ENF	2010	-1.95±1.39	0.44±0.57	7	2.04
				2011	2.49±4.64	-1.64±2.45	4	2.71
				2012	4.16±2.81	-1.77±1.76	9	3.22
AT-Neu	47.1167	11.3175	GRA	2002	0.88±2.21	-0.66±2.48	35	1.55
				2003	0.59±1.53	-0.46±1.67	34	1.46
				2004	0.81±1.64	-0.16±1.83	21	1.41
				2005	0.68±2.46	-0.53±2.51	35	1.66
				2006	0.80±2.80	-1.12±2.69	25	1.7
				2007	0.30±2.13	0.15±0.72	23	1.29
				2008	1.89±1.46	-0.18±0.51	19	0.97
				2009	0.77±1.82	-0.09±0.48	15	0.9
				2010	1.63±2.09	-0.27±1.10	16	1.06
				2011	1.19±2.11	-0.29±0.75	13	1.1
				2012	1.50±2.37	-0.61±1.02	28	1.31
AU-Ade	-13.0769	131.1178	WSA	2007	-0.84±1.02	-0.09±0.20	6	0.87
				2008	-0.65±0.68	0.01±0.31	35	0.66
				2009	-2.50±1.28	0.47±0.29	14	0.98
AU-ASM	-22.283	133.249	ENF	2010	-0.19±0.45	0.17±0.33	11	0.51
				2011	-0.25±0.66	0.06±0.44	33	0.56
				2012	0.14±0.37	-0.04±0.12	35	0.22
				2013	0.04±0.17	0.03±0.09	34	0.18
				2014	-0.02±0.40	0.01±0.12	31	0.25

AU-Cpr	-34.0021	140.5891	SAV	2010	-0.25±0.27	0.20±0.20	15	0.55
				2011	-0.61±0.38	0.10±0.31	36	0.66
				2012	-0.57±0.37	0.05±0.14	36	0.4
				2013	-0.22±0.39	-0.03±0.18	35	0.34
				2014	0.22±0.33	-0.03±0.13	27	0.29
AU-Cum	-33.6152	150.7236	EBF	2012	-1.15±0.64	0.38±0.37	7	0.84
				2013	-1.06±0.63	0.24±0.25	28	0.89
				2014	0.26±0.94	-0.38±0.39	32	0.94
AU-DaP	-14.0633	131.3181	GRA	2007	-0.63±1.57	0.42±1.67	3	1.58
				2008	-0.68±2.45	0.42±0.84	32	1.21
				2009	0.38±1.15	0.06±0.52	30	0.94
				2010	0.14±1.87	-0.39±0.61	26	1.03
				2011	-1.04±1.77	0.21±0.41	33	0.92
				2012	-0.21±1.72	-0.04±0.41	19	0.8
				2013	-1.34±1.99	0.12±0.28	25	0.86
AU-DaS	-14.1593	131.3881	SAV	2008	-0.66±0.87	-0.06±0.68	21	0.98
				2009	-0.40±0.73	-0.07±0.78	25	0.92
				2010	-0.73±1.01	0.13±0.62	23	0.97
				2011	-0.14±1.33	-0.24±0.49	27	0.74
				2012	-0.96±1.25	0.03±0.39	16	0.73
				2013	-0.96±1.35	0.02±0.43	35	0.76
				2014	-0.97±0.72	0.13±0.38	30	0.74
AU-Dry	-15.2588	132.3706	SAV	2008	0.17±0.65	-0.28±0.47	9	0.69
				2009	-0.35±0.80	-0.39±0.58	25	0.86
				2010	-0.74±0.76	0.08±0.22	27	0.7
				2011	-1.09±0.67	0.05±0.26	16	0.63
				2012	-0.88±0.70	0.05±0.19	32	0.56
				2013	-1.12±0.80	0.09±0.30	12	0.63

				2014	-0.68±0.98	-0.03±0.28	32	0.55
AU-Emr	-23.8587	148.4746	GRA	2011	-0.08±0.77	0.01±0.32	16	0.59
				2012	0.01±1.10	-0.07±0.41	34	0.76
				2013	0.23±0.73	-0.05±0.30	34	0.65
AU-Fog	-12.5452	131.3072	WET	2006	-1.71±0.71	-0.08±0.27	16	0.94
				2007	-1.20±1.05	0.03±0.27	23	0.73
				2008	-1.30±0.87	0.09±0.24	15	0.75
AU-Gin	-31.3764	115.7138	WSA	2011	-1.01±0.44	0.02±0.18	7	0.72
				2012	-0.55±0.63	-0.14±0.26	29	0.7
				2013	-0.65±0.67	-0.02±0.27	30	0.53
				2014	-1.00±0.63	0.10±0.19	35	0.6
AU-GWW	-30.1913	120.6541	SAV	2013	-0.32±0.27	0.02±0.14	35	0.3
				2014	-0.36±0.32	0.01±0.14	31	0.32
AU-How	-12.4943	131.1523	WSA	2001	-0.24±1.35	-1.09±0.42	7	1.2
				2002	-1.59±1.27	0.43±0.61	5	0.99
				2003	-2.73±2.08	0.79±1.18	10	1.15
				2004	-1.14±1.43	-0.22±0.68	19	1.14
				2005	-1.68±1.38	-0.02±0.73	17	1.07
				2006	-2.09±0.98	0.26±0.76	11	1.23
				2007	-1.48±0.95	-0.03±0.60	10	1.14
				2008	-1.46±0.76	0.03±0.75	18	1.18
				2009	-2.36±1.02	0.19±1.01	10	1.16
				2010	-2.04±1.04	0.16±0.37	11	0.86
				2011	-1.70±1.16	0.05±0.43	26	0.86
				2012	-1.61±1.50	-0.06±0.48	26	0.85
				2013	-1.76±1.22	0.06±0.45	25	0.86
				2014	-1.66±1.11	0.02±0.46	19	0.88
AU-Lox	-34.4704	140.6551	DBF	2008	-3.85±2.21	0.55±0.57	11	1.49

				2009	-2.77±2.83	0.61±0.84	15	1.41
AU-RDF	-14.5636	132.4776	WSA	2011	-1.27±0.56	0.16±0.35	7	1.03
				2012	1.26±1.41	-0.47±0.66	14	0.89
				2013	0.88±0.13	-0.14±0.13	6	0.59
AU-Rig	-36.6499	145.5759	GRA	2011	-0.16±1.16	0.01±0.49	32	0.9
				2012	-0.33±1.41	0.19±0.34	35	0.78
				2013	-0.20±1.99	0.01±0.50	17	0.92
				2014	-0.36±1.74	-0.01±0.36	26	0.86
AU-Rob	-17.1175	145.6301	EBF	2014	-2.10±1.11	0.07±0.70	7	1.19
AU-Stp	-17.1507	133.3502	GRA	2008	0.04±0.52	0.52±0.50	13	0.35
				2009	-0.03±0.71	0.19±0.19	27	0.55
				2010	-0.04±0.98	0.34±0.51	35	0.6
				2011	-0.49±1.01	0.12±0.55	32	0.83
				2012	-0.09±0.98	0.04±0.24	33	0.46
				2013	0.09±0.96	0.04±0.20	30	0.42
				2014	-0.14±0.94	0.03±0.24	34	0.46
AU-TTE	-22.287	133.64	OSH	2012	0.52±0.24	-0.00±0.08	17	0.21
				2013	0.56±0.25	-0.02±0.10	35	0.21
				2014	0.26±0.47	0.00±0.14	34	0.32
AU-Tum	-35.6566	148.1517	EBF	2001	-1.32±1.80	-0.98±1.75	31	1.75
				2002	-1.09±3.89	-1.48±3.76	31	1.93
				2003	-2.43±3.14	-0.23±3.13	34	2.25
				2004	-1.13±2.33	-1.74±2.36	23	2.04
				2005	-0.84±1.75	-1.85±1.69	34	2.02
				2006	-1.83±1.18	-1.02±1.08	35	2.16
				2007	-1.25±1.22	-1.47±1.08	36	2.15
				2008	-1.89±1.16	-0.63±1.40	36	1.62
				2009	-1.69±1.22	-0.65±1.18	36	1.81

				2010	-1.54±1.33	-0.87±1.14	29	1.93
				2011	-1.67±1.53	-0.54±1.37	32	1.63
				2012	-2.16±3.48	0.14±1.99	22	1.4
				2013	-2.40±2.07	0.04±0.96	36	1.33
				2014	-1.63±1.17	-0.30±0.63	36	1.37
AU-Wac	-37.4259	145.1878	EBF	2005	-4.09±2.59	-0.06±1.59	8	1.82
				2006	-4.75±1.72	0.09±0.91	25	1.3
				2007	-5.25±2.01	0.48±1.01	31	1.57
				2008	-4.73±1.57	0.24±0.80	20	1.35
AU-Whr	-36.6732	145.0294	EBF	2011	-2.14±0.67	0.80±0.54	3	0.73
				2012	-1.04±0.71	0.02±0.28	36	0.66
				2013	-0.92±0.82	0.05±0.32	36	0.65
				2014	-1.28±0.93	0.19±0.37	36	0.82
AU-Wom	-37.4222	144.0944	EBF	2010	-2.44±1.71	-0.48±1.20	26	1.5
				2011	-3.61±1.24	0.36±1.00	29	1.21
				2012	-3.52±1.42	0.07±0.53	31	1.08
				2013	-1.78±1.22	-0.27±0.54	31	1.12
				2014	-3.63±1.98	0.33±0.64	30	1.27
AU-Ync	-34.9893	146.2907	GRA	2012	0.17±0.43	0.08±0.16	5	0.35
				2013	-0.15±0.93	0.05±0.20	28	0.48
				2014	0.67±0.11	-0.07±0.10	6	0.29
BE-Bra	51.3076	4.5198	MF	1999	0.31±1.84	-0.73±0.50	18	1.32
				2000	1.01±1.36	-0.48±0.65	26	1.21
				2001	-0.37±1.54	-0.37±0.80	26	1.35
				2002	-0.15±1.38	0.04±0.61	26	1.23
				2004	0.26±1.82	-0.37±0.67	24	1.23
				2005	-0.66±2.29	0.12±0.65	31	1.32
				2006	0.09±1.57	-0.19±0.80	30	1.34

				2007	-0.46±1.90	-0.01±0.75	29	1.35
				2008	-0.22±1.78	-0.18±0.91	27	1.29
				2009	-0.48±2.29	-0.05±0.69	29	1.33
				2010	0.15±1.66	-0.28±0.61	31	1.12
				2011	0.89±2.12	-0.33±0.60	26	0.92
				2012	0.82±1.96	-0.19±0.49	23	0.88
				2013	-0.94±2.28	0.16±0.42	25	0.92
				2014	-0.76±2.20	0.24±0.43	26	0.98
BE-Lon	50.5516	4.7461	CRO	2004	-2.57±4.77	1.58±3.19	23	1.94
				2005	-1.44±4.16	1.04±3.80	35	1.63
				2006	-0.29±2.44	-0.21±1.36	35	1.58
				2007	-1.30±3.92	0.74±3.37	36	1.74
				2008	-2.27±4.63	1.62±3.78	35	1.68
				2009	-0.88±4.05	0.22±2.96	33	1.66
				2010	0.92±1.55	-1.29±1.23	32	1.52
				2011	-0.78±4.30	-0.03±3.37	33	1.74
				2012	-0.99±3.40	0.16±1.35	35	1.41
				2013	-1.30±3.94	0.31±1.16	35	1.35
				2014	0.20±2.27	-0.39±0.67	34	1.16
BE-Vie	50.305	5.9981	MF	1999	-0.63±2.08	0.69±0.82	21	1.02
				2000	-0.28±1.39	0.18±0.63	30	1.06
				2001	-0.91±1.62	0.49±0.64	31	1.19
				2002	-1.13±1.99	0.84±0.85	28	1.17
				2003	-1.25±1.67	0.79±0.87	23	1.21
				2004	-0.92±1.97	0.75±1.12	22	1.11
				2005	-1.02±1.93	0.45±0.81	31	1.26
				2006	-0.95±1.57	0.73±0.73	29	1.17
				2007	-1.40±1.73	0.98±0.71	31	1.13

				2008	-1.08±1.91	0.86±0.54	24	1.09
				2009	-0.18±2.16	0.46±0.97	19	0.85
				2010	-0.52±2.18	-0.00±0.50	26	0.86
				2011	0.39±1.37	-0.20±0.40	22	0.74
				2012	-0.25±1.54	-0.19±0.37	26	0.91
				2013	-0.30±2.10	-0.02±0.42	19	0.85
				2014	-0.16±2.21	0.08±0.58	16	0.73
BR-Sa1	-2.8567	-54.9589	EBF	2002	0.66±1.44	-0.43±0.51	17	0.72
				2003	-0.05±1.25	0.11±0.63	16	0.74
				2004	-0.28±1.09	0.08±0.57	13	0.68
				2005	-0.39±2.29	0.14±1.04	9	0.82
				2006	-0.28±0.00	0.81±0.00	1	0.59
				2008	-0.84±1.05	0.17±0.70	8	0.82
				2009	0.16±1.19	-0.18±0.50	14	0.67
				2010	0.26±1.05	-0.23±0.51	12	0.76
				2011	-0.47±1.20	0.28±0.50	16	0.75
BR-Sa3	-3.018	-54.9714	EBF	2000	-1.29±0.80	0.41±0.38	10	0.8
				2001	-0.30±1.02	-0.12±0.42	12	0.75
				2002	0.26±1.45	-0.35±0.37	12	0.71
				2003	-0.32±1.17	0.03±0.53	18	0.7
				2004	-0.40±0.91	0.37±0.66	2	0.81
CA-Gro	48.2167	-82.1556	MF	2003	-0.16±0.22	0.45±0.32	4	0.8
				2004	-0.45±1.65	-0.08±0.94	29	1.1
				2005	-0.08±1.09	-0.55±0.75	30	1.2
				2006	0.05±0.98	-0.46±0.36	33	0.96
				2007	-0.09±1.27	-0.45±0.49	33	1.05
				2008	-0.19±1.21	-0.24±0.64	32	1
				2009	-0.11±1.03	-0.19±0.46	32	0.97

				2010	-0.43±1.29	-0.10±0.48	33	1.03
				2011	0.04±0.97	-0.08±0.18	26	0.59
				2012	-0.10±0.92	-0.12±0.23	32	0.66
				2013	-0.33±1.00	-0.12±0.20	29	0.79
				2014	0.24±0.45	-0.01±0.12	14	0.48
CA-Man	55.8796	-98.4808	ENF	1999	-0.10±0.73	-0.16±0.41	28	0.72
				2000	0.03±0.66	-0.12±0.33	30	0.64
				2001	-0.20±0.78	-0.05±0.31	32	0.69
				2002	-0.07±0.74	-0.03±0.13	32	0.42
				2003	-0.05±0.72	-0.08±0.19	29	0.4
				2004	0.28±0.07	-0.00±0.05	5	0.11
				2006	0.20±0.41	0.02±0.08	10	0.33
				2007	0.12±1.17	0.03±0.27	11	0.4
				2008	-0.15±0.75	-0.05±0.26	24	0.54
CA-NS1	55.8792	-98.4839	ENF	2002	-0.60±0.96	0.03±0.29	14	0.5
				2003	-0.45±1.29	0.07±0.23	27	0.38
				2004	-0.19±1.37	-0.01±0.30	25	0.4
				2005	-0.25±1.01	-0.04±0.16	22	0.42
CA-NS2	55.9058	-98.5247	ENF	2001	-0.56±0.79	0.17±0.24	15	0.51
				2002	-0.50±0.96	0.15±0.18	31	0.42
				2003	-0.58±1.07	0.10±0.16	24	0.41
				2004	-0.35±1.19	0.07±0.22	29	0.4
				2005	-1.30±0.81	0.06±0.23	11	0.7
CA-NS3	55.9117	-98.3822	ENF	2001	0.13±0.34	-0.15±0.25	15	0.61
				2002	-0.22±0.85	0.08±0.21	36	0.44
				2003	-0.23±0.99	0.09±0.26	30	0.27
				2004	-0.05±0.97	0.02±0.29	32	0.31
				2005	-0.10±1.04	-0.11±0.43	26	0.4

CA-NS4	55.9144	-98.3806	ENF	2002	0.13±0.14	0.10±0.05	6	0.13
				2003	-0.20±0.68	-0.20±0.27	19	0.46
				2004	-0.14±0.50	-0.08±0.29	22	0.38
				2005	-0.20±0.54	0.00±0.27	27	0.39
CA-NS5	55.8631	-98.485	ENF	2001	0.20±0.66	-0.17±0.58	13	0.56
				2002	-0.31±1.01	0.10±0.25	29	0.5
				2003	-0.53±1.24	-0.03±0.33	27	0.62
				2004	-0.18±0.94	-0.04±0.22	36	0.45
				2005	-0.43±1.05	-0.05±0.27	27	0.6
CA-NS6	55.9167	-98.9644	OSH	2001	0.38±0.19	0.19±0.18	7	0.33
				2002	-0.08±0.77	0.11±0.31	34	0.57
				2003	-0.02±0.83	0.03±0.20	35	0.5
				2004	0.10±0.66	-0.00±0.11	32	0.37
				2005	-0.23±0.85	-0.02±0.18	26	0.54
CA-NS7	56.6358	-99.9483	OSH	2002	0.36±0.59	-0.09±0.16	15	0.4
				2003	0.31±0.74	-0.05±0.18	27	0.32
				2004	0.24±0.66	-0.03±0.14	34	0.33
				2005	0.09±0.77	-0.08±0.21	24	0.4
CA-Oas	53.6289	-106.198	DBF	1999	0.01±1.96	-0.49±1.01	34	0.91
				2000	-0.33±2.27	-0.05±0.77	34	0.97
				2001	-0.49±2.25	0.02±0.46	33	0.94
				2002	-0.28±1.85	-0.31±0.76	35	1.04
				2003	-0.03±1.73	-0.34±0.85	34	0.98
				2004	0.19±1.88	-0.24±0.72	34	0.84
				2005	-0.16±2.18	-0.29±0.71	35	0.94
				2006	-0.27±2.40	-0.25±0.67	33	0.89
				2007	0.27±1.76	-0.52±0.76	34	0.97
				2008	-0.02±2.03	-0.13±0.32	34	0.65

				2009	-0.23±2.22	-0.07±0.32	34	0.64
				2010	-0.14±2.33	-0.11±0.33	34	0.65
CA-Obs	53.9872	-105.118	ENF	1999	-0.24±0.92	-0.06±0.50	22	0.92
				2000	-0.07±0.77	-0.04±0.36	32	0.79
				2001	0.01±0.90	0.06±0.41	25	0.66
				2002	-0.06±0.90	-0.02±0.82	32	0.76
				2003	-0.08±0.77	0.08±0.35	27	0.74
				2004	0.09±0.66	0.01±0.36	29	0.69
				2005	0.00±0.73	-0.07±0.39	31	0.75
				2006	-0.09±0.68	-0.03±0.36	30	0.78
				2007	-0.03±0.80	-0.17±0.65	35	0.75
				2008	0.03±0.93	-0.10±0.14	33	0.54
				2009	0.04±0.91	-0.11±0.26	31	0.57
				2010	-0.05±0.82	-0.06±0.22	32	0.57
CA-Qfo	49.6925	-74.3421	ENF	2003	0.20±0.57	0.08±0.25	9	0.64
				2004	-0.05±0.71	-0.11±0.28	30	0.74
				2005	0.03±0.63	-0.17±0.35	36	0.7
				2006	-0.01±0.65	-0.20±0.33	36	0.73
				2007	0.02±0.61	-0.23±0.38	36	0.72
				2008	0.03±0.63	-0.07±0.15	35	0.52
				2009	0.00±0.58	-0.03±0.16	36	0.48
				2010	-0.04±0.70	-0.09±0.16	29	0.44
CA-SF1	54.485	-105.818	ENF	2003	0.73±1.26	-0.57±0.56	7	1.15
				2004	-0.73±1.21	-0.02±0.40	16	0.93
				2005	-0.38±1.65	-0.29±0.66	16	1.12
				2006	-0.57±1.74	-0.13±0.54	16	1.01
CA-SF2	54.2539	-105.878	ENF	2001	1.04±1.36	-0.81±0.51	8	1.36
				2002	-1.15±1.80	0.06±0.79	16	1.27

				2003	-1.59±0.94	0.15±0.31	11	1.13
				2004	-1.09±1.25	-0.05±0.45	12	1
				2005	-0.77±1.83	-0.13±0.79	15	1.25
CA-SF3	54.0916	-106.005	OSH	2002	0.32±0.20	0.12±0.09	3	0.69
				2003	-0.32±0.60	0.09±0.22	16	0.8
				2004	-0.22±0.58	0.02±0.19	16	0.64
				2005	-0.11±0.66	-0.13±0.26	16	0.7
				2006	-0.15±0.50	-0.12±0.22	16	0.77
CA-TP1	42.6609	-80.5595	ENF	2003	0.21±0.51	-0.37±0.44	14	1.05
				2004	-0.05±0.63	-0.32±0.74	15	1.12
				2005	0.58±0.38	-1.15±1.25	10	1.29
				2006	-0.06±0.44	-0.78±1.11	6	1.54
				2007	0.34±0.32	-0.10±0.29	11	0.56
				2008	-0.14±1.05	-0.13±0.78	10	1.08
				2009	-0.36±0.82	-0.10±0.86	28	1.31
				2010	-0.35±0.85	0.08±0.74	29	1.13
				2011	-0.49±1.05	0.07±0.47	33	0.89
				2012	-0.53±1.32	0.09±0.51	31	0.87
				2013	0.16±1.26	-0.13±0.66	25	0.68
				2014	0.56±0.62	-0.18±0.35	19	0.53
CA-TP2	42.7744	-80.4588	ENF	2003	-1.86±1.76	0.34±0.52	19	1.03
				2004	-1.83±1.88	0.27±0.67	16	1.06
				2005	-1.47±2.42	-0.03±0.92	14	1.2
				2006	-0.98±2.48	-0.03±0.79	15	0.95
				2007	-3.99±2.98	0.95±1.08	11	1.73
CA-TP3	42.7068	-80.3483	ENF	2003	-1.44±1.03	0.71±0.78	18	1.28
				2004	-0.89±0.88	-0.04±1.50	16	1.33
				2005	-1.07±0.73	0.40±0.95	13	1.1

				2006	-2.07±1.27	-0.14±1.39	7	1.91
				2007	-1.10±0.47	1.05±0.77	9	0.8
				2008	-0.83±1.20	-0.17±0.86	36	1.24
				2009	-1.15±1.70	0.21±0.90	36	1.22
				2010	-1.23±1.51	0.20±0.62	35	1.24
				2011	-0.80±1.43	0.20±0.81	32	1.08
				2012	-1.07±1.62	0.10±0.48	35	0.91
				2013	-1.34±1.93	0.19±0.34	35	0.85
				2014	-1.68±2.31	0.27±0.60	33	0.94
CA-TP4	42.7102	-80.3574	ENF	2002	-0.12±0.80	-0.48±0.60	19	1.22
				2003	-0.31±1.13	0.04±0.56	32	1.05
				2004	-0.12±1.19	-0.21±0.68	35	1.12
				2005	-0.22±1.02	-0.05±0.49	33	1.04
				2006	-0.26±1.25	-0.02±0.79	33	0.99
				2007	0.16±0.99	-0.15±0.36	27	0.96
				2008	-0.00±1.23	-0.12±0.88	29	0.99
				2009	0.02±0.92	-0.02±0.59	26	0.96
				2010	0.24±1.27	-0.31±1.07	24	0.9
				2011	-0.52±1.30	0.10±0.39	32	0.79
				2012	-0.07±1.04	-0.14±0.32	31	0.73
				2013	0.02±1.30	-0.05±0.36	23	0.69
				2014	0.04±1.32	-0.17±0.38	29	0.74
CA-TPD	42.6353	-80.5577	DBF	2012	-0.39±2.05	-0.09±0.40	28	0.83
				2013	0.31±1.44	-0.24±0.43	23	0.72
				2014	-0.30±2.42	-0.07±0.29	28	0.63
CG-Tch	-4.2892	11.6564	SAV	2006	0.02±0.75	0.00±0.21	16	0.33
				2007	-0.28±1.08	0.05±0.26	21	0.39
				2008	-0.09±0.68	-0.05±0.22	27	0.43

				2009	-0.58±1.04	0.06±0.28	28	0.45
CH-Cha	47.2102	8.4104	GRA	2005	-0.18±1.21	0.40±1.27	5	1.02
				2006	-0.12±1.82	-0.57±1.36	12	1.3
				2007	0.99±3.01	-0.62±2.07	16	1.5
				2008	0.38±1.88	-0.61±1.27	10	1.51
				2009	0.27±1.43	-0.17±0.85	7	1.2
				2010	0.38±1.38	-0.45±0.53	12	1.17
				2011	0.24±1.78	-0.21±0.75	18	1.17
				2012	0.68±1.26	-0.34±0.86	21	1.22
				2013	-1.10±2.09	0.36±0.87	20	1.14
				2014	-0.65±2.24	0.06±0.77	13	1.38
CH-Dav	46.8153	9.8559	ENF	1999	0.14±1.21	-1.35±0.86	28	1.09
				2000	0.03±1.58	-1.04±0.83	26	1.08
				2001	-0.25±1.36	-0.99±1.02	22	1.35
				2002	-0.19±1.54	-0.89±0.77	23	1.18
				2003	1.11±1.35	-1.93±0.97	25	1.05
				2004	0.22±1.38	-1.46±0.81	30	1.08
				2005	-1.29±0.81	0.29±0.74	10	1.17
				2006	-1.05±1.11	-0.35±0.75	28	1.19
				2007	-1.23±1.21	-0.19±1.14	24	1.17
				2008	-1.19±1.65	0.07±1.15	28	1.13
				2009	-1.19±1.79	-0.15±1.14	28	1.09
				2010	-1.20±1.61	-0.06±1.46	29	1.15
				2011	-2.03±1.79	0.11±0.58	23	0.96
				2012	-1.27±1.29	-0.04±0.53	29	0.91
				2013	-1.51±1.34	0.15±0.50	27	0.89
				2014	-1.94±1.41	0.26±0.58	33	0.94
CH-Fru	47.1158	8.5378	GRA	2005	-0.11±1.74	-0.12±1.79	12	1.25

				2006	-0.26±1.63	-0.38±0.52	19	1.07
				2007	-0.92±1.86	-0.06±0.47	19	1.01
				2008	-1.54±2.74	0.16±0.62	10	1.13
				2009	-0.15±1.86	-0.19±0.47	10	0.98
				2010	-0.35±2.37	-0.02±0.81	10	1.11
				2011	-2.41±2.42	0.57±0.57	10	1.19
				2012	-0.33±2.31	-0.35±0.75	11	1.03
				2013	-0.36±2.35	-0.19±0.42	12	0.96
				2014	0.13±2.15	-0.48±0.96	11	1.2
CH-Lae	47.4781	8.365	MF	2004	-1.43±1.15	0.72±0.06	2	1.57
				2005	-2.93±1.10	-1.31±1.32	7	1.65
				2006	-0.75±1.69	0.04±0.90	10	1.24
				2007	-2.54±2.86	-0.05±0.53	6	1.13
				2008	-2.91±3.17	-0.09±0.47	10	1.12
				2009	-1.72±2.73	0.12±0.66	18	1.19
				2010	-1.40±2.52	-0.22±0.90	5	1.35
				2011	-1.03±2.81	0.09±0.90	10	1.22
				2012	-0.87±3.21	-0.23±0.87	9	1.26
				2013	-2.86±3.09	0.39±0.93	19	1.32
				2014	-3.28±2.32	0.42±0.69	20	1.3
CH-Oe1	47.2858	7.7319	GRA	2002	-1.19±1.76	0.96±1.17	22	1.1
				2003	-0.40±2.25	0.40±1.70	17	0.98
				2004	-1.44±2.33	0.47±0.98	21	1.08
				2005	-0.27±1.21	0.01±0.34	20	0.72
				2006	-0.33±1.15	0.09±0.51	25	0.76
				2007	-0.15±1.27	-0.11±0.52	23	0.75
				2008	0.27±1.56	-0.13±0.64	21	0.81
CH-Oe2	47.2863	7.7343	CRO	2004	-1.09±3.83	0.20±1.88	30	1.51

				2005	-0.14±2.78	0.13±2.23	33	1.44
				2006	1.47±1.54	-2.16±2.40	18	1.5
				2007	0.43±2.39	-0.65±1.27	25	1.45
				2008	0.18±2.36	-0.28±1.42	30	1.48
				2009	-0.27±2.02	0.10±1.42	29	1.3
				2010	1.78±1.28	-2.06±1.84	30	1.42
				2011	-0.53±2.97	0.23±0.79	24	1.02
				2012	0.23±2.89	-0.06±0.99	28	1.18
				2013	0.18±2.61	-0.23±0.70	30	1.08
				2014	-0.87±3.33	0.26±1.08	26	1.2
CN-Cha	42.4025	128.0958	MF	2003	-0.72±1.38	0.08±0.29	25	0.71
				2004	-0.52±1.20	-0.03±0.37	27	0.78
				2005	-0.38±1.59	0.06±0.28	19	0.69
CN-Cng	44.5934	123.5092	GRA	2007	-0.40±0.81	-0.06±0.24	21	0.64
				2008	-0.56±1.17	0.05±0.36	36	0.46
				2009	-0.54±0.96	0.08±0.30	32	0.43
				2010	-0.72±0.73	0.11±0.20	26	0.51
CN-Dan	30.4978	91.0664	GRA	2004	-0.35±0.55	0.06±0.16	32	0.25
				2005	-0.20±0.45	-0.03±0.06	34	0.26
CN-Din	23.1733	112.5361	EBF	2003	-1.39±1.22	-0.01±0.46	18	0.95
				2004	-2.12±0.83	0.24±0.37	21	0.89
				2005	-1.18±1.09	-0.05±0.32	23	0.88
CN-Du2	42.0467	116.2836	GRA	2007	-0.07±0.24	-0.03±0.14	33	0.28
				2008	-0.24±0.37	-0.02±0.27	32	0.36
CN-Du3	42.0551	116.2809	GRA	2009	0.04±0.27	-0.13±0.21	14	0.35
				2010	0.19±0.32	-0.13±0.16	13	0.24
CN-Ha2	37.6086	101.3269	WET	2003	-0.35±0.86	-0.17±0.26	31	0.57
				2004	-0.68±1.17	0.05±0.19	32	0.55

				2005	-0.57±1.09	-0.07±0.22	31	0.6
CN-HaM	37.37	101.18	GRA	2002	-0.00±0.84	-0.13±0.22	33	0.39
				2003	-0.29±0.93	0.03±0.16	35	0.35
				2004	-0.52±1.10	0.12±0.21	36	0.38
CN-Qia	26.7414	115.0581	ENF	2003	-1.21±0.80	0.12±0.48	15	0.86
				2004	-1.23±1.15	0.13±0.41	12	0.91
				2005	-1.15±1.02	0.11±0.27	17	0.9
CN-Sw2	41.7902	111.8971	GRA	2010	-0.93±0.00	0.15±0.00	1	0.31
				2011	0.43±1.37	-0.11±0.55	31	0.66
				2012	-0.48±0.35	0.03±0.39	2	0.54
CZ-BK1	49.5021	18.5369	ENF	2004	-1.88±2.41	0.15±1.24	19	1.45
				2005	-3.26±1.75	-0.04±1.33	14	1.68
				2006	-3.53±1.82	0.50±0.88	12	1.73
				2007	-3.02±2.17	0.32±1.19	18	1.9
				2008	-2.45±2.85	0.43±1.45	21	1.52
				2009	-1.35±2.92	-0.09±1.39	28	1.52
				2010	-2.20±2.56	0.38±1.19	25	1.53
				2011	-2.24±2.90	0.25±0.99	30	1.15
				2012	-2.19±2.28	0.06±0.46	28	1.01
				2013	-2.09±2.51	0.12±0.70	29	1.12
				2014	-2.71±2.53	0.20±0.52	31	1.02
CZ-BK2	49.4944	18.5429	GRA	2006	-0.09±1.43	-1.24±0.76	12	1.52
				2007	-0.22±1.47	-0.76±0.89	13	1.5
				2008	-0.24±1.70	-0.28±0.96	17	1.21
				2009	0.50±1.49	-0.32±0.78	28	0.93
				2010	0.31±1.79	-0.39±0.86	27	0.97
				2011	-0.24±1.57	-0.05±0.41	28	0.84
				2012	0.81±0.72	-0.32±0.37	21	0.76

CZ-wet	49.0247	14.7704	WET	2006	-0.28±2.49	0.03±1.48	18	1.48
				2007	0.19±1.01	0.20±0.68	26	0.97
				2008	-0.19±1.71	0.03±0.70	35	1.15
				2009	-0.32±1.34	0.19±0.76	34	1.18
				2010	-0.46±2.02	0.11±1.00	33	1.2
				2011	0.41±1.03	-0.16±0.39	27	0.74
				2012	0.58±1.11	-0.25±0.35	27	0.76
				2013	0.53±1.43	-0.15±0.50	24	0.64
				2014	0.52±1.24	-0.23±0.52	22	0.81
DE-Akm	53.8662	13.6834	WET	2009	0.80±0.19	0.85±0.25	6	0.68
				2010	-0.25±1.58	0.17±0.40	31	0.86
				2011	-1.14±2.62	0.31±0.49	13	1.06
				2012	-0.08±2.62	0.02±0.58	15	0.97
				2013	-0.02±2.96	-0.00±0.64	29	0.9
				2014	-0.20±2.41	-0.25±0.44	22	1.01
DE-Geb	51.1001	10.9143	CRO	2001	-0.40±3.65	-0.01±1.98	34	1.3
				2002	0.12±2.13	-0.23±2.21	34	1.17
				2003	-0.88±3.08	0.57±1.21	35	1.12
				2004	-0.64±3.04	0.04±1.38	36	1.21
				2005	-0.50±4.07	-0.21±2.67	34	1.23
				2006	-1.61±3.54	1.09±3.55	36	1.31
				2007	-0.34±2.39	-0.29±1.19	33	1.45
				2008	-0.59±3.24	-0.02±1.50	35	1.25
				2009	-0.10±2.05	-0.32±1.70	33	1.13
				2010	-0.78±3.42	0.44±1.62	36	1.2
				2011	0.03±0.98	-0.31±2.38	35	1.19
				2012	-0.29±1.22	0.18±0.44	36	0.83
				2013	-0.77±3.42	0.20±0.75	36	0.91

				2014	-0.77±3.03	0.12±0.60	35	0.99
DE-Gri	50.95	13.5126	GRA	2004	-0.68±1.79	0.23±1.22	34	1.34
				2005	-0.24±1.25	-0.04±0.83	30	1.19
				2006	0.04±0.86	-0.37±1.23	31	1.26
				2007	-0.34±1.63	-0.03±1.39	34	1.25
				2008	-0.08±1.37	-0.12±1.23	34	1.19
				2009	-0.50±1.83	0.08±1.29	35	1.28
				2010	-0.40±1.48	0.10±1.12	35	1.31
				2011	-0.47±1.91	-0.06±1.26	33	1.35
				2012	-0.23±2.07	-0.17±0.89	36	1.07
				2013	-0.18±1.63	-0.01±0.73	35	0.94
				2014	-0.47±1.93	-0.11±0.89	36	1.14
DE-Hai	51.0792	10.453	DBF	2000	-1.36±4.13	0.51±1.57	33	1.15
				2001	-1.76±3.97	0.63±1.44	35	1.24
				2002	-1.62±3.96	0.43±1.75	35	1.29
				2003	-1.25±3.82	0.22±1.35	34	1.2
				2004	-0.96±3.46	-0.05±1.25	35	1.16
				2005	-1.60±3.94	0.42±1.19	35	1.19
				2006	-1.13±3.52	0.29±1.61	34	1.27
				2007	-1.27±3.60	0.17±1.35	34	1.14
				2008	-1.47±4.02	0.14±1.44	36	1.19
				2009	-1.45±3.76	0.03±1.34	35	1.1
				2010	-1.36±3.43	-0.07±0.67	27	0.97
				2011	-1.10±3.47	-0.08±0.49	34	0.91
				2012	-1.23±3.72	0.08±0.50	35	0.88
DE-Kli	50.8931	13.5224	CRO	2004	-1.05±3.03	-0.04±1.57	17	1.55
				2005	-0.32±2.88	-0.21±1.94	32	1.23
				2006	-0.03±2.88	-0.35±1.50	33	1.28

				2007	-0.16±2.34	-0.04±1.95	35	1.25
				2008	-0.08±1.35	0.04±0.76	24	1.36
				2009	-0.04±3.68	-0.74±1.79	23	1.44
				2010	-0.28±2.54	-0.52±1.53	33	1.33
				2011	-0.99±3.00	0.43±1.04	35	1.17
				2012	0.05±1.69	-0.19±0.60	34	0.95
				2013	0.54±1.06	-0.06±0.27	25	0.57
				2014	-0.96±3.56	0.26±0.89	36	1.1
DE-Lkb	49.0996	13.3047	ENF	2009	0.84±0.47	-0.29±0.34	21	0.79
				2010	1.04±0.62	-0.19±0.36	14	0.63
				2011	0.78±0.71	-0.11±0.28	27	0.67
				2012	0.72±0.70	-0.18±0.30	19	0.73
				2013	0.35±0.92	-0.09±0.29	18	0.8
DE-Lnf	51.3282	10.3678	DBF	2002	-2.37±3.80	0.37±1.45	25	1.45
				2003	-1.20±3.52	0.29±1.06	33	1.21
				2004	-0.52±2.84	-0.32±0.88	33	1.2
				2005	-1.67±3.81	0.35±1.29	35	1.29
				2006	-1.37±3.49	-0.04±1.09	34	1.19
				2010	-1.81±4.27	0.29±0.79	32	0.99
				2011	-0.35±2.65	-0.19±0.51	28	0.87
				2012	-1.61±3.80	0.31±0.65	33	0.9
DE-Obe	50.7867	13.7213	ENF	2008	-0.66±1.91	-0.07±0.66	25	1.22
				2009	-0.16±1.91	-0.18±0.68	25	1.08
				2010	-0.20±1.63	0.00±0.85	28	1.11
				2011	-0.58±2.02	-0.01±0.31	26	0.75
				2012	-0.55±2.14	0.05±0.40	24	0.81
				2013	-0.70±2.31	0.12±0.58	31	0.87
				2014	-0.91±2.06	0.10±0.33	25	0.79

DE-RuR	50.6219	6.3041	GRA	2011	-0.31±1.32	-0.24±0.41	15	1.02
				2012	-0.61±1.63	0.11±0.49	27	0.87
				2013	-0.27±1.54	-0.04±0.58	34	0.87
				2014	0.05±1.36	-0.22±0.66	31	0.94
DE-RuS	50.8659	6.4472	CRO	2011	-5.35±3.27	1.36±1.20	17	2.34
				2012	-1.56±3.54	-0.27±1.25	19	1.88
				2013	-1.97±3.99	0.30±1.13	33	1.49
				2014	-2.30±3.74	0.38±1.28	30	1.7
DE-Seh	50.8706	6.4497	CRO	2007	-2.83±3.95	1.88±2.90	15	1.76
				2008	-0.99±3.67	0.02±1.30	33	1.44
				2009	-0.94±3.04	0.18±0.84	35	1.23
				2010	-2.02±2.61	0.50±1.43	28	1.67
DE-SfN	47.8064	11.3275	WET	2012	-0.58±0.82	-0.12±0.25	13	0.78
				2013	-0.27±0.44	-0.02±0.35	23	0.69
				2014	-0.91±0.90	0.07±0.24	19	0.73
DE-Spw	51.8923	14.0337	WET	2010	-1.37±1.71	-1.05±1.57	6	1.43
				2011	-0.64±2.64	0.19±0.45	28	0.72
				2012	-0.58±2.36	0.11±0.31	29	0.67
				2013	0.11±1.45	-0.14±0.30	27	0.59
				2014	-0.56±2.21	-0.08±0.34	30	0.75
DE-Tha	50.9624	13.5652	ENF	1999	-1.27±2.04	0.77±0.76	24	1.09
				2000	-1.59±1.96	0.65±1.02	26	1.2
				2001	-1.44±1.89	0.60±0.63	29	1.06
				2002	-1.58±1.83	0.69±0.90	31	1.18
				2003	-0.56±1.27	0.09±1.05	24	1.11
				2004	-1.26±1.94	0.48±0.86	30	1.25
				2005	-0.88±1.67	0.27±1.08	24	1.1
				2006	-0.80±1.55	0.16±0.91	27	1.12

				2007	-1.09±1.55	0.13±1.00	25	1.16
				2008	-0.70±1.26	0.55±0.55	19	0.96
				2009	-1.19±2.24	0.41±0.54	23	1.03
				2010	-0.97±1.83	0.49±1.13	22	1.06
				2011	-1.44±1.89	0.04±0.38	30	0.87
				2012	-1.70±2.26	0.20±0.31	29	0.87
				2013	-0.68±1.66	0.15±0.27	26	0.7
				2014	-1.60±1.92	0.08±0.37	29	0.88
DE-Zrk	53.8759	12.889	WET	2013	0.39±0.56	-0.39±0.56	17	1.04
				2014	0.41±0.49	-0.07±0.43	26	0.67
DK-Eng	55.6905	12.1918	GRA	2005	-0.04±1.19	-0.22±0.49	23	0.95
				2006	0.09±0.76	-0.08±0.30	30	0.72
				2007	-0.52±0.92	0.06±0.45	31	0.93
				2008	-0.73±0.94	-0.07±1.31	16	1.39
DK-Fou	56.4842	9.5872	CRO	2005	-0.89±1.36	0.02±0.44	24	0.97
DK-NuF	64.1308	-51.3861	WET	2008	-0.36±0.74	0.06±0.17	13	0.4
				2009	-0.09±0.56	0.05±0.10	15	0.36
				2010	-0.19±0.92	0.05±0.24	15	0.43
				2011	0.28±0.45	0.01±0.21	15	0.35
				2012	-0.05±0.89	0.02±0.17	12	0.43
				2013	-0.12±0.78	-0.05±0.14	16	0.42
				2014	-0.65±0.80	-0.20±0.23	6	0.61
DK-Sor	55.4859	11.6446	DBF	1999	0.14±2.60	-0.66±0.77	35	1.19
				2000	-0.12±2.90	-0.11±1.20	34	1.23
				2001	-0.35±2.88	-0.14±0.85	36	1.17
				2002	-0.38±2.75	-0.13±0.90	36	1.23
				2003	-0.38±2.88	-0.29±0.78	33	1.28
				2004	-0.36±2.97	-0.17±0.92	31	1.14

				2005	-0.33±2.93	-0.22±1.03	34	1.19
				2006	-0.16±2.70	-0.35±0.82	35	1.24
				2007	-0.33±2.37	-0.02±0.87	33	1.19
				2008	-0.57±3.02	0.06±0.95	33	1.22
				2009	-0.86±2.86	0.08±0.78	36	1.29
				2010	-0.33±2.38	-0.02±0.70	34	1.1
				2011	-0.80±2.88	0.21±1.25	35	1.14
				2012	-0.70±2.74	0.08±0.35	35	0.88
				2013	-1.04±3.14	0.19±0.50	33	0.9
				2014	-1.31±3.32	-0.12±0.60	27	1.24
DK-ZaF	74.4814	-20.5545	WET	2008	0.70±0.00	-0.67±0.00	1	0.48
				2009	0.41±0.08	-0.56±0.06	3	0.44
				2010	0.40±0.17	-0.51±0.08	5	0.43
				2011	0.49±0.18	-0.62±0.21	4	0.44
DK-ZaH	74.4733	-20.5503	GRA	2000	-0.18±0.37	0.05±0.15	9	0.22
				2001	0.06±0.50	-0.12±0.24	9	0.26
				2002	-0.04±0.40	-0.07±0.22	8	0.25
				2003	-0.12±0.48	-0.11±0.24	9	0.23
				2004	-0.10±0.47	-0.07±0.21	9	0.2
				2005	-0.34±0.45	0.09±0.22	10	0.22
				2006	-0.25±0.38	0.09±0.16	9	0.19
				2007	-0.18±0.40	0.12±0.24	15	0.19
				2008	-0.05±0.39	-0.01±0.23	17	0.17
				2009	-0.06±0.29	0.04±0.12	16	0.16
				2010	0.05±0.38	-0.05±0.14	19	0.16
				2011	0.08±0.01	-0.16±0.14	2	0.26
				2012	-0.08±0.43	0.00±0.13	14	0.15
				2013	0.01±0.35	-0.02±0.20	18	0.19

				2014	0.01±0.33	-0.06±0.11	15	0.31
ES-Amo	36.8336	-2.2523	OSH	2007	0.91±0.76	-0.27±0.43	14	0.44
				2008	0.89±0.64	-0.81±0.64	9	0.55
				2009	0.48±0.65	0.10±0.27	35	0.43
				2010	0.37±0.69	0.00±0.09	36	0.35
				2011	0.46±0.81	-0.04±0.20	30	0.35
				2012	0.63±0.73	-0.06±0.14	35	0.34
ES-LgS	37.0979	-2.9658	OSH	2007	-0.32±0.63	-0.04±0.22	36	0.58
				2008	-0.25±0.59	-0.12±0.13	32	0.63
				2009	-0.43±0.88	0.04±0.26	16	0.56
ES-LJu	36.9266	-2.7521	OSH	2004	-0.03±0.43	0.18±0.31	16	0.48
				2005	0.20±0.16	0.08±0.11	19	0.44
				2006	0.08±0.34	0.01±0.21	28	0.51
				2007	0.01±0.52	-0.03±0.28	26	0.57
				2008	0.00±0.56	-0.23±0.38	29	0.7
				2009	0.01±0.55	-0.01±0.39	36	0.49
				2010	-0.08±0.54	0.04±0.24	30	0.51
				2011	-0.18±0.54	0.06±0.14	36	0.38
				2012	0.15±0.40	-0.01±0.16	28	0.33
				2013	0.01±0.46	-0.02±0.17	26	0.35
ES-Ln2	36.9695	-3.4758	OSH	2009	0.50±0.27	-0.18±0.27	19	0.54
FI-Hyy	61.8474	24.2948	ENF	1999	-0.25±1.17	-0.12±0.63	34	0.87
				2000	-0.56±1.34	-0.02±0.34	31	1.02
				2001	-0.52±1.44	0.12±0.32	35	0.89
				2002	-0.52±1.29	-0.14±0.46	34	0.88
				2003	-0.28±1.36	-0.22±0.65	35	0.84
				2004	-0.59±1.44	-0.04±0.49	35	0.88
				2005	-0.91±1.30	0.08±0.50	28	1.02

				2006	-0.46±1.60	-0.14±0.52	33	0.94
				2007	-0.58±1.44	0.07±0.43	36	0.91
				2008	-0.61±1.54	0.12±0.40	35	0.82
				2009	-0.75±1.69	0.09±0.38	36	0.83
				2010	-0.44±1.23	0.14±0.34	31	0.87
				2011	-0.70±1.81	0.20±0.57	35	0.85
				2012	-0.50±1.58	0.05±0.17	34	0.53
				2013	-0.59±1.79	0.09±0.26	34	0.58
				2014	-0.75±1.53	0.05±0.20	34	0.63
FI-Jok	60.8986	23.5135	CRO	2000	0.59±0.35	-0.20±0.32	24	0.6
				2001	0.53±0.92	-0.35±0.52	33	0.8
				2002	0.16±0.53	-0.17±0.54	32	0.72
				2003	-0.01±0.89	0.03±0.15	11	0.54
FI-Let	60.6418	23.9595	ENF	2009	1.28±0.68	-0.17±0.43	8	0.51
				2010	0.17±1.51	-0.09±0.33	26	0.72
				2011	0.42±1.78	-0.17±0.30	21	0.69
				2012	0.29±1.69	-0.14±0.38	29	0.71
FI-Lom	67.9972	24.2092	WET	2007	0.01±0.94	-0.08±0.18	30	0.5
				2008	-0.10±1.03	0.03±0.19	33	0.42
				2009	0.00±1.13	-0.04±0.35	32	0.48
FI-Sod	67.3624	26.6386	ENF	2001	0.21±0.91	-0.01±0.69	32	0.75
				2002	-0.11±1.21	0.10±0.76	22	0.98
				2003	0.42±0.84	-0.17±0.47	32	0.62
				2004	0.38±0.86	-0.13±0.52	35	0.68
				2005	0.51±0.83	-0.22±0.41	34	0.62
				2006	0.16±0.83	-0.04±0.50	36	0.67
				2007	0.32±0.98	-0.07±0.63	32	0.6
				2008	0.20±0.77	0.04±0.38	36	0.58

				2009	0.22±0.93	-0.10±0.53	33	0.7
				2010	0.36±0.83	-0.12±0.34	28	0.51
				2011	0.32±0.78	-0.05±0.25	34	0.58
				2012	0.35±0.68	0.01±0.19	31	0.34
				2013	0.25±0.85	-0.06±0.20	32	0.43
				2014	0.31±0.87	-0.04±0.15	34	0.42
FR-Fon	48.4764	2.7801	DBF	2005	-2.21±3.13	0.44±1.04	27	1.51
				2006	-1.62±3.13	0.52±1.09	33	1.34
				2007	-1.91±3.73	0.63±1.77	36	1.39
				2008	-1.59±3.55	0.39±1.33	34	1.19
				2009	-1.69±3.44	0.23±1.08	35	1.25
				2010	-1.56±3.49	0.25±1.26	33	1.22
				2011	-1.76±3.68	0.05±0.94	34	1.11
				2012	-1.42±2.96	-0.07±0.57	36	1.01
				2013	-1.03±3.32	0.05±0.49	31	0.87
				2014	-0.47±2.45	-0.14±0.39	19	0.79
FR-Gri	48.8442	1.9519	CRO	2004	-0.87±3.88	-0.01±3.01	20	1.92
				2005	-1.02±3.72	0.64±3.70	31	1.6
				2006	-0.67±3.25	0.25±1.71	32	1.49
				2007	-0.84±3.20	0.13±2.69	32	1.86
				2008	-1.89±3.25	1.55±2.66	21	1.58
				2009	-1.44±3.76	0.32±1.02	14	1.22
				2010	-1.12±3.01	0.30±0.81	18	1.03
				2011	-1.98±3.16	0.77±1.06	18	1.52
				2012	-1.82±2.68	-0.06±0.73	12	1.43
				2013	-0.83±2.77	-0.07±0.81	26	1.25
				2014	-1.61±3.61	0.23±1.12	20	1.48
FR-LBr	44.7171	-0.7693	ENF	1999	-0.75±1.00	0.01±0.53	15	1.17

				2000	-0.51±2.24	-0.12±0.91	12	1.47
				2001	-1.14±1.93	0.30±0.95	23	1.32
				2002	0.29±0.93	-0.87±0.59	24	1.3
				2003	-0.12±1.51	-0.42±1.43	10	1.43
				2004	-1.64±1.78	0.28±0.68	21	1.07
				2005	-0.40±1.50	-0.17±0.44	29	0.83
				2006	-0.69±1.57	0.03±0.49	29	0.94
				2007	-2.09±1.63	0.53±0.57	19	1.04
				2008	-0.78±1.45	0.05±0.63	15	0.99
FR-Pue	43.7413	3.5957	EBF	2000	-0.16±1.21	0.09±0.88	14	0.98
				2001	-0.71±1.36	0.14±0.92	34	0.96
				2002	-0.65±1.00	0.11±0.65	35	1.04
				2003	-0.31±1.32	-0.23±1.03	34	0.97
				2004	-0.95±1.24	0.33±0.93	35	0.95
				2005	-0.16±1.36	-0.33±0.99	35	1.03
				2006	-0.26±1.14	-0.14±0.80	33	1.05
				2007	-1.02±1.16	0.21±0.87	36	1.07
				2008	-0.78±1.03	0.03±0.72	36	1.05
				2009	-0.81±0.98	0.04±0.79	34	1.07
				2010	-0.46±0.90	-0.13±0.68	33	0.95
				2011	-0.76±1.22	0.25±0.60	29	0.88
				2012	-0.66±1.02	-0.04±0.31	30	0.78
				2013	-0.54±0.44	-0.06±0.23	35	0.66
				2014	-0.50±0.57	0.05±0.18	35	0.69
GF-Guy	5.2788	-52.9249	EBF	2004	-0.30±0.91	-0.12±0.84	23	0.76
				2005	-0.45±1.29	0.00±1.30	22	0.72
				2006	-0.23±0.92	-0.15±0.84	35	0.77
				2007	-0.69±0.98	0.33±0.86	34	0.68

				2008	-0.91±0.76	0.45±0.68	21	0.8
				2009	-1.02±1.10	0.57±1.06	20	0.74
				2010	-0.24±1.14	-0.15±1.12	36	0.79
				2011	-0.65±0.90	0.24±0.71	13	0.74
				2012	-0.59±1.00	0.04±0.66	35	0.62
				2013	-0.47±0.83	0.01±0.57	35	0.59
				2014	-0.13±0.85	-0.11±0.44	36	0.68
GH-Ank	5.2685	-2.6942	EBF	2011	-2.56±1.06	0.33±0.55	8	0.83
				2012	-1.77±0.40	-0.04±0.26	9	0.72
				2014	-1.69±0.04	-0.00±0.17	2	0.97
IT-BCi	40.5238	14.9574	CRO	2004	-1.41±3.80	0.44±2.47	21	2.41
				2005	-1.15±3.83	0.43±3.29	35	2.12
				2006	-0.95±4.23	0.12±3.84	33	2.21
				2007	-0.99±3.30	0.28±2.69	34	2.04
				2008	-1.42±4.26	0.79±3.76	30	1.96
				2009	-0.20±2.53	0.13±2.67	34	1.76
				2010	-1.43±3.87	0.94±3.78	33	1.85
				2011	-0.75±4.69	0.24±2.17	21	1.94
				2012	-0.32±2.15	0.06±1.09	28	1.49
				2013	-0.13±2.17	-0.11±1.05	32	1.37
				2014	-0.13±2.28	0.01±1.13	31	1.37
IT-CA1	42.3804	12.0266	DBF	2011	-1.42±1.96	0.11±0.48	15	1.03
				2012	-0.48±2.17	0.03±0.81	26	0.89
				2013	0.15±0.57	-0.23±0.31	17	0.62
				2014	-0.78±1.46	-0.06±0.27	28	1.01
IT-CA2	42.3772	12.026	CRO	2011	-0.81±2.07	0.12±1.08	10	1.34
				2012	-0.08±1.44	-0.20±0.57	24	1.09
				2013	-0.94±2.35	0.23±0.64	24	1

				2014	0.55±1.98	-0.33±0.71	7	1.3
IT-CA3	42.38	12.0222	DBF	2011	0.45±0.27	-0.11±0.09	4	0.39
				2012	-0.36±0.84	0.02±0.34	16	0.85
				2013	-0.50±1.25	0.05±0.24	18	0.69
				2014	-1.81±2.00	0.46±0.95	5	1.25
IT-Col	41.8494	13.5881	DBF	1999	-3.87±3.29	0.85±0.97	14	1.6
				2000	-1.37±3.67	0.86±1.09	9	1.09
				2001	-0.50±2.44	0.18±0.47	26	1.1
				2002	0.07±2.03	0.04±0.64	8	1.27
				2003	-6.47±0.00	0.35±0.00	1	1.53
				2004	-1.55±3.22	0.85±1.14	14	1.24
				2005	-1.91±3.60	0.37±0.82	20	1.18
				2006	-0.24±2.54	0.05±0.66	10	1.08
				2007	-1.30±3.25	0.19±0.81	27	1.26
				2008	-1.23±3.15	0.16±0.79	26	1.24
				2009	-1.69±4.01	0.30±0.56	19	0.88
				2010	-2.66±4.71	0.18±0.70	9	1.08
				2011	-1.28±3.02	-0.05±0.38	31	0.93
				2012	-1.89±3.59	0.04±0.37	13	0.99
				2013	-0.68±2.88	0.00±0.28	19	0.81
				2014	-0.02±2.49	-0.03±0.40	22	0.75
IT-Cp2	41.7043	12.3573	EBF	2012	-1.69±1.16	0.03±0.51	18	1.04
				2013	-1.60±1.31	0.01±0.51	33	0.99
				2014	-2.07±0.82	-0.05±0.34	28	1.15
IT-Cpz	41.7053	12.3761	EBF	2000	-1.69±1.11	-0.12±0.89	27	1.44
				2001	-1.42±1.17	-0.06±0.83	24	1.4
				2002	-1.10±1.08	-0.58±0.94	21	1.44
				2003	-1.39±1.33	-0.01±0.91	30	1.26

				2004	-1.63±1.34	0.03±0.96	25	1.35
				2005	-1.53±1.09	0.02±0.37	26	0.77
				2006	-1.07±0.96	-0.17±0.40	31	0.87
				2007	-1.06±0.99	-0.12±0.36	23	0.92
				2008	-2.14±1.54	0.23±0.62	21	1.16
IT-Isp	45.8126	8.6336	DBF	2013	-0.58±3.33	0.10±0.82	4	1.15
				2014	1.63±2.07	-0.35±0.34	6	0.89
IT-La2	45.9542	11.2853	ENF	2000	-1.87±1.98	0.02±0.54	10	1.14
				2001	-2.46±2.27	0.17±0.41	17	1.05
				2002	-1.18±1.72	-0.01±0.47	9	0.99
IT-Lav	45.9562	11.2813	ENF	2003	-4.49±2.79	0.40±1.47	31	2.08
				2004	-4.60±2.88	1.04±1.45	30	1.99
				2005	-4.69±2.87	0.67±1.55	24	1.99
				2006	-4.70±3.10	0.77±1.31	34	1.98
				2007	-4.86±2.33	0.86±0.89	32	1.87
				2008	-4.98±2.82	1.18±1.48	29	1.94
				2009	-6.11±4.04	1.81±1.97	32	1.85
				2010	-5.16±3.40	1.41±1.91	32	1.94
				2011	-4.96±2.97	0.64±0.81	28	1.61
				2012	-4.90±3.04	0.53±0.91	28	1.49
				2013	-4.69±2.37	0.38±0.56	34	1.37
				2014	-6.05±2.69	0.74±0.65	27	1.49
IT-MBo	46.0147	11.0458	GRA	2003	0.07±1.32	-0.46±1.37	33	1.06
				2004	-0.09±1.90	-0.17±0.98	36	1.1
				2005	-0.12±1.89	-0.31±0.99	36	1.11
				2006	-0.27±2.00	-0.02±0.96	35	1.05
				2007	-0.45±1.64	0.07±0.99	36	1.17
				2008	0.06±1.41	-0.01±1.07	33	1

				2009	0.01±1.93	-0.65±1.15	35	1.2
				2010	-0.26±1.83	-0.18±0.89	32	1.14
				2011	-0.47±2.13	0.04±0.57	35	0.88
				2012	-0.04±1.69	-0.15±0.47	36	0.85
				2013	0.10±1.89	-0.23±0.46	33	0.8
IT-PT1	45.2009	9.061	DBF	2002	-2.44±3.24	0.30±0.56	27	1.13
				2003	-1.25±2.78	0.11±0.38	35	0.92
				2004	-2.17±3.27	0.11±0.51	29	0.99
IT-Ren	46.5869	11.4337	ENF	1999	-0.77±1.13	-0.51±0.86	20	1.13
				2001	0.62±0.00	0.11±0.00	1	0.42
				2002	-1.87±1.87	0.48±0.71	22	1.18
				2003	-1.69±2.08	0.75±0.69	19	1.1
				2005	-1.18±2.04	0.40±0.67	15	1
				2006	-2.43±2.99	0.91±1.03	10	1.09
				2007	-1.23±2.10	0.07±0.29	16	0.71
				2008	-0.74±1.63	0.22±0.31	11	0.63
				2009	-1.53±3.02	0.28±0.62	20	0.8
				2010	-1.71±2.58	0.23±0.49	16	0.89
				2011	-1.24±2.24	0.20±0.33	12	0.68
				2012	-0.92±2.34	0.06±0.40	17	0.69
				2013	-1.14±2.49	0.05±0.45	17	0.82
IT-Ro1	42.4081	11.93	DBF	2000	0.47±1.40	-1.18±1.43	21	1.58
				2001	-0.80±1.92	-0.18±1.37	25	1.66
				2002	0.21±1.62	-1.30±1.06	31	1.76
				2003	1.12±0.93	-1.06±0.92	25	1.32
				2004	-0.61±2.45	-0.24±0.77	25	1.28
				2005	-0.86±2.61	-0.08±0.47	28	1.03
				2006	-0.87±2.15	-0.04±0.49	31	1.06

				2007	-1.26±2.96	-0.08±0.49	11	1.07
				2008	-0.81±3.13	-0.18±0.84	23	1.31
IT-Ro2	42.3903	11.9209	DBF	2002	-1.01±2.69	-0.32±1.40	29	1.78
				2003	-0.85±3.03	0.19±1.74	28	1.44
				2004	-1.71±3.36	0.68±1.52	29	1.56
				2005	-2.27±3.77	0.82±2.10	32	1.7
				2006	-1.44±3.41	0.71±1.91	27	1.44
				2007	-1.06±2.19	-0.07±0.45	24	1.28
				2008	-2.32±3.26	0.07±0.61	20	1.28
				2010	-2.68±3.74	0.39±0.83	34	1.33
				2011	-3.06±3.48	0.02±0.61	16	1.33
				2012	-0.06±2.15	-0.50±0.70	23	1.16
IT-SR2	43.732	10.291	ENF	2013	-1.26±1.50	0.11±0.49	31	0.89
				2014	-0.69±1.38	-0.08±0.46	33	1.01
IT-SRo	43.7279	10.2844	ENF	1999	-1.00±1.48	0.15±1.17	36	1.31
				2000	-1.07±1.23	0.54±0.92	32	1.32
				2001	-1.03±1.20	0.36±0.94	36	1.36
				2002	-0.99±1.30	0.44±0.78	27	1.39
				2003	-1.04±1.48	0.07±1.01	35	1.31
				2004	-1.29±1.27	0.78±0.88	33	1.24
				2005	-1.18±0.72	0.41±0.49	20	1.23
				2006	-1.58±1.70	0.81±1.10	35	1.28
				2007	-1.42±1.52	0.58±1.14	34	1.39
				2008	-0.23±2.06	-0.85±1.90	21	1.44
				2009	-1.35±1.81	0.53±1.12	28	1.07
				2010	-1.25±1.68	0.20±0.64	23	1
				2011	-0.83±1.46	-0.08±0.56	32	1.03
				2012	0.43±1.72	-0.36±0.61	35	1.1

IT-Tor	45.8444	7.5781	GRA	2008	-1.16±3.09	0.30±1.43	10	1.27
				2009	-0.21±2.30	-0.08±1.06	25	1.1
				2010	-0.44±2.21	0.05±0.82	26	1.04
				2011	-0.73±1.77	0.15±0.34	26	0.84
				2012	0.11±1.86	-0.10±0.28	29	0.7
				2013	0.14±1.96	-0.06±0.43	21	0.82
				2014	0.32±1.71	-0.04±0.28	28	0.71
JP-MBF	44.3869	142.3186	DBF	2004	-1.15±2.29	-0.37±0.69	20	1.28
				2005	-1.01±2.21	-0.16±0.58	30	1.17
JP-SMF	35.2617	137.0788	MF	2002	0.84±0.86	0.03±0.48	2	0.66
				2003	0.32±1.26	-0.13±0.40	8	0.95
				2004	0.62±0.68	-0.02±0.54	5	0.82
				2005	1.85±0.26	-0.88±0.46	4	1.04
				2006	0.46±1.49	-0.31±0.45	12	1.02
MY-PSO	2.973	102.3062	EBF	2003	-2.64±1.02	-0.11±0.94	19	0.44
				2004	-2.86±0.62	0.10±0.38	14	0.51
				2005	-2.63±0.59	-0.05±0.38	17	0.49
				2006	-2.94±0.57	0.12±0.39	16	0.43
				2007	-3.09±0.82	0.29±0.54	19	0.55
				2008	-2.48±0.55	-0.11±0.37	15	0.5
				2009	-2.48±0.74	-0.12±0.46	15	0.55
NL-Hor	52.2404	5.0713	GRA	2004	-0.32±1.41	0.03±0.47	14	0.95
				2005	-0.28±1.33	-0.31±0.62	27	1.06
				2006	0.63±0.33	-0.02±0.62	9	0.79
				2007	-0.69±1.66	0.19±0.78	30	1.05
				2008	-0.50±1.27	0.07±0.34	30	0.75
				2009	-0.64±1.77	-0.14±0.27	23	0.83
				2010	-0.42±1.53	-0.07±0.33	34	0.68

				2011	-1.16±1.71	0.01±0.26	21	0.83
NL-Loo	52.1666	5.7436	ENF	1999	-0.55±1.61	-0.19±0.93	25	1.12
				2000	-0.49±1.43	-0.20±0.68	34	1.09
				2001	-0.29±1.07	-0.12±0.66	31	1.05
				2002	-0.98±1.23	0.47±0.82	27	1.08
				2003	-1.17±1.31	0.57±0.60	31	1.14
				2004	-1.35±1.52	0.72±0.68	29	1.11
				2005	-1.32±1.46	0.59±0.72	31	1.11
				2006	-0.68±1.26	0.05±0.57	33	1.17
				2007	-0.62±1.20	-0.01±0.74	25	1
				2008	-1.22±1.20	0.60±0.57	28	1.06
				2009	-1.91±1.41	0.92±0.44	8	1.17
				2010	-1.31±1.32	0.71±0.66	29	1
				2011	-1.32±1.36	0.57±0.70	31	0.91
				2012	-1.46±1.53	0.30±0.35	36	0.81
				2013	-1.17±1.38	0.21±0.30	29	0.8
				2014	-1.51±1.40	0.08±0.34	28	0.89
NO-Adv	78.186	15.923	WET	2012	0.33±0.00	-0.30±0.00	1	0.41
				2013	-0.41±0.88	0.28±0.81	22	0.51
NO-Blv	78.9216	11.8311	SNO	2008	-0.02±0.12	-0.16±0.16	30	0.5
				2009	0.07±0.10	-0.19±0.12	7	0.45
PA-SPn	9.3181	-79.6346	DBF	2007	-0.88±0.46	0.15±0.37	6	0.76
				2008	-1.04±0.84	0.28±0.40	15	0.87
				2009	-0.68±1.12	0.04±0.55	12	1.06
PA-SPs	9.3138	-79.6314	GRA	2007	0.63±1.40	-0.29±0.75	18	0.93
				2008	0.89±1.31	-0.22±0.51	24	0.96
				2009	0.83±1.51	-0.16±0.62	17	0.98
RU-Che	68.613	161.3414	WET	2002	-0.44±1.22	0.03±0.18	12	0.48

				2003	-0.07±1.01	-0.07±0.17	19	0.47
				2004	-0.13±0.83	-0.08±0.19	18	0.47
				2005	0.28±0.39	0.01±0.19	6	0.3
RU-Cok	70.8291	147.4943	OSH	2003	-0.63±1.12	0.08±0.18	14	0.46
				2004	-1.38±0.89	0.41±0.29	7	0.72
				2005	-1.79±0.76	0.23±0.22	6	0.74
				2006	-0.86±0.49	0.13±0.38	4	0.57
				2007	-0.56±0.94	-0.12±0.17	7	0.62
				2008	-0.62±0.95	0.07±0.15	13	0.49
				2009	-0.61±1.09	-0.03±0.10	5	0.41
				2010	-0.57±1.24	-0.06±0.26	8	0.49
				2011	-1.24±1.18	-0.26±0.39	2	0.82
				2012	-0.91±1.32	0.18±0.22	7	0.57
				2013	-0.31±1.48	0.04±0.44	9	0.58
RU-Fyo	56.4615	32.9221	ENF	1999	1.00±1.26	-1.09±1.32	34	1
				2000	0.91±1.01	-1.01±1.07	35	1.04
				2001	1.03±1.25	-1.25±1.48	32	0.99
				2002	0.65±0.73	-0.64±0.78	26	0.74
				2003	0.54±1.11	-0.59±0.82	33	0.87
				2004	0.41±1.15	-0.85±1.12	33	1.02
				2005	0.24±1.01	-0.67±0.84	35	0.99
				2006	0.40±1.67	-0.63±1.24	34	1.07
				2007	0.08±1.28	-0.27±0.87	33	0.94
				2008	0.30±1.13	-0.60±1.05	32	1.01
				2009	0.18±1.27	-0.61±0.77	35	1.03
				2010	0.57±1.39	-0.85±1.56	24	1.17
				2011	0.17±1.10	-0.65±1.10	26	1.15
				2012	0.11±1.05	-0.18±0.36	35	0.75

				2013	0.30±1.05	-0.36±0.47	35	0.82
				2014	-0.21±1.36	-0.11±0.53	35	0.82
RU-Ha1	54.7252	90.0022	GRA	2002	-0.14±0.43	0.02±0.12	18	0.37
				2003	-0.48±0.58	-0.00±0.21	19	0.57
				2004	-0.53±0.87	0.07±0.18	23	0.48
SD-Dem	13.2829	30.4783	SAV	2005	-3.40±1.09	1.72±0.85	4	1.36
				2007	-0.78±1.08	-0.02±0.25	14	0.74
				2008	-0.30±0.87	-0.01±0.19	29	0.44
				2009	-0.23±0.73	-0.05±0.24	30	0.43
SN-Dhr	15.4028	-15.4322	SAV	2010	-3.76±2.10	0.44±1.11	6	1.45
				2011	-1.15±1.78	-0.25±0.74	3	1.21
				2012	-0.34±1.13	-0.04±0.33	26	0.63
				2013	-0.34±1.14	-0.10±0.33	6	0.71
US-AR1	36.4267	-99.42	GRA	2009	0.45±0.93	-0.41±0.78	24	1.15
				2010	-0.21±1.46	-0.10±0.55	36	0.78
				2011	0.52±0.84	-0.04±0.30	36	0.49
				2012	0.25±0.70	0.02±0.30	24	0.46
US-AR2	36.6358	-99.5975	GRA	2009	0.01±0.56	-0.12±0.28	22	0.53
				2010	-0.25±1.29	0.10±0.45	34	0.49
				2011	0.13±0.55	0.06±0.24	35	0.34
				2012	0.09±0.47	-0.07±0.20	17	0.42
US-ARb	35.5497	-98.0402	GRA	2005	-0.15±2.21	-0.12±0.54	21	0.91
				2006	0.35±1.54	-0.13±0.38	28	0.78
US-ARc	35.5465	-98.04	GRA	2005	-0.58±1.87	-0.10±0.36	29	0.94
				2006	0.17±1.62	-0.11±0.38	29	0.85
US-ARM	36.6058	-97.4888	CRO	2003	-0.04±1.94	0.05±0.90	34	1.19
				2004	-0.31±1.54	0.07±0.99	30	1.32
				2005	0.10±1.26	0.15±0.81	31	1.22

				2006	-0.20±1.45	0.41±1.01	31	1.14
				2007	-0.44±1.67	0.38±1.39	28	1.35
				2008	-0.97±2.35	-0.03±1.50	20	1.28
				2009	-0.14±1.67	0.16±0.51	26	0.87
				2010	-0.33±2.43	-0.03±0.87	26	1.03
				2011	-0.23±1.27	0.19±0.56	24	0.95
				2012	0.39±1.63	-0.02±0.36	21	0.73
US-Atq	70.4696	-157.409	WET	2003	0.10±0.58	-0.10±0.77	22	0.39
				2004	-0.22±0.72	0.08±0.35	27	0.45
				2005	-0.13±0.60	0.02±0.20	31	0.33
				2006	-0.06±0.57	0.01±0.17	32	0.3
				2007	-0.00±0.54	-0.05±0.15	32	0.28
				2008	-0.22±0.70	0.06±0.15	24	0.31
US-Blo	38.8953	-120.633	ENF	1999	-2.69±1.01	0.40±0.41	7	1.05
				2000	-0.29±0.00	0.02±0.00	1	0.79
				2001	0.47±0.60	-0.47±0.39	5	0.96
				2002	0.63±0.79	-0.47±0.43	3	0.8
				2003	0.05±0.87	-0.38±0.29	3	0.87
				2004	-0.07±0.50	-0.29±0.37	5	1
				2005	-1.18±1.22	-0.10±0.41	17	1.07
				2006	-1.30±1.68	0.26±0.40	2	0.82
US-Cop	38.09	-109.39	GRA	2001	-0.10±0.15	-0.10±0.20	24	0.34
				2002	-0.02±0.15	0.00±0.06	27	0.16
				2003	-0.09±0.33	0.01±0.15	27	0.21
				2006	-0.10±0.35	-0.02±0.10	21	0.19
				2007	-0.19±0.34	0.03±0.07	25	0.25
US-CRT	41.6285	-83.3471	CRO	2011	0.19±2.42	-0.09±0.61	35	1
				2012	0.00±2.73	-0.09±0.76	25	1.06

				2013	0.17±2.63	0.02±1.09	29	1.2
US-GBT	41.3658	-106.24	ENF	2001	-0.95±0.42	0.11±0.28	5	0.44
				2002	-1.34±1.01	0.17±0.41	26	0.7
				2003	-1.08±1.33	0.02±0.49	23	0.82
US-GLE	41.3665	-106.24	ENF	2005	-1.16±1.51	0.73±1.09	20	1.16
				2006	0.16±1.75	-0.32±1.50	21	0.85
				2007	1.01±1.17	-0.94±0.76	14	0.79
				2008	1.09±0.86	-0.95±0.89	22	0.7
				2009	1.05±0.96	-0.87±0.72	16	0.61
				2010	0.90±0.70	-0.93±0.55	28	0.72
				2011	0.37±0.98	-0.51±0.79	30	0.68
				2012	-0.03±0.57	-0.05±0.22	34	0.58
				2013	-0.00±0.61	-0.08±0.15	33	0.55
				2014	-0.01±0.85	-0.04±0.25	31	0.58
US-Goo	34.2547	-89.8735	GRA	2002	-0.05±1.25	-0.05±0.44	13	0.94
				2003	-1.12±0.61	0.10±0.44	13	0.83
				2004	-0.65±1.23	0.08±0.35	28	0.89
				2005	-0.10±0.71	0.03±0.22	22	0.71
				2006	0.07±1.01	-0.24±0.43	31	0.91
US-Ha1	42.5378	-72.1715	DBF	1999	0.66±1.75	-0.91±1.24	22	1.29
				2000	0.93±1.47	-0.68±0.99	19	1.04
				2001	0.10±2.72	-0.47±0.82	21	1.28
				2002	-0.29±3.24	-0.74±1.22	8	1.48
				2003	0.64±1.39	-0.37±0.55	13	1.08
				2004	-0.42±3.25	-0.28±1.10	25	1.38
				2005	0.48±1.74	-0.14±0.33	18	0.82
				2006	-0.06±3.07	-0.00±0.83	22	0.86
				2007	-0.55±2.80	-0.07±0.44	19	1.04

				2008	0.01±1.76	0.01±0.26	13	0.74
				2009	-0.13±3.79	-0.14±0.43	5	0.81
				2010	2.53±1.59	-1.07±1.24	6	1.33
				2011	-1.03±3.92	-0.04±1.21	10	1.37
				2012	1.26±0.72	-0.24±0.36	17	0.63
US-IB2	41.8406	-88.241	GRA	2004	1.13±0.57	-0.38±0.49	9	0.67
				2005	-0.68±2.01	0.19±0.81	30	0.96
				2006	-0.12±1.85	-0.26±1.07	29	0.9
				2007	-0.66±2.23	-0.03±0.87	29	1.04
				2008	-0.68±1.94	0.16±0.46	30	0.75
				2009	-0.68±2.23	0.01±0.34	33	0.7
				2010	0.06±1.16	-0.14±0.37	28	0.66
				2011	-0.76±1.88	-0.05±0.44	33	0.77
US-Ivo	68.4865	-155.75	WET	2004	0.14±0.64	-0.10±0.16	32	0.37
				2005	-0.06±0.77	-0.01±0.16	30	0.4
				2006	-0.04±0.74	-0.01±0.13	28	0.39
				2007	-0.42±0.98	0.03±0.30	22	0.5
US-KS1	28.4583	-80.6709	ENF	2002	-0.98±0.64	0.00±0.25	21	0.73
US-KS2	28.6086	-80.6715	CSH	2003	-0.86±1.02	0.14±0.84	24	1.03
				2004	-0.60±0.72	-0.07±0.31	36	0.76
				2005	-0.97±1.02	0.12±0.41	36	0.76
				2006	-0.90±0.67	-0.01±0.31	36	0.69
US-Lin	36.3566	-119.842	CRO	2009	-1.19±0.28	0.38±0.17	5	0.73
				2010	-0.84±0.34	0.02±0.30	21	1
US-Los	46.0827	-89.9792	WET	2000	0.96±0.65	-0.57±0.52	5	0.82
				2001	0.58±0.51	-0.31±0.26	24	0.7
				2002	0.17±0.72	-0.41±0.79	31	0.89
				2003	0.32±0.73	-0.35±0.50	26	0.81

				2004	0.04±1.05	-0.30±0.54	30	0.95
				2005	0.29±0.91	-0.21±0.34	31	0.66
				2006	0.27±0.86	-0.13±0.17	29	0.57
				2007	-0.13±1.84	-0.50±0.38	5	1.16
				2008	-0.62±1.55	-0.36±0.33	8	1.22
				2010	0.14±1.51	-0.70±0.49	7	1.33
				2014	-0.22±1.17	-0.07±0.25	31	0.61
US-Me1	44.5794	-121.5	ENF	2004	0.07±0.28	-0.04±0.12	16	0.37
				2005	-0.56±0.19	0.14±0.06	14	0.36
US-Me2	44.4523	-121.557	ENF	2002	-0.95±1.70	-0.66±0.96	24	1.46
				2003	-2.37±2.03	0.80±1.76	10	1.45
				2004	-2.03±1.08	0.36±0.59	25	1.41
				2005	-1.26±1.56	-0.20±1.08	33	1.49
				2006	-1.95±0.84	0.51±0.69	16	1.48
				2007	-2.18±1.66	0.38±1.07	28	1.32
				2008	-1.93±1.29	0.32±0.87	29	1.33
				2009	-2.50±1.26	0.93±1.01	33	1.19
				2010	-1.86±1.48	0.15±0.52	28	1.07
				2011	-3.36±1.64	0.56±0.48	18	1.15
				2012	-2.57±2.15	0.13±0.75	10	1.22
				2013	0.01±1.65	-0.52±0.54	22	1.1
				2014	0.37±1.44	-0.46±0.63	15	1.24
US-Me3	44.3154	-121.608	ENF	2004	-0.21±0.69	-0.23±0.51	32	0.84
				2005	-0.05±0.63	-0.39±0.36	25	0.85
				2006	-0.18±0.48	-0.10±0.21	26	0.53
				2007	-0.35±0.75	-0.09±0.27	27	0.57
				2008	-0.27±0.70	-0.05±0.27	35	0.58
				2009	-0.46±0.63	-0.02±0.29	26	0.53

US-Me4	44.4992	-121.622	ENF	1999	-1.12±1.39	-0.06±0.50	7	1.17
				2000	-3.15±1.59	0.44±0.84	17	1.25
US-Me5	44.4372	-121.567	ENF	2000	-0.91±0.84	0.04±0.23	27	0.73
				2001	-0.30±0.80	-0.12±0.23	33	0.66
				2002	-0.41±0.64	-0.12±0.21	32	0.69
US-Me6	44.3233	-121.608	ENF	2010	-0.99±1.39	-0.01±0.48	6	0.82
				2011	-1.03±0.79	0.07±0.21	22	0.55
				2012	-0.90±0.21	0.14±0.24	6	0.52
				2013	-0.38±0.91	-0.08±0.30	23	0.67
				2014	-0.55±1.17	-0.08±0.43	24	0.69
US-MMS	39.3232	-86.4131	DBF	1999	-0.76±2.76	-0.17±0.76	32	1.12
				2000	-0.59±3.21	0.16±1.04	25	1.06
				2001	0.04±2.38	-0.21±0.88	26	0.99
				2002	-0.27±2.46	-0.15±0.96	29	1.06
				2003	-0.24±2.33	-0.17±0.88	27	1.1
				2004	-1.31±3.26	0.25±0.99	31	1.12
				2005	-0.71±2.40	-0.15±0.76	31	1.12
				2006	-0.48±2.70	-0.13±0.98	30	1.13
				2007	-0.15±2.18	-0.29±1.42	26	1.2
				2008	-0.21±2.39	-0.28±1.08	29	1.12
				2009	-0.49±2.95	-0.14±1.29	32	1.13
				2010	-0.02±2.29	-0.33±1.10	28	1.28
				2011	-0.31±2.73	-0.04±0.82	29	0.99
				2012	-0.47±2.74	-0.15±0.45	29	0.91
				2013	-0.79±2.74	-0.16±0.47	34	0.78
				2014	-0.56±2.83	-0.09±0.40	33	0.79
US-Myb	38.0498	-121.765	WET	2011	-0.40±1.68	-0.24±0.89	19	1.37
				2012	-1.81±2.39	0.21±0.60	17	1.17

				2013	-0.19±1.69	-0.48±0.56	24	1.31
				2014	-1.23±2.78	0.21±0.81	15	1.34
US-Ne1	41.1651	-96.4766	CRO	2001	-0.66±3.75	0.94±1.63	11	1.44
				2002	0.48±2.54	0.57±2.02	23	0.88
				2003	0.57±2.08	0.23±1.34	26	1
				2004	0.01±3.09	0.33±0.95	23	1.07
				2005	0.77±1.84	0.25±0.97	19	1.05
				2006	0.77±3.12	-0.42±1.56	26	1.08
				2007	-0.15±4.23	0.25±1.86	27	1.27
				2008	0.62±2.57	0.22±1.31	25	0.88
				2009	-0.84±4.77	0.19±0.63	26	0.79
				2010	0.37±2.89	-0.13±0.33	20	0.54
				2011	1.90±1.66	-0.44±0.81	24	0.61
				2012	0.96±2.43	-0.11±0.26	20	0.55
				2013	1.03±0.61	-0.03±0.17	13	0.34
US-Ne2	41.1649	-96.4701	CRO	2001	-6.22±4.73	3.33±2.25	11	2.85
				2002	-0.12±2.43	0.29±1.71	18	1.72
				2003	-0.60±4.74	1.04±3.12	31	1.25
				2004	0.32±2.25	-0.00±0.98	32	1.2
				2005	-0.47±3.78	0.78±1.52	32	1.22
				2006	0.30±2.02	-0.46±1.22	34	1.43
				2007	0.08±2.84	0.19±1.22	30	1.25
				2008	0.50±1.57	-0.23±1.33	29	1.02
				2009	-0.09±3.14	0.58±0.99	28	0.79
				2010	-0.01±3.43	0.04±0.54	30	0.81
				2011	0.12±3.72	0.16±0.60	29	0.79
				2012	-0.53±4.26	0.49±0.93	30	0.92
				2013	1.03±0.56	0.01±0.27	15	0.49

US-Ne3	41.1797	-96.4397	CRO	2001	-5.49±5.23	4.48±4.10	13	2.15
				2002	0.67±1.47	-0.17±0.71	31	1.08
				2003	-0.74±4.49	1.01±3.32	29	1.21
				2004	0.54±1.77	0.01±0.64	29	0.95
				2005	-0.86±4.11	0.81±2.16	33	1.23
				2006	0.30±2.00	-0.55±1.14	32	1.29
				2007	-0.47±3.72	0.34±2.26	33	1.28
				2008	0.36±1.90	-0.47±1.31	32	1.1
				2009	-0.87±4.17	0.57±1.42	31	1.08
				2010	0.24±2.35	-0.30±0.72	33	0.95
				2011	-0.42±3.85	0.25±0.72	32	0.89
				2012	0.49±1.72	-0.05±0.49	26	0.8
				2013	0.93±0.69	-0.06±0.28	15	0.41
US-NR1	40.0329	-105.546	ENF	1999	-0.95±1.37	-0.10±0.62	19	1.12
				2000	-0.51±1.14	-0.19±0.40	33	0.95
				2001	-0.49±1.15	-0.09±0.45	31	0.99
				2002	-0.29±0.91	-0.30±0.45	32	1.03
				2003	-0.20±0.87	-0.39±0.46	30	0.99
				2004	-0.41±1.17	-0.42±0.57	30	1.15
				2005	-0.29±0.99	-0.47±0.39	33	1.08
				2006	-0.30±1.13	-0.37±0.44	29	0.97
				2007	-0.32±1.15	-0.25±0.47	30	0.9
				2008	-0.33±1.11	-0.21±0.53	32	0.93
				2009	-0.34±1.12	-0.19±0.48	28	0.95
				2010	-0.32±0.98	-0.22±0.40	32	0.94
				2011	-0.41±1.29	-0.09±0.53	32	0.79
				2012	-0.46±1.07	-0.14±0.27	33	0.69
				2013	-0.37±1.19	-0.08±0.28	33	0.64

				2014	-0.29±1.25	-0.08±0.28	28	0.62
US-Oho	41.5545	-83.8438	DBF	2004	-2.75±3.91	1.13±1.47	29	1.27
				2005	-1.60±2.75	0.58±0.98	25	1.2
				2006	-2.03±3.47	0.21±1.01	34	1.2
				2007	-1.77±3.73	0.55±1.45	23	1.25
				2008	-2.22±3.31	0.37±1.14	23	1.32
				2009	-1.93±3.88	0.22±1.45	30	1.16
				2010	-0.52±2.41	-0.09±0.52	26	0.85
				2011	-1.70±3.20	0.25±0.46	30	0.78
				2012	-1.63±3.63	0.03±0.48	31	0.84
				2013	-1.40±3.11	0.12±0.54	27	0.79
US-ORv	40.0201	-83.0183	WET	2011	0.54±0.38	-0.09±0.24	16	0.62
US-PFa	45.9459	-90.2723	MF	1999	0.38±0.57	-0.78±1.07	31	1.09
				2000	0.26±0.67	-0.61±0.65	33	1.06
				2001	0.47±0.59	-0.58±0.60	29	0.92
				2002	0.41±0.43	-0.53±0.73	27	0.8
				2003	0.31±0.93	-0.84±0.95	27	1.1
				2004	0.31±0.65	-0.61±0.71	31	0.93
				2005	0.05±0.56	-0.30±0.57	20	1.01
				2006	0.08±0.32	-0.16±0.67	25	0.82
				2007	-0.30±0.98	-0.11±0.62	31	1.06
				2008	-0.13±0.92	-0.11±0.59	31	0.88
				2009	-0.13±0.73	-0.04±0.69	30	0.91
				2010	0.22±0.20	-0.14±0.43	22	0.76
				2011	-0.02±0.83	-0.16±0.31	30	0.68
				2012	0.11±0.36	-0.13±0.30	28	0.68
				2013	0.09±0.80	-0.14±0.29	27	0.58
				2014	0.12±0.74	-0.22±0.46	31	0.64

US-Prr	65.1237	-147.488	ENF	2010	0.23±0.18	0.03±0.07	8	0.17
				2011	-0.18±0.71	0.03±0.09	18	0.32
				2012	-0.31±0.71	0.08±0.12	12	0.41
				2013	0.22±0.18	0.01±0.06	19	0.26
				2014	-0.38±0.78	0.06±0.20	24	0.6
US-SRC	31.9083	-110.84	OSH	2008	0.02±0.28	-0.18±0.21	20	0.35
				2009	-0.03±0.23	-0.08±0.17	33	0.25
				2010	-0.36±0.37	0.18±0.27	33	0.34
				2011	0.05±0.33	-0.04±0.10	34	0.2
				2012	-0.13±0.32	0.04±0.11	28	0.21
				2013	-0.24±0.18	0.01±0.09	20	0.18
				2014	-0.40±0.24	0.06±0.07	15	0.2
US-SRG	31.7894	-110.828	GRA	2008	-0.21±1.21	-0.25±0.75	28	0.74
				2009	0.17±0.50	0.05±0.40	35	0.47
				2010	-0.23±0.88	-0.07±0.73	36	0.67
				2011	0.26±0.59	-0.24±0.54	36	0.46
				2012	-0.27±0.95	0.08±0.33	36	0.4
				2013	0.04±0.64	0.01±0.21	36	0.33
				2014	-0.02±0.71	-0.11±0.29	36	0.43
US-SRM	31.8214	-110.866	WSA	2004	0.15±0.39	0.01±0.23	33	0.35
				2005	0.05±0.54	0.01±0.38	34	0.42
				2006	0.42±0.66	-0.38±0.42	35	0.41
				2007	0.27±0.50	-0.28±0.32	30	0.4
				2008	-0.05±0.71	-0.00±0.31	31	0.42
				2009	0.25±0.34	-0.00±0.29	36	0.35
				2010	-0.14±0.54	0.04±0.40	36	0.46
				2011	-0.06±0.75	-0.02±0.32	35	0.38
				2012	-0.10±0.52	0.05±0.18	34	0.28

				2013	0.05±0.45	-0.00±0.15	29	0.27
				2014	0.07±0.41	-0.05±0.15	28	0.26
US-Sta	41.3966	-106.802	OSH	2005	-0.44±0.13	0.05±0.05	5	0.2
				2006	-0.24±0.12	-0.00±0.20	2	0.23
				2007	-0.87±0.72	0.05±0.23	26	0.37
				2008	-0.87±0.67	0.00±0.18	33	0.37
				2009	-1.03±1.04	0.09±0.32	32	0.36
US-Syv	46.242	-89.3477	MF	2001	0.47±0.55	-0.20±0.96	11	0.98
				2002	0.04±0.82	-0.26±0.83	28	1.02
				2003	-0.41±1.18	-0.24±0.63	23	1.13
				2004	-0.01±1.60	-0.74±0.74	28	1.28
				2005	0.18±1.34	-0.56±0.50	21	1.09
				2006	0.39±0.82	-0.35±0.39	31	0.89
				2007	-1.32±1.49	-0.25±0.50	4	1.58
				2012	-0.20±2.06	-0.18±0.53	16	1.02
				2013	0.11±1.74	-0.34±0.71	19	0.92
				2014	-0.42±1.71	-0.06±0.54	33	0.84
US-Ton	38.4316	-120.966	WSA	2001	0.09±0.92	-0.09±0.67	18	0.82
				2002	-0.49±1.40	0.32±1.03	30	0.87
				2003	-0.39±1.17	0.04±0.72	26	1
				2004	-0.46±1.25	0.04±0.94	22	0.91
				2005	-0.53±1.19	-0.01±1.18	18	1.01
				2006	-0.40±0.98	0.11±0.68	27	0.89
				2007	-0.14±1.29	-0.29±0.69	26	0.94
				2008	-0.32±1.31	0.16±0.92	29	0.92
				2009	-0.29±1.11	-0.06±0.73	32	0.92
				2010	-0.68±1.25	0.23±0.97	30	1.02
				2011	-0.50±0.87	0.07±0.29	23	0.69

				2012	-0.38±1.17	-0.01±0.45	29	0.69
				2013	-0.06±1.02	-0.10±0.37	25	0.52
				2014	-0.10±1.38	-0.05±0.34	27	0.63
US-Tw1	38.1074	-121.647	WET	2012	-5.48±2.87	1.54±0.24	6	1.93
				2013	-2.57±3.35	0.93±1.21	19	1.93
				2014	-2.69±2.74	1.29±1.47	16	1.86
US-Tw2	38.1047	-121.643	CRO	2012	0.76±2.62	-0.84±0.58	22	1.6
				2013	0.65±1.10	0.32±0.61	9	1.1
US-Tw3	38.1159	-121.647	CRO	2013	-1.56±3.74	-0.07±1.56	14	2.1
				2014	-2.07±2.98	0.08±1.16	20	2.02
US-Tw4	38.103	-121.641	WET	2013	1.58±0.12	-0.03±0.00	2	0.3
				2014	0.48±1.19	-0.97±1.51	23	1.61
US-Twt	38.1087	-121.653	CRO	2009	-2.14±3.79	0.99±3.54	21	3.07
				2010	-1.18±5.31	-0.15±3.49	27	2.62
				2011	0.31±4.10	-0.39±0.97	30	1.7
				2012	-0.22±4.06	-0.07±0.98	29	1.62
				2013	-0.16±5.21	0.04±1.98	25	2.19
				2014	0.56±5.56	-1.04±1.84	22	2.41
US-UMB	45.5598	-84.7138	DBF	2000	-0.18±1.79	-0.34±0.79	27	0.84
				2001	0.24±1.08	-0.43±1.08	27	0.78
				2002	-0.00±1.56	-0.37±0.84	28	0.69
				2003	0.34±1.37	-0.19±0.66	25	0.53
				2004	0.51±1.60	-0.54±0.81	24	0.59
				2005	-0.21±2.34	-0.23±0.61	17	0.72
				2006	0.07±1.99	0.00±0.51	19	0.61
				2007	0.14±1.85	-0.38±0.59	25	0.62
				2008	-0.36±2.35	-0.20±0.80	30	0.77
				2009	0.68±0.77	-0.14±0.45	21	0.49

				2010	0.33±1.44	-0.06±0.32	26	0.63
				2011	0.39±1.54	0.05±0.21	25	0.36
				2012	-0.33±2.13	-0.08±0.19	26	0.51
				2013	-0.08±2.00	-0.01±0.35	26	0.44
				2014	0.04±1.94	-0.09±0.26	25	0.48
US-UMd	45.5625	-84.6975	DBF	2007	-1.15±2.39	-0.31±0.84	17	1.01
				2008	-0.89±2.53	0.18±0.68	34	0.87
				2009	-0.72±2.38	0.13±0.71	34	0.86
				2010	-0.43±2.08	0.04±0.56	32	0.89
				2011	0.04±1.55	0.03±0.31	29	0.53
				2012	-0.89±2.53	0.05±0.32	33	0.58
				2013	-0.25±2.04	-0.17±0.50	31	0.6
				2014	-0.84±2.69	0.01±0.54	27	0.83
US-Var	38.4133	-120.951	GRA	2000	-0.51±0.66	1.01±0.55	6	0.74
				2001	-0.28±1.44	0.27±0.68	34	1.18
				2002	0.00±1.40	0.07±0.80	36	1.06
				2003	0.06±1.53	-0.14±0.90	34	1.24
				2004	0.08±1.34	0.07±0.83	34	0.94
				2005	0.02±1.97	-0.25±0.78	27	1.31
				2006	0.21±0.94	-0.16±0.61	33	1.08
				2007	-0.20±1.49	0.20±0.61	33	1.12
				2008	0.28±1.01	0.04±0.62	31	1.01
				2009	0.04±1.73	-0.09±0.85	35	1.13
				2010	0.23±1.21	-0.19±0.56	30	1.21
				2011	0.48±1.34	-0.52±0.91	35	1.02
				2012	0.51±1.33	-0.19±0.41	32	0.78
				2013	0.03±0.95	0.04±0.22	36	0.67
				2014	0.60±0.75	-0.02±0.20	26	0.61

US-WCr	45.8059	-90.0799	DBF	1999	0.20±2.17	-0.52±0.96	27	0.96
				2000	-0.40±2.41	-0.47±0.89	32	1.14
				2001	0.56±1.75	-0.79±1.53	27	0.97
				2002	0.18±1.50	-0.29±0.43	23	0.75
				2003	-0.35±2.86	-0.40±1.10	30	0.98
				2004	0.67±1.86	-0.64±0.60	21	0.78
				2005	-0.29±3.19	-0.19±1.29	31	0.97
				2006	-0.62±2.67	-0.09±0.75	26	0.98
				2010	-0.13±0.26	0.79±0.21	4	0.42
				2011	-0.62±1.65	0.27±0.29	26	0.63
				2012	-0.96±2.36	-0.10±0.45	31	0.87
				2013	-1.22±3.43	-0.02±0.87	32	0.96
				2014	0.08±2.73	-0.19±0.40	20	0.68
US-Whs	31.7438	-110.052	OSH	2007	0.10±0.33	-0.05±0.20	18	0.36
				2008	-0.11±0.49	0.16±0.24	36	0.29
				2009	0.13±0.18	0.06±0.17	36	0.24
				2010	-0.07±0.34	0.10±0.21	36	0.34
				2011	0.10±0.31	0.03±0.19	36	0.25
				2012	0.14±0.25	0.00±0.07	36	0.15
				2013	0.11±0.30	-0.01±0.08	36	0.18
				2014	-0.07±0.40	0.06±0.07	35	0.2
US-Wi0	46.6188	-91.0814	ENF	2002	-2.08±1.73	-0.07±0.54	20	1.17
US-Wi1	46.7305	-91.2329	DBF	2003	1.04±1.92	-1.12±0.82	5	1.85
US-Wi2	46.6869	-91.1528	ENF	2003	-2.33±1.66	1.21±0.94	2	1.3
US-Wi3	46.6347	-91.0987	DBF	2002	-4.53±2.87	0.82±0.40	17	1.44
				2004	-3.15±3.34	0.39±0.53	21	1.14
US-Wi4	46.7393	-91.1663	ENF	2002	-4.18±1.25	0.34±0.35	17	1.2
				2003	-5.11±1.27	0.01±0.55	4	1.03

				2004	-4.20±1.85	0.42±0.40	20	1.21
				2005	-4.23±1.52	0.25±0.53	17	1.16
US-Wi5	46.6531	-91.0858	ENF	2004	-1.92±1.96	-0.07±0.44	22	1.21
US-Wi6	46.6249	-91.2982	OSH	2002	-1.03±1.85	0.08±0.33	17	0.96
US-Wi7	46.6491	-91.0693	OSH	2005	-0.82±1.16	-0.51±0.49	13	1.41
US-Wi8	46.7223	-91.2524	DBF	2002	0.56±0.88	-0.91±0.64	18	1.68
US-Wi9	46.6188	-91.0814	ENF	2004	-0.47±0.74	0.01±0.30	7	0.8
				2005	-1.73±0.82	-0.26±0.31	6	1.44
US-Wkg	31.7365	-109.942	GRA	2004	0.03±0.42	0.23±0.33	21	0.37
				2005	0.17±0.28	0.11±0.18	33	0.3
				2006	-0.02±0.58	0.12±0.30	34	0.4
				2007	0.04±0.25	0.11±0.23	33	0.33
				2008	0.13±0.22	0.08±0.24	29	0.23
				2009	-0.00±0.37	0.29±0.32	36	0.31
				2010	-0.08±0.54	0.21±0.28	29	0.36
				2011	0.30±0.30	-0.08±0.15	31	0.21
				2012	0.12±0.43	0.04±0.13	33	0.18
				2013	0.28±0.32	-0.05±0.11	34	0.19
				2014	0.05±0.50	0.02±0.09	29	0.21
US-WPT	41.4646	-82.9962	WET	2011	0.38±1.19	0.08±0.35	27	0.61
				2012	0.81±0.78	-0.18±0.37	28	0.62
				2013	0.87±0.92	-0.20±0.34	31	0.62
ZM-Mon	-15.4378	23.2528	DBF	2000	-0.15±0.00	-0.17±0.00	1	0.81
				2007	0.05±1.82	-0.23±0.82	8	1.05
				2008	0.32±0.97	-0.19±0.28	21	0.8
				2009	-0.31±0.19	-0.06±0.33	6	0.89
