# Supplementary material for: Global terrestrial carbon fluxes of 1999–2019 estimated by upscaling eddy covariance data with a random forest
Source: Sci Data. 2020 Sep 24;7:313. doi: 10.1038/s41597-020-00653-5 (PMC7518252; doi:10.1038/s41597-020-00653-5)
Supplement: Supplementary file 1 — Supplementary information [file 41597_2020_653_MOESM1_ESM.zip › scidata/Supplementary File 5 (2).pdf]

NEE Time Series

This document contains plots of the time series variations of observed NEE and model predictions. Dark dots are observations. Horizontal blue bars are the means (predictions) and vertical blue lines are the standard deviations of flux values in the terminal nodes of 500 trees.

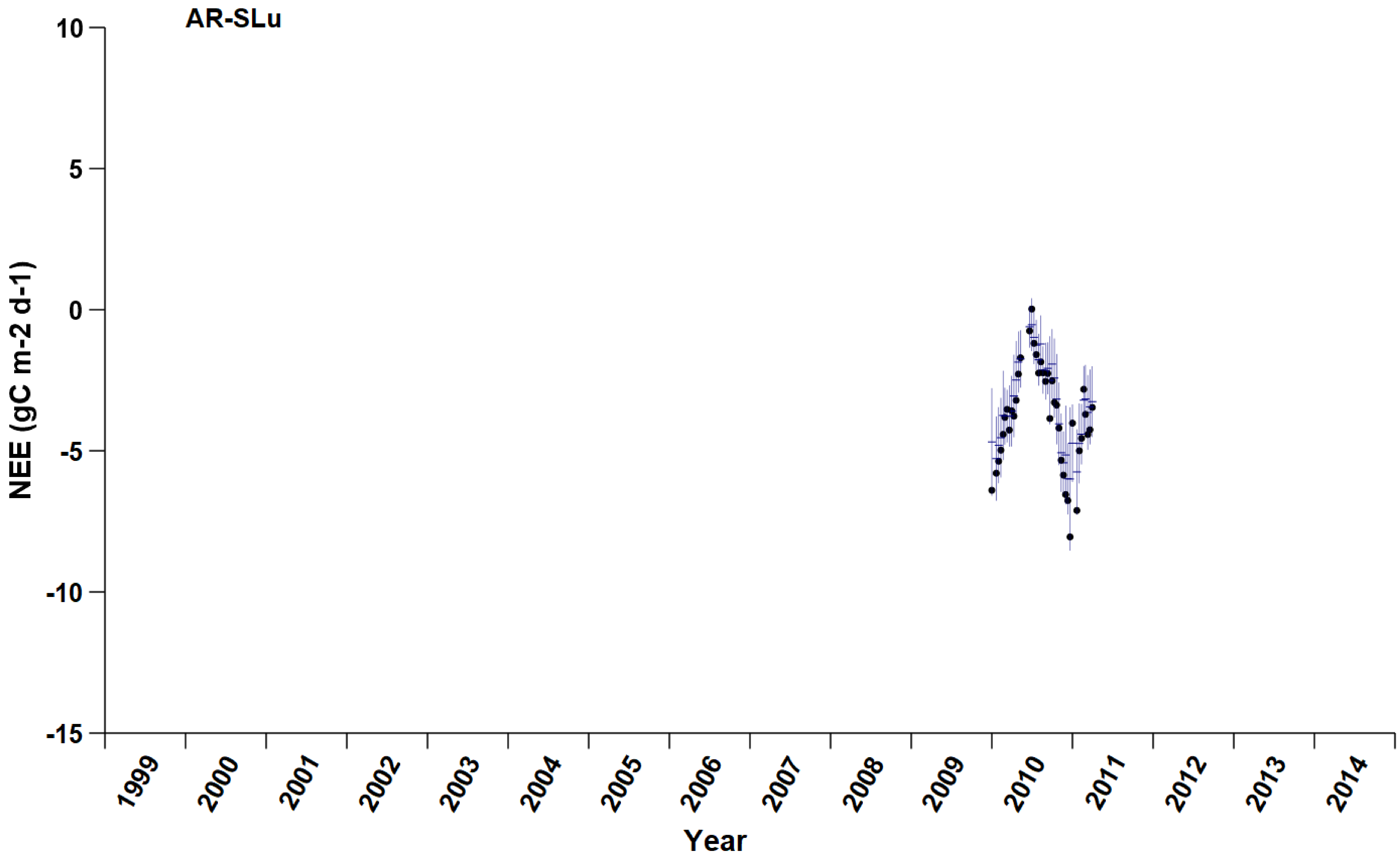
AR-SLu
NEE (gC m-2 d-1)
10
5
0
-5
-10
-15
1999
2000
2001
2002
2003
2004
2005
2006
2007
2008
2009
2010
2011
2012
2013
2014
Year

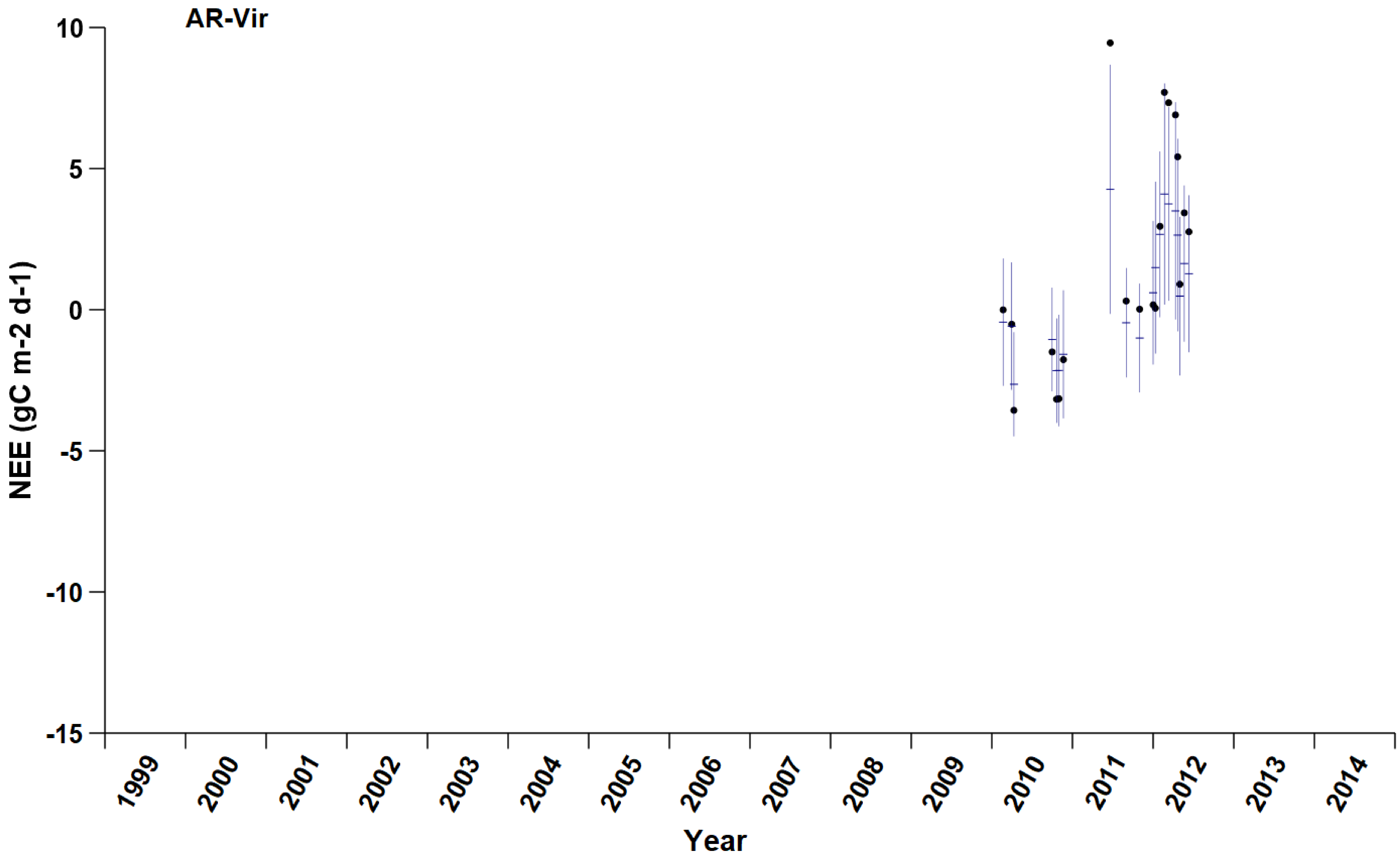
AR-Vir
NEE (gC m-2 d-1)
10
5
0
-5
-10
-15
1999
2000
2001
2002
2003
2004
2005
2006
2007
2008
2009
2010
2011
2012
2013
2014
Year

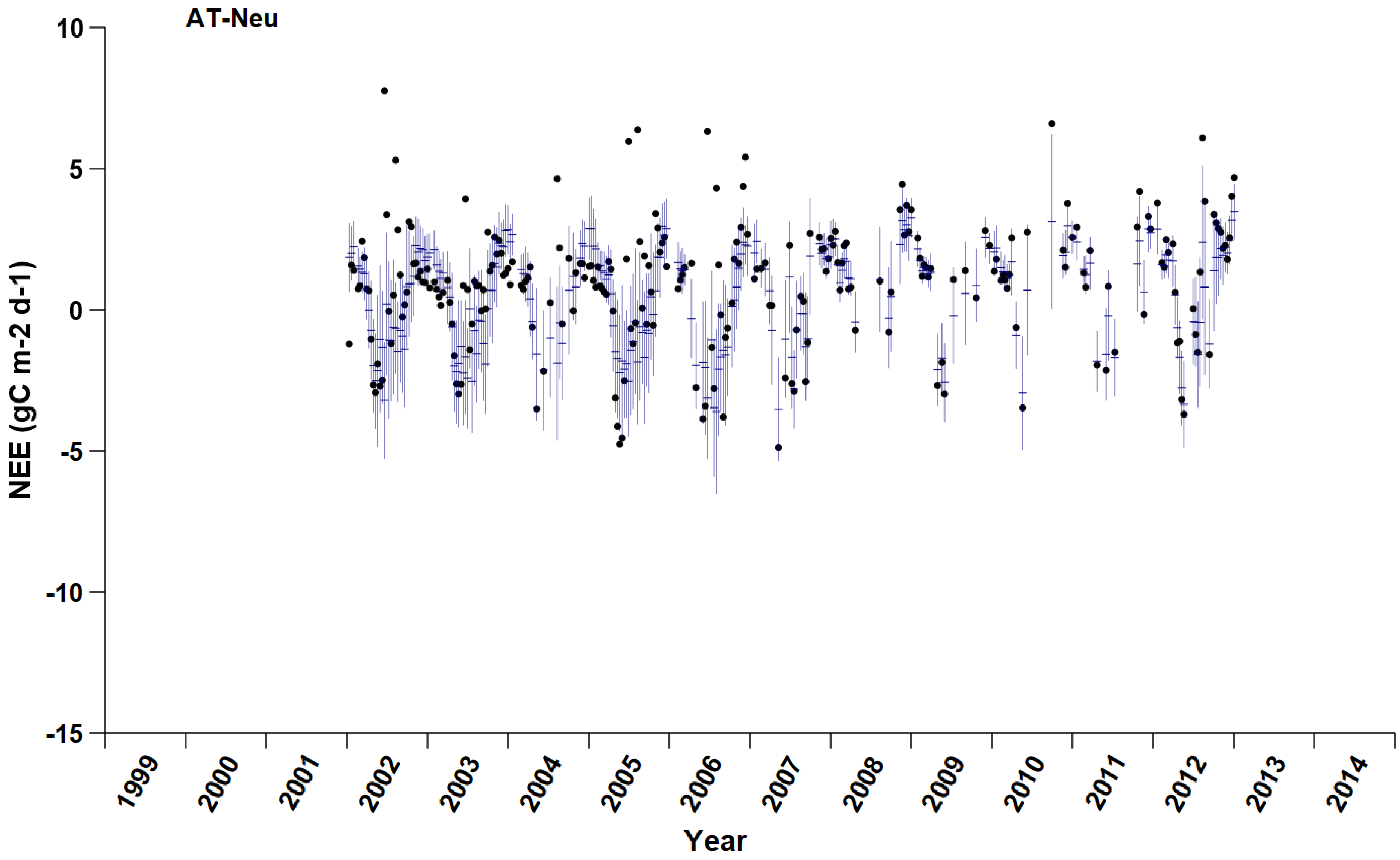
AT-Neu
NEE (gC m-2 d-1)
10
5
0
-5
-10
-15
1999
2000
2001
2002
2003
2004
2005
2006
2007
2008
2009
2010
2011
2012
2013
2014
Year

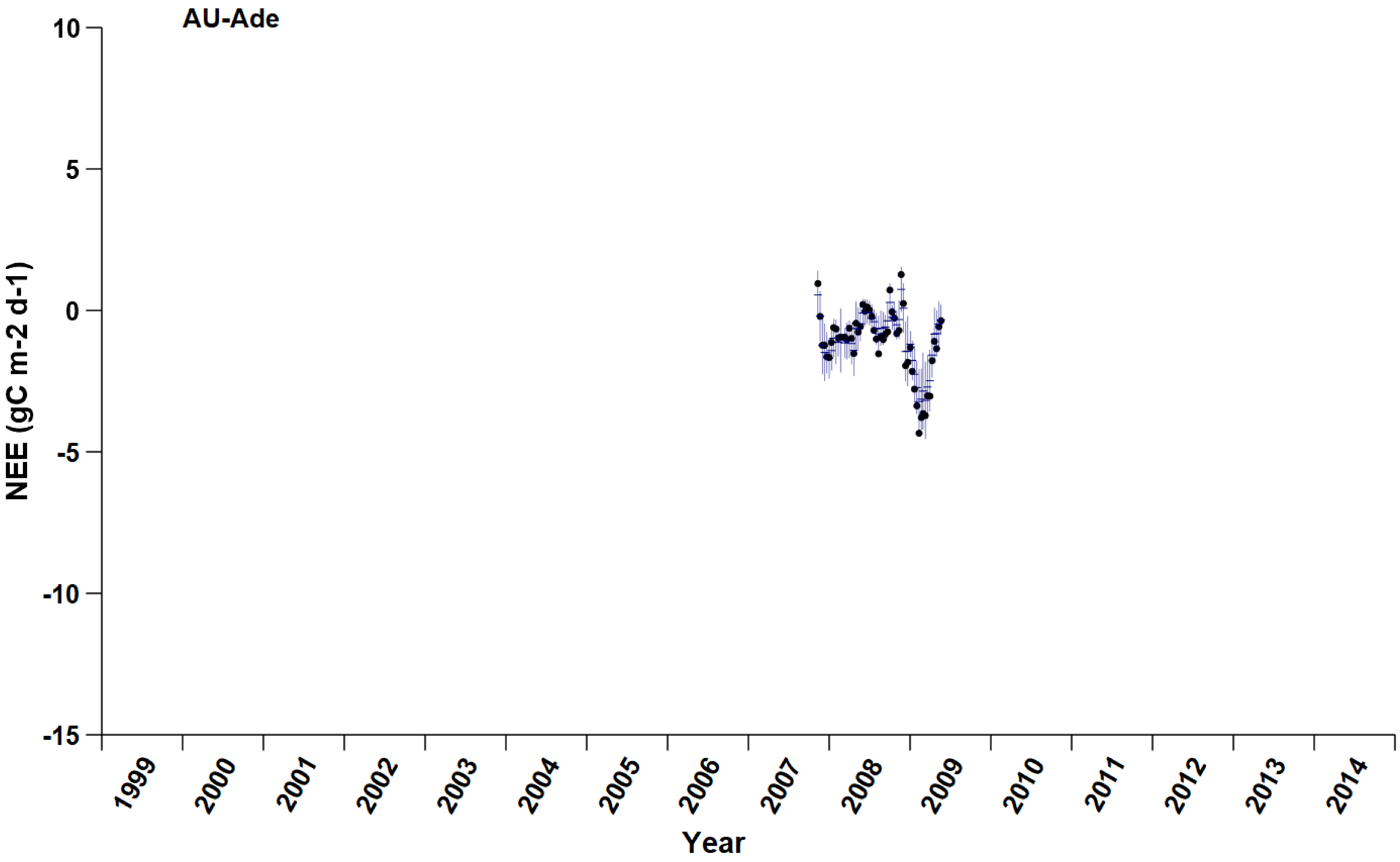
AU-Ade
10
5
0
-5
-10
-15
NEE (gC m-2 d-1)
1999
2000
2001
2002
2003
2004
2005
2006
2007
2008
2009
2010
2011
2012
2013
2014
Year

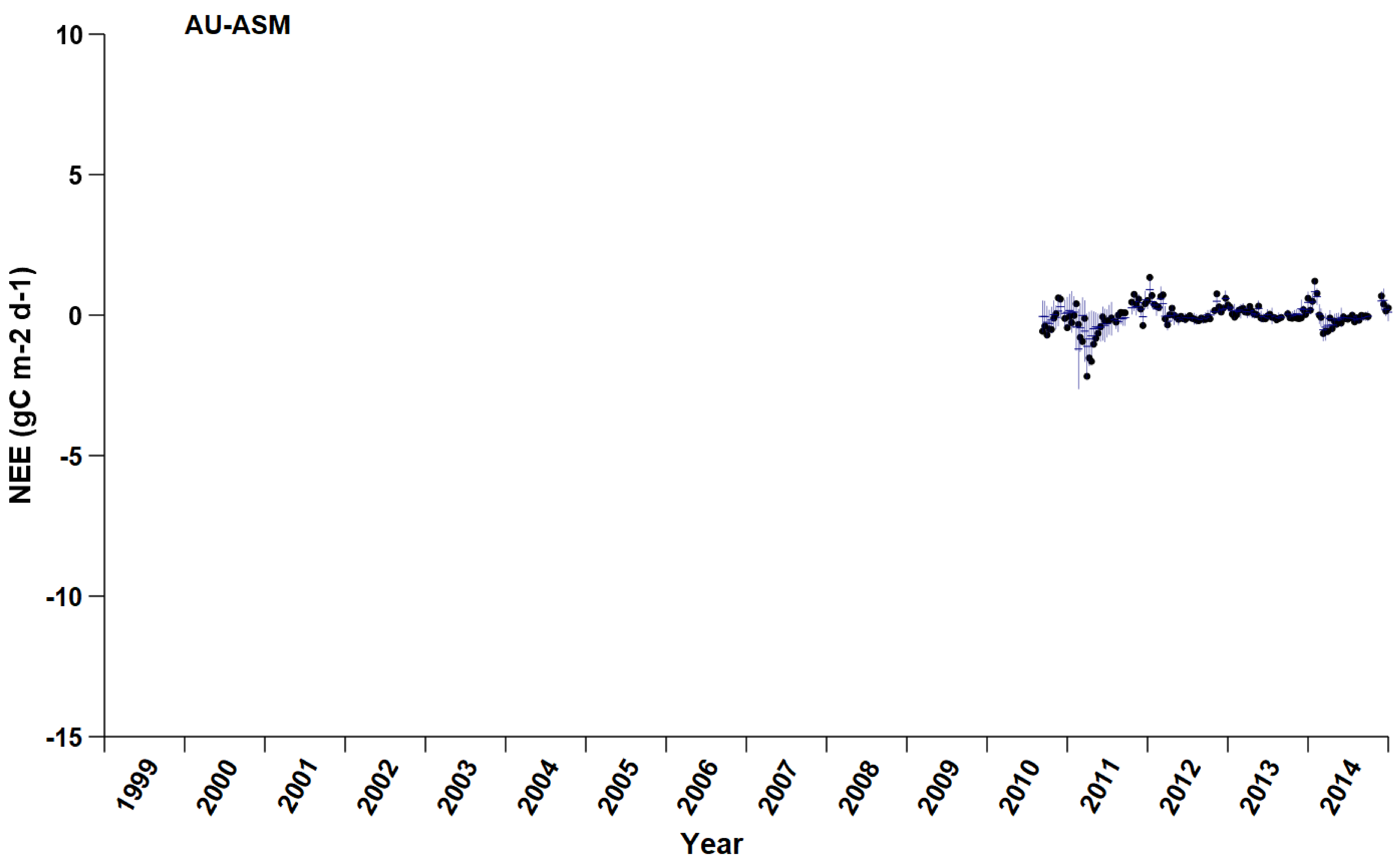

AU-ASM
NEE (gC m-2 d-1)
10
5
0
-5
-10
-15
1999
2000
2001
2002
2003
2004
2005
2006
2007
2008
2009
2010
2011
2012
2013
2014
Year

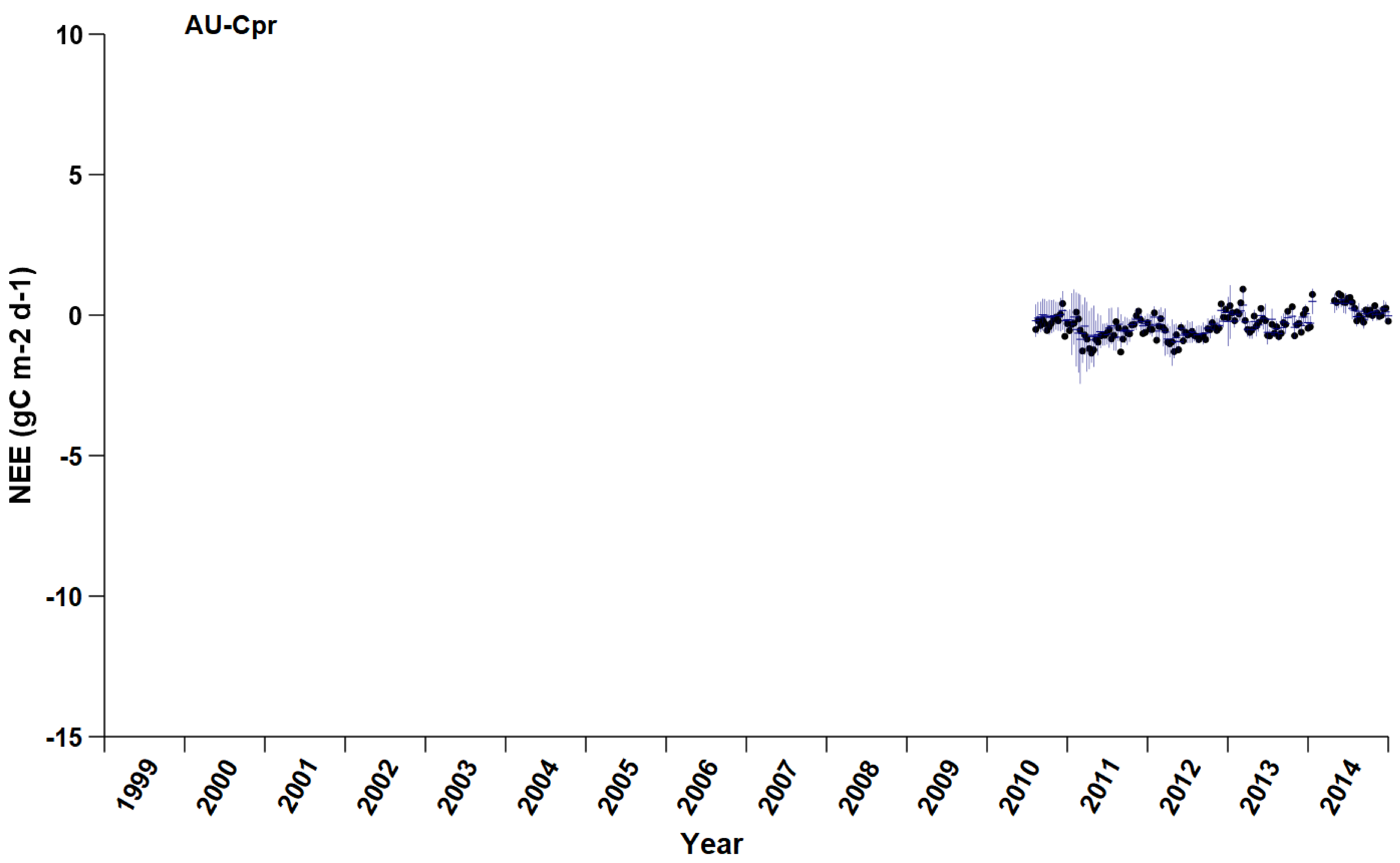

AU-Cpr
NEE (gC m-2 d-1)
10
5
0
-5
-10
-15
1999
2000
2001
2002
2003
2004
2005
2006
2007
2008
2009
2010
2011
2012
2013
2014
Year

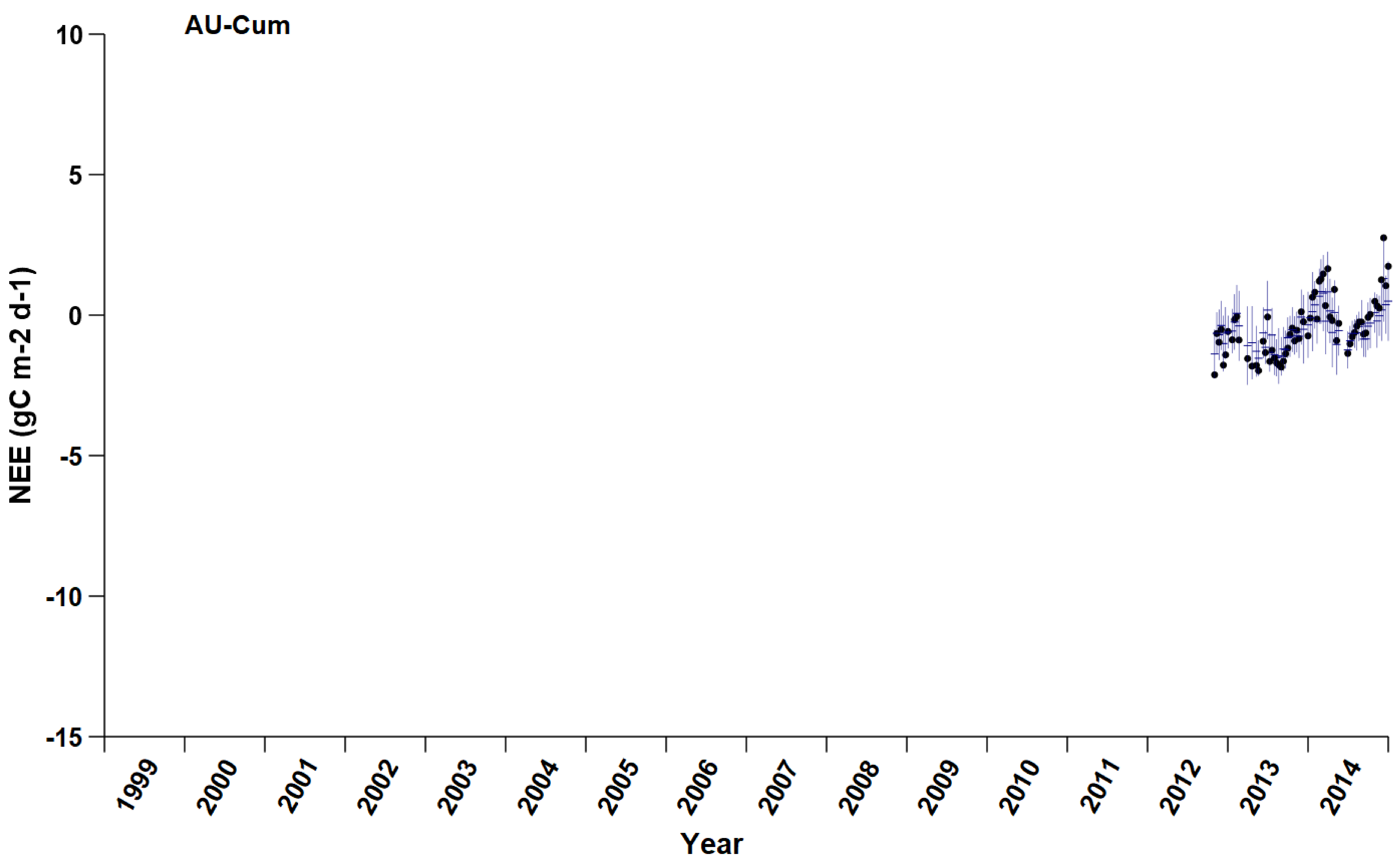
AU-Cum
NEE (gC m-2 d-1)
10
5
0
-5
-10
-15
1999
2000
2001
2002
2003
2004
2005
2006
2007
2008
2009
2010
2011
2012
2013
2014
Year

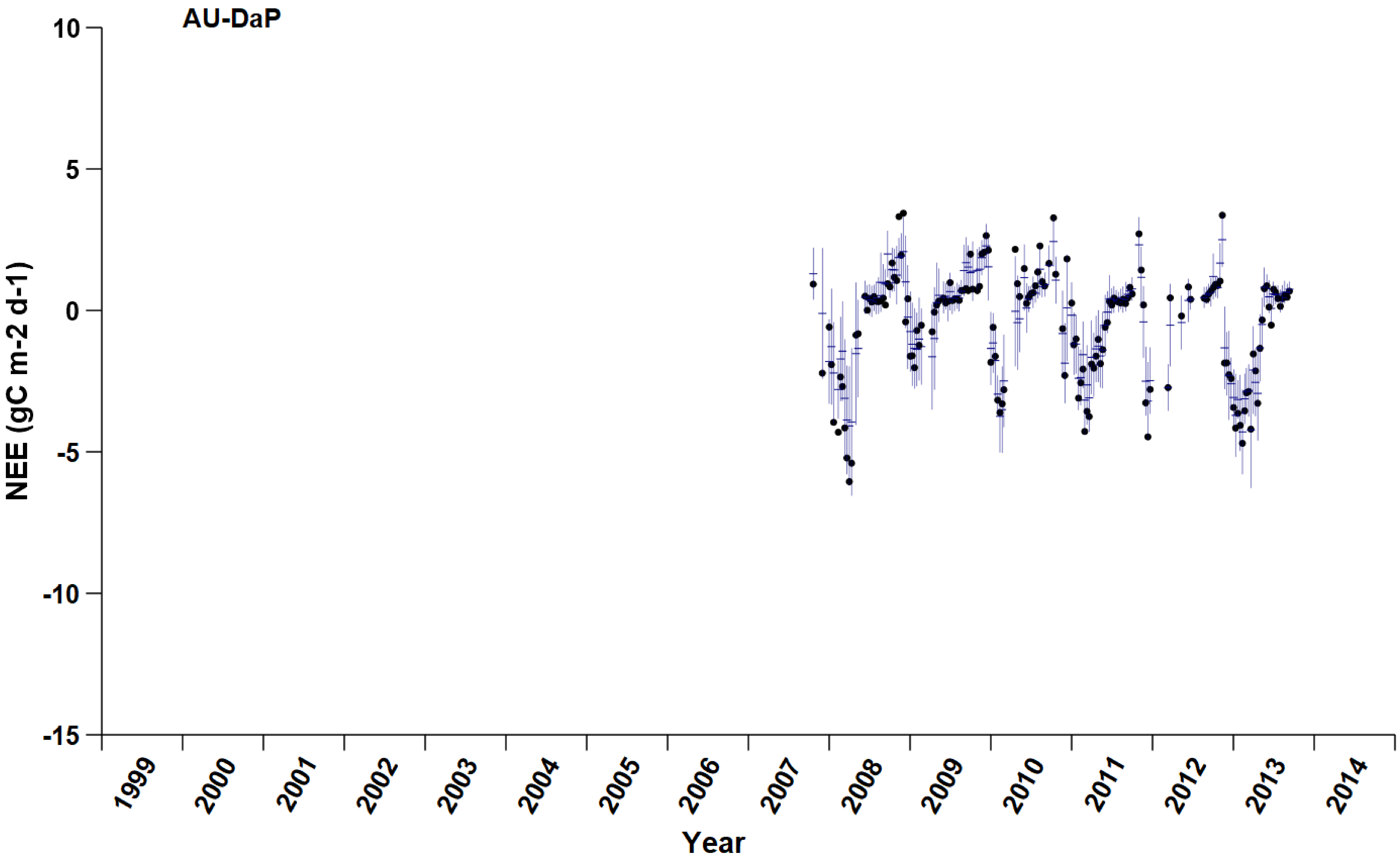
AU-DaP
NEE (gC m-2 d-1)
10
5
0
-5
-10
-15
1999
2000
2001
2002
2003
2004
2005
2006
2007
2008
2009
2010
2011
2012
2013
2014
Year

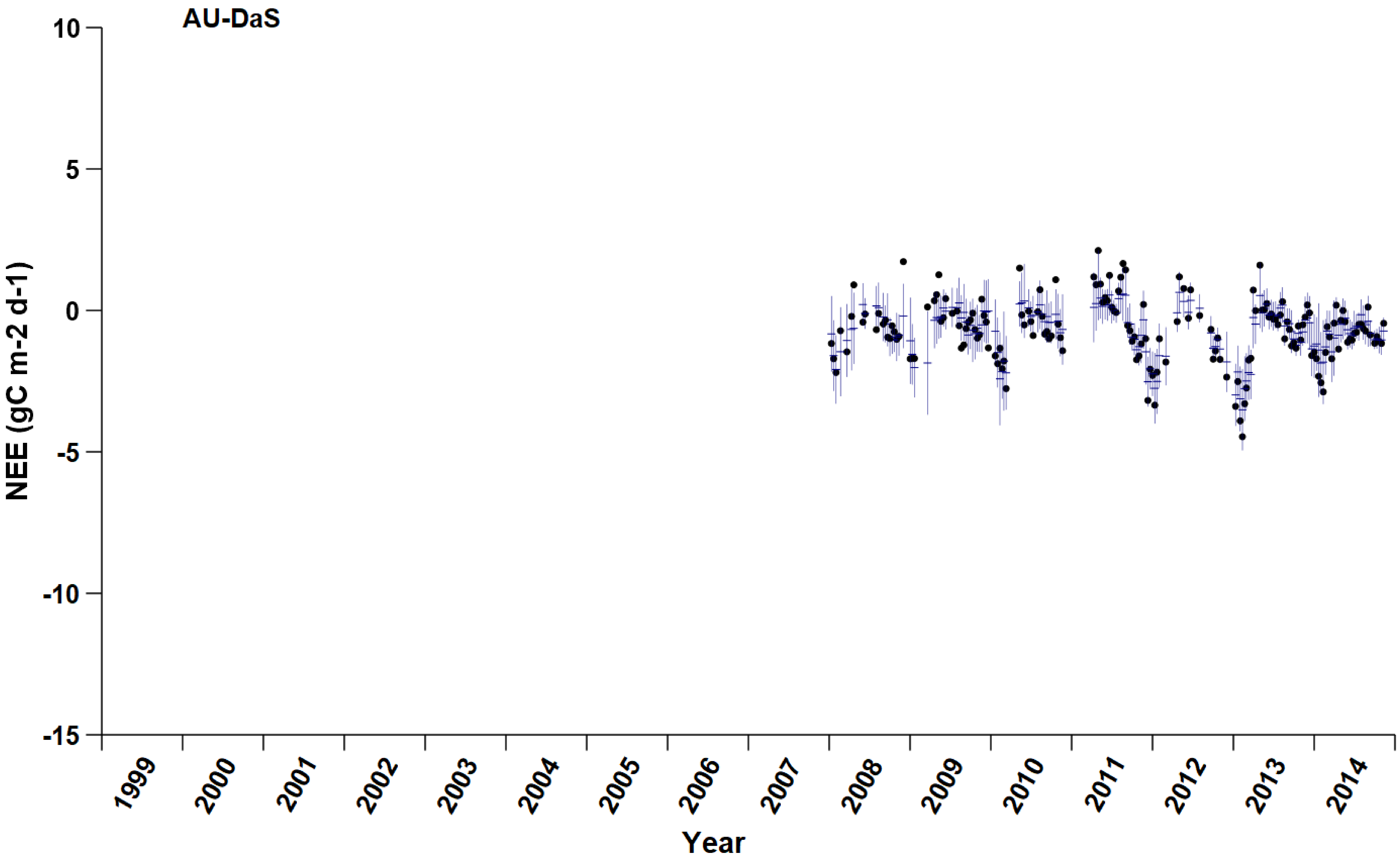
AU-DaS
NEE (gC m-2 d-1)
10
5
0
-5
-10
-15
1999
2000
2001
2002
2003
2004
2005
2006
2007
2008
2009
2010
2011
2012
2013
2014
Year

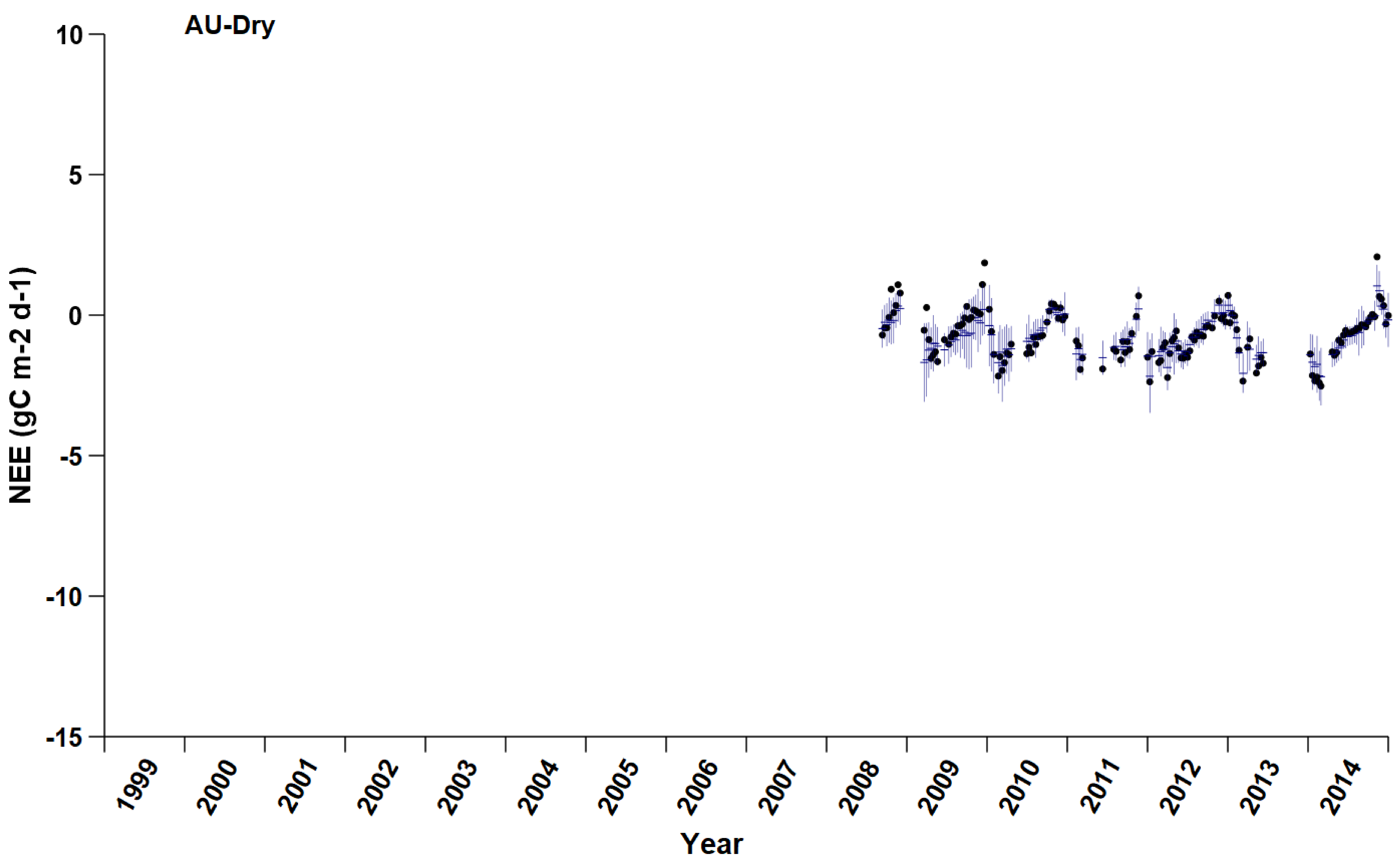

AU-Dry
NEE (gC m-2 d-1)
10
5
0
-5
-10
-15
1999
2000
2001
2002
2003
2004
2005
2006
2007
2008
2009
2010
2011
2012
2013
2014
Year

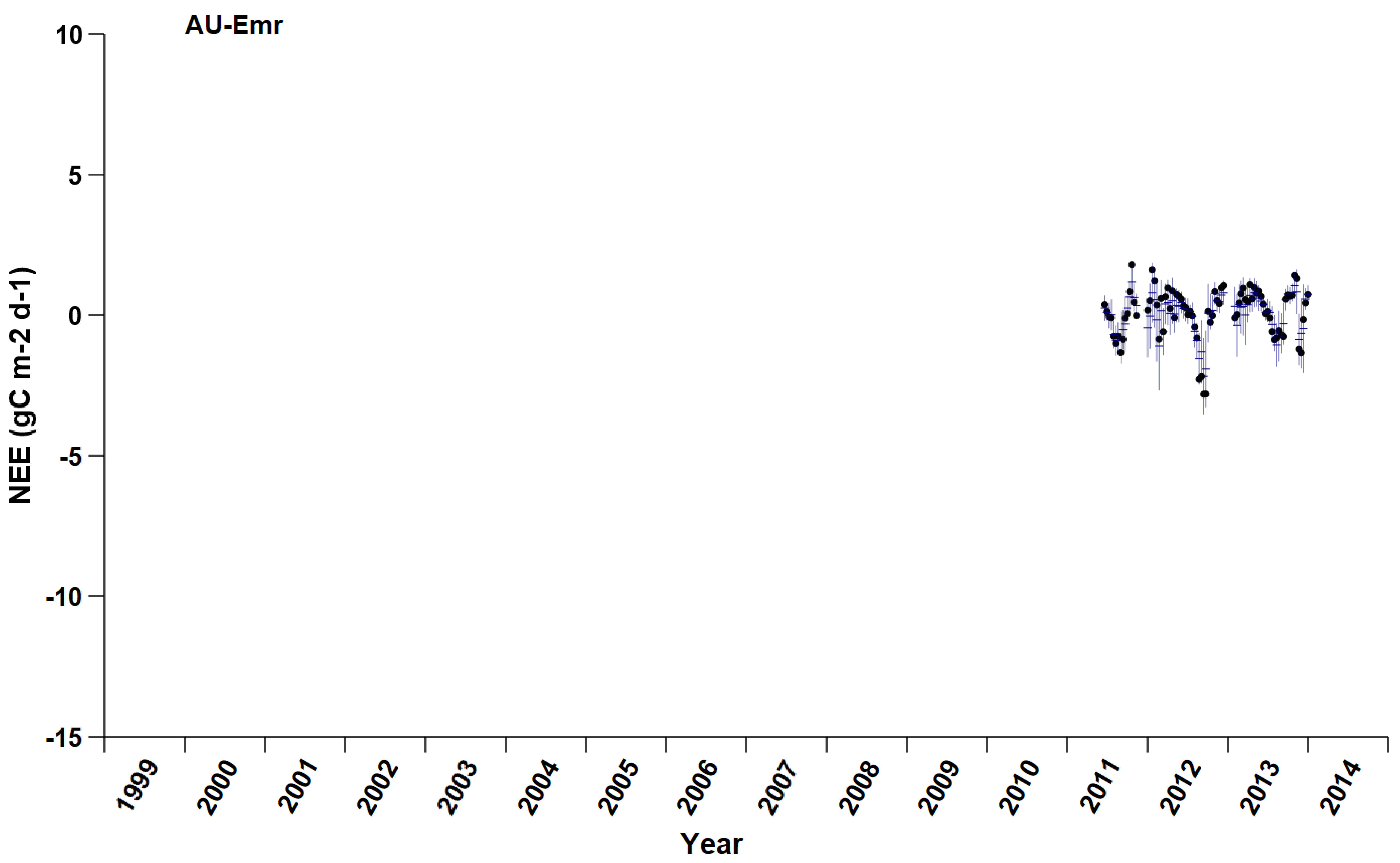

AU-Emr
NEE (gC m-2 d-1)
10
5
0
-5
-10
-15
1999
2000
2001
2002
2003
2004
2005
2006
2007
2008
2009
2010
2011
2012
2013
2014
Year

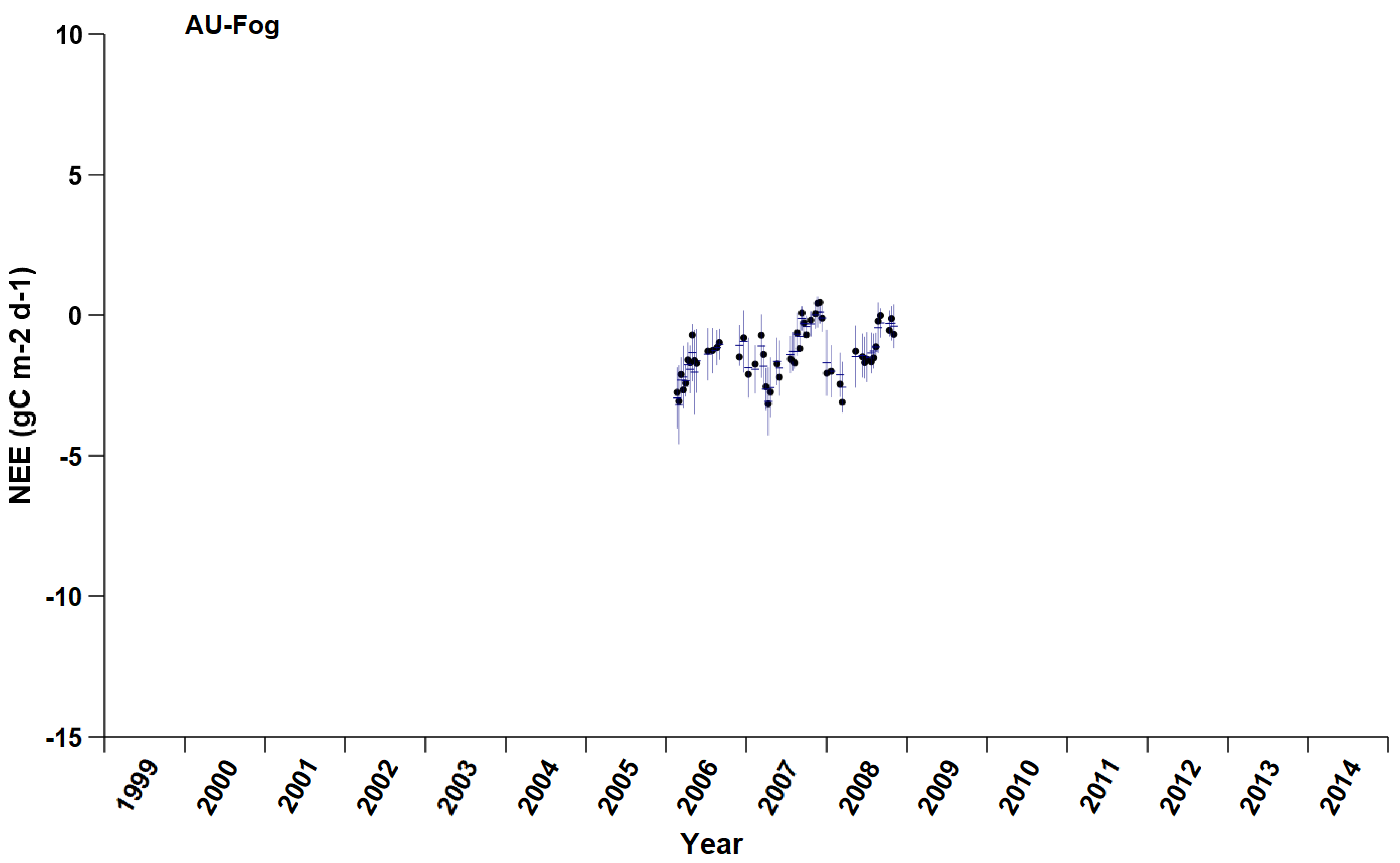
AU-Fog
NEE (gC m-2 d-1)
10
5
0
-5
-10
-15
1999
2000
2001
2002
2003
2004
2005
2006
2007
2008
2009
2010
2011
2012
2013
2014
Year

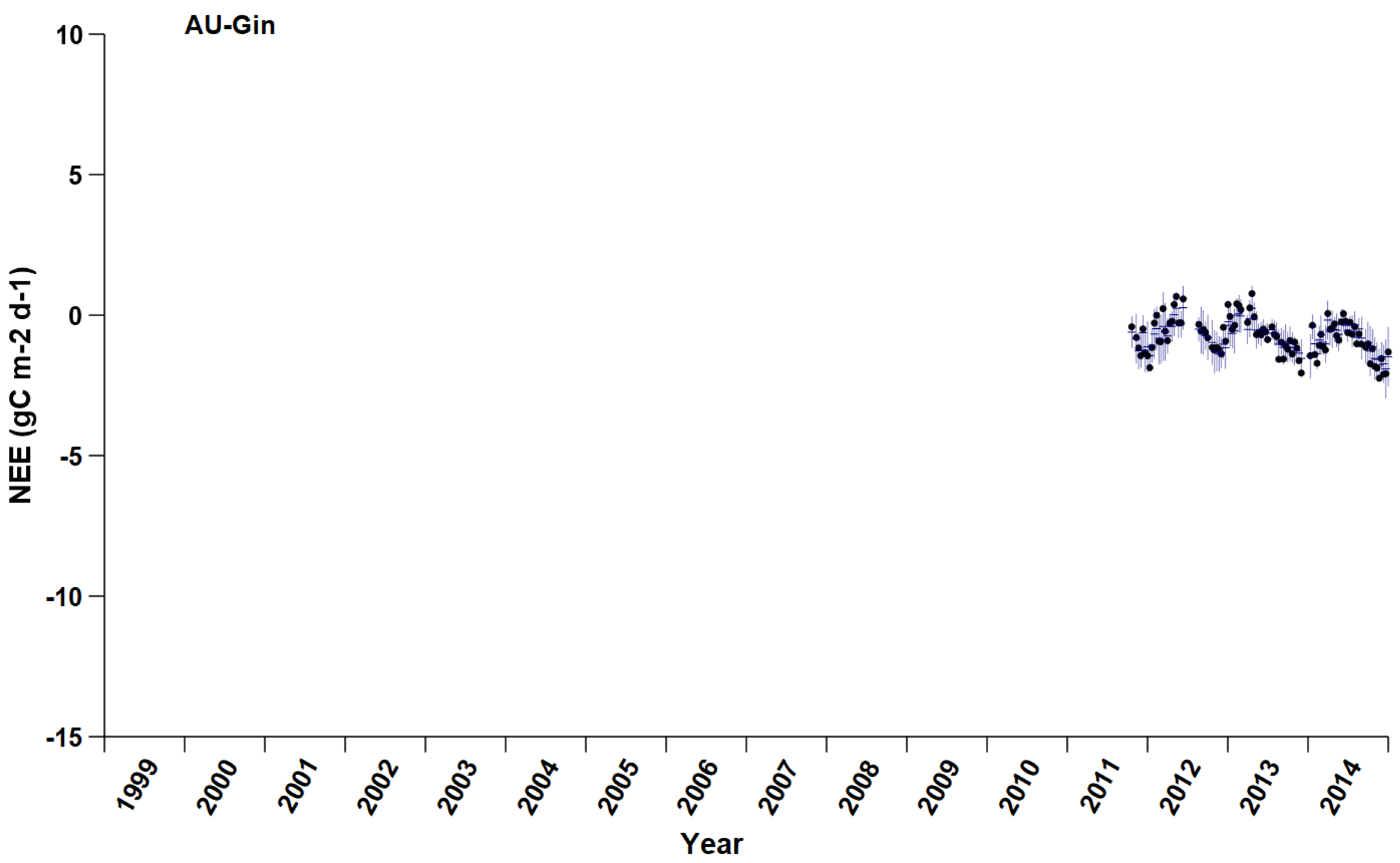

AU-Gin
NEE (gC m-2 d-1)
10
5
0
-5
-10
-15
1999
2000
2001
2002
2003
2004
2005
2006
2007
2008
2009
2010
2011
2012
2013
2014
Year

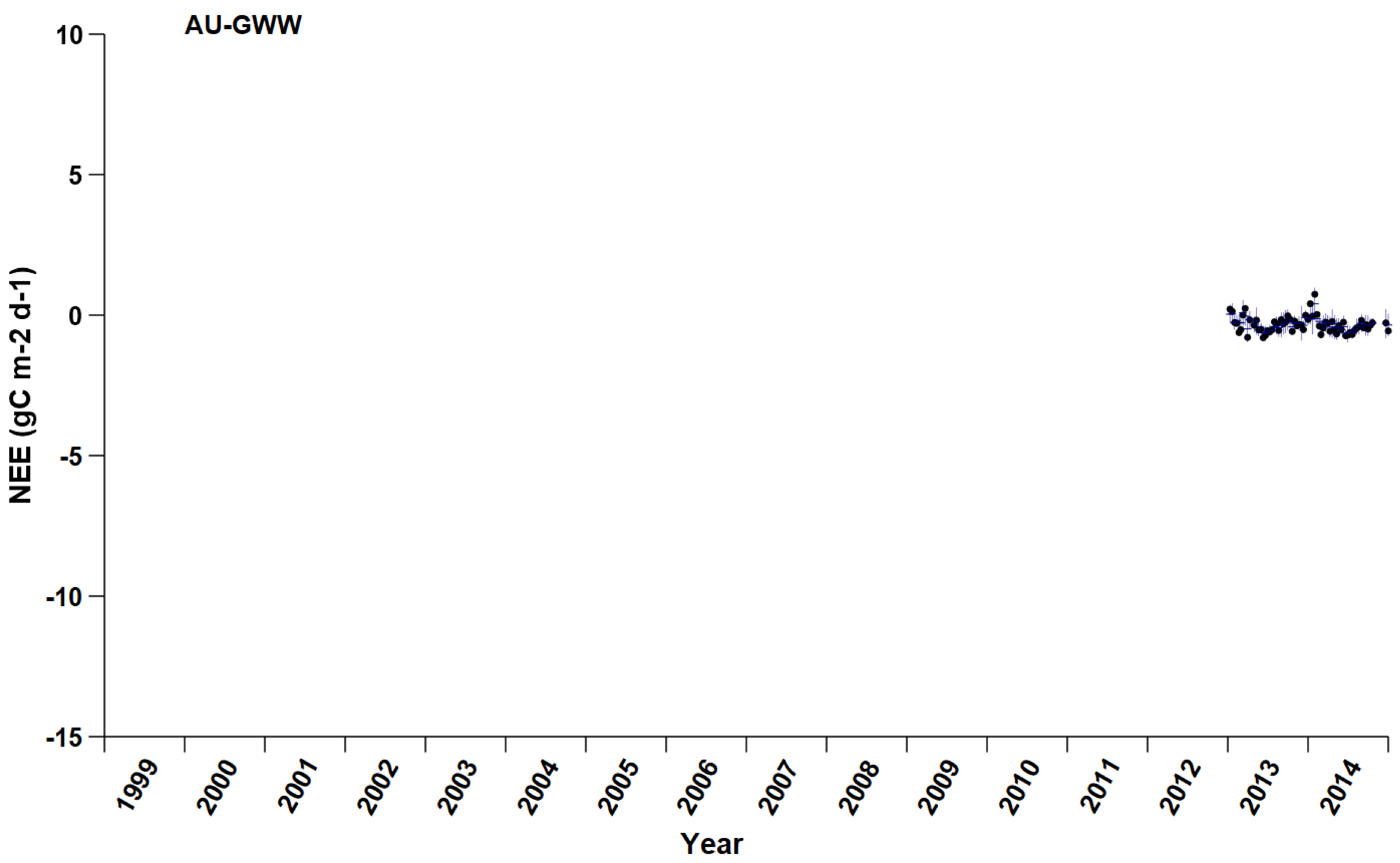

AU-GWW
NEE (gC m-2 d-1)
10
5
0
-5
-10
-15
1999
2000
2001
2002
2003
2004
2005
2006
2007
2008
2009
2010
2011
2012
2013
2014
Year

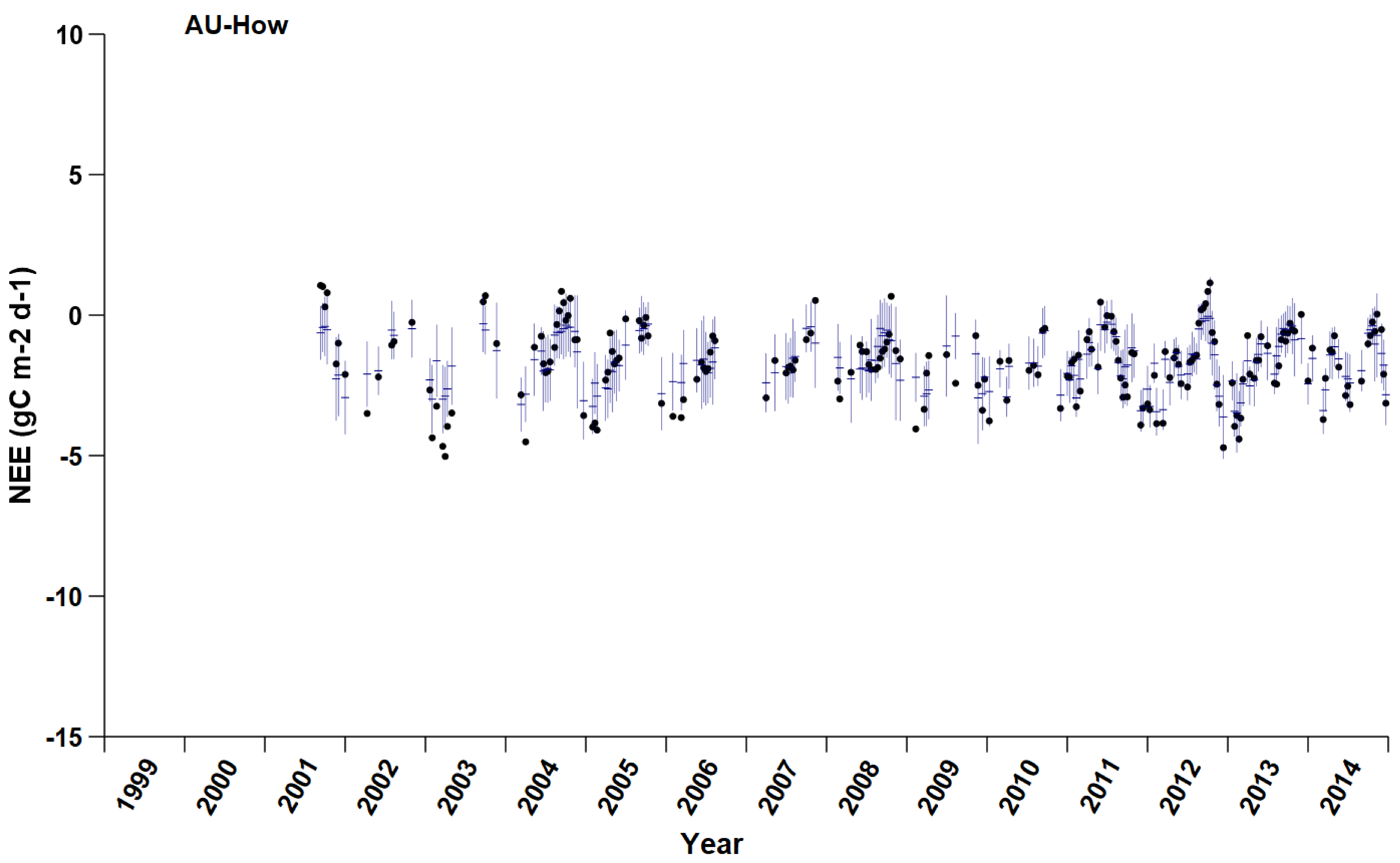
AU-How
NEE (gC m-2 d-1)
10
5
0
-5
-10
-15
1999
2000
2001
2002
2003
2004
2005
2006
2007
2008
2009
2010
2011
2012
2013
2014
Year

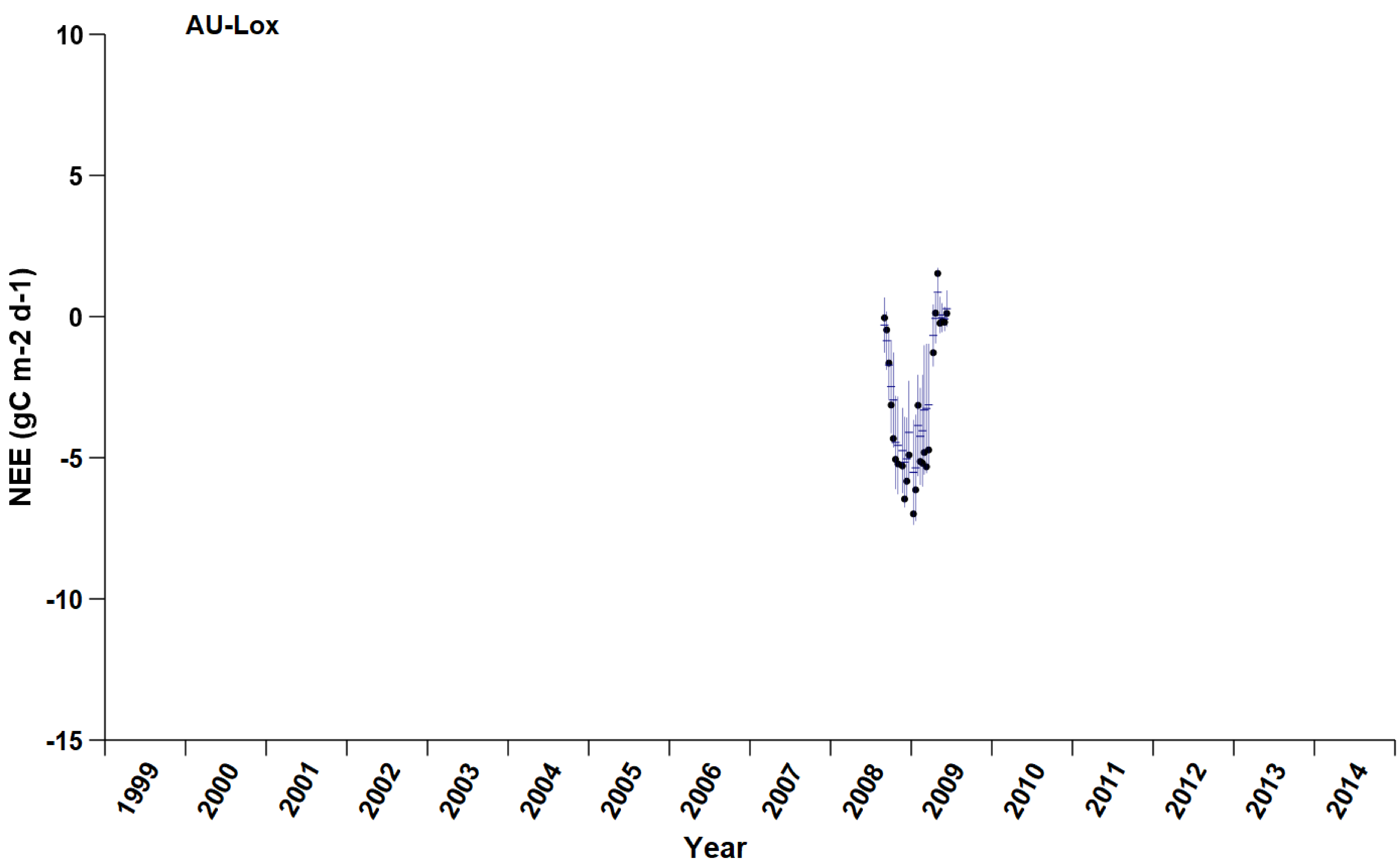
AU-Lox
10
5
0
-5
-10
-15
NEE (gC m-2 d-1)
1999
2000
2001
2002
2003
2004
2005
2006
2007
2008
2009
2010
2011
2012
2013
2014
Year

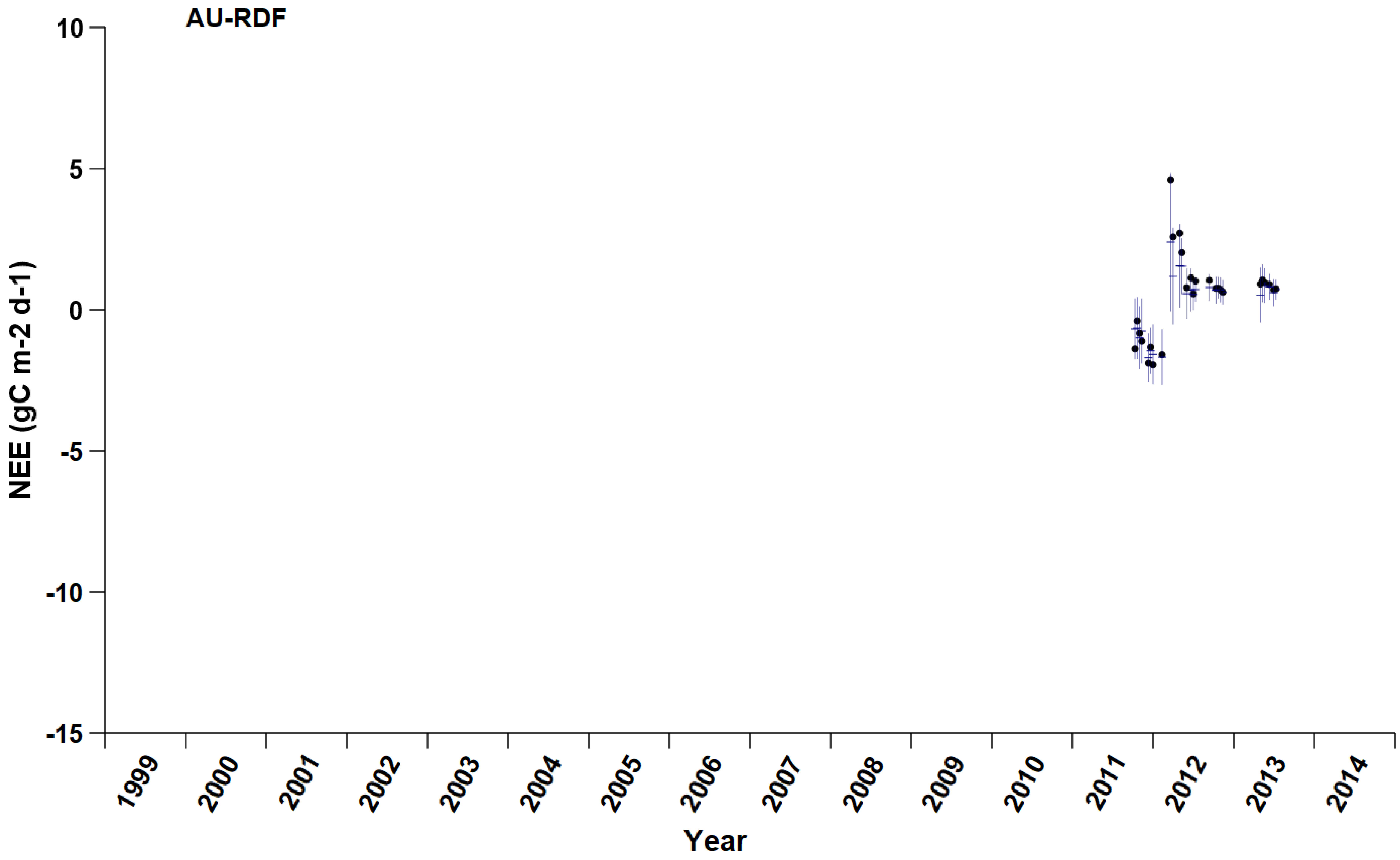

AU-RDF
10
5
0
-5
-10
-15
NEE (gC m-2 d-1)
1999
2000
2001
2002
2003
2004
2005
2006
2007
2008
2009
2010
2011
2012
2013
2014
Year

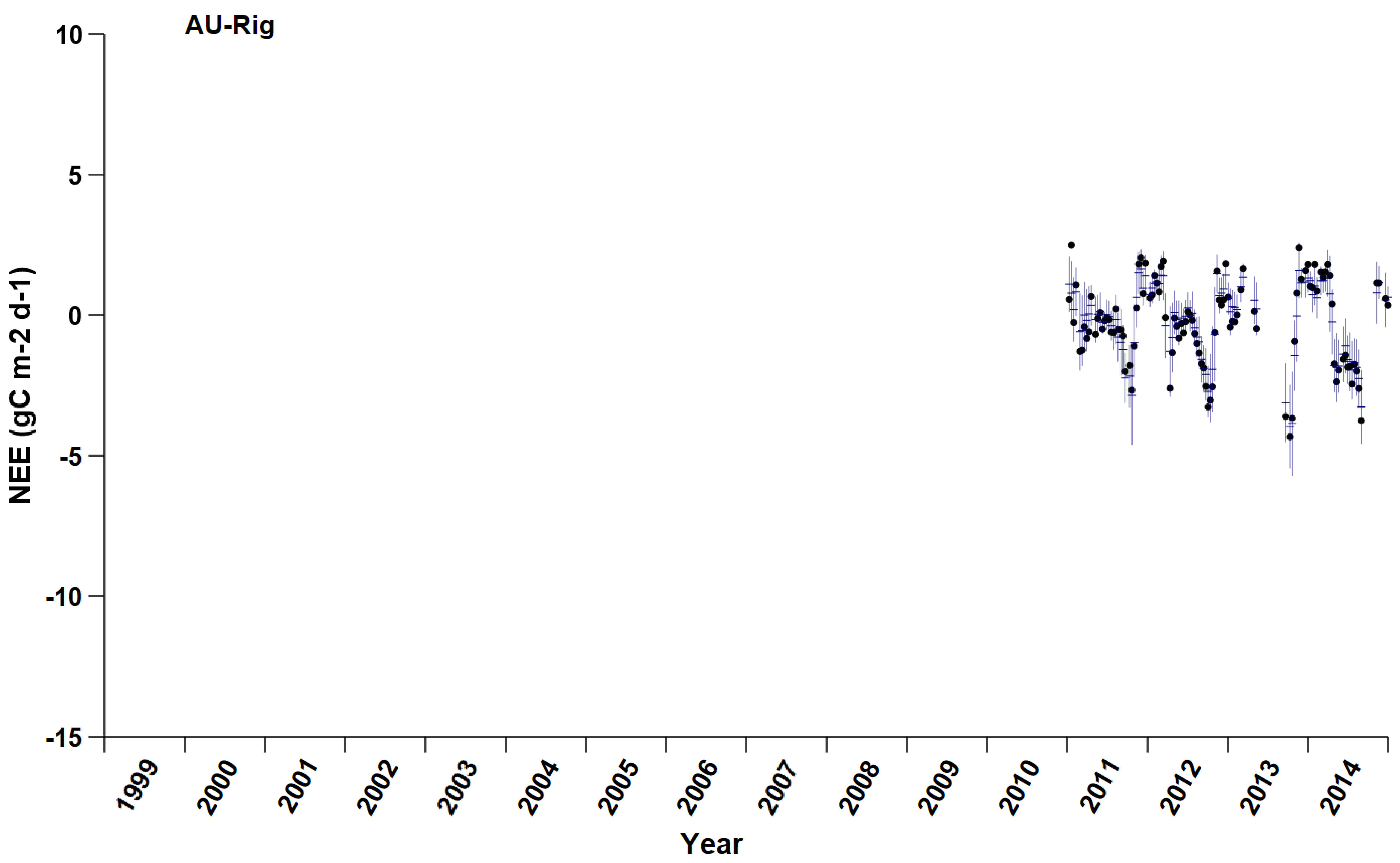
AU-Rig
NEE (gC m-2 d-1)
10
5
0
-5
-10
-15
1999
2000
2001
2002
2003
2004
2005
2006
2007
2008
2009
2010
2011
2012
2013
2014
Year

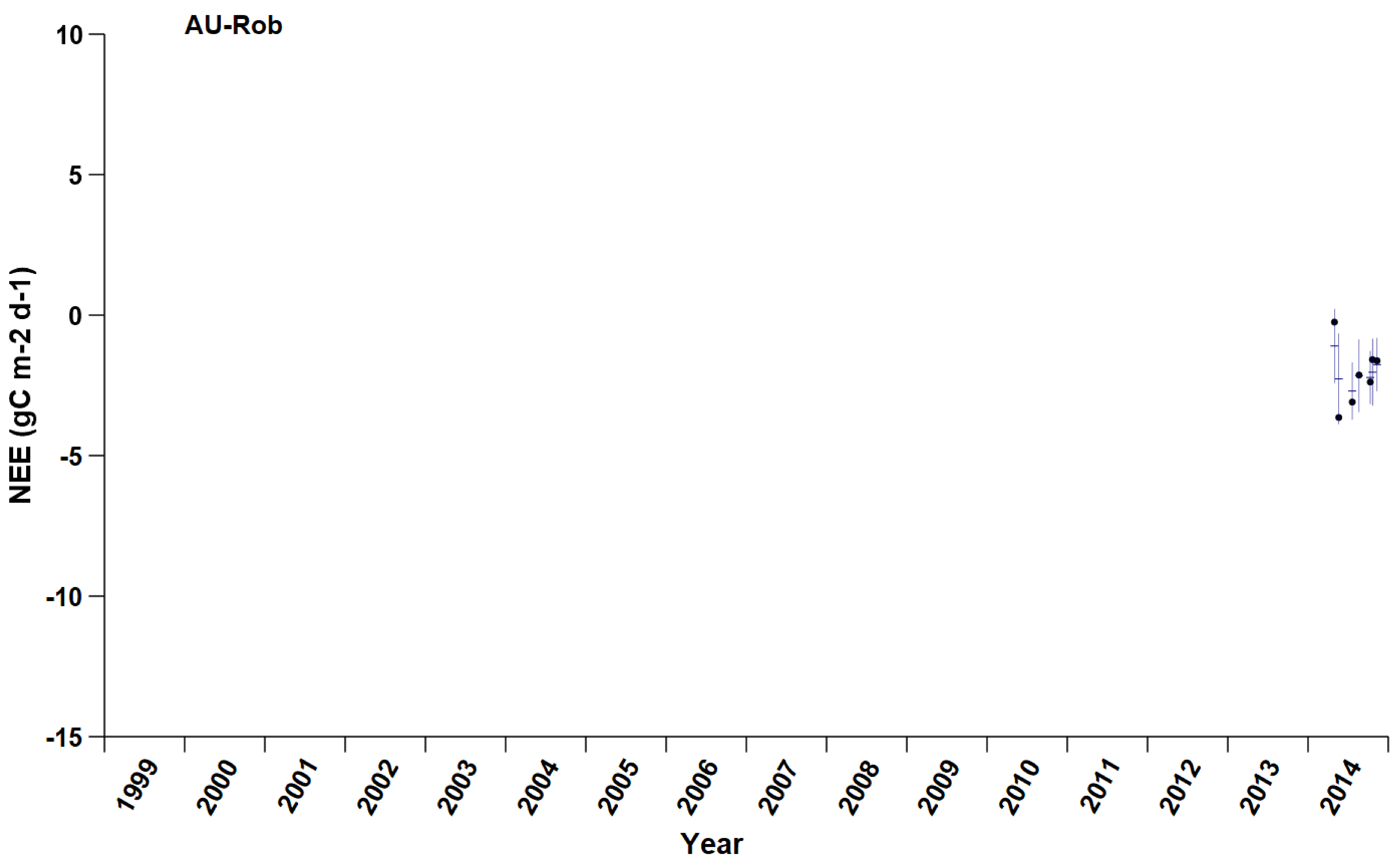

AU-Rob
NEE (gC m-2 d-1)
10
5
0
-5
-10
-15
1999
2000
2001
2002
2003
2004
2005
2006
2007
2008
2009
2010
2011
2012
2013
2014
Year

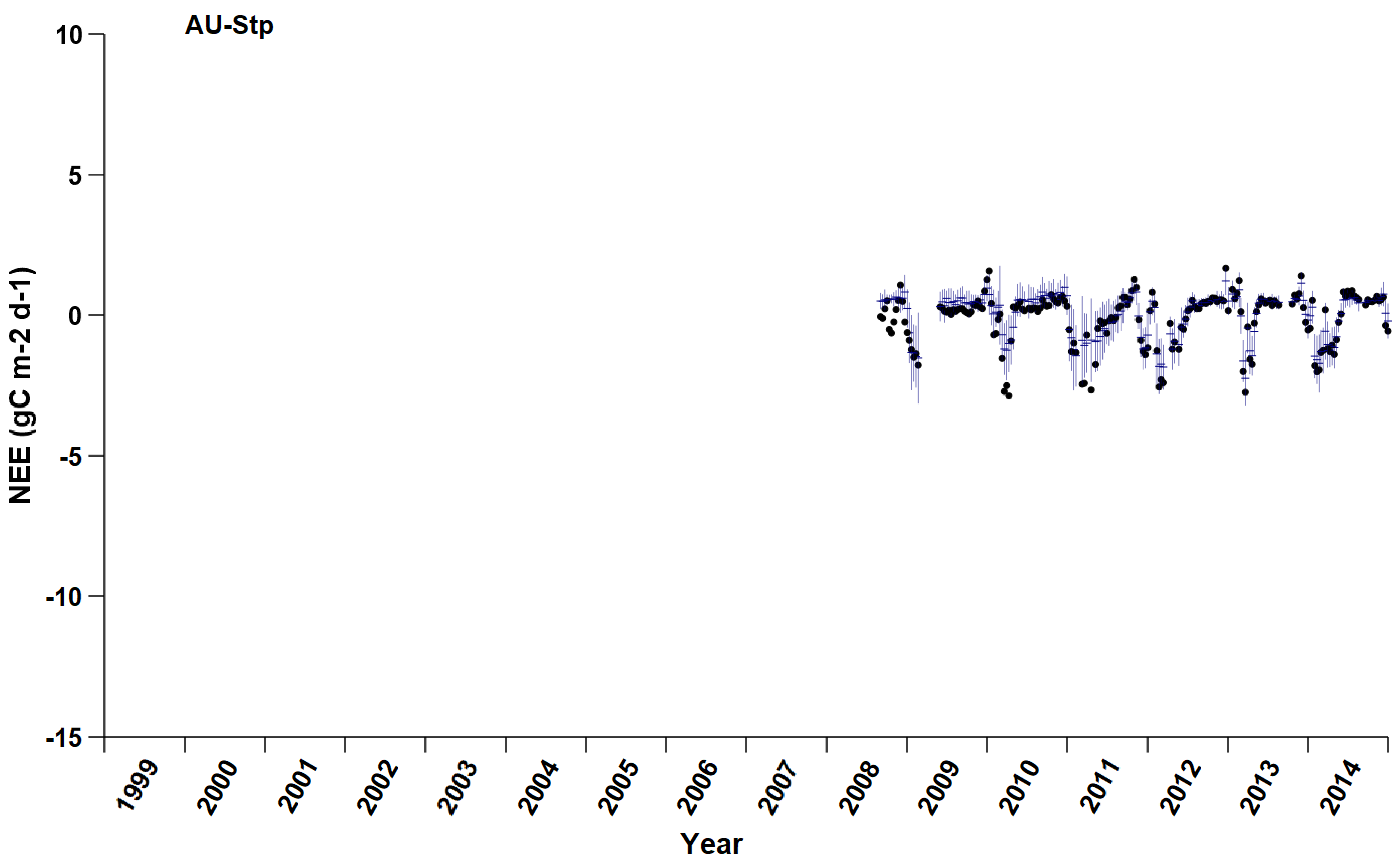
AU-Stp
NEE (gC m-2 d-1)
10
5
0
-5
-10
-15
1999
2000
2001
2002
2003
2004
2005
2006
2007
2008
2009
2010
2011
2012
2013
2014
Year

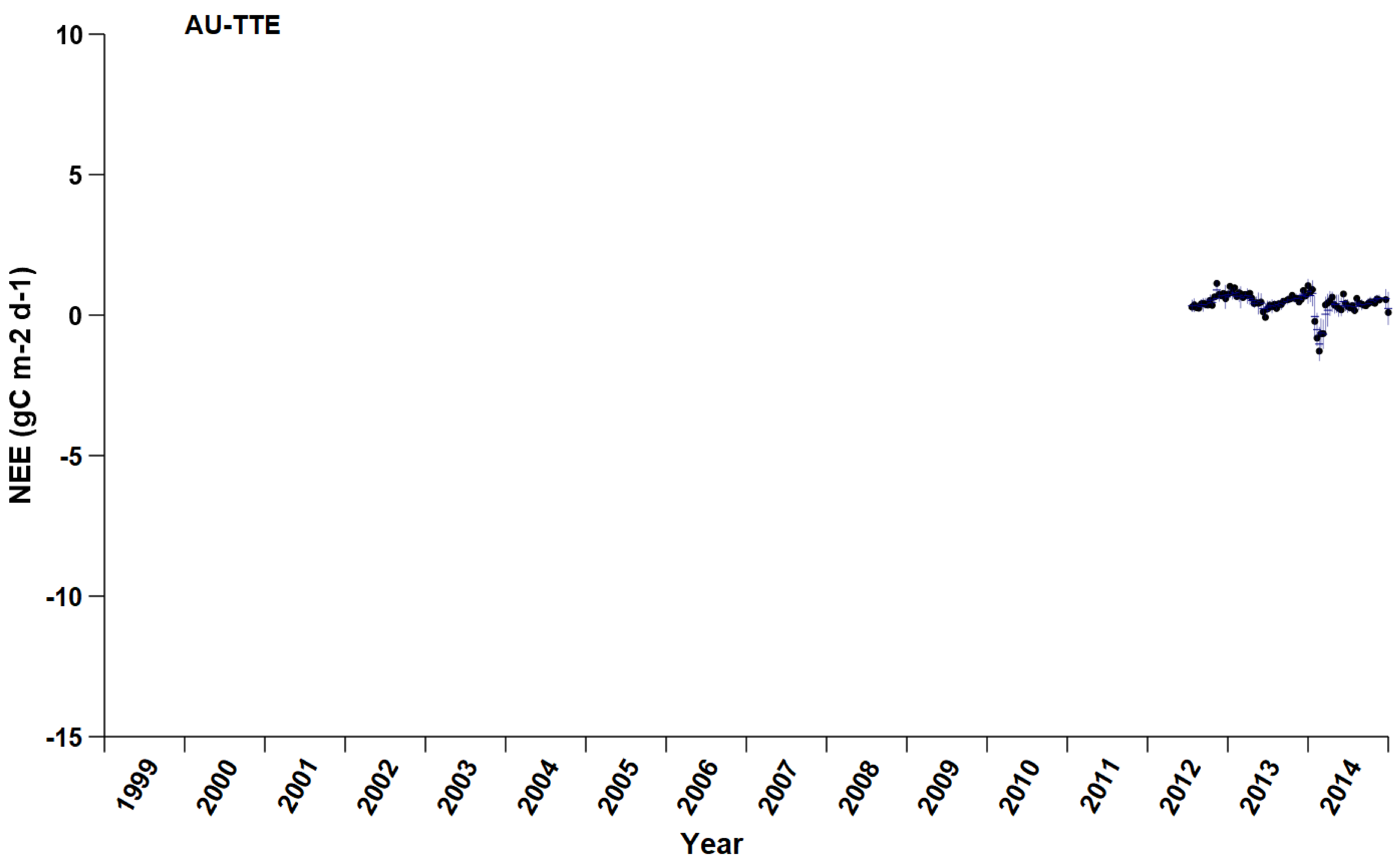
AU-TTE
NEE (gC m-2 d-1)
10
5
0
-5
-10
-15
1999
2000
2001
2002
2003
2004
2005
2006
2007
2008
2009
2010
2011
2012
2013
2014
Year

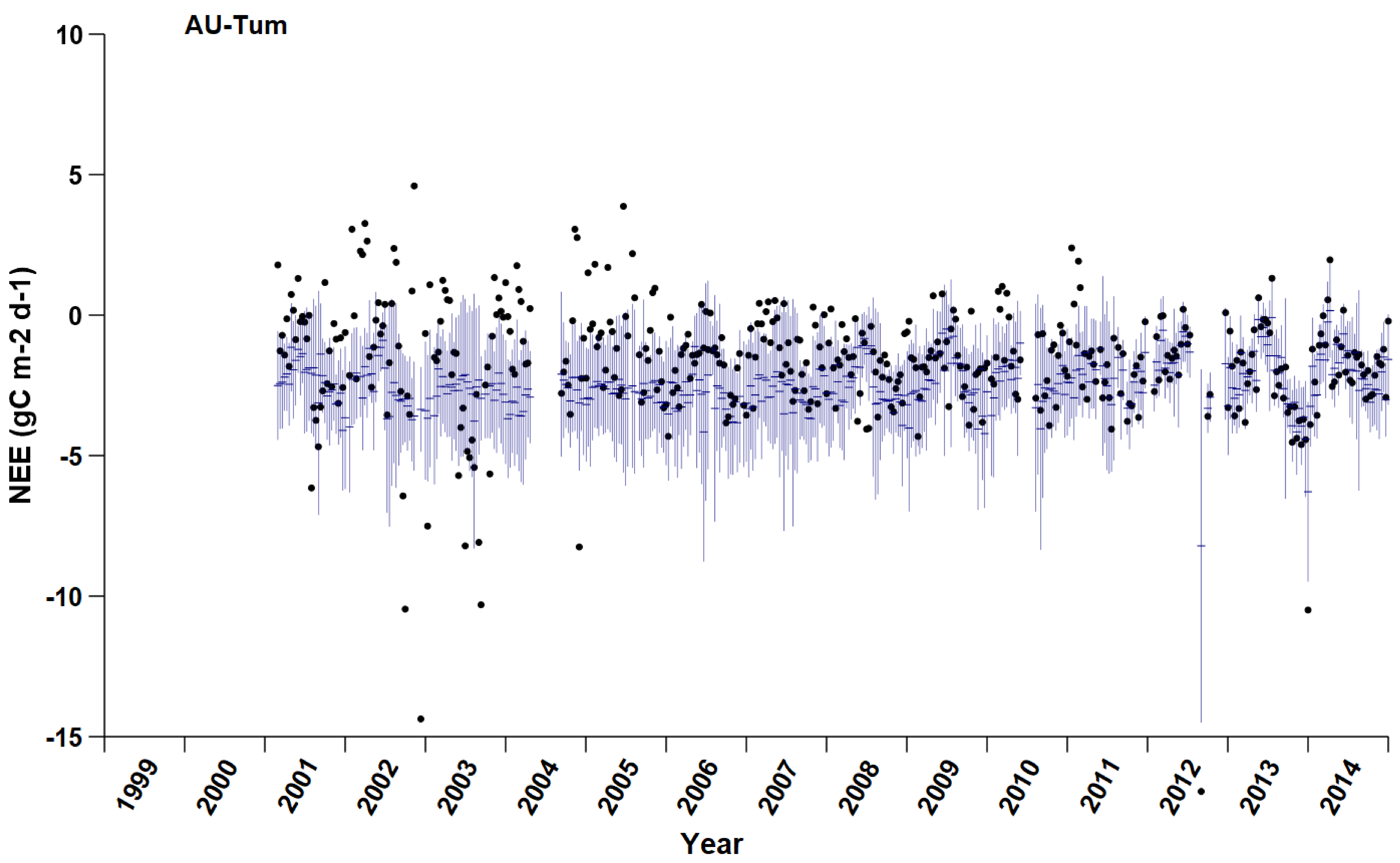
AU-Tum
NEE (gC m-2 d-1)
10
5
0
-5
-10
-15
1999
2000
2001
2002
2003
2004
2005
2006
2007
2008
2009
2010
2011
2012
2013
2014
Year

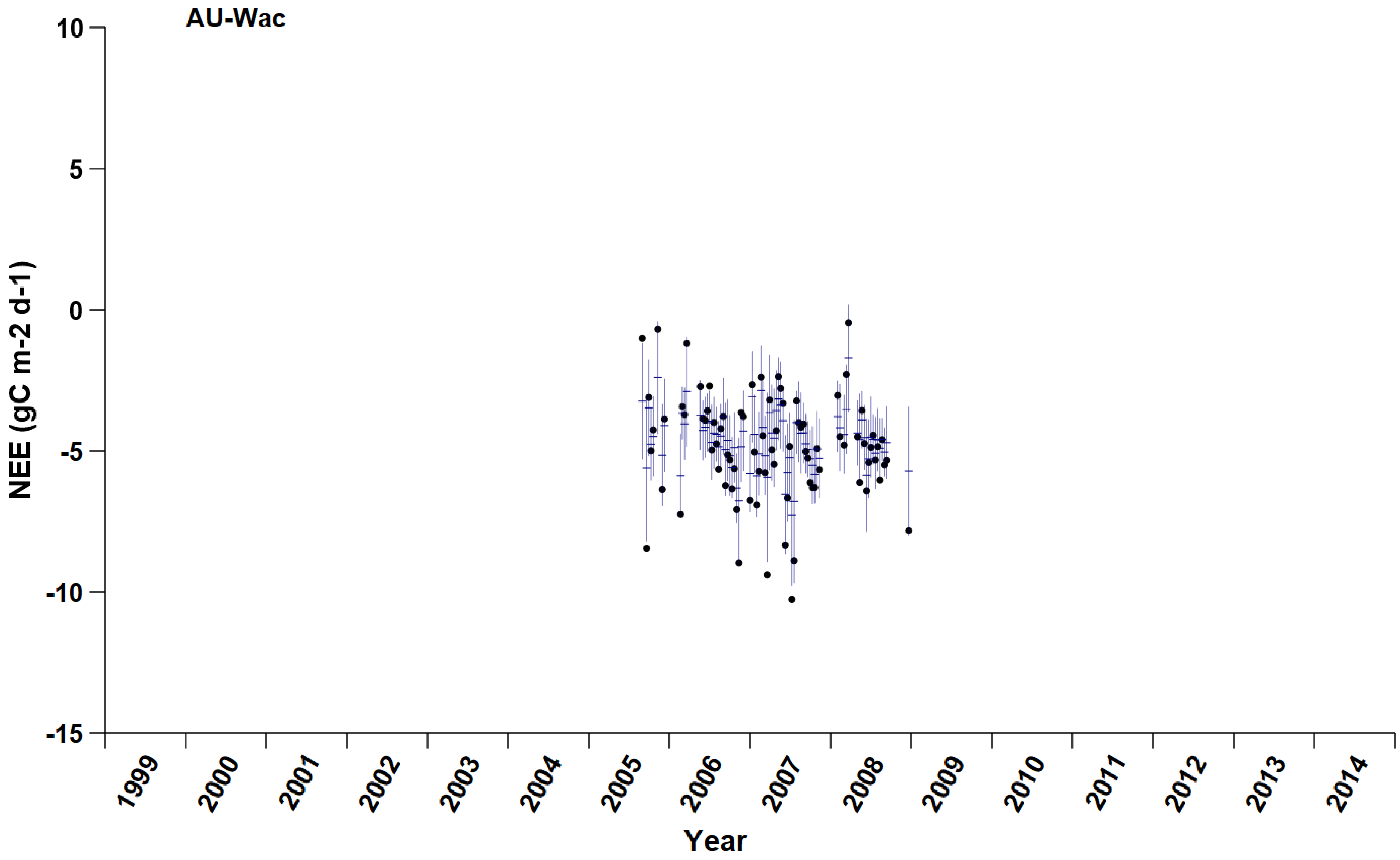

AU-Wac
NEE (gC m-2 d-1)
10
5
0
-5
-10
-15
1999
2000
2001
2002
2003
2004
2005
2006
2007
2008
2009
2010
2011
2012
2013
2014
Year

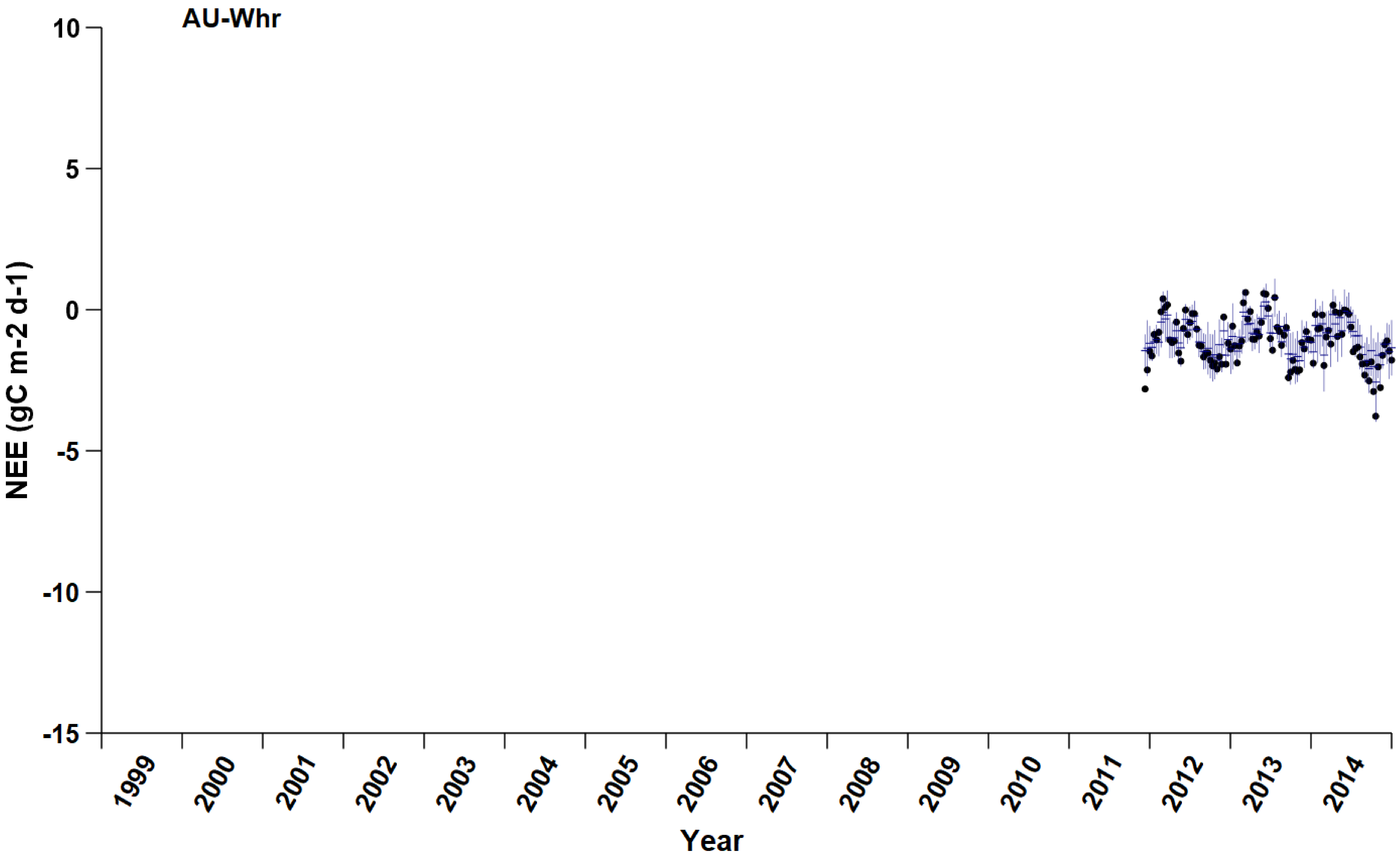

AU-Whr
NEE (gC m-2 d-1)
10
5
0
-5
-10
-15
1999
2000
2001
2002
2003
2004
2005
2006
2007
2008
2009
2010
2011
2012
2013
2014
Year

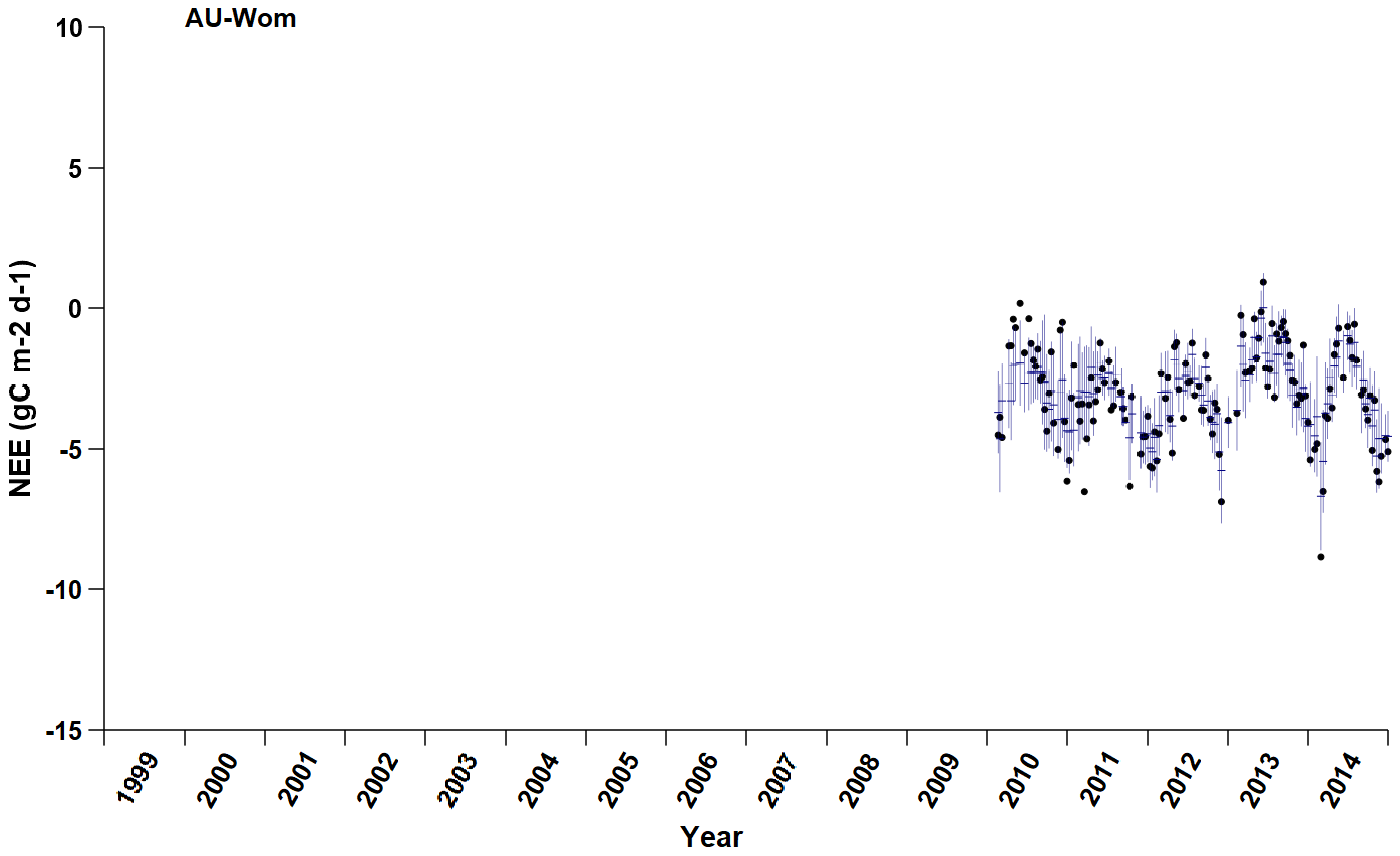
AU-Wom
10
5
0
-5
-10
-15
NEE (gC m-2 d-1)
1999
2000
2001
2002
2003
2004
2005
2006
2007
2008
2009
2010
2011
2012
2013
2014
Year

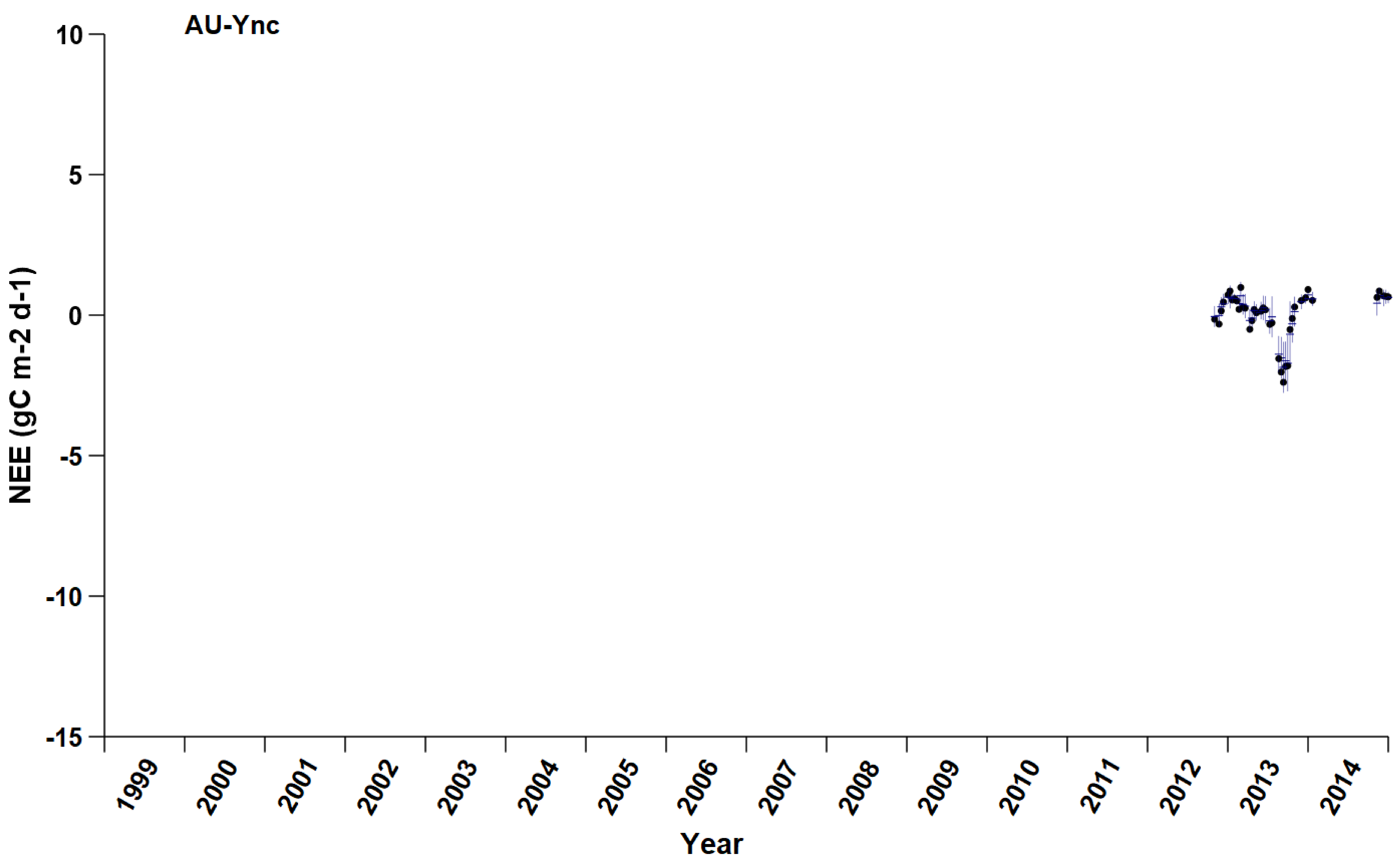
AU-Ync
NEE (gC m-2 d-1)
10
5
0
-5
-10
-15
1999
2000
2001
2002
2003
2004
2005
2006
2007
2008
2009
2010
2011
2012
2013
2014
Year

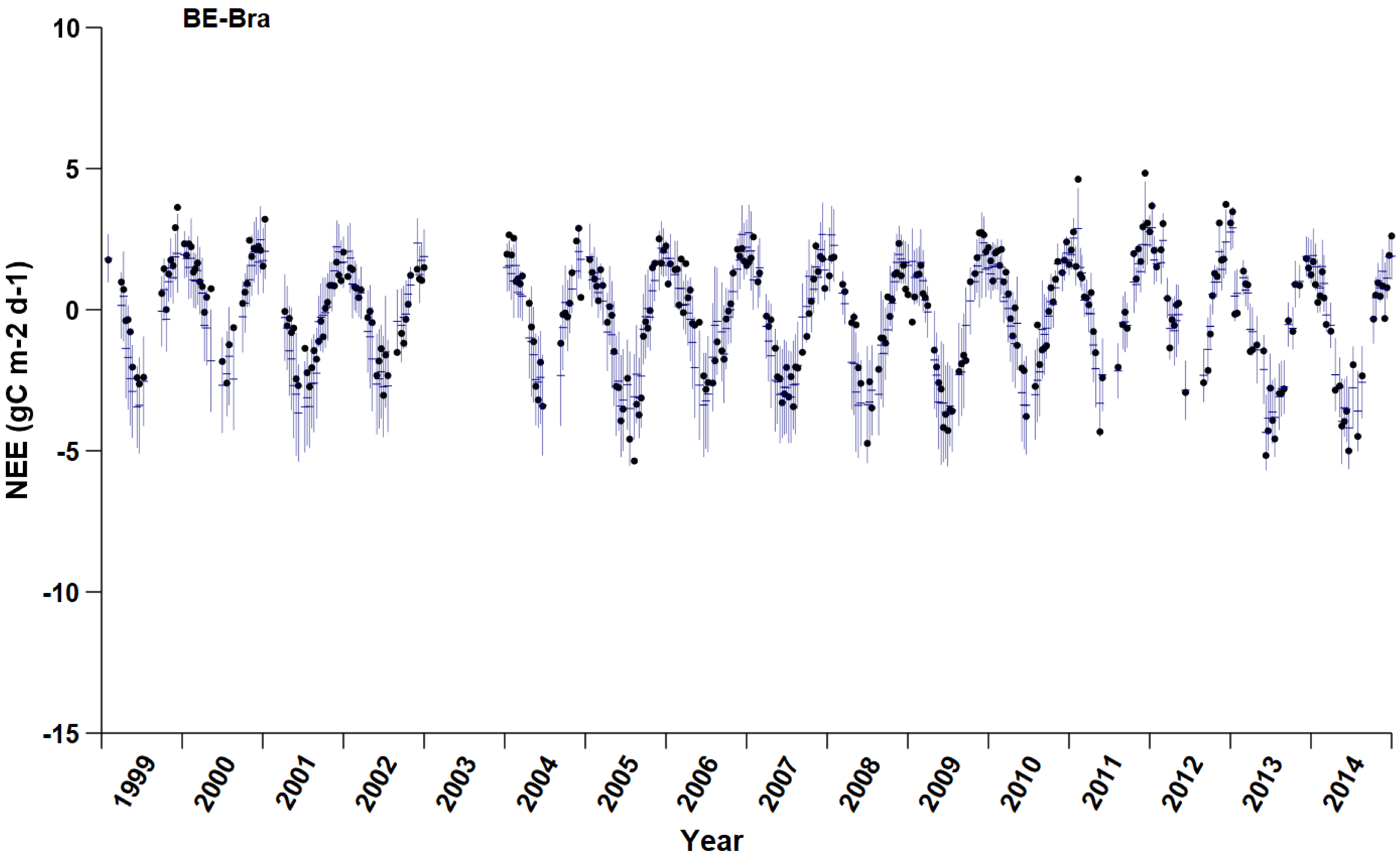

BE-Bra
10
5
0
-5
-10
-15
NEE (gC m-2 d-1)
1999
2000
2001
2002
2003
2004
2005
2006
2007
2008
2009
2010
2011
2012
2013
2014
Year

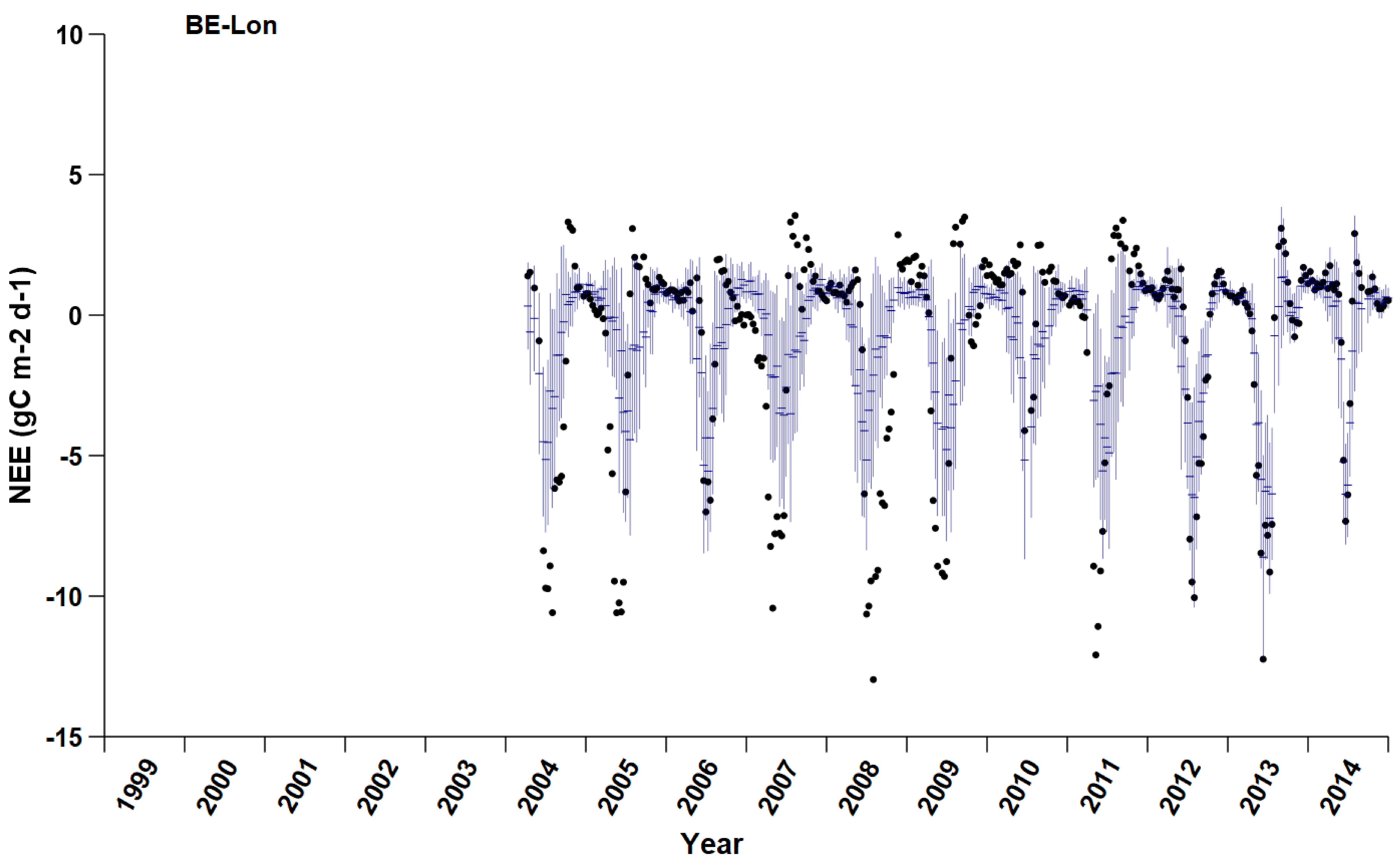

BE-Lon
10
5
0
-5
-10
-15
NEE (gC m-2 d-1)
1999
2000
2001
2002
2003
2004
2005
2006
2007
2008
2009
2010
2011
2012
2013
2014
Year

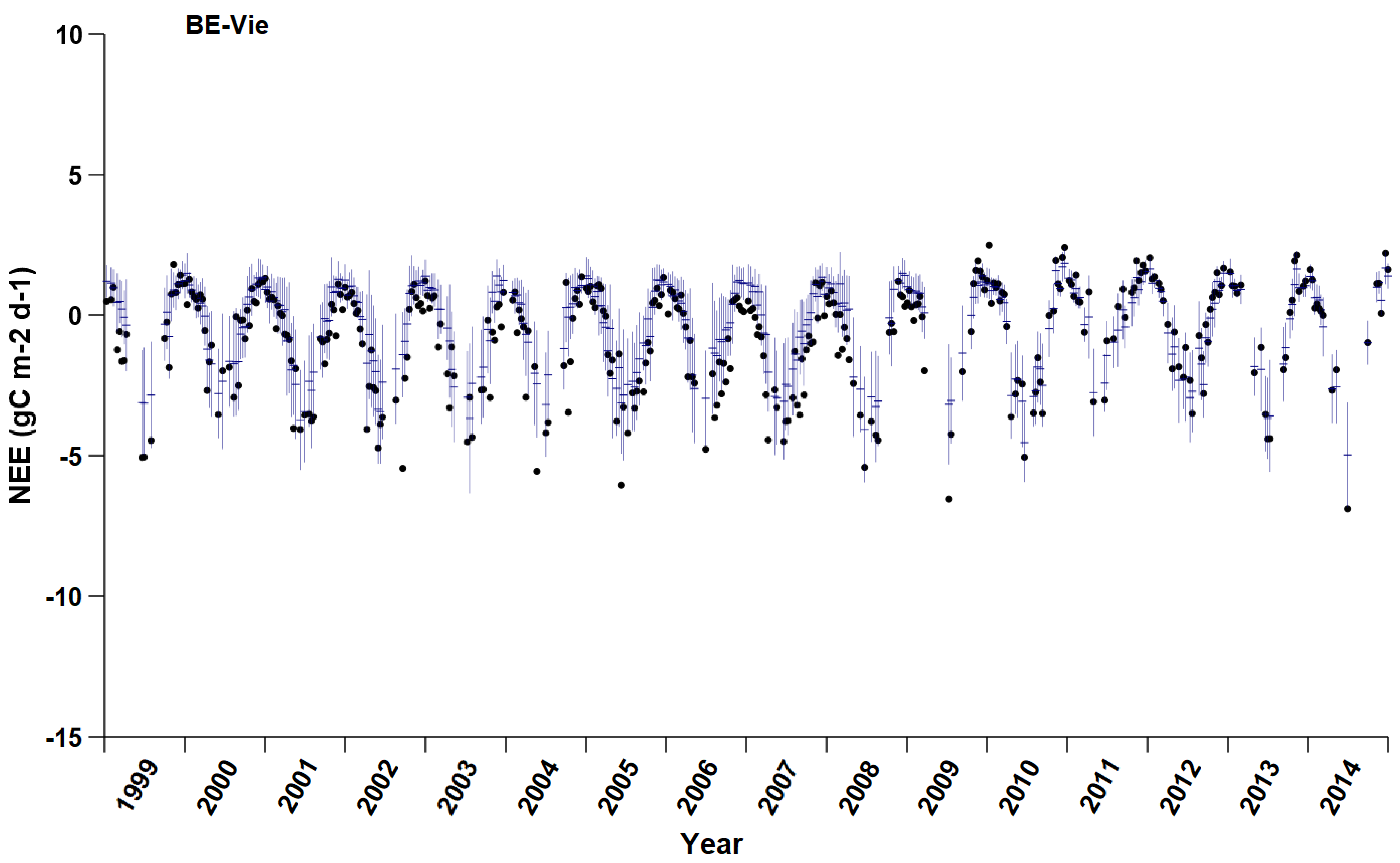

BE-Vie
10
5
0
-5
-10
-15
NEE (gC m-2 d-1)
1999
2000
2001
2002
2003
2004
2005
2006
2007
2008
2009
2010
2011
2012
2013
2014
Year

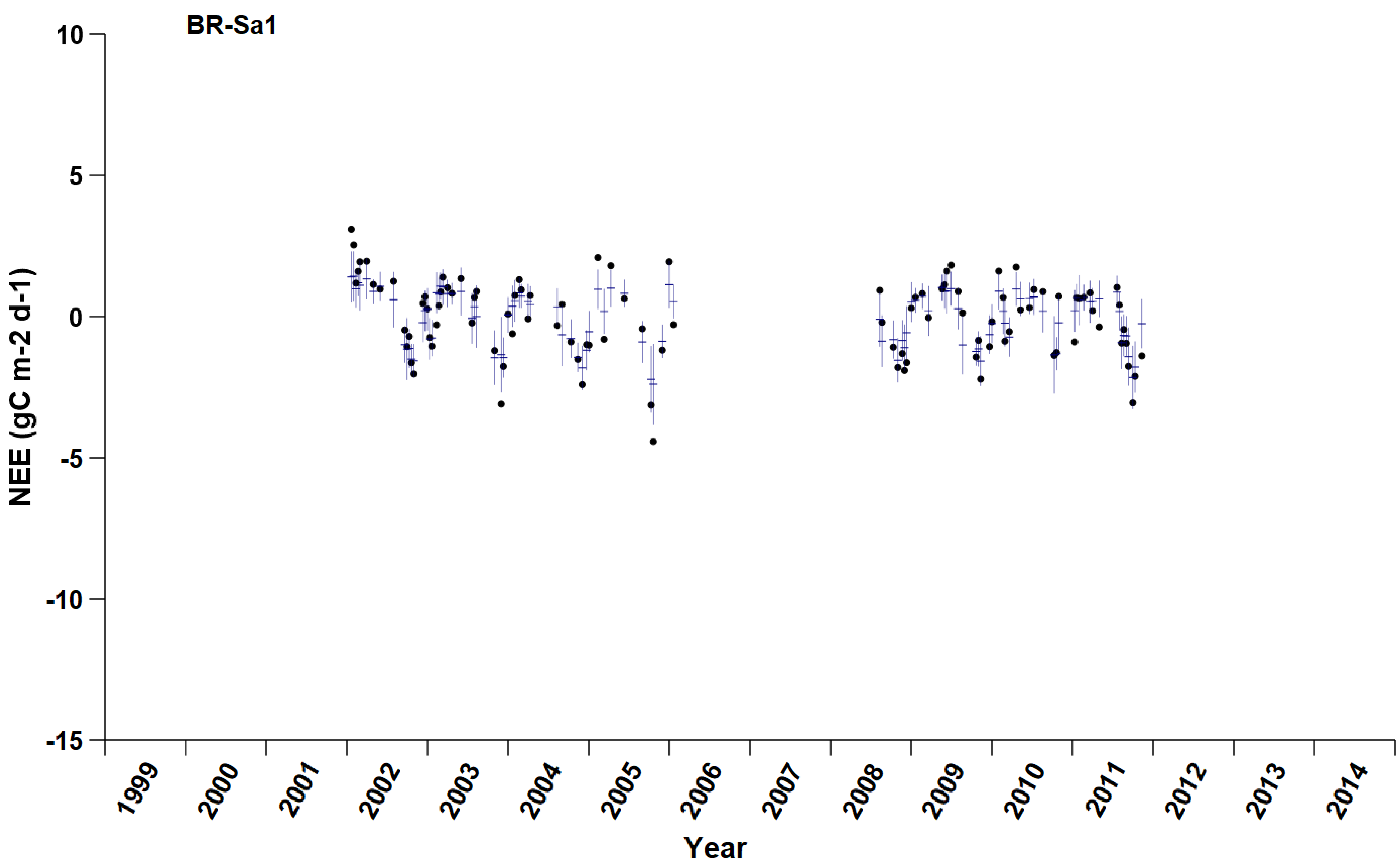
BR-Sa1
NEE (gC m-2 d-1)
10
5
0
-5
-10
-15
1999
2000
2001
2002
2003
2004
2005
2006
2007
2008
2009
2010
2011
2012
2013
2014
Year

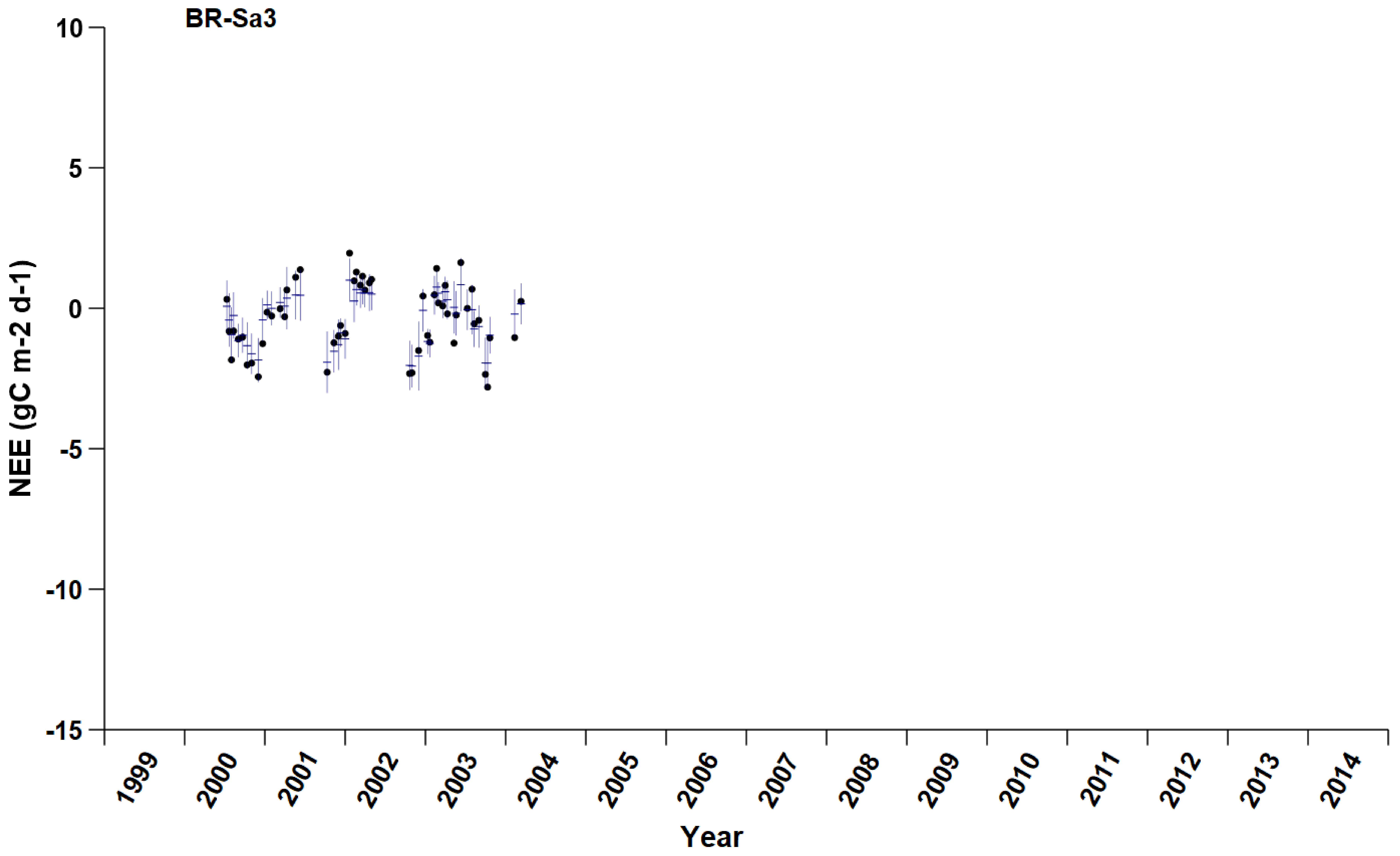
BR-Sa3
NEE (gC m-2 d-1)
10
5
0
-5
-10
-15
1999
2000
2001
2002
2003
2004
2005
2006
2007
2008
2009
2010
2011
2012
2013
2014
Year

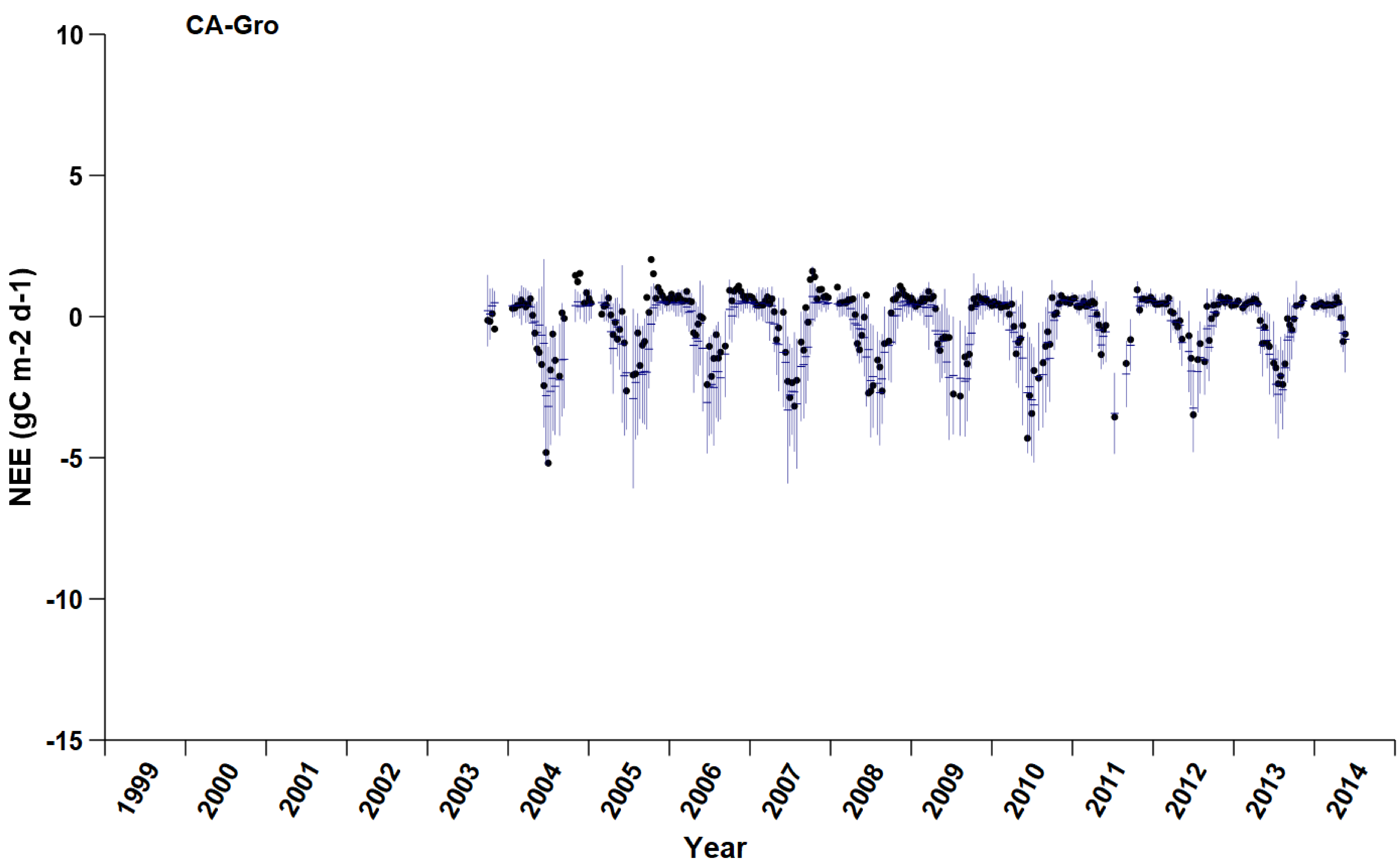
CA-Gro
10
5
0
-5
-10
-15
NEE (gC m-2 d-1)
1999
2000
2001
2002
2003
2004
2005
2006
2007
2008
2009
2010
2011
2012
2013
2014
Year

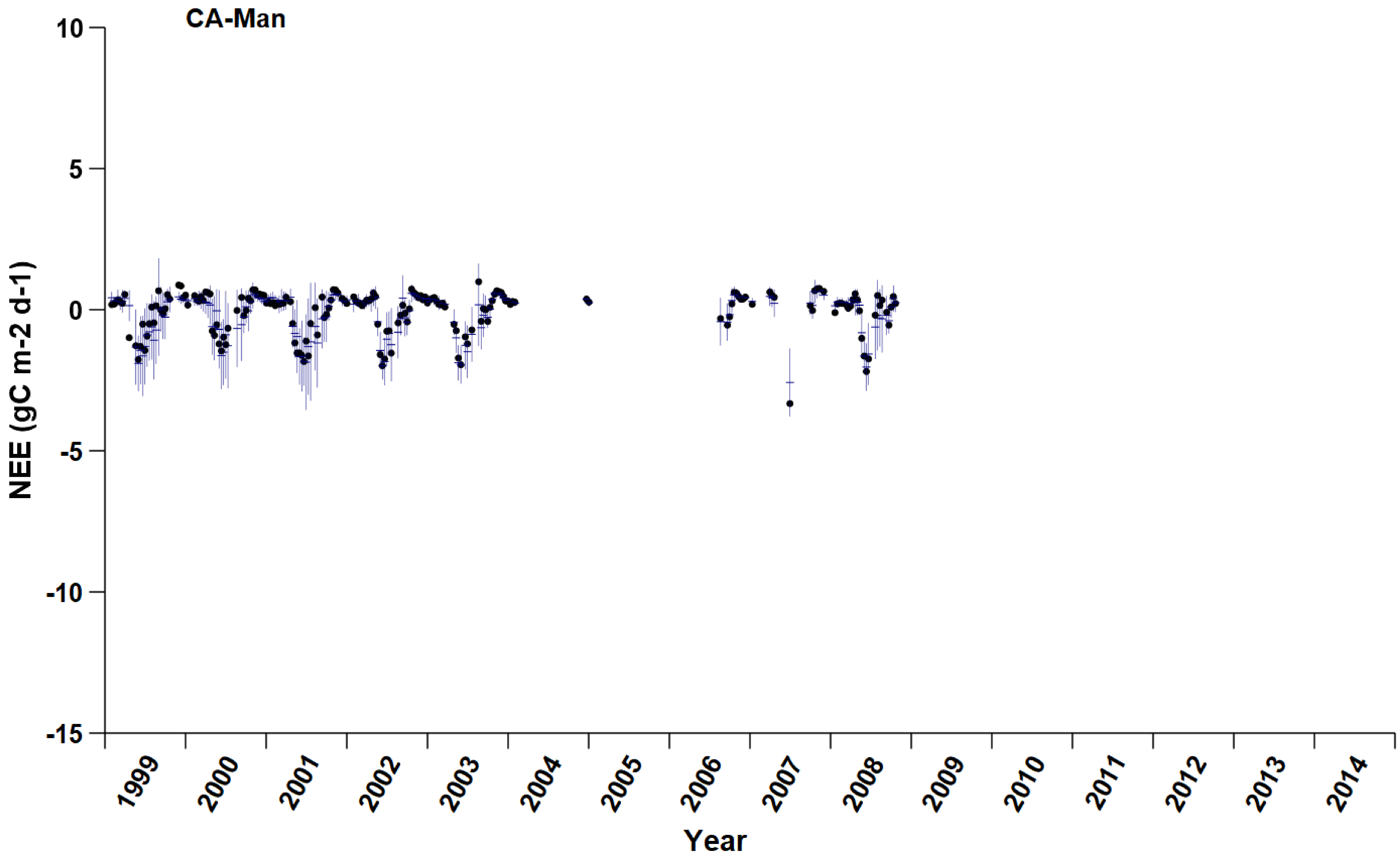
CA-Man
NEE (gC m-2 d-1)
10
5
0
-5
-10
-15
1999
2000
2001
2002
2003
2004
2005
2006
2007
2008
2009
2010
2011
2012
2013
2014
Year

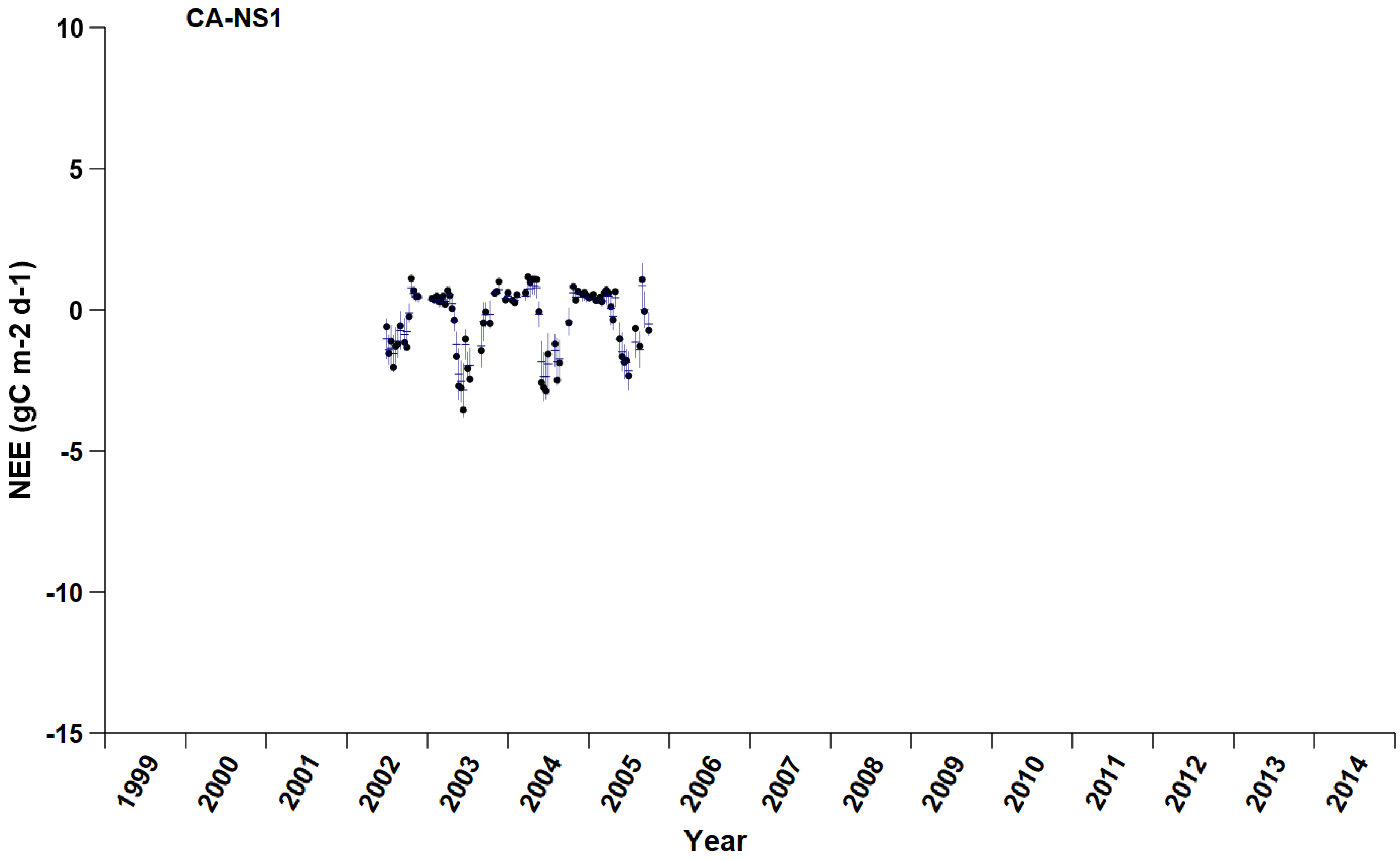
CA-NS1
NEE (gC m-2 d-1)
10
5
0
-5
-10
-15
1999
2000
2001
2002
2003
2004
2005
2006
2007
2008
2009
2010
2011
2012
2013
2014
Year

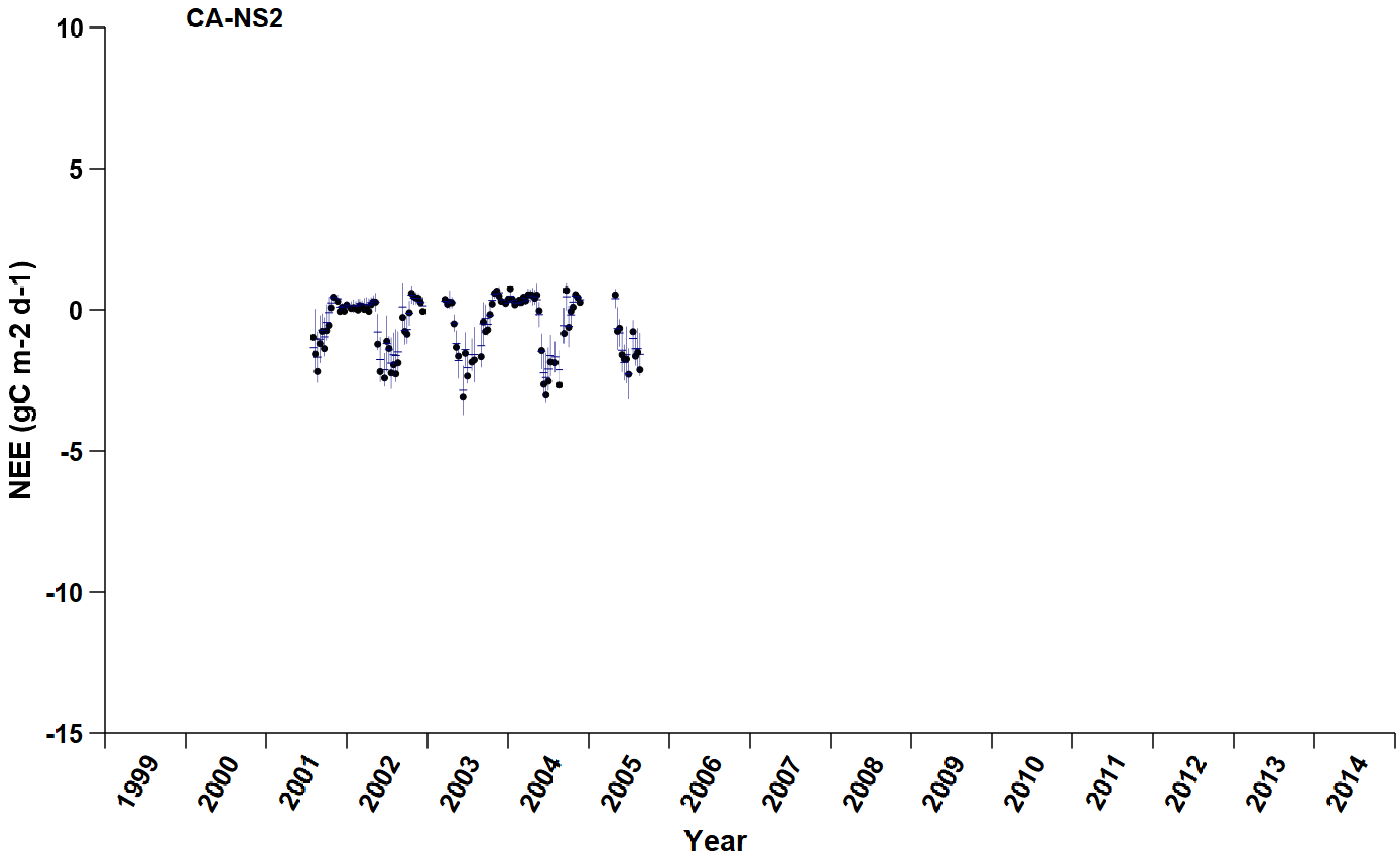
CA-NS2
10
5
0
-5
-10
-15
NEE (gC m-2 d-1)
1999
2000
2001
2002
2003
2004
2005
2006
2007
2008
2009
2010
2011
2012
2013
2014
Year

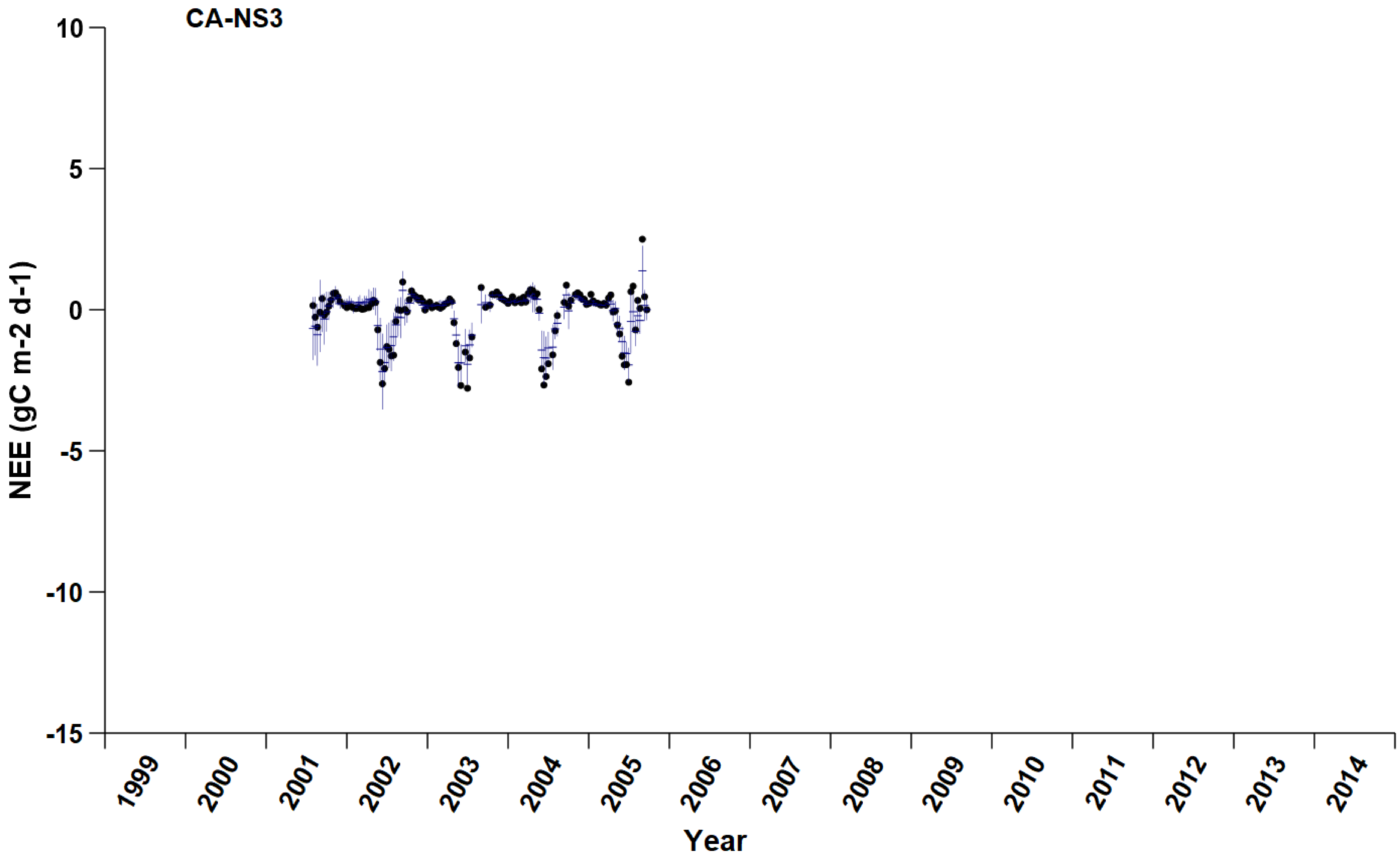

CA-NS3
10
5
0
-5
-10
-15
NEE (gC m-2 d-1)
1999
2000
2001
2002
2003
2004
2005
2006
2007
2008
2009
2010
2011
2012
2013
2014
Year

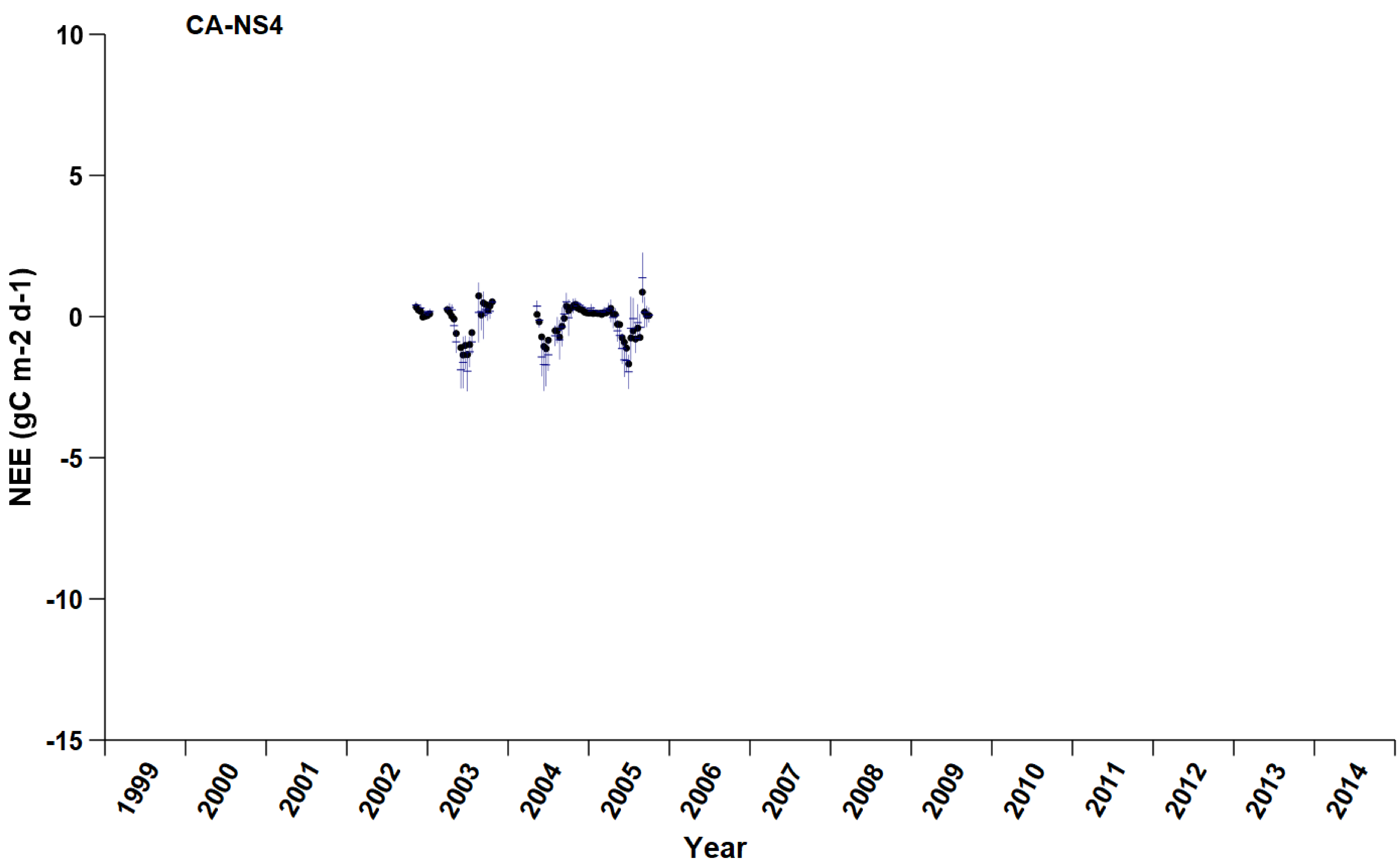
CA-NS4
10
5
0
-5
-10
-15
NEE (gC m-2 d-1)
1999
2000
2001
2002
2003
2004
2005
2006
2007
2008
2009
2010
2011
2012
2013
2014
Year

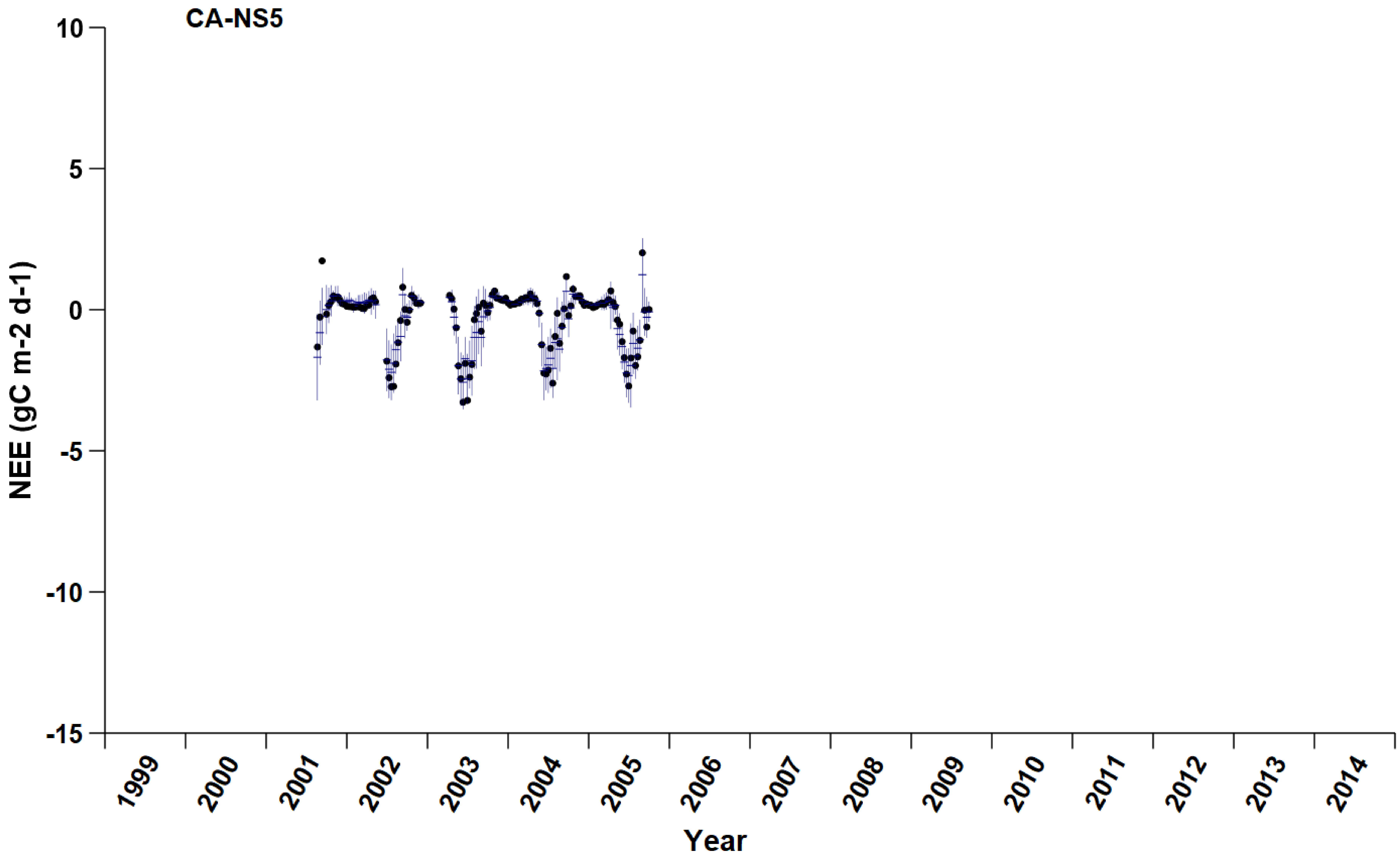
CA-NS5
10
5
0
-5
-10
-15
NEE (gC m-2 d-1)
1999
2000
2001
2002
2003
2004
2005
2006
2007
2008
2009
2010
2011
2012
2013
2014
Year

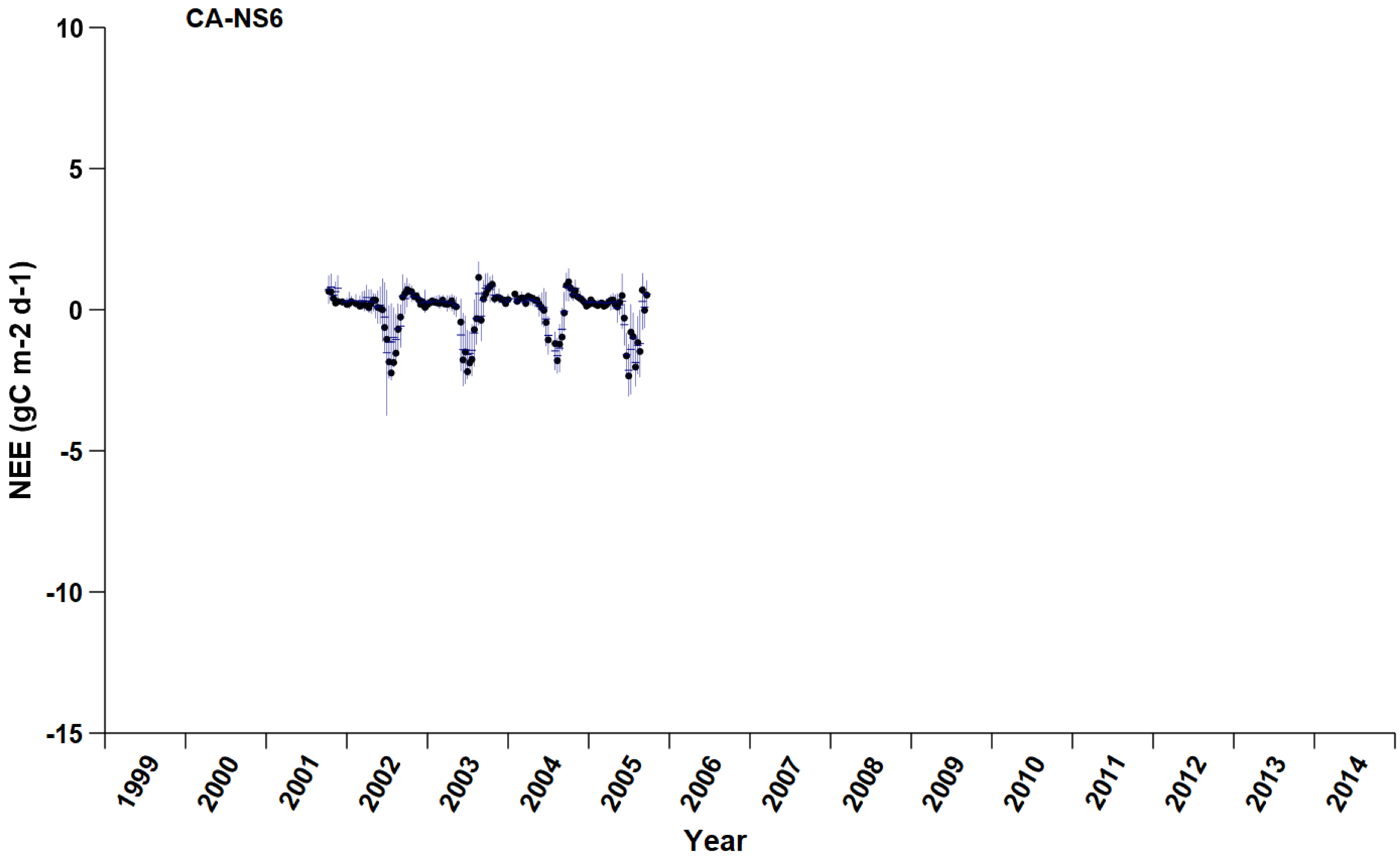

CA-NS6
10
5
0
-5
-10
-15
NEE (gC m-2 d-1)
1999
2000
2001
2002
2003
2004
2005
2006
2007
2008
2009
2010
2011
2012
2013
2014
Year

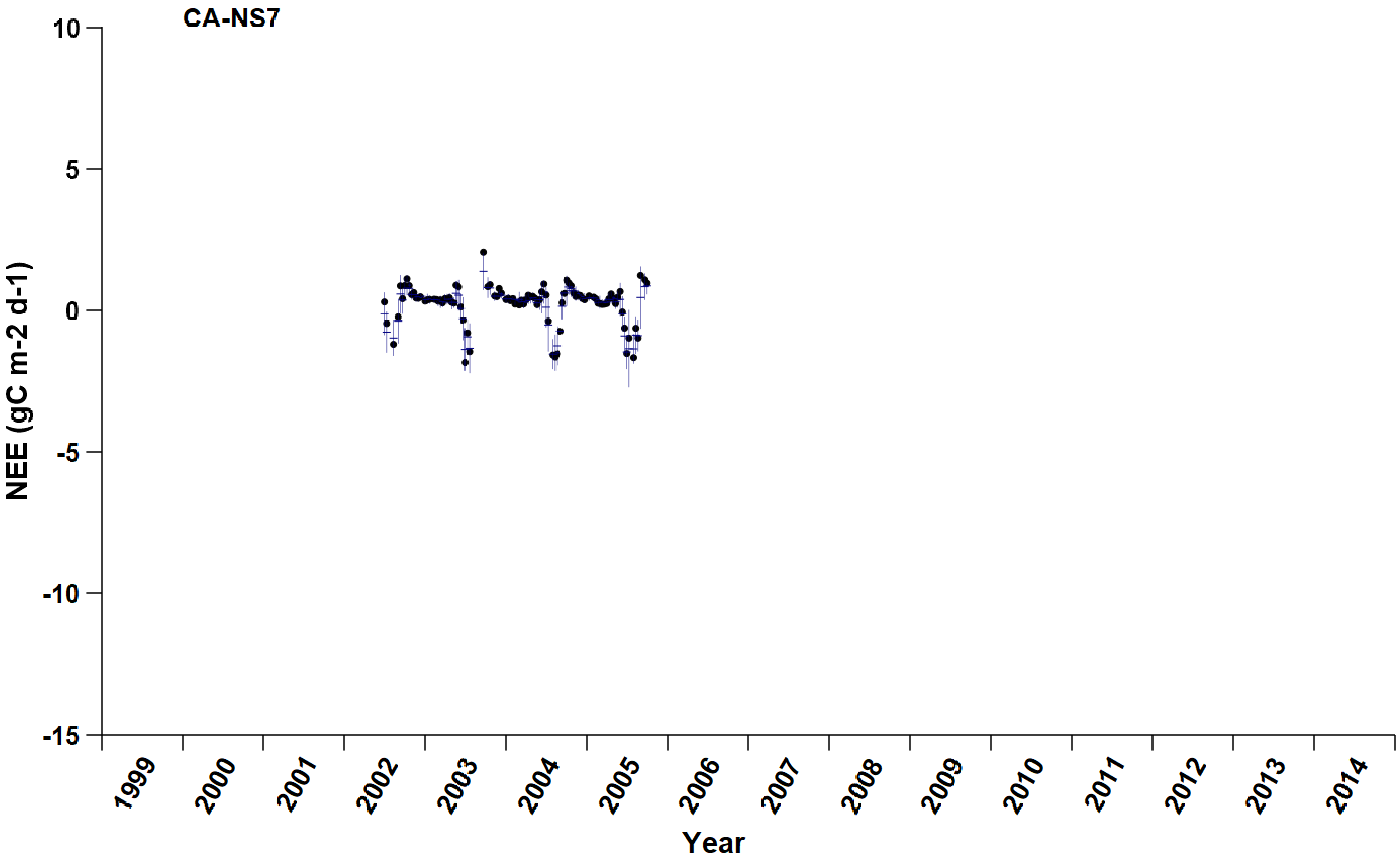

CA-NS7
NEE (gC m-2 d-1)
10
5
0
-5
-10
-15
1999
2000
2001
2002
2003
2004
2005
2006
2007
2008
2009
2010
2011
2012
2013
2014
Year

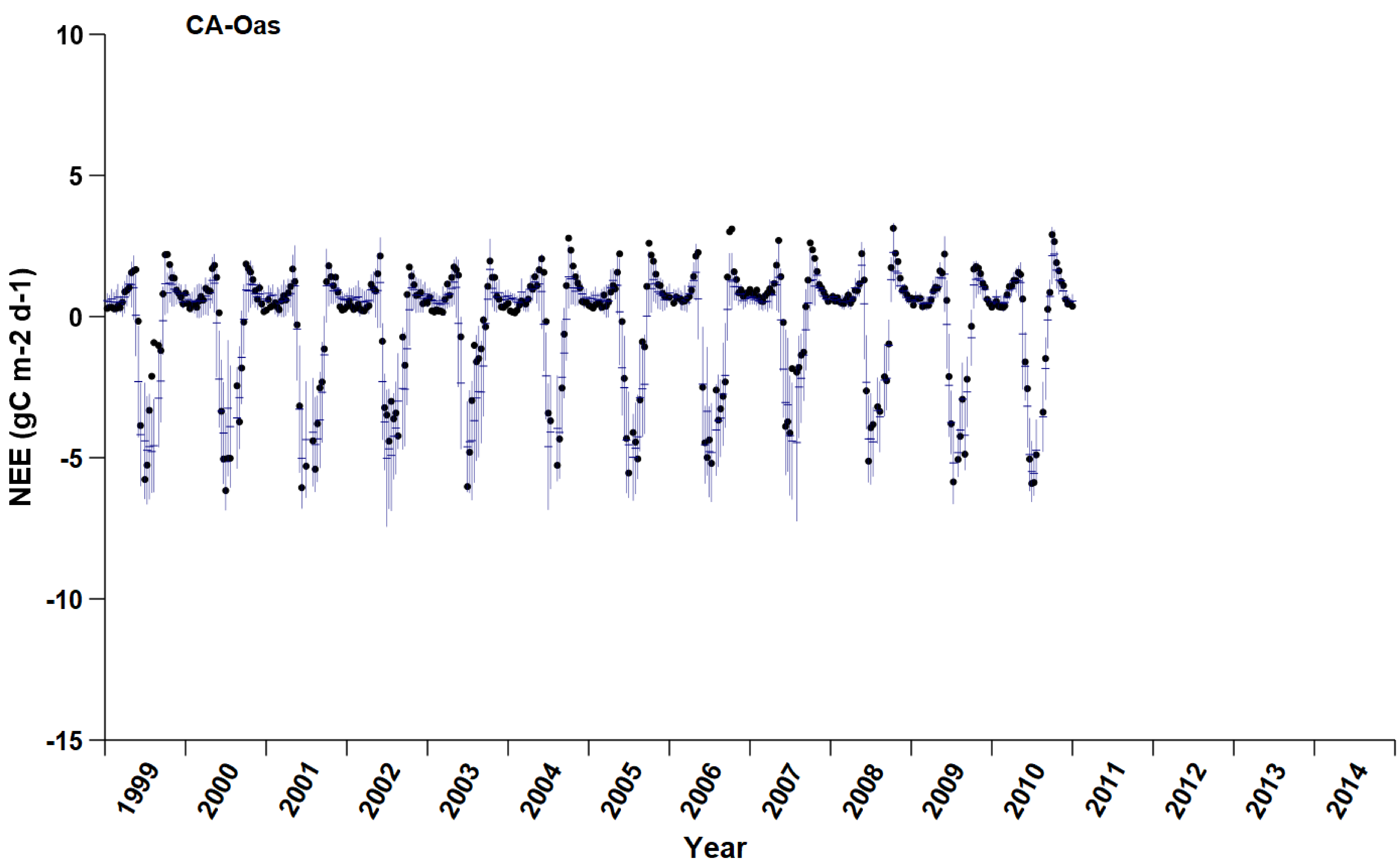
CA-Oas
NEE (gC m-2 d-1)
10
5
0
-5
-10
-15
1999
2000
2001
2002
2003
2004
2005
2006
2007
2008
2009
2010
2011
2012
2013
2014
Year

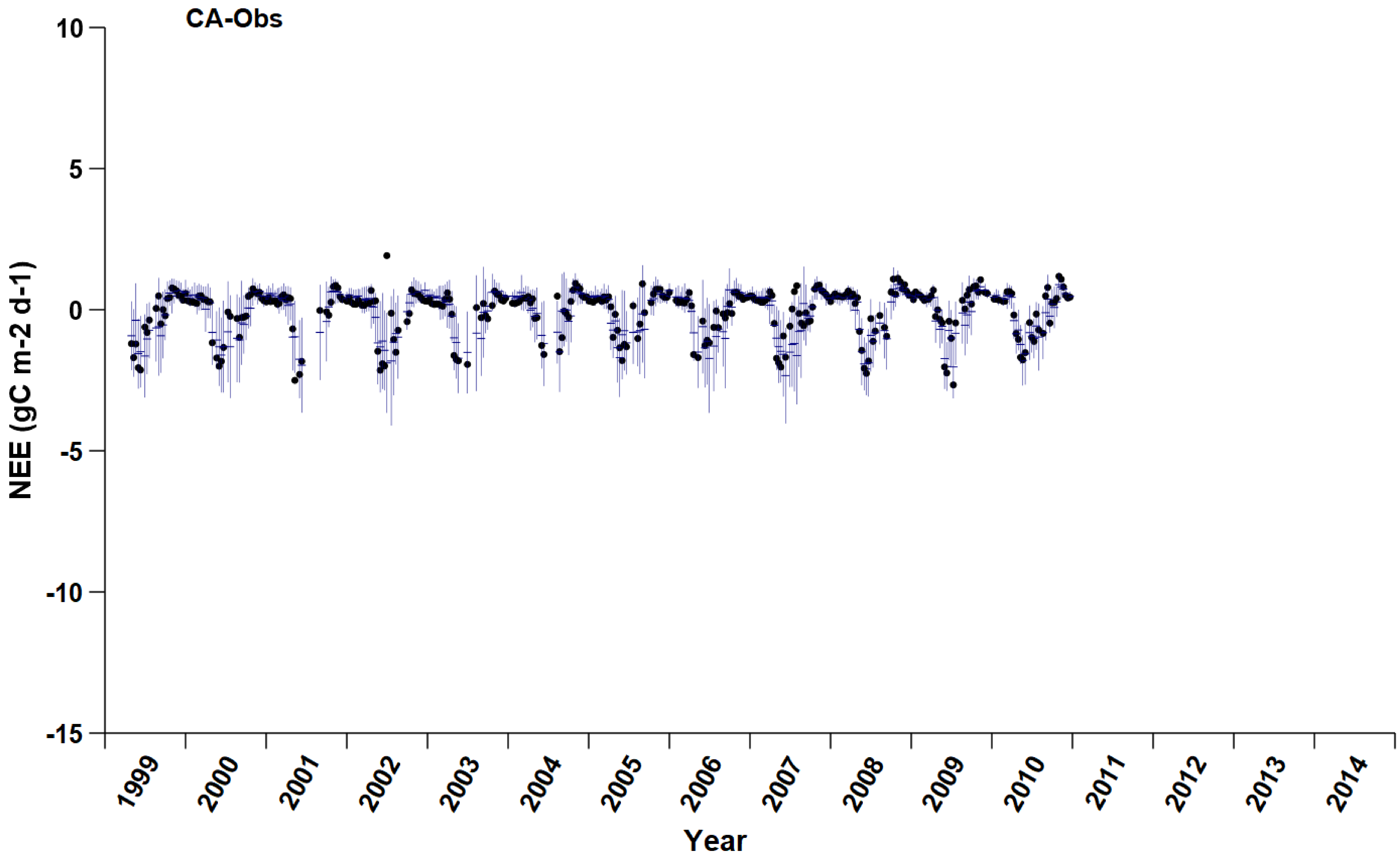

CA-Obs
10
5
0
-5
-10
-15
NEE (gC m-2 d-1)
1999
2000
2001
2002
2003
2004
2005
2006
2007
2008
2009
2010
2011
2012
2013
2014
Year

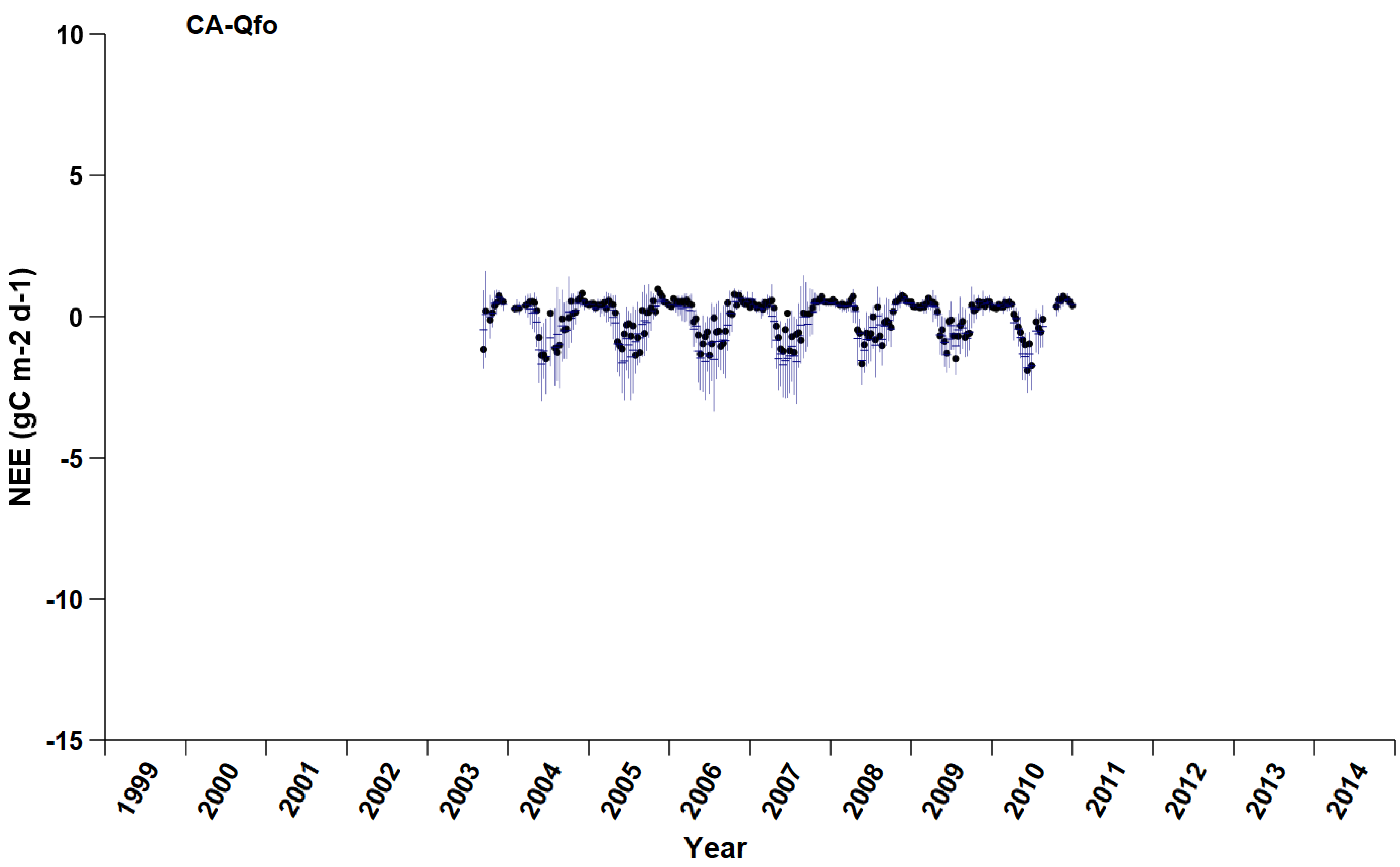
CA-Qfo
NEE (gC m-2 d-1)
10
5
0
-5
-10
-15
1999
2000
2001
2002
2003
2004
2005
2006
2007
2008
2009
2010
2011
2012
2013
2014
Year

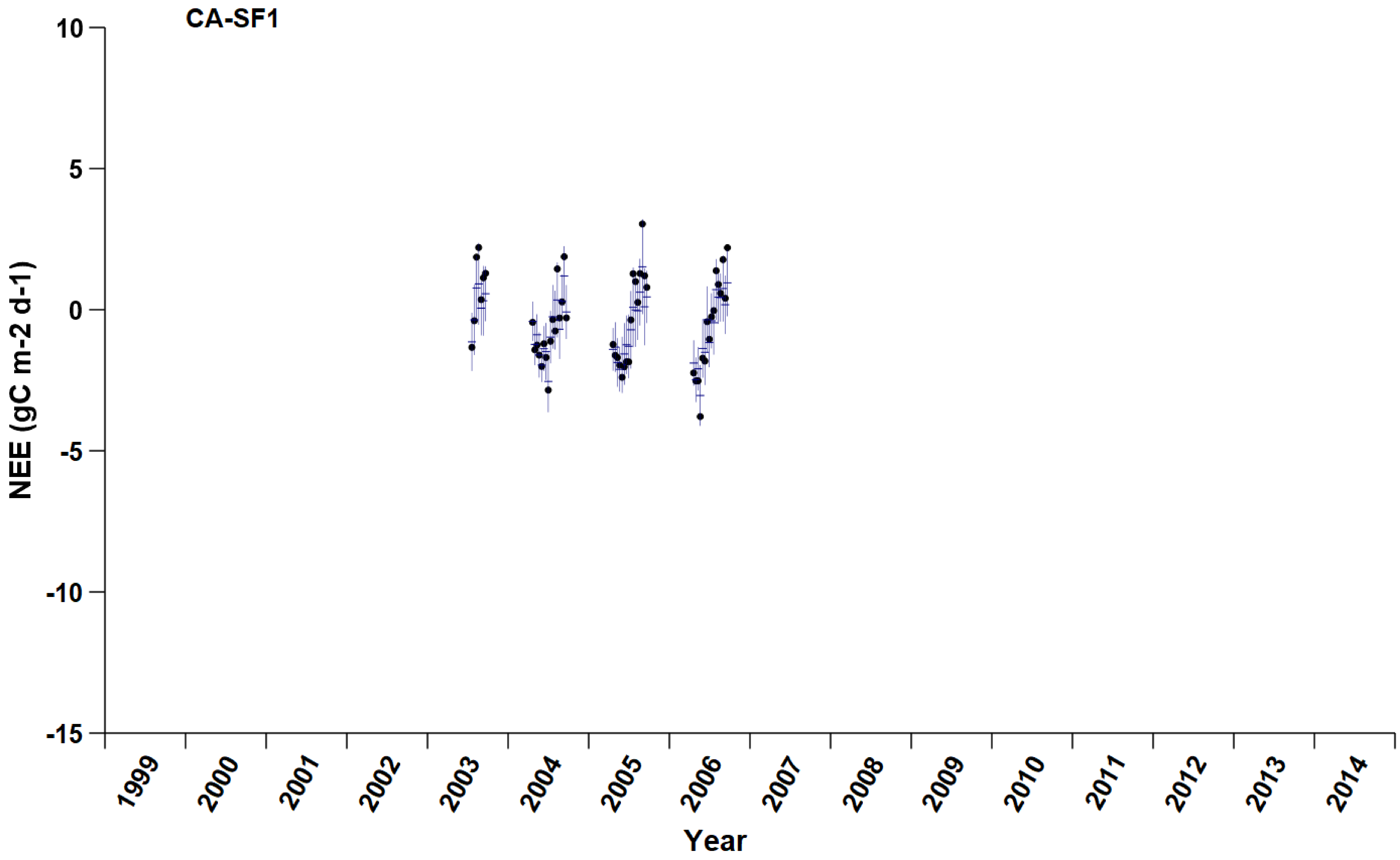

CA-SF1
10
5
0
-5
-10
-15
NEE (gC m-2 d-1)
1999
2000
2001
2002
2003
2004
2005
2006
2007
2008
2009
2010
2011
2012
2013
2014
Year

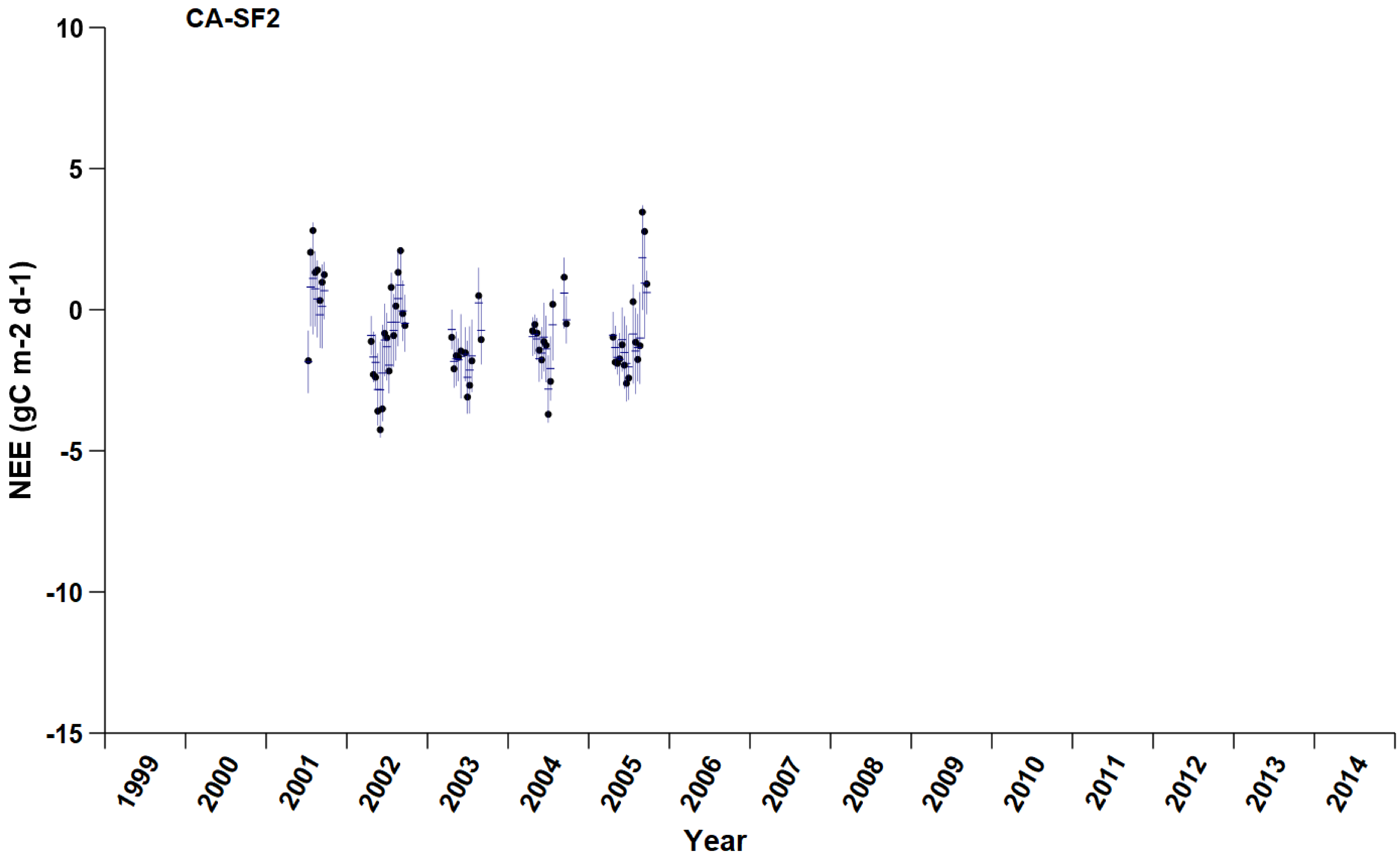

CA-SF2
10
5
0
-5
-10
-15
NEE (gC m-2 d-1)
1999
2000
2001
2002
2003
2004
2005
2006
2007
2008
2009
2010
2011
2012
2013
2014
Year

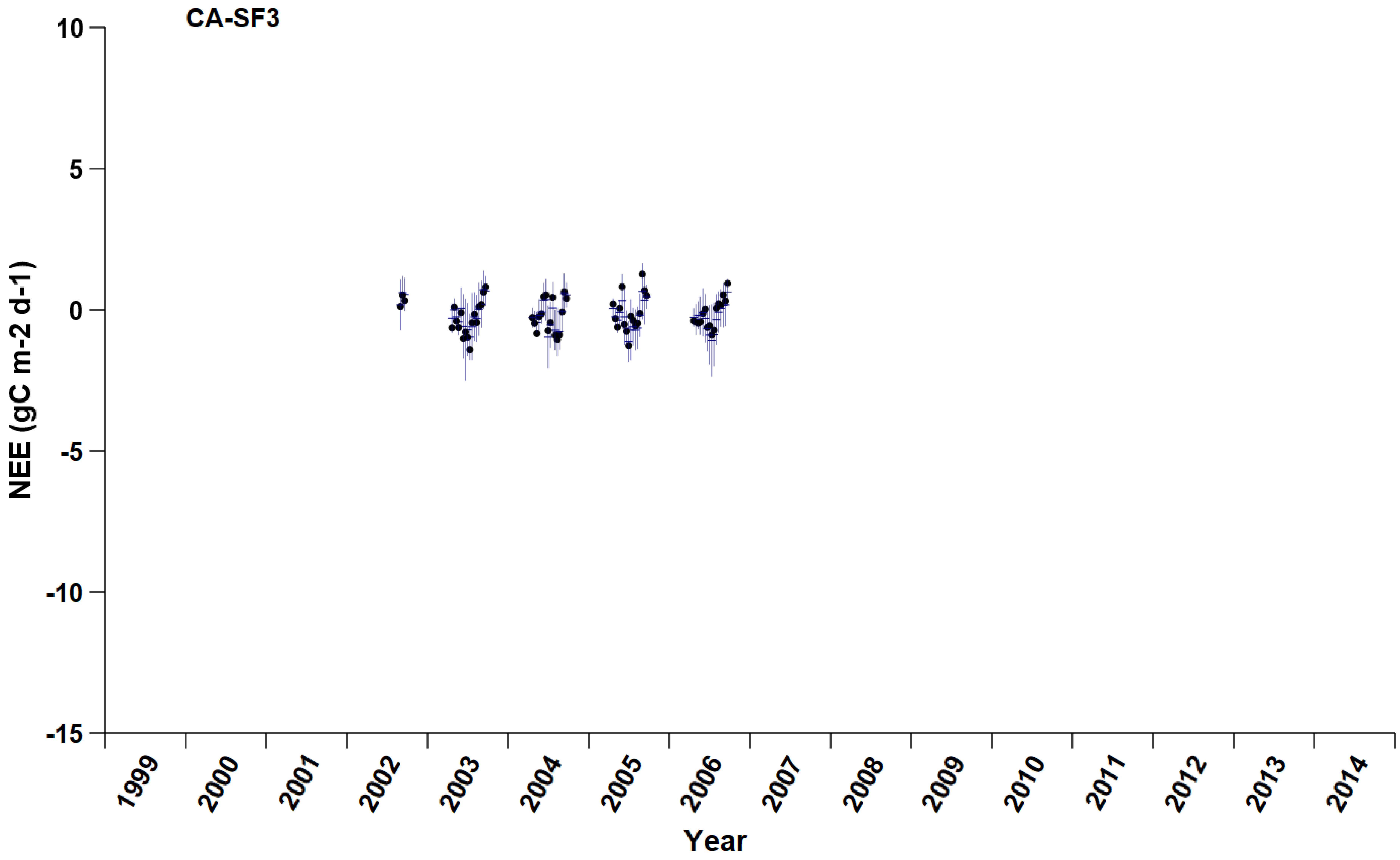
CA-SF3
10
5
0
-5
-10
-15
NEE (gC m-2 d-1)
1999
2000
2001
2002
2003
2004
2005
2006
2007
2008
2009
2010
2011
2012
2013
2014
Year

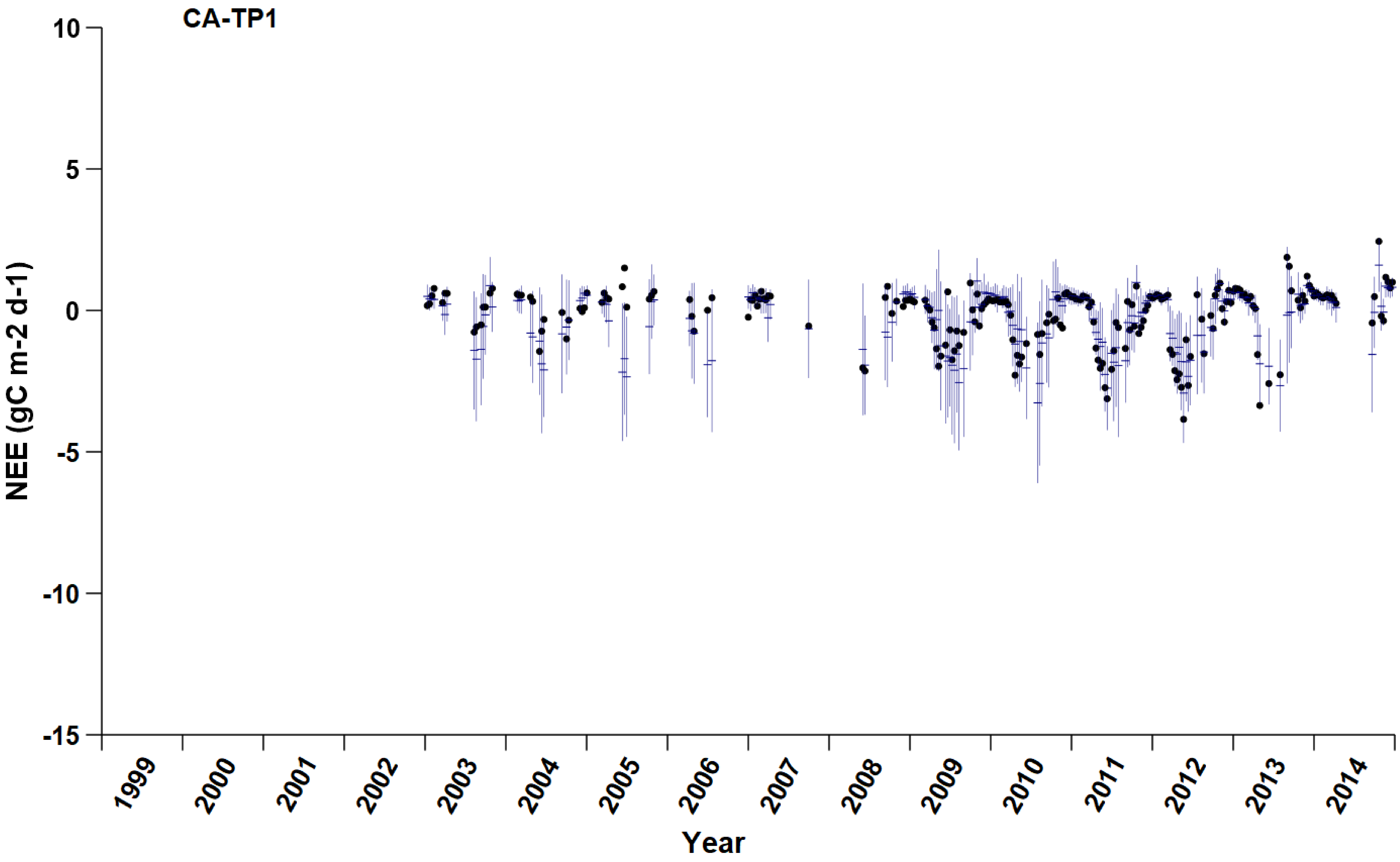

CA-TP1
NEE (gC m-2 d-1)
10
5
0
-5
-10
-15
1999
2000
2001
2002
2003
2004
2005
2006
2007
2008
2009
2010
2011
2012
2013
2014
Year

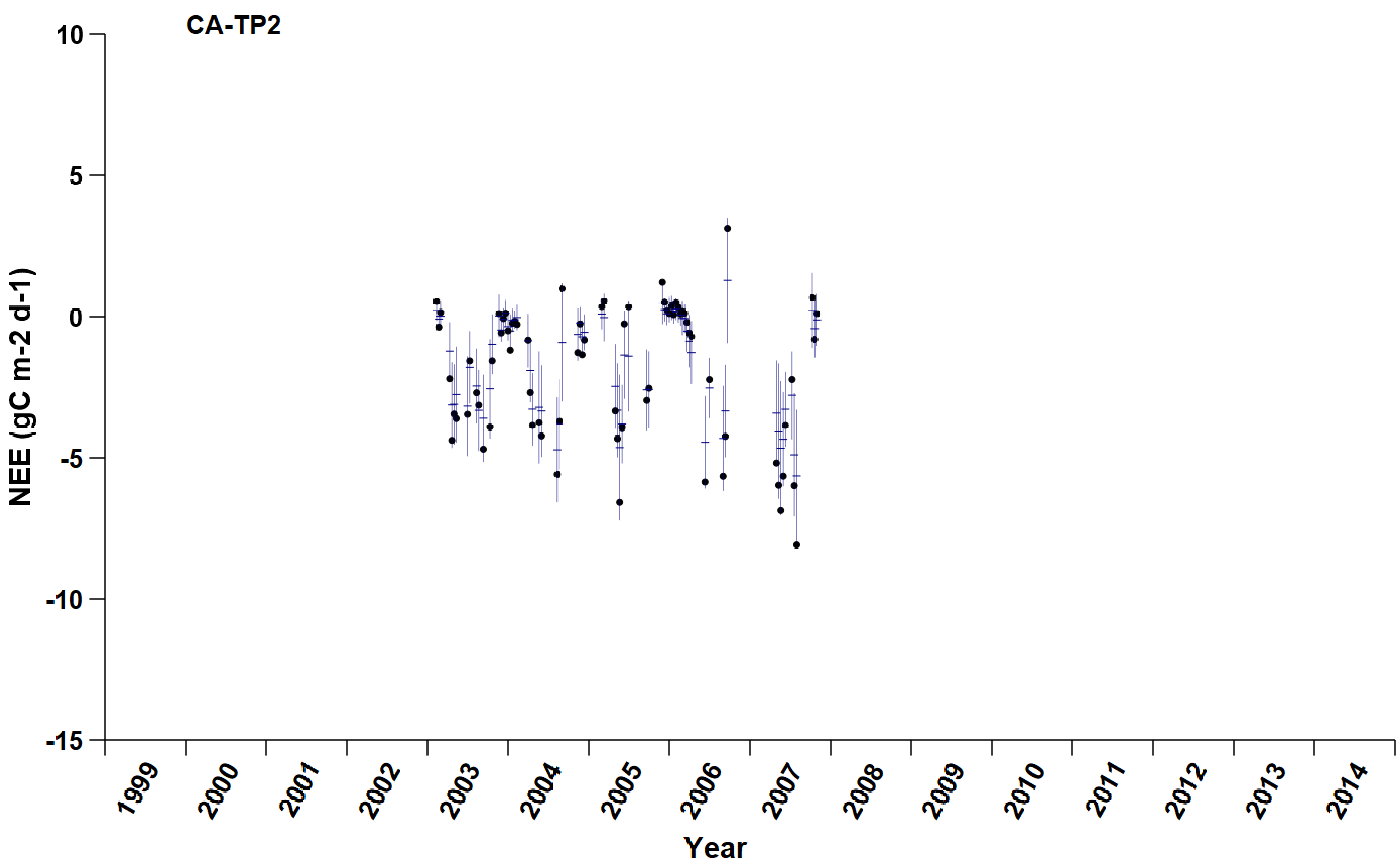
CA-TP2
NEE (gC m-2 d-1)
10
5
0
-5
-10
-15
1999
2000
2001
2002
2003
2004
2005
2006
2007
2008
2009
2010
2011
2012
2013
2014
Year

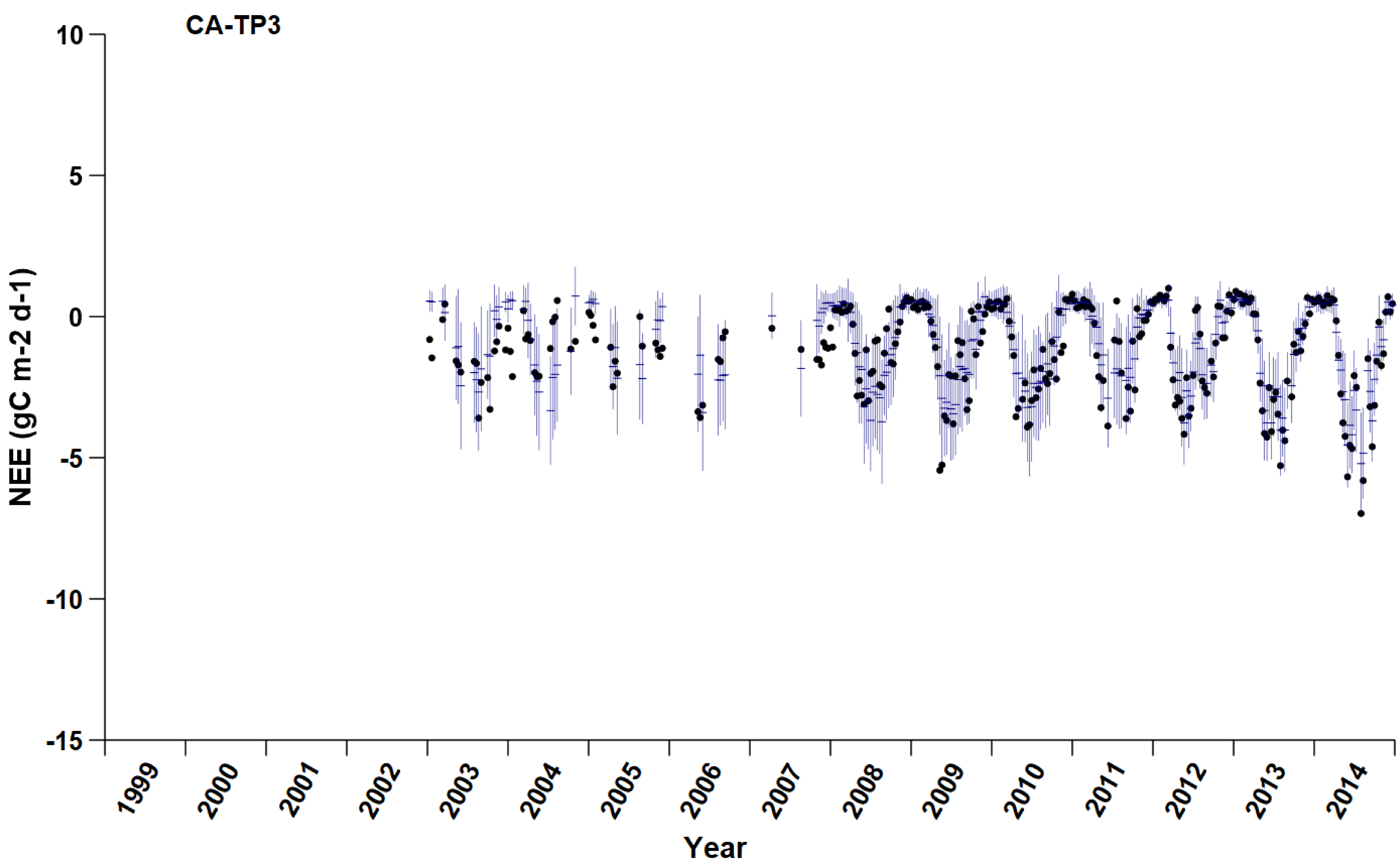

CA-TP3
10
5
0
-5
-10
-15
NEE (gC m-2 d-1)
1999
2000
2001
2002
2003
2004
2005
2006
2007
2008
2009
2010
2011
2012
2013
2014
Year

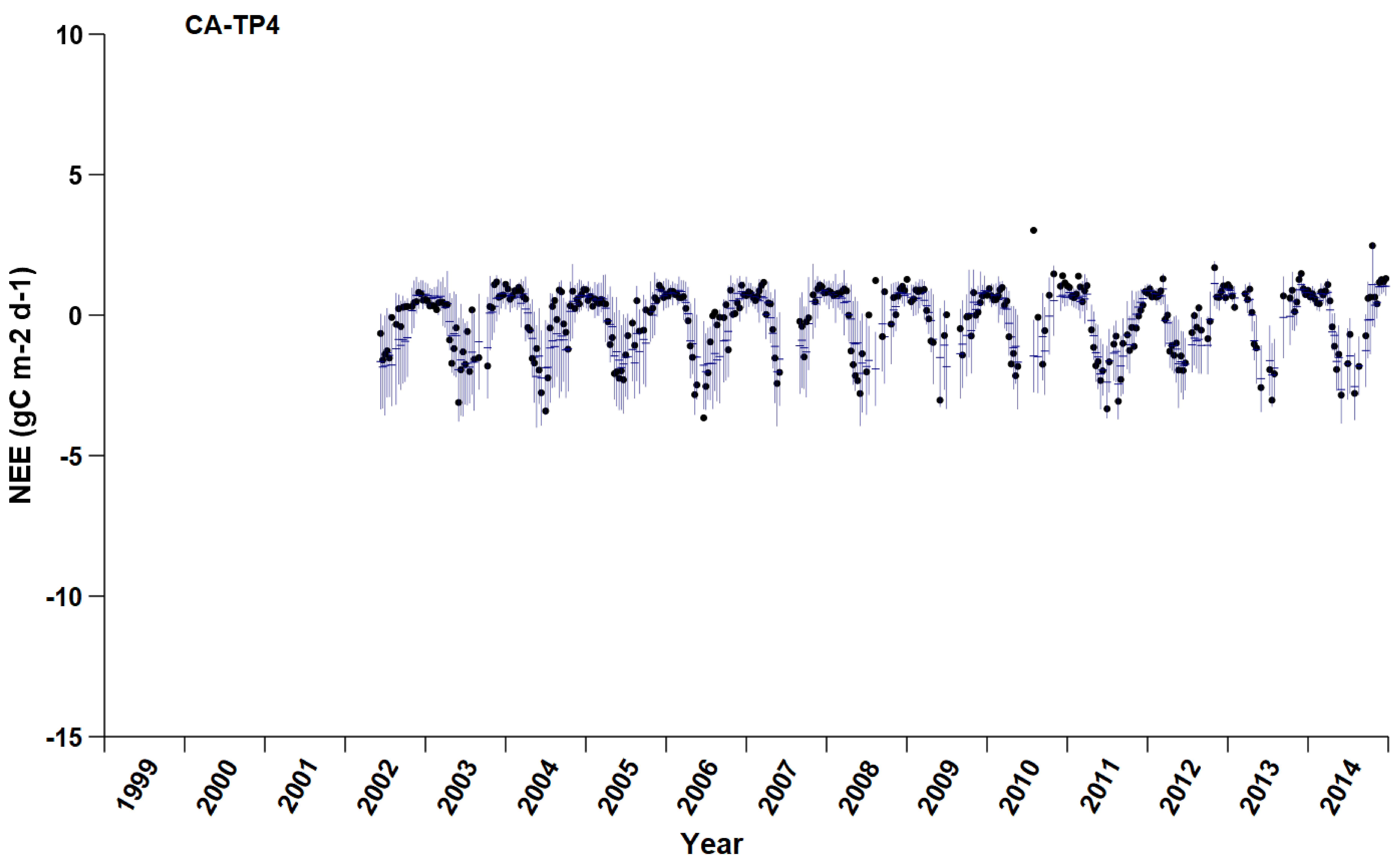

CA-TP4
NEE (gC m-2 d-1)
10
5
0
-5
-10
-15
1999
2000
2001
2002
2003
2004
2005
2006
2007
2008
2009
2010
2011
2012
2013
2014
Year

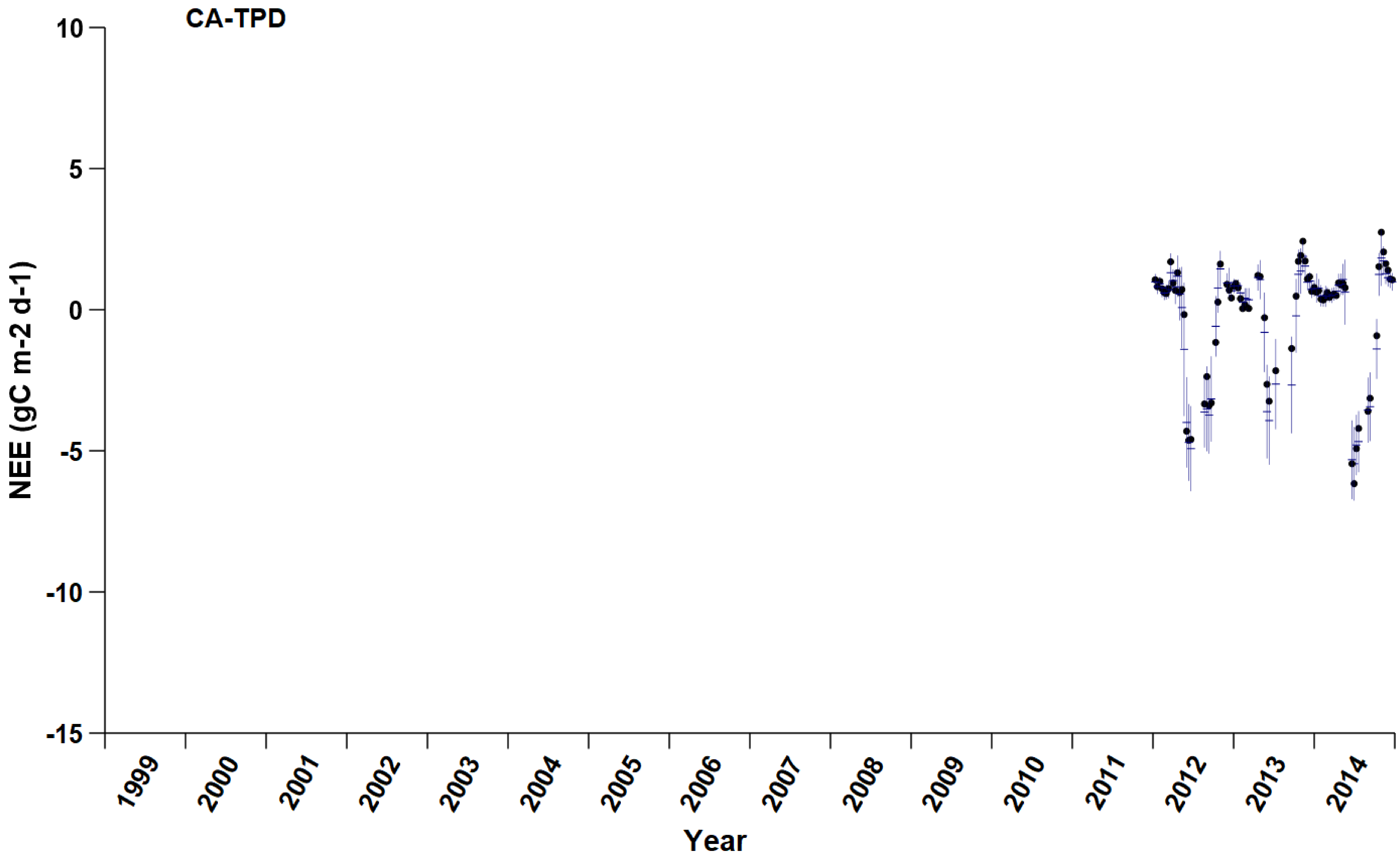
CA-TPD
NEE (gC m-2 d-1)
10
5
0
-5
-10
-15
1999
2000
2001
2002
2003
2004
2005
2006
2007
2008
2009
2010
2011
2012
2013
2014
Year

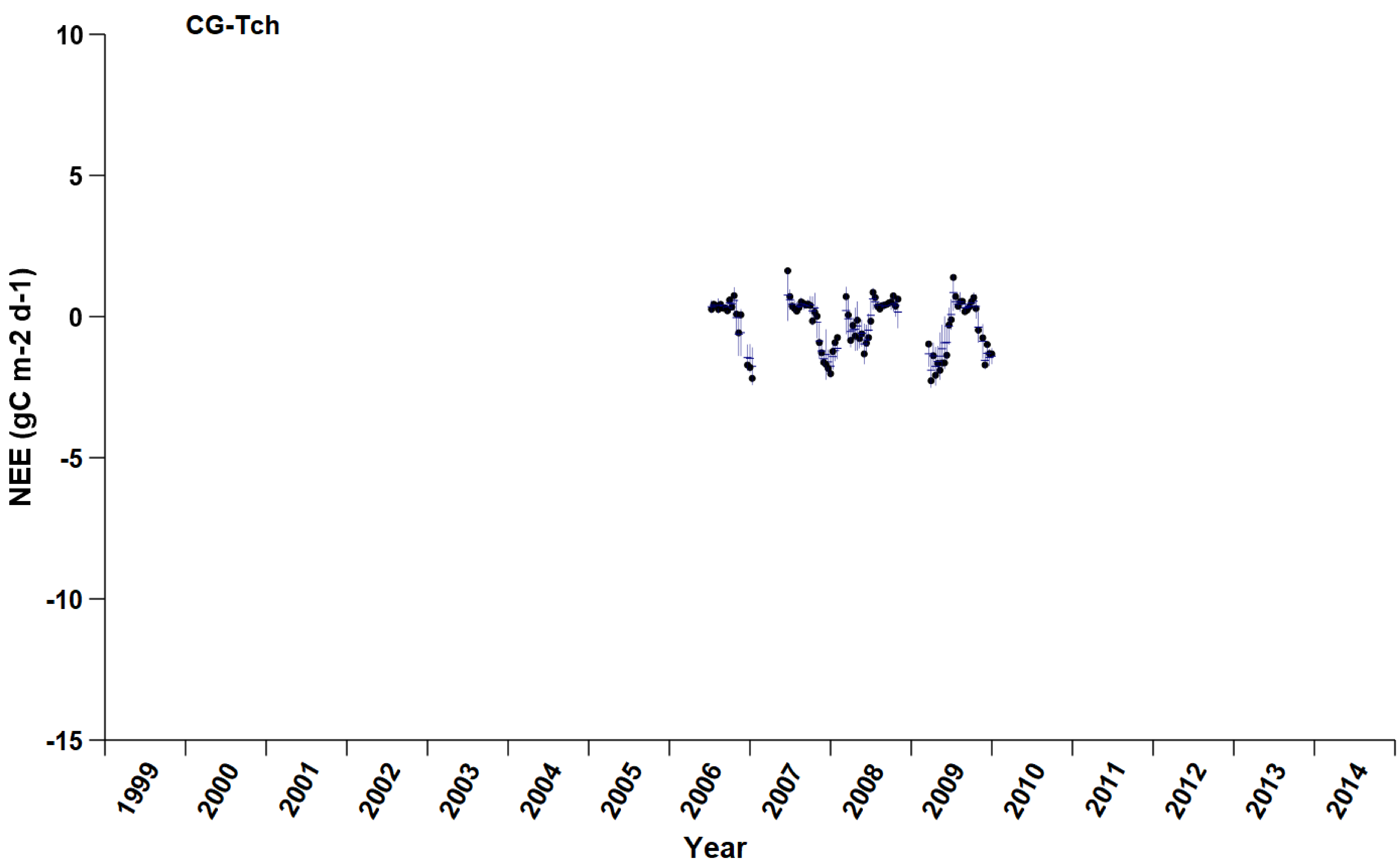
CG-Tch
NEE (gC m-2 d-1)
10
5
0
-5
-10
-15
1999
2000
2001
2002
2003
2004
2005
2006
2007
2008
2009
2010
2011
2012
2013
2014
Year

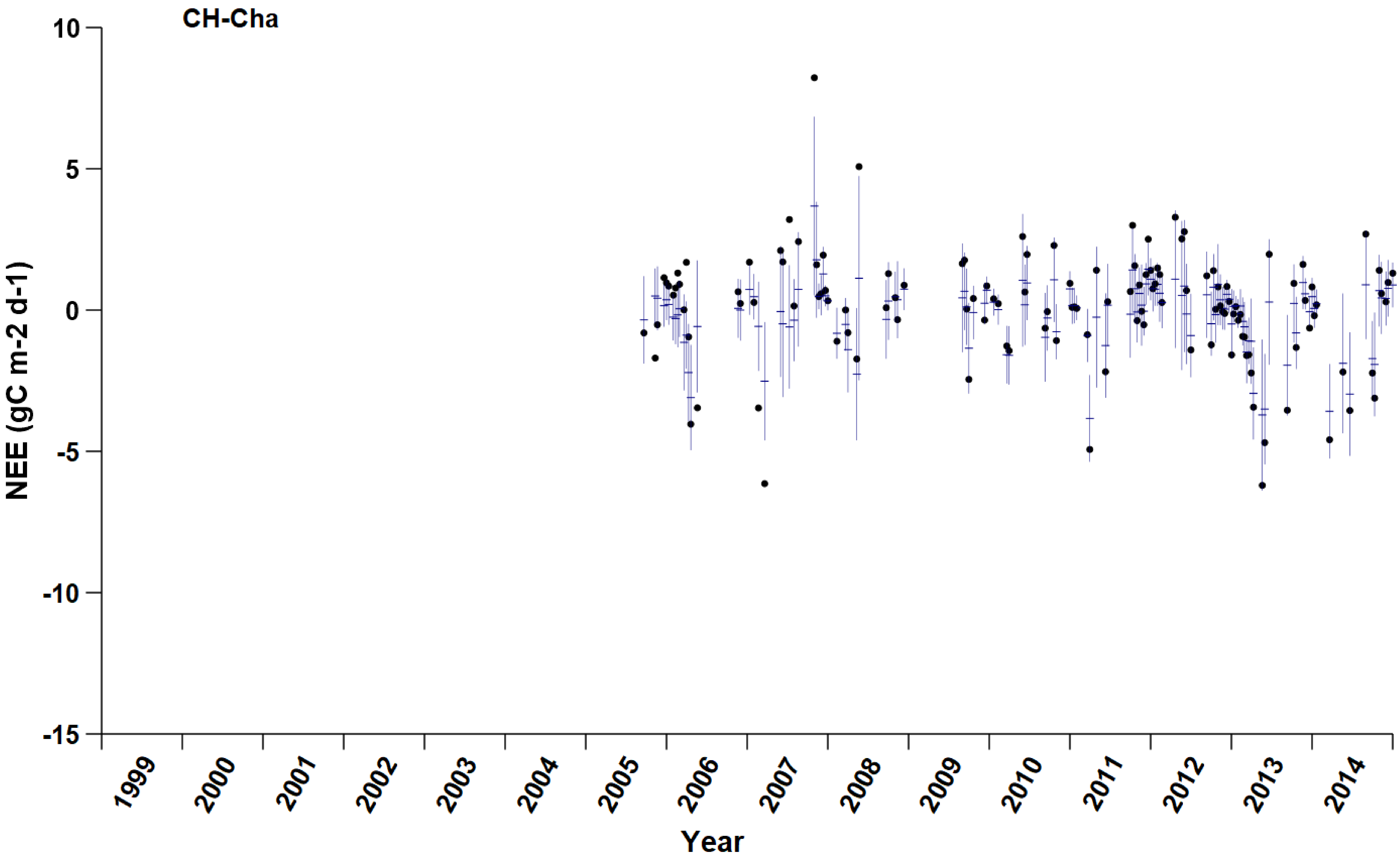
CH-Cha
NEE (gC m-2 d-1)
10
5
0
-5
-10
-15
1999
2000
2001
2002
2003
2004
2005
2006
2007
2008
2009
2010
2011
2012
2013
2014
Year

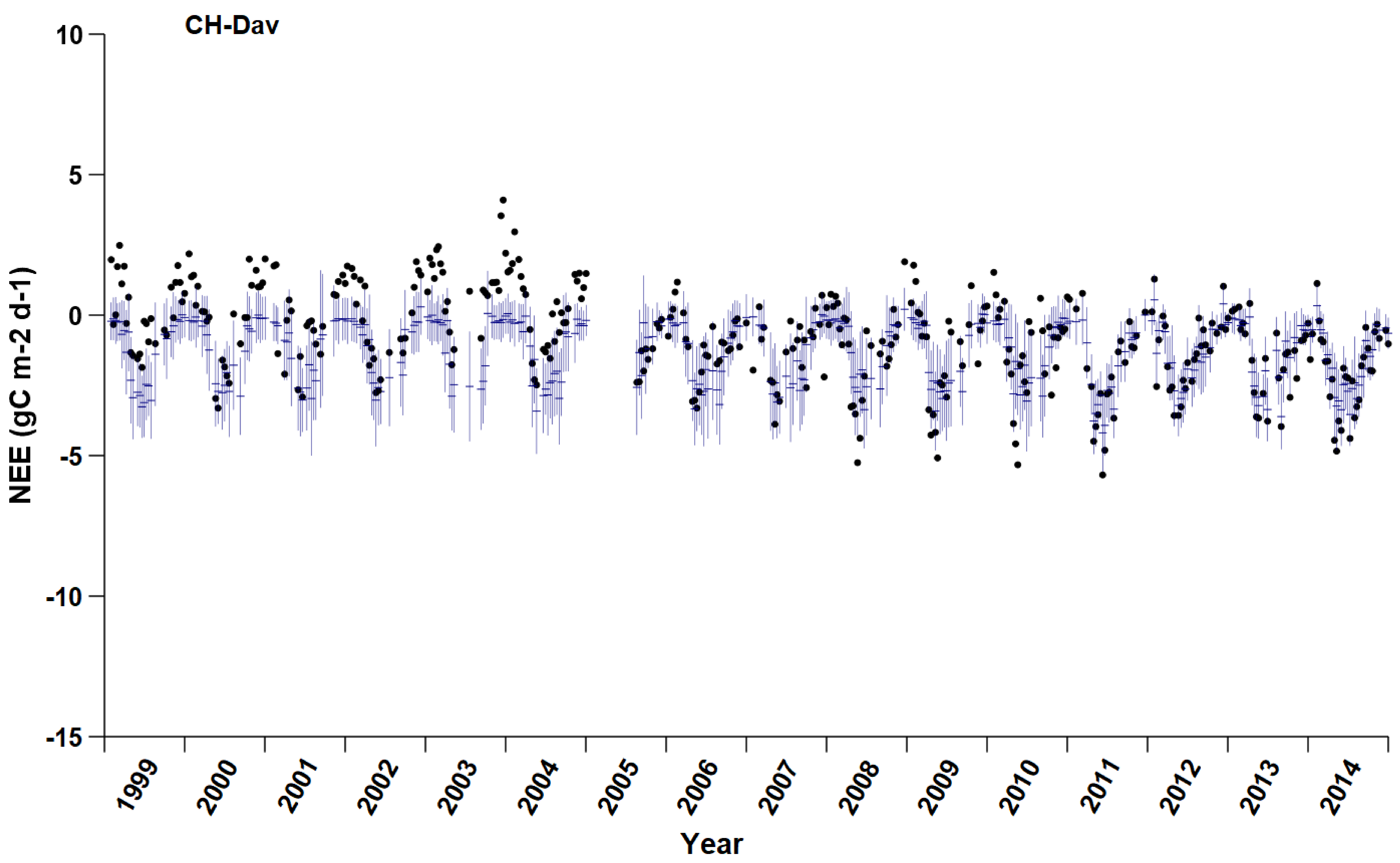
CH-Dav
NEE (gC m-2 d-1)
10
5
0
-5
-10
-15
1999
2000
2001
2002
2003
2004
2005
2006
2007
2008
2009
2010
2011
2012
2013
2014
Year

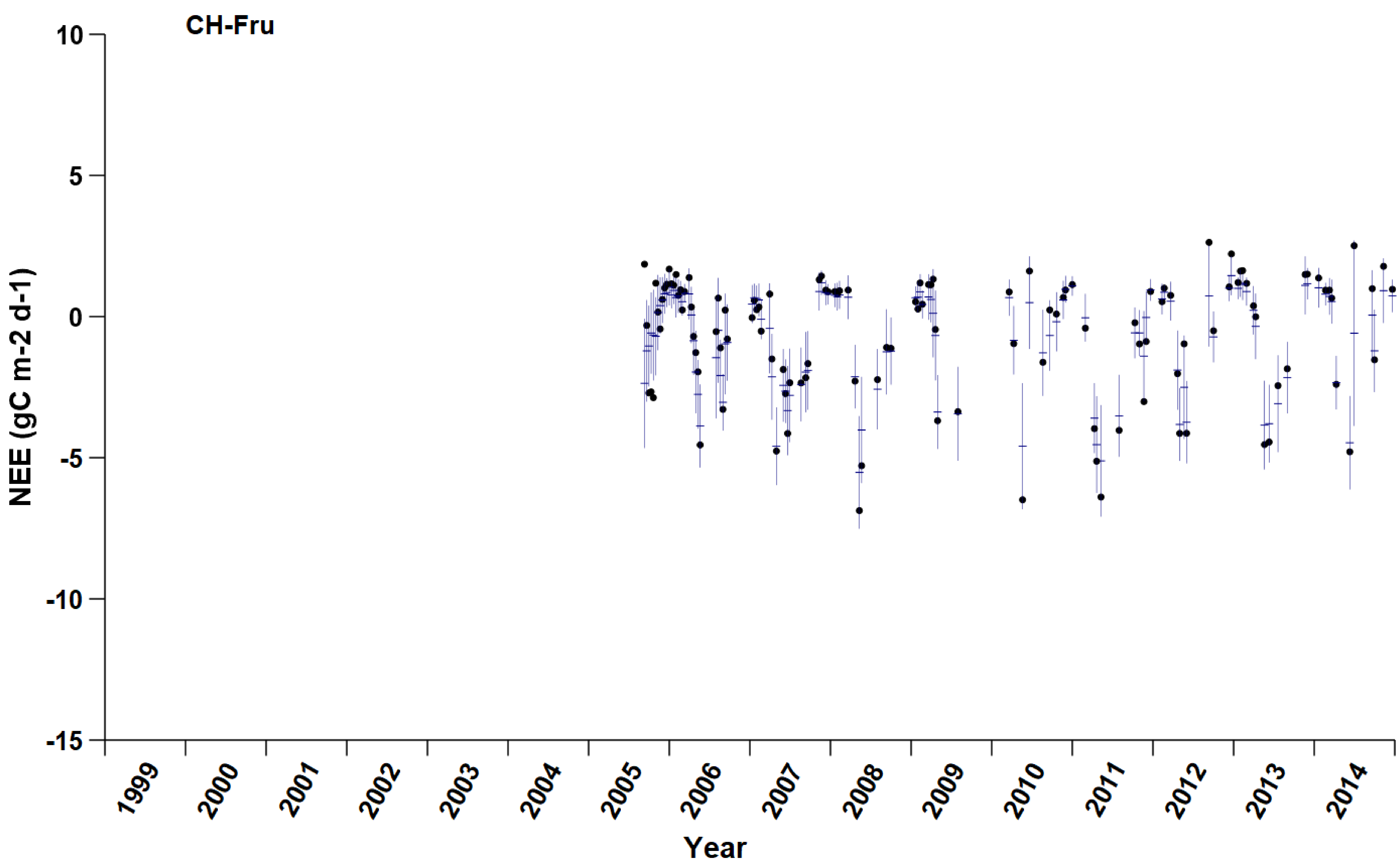

CH-Fru
NEE (gC m-2 d-1)
10
5
0
-5
-10
-15
1999
2000
2001
2002
2003
2004
2005
2006
2007
2008
2009
2010
2011
2012
2013
2014
Year

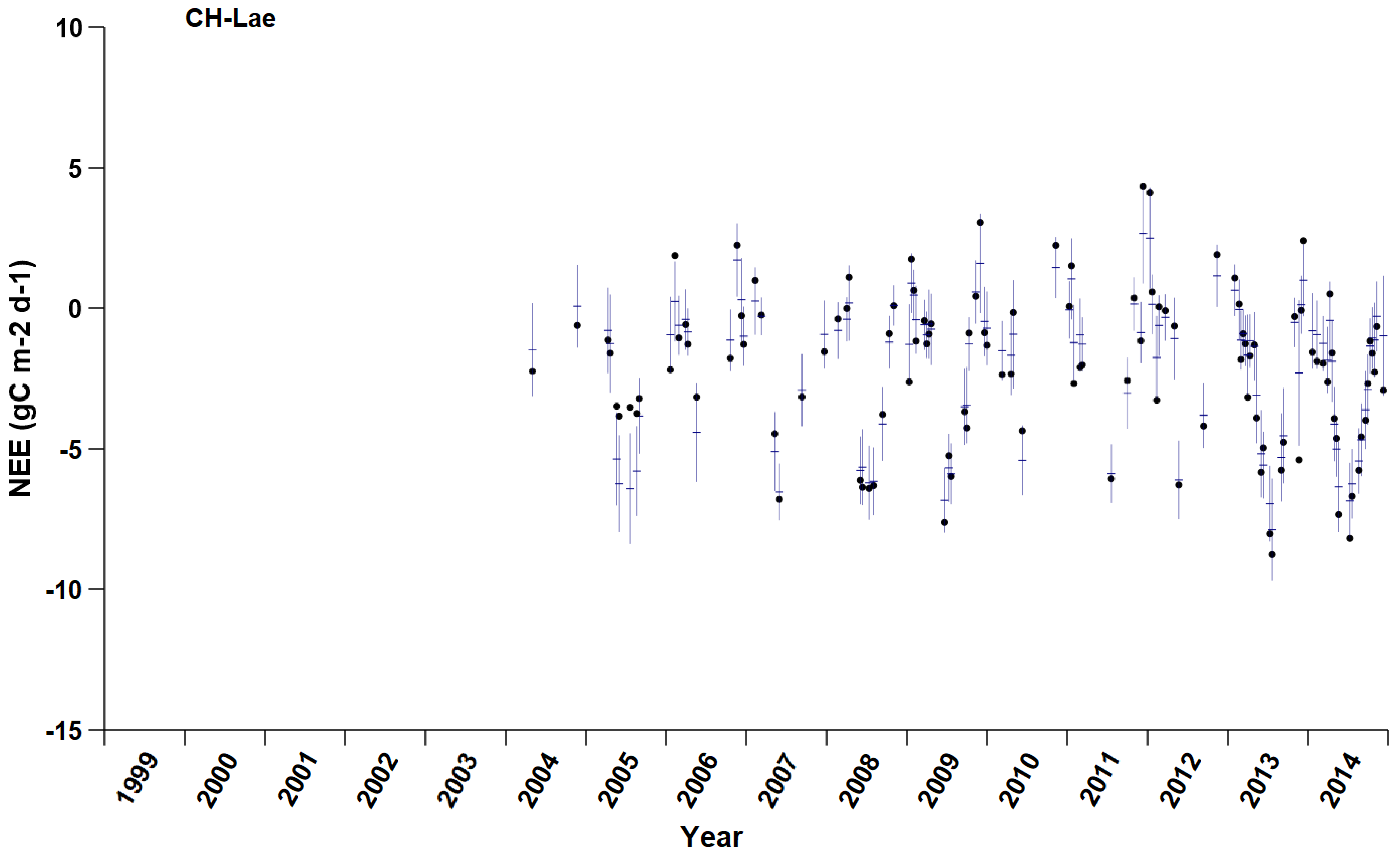
CH-Lae
NEE (gC m-2 d-1)
10
5
0
-5
-10
-15
1999
2000
2001
2002
2003
2004
2005
2006
2007
2008
2009
2010
2011
2012
2013
2014
Year

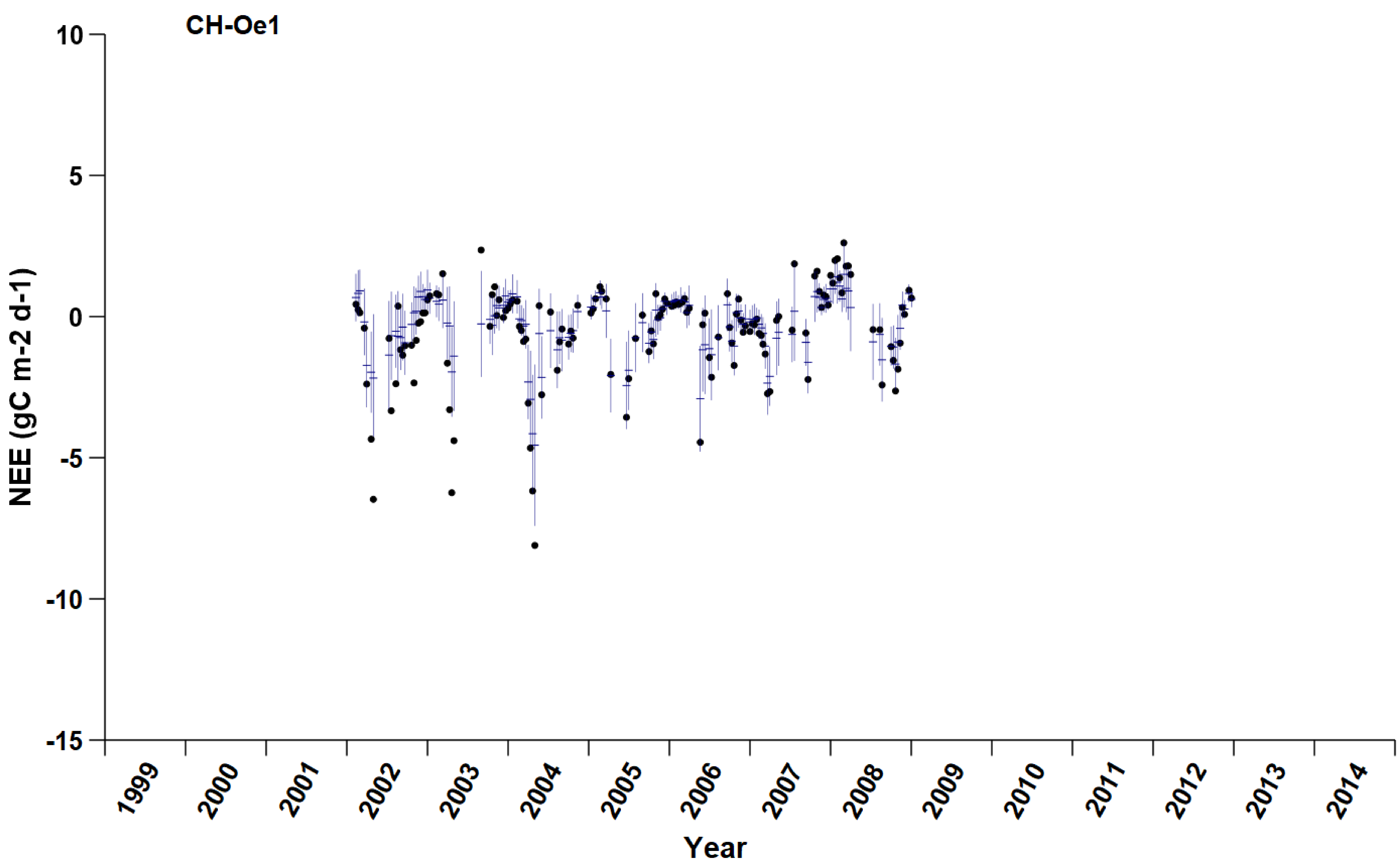
CH-Oe1
NEE (gC m-2 d-1)
10
5
0
-5
-10
-15
1999
2000
2001
2002
2003
2004
2005
2006
2007
2008
2009
2010
2011
2012
2013
2014
Year

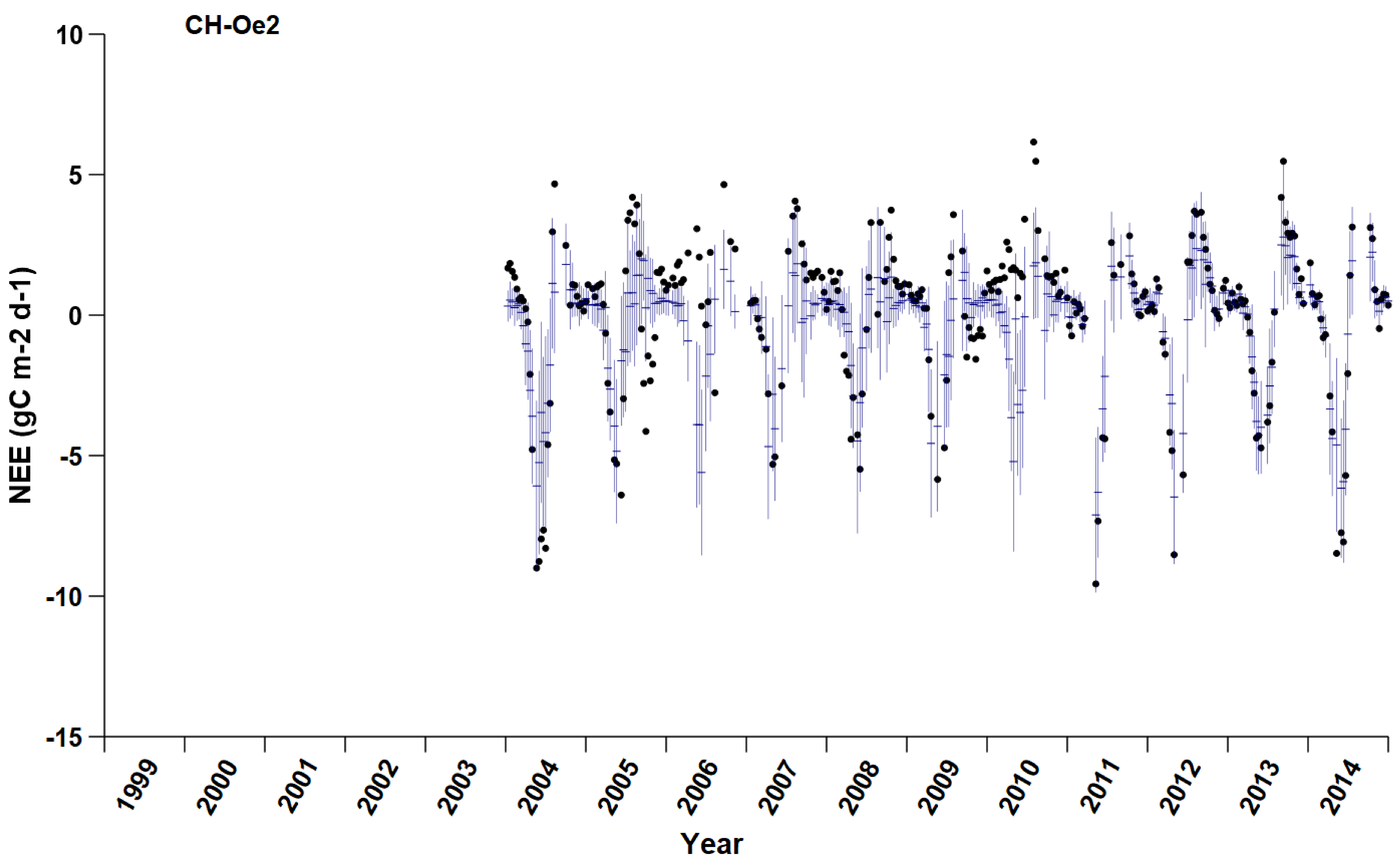

CH-Oe2
NEE (gC m-2 d-1)
10
5
0
-5
-10
-15
1999
2000
2001
2002
2003
2004
2005
2006
2007
2008
2009
2010
2011
2012
2013
2014
Year

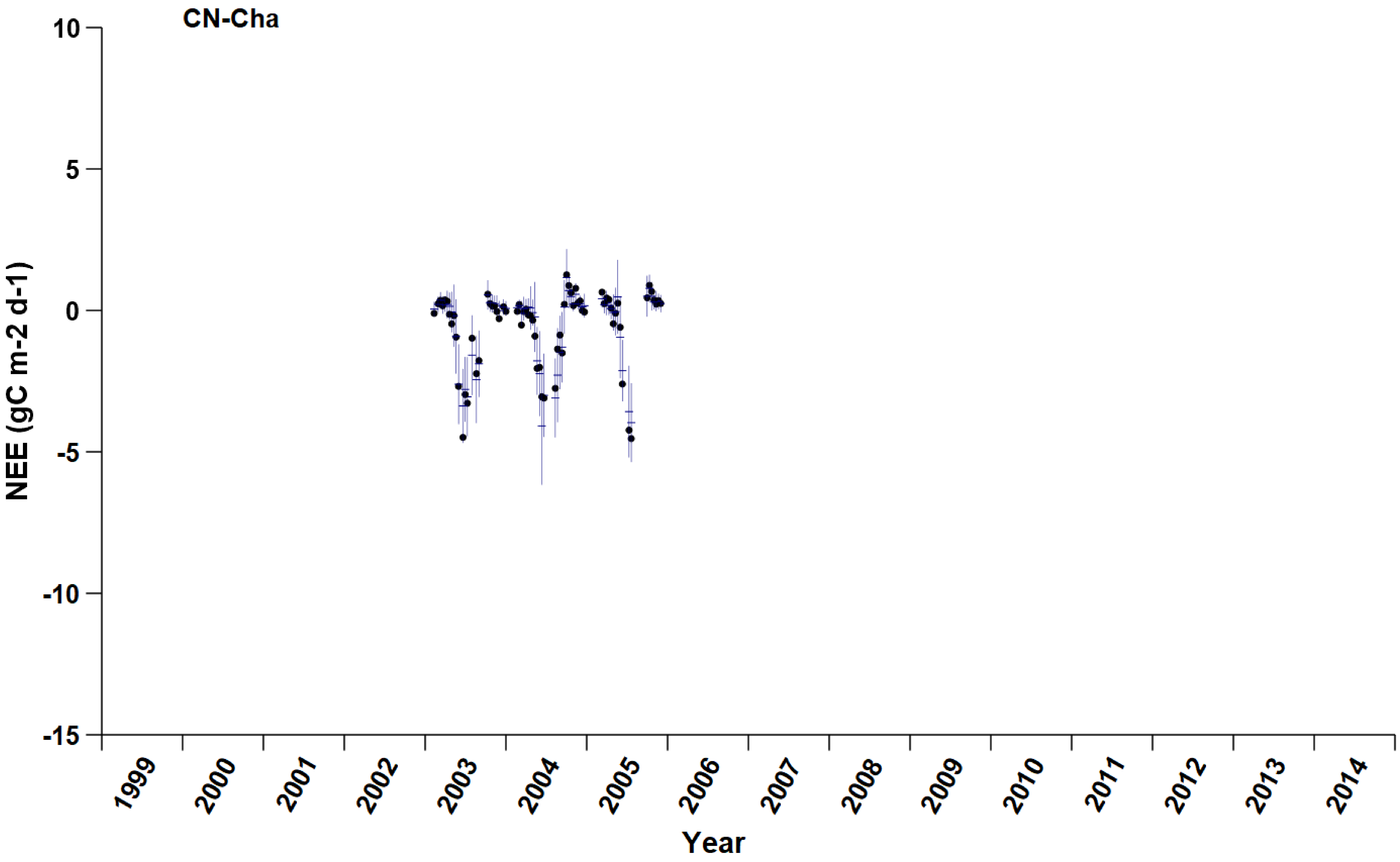

CN-Cha
NEE (gC m-2 d-1)
10
5
0
-5
-10
-15
1999
2000
2001
2002
2003
2004
2005
2006
2007
2008
2009
2010
2011
2012
2013
2014
Year

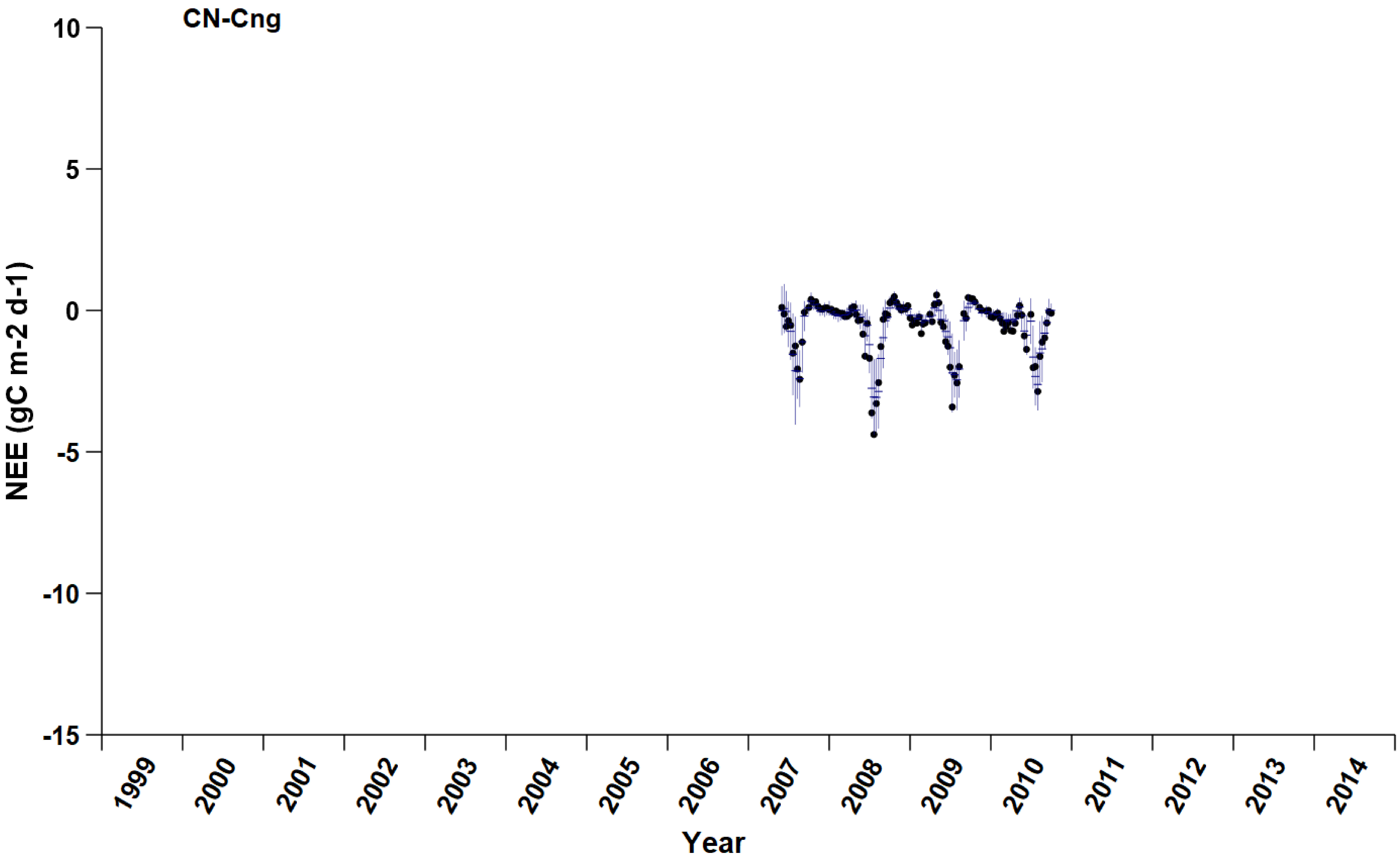

CN-Cng
10
5
0
-5
-10
-15
NEE (gC m-2 d-1)
1999
2000
2001
2002
2003
2004
2005
2006
2007
2008
2009
2010
2011
2012
2013
2014
Year

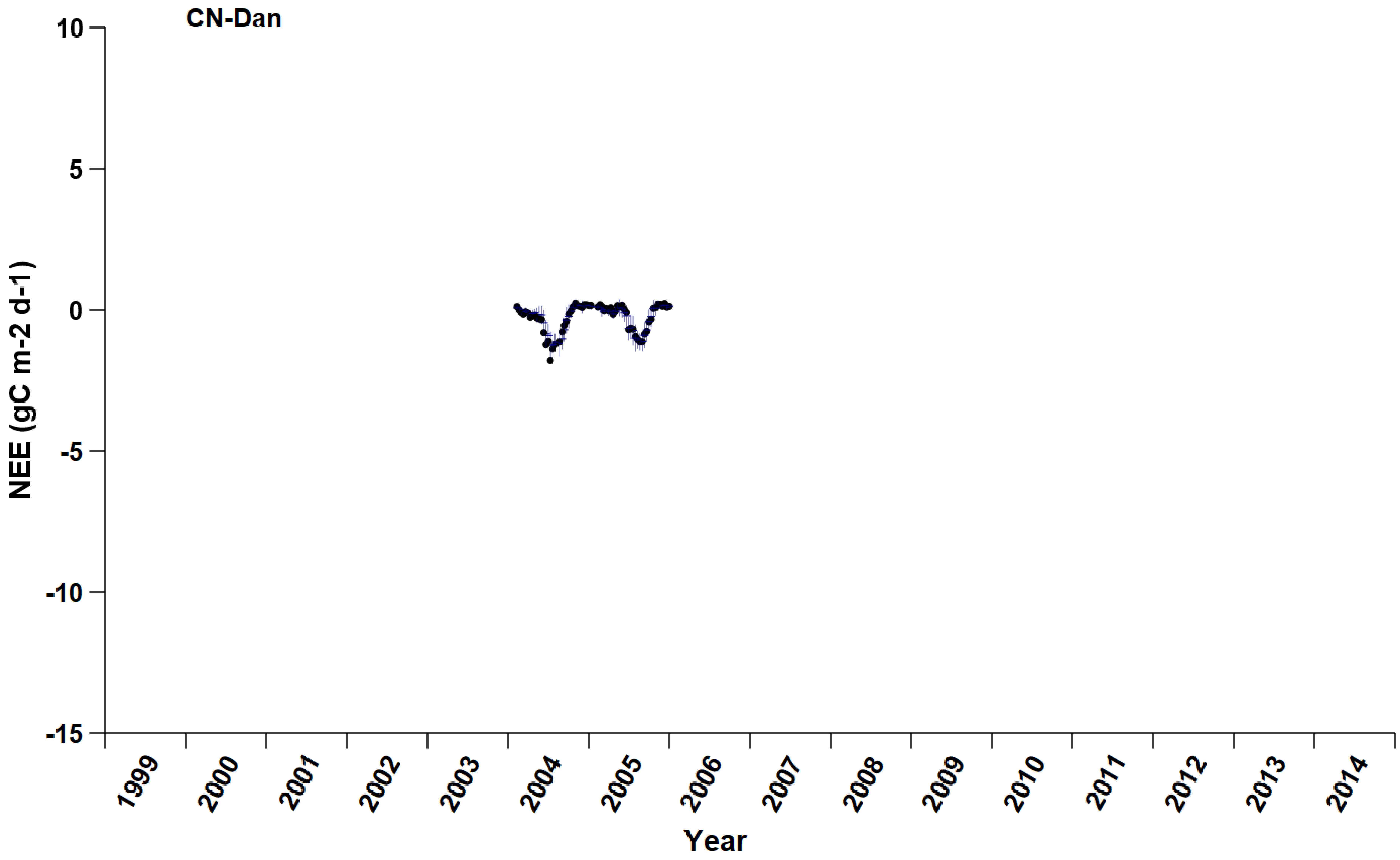

CN-Dan
NEE (gC m-2 d-1)
10
5
0
-5
-10
-15
1999
2000
2001
2002
2003
2004
2005
2006
2007
2008
2009
2010
2011
2012
2013
2014
Year

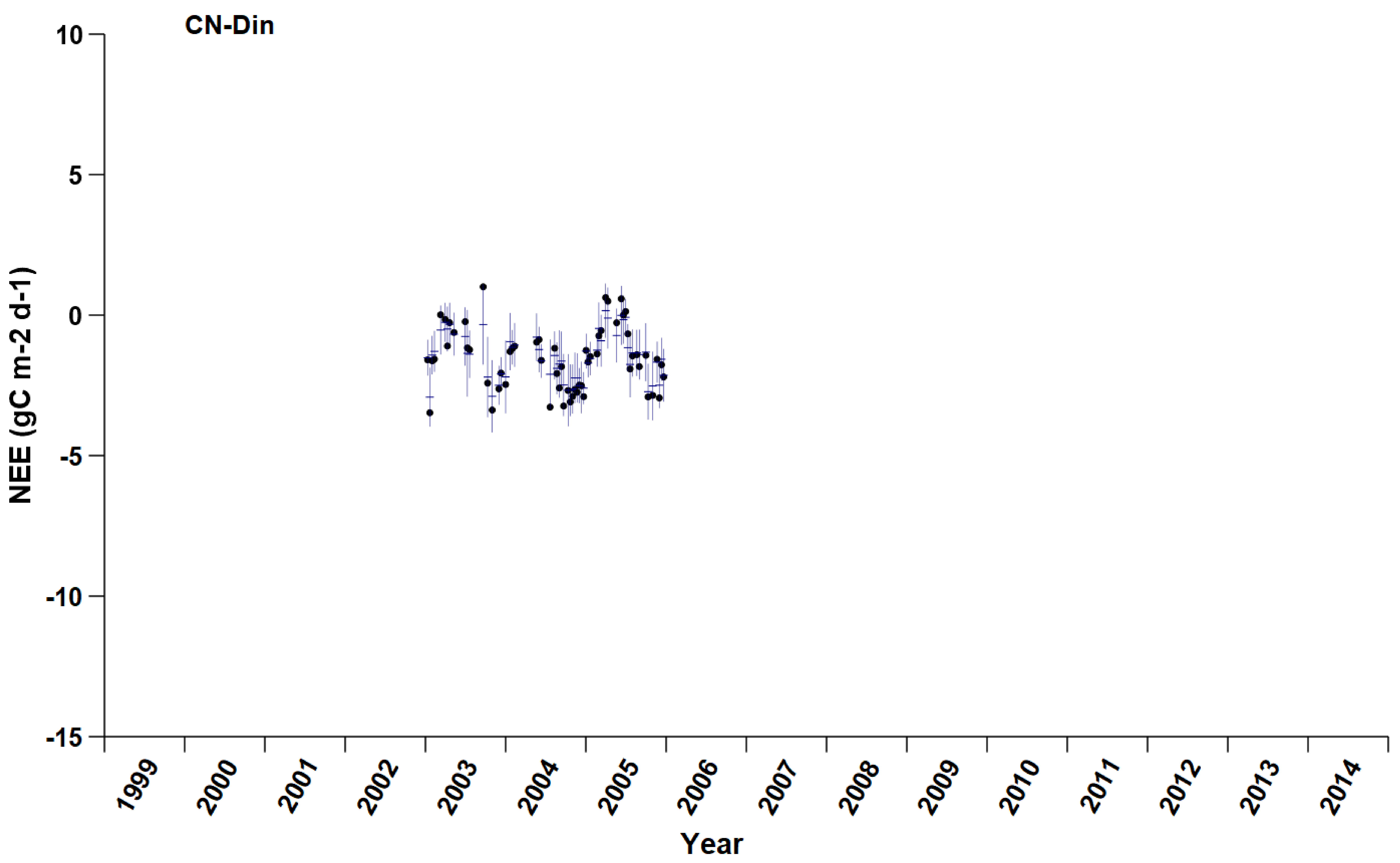
CN-Din
NEE (gC m-2 d-1)
10
5
0
-5
-10
-15
1999
2000
2001
2002
2003
2004
2005
2006
2007
2008
2009
2010
2011
2012
2013
2014
Year

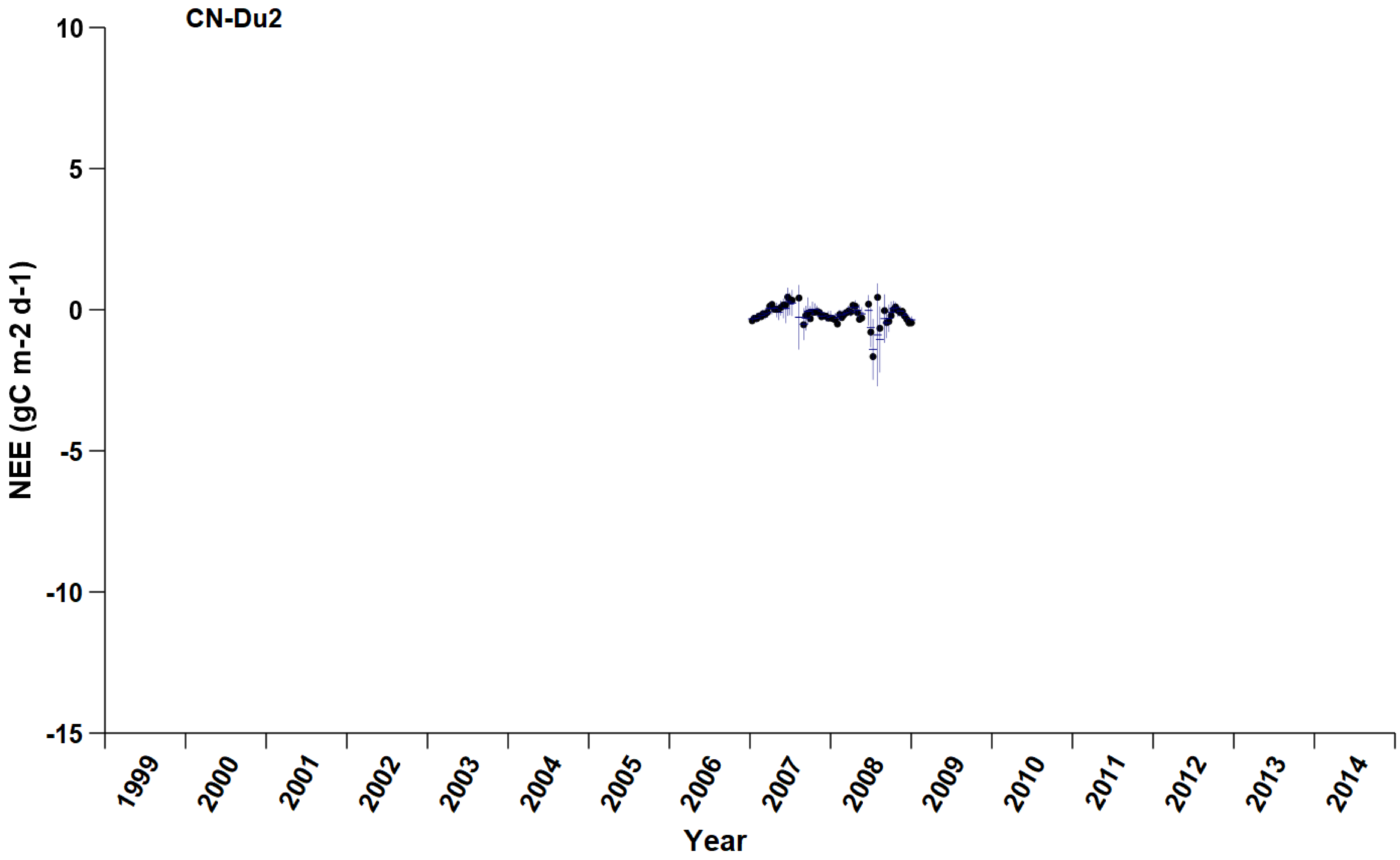

CN-Du2
NEE (gC m-2 d-1)
10
5
0
-5
-10
-15
1999
2000
2001
2002
2003
2004
2005
2006
2007
2008
2009
2010
2011
2012
2013
2014
Year

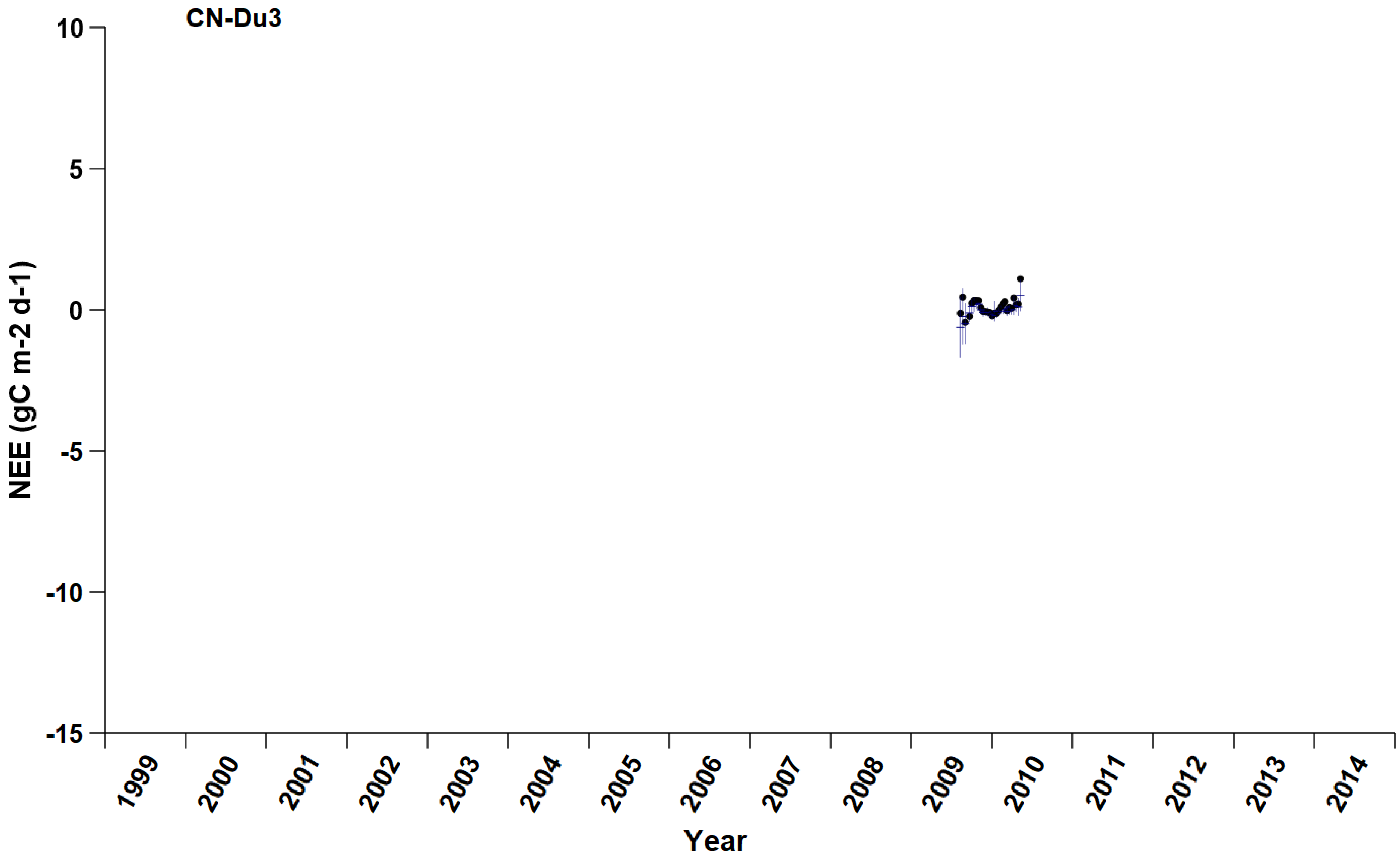

CN-Du3
NEE (gC m-2 d-1)
10
5
0
-5
-10
-15
1999
2000
2001
2002
2003
2004
2005
2006
2007
2008
2009
2010
2011
2012
2013
2014
Year

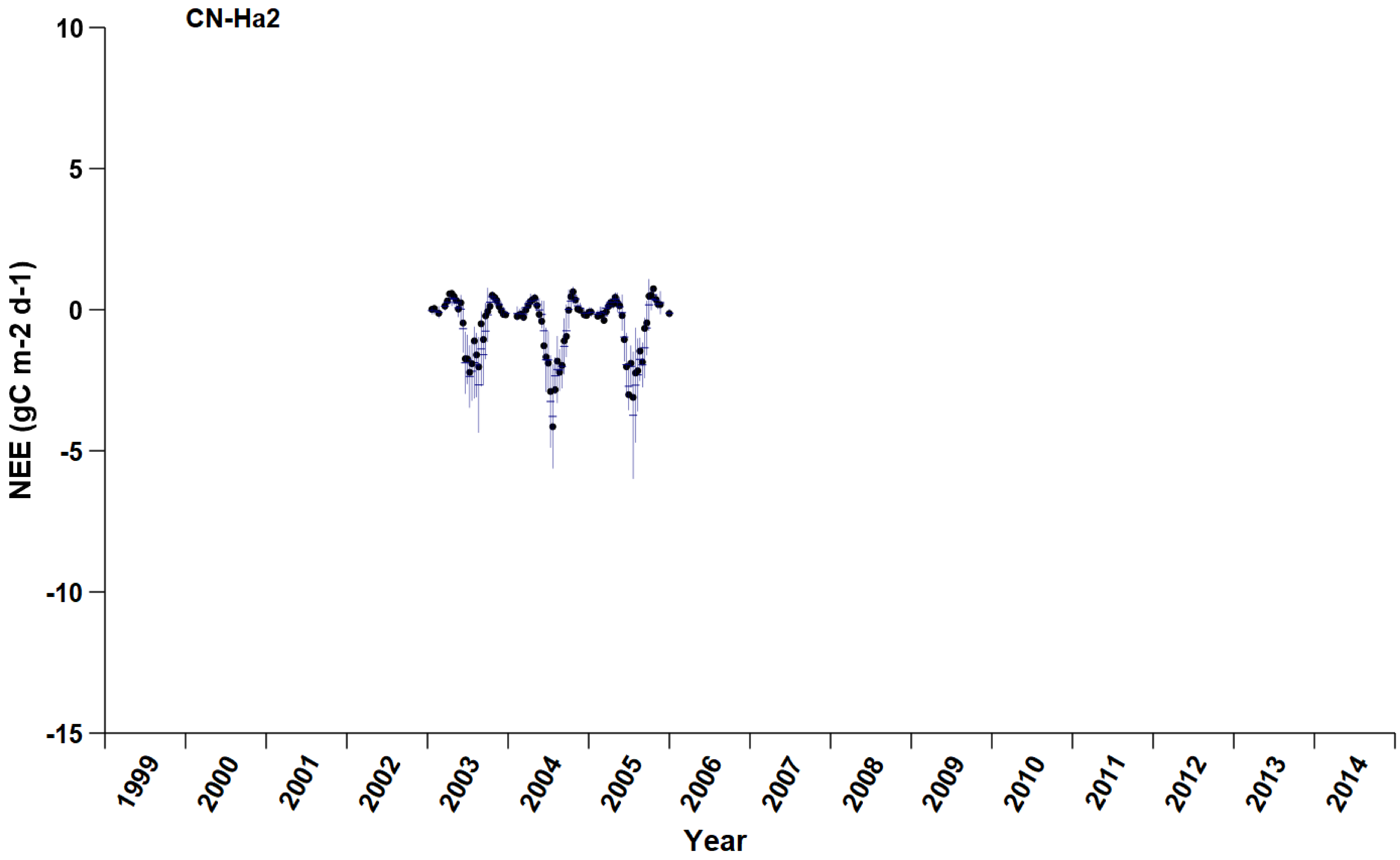

CN-Ha2
NEE (gC m-2 d-1)
10
5
0
-5
-10
-15
1999
2000
2001
2002
2003
2004
2005
2006
2007
2008
2009
2010
2011
2012
2013
2014
Year

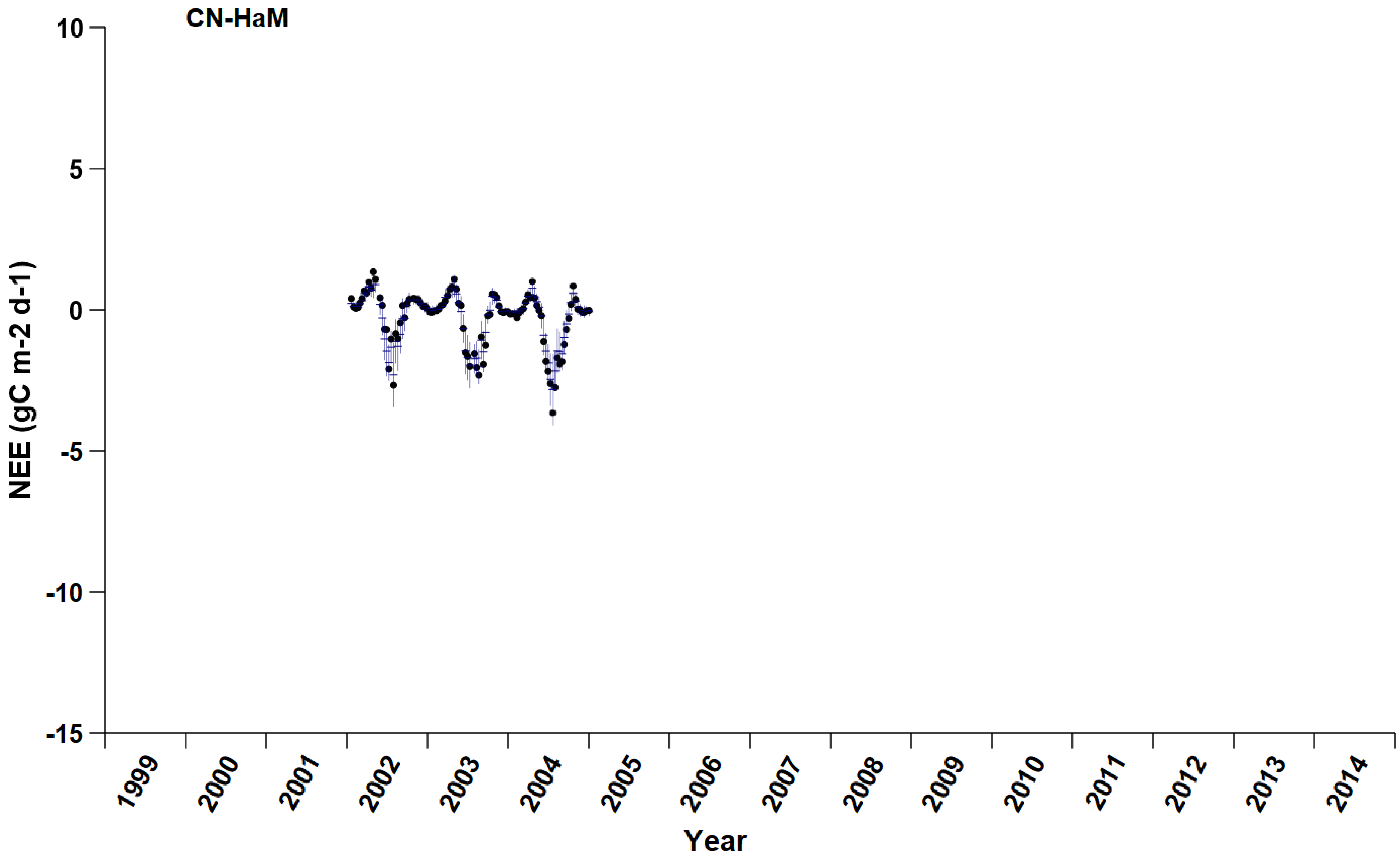

CN-HaM
10
5
0
-5
-10
-15
NEE (gC m-2 d-1)
1999
2000
2001
2002
2003
2004
2005
2006
2007
2008
2009
2010
2011
2012
2013
2014
Year

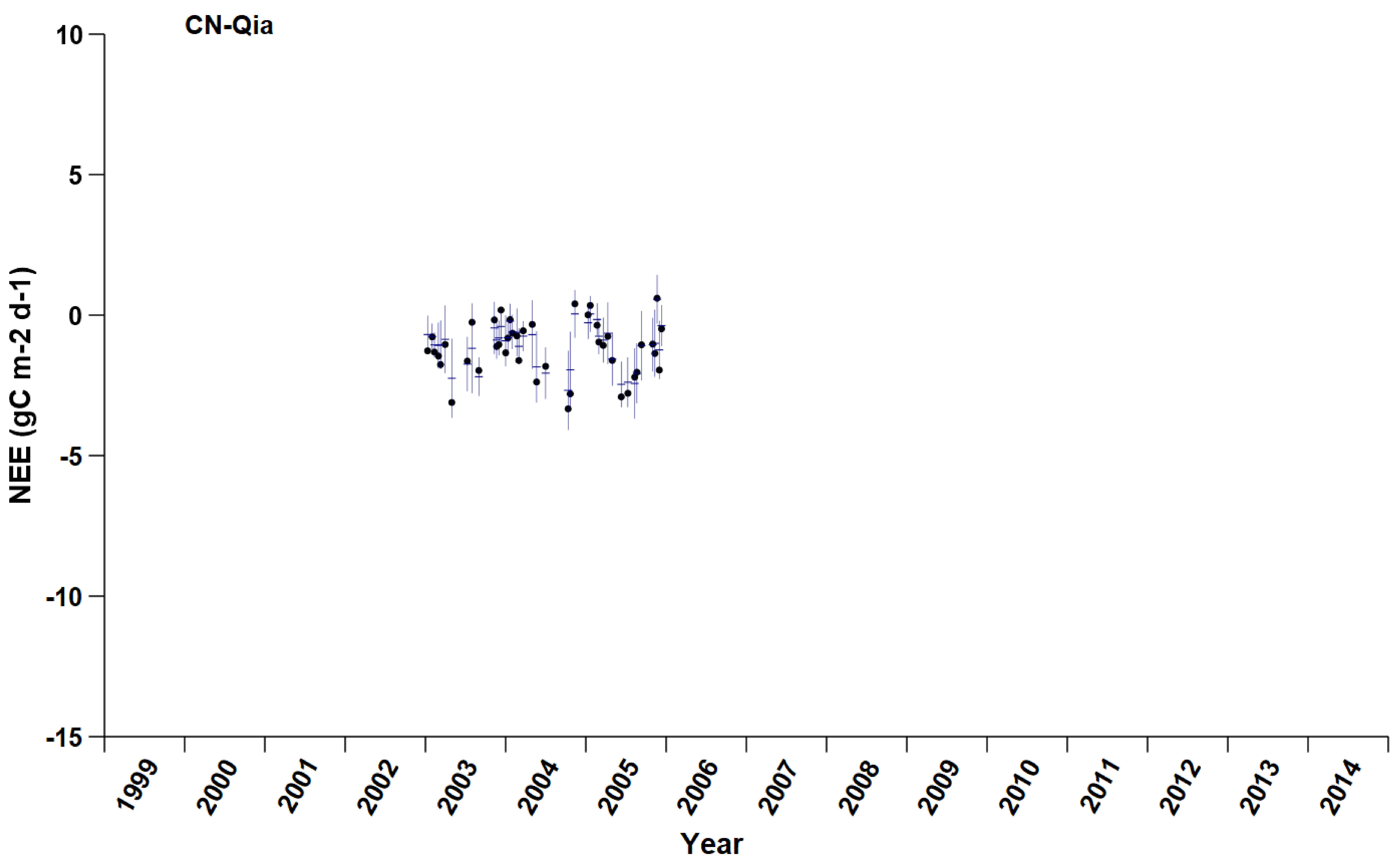

CN-Qia
10
5
0
-5
-10
-15
NEE (gC m-2 d-1)
1999
2000
2001
2002
2003
2004
2005
2006
2007
2008
2009
2010
2011
2012
2013
2014
Year

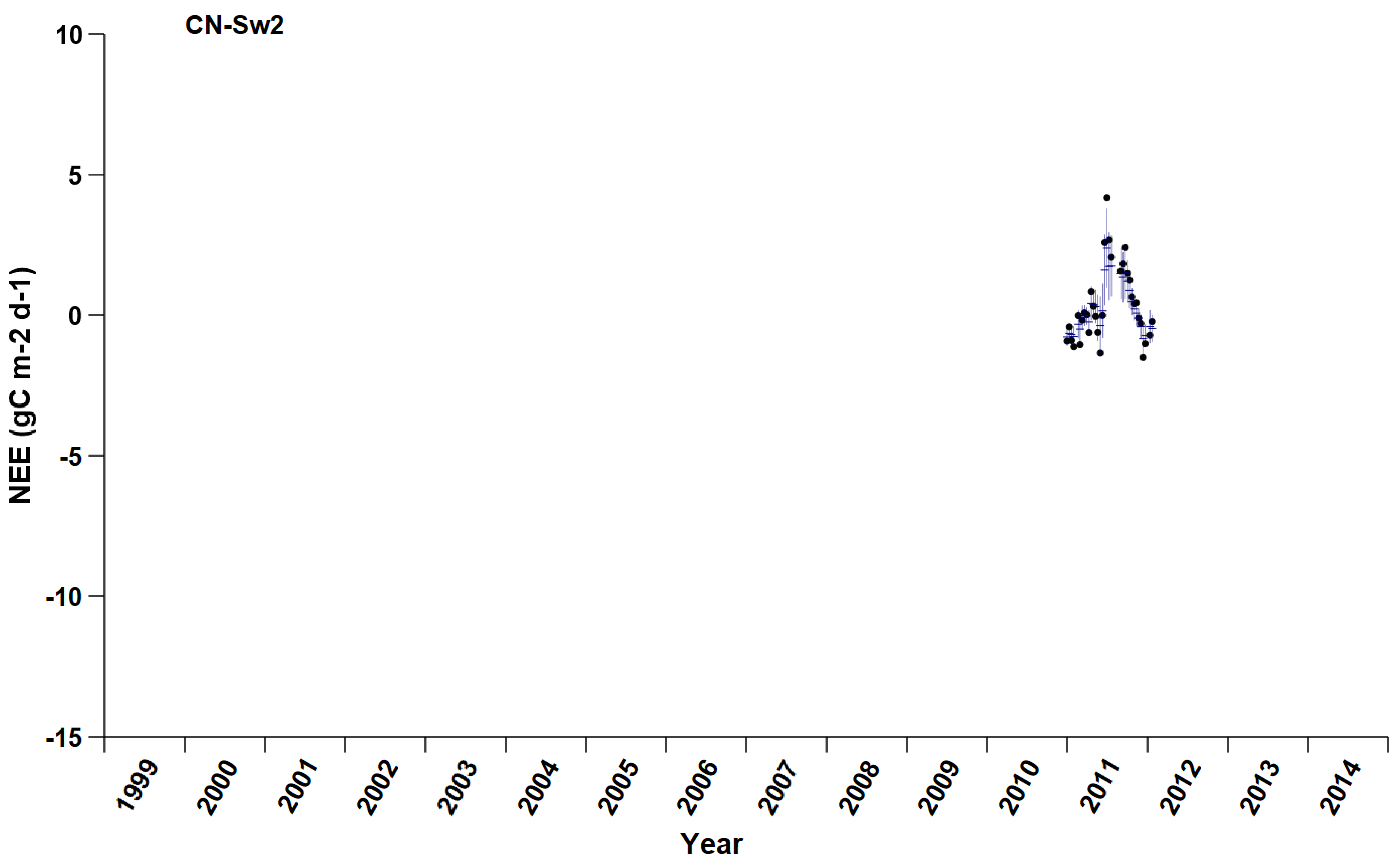

CN-Sw2
NEE (gC m-2 d-1)
10
5
0
-5
-10
-15
1999
2000
2001
2002
2003
2004
2005
2006
2007
2008
2009
2010
2011
2012
2013
2014
Year

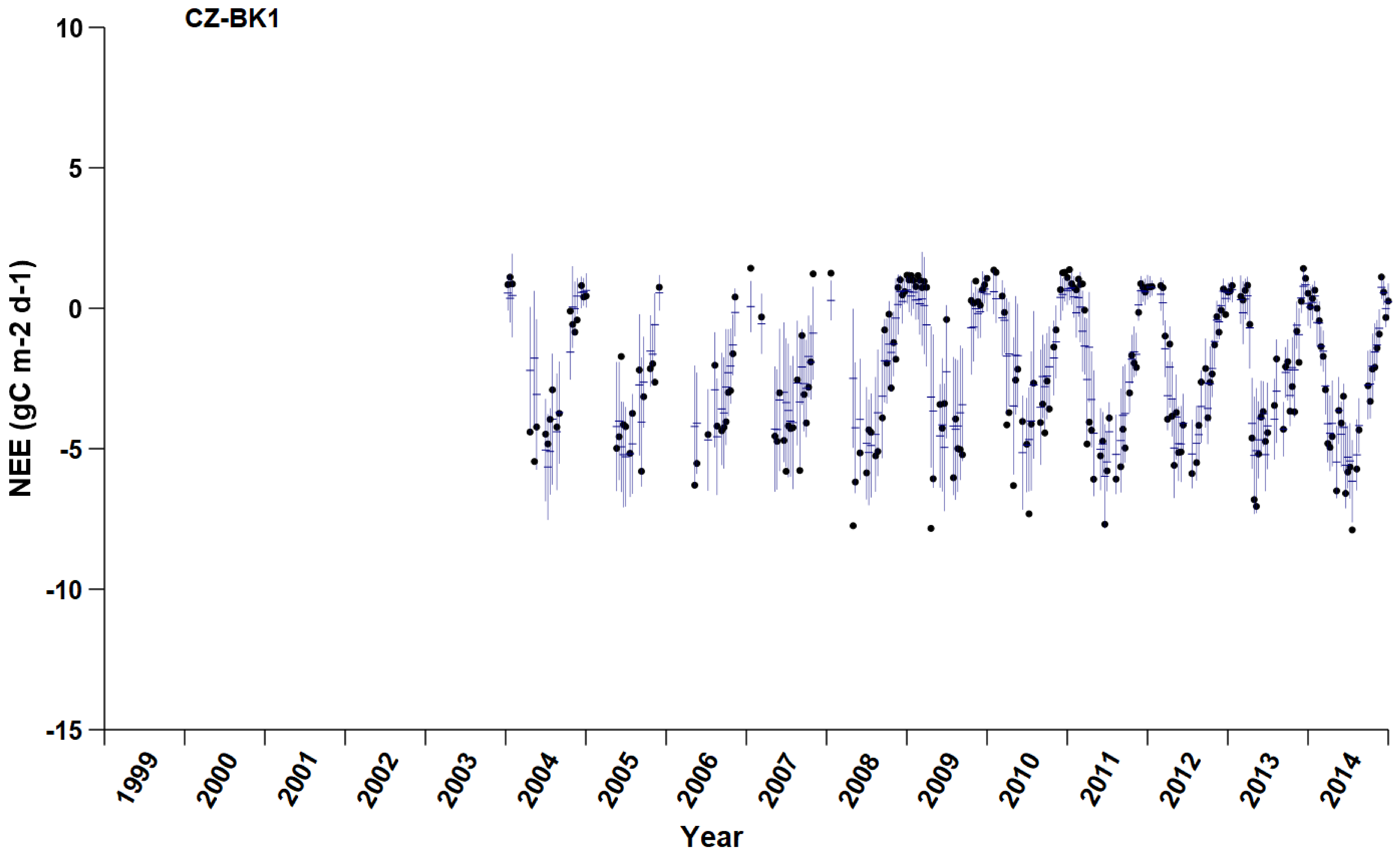
CZ-BK1
NEE (gC m-2 d-1)
10
5
0
-5
-10
-15
1999
2000
2001
2002
2003
2004
2005
2006
2007
2008
2009
2010
2011
2012
2013
2014
Year

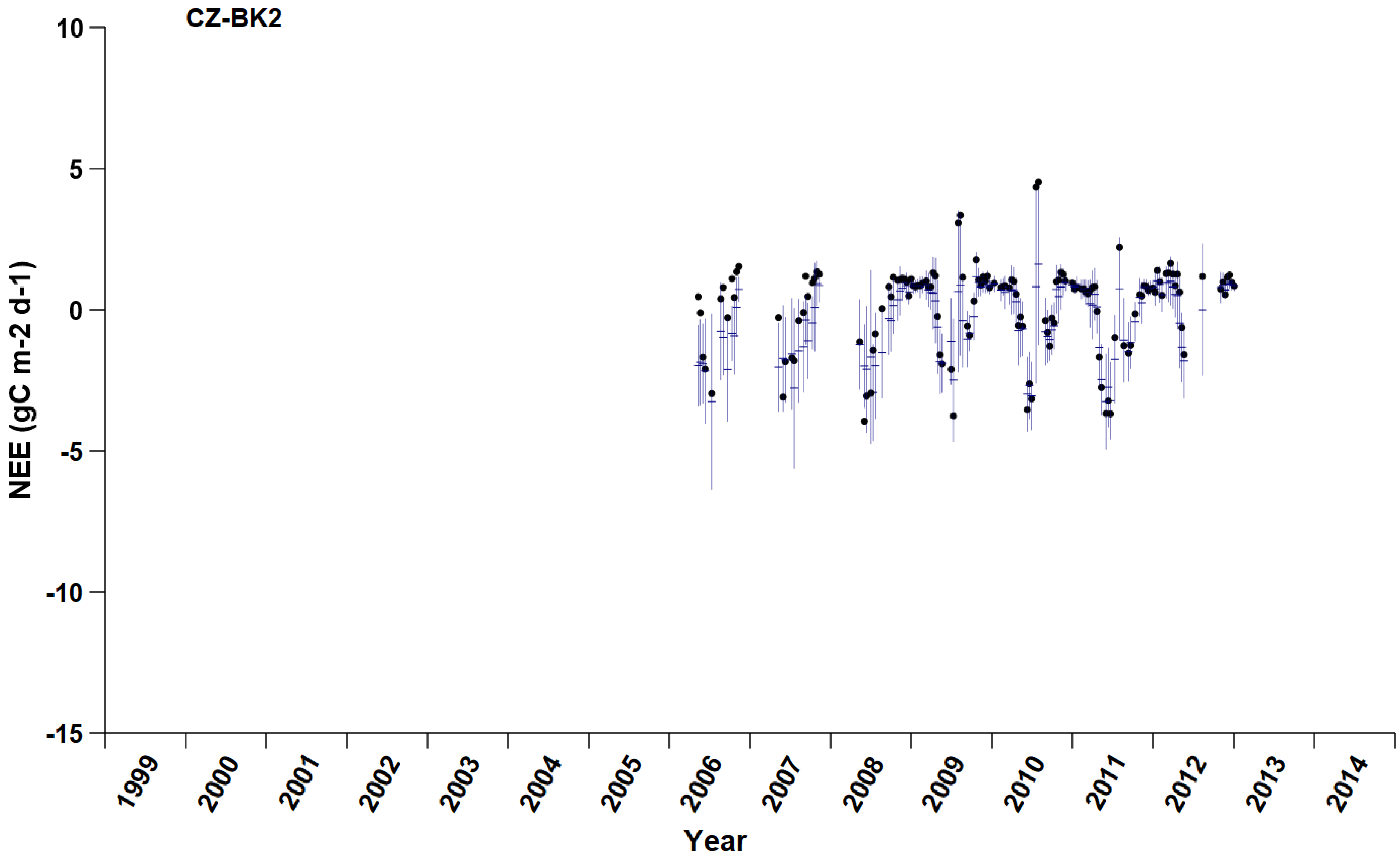

CZ-BK2
NEE (gC m-2 d-1)
10
5
0
-5
-10
-15
1999
2000
2001
2002
2003
2004
2005
2006
2007
2008
2009
2010
2011
2012
2013
2014
Year

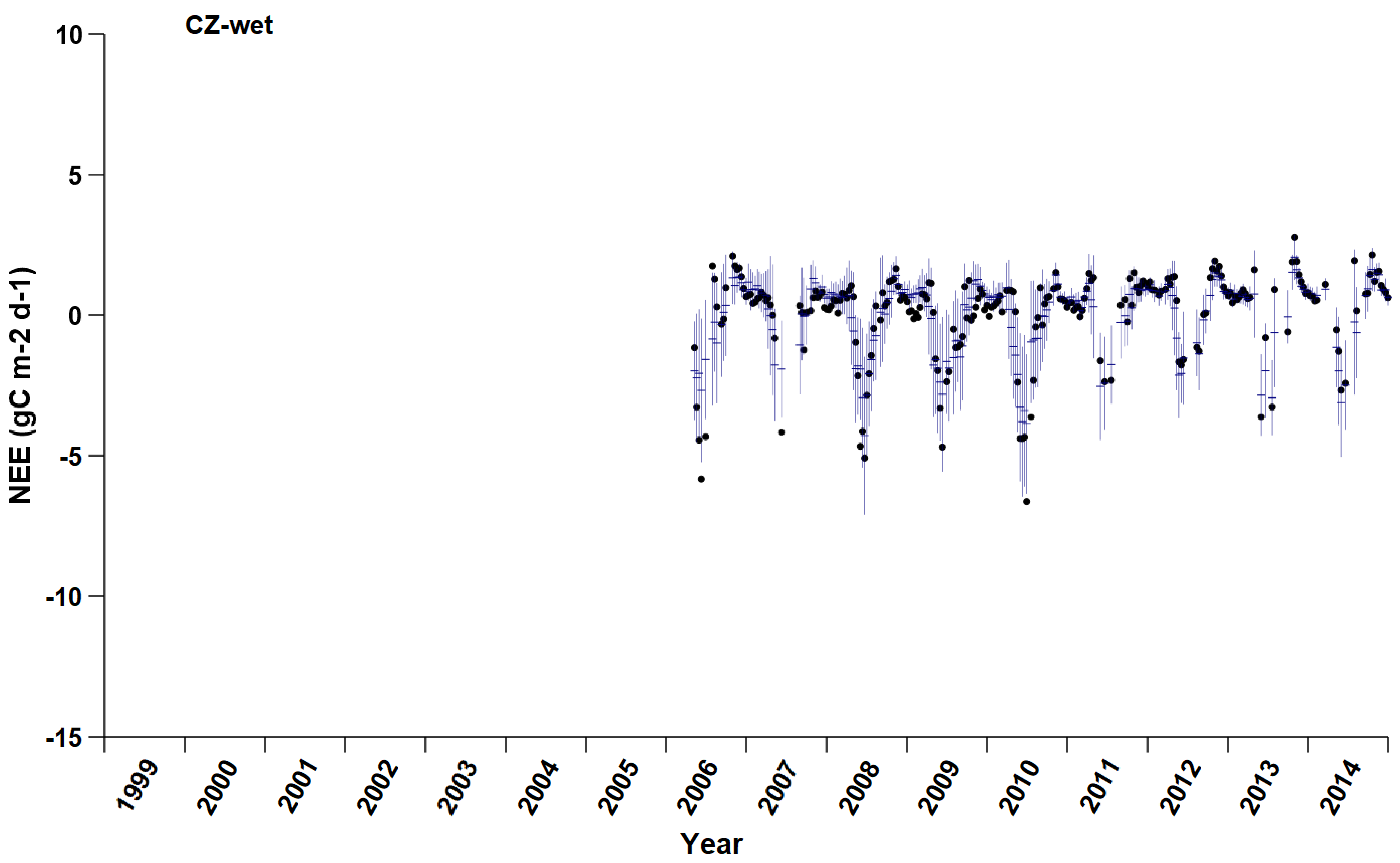

CZ-wet
NEE (gC m-2 d-1)
10
5
0
-5
-10
-15
1999
2000
2001
2002
2003
2004
2005
2006
2007
2008
2009
2010
2011
2012
2013
2014
Year

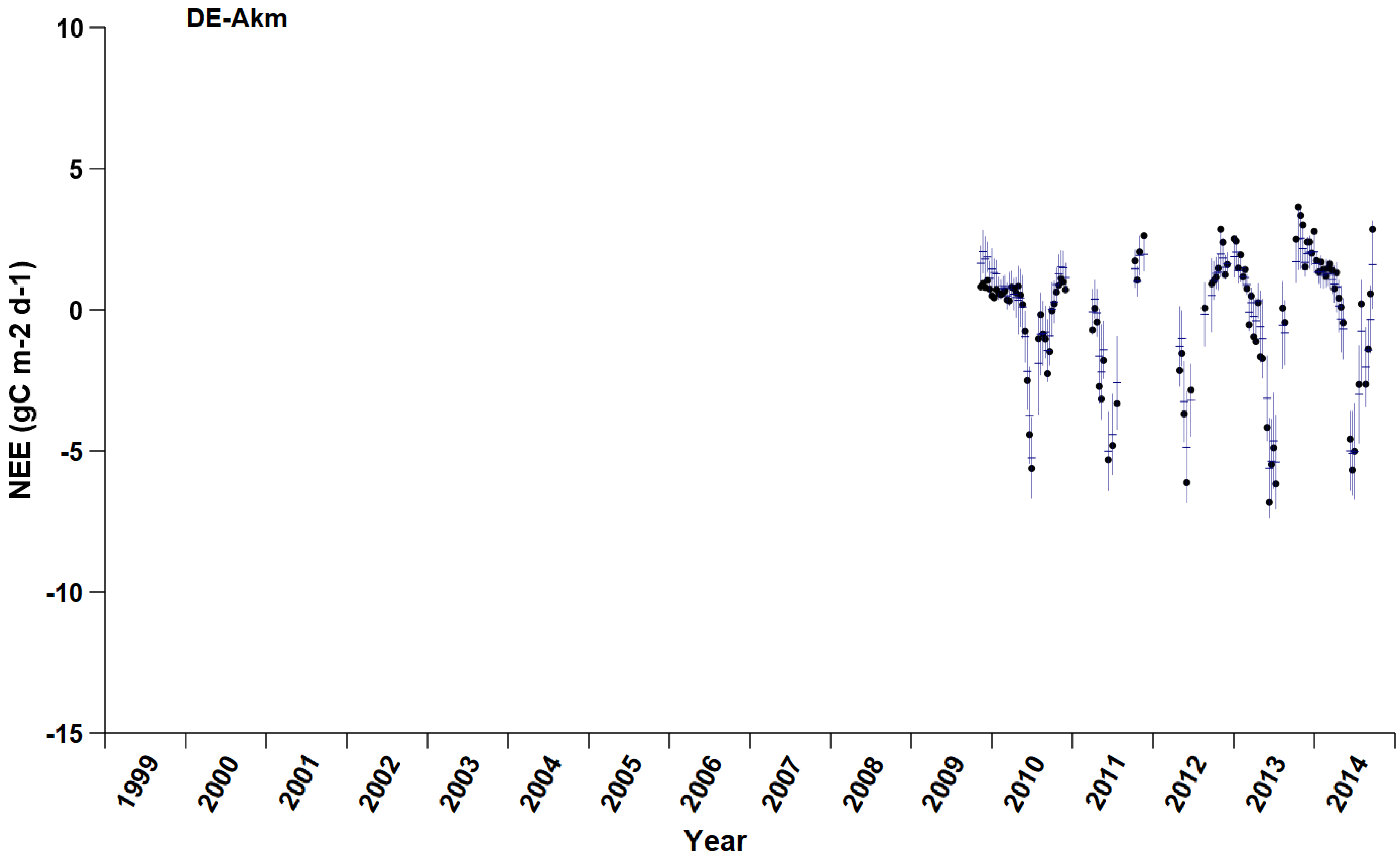
DE-Akm
NEE (gC m-2 d-1)
10
5
0
-5
-10
-15
1999
2000
2001
2002
2003
2004
2005
2006
2007
2008
2009
2010
2011
2012
2013
2014
Year

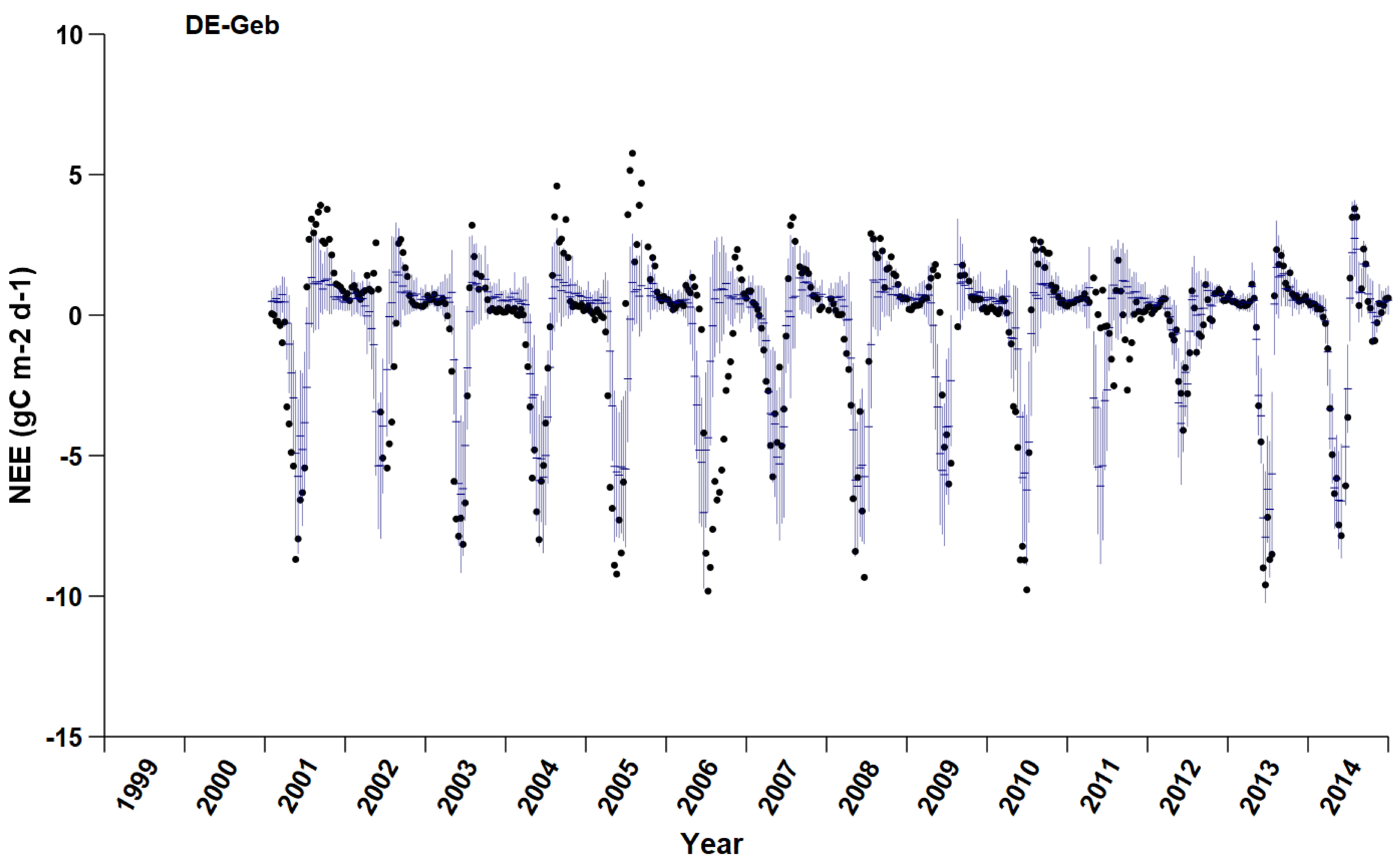
DE-Geb
NEE (gC m-2 d-1)
10
5
0
-5
-10
-15
1999
2000
2001
2002
2003
2004
2005
2006
2007
2008
2009
2010
2011
2012
2013
2014
Year

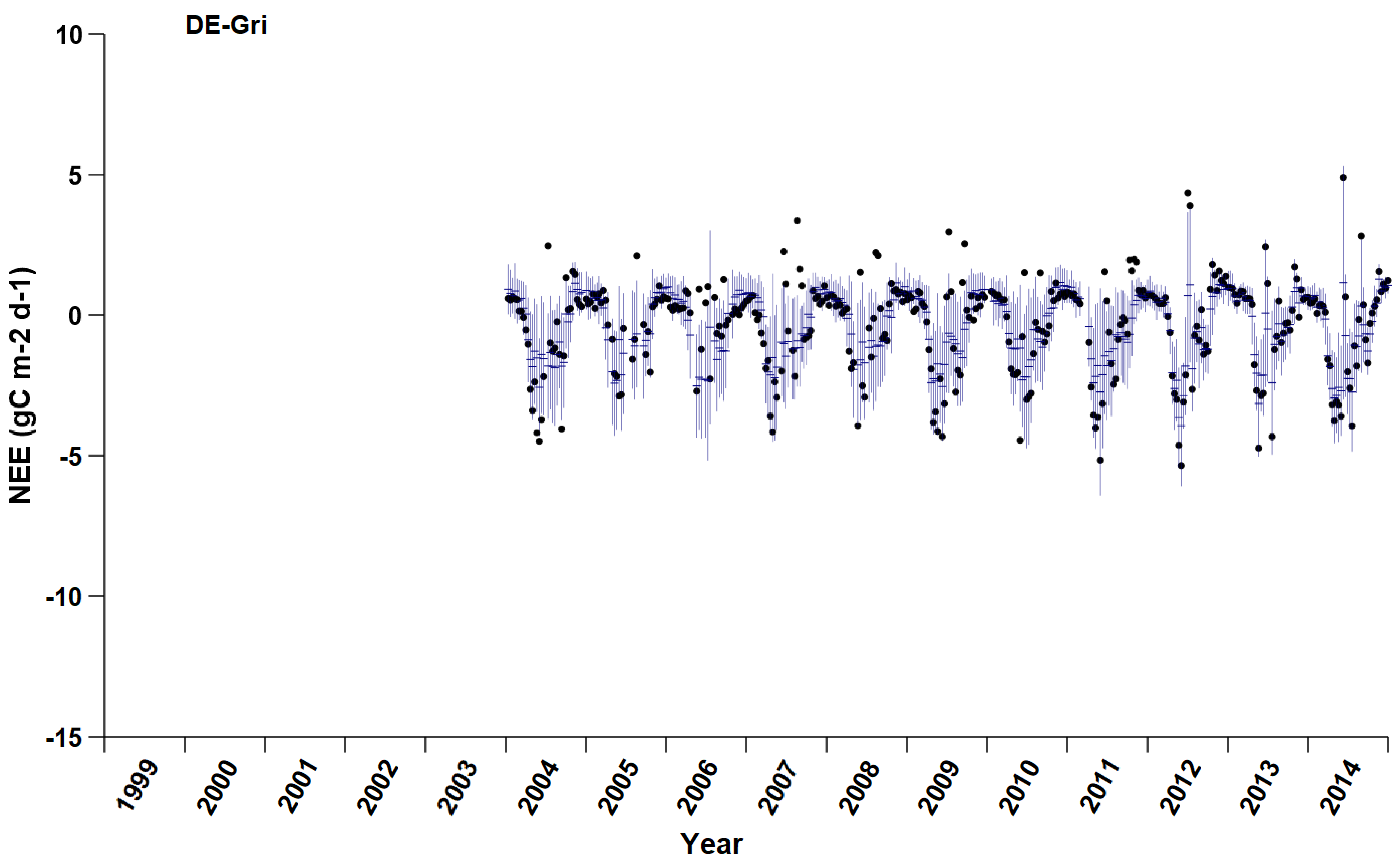
DE-Gri
NEE (gC m-2 d-1)
10
5
0
-5
-10
-15
1999
2000
2001
2002
2003
2004
2005
2006
2007
2008
2009
2010
2011
2012
2013
2014
Year

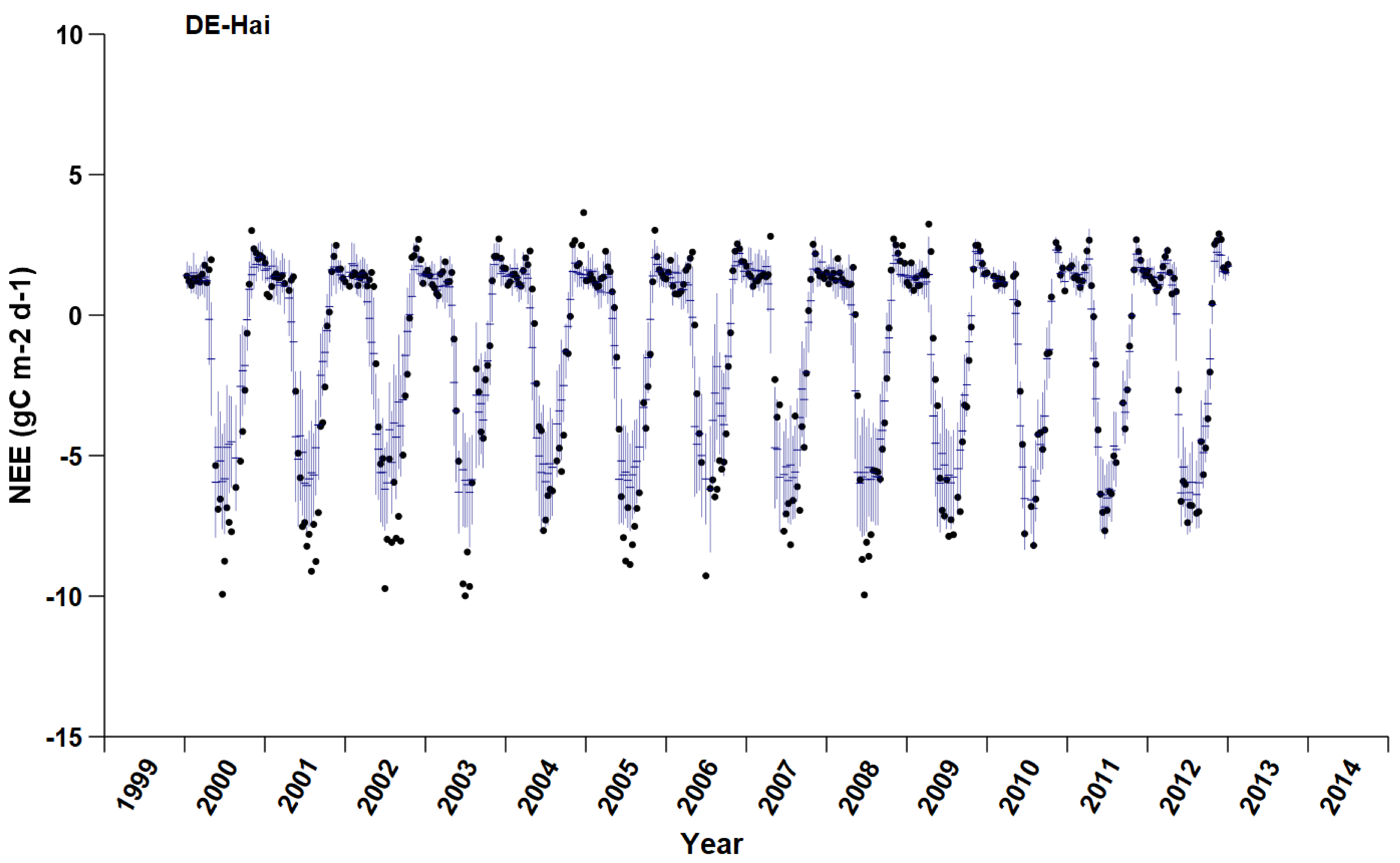
DE-Hai
NEE (gC m-2 d-1)
10
5
0
-5
-10
-15
1999
2000
2001
2002
2003
2004
2005
2006
2007
2008
2009
2010
2011
2012
2013
2014
Year

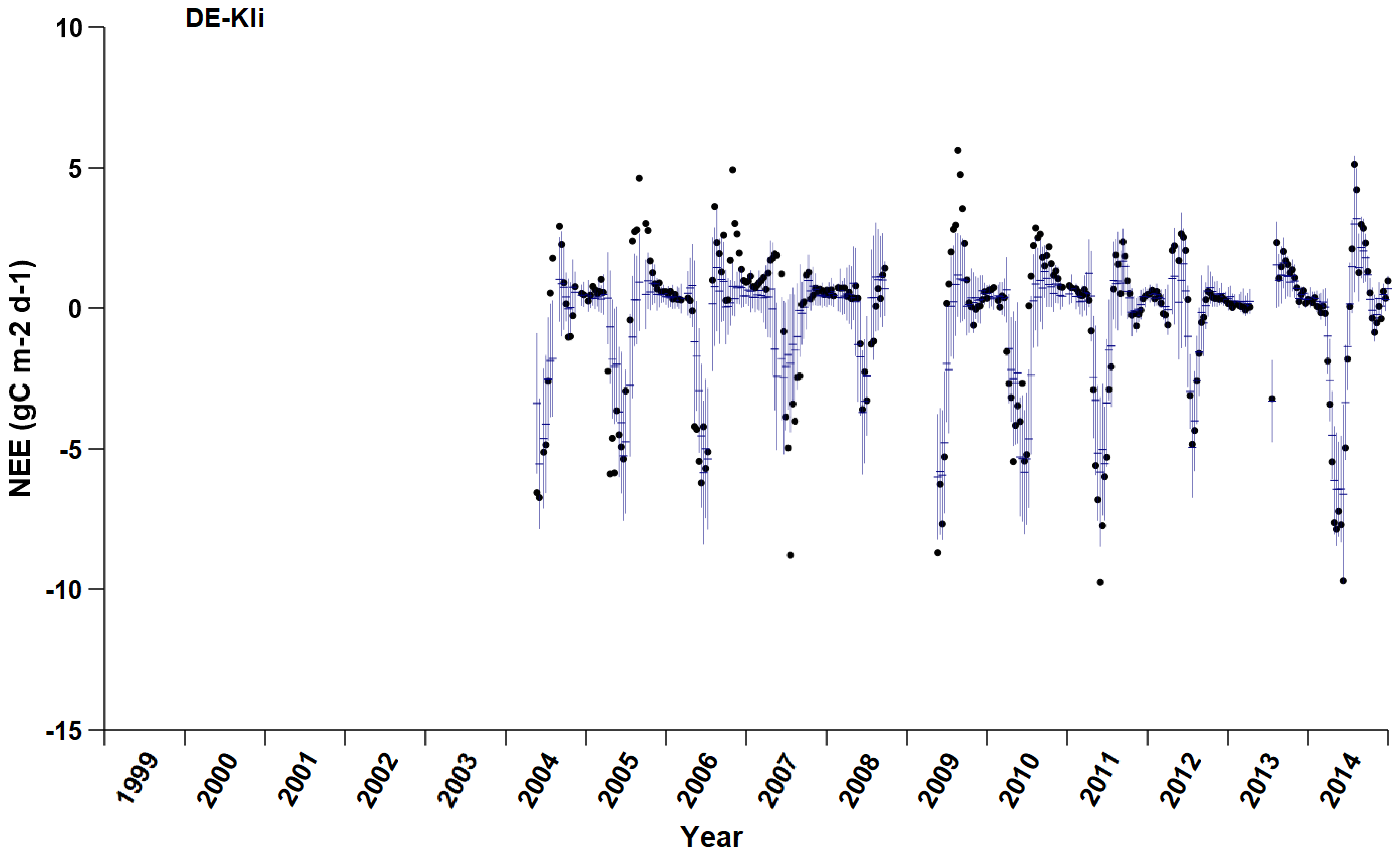
DE-Kli
NEE (gC m-2 d-1)
10
5
0
-5
-10
-15
1999
2000
2001
2002
2003
2004
2005
2006
2007
2008
2009
2010
2011
2012
2013
2014
Year

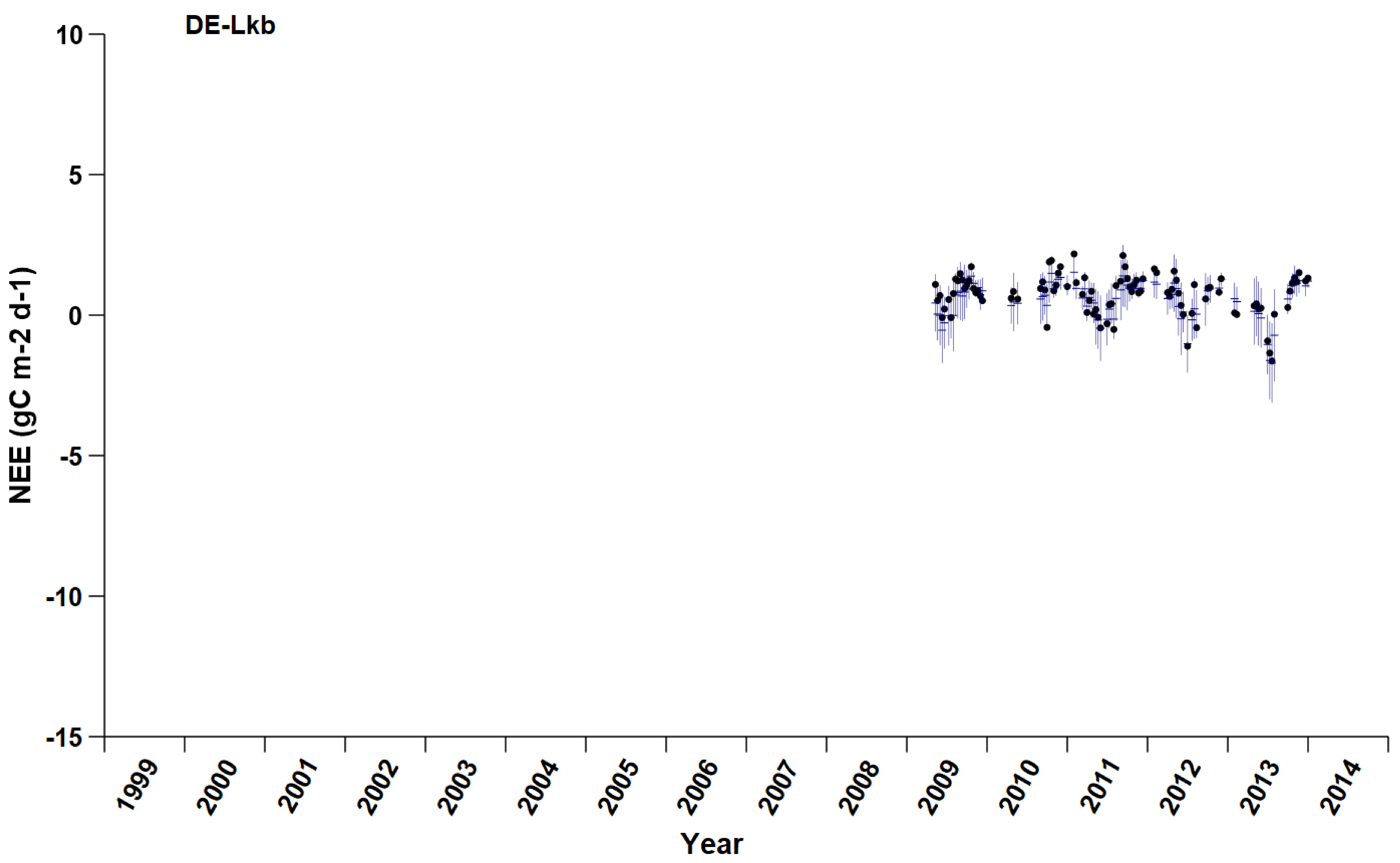
DE-Lkb
NEE (gC m-2 d-1)
10
5
0
-5
-10
-15
1999
2000
2001
2002
2003
2004
2005
2006
2007
2008
2009
2010
2011
2012
2013
2014
Year

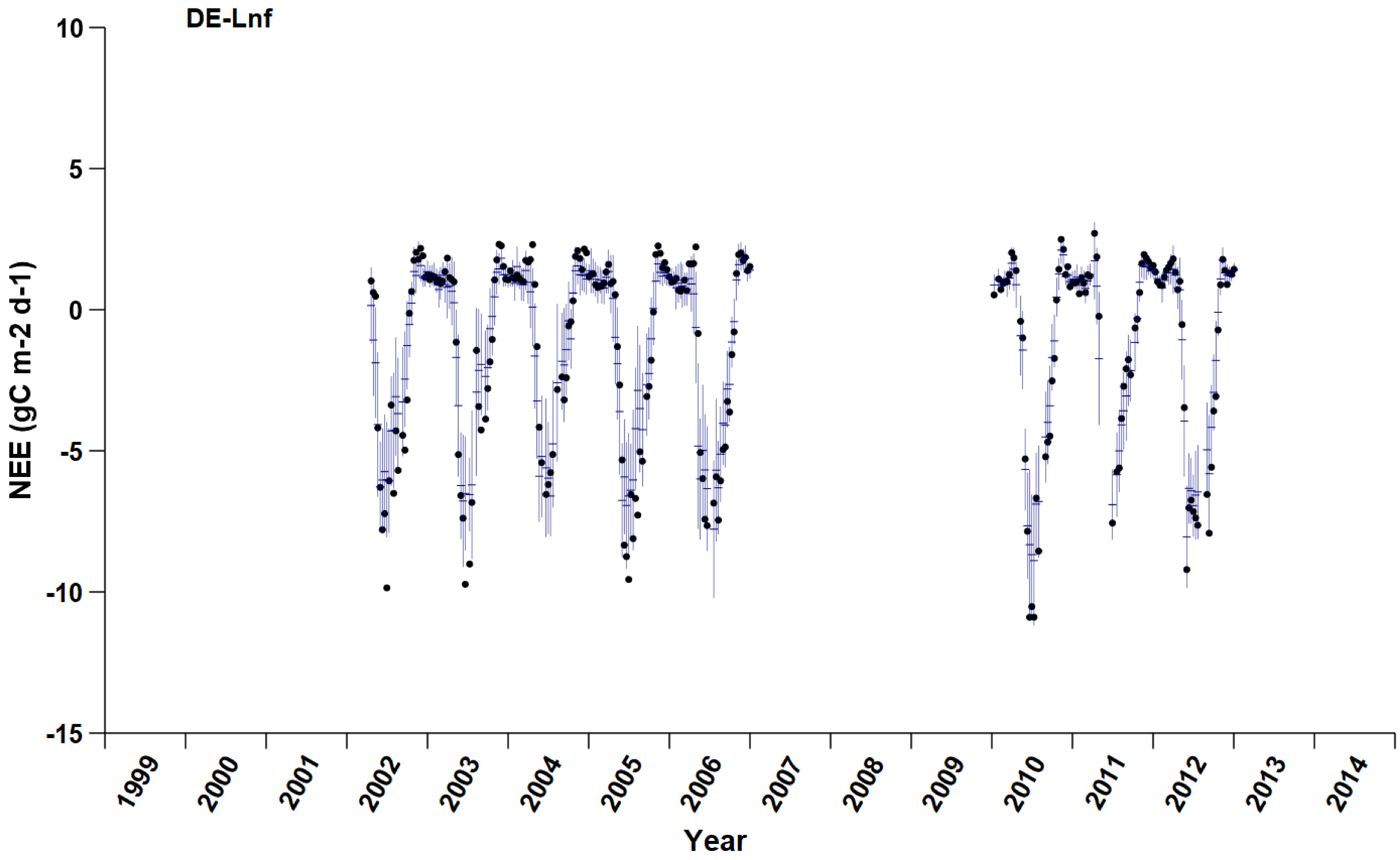
DE-Lnf
NEE (gC m-2 d-1)
10
5
0
-5
-10
-15
1999
2000
2001
2002
2003
2004
2005
2006
2007
2008
2009
2010
2011
2012
2013
2014
Year

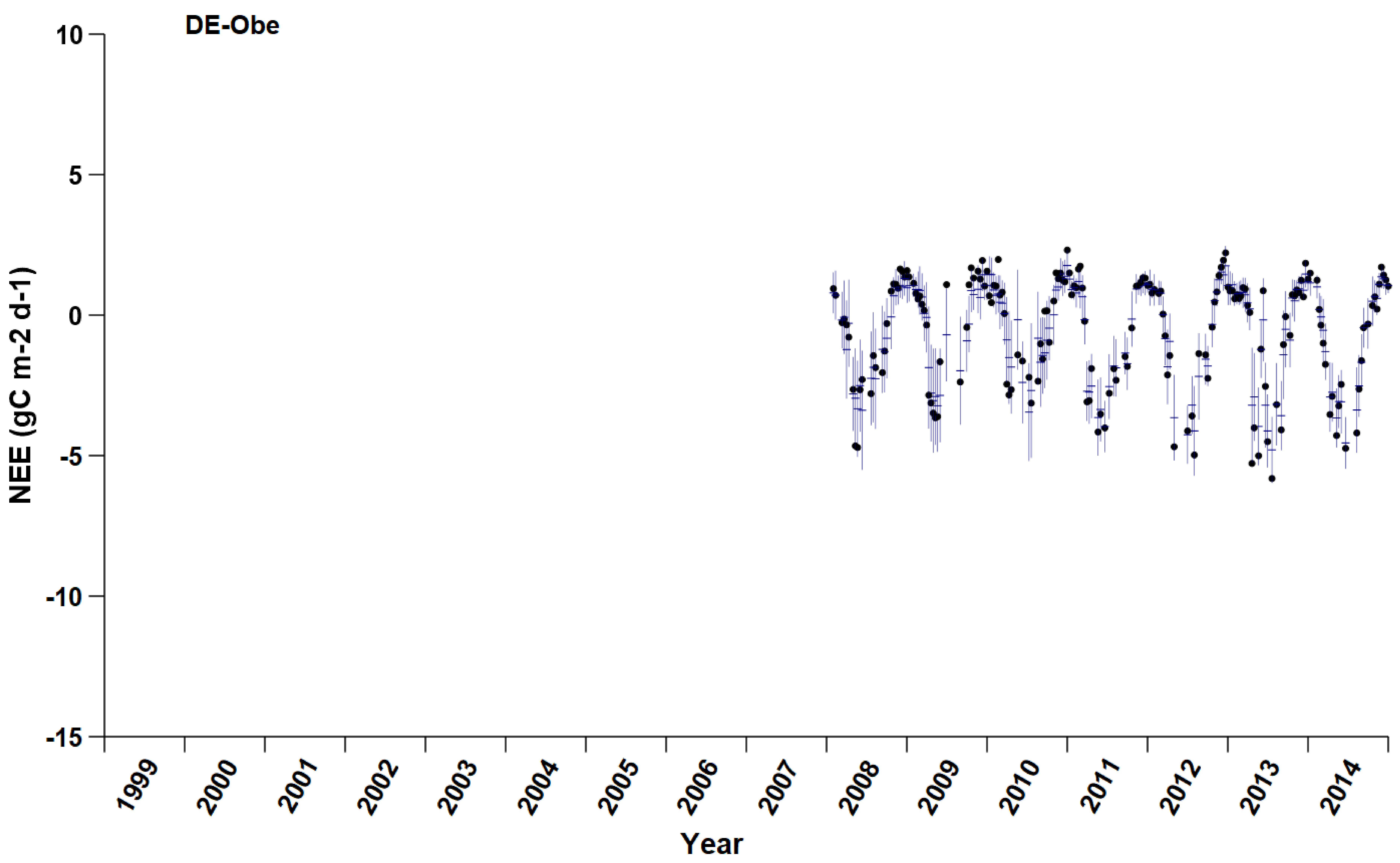
DE-Obe
NEE (gC m-2 d-1)
10
5
0
-5
-10
-15
1999
2000
2001
2002
2003
2004
2005
2006
2007
2008
2009
2010
2011
2012
2013
2014
Year

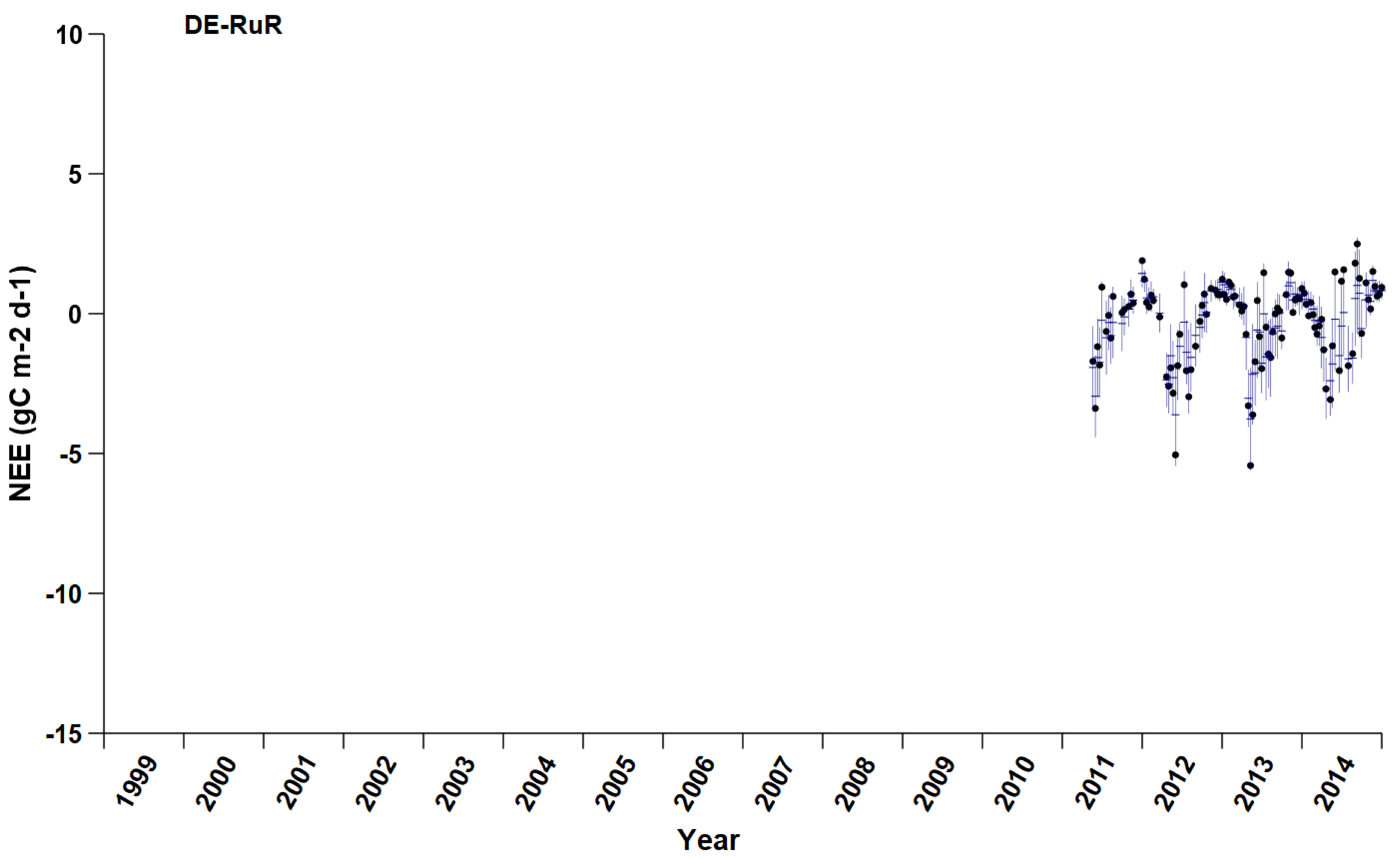
DE-RuR
NEE (gC m-2 d-1)
10
5
0
-5
-10
-15
1999
2000
2001
2002
2003
2004
2005
2006
2007
2008
2009
2010
2011
2012
2013
2014
Year

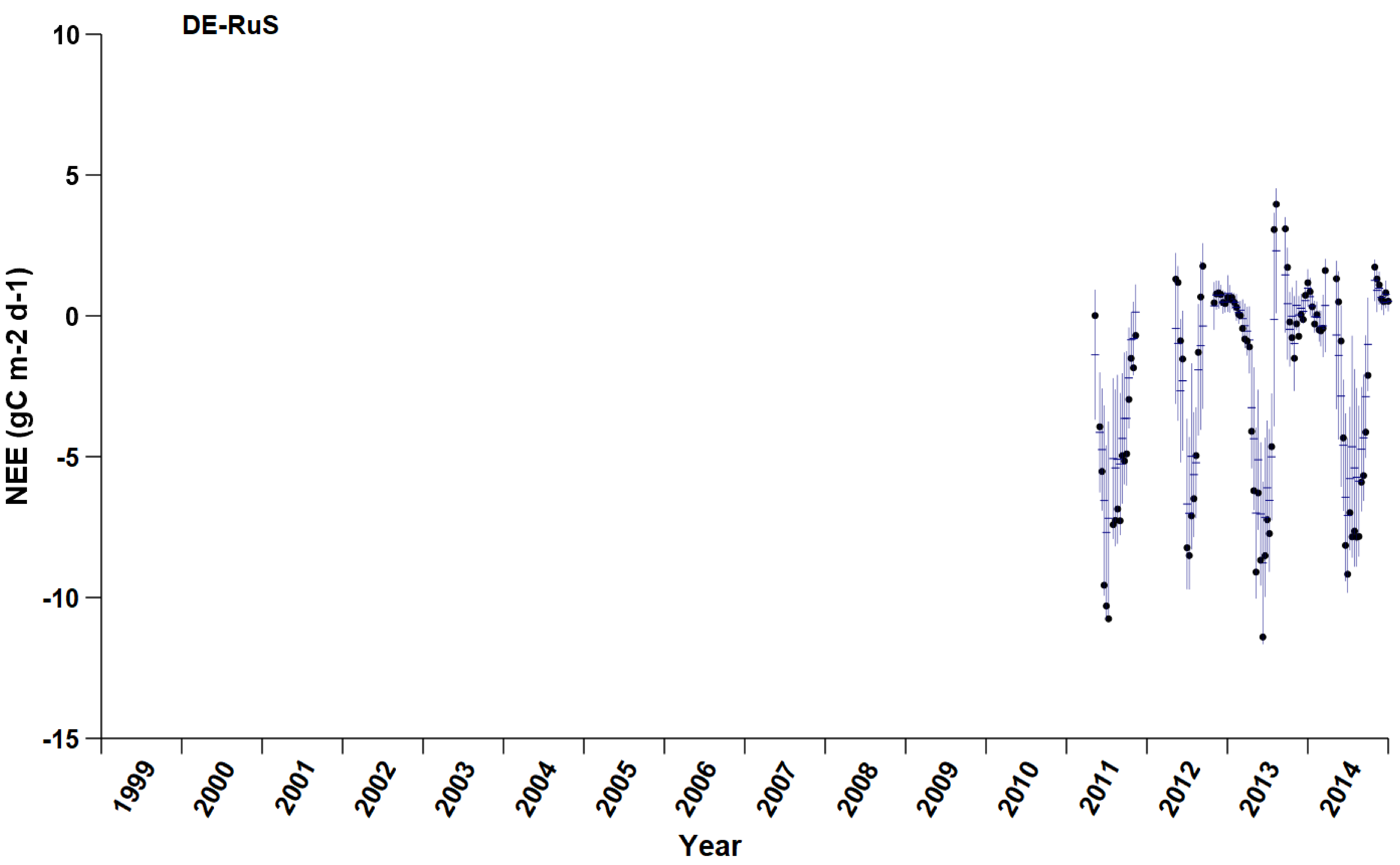
DE-RuS
NEE (gC m-2 d-1)
10
5
0
-5
-10
-15
1999
2000
2001
2002
2003
2004
2005
2006
2007
2008
2009
2010
2011
2012
2013
2014
Year

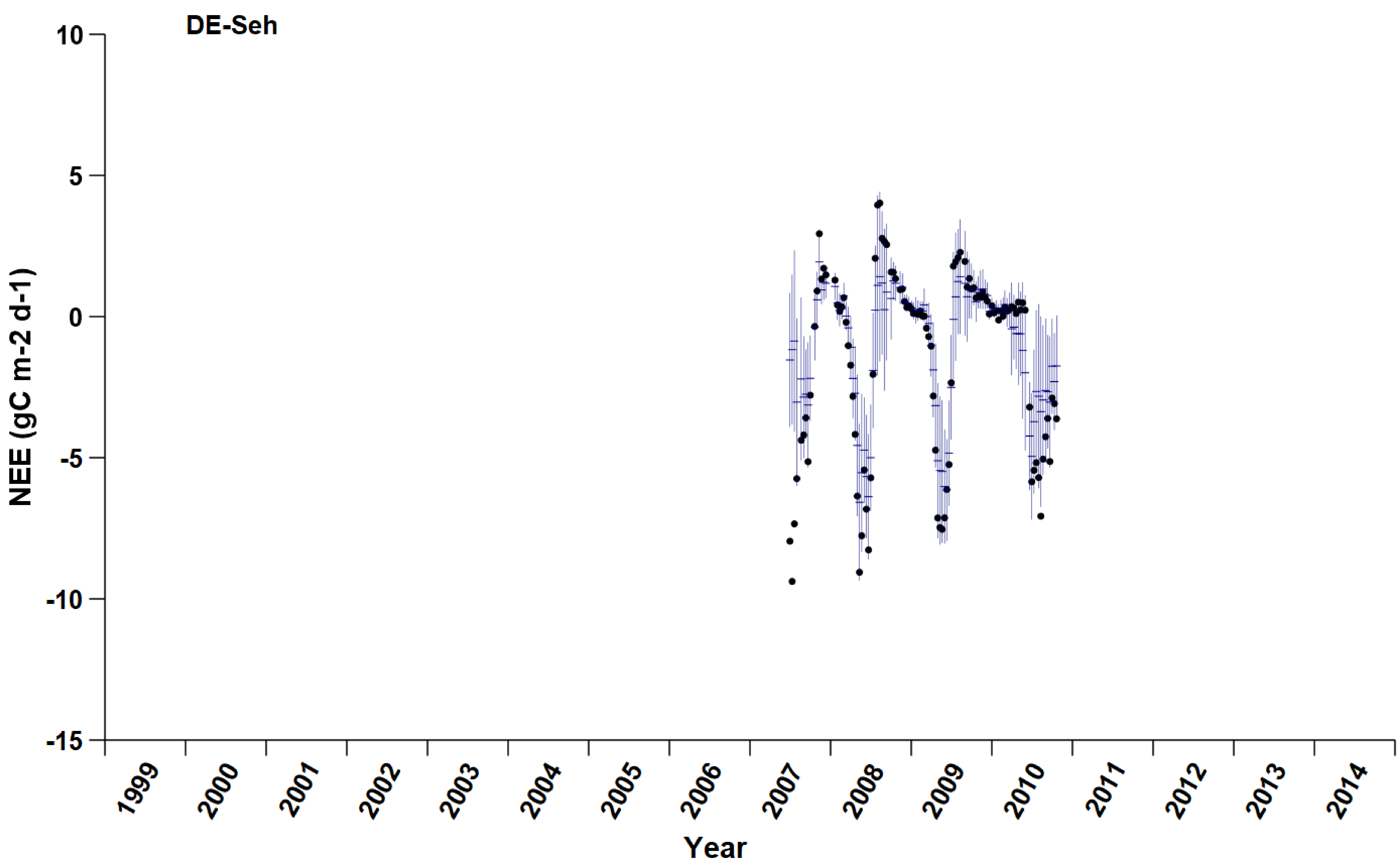

DE-Seh
10
5
0
-5
-10
-15
NEE (gC m-2 d-1)
1999
2000
2001
2002
2003
2004
2005
2006
2007
2008
2009
2010
2011
2012
2013
2014
Year

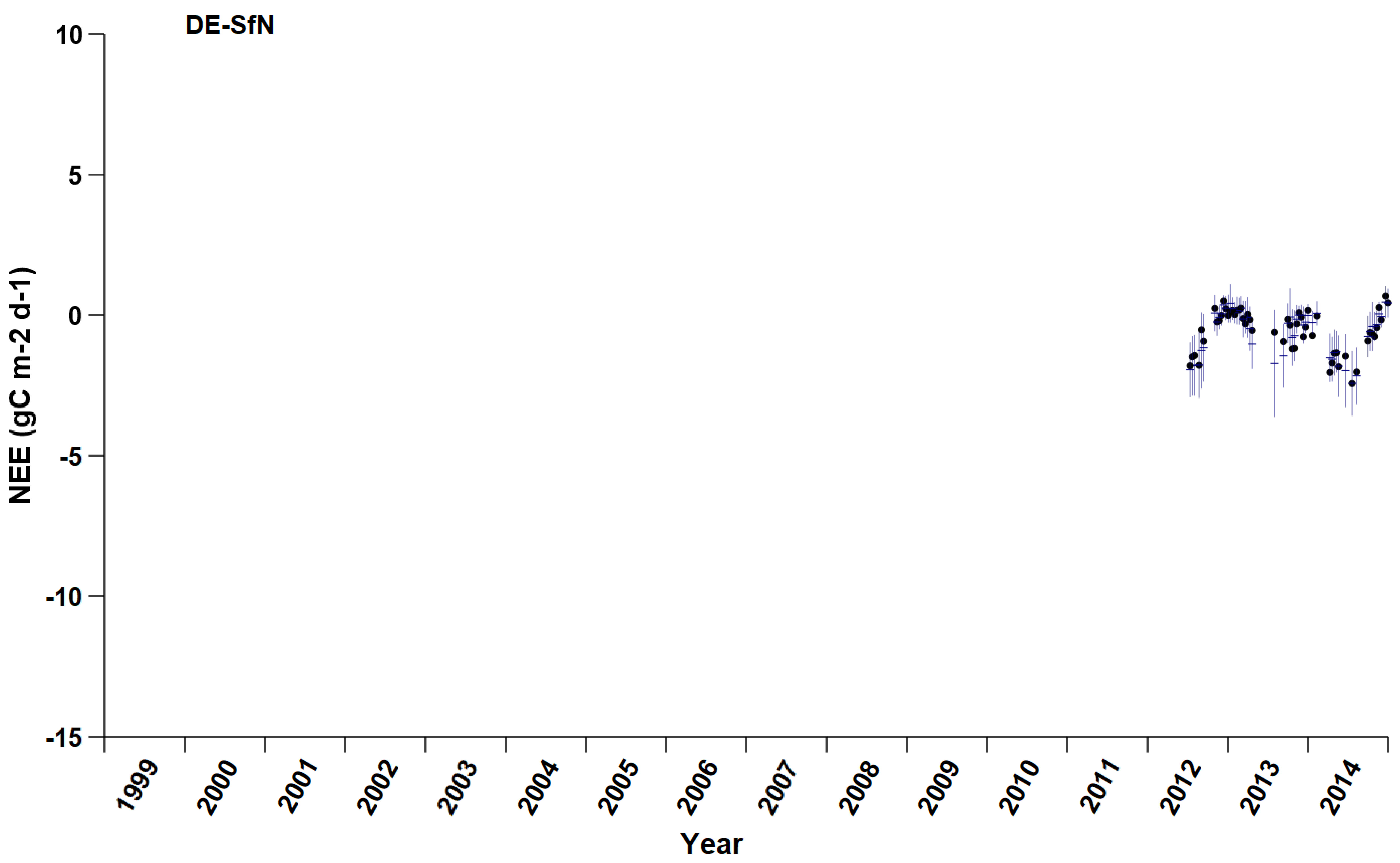
DE-SfN
NEE (gC m-2 d-1)
10
5
0
-5
-10
-15
1999
2000
2001
2002
2003
2004
2005
2006
2007
2008
2009
2010
2011
2012
2013
2014
Year

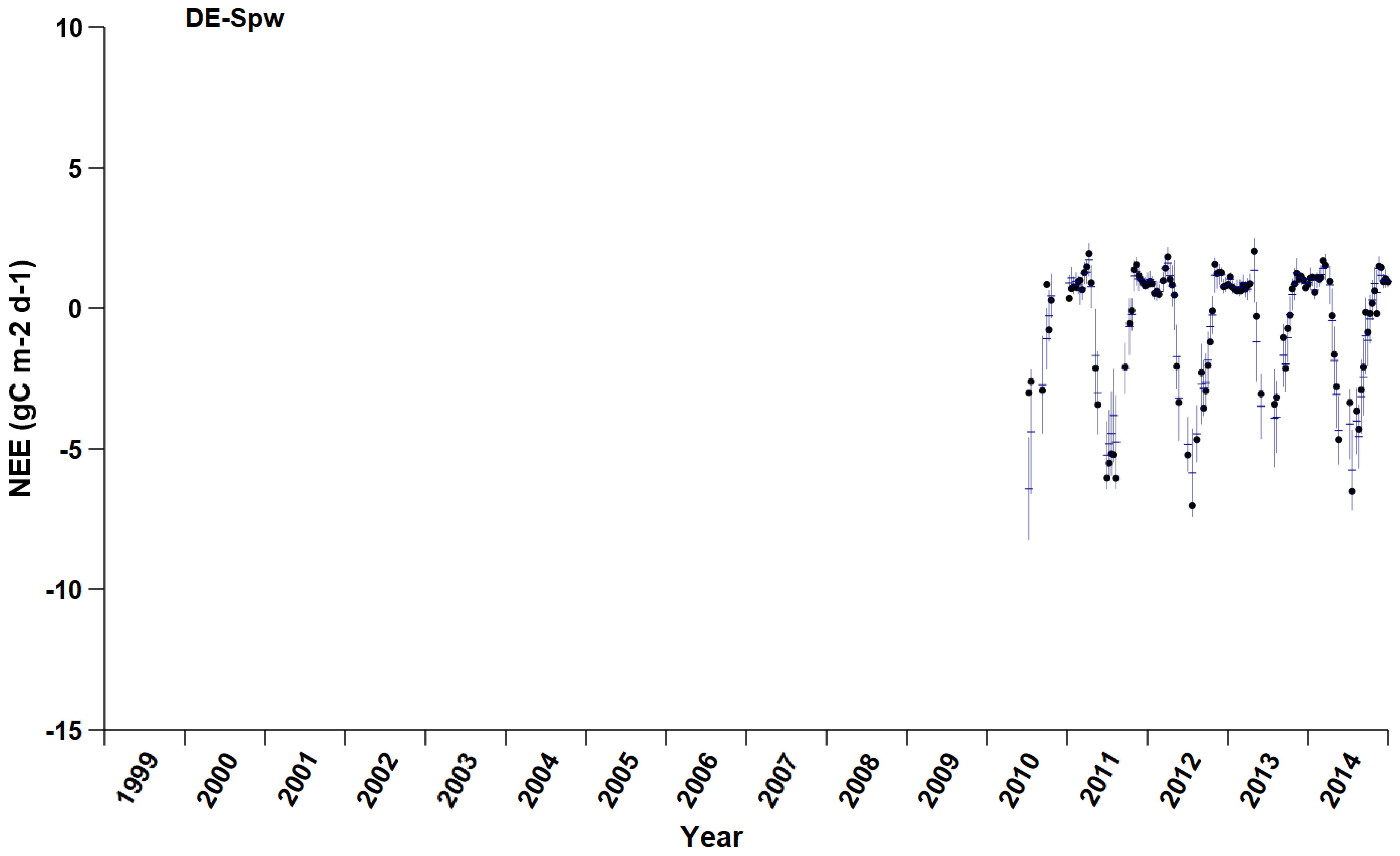
DE-Spw
NEE (gC m-2 d-1)
10
5
0
-5
-10
-15
1999
2000
2001
2002
2003
2004
2005
2006
2007
2008
2009
2010
2011
2012
2013
2014
Year

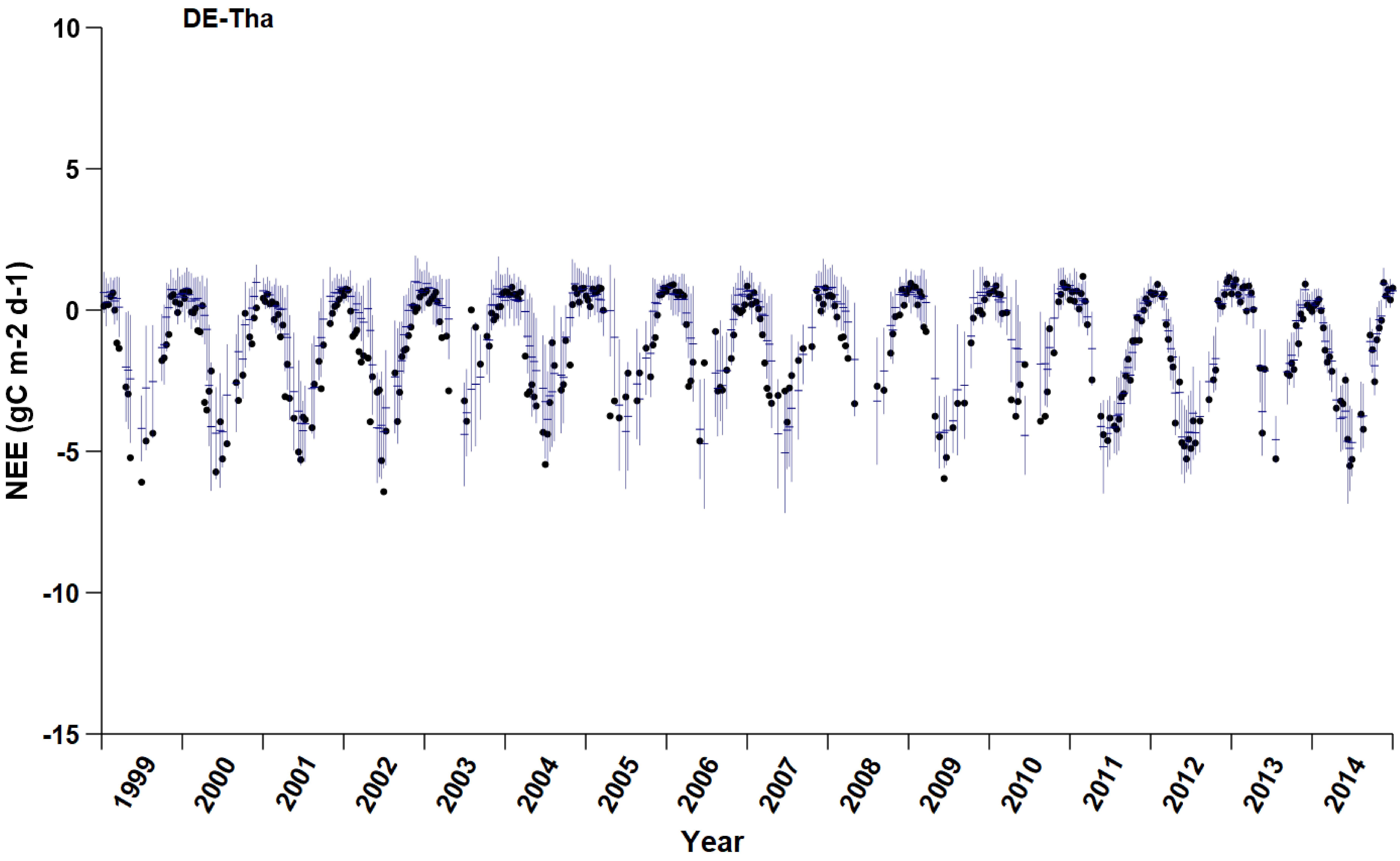
DE-Tha
NEE (gC m-2 d-1)
10
5
0
-5
-10
-15
1999
2000
2001
2002
2003
2004
2005
2006
2007
2008
2009
2010
2011
2012
2013
2014
Year

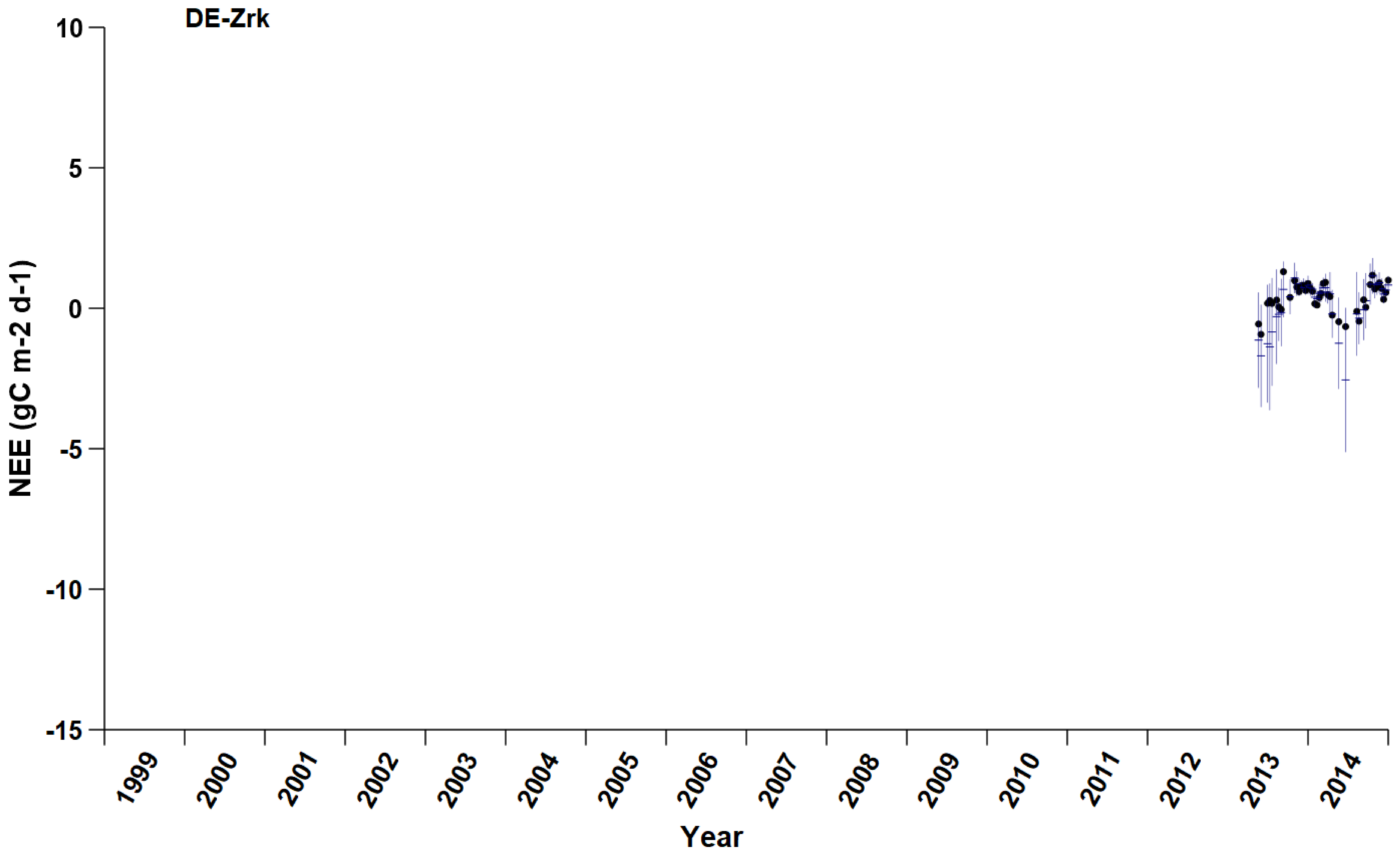
DE-Zrk
NEE (gC m-2 d-1)
10
5
0
-5
-10
-15
1999
2000
2001
2002
2003
2004
2005
2006
2007
2008
2009
2010
2011
2012
2013
2014
Year

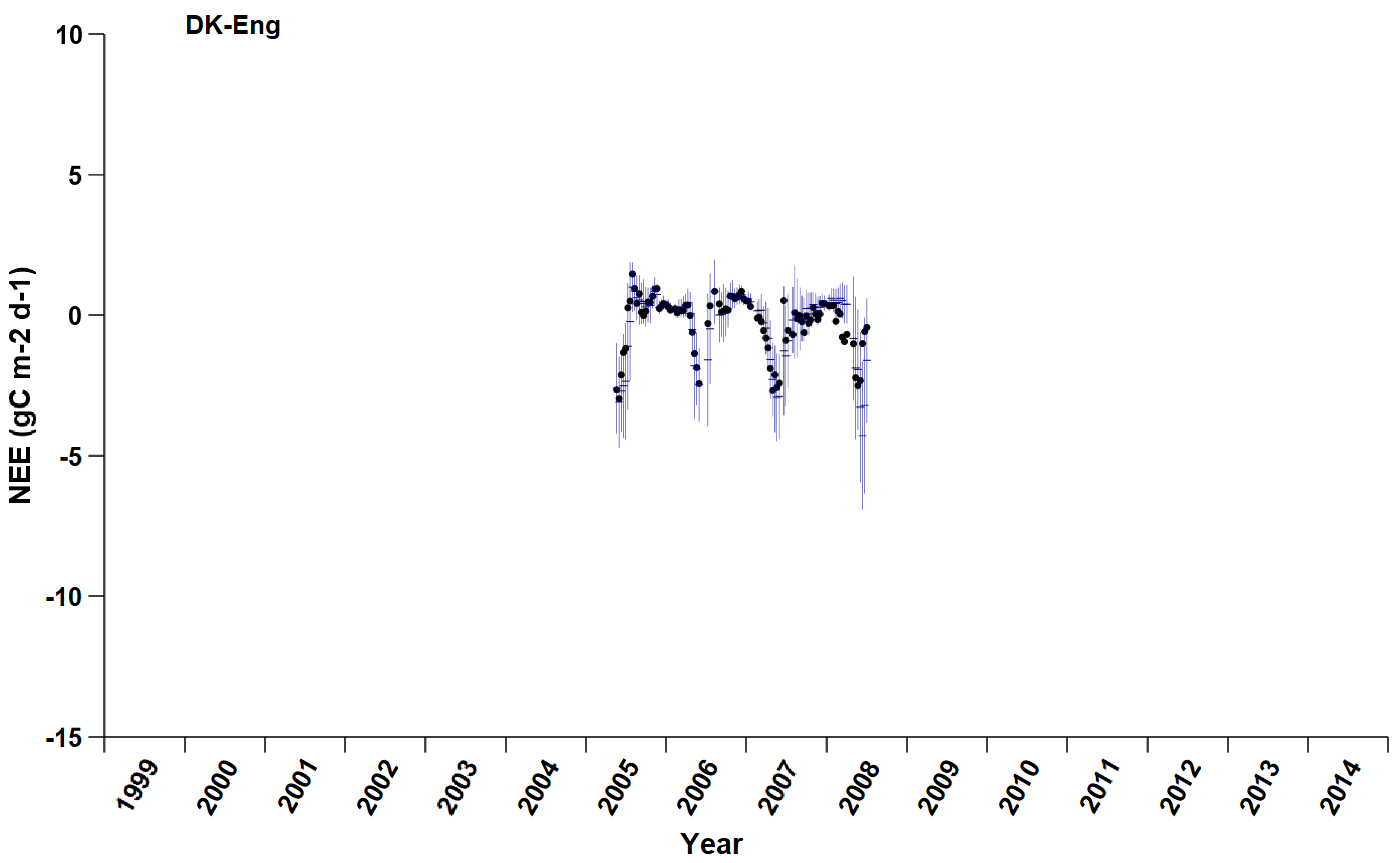
DK-Eng
10
5
0
-5
-10
-15
NEE (gC m-2 d-1)
1999
2000
2001
2002
2003
2004
2005
2006
2007
2008
2009
2010
2011
2012
2013
2014
Year

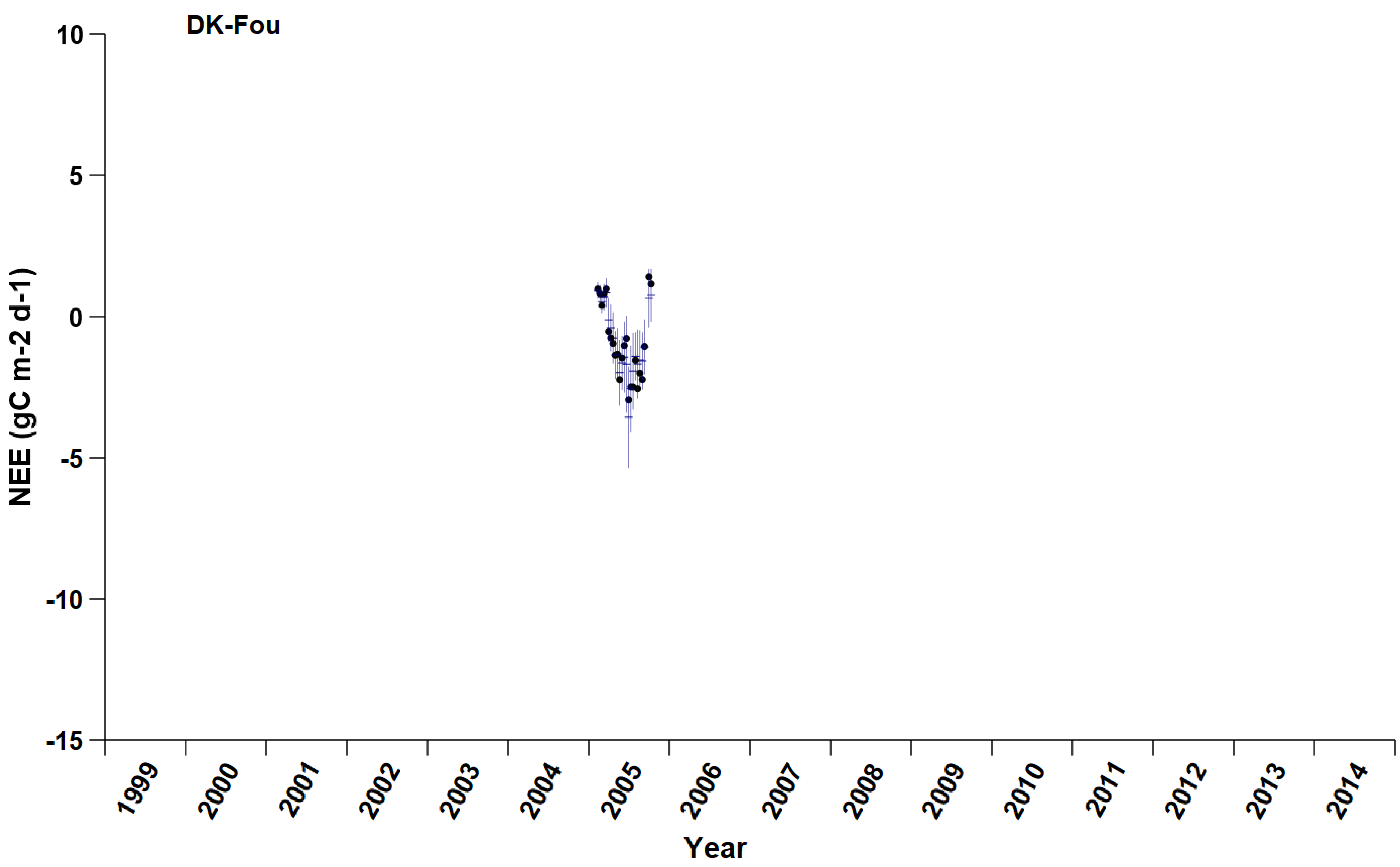

DK-Fou
NEE (gC m-2 d-1)
10
5
0
-5
-10
-15
1999
2000
2001
2002
2003
2004
2005
2006
2007
2008
2009
2010
2011
2012
2013
2014
Year

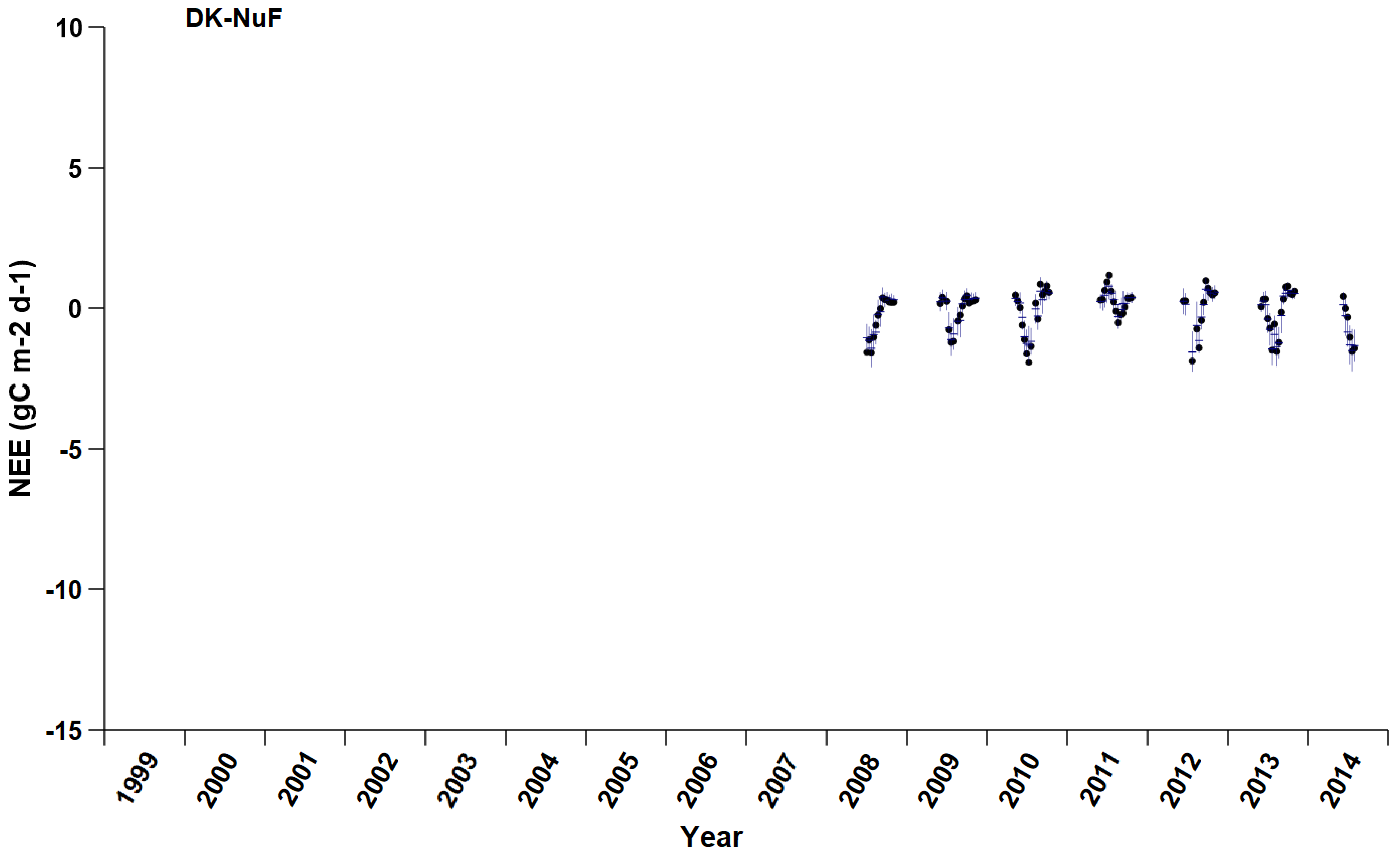
DK-NuF
NEE (gC m-2 d-1)
10
5
0
-5
-10
-15
1999
2000
2001
2002
2003
2004
2005
2006
2007
2008
2009
2010
2011
2012
2013
2014
Year

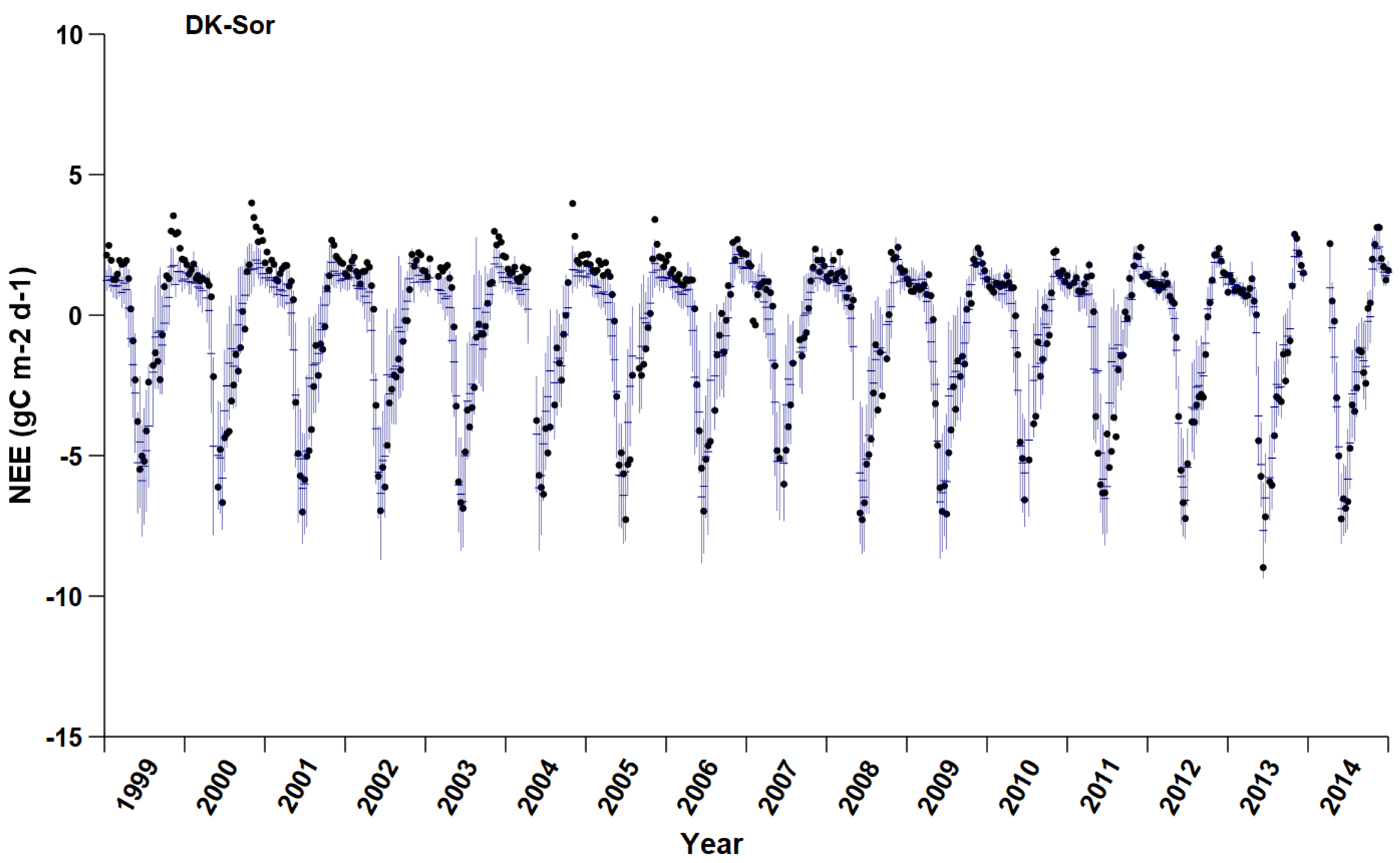
DK-Sor
NEE (gC m-2 d-1)
10
5
0
-5
-10
-15
1999
2000
2001
2002
2003
2004
2005
2006
2007
2008
2009
2010
2011
2012
2013
2014
Year

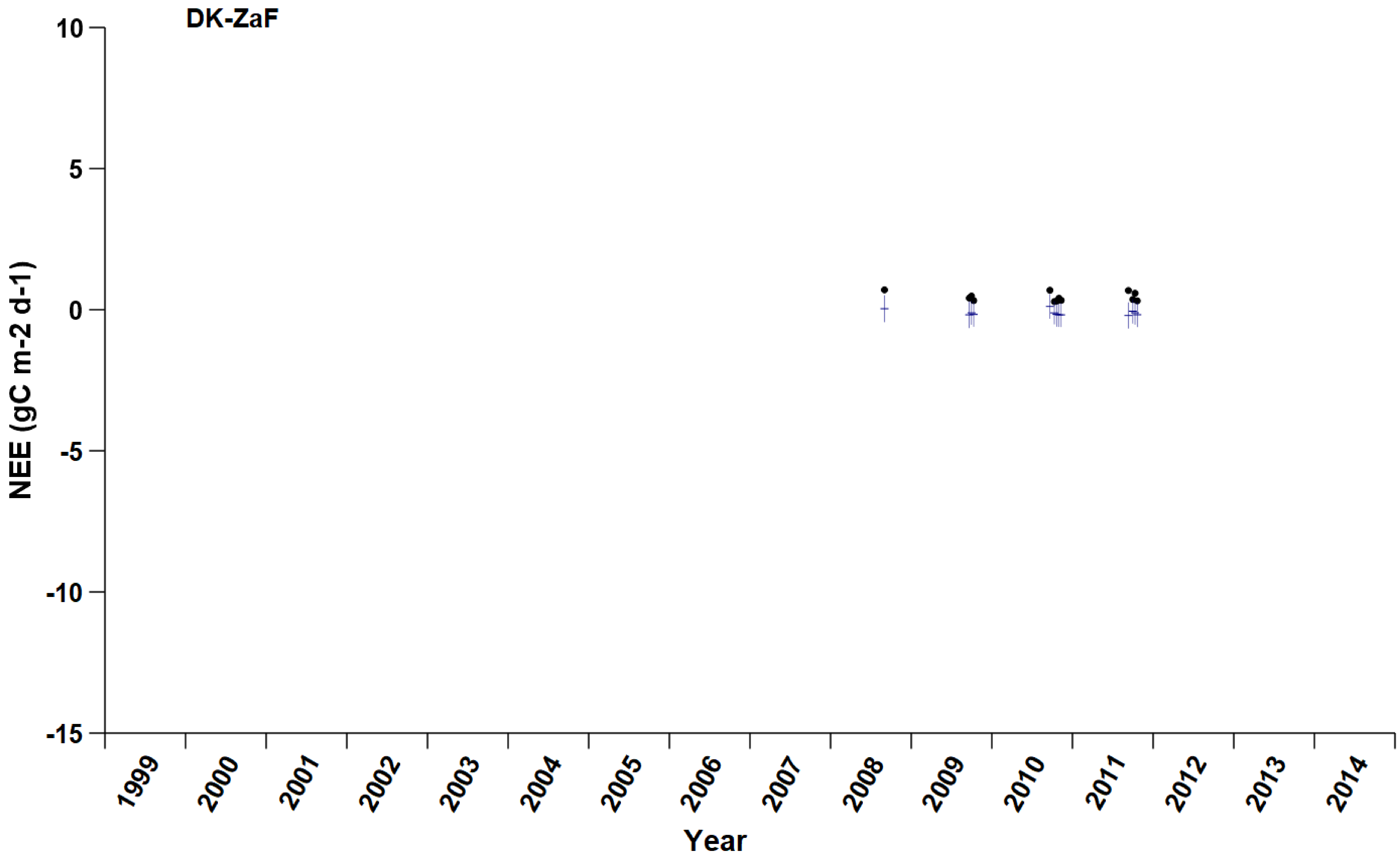

DK-ZaF
10
5
0
-5
-10
-15
NEE (gC m-2 d-1)
1999
2000
2001
2002
2003
2004
2005
2006
2007
2008
2009
2010
2011
2012
2013
2014
Year

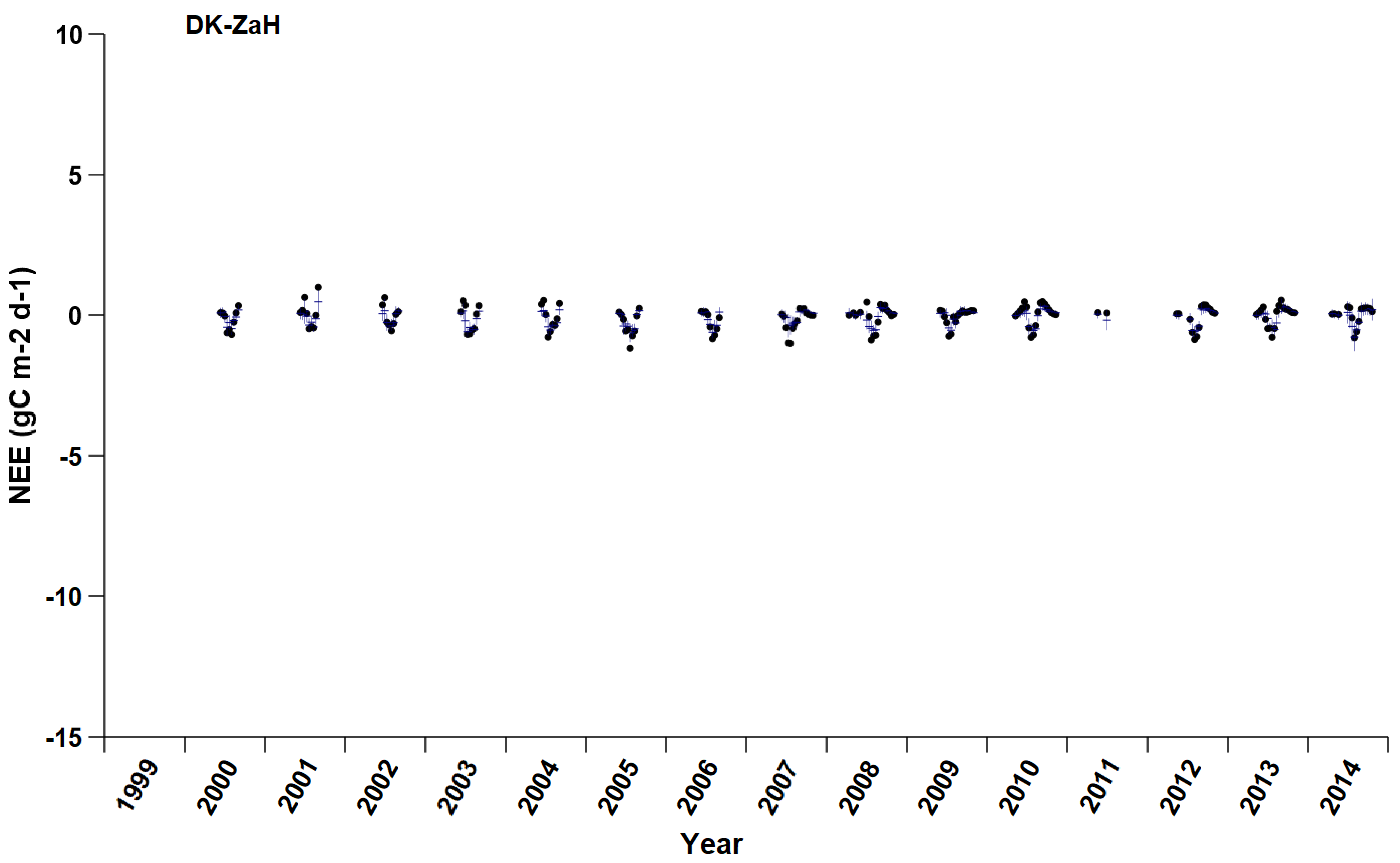
DK-ZaH
NEE (gC m-2 d-1)
10
5
0
-5
-10
-15
1999
2000
2001
2002
2003
2004
2005
2006
2007
2008
2009
2010
2011
2012
2013
2014
Year

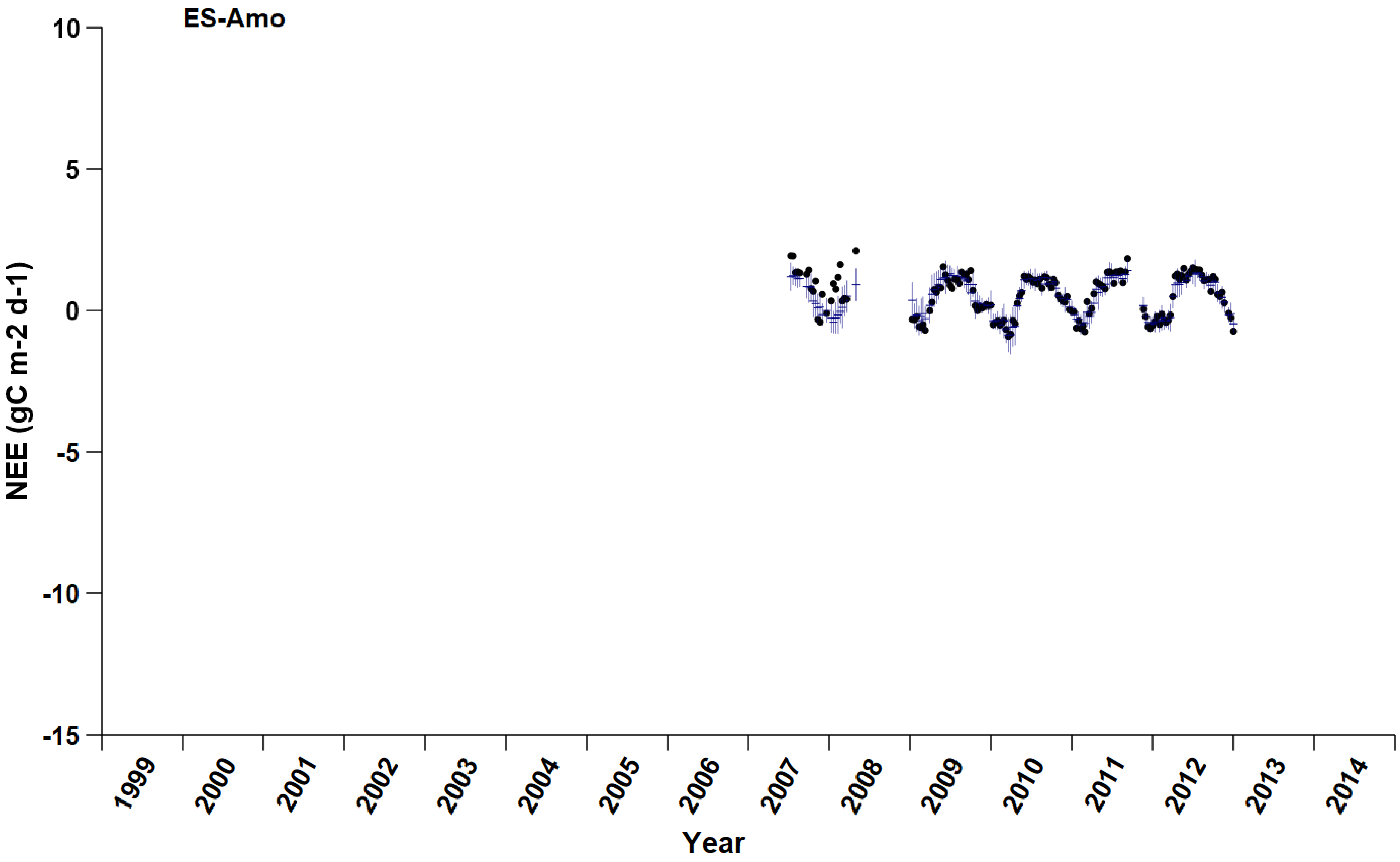
ES-Amo
NEE (gC m-2 d-1)
10
5
0
-5
-10
-15
1999
2000
2001
2002
2003
2004
2005
2006
2007
2008
2009
2010
2011
2012
2013
2014
Year

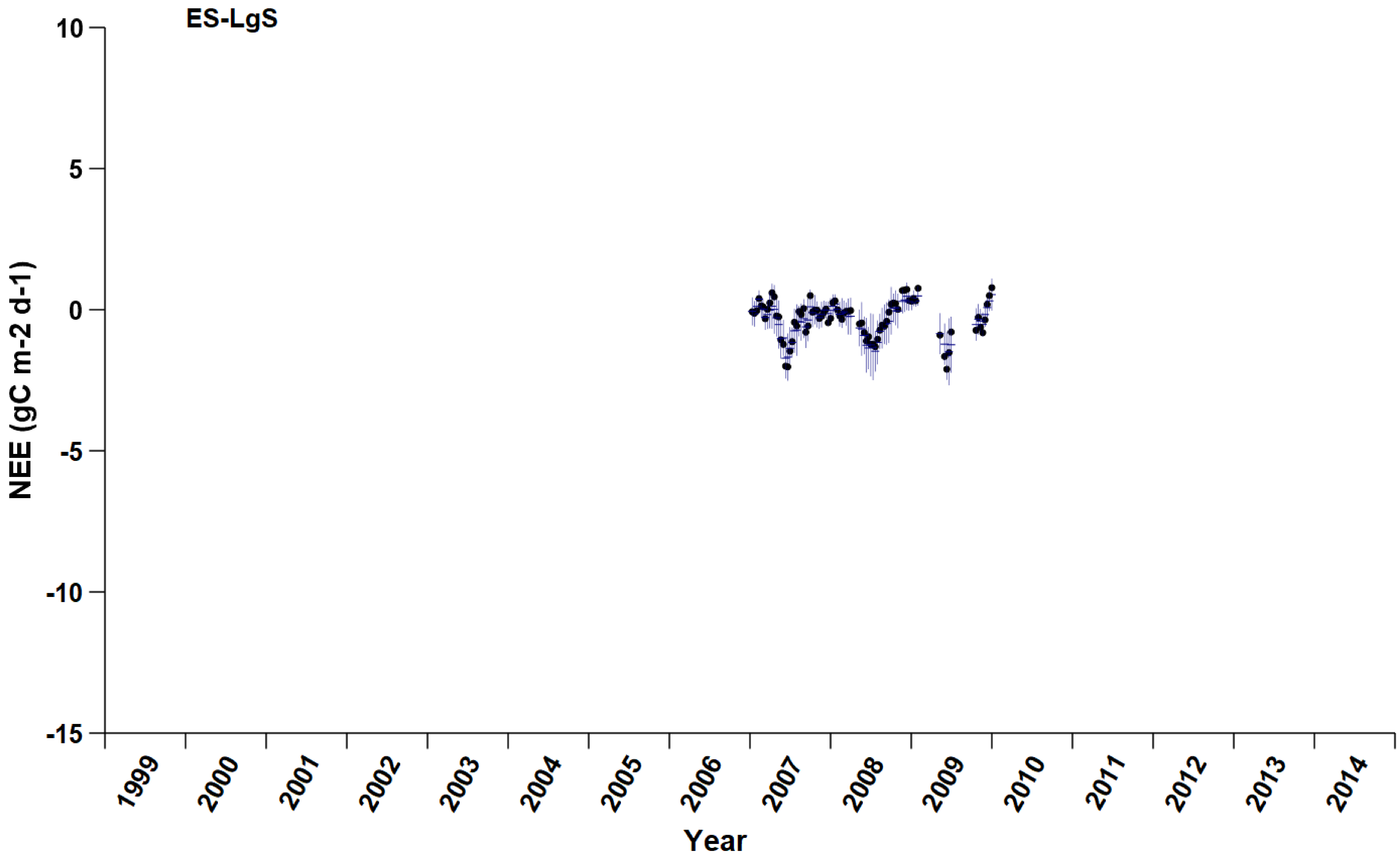

ES-LgS
10
5
0
-5
-10
-15
NEE (gC m-2 d-1)
1999
2000
2001
2002
2003
2004
2005
2006
2007
2008
2009
2010
2011
2012
2013
2014
Year

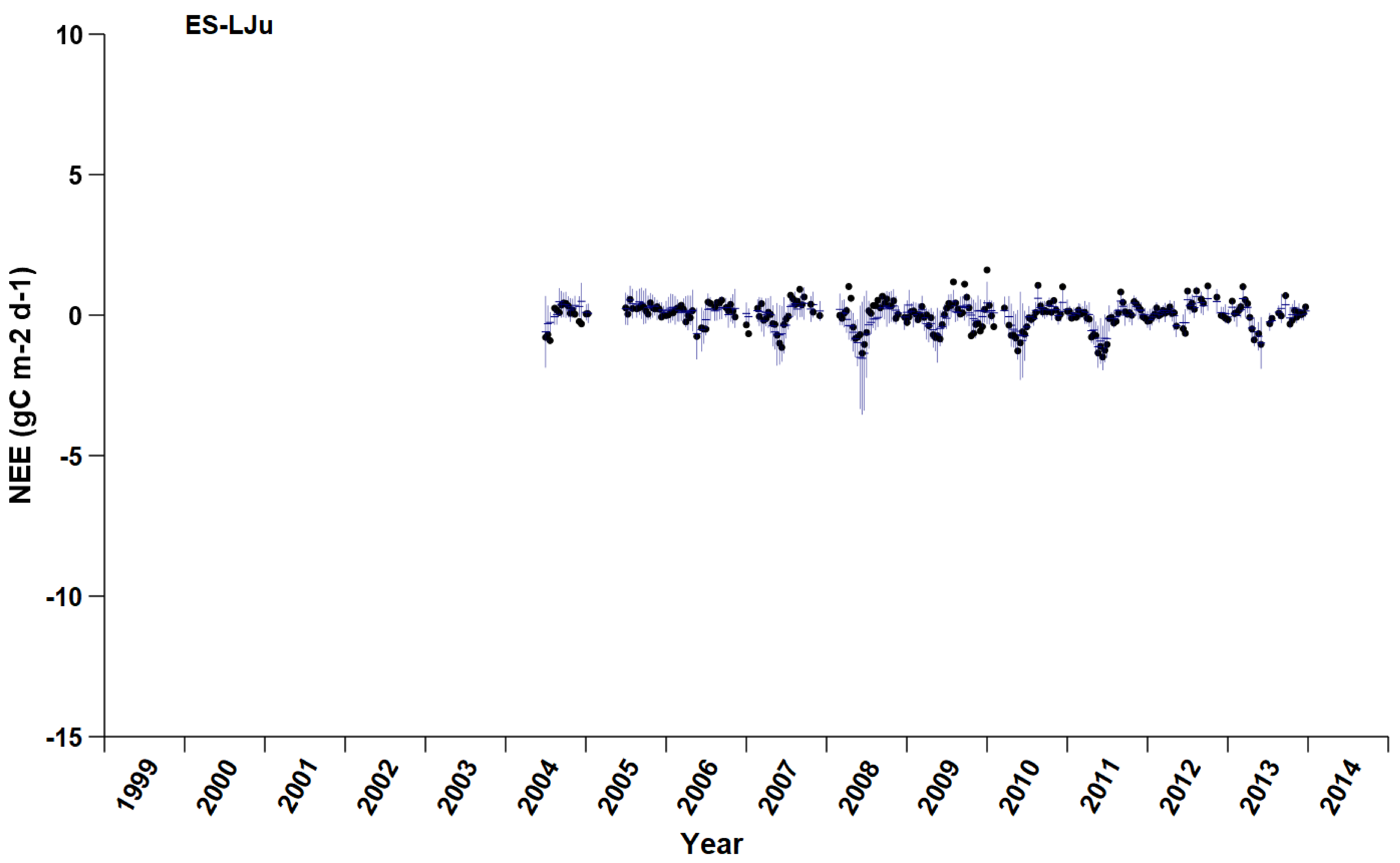

ES-LJu
NEE (gC m-2 d-1)
10
5
0
-5
-10
-15
1999
2000
2001
2002
2003
2004
2005
2006
2007
2008
2009
2010
2011
2012
2013
2014
Year

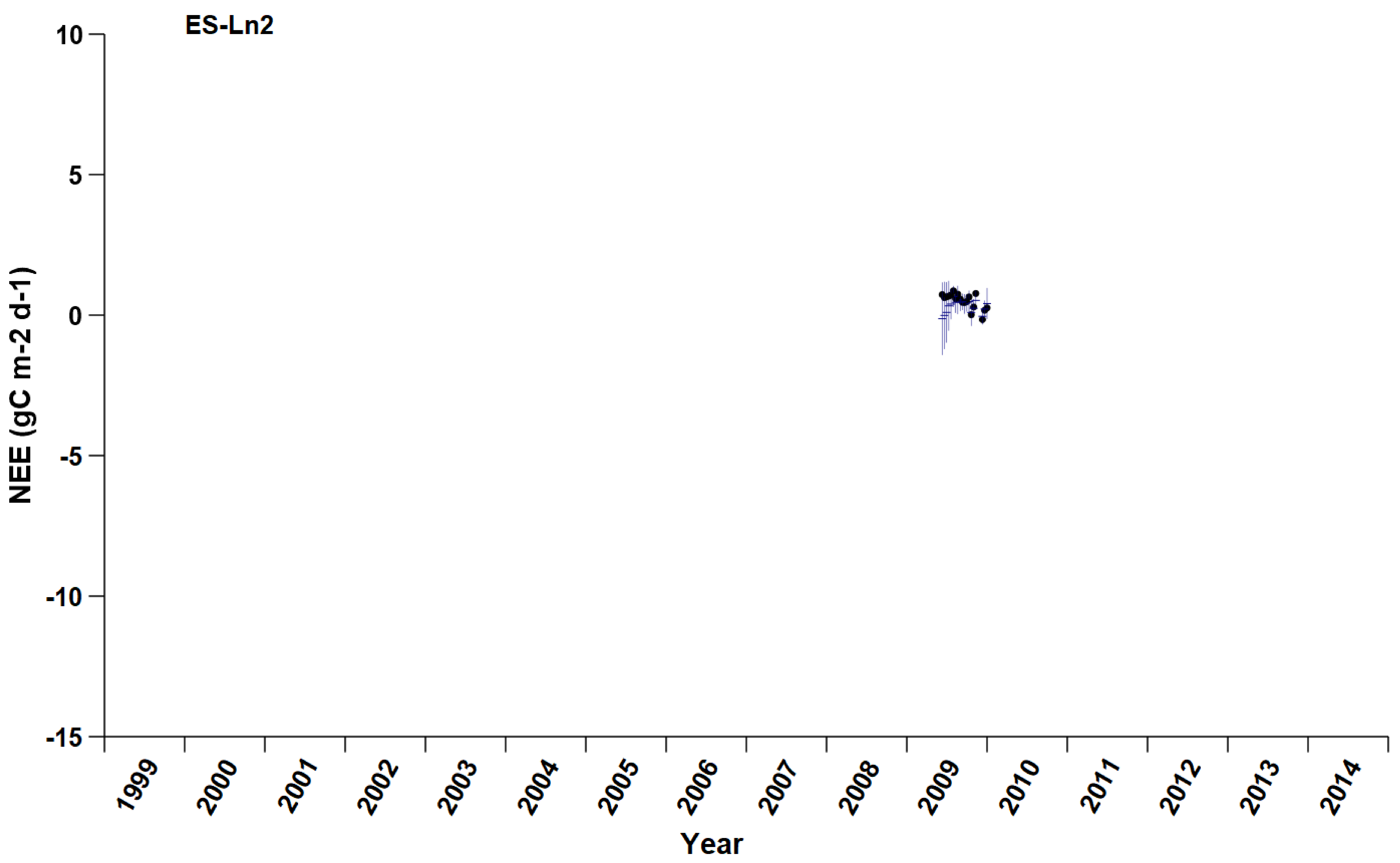
ES-Ln2
NEE (gC m-2 d-1)
10
5
0
-5
-10
-15
1999
2000
2001
2002
2003
2004
2005
2006
2007
2008
2009
2010
2011
2012
2013
2014
Year

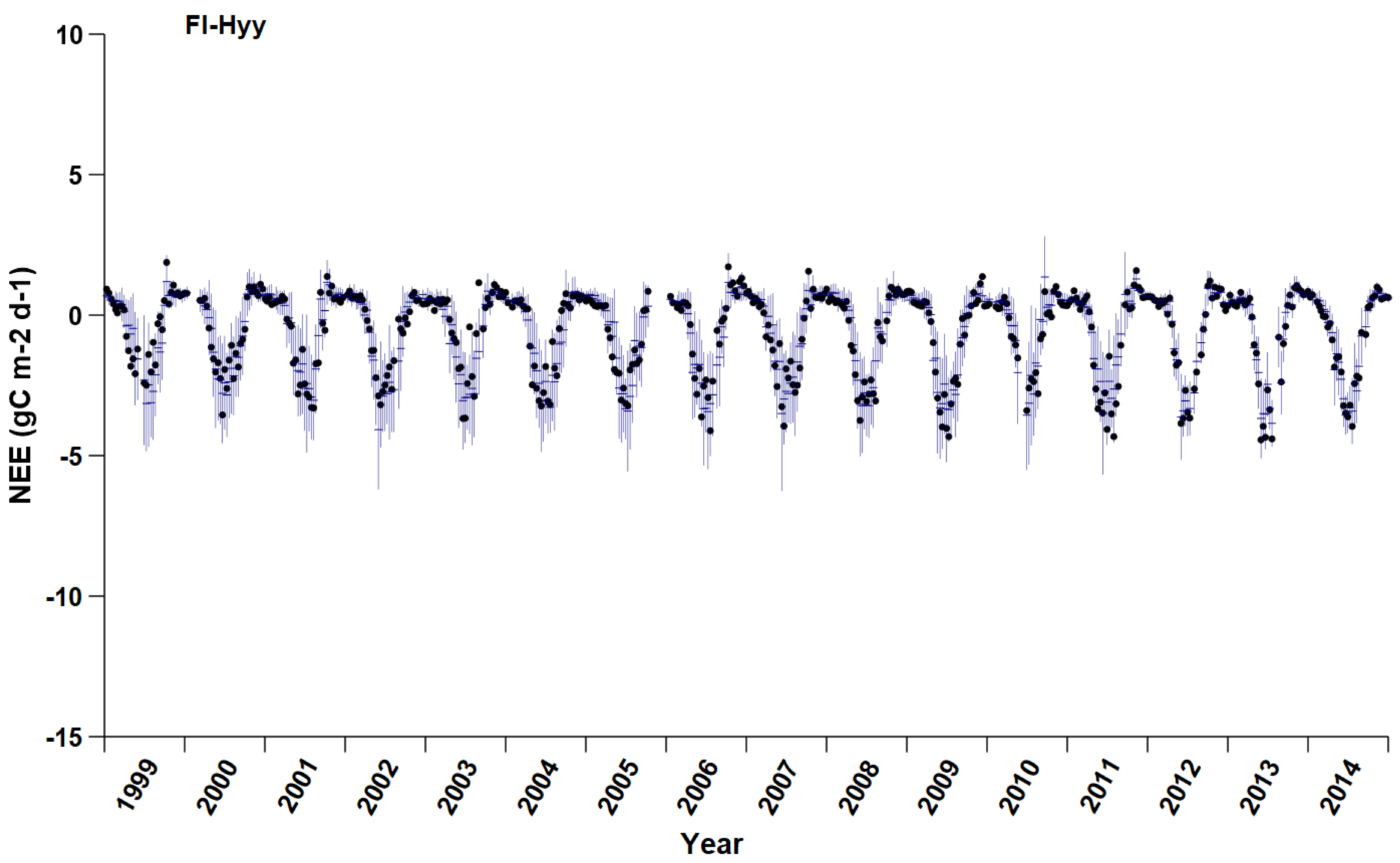
FI-Hyy
NEE (gC m-2 d-1)
10
5
0
-5
-10
-15
1999
2000
2001
2002
2003
2004
2005
2006
2007
2008
2009
2010
2011
2012
2013
2014
Year

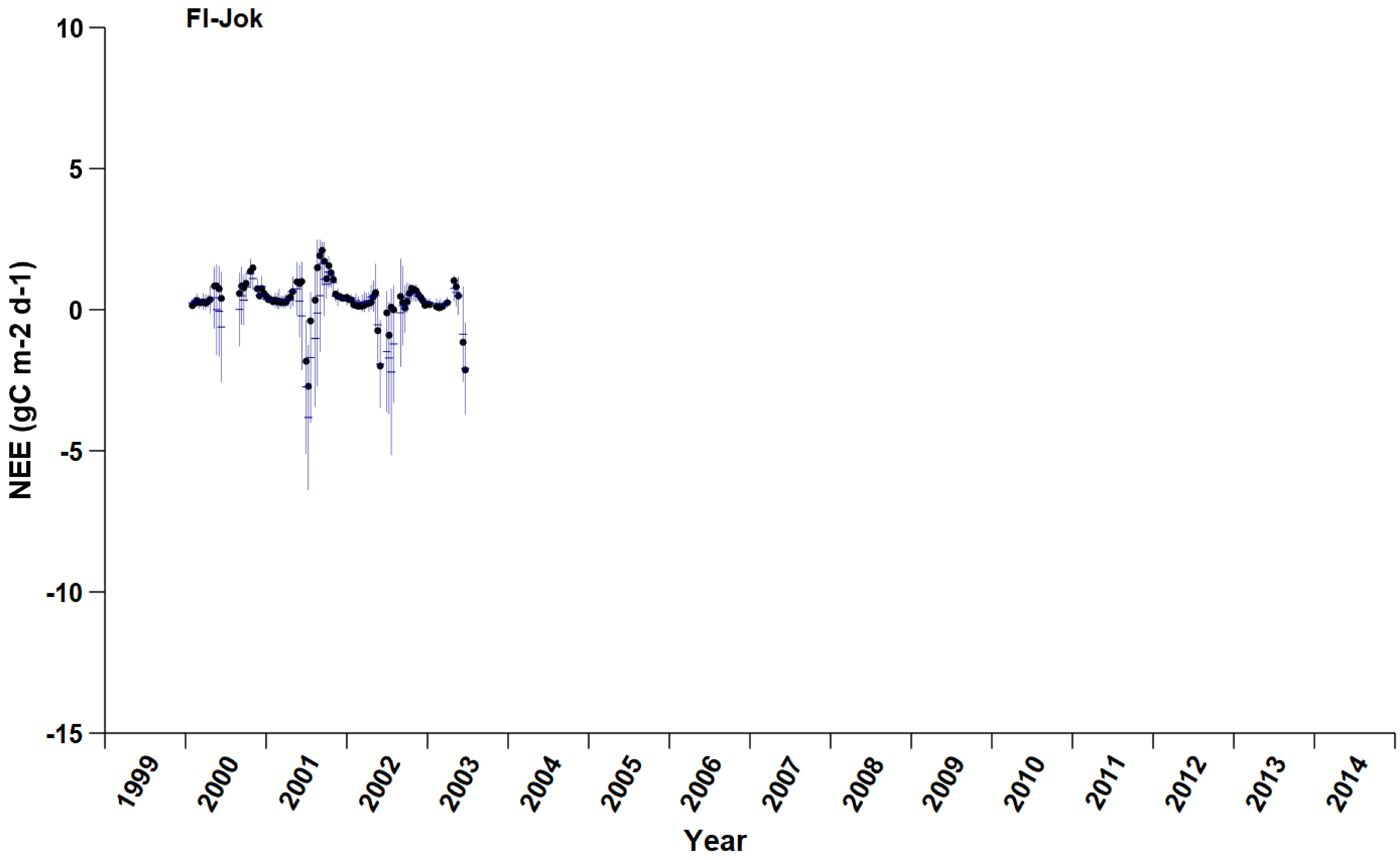
FI-Jok
10
5
0
-5
-10
-15
NEE (gC m-2 d-1)
1999
2000
2001
2002
2003
2004
2005
2006
2007
2008
2009
2010
2011
2012
2013
2014
Year

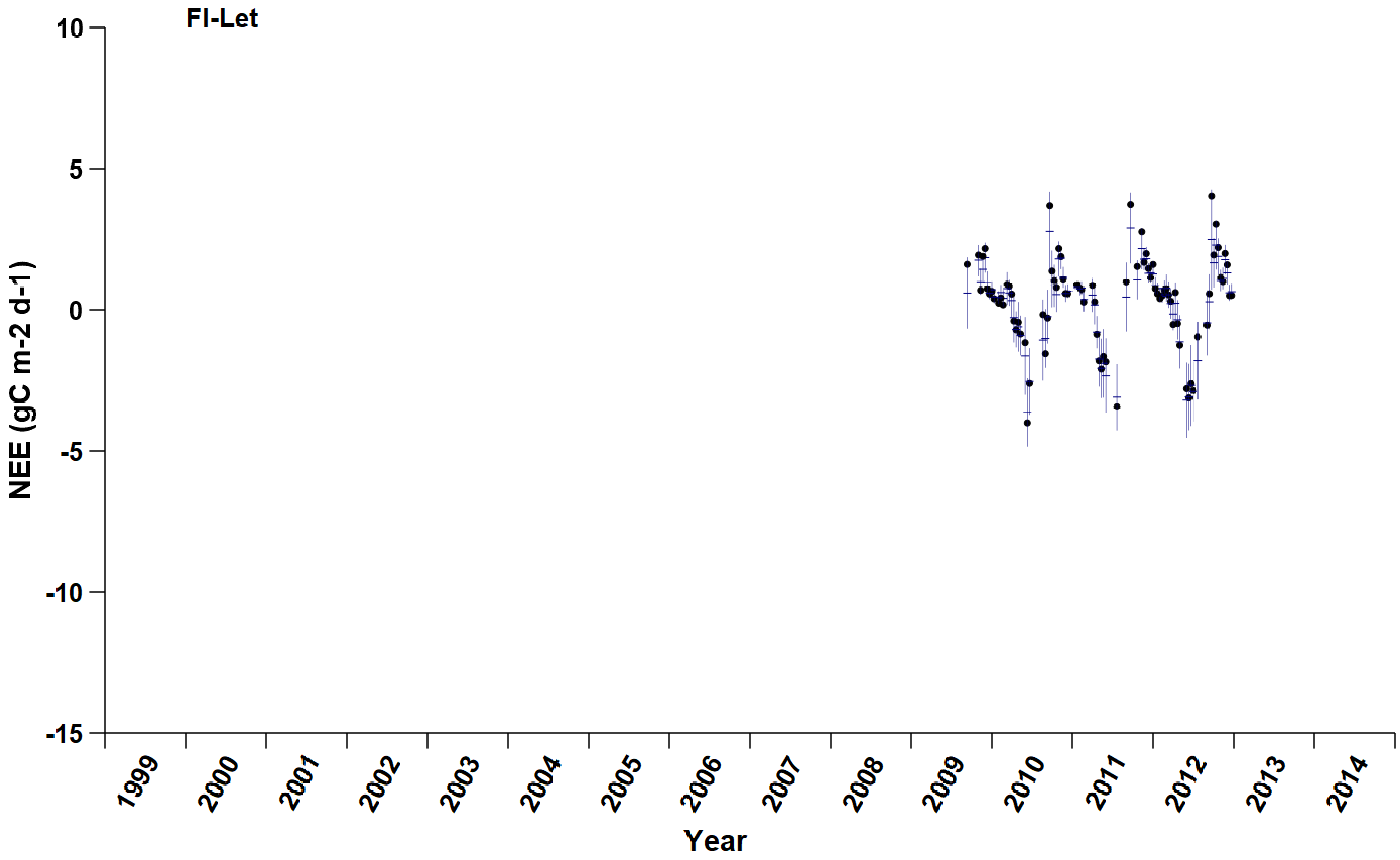
FI-Let
NEE (gC m-2 d-1)
10
5
0
-5
-10
-15
1999
2000
2001
2002
2003
2004
2005
2006
2007
2008
2009
2010
2011
2012
2013
2014
Year

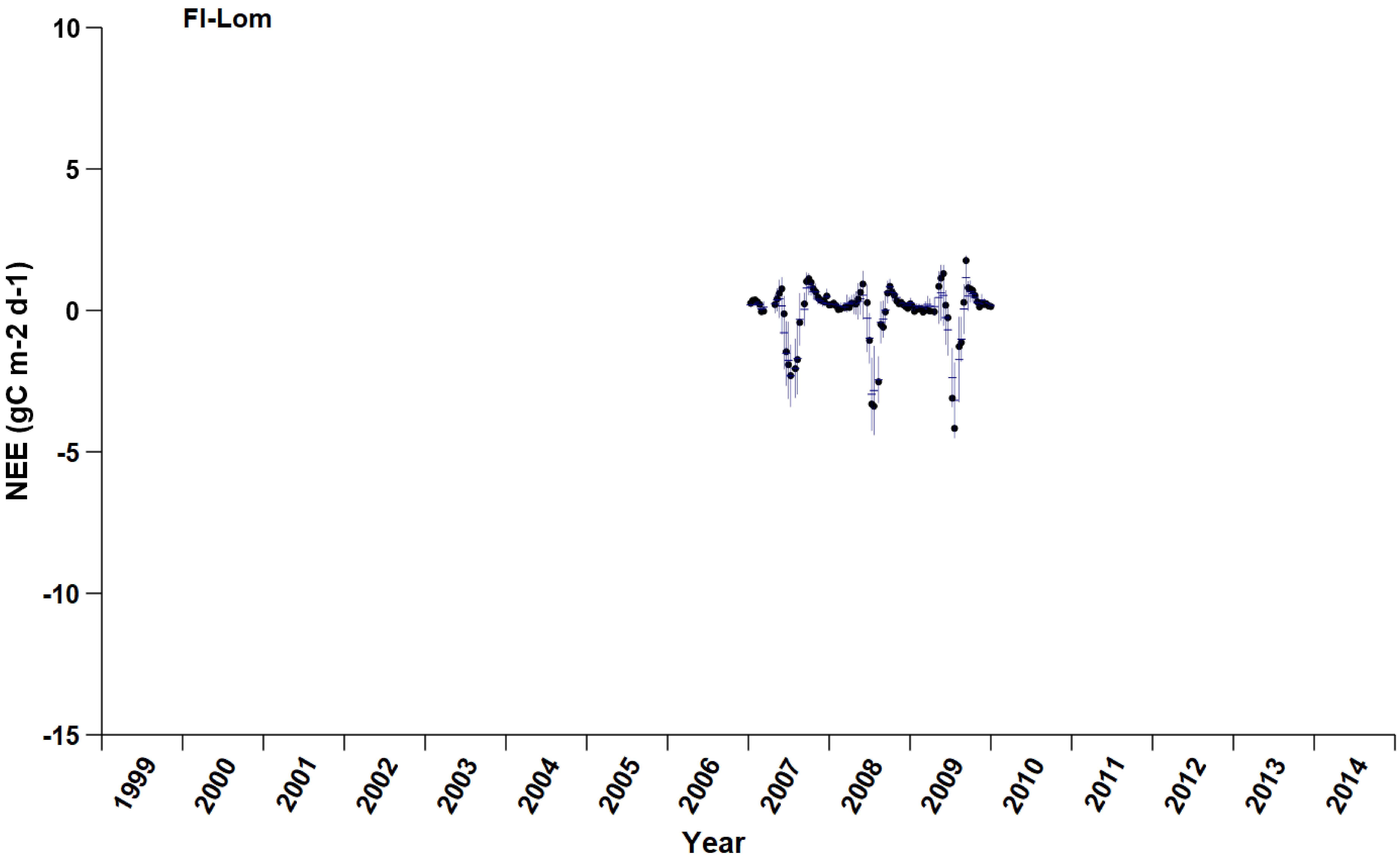

FI-Lom
NEE (gC m-2 d-1)
10
5
0
-5
-10
-15
1999
2000
2001
2002
2003
2004
2005
2006
2007
2008
2009
2010
2011
2012
2013
2014
Year

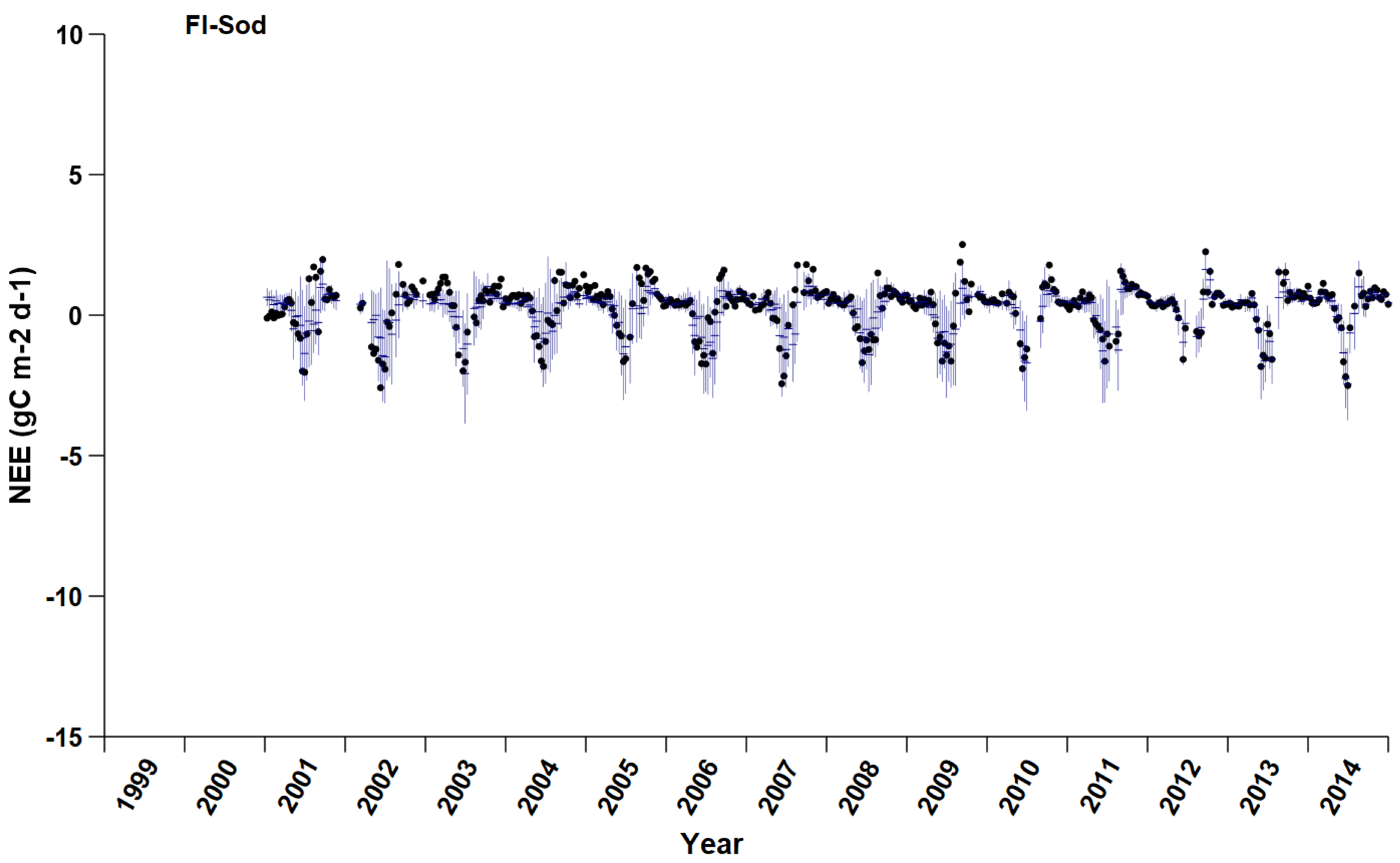

FI-Sod
NEE (gC m-2 d-1)
10
5
0
-5
-10
-15
1999
2000
2001
2002
2003
2004
2005
2006
2007
2008
2009
2010
2011
2012
2013
2014
Year

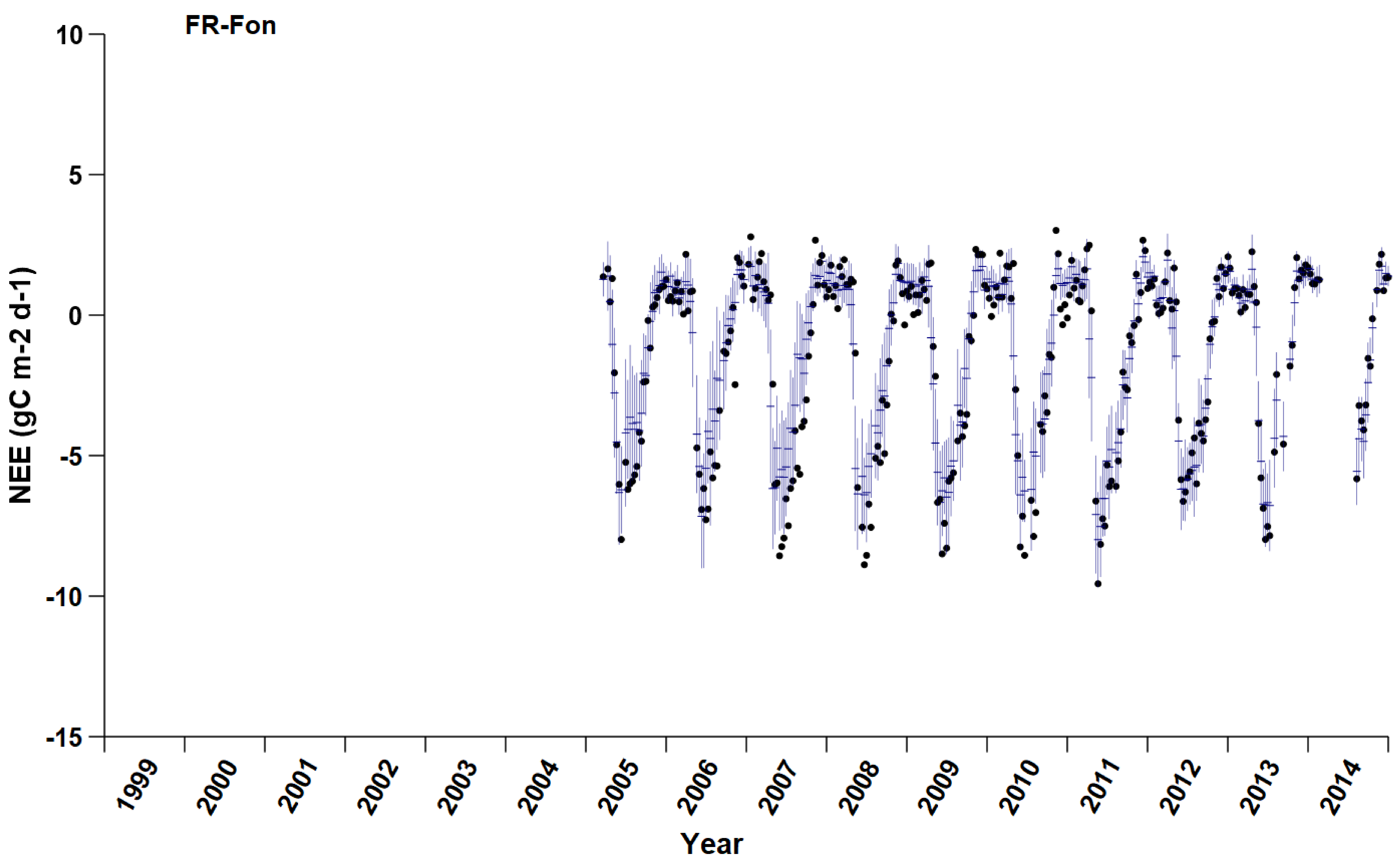

FR-Fon
10
5
0
-5
-10
-15
NEE (gC m-2 d-1)
1999
2000
2001
2002
2003
2004
2005
2006
2007
2008
2009
2010
2011
2012
2013
2014
Year

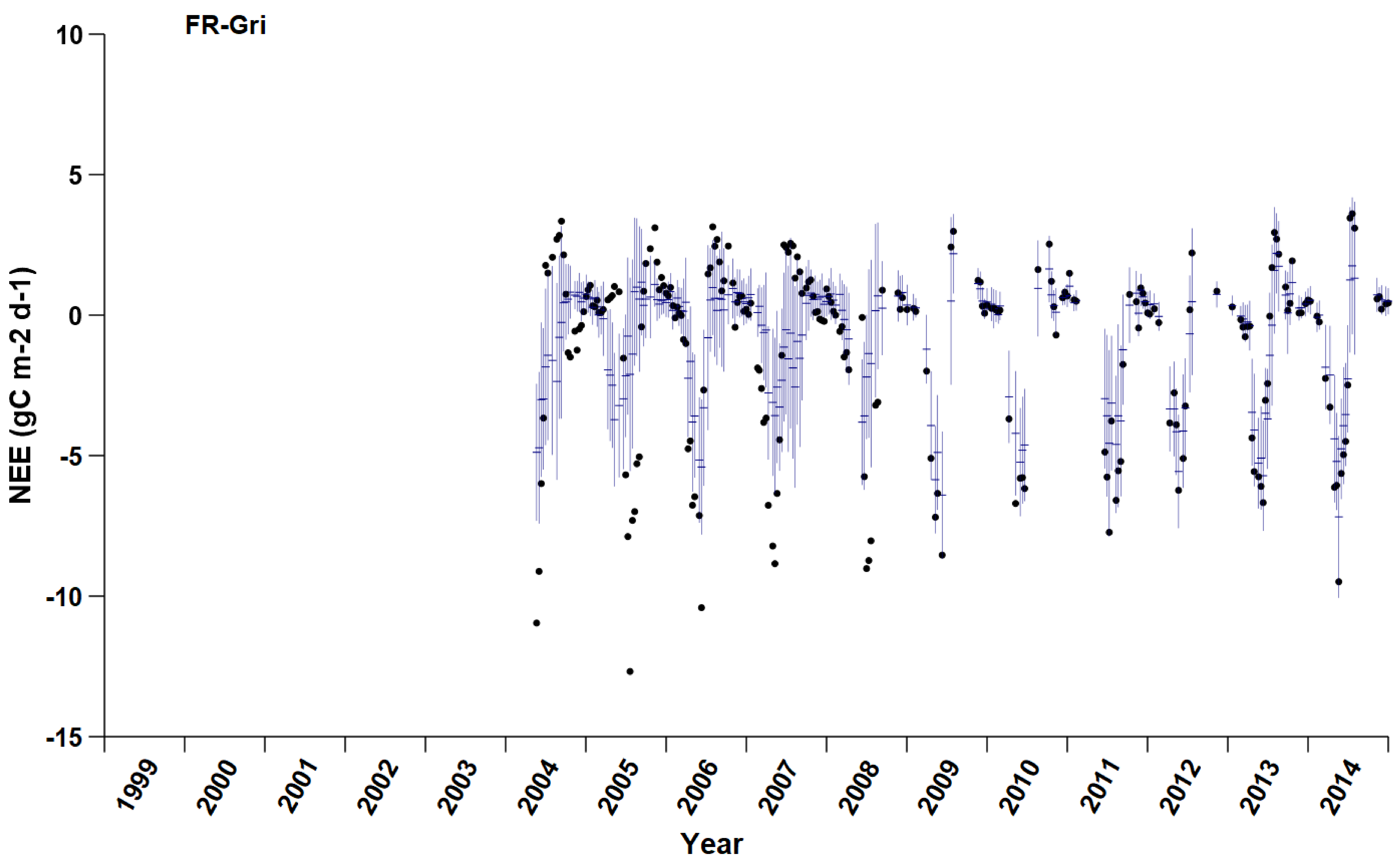
FR-Gri
NEE (gC m-2 d-1)
10
5
0
-5
-10
-15
1999
2000
2001
2002
2003
2004
2005
2006
2007
2008
2009
2010
2011
2012
2013
2014
Year

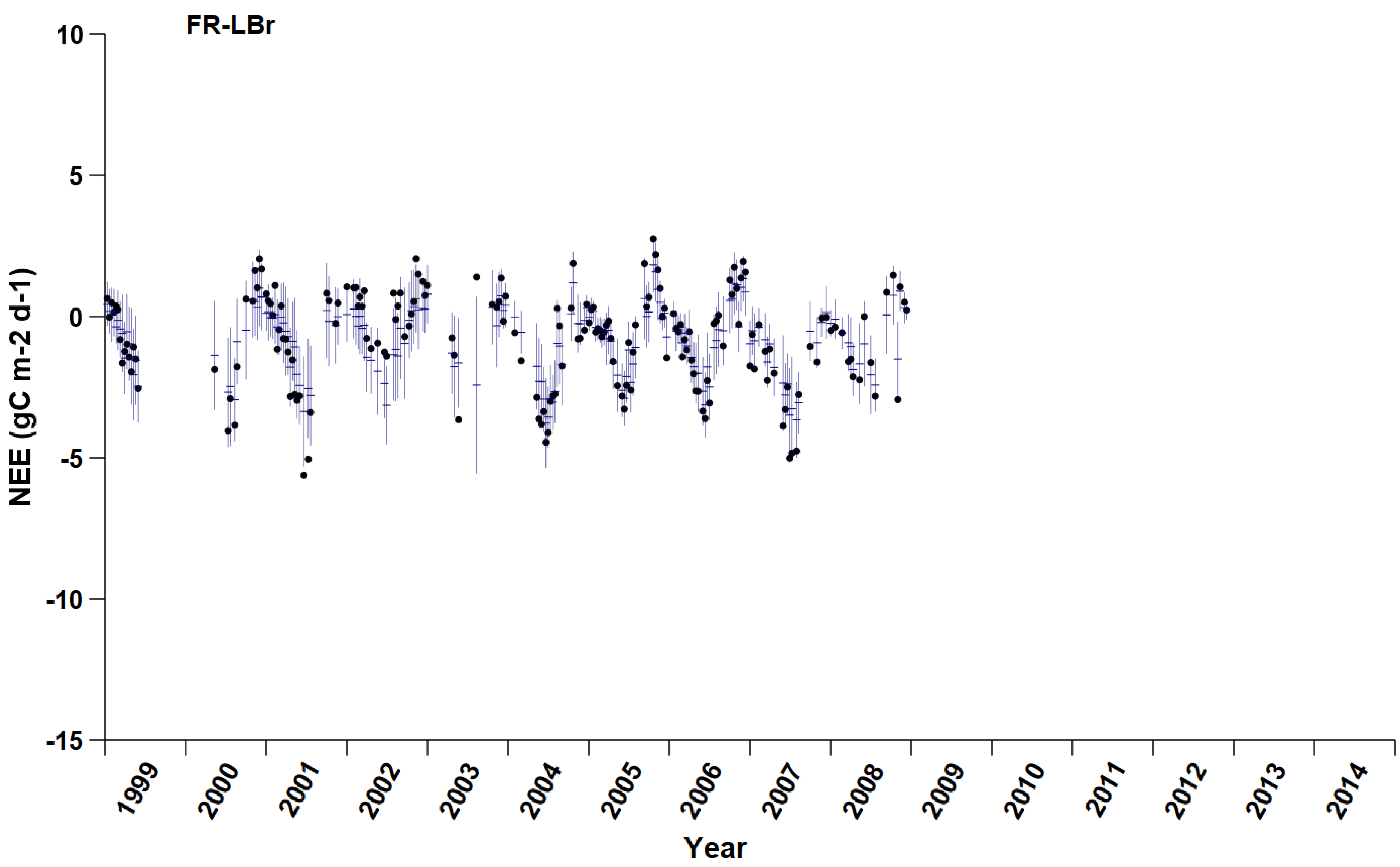
FR-LBr
NEE (gC m-2 d-1)
10
5
0
-5
-10
-15
1999
2000
2001
2002
2003
2004
2005
2006
2007
2008
2009
2010
2011
2012
2013
2014
Year

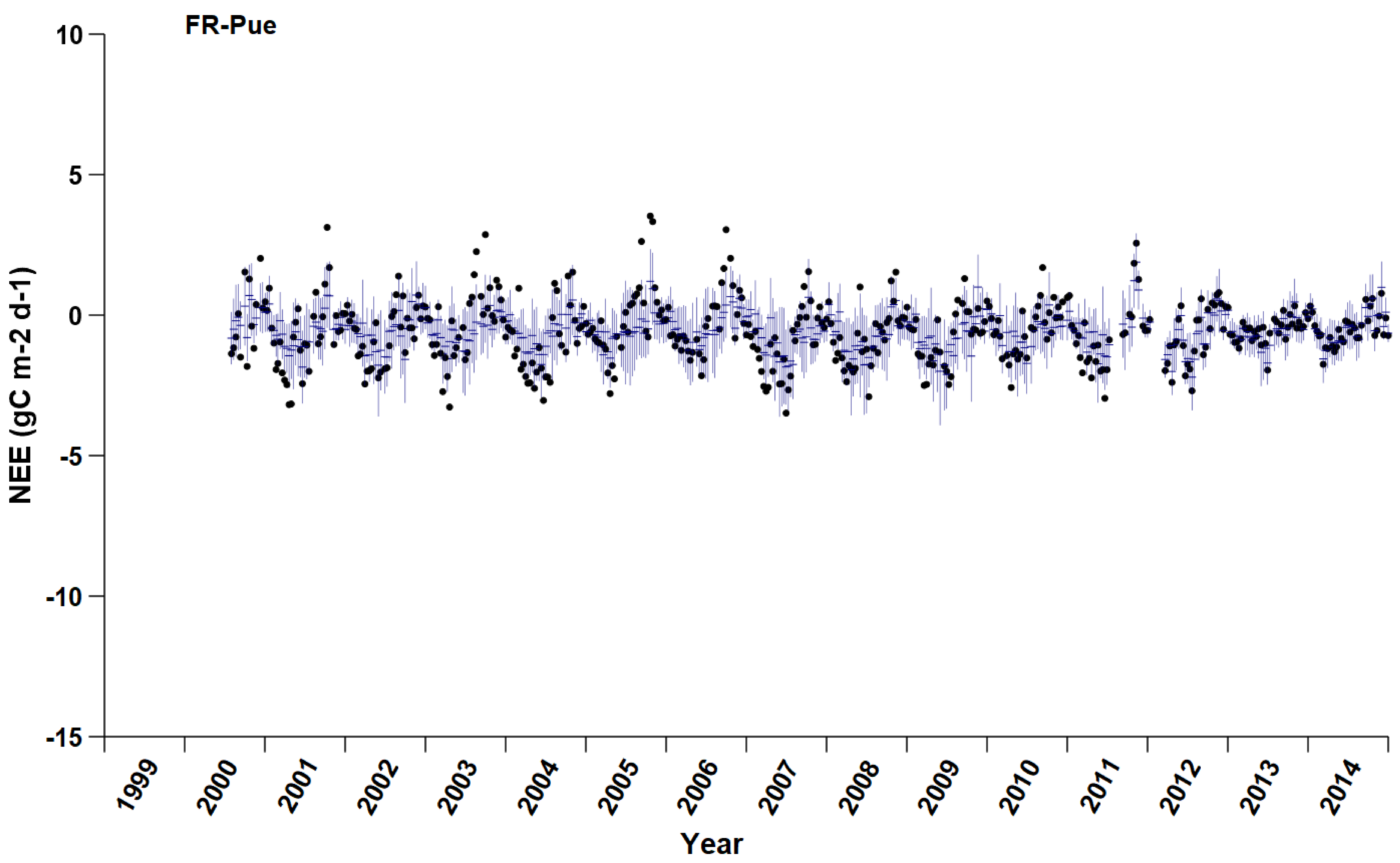

FR-Pue
10
5
0
-5
-10
-15
NEE (gC m-2 d-1)
1999
2000
2001
2002
2003
2004
2005
2006
2007
2008
2009
2010
2011
2012
2013
2014
Year

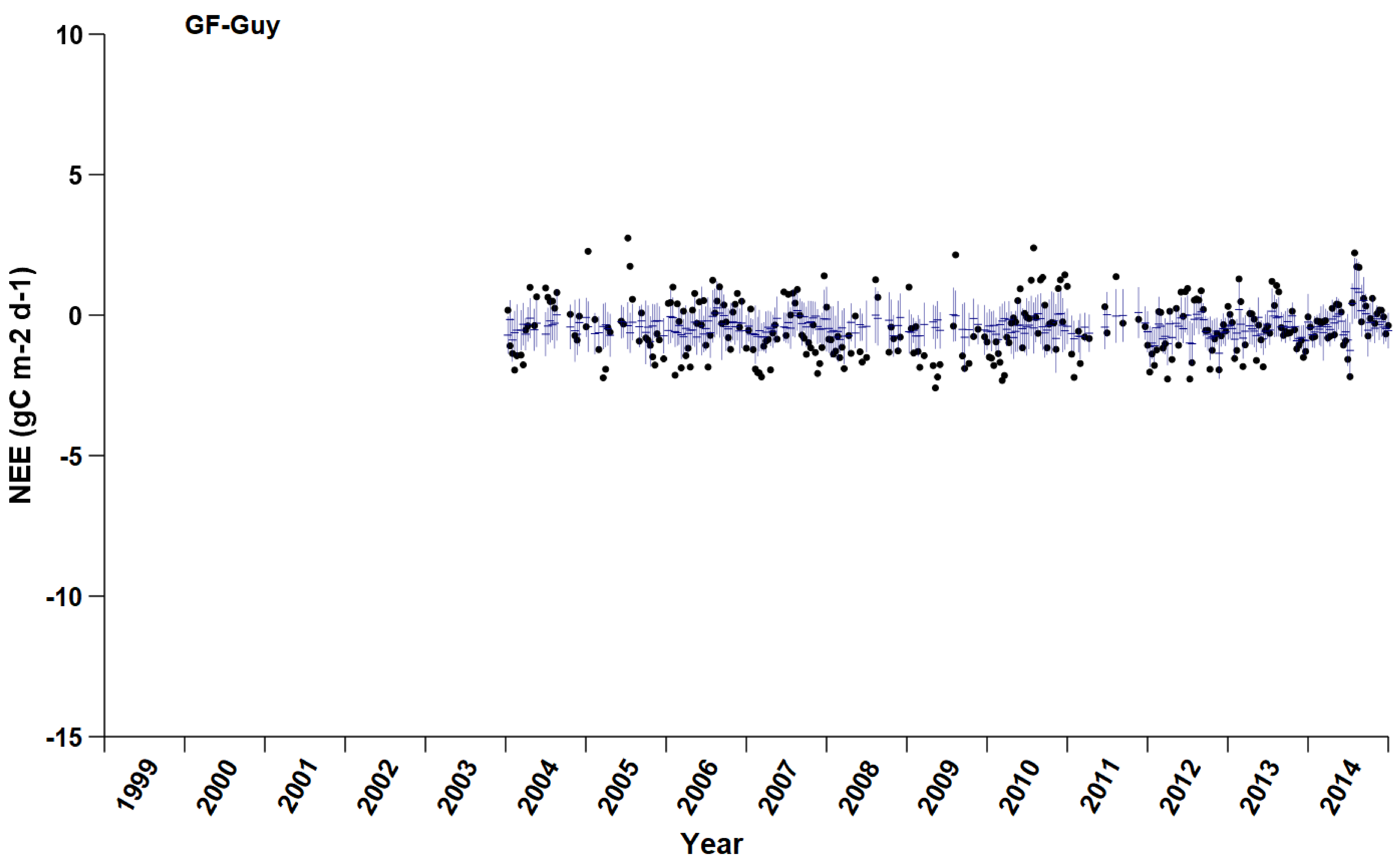
GF-Guy
10
5
0
-5
-10
-15
NEE (gC m-2 d-1)
1999
2000
2001
2002
2003
2004
2005
2006
2007
2008
2009
2010
2011
2012
2013
2014
Year

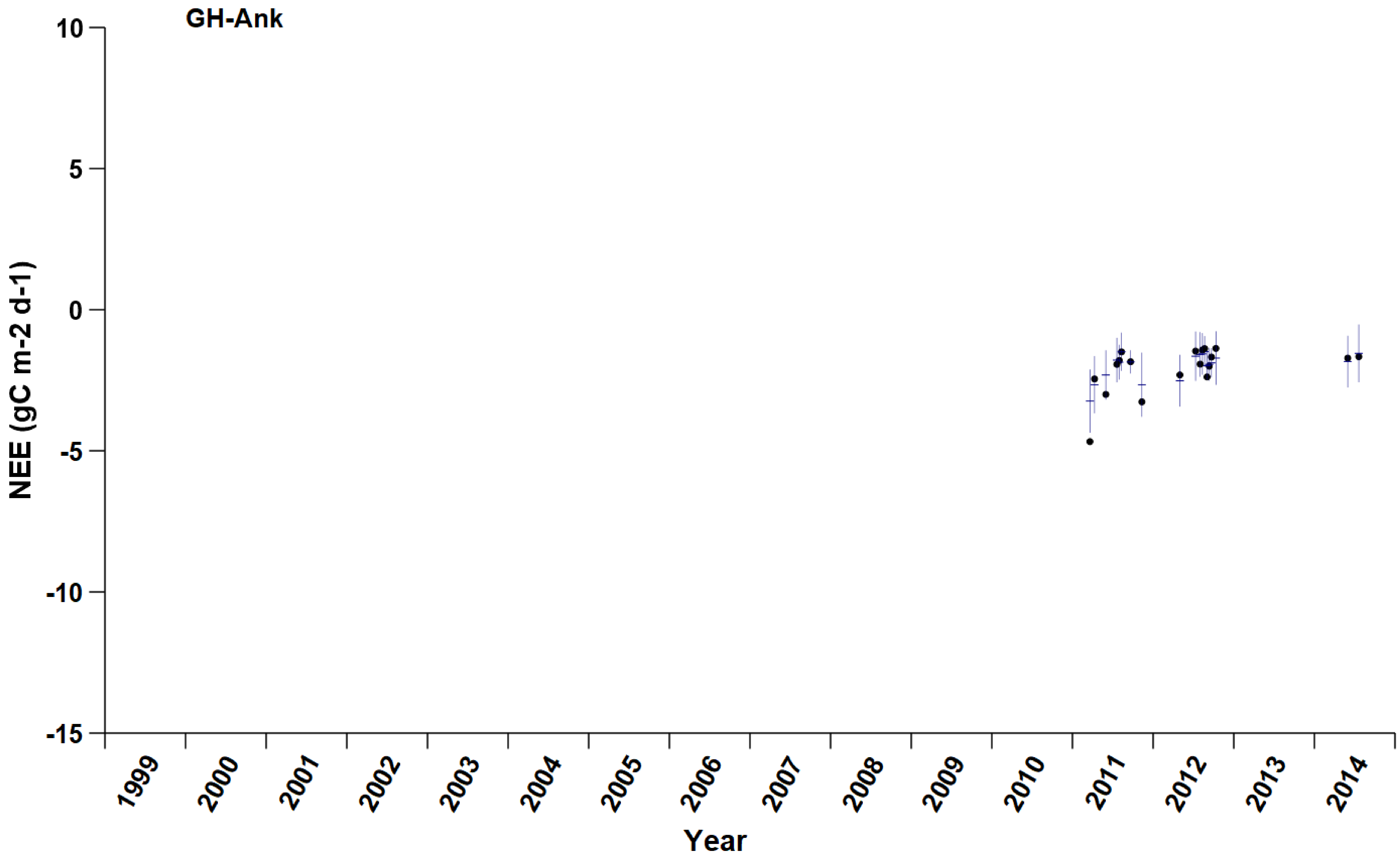

GH-Ank
10
5
0
-5
-10
-15
NEE (gC m-2 d-1)
1999
2000
2001
2002
2003
2004
2005
2006
2007
2008
2009
2010
2011
2012
2013
2014
Year

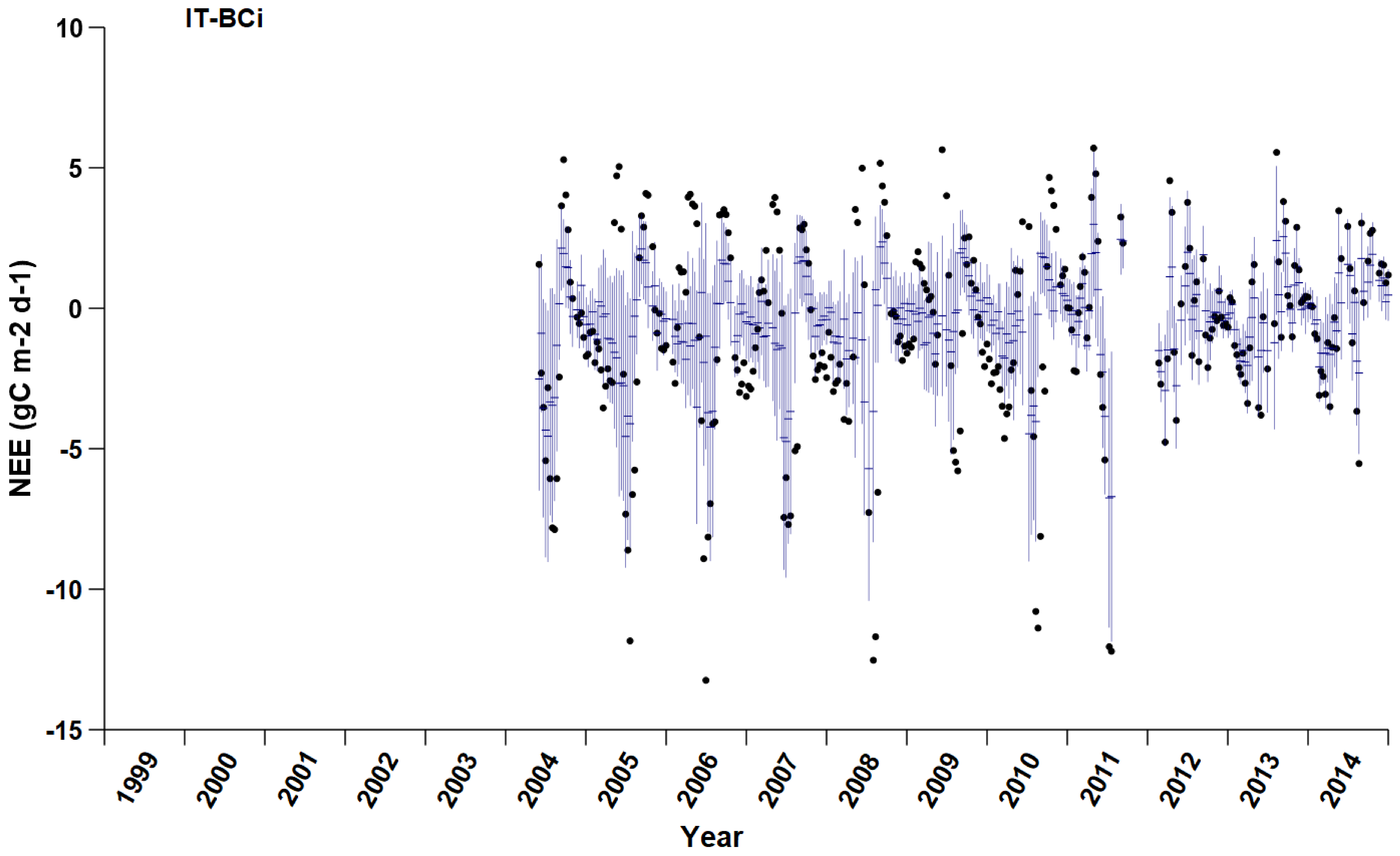
IT-BCi
NEE (gC m-2 d-1)
10
5
0
-5
-10
-15
1999
2000
2001
2002
2003
2004
2005
2006
2007
2008
2009
2010
2011
2012
2013
2014
Year

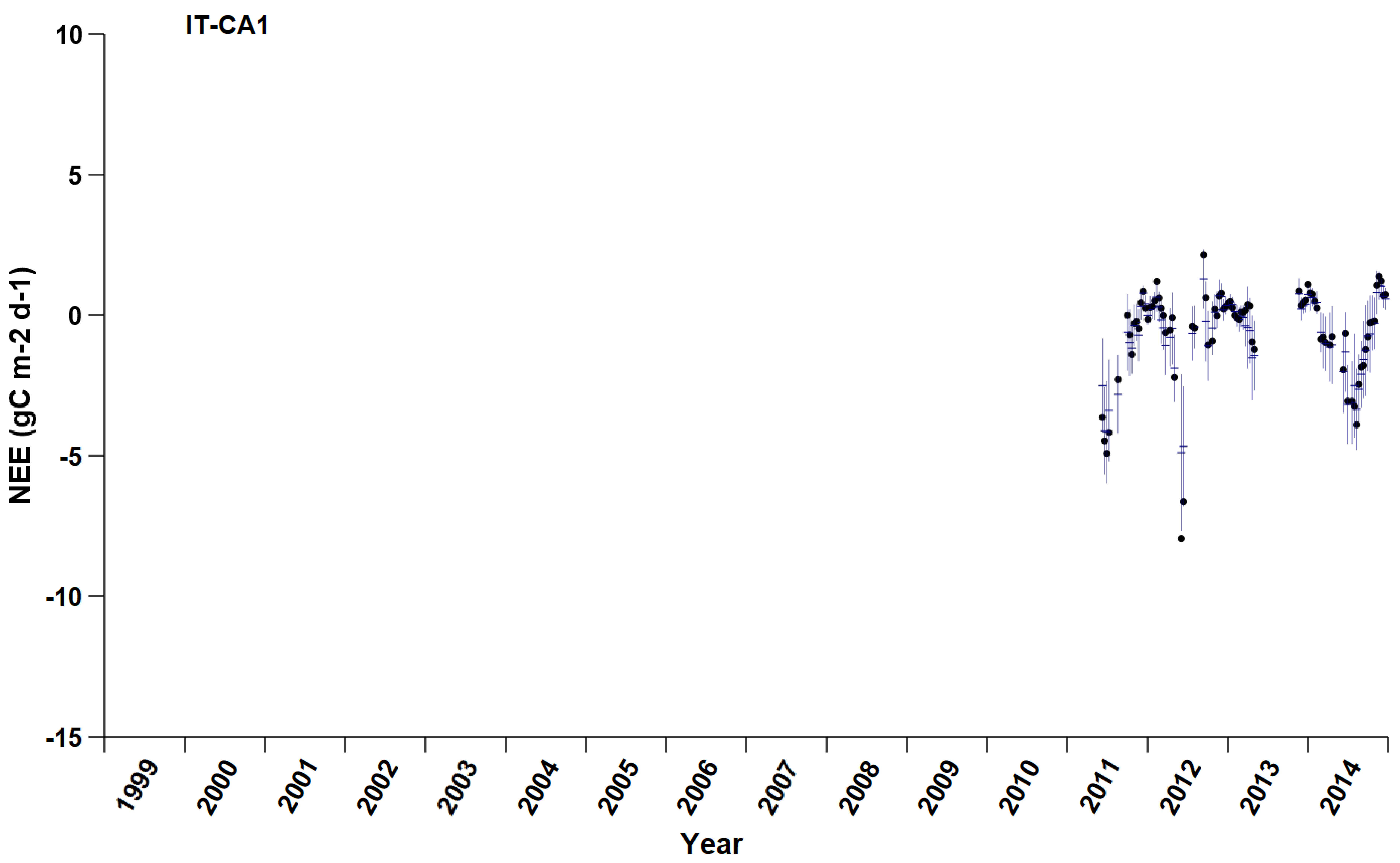
IT-CA1
NEE (gC m-2 d-1)
10
5
0
-5
-10
-15
1999
2000
2001
2002
2003
2004
2005
2006
2007
2008
2009
2010
2011
2012
2013
2014
Year

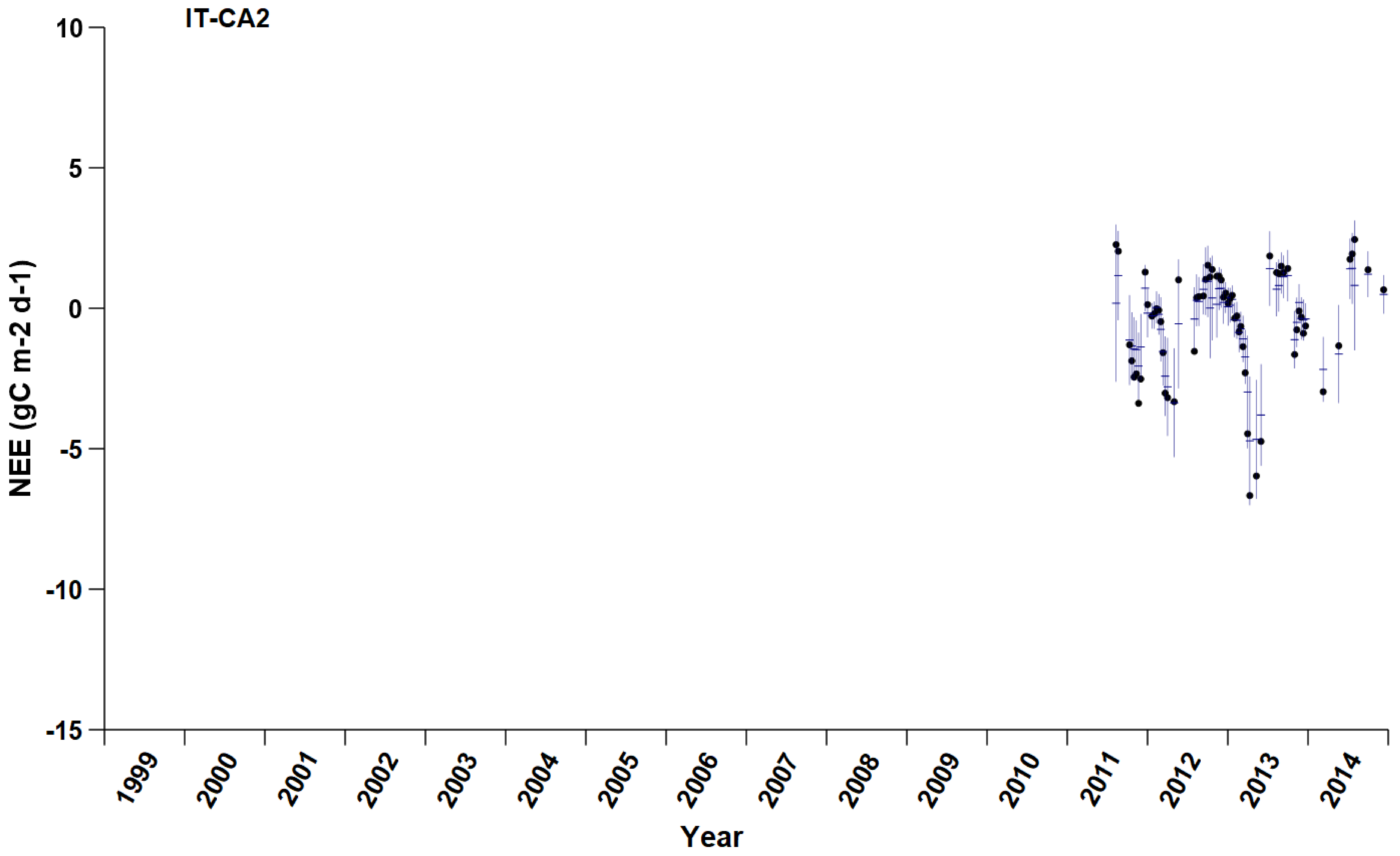

IT-CA2
10
5
0
-5
-10
-15
NEE (gC m-2 d-1)
1999
2000
2001
2002
2003
2004
2005
2006
2007
2008
2009
2010
2011
2012
2013
2014
Year

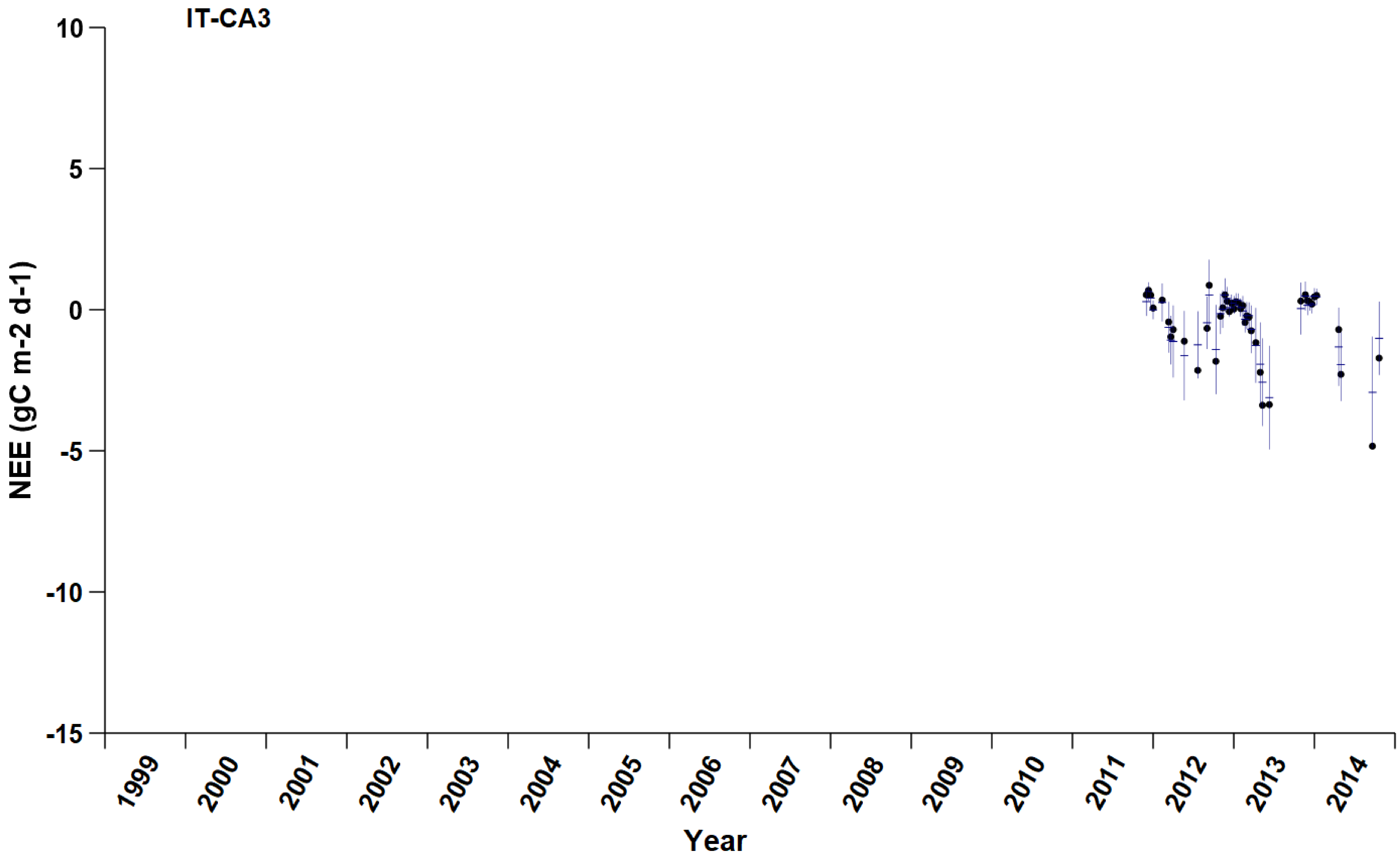
IT-CA3
NEE (gC m-2 d-1)
10
5
0
-5
-10
-15
1999
2000
2001
2002
2003
2004
2005
2006
2007
2008
2009
2010
2011
2012
2013
2014
Year

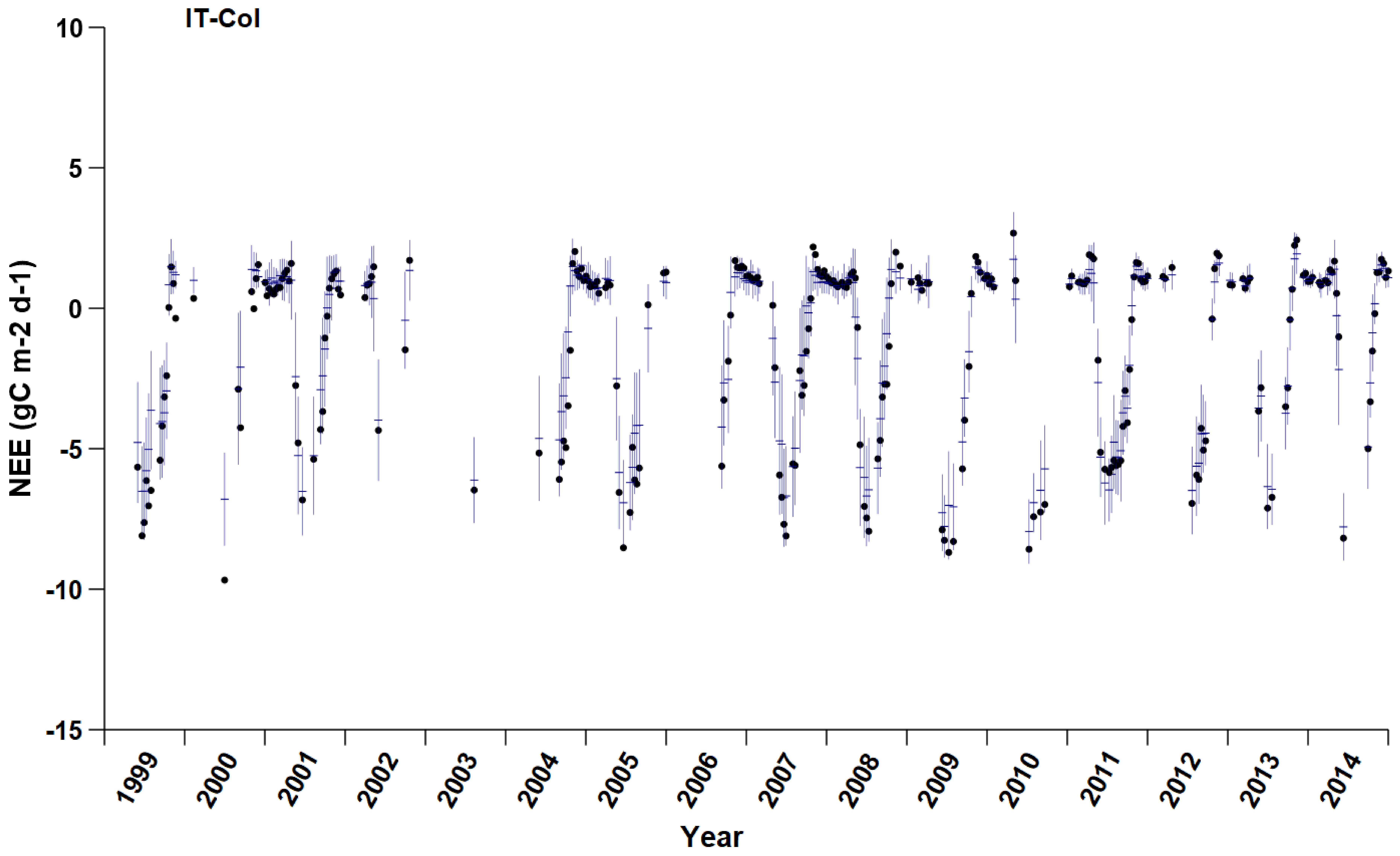

IT-Col
NEE (gC m-2 d-1)
10
5
0
-5
-10
-15
1999
2000
2001
2002
2003
2004
2005
2006
2007
2008
2009
2010
2011
2012
2013
2014
Year

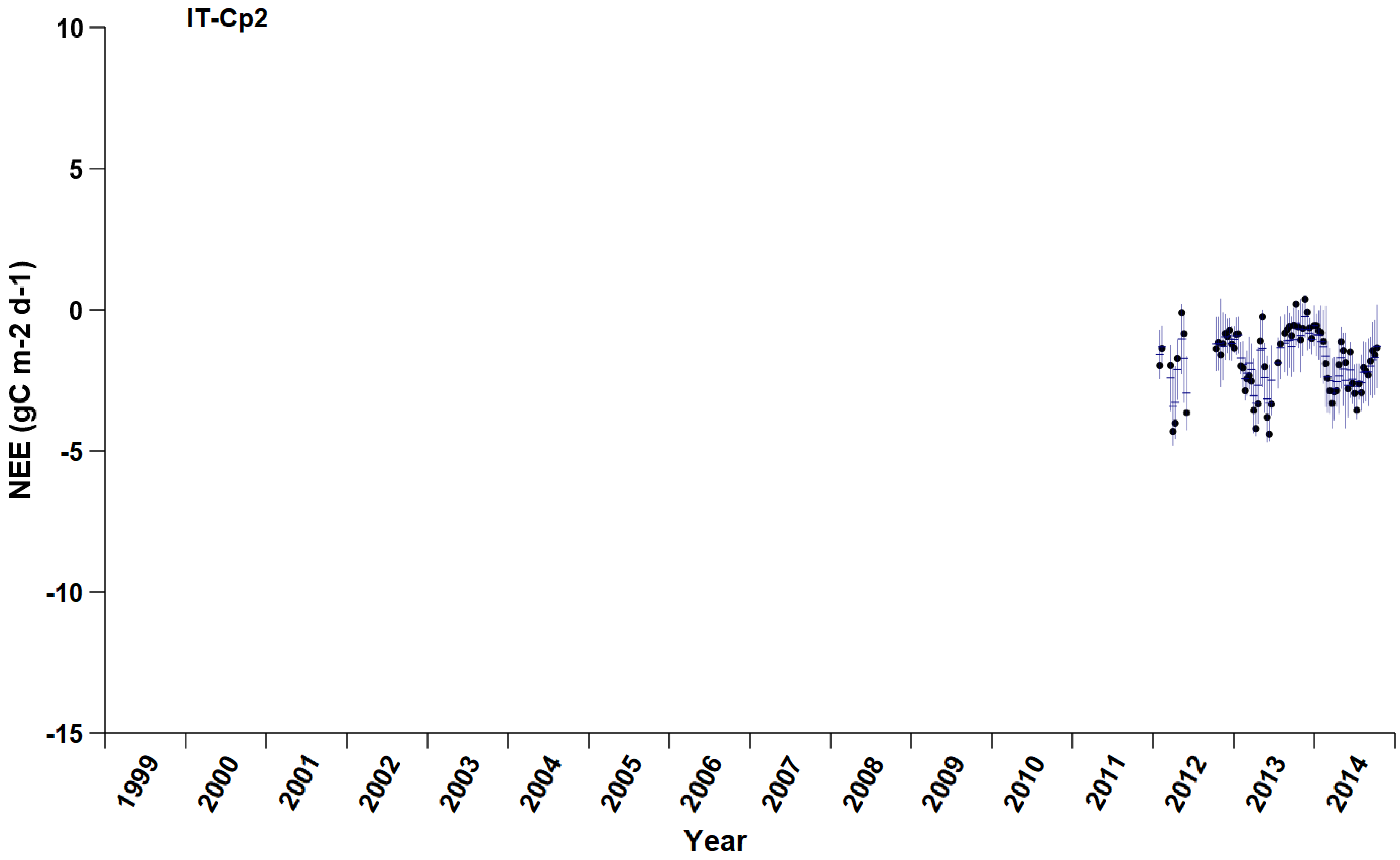
IT-Cp2
10
5
0
-5
-10
-15
NEE (gC m-2 d-1)
1999
2000
2001
2002
2003
2004
2005
2006
2007
2008
2009
2010
2011
2012
2013
2014
Year

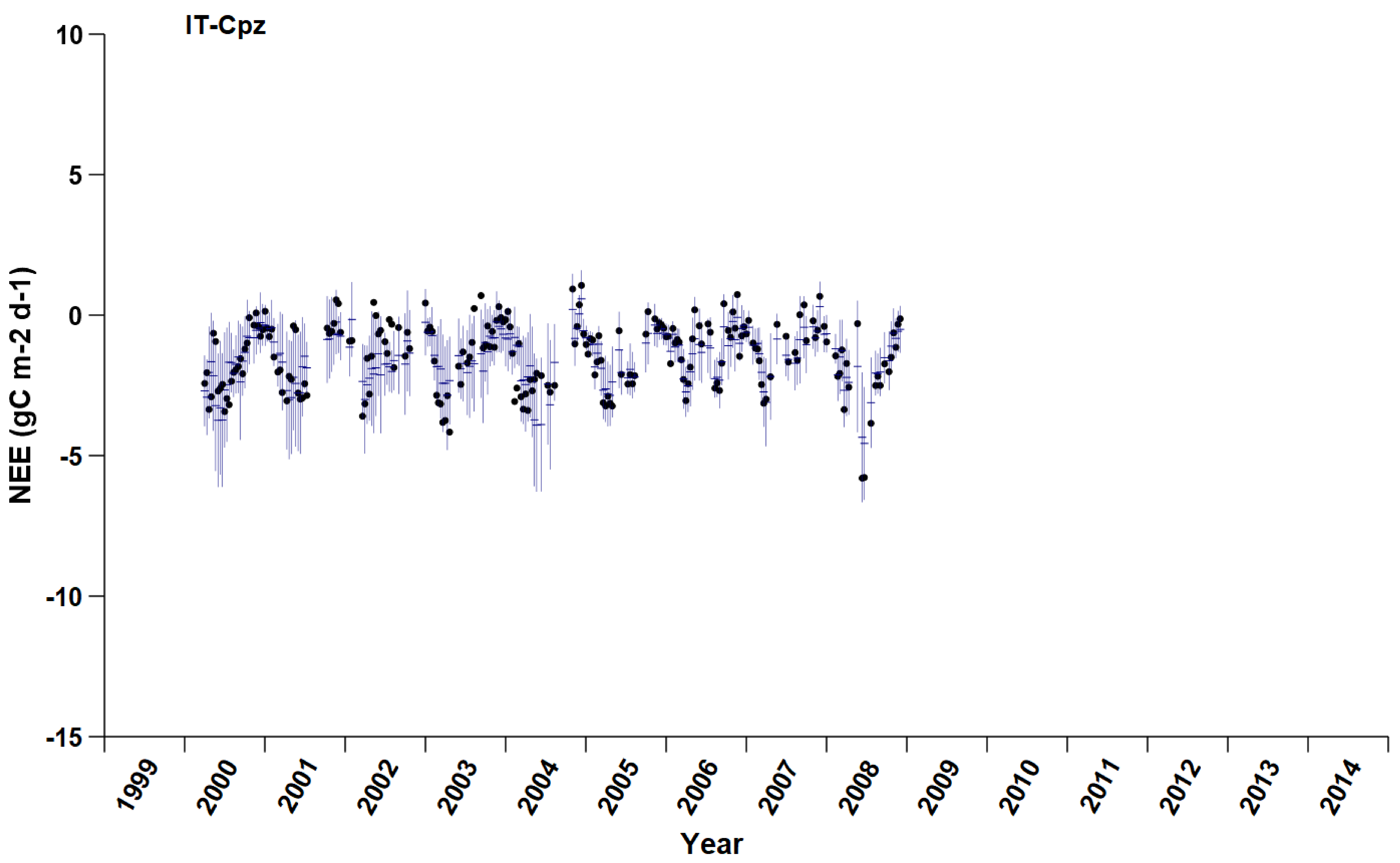

IT-Cpz
10
5
0
-5
-10
-15
NEE (gC m-2 d-1)
1999
2000
2001
2002
2003
2004
2005
2006
2007
2008
2009
2010
2011
2012
2013
2014
Year

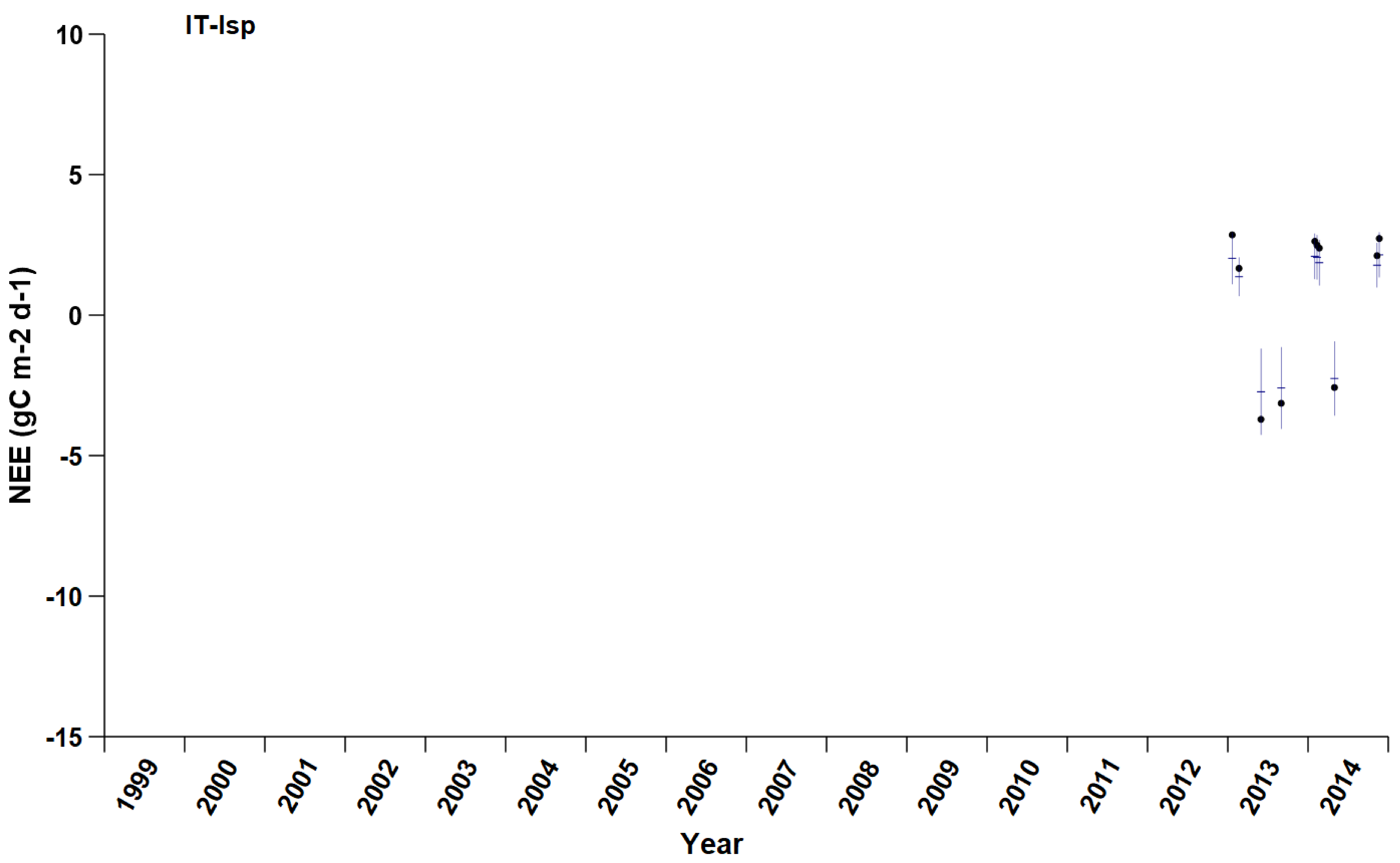

IT-Isp
NEE (gC m-2 d-1)
10
5
0
-5
-10
-15
1999
2000
2001
2002
2003
2004
2005
2006
2007
2008
2009
2010
2011
2012
2013
2014
Year

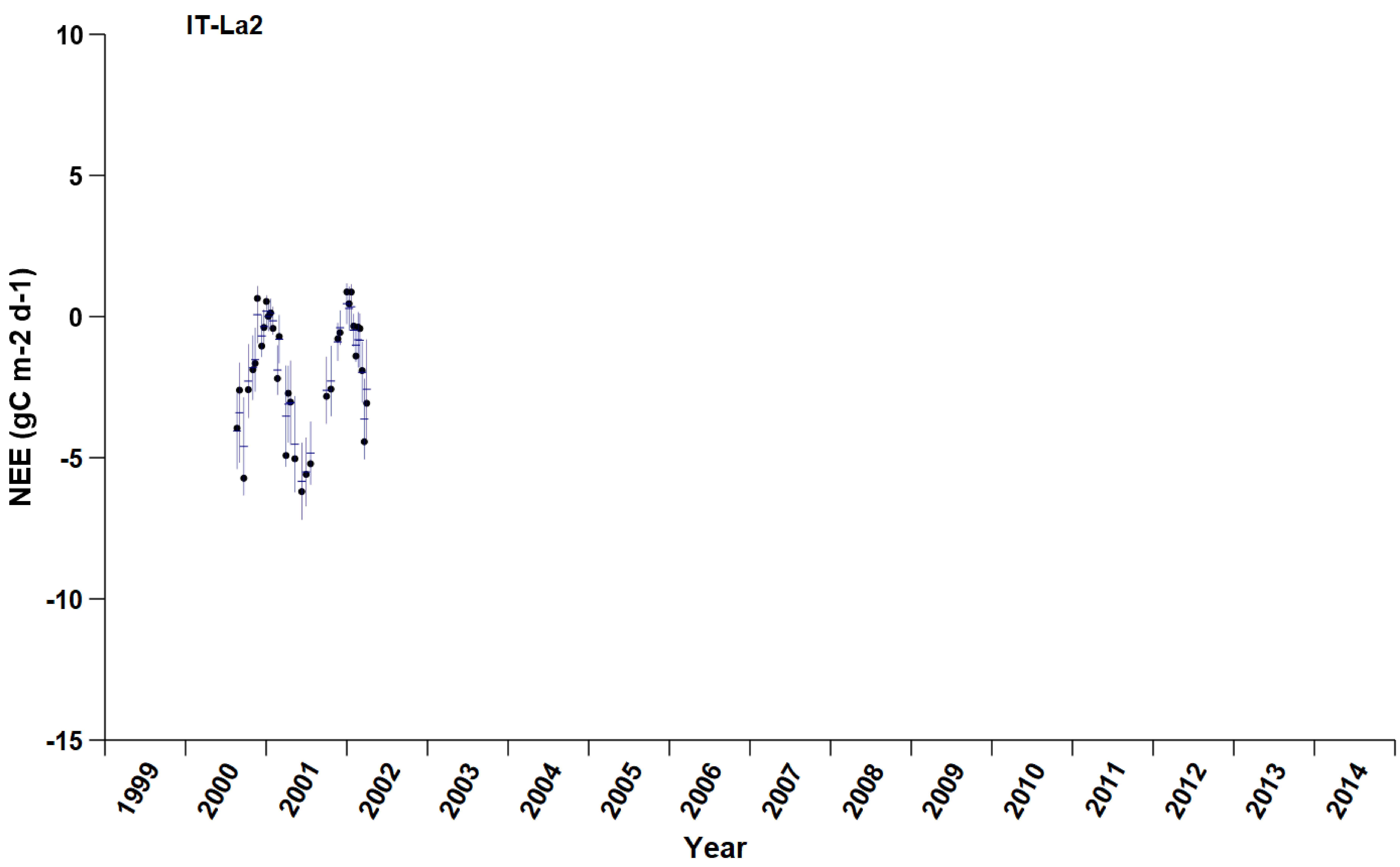
IT-La2
NEE (gC m-2 d-1)
10
5
0
-5
-10
-15
1999
2000
2001
2002
2003
2004
2005
2006
2007
2008
2009
2010
2011
2012
2013
2014
Year

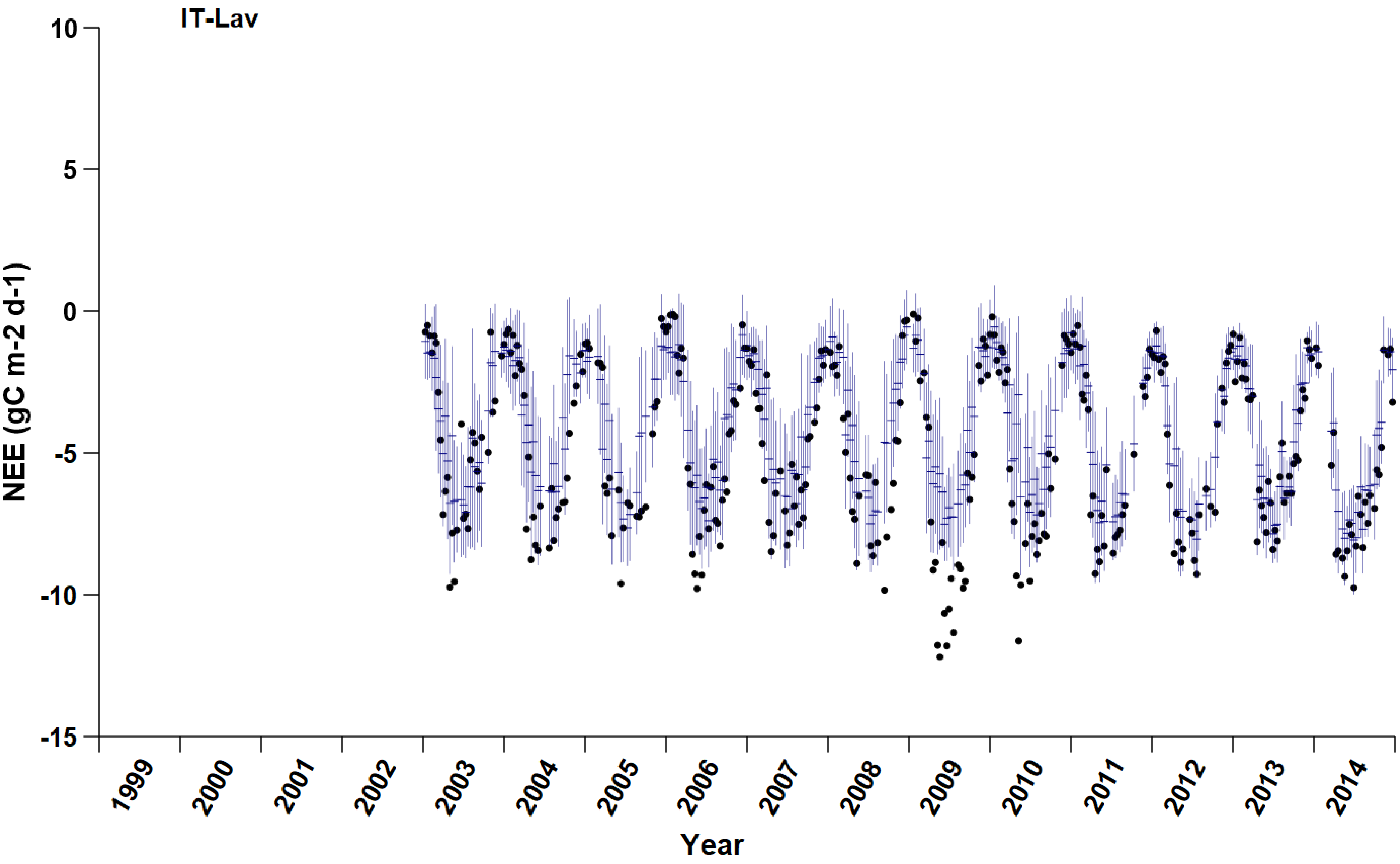

IT-Lav
NEE (gC m-2 d-1)
10
5
0
-5
-10
-15
1999
2000
2001
2002
2003
2004
2005
2006
2007
2008
2009
2010
2011
2012
2013
2014
Year

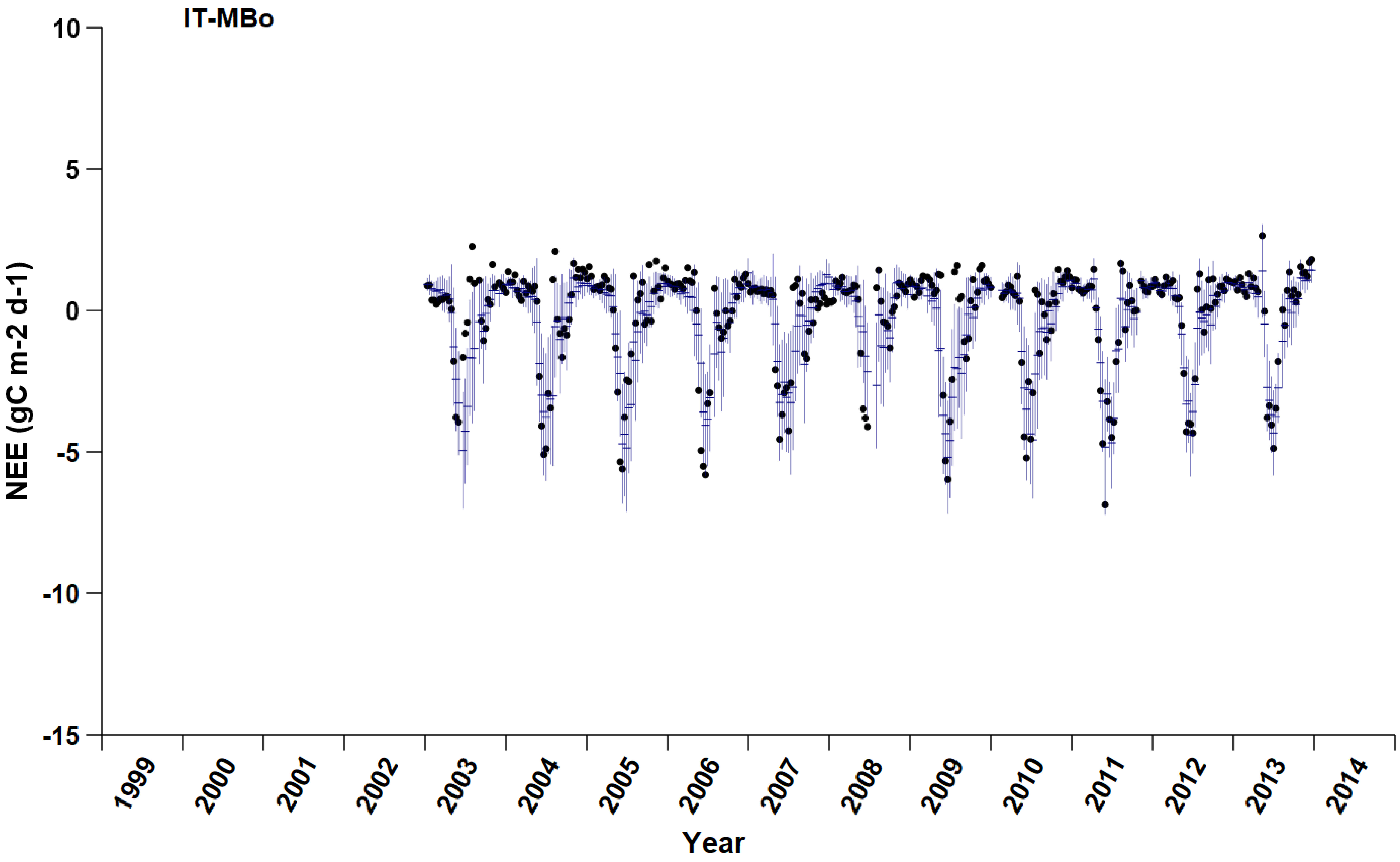

IT-MBo
NEE (gC m-2 d-1)
10
5
0
-5
-10
-15
1999
2000
2001
2002
2003
2004
2005
2006
2007
2008
2009
2010
2011
2012
2013
2014
Year

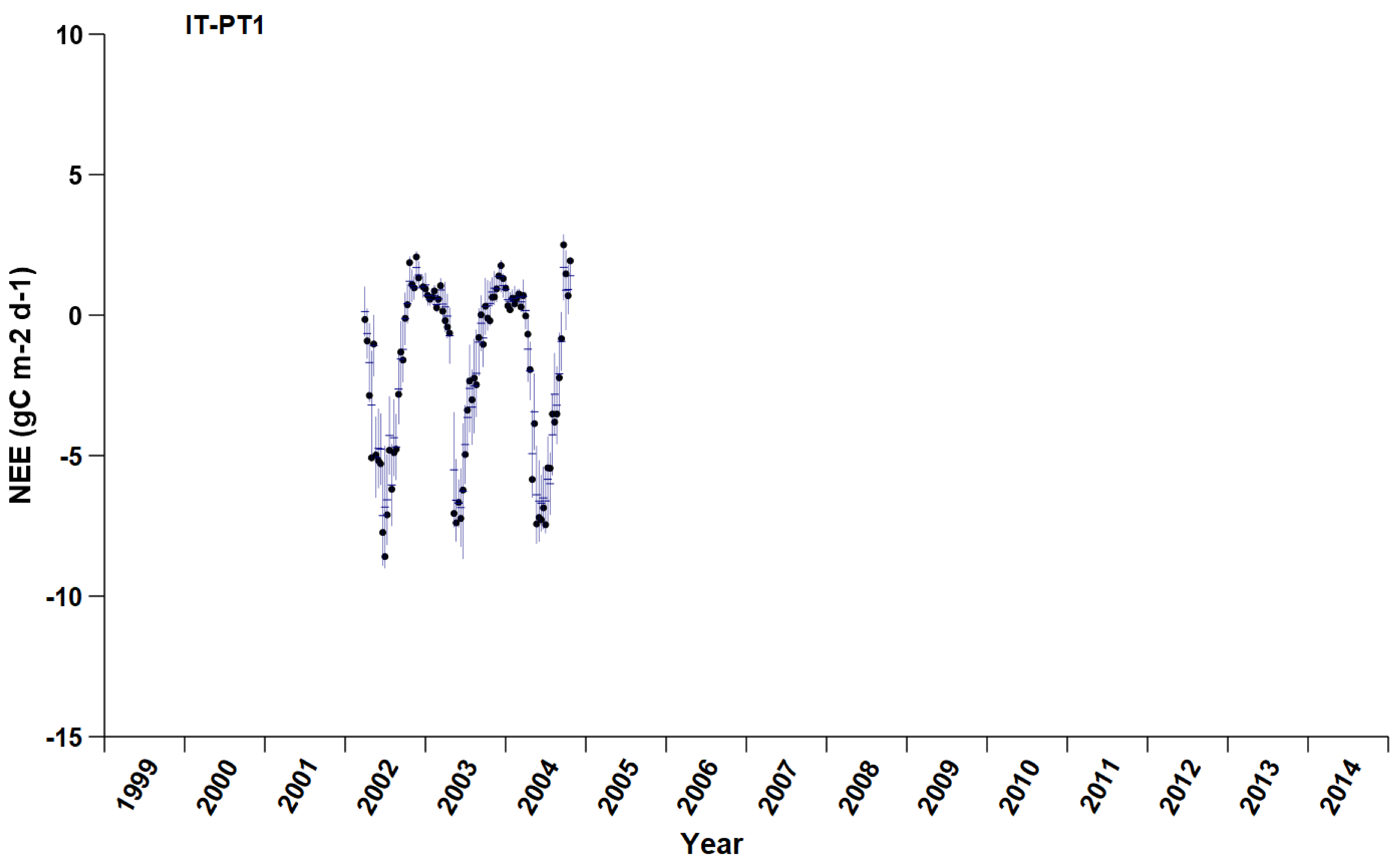

IT-PT1
NEE (gC m-2 d-1)
10
5
0
-5
-10
-15
1999
2000
2001
2002
2003
2004
2005
2006
2007
2008
2009
2010
2011
2012
2013
2014
Year

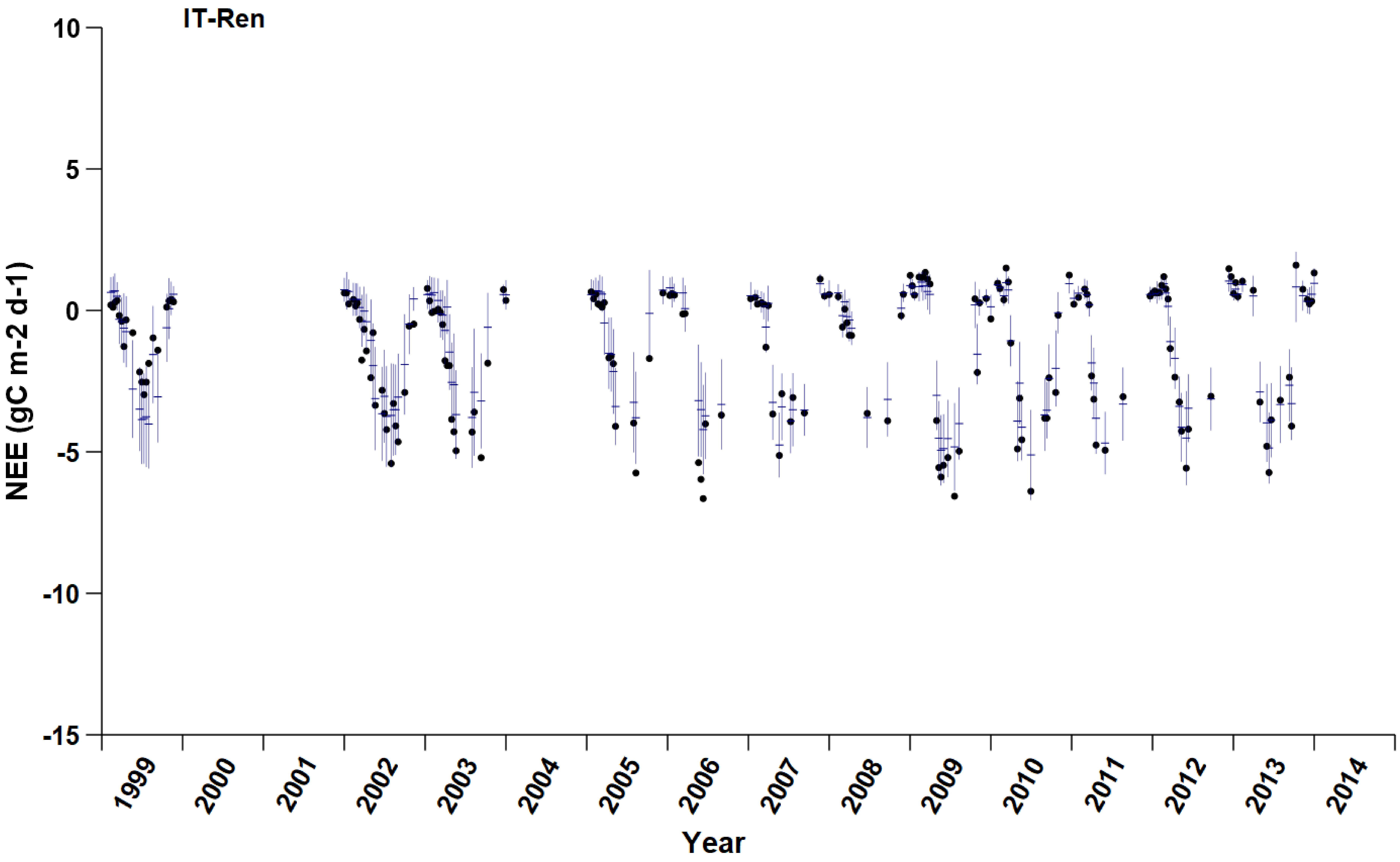
IT-Ren
NEE (gC m-2 d-1)
10
5
0
-5
-10
-15
1999
2000
2001
2002
2003
2004
2005
2006
2007
2008
2009
2010
2011
2012
2013
2014
Year

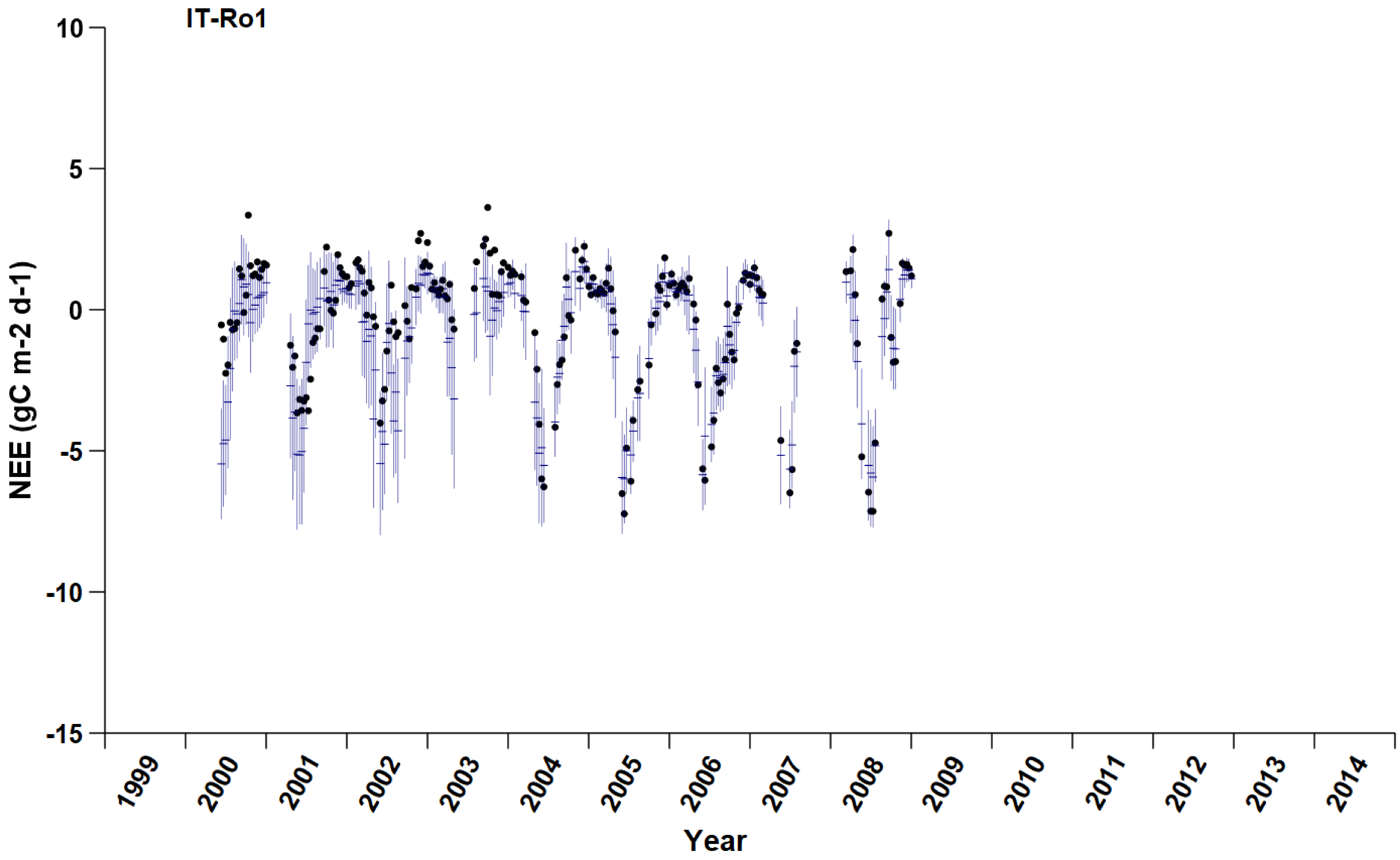

IT-Ro1
NEE (gC m-2 d-1)
10
5
0
-5
-10
-15
1999
2000
2001
2002
2003
2004
2005
2006
2007
2008
2009
2010
2011
2012
2013
2014
Year

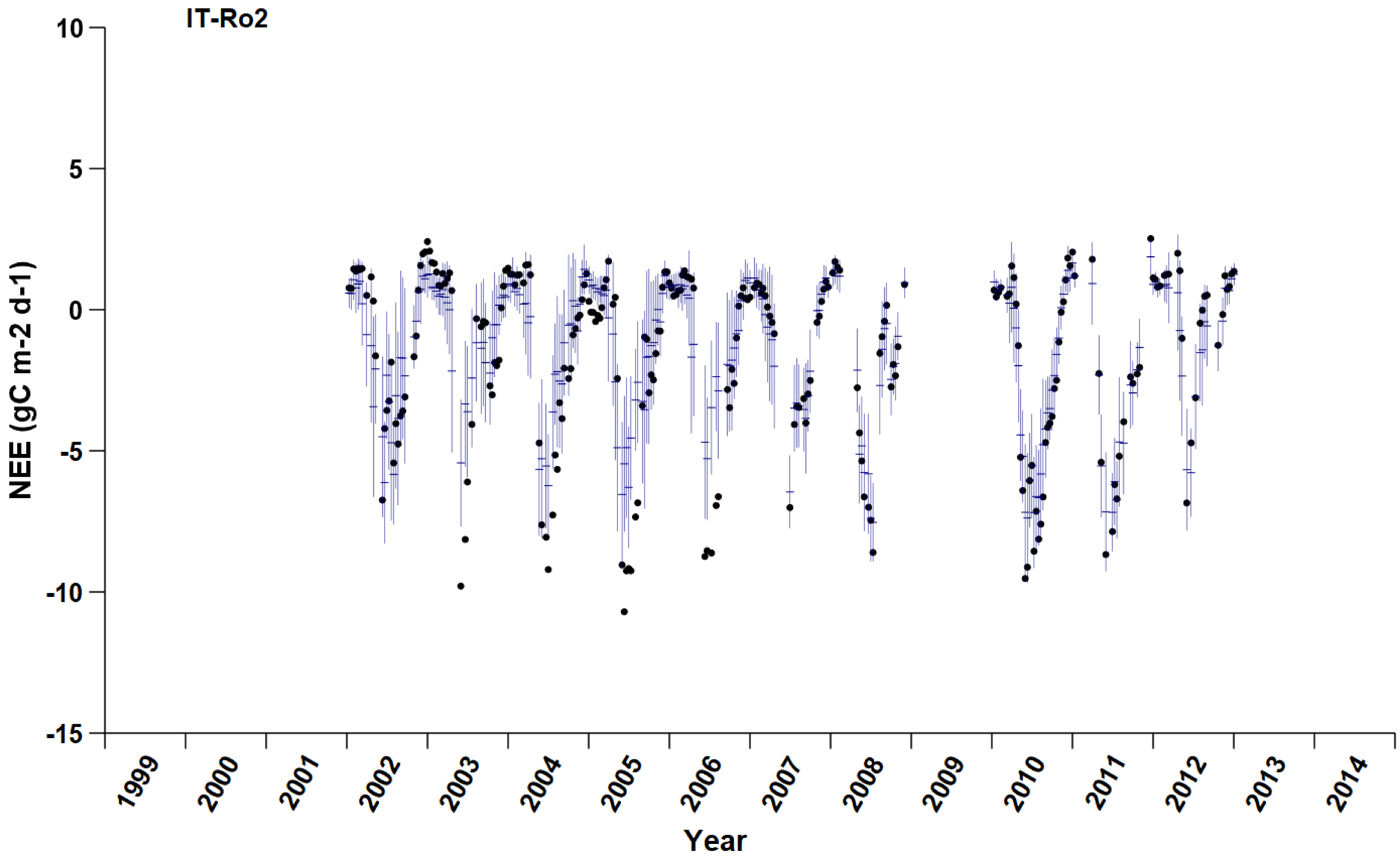
IT-Ro2
NEE (gC m-2 d-1)
10
5
0
-5
-10
-15
1999
2000
2001
2002
2003
2004
2005
2006
2007
2008
2009
2010
2011
2012
2013
2014
Year

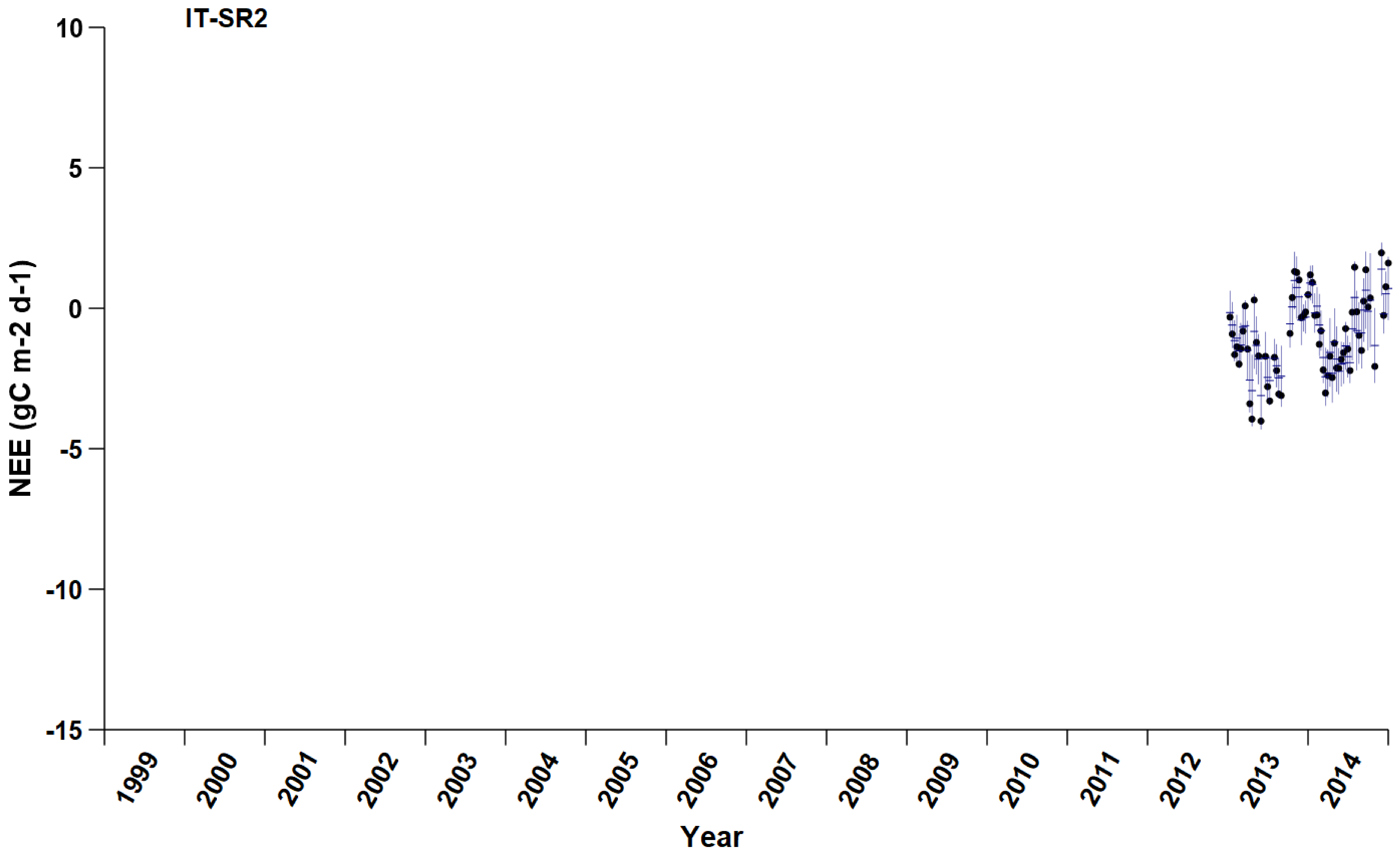
IT-SR2
NEE (gC m-2 d-1)
10
5
0
-5
-10
-15
1999
2000
2001
2002
2003
2004
2005
2006
2007
2008
2009
2010
2011
2012
2013
2014
Year

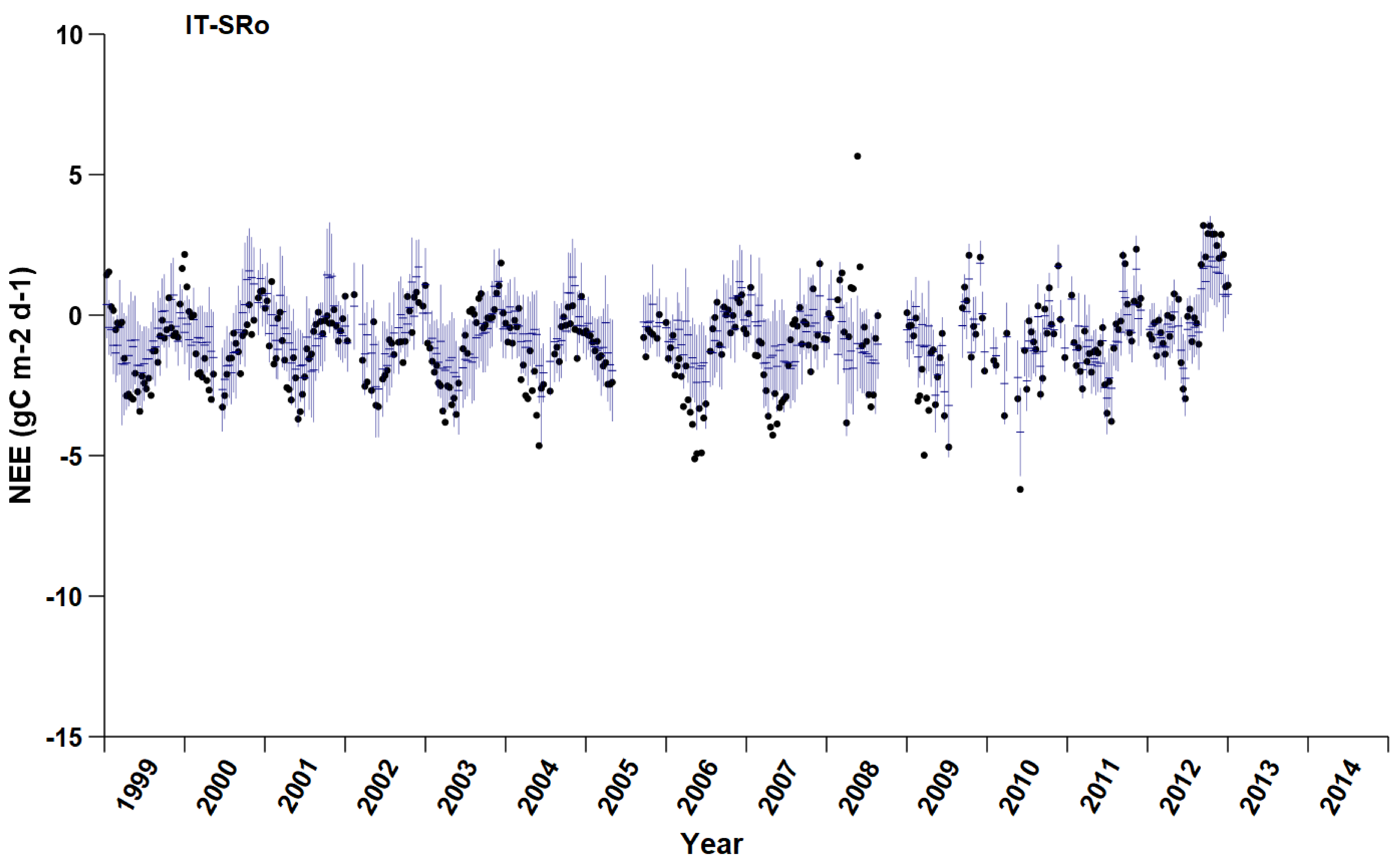

IT-SRo
NEE (gC m-2 d-1)
10
5
0
-5
-10
-15
1999
2000
2001
2002
2003
2004
2005
2006
2007
2008
2009
2010
2011
2012
2013
2014
Year

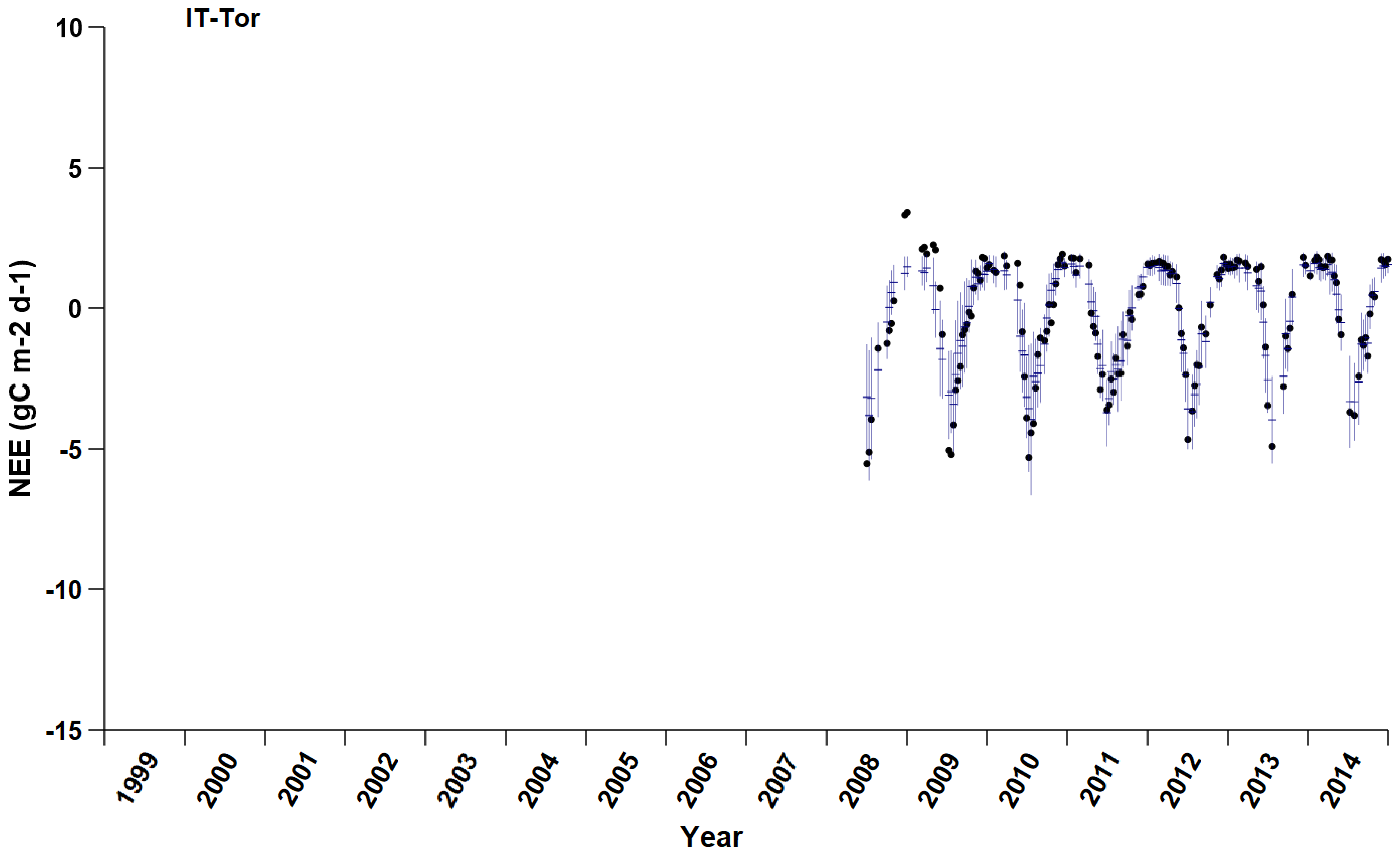

IT-Tor
NEE (gC m-2 d-1)
10
5
0
-5
-10
-15
1999
2000
2001
2002
2003
2004
2005
2006
2007
2008
2009
2010
2011
2012
2013
2014
Year

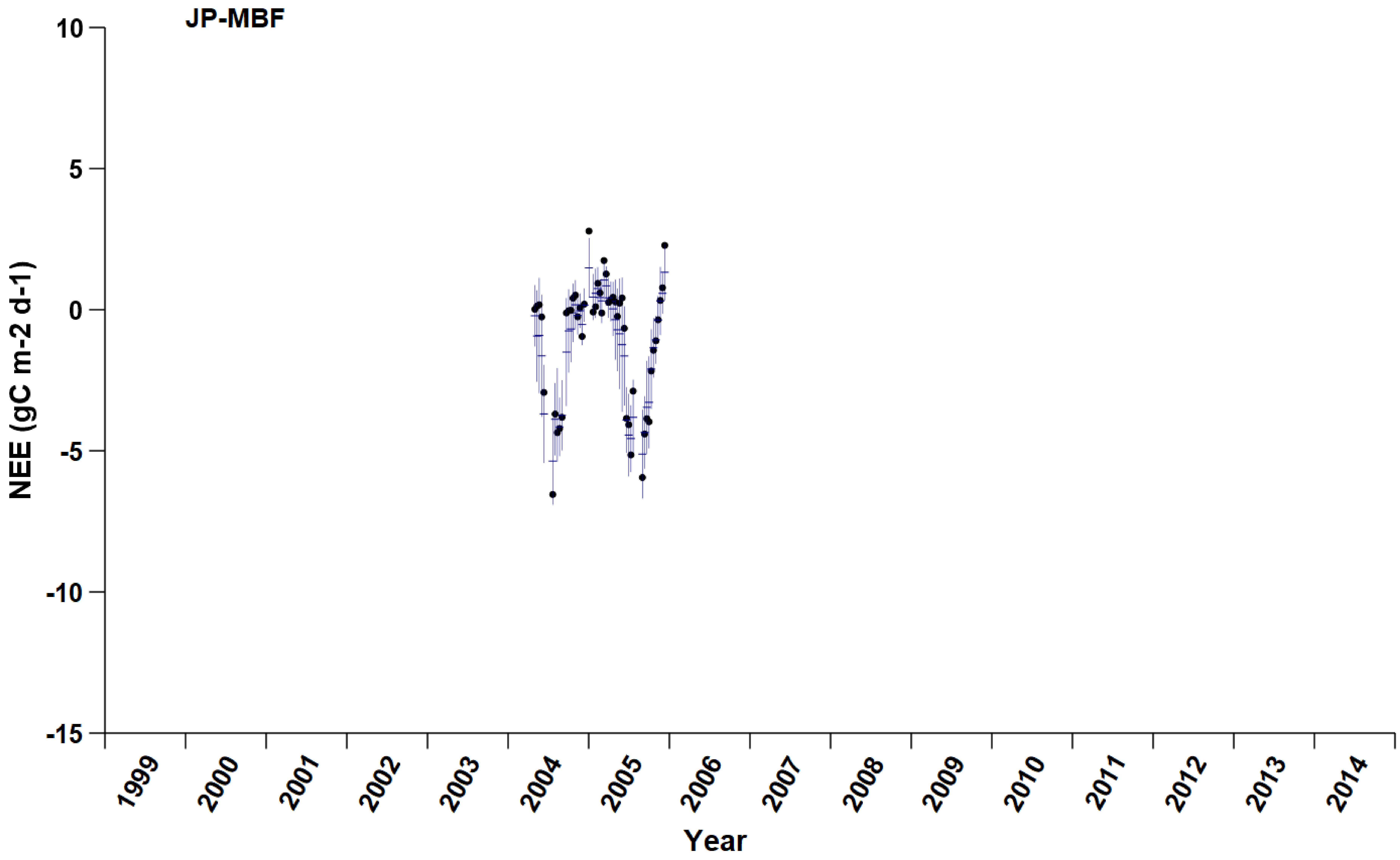

JP-MBF
10
5
0
-5
-10
-15
NEE (gC m-2 d-1)
1999
2000
2001
2002
2003
2004
2005
2006
2007
2008
2009
2010
2011
2012
2013
2014
Year

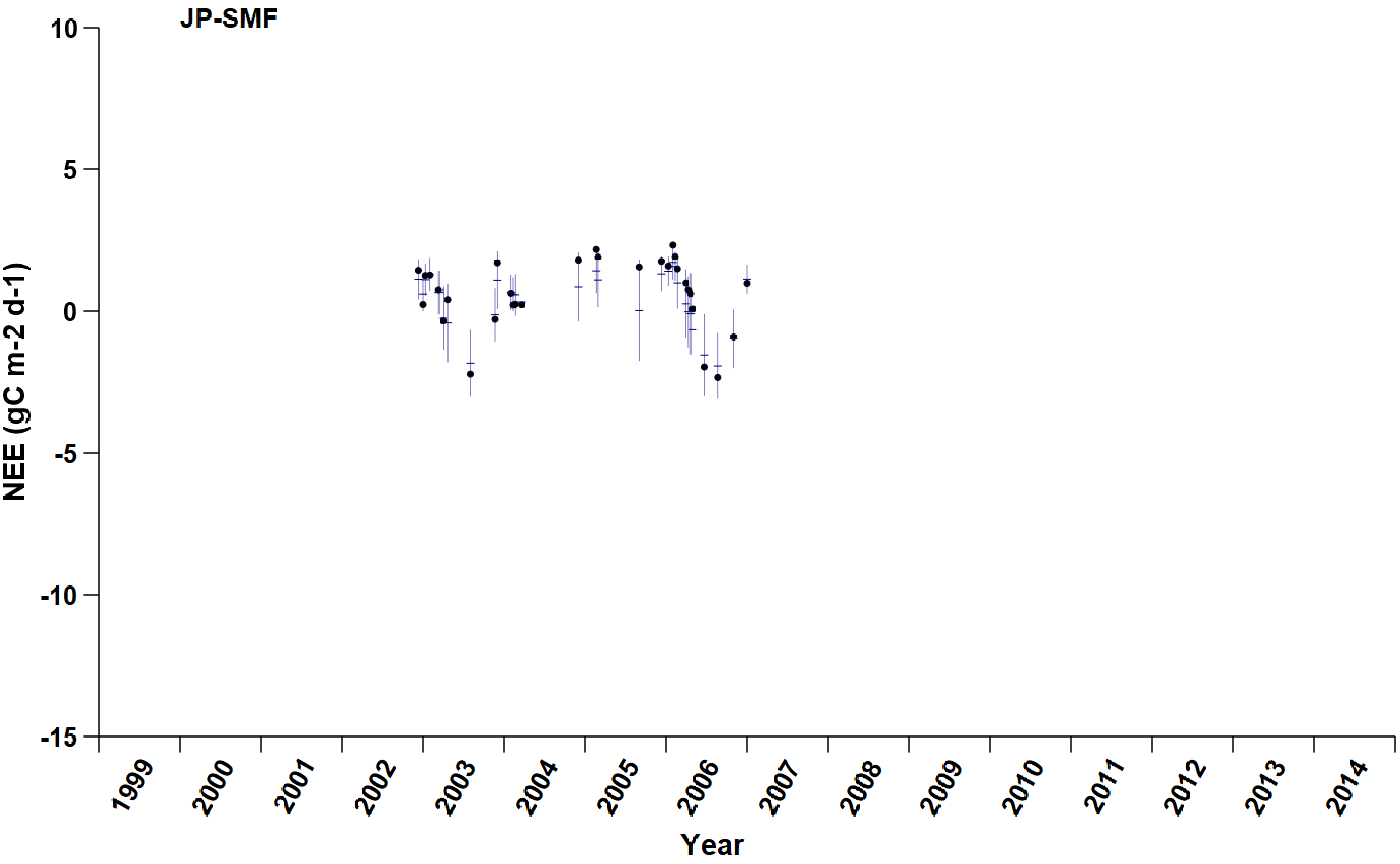

JP-SMF
10
5
0
-5
-10
-15
NEE (gC m-2 d-1)
1999
2000
2001
2002
2003
2004
2005
2006
2007
2008
2009
2010
2011
2012
2013
2014
Year

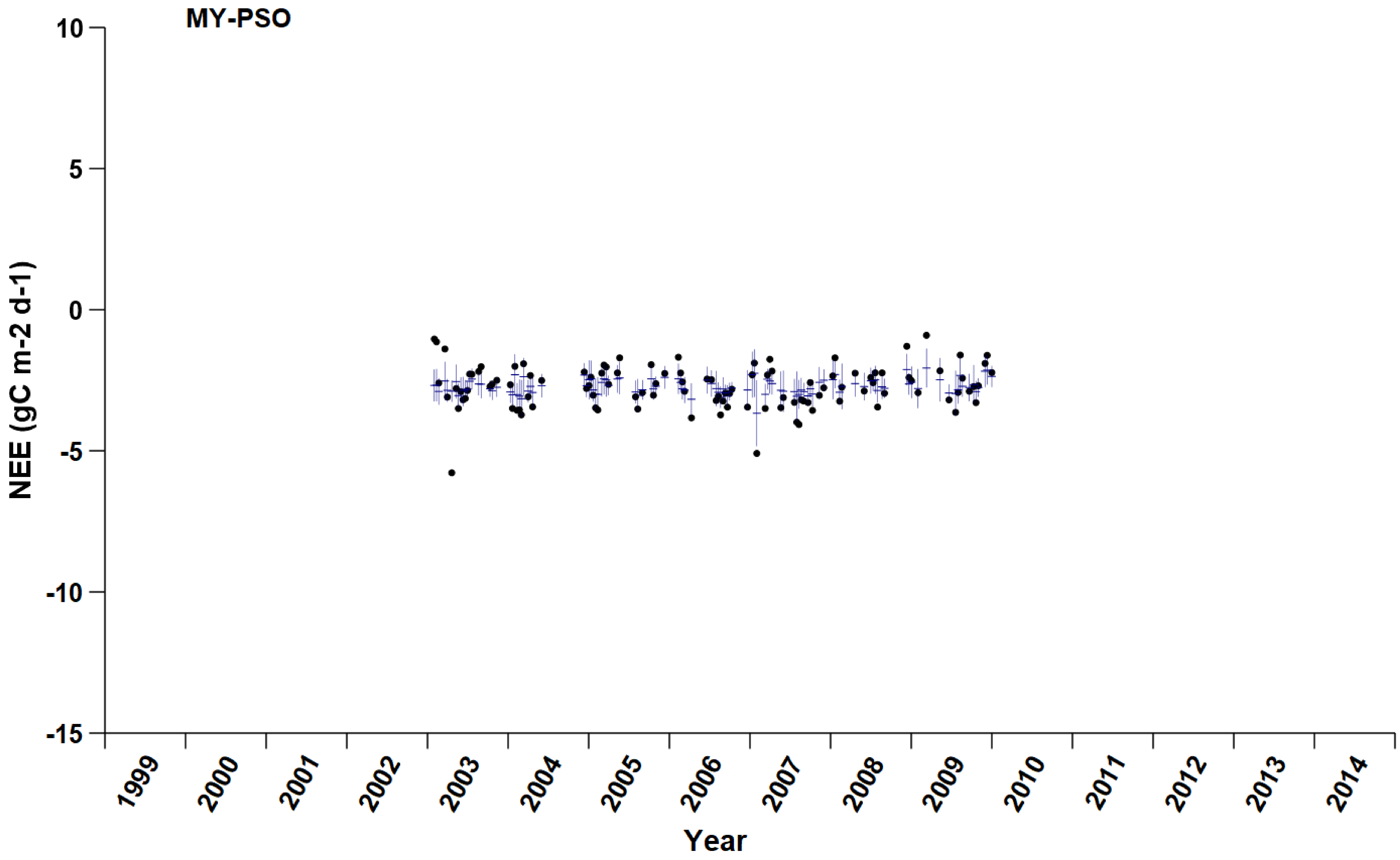
MY-PSO
10
5
0
-5
-10
-15
NEE (gC m-2 d-1)
1999
2000
2001
2002
2003
2004
2005
2006
2007
2008
2009
2010
2011
2012
2013
2014
Year

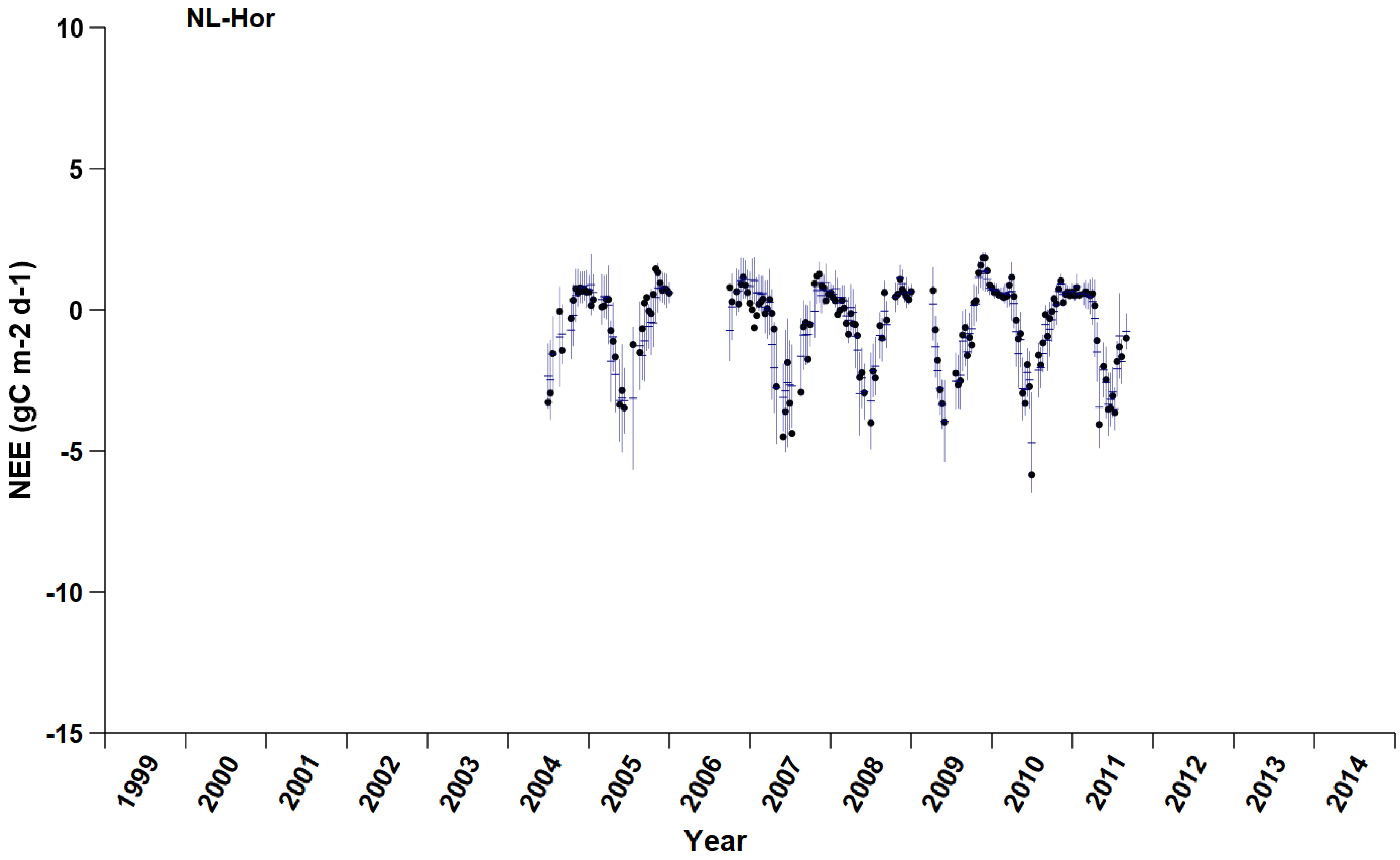

NL-Hor
10
5
0
-5
-10
-15
NEE (gC m-2 d-1)
1999
2000
2001
2002
2003
2004
2005
2006
2007
2008
2009
2010
2011
2012
2013
2014
Year

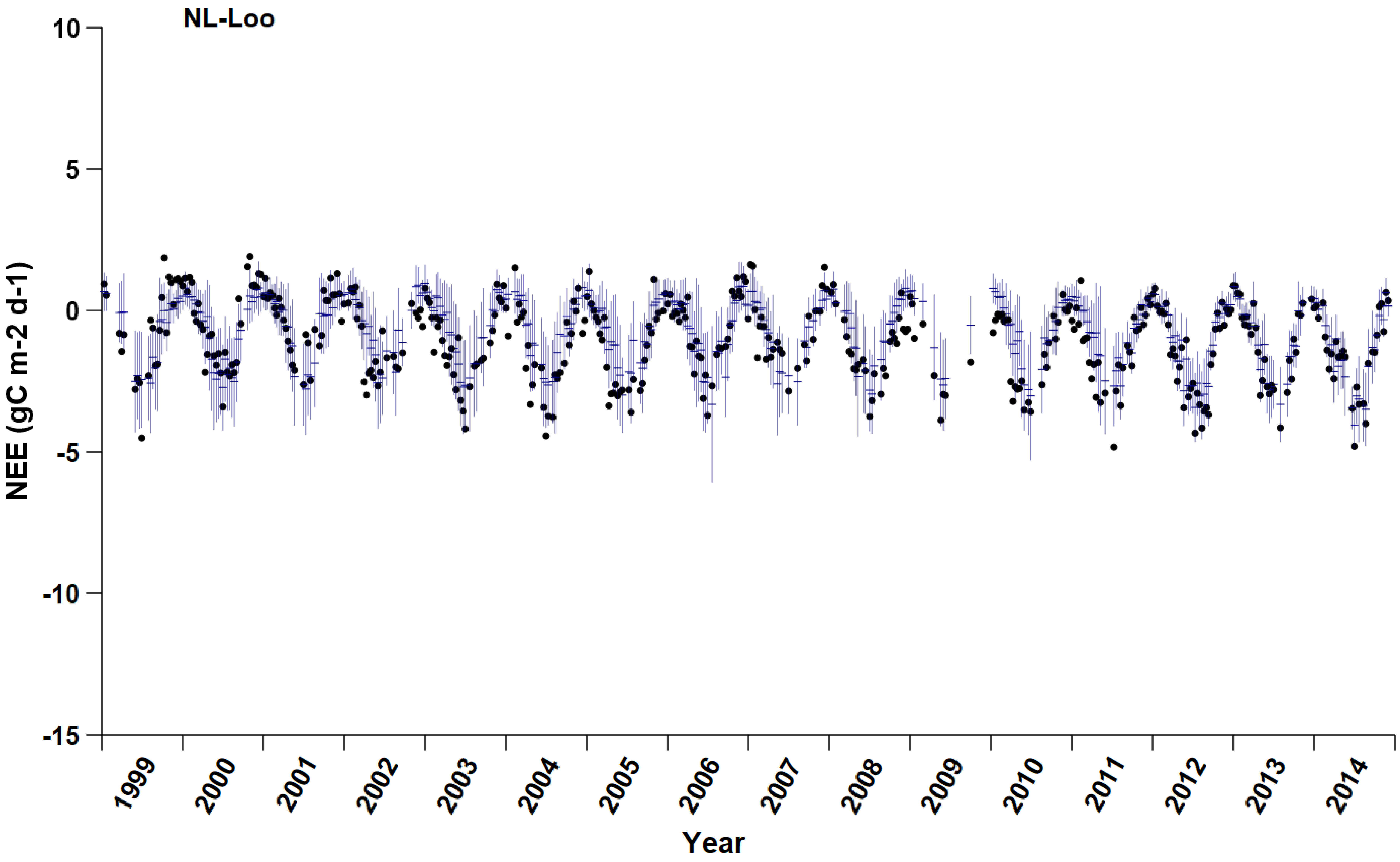

NL-Loo
10
5
0
-5
-10
-15
NEE (gC m-2 d-1)
1999
2000
2001
2002
2003
2004
2005
2006
2007
2008
2009
2010
2011
2012
2013
2014
Year

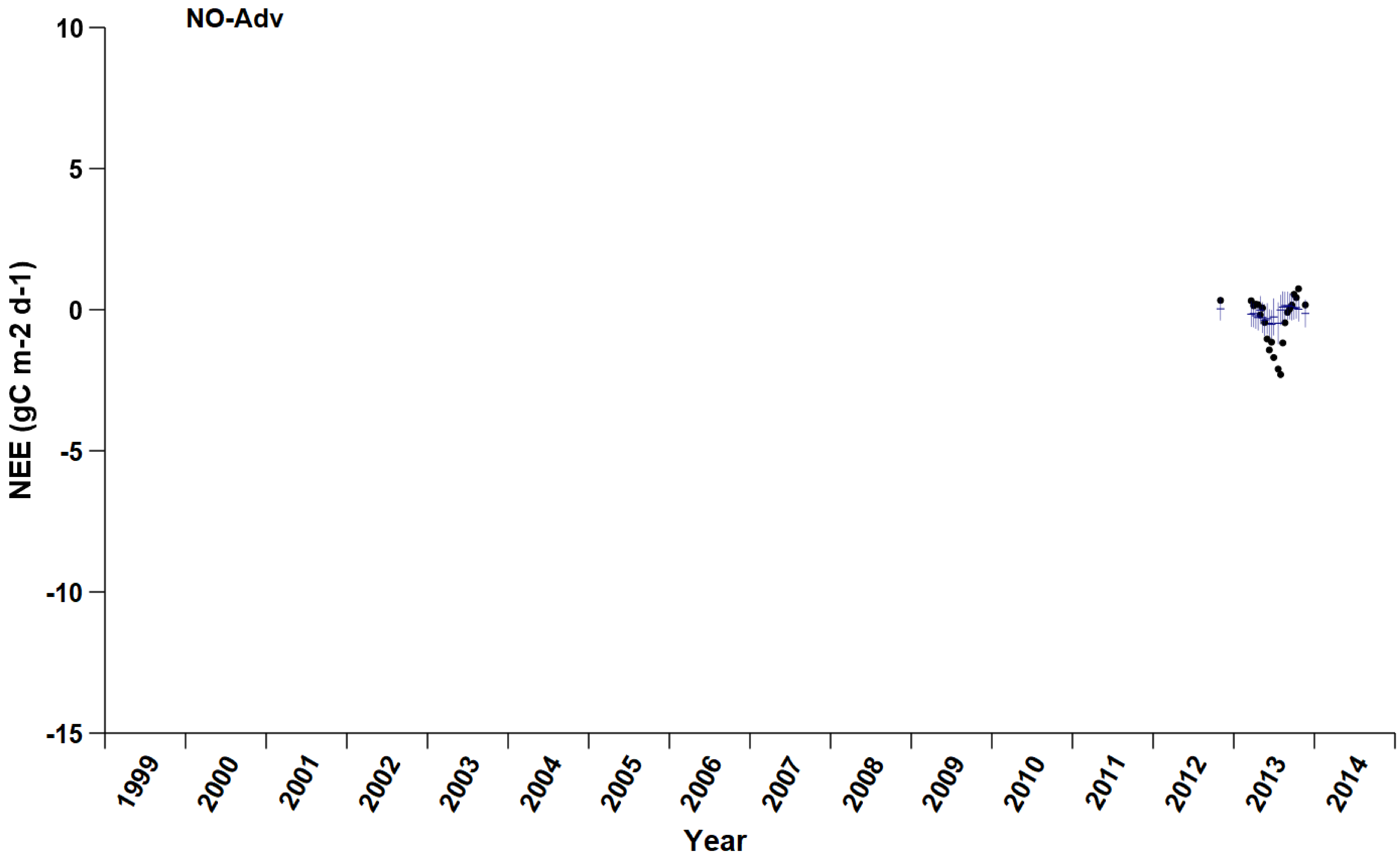

NO-Adv
NEE (gC m-2 d-1)
10
5
0
-5
-10
-15
1999
2000
2001
2002
2003
2004
2005
2006
2007
2008
2009
2010
2011
2012
2013
2014
Year

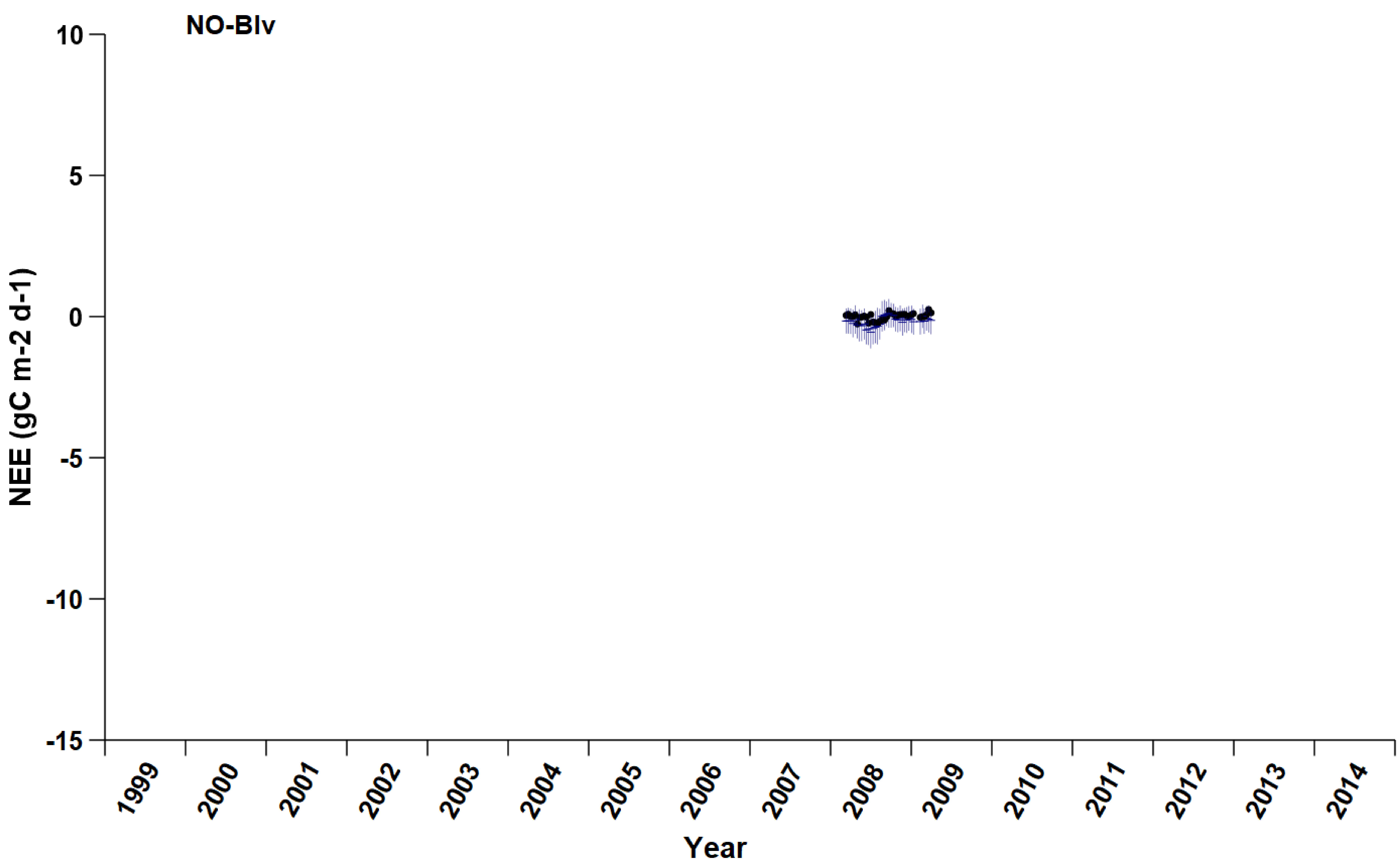

NO-Blv
10
5
0
-5
-10
-15
NEE (gC m-2 d-1)
1999
2000
2001
2002
2003
2004
2005
2006
2007
2008
2009
2010
2011
2012
2013
2014
Year

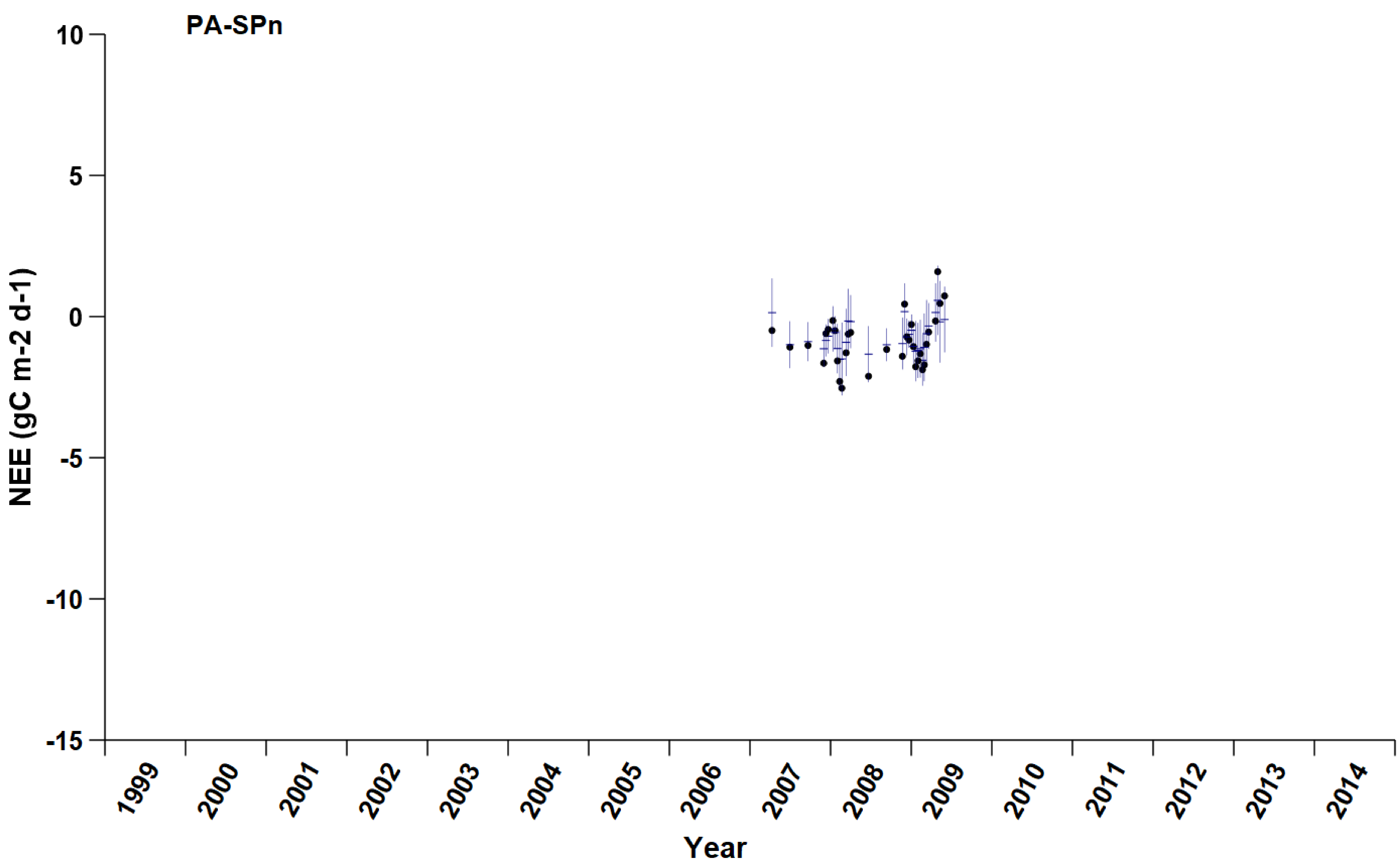
PA-SPn
NEE (gC m-2 d-1)
10
5
0
-5
-10
-15
1999
2000
2001
2002
2003
2004
2005
2006
2007
2008
2009
2010
2011
2012
2013
2014
Year

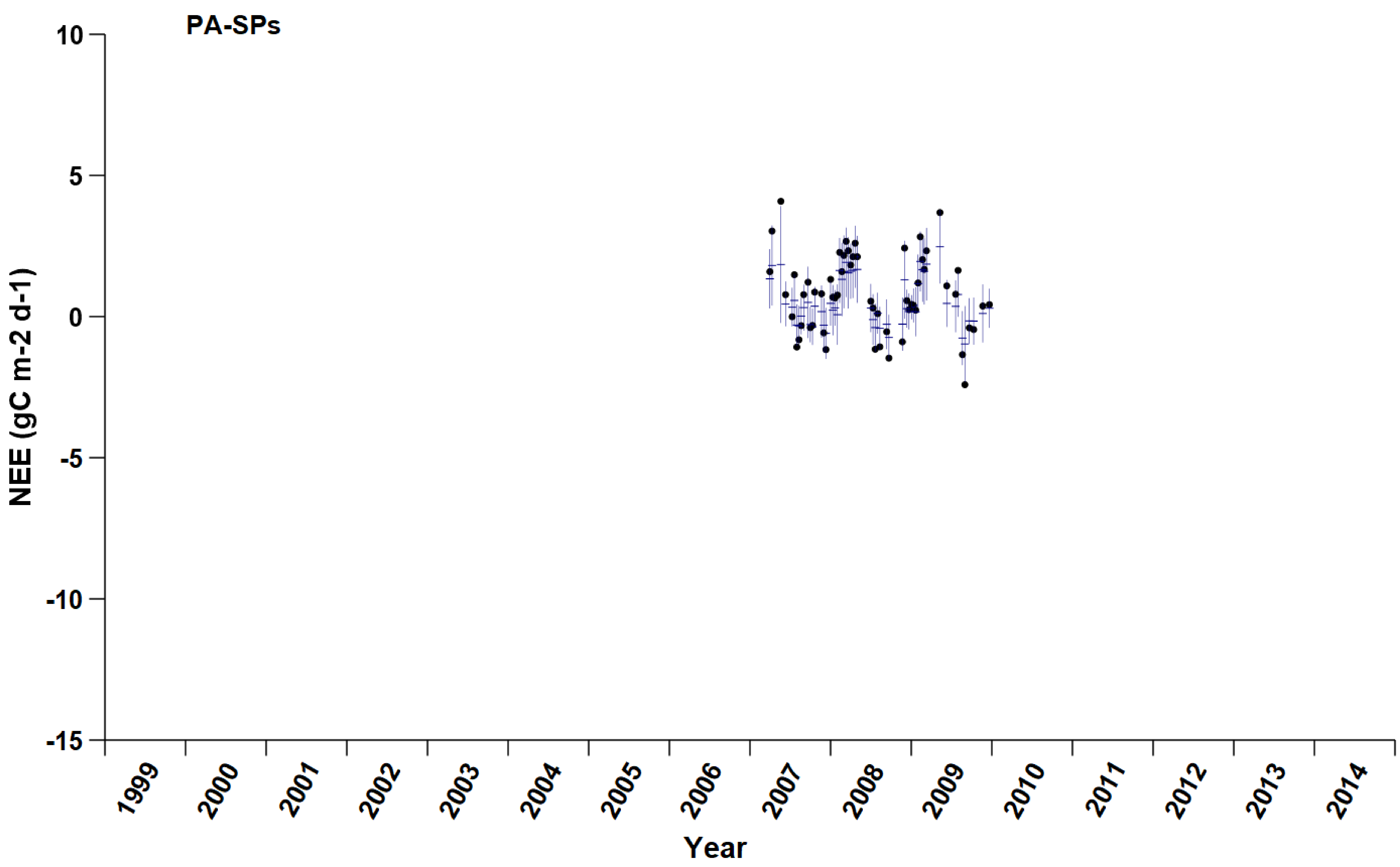

PA-SPs
10
5
0
-5
-10
-15
NEE (gC m-2 d-1)
1999
2000
2001
2002
2003
2004
2005
2006
2007
2008
2009
2010
2011
2012
2013
2014
Year

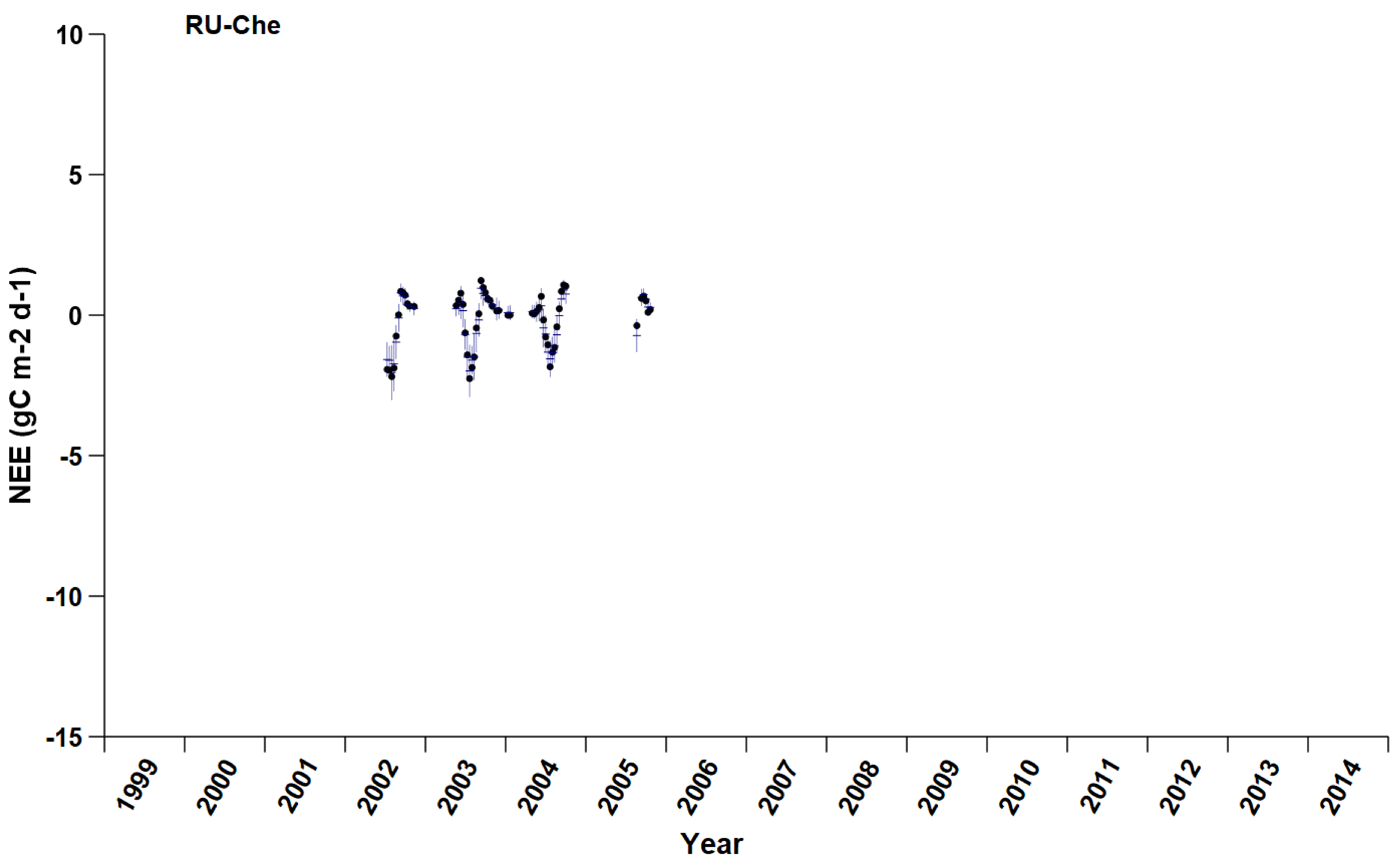

RU-Che
NEE (gC m-2 d-1)
10
5
0
-5
-10
-15
1999
2000
2001
2002
2003
2004
2005
2006
2007
2008
2009
2010
2011
2012
2013
2014
Year

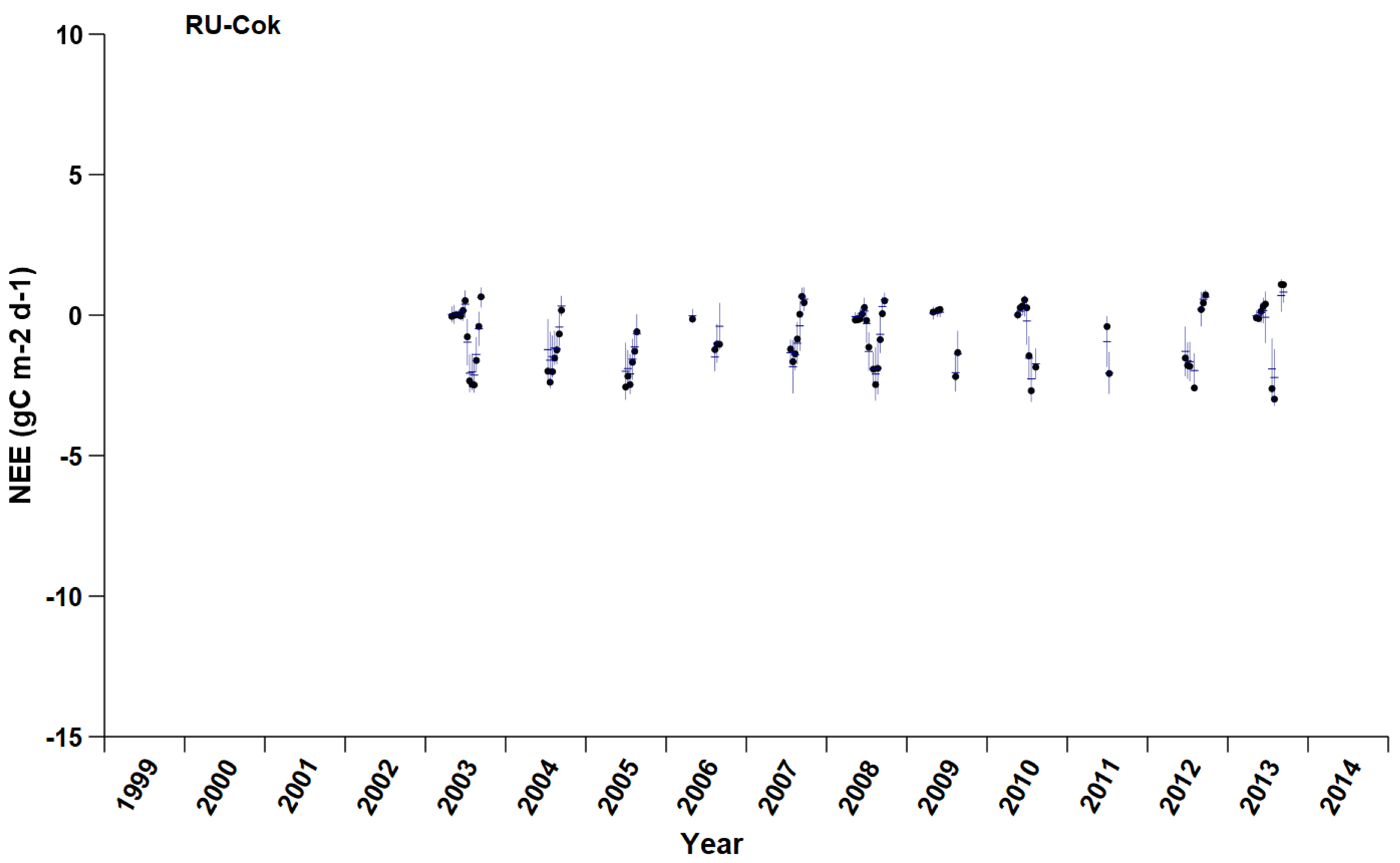
RU-Cok
NEE (gC m-2 d-1)
10
5
0
-5
-10
-15
1999
2000
2001
2002
2003
2004
2005
2006
2007
2008
2009
2010
2011
2012
2013
2014
Year

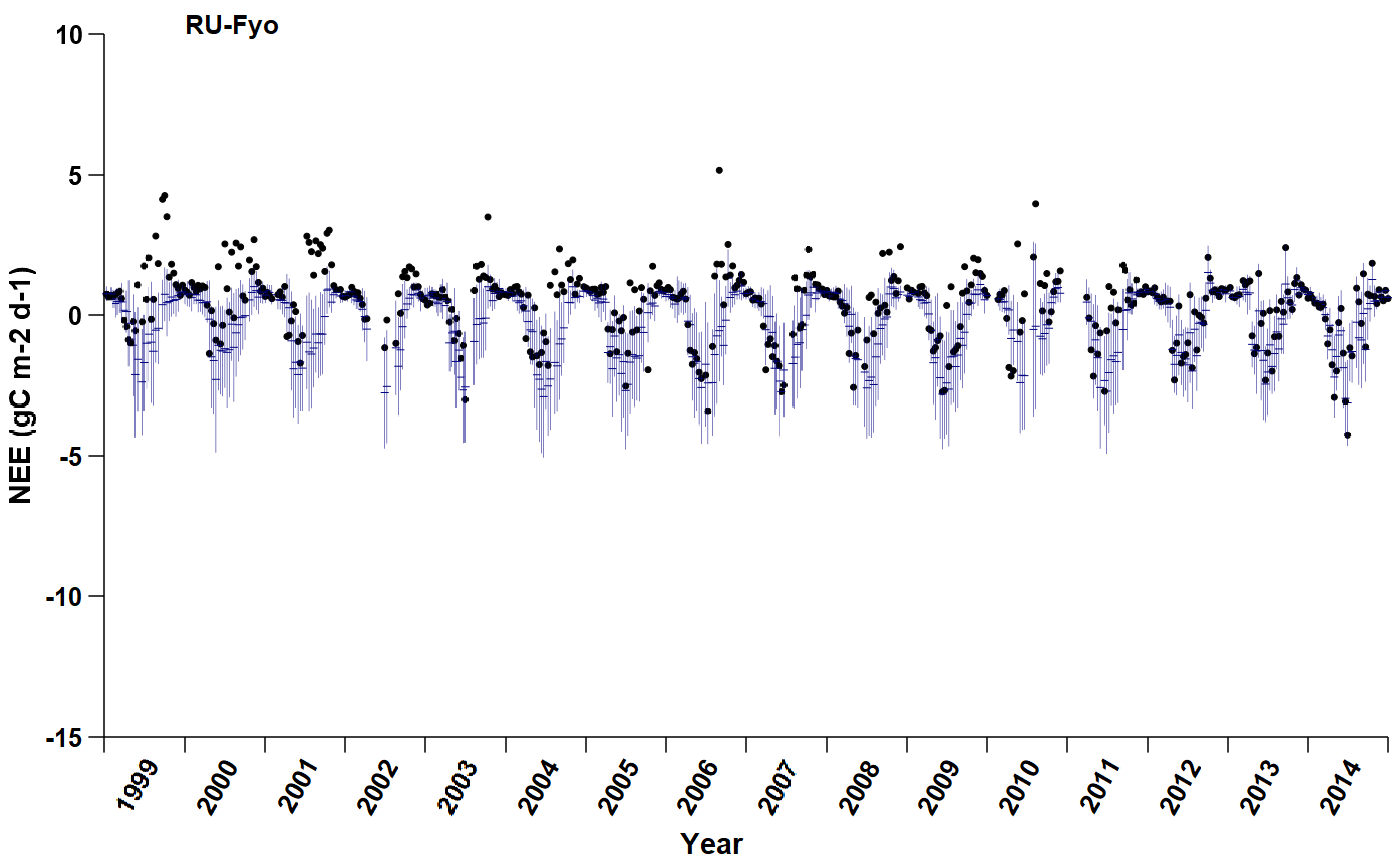
RU-Fyo
NEE (gC m-2 d-1)
10
5
0
-5
-10
-15
1999
2000
2001
2002
2003
2004
2005
2006
2007
2008
2009
2010
2011
2012
2013
2014
Year

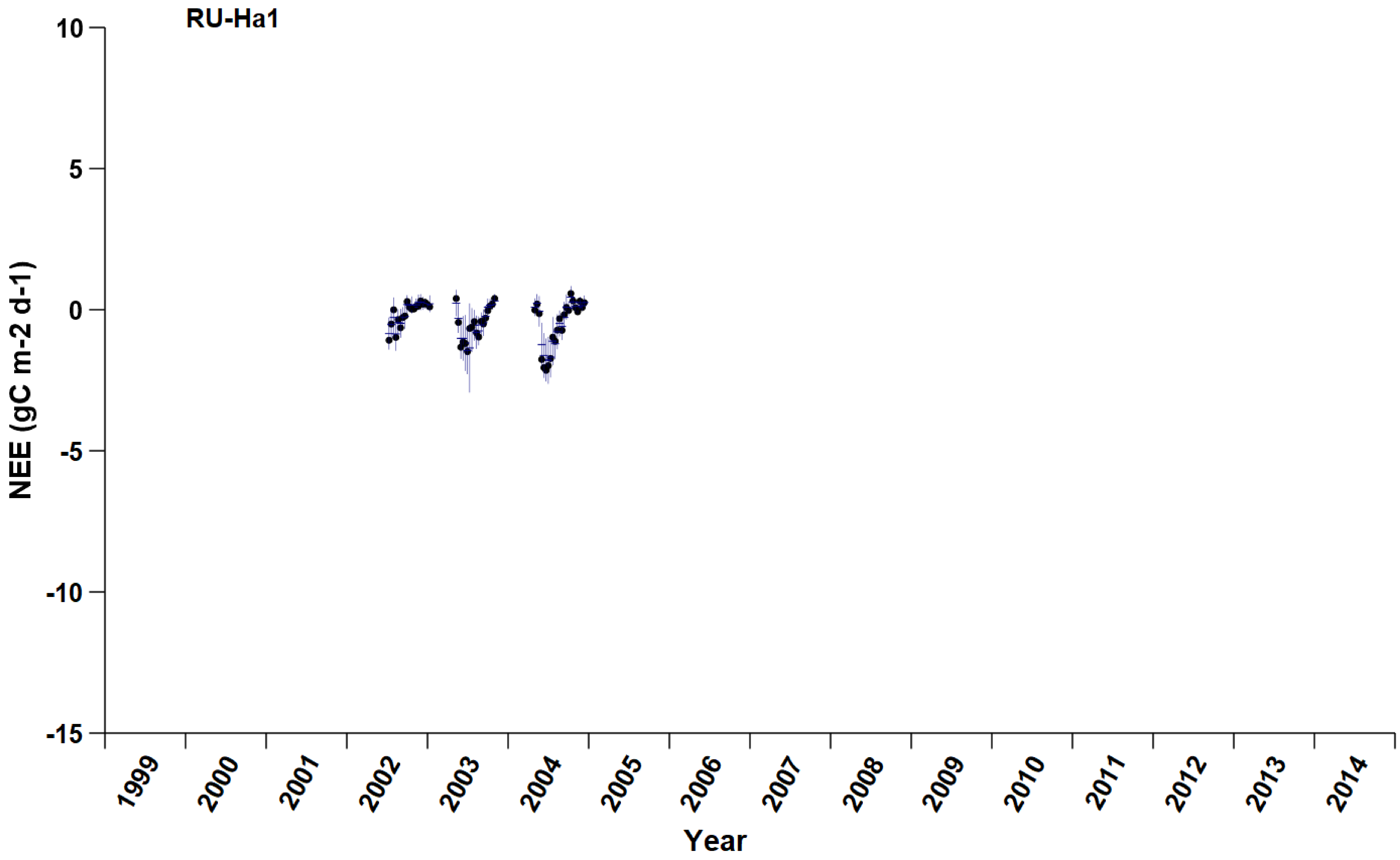
RU-Ha1
NEE (gC m-2 d-1)
10
5
0
-5
-10
-15
1999
2000
2001
2002
2003
2004
2005
2006
2007
2008
2009
2010
2011
2012
2013
2014
Year

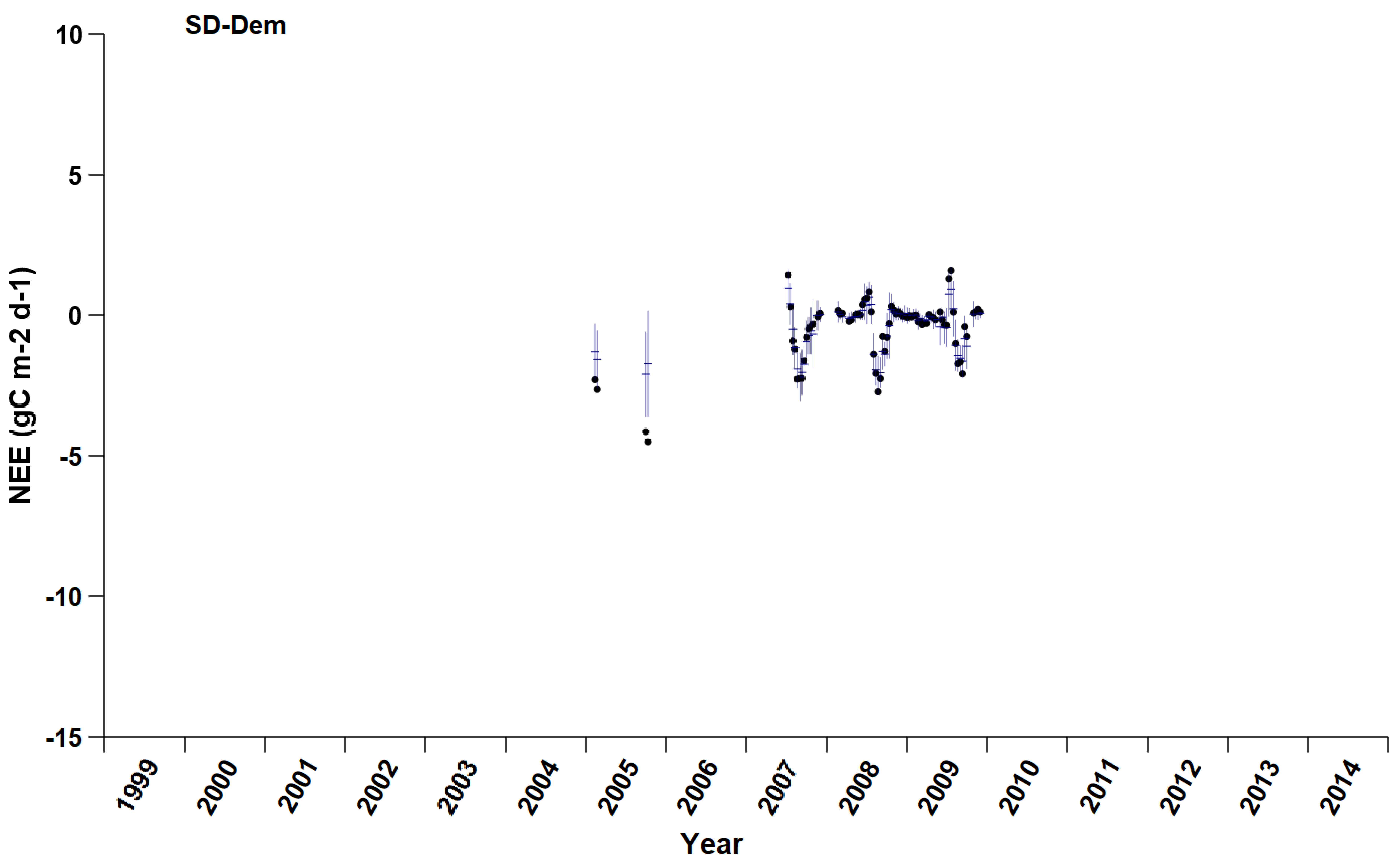
SD-Dem
NEE (gC m-2 d-1)
10
5
0
-5
-10
-15
1999
2000
2001
2002
2003
2004
2005
2006
2007
2008
2009
2010
2011
2012
2013
2014
Year

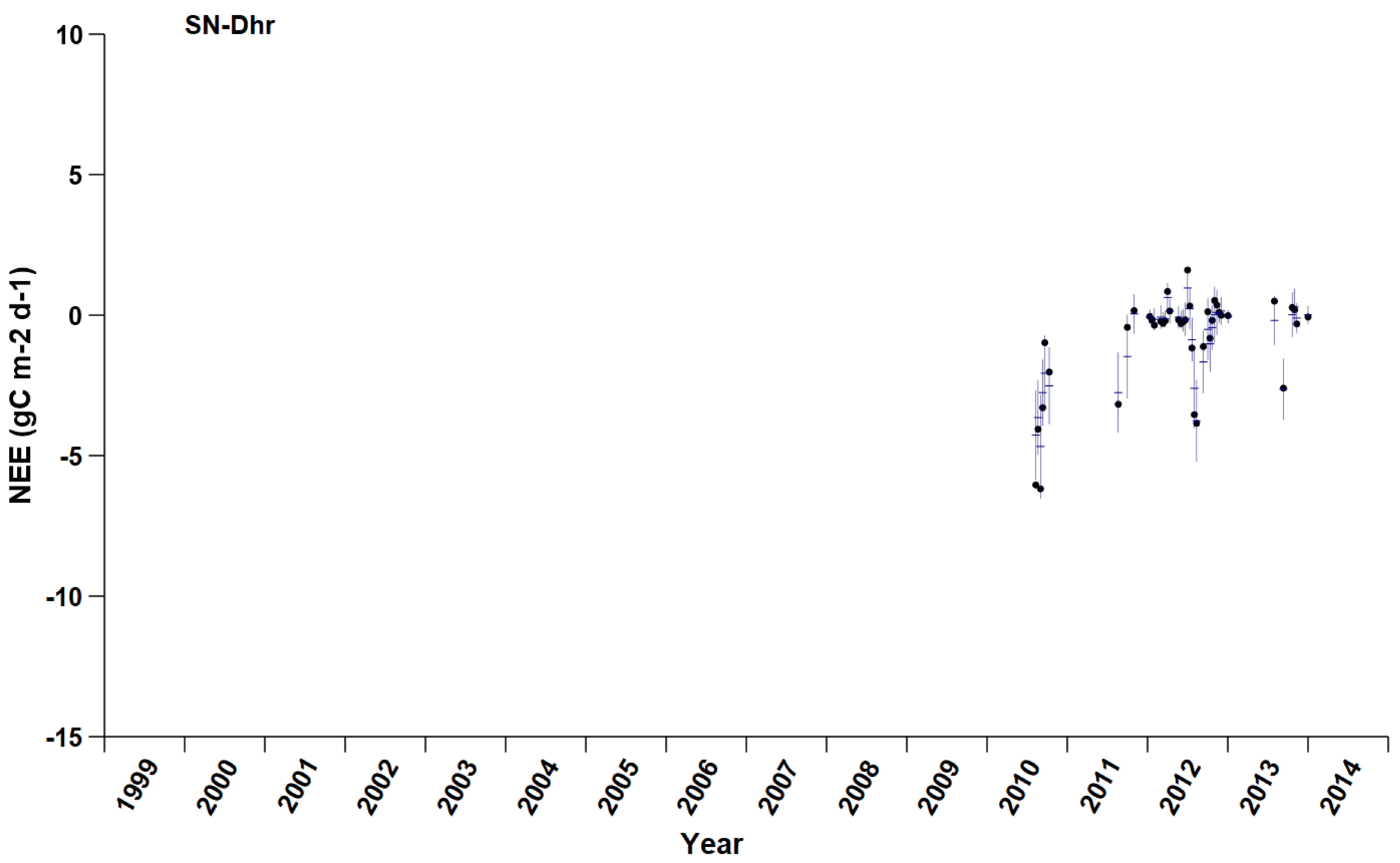

SN-Dhr
NEE (gC m-2 d-1)
10
5
0
-5
-10
-15
1999
2000
2001
2002
2003
2004
2005
2006
2007
2008
2009
2010
2011
2012
2013
2014
Year

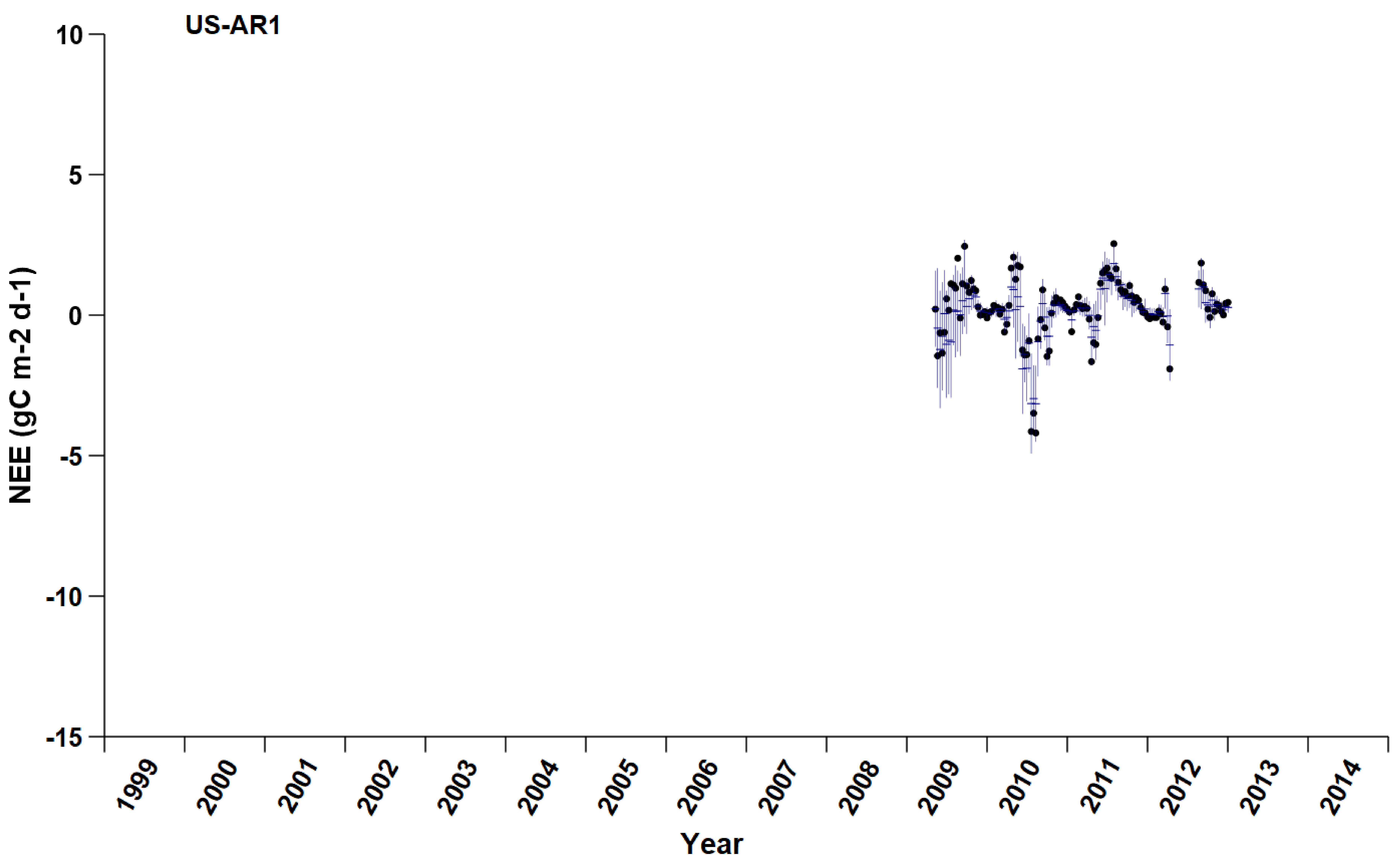
US-AR1
10
5
0
-5
-10
-15
NEE (gC m-2 d-1)
1999
2000
2001
2002
2003
2004
2005
2006
2007
2008
2009
2010
2011
2012
2013
2014
Year

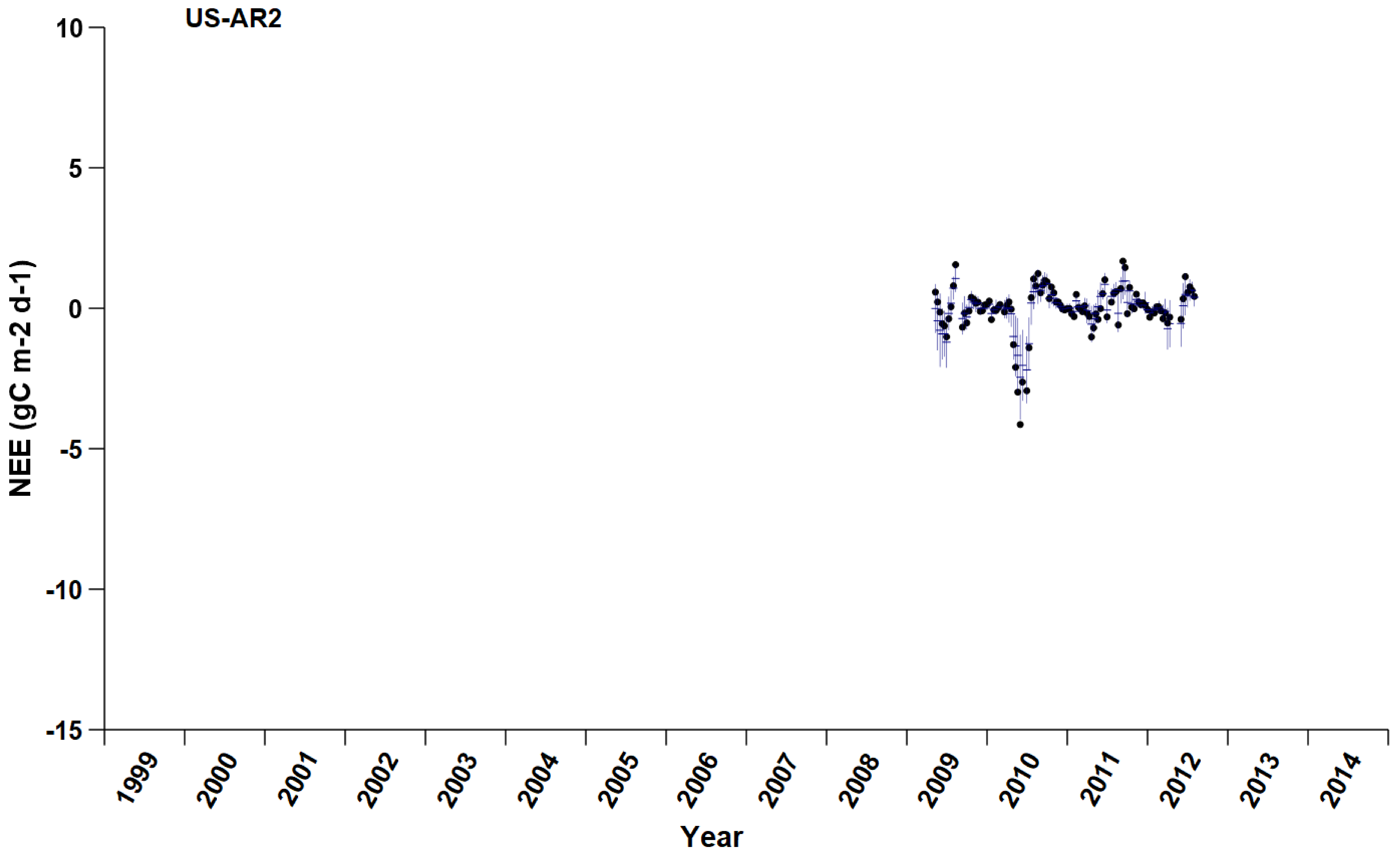
US-AR2
NEE (gC m-2 d-1)
10
5
0
-5
-10
-15
1999
2000
2001
2002
2003
2004
2005
2006
2007
2008
2009
2010
2011
2012
2013
2014
Year

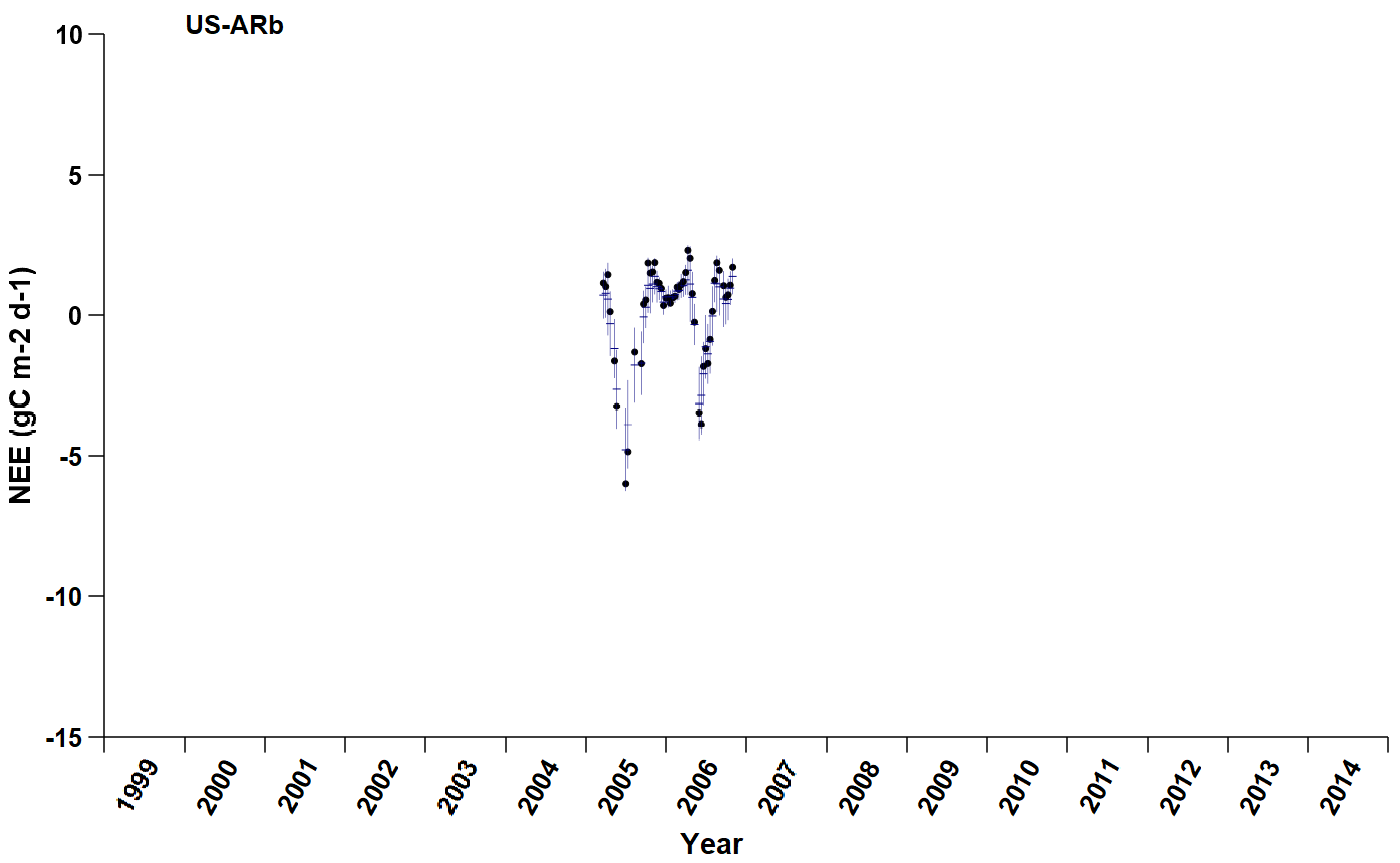
US-ARb
NEE (gC m-2 d-1)
10
5
0
-5
-10
-15
1999
2000
2001
2002
2003
2004
2005
2006
2007
2008
2009
2010
2011
2012
2013
2014
Year

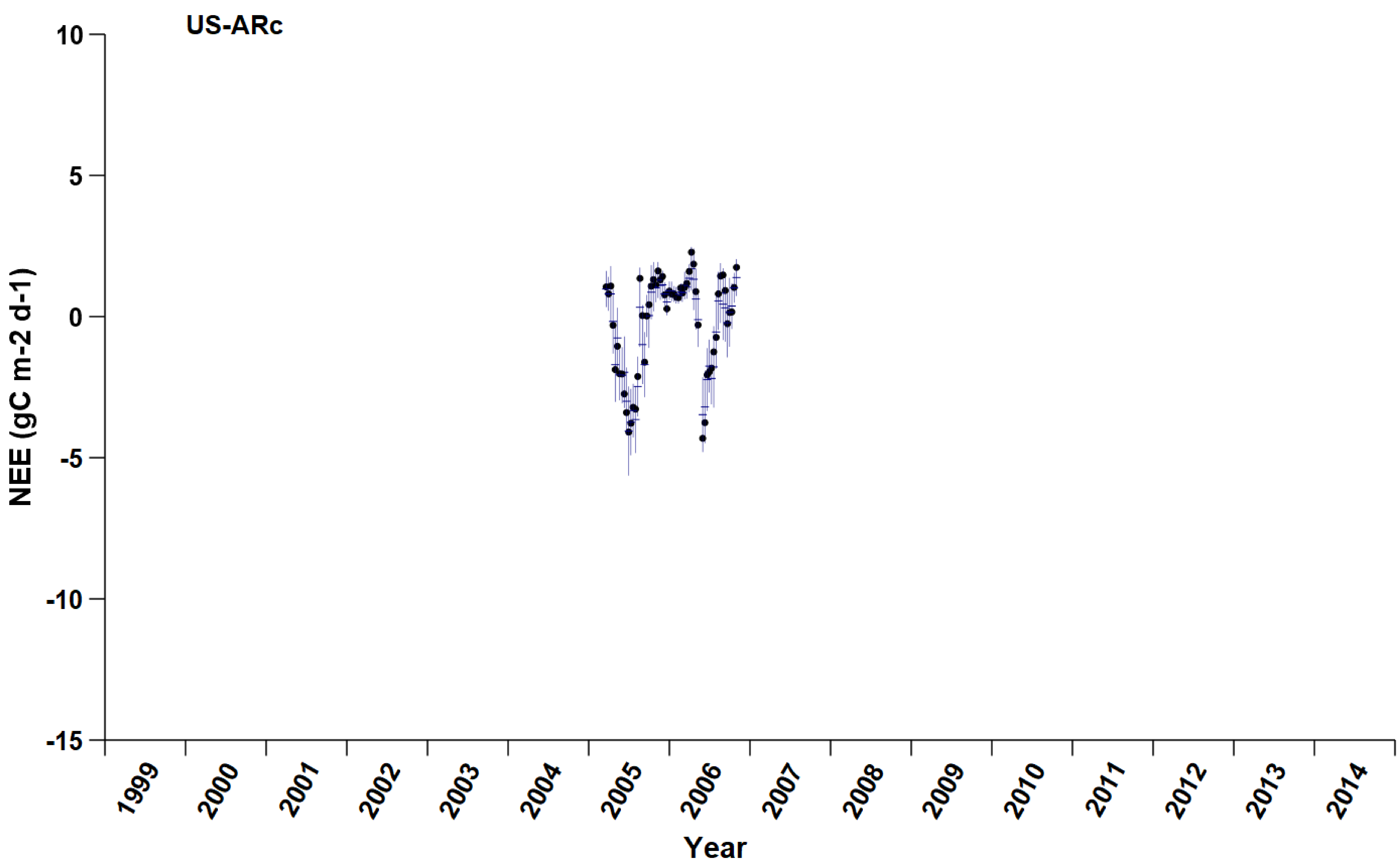

US-ARc
NEE (gC m-2 d-1)
10
5
0
-5
-10
-15
1999
2000
2001
2002
2003
2004
2005
2006
2007
2008
2009
2010
2011
2012
2013
2014
Year

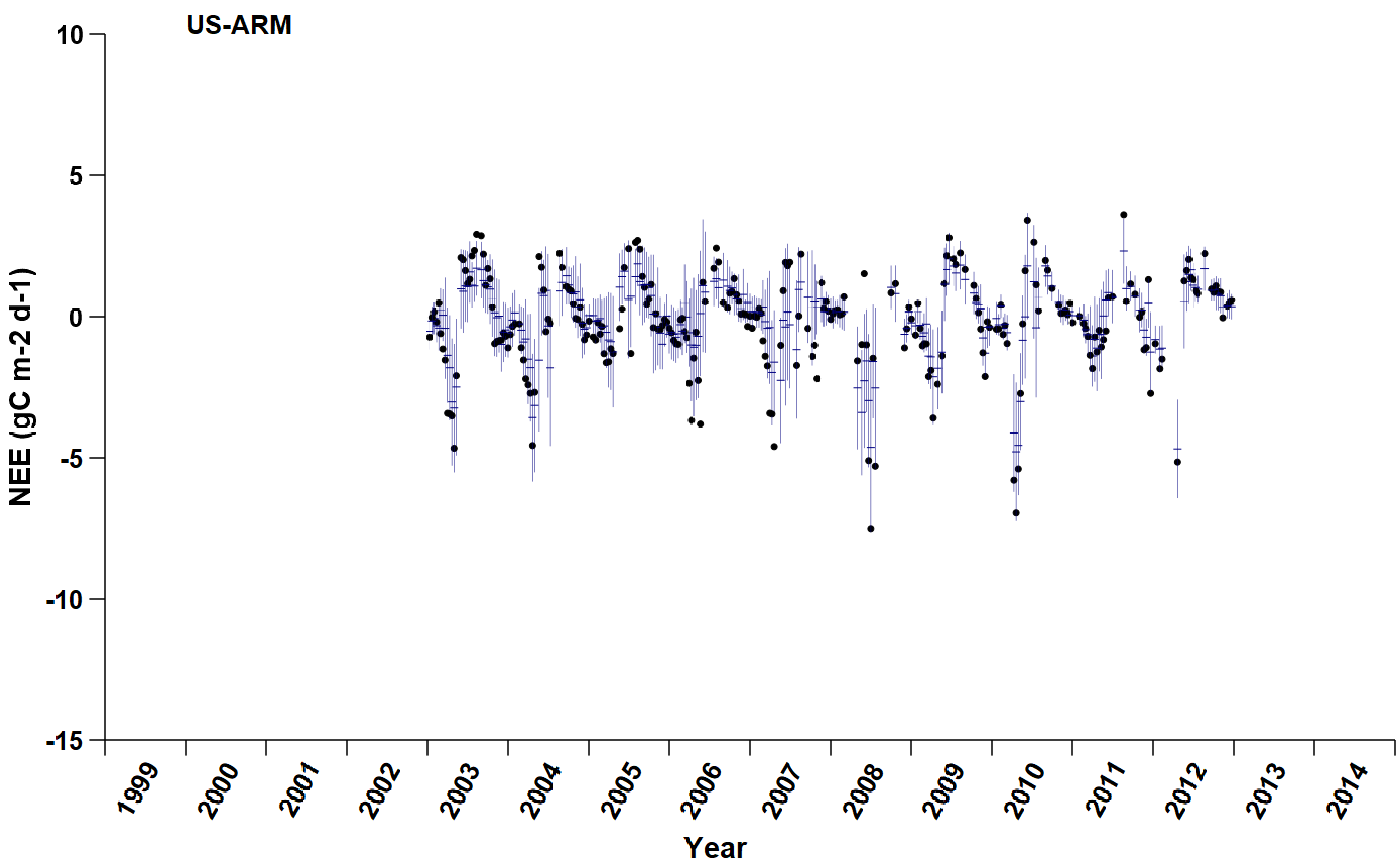
US-ARM
10
5
0
-5
-10
-15
NEE (gC m-2 d-1)
1999
2000
2001
2002
2003
2004
2005
2006
2007
2008
2009
2010
2011
2012
2013
2014
Year

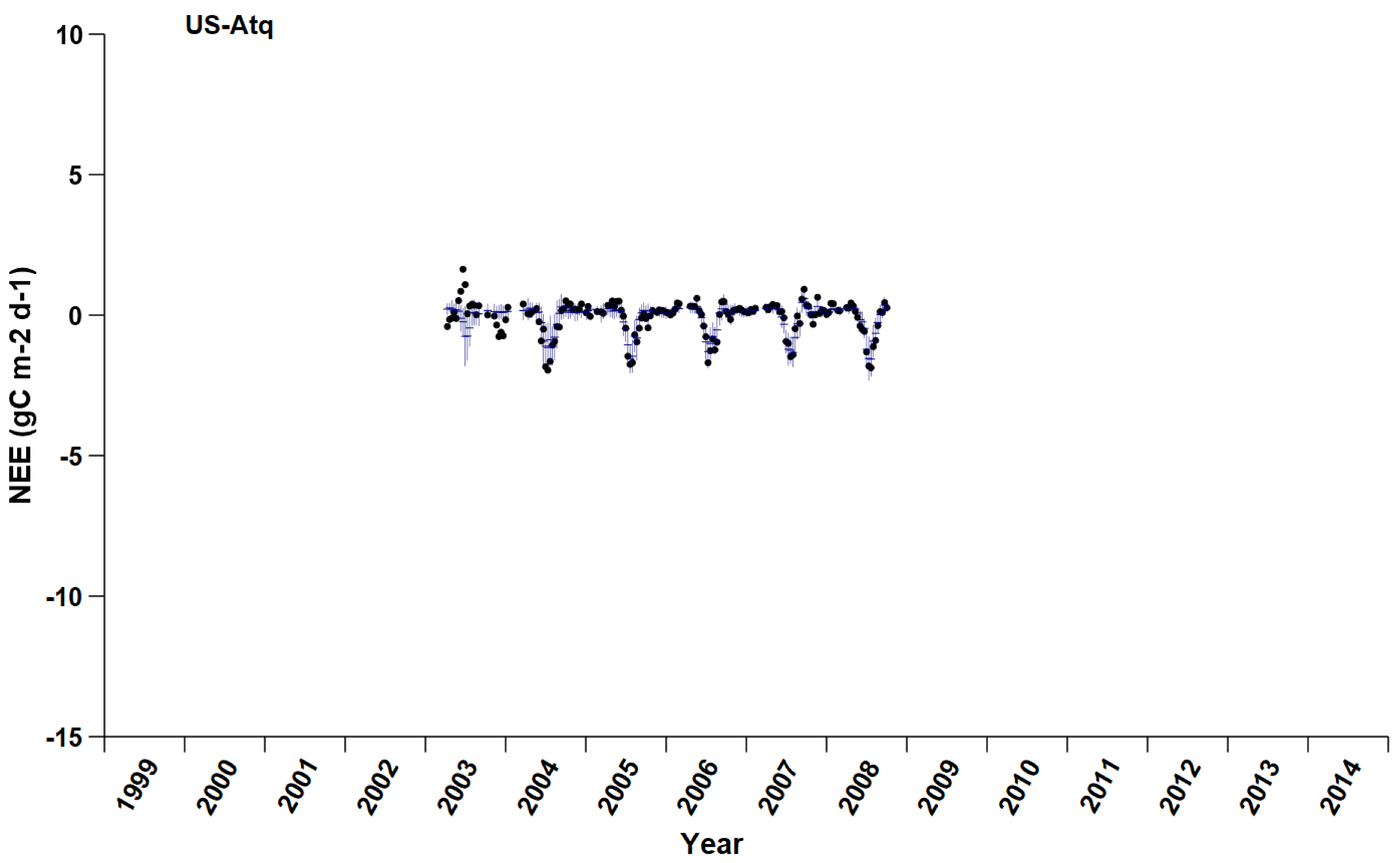

US-Atq
10
5
0
-5
-10
-15
NEE (gC m-2 d-1)
1999
2000
2001
2002
2003
2004
2005
2006
2007
2008
2009
2010
2011
2012
2013
2014
Year

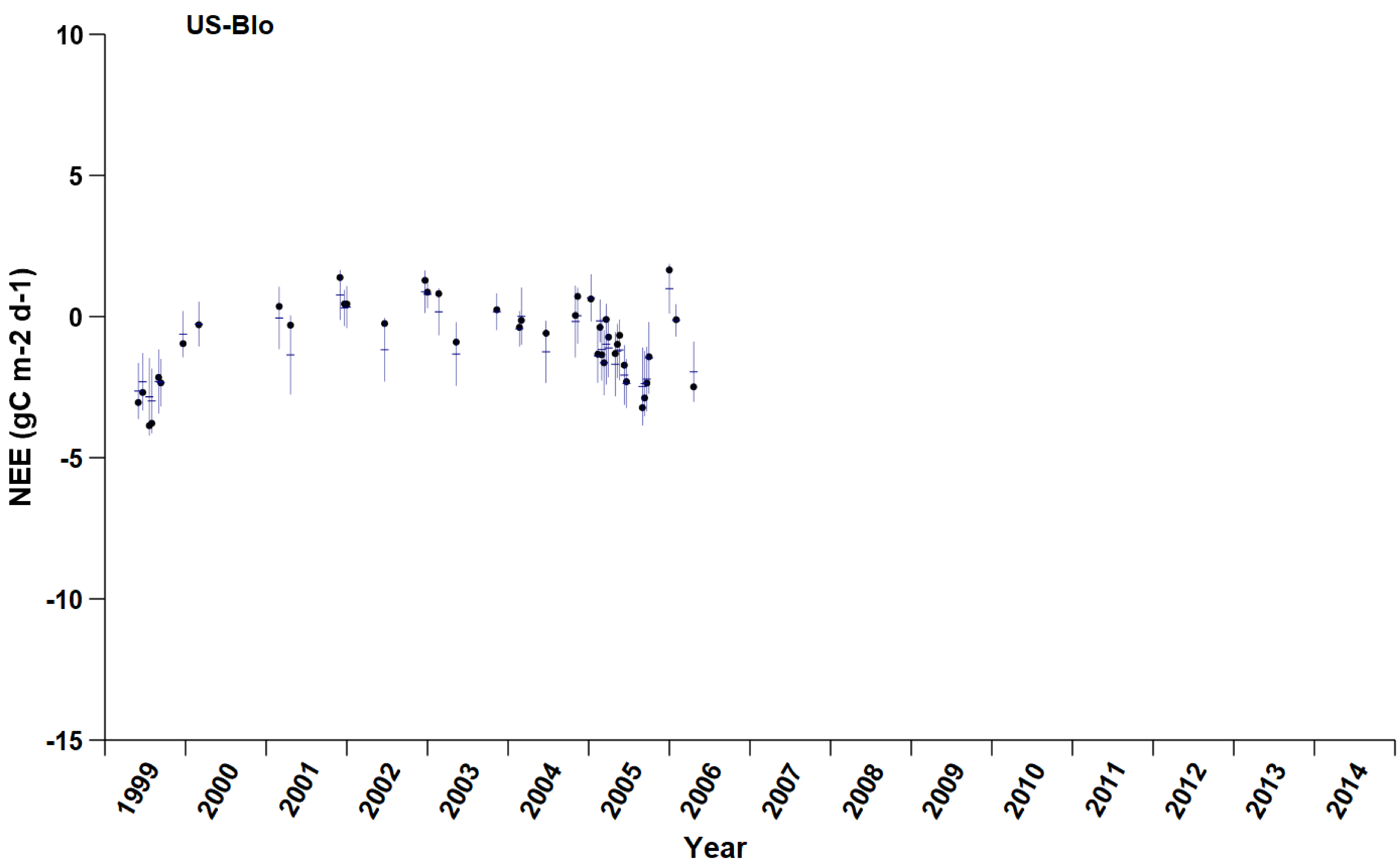

US-Blo
NEE (gC m-2 d-1)
10
5
0
-5
-10
-15
1999
2000
2001
2002
2003
2004
2005
2006
2007
2008
2009
2010
2011
2012
2013
2014
Year

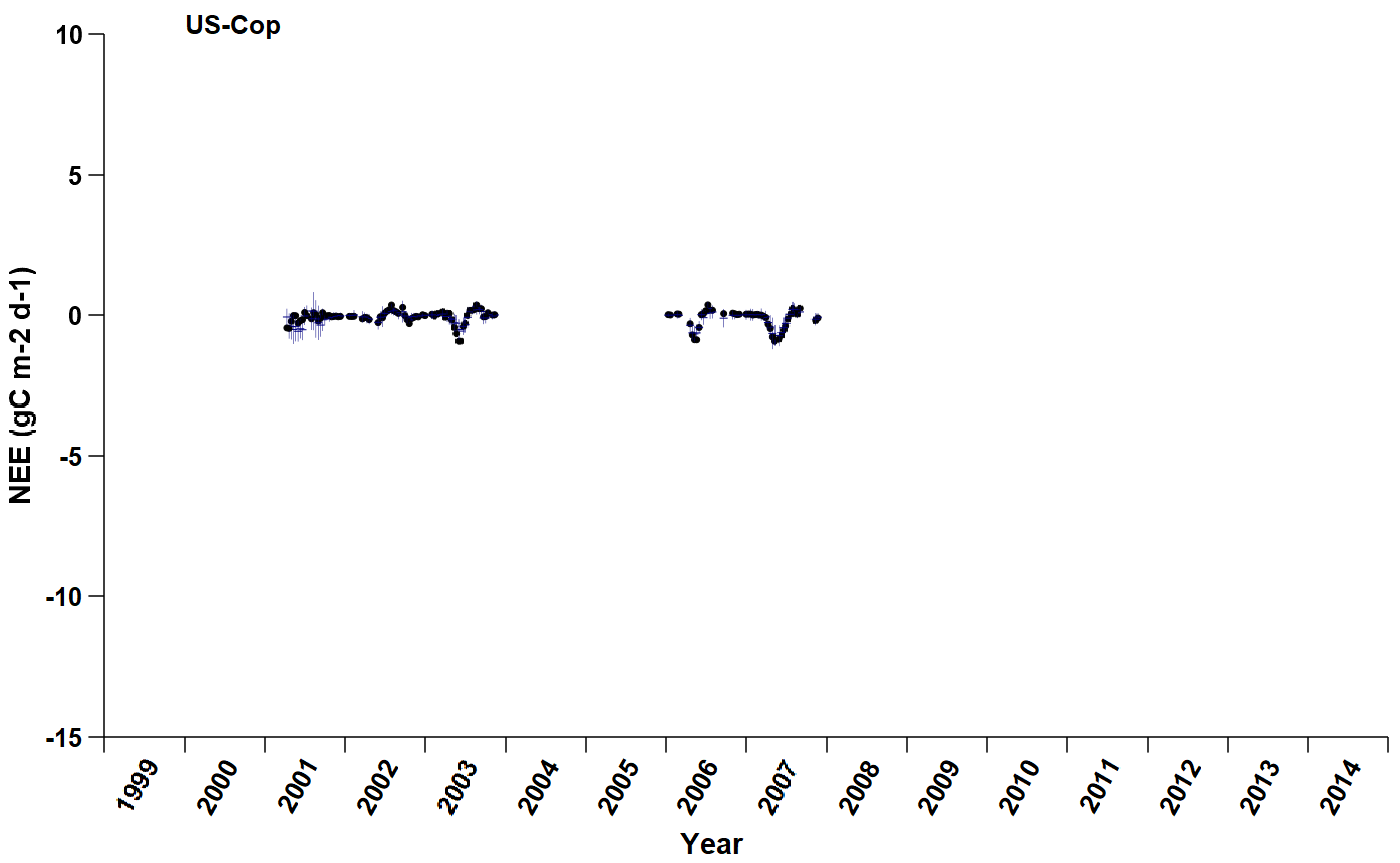
US-Cop
10
5
0
-5
-10
-15
NEE (gC m-2 d-1)
1999
2000
2001
2002
2003
2004
2005
2006
2007
2008
2009
2010
2011
2012
2013
2014
Year

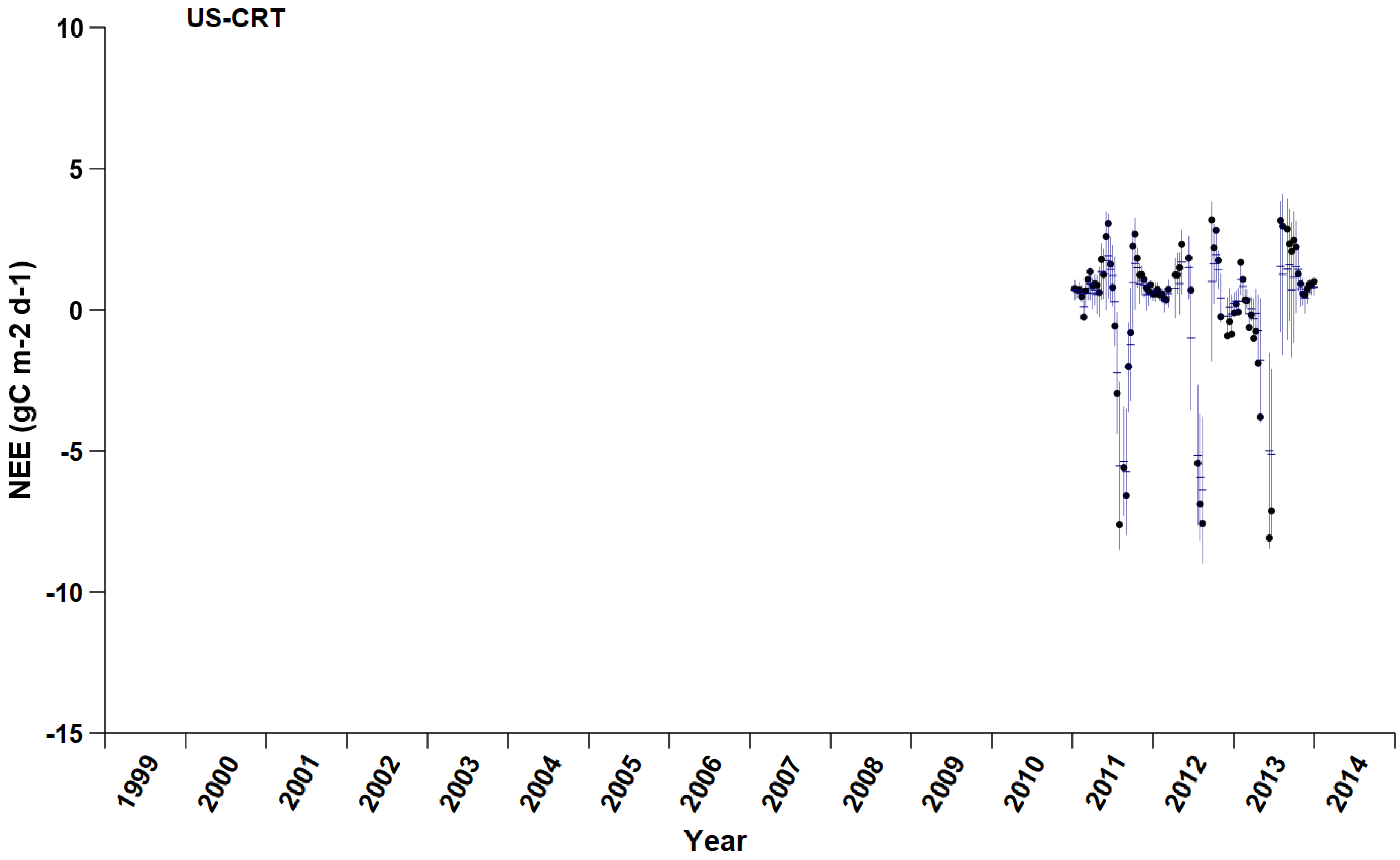
US-CRT
NEE (gC m-2 d-1)
10
5
0
-5
-10
-15
1999
2000
2001
2002
2003
2004
2005
2006
2007
2008
2009
2010
2011
2012
2013
2014
Year

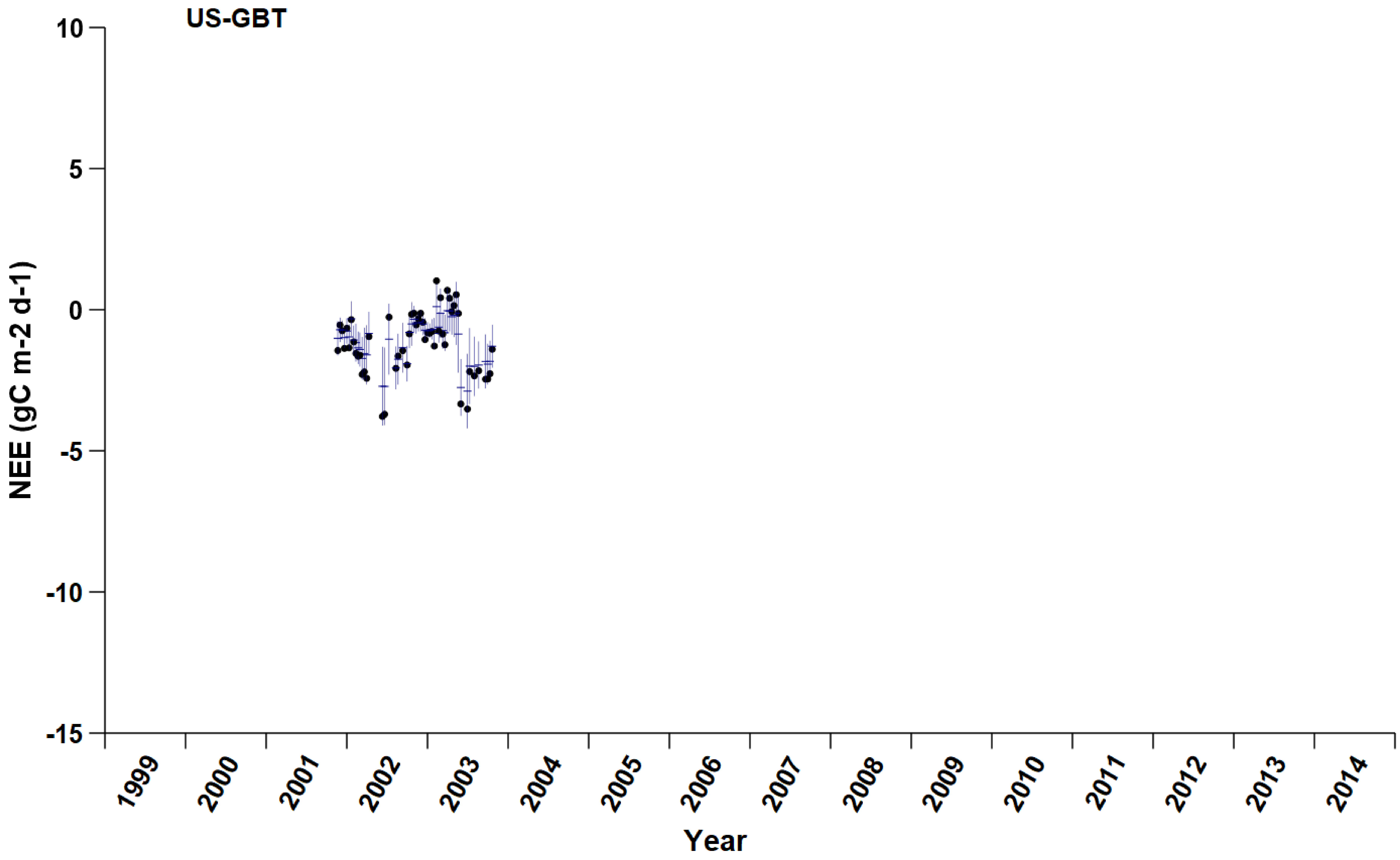

US-GBT
NEE (gC m-2 d-1)
10
5
0
-5
-10
-15
1999
2000
2001
2002
2003
2004
2005
2006
2007
2008
2009
2010
2011
2012
2013
2014
Year

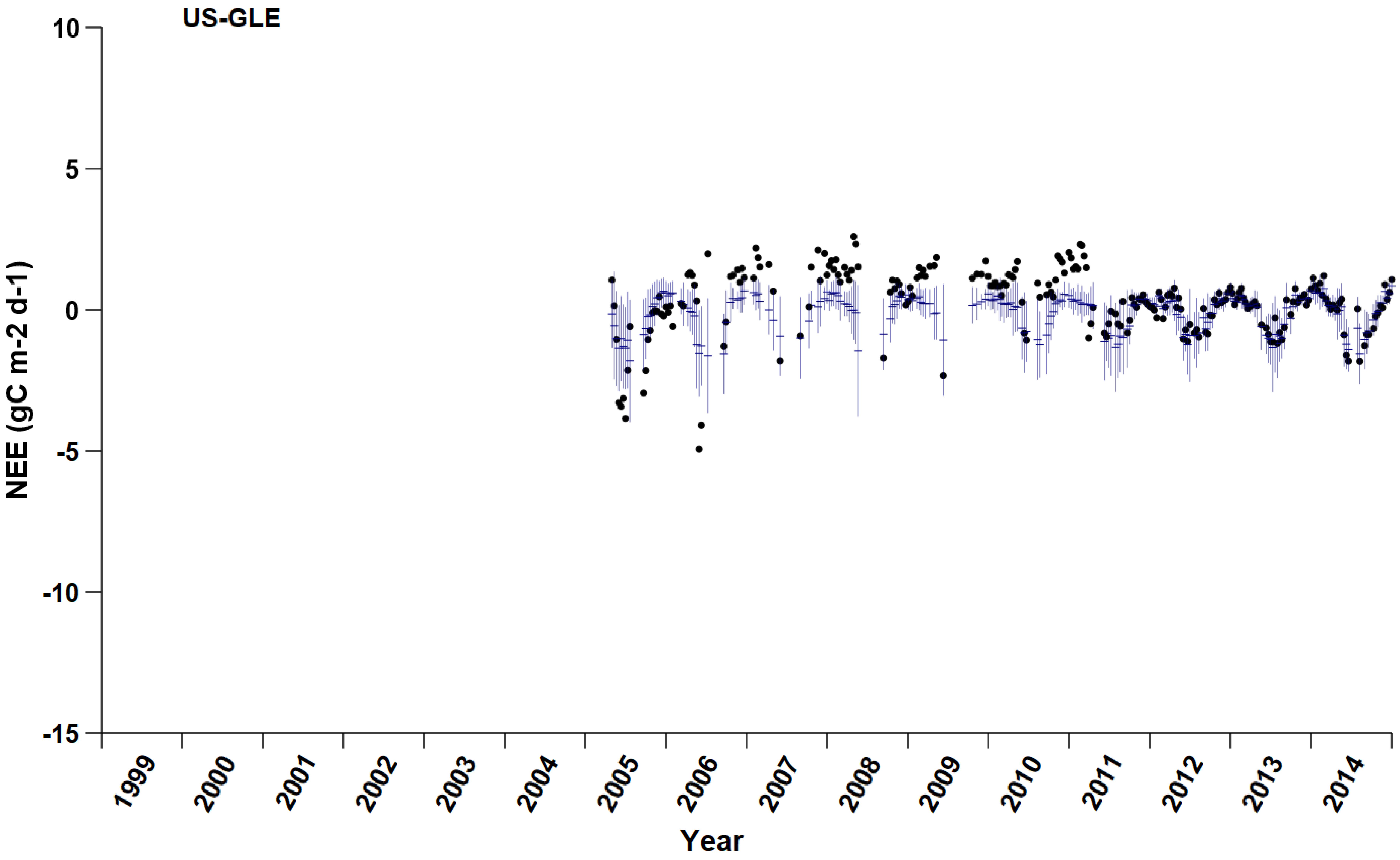
US-GLE
NEE (gC m-2 d-1)
10
5
0
-5
-10
-15
1999
2000
2001
2002
2003
2004
2005
2006
2007
2008
2009
2010
2011
2012
2013
2014
Year

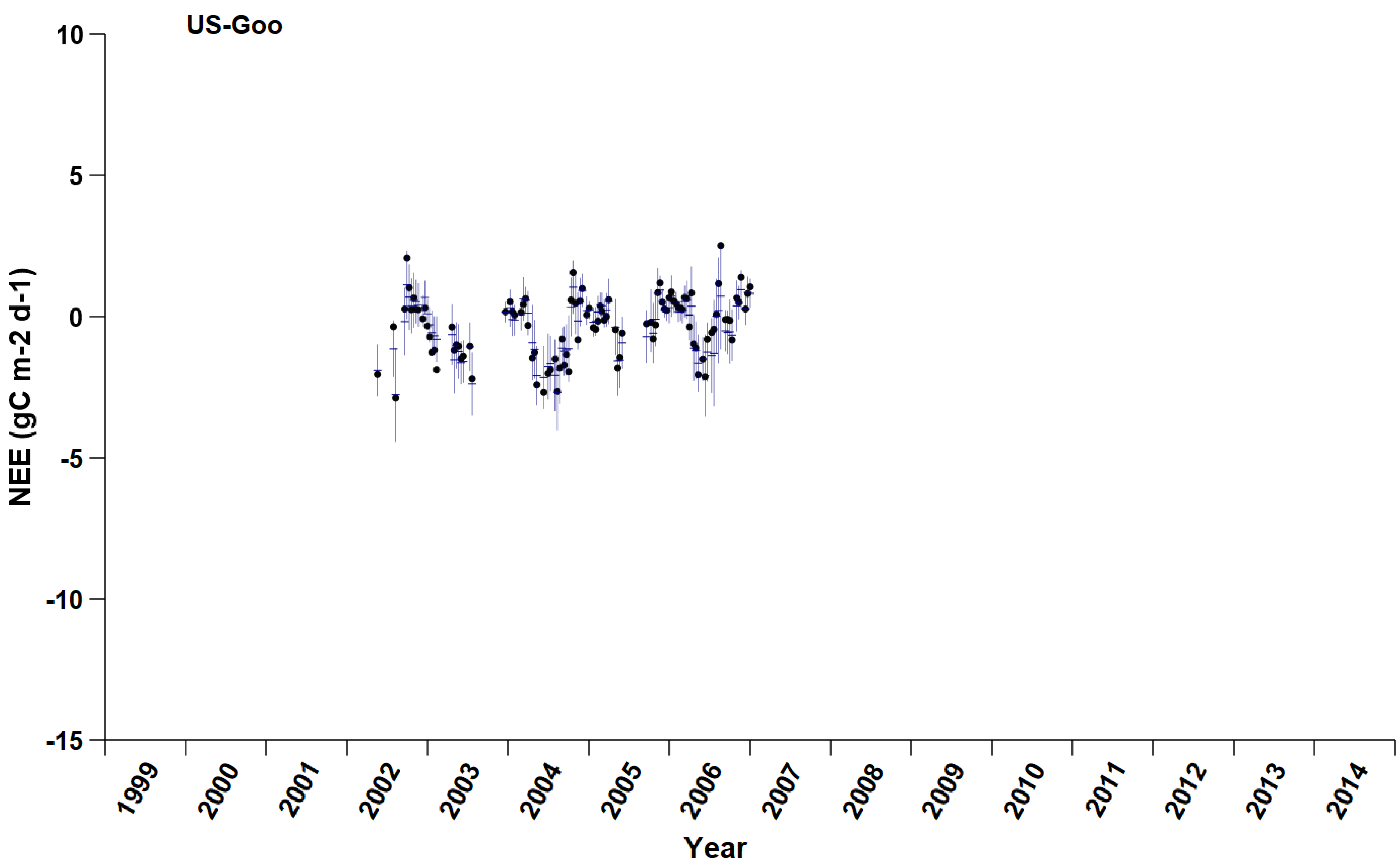
US-Goo
NEE (gC m-2 d-1)
10
5
0
-5
-10
-15
1999
2000
2001
2002
2003
2004
2005
2006
2007
2008
2009
2010
2011
2012
2013
2014
Year

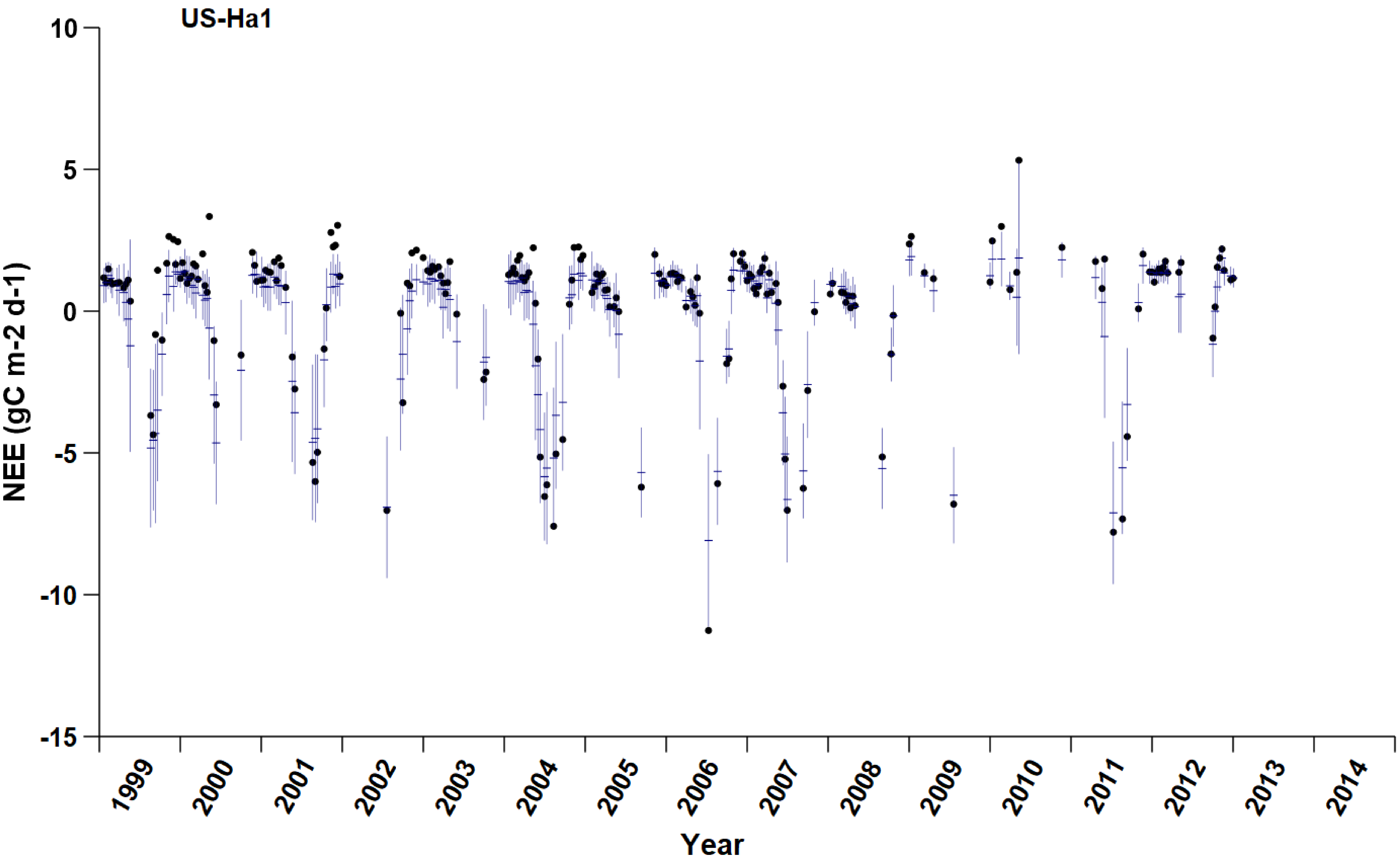

US-Ha1
NEE (gC m-2 d-1)
10
5
0
-5
-10
-15
1999
2000
2001
2002
2003
2004
2005
2006
2007
2008
2009
2010
2011
2012
2013
2014
Year

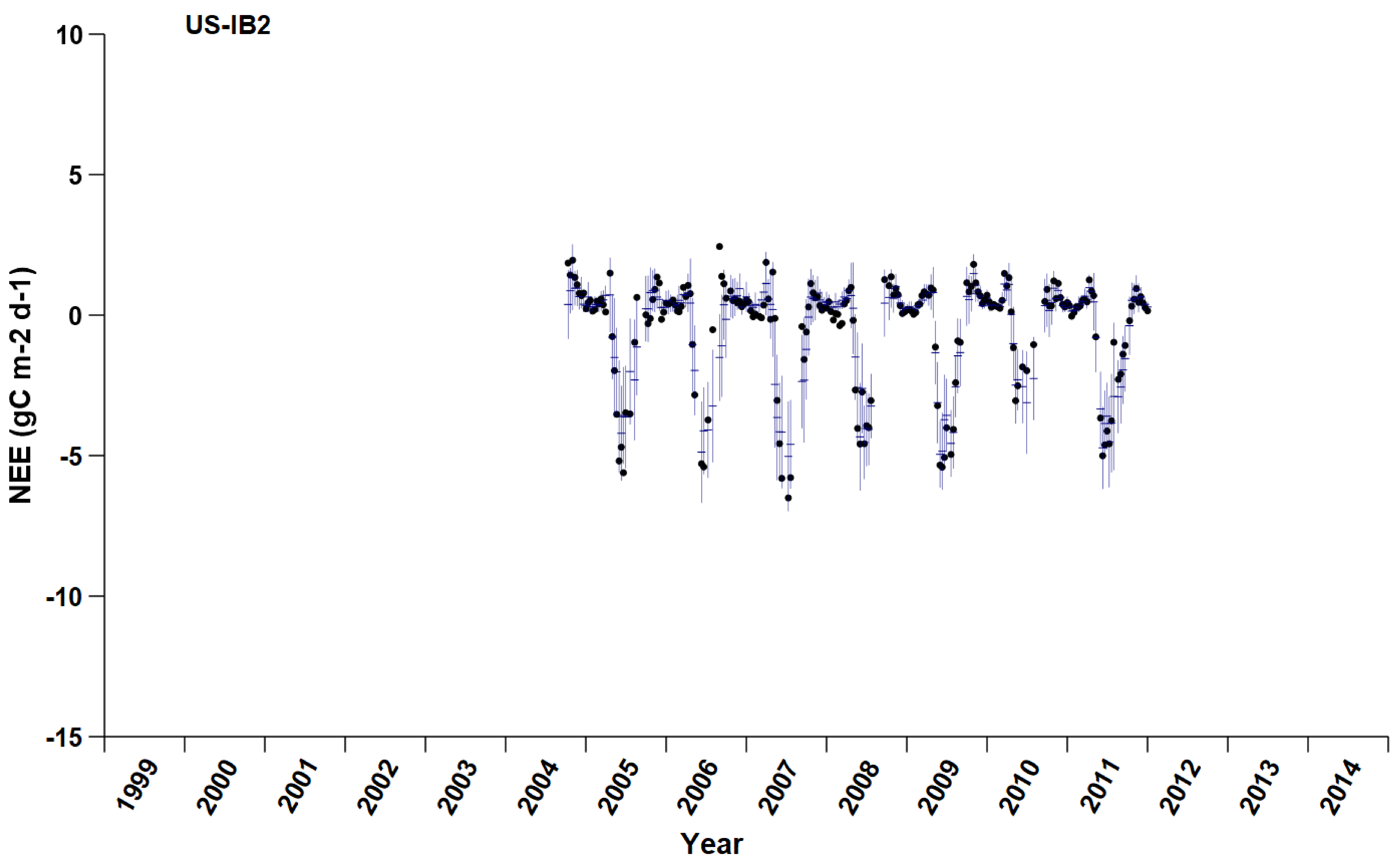

US-IB2
10
5
0
-5
-10
-15
NEE (gC m-2 d-1)
1999
2000
2001
2002
2003
2004
2005
2006
2007
2008
2009
2010
2011
2012
2013
2014
Year

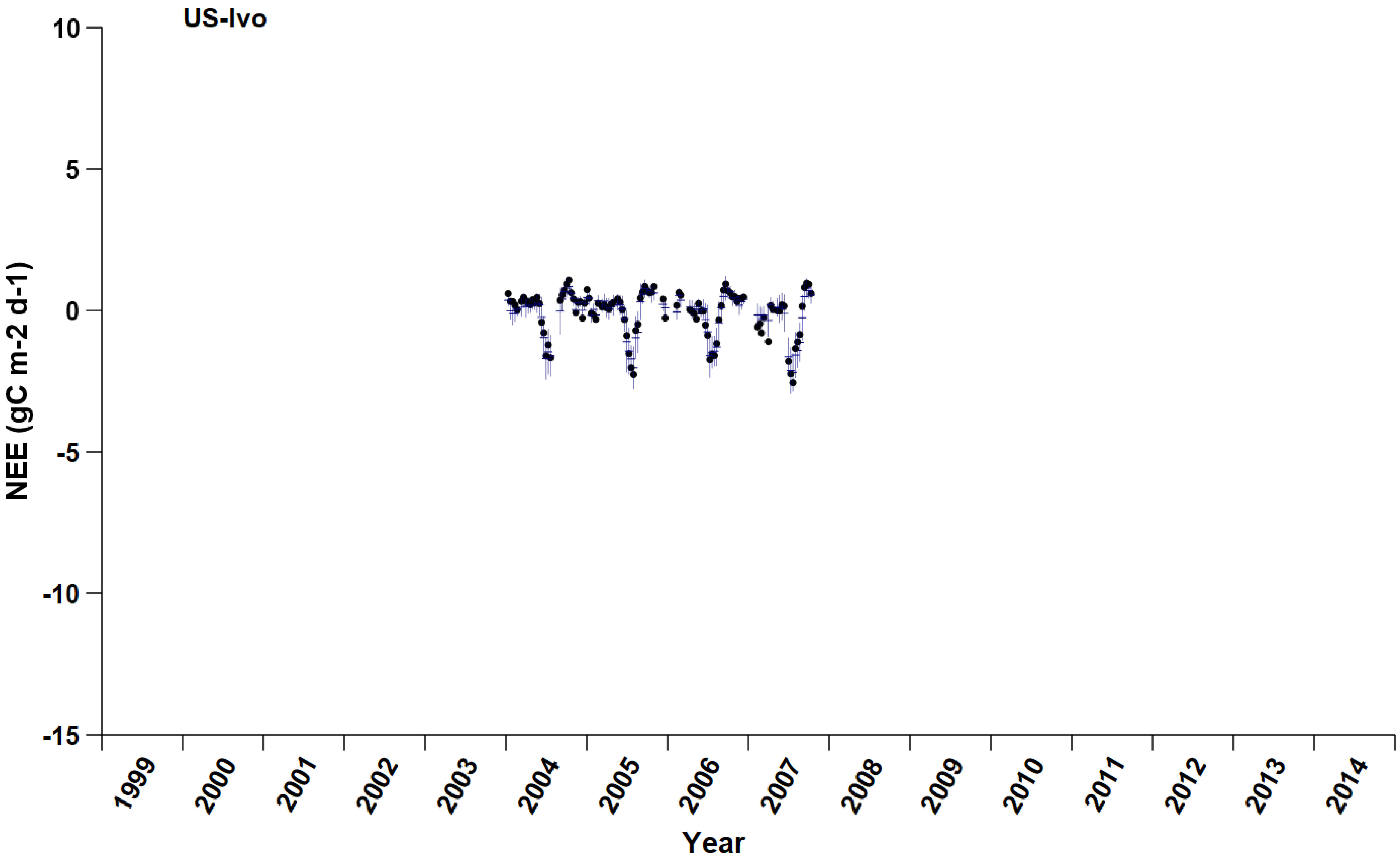

US-Ivo
10
5
0
-5
-10
-15
NEE (gC m-2 d-1)
1999
2000
2001
2002
2003
2004
2005
2006
2007
2008
2009
2010
2011
2012
2013
2014
Year

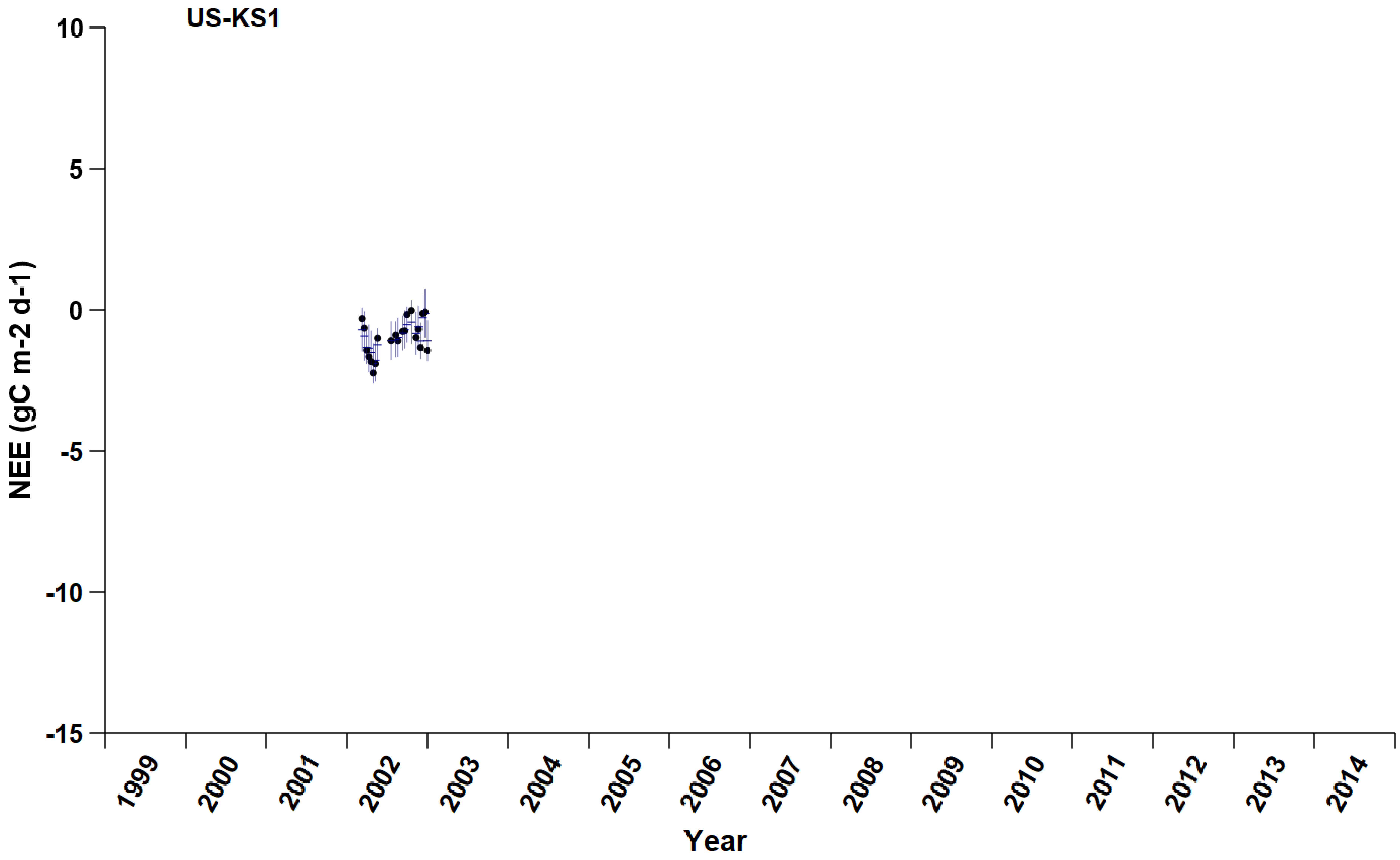

US-KS1
10
5
0
-5
-10
-15
NEE (gC m-2 d-1)
1999
2000
2001
2002
2003
2004
2005
2006
2007
2008
2009
2010
2011
2012
2013
2014
Year

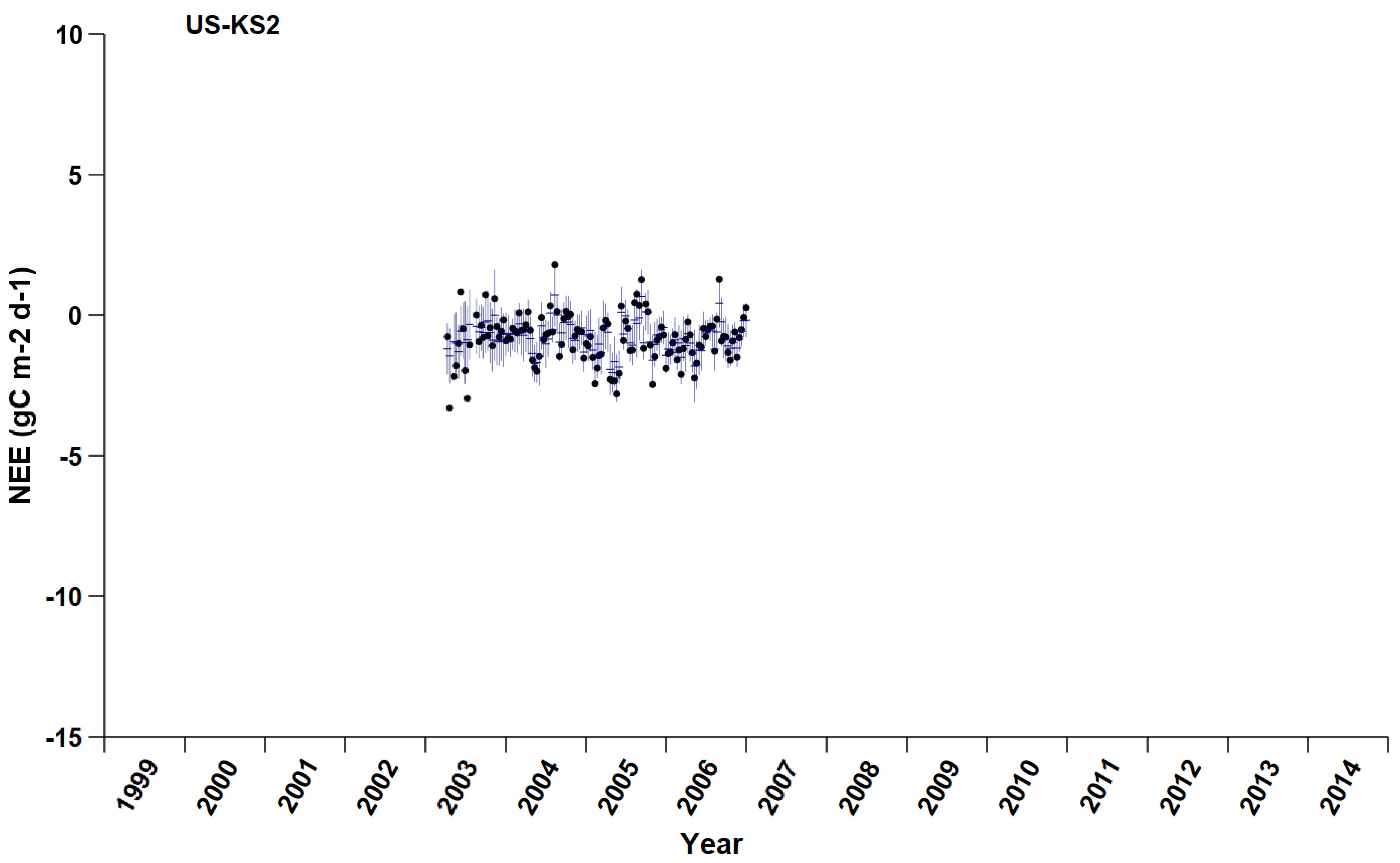
US-KS2
NEE (gC m-2 d-1)
10
5
0
-5
-10
-15
1999
2000
2001
2002
2003
2004
2005
2006
2007
2008
2009
2010
2011
2012
2013
2014
Year

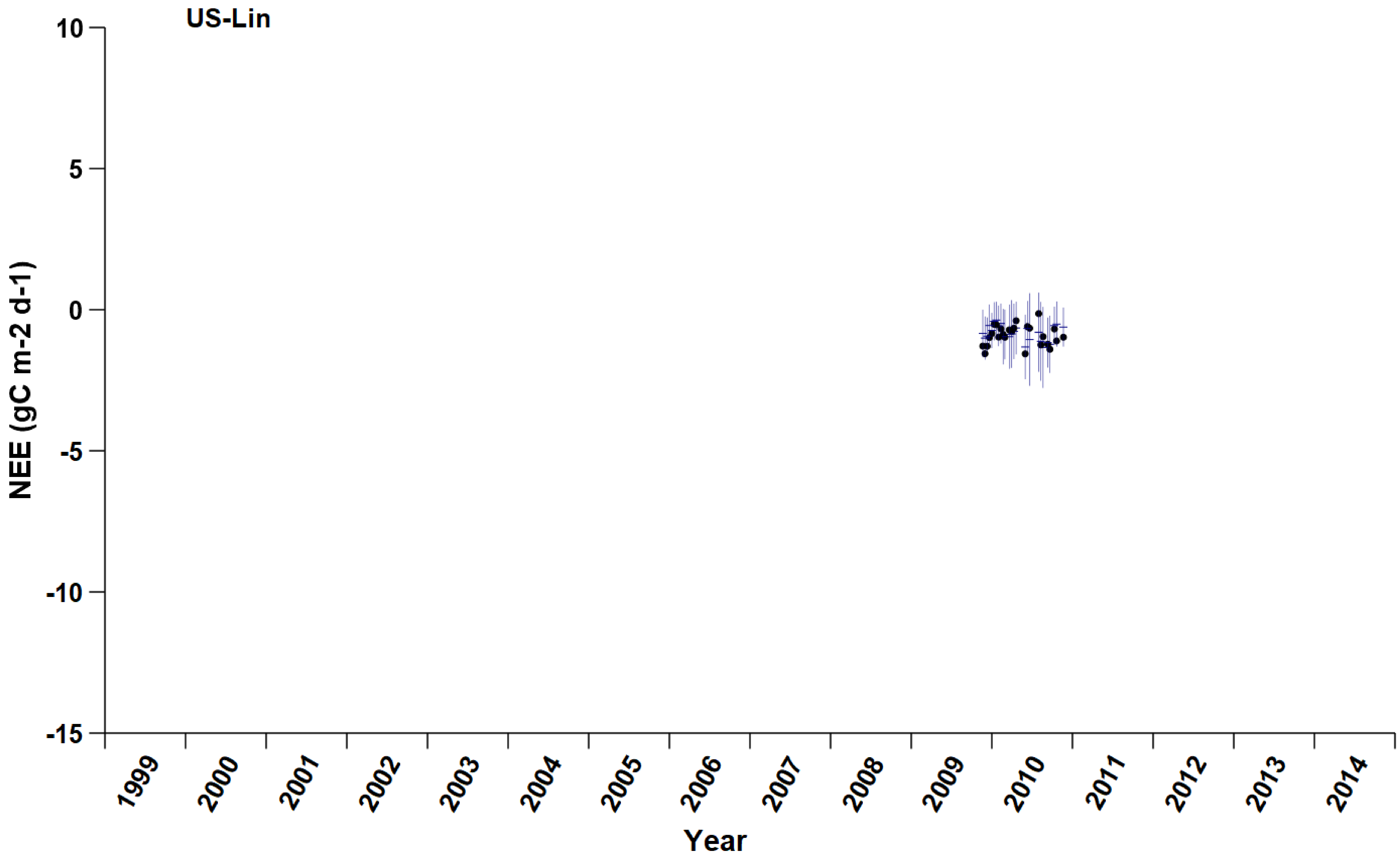

US-Lin
10
5
0
-5
-10
-15
NEE (gC m-2 d-1)
1999
2000
2001
2002
2003
2004
2005
2006
2007
2008
2009
2010
2011
2012
2013
2014
Year

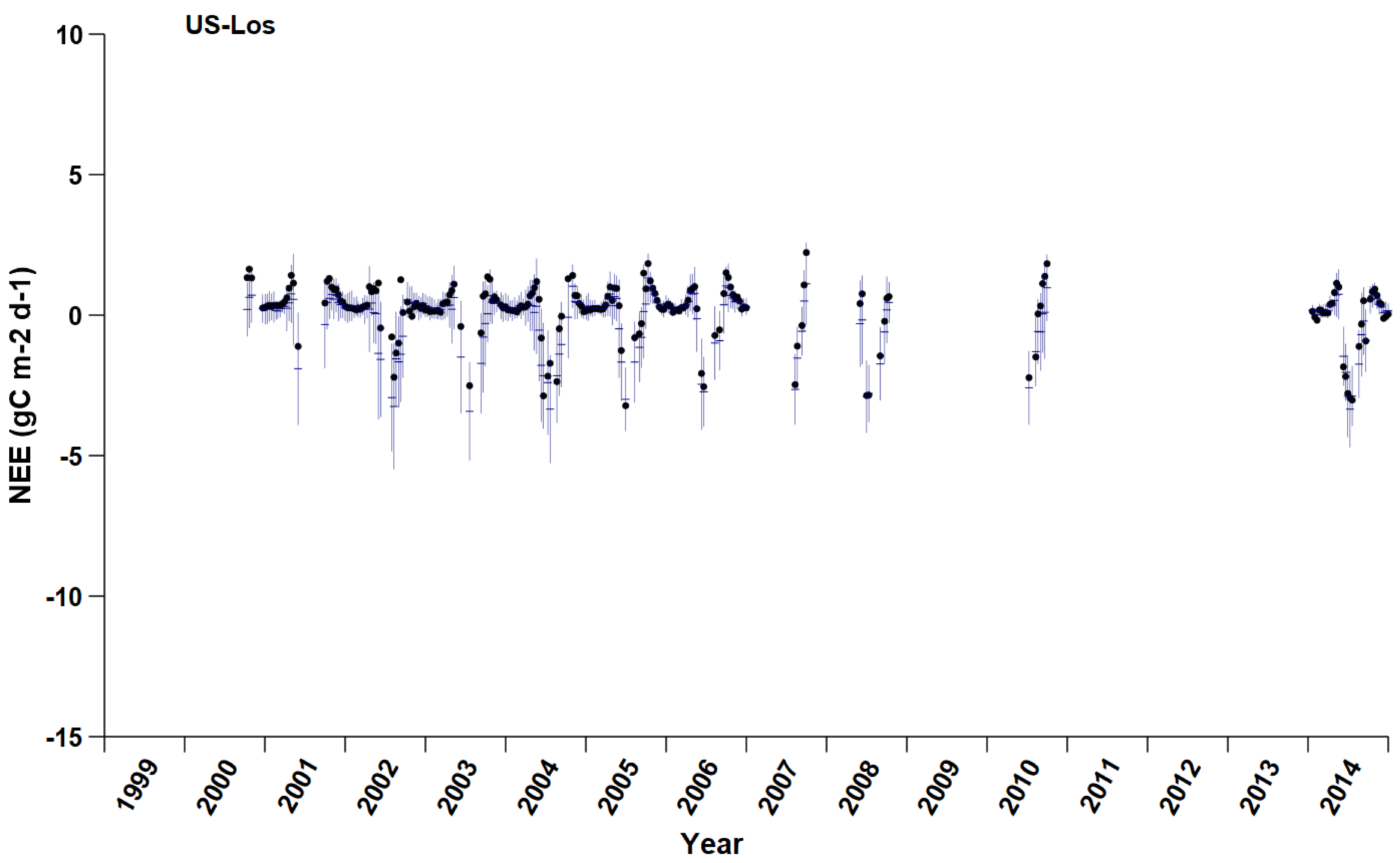

US-Los
10
5
0
-5
-10
-15
NEE (gC m-2 d-1)
1999
2000
2001
2002
2003
2004
2005
2006
2007
2008
2009
2010
2011
2012
2013
2014
Year

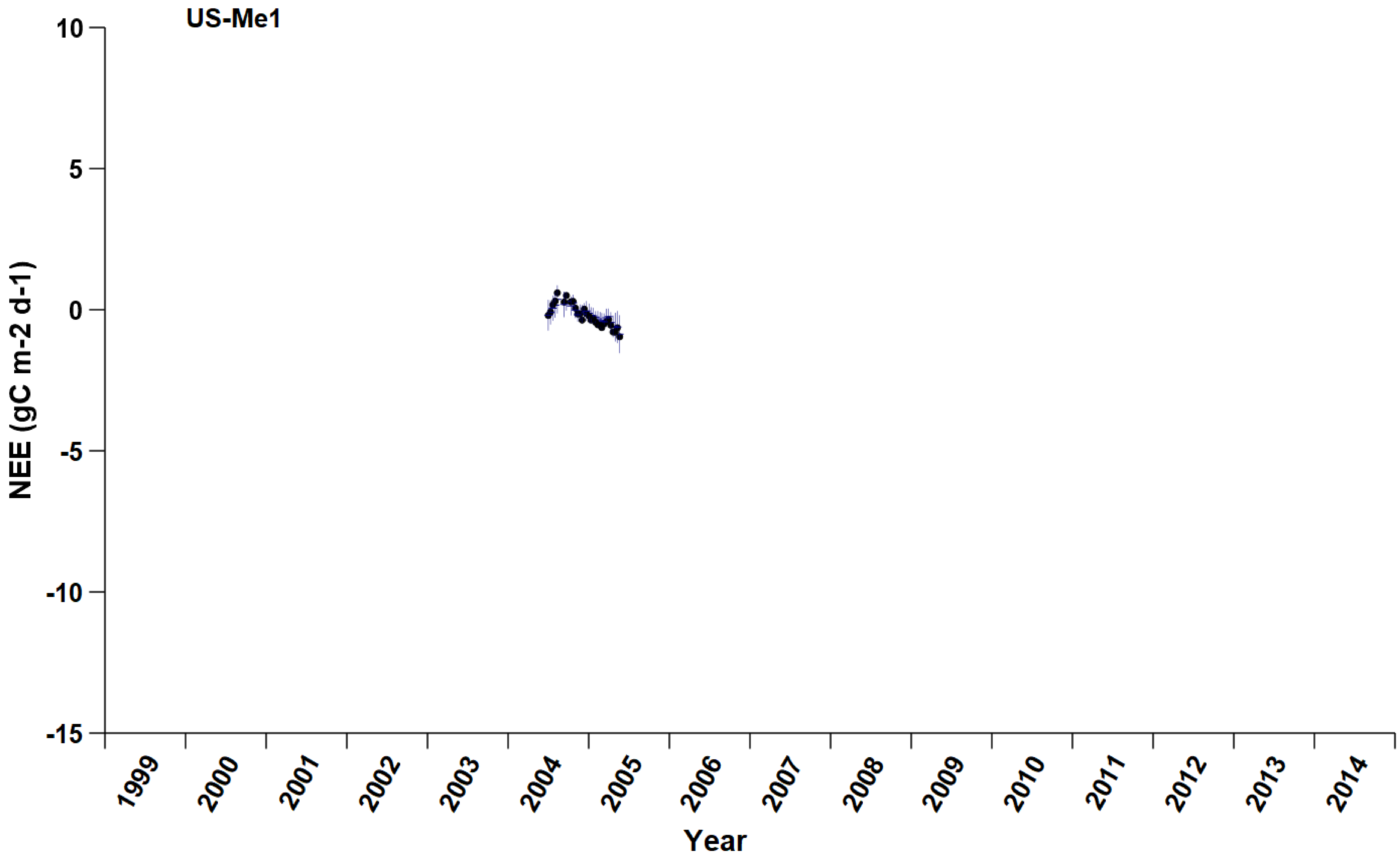
US-Me1
NEE (gC m-2 d-1)
10
5
0
-5
-10
-15
1999
2000
2001
2002
2003
2004
2005
2006
2007
2008
2009
2010
2011
2012
2013
2014
Year

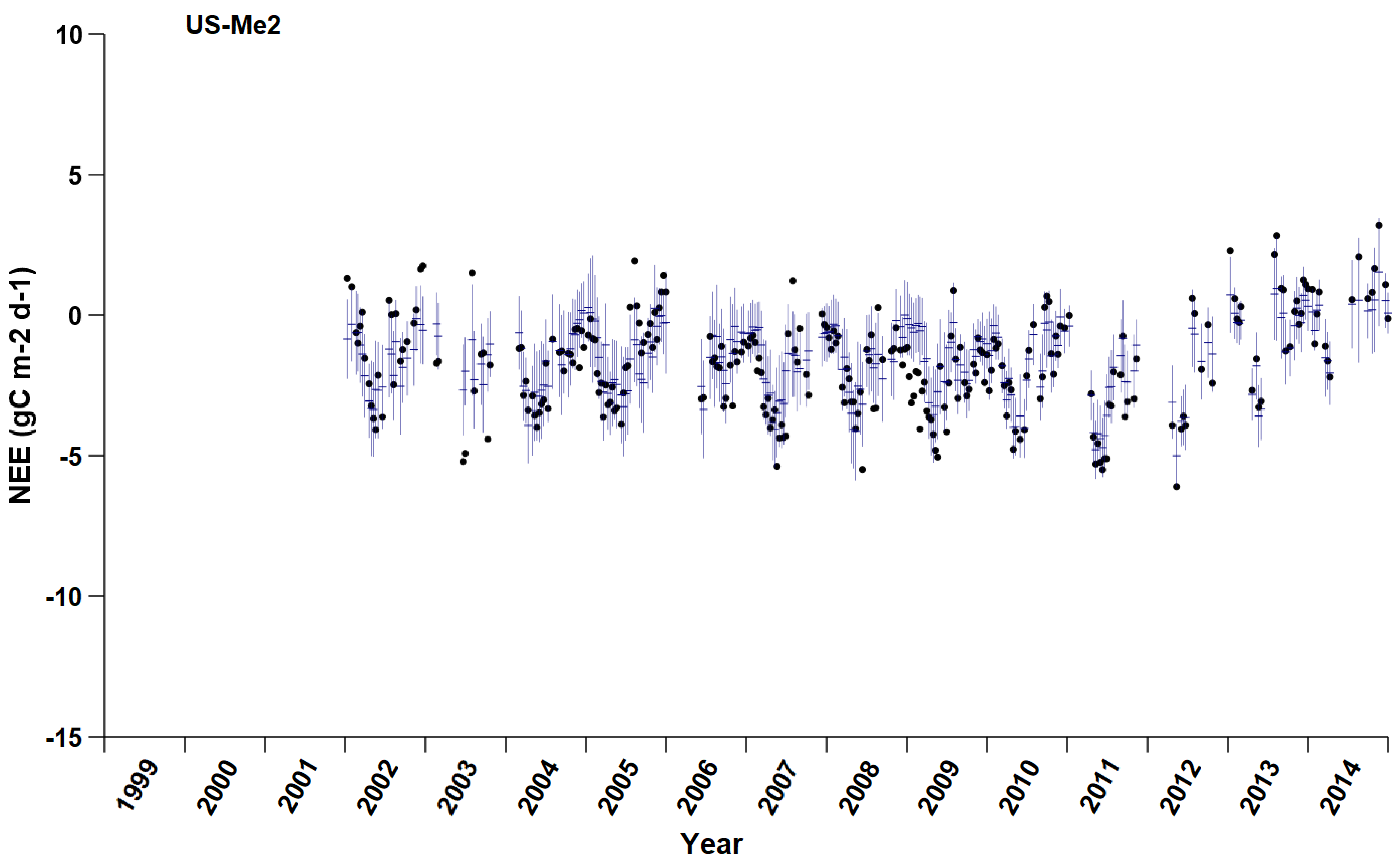

US-Me2
10
5
0
-5
-10
-15
NEE (gC m-2 d-1)
1999
2000
2001
2002
2003
2004
2005
2006
2007
2008
2009
2010
2011
2012
2013
2014
Year

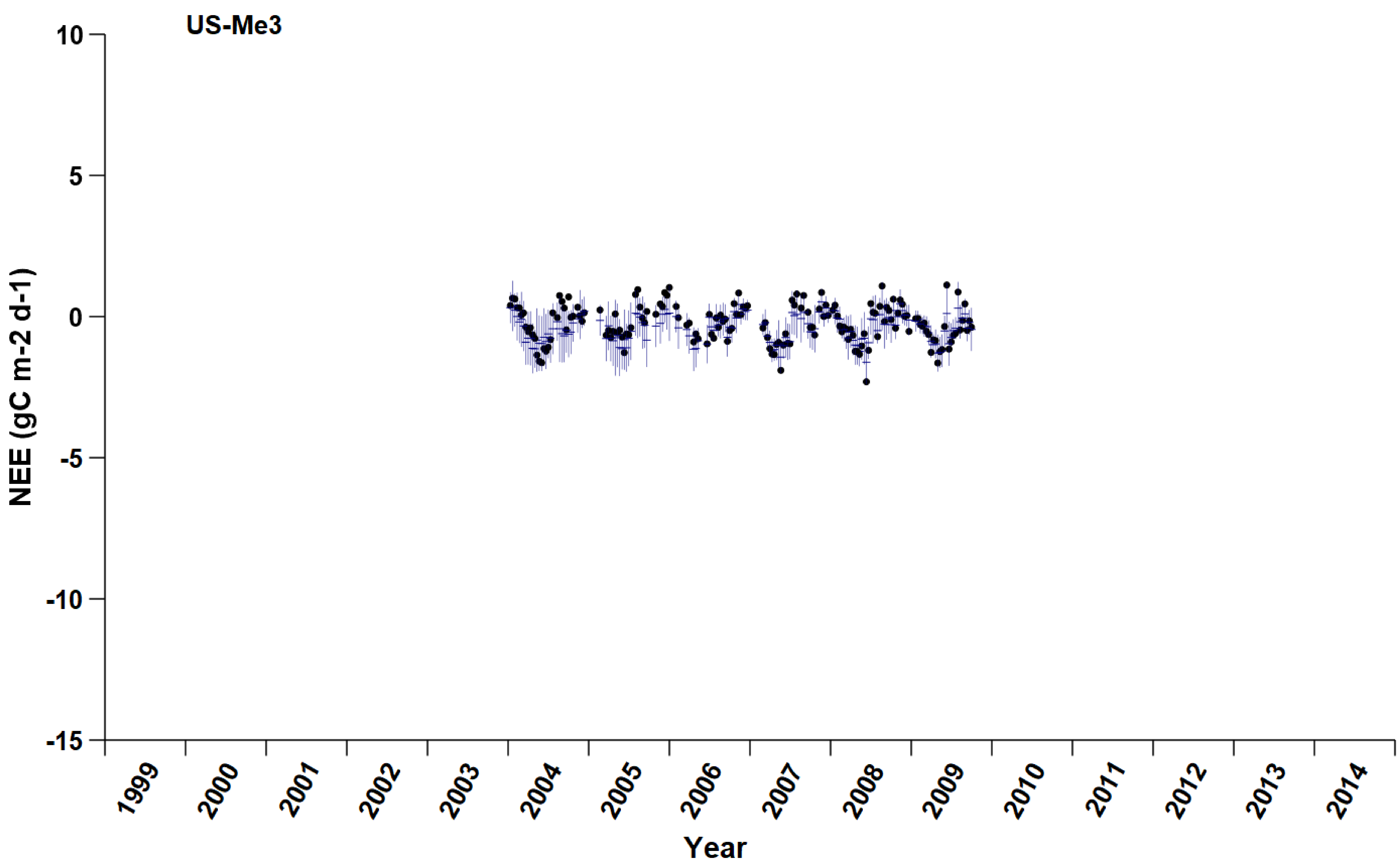
US-Me3
10
5
0
-5
-10
-15
NEE (gC m-2 d-1)
1999
2000
2001
2002
2003
2004
2005
2006
2007
2008
2009
2010
2011
2012
2013
2014
Year

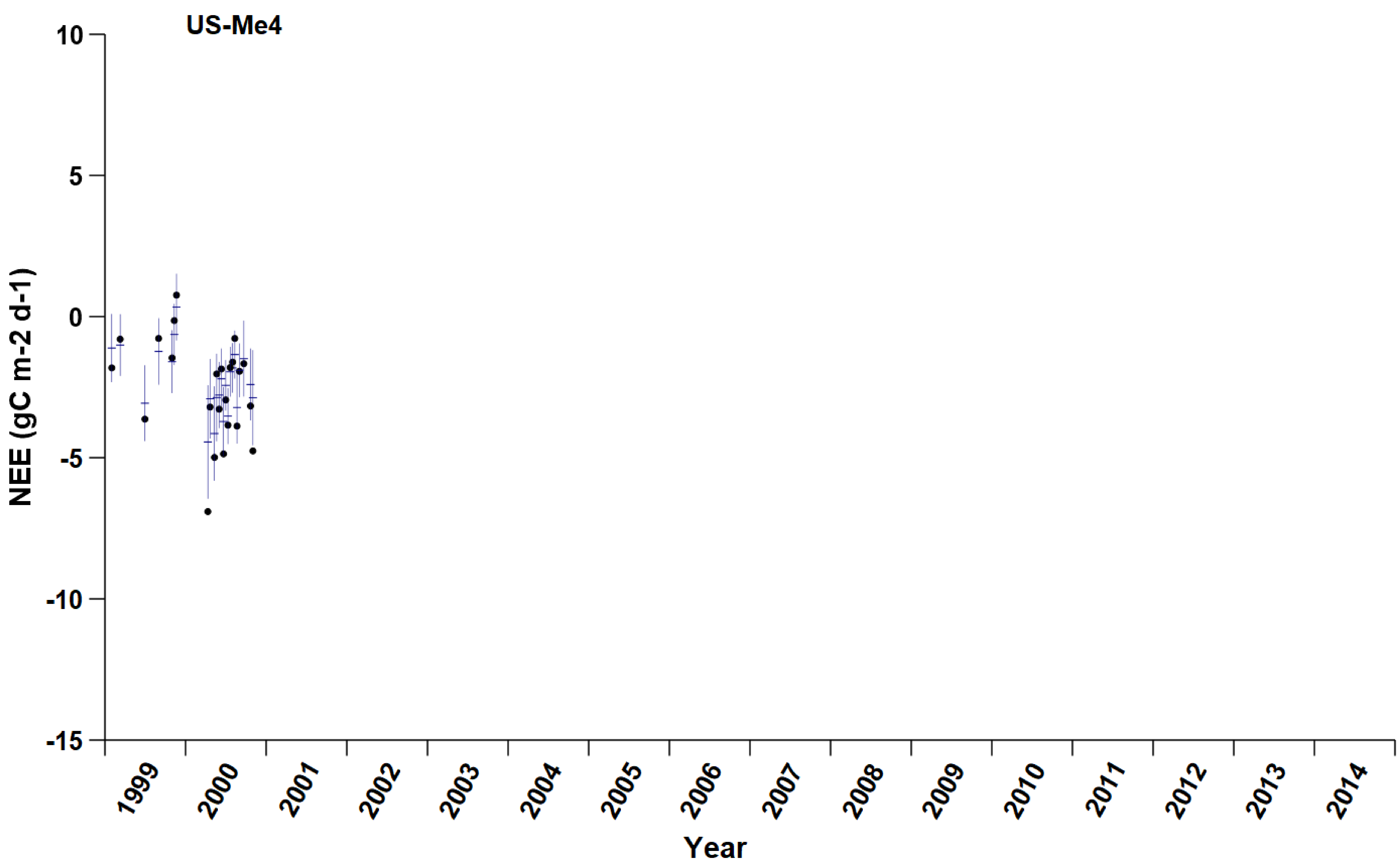
US-Me4
NEE (gC m-2 d-1)
10
5
0
-5
-10
-15
1999
2000
2001
2002
2003
2004
2005
2006
2007
2008
2009
2010
2011
2012
2013
2014
Year

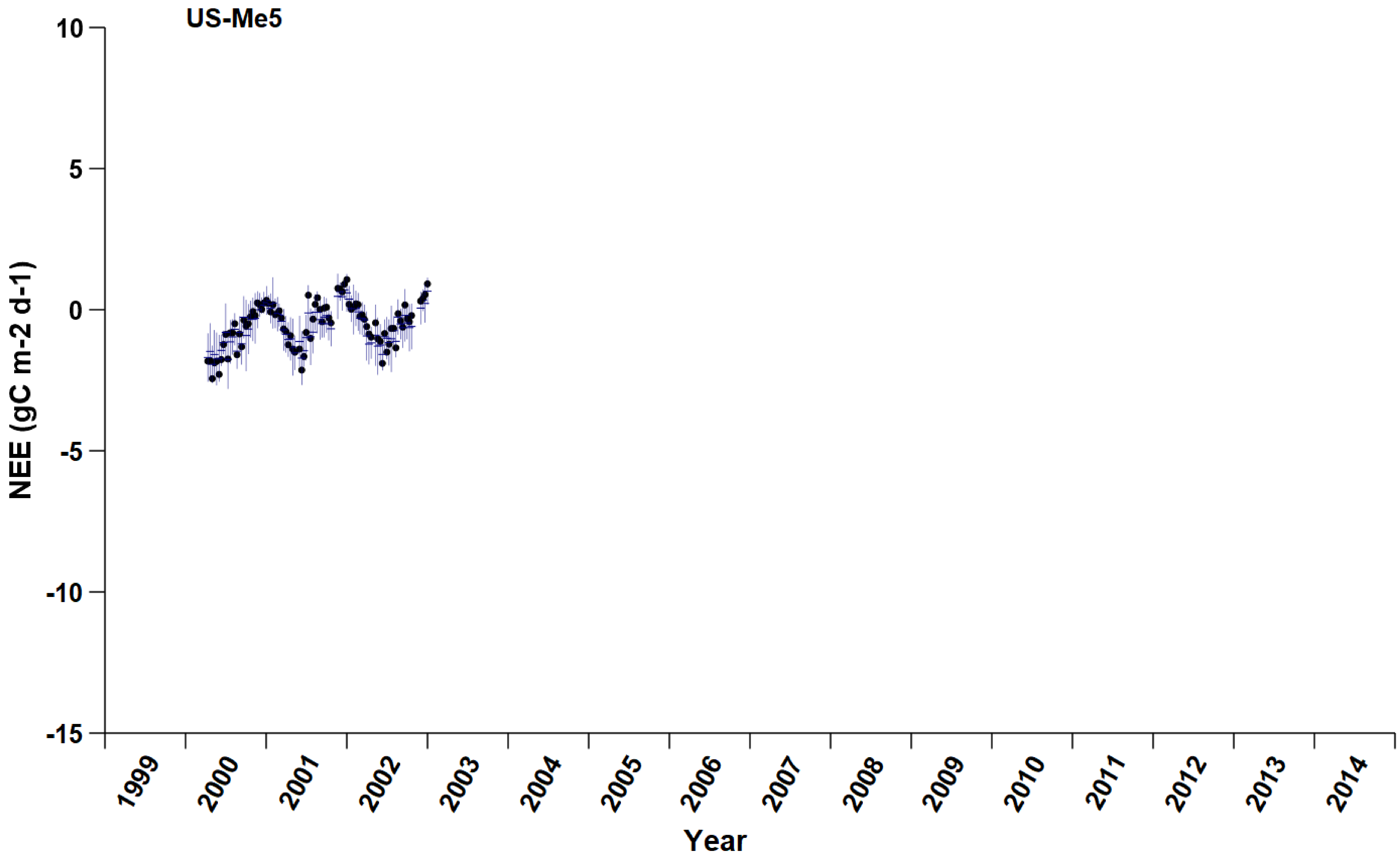

US-Me5
10
5
0
-5
-10
-15
NEE (gC m-2 d-1)
1999
2000
2001
2002
2003
2004
2005
2006
2007
2008
2009
2010
2011
2012
2013
2014
Year

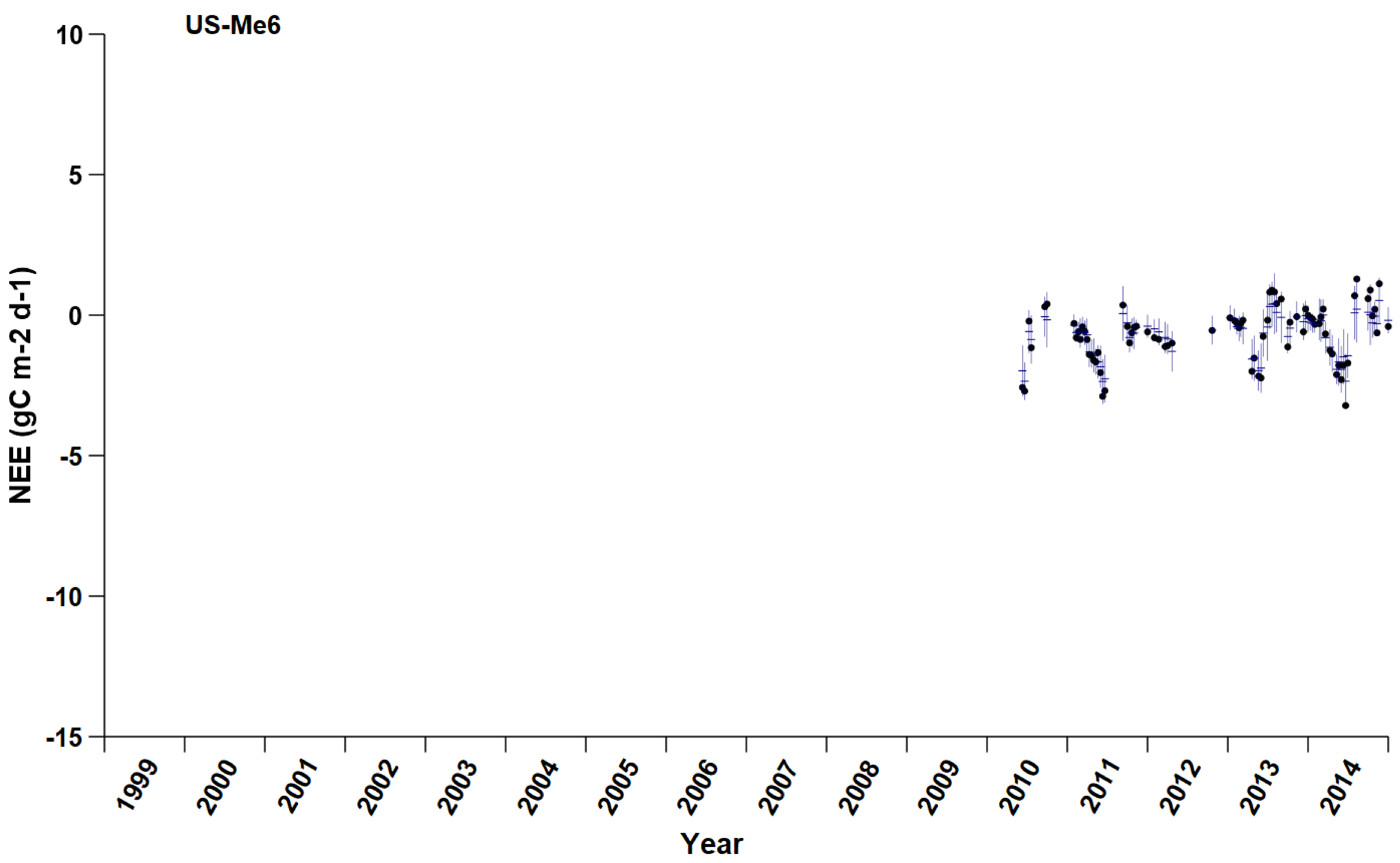

US-Me6
NEE (gC m-2 d-1)
10
5
0
-5
-10
-15
1999
2000
2001
2002
2003
2004
2005
2006
2007
2008
2009
2010
2011
2012
2013
2014
Year

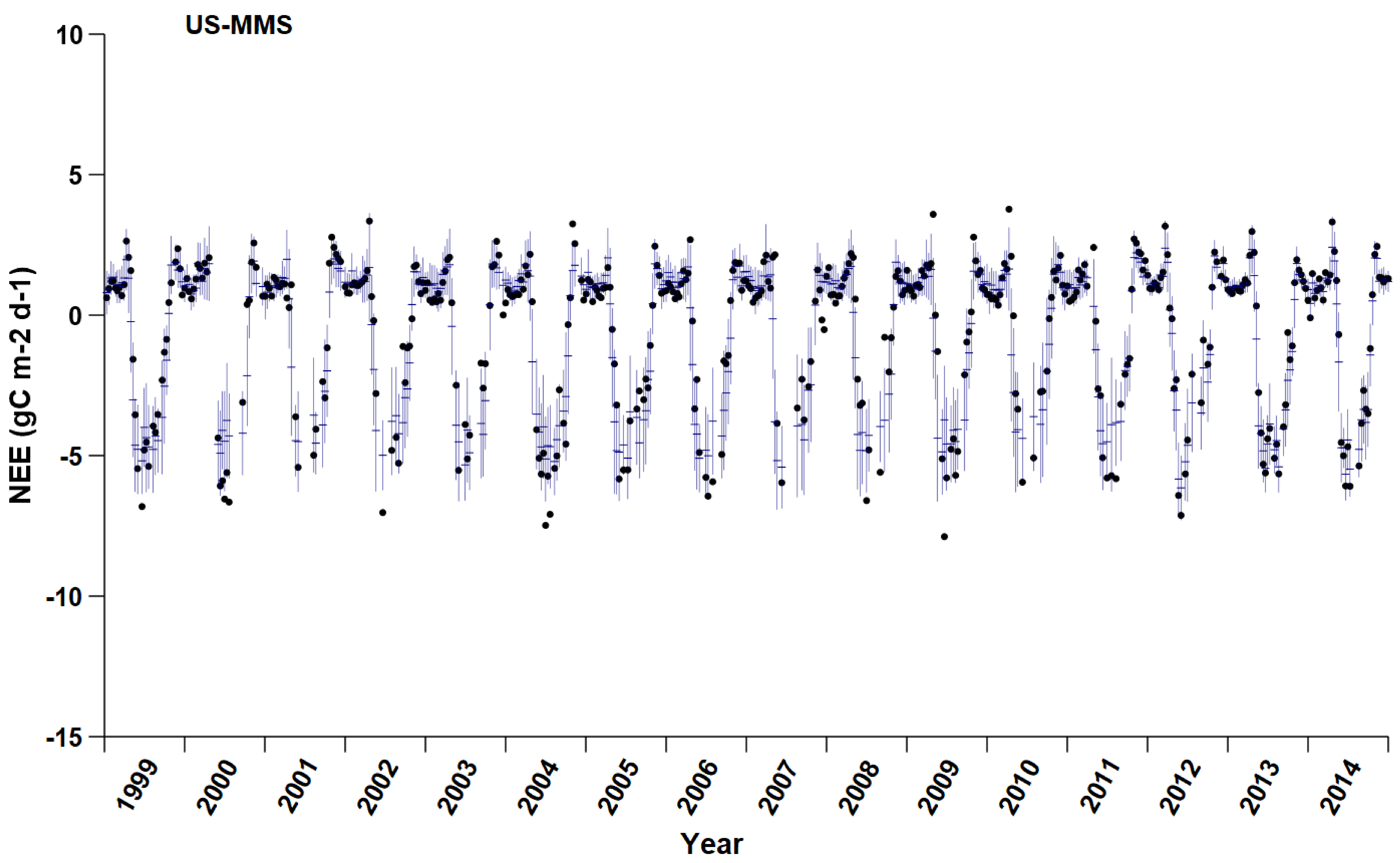

US-MMS
NEE (gC m-2 d-1)
10
5
0
-5
-10
-15
1999
2000
2001
2002
2003
2004
2005
2006
2007
2008
2009
2010
2011
2012
2013
2014
Year

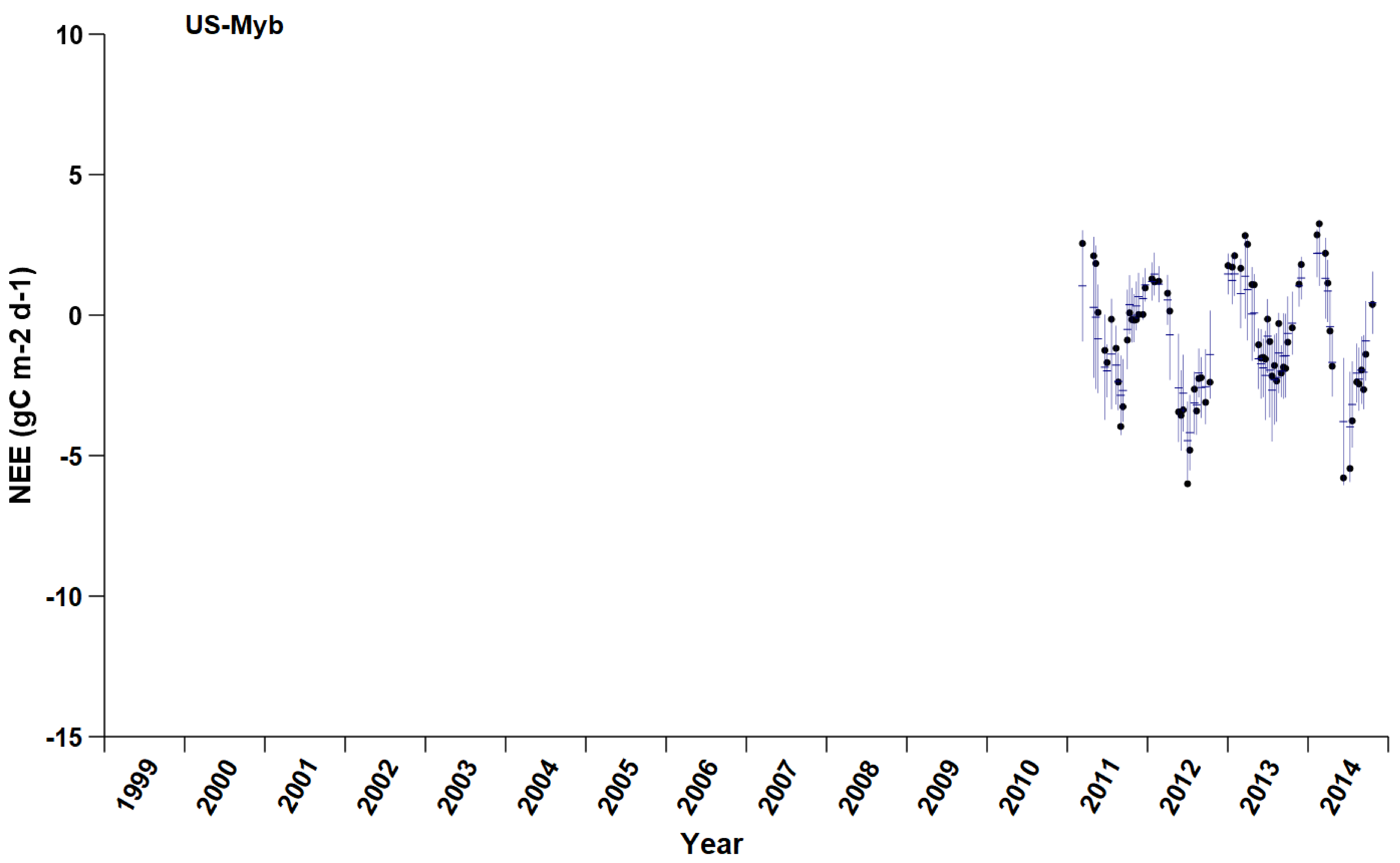

US-Myb
NEE (gC m-2 d-1)
10
5
0
-5
-10
-15
1999
2000
2001
2002
2003
2004
2005
2006
2007
2008
2009
2010
2011
2012
2013
2014
Year

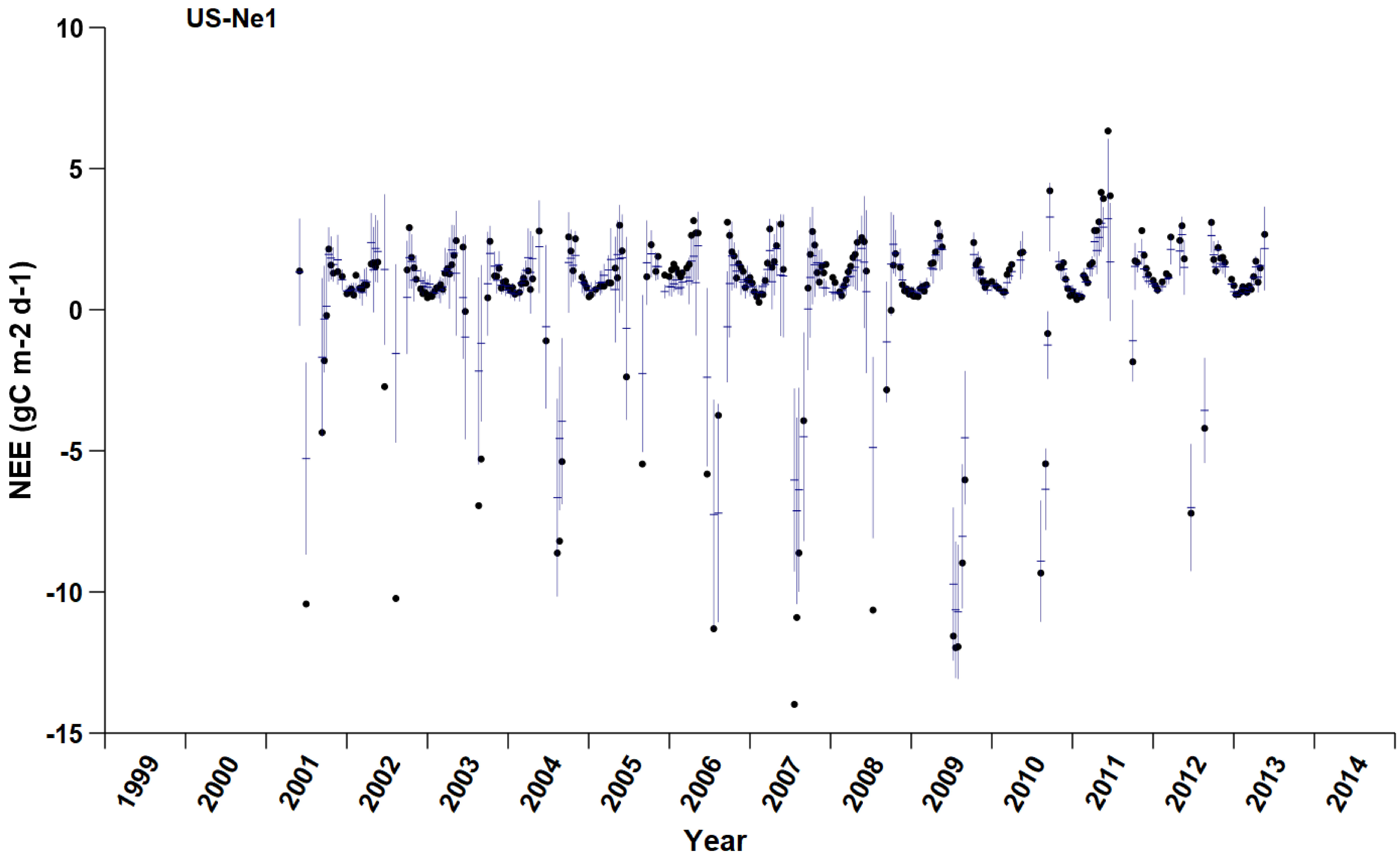

US-Ne1
NEE (gC m-2 d-1)
10
5
0
-5
-10
-15
1999
2000
2001
2002
2003
2004
2005
2006
2007
2008
2009
2010
2011
2012
2013
2014
Year

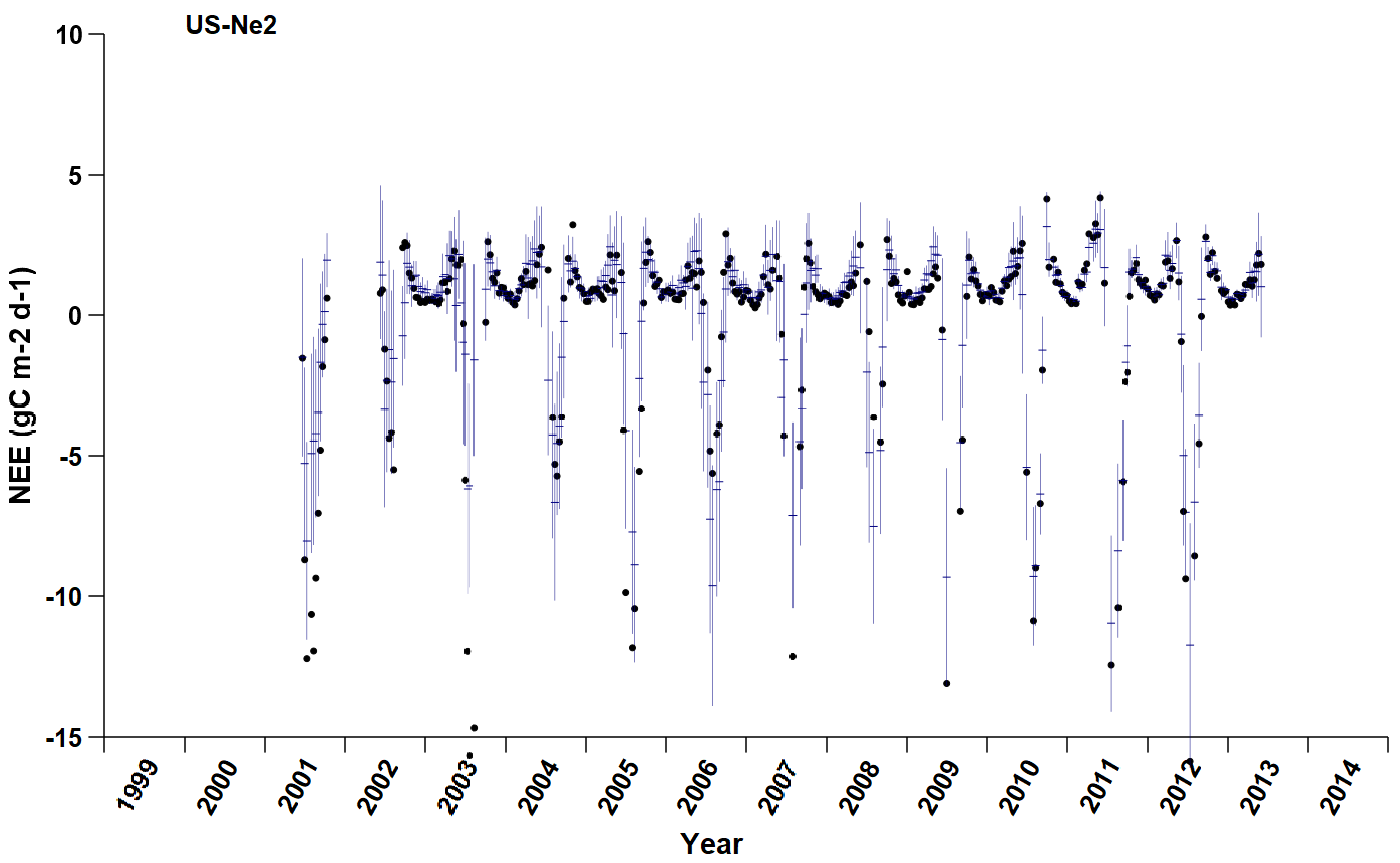
US-Ne2
NEE (gC m-2 d-1)
10
5
0
-5
-10
-15
1999
2000
2001
2002
2003
2004
2005
2006
2007
2008
2009
2010
2011
2012
2013
2014
Year

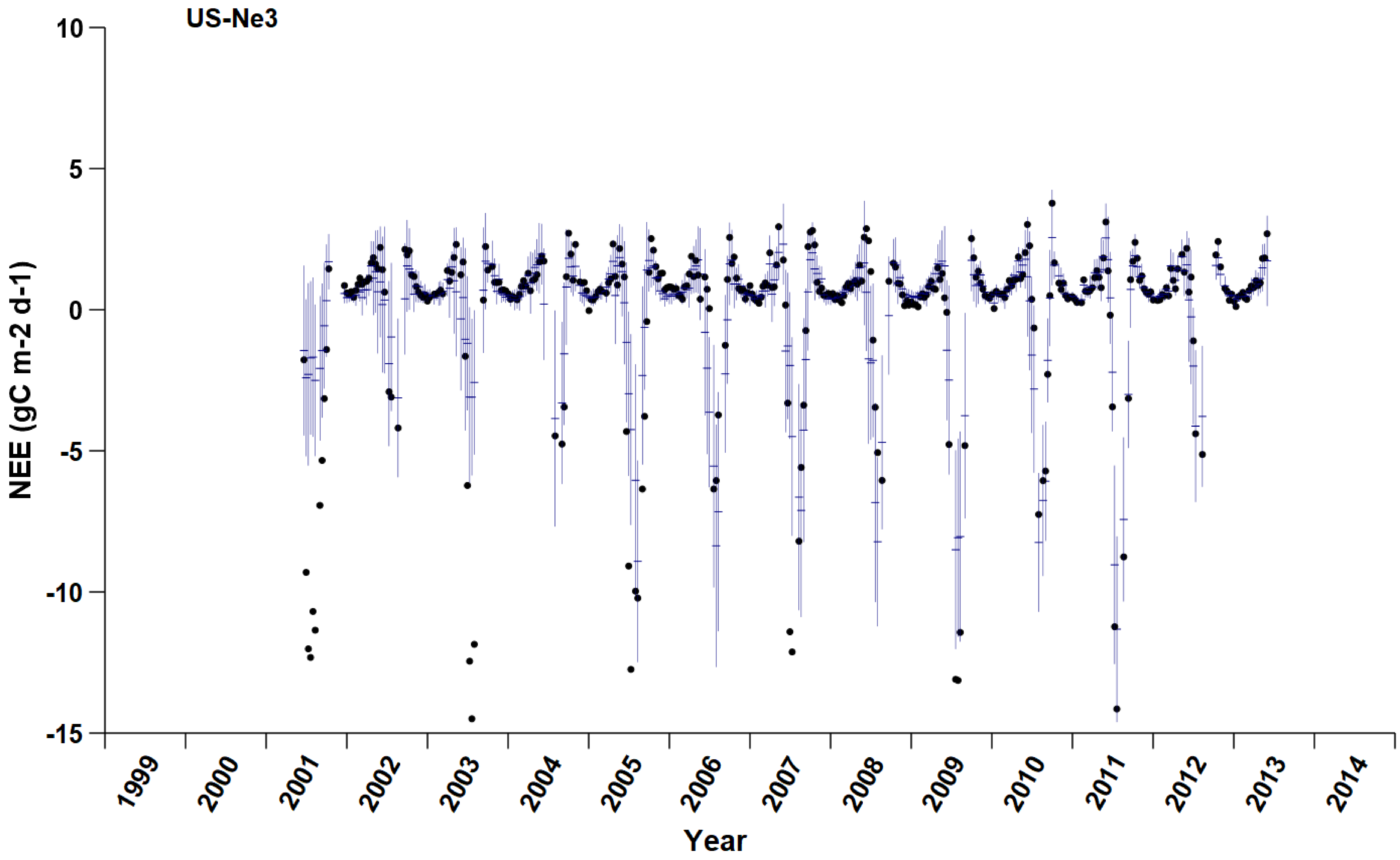

US-Ne3
NEE (gC m-2 d-1)
10
5
0
-5
-10
-15
1999
2000
2001
2002
2003
2004
2005
2006
2007
2008
2009
2010
2011
2012
2013
2014
Year

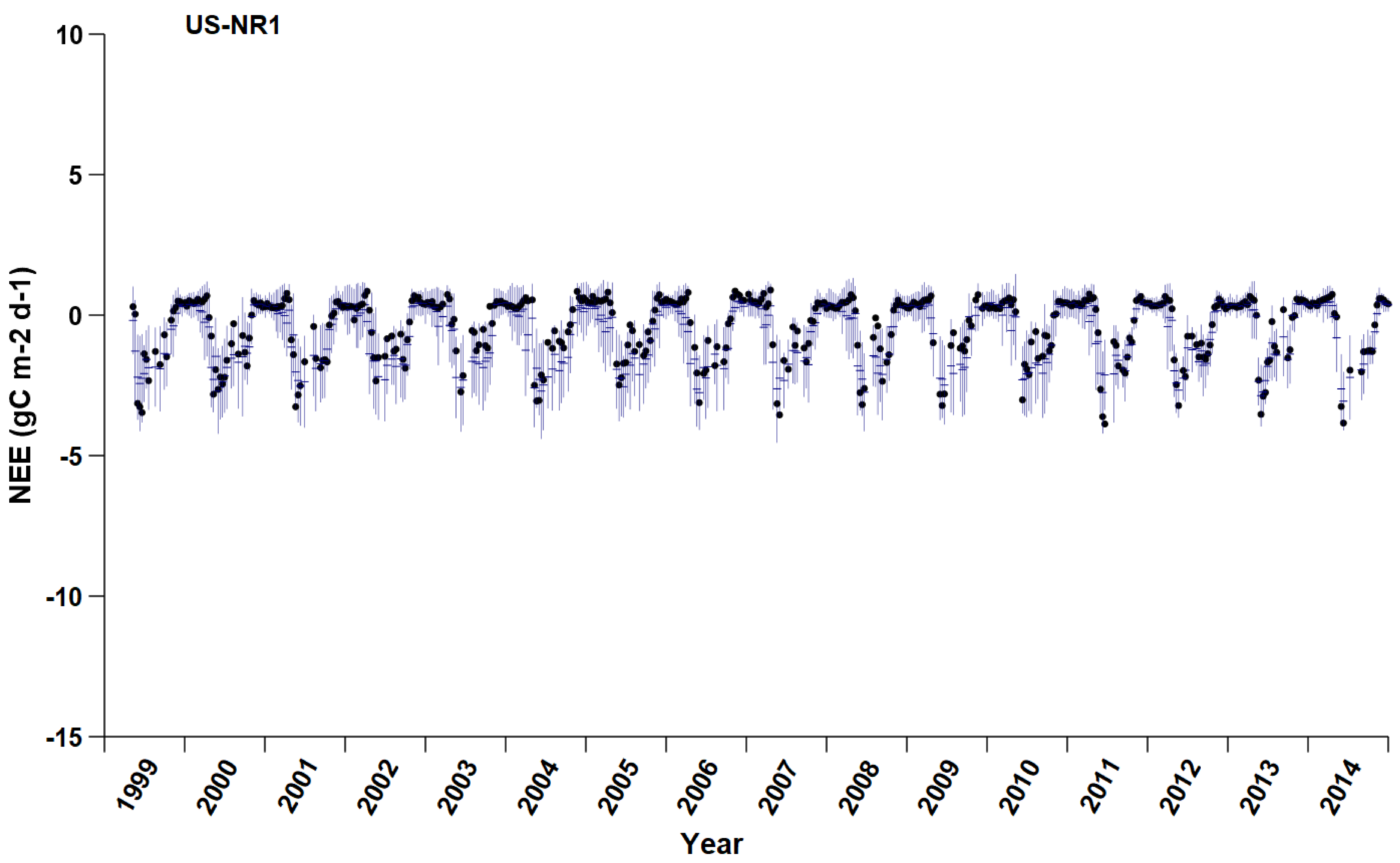

US-NR1
NEE (gC m-2 d-1)
10
5
0
-5
-10
-15
1999
2000
2001
2002
2003
2004
2005
2006
2007
2008
2009
2010
2011
2012
2013
2014
Year

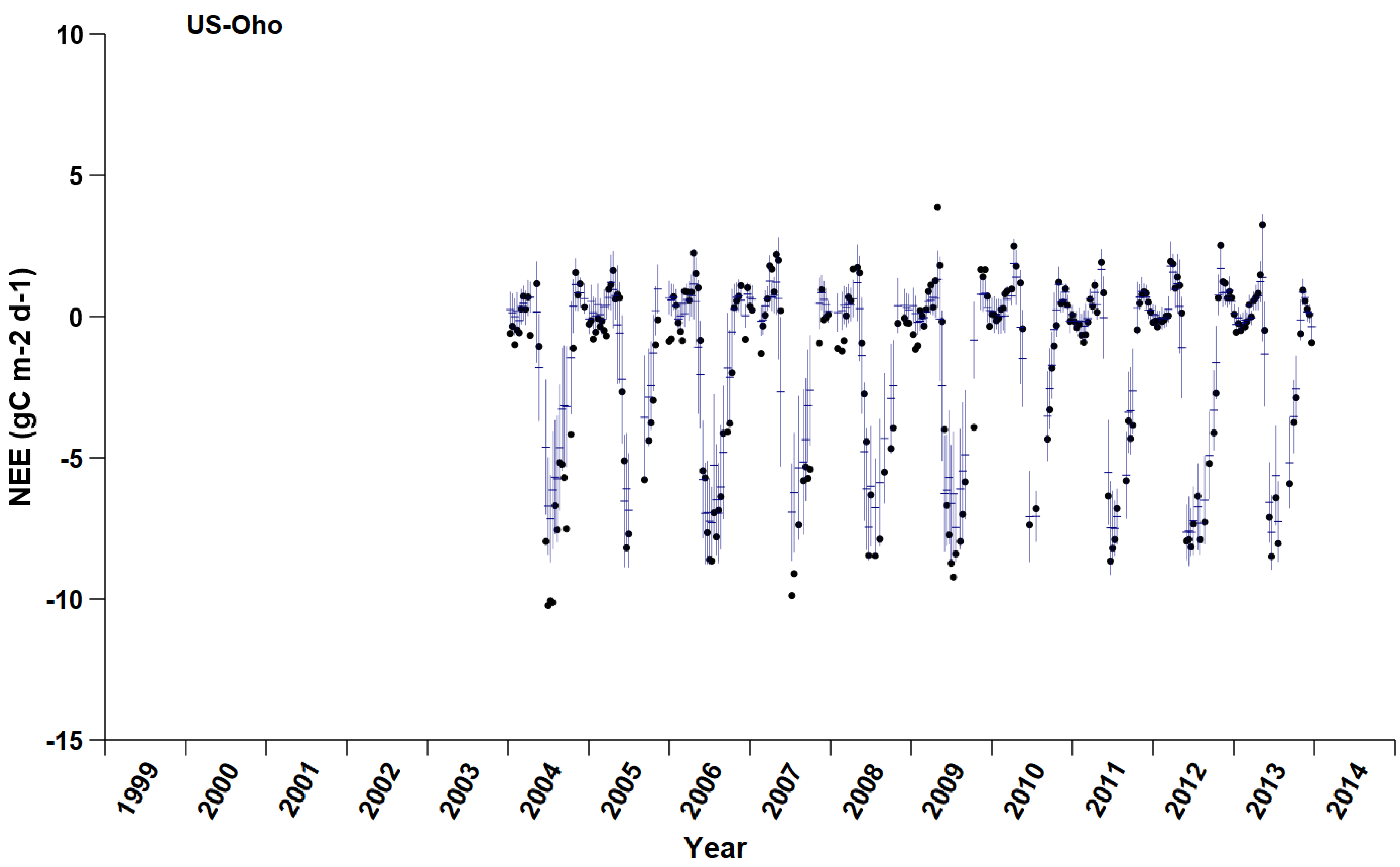

US-Oho
10
5
0
-5
-10
-15
NEE (gC m-2 d-1)
1999
2000
2001
2002
2003
2004
2005
2006
2007
2008
2009
2010
2011
2012
2013
2014
Year

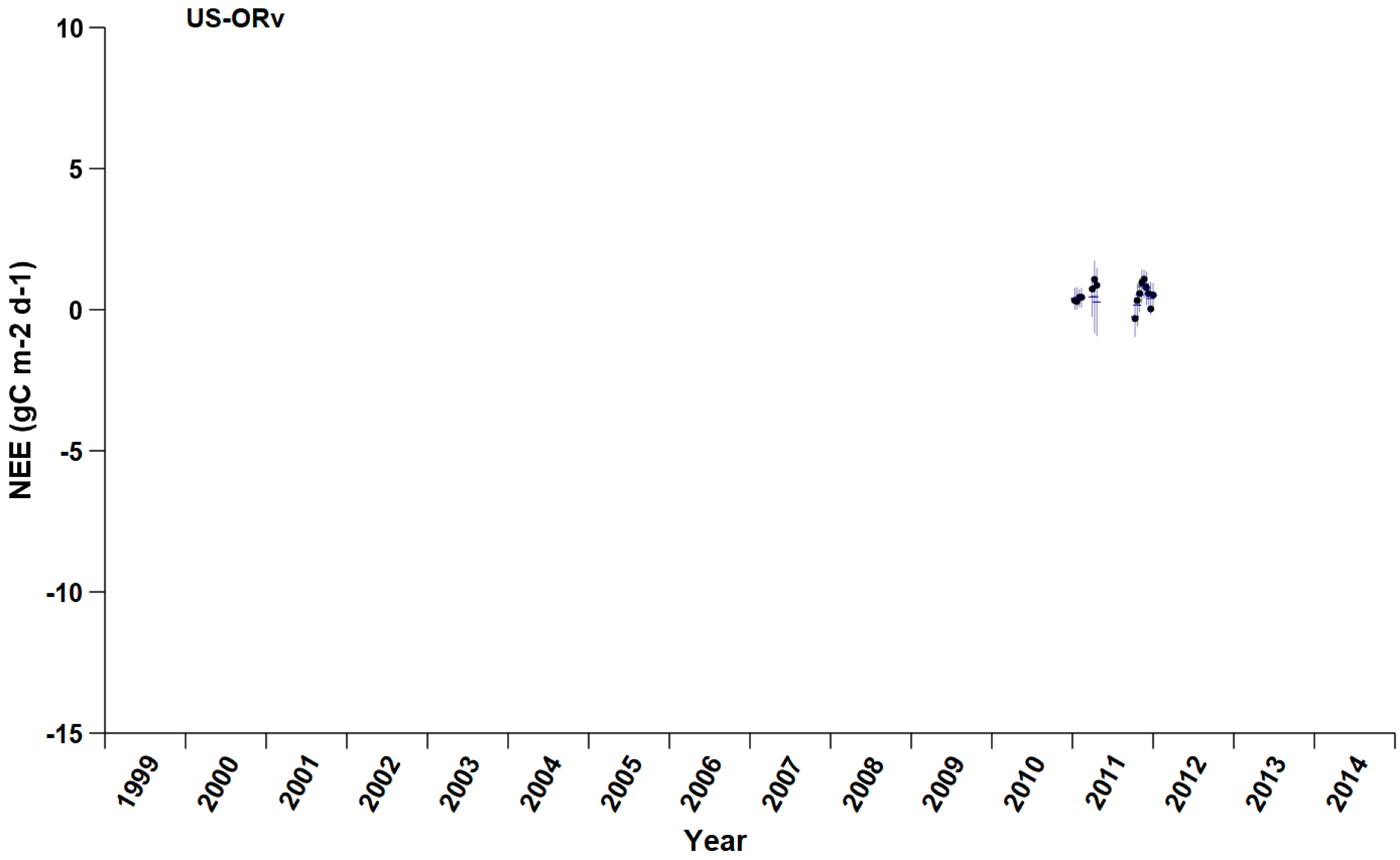

US-ORv
10
5
0
-5
-10
-15
NEE (gC m-2 d-1)
1999
2000
2001
2002
2003
2004
2005
2006
2007
2008
2009
2010
2011
2012
2013
2014
Year

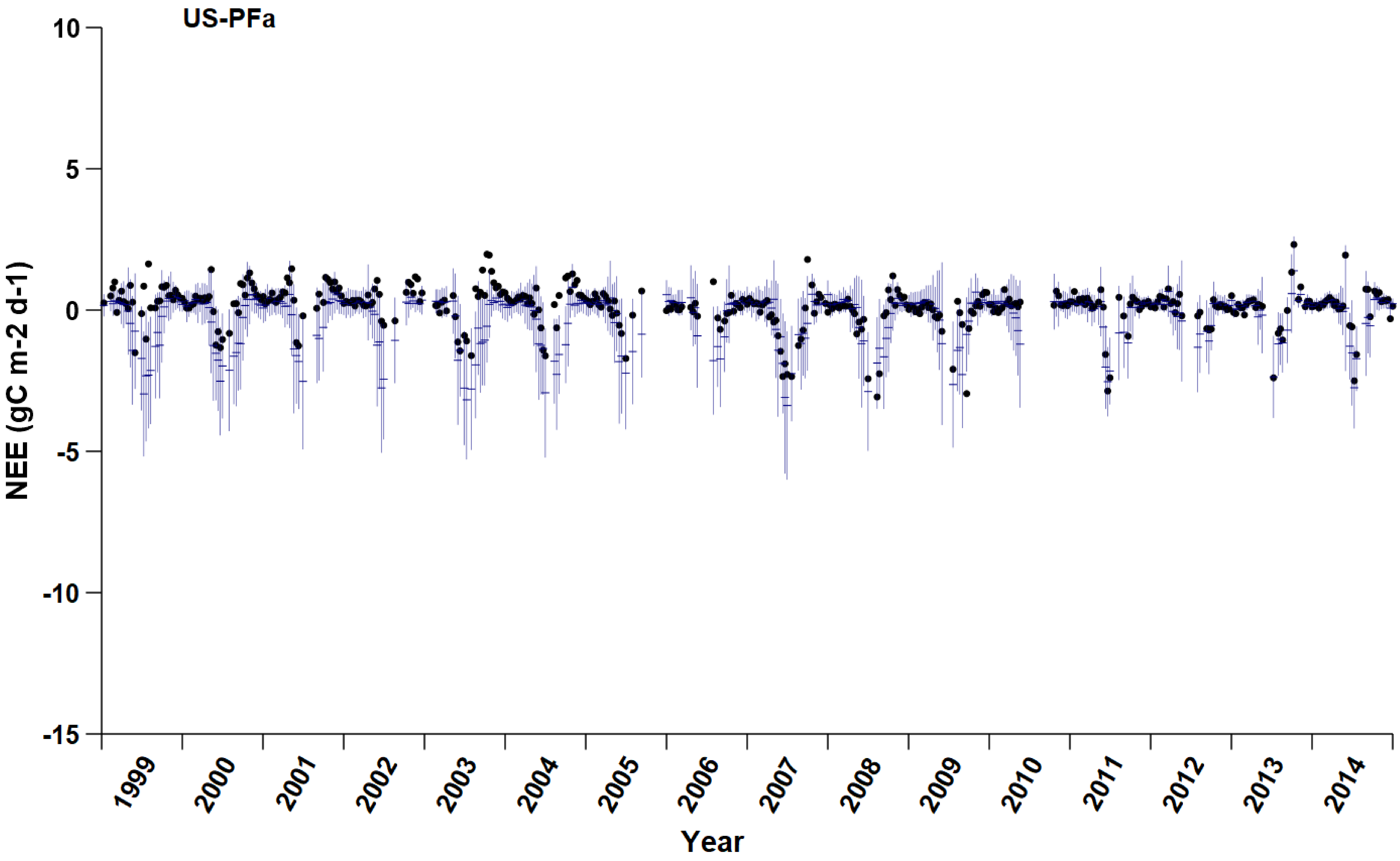

US-PFa
NEE (gC m-2 d-1)
10
5
0
-5
-10
-15
1999
2000
2001
2002
2003
2004
2005
2006
2007
2008
2009
2010
2011
2012
2013
2014
Year

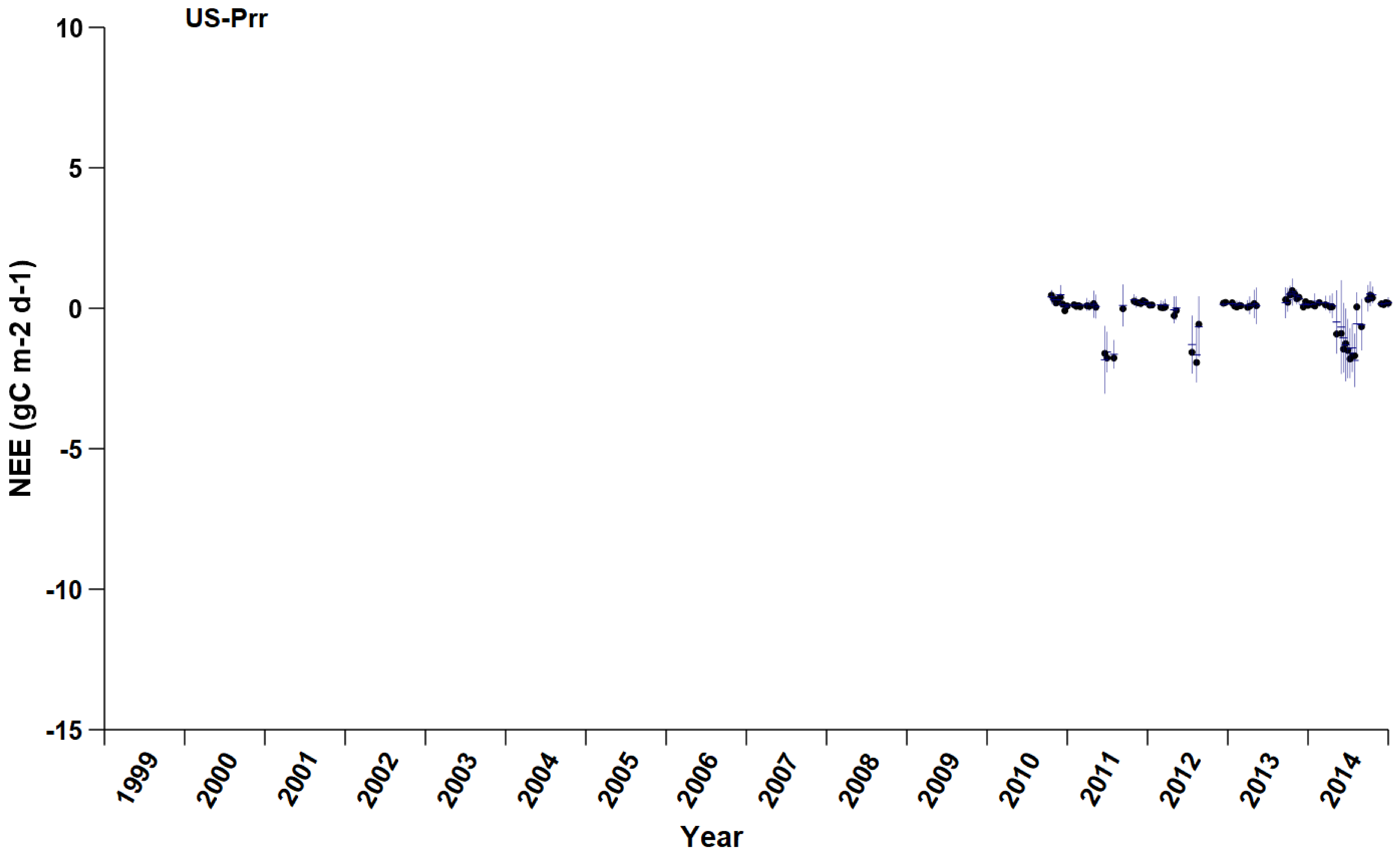

US-Prr
10
5
0
-5
-10
-15
NEE (gC m-2 d-1)
1999
2000
2001
2002
2003
2004
2005
2006
2007
2008
2009
2010
2011
2012
2013
2014
Year

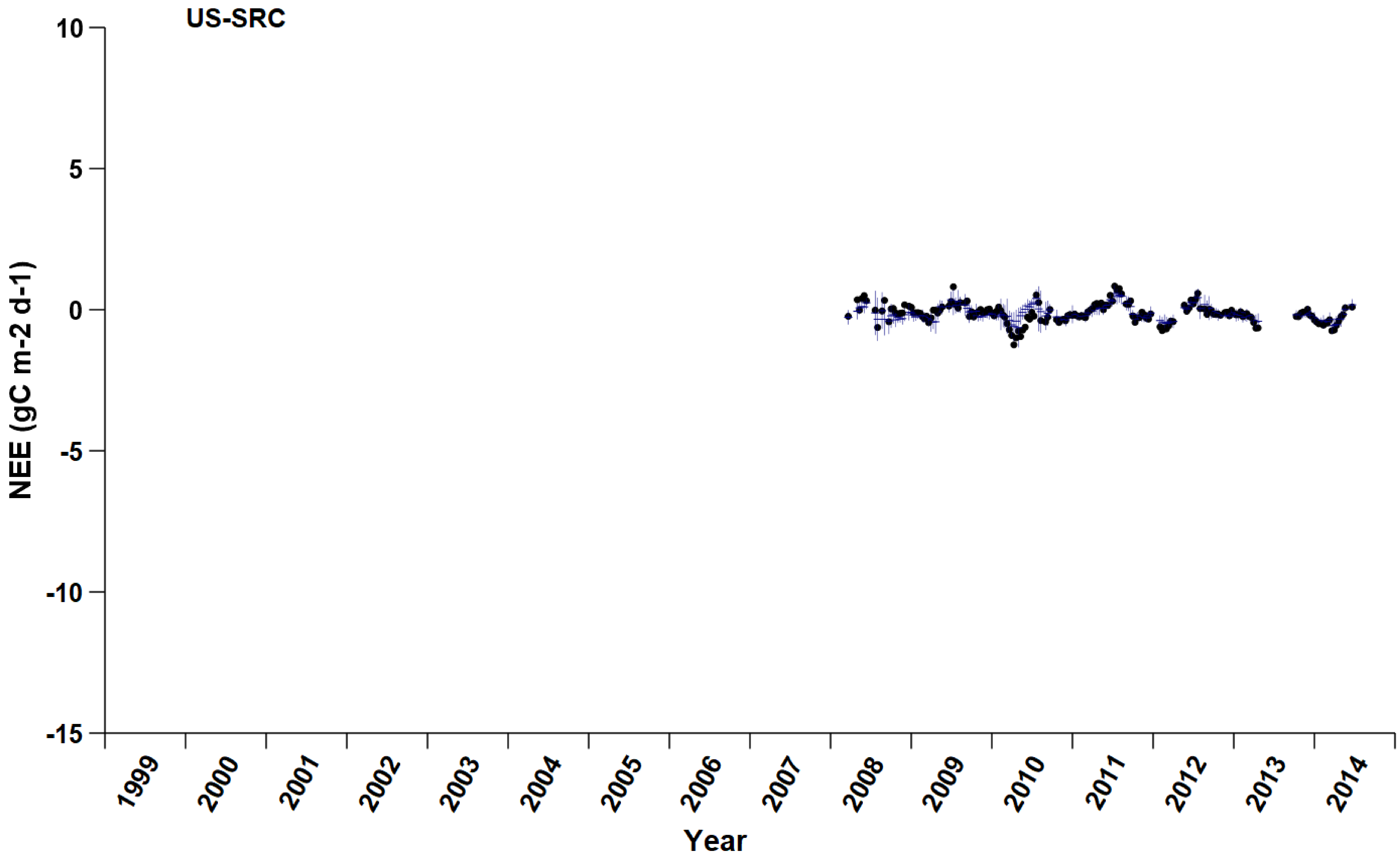
US-SRC
10
5
0
-5
-10
-15
NEE (gC m-2 d-1)
1999
2000
2001
2002
2003
2004
2005
2006
2007
2008
2009
2010
2011
2012
2013
2014
Year

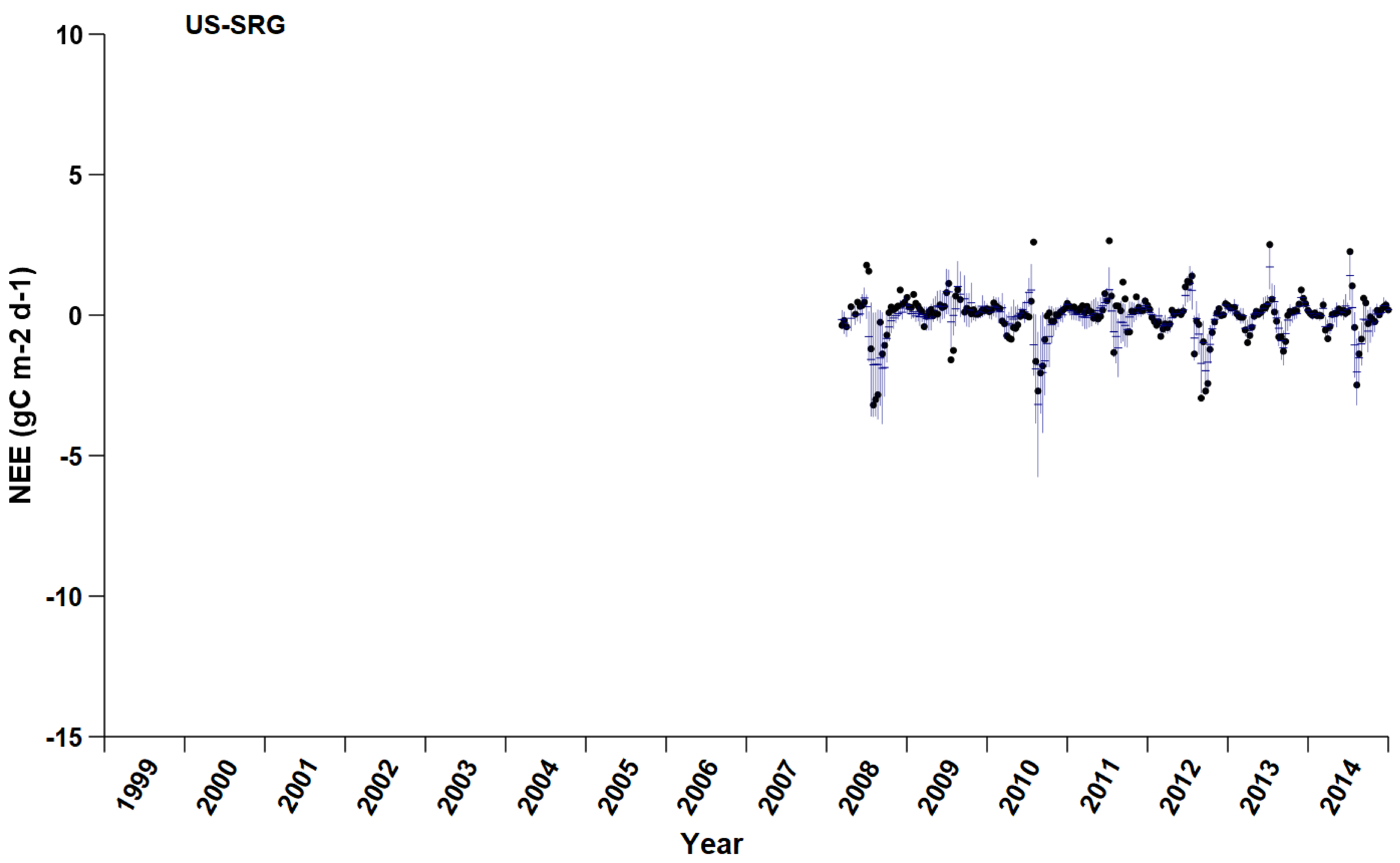

US-SRG
10
5
0
-5
-10
-15
NEE (gC m-2 d-1)
1999
2000
2001
2002
2003
2004
2005
2006
2007
2008
2009
2010
2011
2012
2013
2014
Year

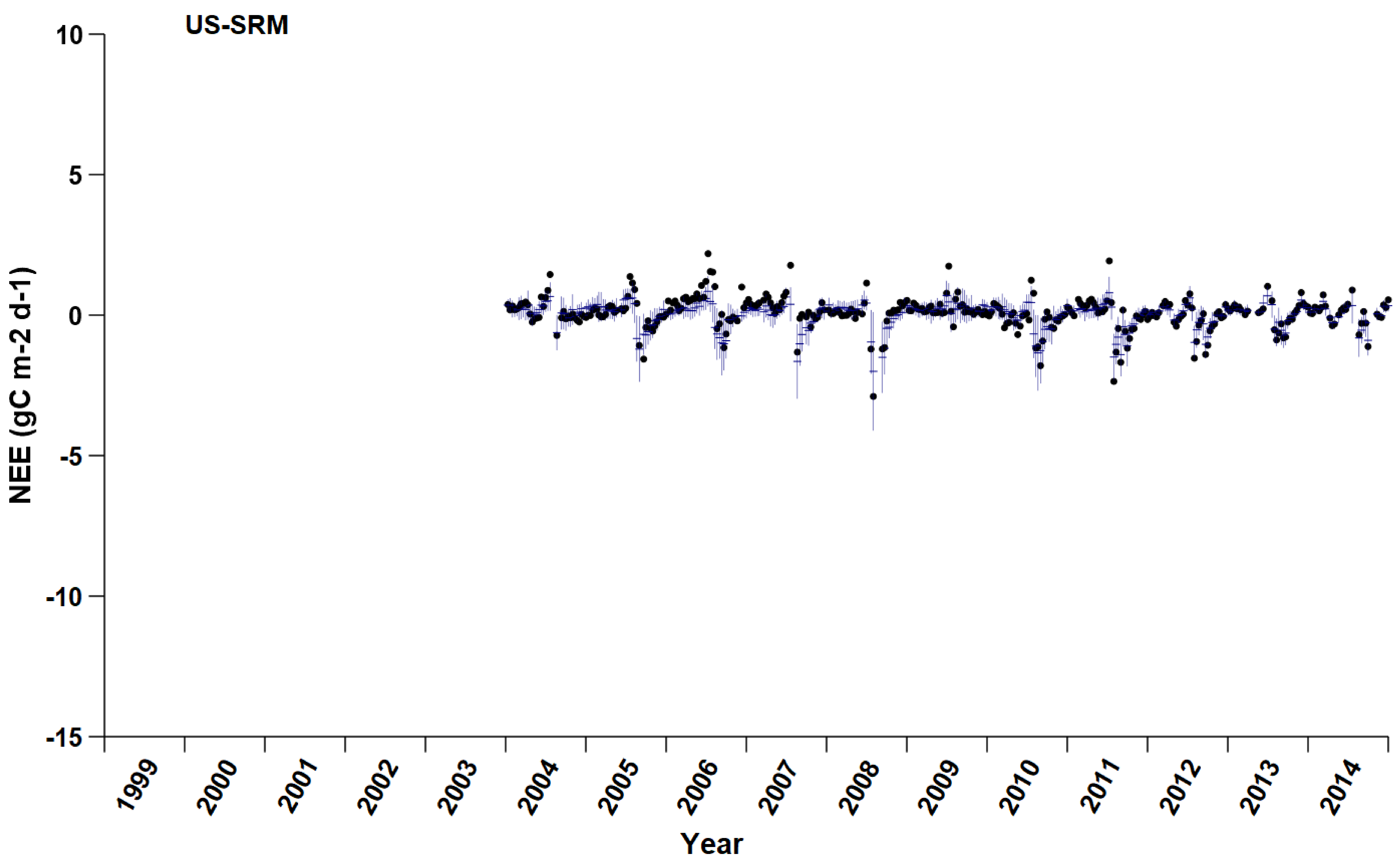
US-SRM
10
5
0
-5
-10
-15
NEE (gC m-2 d-1)
1999
2000
2001
2002
2003
2004
2005
2006
2007
2008
2009
2010
2011
2012
2013
2014
Year

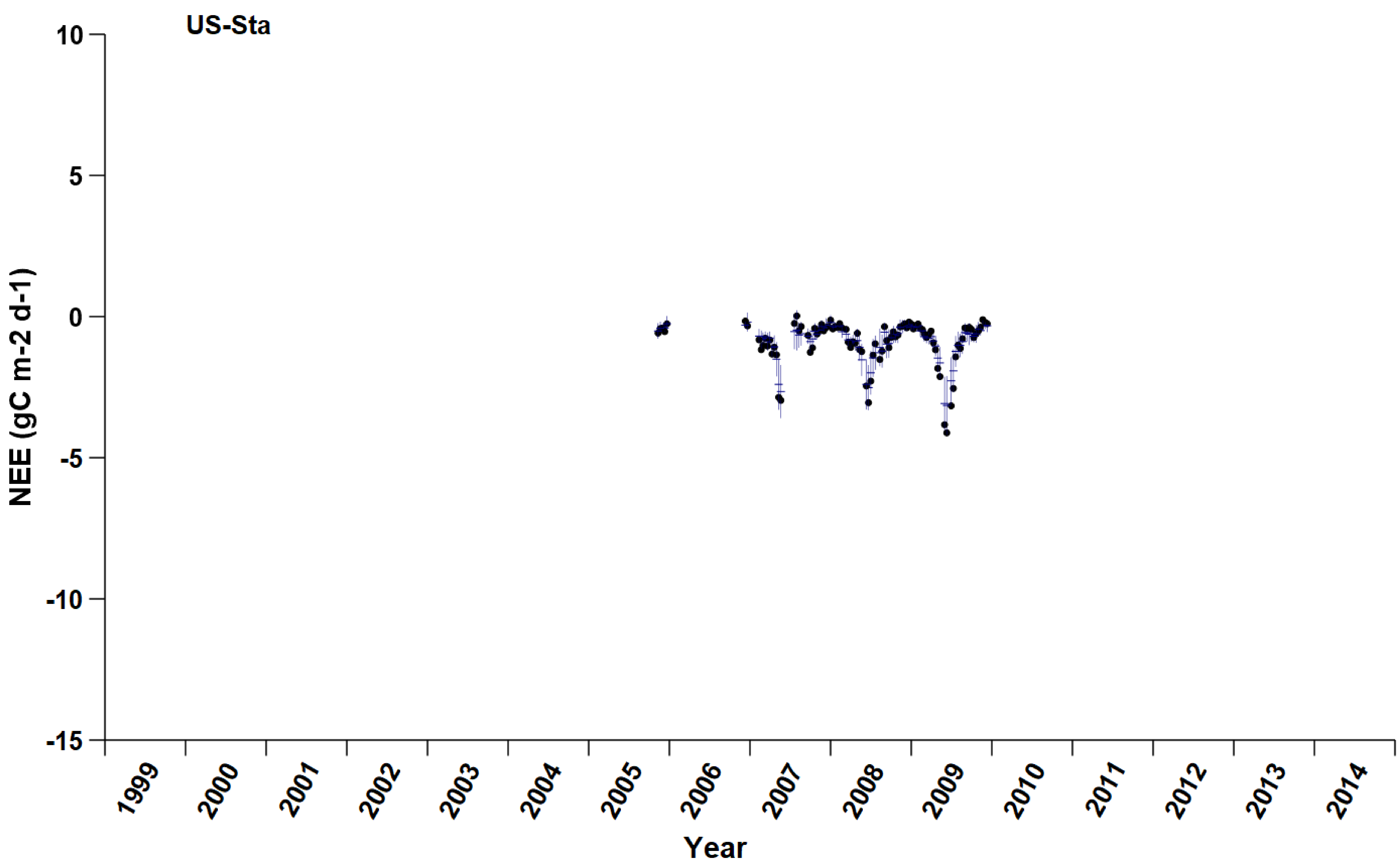

US-Sta
10
5
0
-5
-10
-15
NEE (gC m-2 d-1)
1999
2000
2001
2002
2003
2004
2005
2006
2007
2008
2009
2010
2011
2012
2013
2014
Year

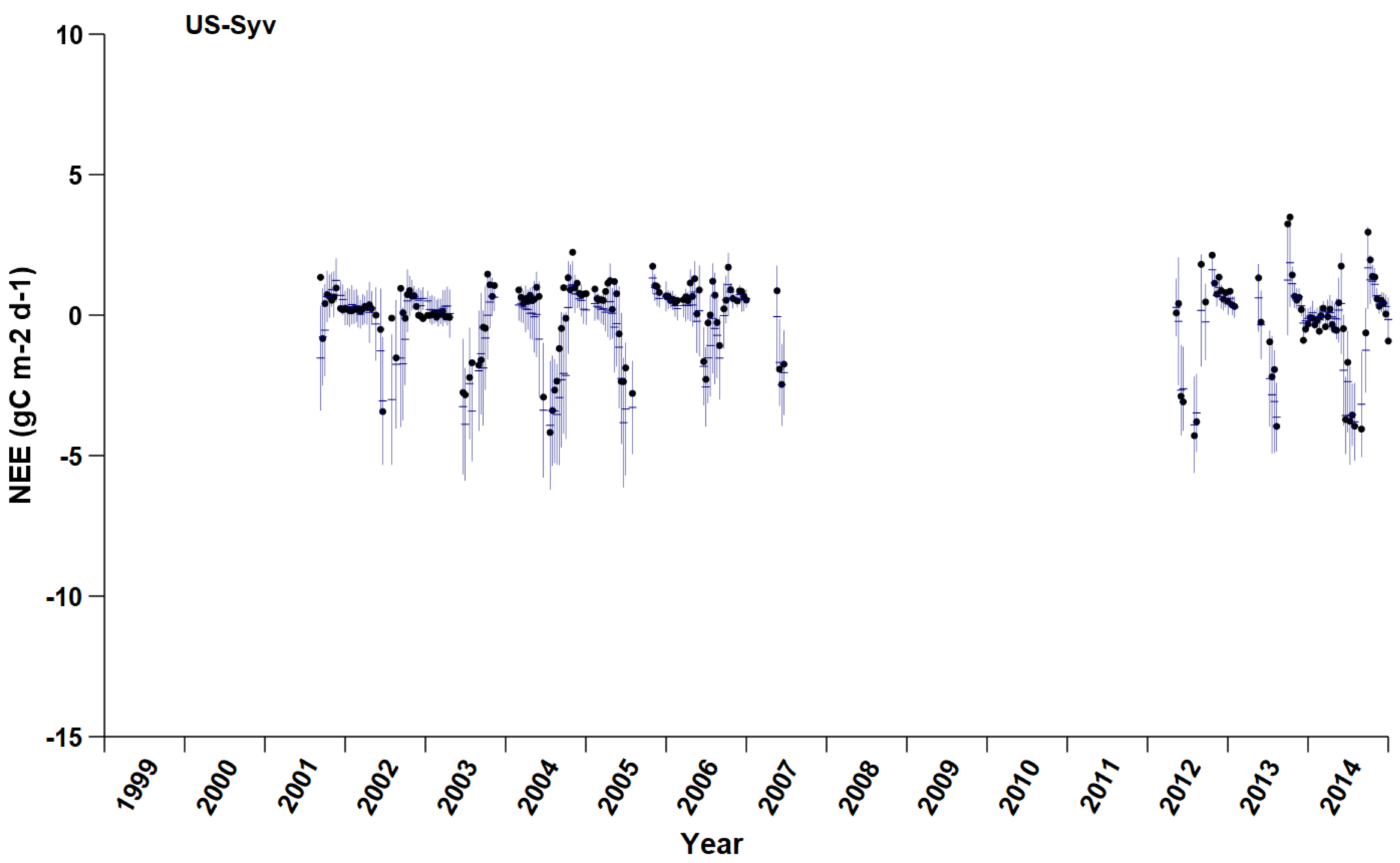

US-Syv
NEE (gC m-2 d-1)
10
5
0
-5
-10
-15
1999
2000
2001
2002
2003
2004
2005
2006
2007
2008
2009
2010
2011
2012
2013
2014
Year

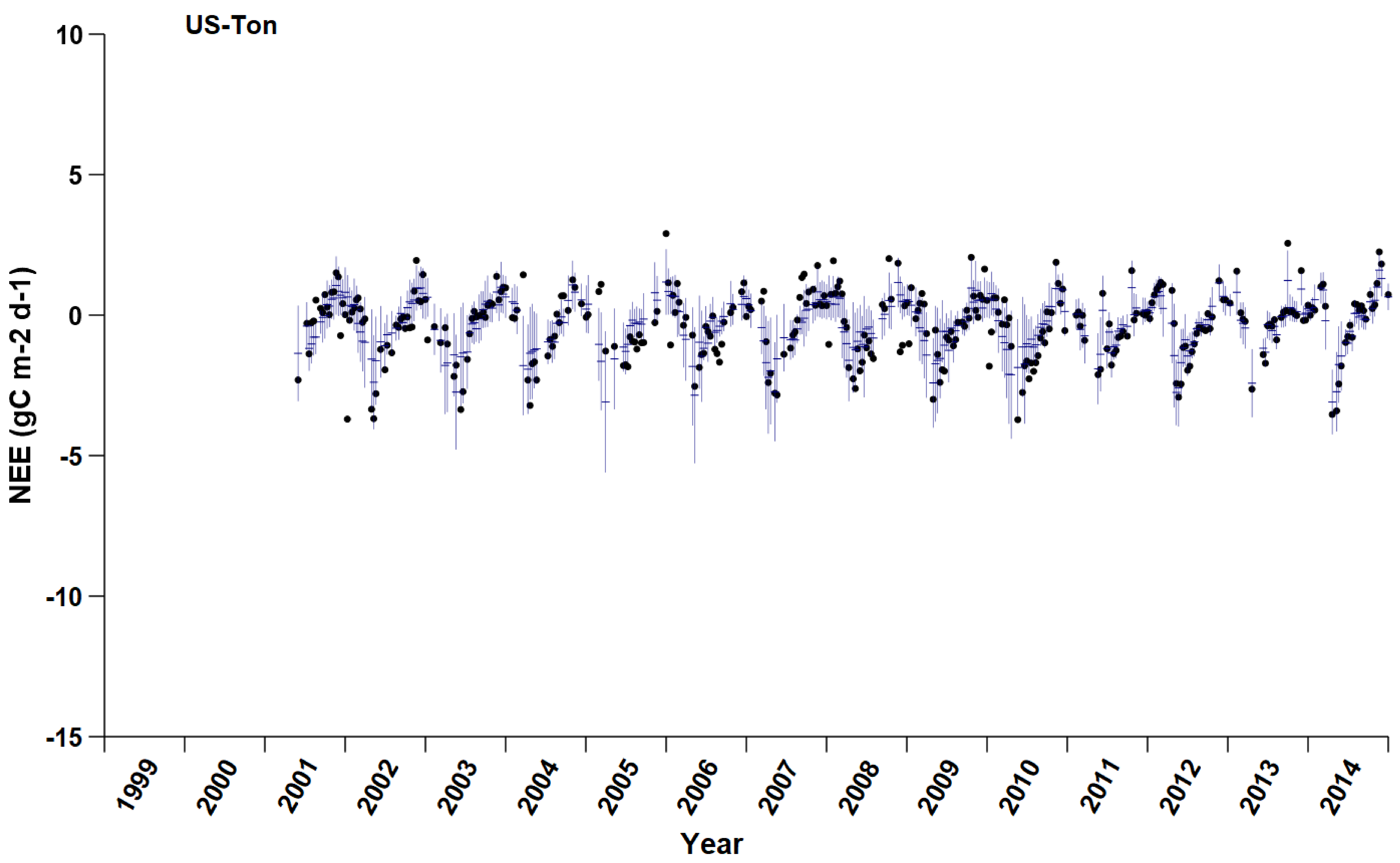

US-Ton
10
5
0
-5
-10
-15
NEE (gC m-2 d-1)
1999
2000
2001
2002
2003
2004
2005
2006
2007
2008
2009
2010
2011
2012
2013
2014
Year

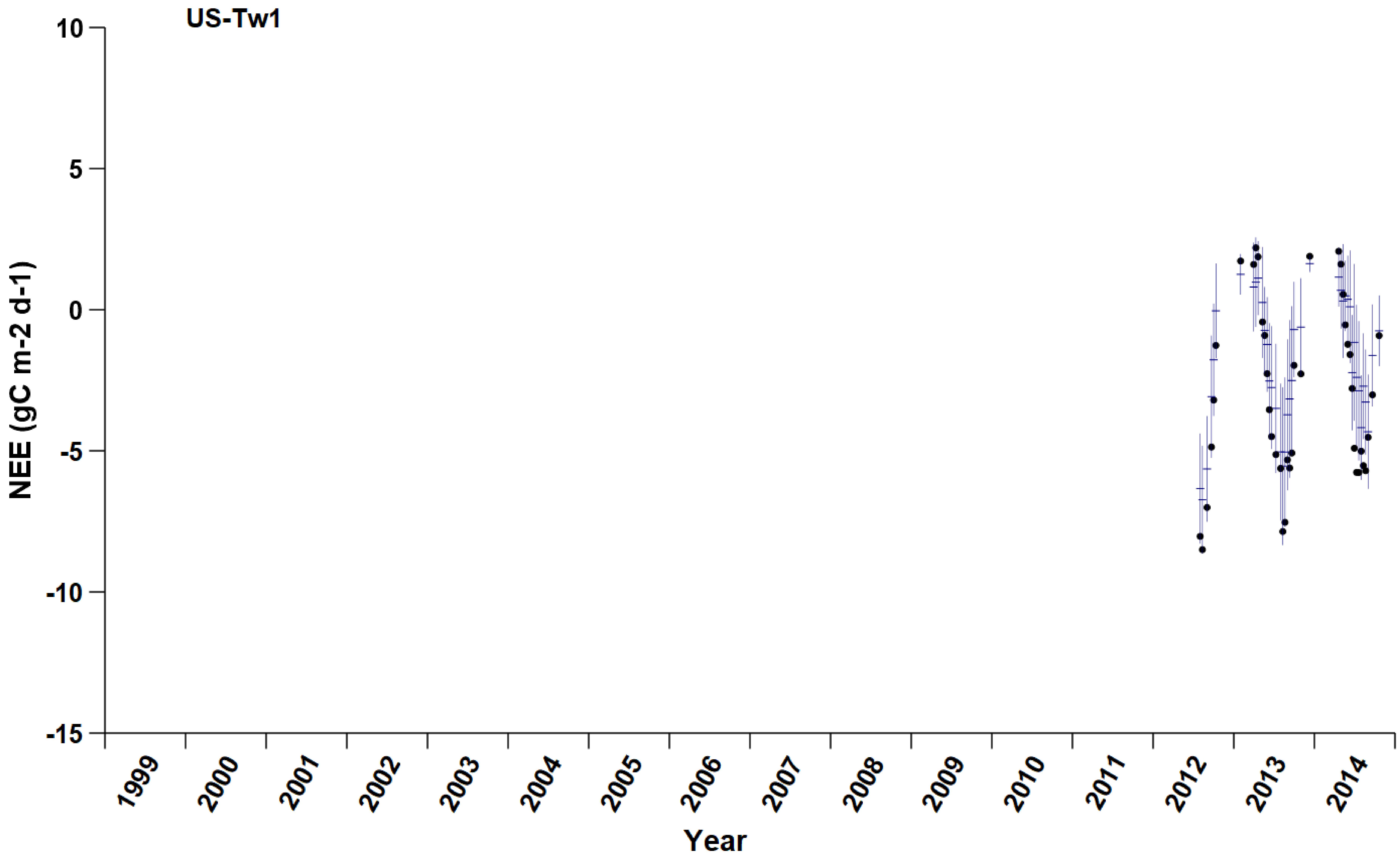

US-Tw1
NEE (gC m-2 d-1)
10
5
0
-5
-10
-15
1999
2000
2001
2002
2003
2004
2005
2006
2007
2008
2009
2010
2011
2012
2013
2014
Year

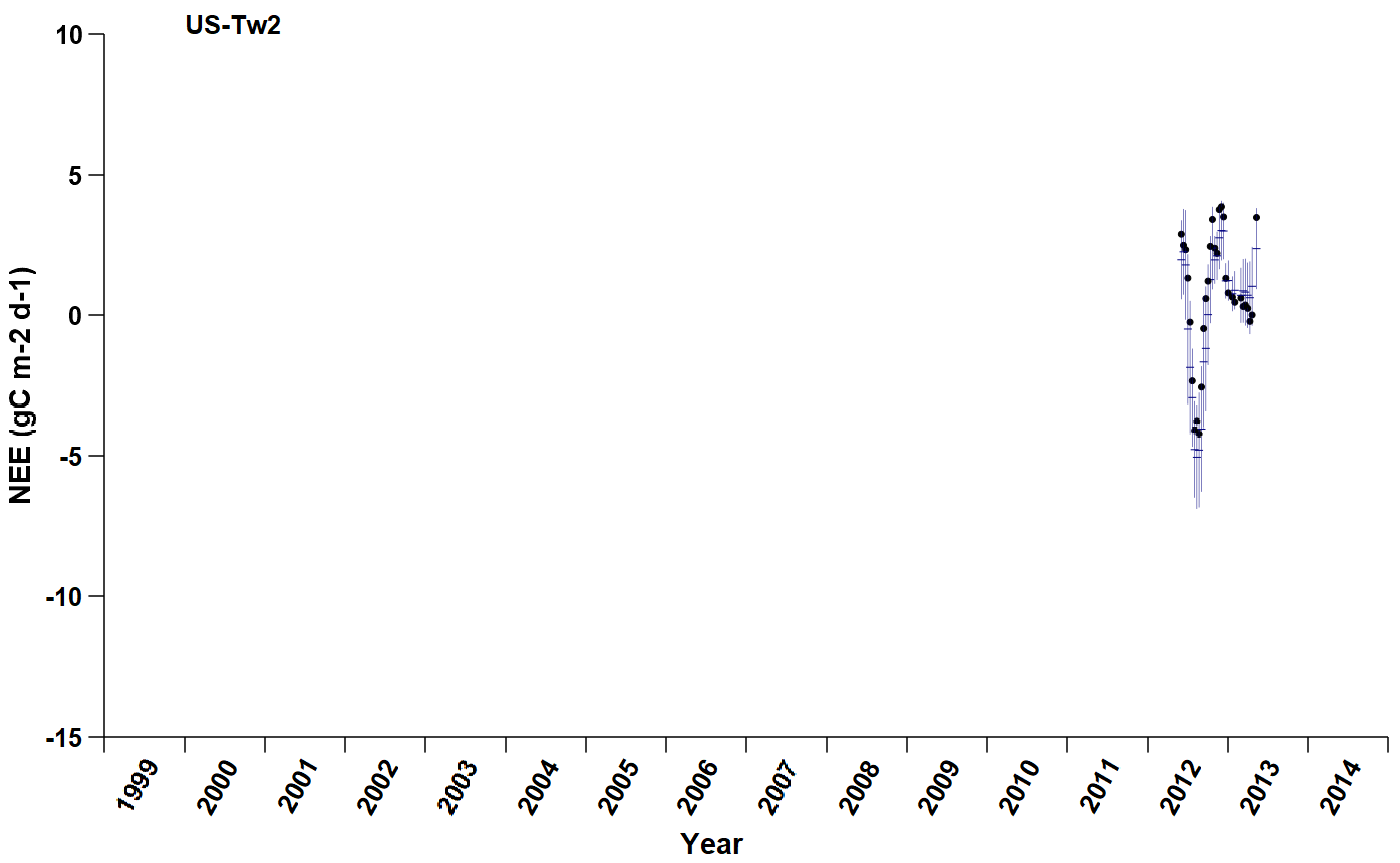
US-Tw2
10
5
0
-5
-10
-15
NEE (gC m-2 d-1)
1999
2000
2001
2002
2003
2004
2005
2006
2007
2008
2009
2010
2011
2012
2013
2014
Year

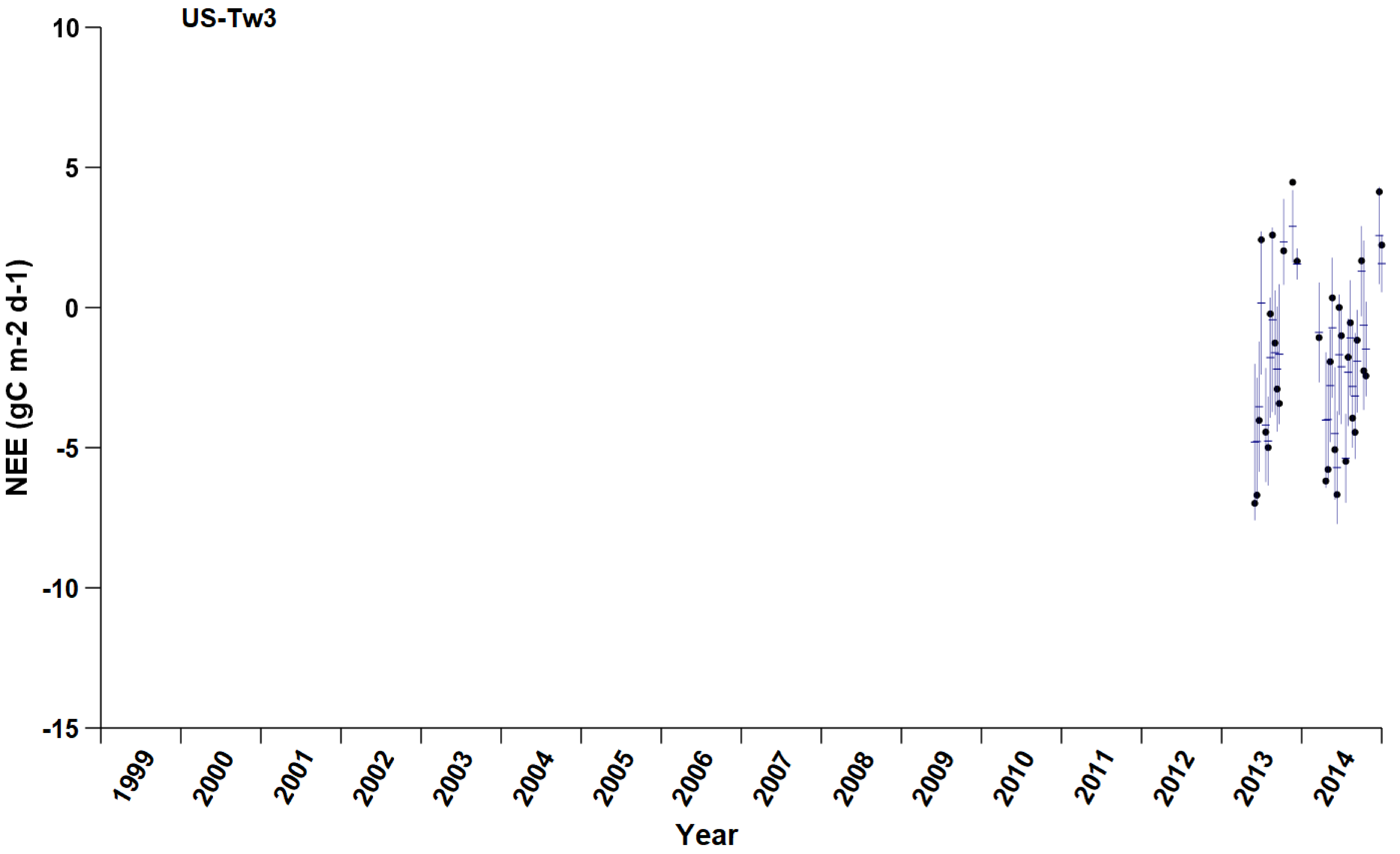

US-Tw3
NEE (gC m-2 d-1)
10
5
0
-5
-10
-15
1999
2000
2001
2002
2003
2004
2005
2006
2007
2008
2009
2010
2011
2012
2013
2014
Year

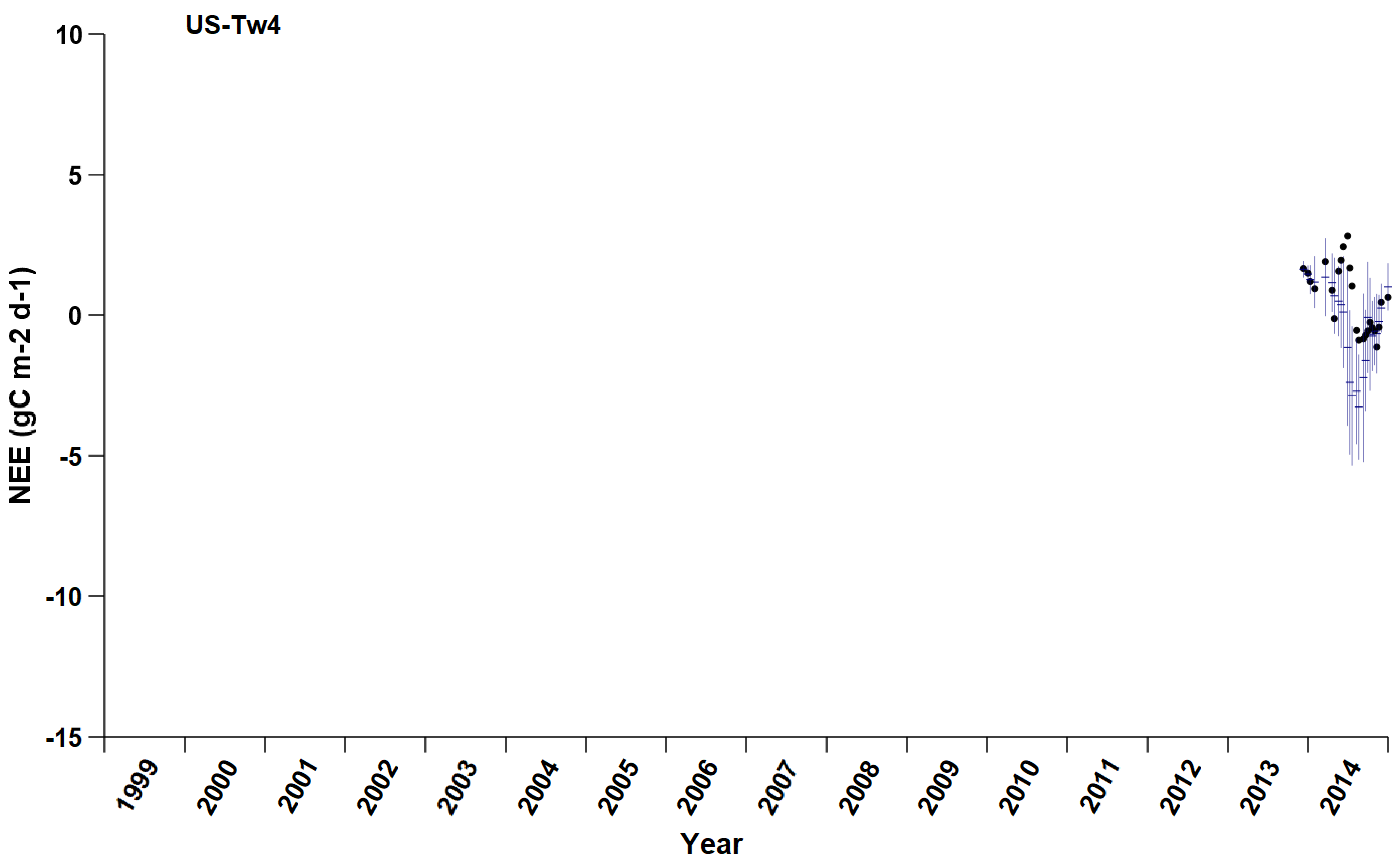
US-Tw4
NEE (gC m-2 d-1)
10
5
0
-5
-10
-15
1999
2000
2001
2002
2003
2004
2005
2006
2007
2008
2009
2010
2011
2012
2013
2014
Year

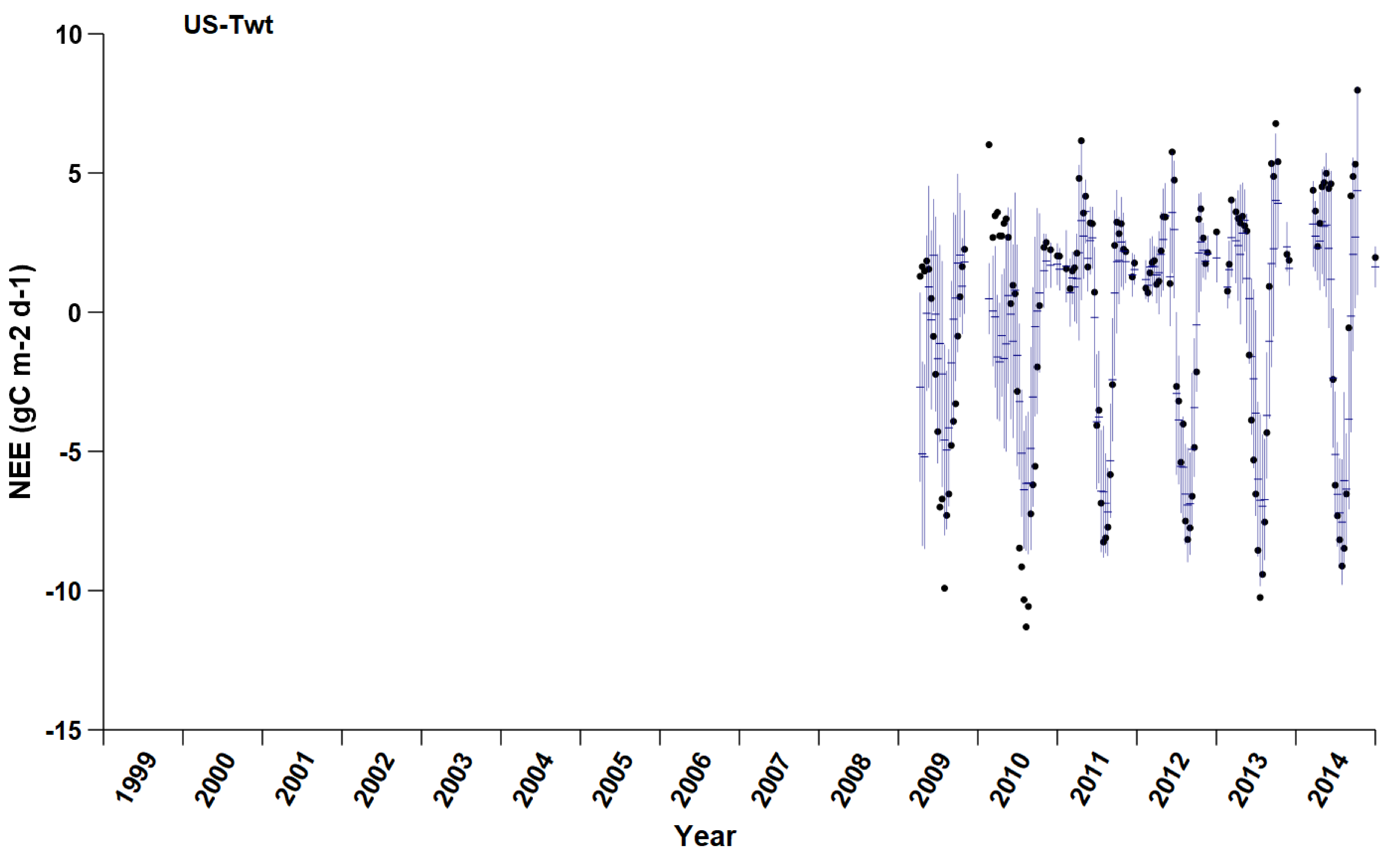

US-Twt
NEE (gC m-2 d-1)
10
5
0
-5
-10
-15
1999
2000
2001
2002
2003
2004
2005
2006
2007
2008
2009
2010
2011
2012
2013
2014
Year

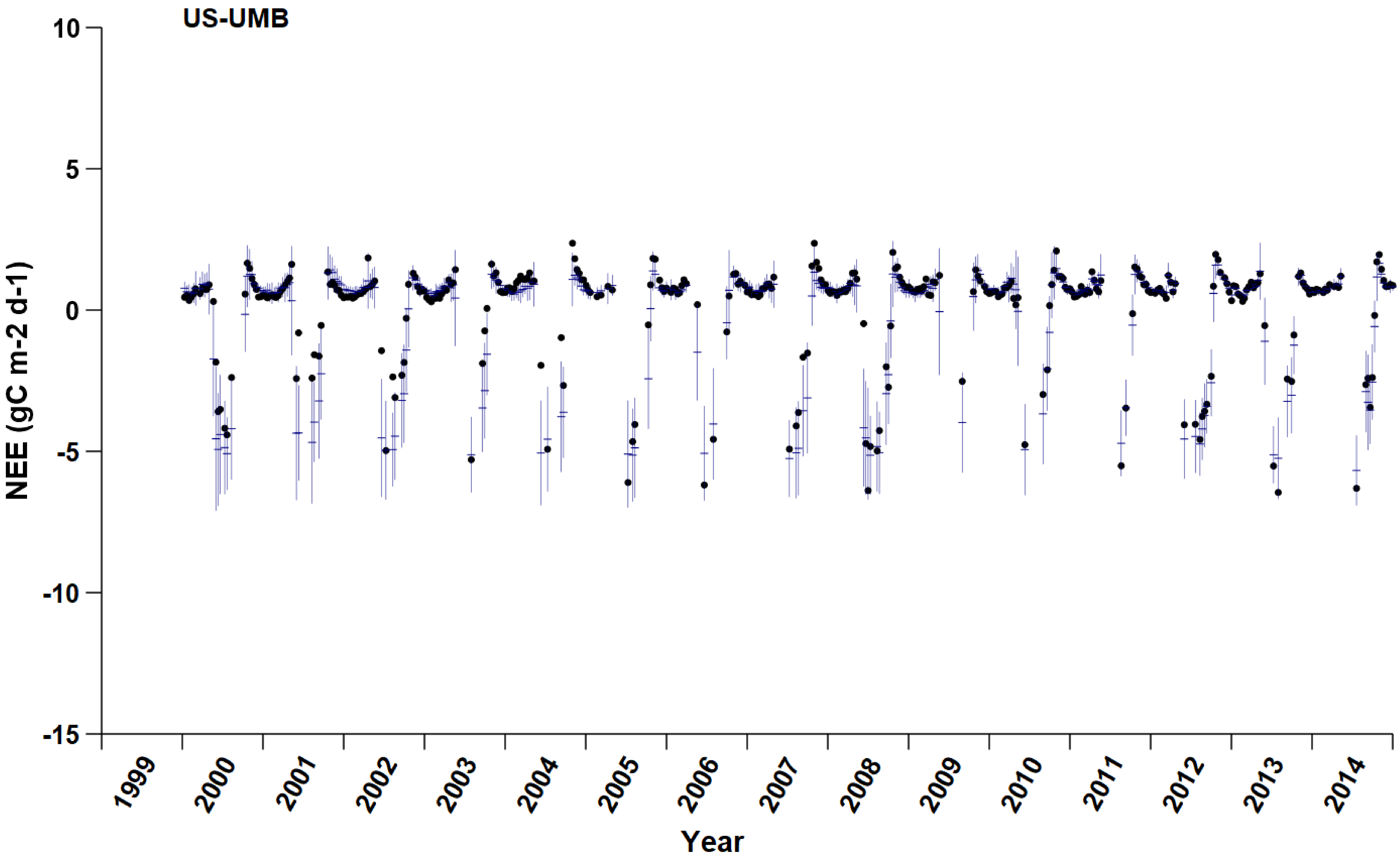
US-UMB
NEE (gC m-2 d-1)
10
5
0
-5
-10
-15
1999
2000
2001
2002
2003
2004
2005
2006
2007
2008
2009
2010
2011
2012
2013
2014
Year

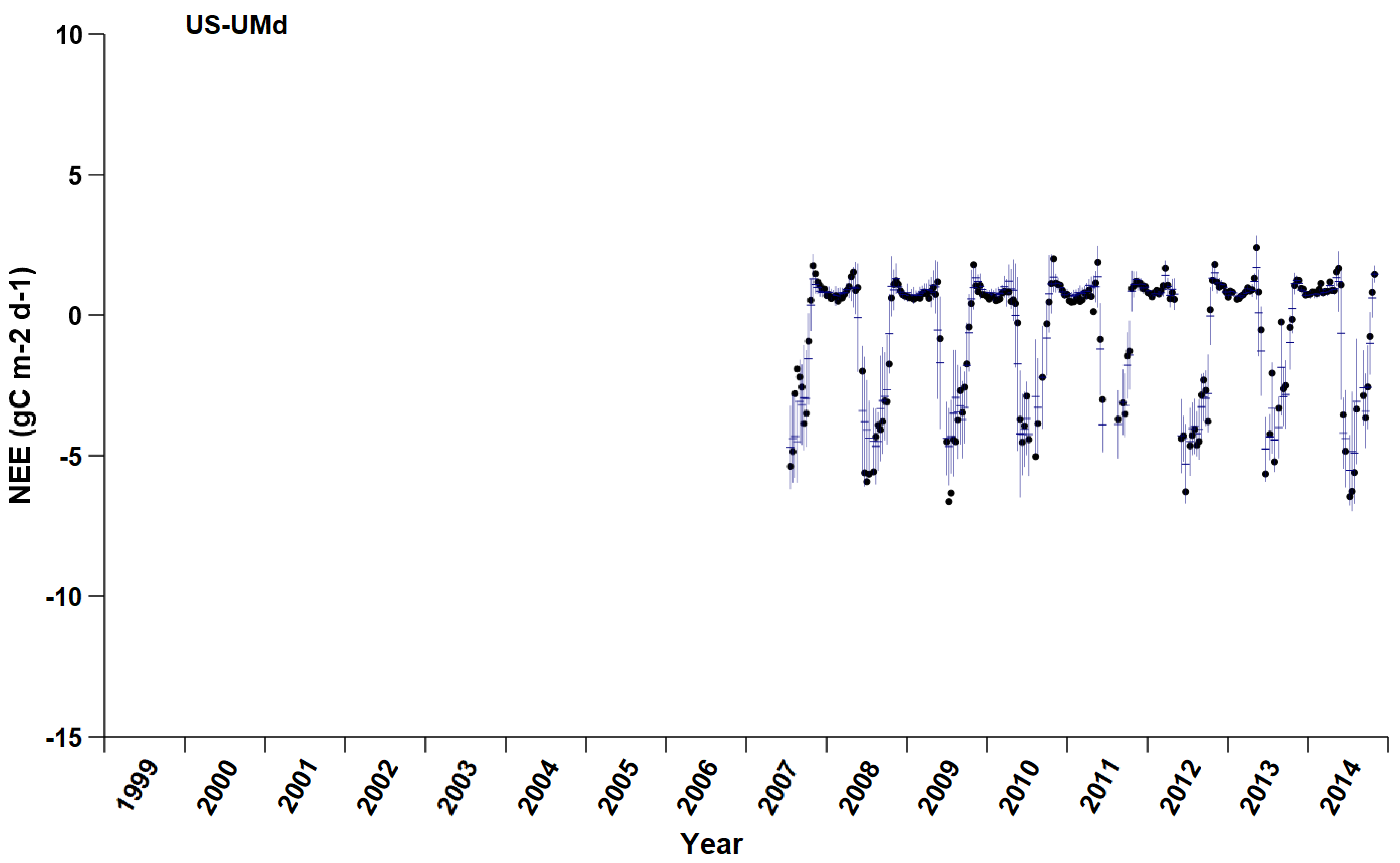

US-UMd
NEE (gC m-2 d-1)
10
5
0
-5
-10
-15
1999
2000
2001
2002
2003
2004
2005
2006
2007
2008
2009
2010
2011
2012
2013
2014
Year

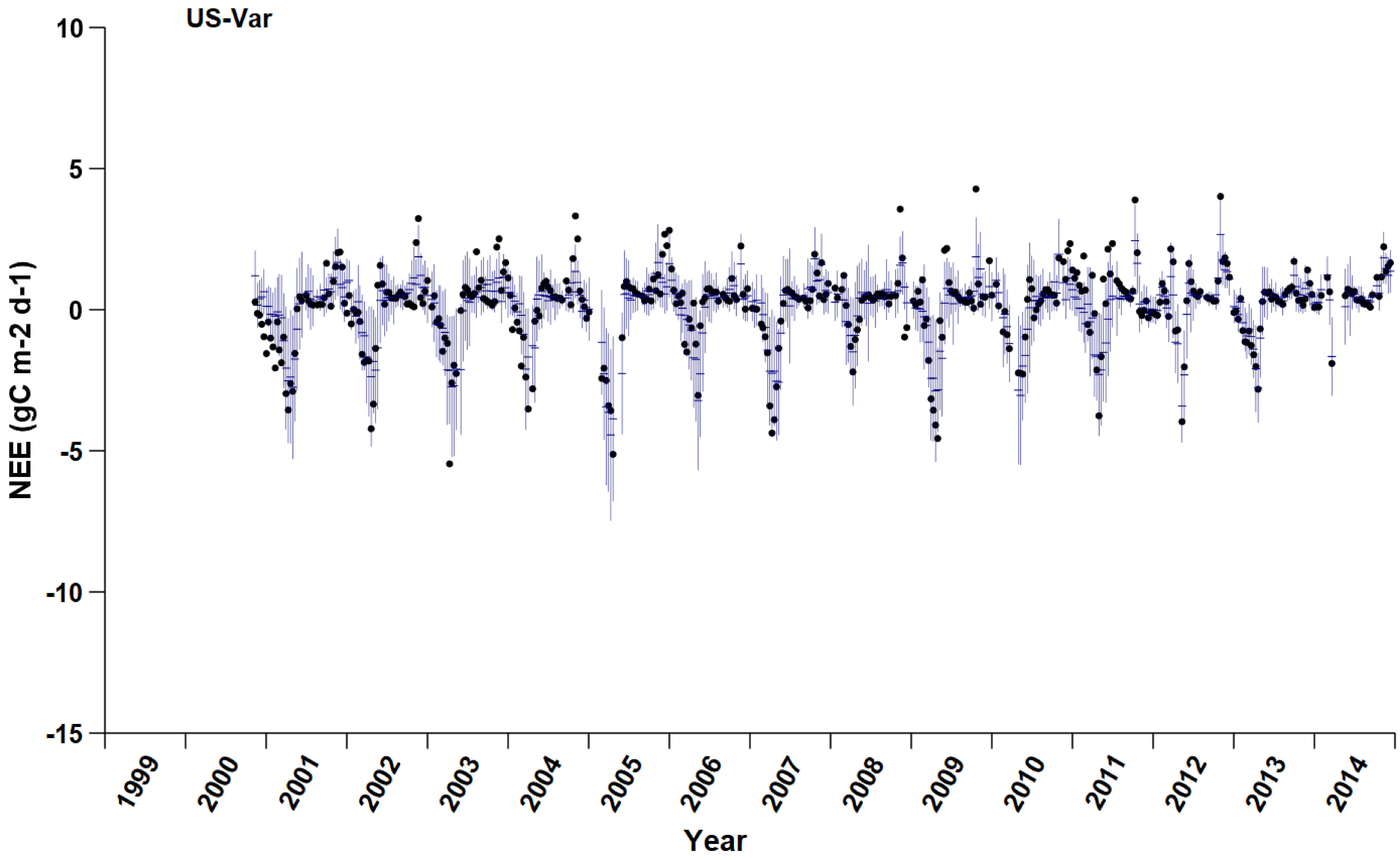
US-Var
NEE (gC m-2 d-1)
10
5
0
-5
-10
-15
1999
2000
2001
2002
2003
2004
2005
2006
2007
2008
2009
2010
2011
2012
2013
2014
Year

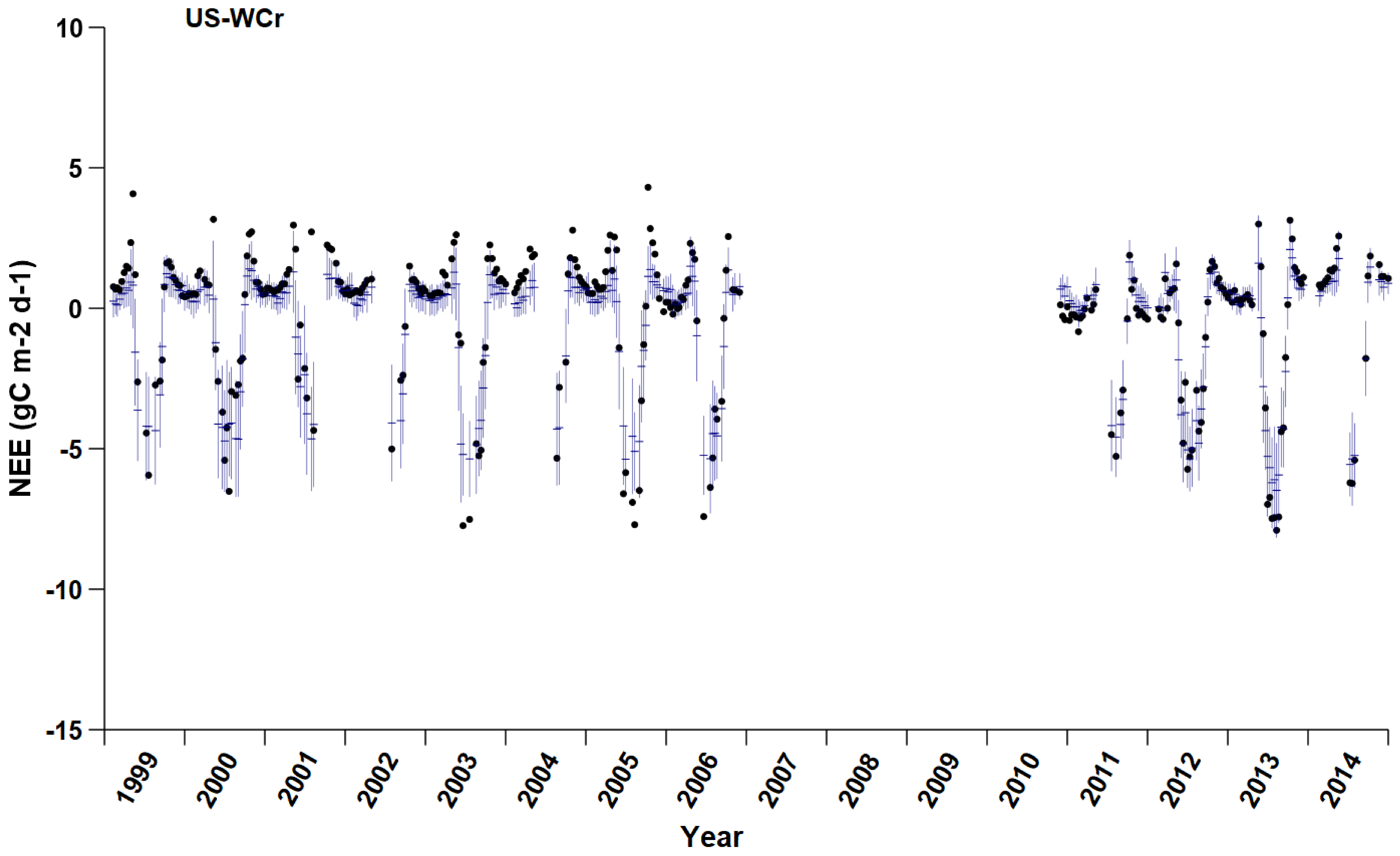
US-WCr
NEE (gC m-2 d-1)
10
5
0
-5
-10
-15
1999
2000
2001
2002
2003
2004
2005
2006
2007
2008
2009
2010
2011
2012
2013
2014
Year

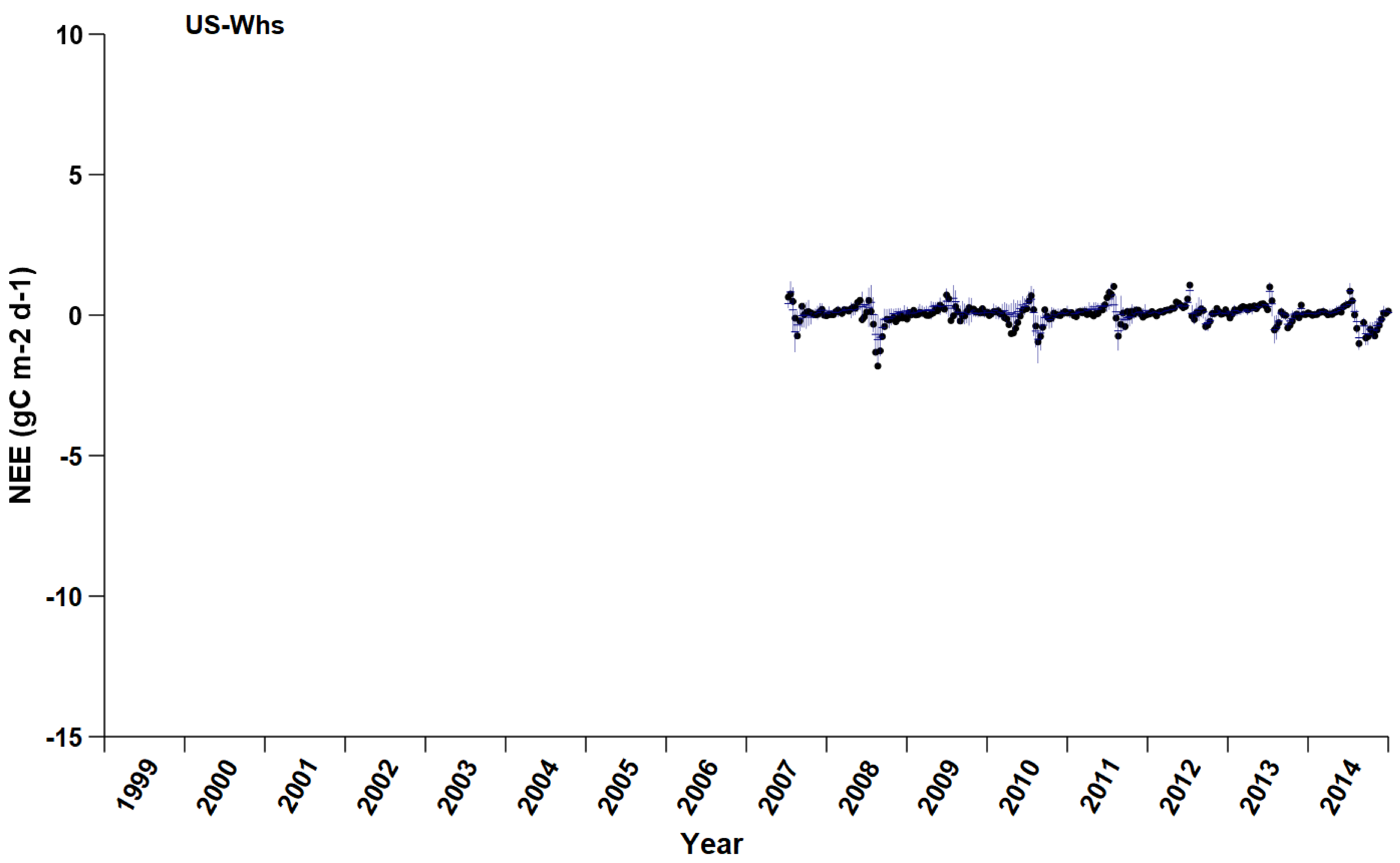

US-Whs
NEE (gC m-2 d-1)
10
5
0
-5
-10
-15
1999
2000
2001
2002
2003
2004
2005
2006
2007
2008
2009
2010
2011
2012
2013
2014
Year

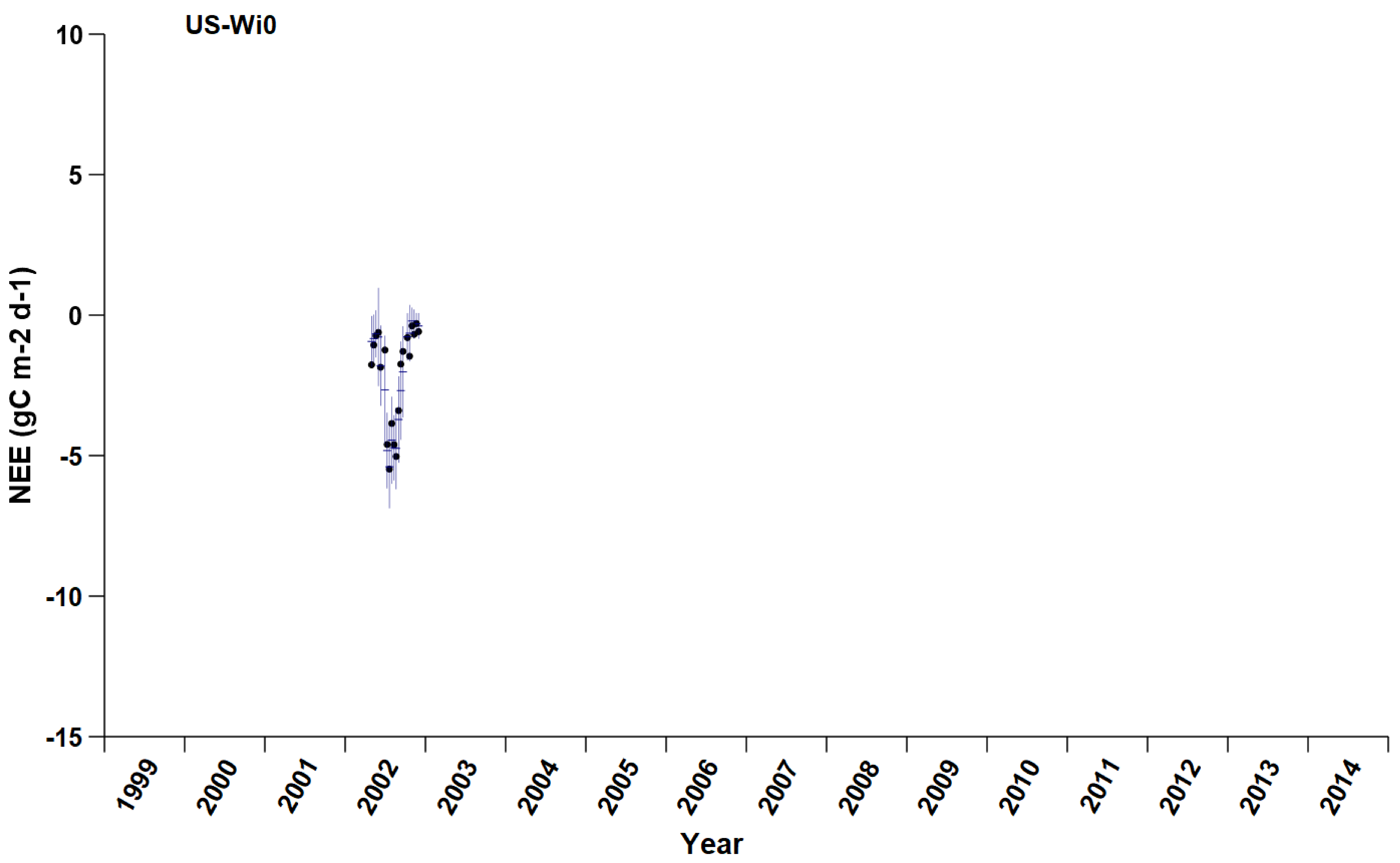
US-Wi0
10
5
0
-5
-10
-15
NEE (gC m-2 d-1)
1999
2000
2001
2002
2003
2004
2005
2006
2007
2008
2009
2010
2011
2012
2013
2014
Year

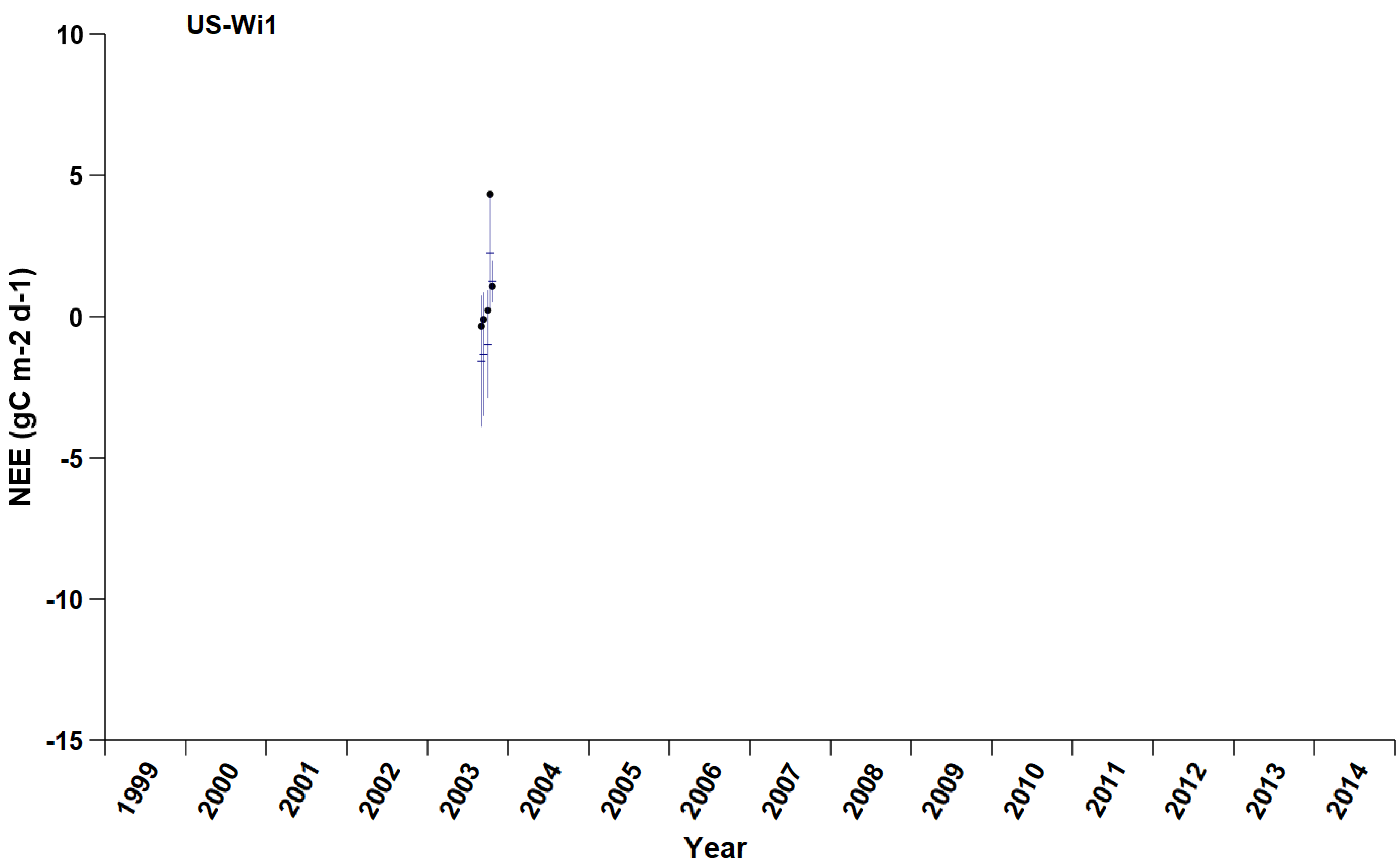
US-Wi1
NEE (gC m-2 d-1)
10
5
0
-5
-10
-15
1999
2000
2001
2002
2003
2004
2005
2006
2007
2008
2009
2010
2011
2012
2013
2014
Year

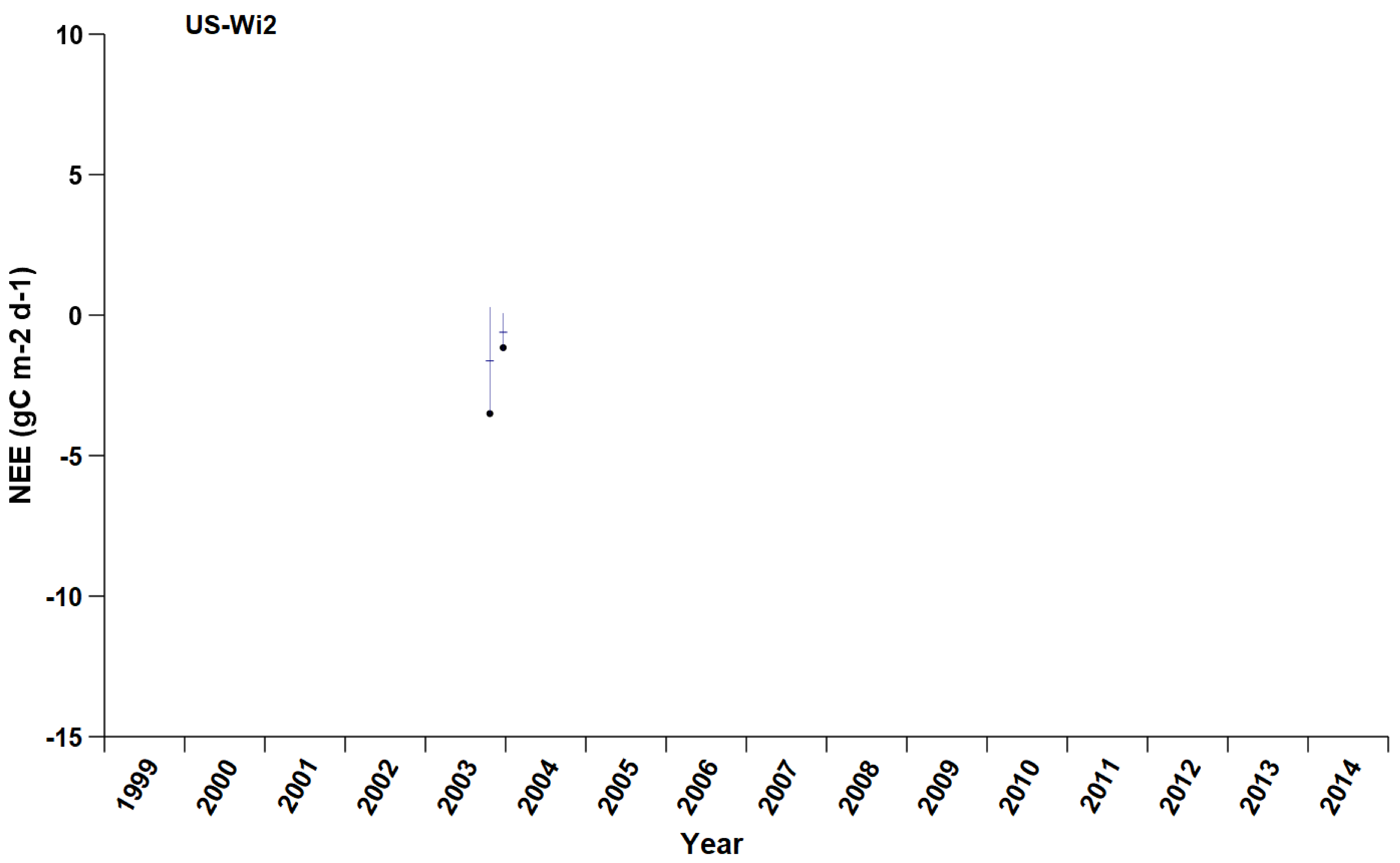
US-Wi2
NEE (gC m-2 d-1)
10
5
0
-5
-10
-15
1999
2000
2001
2002
2003
2004
2005
2006
2007
2008
2009
2010
2011
2012
2013
2014
Year

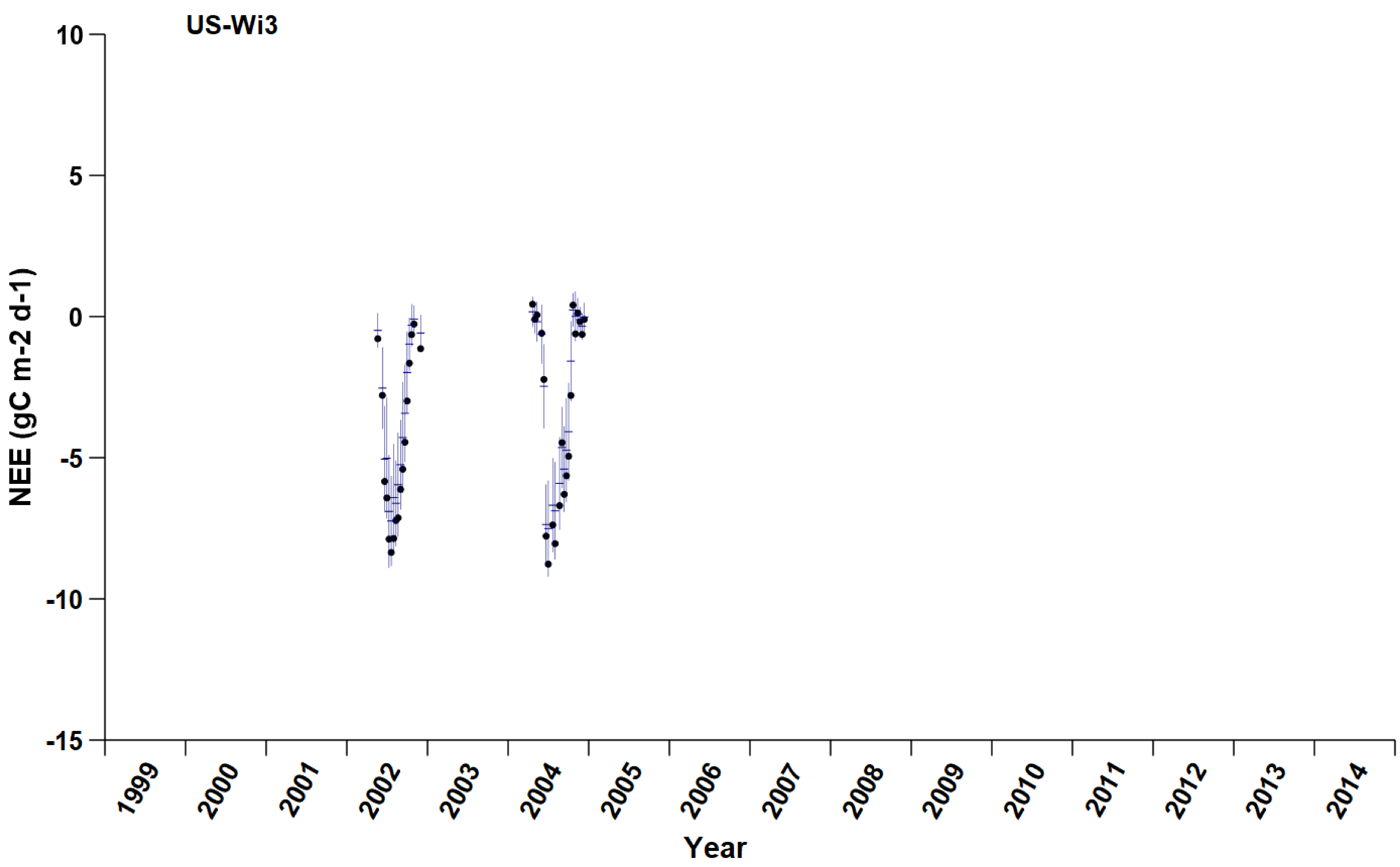

US-Wi3
NEE (gC m-2 d-1)
10
5
0
-5
-10
-15
1999
2000
2001
2002
2003
2004
2005
2006
2007
2008
2009
2010
2011
2012
2013
2014
Year

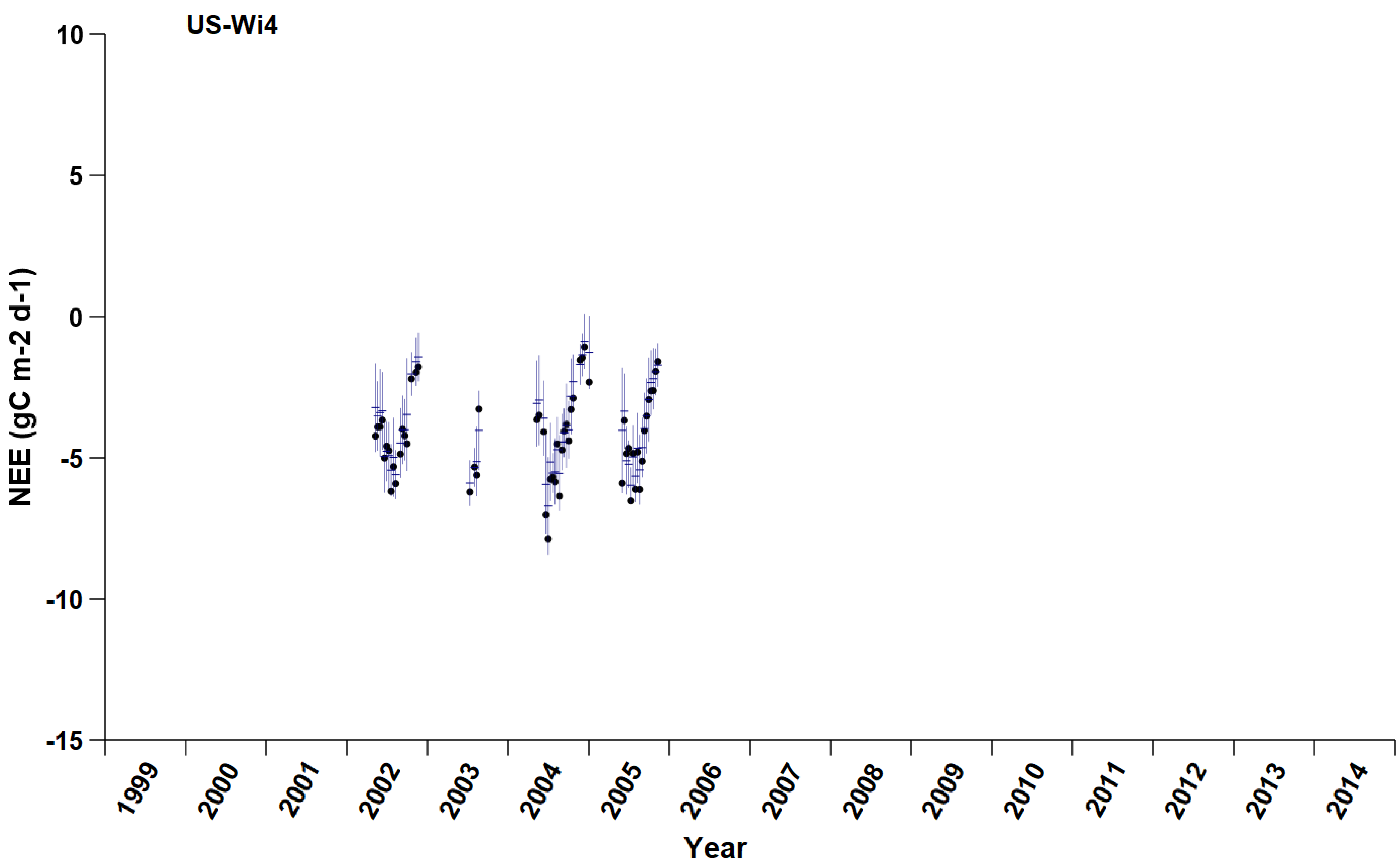
US-Wi4
NEE (gC m-2 d-1)
10
5
0
-5
-10
-15
1999
2000
2001
2002
2003
2004
2005
2006
2007
2008
2009
2010
2011
2012
2013
2014
Year

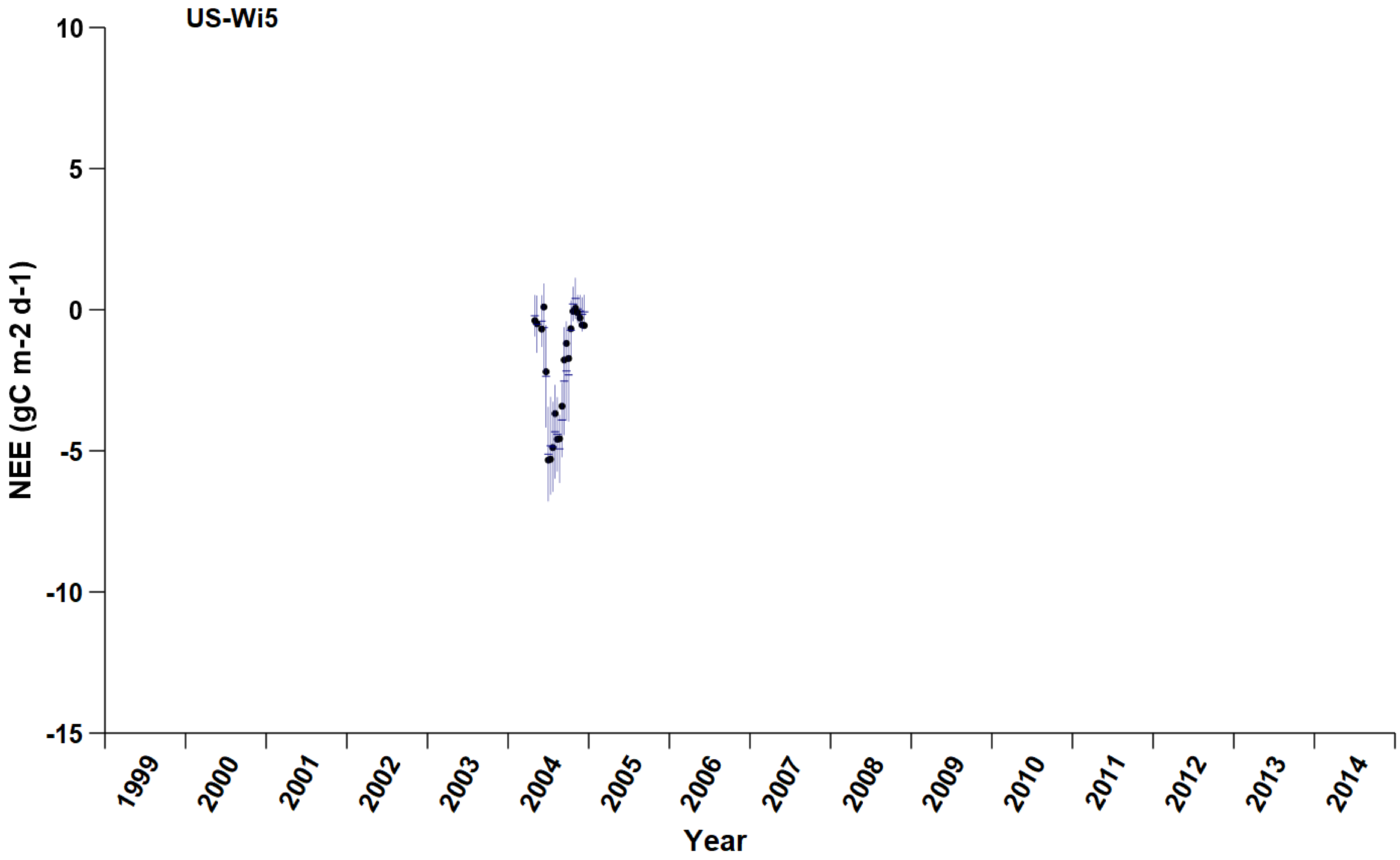
US-Wi5
NEE (gC m-2 d-1)
10
5
0
-5
-10
-15
1999
2000
2001
2002
2003
2004
2005
2006
2007
2008
2009
2010
2011
2012
2013
2014
Year

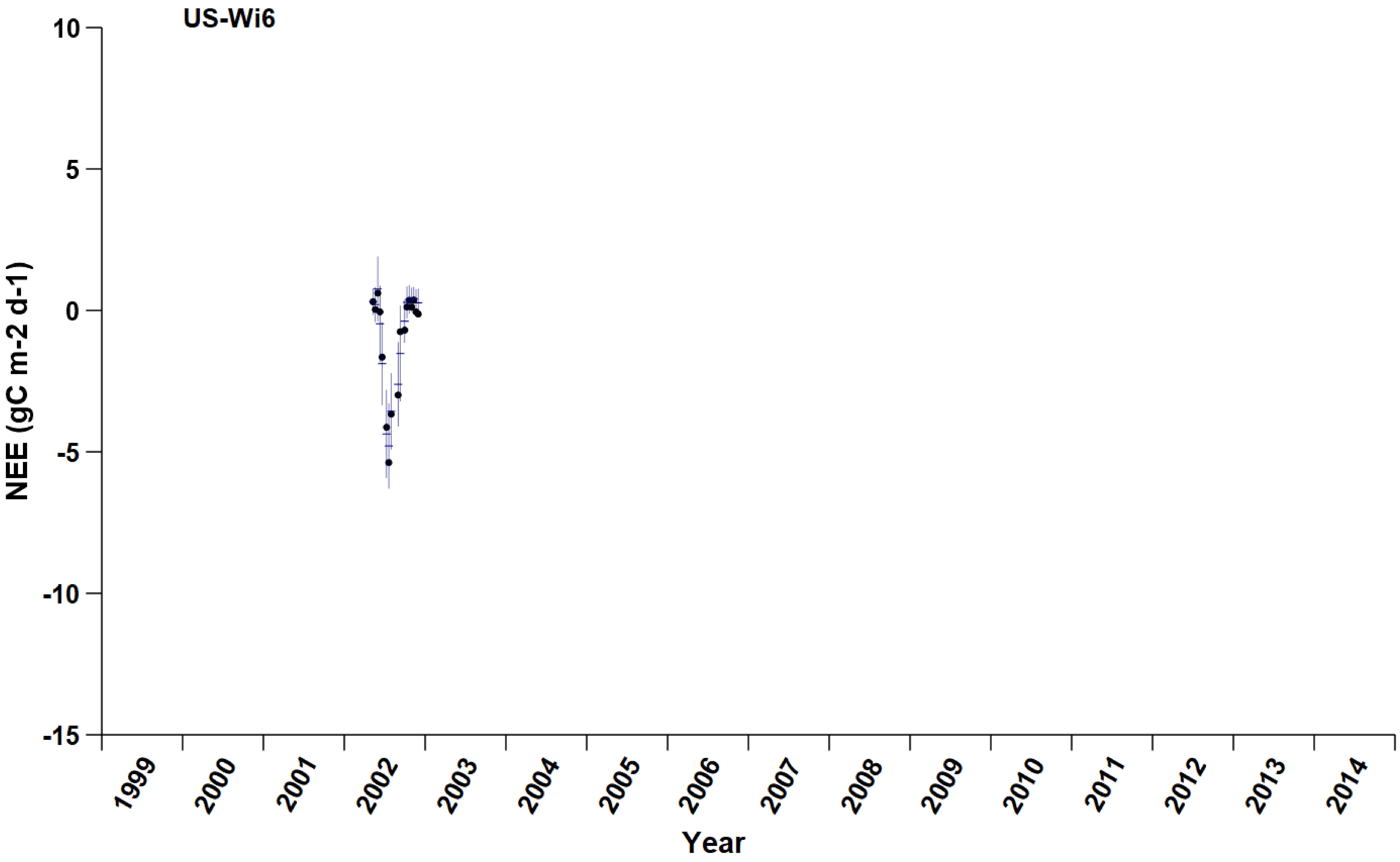

US-Wi6
NEE (gC m-2 d-1)
10
5
0
-5
-10
-15
1999
2000
2001
2002
2003
2004
2005
2006
2007
2008
2009
2010
2011
2012
2013
2014
Year

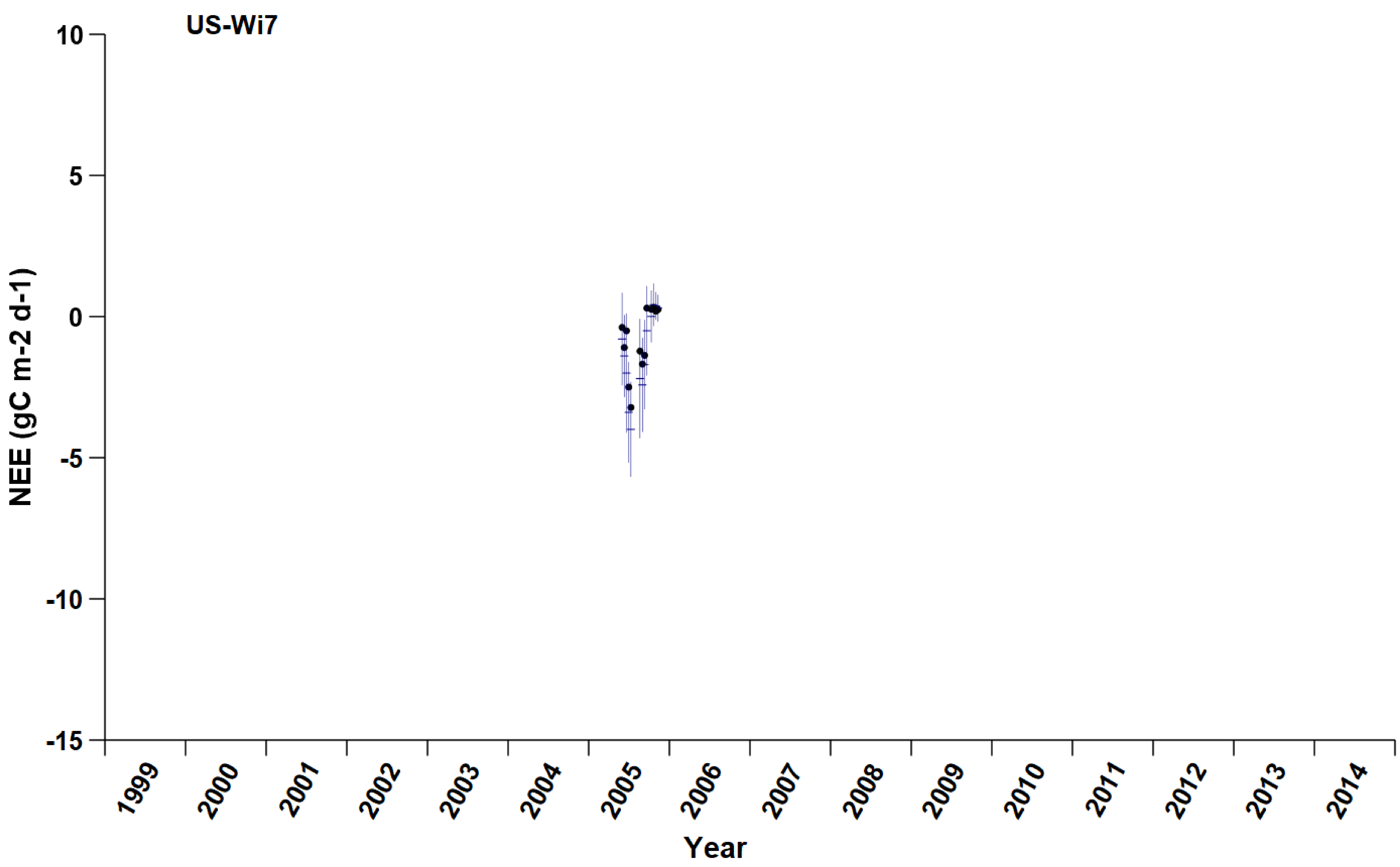

US-Wi7
NEE (gC m-2 d-1)
10
5
0
-5
-10
-15
1999
2000
2001
2002
2003
2004
2005
2006
2007
2008
2009
2010
2011
2012
2013
2014
Year

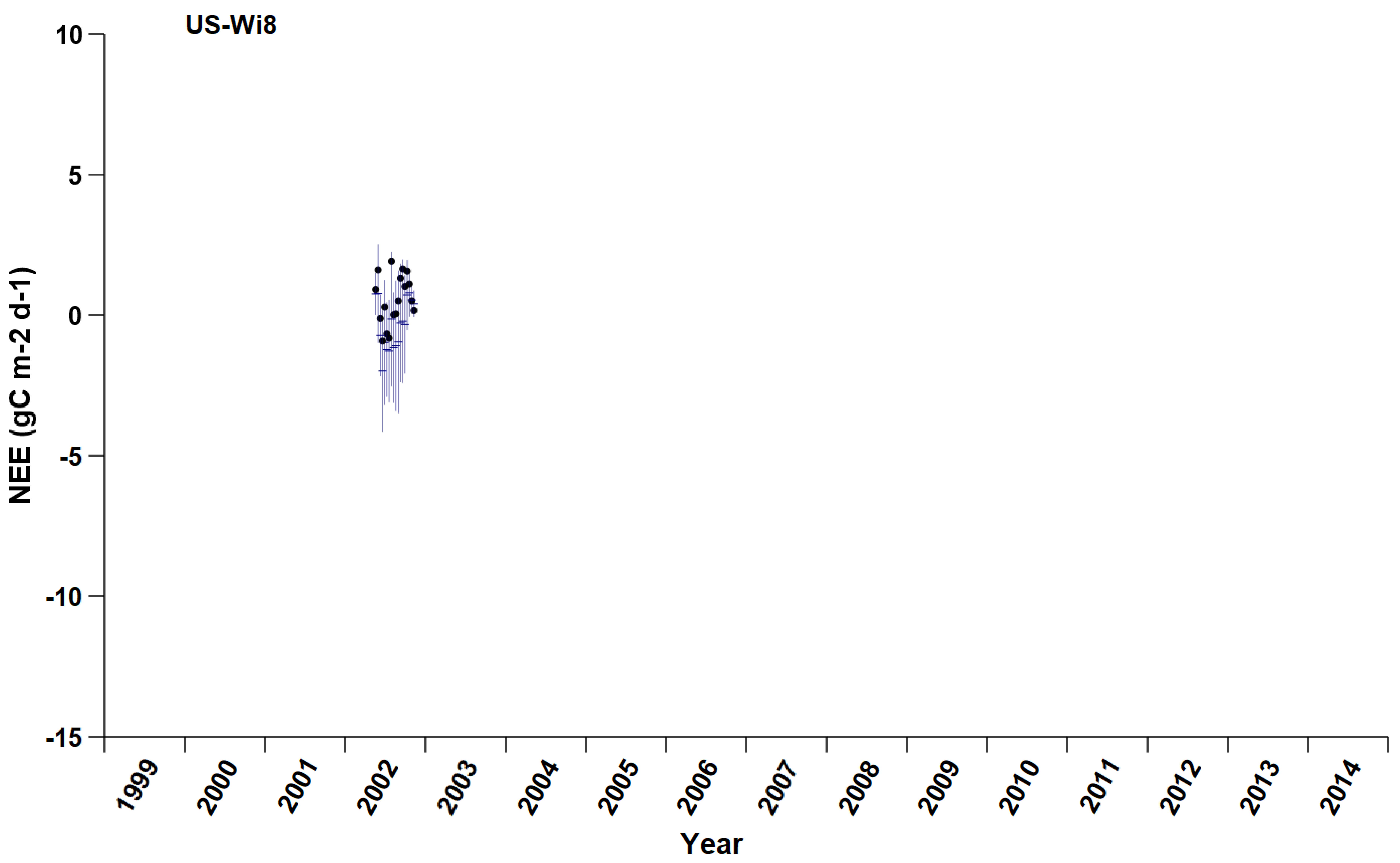
US-Wi8
10
5
0
-5
-10
-15
NEE (gC m-2 d-1)
1999
2000
2001
2002
2003
2004
2005
2006
2007
2008
2009
2010
2011
2012
2013
2014
Year

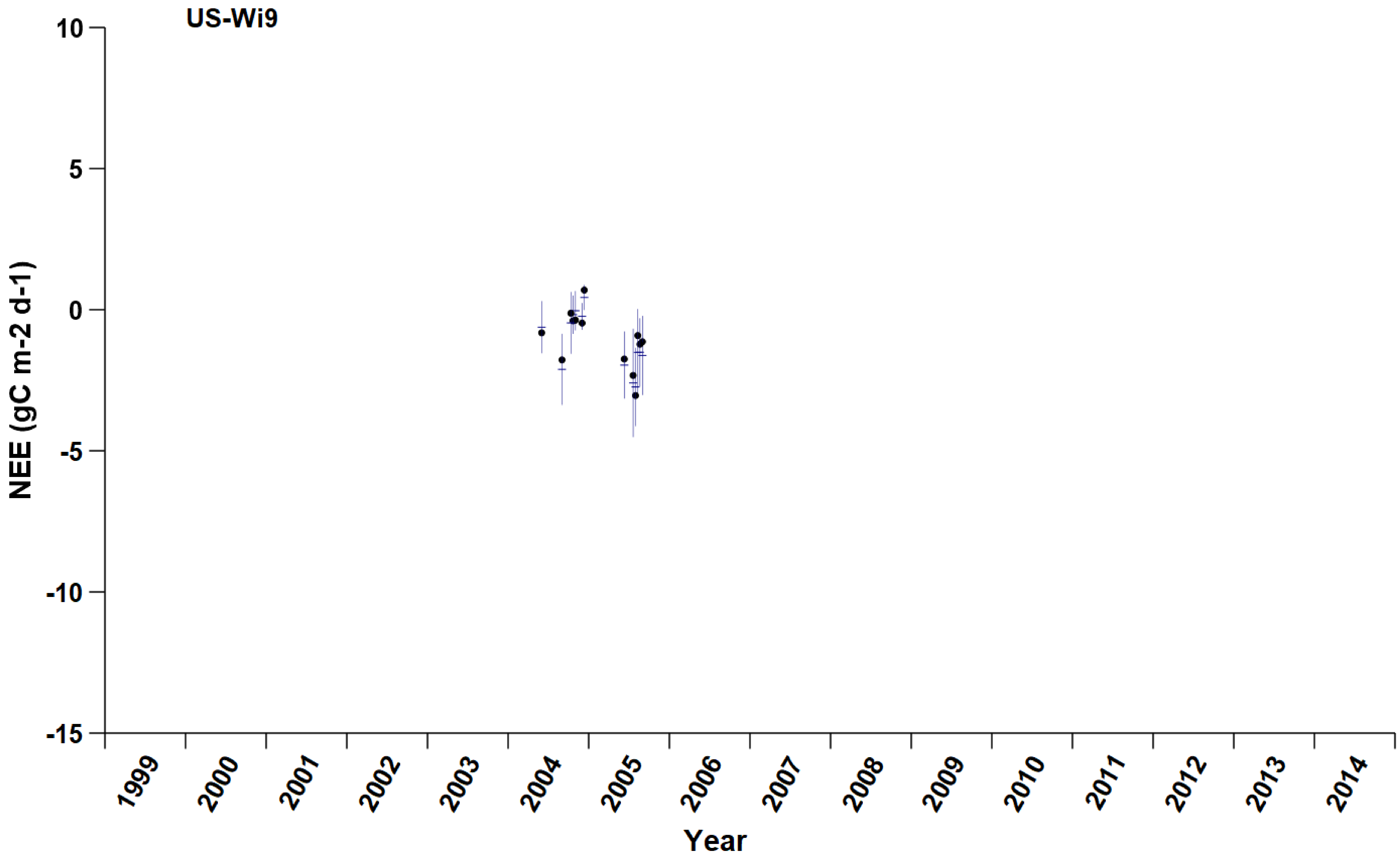
US-Wi9
NEE (gC m-2 d-1)
10
5
0
-5
-10
-15
1999
2000
2001
2002
2003
2004
2005
2006
2007
2008
2009
2010
2011
2012
2013
2014
Year

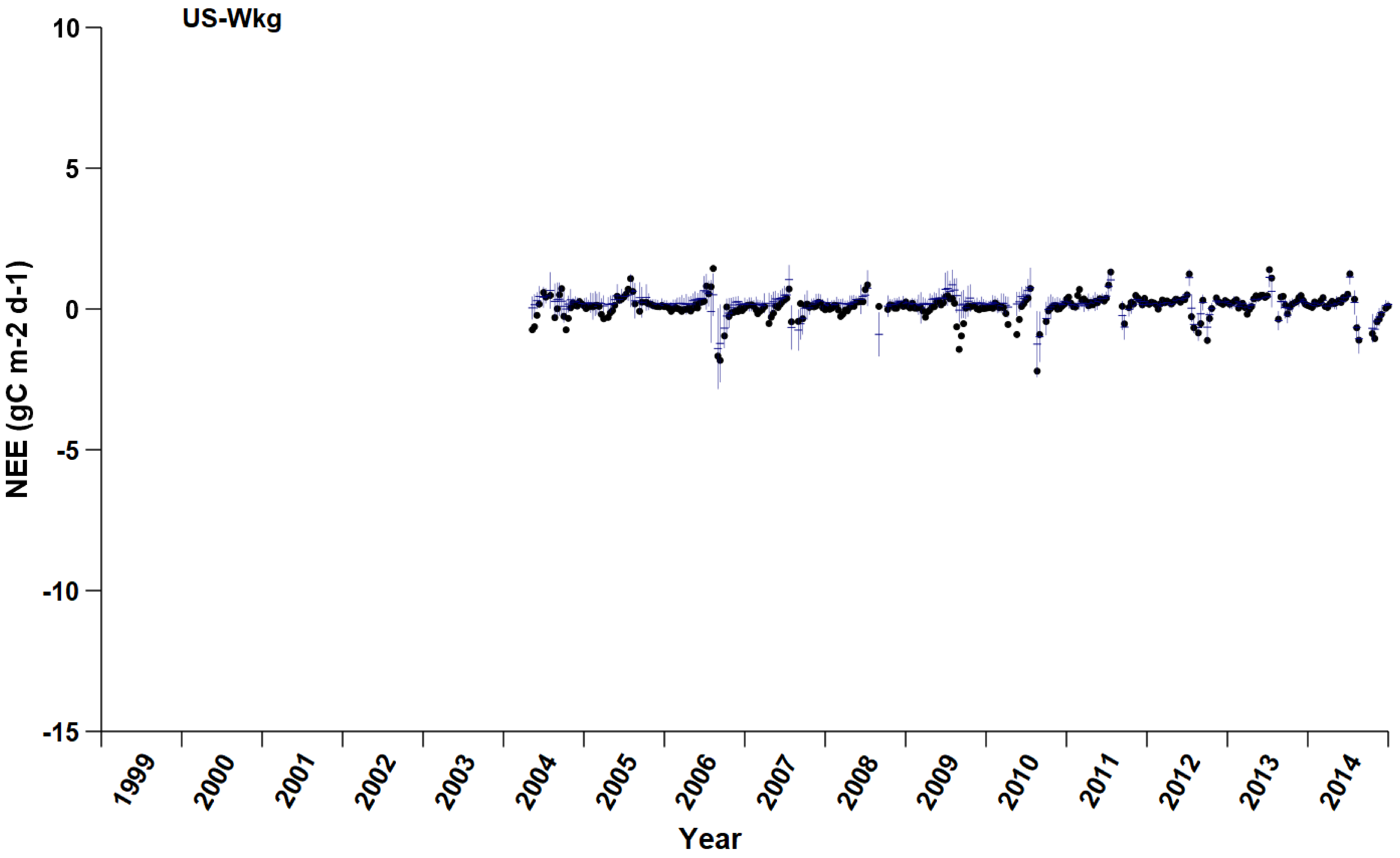

US-Wkg
10
5
0
-5
-10
-15
NEE (gC m-2 d-1)
1999
2000
2001
2002
2003
2004
2005
2006
2007
2008
2009
2010
2011
2012
2013
2014
Year

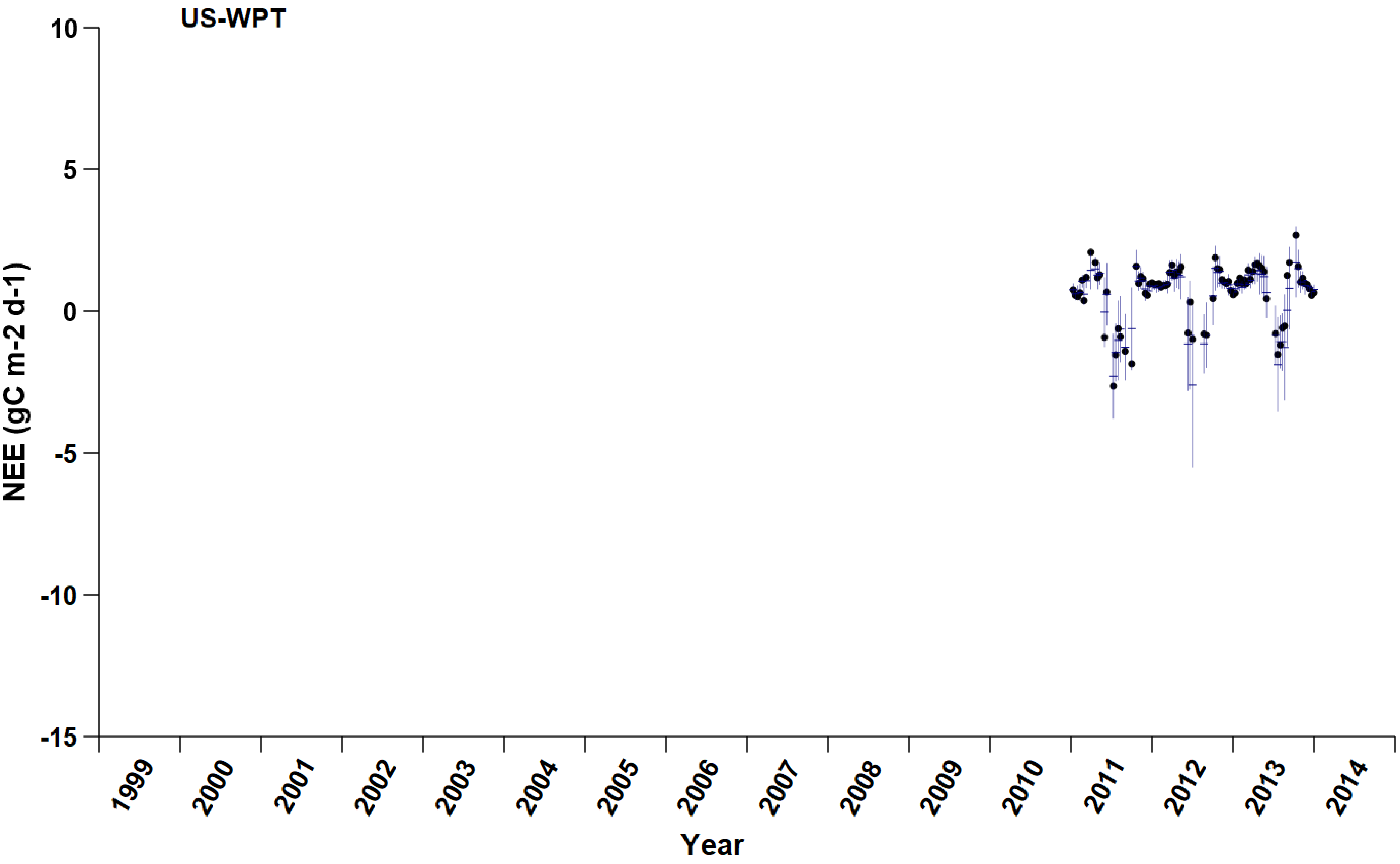
US-WPT
NEE (gC m-2 d-1)
10
5
0
-5
-10
-15
1999
2000
2001
2002
2003
2004
2005
2006
2007
2008
2009
2010
2011
2012
2013
2014
Year

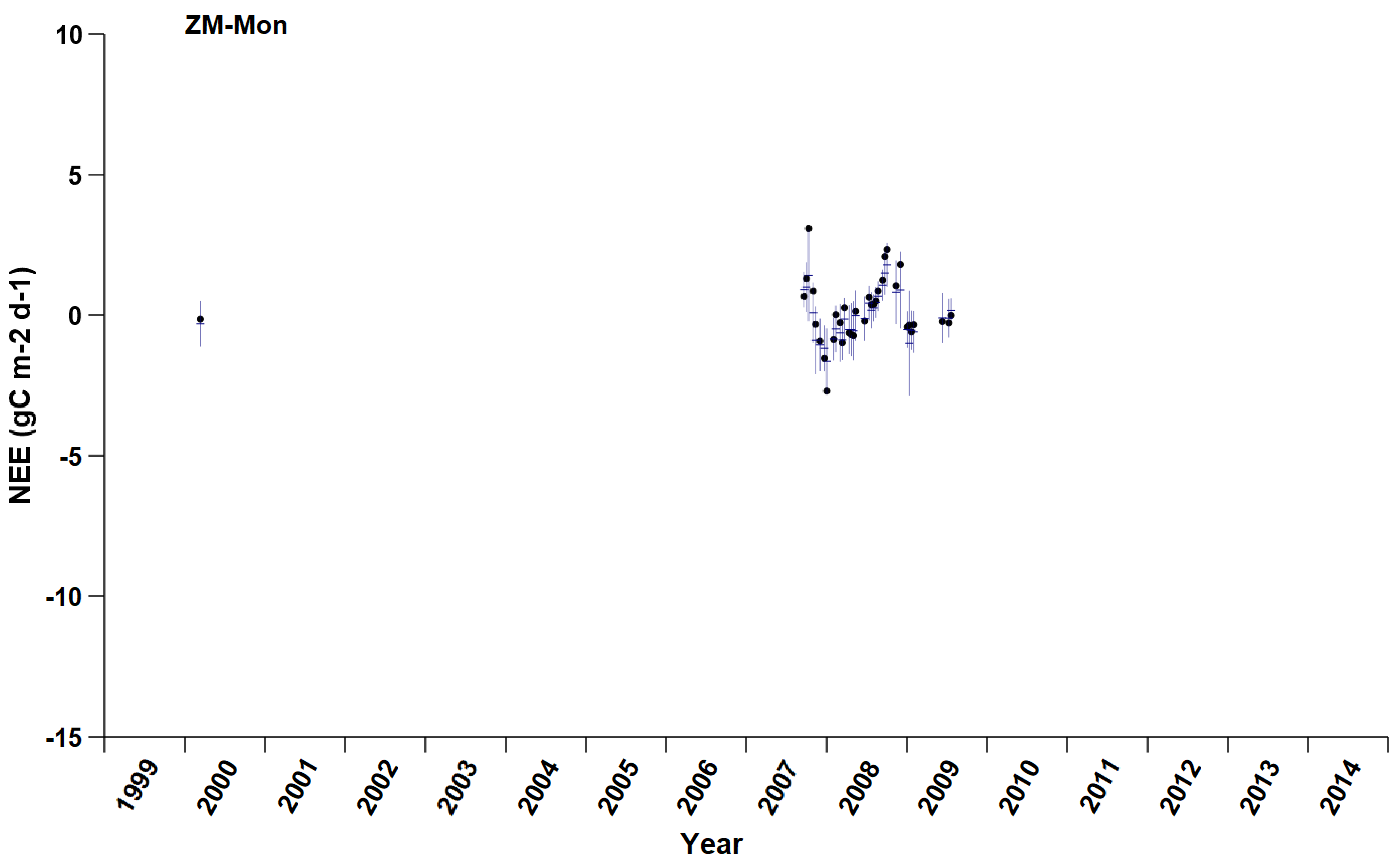

ZM-Mon
NEE (gC m-2 d-1)
10
5
0
-5
-10
-15
1999
2000
2001
2002
2003
2004
2005
2006
2007
2008
2009
2010
2011
2012
2013
2014
Year
