# Supplementary material for: Global terrestrial carbon fluxes of 1999–2019 estimated by upscaling eddy covariance data with a random forest
Source: Sci Data. 2020 Sep 24;7:313. doi: 10.1038/s41597-020-00653-5 (PMC7518252; doi:10.1038/s41597-020-00653-5)
Supplement: Supplementary file 1 — Supplementary information [file 41597_2020_653_MOESM1_ESM.zip › scidata/Supplementary File 3 (2).pdf]

GPP Time Series

This document contains plots of the time series variations of observed GPP and model predictions. Dark dots are observations. Horizontal blue bars are the means (predictions) and vertical blue lines are the standard deviations of flux values in the terminal nodes of 500 trees.

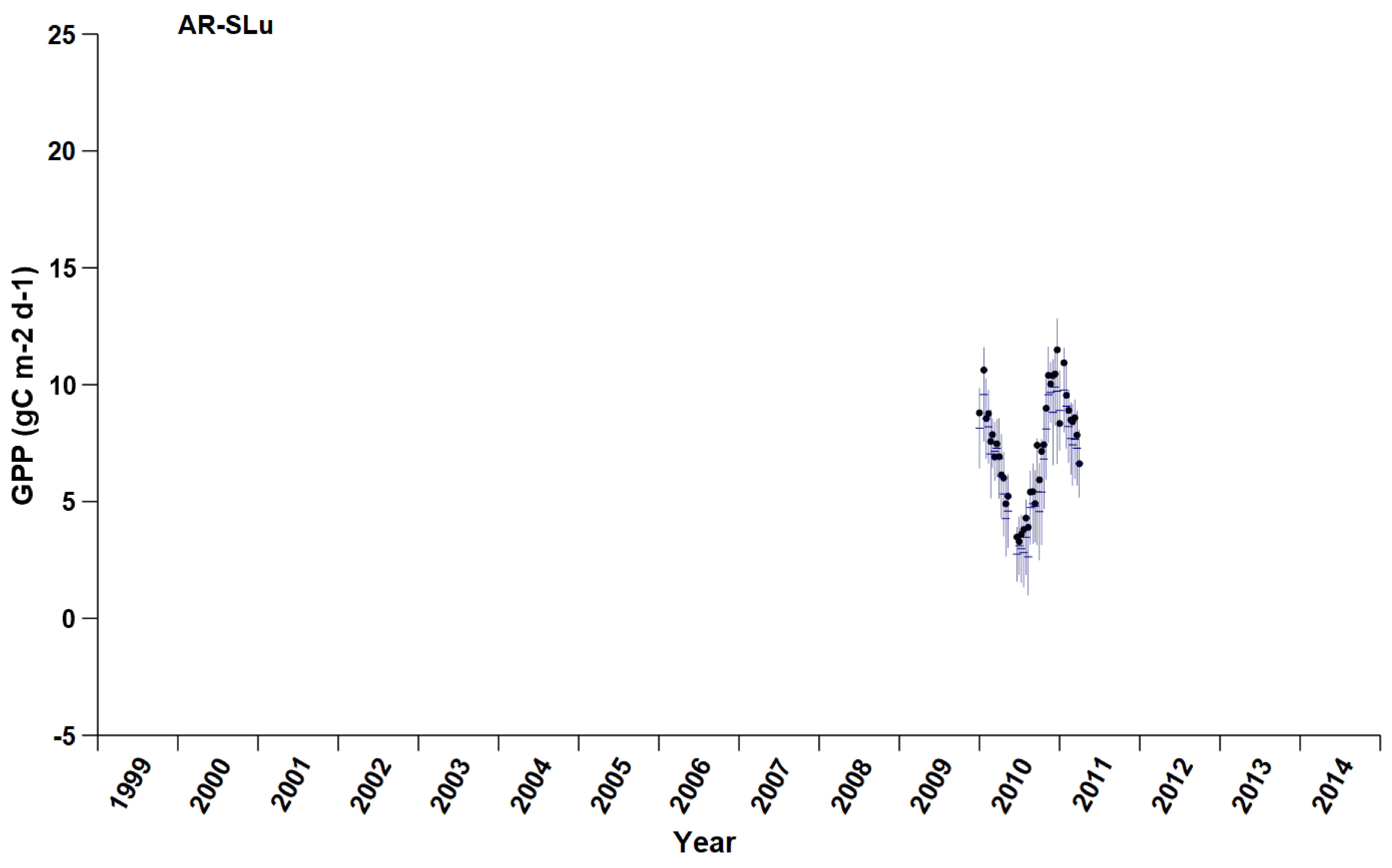

AR-SLu
GPP (gC m-2 d-1)
25
20
15
10
5
0
-5
1999
2000
2001
2002
2003
2004
2005
2006
2007
2008
2009
2010
2011
2012
2013
2014
Year

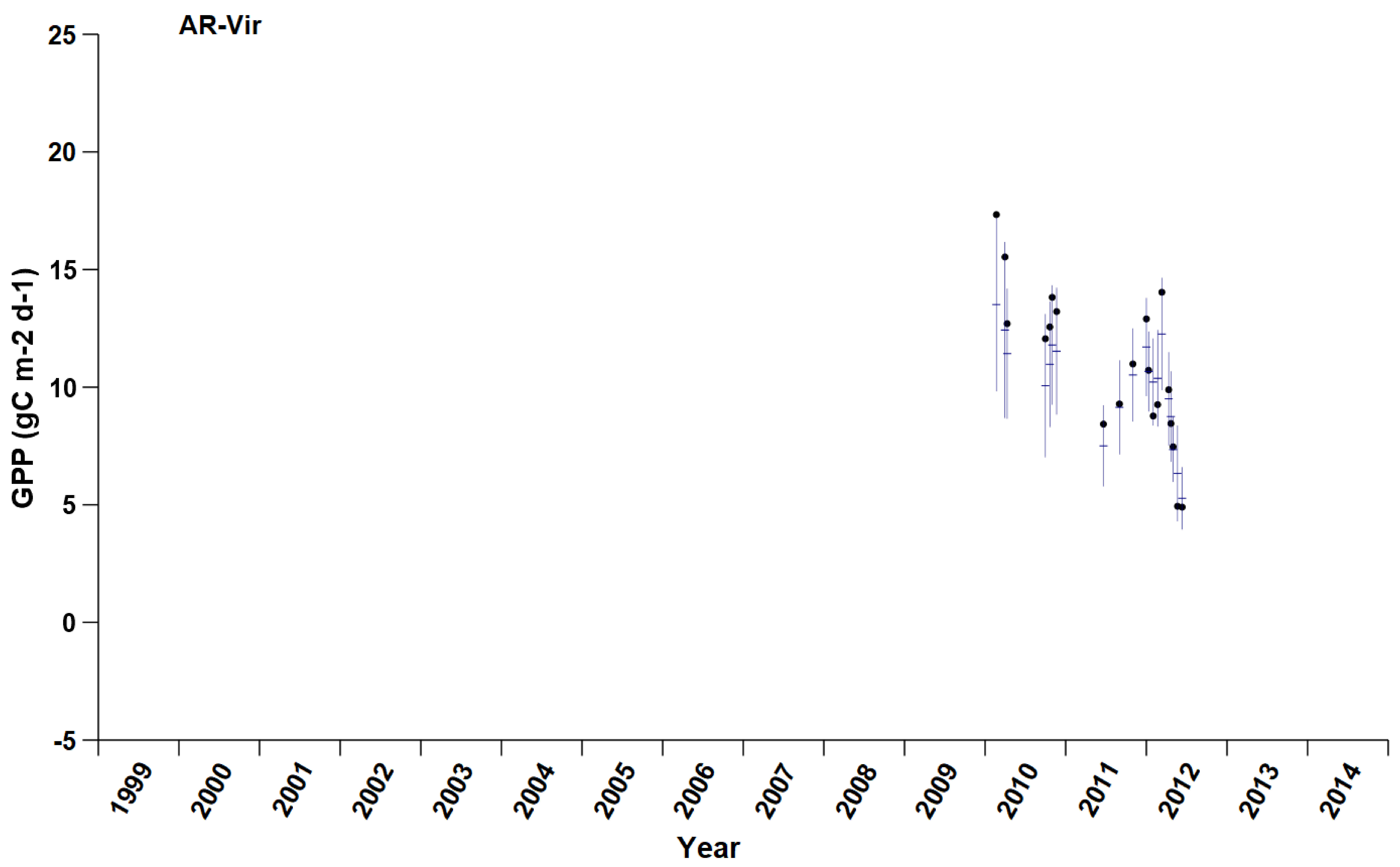

AR-Vir
GPP (gC m-2 d-1)
25
20
15
10
5
0
-5
1999
2000
2001
2002
2003
2004
2005
2006
2007
2008
2009
2010
2011
2012
2013
2014
Year

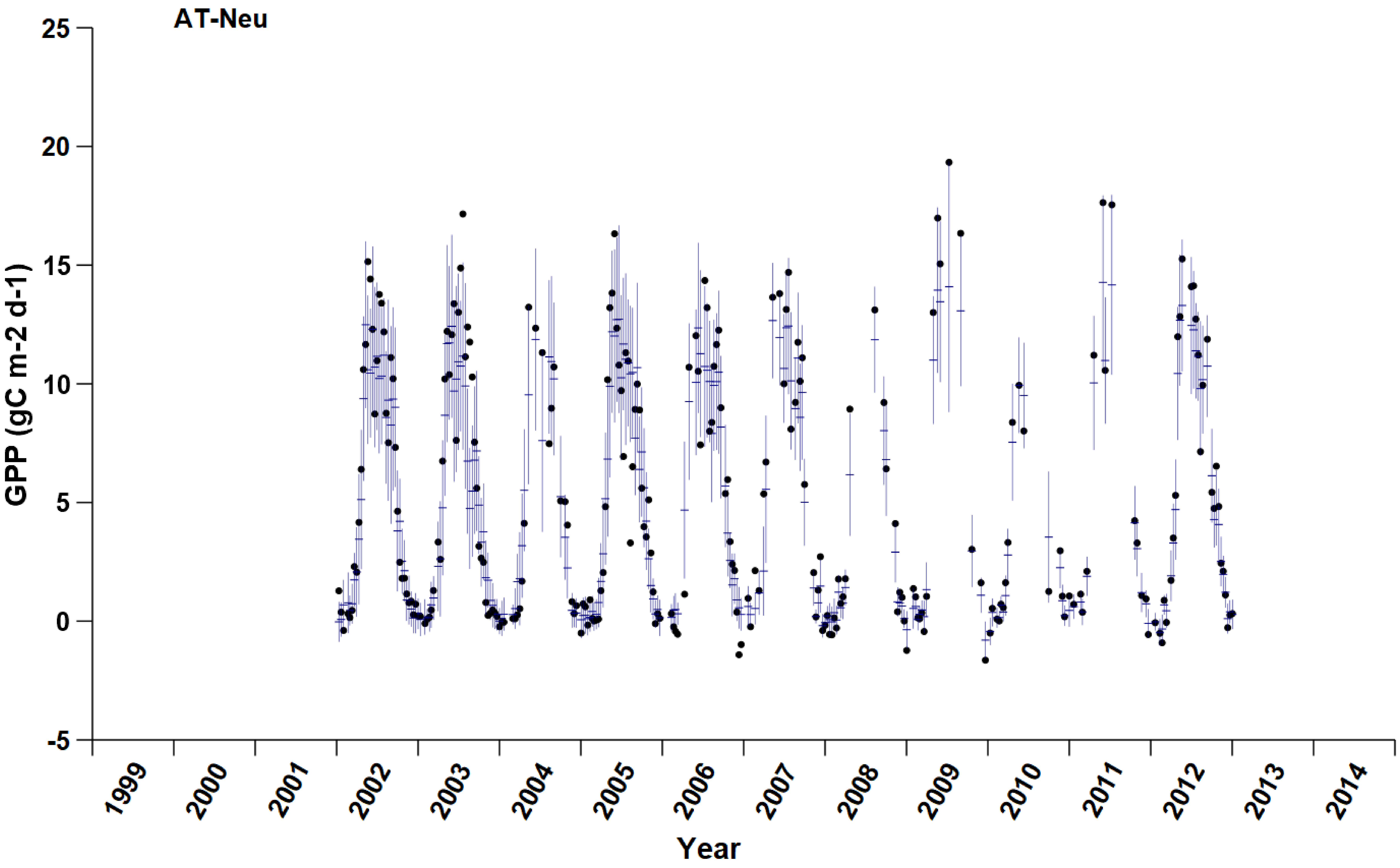

AT-Neu
25
20
15
10
5
0
-5
GPP (gC m-2 d-1)
1999
2000
2001
2002
2003
2004
2005
2006
2007
2008
2009
2010
2011
2012
2013
2014
Year

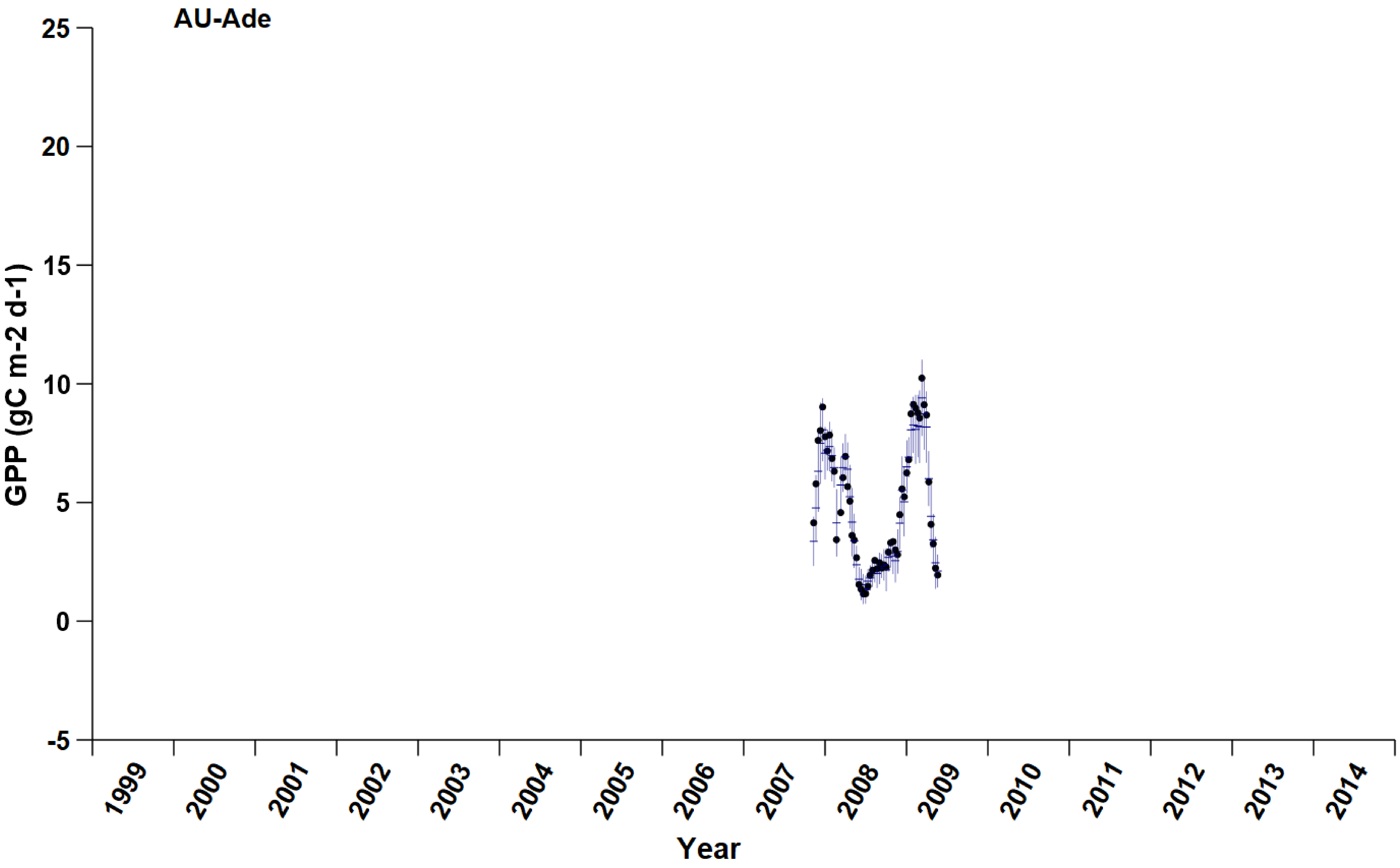
AU-Ade
GPP (gC m-2 d-1)
25
20
15
10
5
0
-5
1999
2000
2001
2002
2003
2004
2005
2006
2007
2008
2009
2010
2011
2012
2013
2014
Year

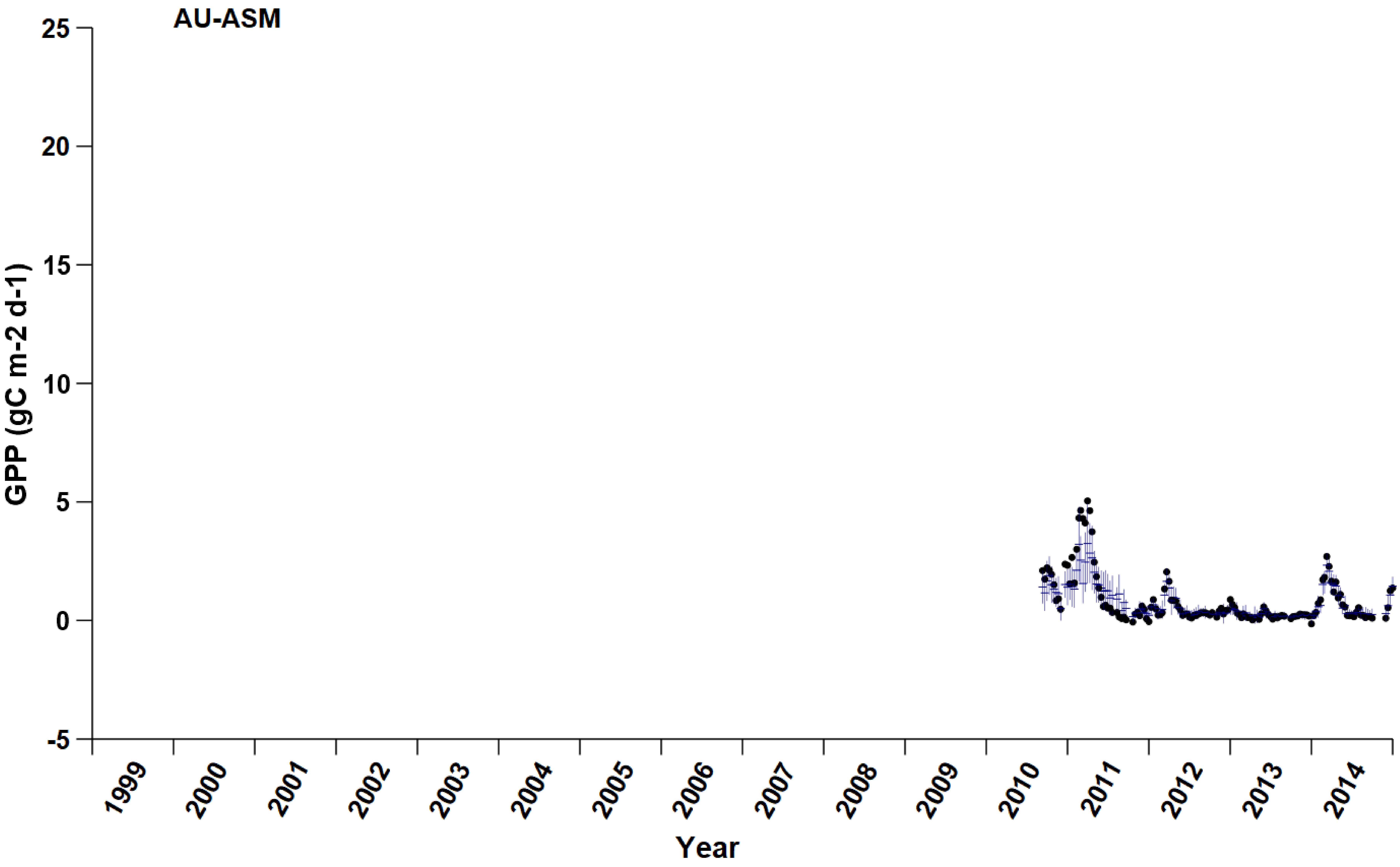
AU-ASM
GPP (gC m-2 d-1)
25
20
15
10
5
0
-5
1999
2000
2001
2002
2003
2004
2005
2006
2007
2008
2009
2010
2011
2012
2013
2014
Year

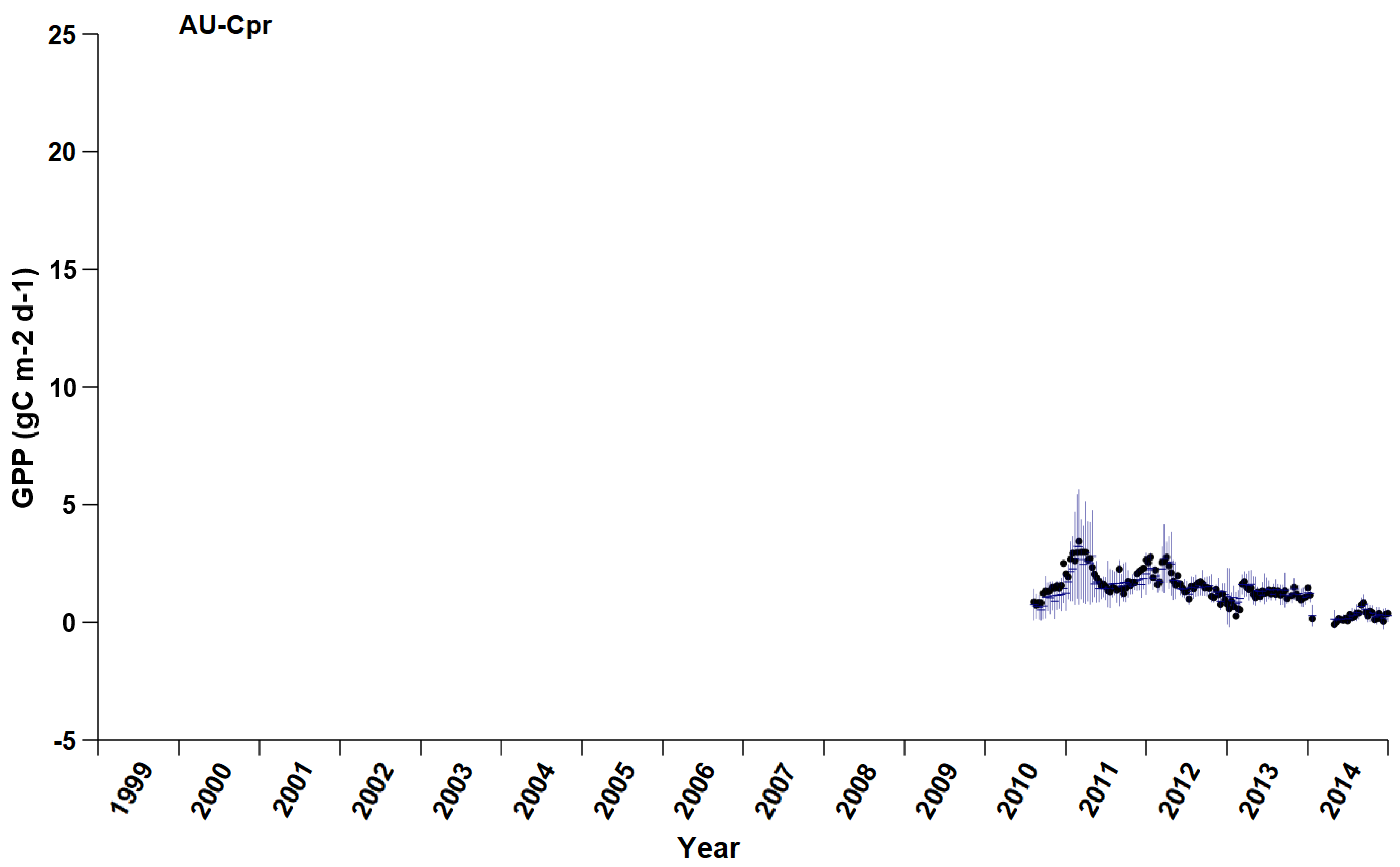

AU-Cpr
GPP (gC m-2 d-1)
25
20
15
10
5
0
-5
1999
2000
2001
2002
2003
2004
2005
2006
2007
2008
2009
2010
2011
2012
2013
2014
Year

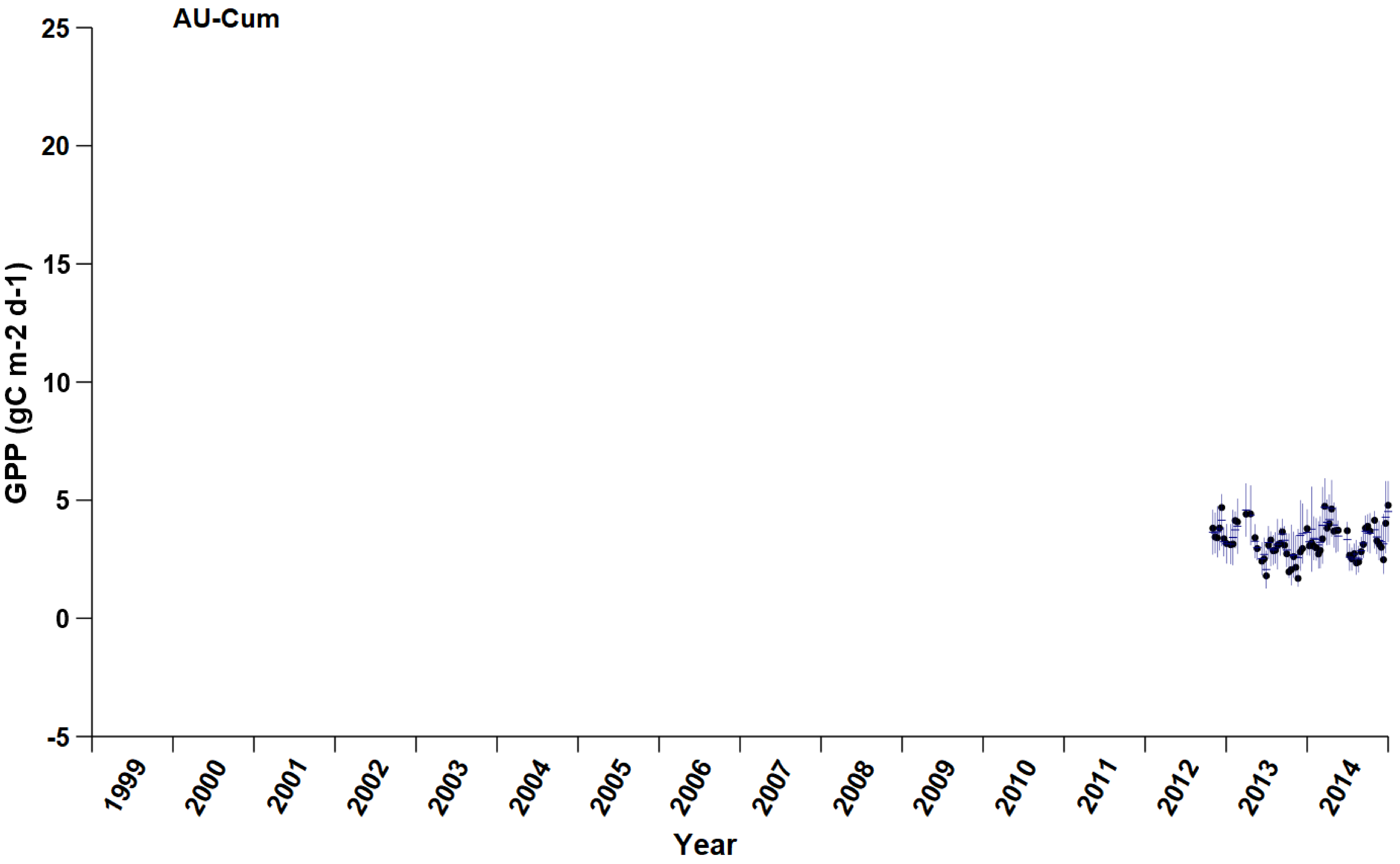

AU-Cum
GPP (gC m-2 d-1)
25
20
15
10
5
0
-5
1999
2000
2001
2002
2003
2004
2005
2006
2007
2008
2009
2010
2011
2012
2013
2014
Year

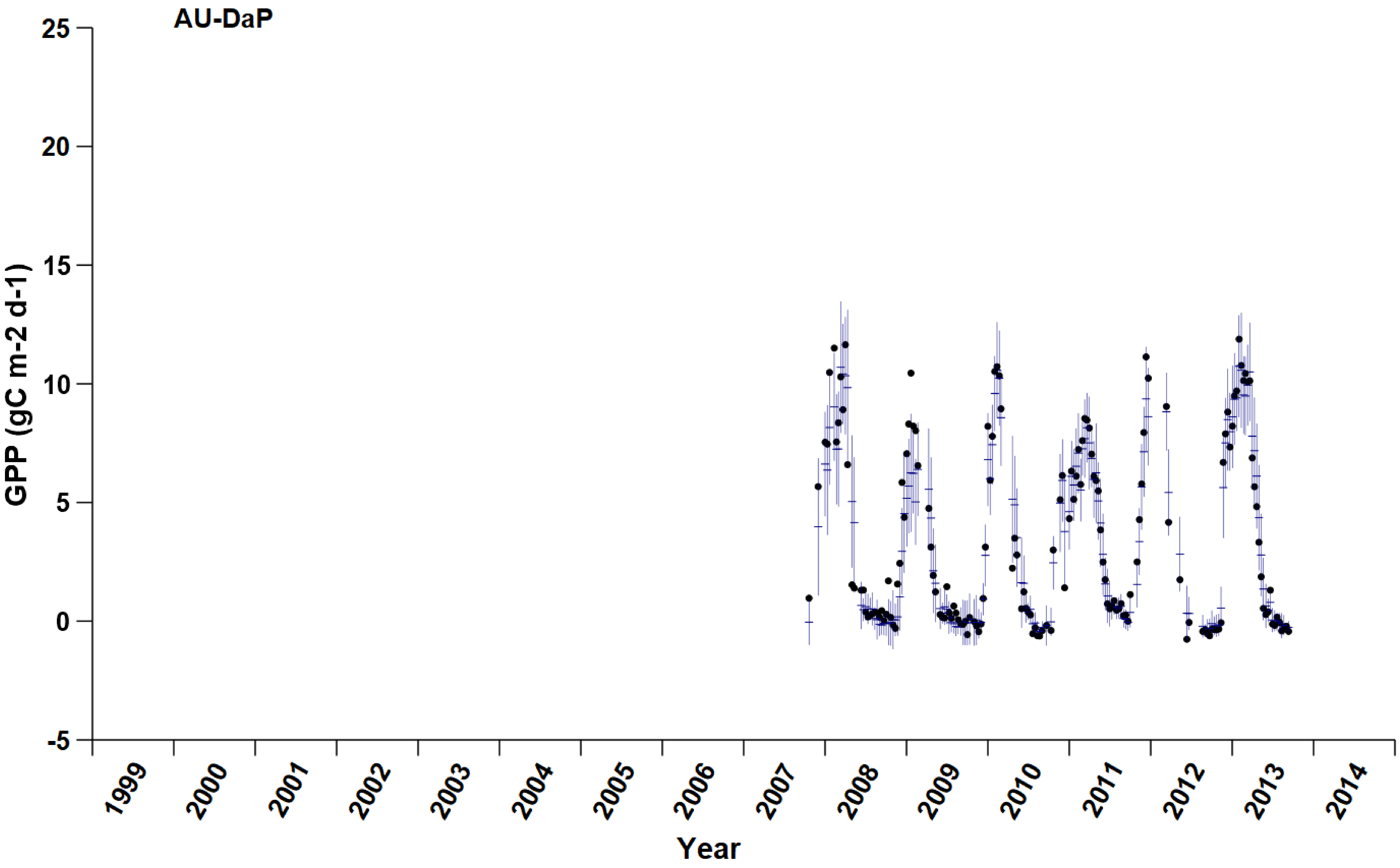

AU-DaP
GPP (gC m-2 d-1)
25
20
15
10
5
0
-5
1999
2000
2001
2002
2003
2004
2005
2006
2007
2008
2009
2010
2011
2012
2013
2014
Year

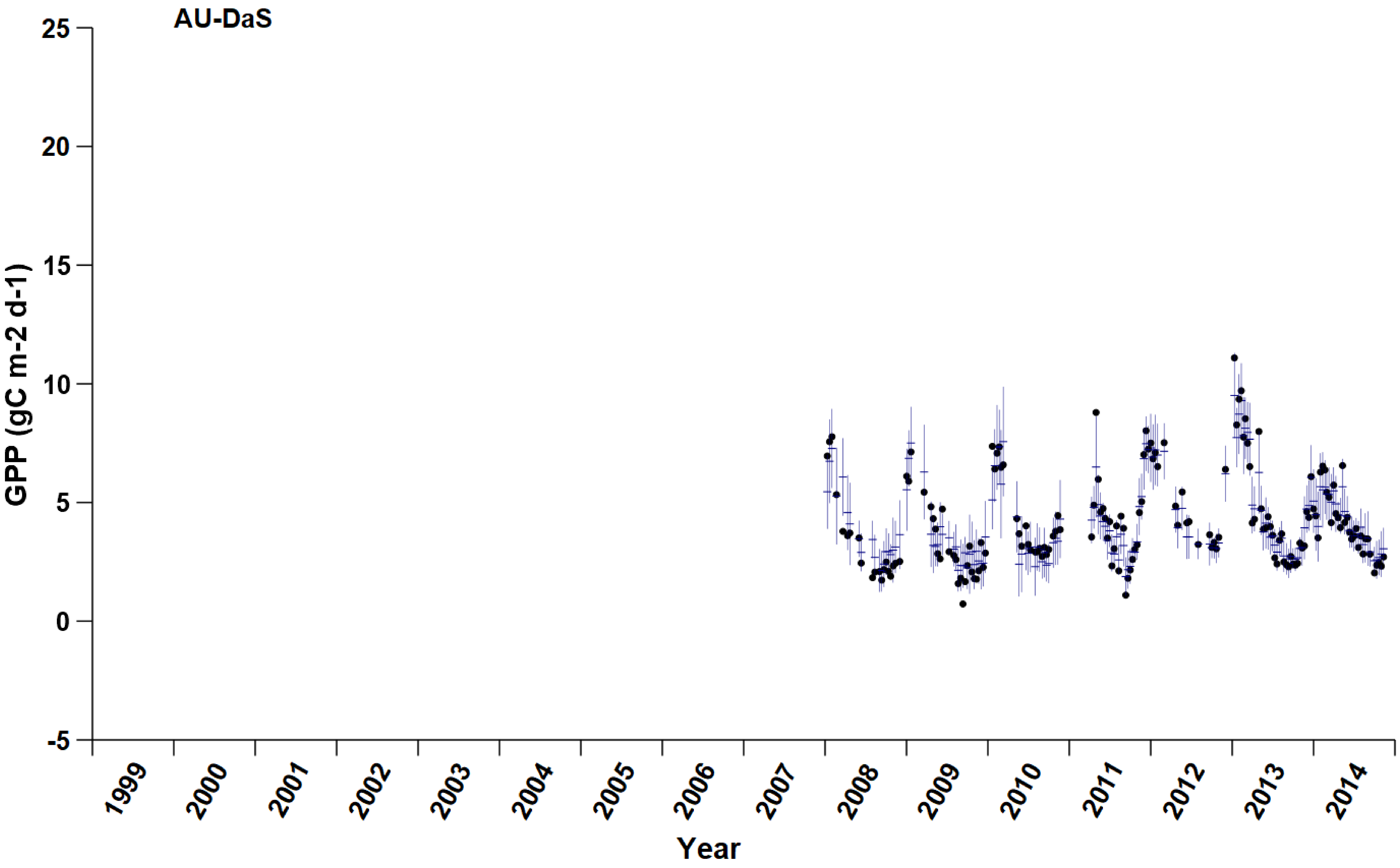
AU-DaS
GPP (gC m-2 d-1)
25
20
15
10
5
0
-5
1999
2000
2001
2002
2003
2004
2005
2006
2007
2008
2009
2010
2011
2012
2013
2014
Year

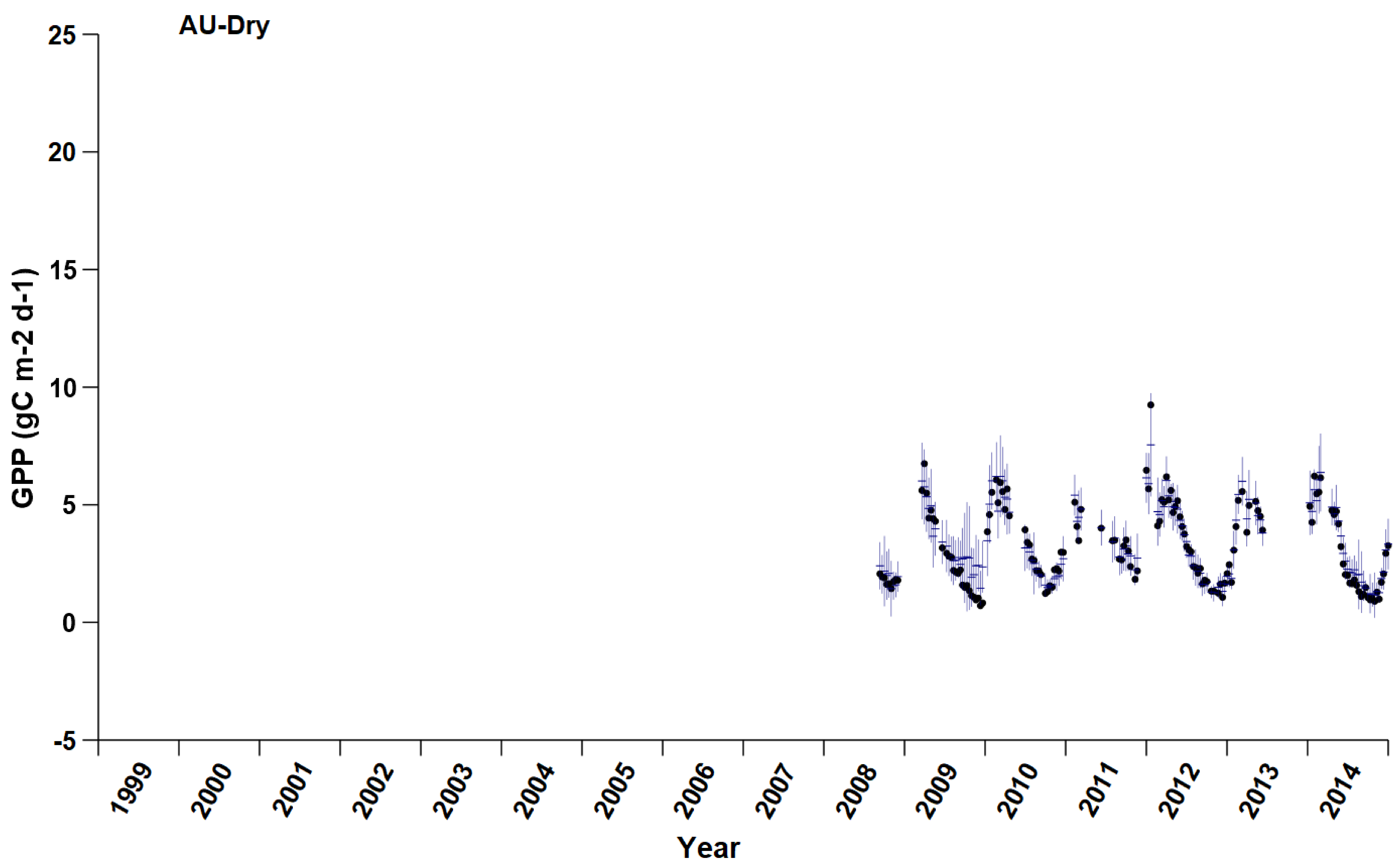
AU-Dry
GPP (gC m-2 d-1)
25
20
15
10
5
0
-5
1999
2000
2001
2002
2003
2004
2005
2006
2007
2008
2009
2010
2011
2012
2013
2014
Year

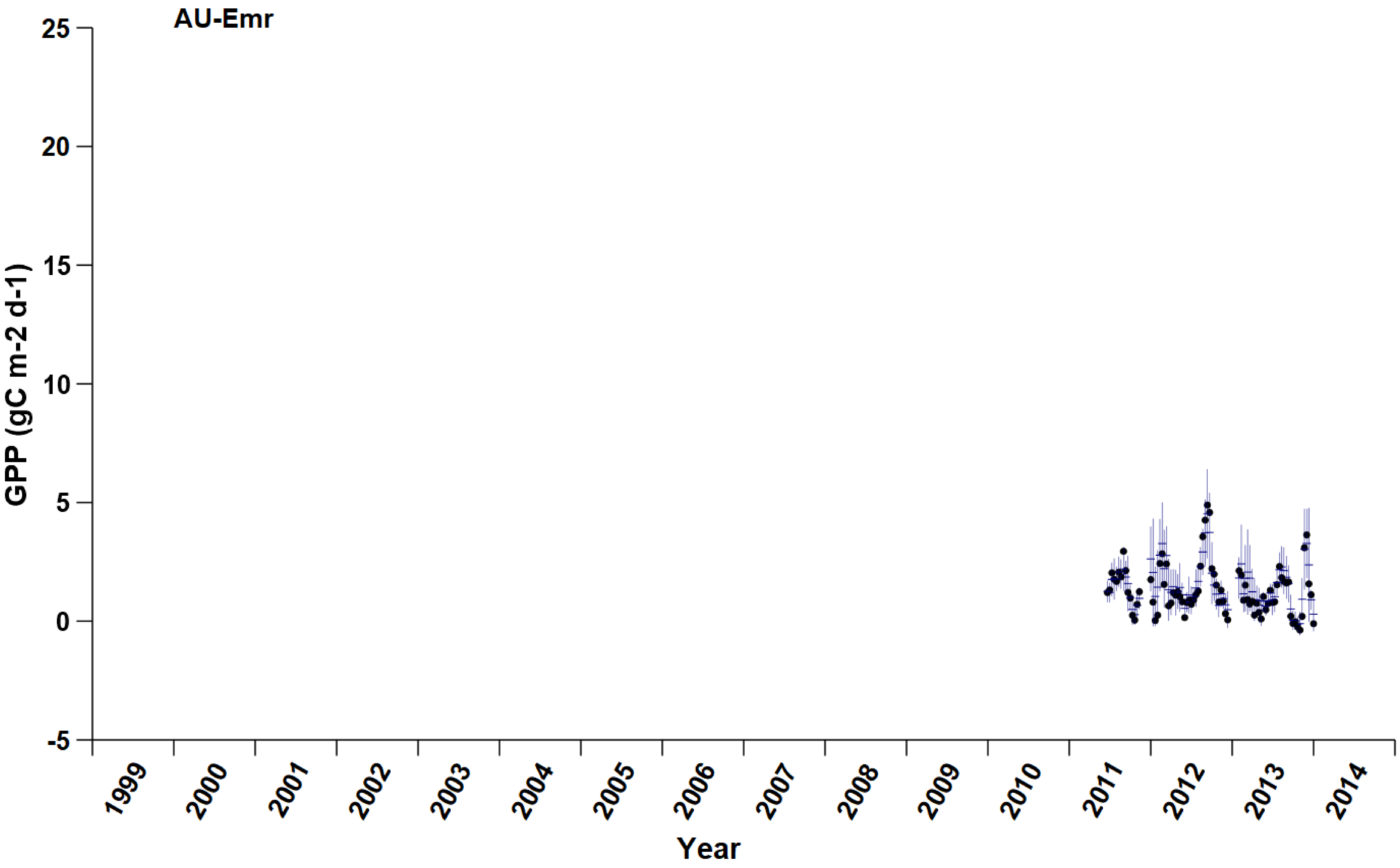

AU-Emr
GPP (gC m-2 d-1)
25
20
15
10
5
0
-5
1999
2000
2001
2002
2003
2004
2005
2006
2007
2008
2009
2010
2011
2012
2013
2014
Year

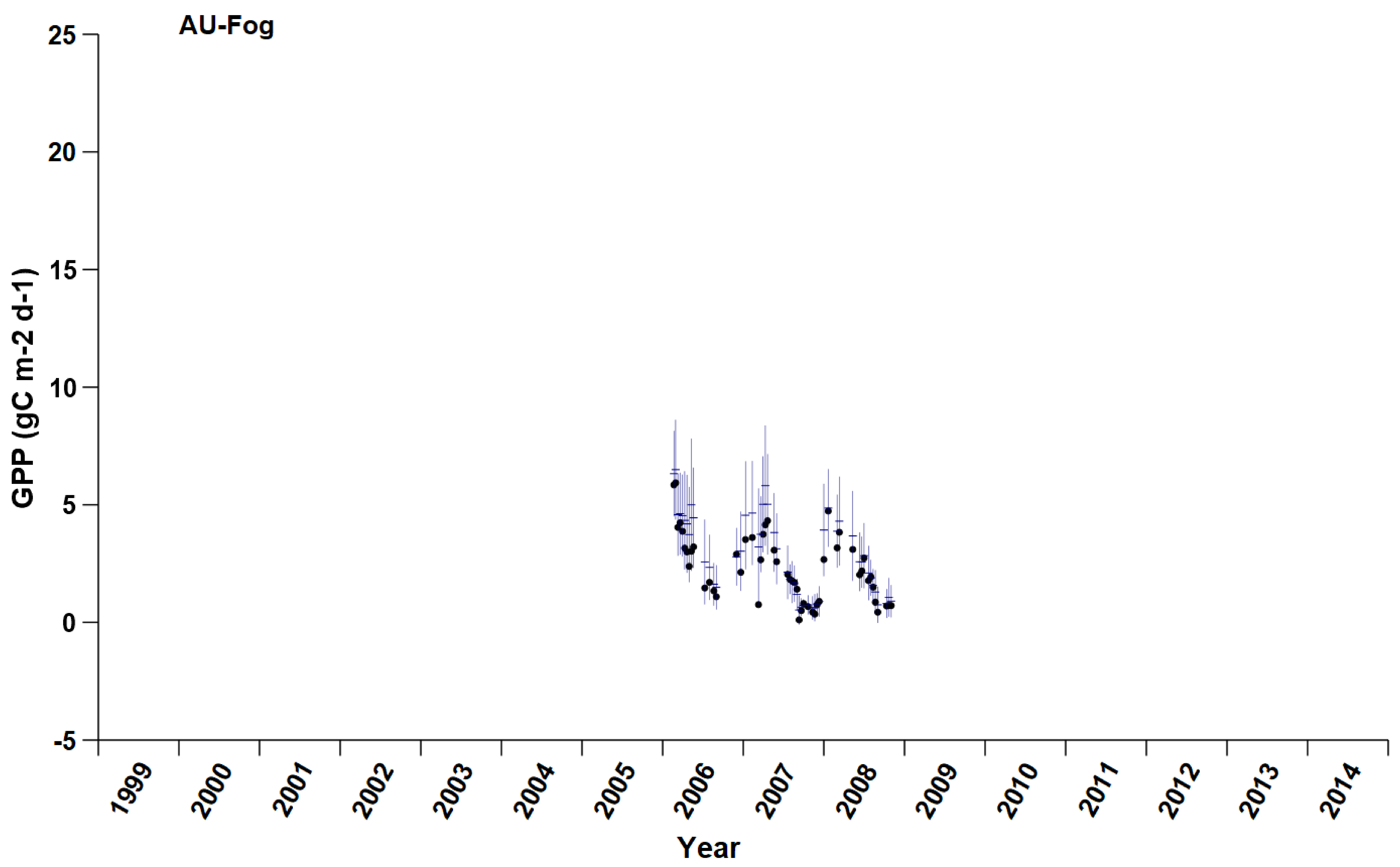
AU-Fog
GPP (gC m-2 d-1)
25
20
15
10
5
0
-5
1999
2000
2001
2002
2003
2004
2005
2006
2007
2008
2009
2010
2011
2012
2013
2014
Year

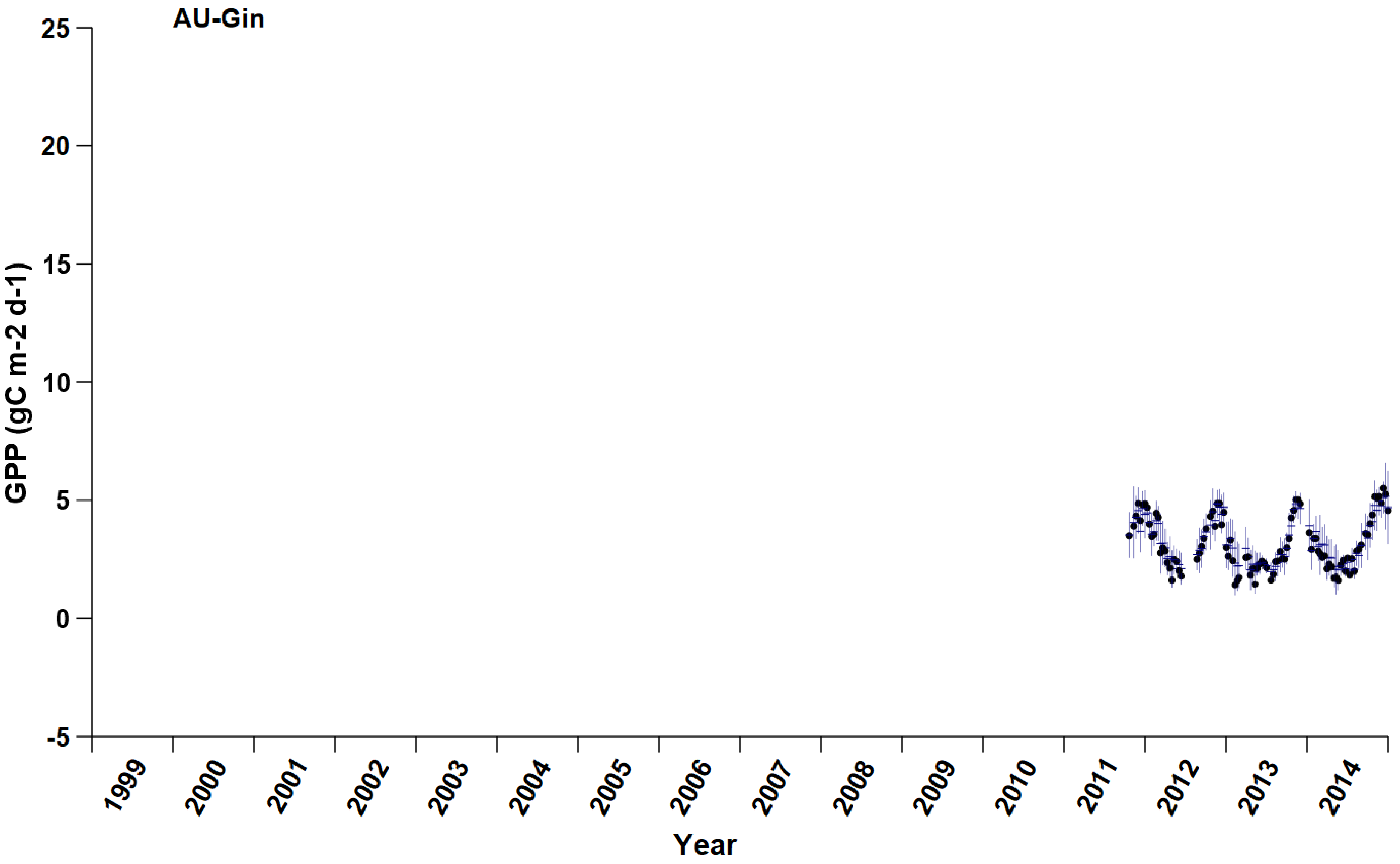
AU-Gin
GPP (gC m-2 d-1)
25
20
15
10
5
0
-5
1999
2000
2001
2002
2003
2004
2005
2006
2007
2008
2009
2010
2011
2012
2013
2014
Year

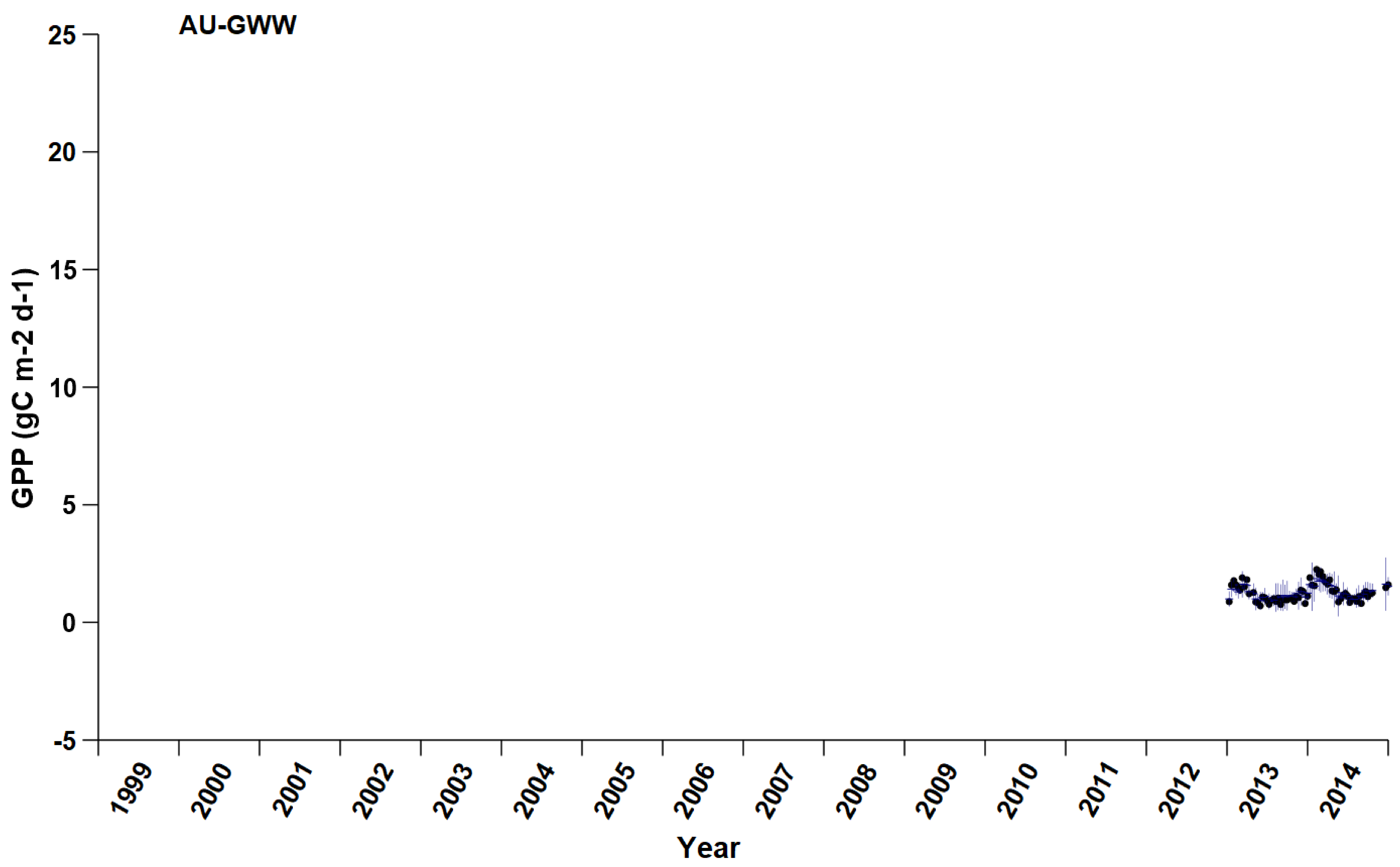

AU-GWW
GPP (gC m-2 d-1)
25
20
15
10
5
0
-5
1999
2000
2001
2002
2003
2004
2005
2006
2007
2008
2009
2010
2011
2012
2013
2014
Year

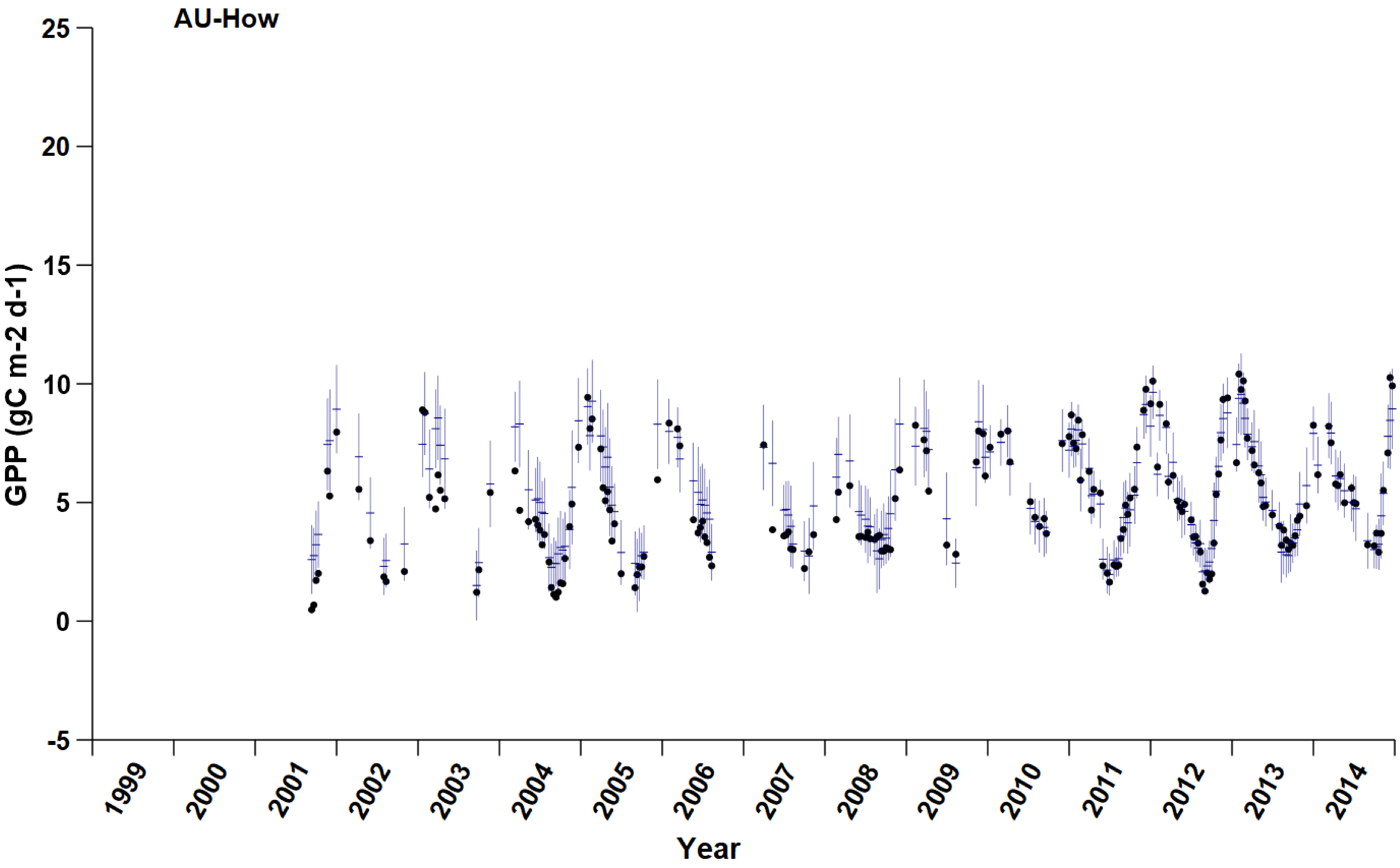
AU-How
GPP (gC m-2 d-1)
25
20
15
10
5
0
-5
1999
2000
2001
2002
2003
2004
2005
2006
2007
2008
2009
2010
2011
2012
2013
2014
Year

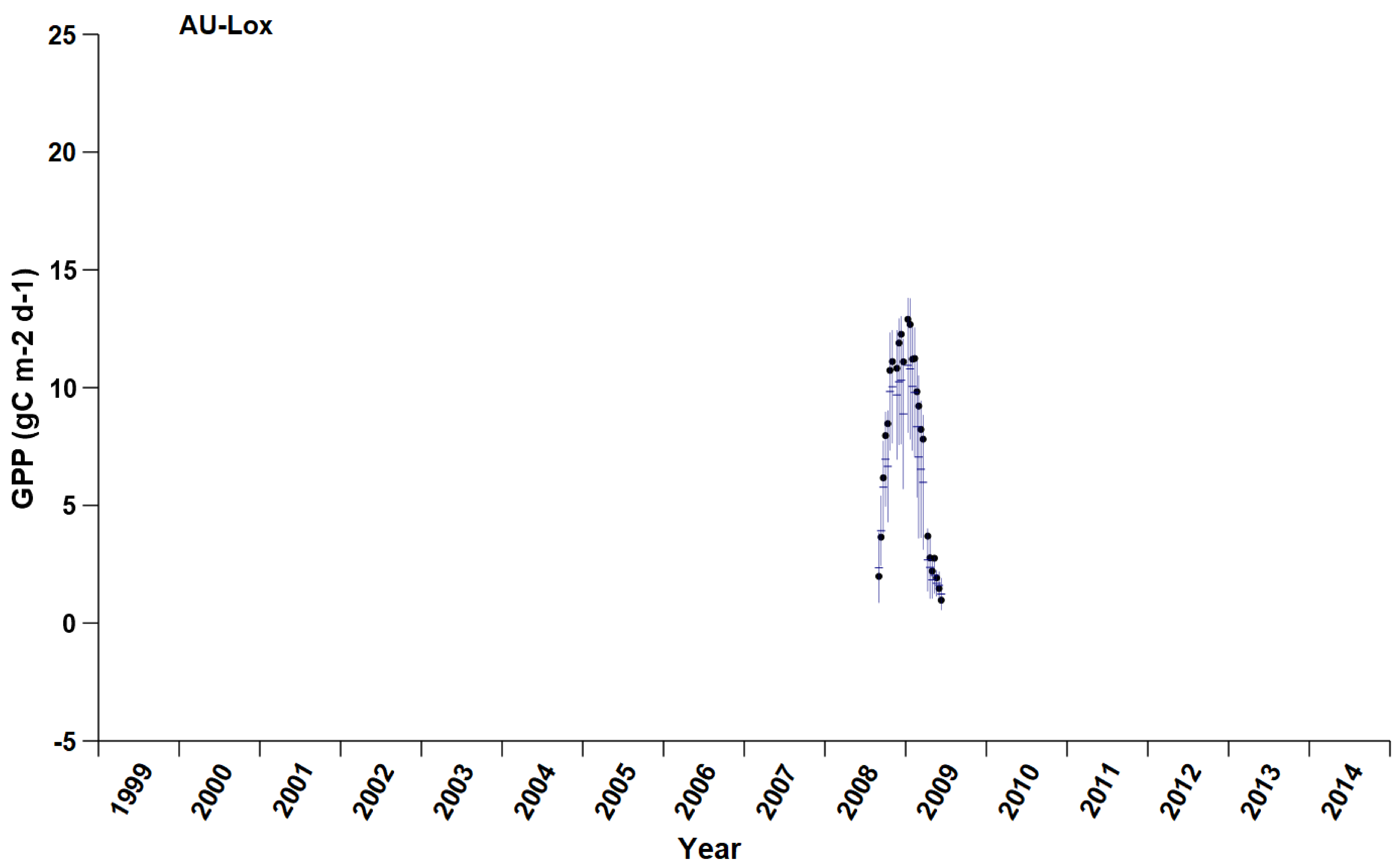
AU-Lox
GPP (gC m-2 d-1)
25
20
15
10
5
0
-5
1999
2000
2001
2002
2003
2004
2005
2006
2007
2008
2009
2010
2011
2012
2013
2014
Year

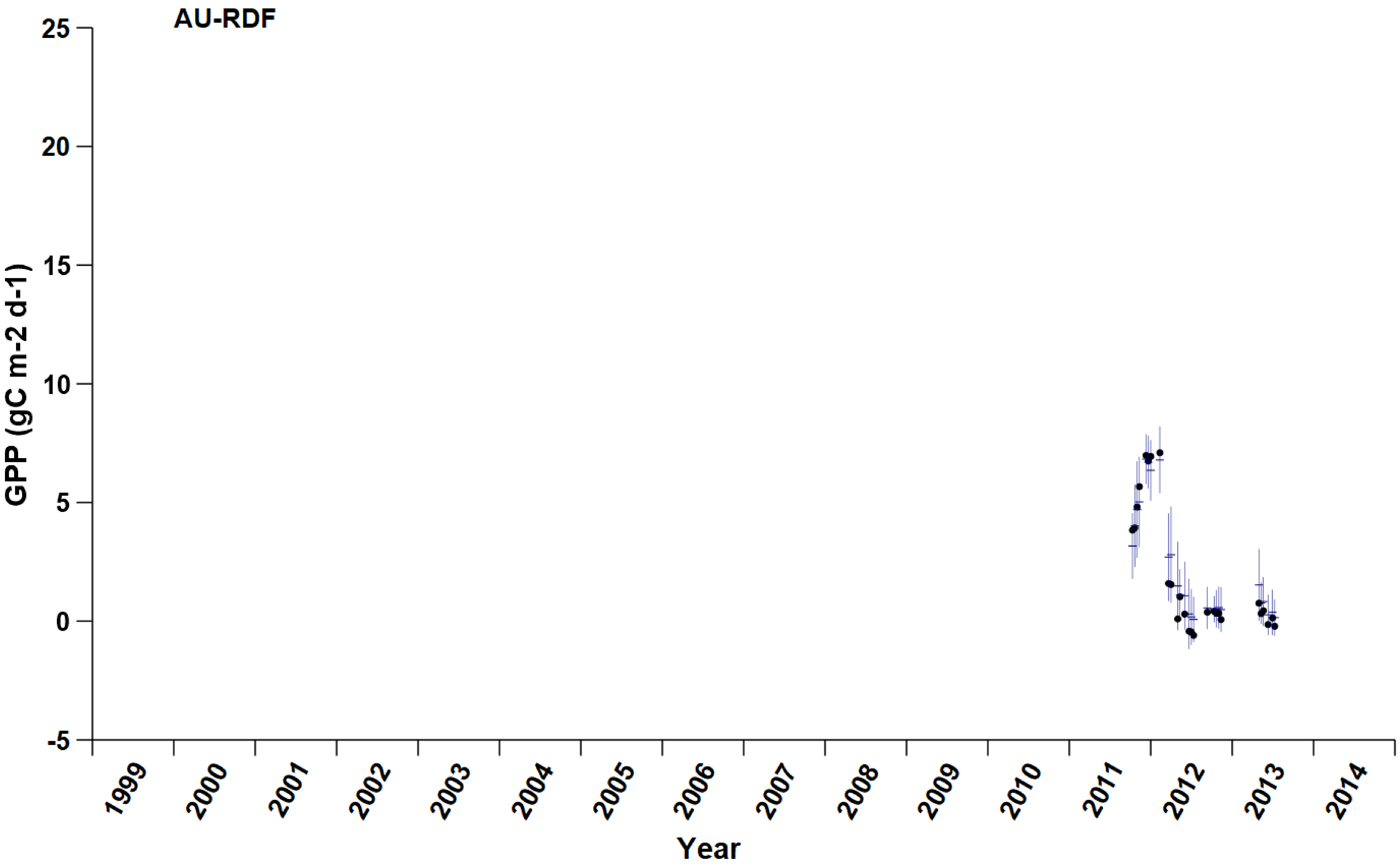

AU-RDF
GPP (gC m-2 d-1)
25
20
15
10
5
0
-5
1999
2000
2001
2002
2003
2004
2005
2006
2007
2008
2009
2010
2011
2012
2013
2014
Year

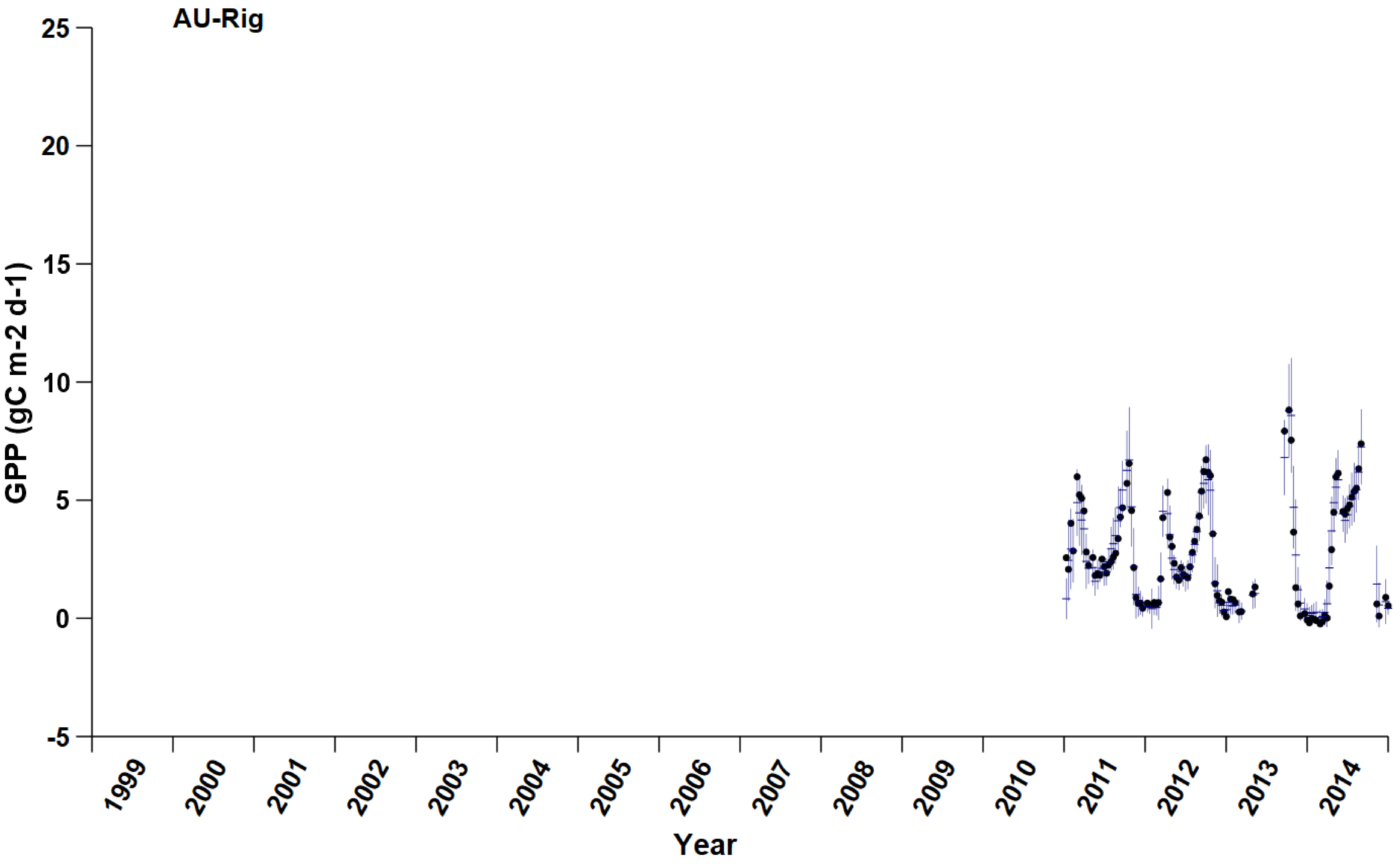

AU-Rig
GPP (gC m-2 d-1)
25
20
15
10
5
0
-5
1999
2000
2001
2002
2003
2004
2005
2006
2007
2008
2009
2010
2011
2012
2013
2014
Year

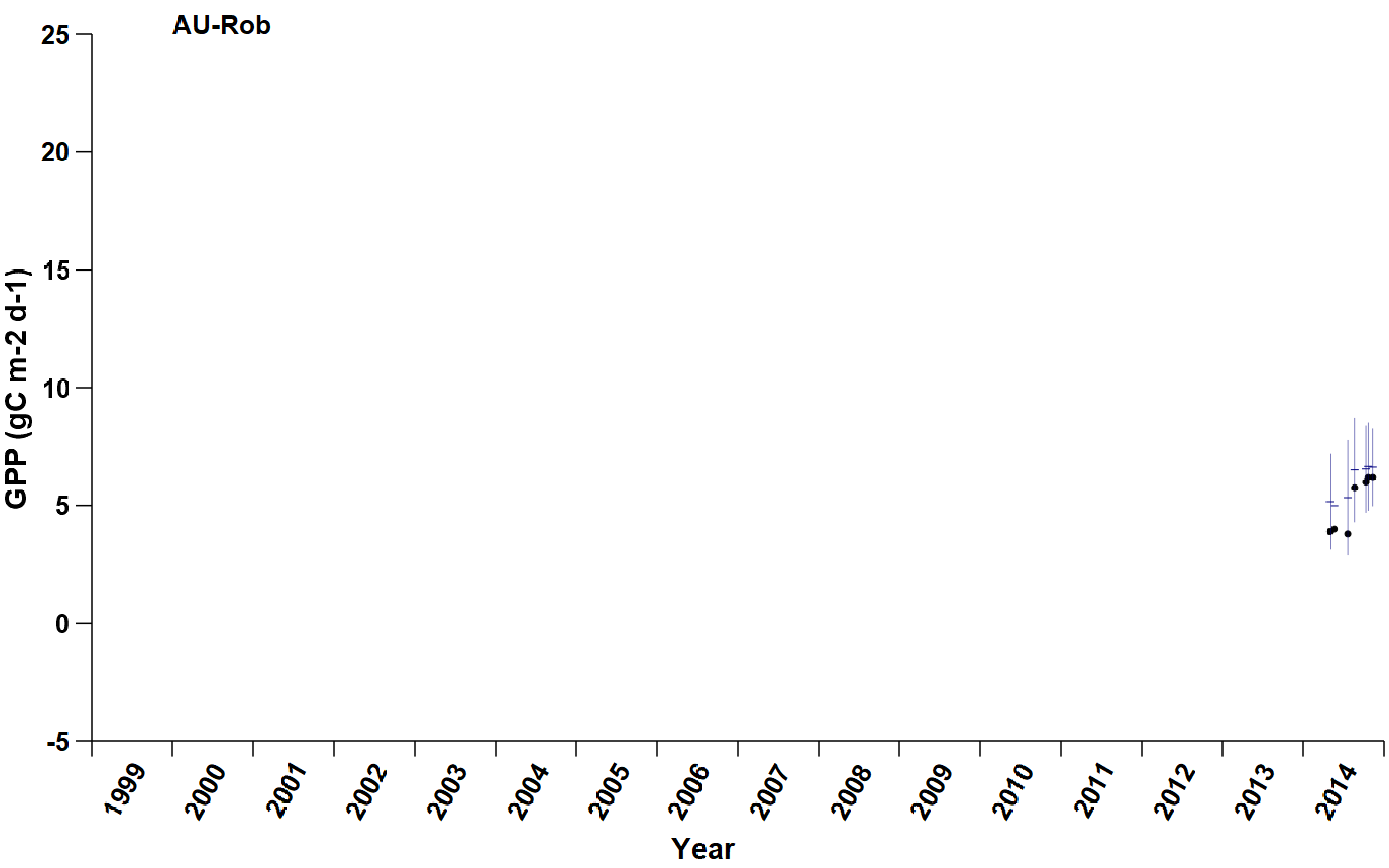

AU-Rob
GPP (gC m-2 d-1)
25
20
15
10
5
0
-5
1999
2000
2001
2002
2003
2004
2005
2006
2007
2008
2009
2010
2011
2012
2013
2014
Year

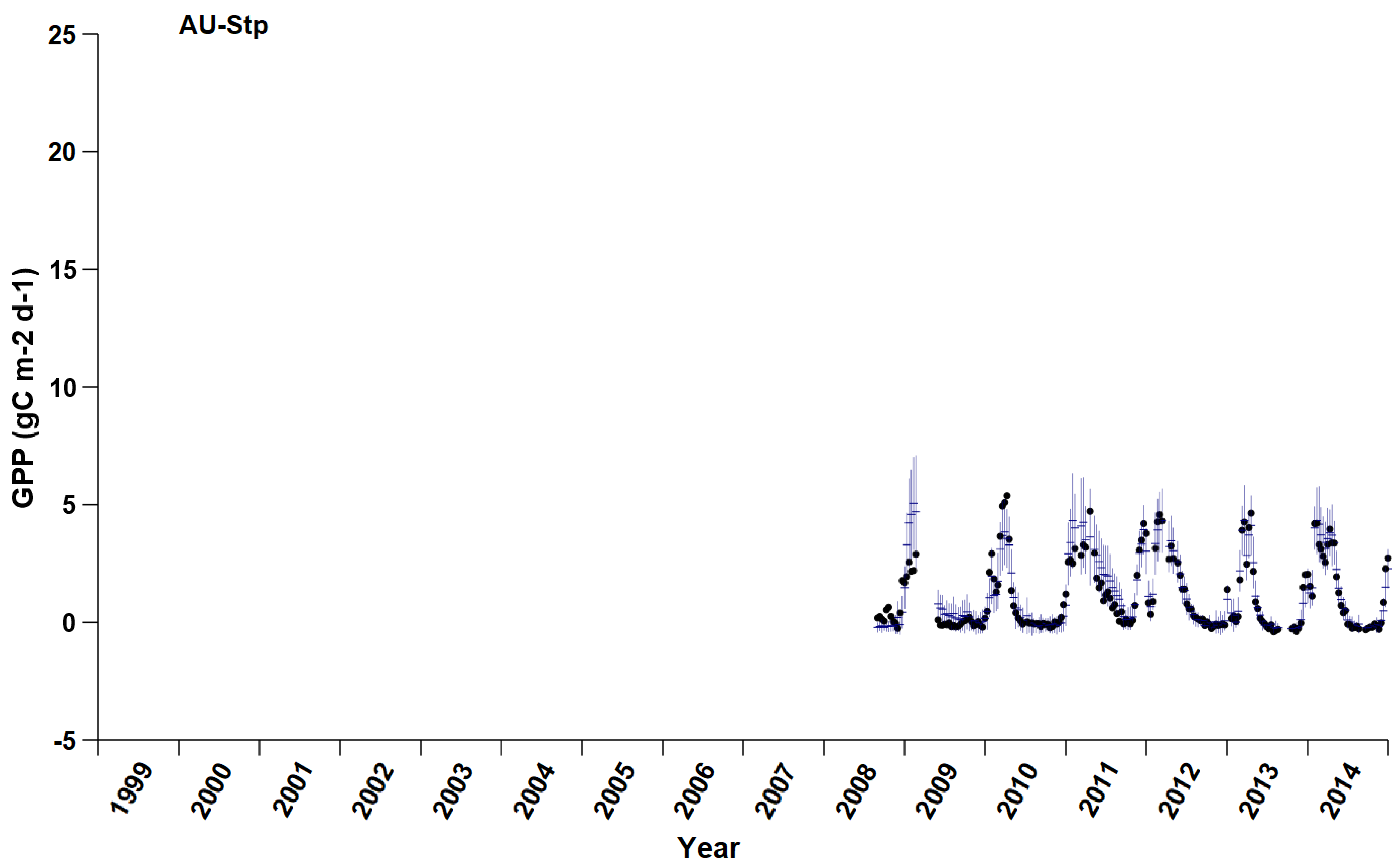

AU-Stp
GPP (gC m-2 d-1)
25
20
15
10
5
0
-5
1999
2000
2001
2002
2003
2004
2005
2006
2007
2008
2009
2010
2011
2012
2013
2014
Year

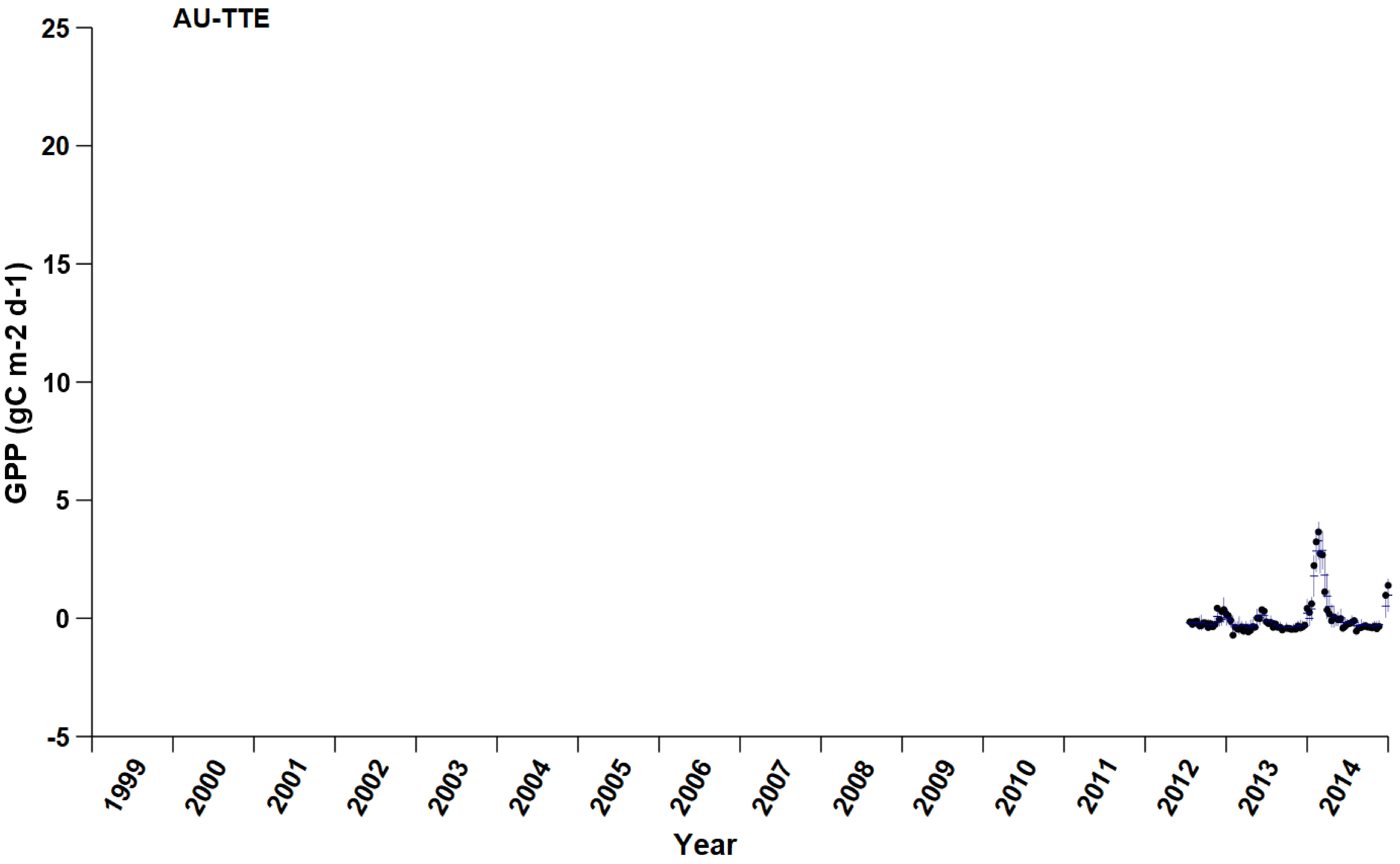
AU-TTE
GPP (gC m-2 d-1)
25
20
15
10
5
0
-5
1999
2000
2001
2002
2003
2004
2005
2006
2007
2008
2009
2010
2011
2012
2013
2014
Year

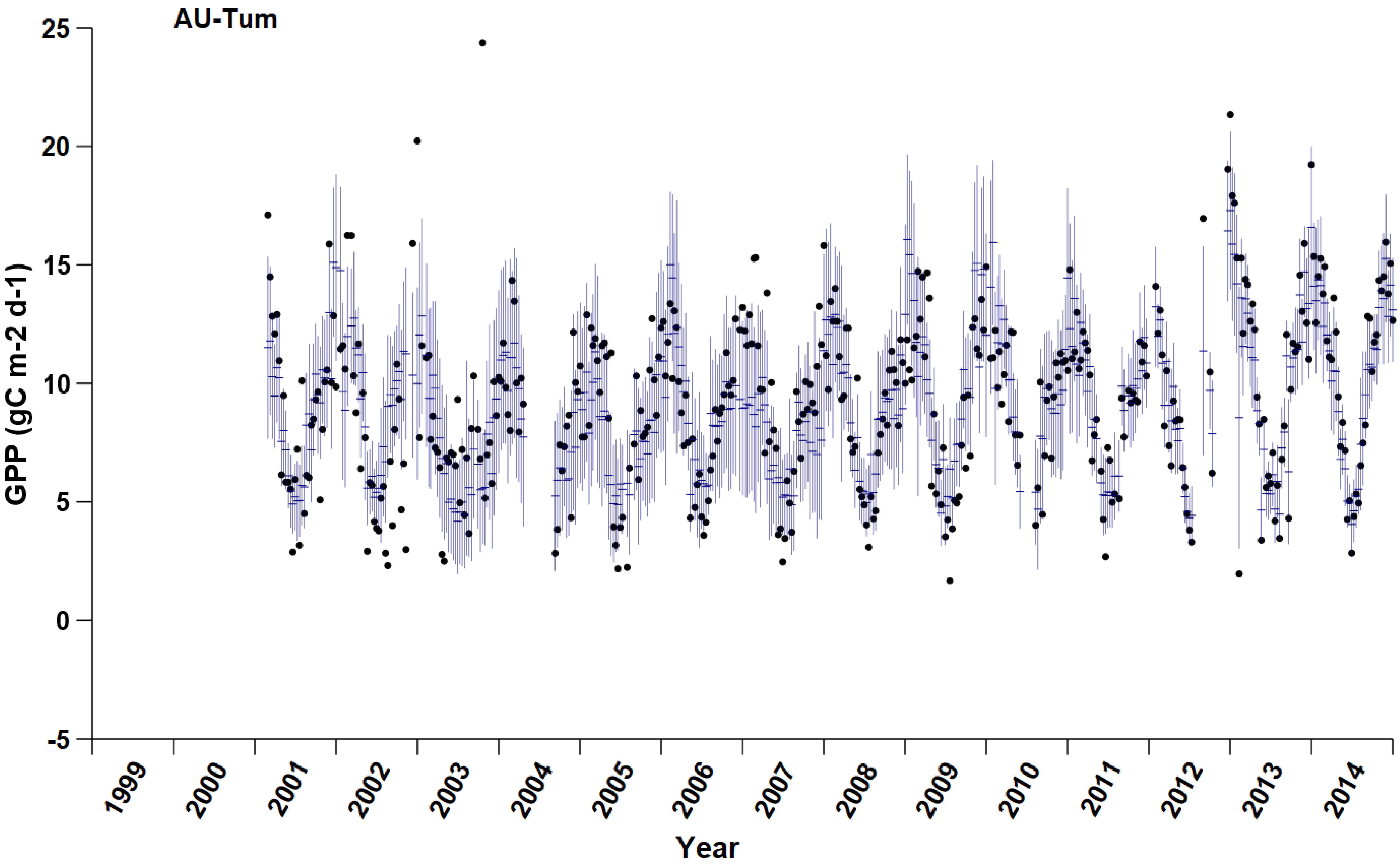
AU-Tum
GPP (gC m-2 d-1)
25
20
15
10
5
0
-5
1999
2000
2001
2002
2003
2004
2005
2006
2007
2008
2009
2010
2011
2012
2013
2014
Year

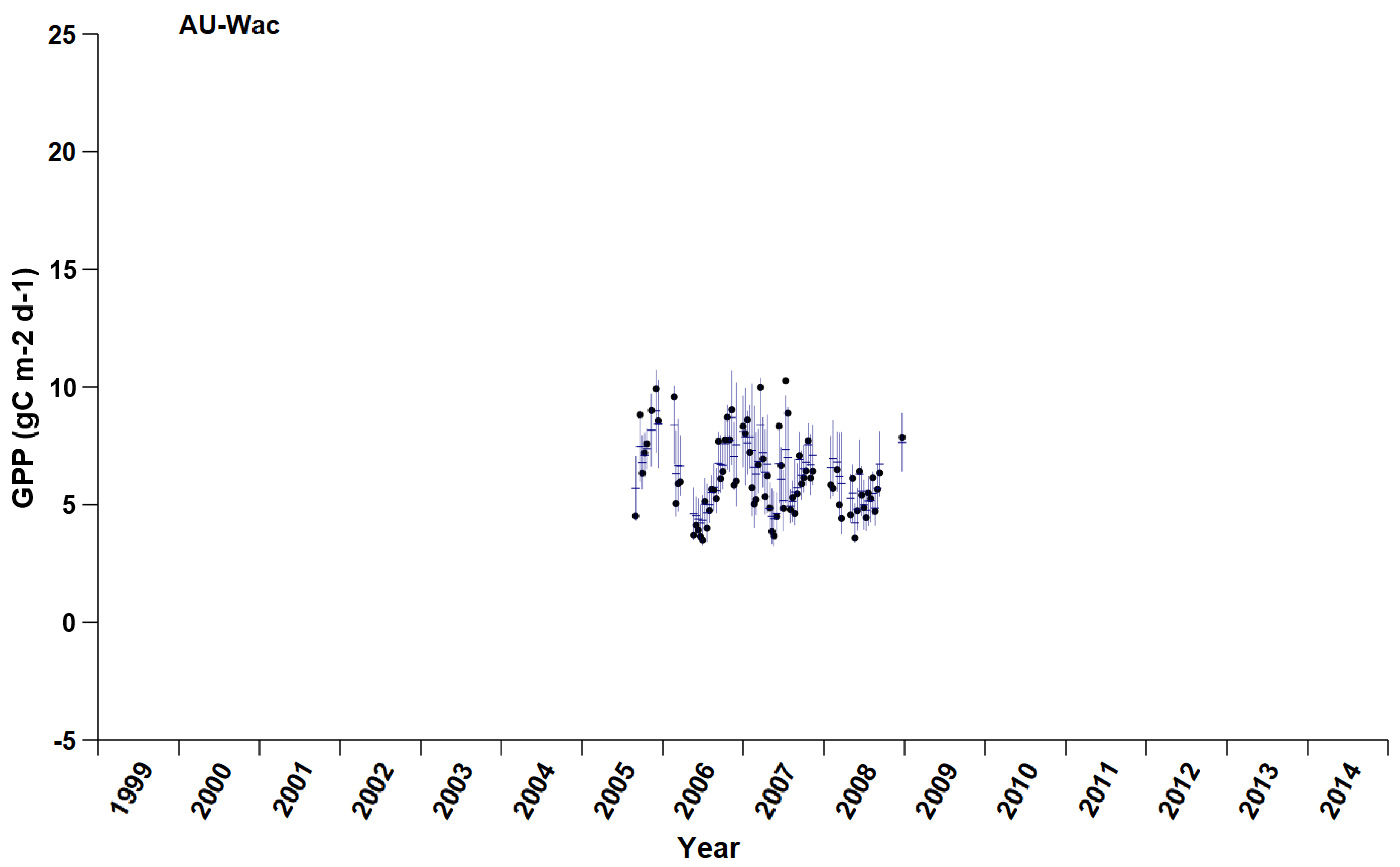

AU-Wac
GPP (gC m-2 d-1)
25
20
15
10
5
0
-5
1999
2000
2001
2002
2003
2004
2005
2006
2007
2008
2009
2010
2011
2012
2013
2014
Year

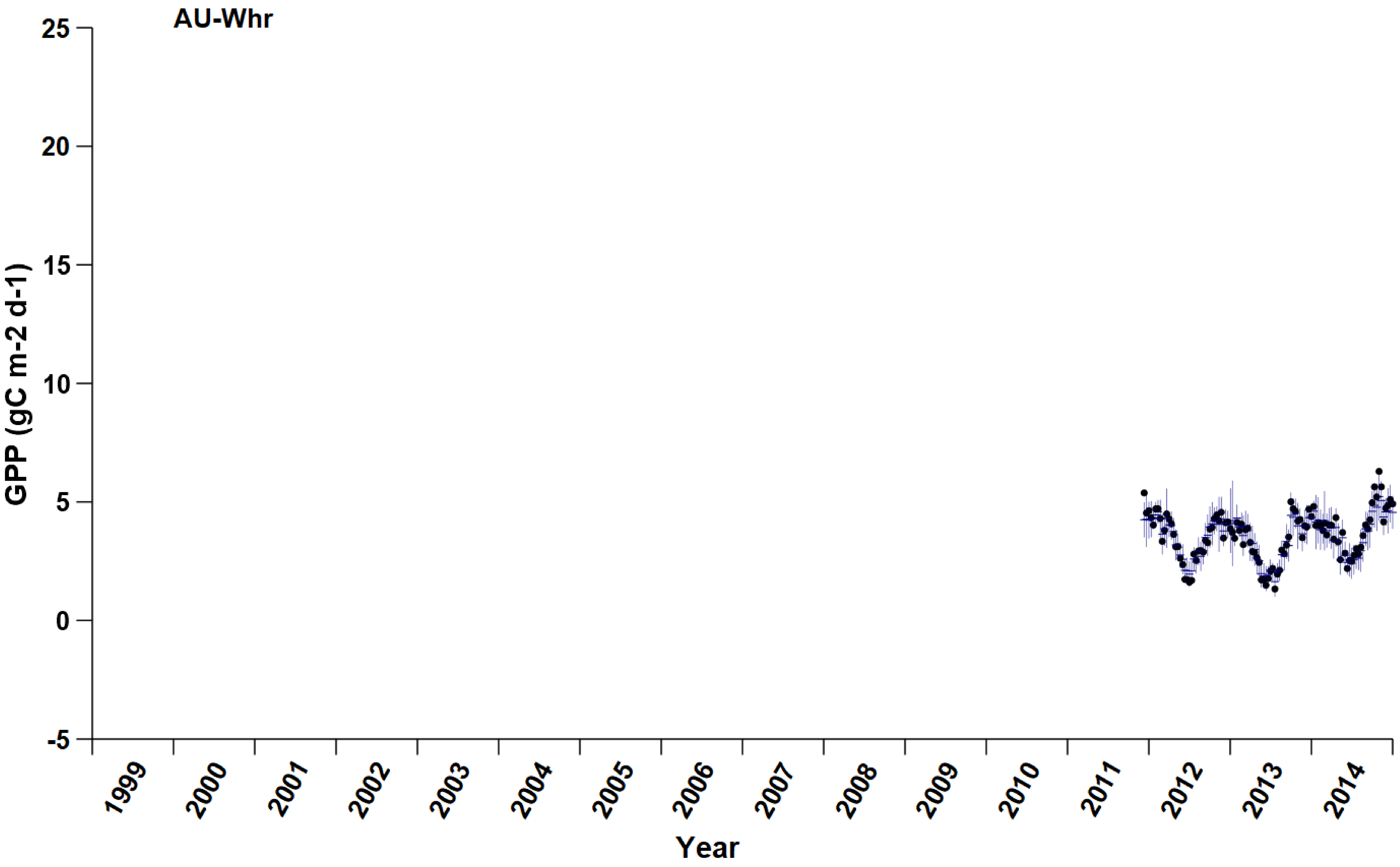
AU-Whr
GPP (gC m-2 d-1)
25
20
15
10
5
0
-5
1999
2000
2001
2002
2003
2004
2005
2006
2007
2008
2009
2010
2011
2012
2013
2014
Year

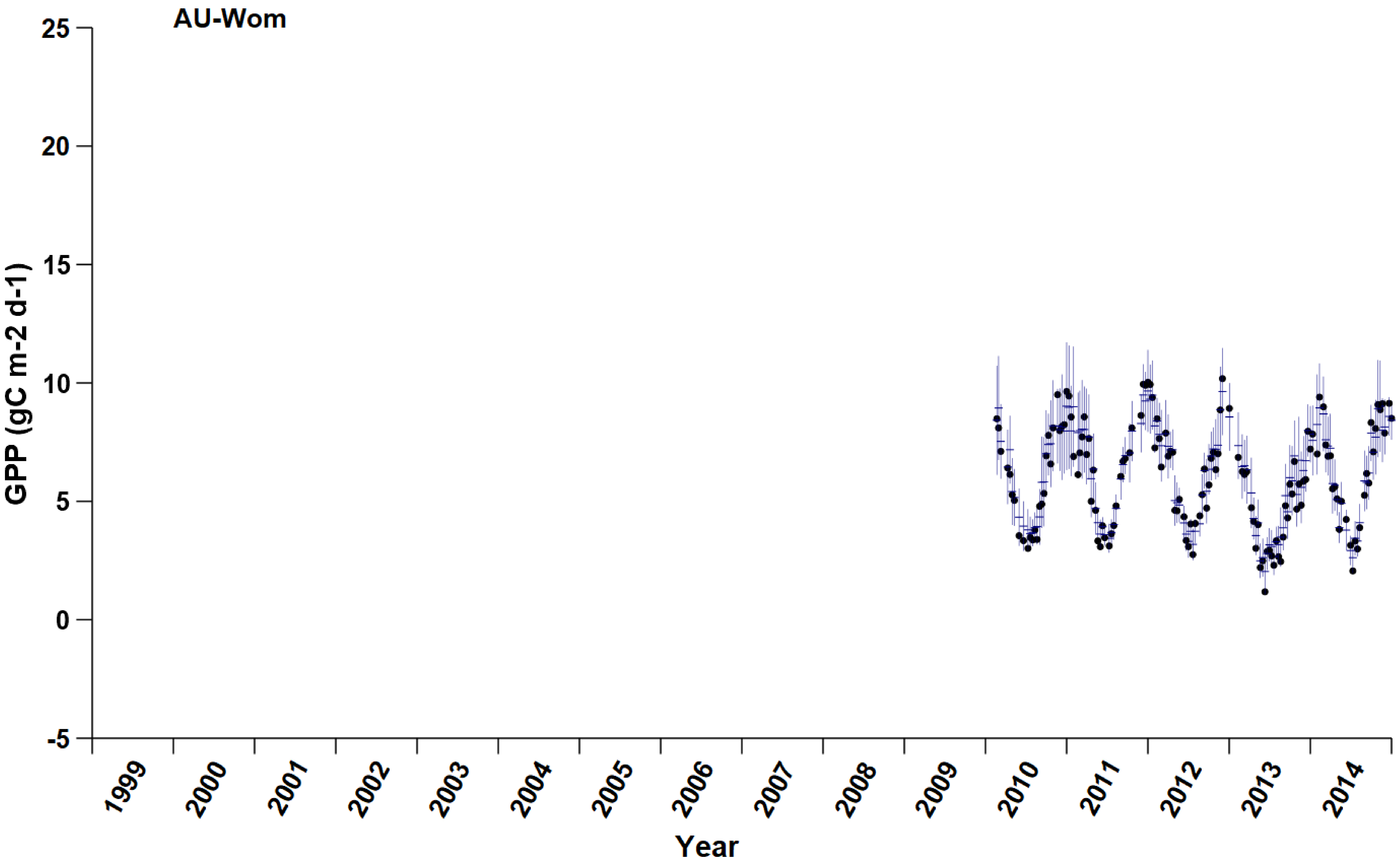
AU-Wom
GPP (gC m-2 d-1)
25
20
15
10
5
0
-5
1999
2000
2001
2002
2003
2004
2005
2006
2007
2008
2009
2010
2011
2012
2013
2014
Year

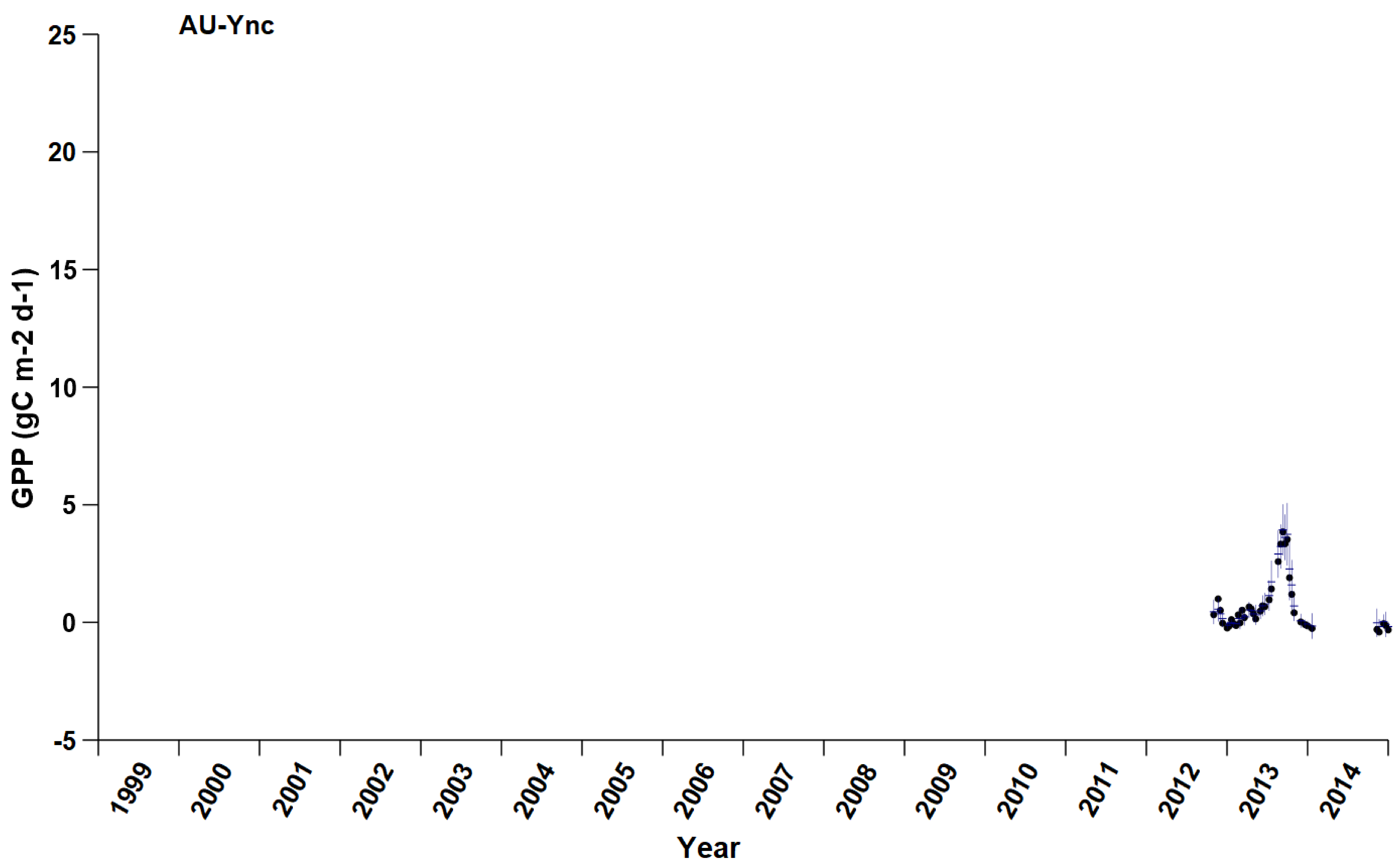

AU-Ync
GPP (gC m-2 d-1)
25
20
15
10
5
0
-5
1999
2000
2001
2002
2003
2004
2005
2006
2007
2008
2009
2010
2011
2012
2013
2014
Year

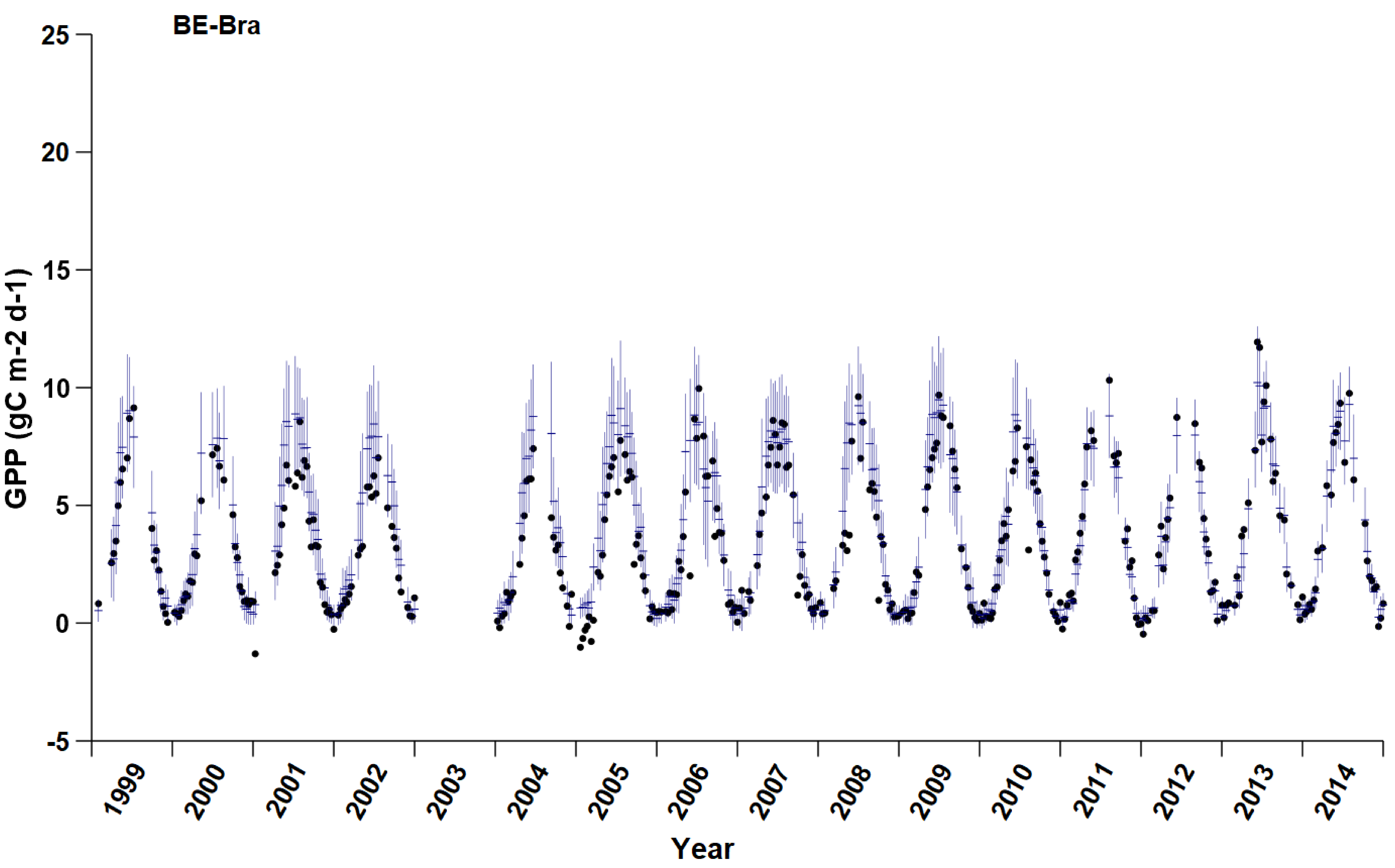

BE-Bra
GPP (gC m-2 d-1)
25
20
15
10
5
0
-5
1999
2000
2001
2002
2003
2004
2005
2006
2007
2008
2009
2010
2011
2012
2013
2014
Year

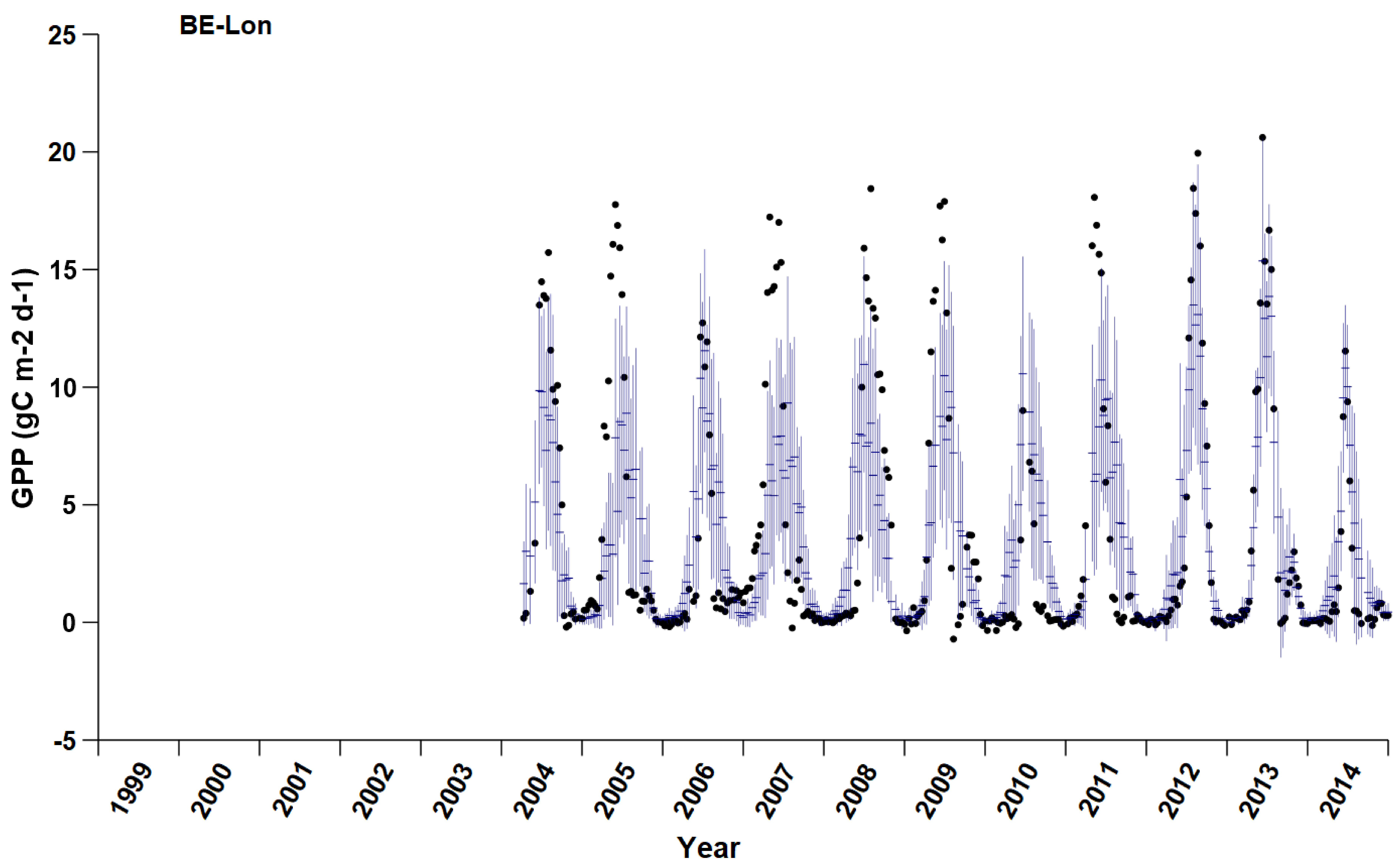
BE-Lon
GPP (gC m-2 d-1)
25
20
15
10
5
0
-5
1999
2000
2001
2002
2003
2004
2005
2006
2007
2008
2009
2010
2011
2012
2013
2014
Year

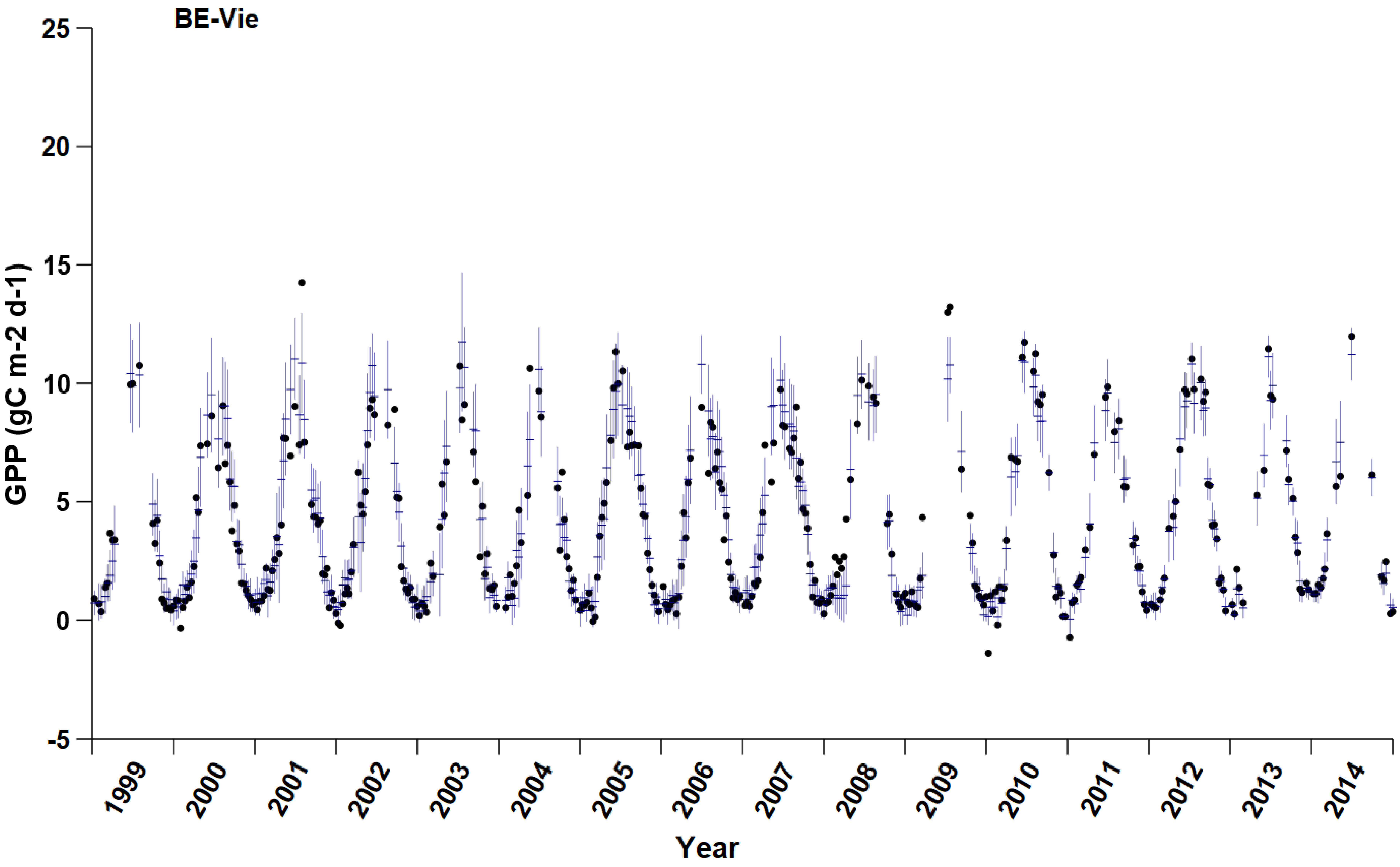

BE-Vie
GPP (gC m-2 d-1)
25
20
15
10
5
0
-5
1999
2000
2001
2002
2003
2004
2005
2006
2007
2008
2009
2010
2011
2012
2013
2014
Year

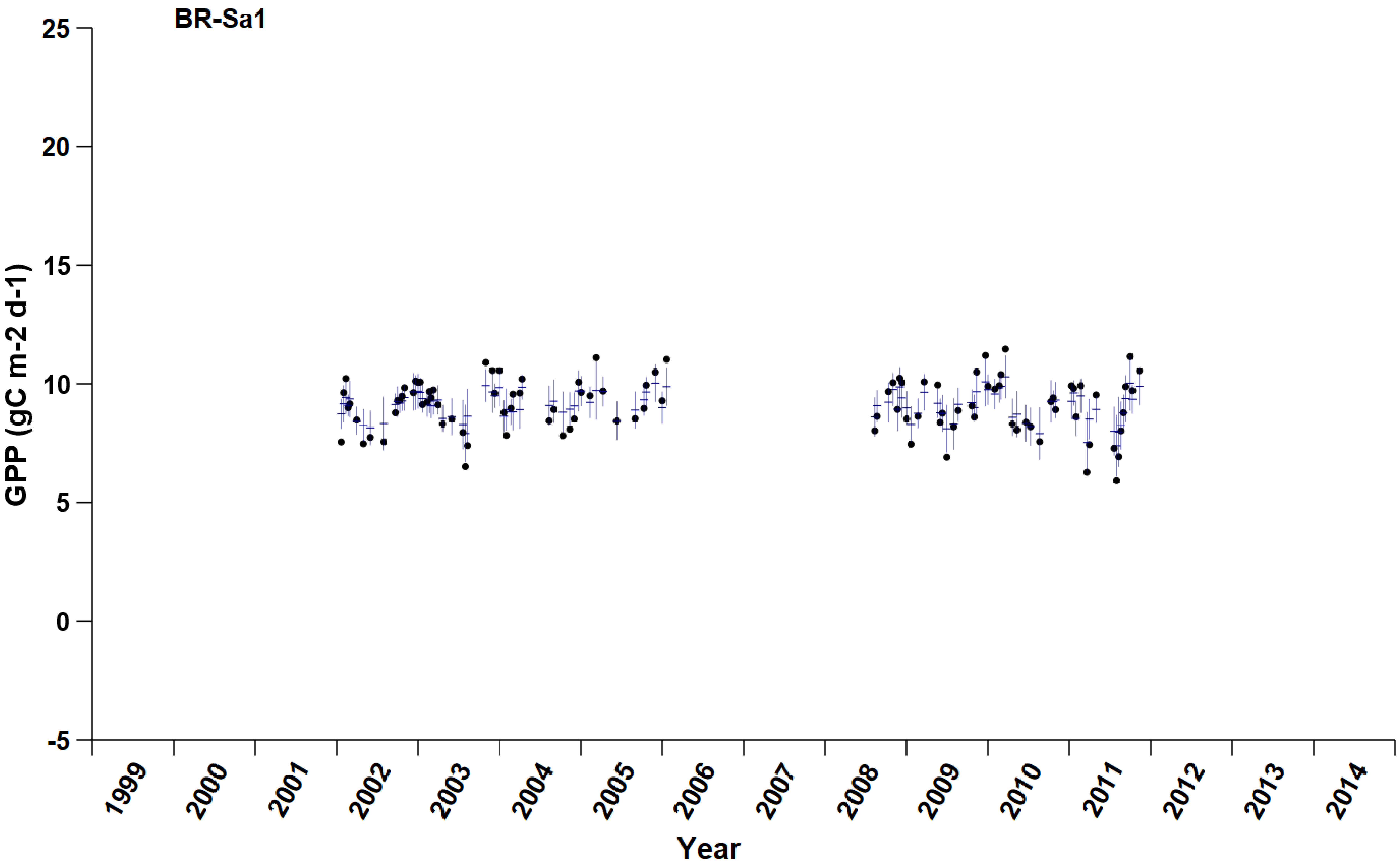
BR-Sa1
GPP (gC m-2 d-1)
25
20
15
10
5
0
-5
1999
2000
2001
2002
2003
2004
2005
2006
2007
2008
2009
2010
2011
2012
2013
2014
Year

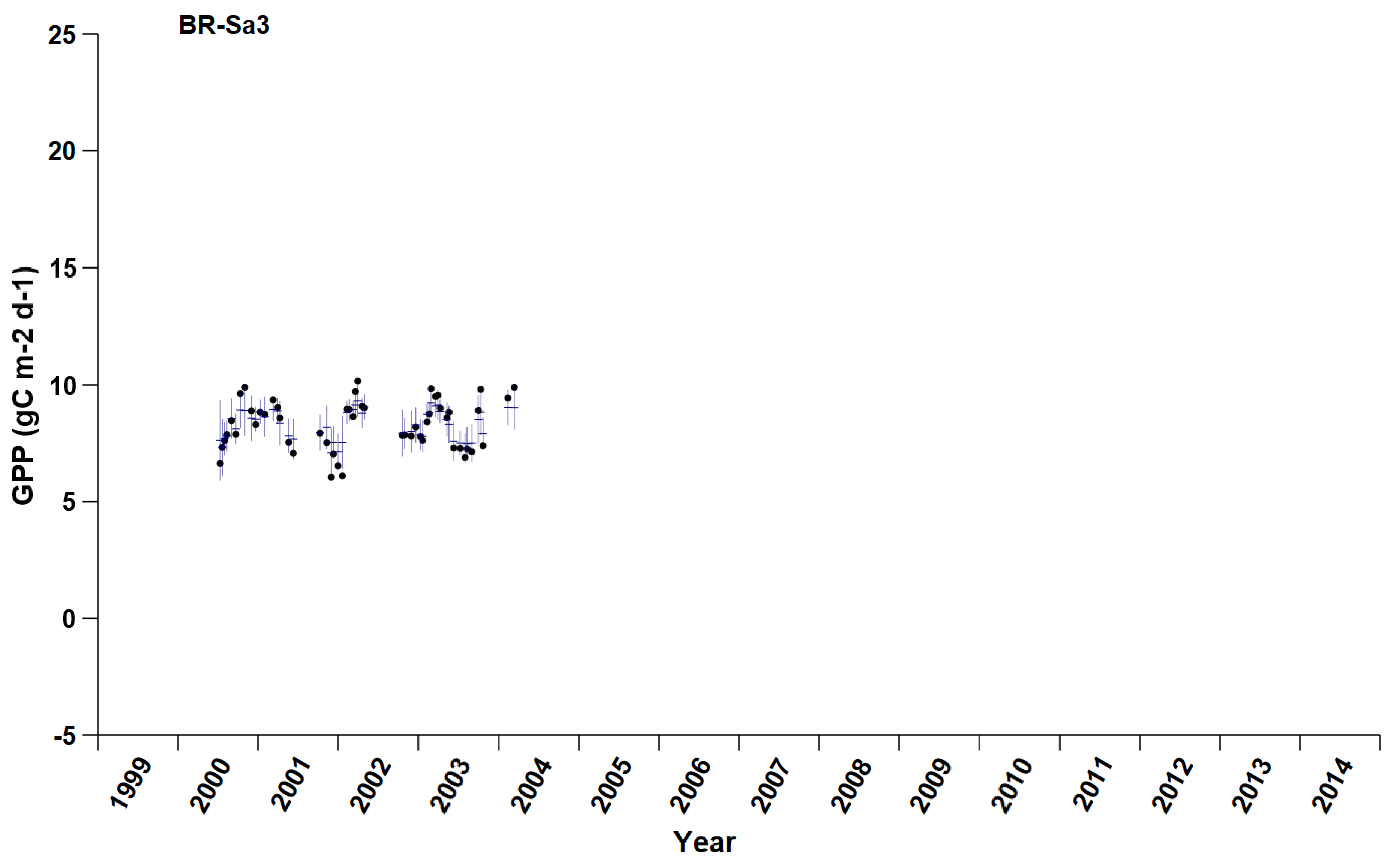
BR-Sa3
GPP (gC m-2 d-1)
25
20
15
10
5
0
-5
1999
2000
2001
2002
2003
2004
2005
2006
2007
2008
2009
2010
2011
2012
2013
2014
Year

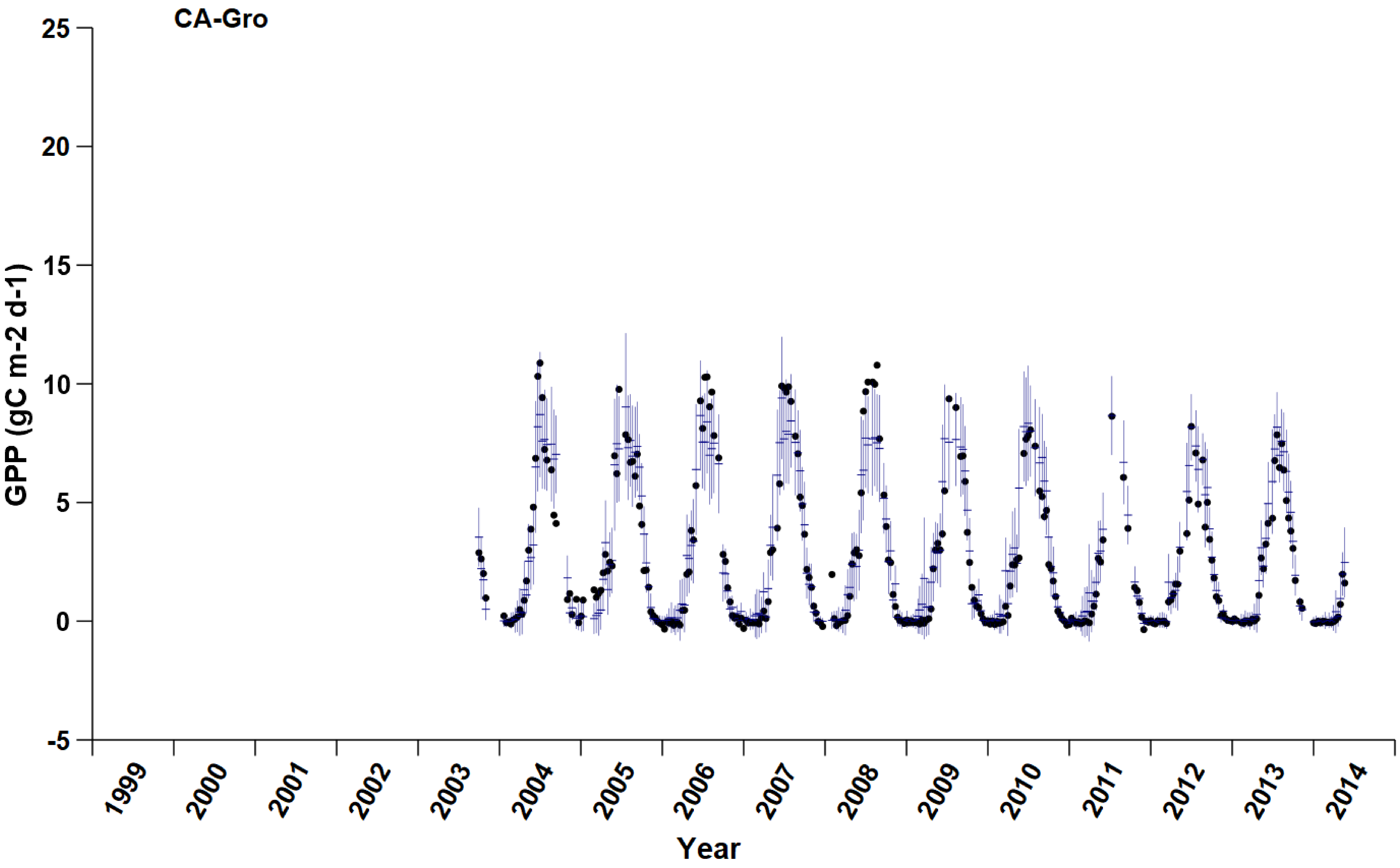
CA-Gro
GPP (gC m-2 d-1)
25
20
15
10
5
0
-5
1999
2000
2001
2002
2003
2004
2005
2006
2007
2008
2009
2010
2011
2012
2013
2014
Year

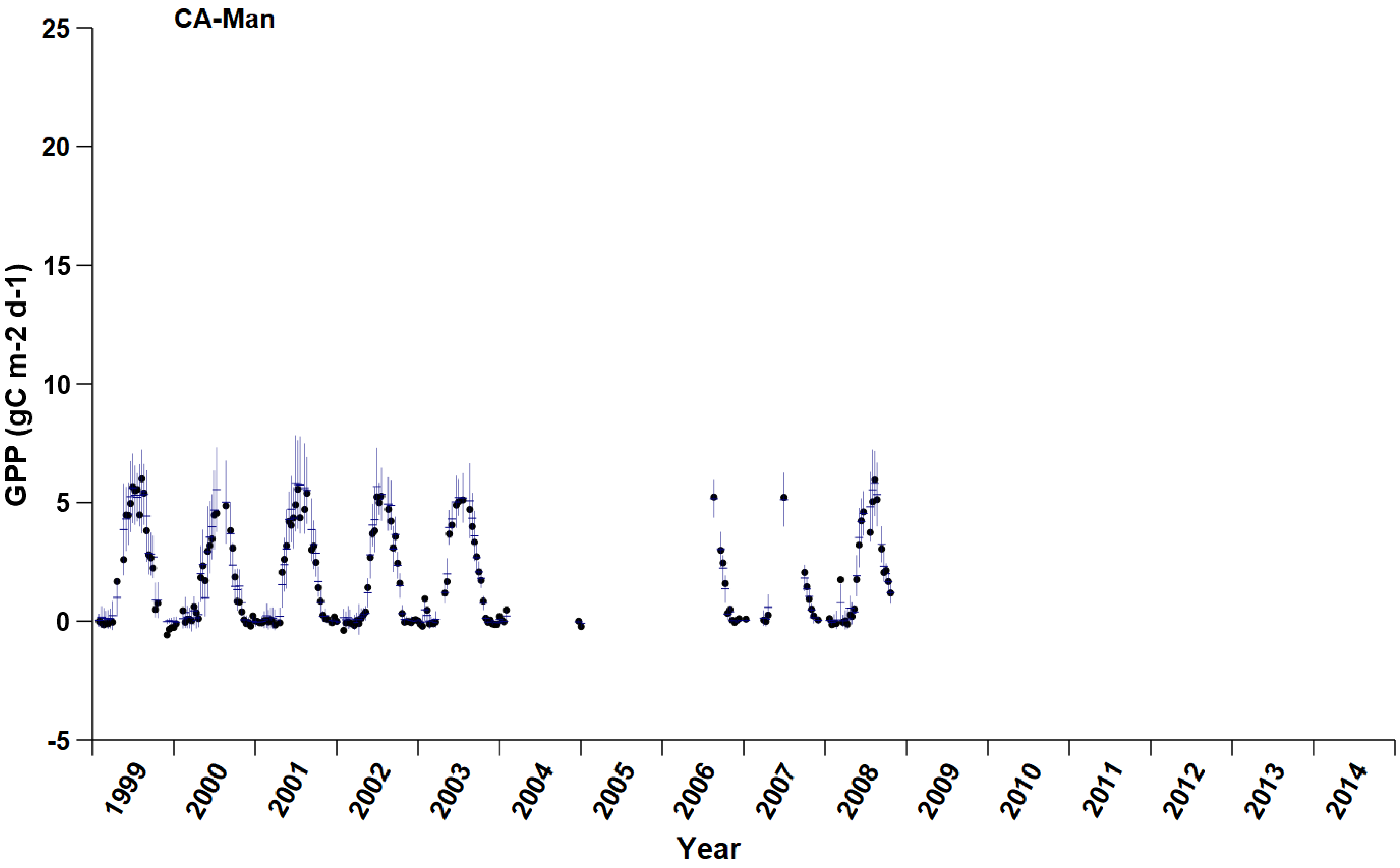

CA-Man
GPP (gC m-2 d-1)
25
20
15
10
5
0
-5
1999
2000
2001
2002
2003
2004
2005
2006
2007
2008
2009
2010
2011
2012
2013
2014
Year

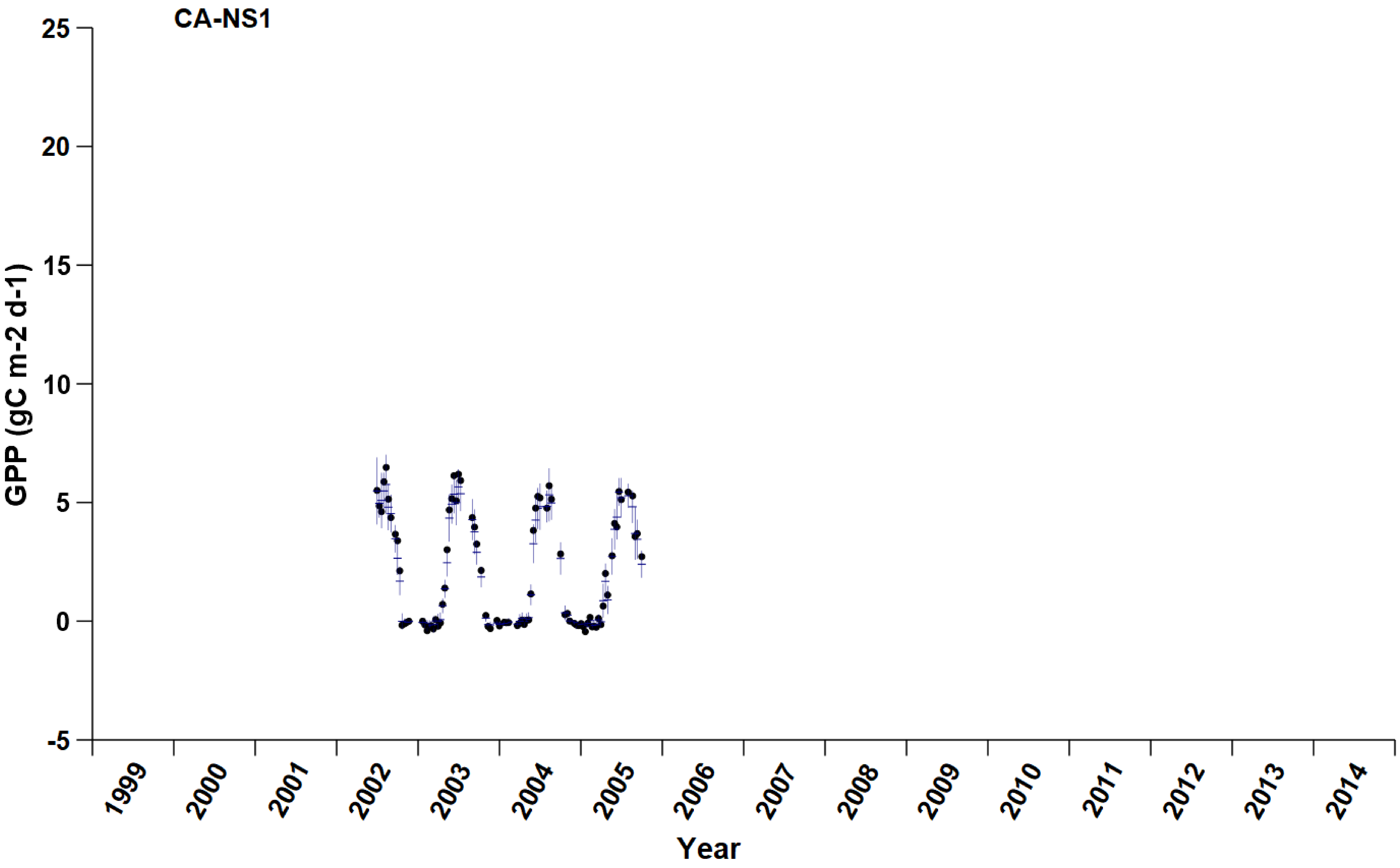

CA-NS1
GPP (gC m-2 d-1)
25
20
15
10
5
0
-5
1999
2000
2001
2002
2003
2004
2005
2006
2007
2008
2009
2010
2011
2012
2013
2014
Year

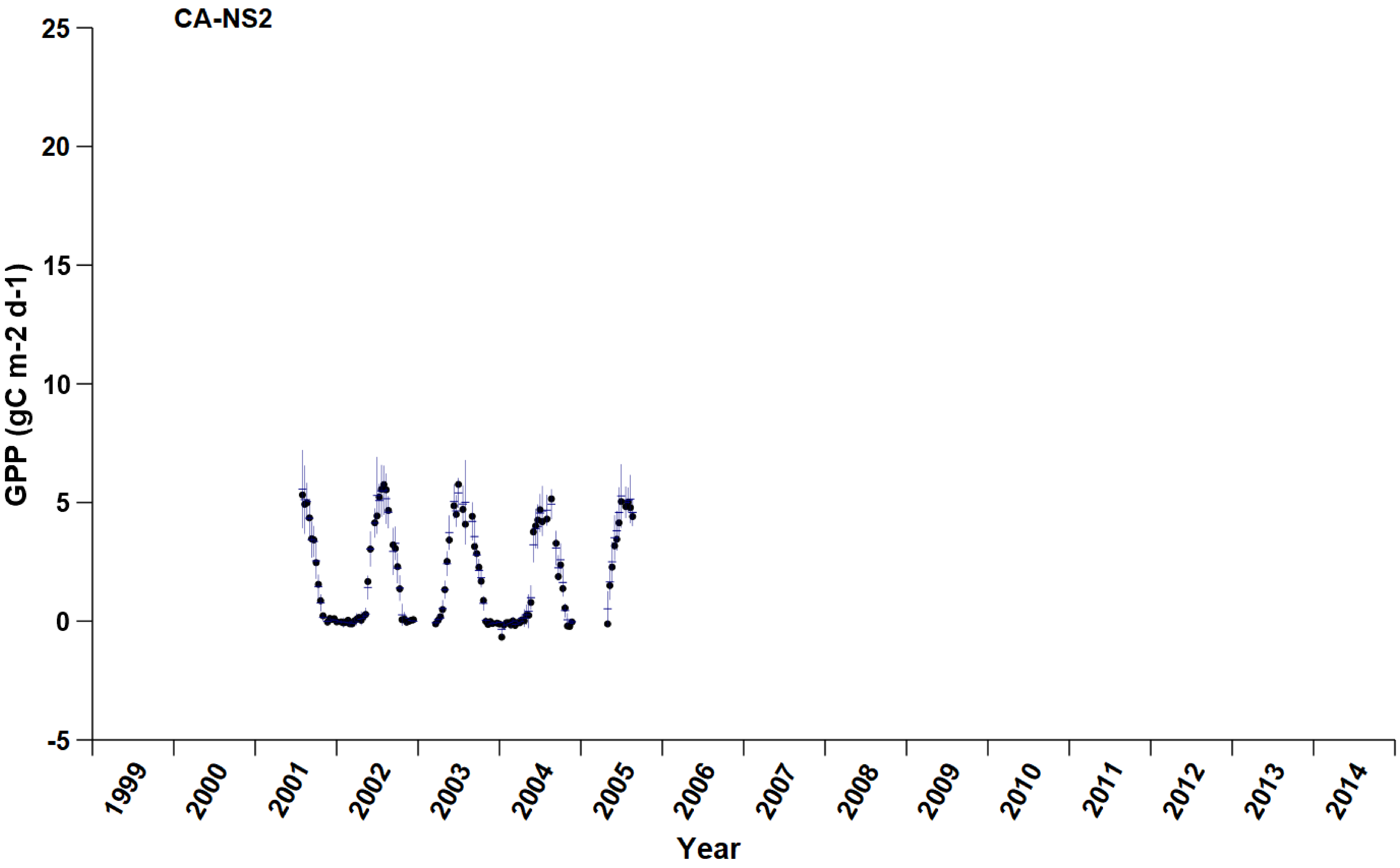

CA-NS2
GPP (gC m-2 d-1)
25
20
15
10
5
0
-5
1999
2000
2001
2002
2003
2004
2005
2006
2007
2008
2009
2010
2011
2012
2013
2014
Year

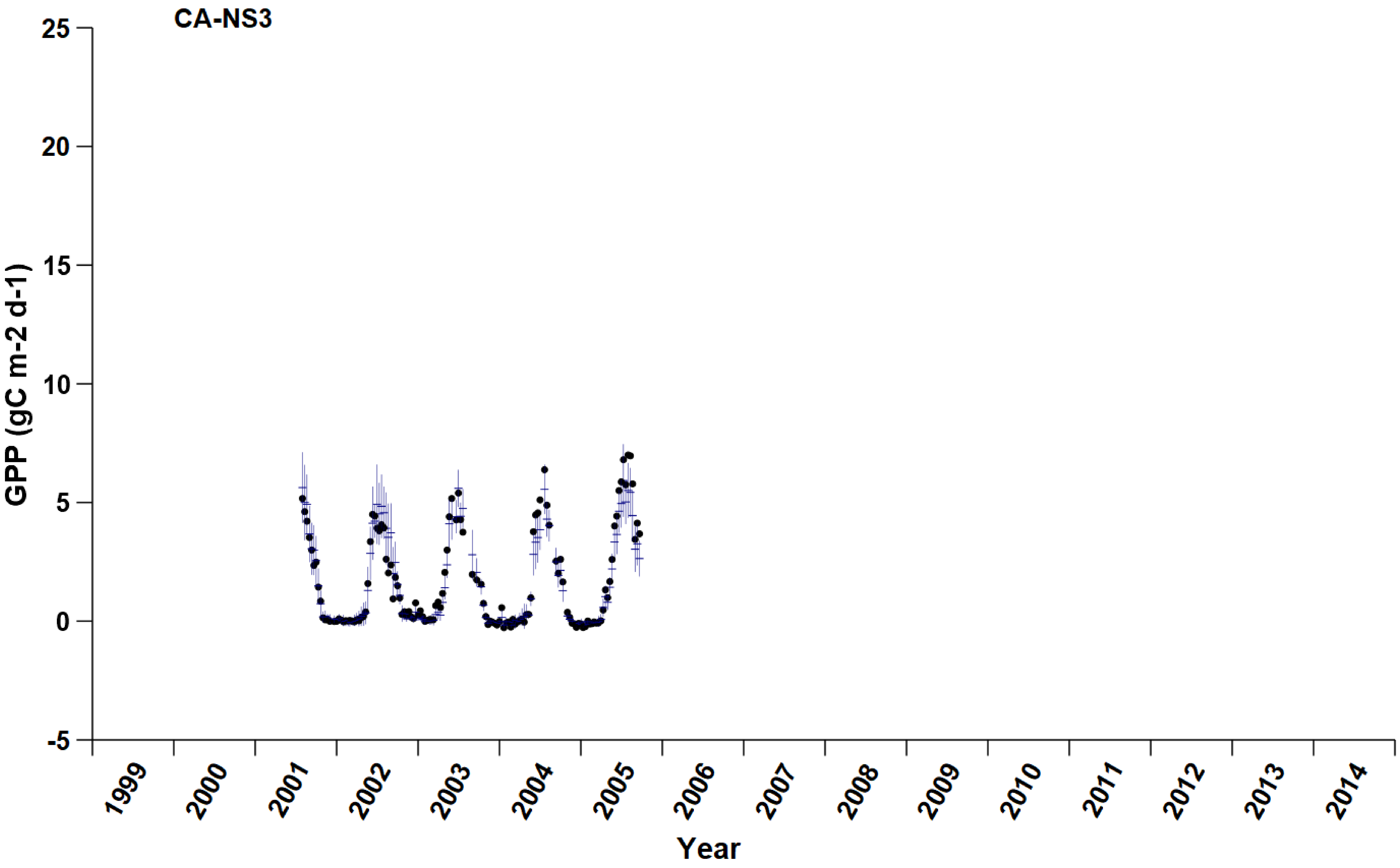

CA-NS3
GPP (gC m-2 d-1)
25
20
15
10
5
0
-5
1999
2000
2001
2002
2003
2004
2005
2006
2007
2008
2009
2010
2011
2012
2013
2014
Year

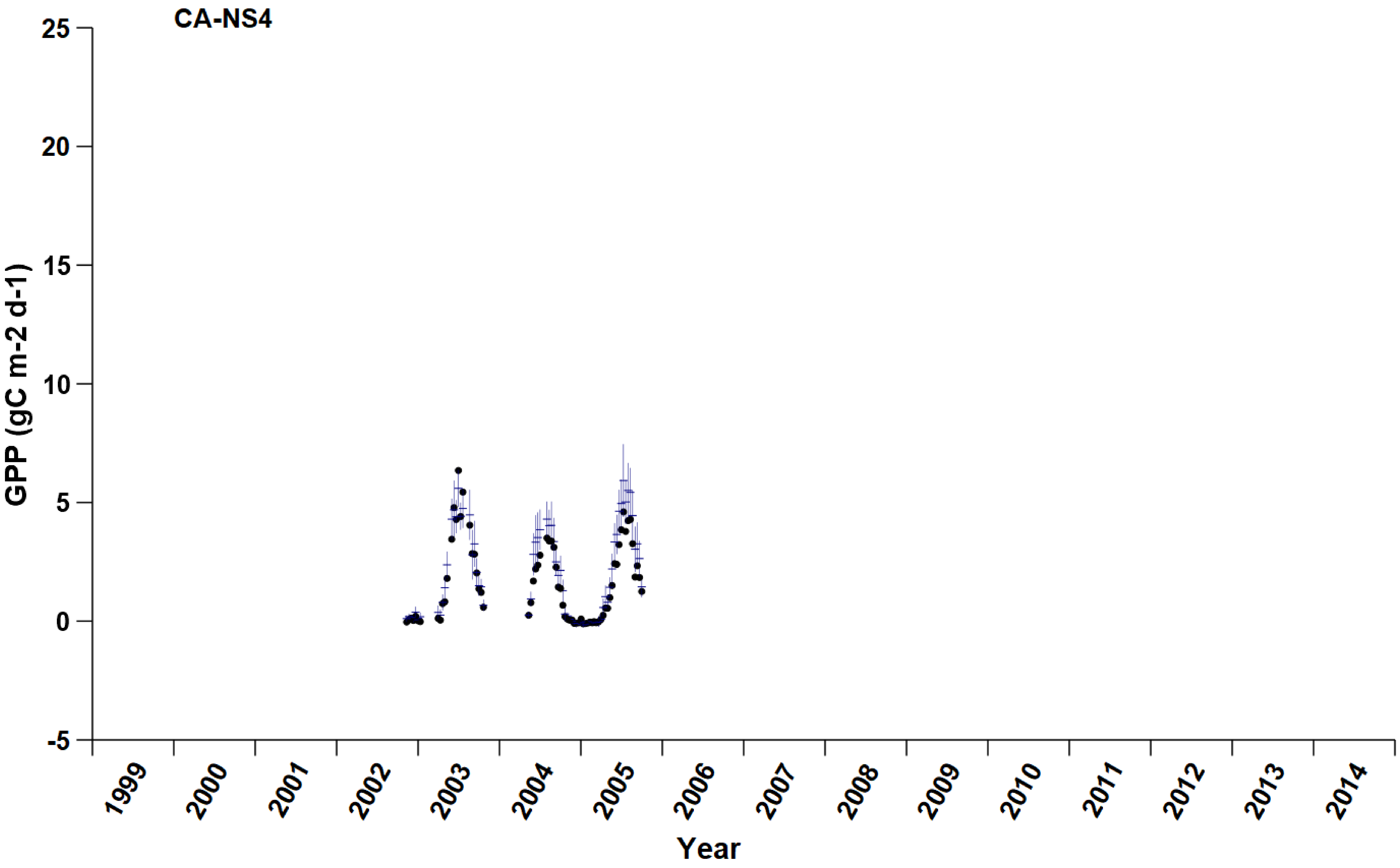
CA-NS4
GPP (gC m-2 d-1)
25
20
15
10
5
0
-5
1999
2000
2001
2002
2003
2004
2005
2006
2007
2008
2009
2010
2011
2012
2013
2014
Year

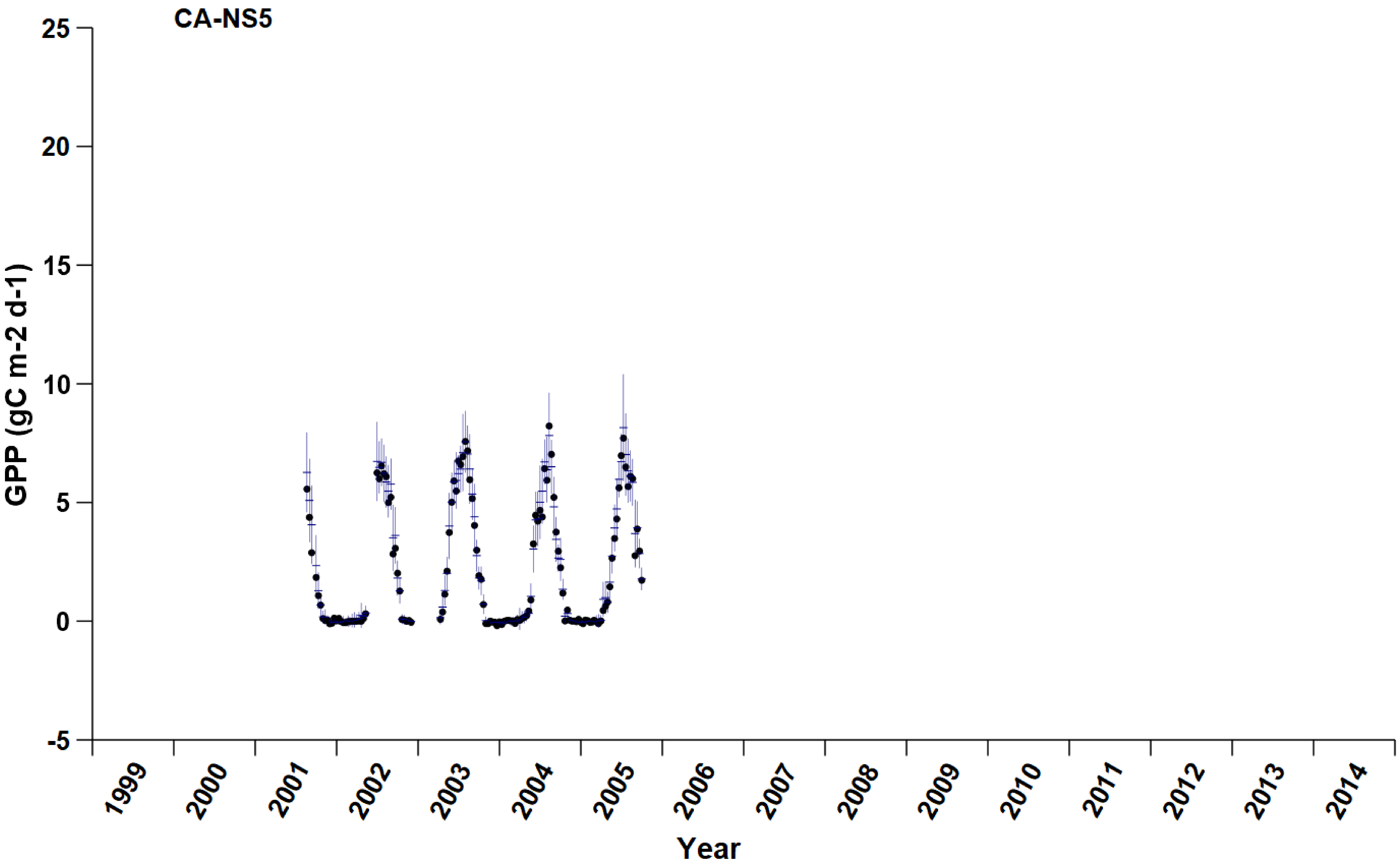
CA-NS5
GPP (gC m-2 d-1)
25
20
15
10
5
0
-5
1999
2000
2001
2002
2003
2004
2005
2006
2007
2008
2009
2010
2011
2012
2013
2014
Year

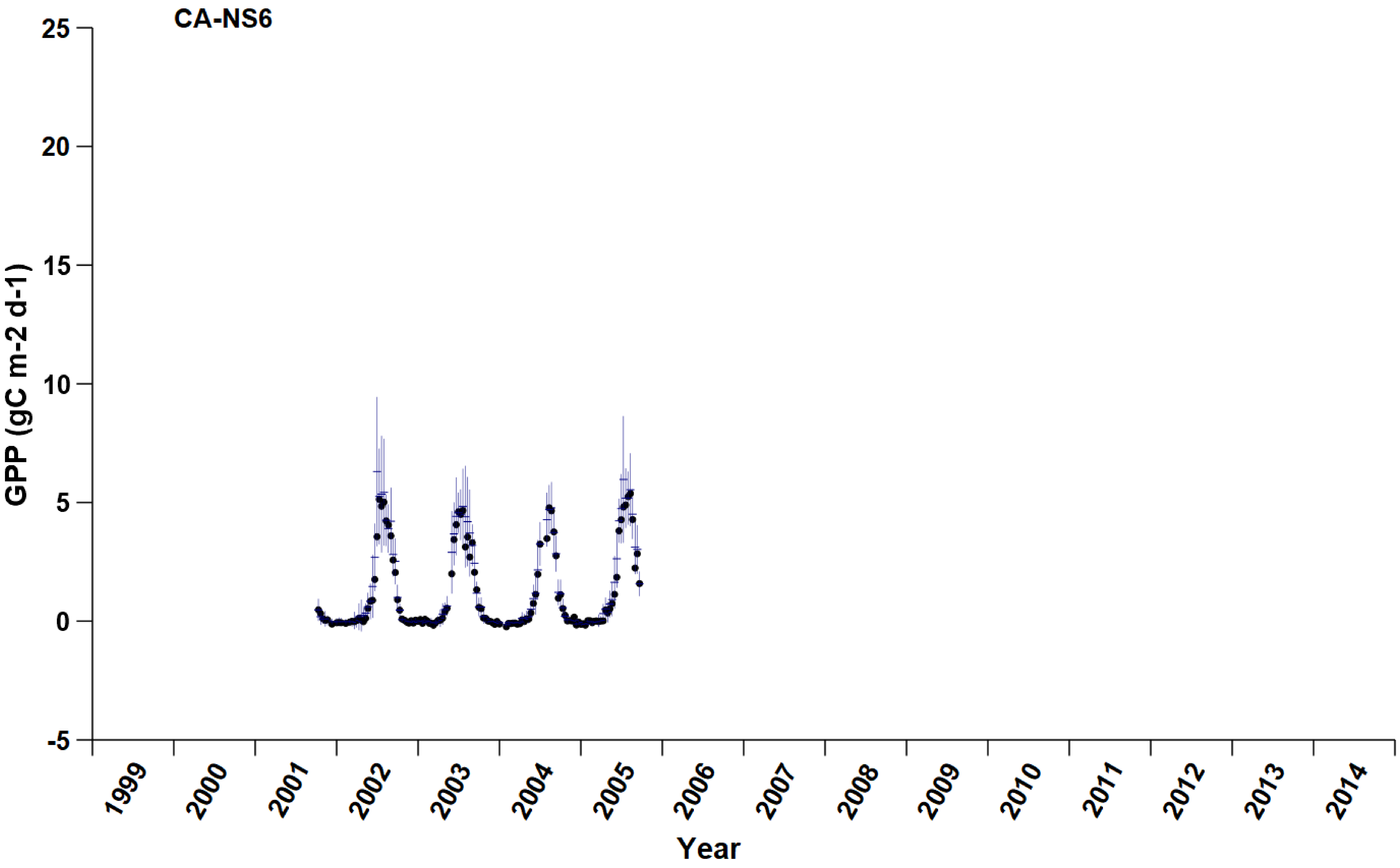

CA-NS6
GPP (gC m-2 d-1)
25
20
15
10
5
0
-5
1999
2000
2001
2002
2003
2004
2005
2006
2007
2008
2009
2010
2011
2012
2013
2014
Year

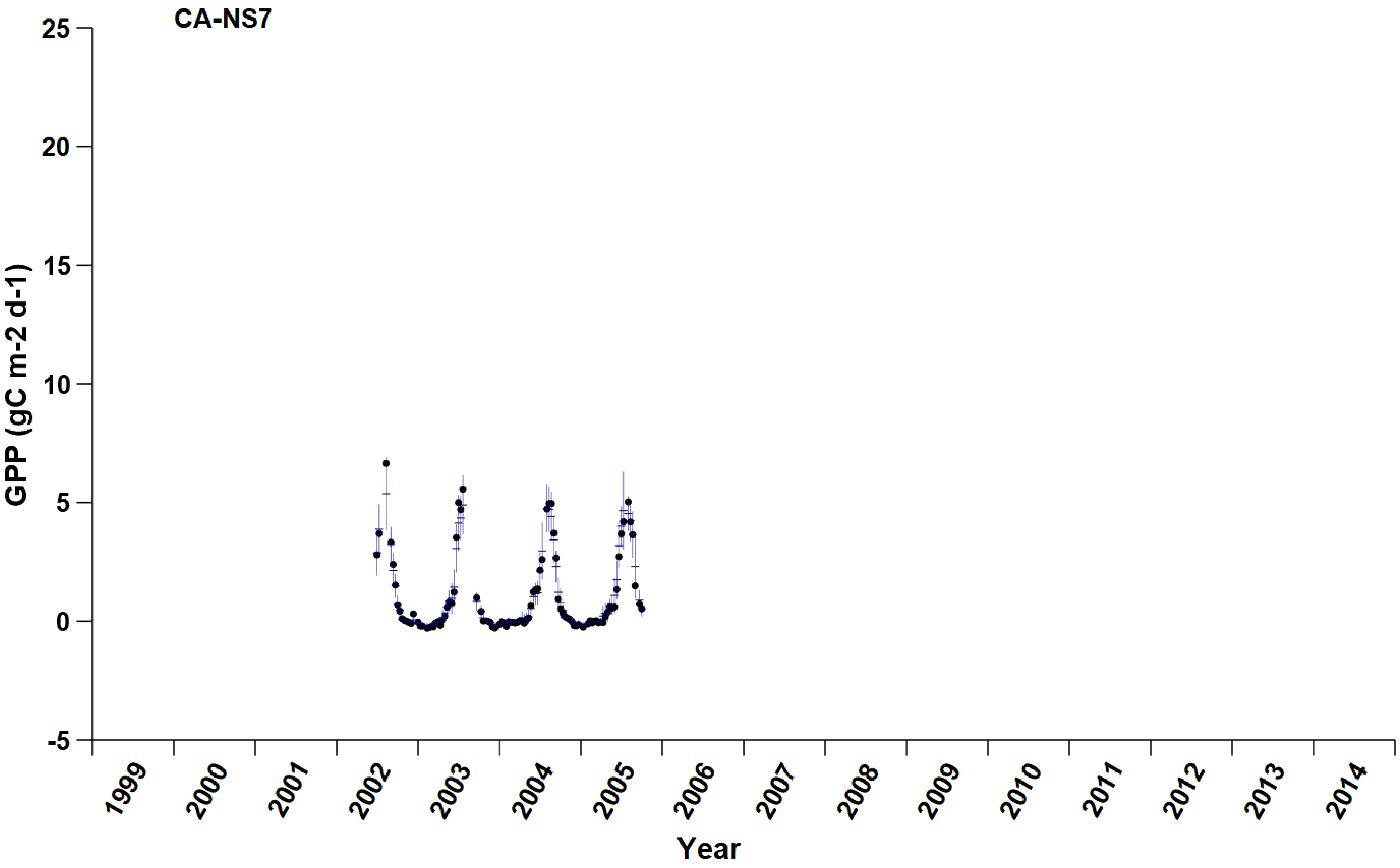

CA-NS7
GPP (gC m-2 d-1)
25
20
15
10
5
0
-5
1999
2000
2001
2002
2003
2004
2005
2006
2007
2008
2009
2010
2011
2012
2013
2014
Year

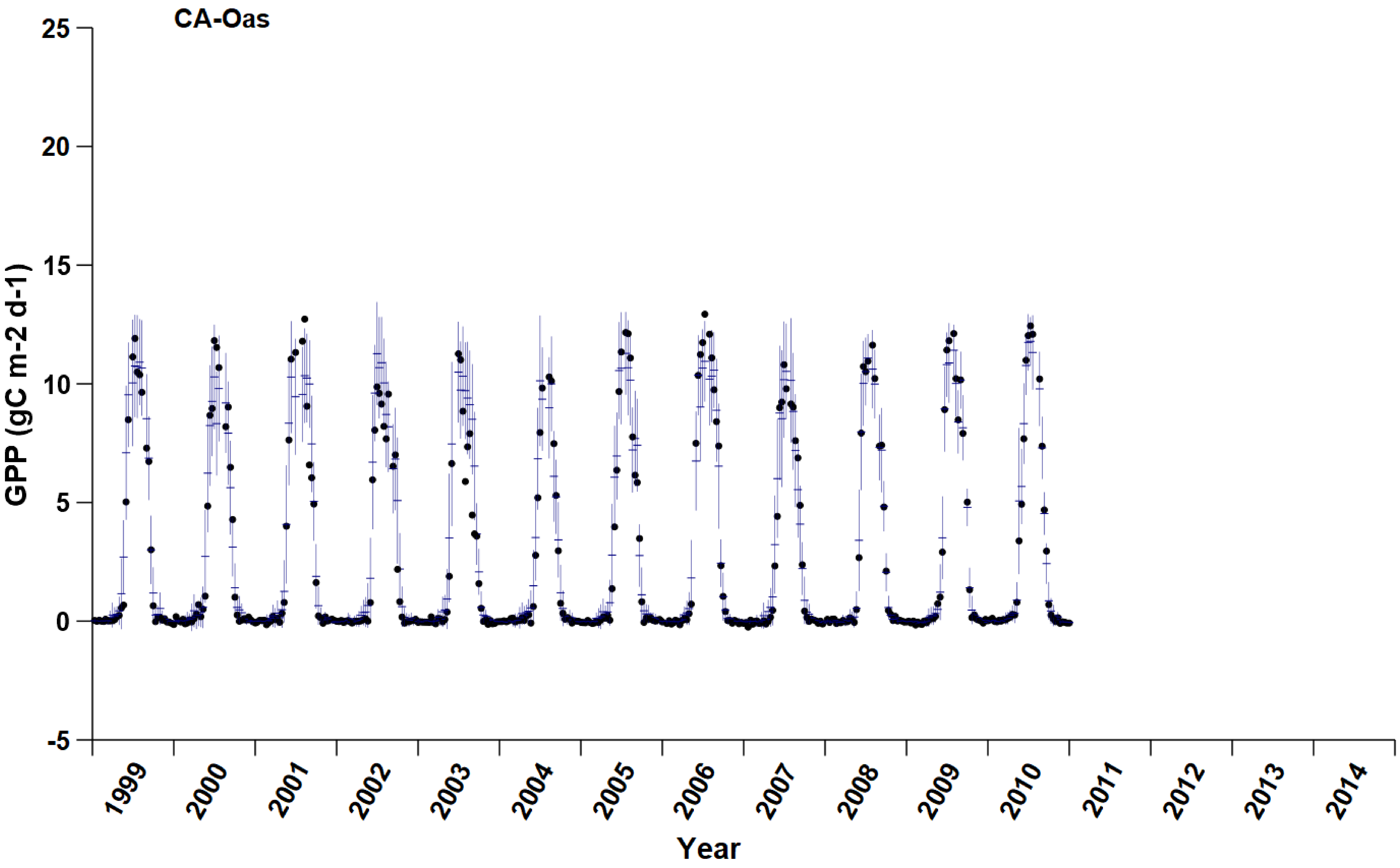

CA-Oas
GPP (gC m-2 d-1)
25
20
15
10
5
0
-5
1999
2000
2001
2002
2003
2004
2005
2006
2007
2008
2009
2010
2011
2012
2013
2014
Year

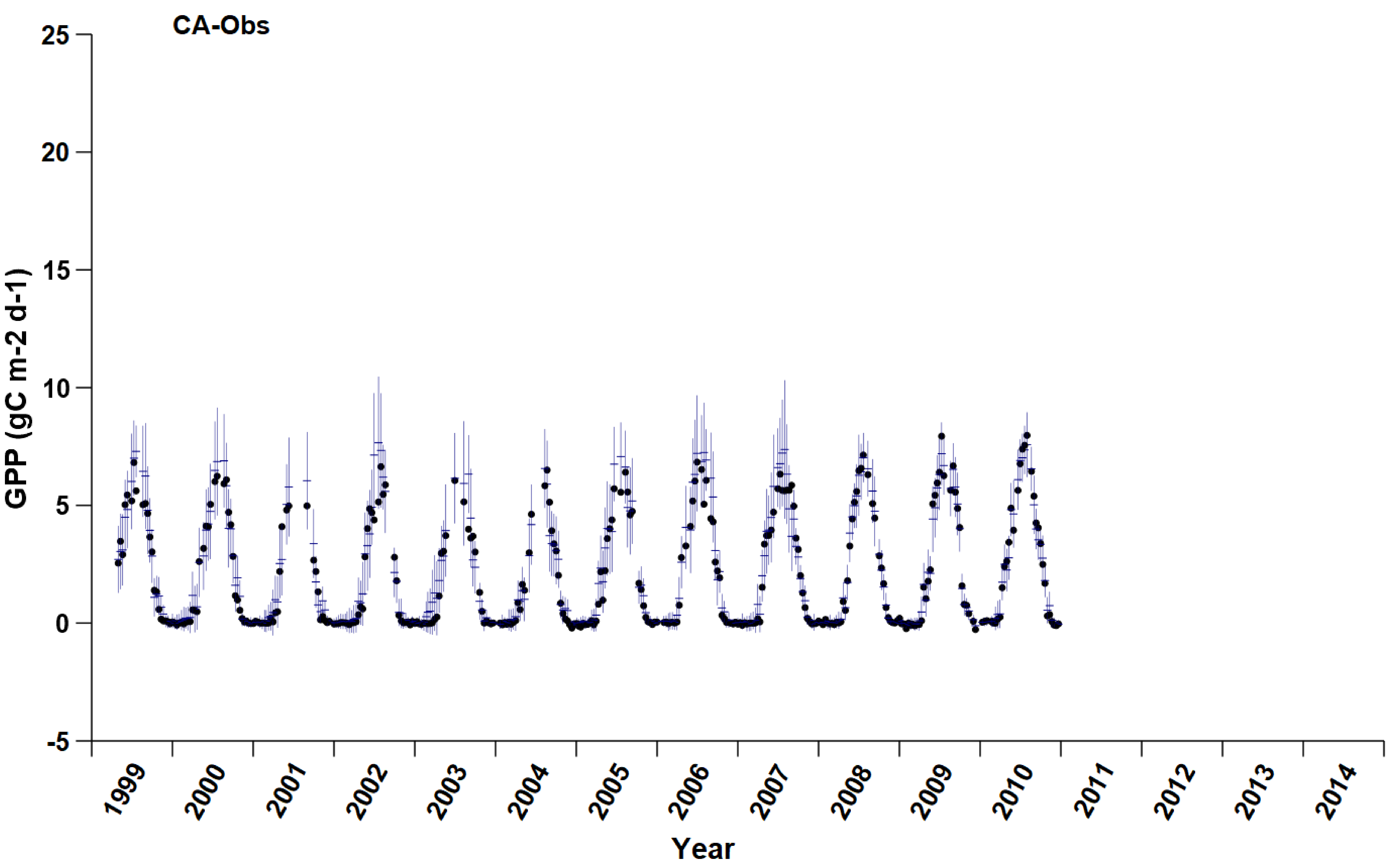

CA-Obs
GPP (gC m-2 d-1)
25
20
15
10
5
0
-5
1999
2000
2001
2002
2003
2004
2005
2006
2007
2008
2009
2010
2011
2012
2013
2014
Year

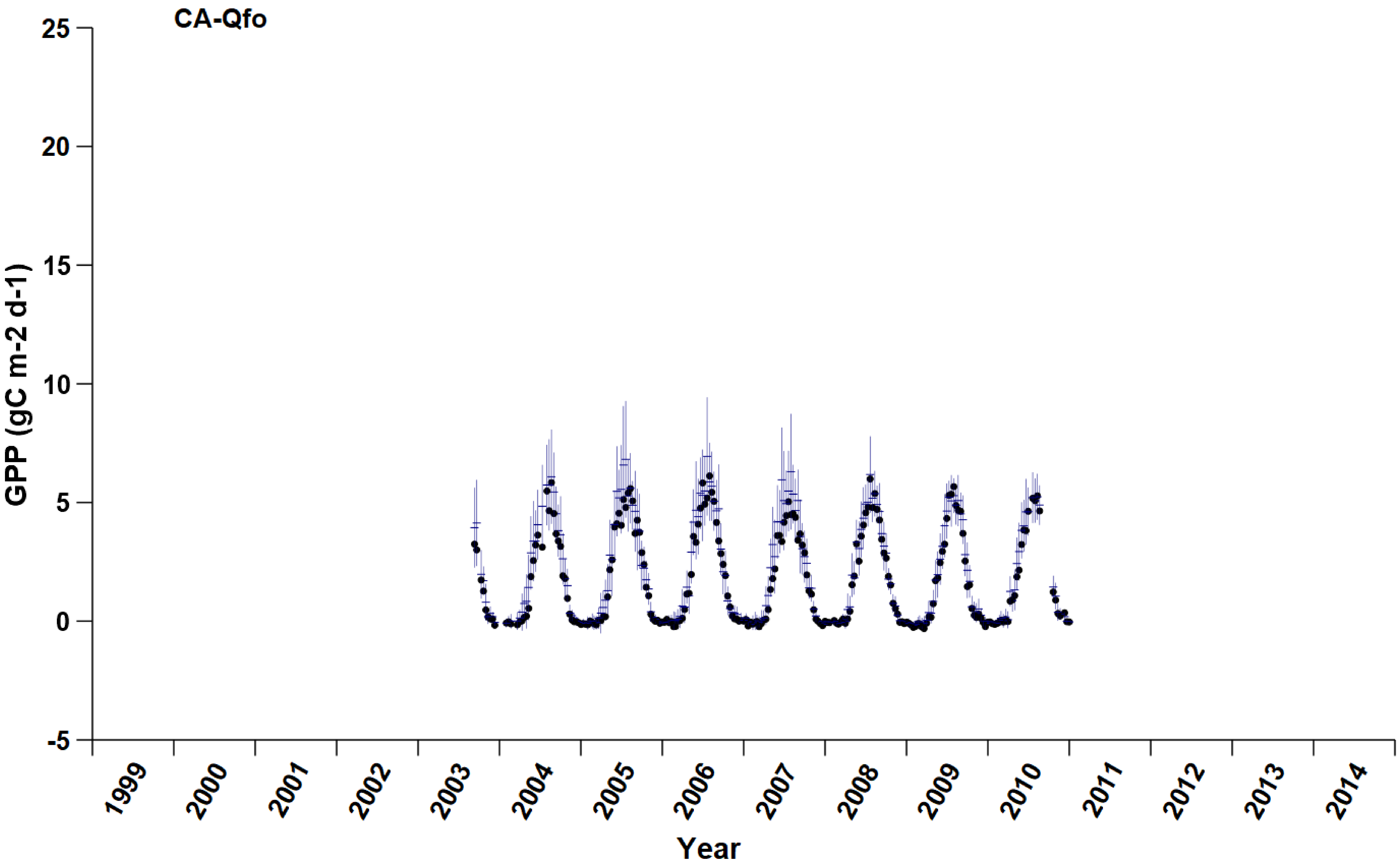

CA-Qfo
GPP (gC m-2 d-1)
25
20
15
10
5
0
-5
1999
2000
2001
2002
2003
2004
2005
2006
2007
2008
2009
2010
2011
2012
2013
2014
Year

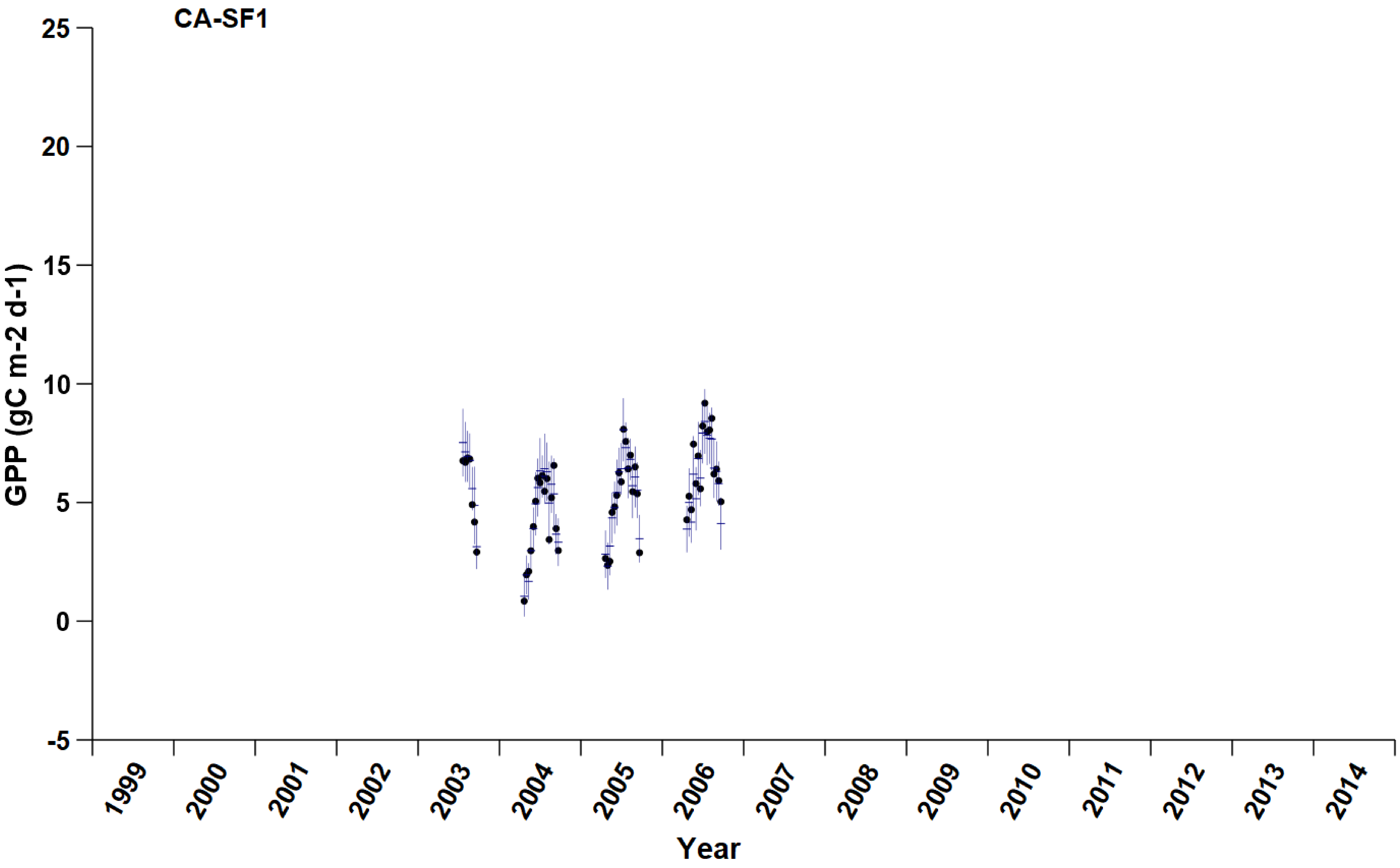

CA-SF1
GPP (gC m-2 d-1)
25
20
15
10
5
0
-5
1999
2000
2001
2002
2003
2004
2005
2006
2007
2008
2009
2010
2011
2012
2013
2014
Year

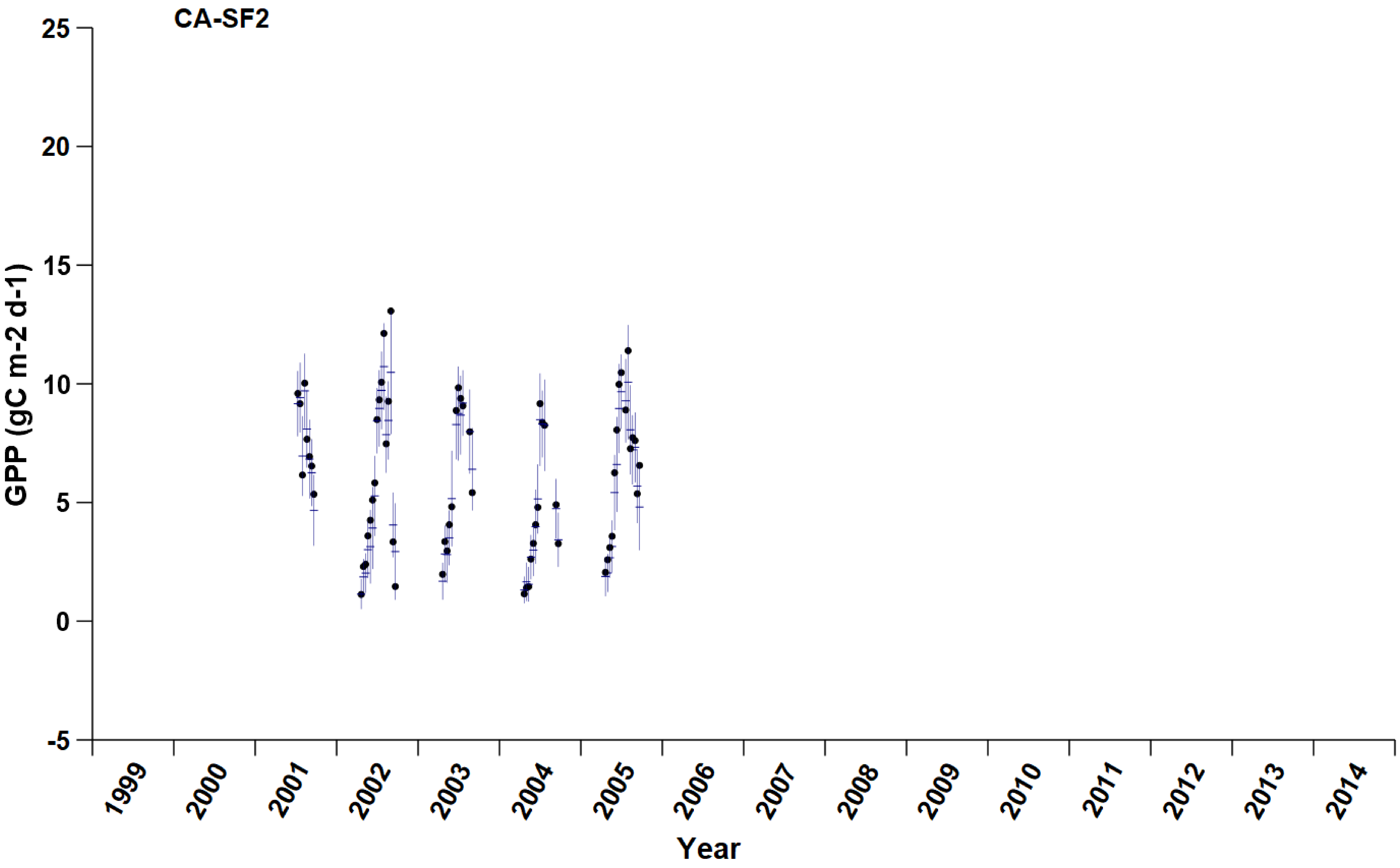
CA-SF2
GPP (gC m-2 d-1)
25
20
15
10
5
0
-5
1999
2000
2001
2002
2003
2004
2005
2006
2007
2008
2009
2010
2011
2012
2013
2014
Year

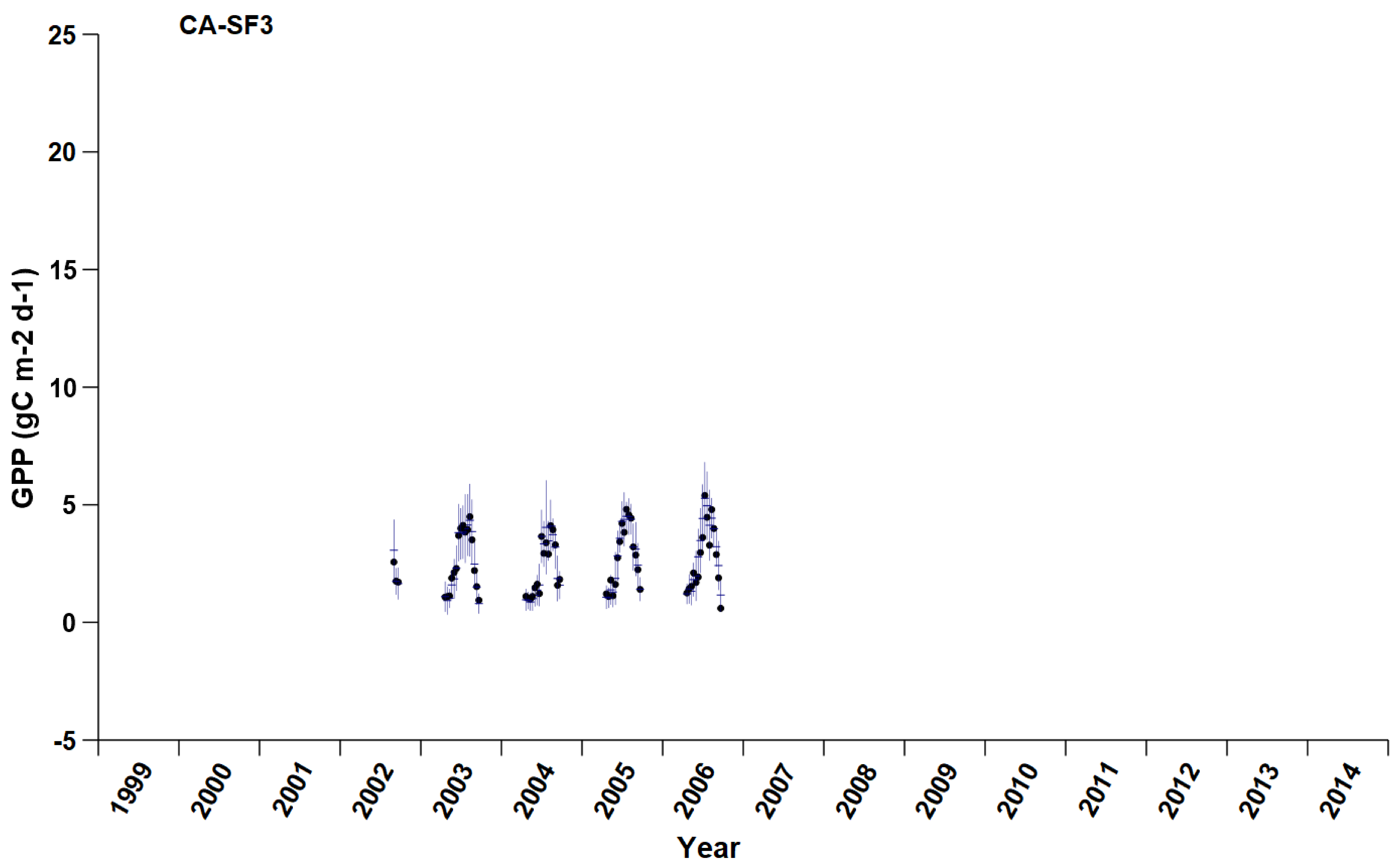
CA-SF3
GPP (gC m-2 d-1)
25
20
15
10
5
0
-5
1999
2000
2001
2002
2003
2004
2005
2006
2007
2008
2009
2010
2011
2012
2013
2014
Year

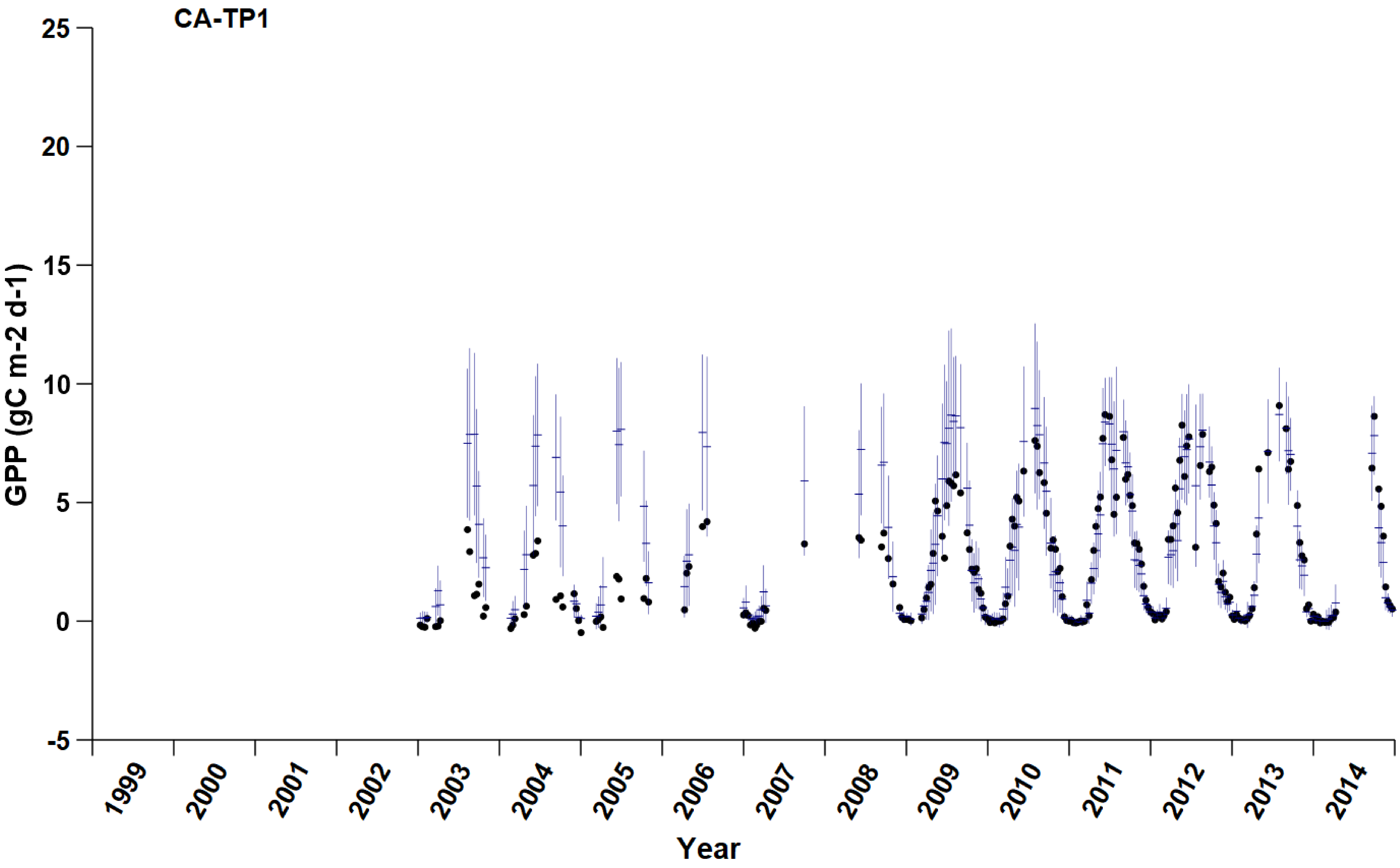
CA-TP1
GPP (gC m-2 d-1)
25
20
15
10
5
0
-5
1999
2000
2001
2002
2003
2004
2005
2006
2007
2008
2009
2010
2011
2012
2013
2014
Year

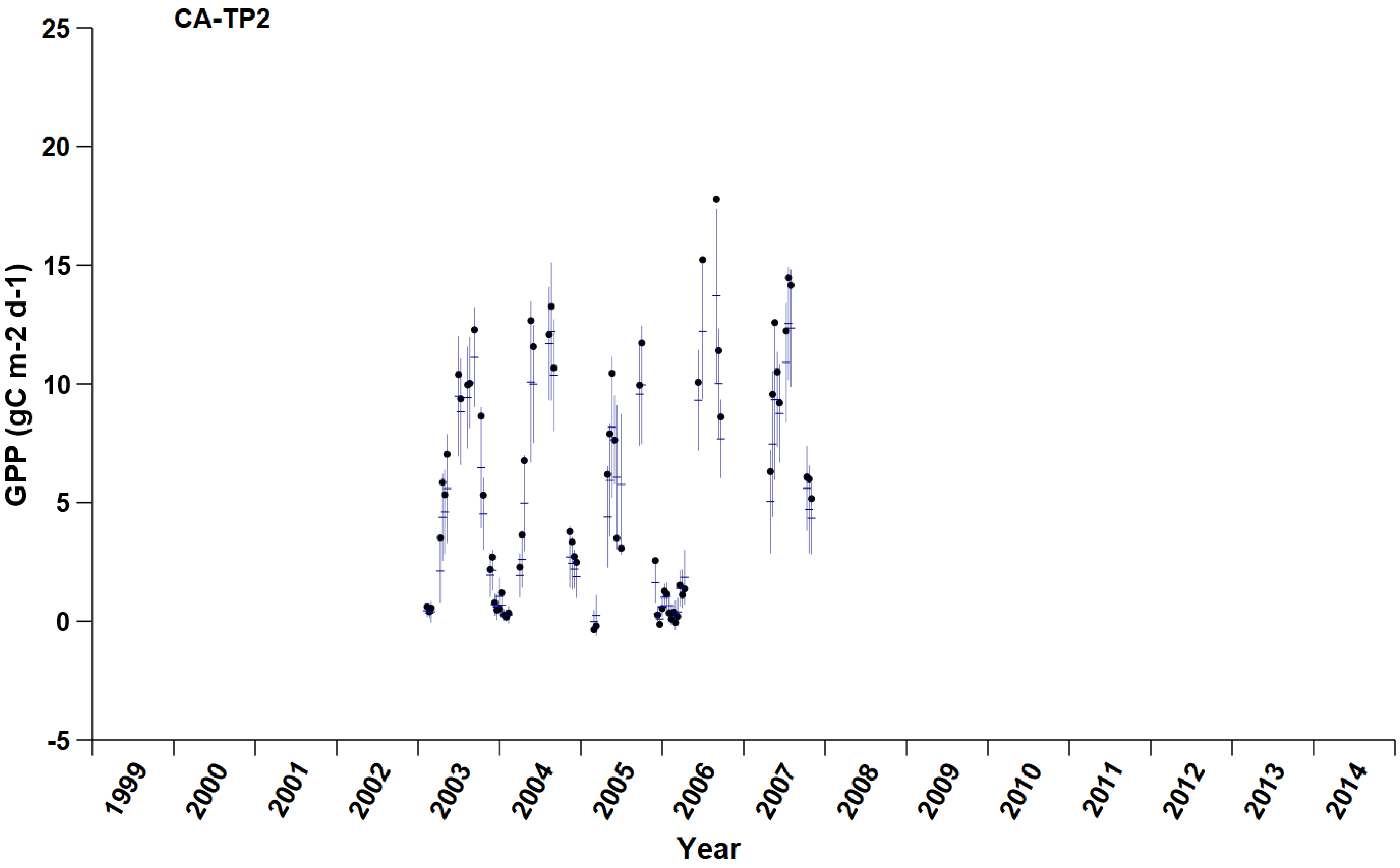
CA-TP2
GPP (gC m-2 d-1)
25
20
15
10
5
0
-5
1999
2000
2001
2002
2003
2004
2005
2006
2007
2008
2009
2010
2011
2012
2013
2014
Year

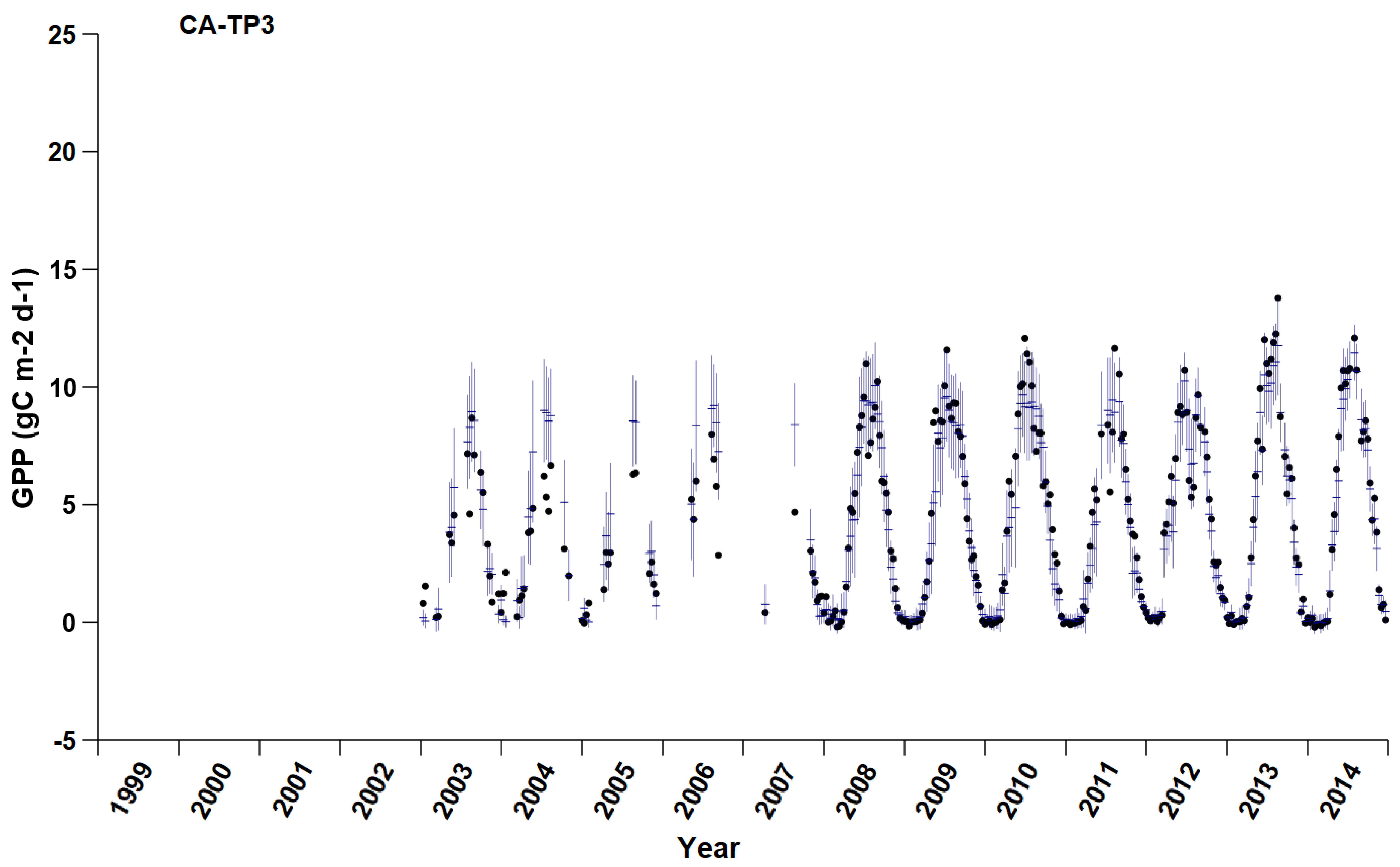

CA-TP3
GPP (gC m-2 d-1)
25
20
15
10
5
0
-5
1999
2000
2001
2002
2003
2004
2005
2006
2007
2008
2009
2010
2011
2012
2013
2014
Year

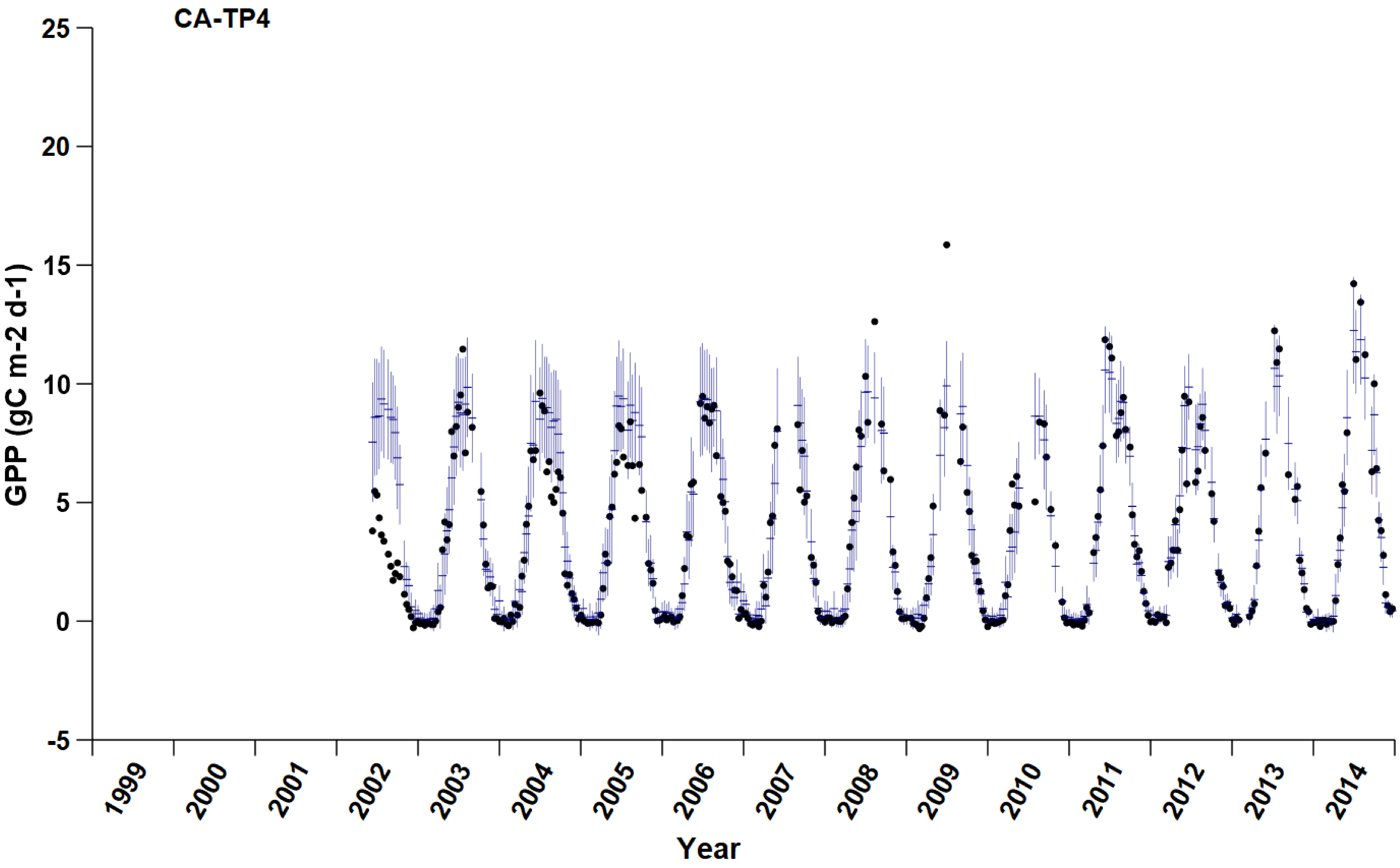
CA-TP4
GPP (gC m-2 d-1)
25
20
15
10
5
0
-5
1999
2000
2001
2002
2003
2004
2005
2006
2007
2008
2009
2010
2011
2012
2013
2014
Year

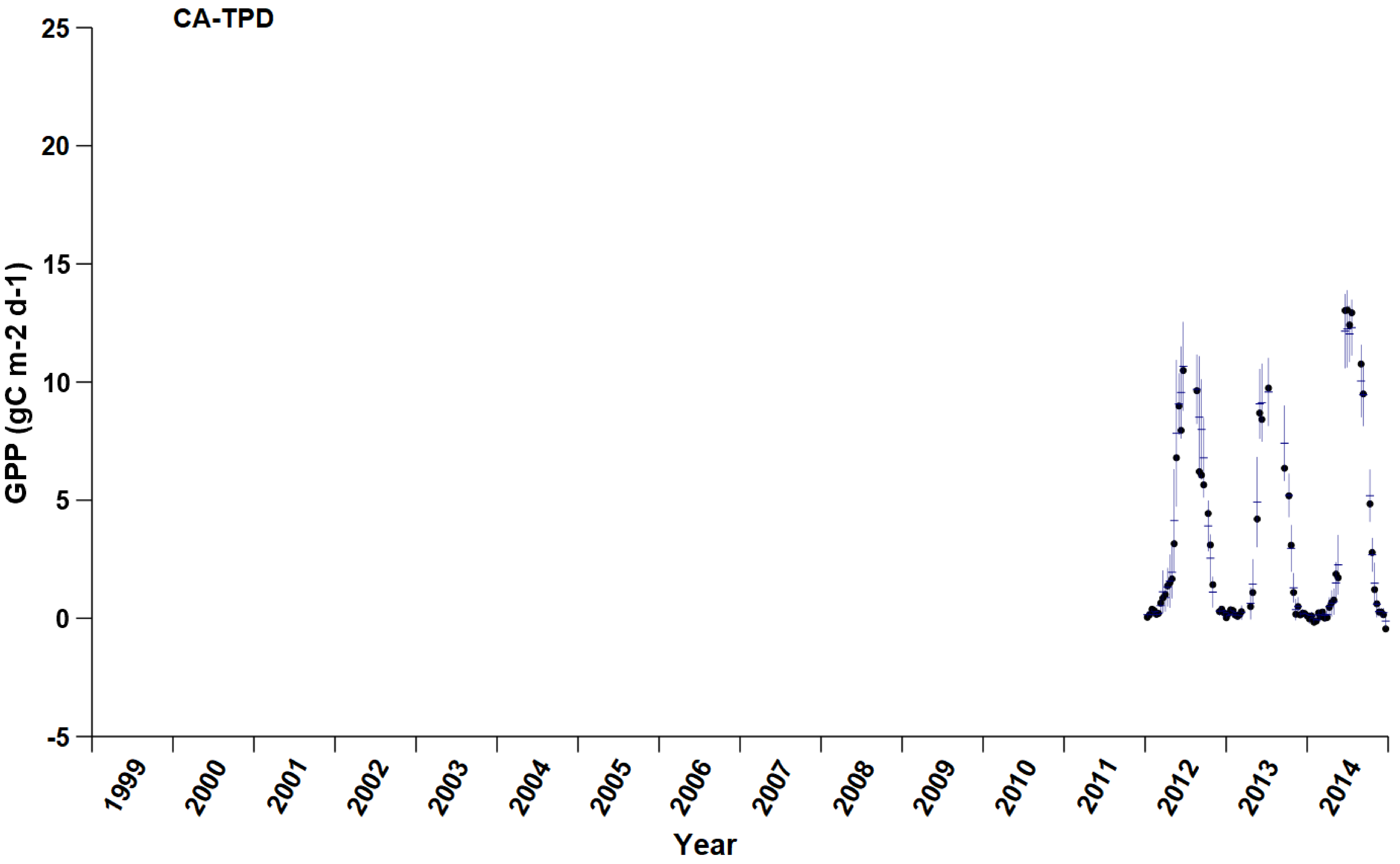
CA-TPD
GPP (gC m-2 d-1)
25
20
15
10
5
0
-5
1999
2000
2001
2002
2003
2004
2005
2006
2007
2008
2009
2010
2011
2012
2013
2014
Year

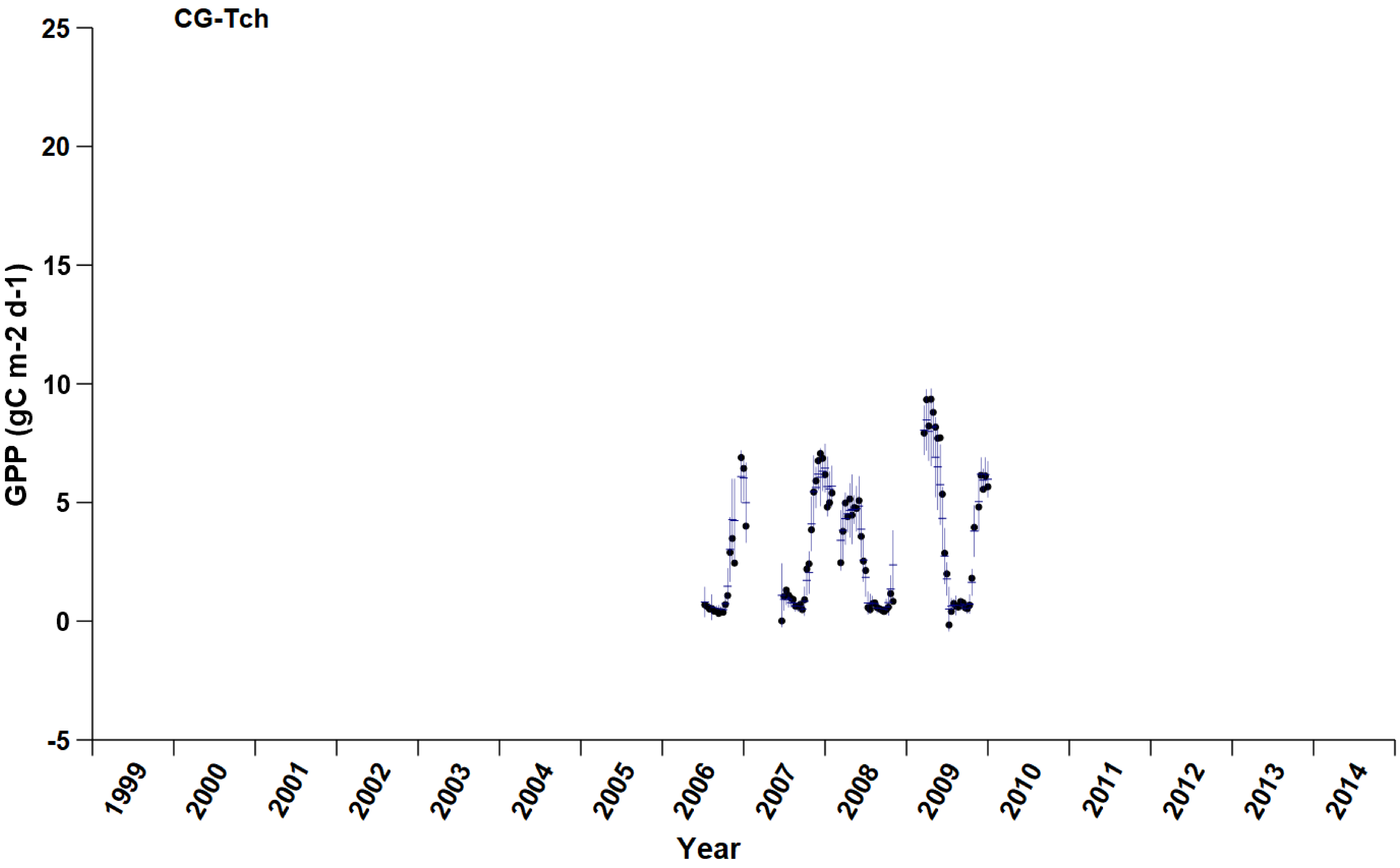
CG-Tch
GPP (gC m-2 d-1)
25
20
15
10
5
0
-5
1999
2000
2001
2002
2003
2004
2005
2006
2007
2008
2009
2010
2011
2012
2013
2014
Year

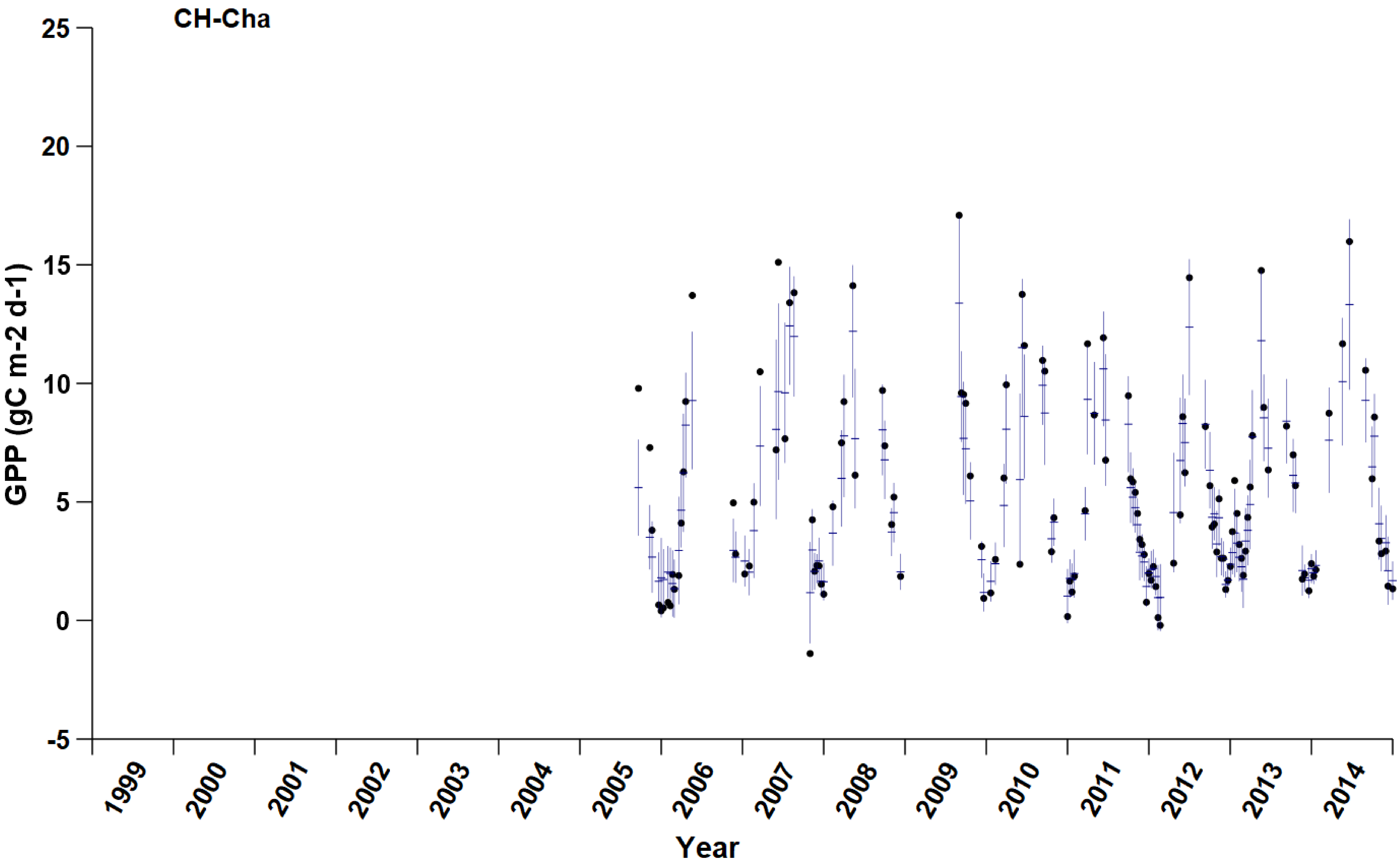

CH-Cha
GPP (gC m-2 d-1)
25
20
15
10
5
0
-5
1999
2000
2001
2002
2003
2004
2005
2006
2007
2008
2009
2010
2011
2012
2013
2014
Year

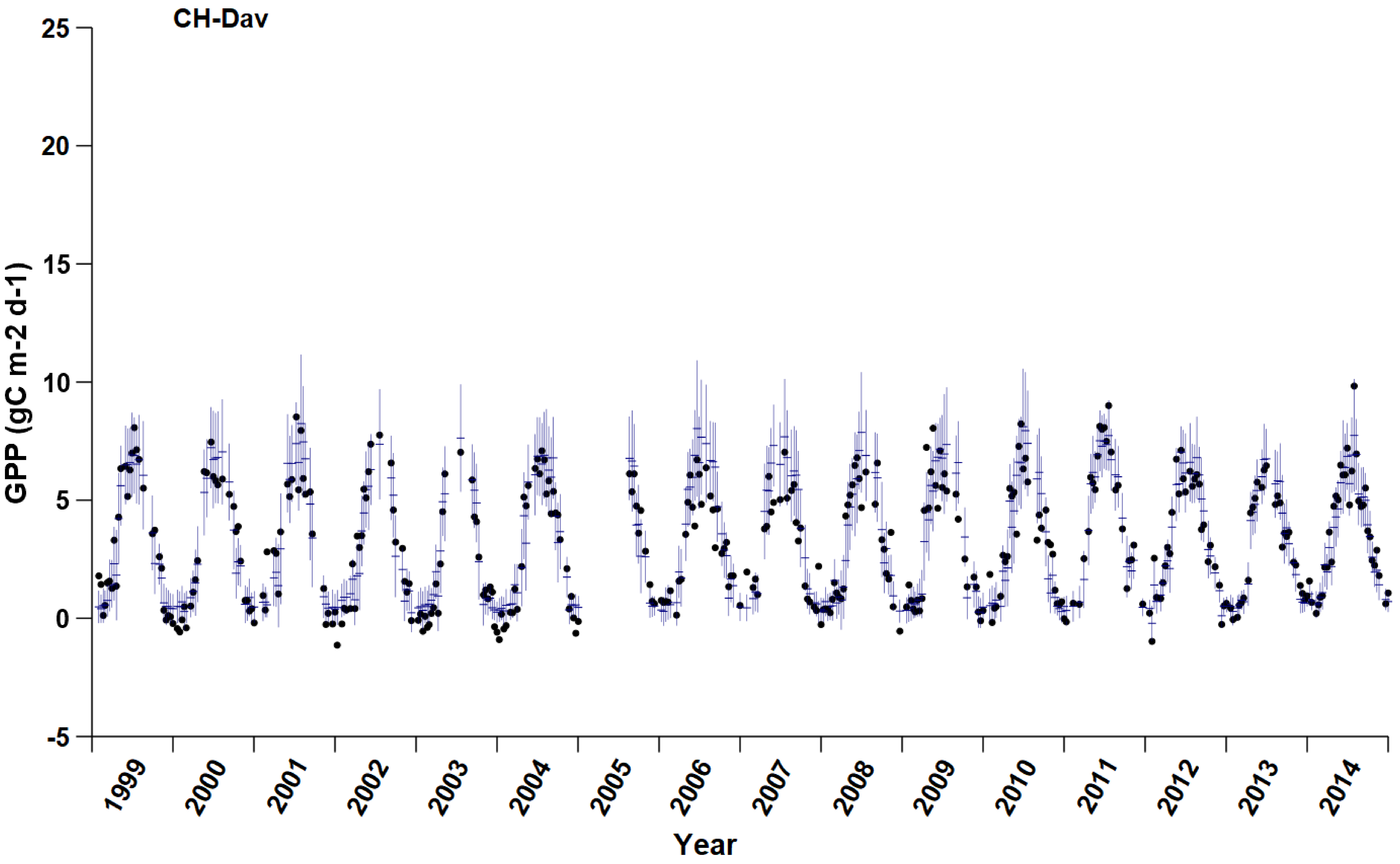
CH-Dav
GPP (gC m-2 d-1)
25
20
15
10
5
0
-5
1999
2000
2001
2002
2003
2004
2005
2006
2007
2008
2009
2010
2011
2012
2013
2014
Year

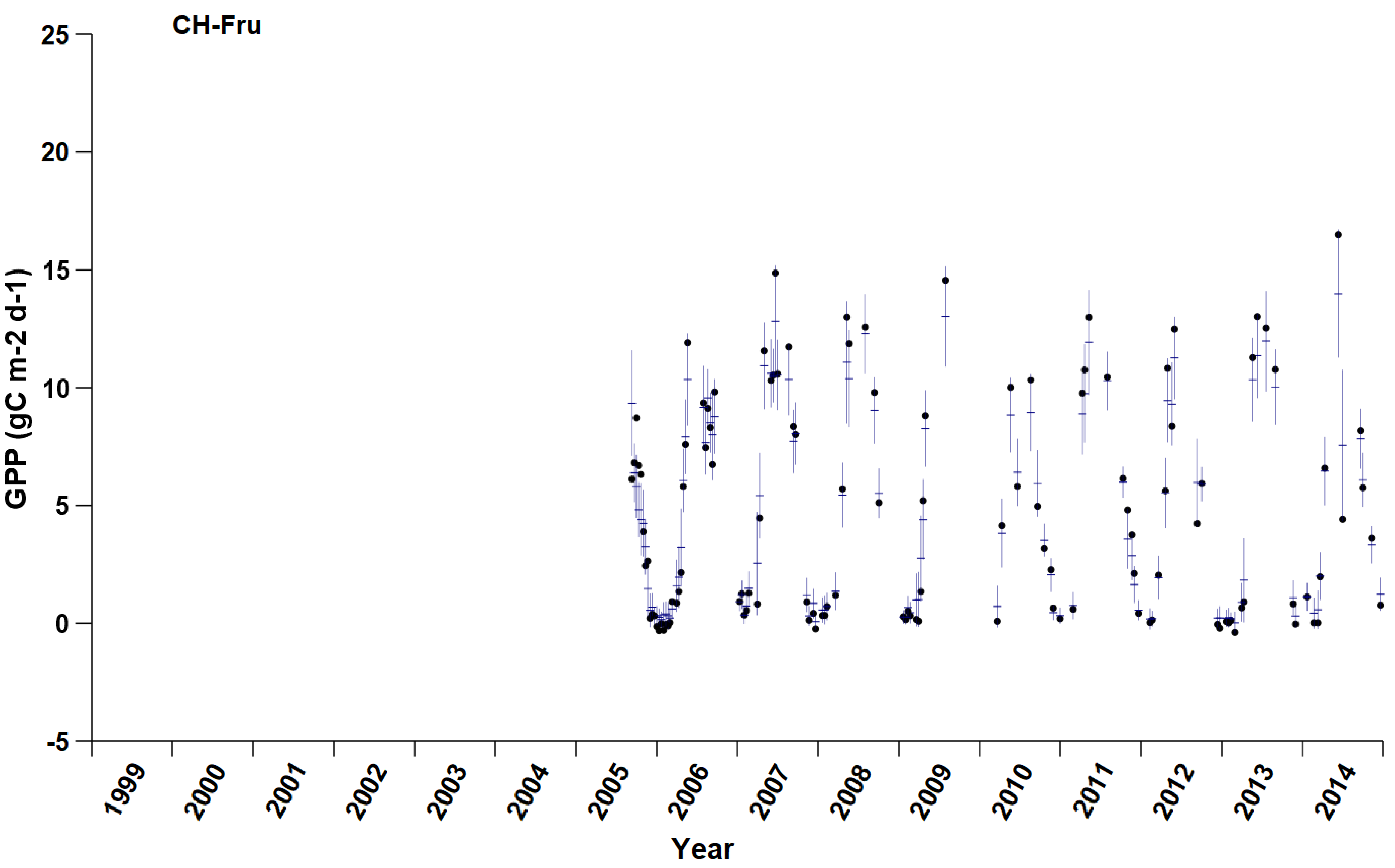

CH-Fru
GPP (gC m-2 d-1)
25
20
15
10
5
0
-5
1999
2000
2001
2002
2003
2004
2005
2006
2007
2008
2009
2010
2011
2012
2013
2014
Year

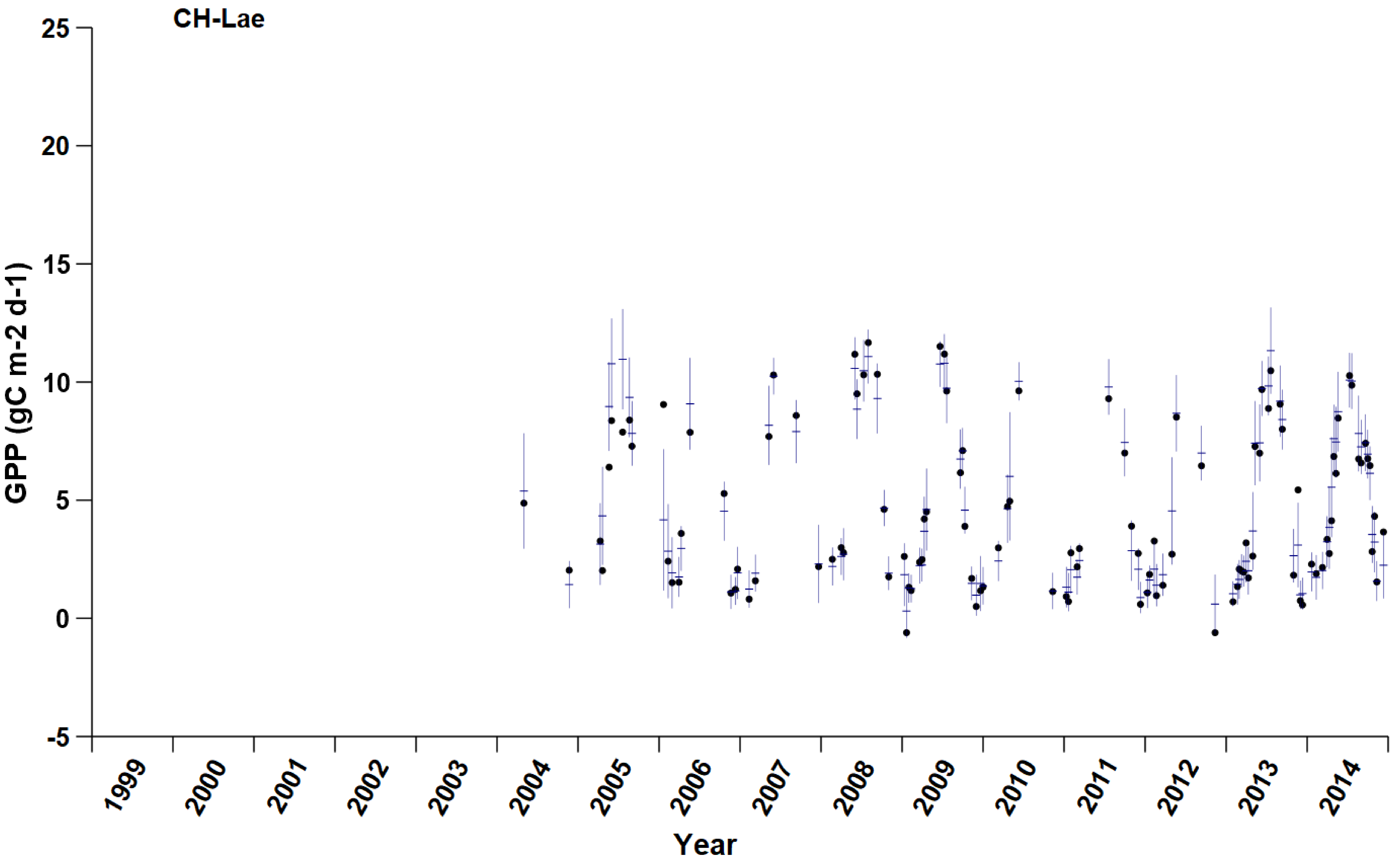
CH-Lae
GPP (gC m-2 d-1)
25
20
15
10
5
0
-5
1999
2000
2001
2002
2003
2004
2005
2006
2007
2008
2009
2010
2011
2012
2013
2014
Year

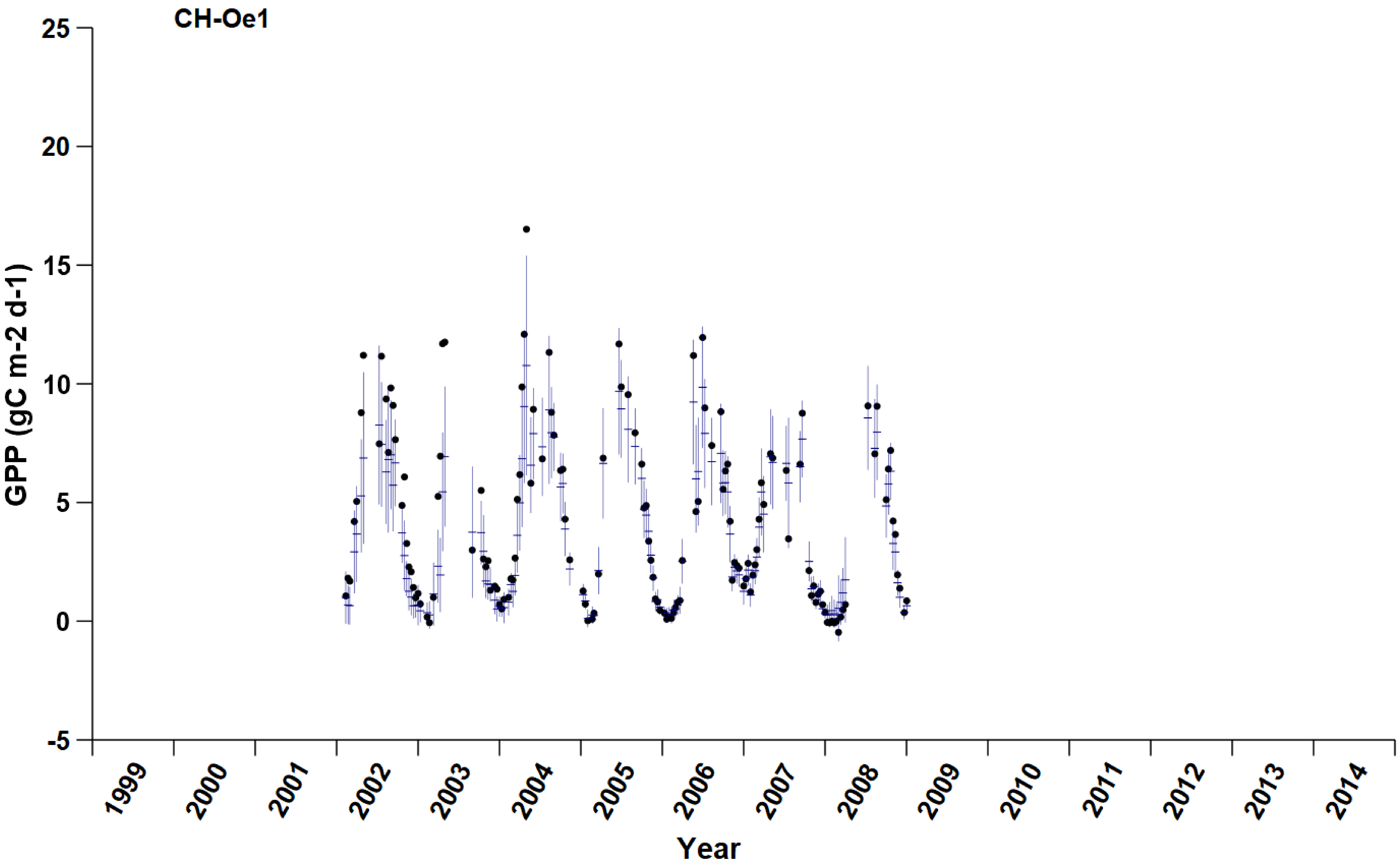

CH-Oe1
GPP (gC m-2 d-1)
25
20
15
10
5
0
-5
1999
2000
2001
2002
2003
2004
2005
2006
2007
2008
2009
2010
2011
2012
2013
2014
Year

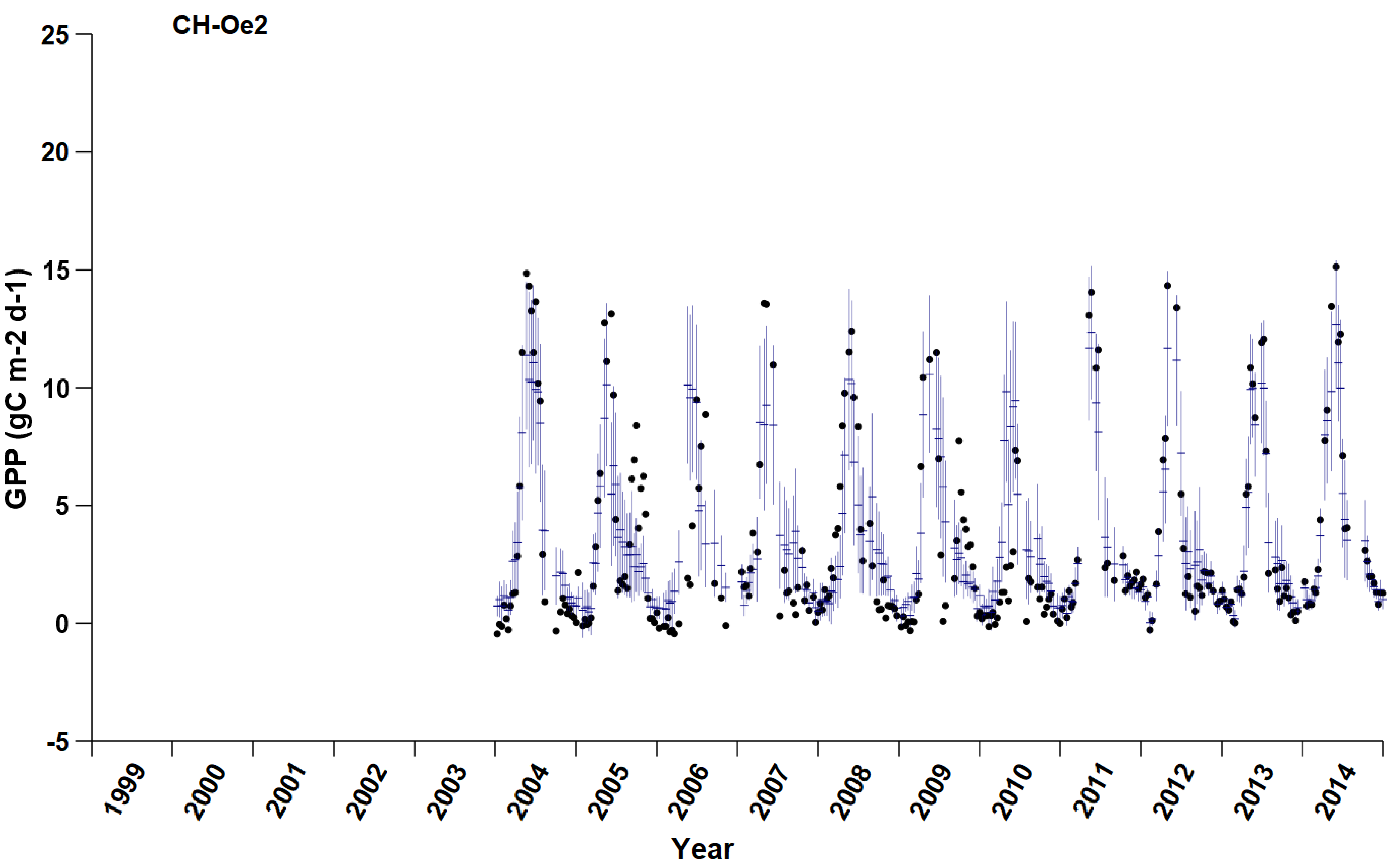

CH-Oe2
GPP (gC m-2 d-1)
25
20
15
10
5
0
-5
1999
2000
2001
2002
2003
2004
2005
2006
2007
2008
2009
2010
2011
2012
2013
2014
Year

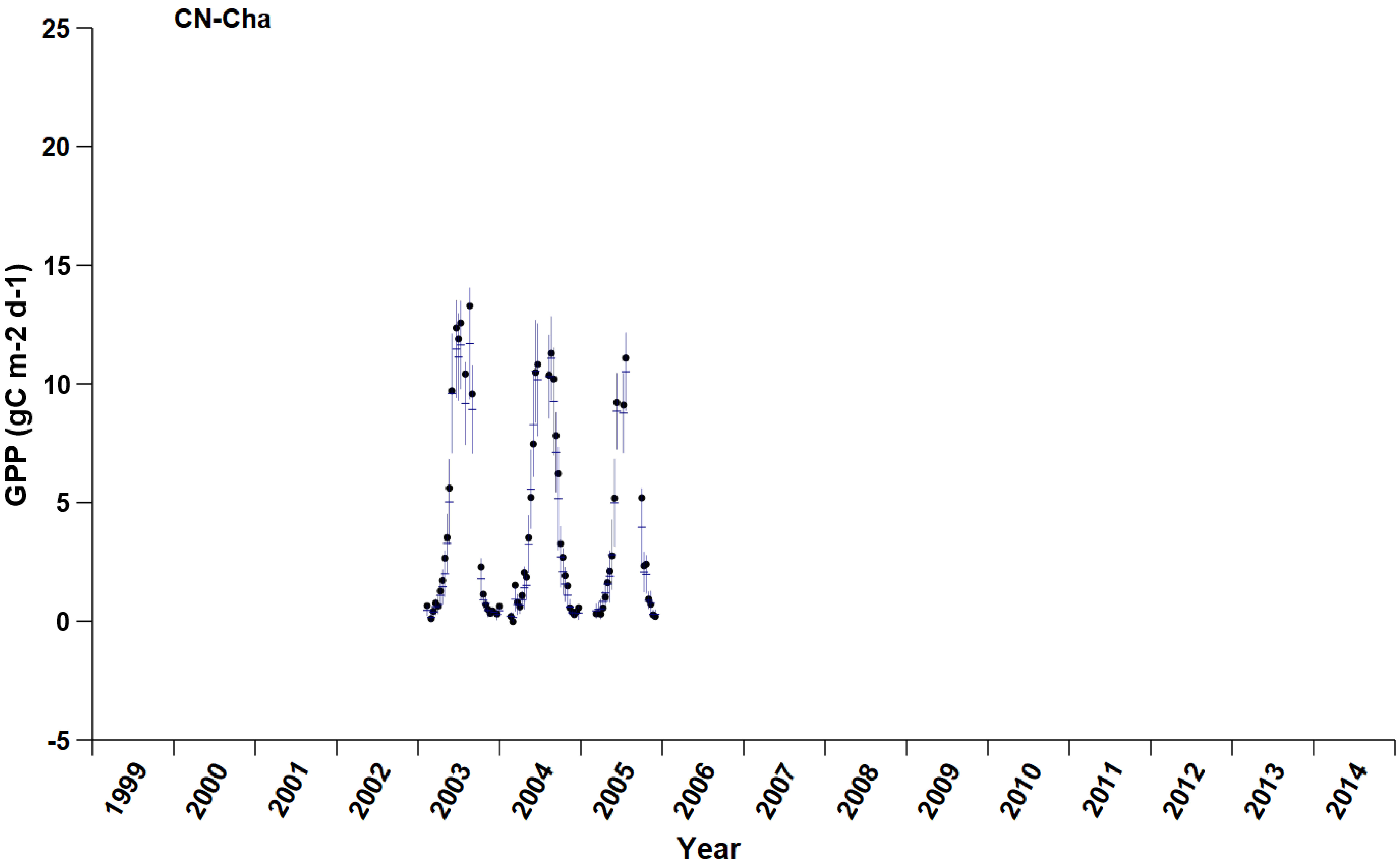
CN-Cha
GPP (gC m-2 d-1)
25
20
15
10
5
0
-5
1999
2000
2001
2002
2003
2004
2005
2006
2007
2008
2009
2010
2011
2012
2013
2014
Year

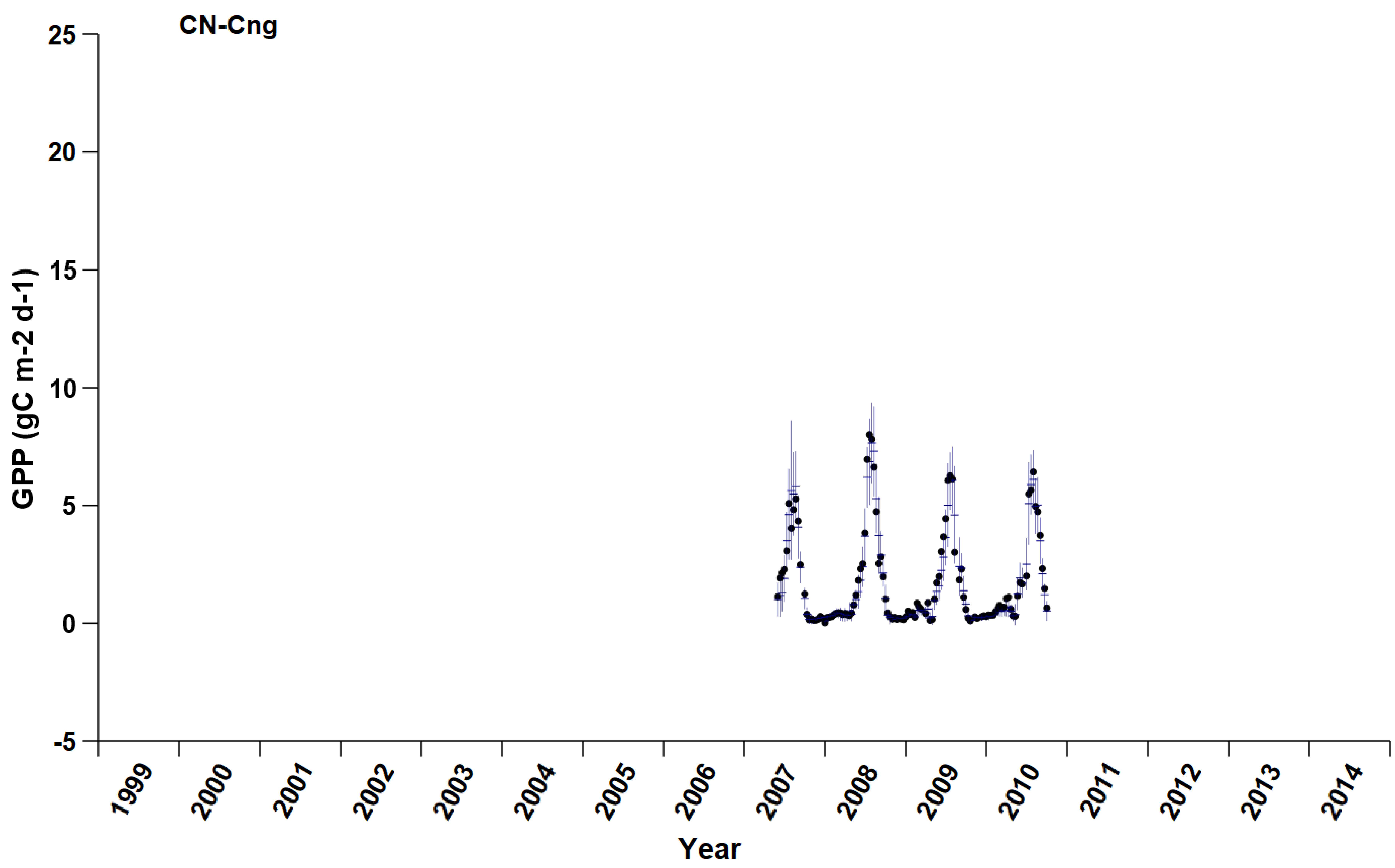

CN-Cng
GPP (gC m-2 d-1)
25
20
15
10
5
0
-5
1999
2000
2001
2002
2003
2004
2005
2006
2007
2008
2009
2010
2011
2012
2013
2014
Year

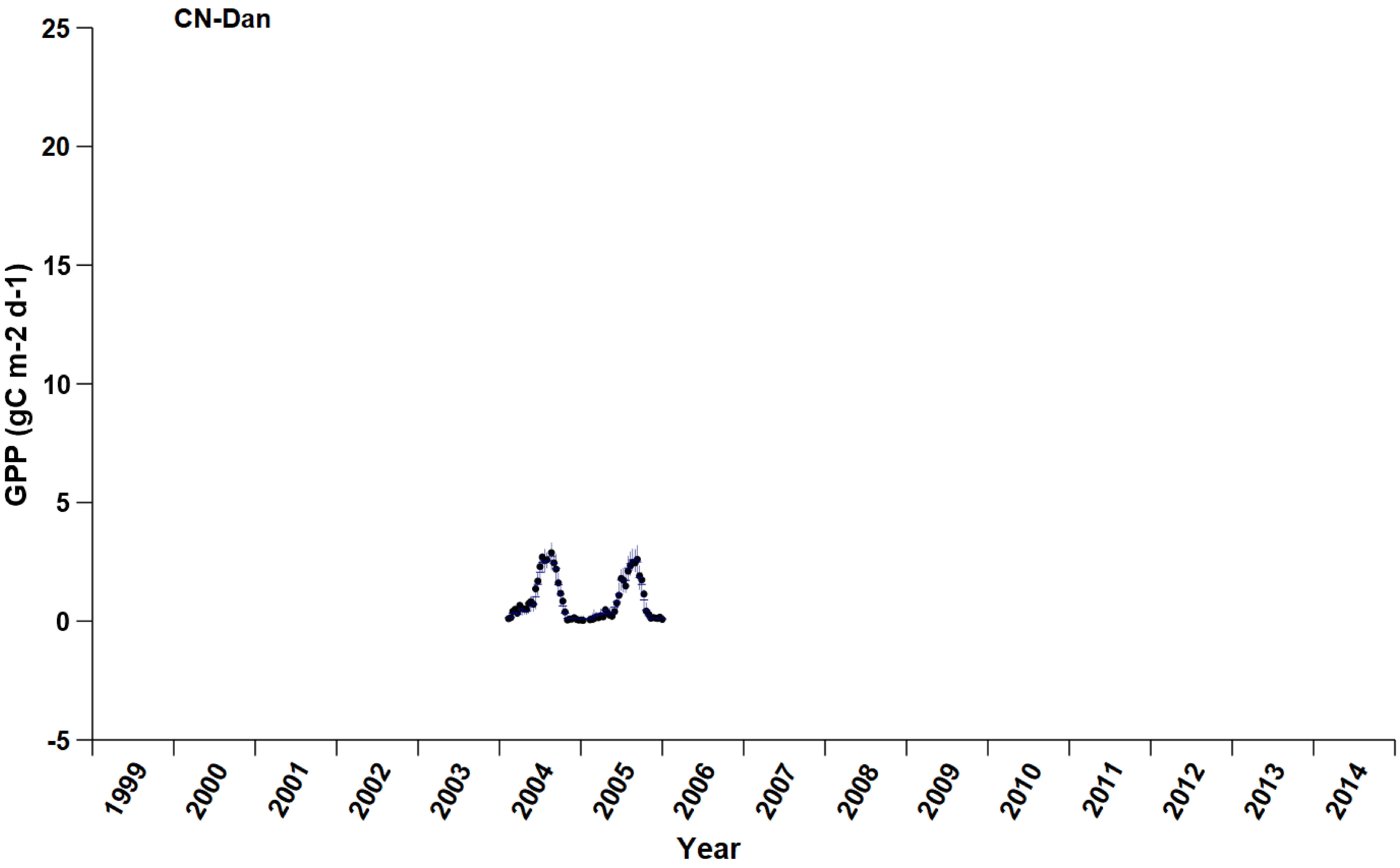
CN-Dan
25
20
15
10
5
0
-5
GPP (gC m-2 d-1)
1999
2000
2001
2002
2003
2004
2005
2006
2007
2008
2009
2010
2011
2012
2013
2014
Year

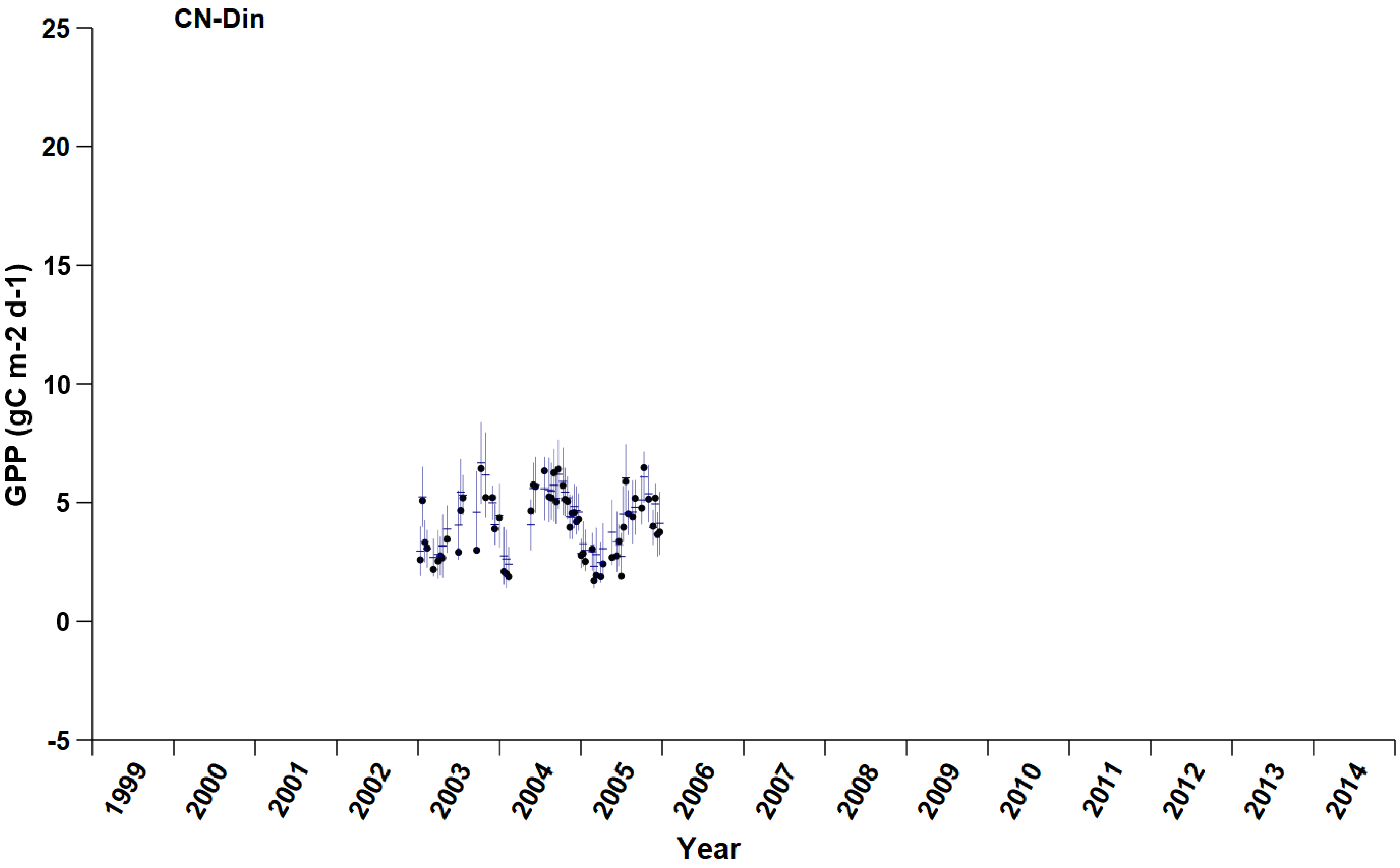

CN-Din
GPP (gC m-2 d-1)
25
20
15
10
5
0
-5
1999
2000
2001
2002
2003
2004
2005
2006
2007
2008
2009
2010
2011
2012
2013
2014
Year

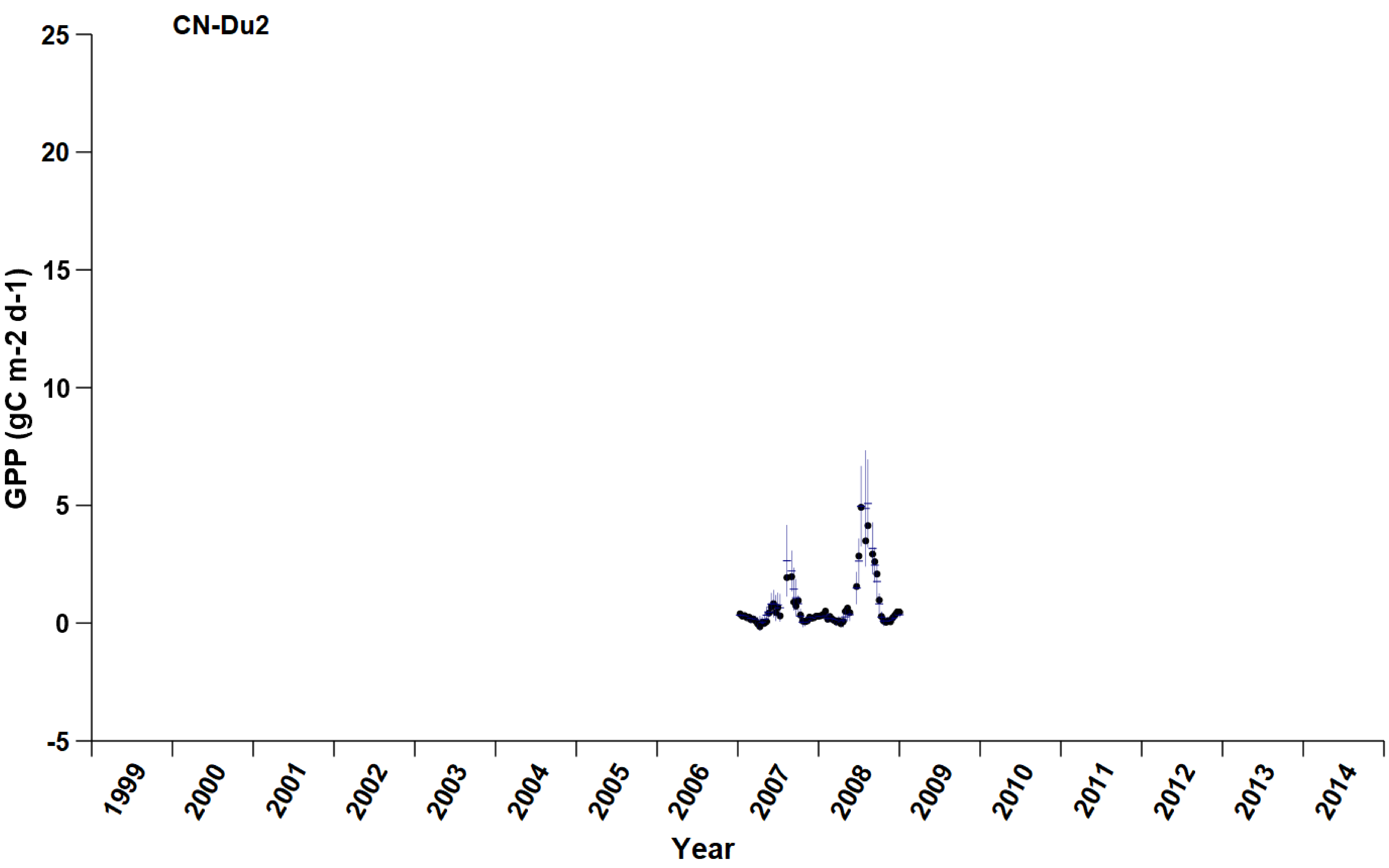

CN-Du2
GPP (gC m-2 d-1)
25
20
15
10
5
0
-5
1999
2000
2001
2002
2003
2004
2005
2006
2007
2008
2009
2010
2011
2012
2013
2014
Year

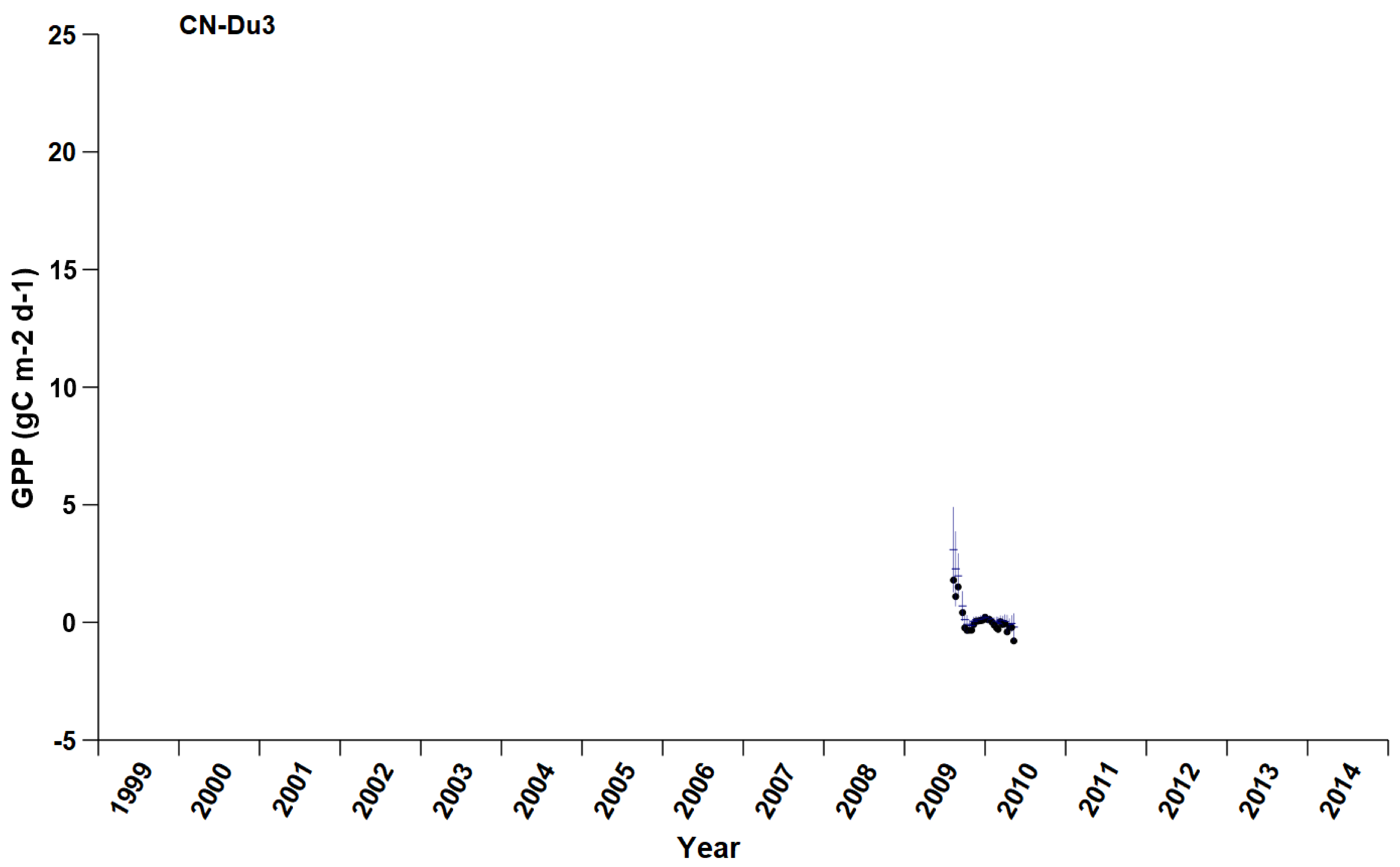
CN-Du3
GPP (gC m-2 d-1)
25
20
15
10
5
0
-5
1999
2000
2001
2002
2003
2004
2005
2006
2007
2008
2009
2010
2011
2012
2013
2014
Year

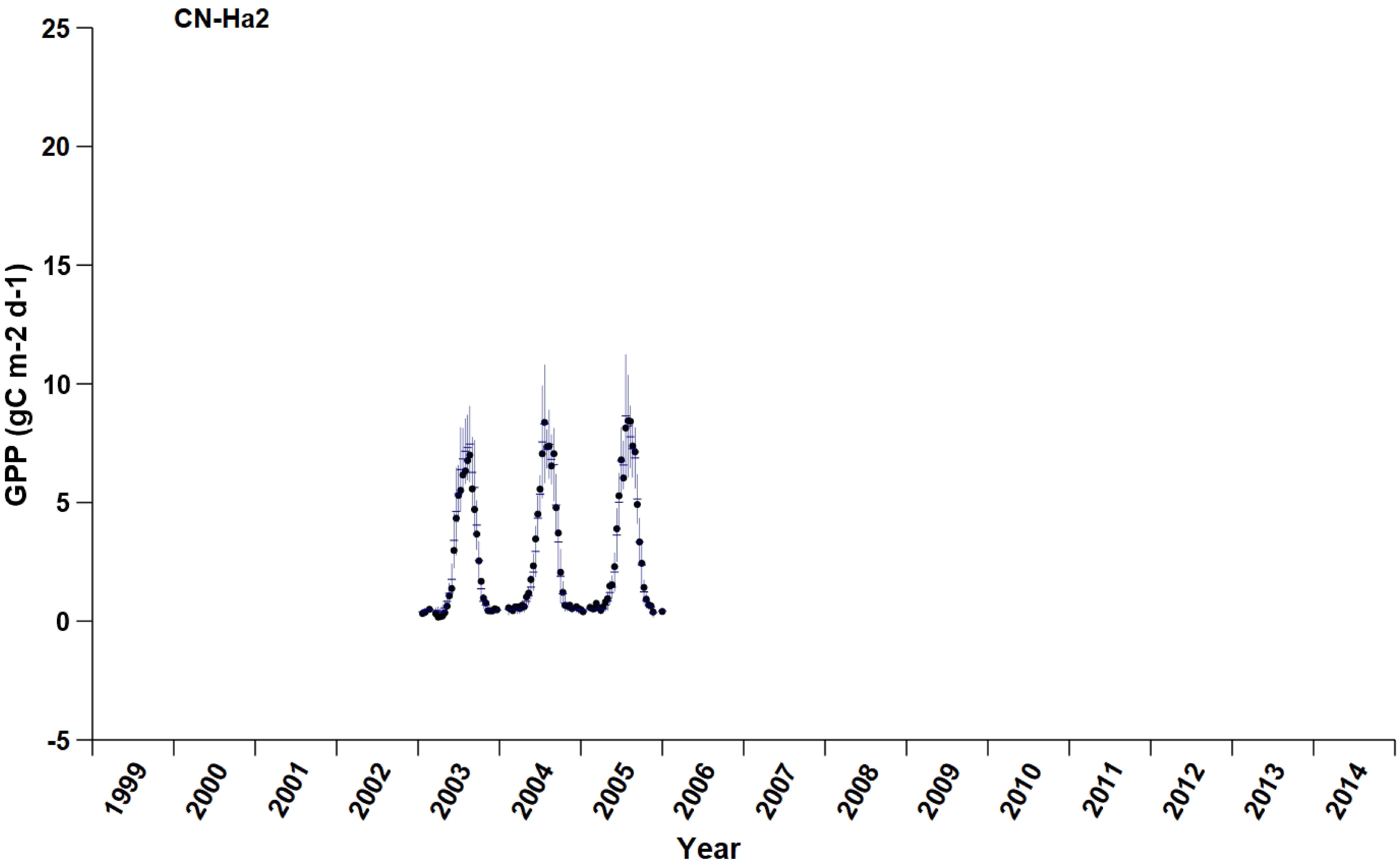

CN-Ha2
GPP (gC m-2 d-1)
25
20
15
10
5
0
-5
1999
2000
2001
2002
2003
2004
2005
2006
2007
2008
2009
2010
2011
2012
2013
2014
Year

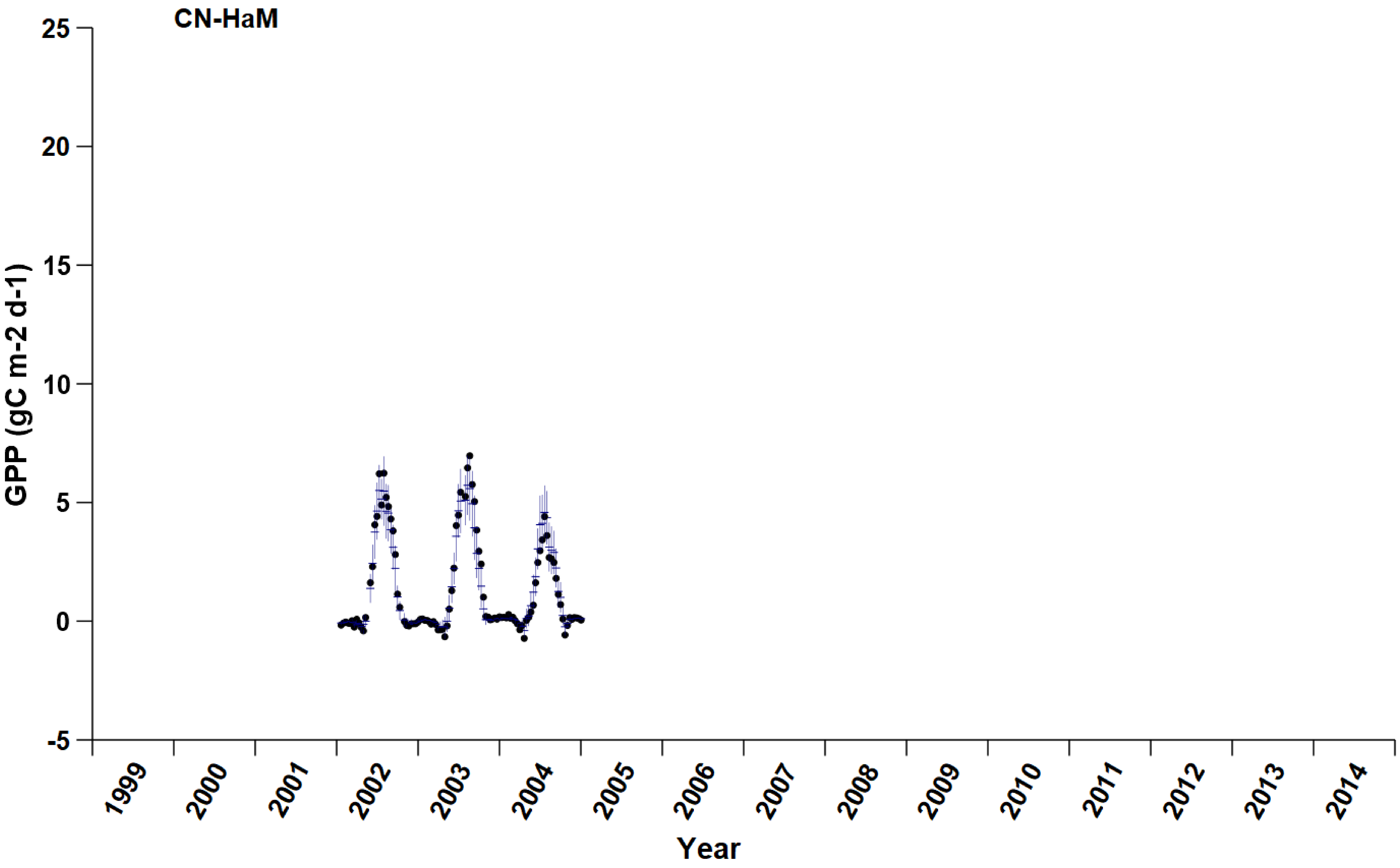

CN-HaM
GPP (gC m-2 d-1)
25
20
15
10
5
0
-5
1999
2000
2001
2002
2003
2004
2005
2006
2007
2008
2009
2010
2011
2012
2013
2014
Year

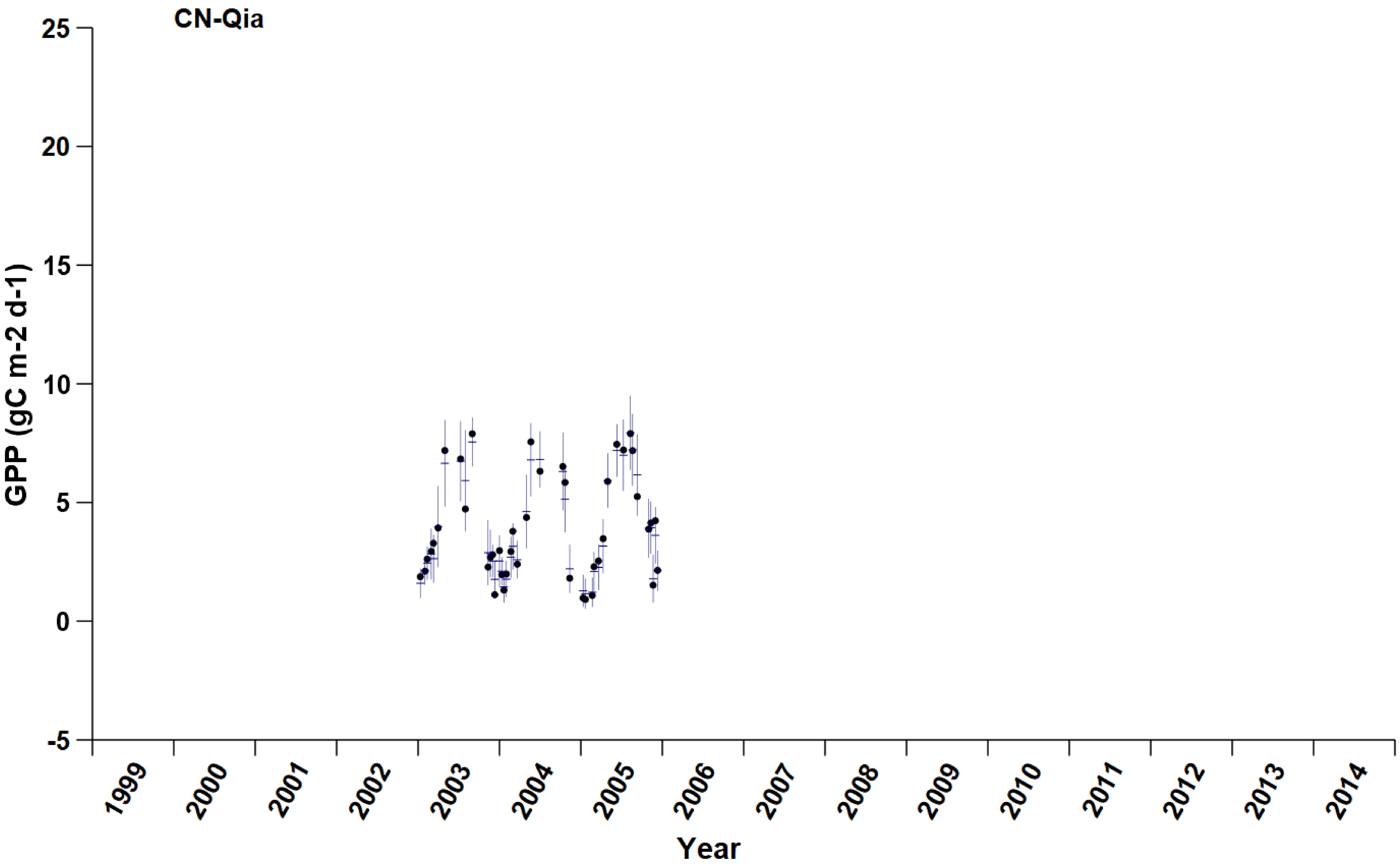
CN-Qia
GPP (gC m-2 d-1)
25
20
15
10
5
0
-5
1999
2000
2001
2002
2003
2004
2005
2006
2007
2008
2009
2010
2011
2012
2013
2014
Year

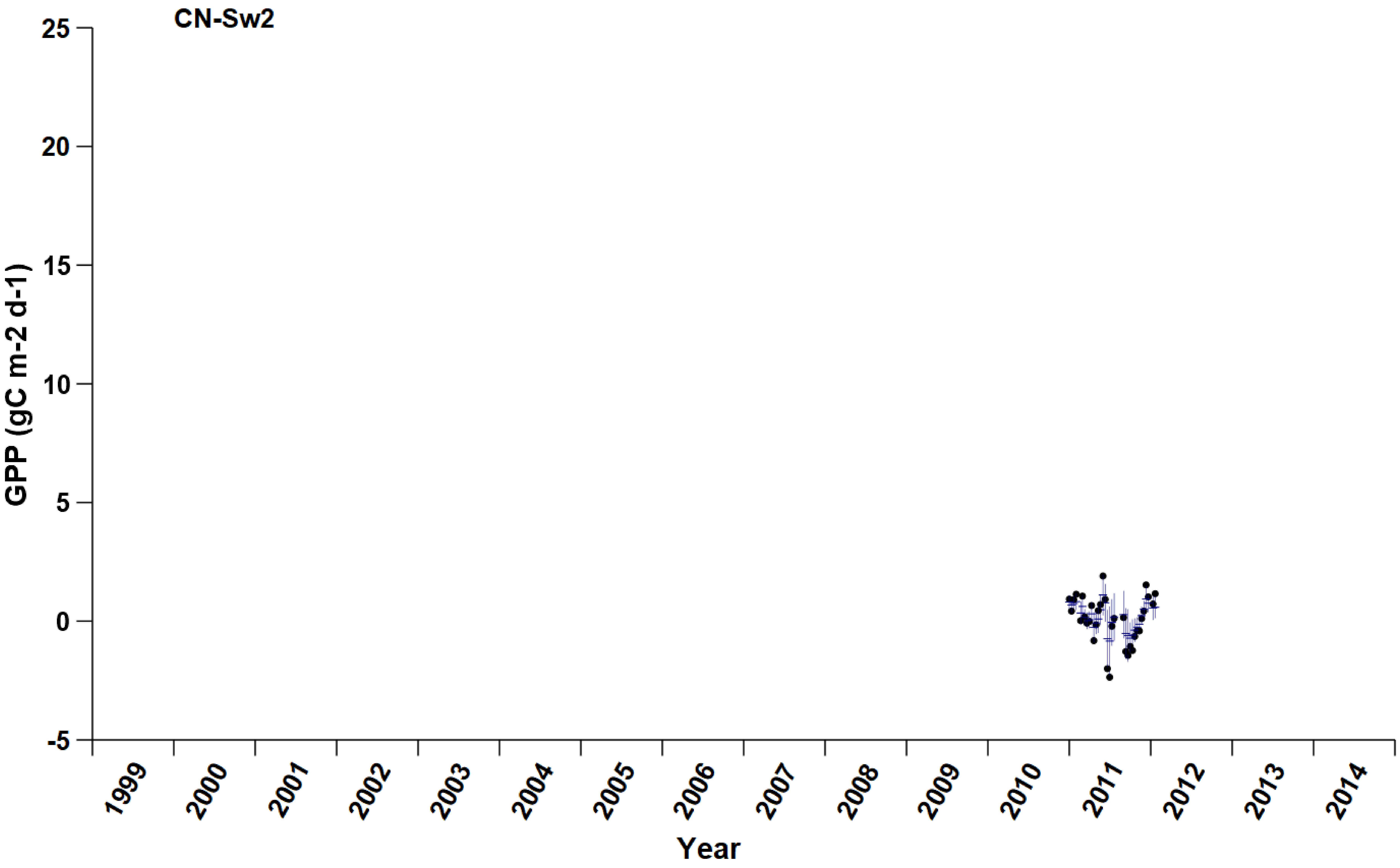
CN-Sw2
GPP (gC m-2 d-1)
25
20
15
10
5
0
-5
1999
2000
2001
2002
2003
2004
2005
2006
2007
2008
2009
2010
2011
2012
2013
2014
Year

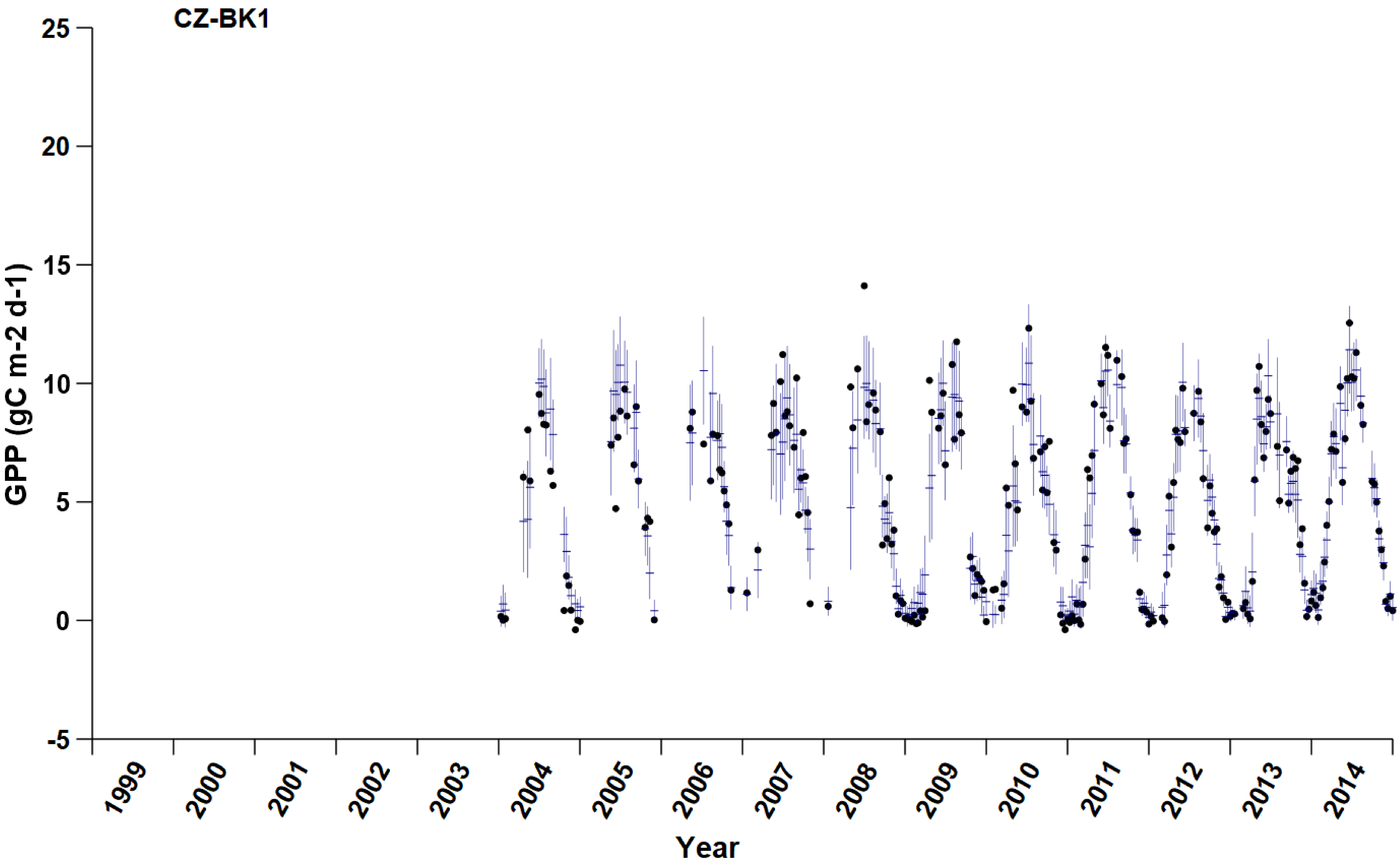
CZ-BK1
GPP (gC m-2 d-1)
25
20
15
10
5
0
-5
1999
2000
2001
2002
2003
2004
2005
2006
2007
2008
2009
2010
2011
2012
2013
2014
Year

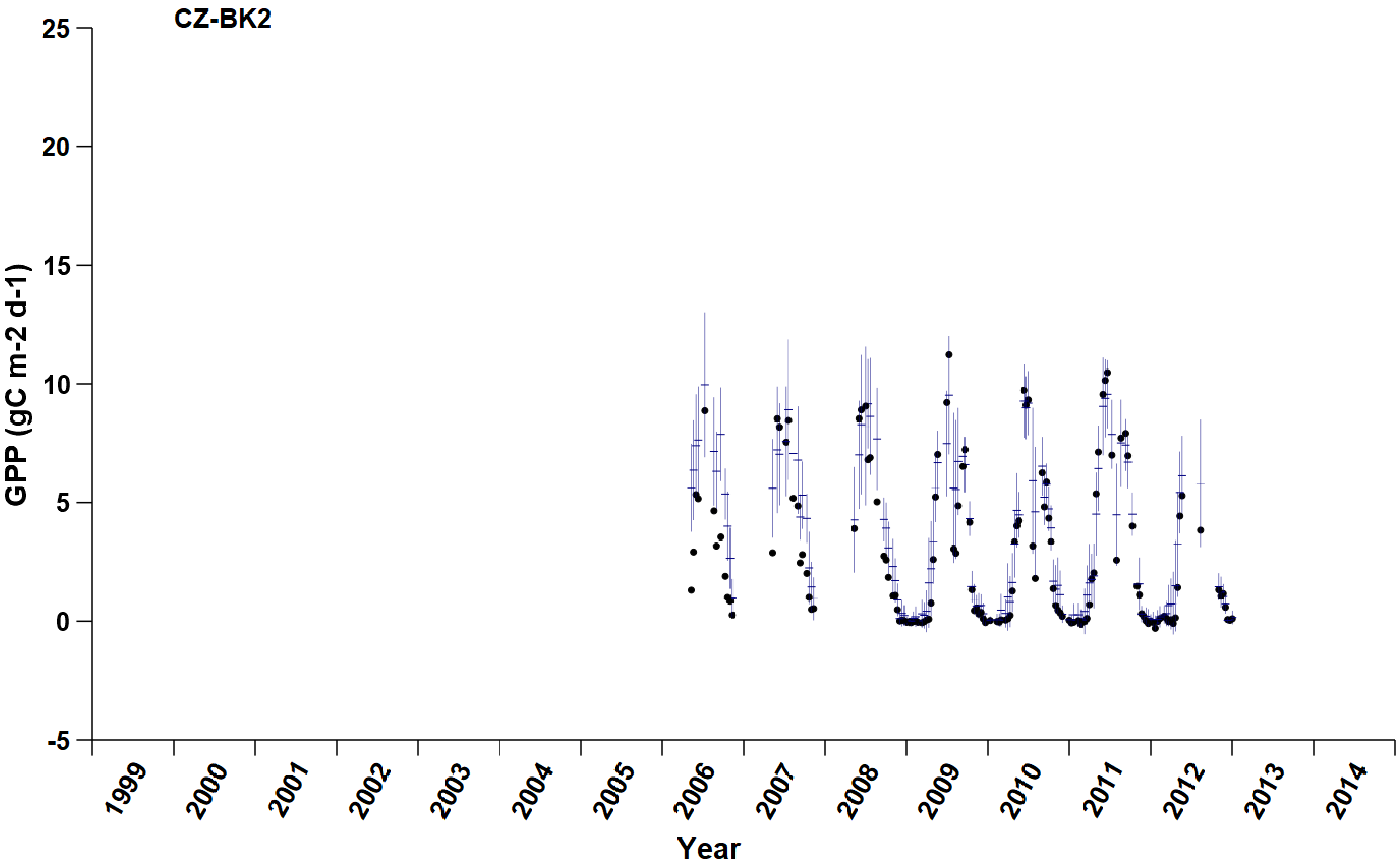
CZ-BK2
GPP (gC m-2 d-1)
25
20
15
10
5
0
-5
1999
2000
2001
2002
2003
2004
2005
2006
2007
2008
2009
2010
2011
2012
2013
2014
Year

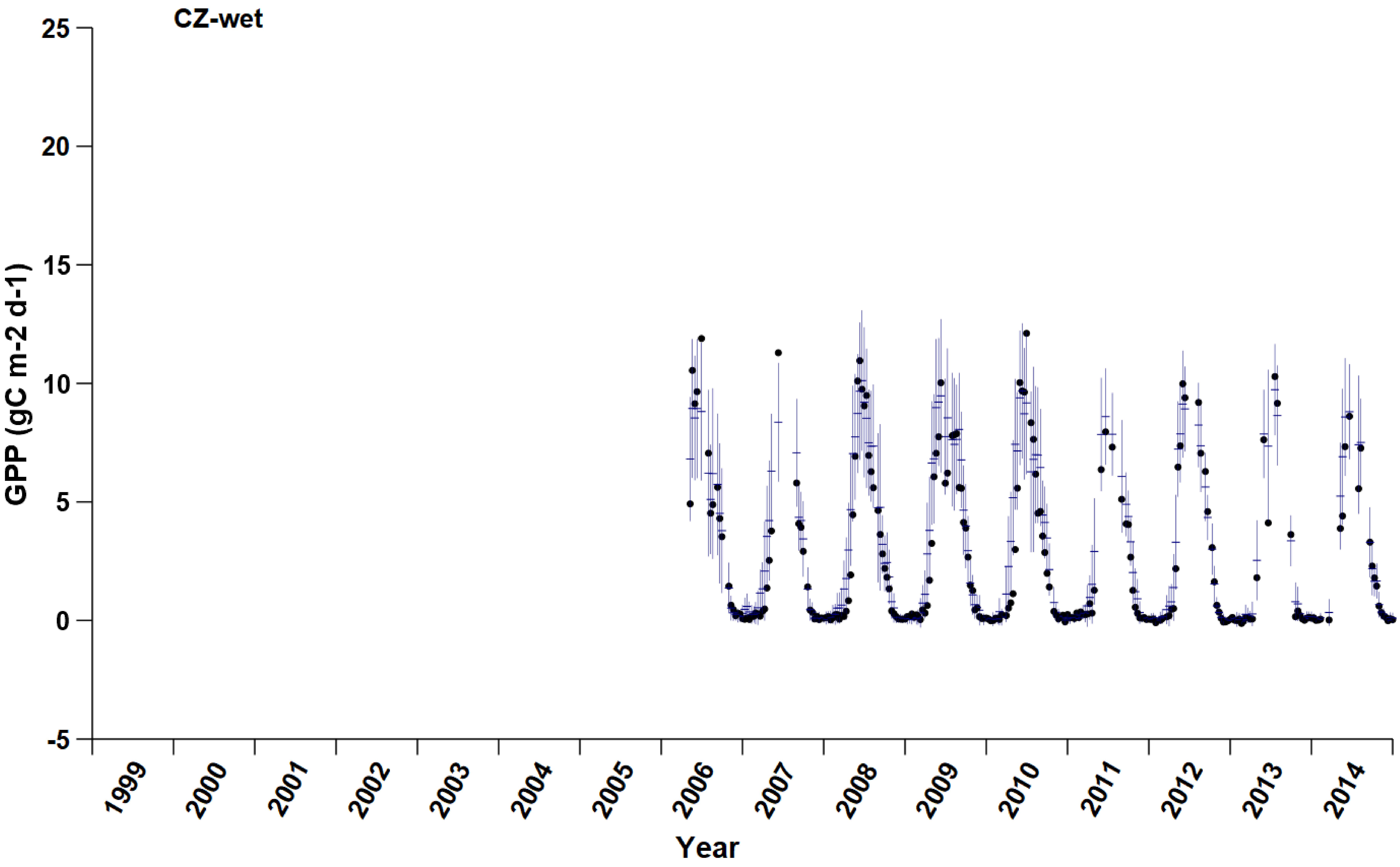

CZ-wet
GPP (gC m-2 d-1)
25
20
15
10
5
0
-5
1999
2000
2001
2002
2003
2004
2005
2006
2007
2008
2009
2010
2011
2012
2013
2014
Year

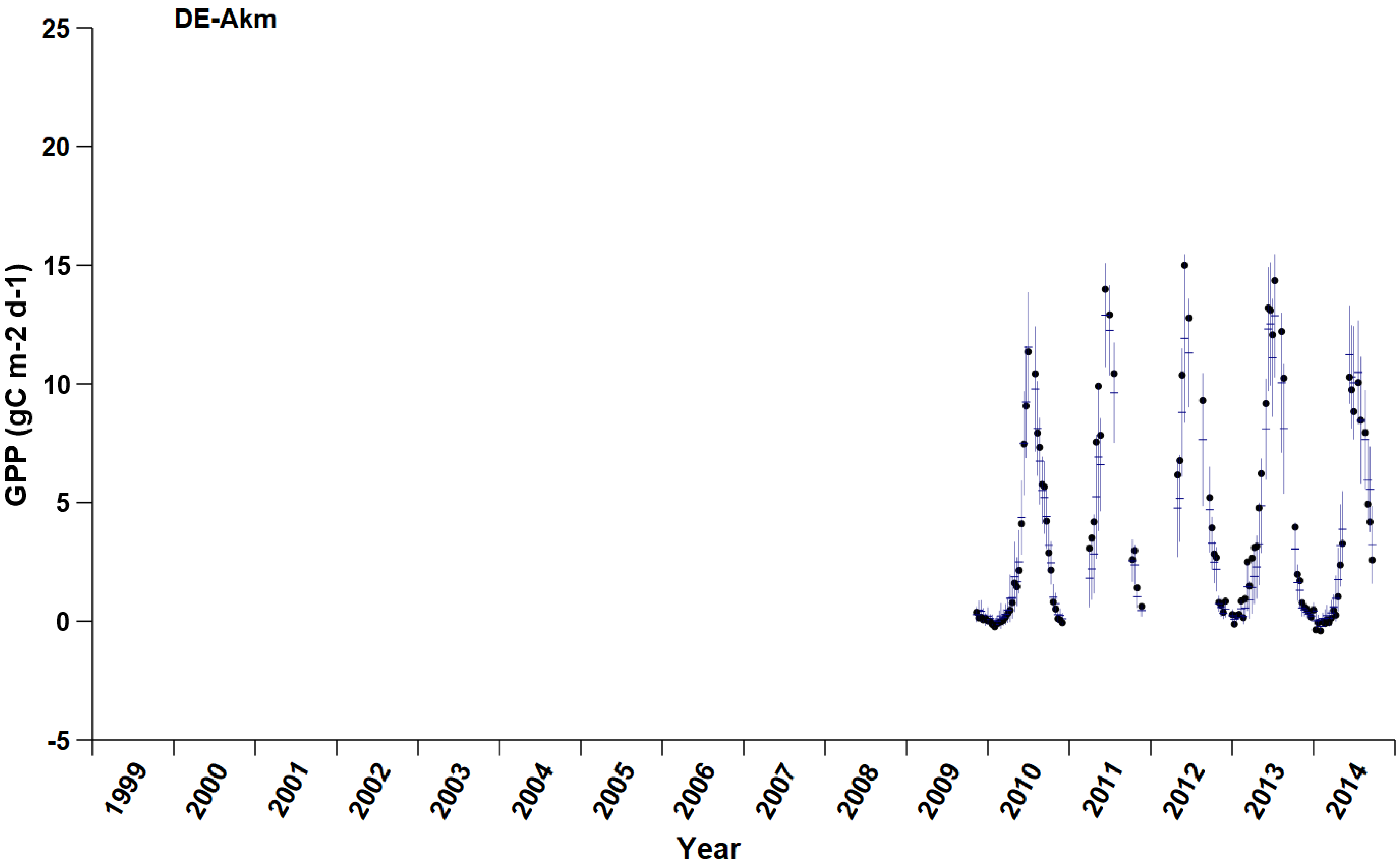
DE-Akm
GPP (gC m-2 d-1)
25
20
15
10
5
0
-5
1999
2000
2001
2002
2003
2004
2005
2006
2007
2008
2009
2010
2011
2012
2013
2014
Year

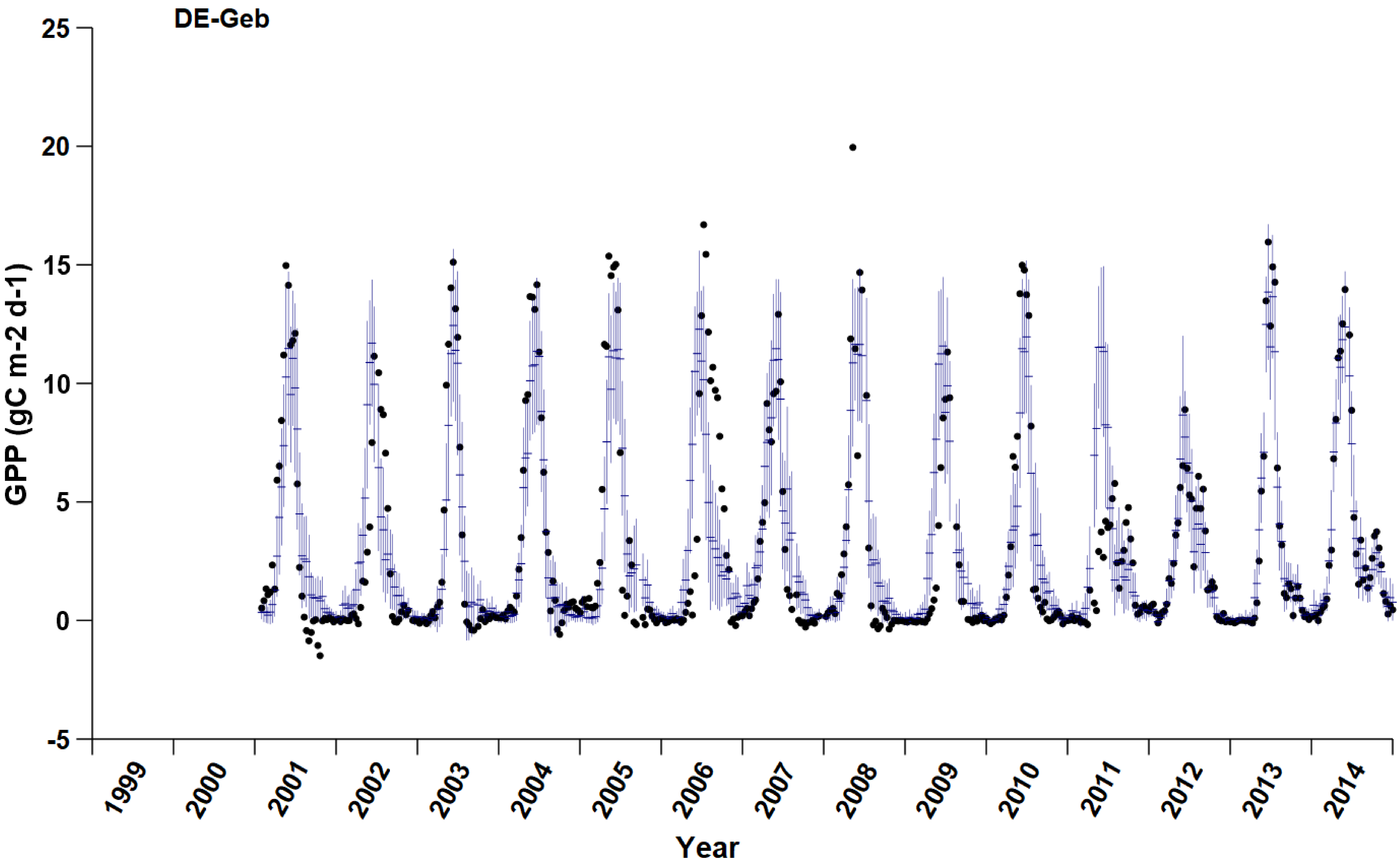
DE-Geb
GPP (gC m-2 d-1)
25
20
15
10
5
0
-5
1999
2000
2001
2002
2003
2004
2005
2006
2007
2008
2009
2010
2011
2012
2013
2014
Year

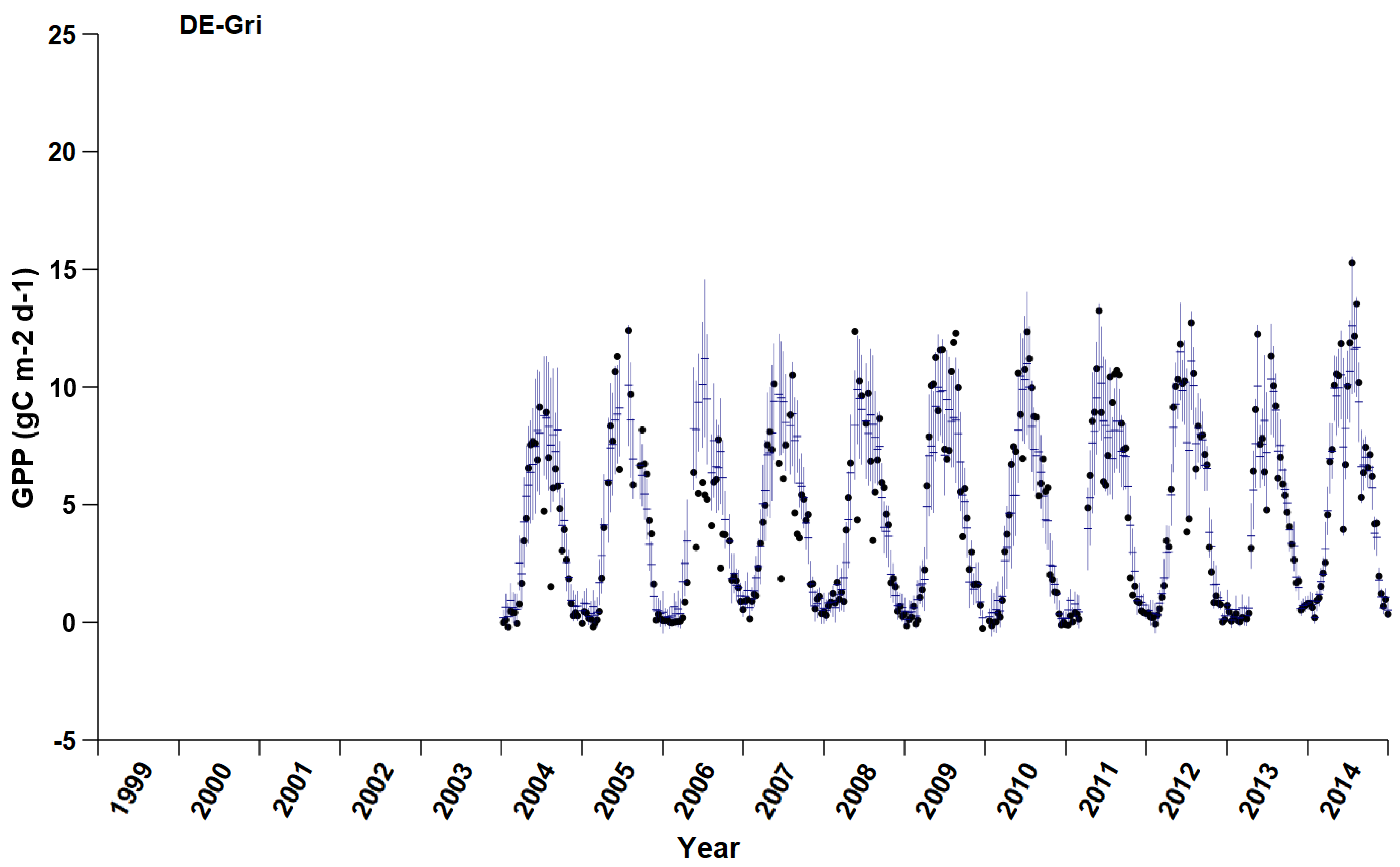

DE-Gri
GPP (gC m-2 d-1)
25
20
15
10
5
0
-5
1999
2000
2001
2002
2003
2004
2005
2006
2007
2008
2009
2010
2011
2012
2013
2014
Year

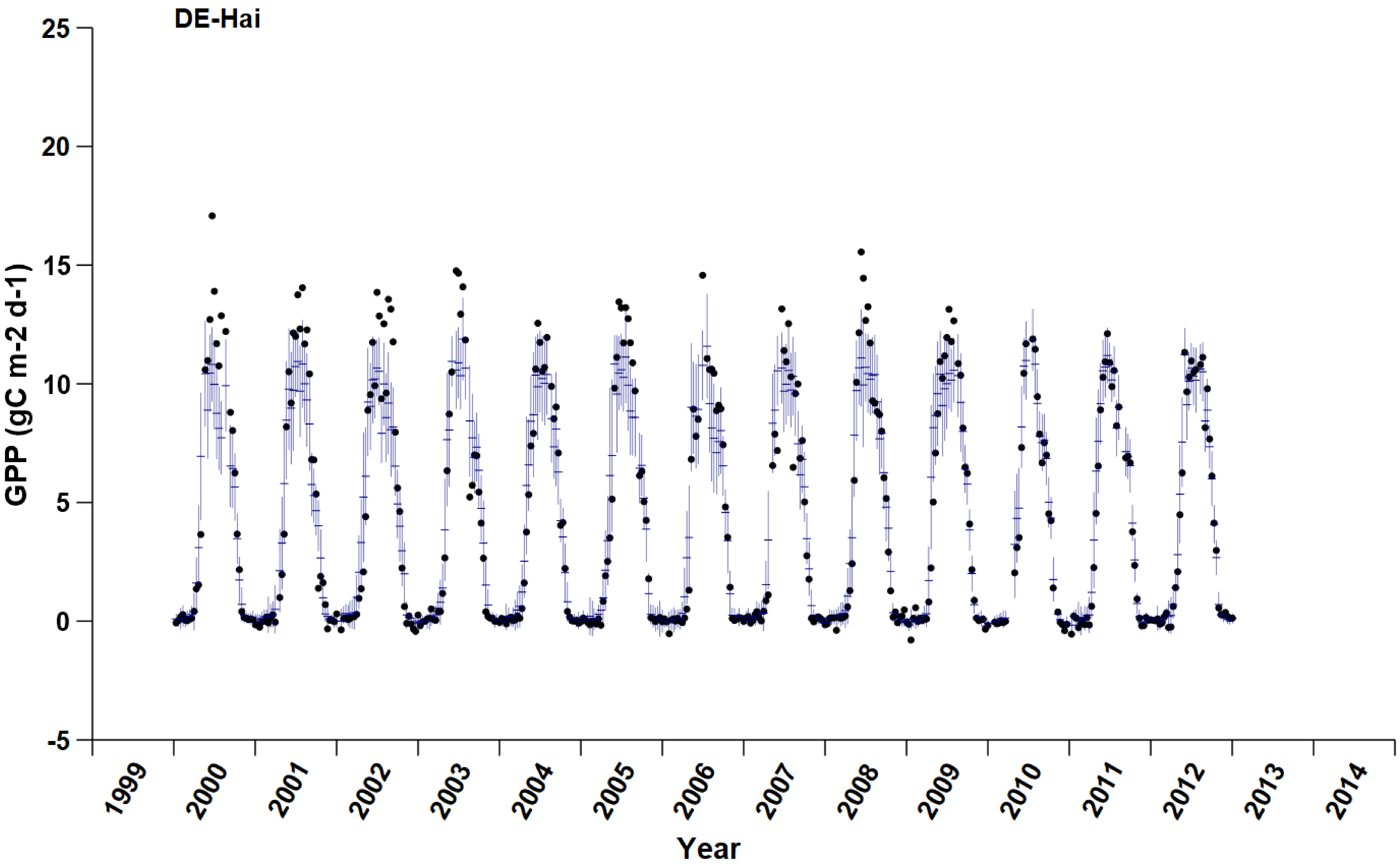

DE-Hai
GPP (gC m-2 d-1)
25
20
15
10
5
0
-5
1999
2000
2001
2002
2003
2004
2005
2006
2007
2008
2009
2010
2011
2012
2013
2014
Year

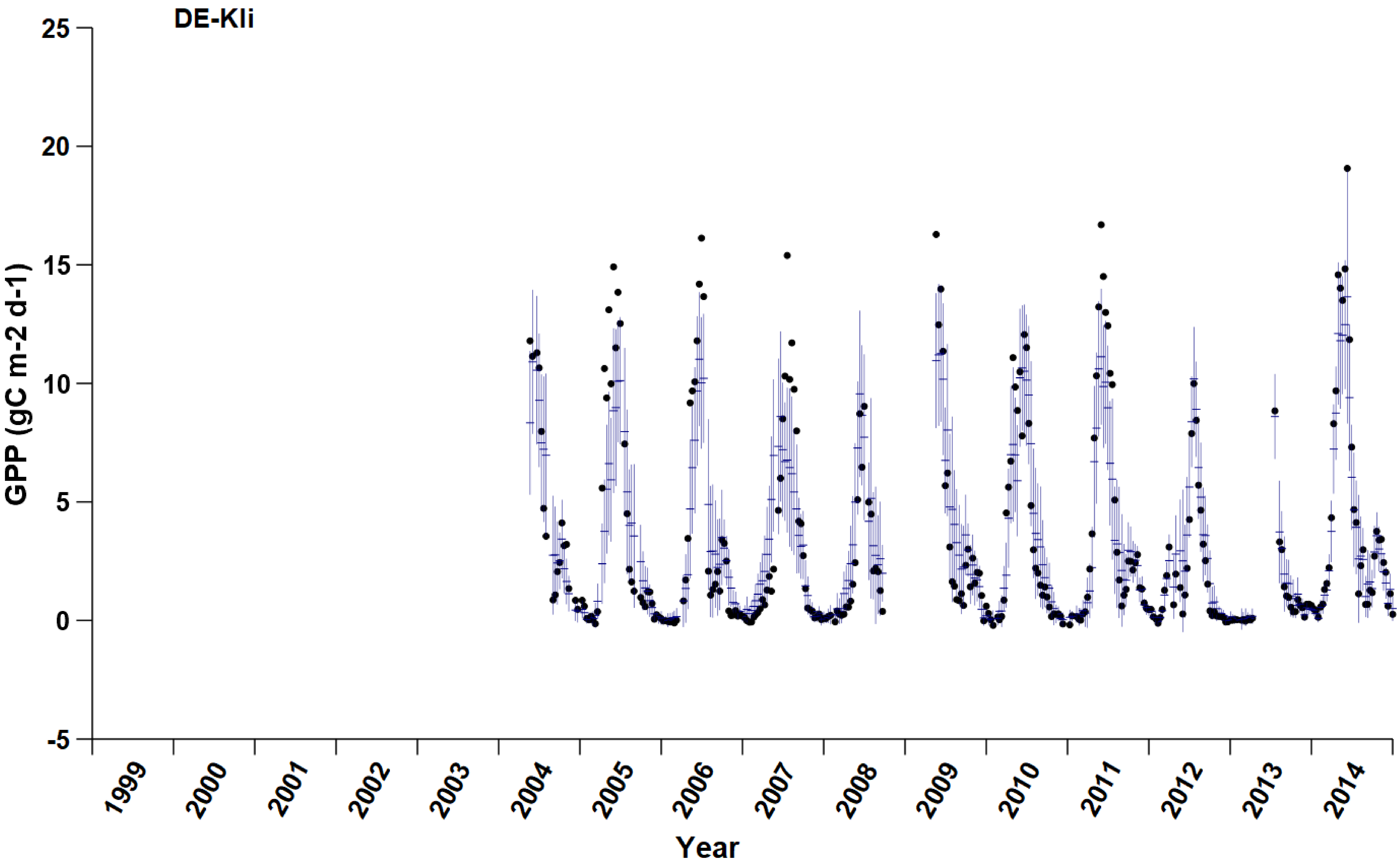
DE-Kli
GPP (gC m-2 d-1)
25
20
15
10
5
0
-5
1999
2000
2001
2002
2003
2004
2005
2006
2007
2008
2009
2010
2011
2012
2013
2014
Year

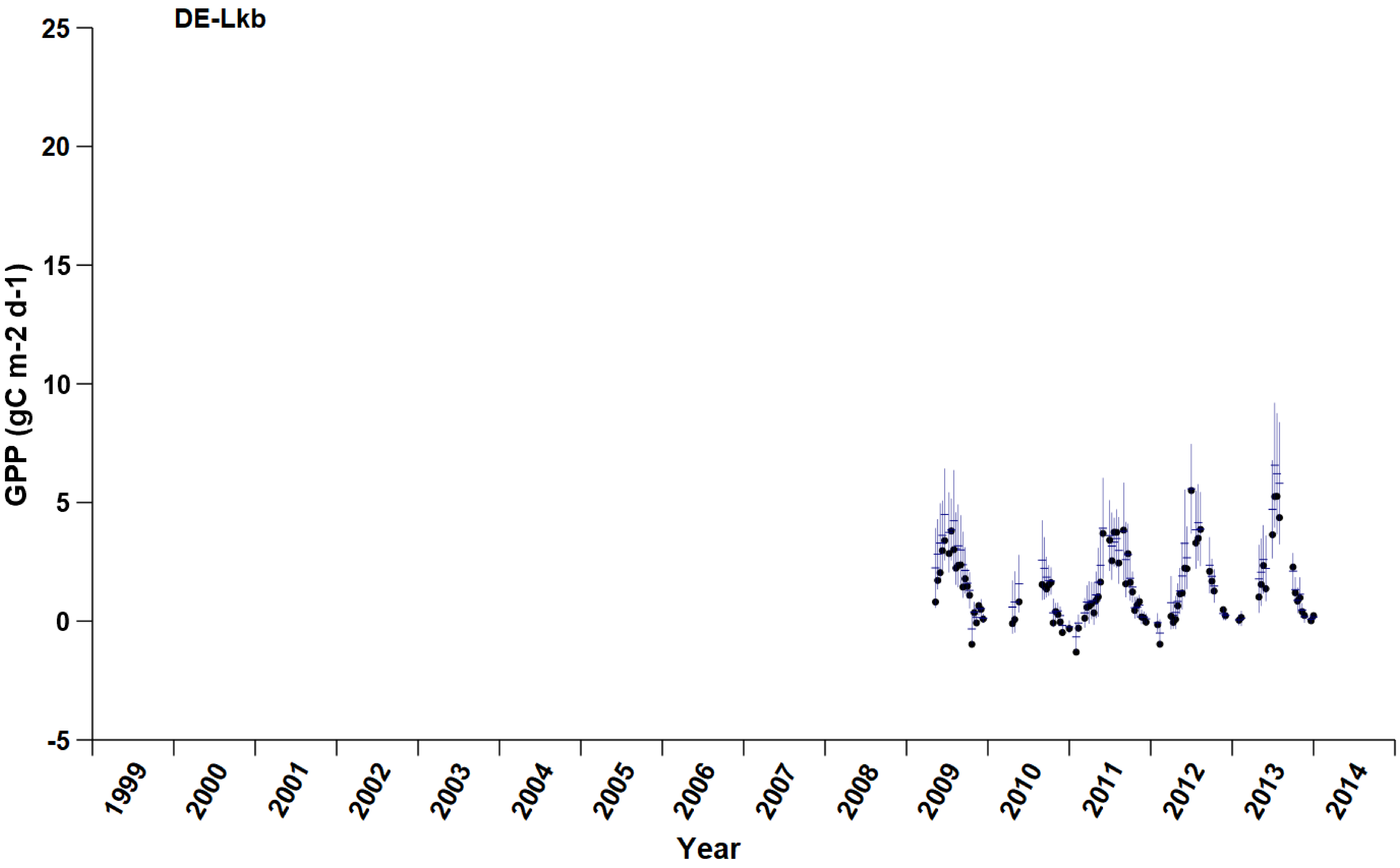

DE-Lkb
GPP (gC m-2 d-1)
25
20
15
10
5
0
-5
1999
2000
2001
2002
2003
2004
2005
2006
2007
2008
2009
2010
2011
2012
2013
2014
Year

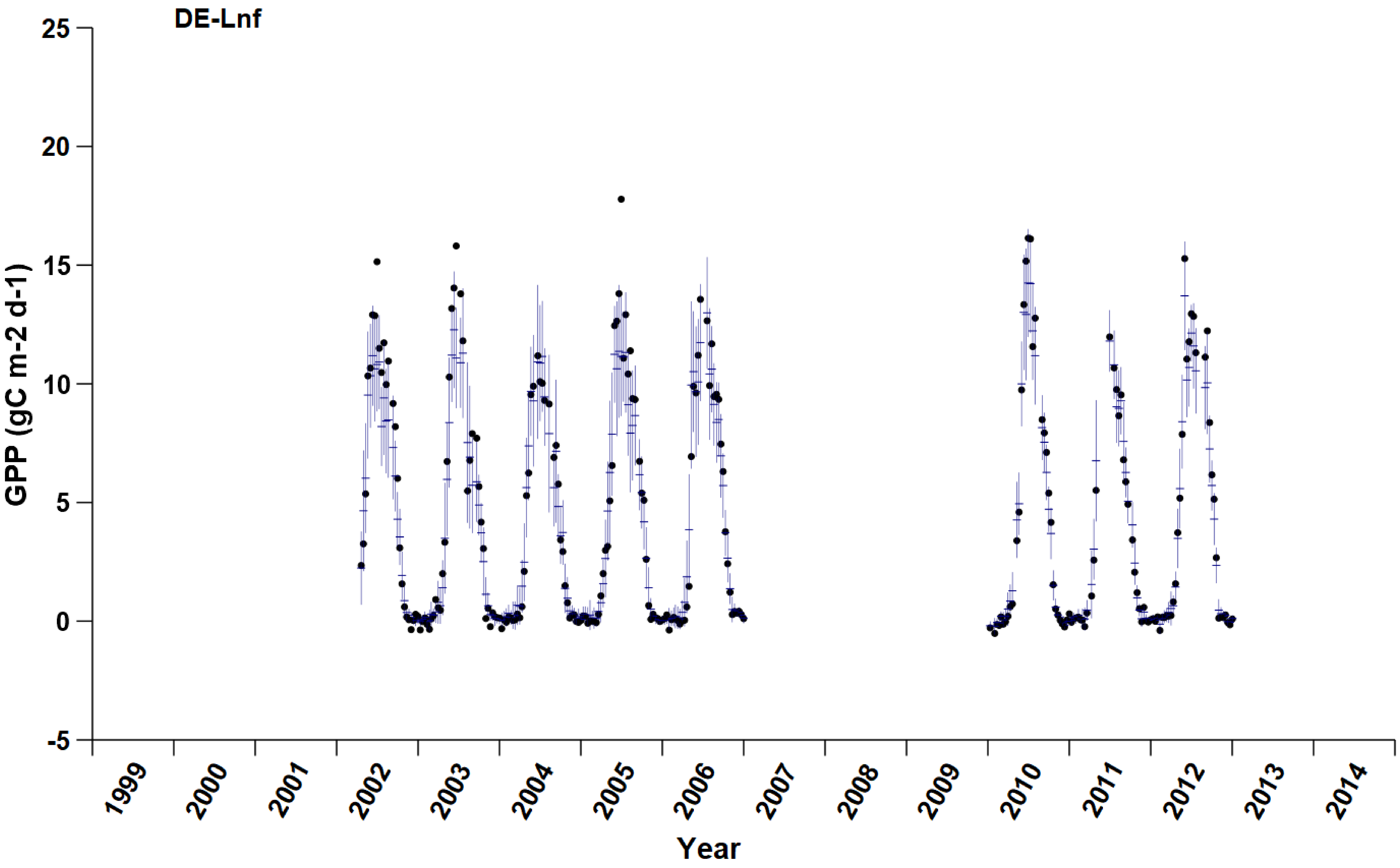
DE-Lnf
GPP (gC m-2 d-1)
25
20
15
10
5
0
-5
1999
2000
2001
2002
2003
2004
2005
2006
2007
2008
2009
2010
2011
2012
2013
2014
Year

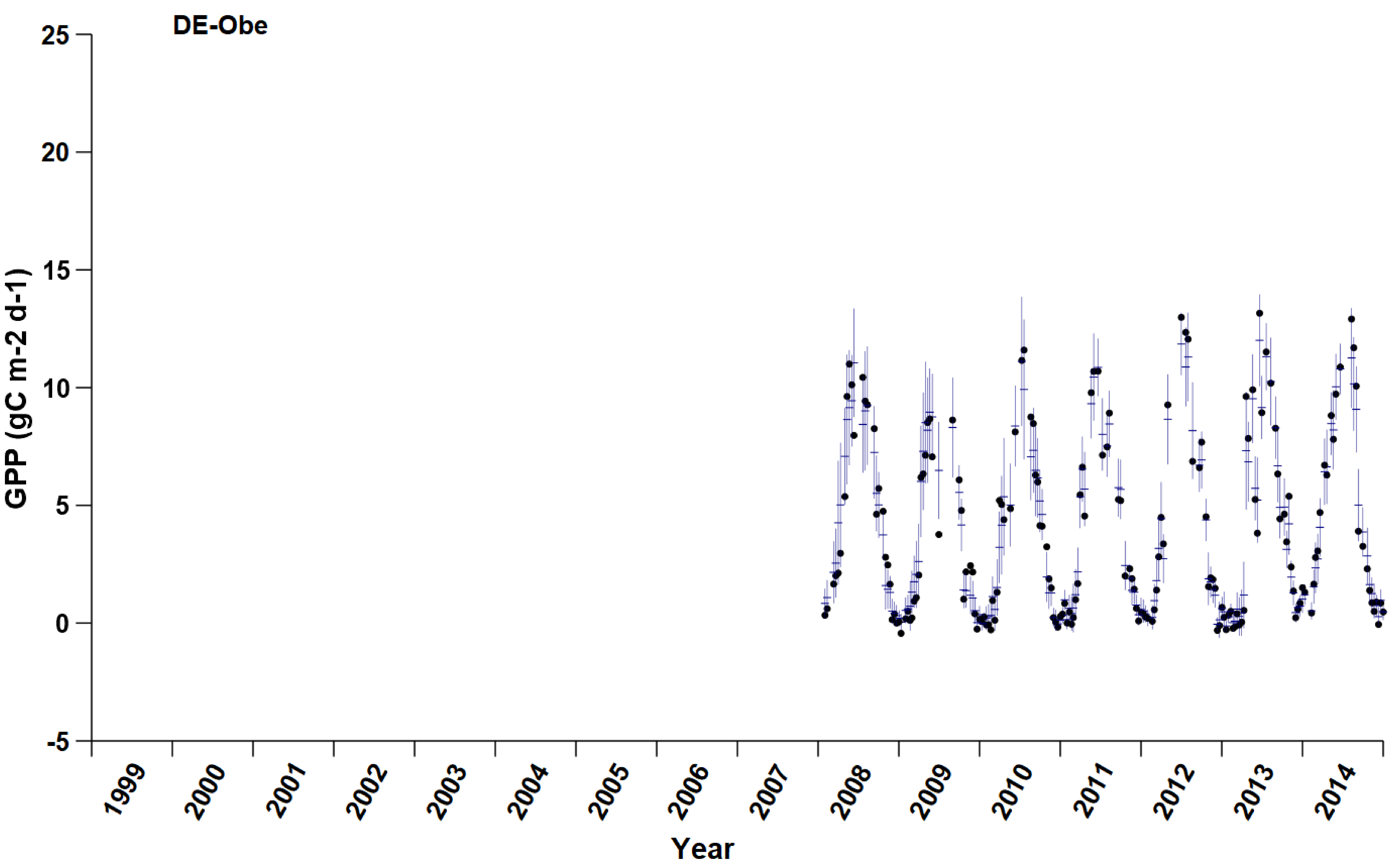

DE-Obe
GPP (gC m-2 d-1)
25
20
15
10
5
0
-5
1999
2000
2001
2002
2003
2004
2005
2006
2007
2008
2009
2010
2011
2012
2013
2014
Year

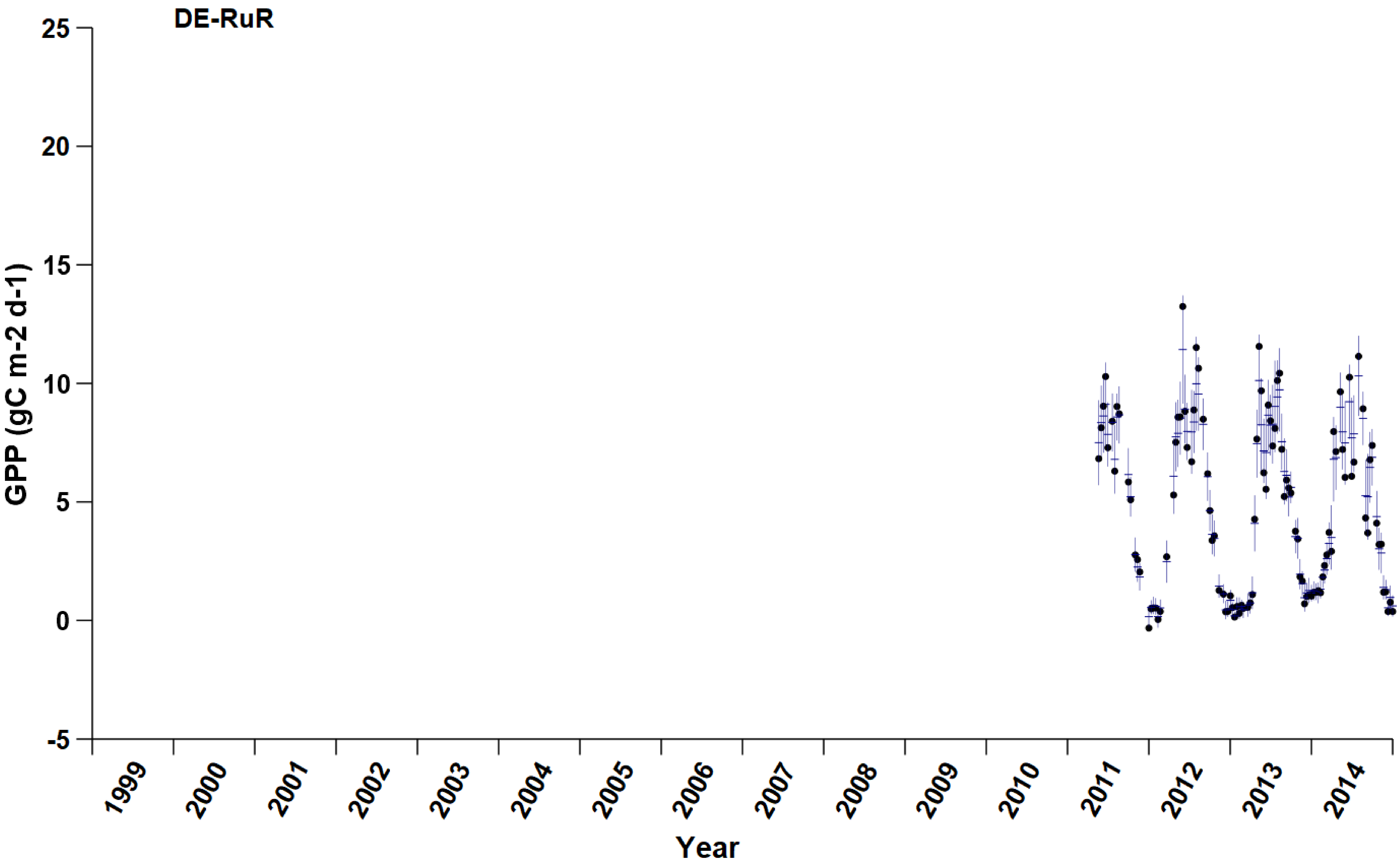

DE-RuR
GPP (gC m-2 d-1)
25
20
15
10
5
0
-5
1999
2000
2001
2002
2003
2004
2005
2006
2007
2008
2009
2010
2011
2012
2013
2014
Year

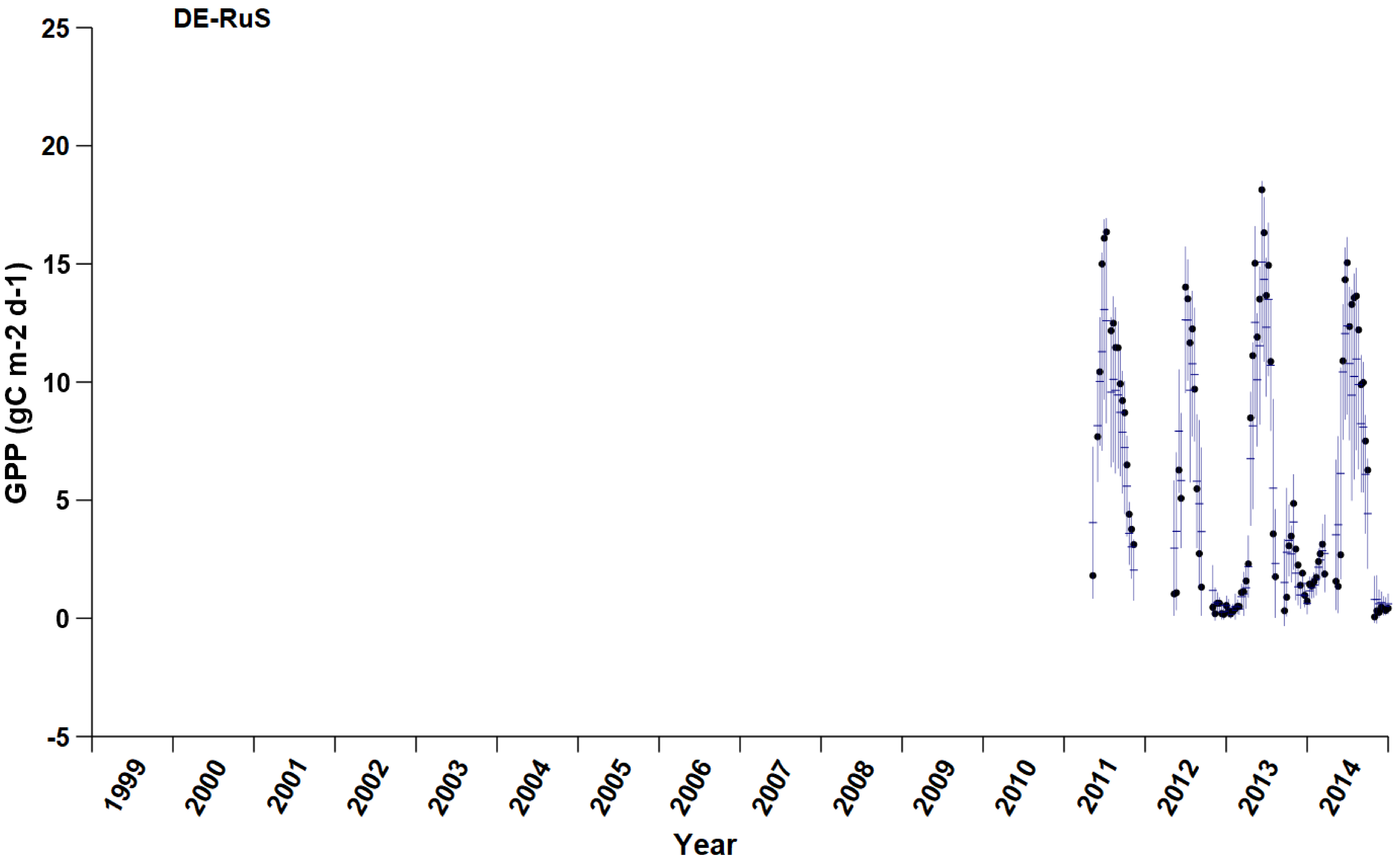
DE-RuS
GPP (gC m-2 d-1)
25
20
15
10
5
0
-5
1999
2000
2001
2002
2003
2004
2005
2006
2007
2008
2009
2010
2011
2012
2013
2014
Year

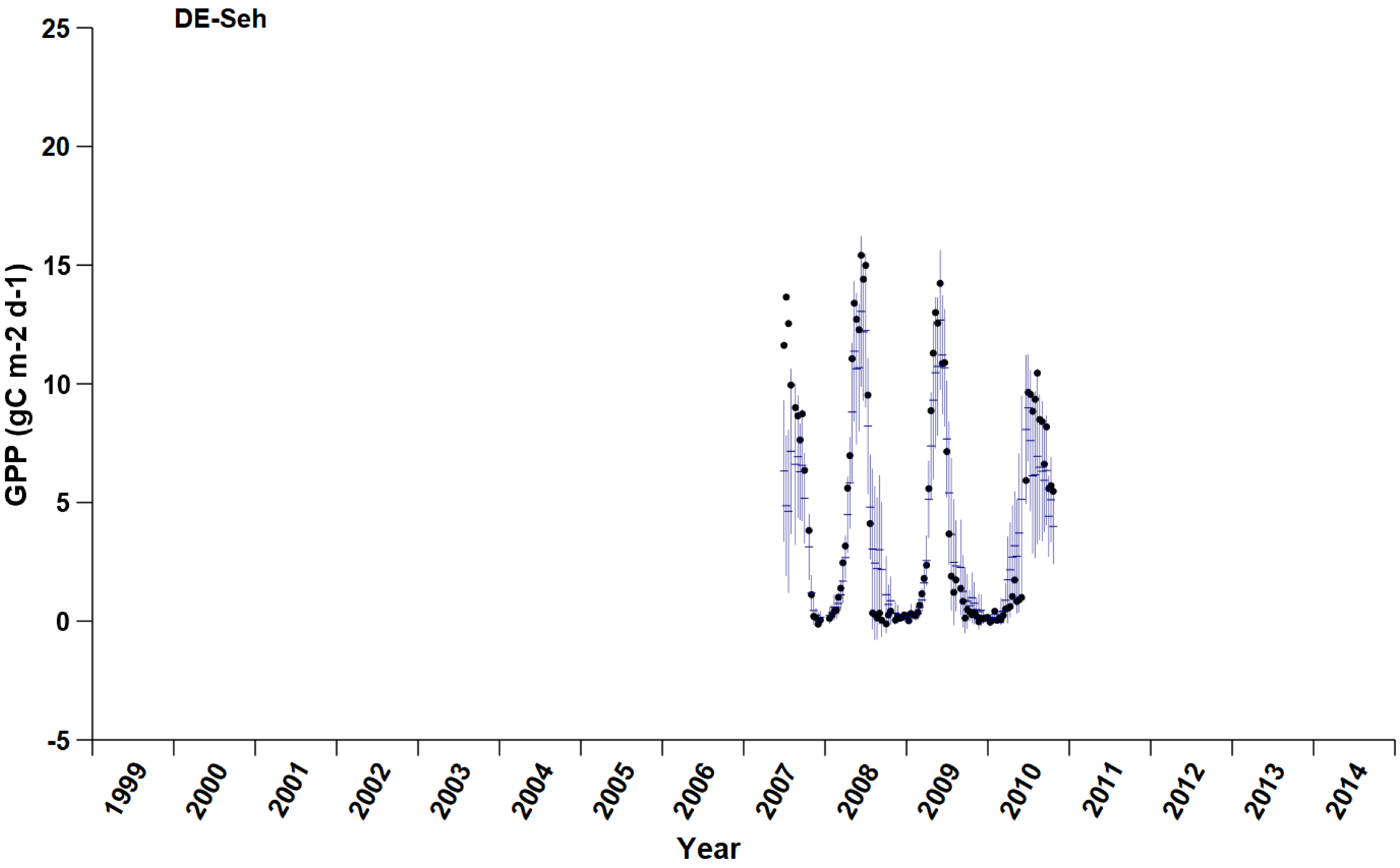

DE-Seh
GPP (gC m-2 d-1)
25
20
15
10
5
0
-5
1999
2000
2001
2002
2003
2004
2005
2006
2007
2008
2009
2010
2011
2012
2013
2014
Year

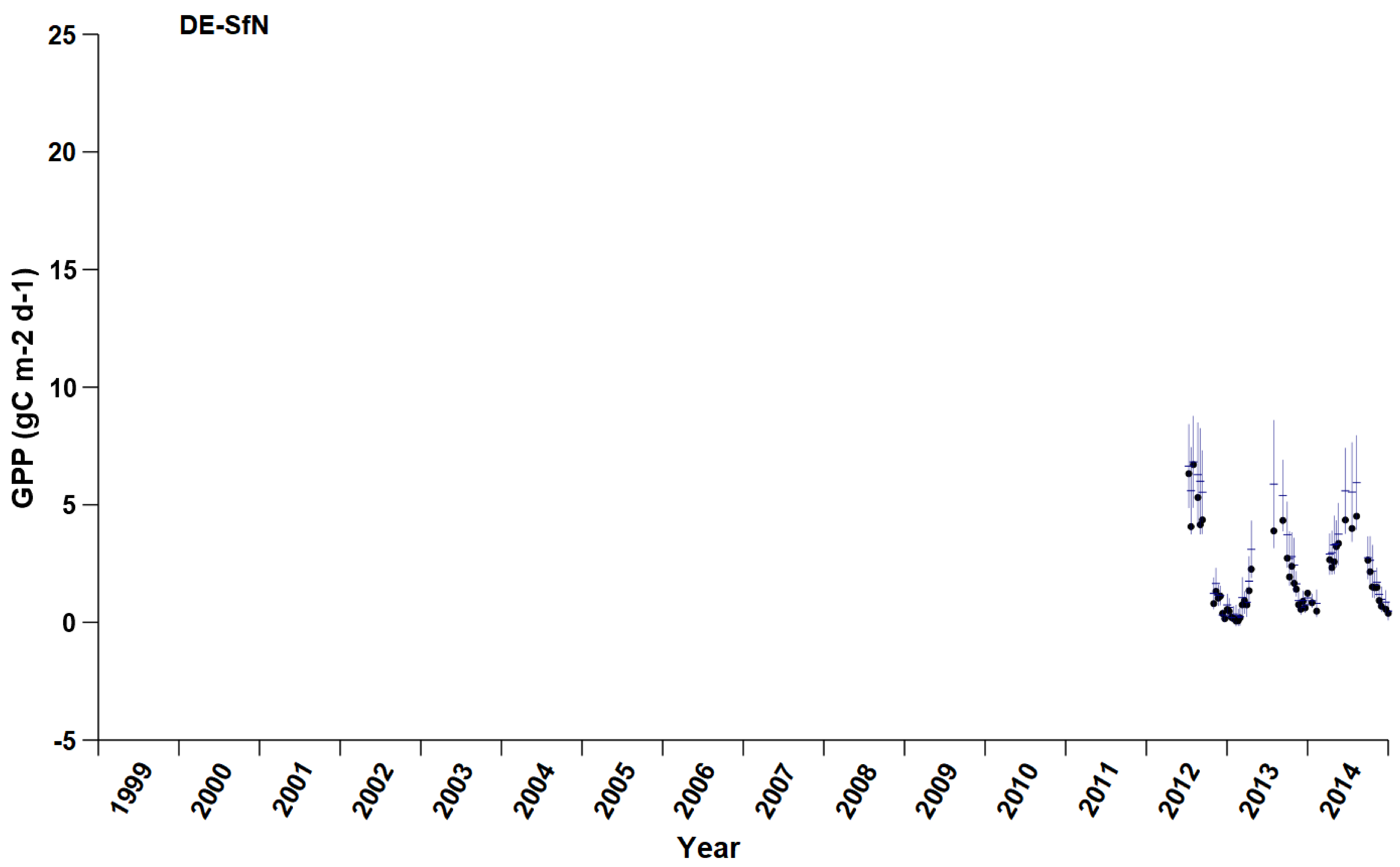
DE-SfN
GPP (gC m-2 d-1)
25
20
15
10
5
0
-5
1999
2000
2001
2002
2003
2004
2005
2006
2007
2008
2009
2010
2011
2012
2013
2014
Year

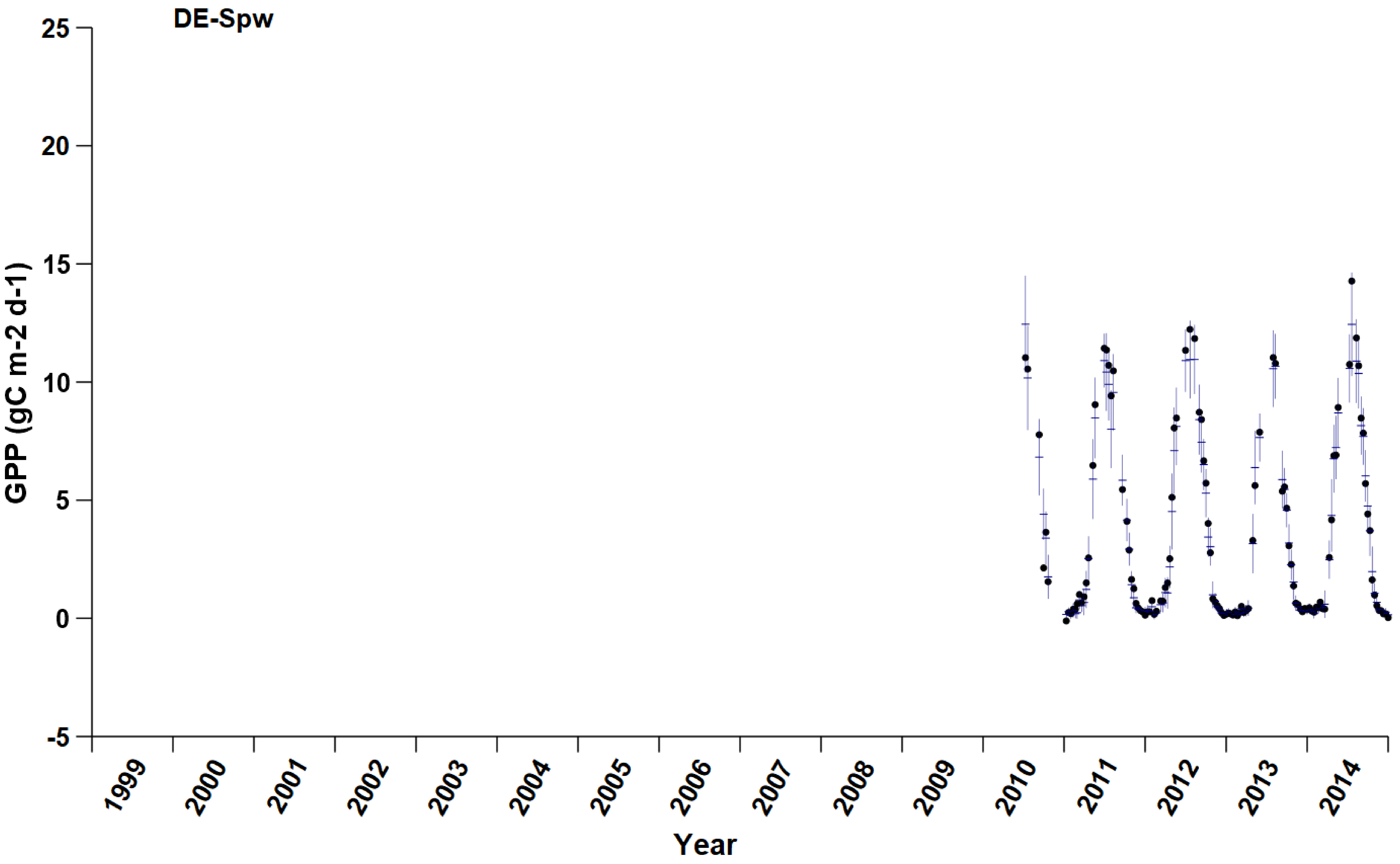
DE-Spw
GPP (gC m-2 d-1)
25
20
15
10
5
0
-5
1999
2000
2001
2002
2003
2004
2005
2006
2007
2008
2009
2010
2011
2012
2013
2014
Year

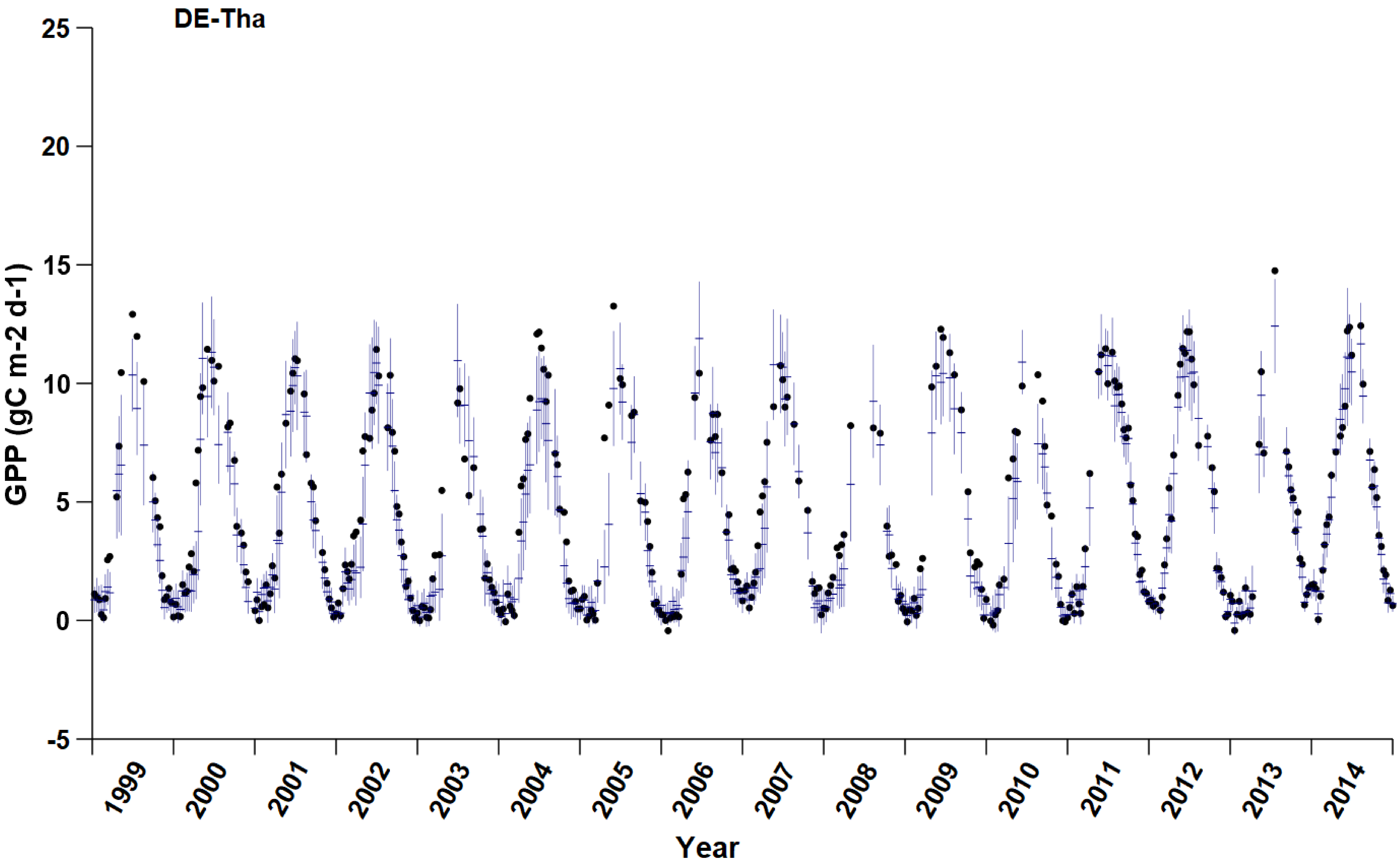

DE-Tha
GPP (gC m-2 d-1)
25
20
15
10
5
0
-5
1999
2000
2001
2002
2003
2004
2005
2006
2007
2008
2009
2010
2011
2012
2013
2014
Year

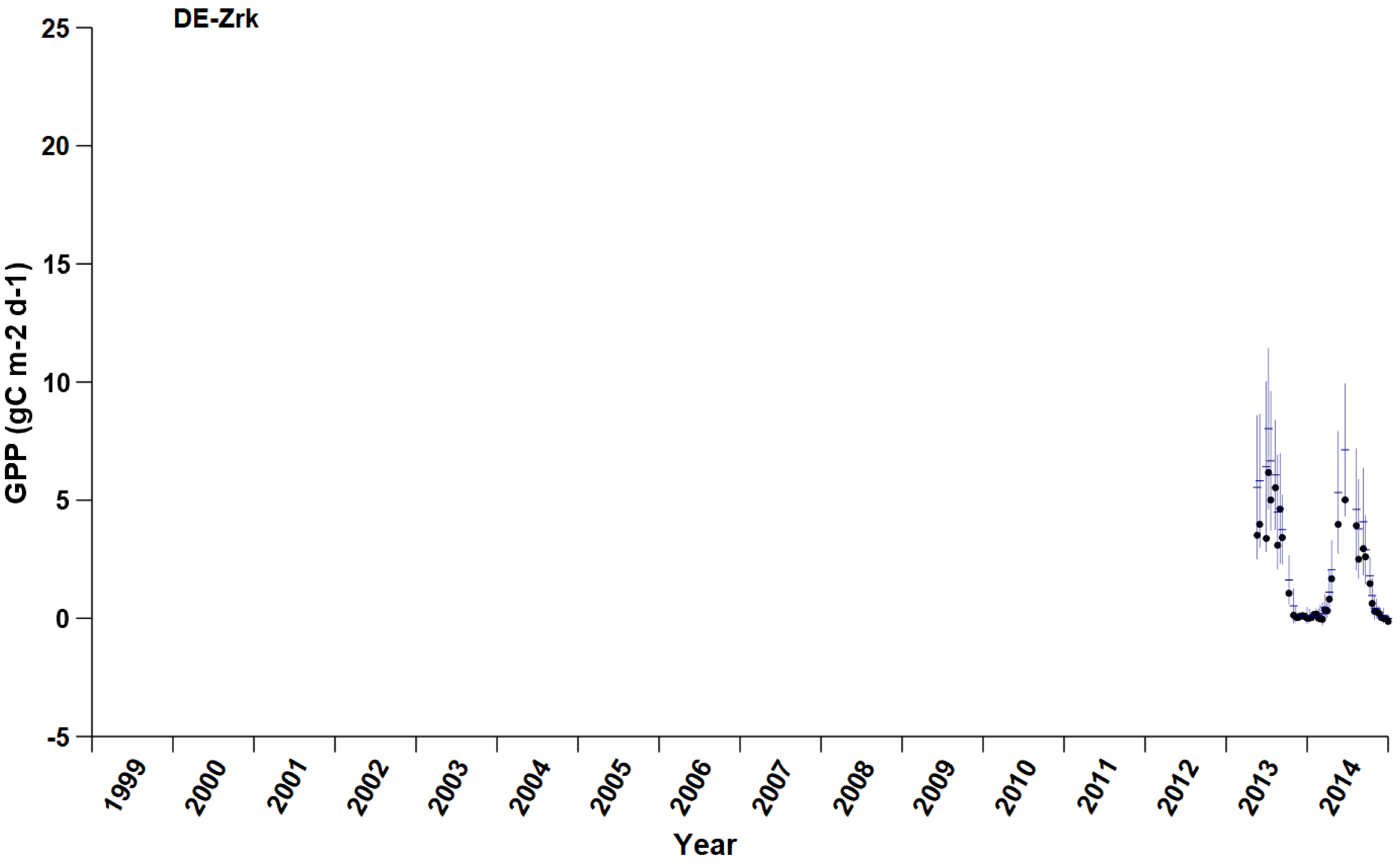

DE-Zrk
GPP (gC m-2 d-1)
25
20
15
10
5
0
-5
1999
2000
2001
2002
2003
2004
2005
2006
2007
2008
2009
2010
2011
2012
2013
2014
Year

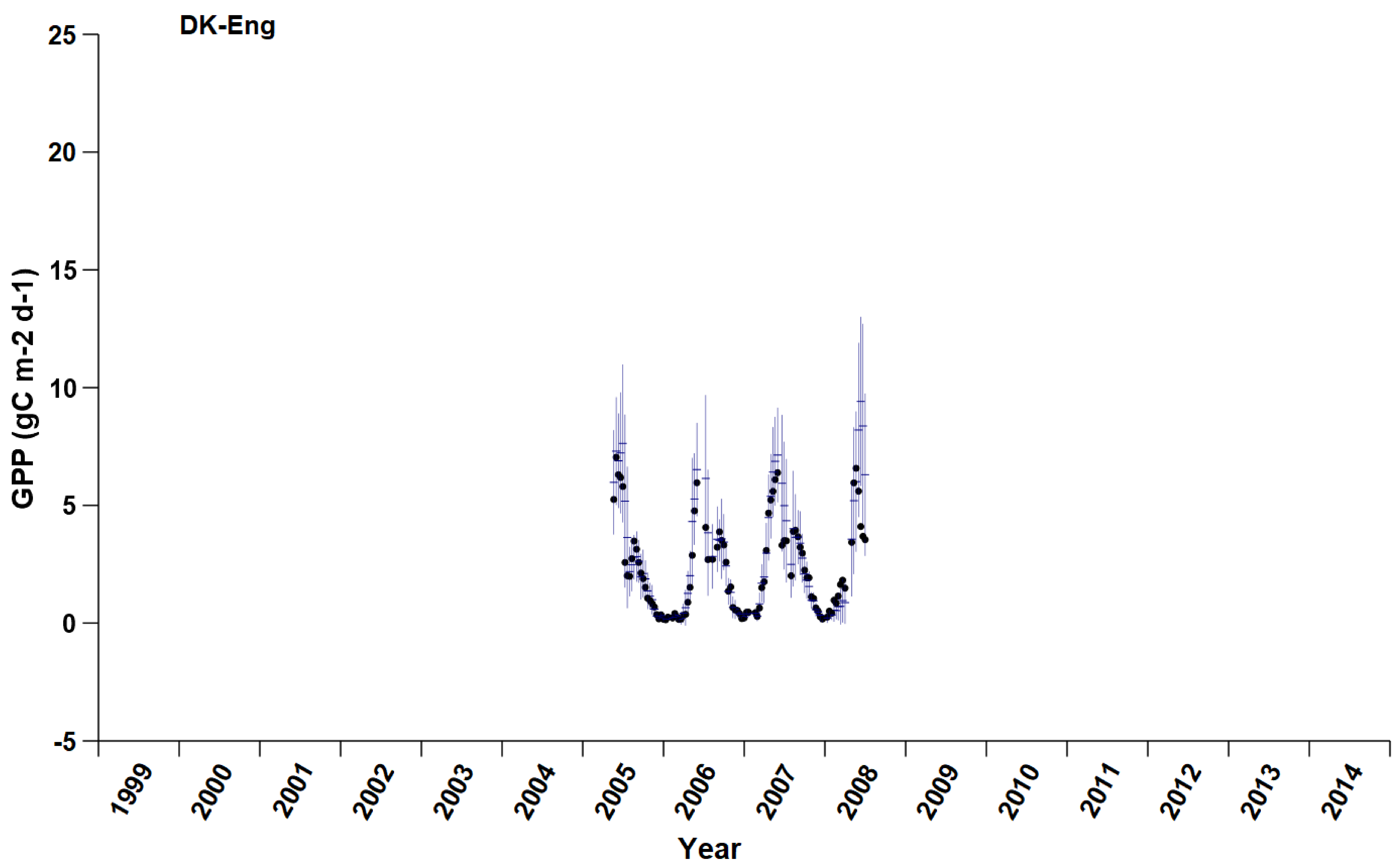
DK-Eng
GPP (gC m-2 d-1)
25
20
15
10
5
0
-5
1999
2000
2001
2002
2003
2004
2005
2006
2007
2008
2009
2010
2011
2012
2013
2014
Year

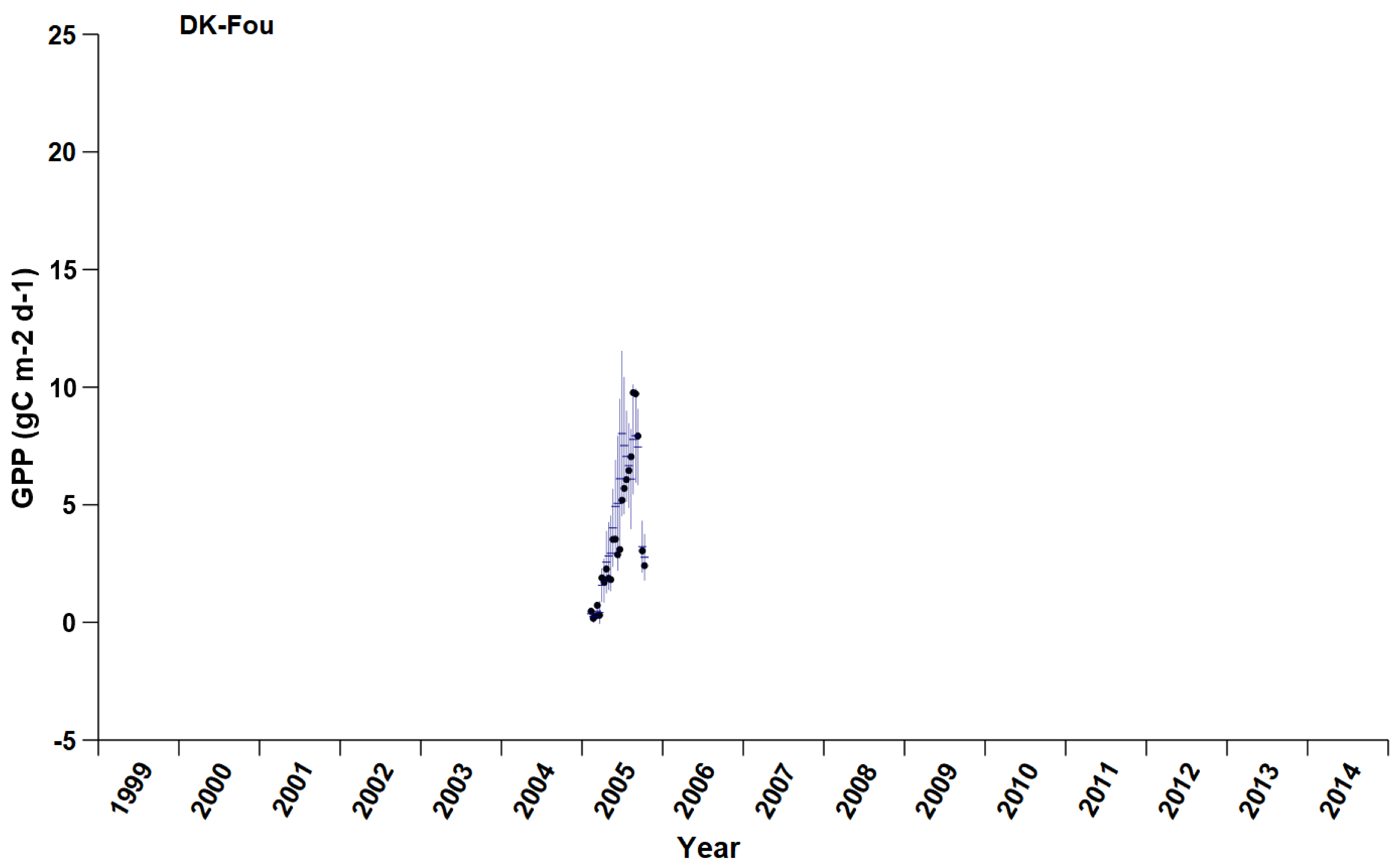

DK-Fou
GPP (gC m-2 d-1)
25
20
15
10
5
0
-5
1999
2000
2001
2002
2003
2004
2005
2006
2007
2008
2009
2010
2011
2012
2013
2014
Year

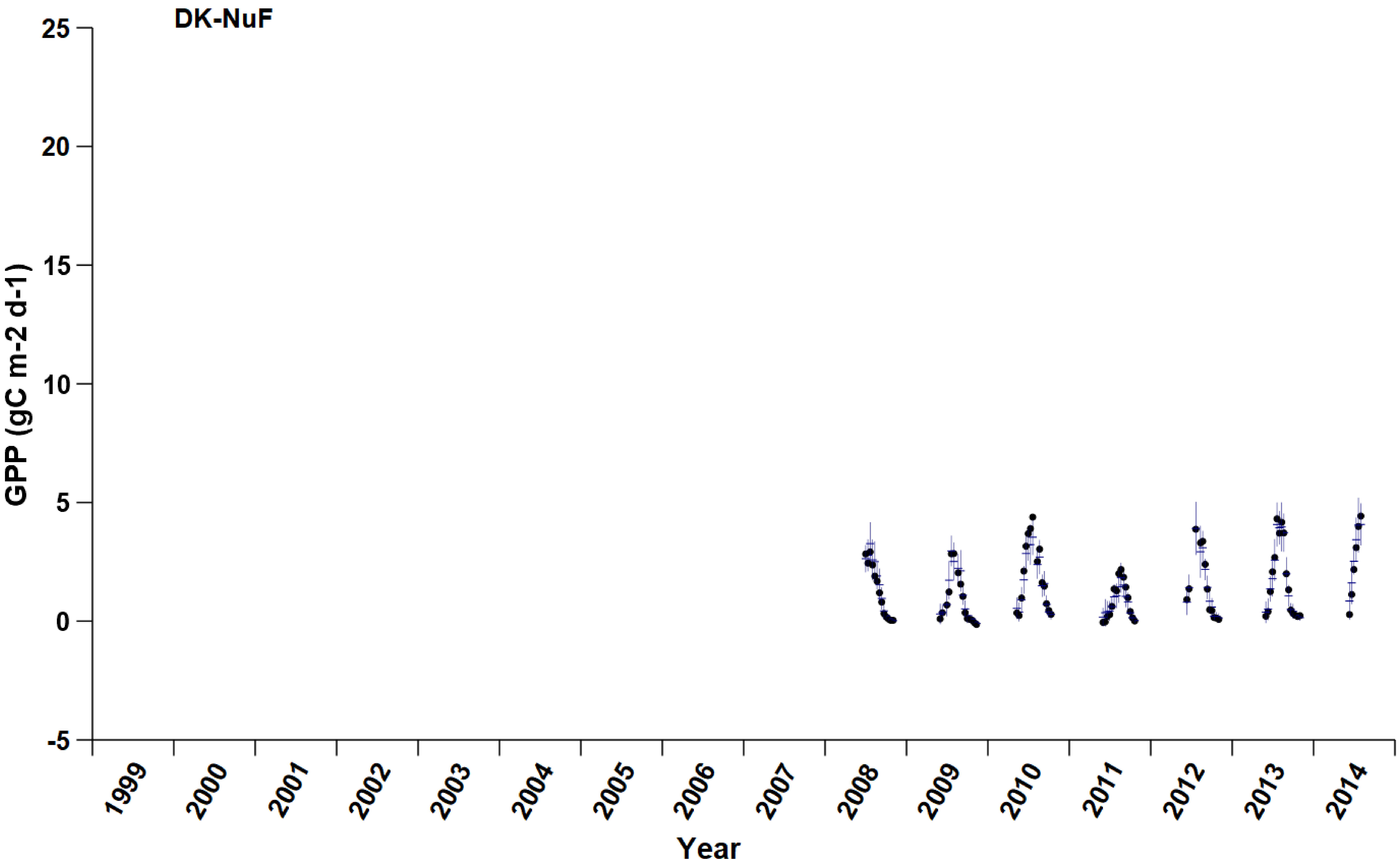
DK-NuF
GPP (gC m-2 d-1)
25
20
15
10
5
0
-5
1999
2000
2001
2002
2003
2004
2005
2006
2007
2008
2009
2010
2011
2012
2013
2014
Year

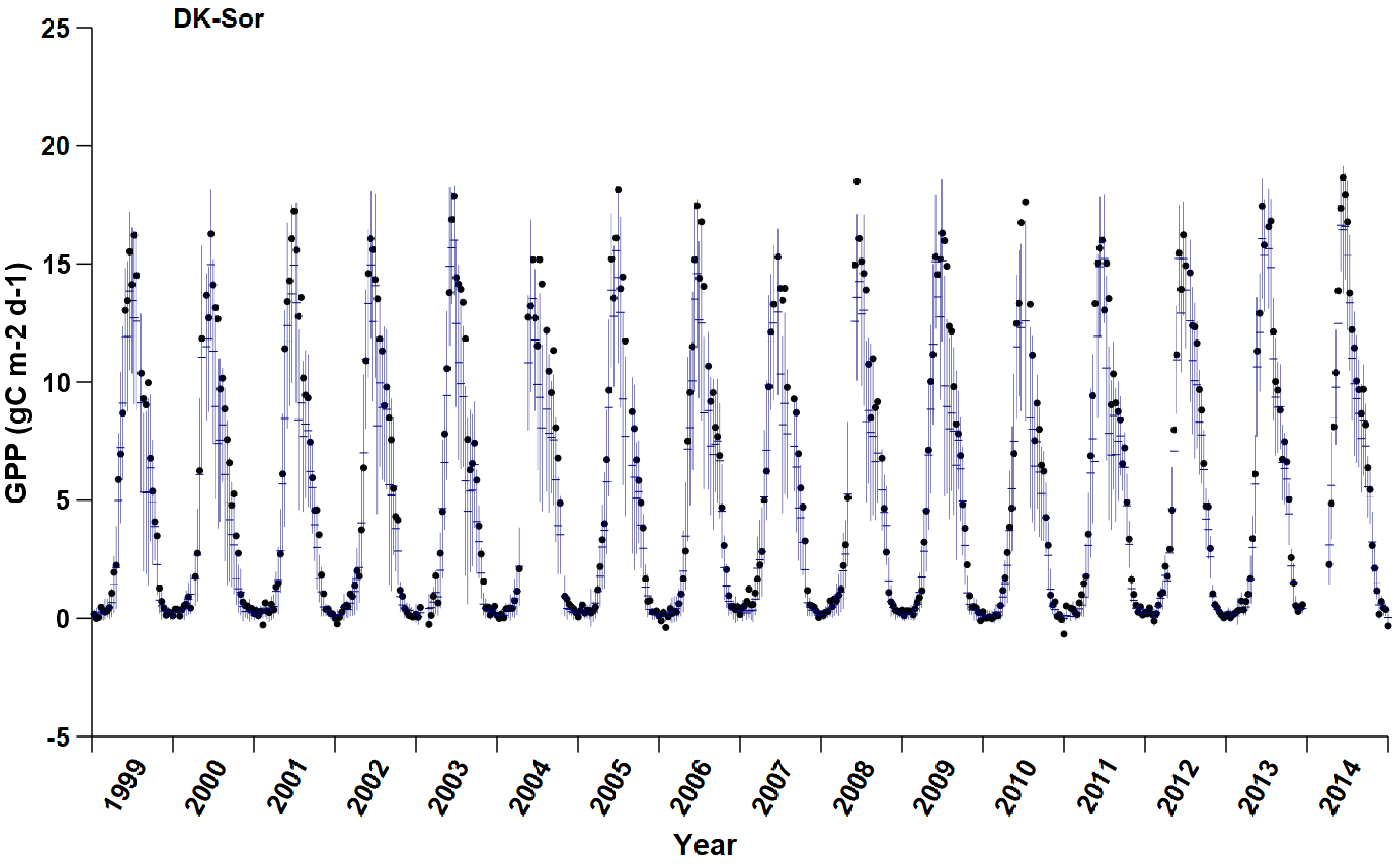
DK-Sor
GPP (gC m-2 d-1)
25
20
15
10
5
0
-5
1999
2000
2001
2002
2003
2004
2005
2006
2007
2008
2009
2010
2011
2012
2013
2014
Year

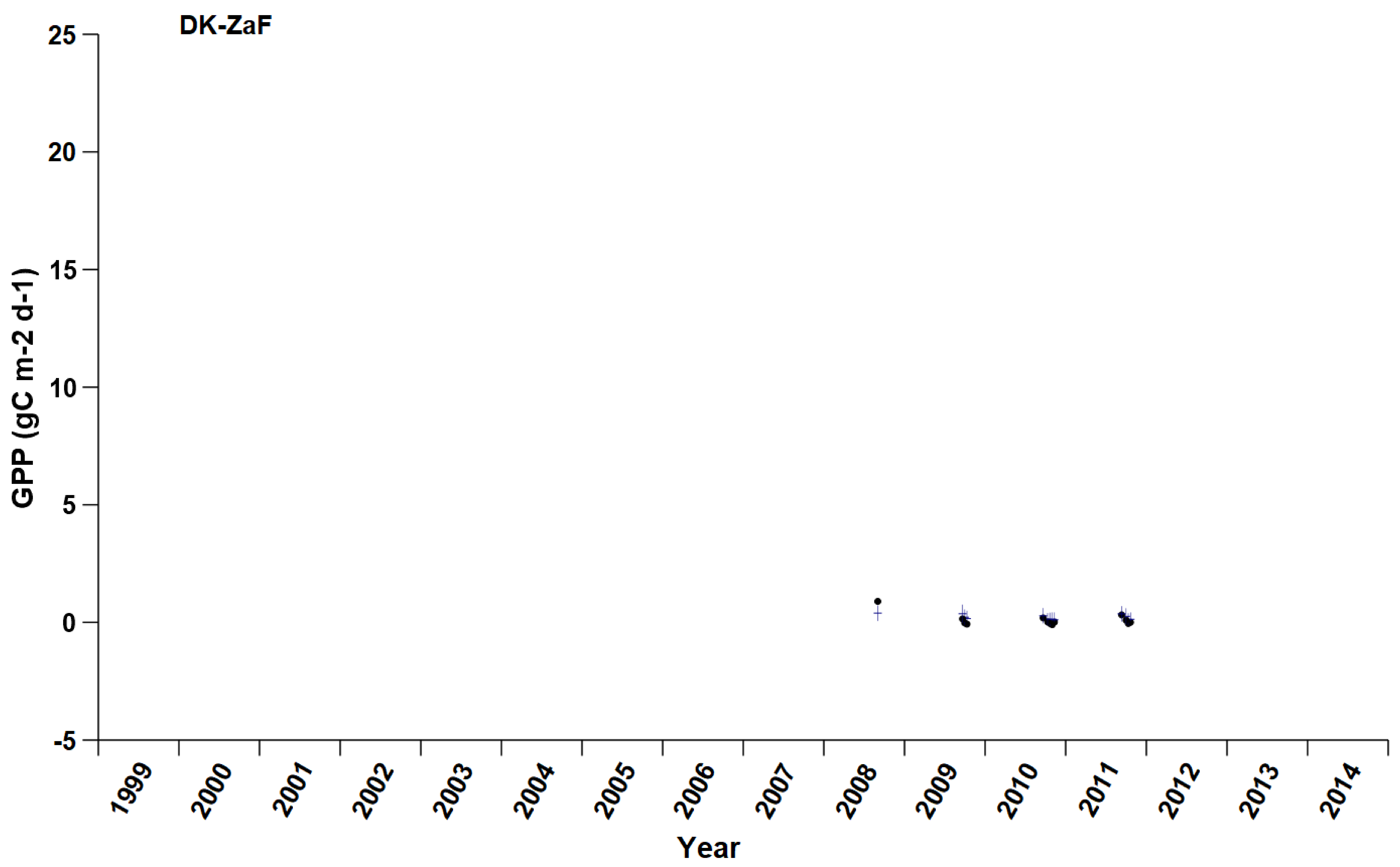

DK-ZaF
GPP (gC m-2 d-1)
25
20
15
10
5
0
-5
1999
2000
2001
2002
2003
2004
2005
2006
2007
2008
2009
2010
2011
2012
2013
2014
Year

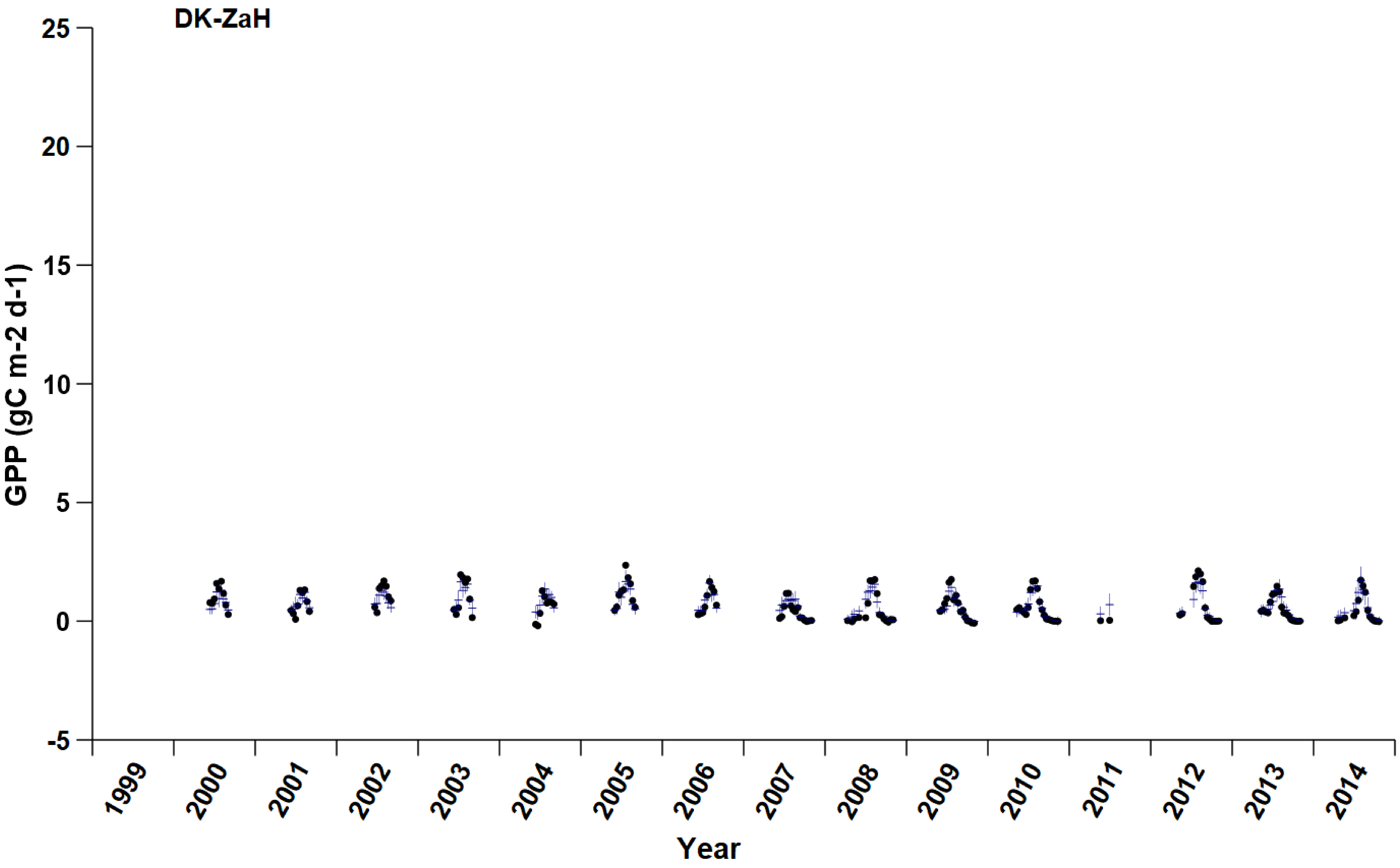
DK-ZaH
GPP (gC m-2 d-1)
25
20
15
10
5
0
-5
1999
2000
2001
2002
2003
2004
2005
2006
2007
2008
2009
2010
2011
2012
2013
2014
Year

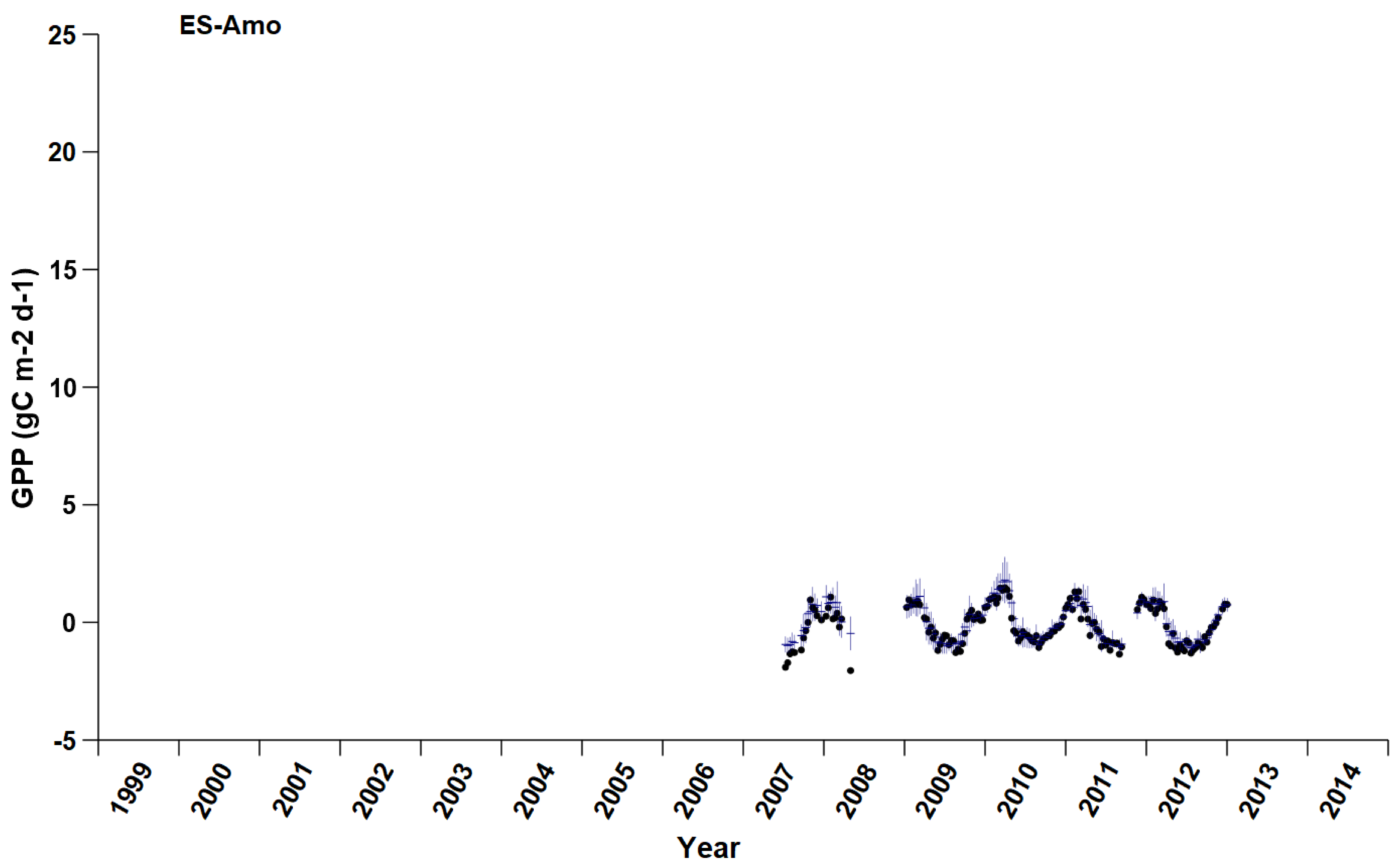
ES-Amo
GPP (gC m-2 d-1)
25
20
15
10
5
0
-5
1999
2000
2001
2002
2003
2004
2005
2006
2007
2008
2009
2010
2011
2012
2013
2014
Year

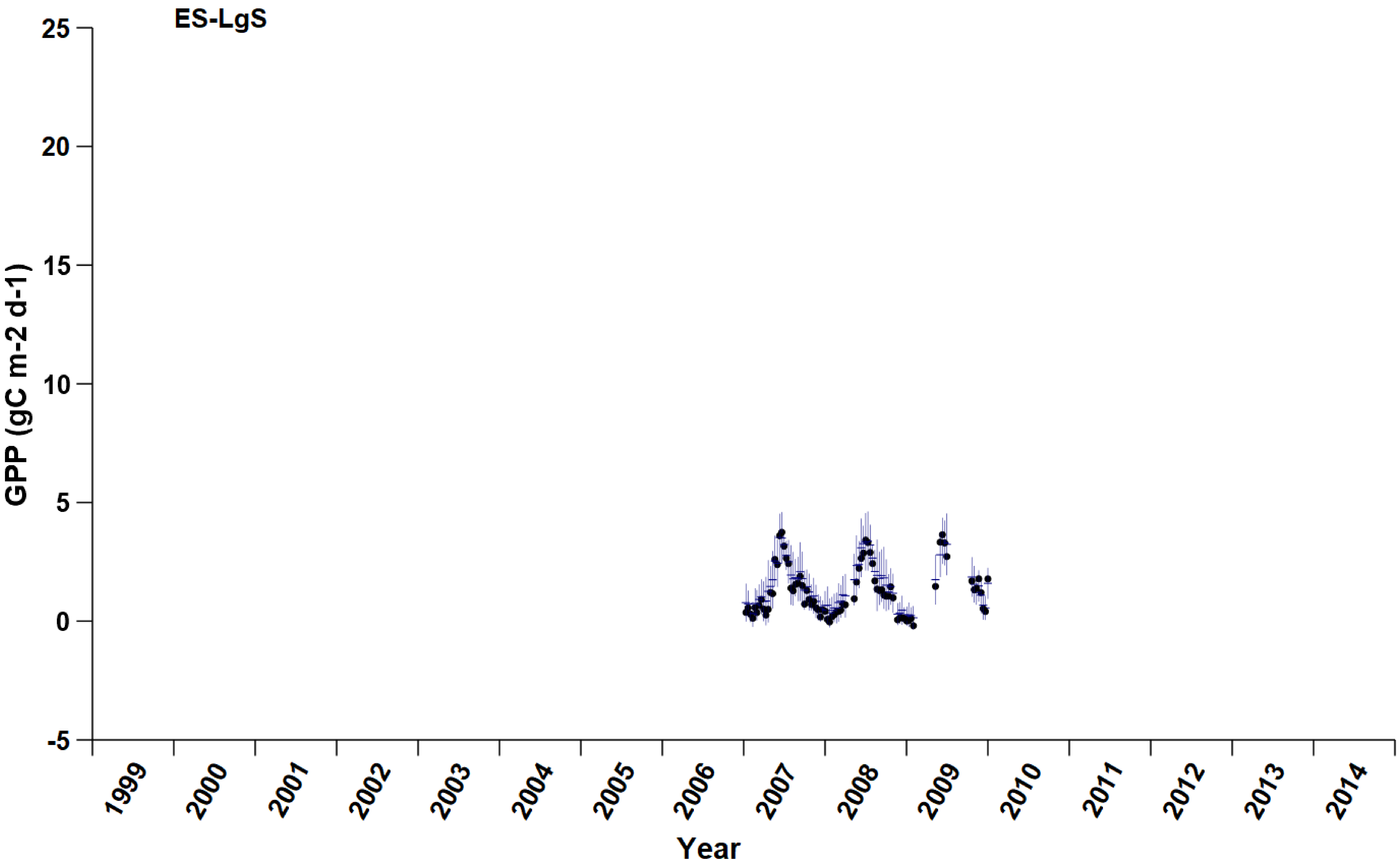

ES-LgS
GPP (gC m-2 d-1)
25
20
15
10
5
0
-5
1999
2000
2001
2002
2003
2004
2005
2006
2007
2008
2009
2010
2011
2012
2013
2014
Year

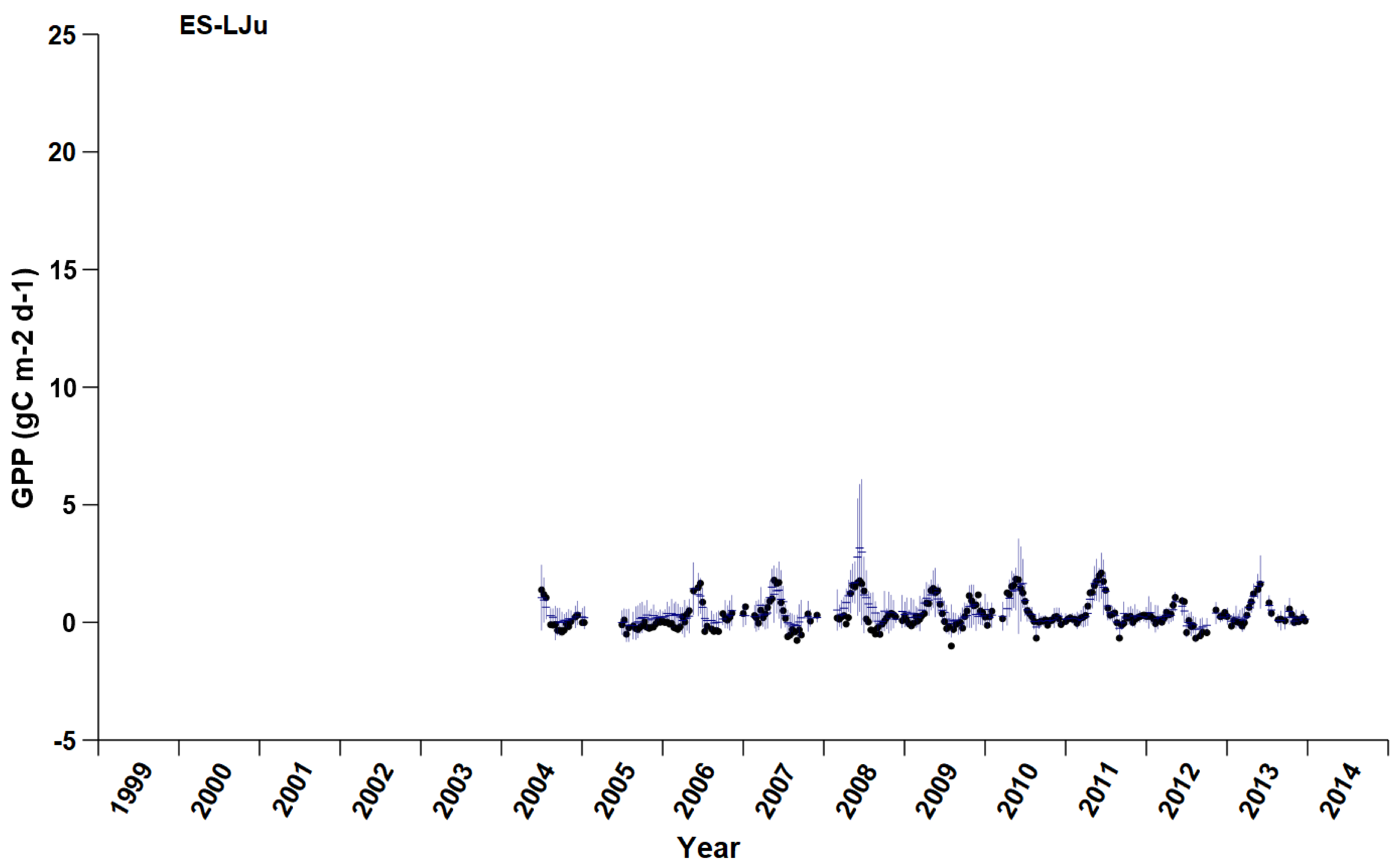

ES-LJu
25
20
15
10
5
0
-5
GPP (gC m-2 d-1)
1999
2000
2001
2002
2003
2004
2005
2006
2007
2008
2009
2010
2011
2012
2013
2014
Year

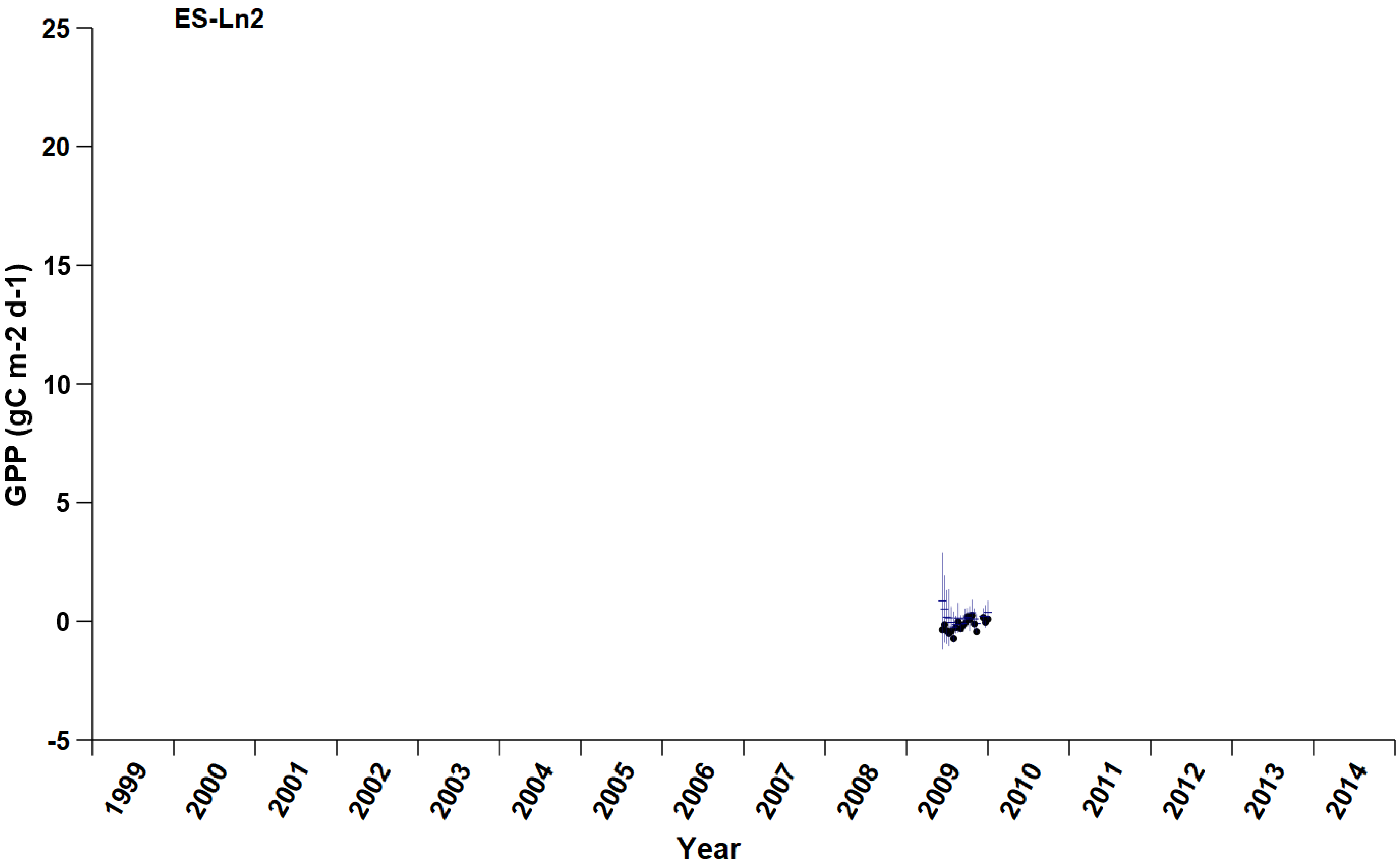

ES-Ln2
GPP (gC m-2 d-1)
25
20
15
10
5
0
-5
1999
2000
2001
2002
2003
2004
2005
2006
2007
2008
2009
2010
2011
2012
2013
2014
Year

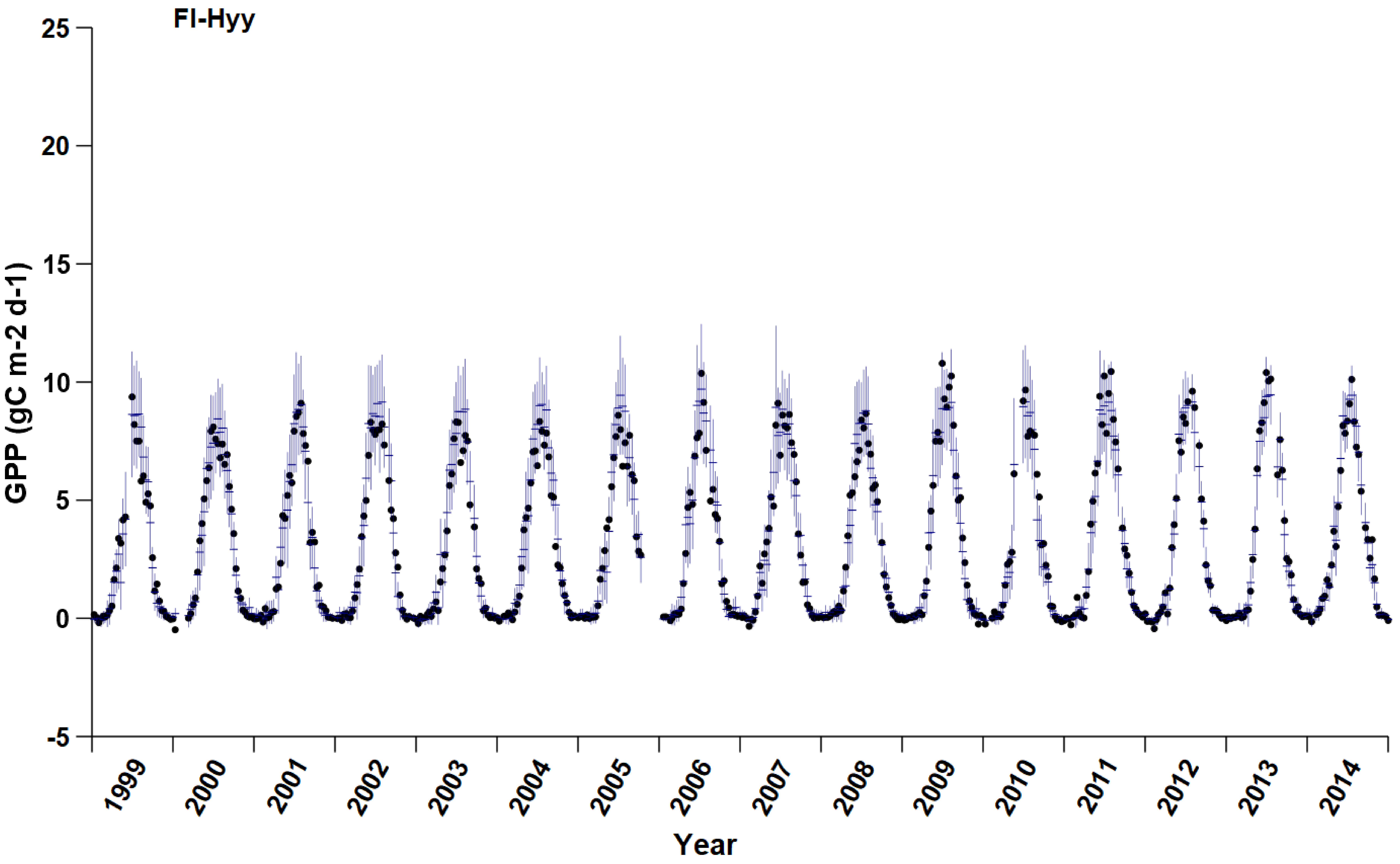

FI-Hyy
GPP (gC m-2 d-1)
25
20
15
10
5
0
-5
1999
2000
2001
2002
2003
2004
2005
2006
2007
2008
2009
2010
2011
2012
2013
2014
Year

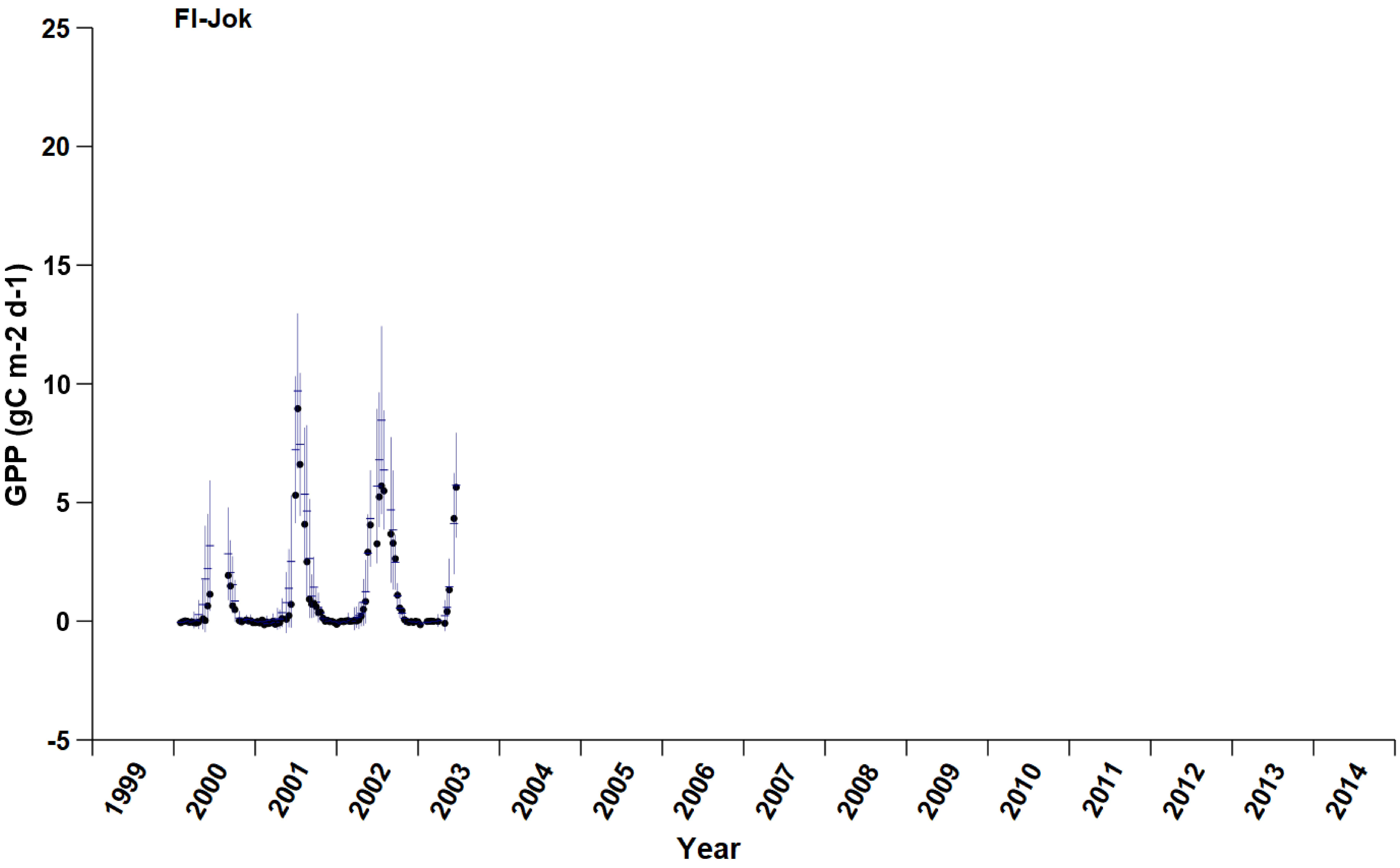

FI-Jok
GPP (gC m-2 d-1)
25
20
15
10
5
0
-5
1999
2000
2001
2002
2003
2004
2005
2006
2007
2008
2009
2010
2011
2012
2013
2014
Year

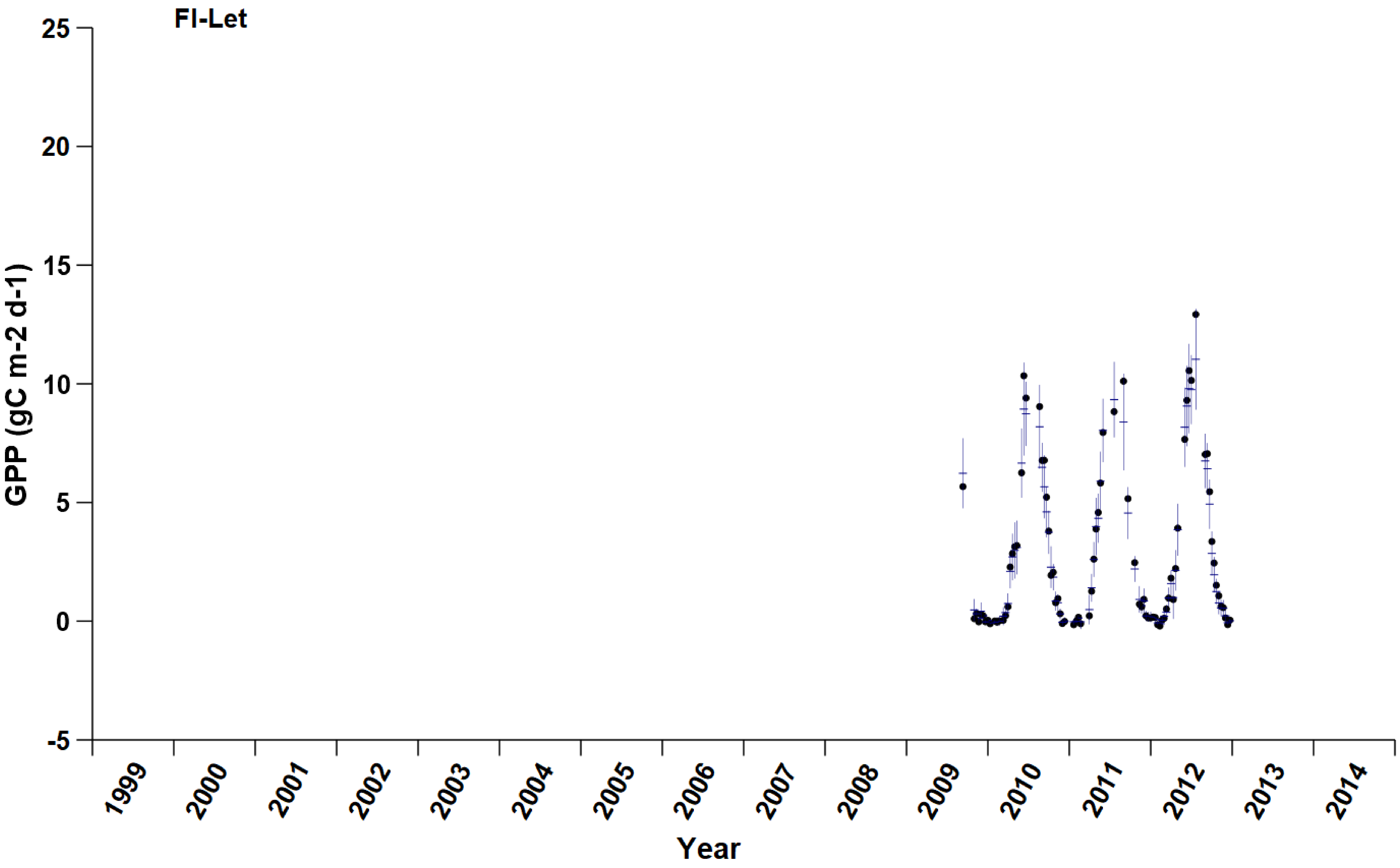
FI-Let
GPP (gC m-2 d-1)
25
20
15
10
5
0
-5
1999
2000
2001
2002
2003
2004
2005
2006
2007
2008
2009
2010
2011
2012
2013
2014
Year

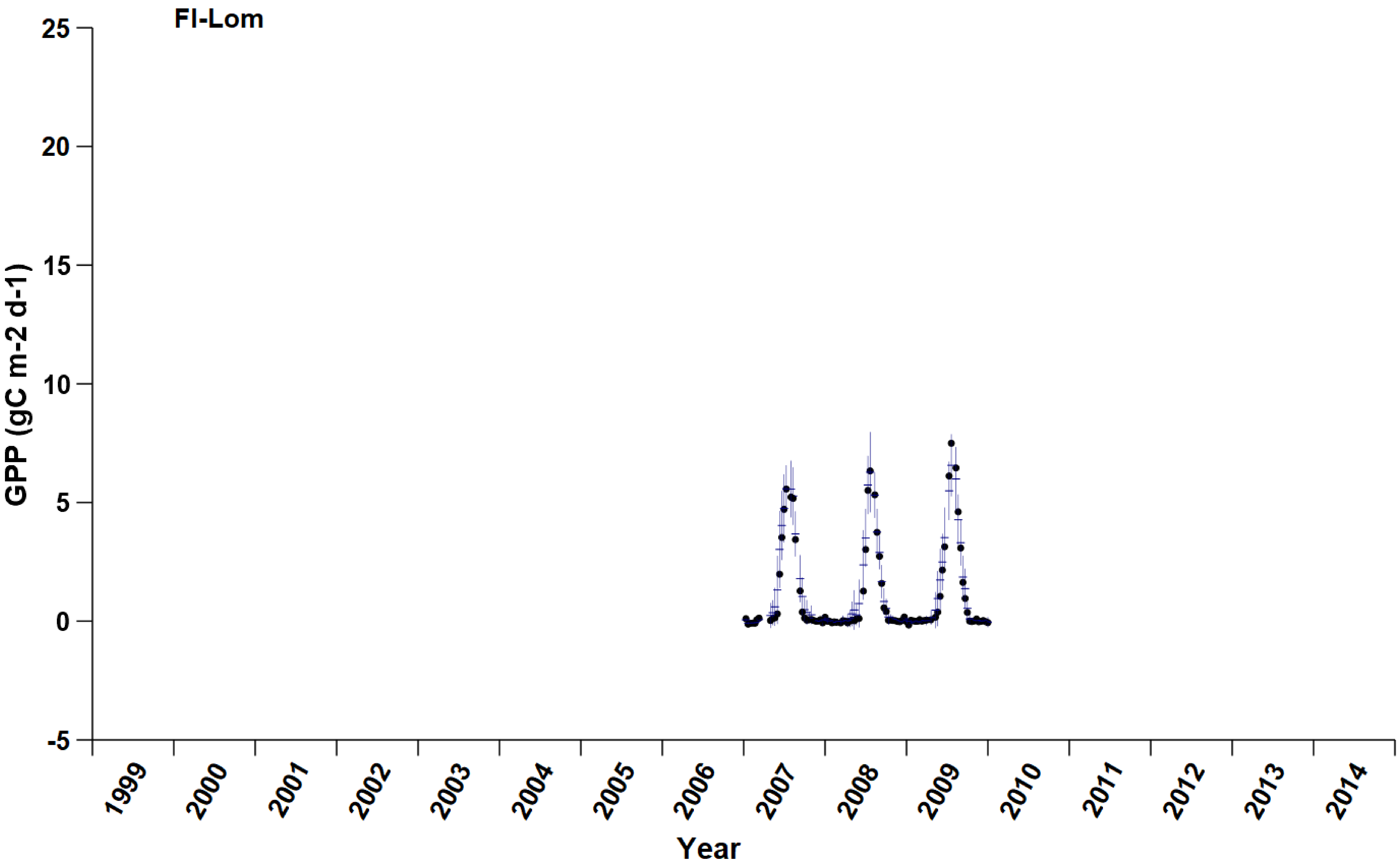
FI-Lom
GPP (gC m-2 d-1)
25
20
15
10
5
0
-5
1999
2000
2001
2002
2003
2004
2005
2006
2007
2008
2009
2010
2011
2012
2013
2014
Year

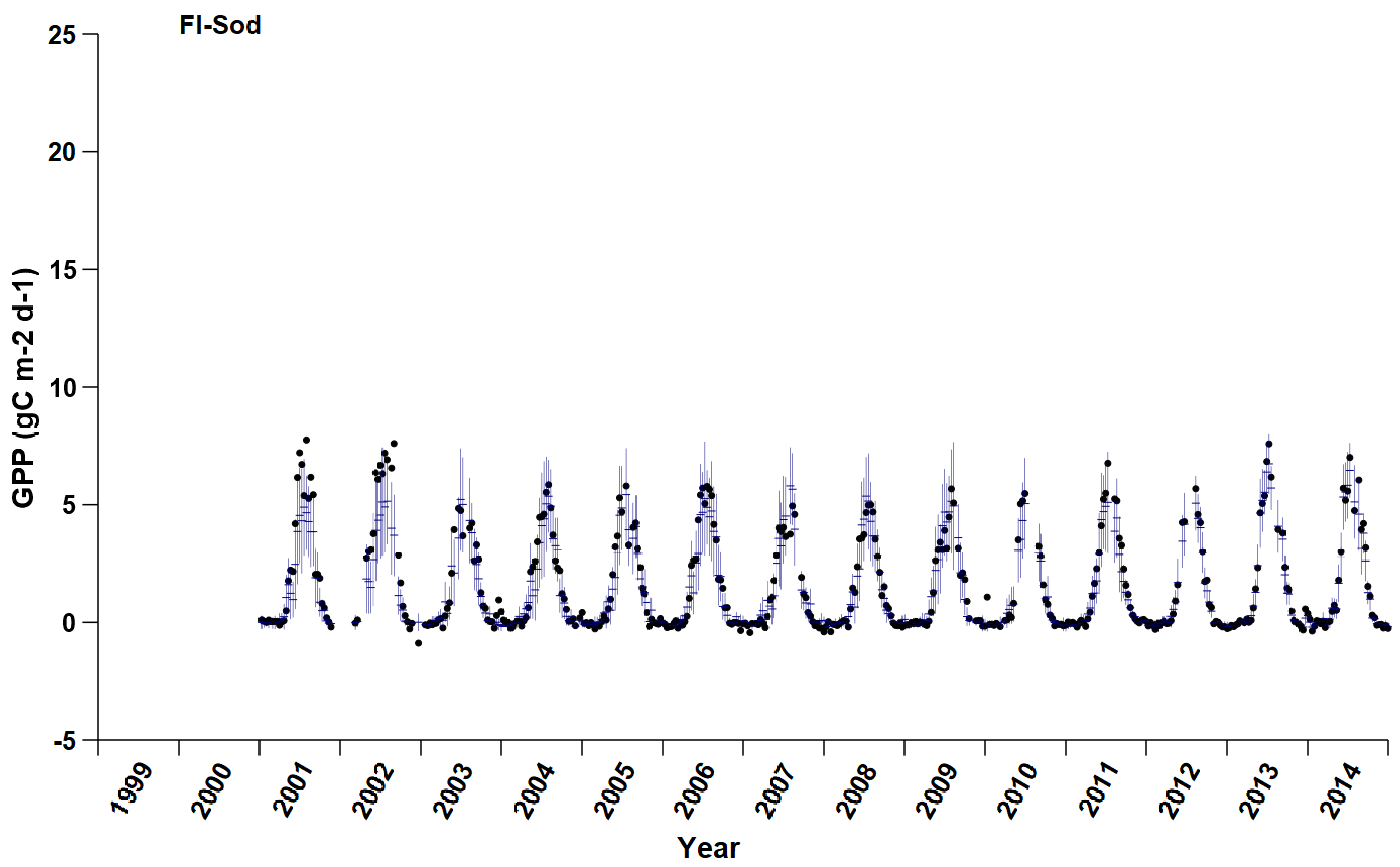
FI-Sod
GPP (gC m-2 d-1)
25
20
15
10
5
0
-5
1999
2000
2001
2002
2003
2004
2005
2006
2007
2008
2009
2010
2011
2012
2013
2014
Year

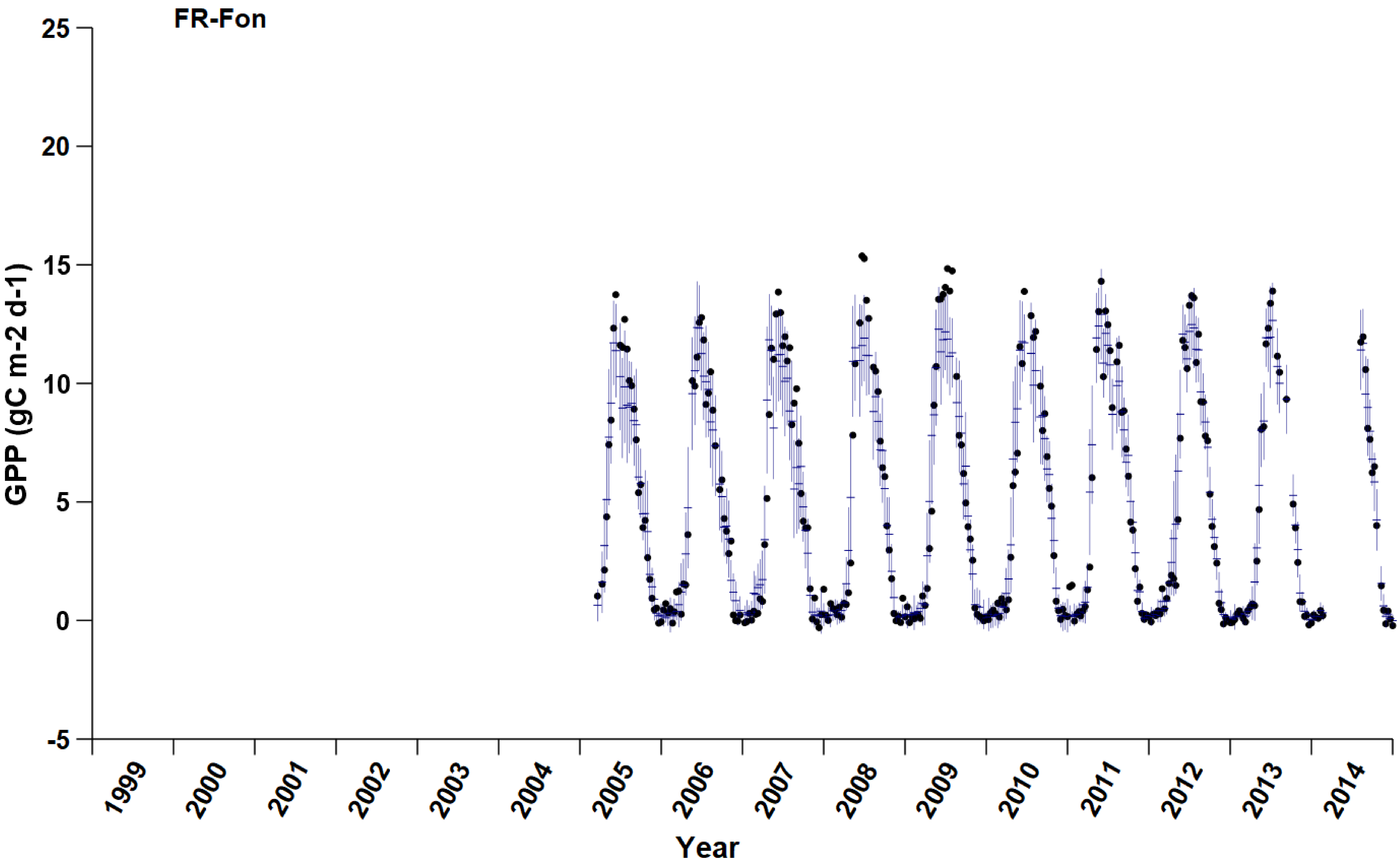
FR-Fon
GPP (gC m-2 d-1)
25
20
15
10
5
0
-5
1999
2000
2001
2002
2003
2004
2005
2006
2007
2008
2009
2010
2011
2012
2013
2014
Year

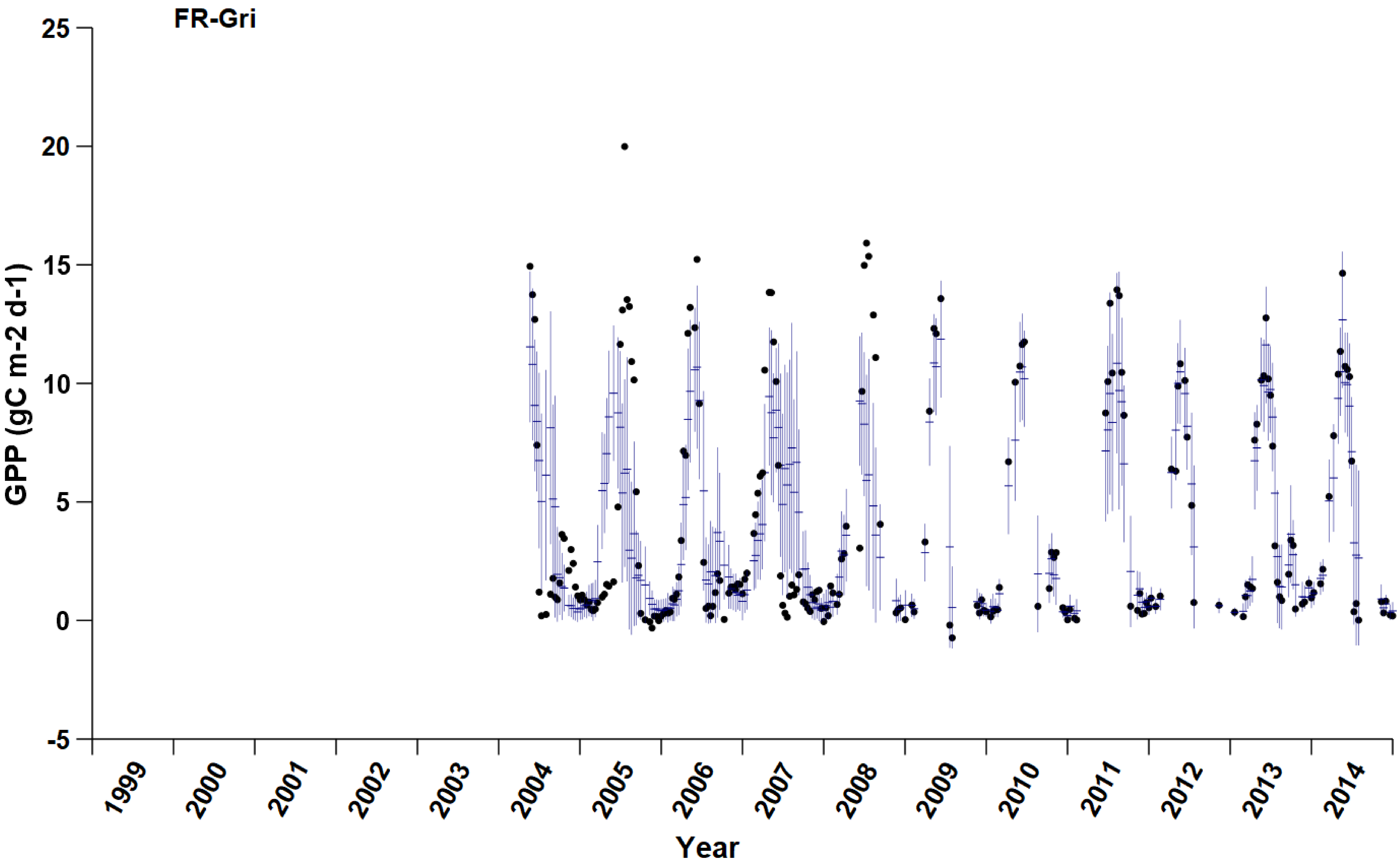

FR-Gri
GPP (gC m-2 d-1)
25
20
15
10
5
0
-5
1999
2000
2001
2002
2003
2004
2005
2006
2007
2008
2009
2010
2011
2012
2013
2014
Year

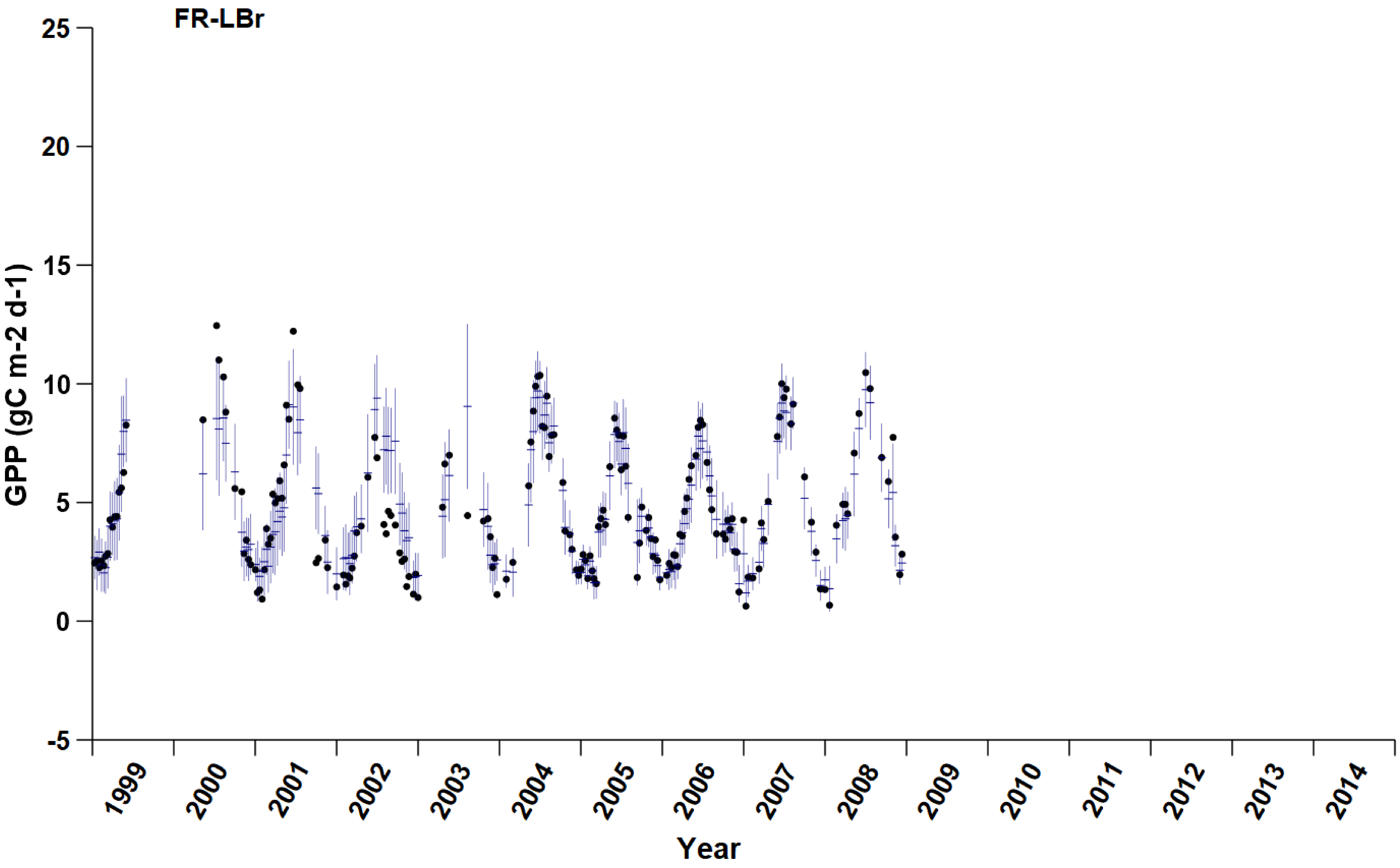
FR-LBr
GPP (gC m-2 d-1)
25
20
15
10
5
0
-5
1999
2000
2001
2002
2003
2004
2005
2006
2007
2008
2009
2010
2011
2012
2013
2014
Year

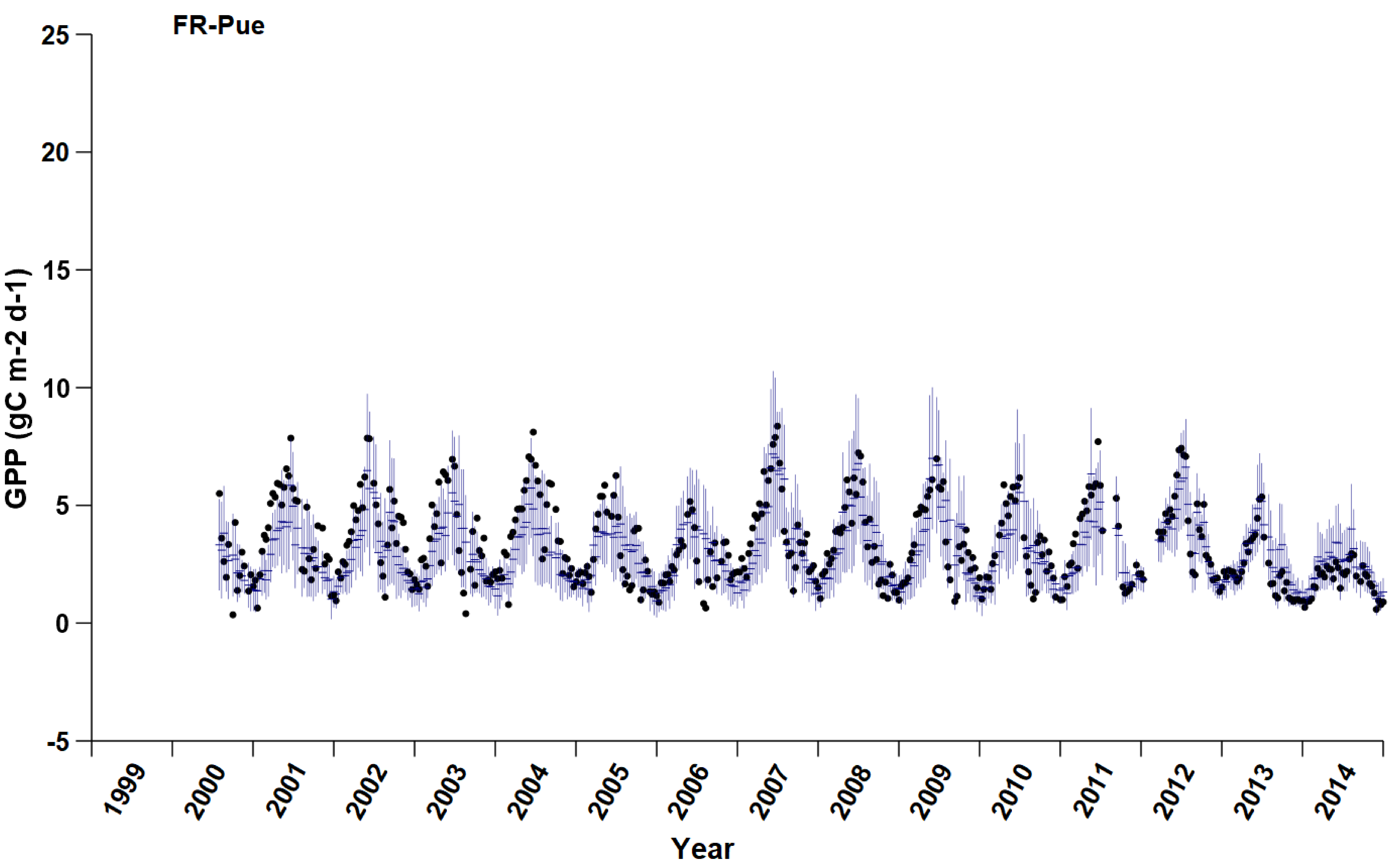
FR-Pue
GPP (gC m-2 d-1)
25
20
15
10
5
0
-5
1999
2000
2001
2002
2003
2004
2005
2006
2007
2008
2009
2010
2011
2012
2013
2014
Year

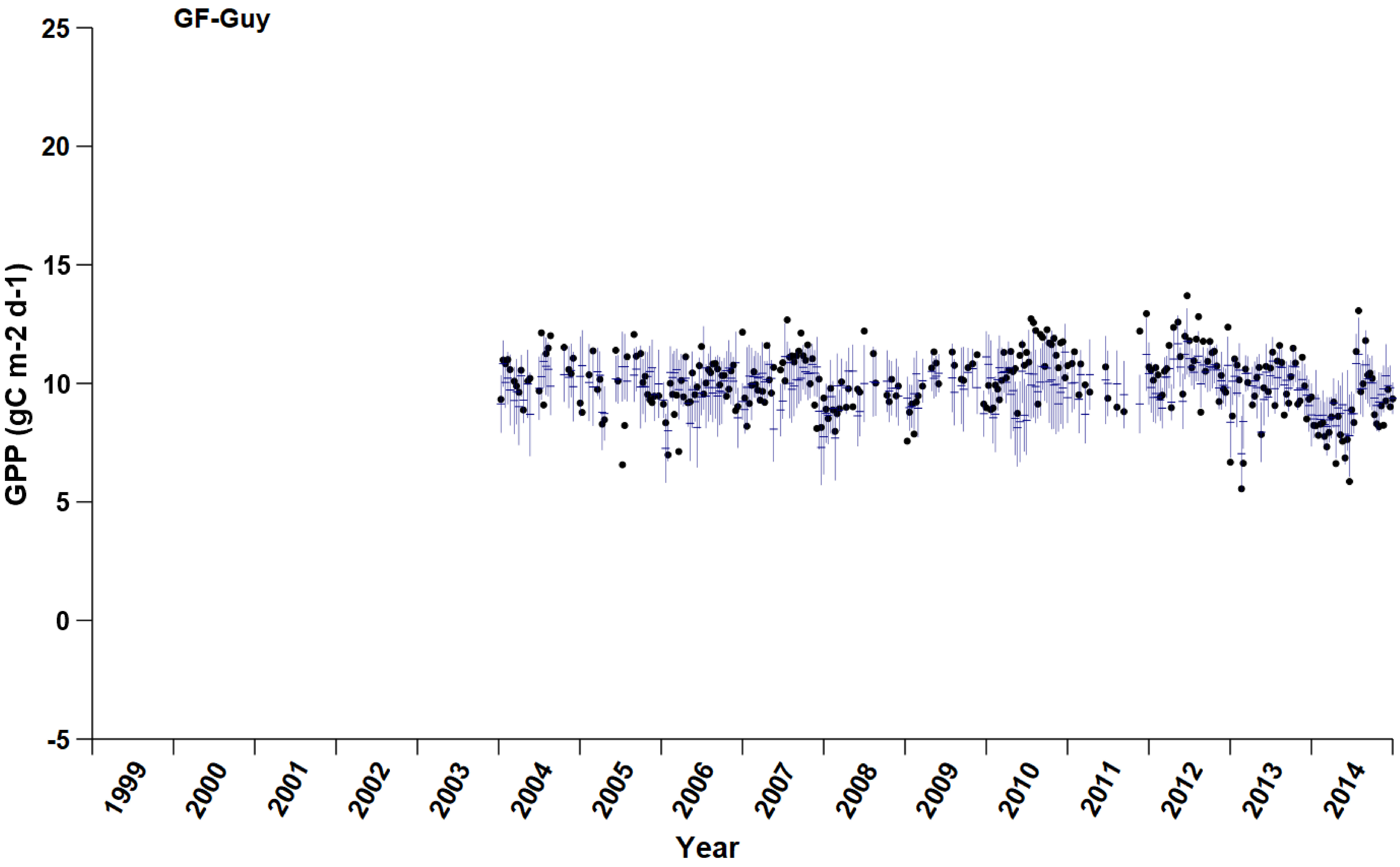
GF-Guy
GPP (gC m-2 d-1)
25
20
15
10
5
0
-5
1999
2000
2001
2002
2003
2004
2005
2006
2007
2008
2009
2010
2011
2012
2013
2014
Year

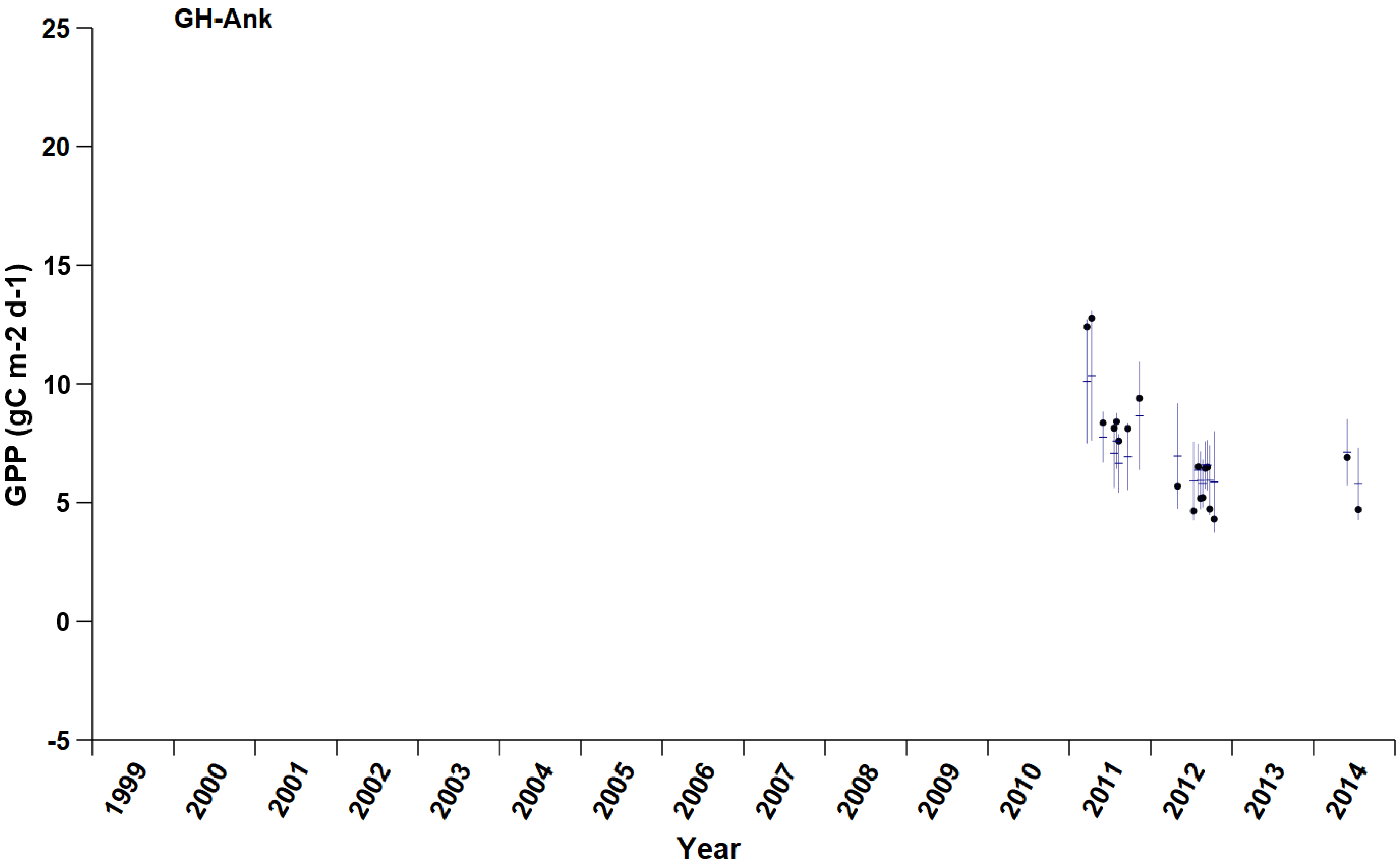
GH-Ank
GPP (gC m-2 d-1)
25
20
15
10
5
0
-5
1999
2000
2001
2002
2003
2004
2005
2006
2007
2008
2009
2010
2011
2012
2013
2014
Year

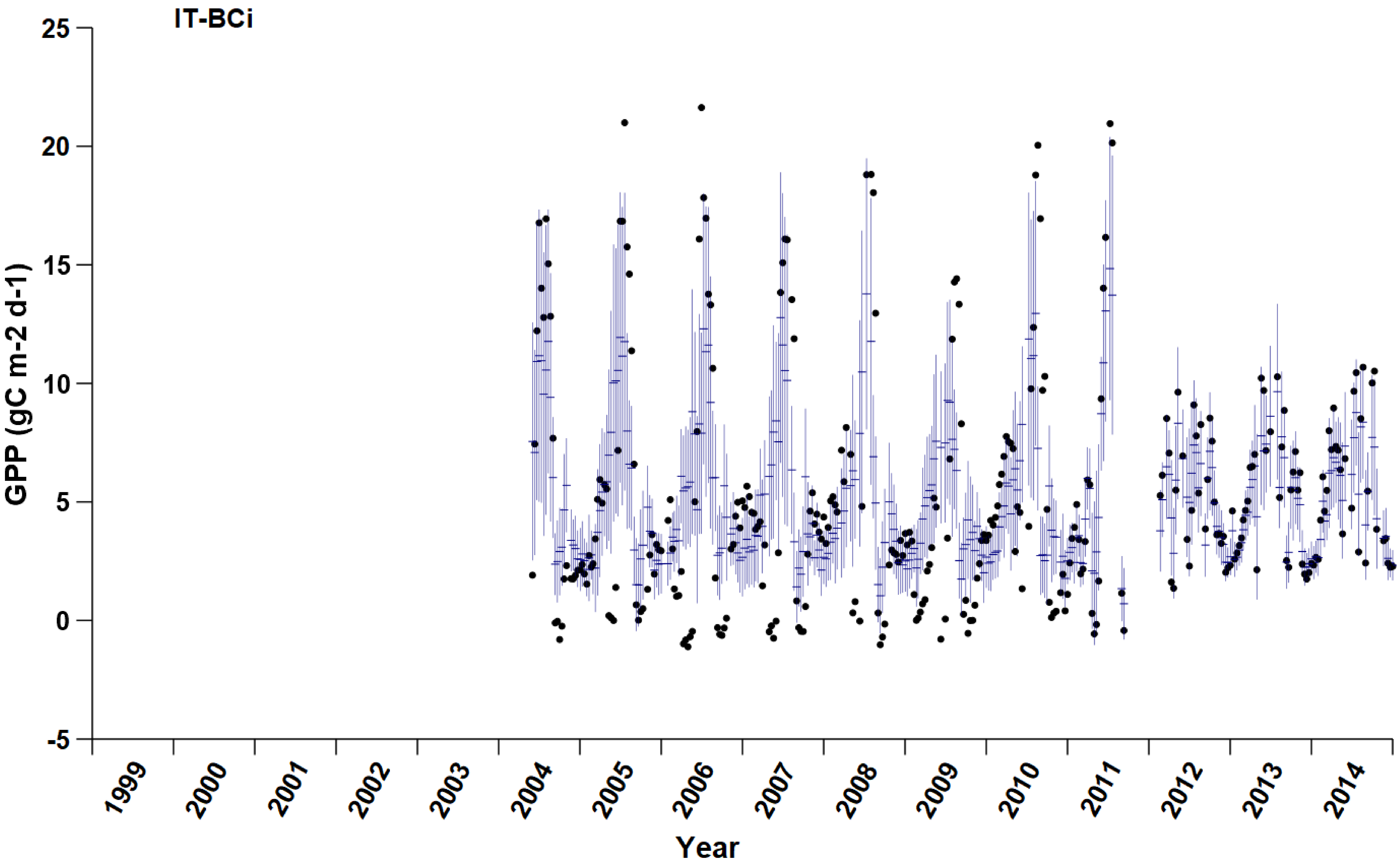

IT-BCi
GPP (gC m-2 d-1)
25
20
15
10
5
0
-5
1999
2000
2001
2002
2003
2004
2005
2006
2007
2008
2009
2010
2011
2012
2013
2014
Year

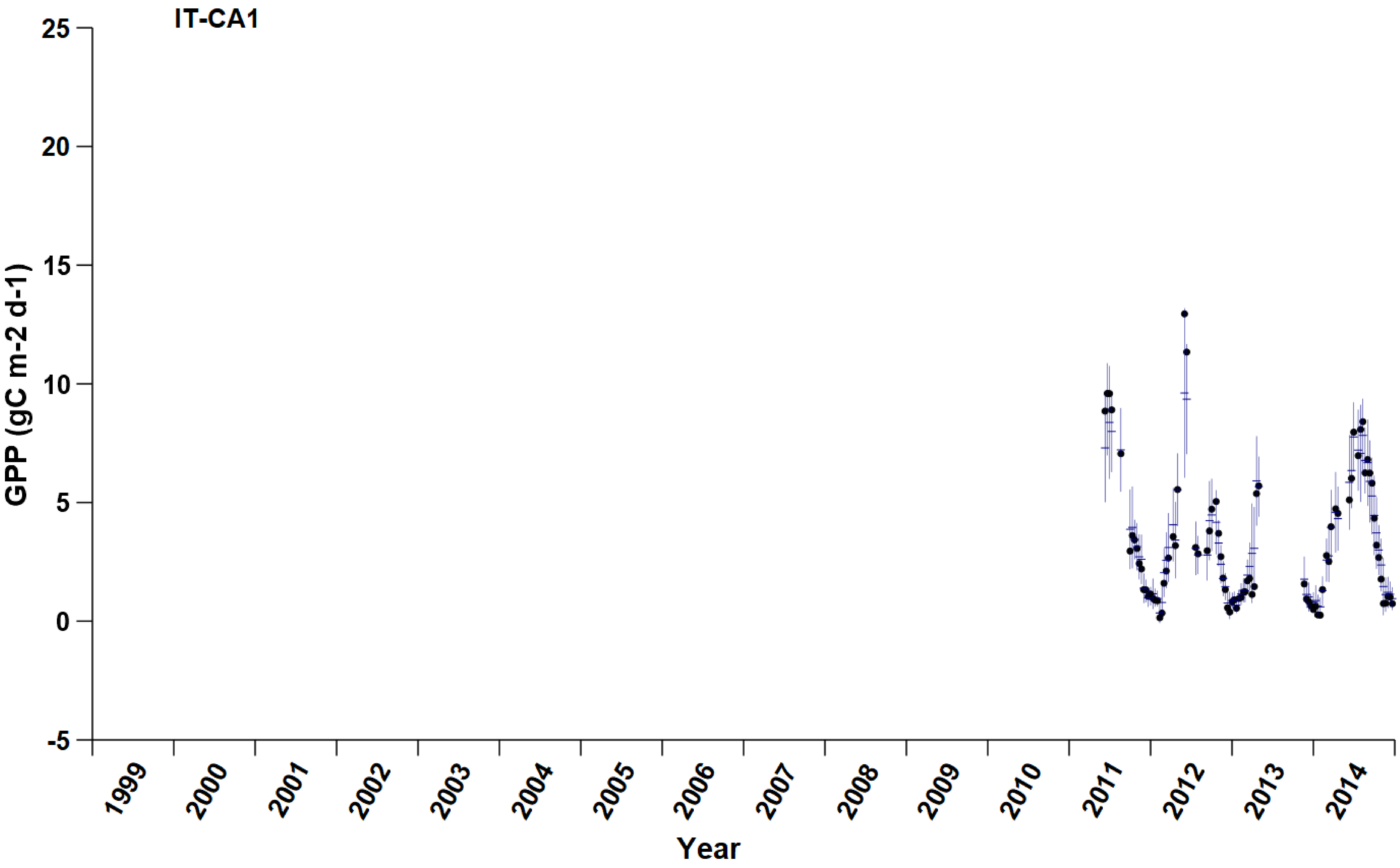
IT-CA1
GPP (gC m-2 d-1)
25
20
15
10
5
0
-5
1999
2000
2001
2002
2003
2004
2005
2006
2007
2008
2009
2010
2011
2012
2013
2014
Year

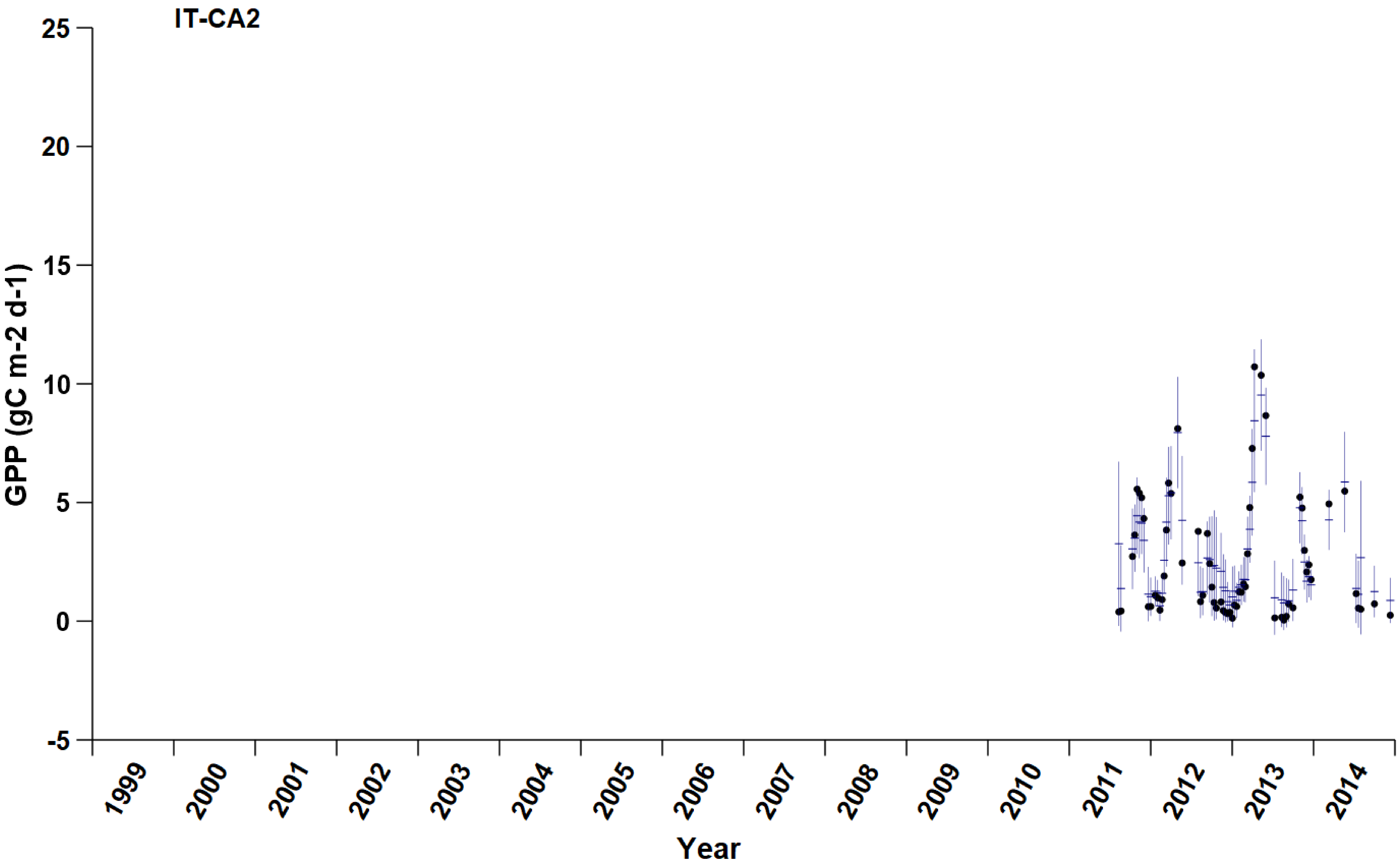

IT-CA2
GPP (gC m-2 d-1)
25
20
15
10
5
0
-5
1999
2000
2001
2002
2003
2004
2005
2006
2007
2008
2009
2010
2011
2012
2013
2014
Year

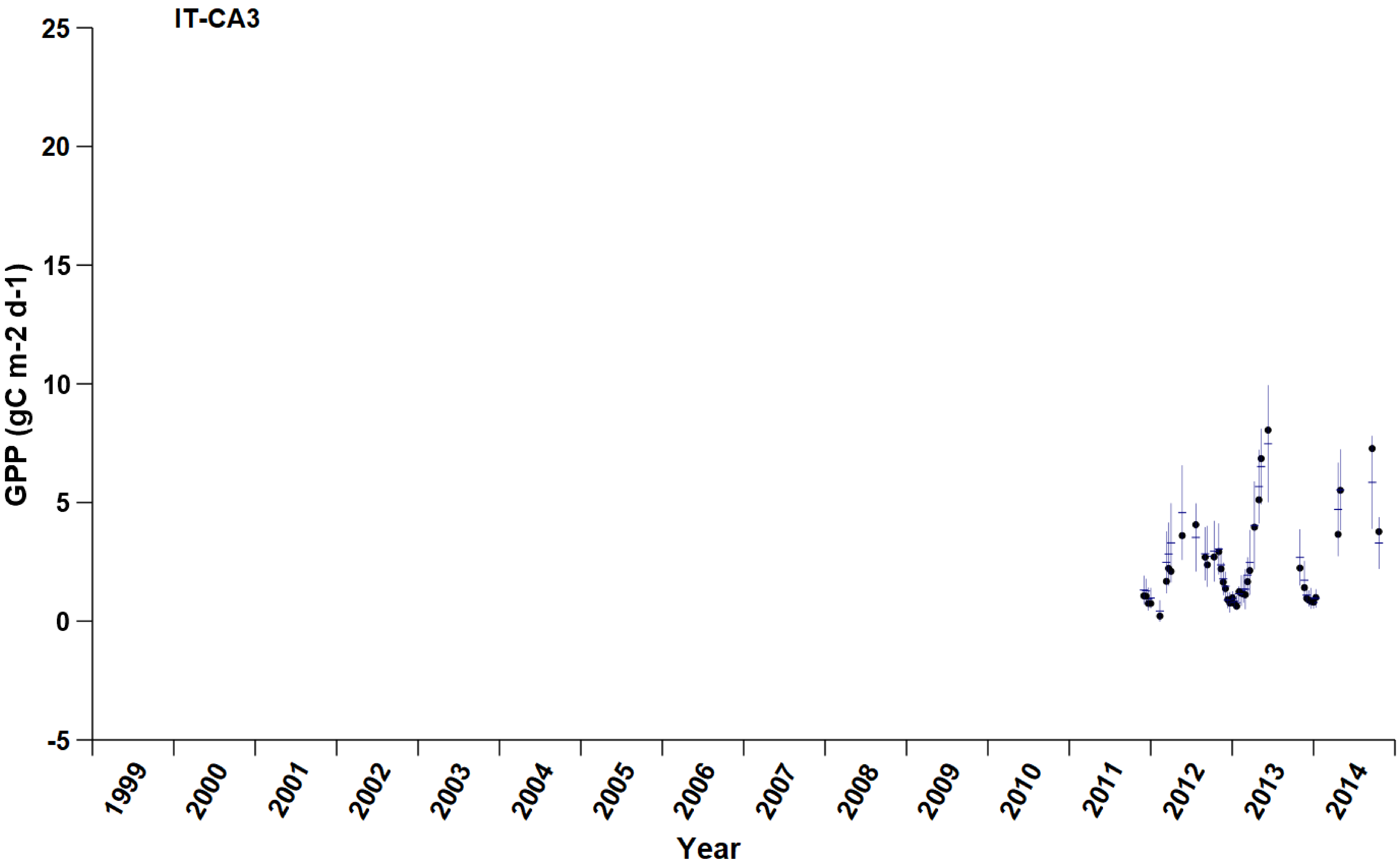
IT-CA3
GPP (gC m-2 d-1)
25
20
15
10
5
0
-5
1999
2000
2001
2002
2003
2004
2005
2006
2007
2008
2009
2010
2011
2012
2013
2014
Year

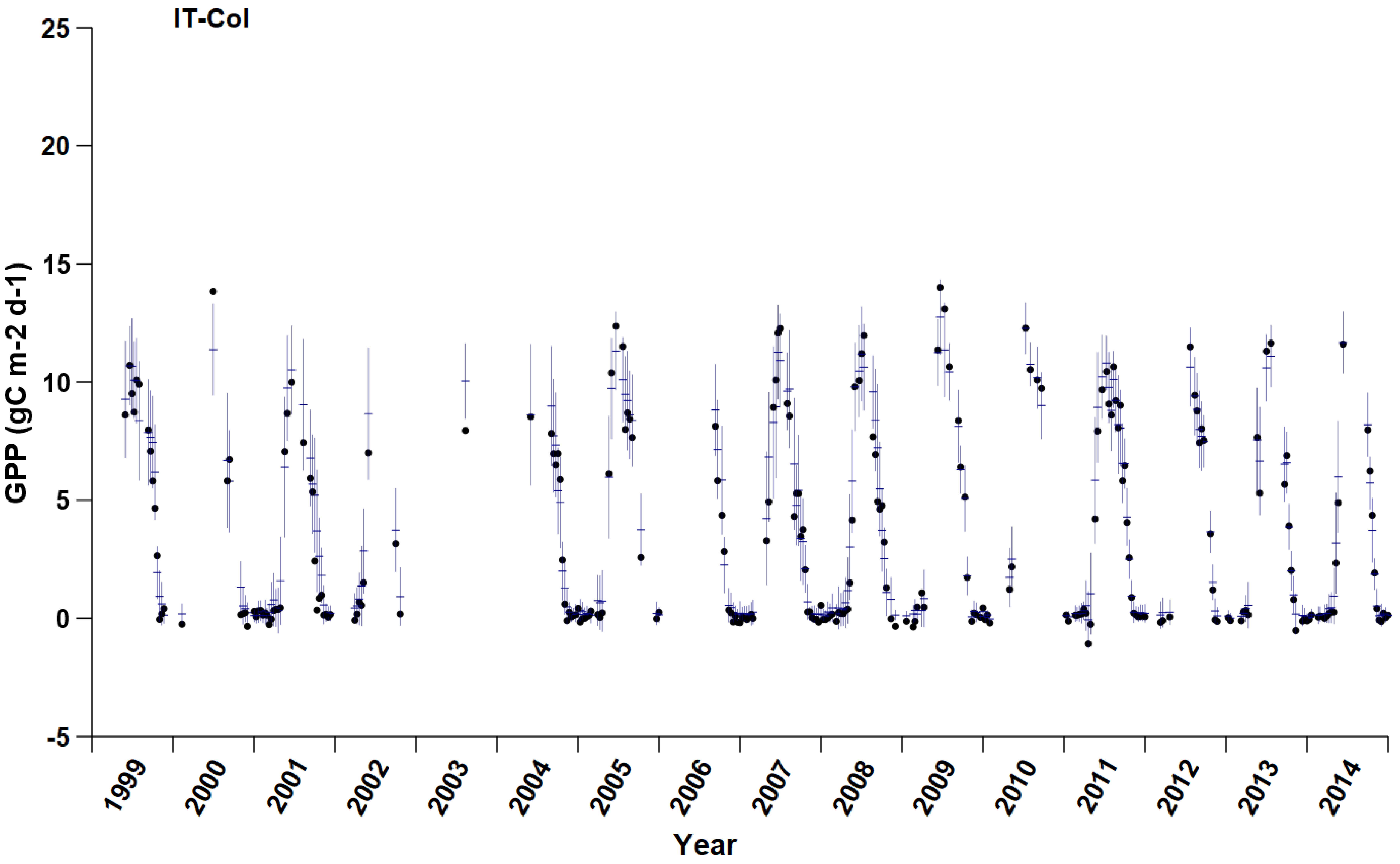
IT-Col
GPP (gC m-2 d-1)
25
20
15
10
5
0
-5
1999
2000
2001
2002
2003
2004
2005
2006
2007
2008
2009
2010
2011
2012
2013
2014
Year

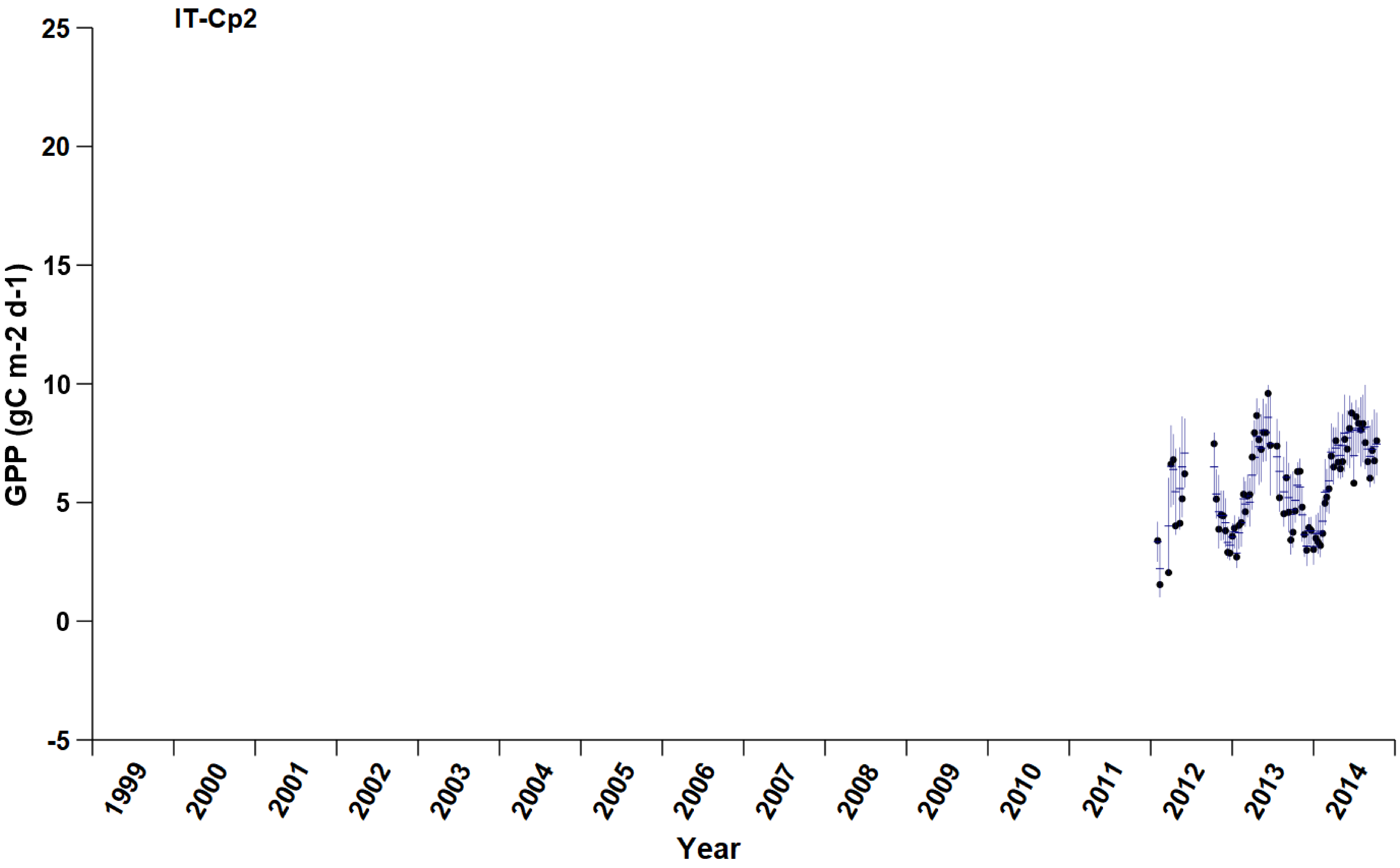

IT-Cp2
GPP (gC m-2 d-1)
25
20
15
10
5
0
-5
1999
2000
2001
2002
2003
2004
2005
2006
2007
2008
2009
2010
2011
2012
2013
2014
Year

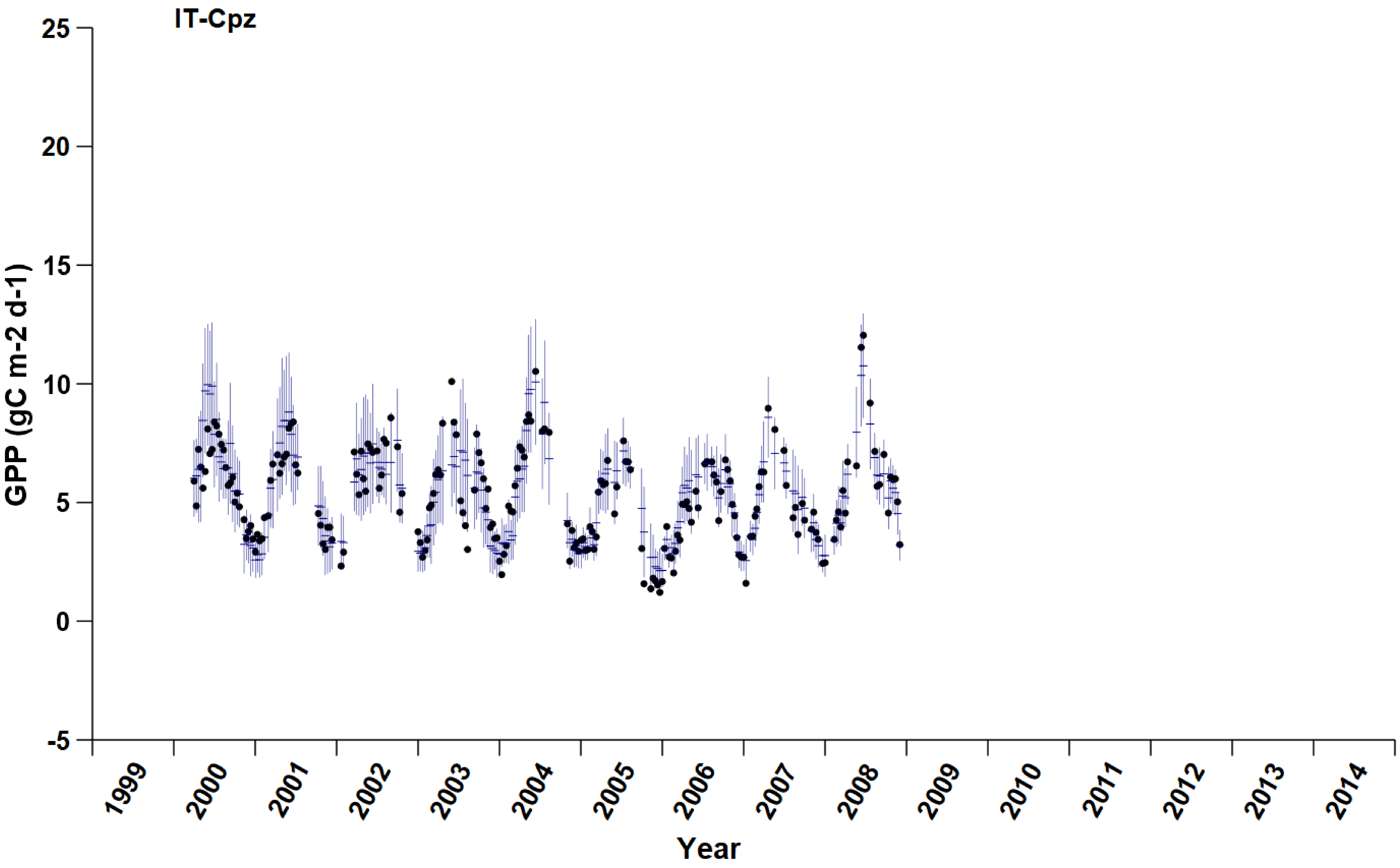
IT-Cpz
GPP (gC m-2 d-1)
25
20
15
10
5
0
-5
1999
2000
2001
2002
2003
2004
2005
2006
2007
2008
2009
2010
2011
2012
2013
2014
Year

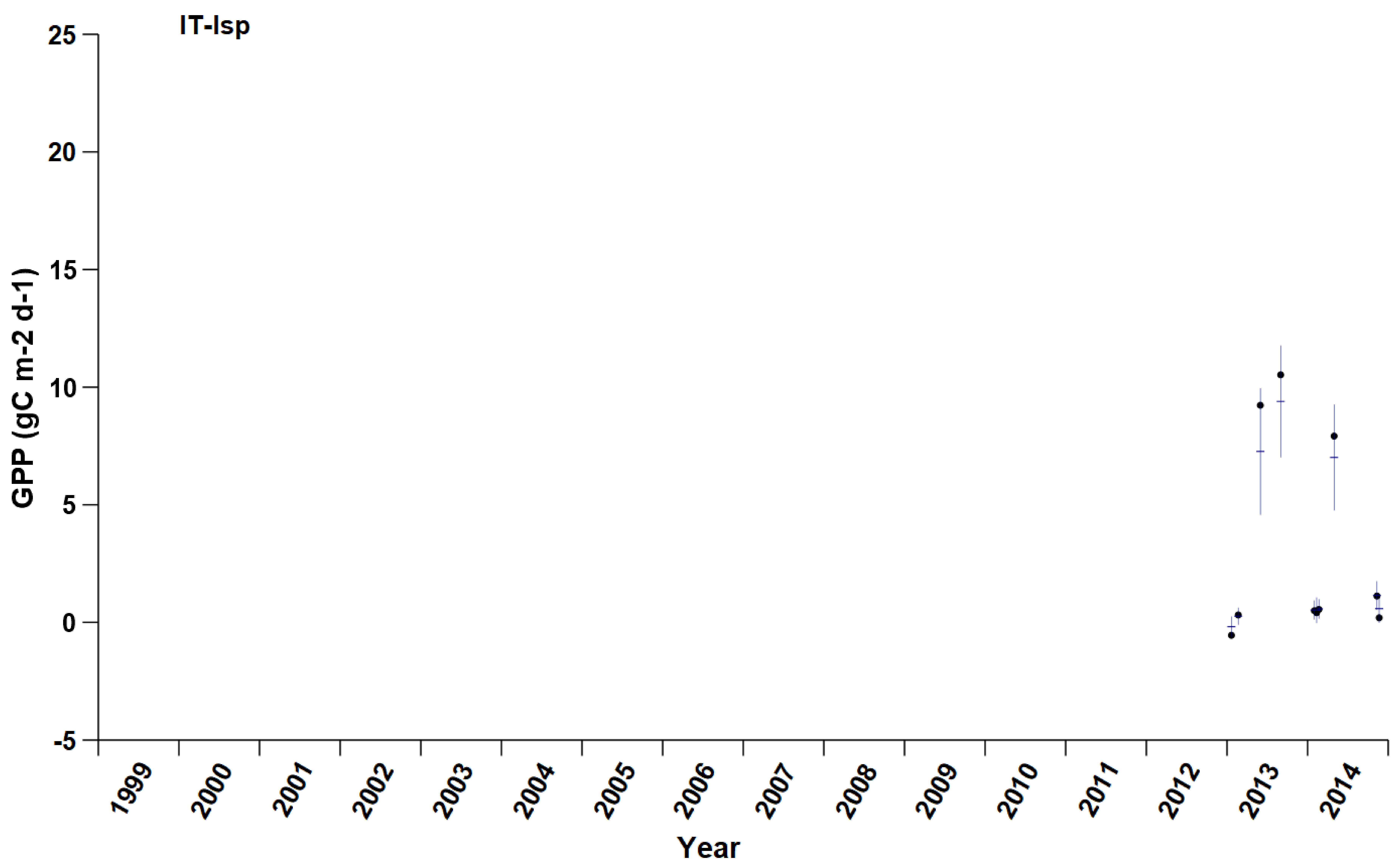
IT-Isp
GPP (gC m-2 d-1)
25
20
15
10
5
0
-5
1999
2000
2001
2002
2003
2004
2005
2006
2007
2008
2009
2010
2011
2012
2013
2014
Year

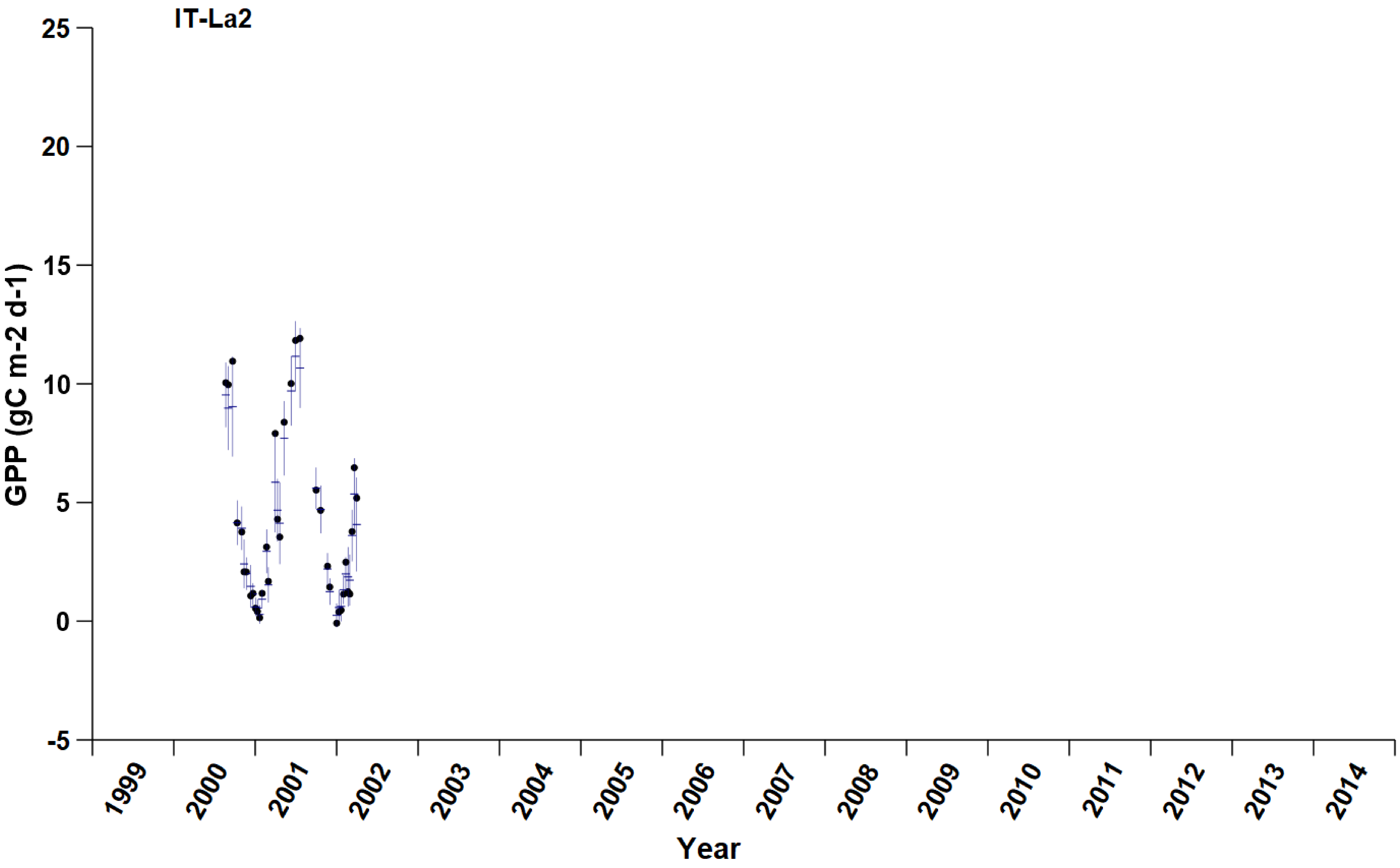
IT-La2
GPP (gC m-2 d-1)
25
20
15
10
5
0
-5
1999
2000
2001
2002
2003
2004
2005
2006
2007
2008
2009
2010
2011
2012
2013
2014
Year

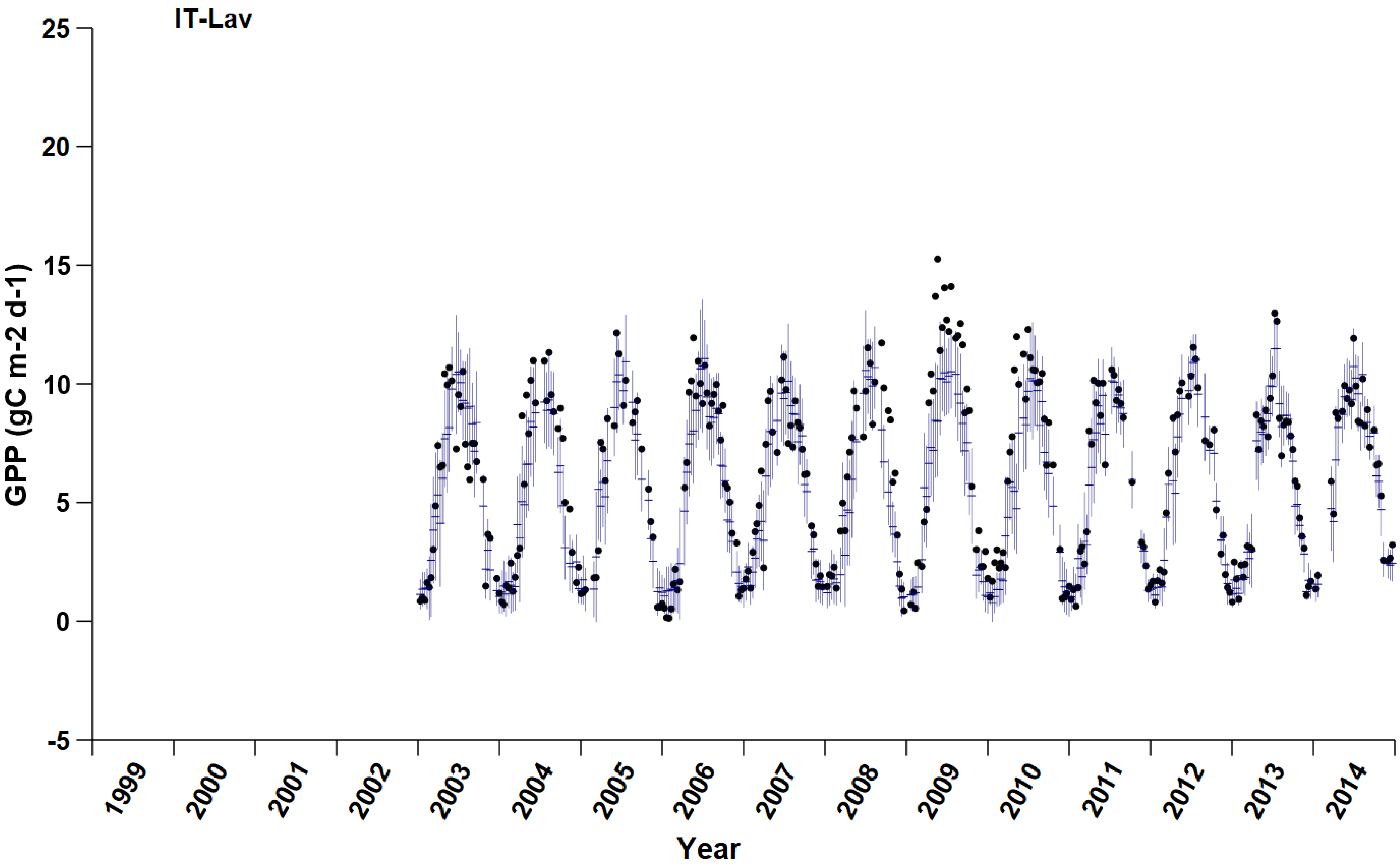
IT-Lav
GPP (gC m-2 d-1)
25
20
15
10
5
0
-5
1999
2000
2001
2002
2003
2004
2005
2006
2007
2008
2009
2010
2011
2012
2013
2014
Year

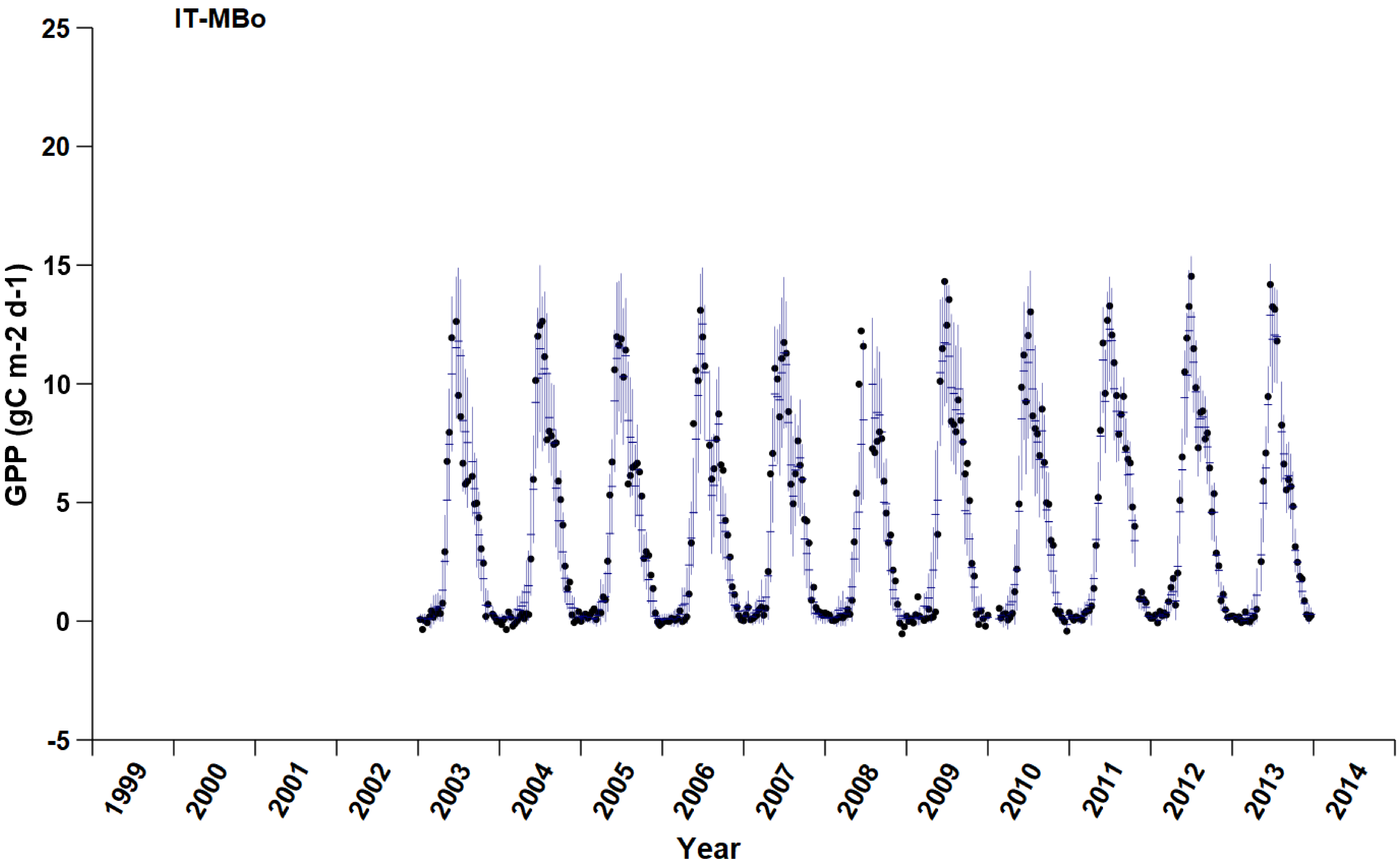
IT-MBo
GPP (gC m-2 d-1)
25
20
15
10
5
0
-5
1999
2000
2001
2002
2003
2004
2005
2006
2007
2008
2009
2010
2011
2012
2013
2014
Year

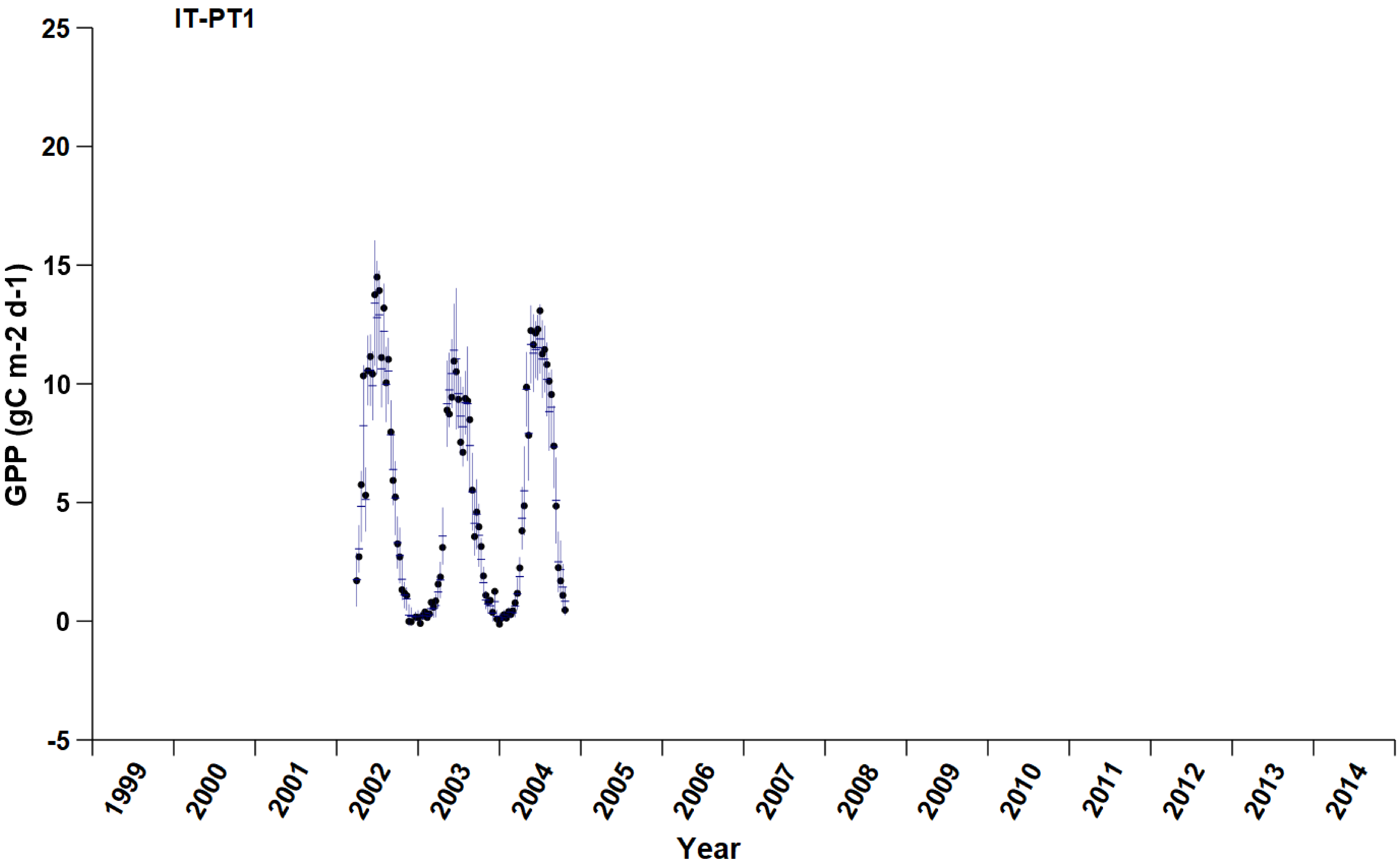

IT-PT1
GPP (gC m-2 d-1)
25
20
15
10
5
0
-5
1999
2000
2001
2002
2003
2004
2005
2006
2007
2008
2009
2010
2011
2012
2013
2014
Year

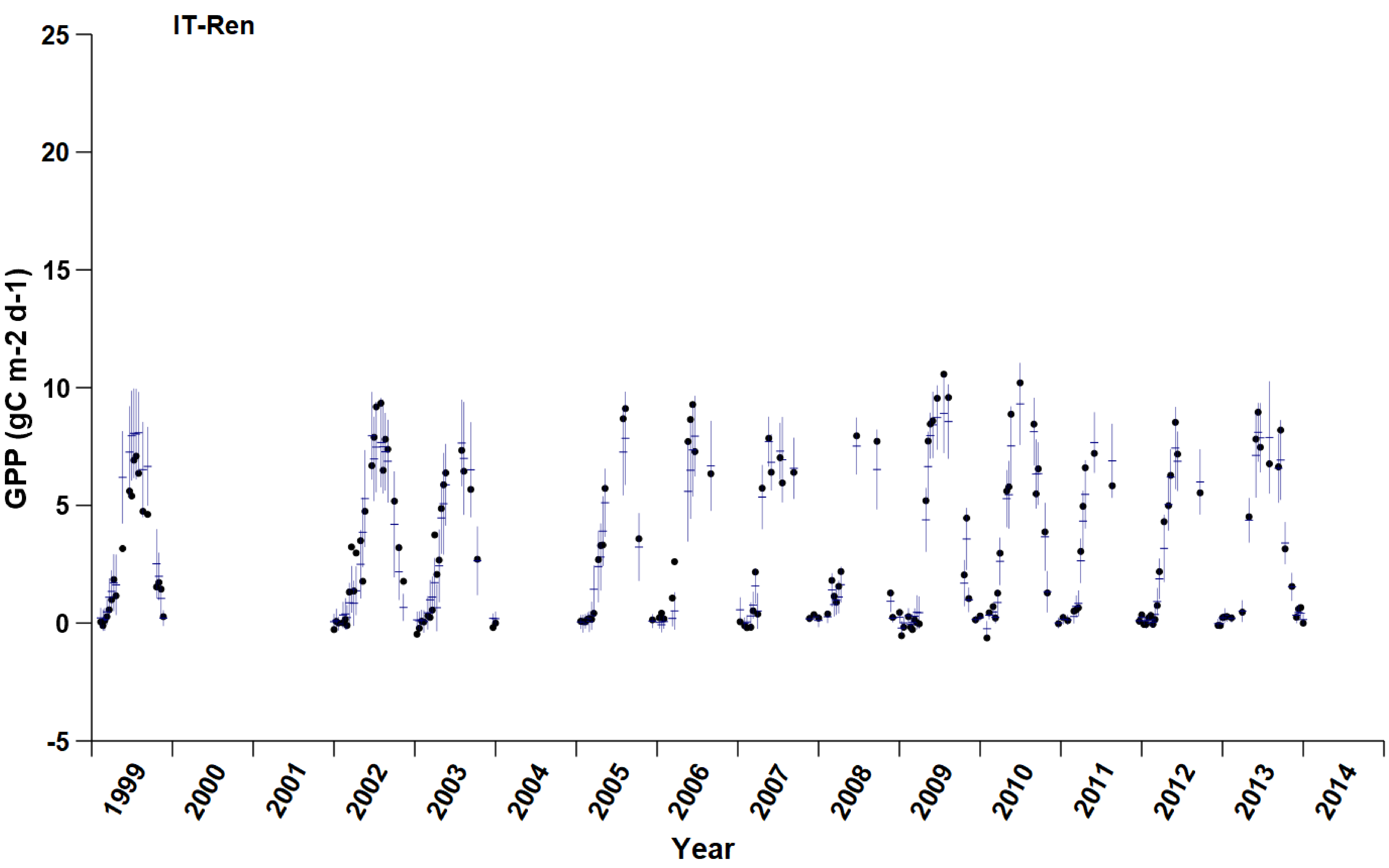

IT-Ren
GPP (gC m-2 d-1)
25
20
15
10
5
0
-5
1999
2000
2001
2002
2003
2004
2005
2006
2007
2008
2009
2010
2011
2012
2013
2014
Year

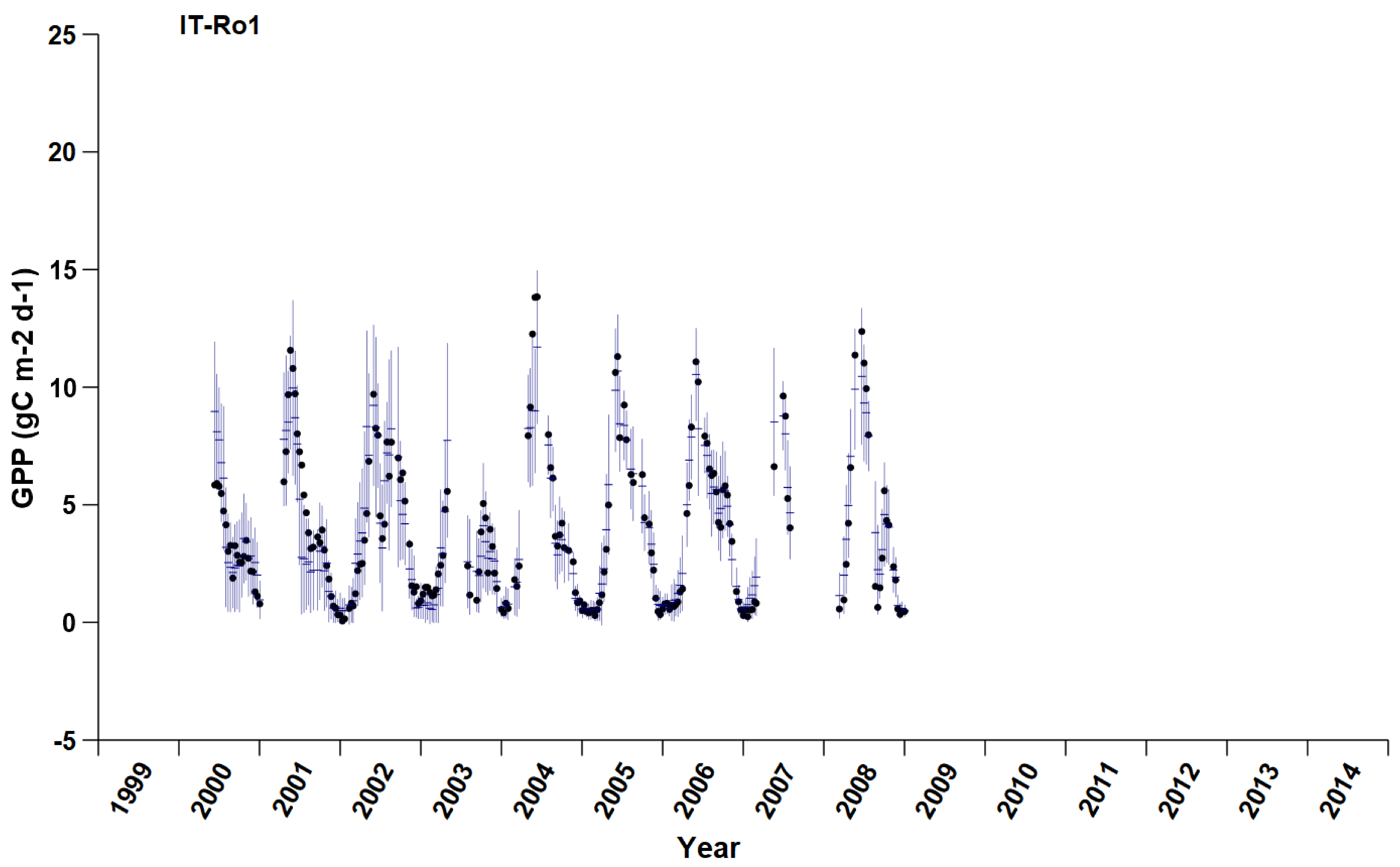
IT-Ro1
GPP (gC m-2 d-1)
25
20
15
10
5
0
-5
1999
2000
2001
2002
2003
2004
2005
2006
2007
2008
2009
2010
2011
2012
2013
2014
Year

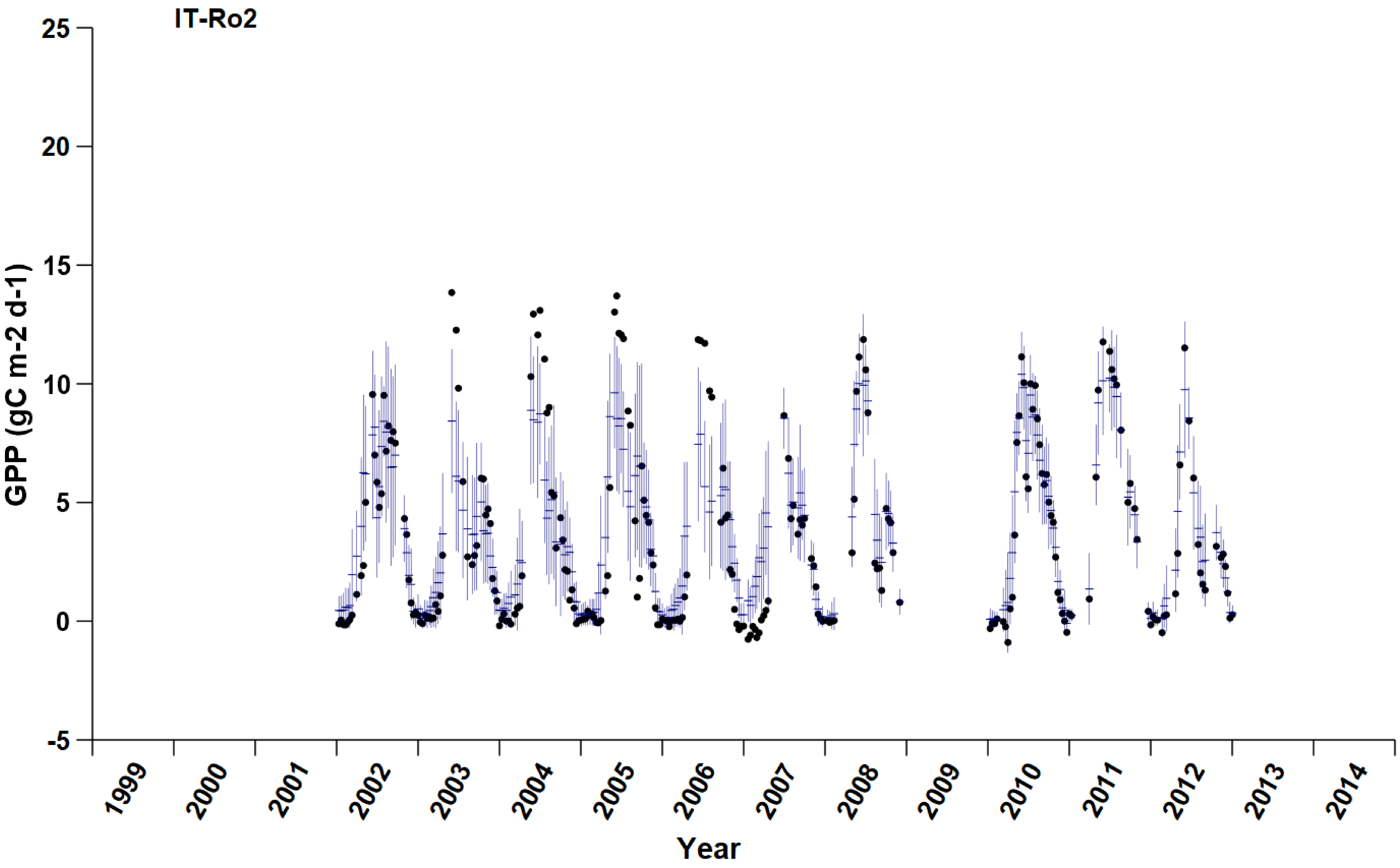
IT-Ro2
GPP (gC m-2 d-1)
25
20
15
10
5
0
-5
1999
2000
2001
2002
2003
2004
2005
2006
2007
2008
2009
2010
2011
2012
2013
2014
Year

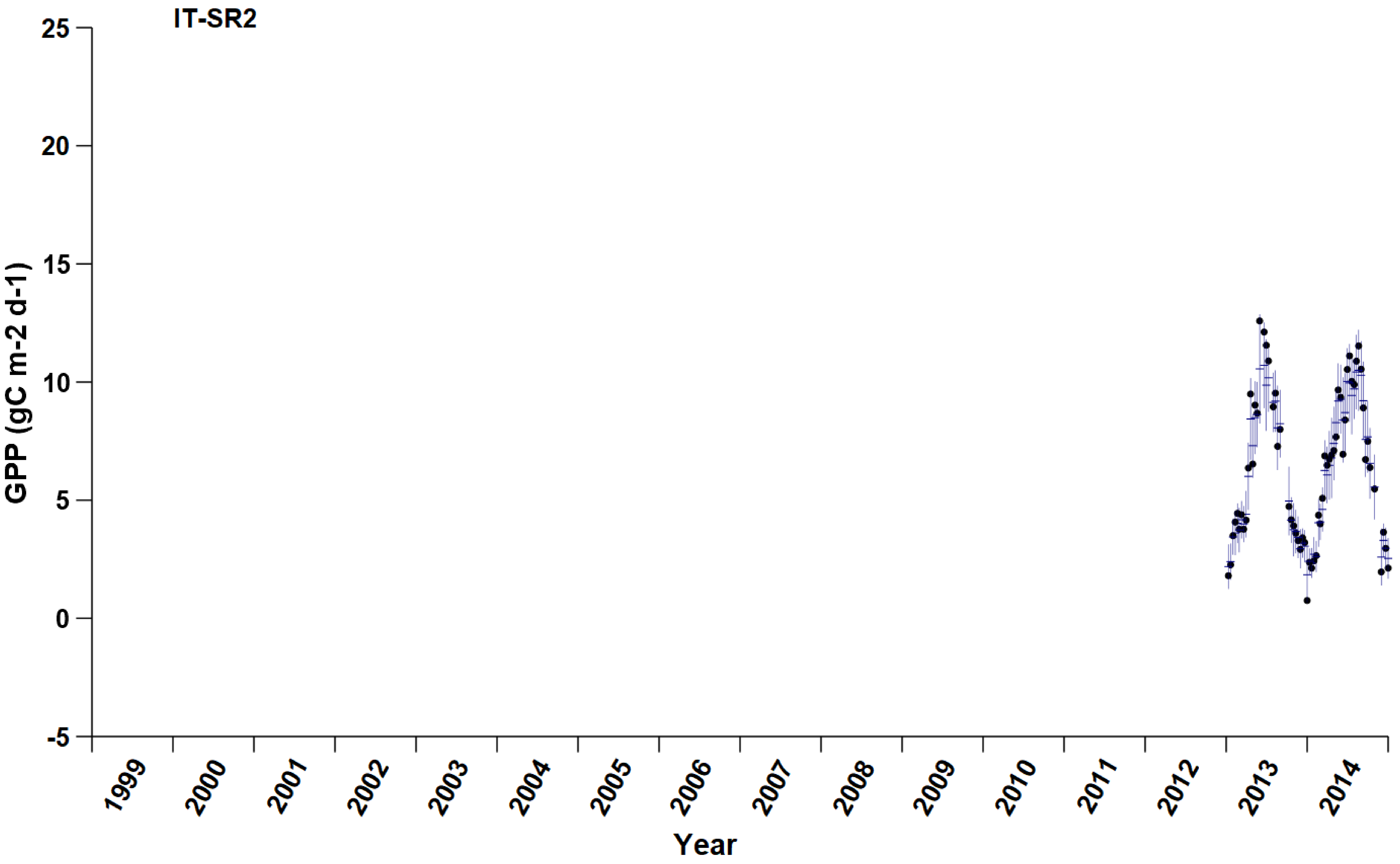
IT-SR2
GPP (gC m-2 d-1)
25
20
15
10
5
0
-5
1999
2000
2001
2002
2003
2004
2005
2006
2007
2008
2009
2010
2011
2012
2013
2014
Year

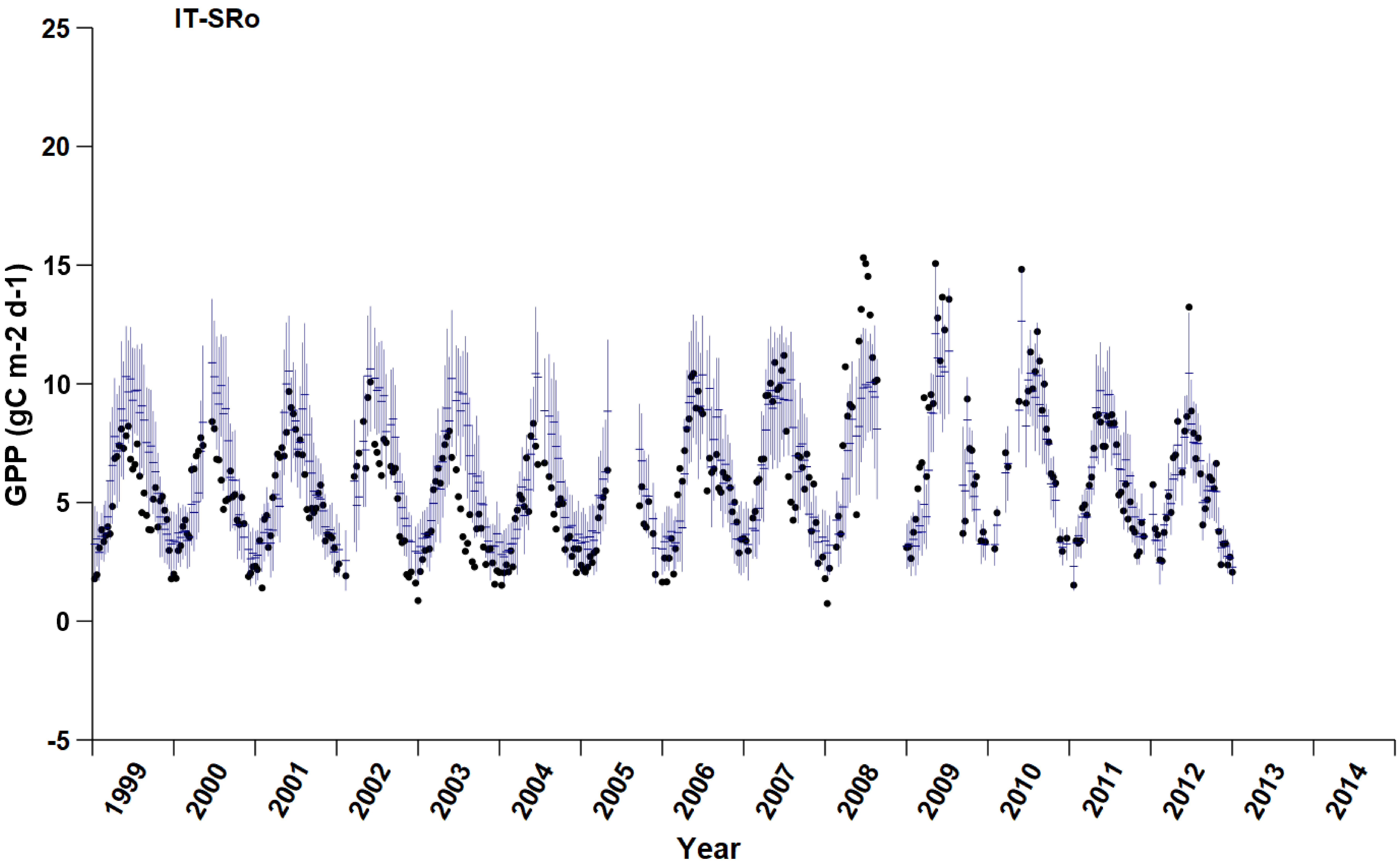

IT-SRo
GPP (gC m-2 d-1)
25
20
15
10
5
0
-5
1999
2000
2001
2002
2003
2004
2005
2006
2007
2008
2009
2010
2011
2012
2013
2014
Year

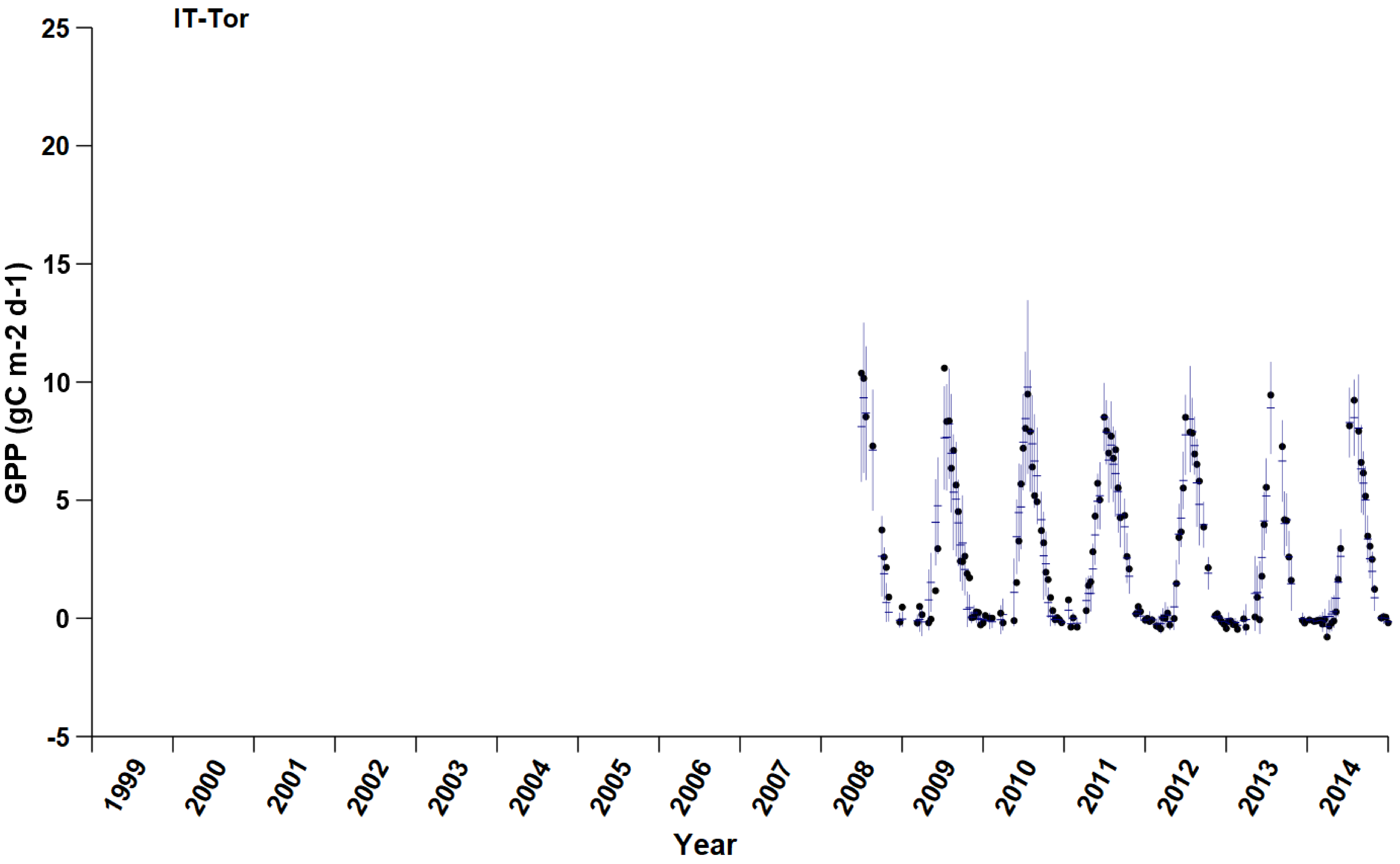
IT-Tor
GPP (gC m-2 d-1)
25
20
15
10
5
0
-5
1999
2000
2001
2002
2003
2004
2005
2006
2007
2008
2009
2010
2011
2012
2013
2014
Year

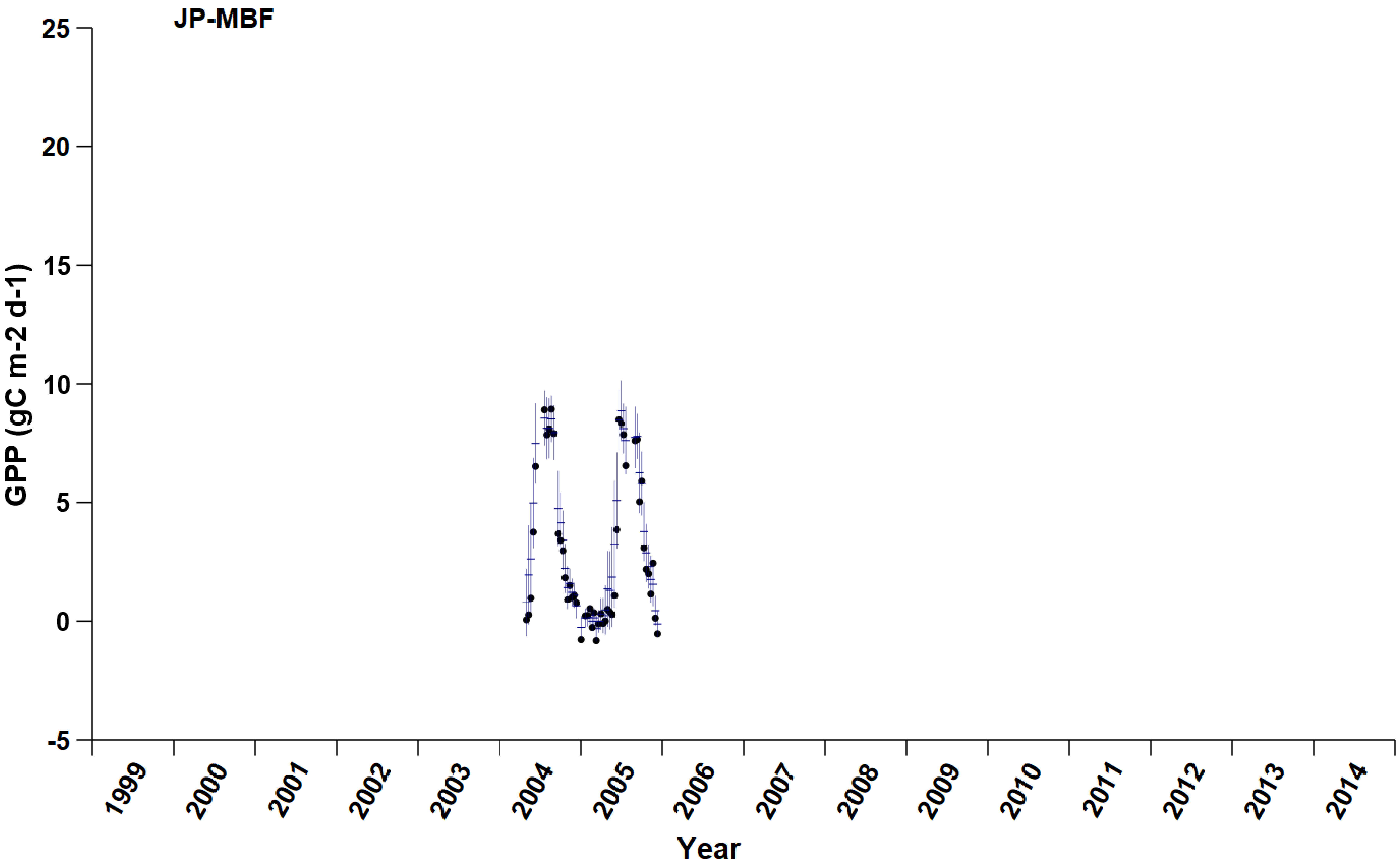
JP-MBF
GPP (gC m-2 d-1)
25
20
15
10
5
0
-5
1999
2000
2001
2002
2003
2004
2005
2006
2007
2008
2009
2010
2011
2012
2013
2014
Year

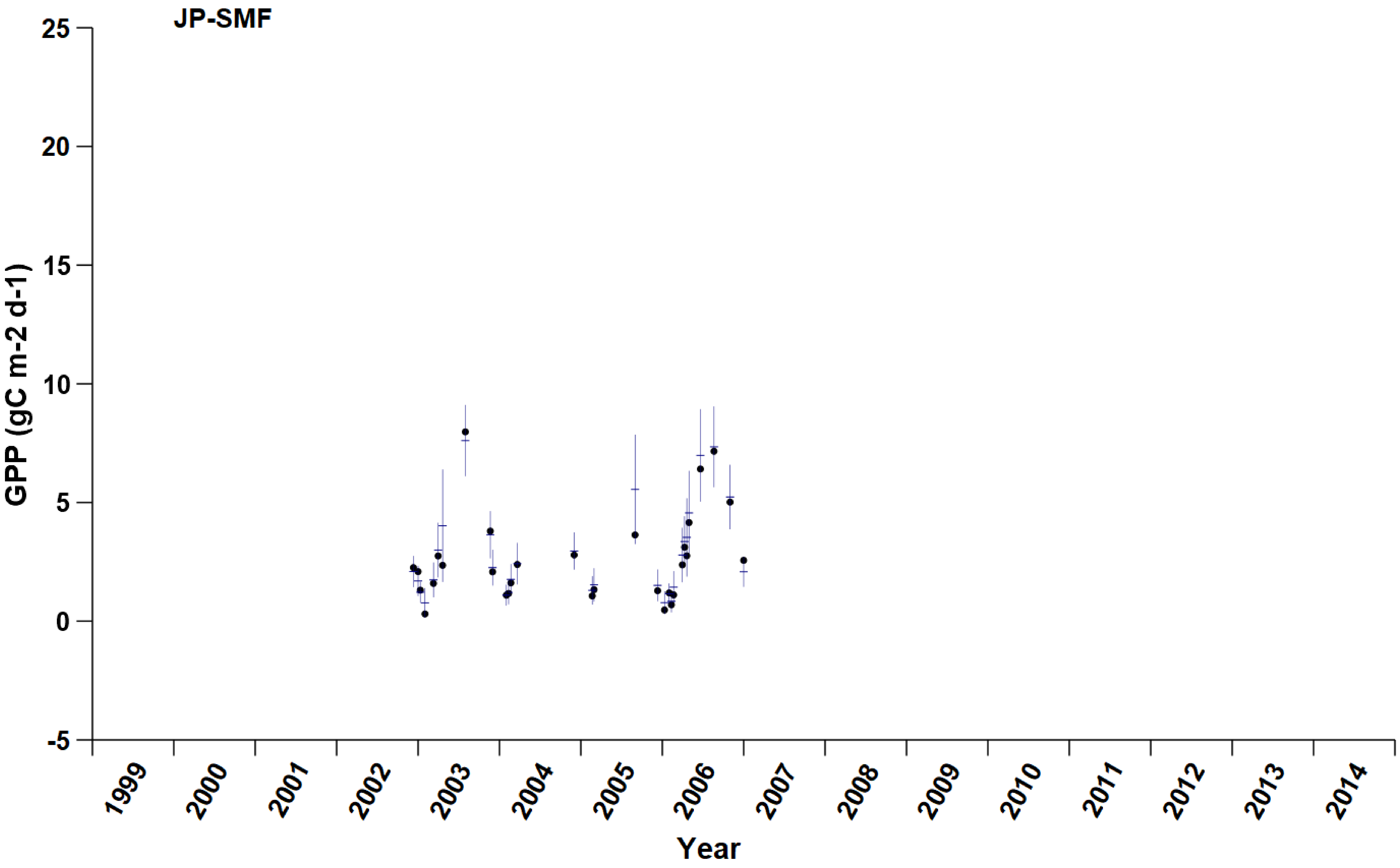

JP-SMF
GPP (gC m-2 d-1)
25
20
15
10
5
0
-5
1999
2000
2001
2002
2003
2004
2005
2006
2007
2008
2009
2010
2011
2012
2013
2014
Year

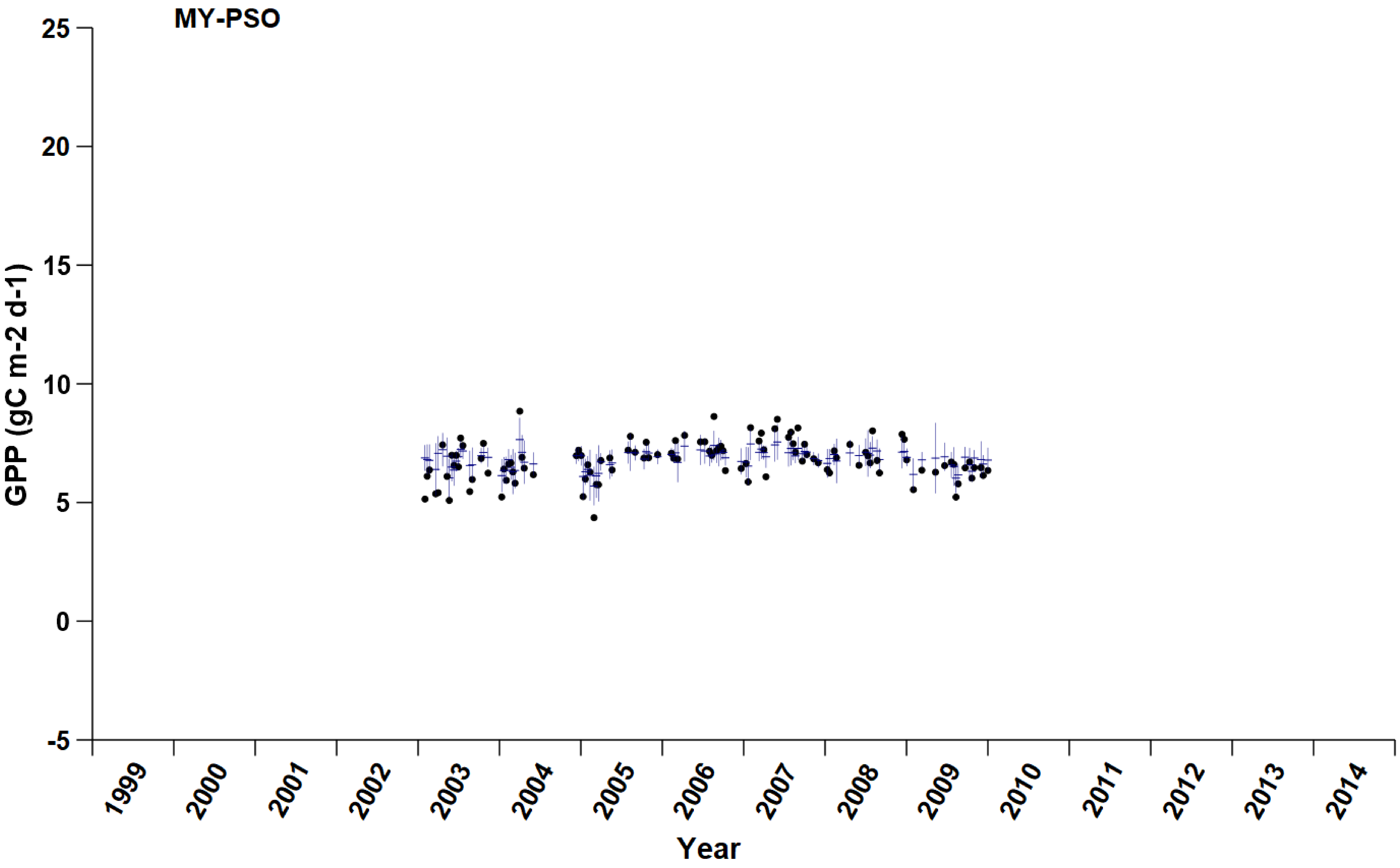
MY-PSO
GPP (gC m-2 d-1)
25
20
15
10
5
0
-5
1999
2000
2001
2002
2003
2004
2005
2006
2007
2008
2009
2010
2011
2012
2013
2014
Year

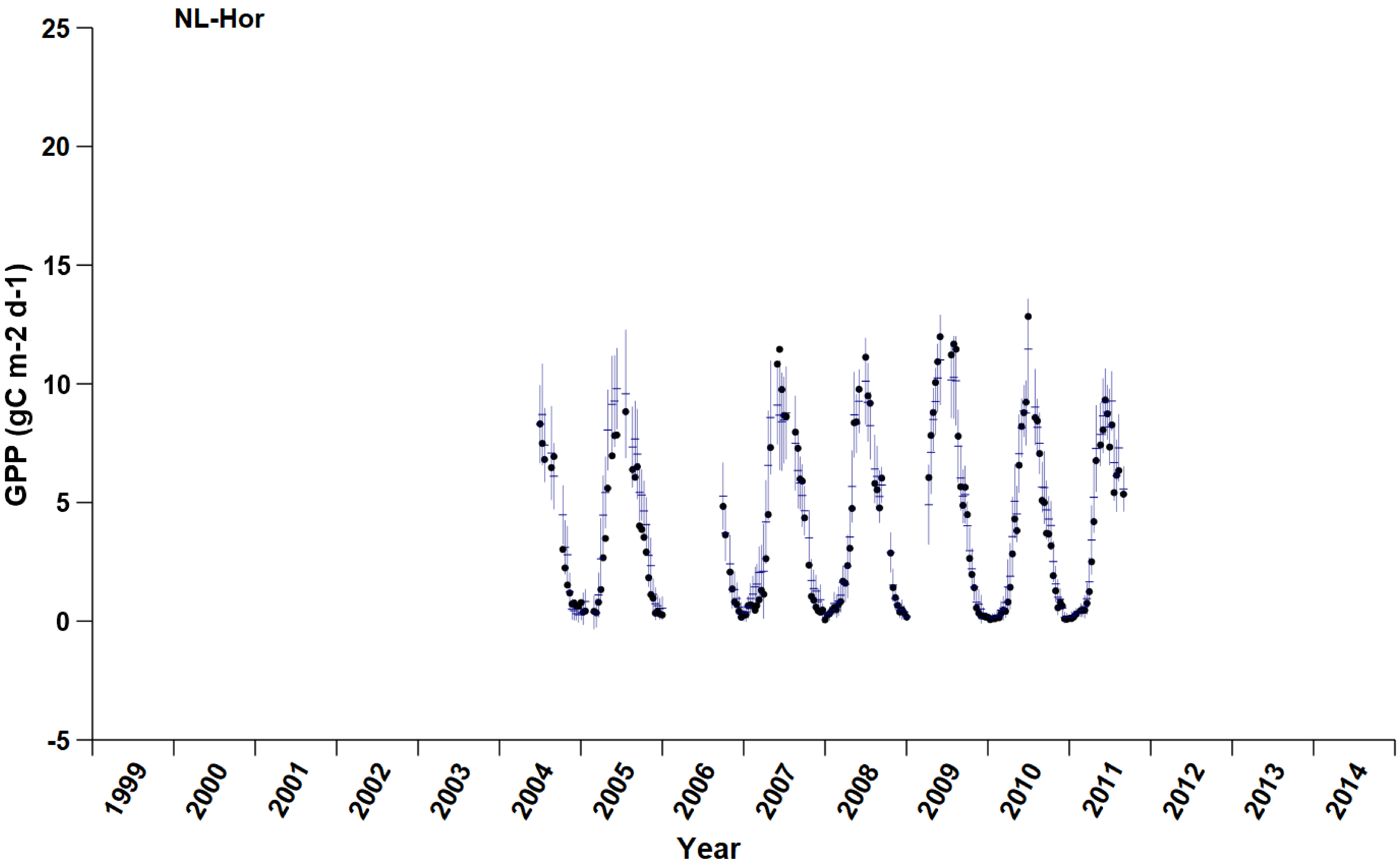
NL-Hor
GPP (gC m-2 d-1)
25
20
15
10
5
0
-5
1999
2000
2001
2002
2003
2004
2005
2006
2007
2008
2009
2010
2011
2012
2013
2014
Year

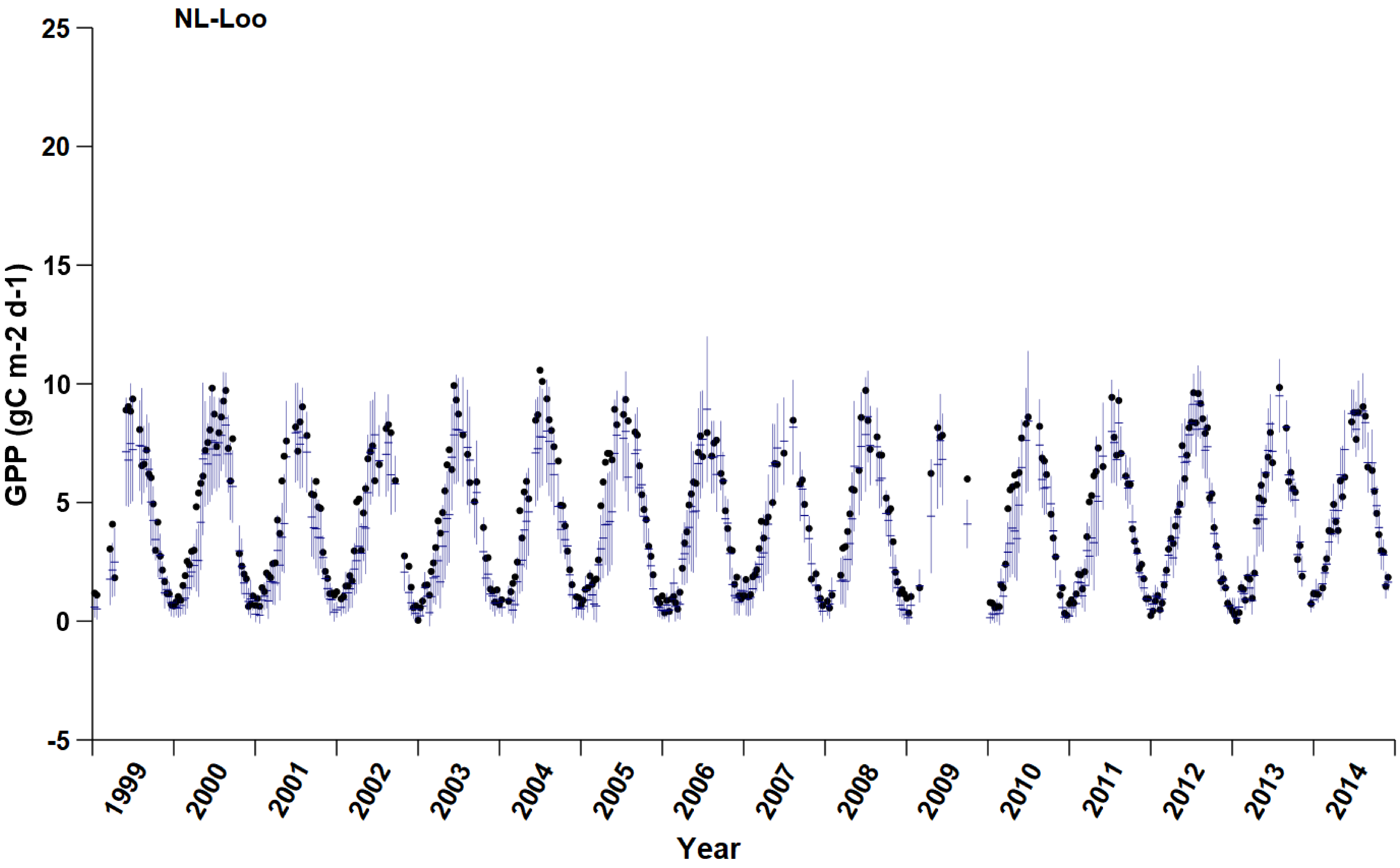
NL-Loo
GPP (gC m-2 d-1)
25
20
15
10
5
0
-5
1999
2000
2001
2002
2003
2004
2005
2006
2007
2008
2009
2010
2011
2012
2013
2014
Year

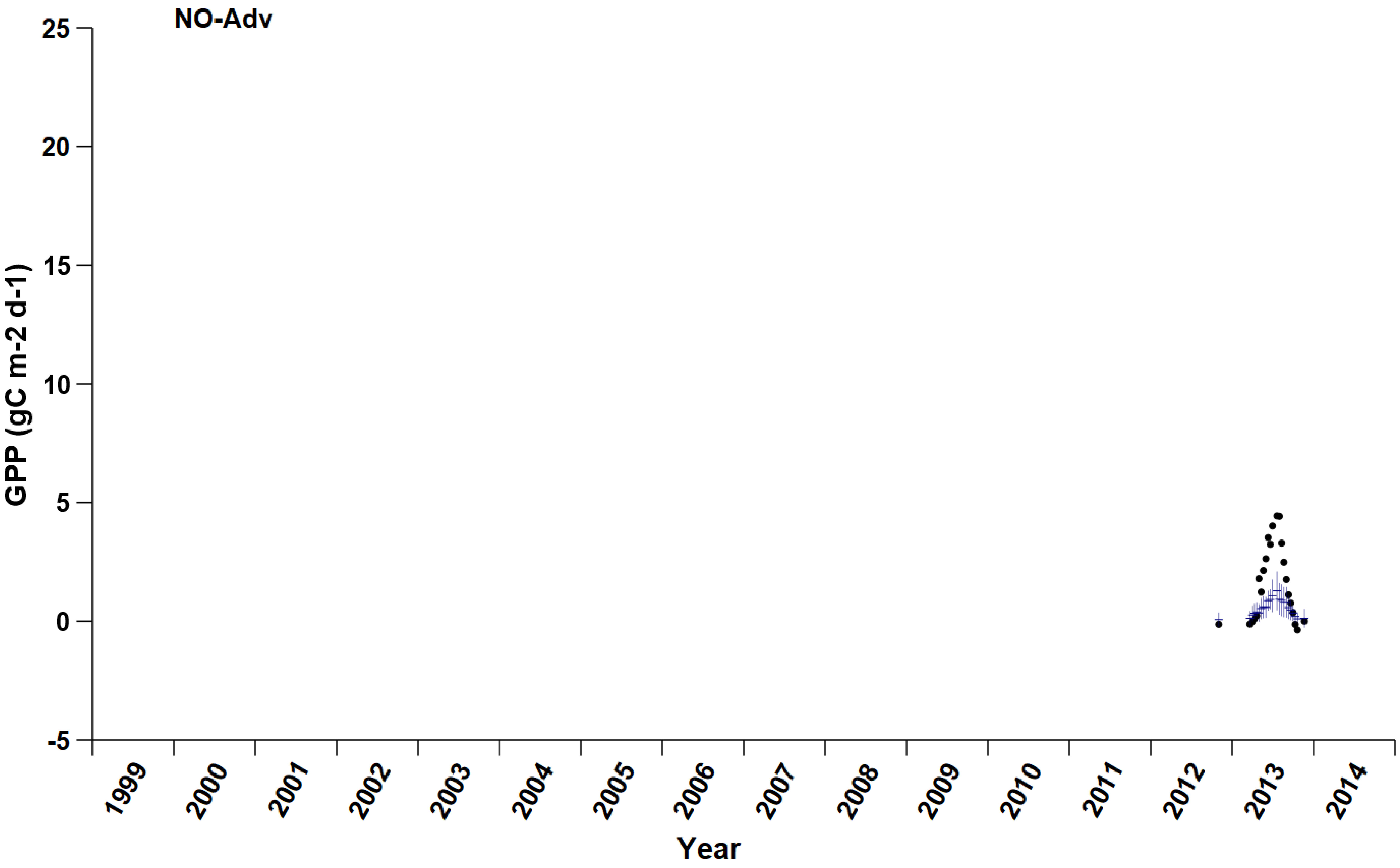
NO-Adv
GPP (gC m-2 d-1)
25
20
15
10
5
0
-5
1999
2000
2001
2002
2003
2004
2005
2006
2007
2008
2009
2010
2011
2012
2013
2014
Year

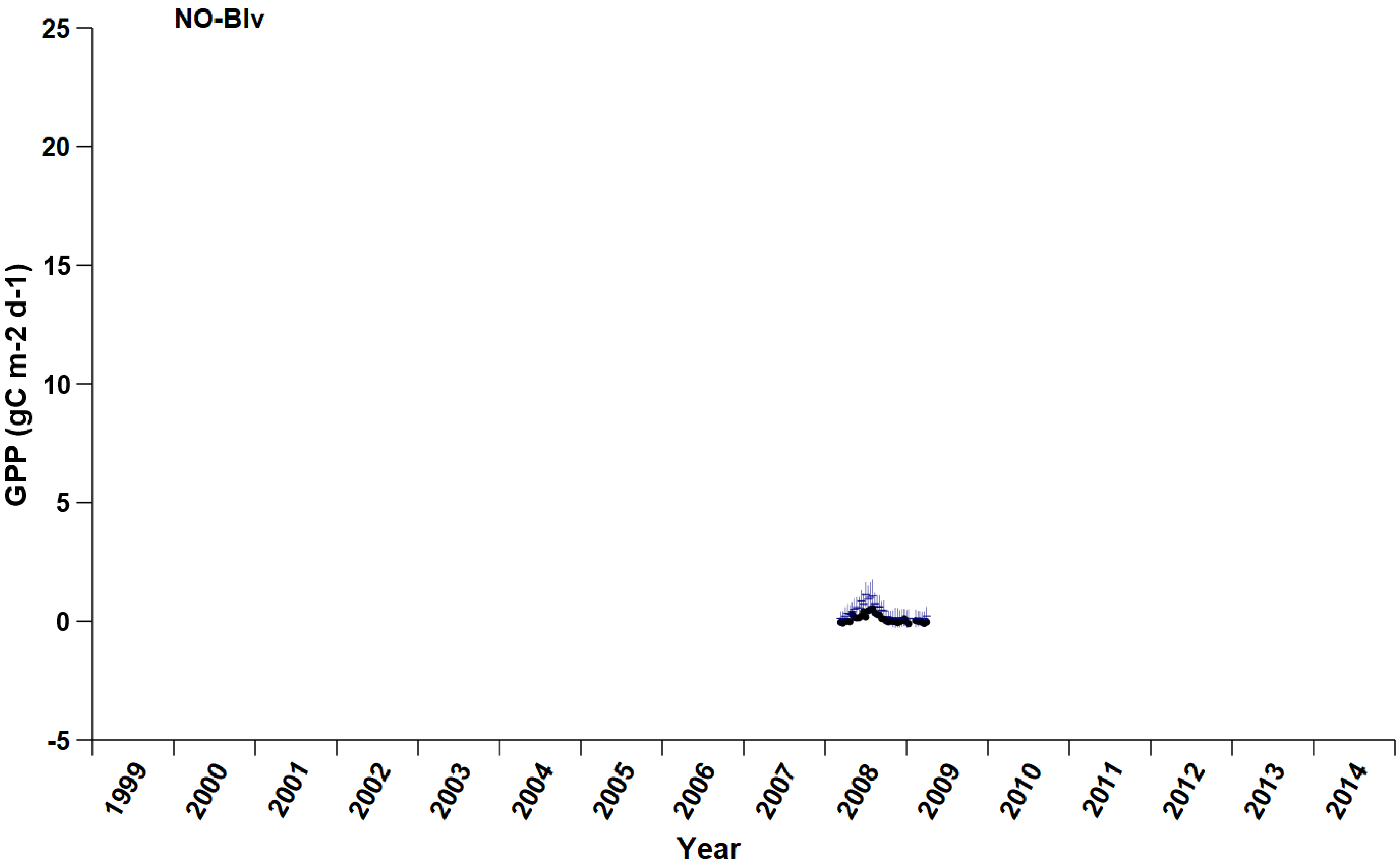

NO-Blv
GPP (gC m-2 d-1)
25
20
15
10
5
0
-5
1999
2000
2001
2002
2003
2004
2005
2006
2007
2008
2009
2010
2011
2012
2013
2014
Year

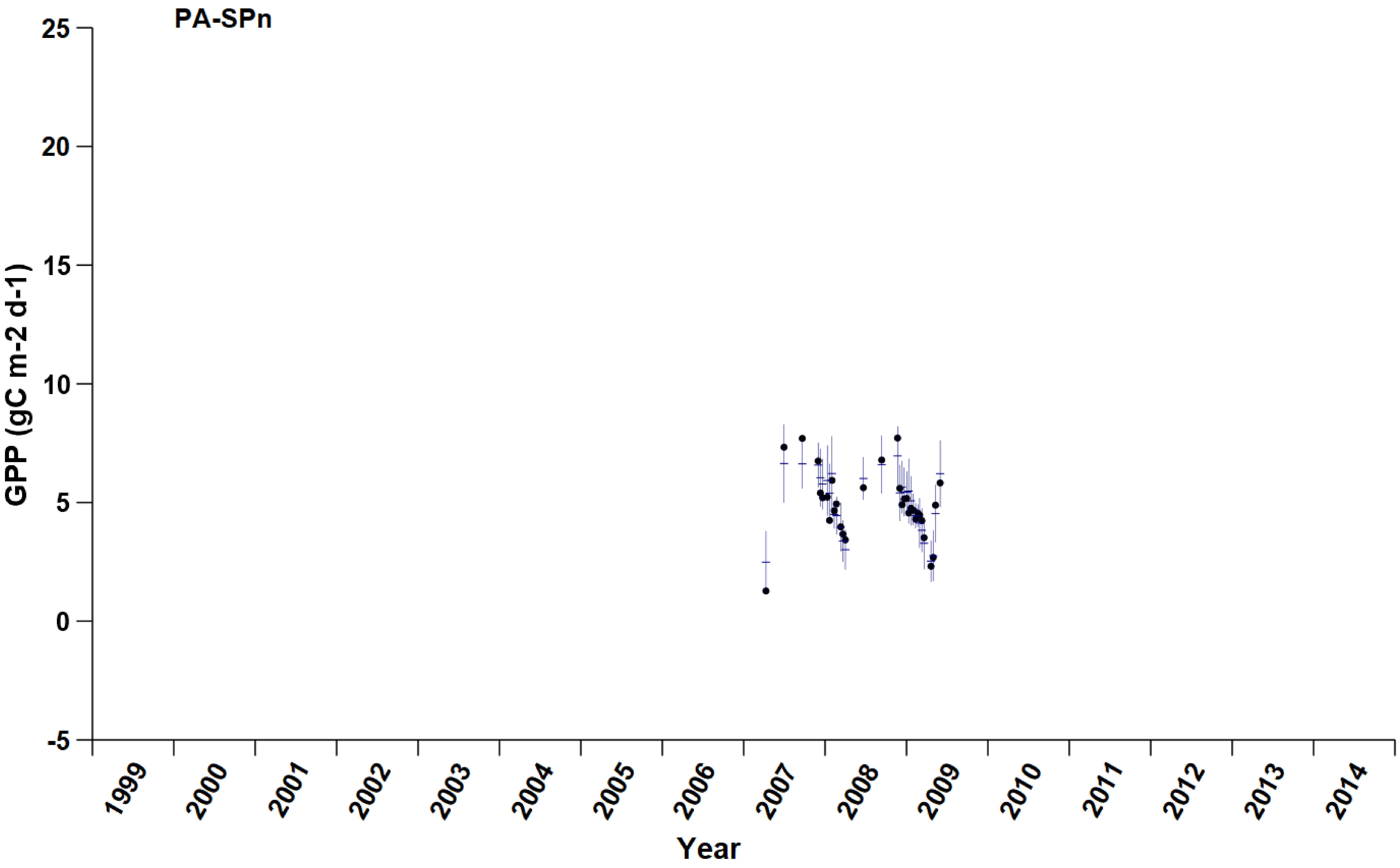

PA-SPn
GPP (gC m-2 d-1)
25
20
15
10
5
0
-5
1999
2000
2001
2002
2003
2004
2005
2006
2007
2008
2009
2010
2011
2012
2013
2014
Year

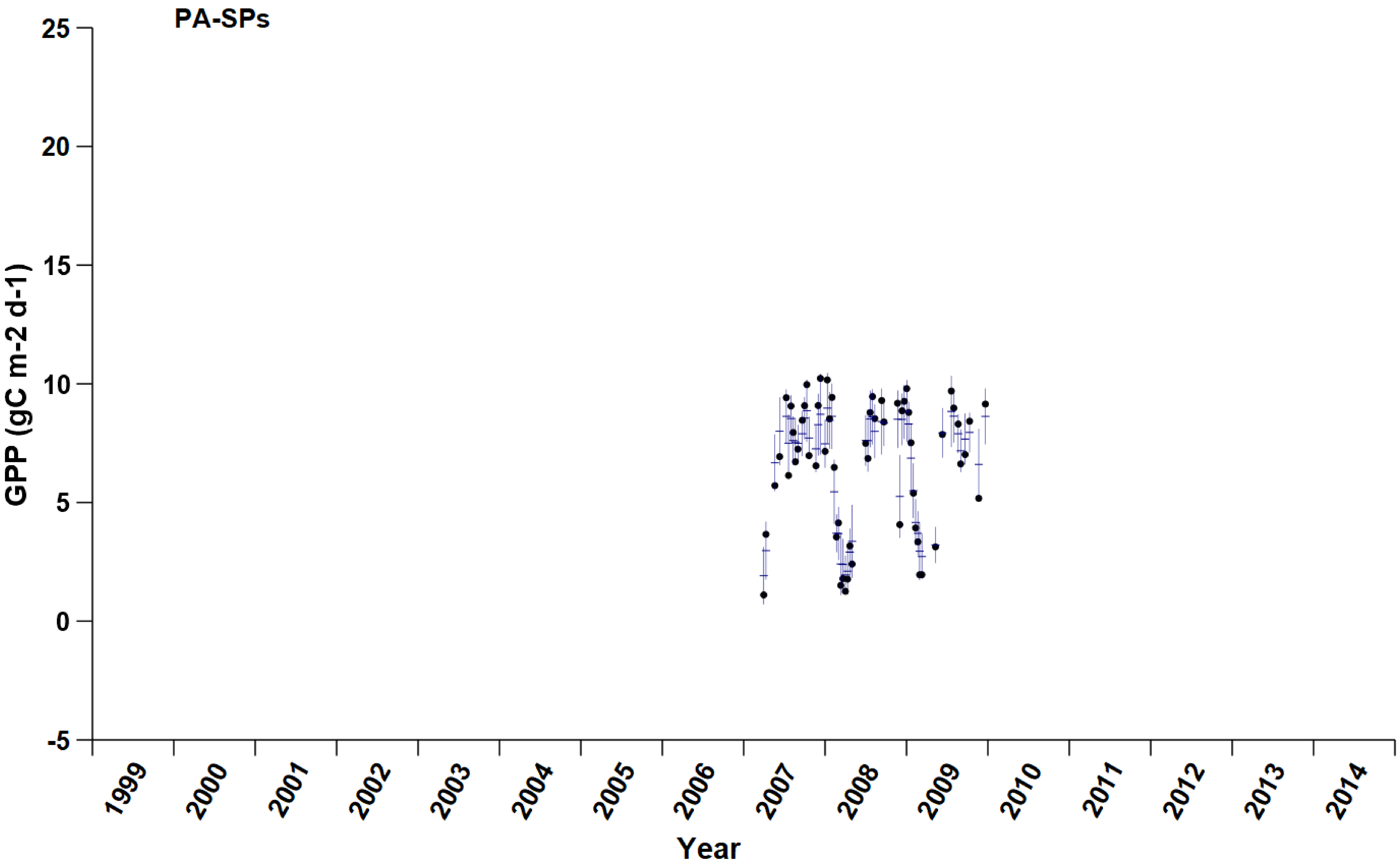

PA-SPs
GPP (gC m-2 d-1)
25
20
15
10
5
0
-5
1999
2000
2001
2002
2003
2004
2005
2006
2007
2008
2009
2010
2011
2012
2013
2014
Year

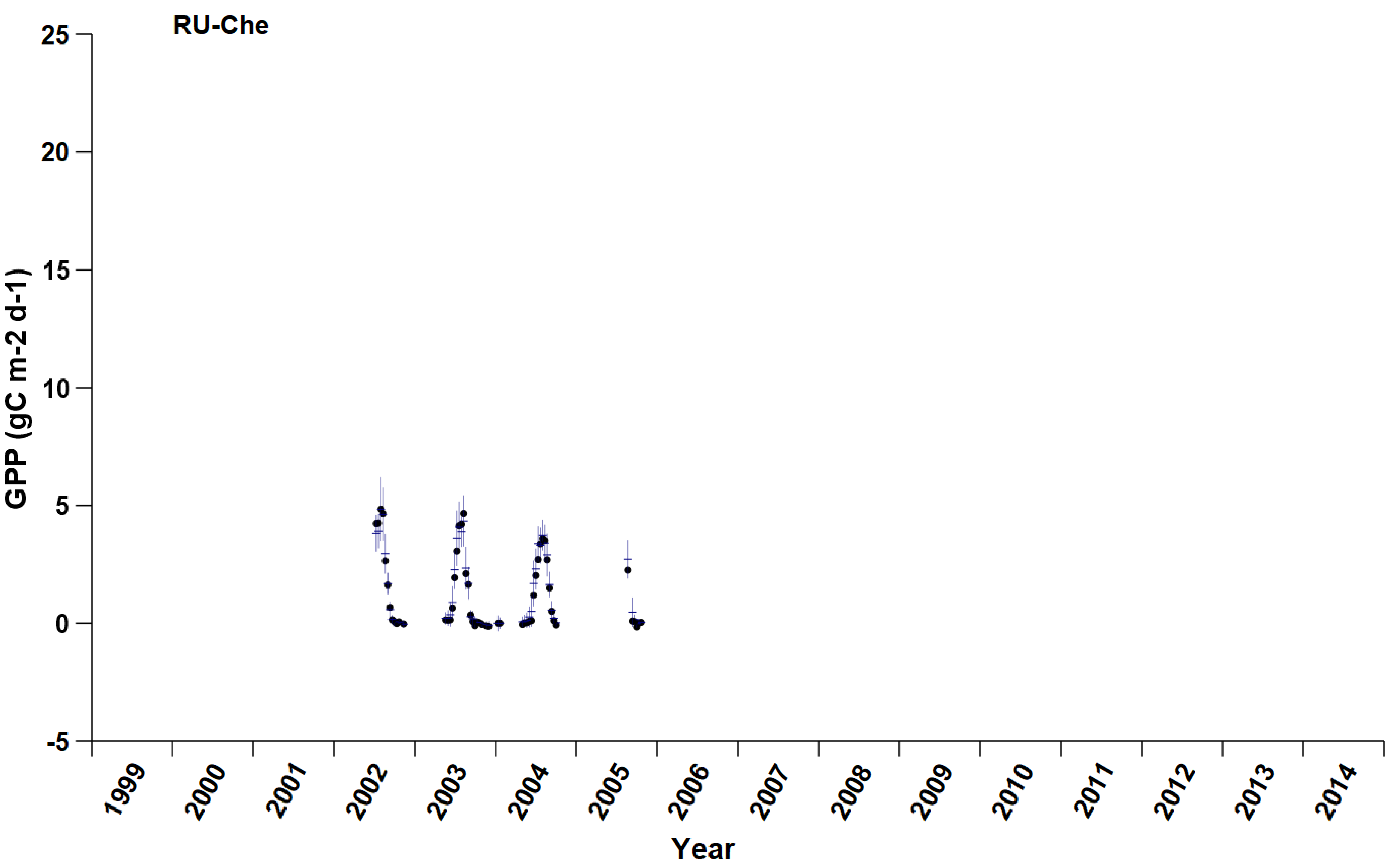

RU-Che
GPP (gC m-2 d-1)
25
20
15
10
5
0
-5
1999
2000
2001
2002
2003
2004
2005
2006
2007
2008
2009
2010
2011
2012
2013
2014
Year

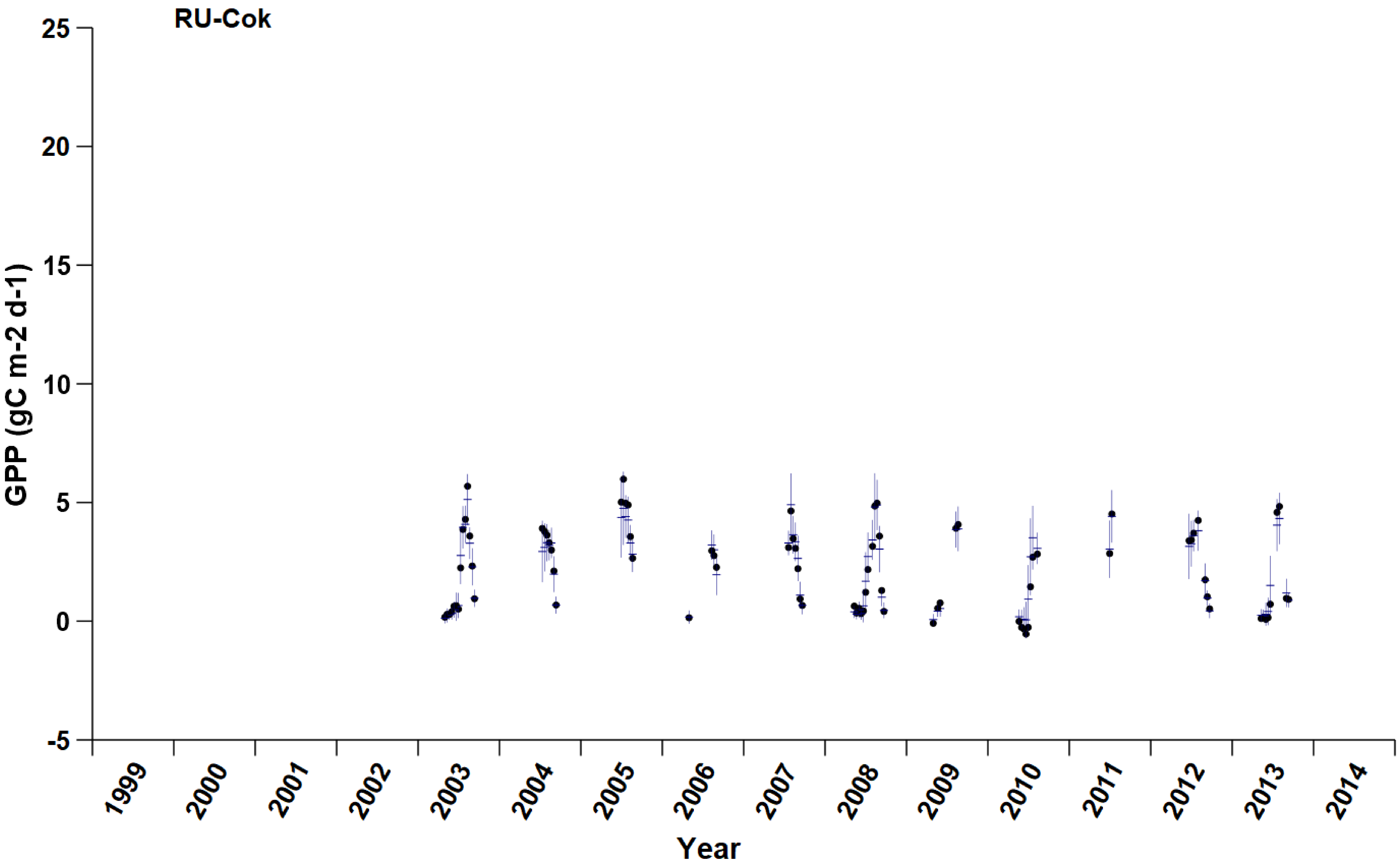

RU-Cok
GPP (gC m-2 d-1)
25
20
15
10
5
0
-5
1999
2000
2001
2002
2003
2004
2005
2006
2007
2008
2009
2010
2011
2012
2013
2014
Year

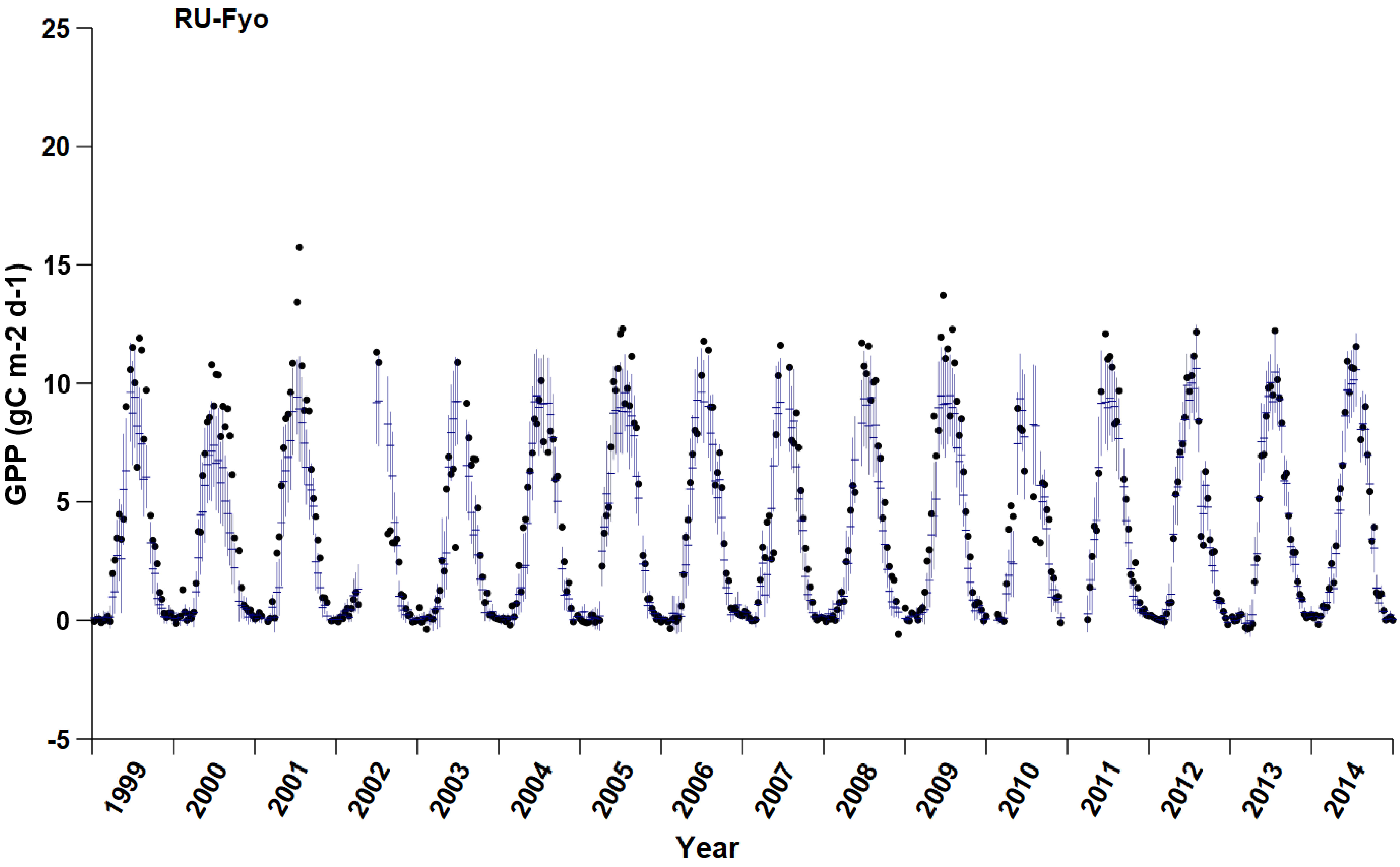

RU-Fyo
GPP (gC m-2 d-1)
25
20
15
10
5
0
-5
1999
2000
2001
2002
2003
2004
2005
2006
2007
2008
2009
2010
2011
2012
2013
2014
Year

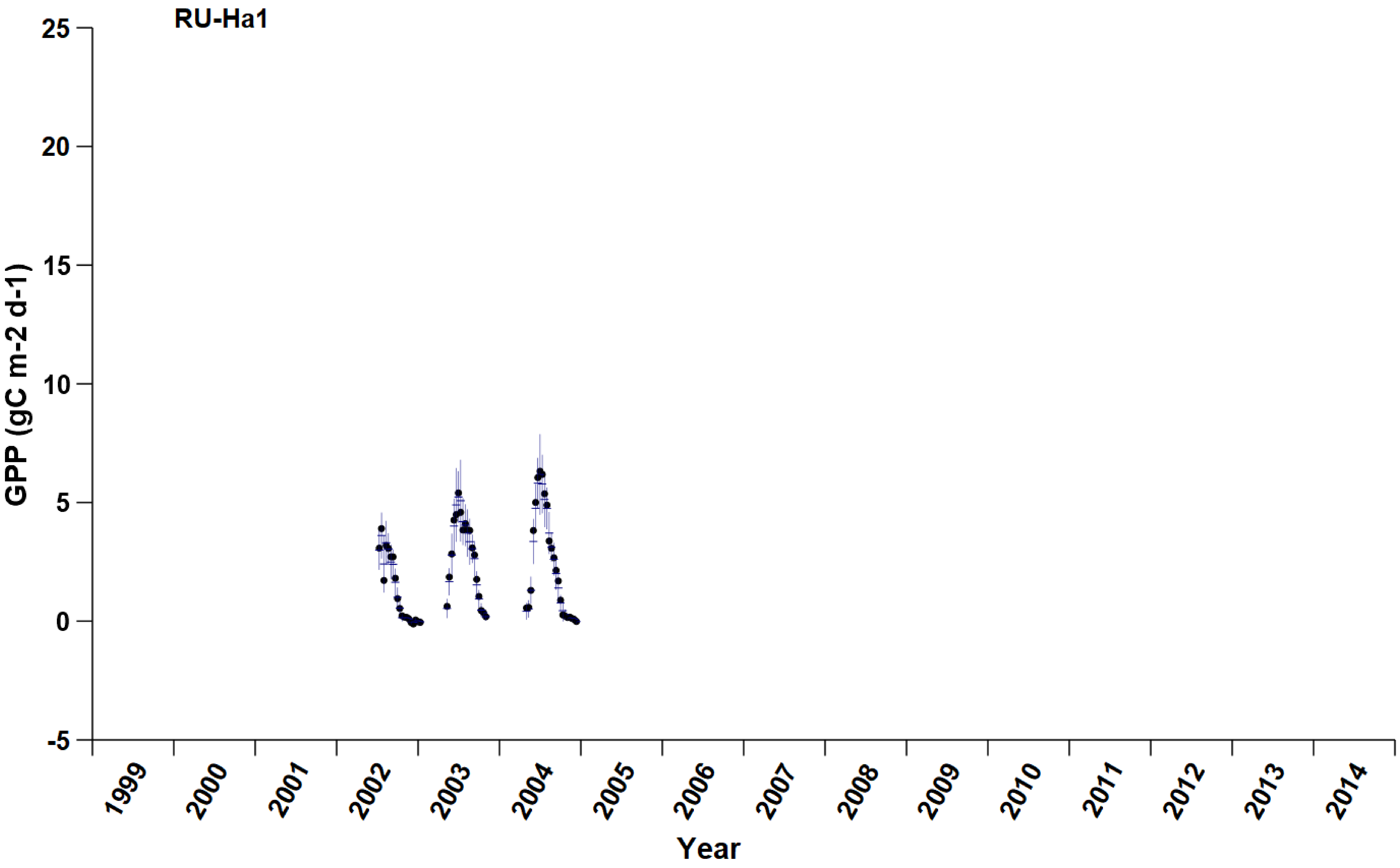
RU-Ha1
GPP (gC m-2 d-1)
25
20
15
10
5
0
-5
1999
2000
2001
2002
2003
2004
2005
2006
2007
2008
2009
2010
2011
2012
2013
2014
Year

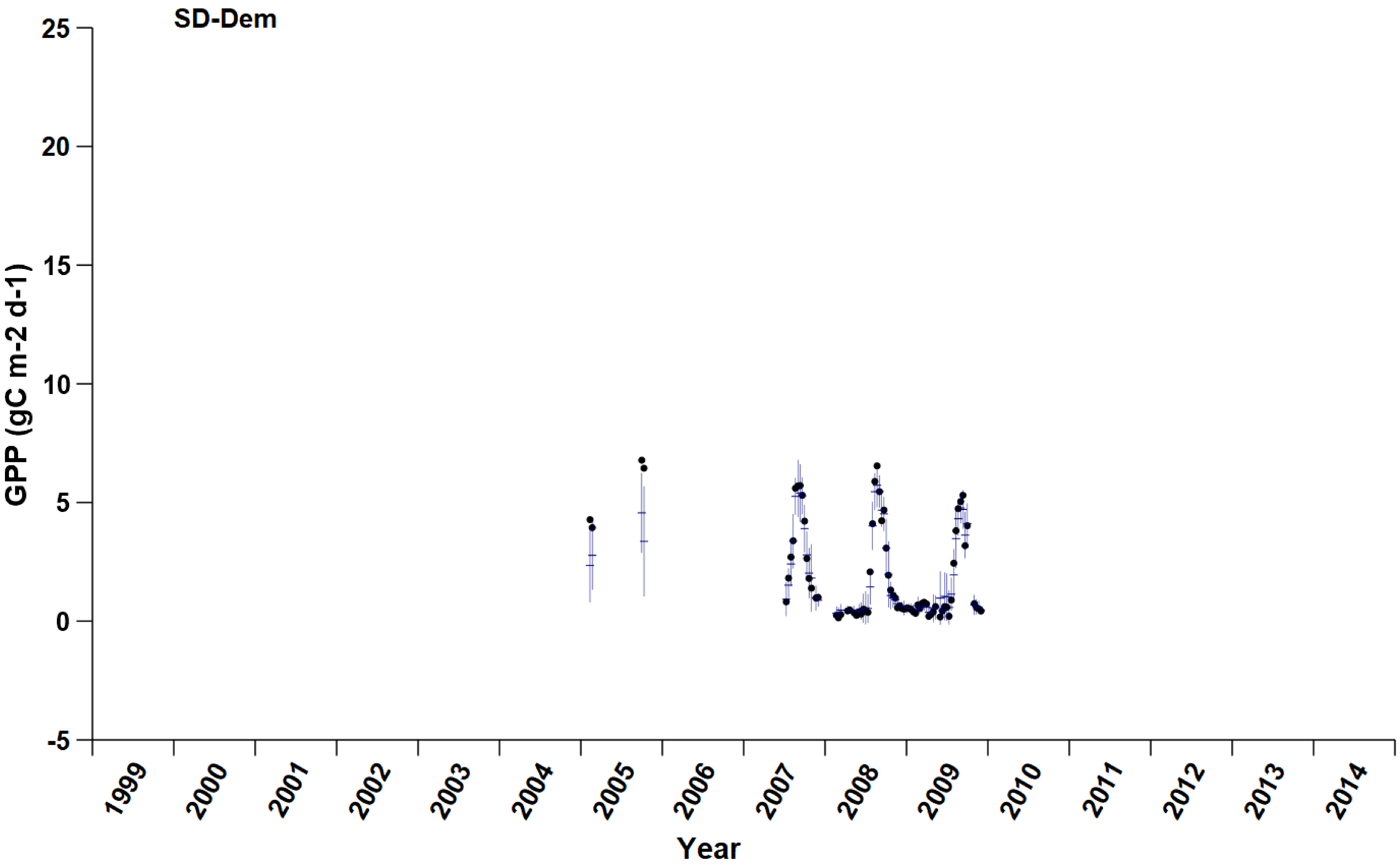

SD-Dem
GPP (gC m-2 d-1)
25
20
15
10
5
0
-5
1999
2000
2001
2002
2003
2004
2005
2006
2007
2008
2009
2010
2011
2012
2013
2014
Year

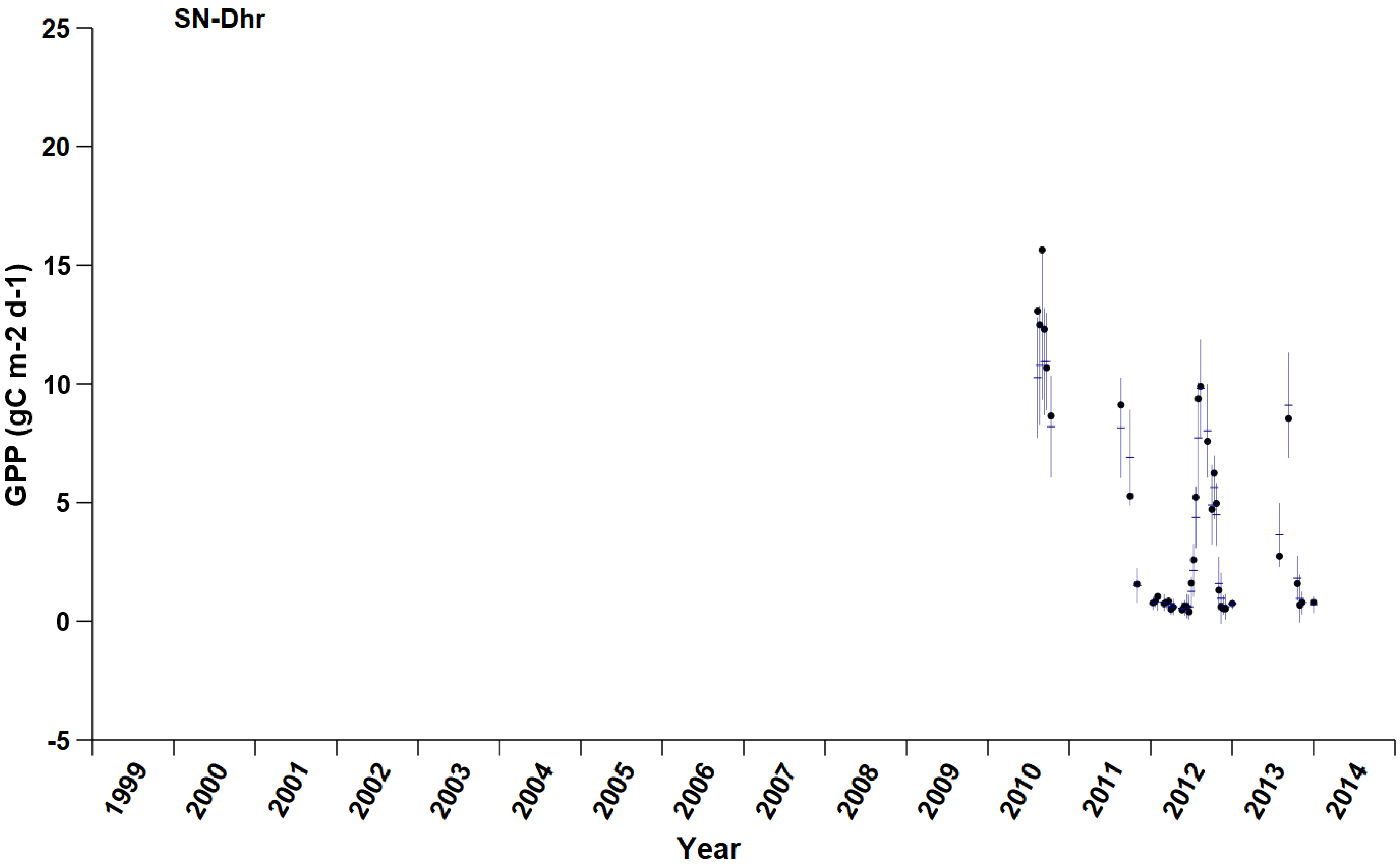
SN-Dhr
GPP (gC m-2 d-1)
25
20
15
10
5
0
-5
1999
2000
2001
2002
2003
2004
2005
2006
2007
2008
2009
2010
2011
2012
2013
2014
Year

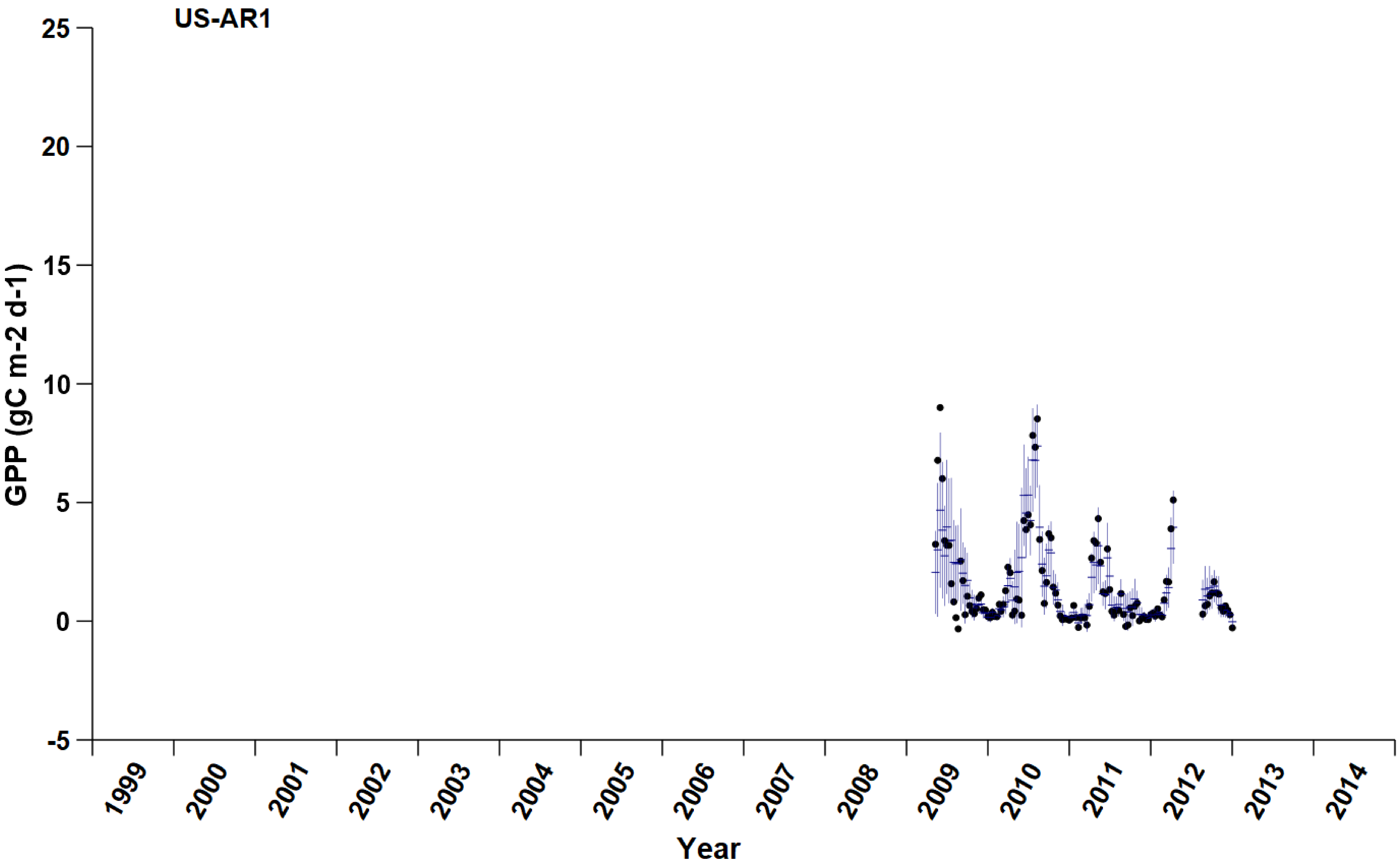

US-AR1
GPP (gC m-2 d-1)
25
20
15
10
5
0
-5
1999
2000
2001
2002
2003
2004
2005
2006
2007
2008
2009
2010
2011
2012
2013
2014
Year

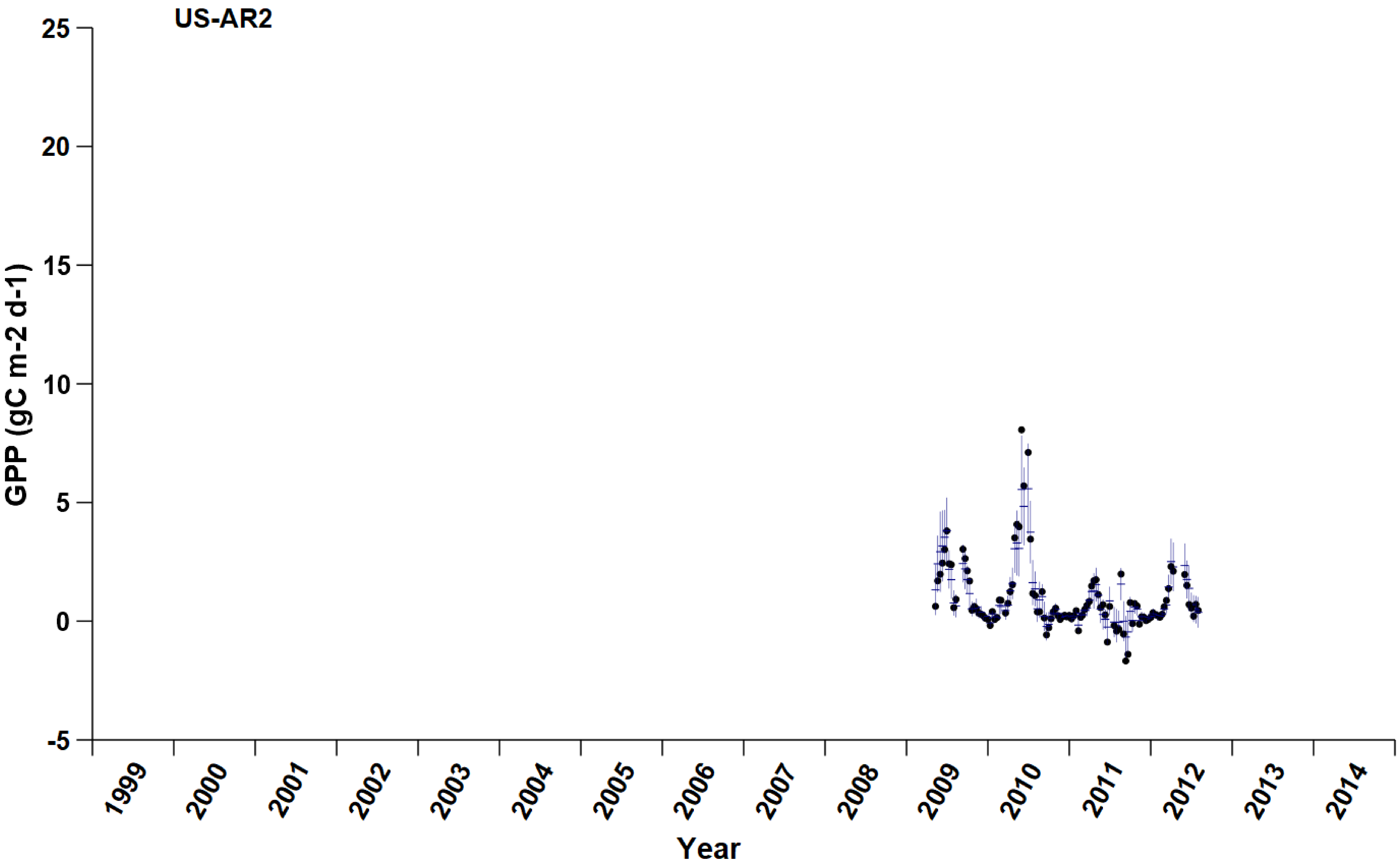

US-AR2
GPP (gC m-2 d-1)
25
20
15
10
5
0
-5
1999
2000
2001
2002
2003
2004
2005
2006
2007
2008
2009
2010
2011
2012
2013
2014
Year

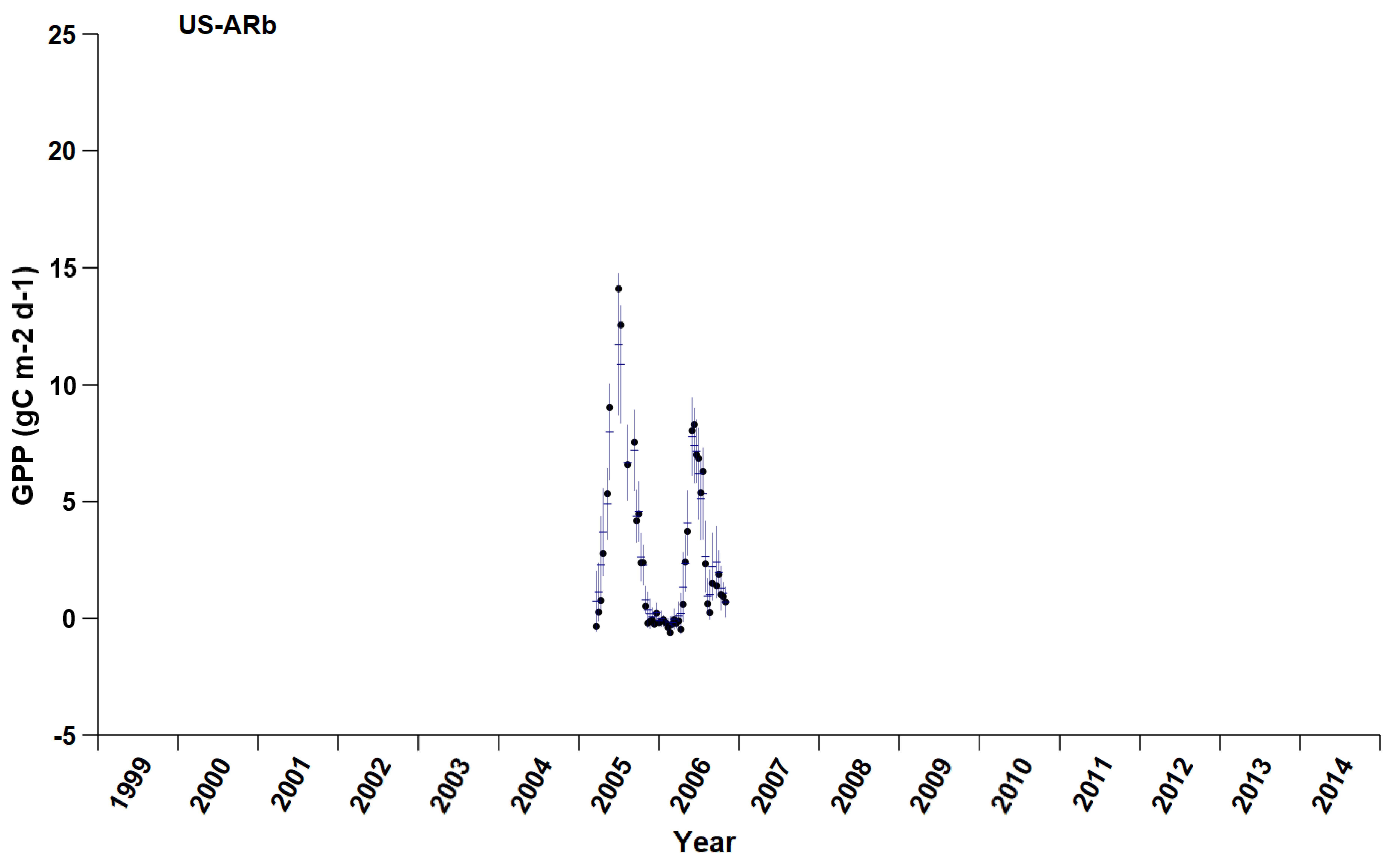

US-ARb
GPP (gC m-2 d-1)
25
20
15
10
5
0
-5
1999
2000
2001
2002
2003
2004
2005
2006
2007
2008
2009
2010
2011
2012
2013
2014
Year

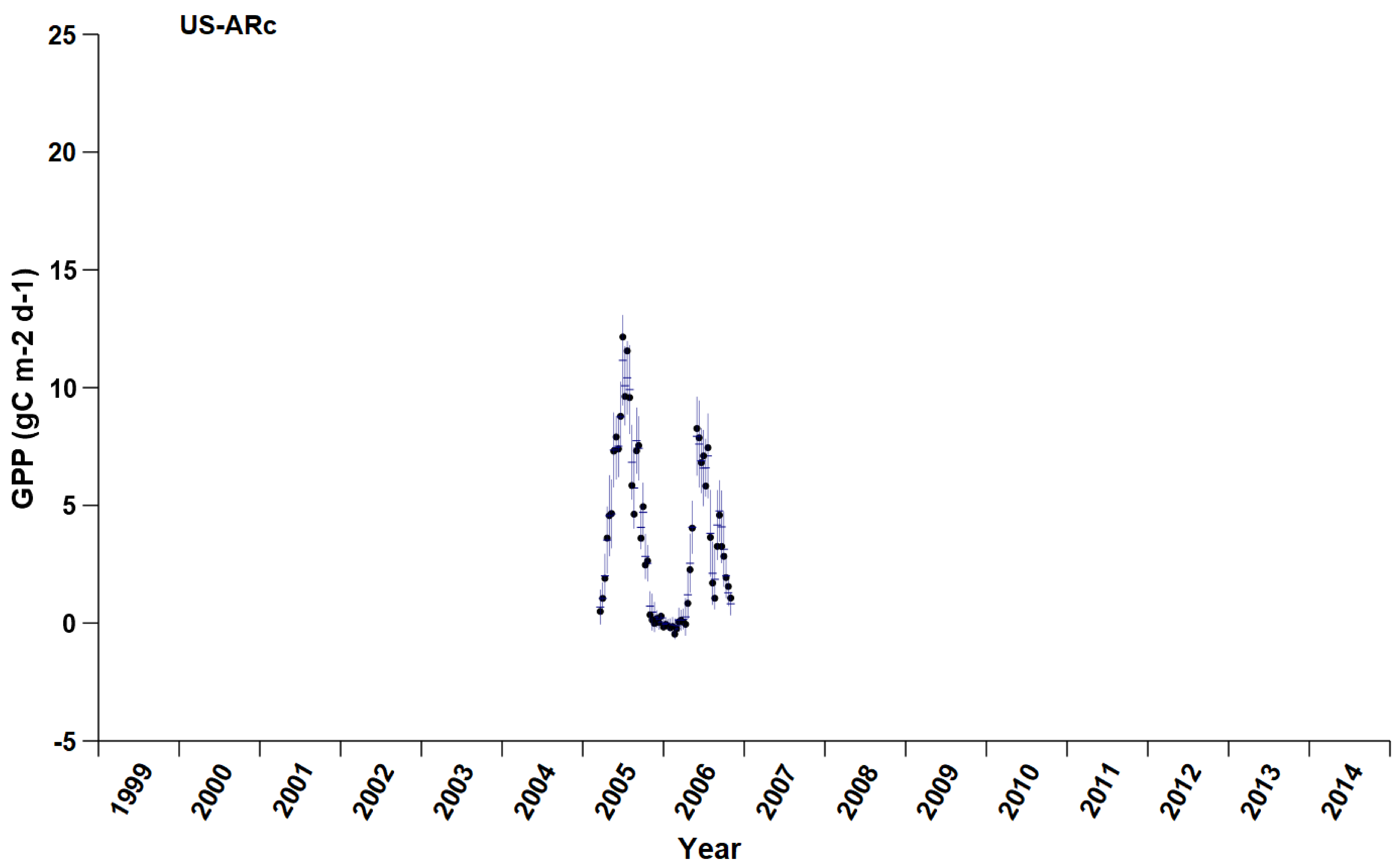
US-ARc
25
20
15
10
5
0
-5
GPP (gC m-2 d-1)
1999
2000
2001
2002
2003
2004
2005
2006
2007
2008
2009
2010
2011
2012
2013
2014
Year

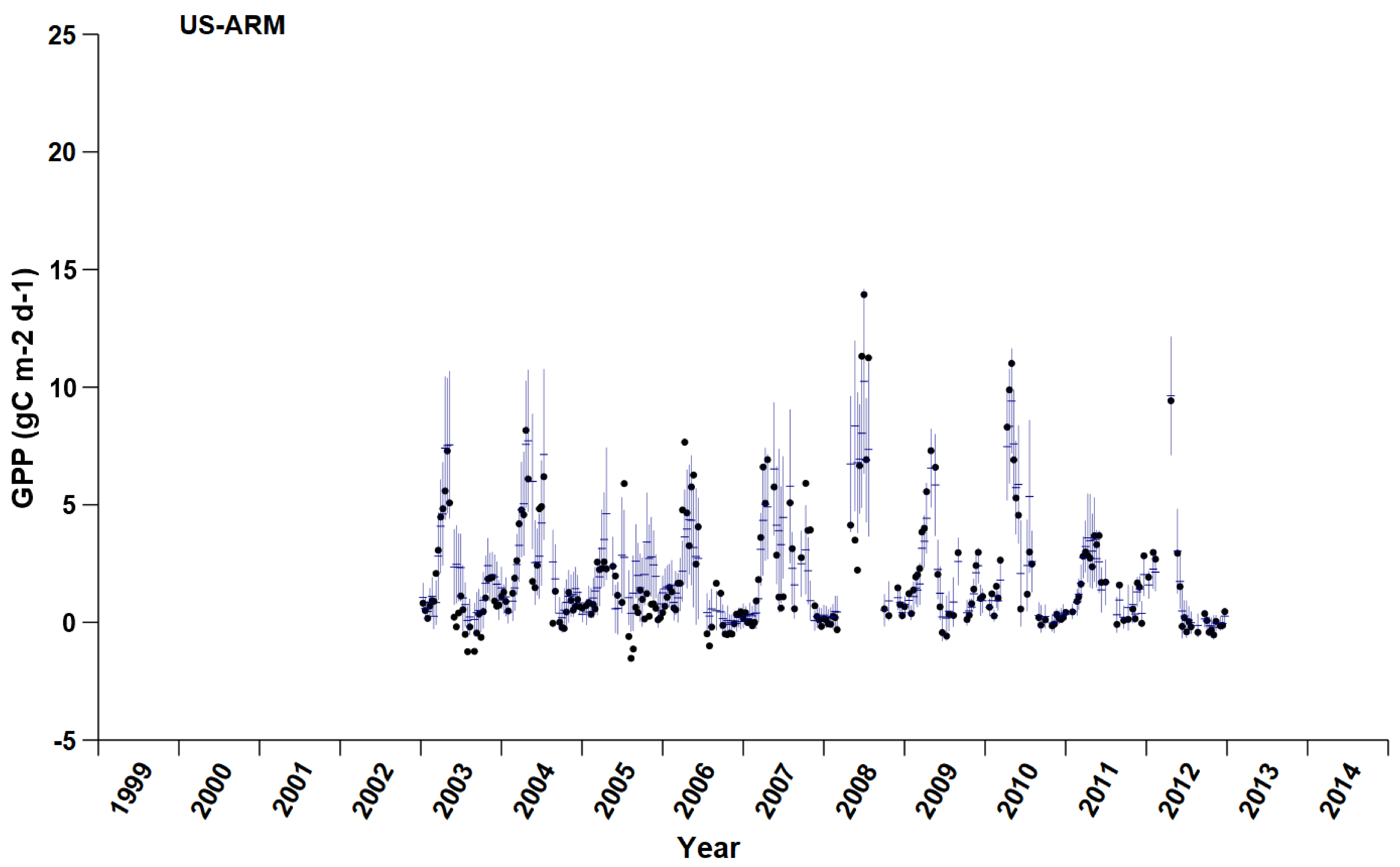

US-ARM
GPP (gC m-2 d-1)
25
20
15
10
5
0
-5
1999
2000
2001
2002
2003
2004
2005
2006
2007
2008
2009
2010
2011
2012
2013
2014
Year

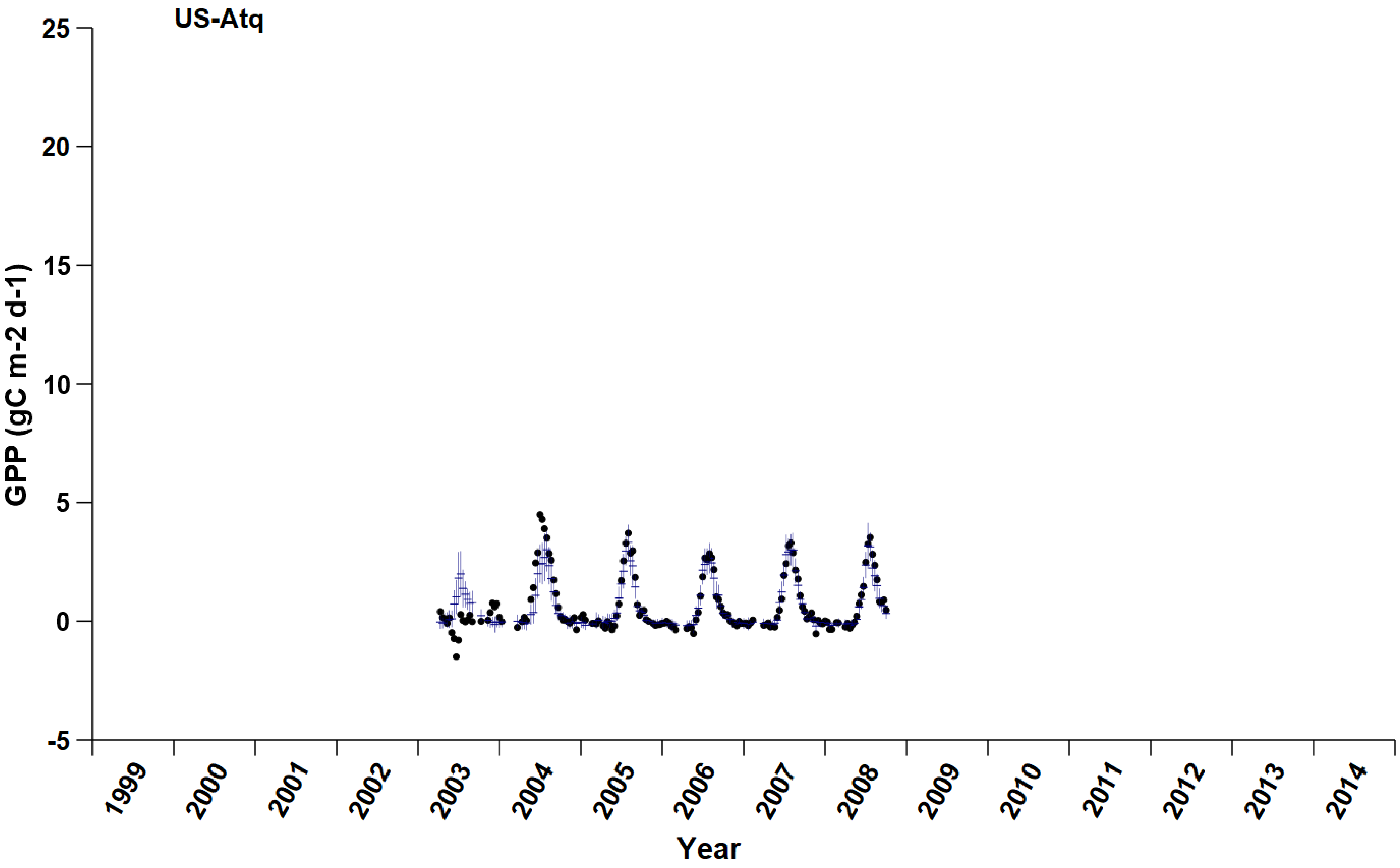

US-Atq
GPP (gC m-2 d-1)
25
20
15
10
5
0
-5
1999
2000
2001
2002
2003
2004
2005
2006
2007
2008
2009
2010
2011
2012
2013
2014
Year

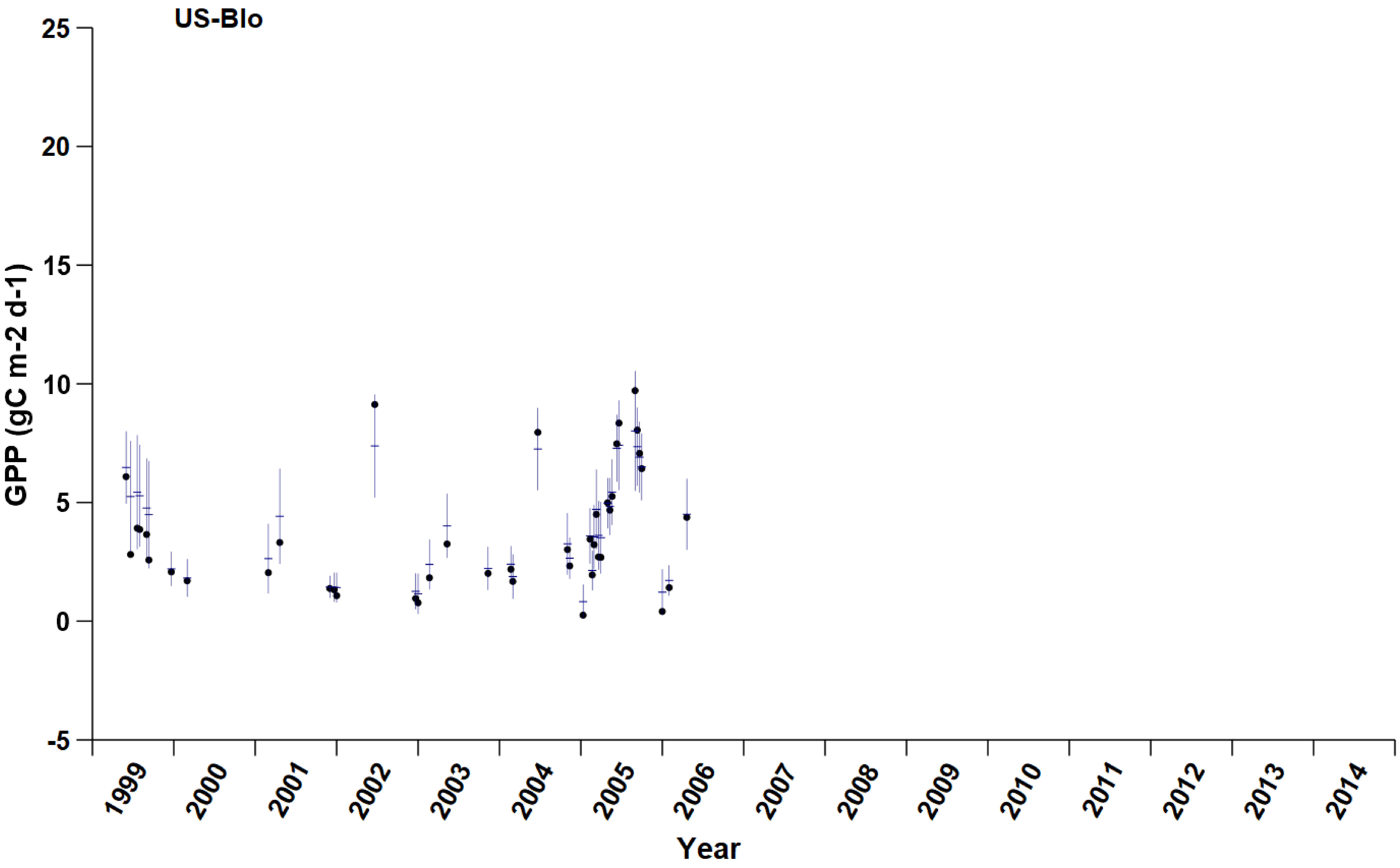

US-Blo
GPP (gC m-2 d-1)
25
20
15
10
5
0
-5
1999
2000
2001
2002
2003
2004
2005
2006
2007
2008
2009
2010
2011
2012
2013
2014
Year

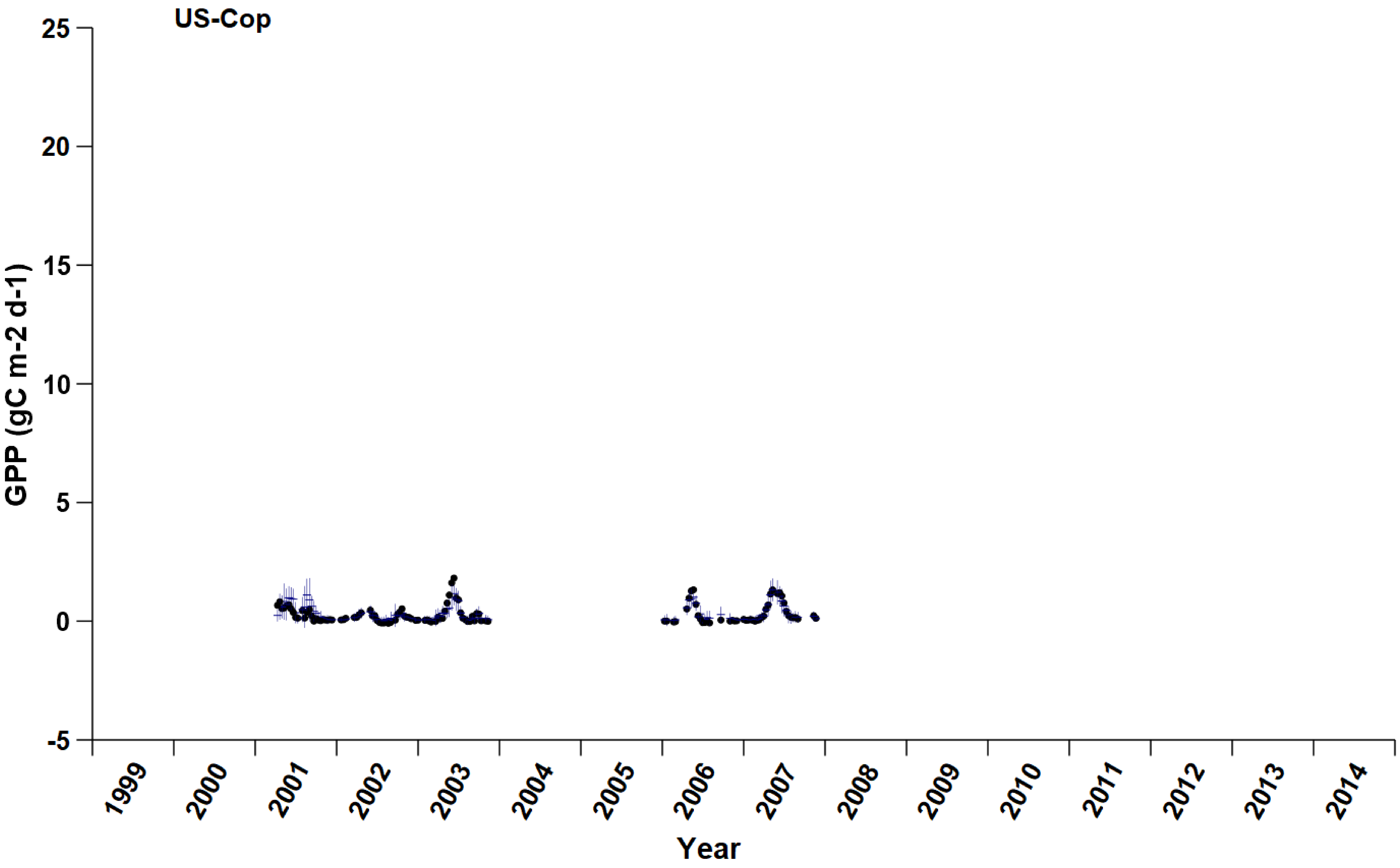
US-Cop
GPP (gC m-2 d-1)
25
20
15
10
5
0
-5
1999
2000
2001
2002
2003
2004
2005
2006
2007
2008
2009
2010
2011
2012
2013
2014
Year

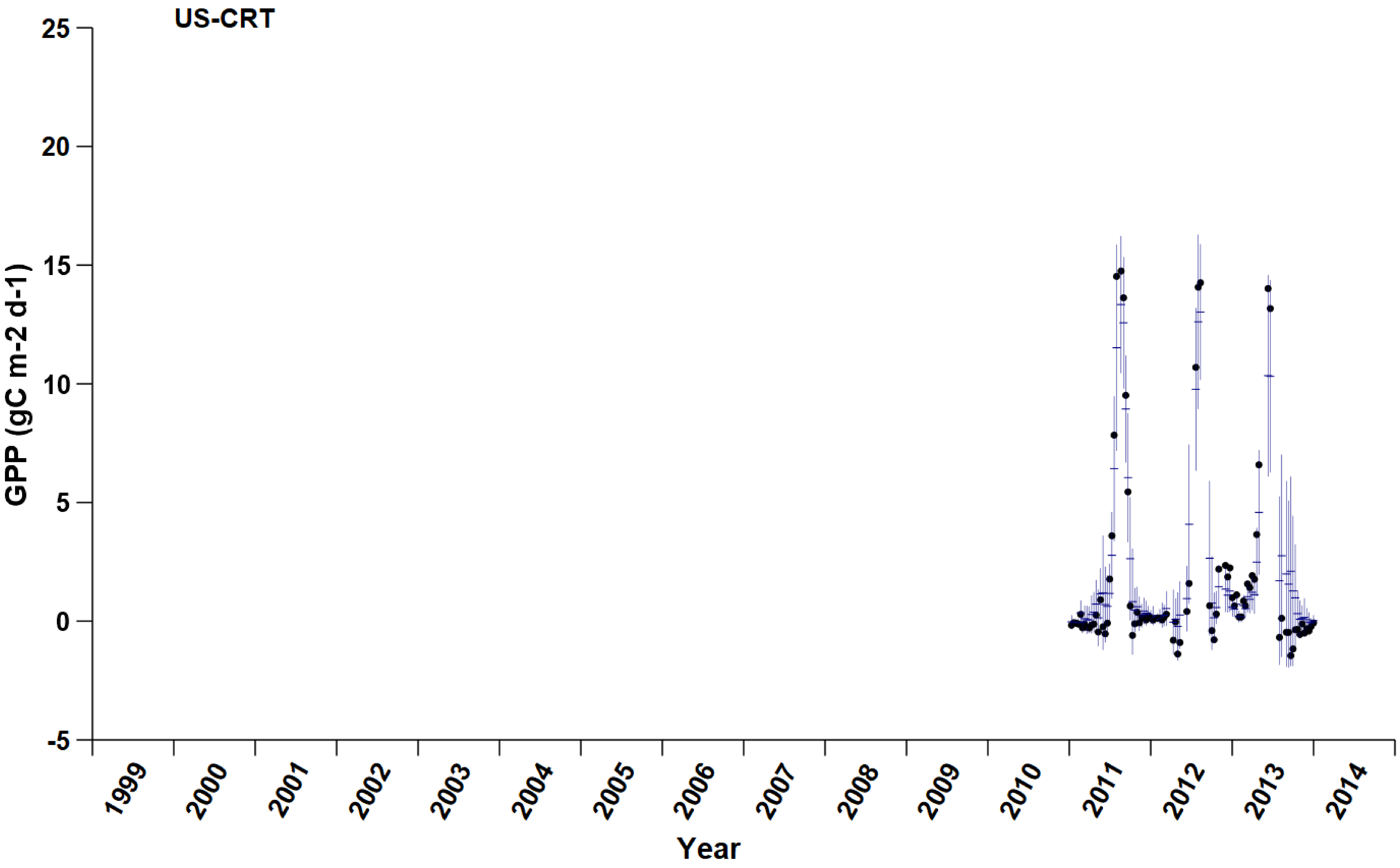

US-CRT
GPP (gC m-2 d-1)
25
20
15
10
5
0
-5
1999
2000
2001
2002
2003
2004
2005
2006
2007
2008
2009
2010
2011
2012
2013
2014
Year

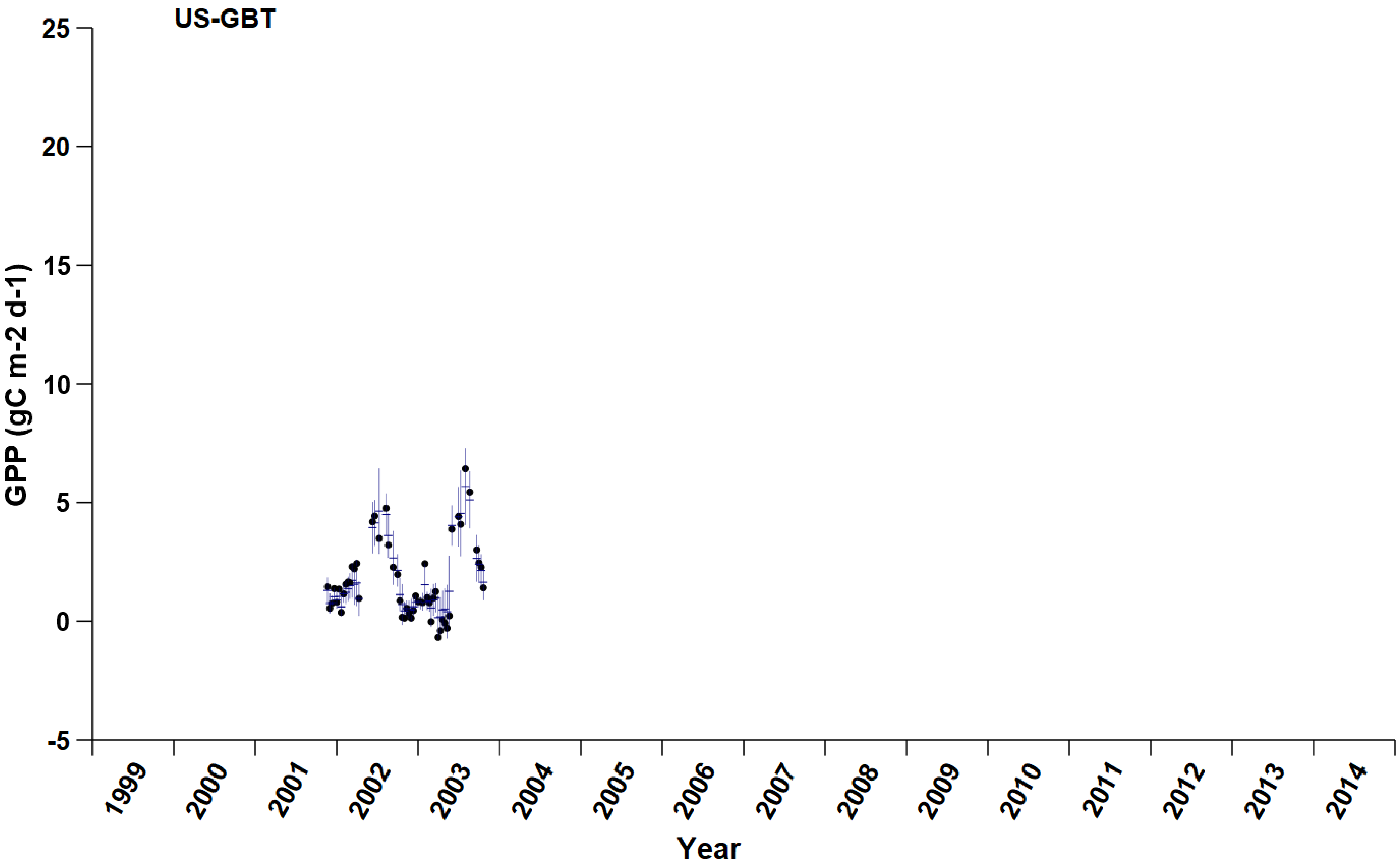

US-GBT
GPP (gC m-2 d-1)
25
20
15
10
5
0
-5
1999
2000
2001
2002
2003
2004
2005
2006
2007
2008
2009
2010
2011
2012
2013
2014
Year

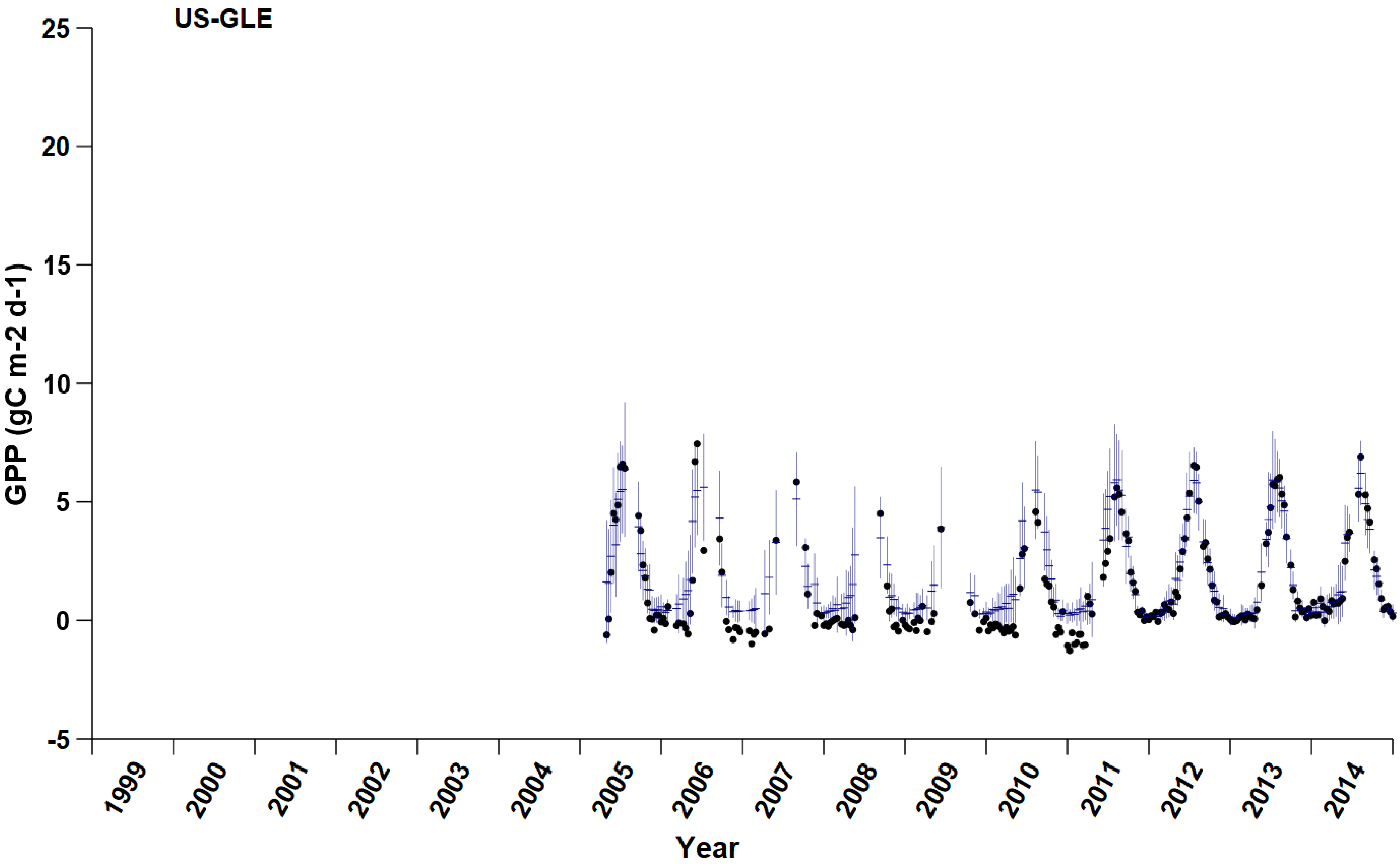

US-GLE
GPP (gC m-2 d-1)
25
20
15
10
5
0
-5
1999
2000
2001
2002
2003
2004
2005
2006
2007
2008
2009
2010
2011
2012
2013
2014
Year

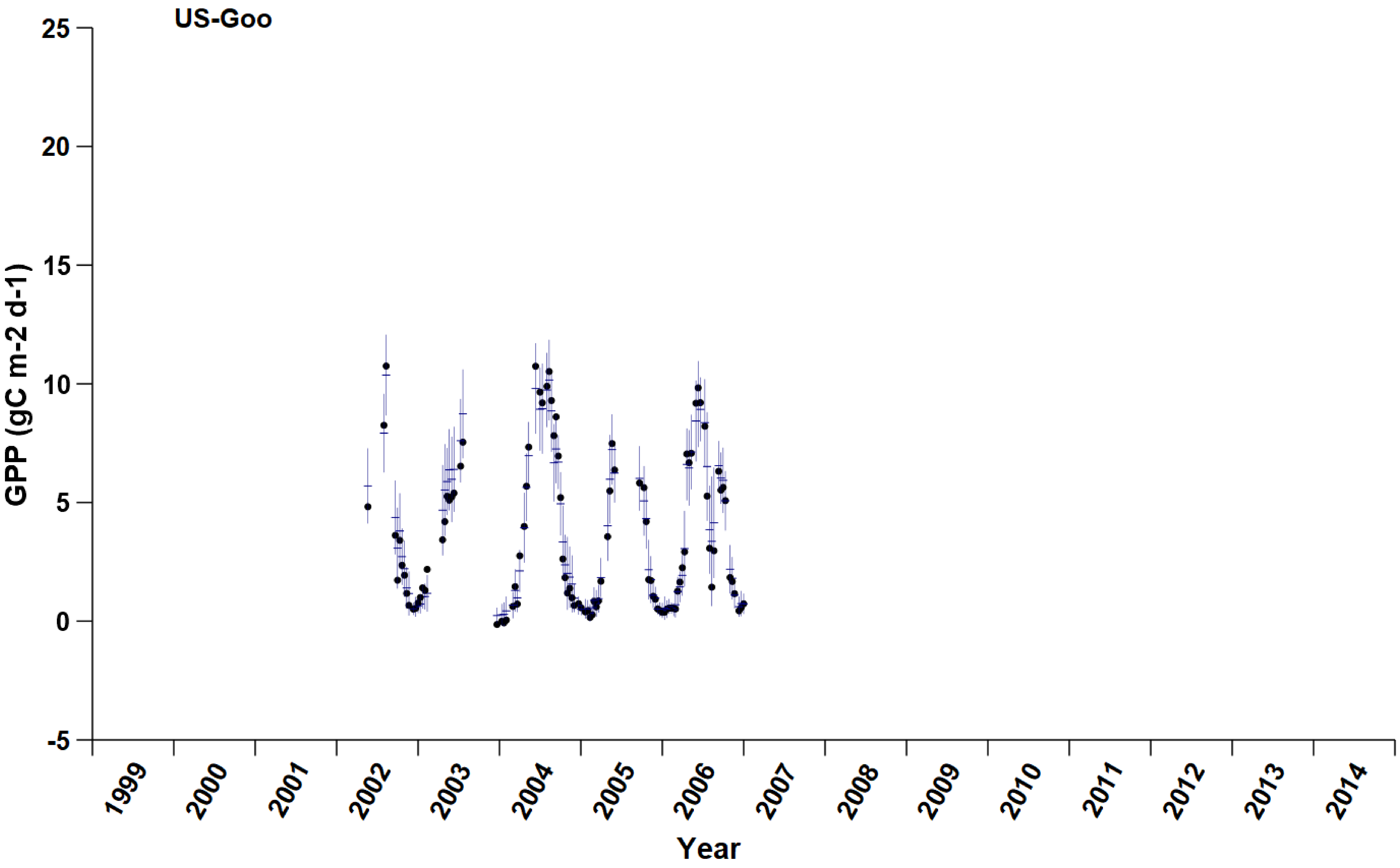
US-Goo
25
20
15
10
5
0
-5
GPP (gC m-2 d-1)
1999
2000
2001
2002
2003
2004
2005
2006
2007
2008
2009
2010
2011
2012
2013
2014
Year

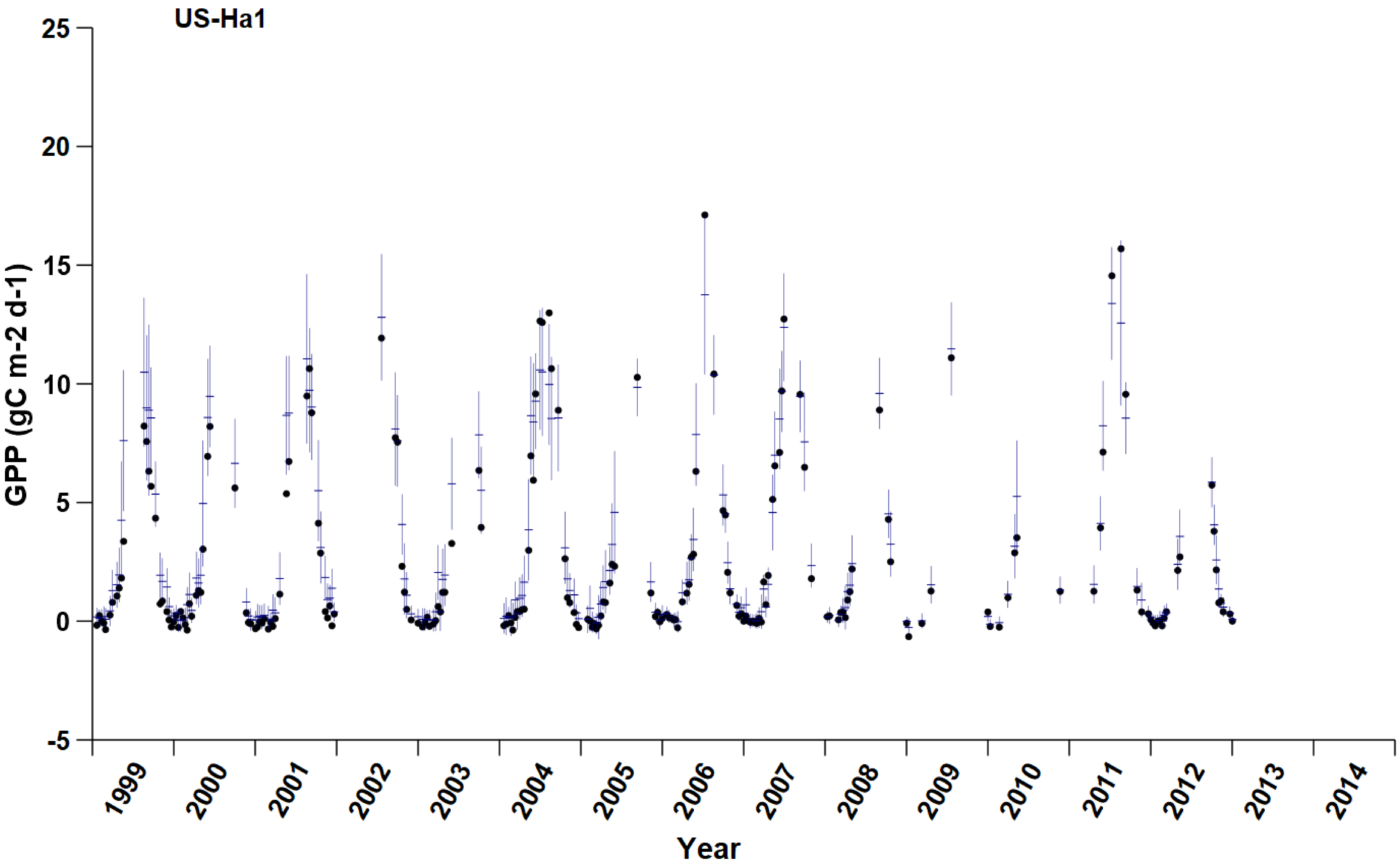
US-Ha1
GPP (gC m-2 d-1)
25
20
15
10
5
0
-5
1999
2000
2001
2002
2003
2004
2005
2006
2007
2008
2009
2010
2011
2012
2013
2014
Year

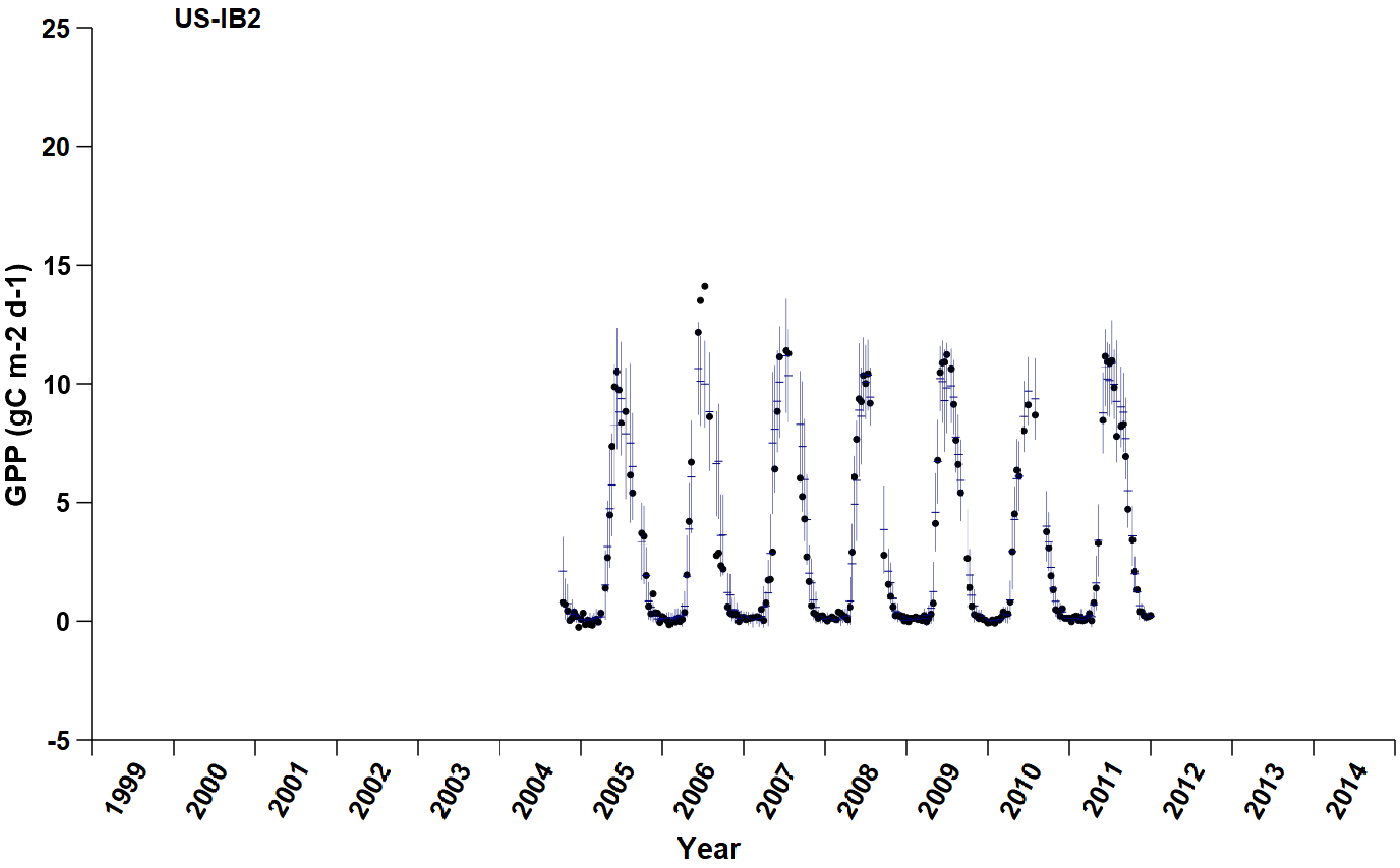

US-IB2
GPP (gC m-2 d-1)
25
20
15
10
5
0
-5
1999
2000
2001
2002
2003
2004
2005
2006
2007
2008
2009
2010
2011
2012
2013
2014
Year

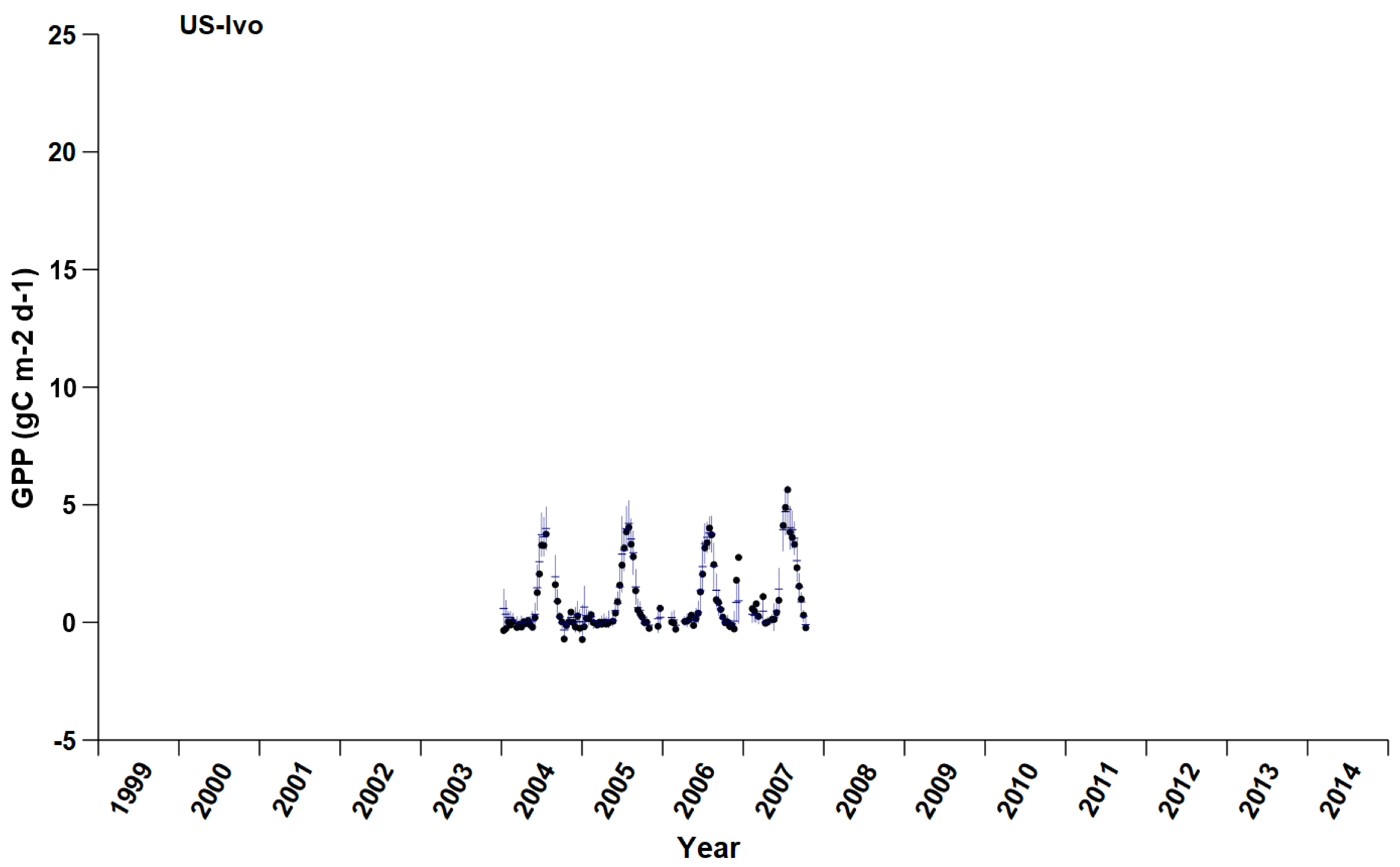

US-Ivo
GPP (gC m-2 d-1)
25
20
15
10
5
0
-5
1999
2000
2001
2002
2003
2004
2005
2006
2007
2008
2009
2010
2011
2012
2013
2014
Year

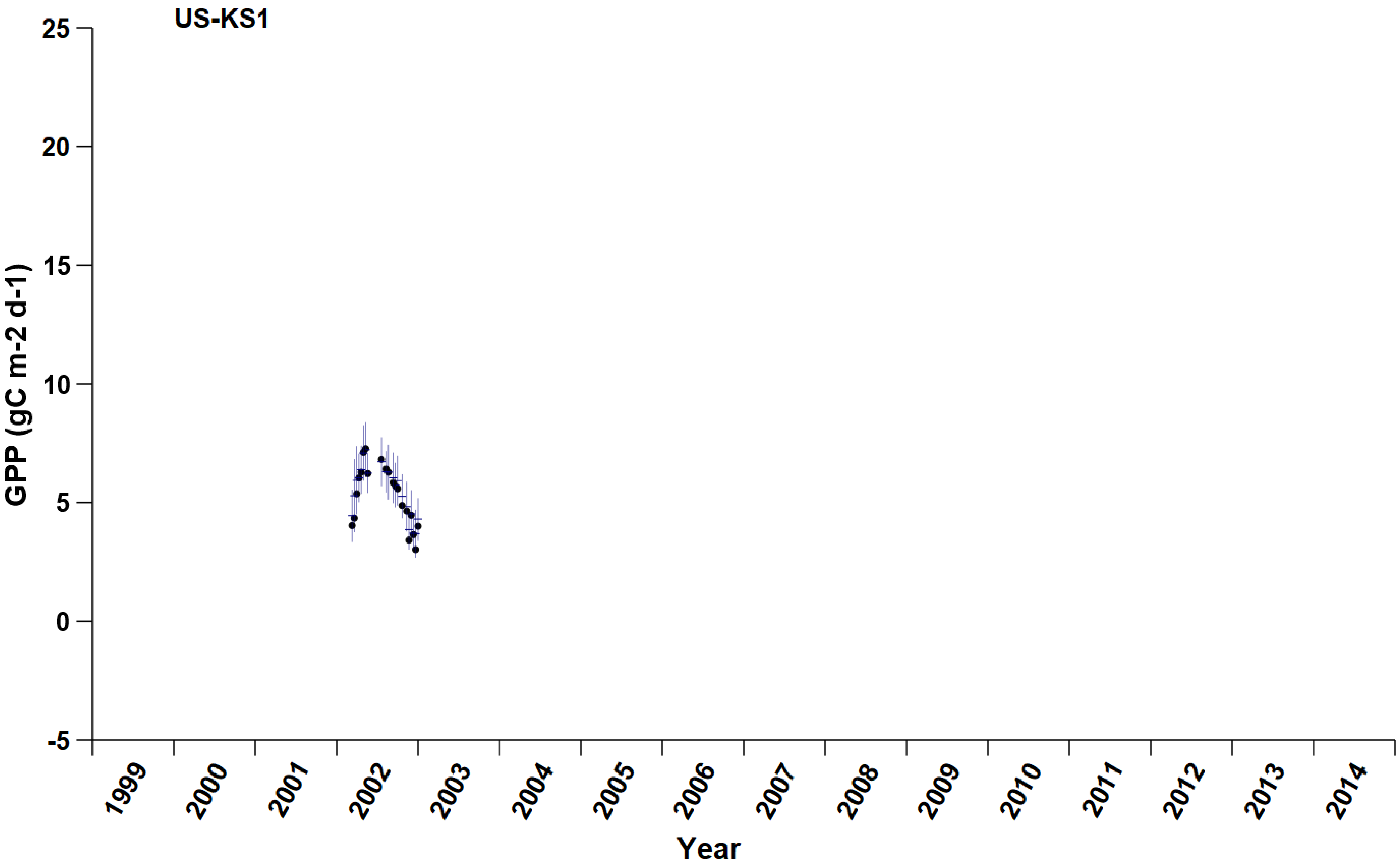
US-KS1
GPP (gC m-2 d-1)
25
20
15
10
5
0
-5
1999
2000
2001
2002
2003
2004
2005
2006
2007
2008
2009
2010
2011
2012
2013
2014
Year

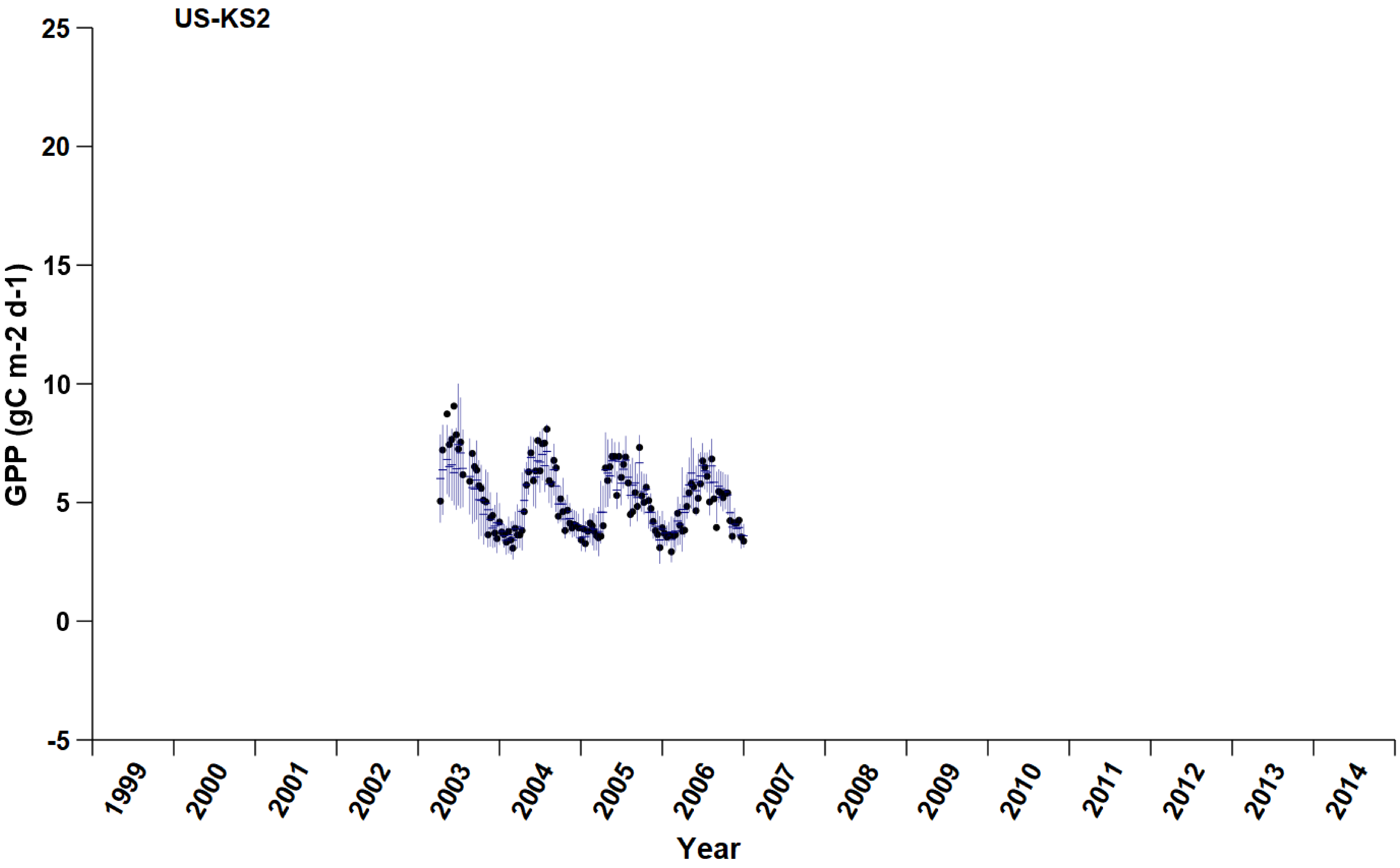

US-KS2
GPP (gC m-2 d-1)
25
20
15
10
5
0
-5
1999
2000
2001
2002
2003
2004
2005
2006
2007
2008
2009
2010
2011
2012
2013
2014
Year

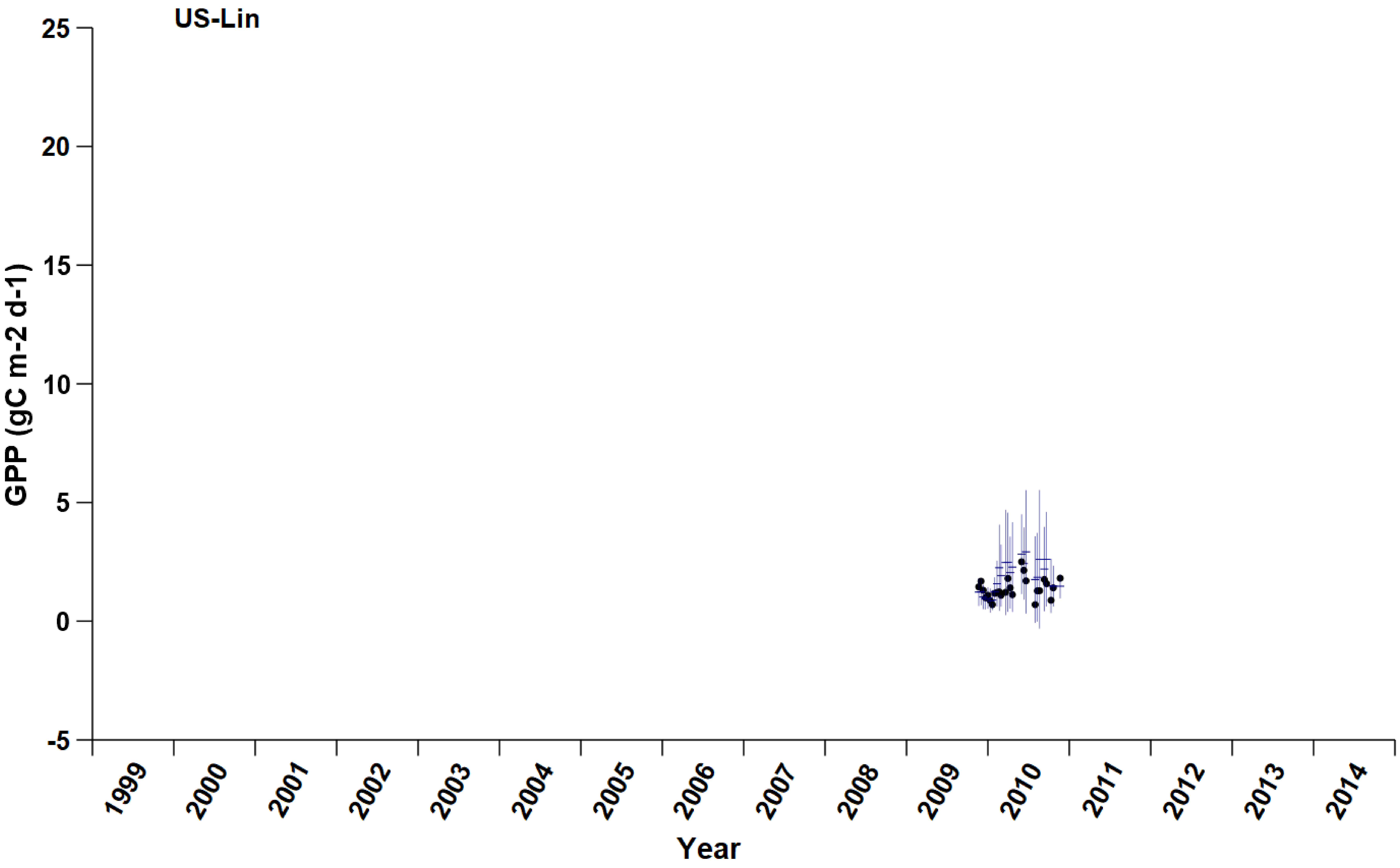

US-Lin
GPP (gC m-2 d-1)
25
20
15
10
5
0
-5
1999
2000
2001
2002
2003
2004
2005
2006
2007
2008
2009
2010
2011
2012
2013
2014
Year

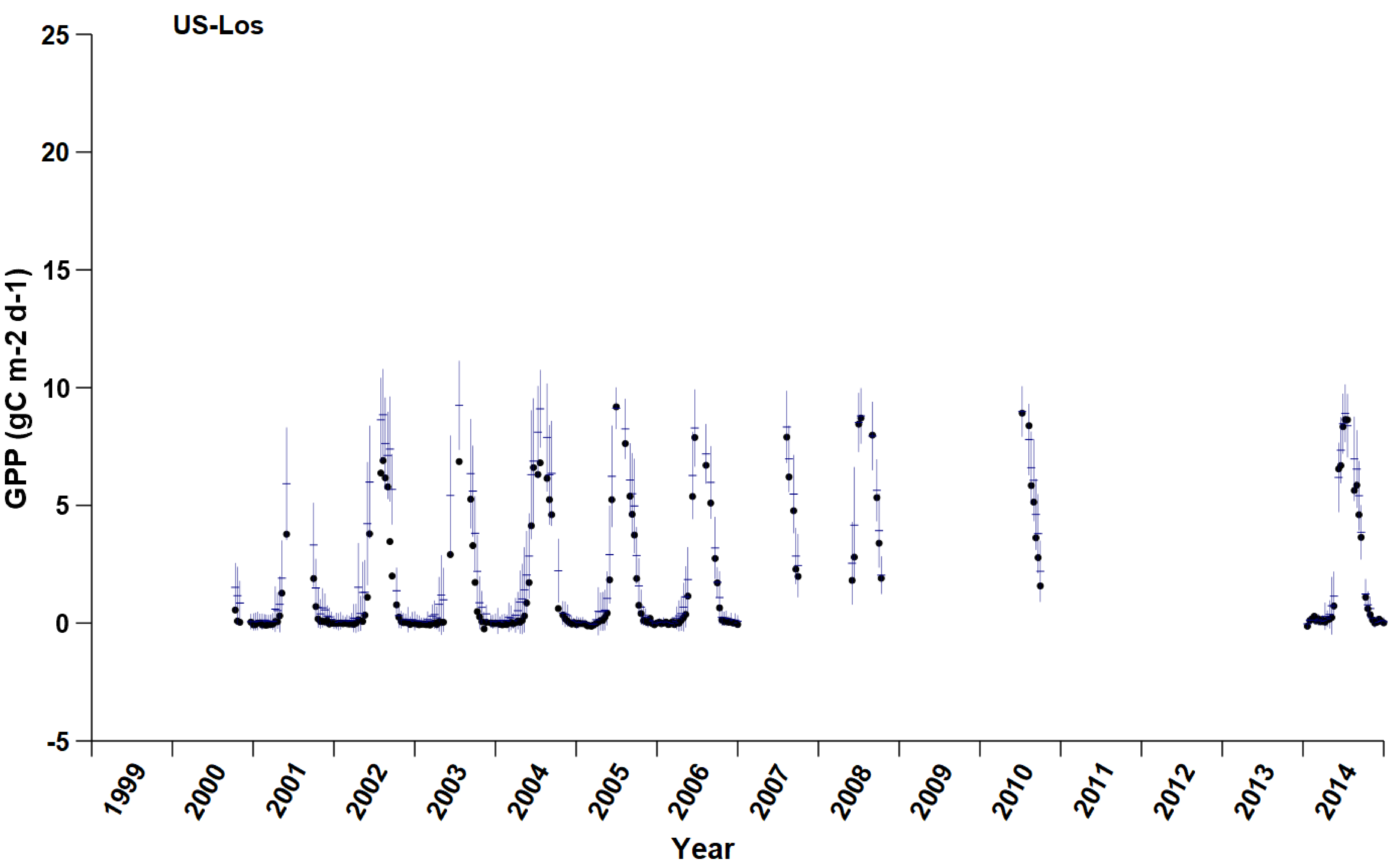

US-Los
GPP (gC m-2 d-1)
25
20
15
10
5
0
-5
1999
2000
2001
2002
2003
2004
2005
2006
2007
2008
2009
2010
2011
2012
2013
2014
Year

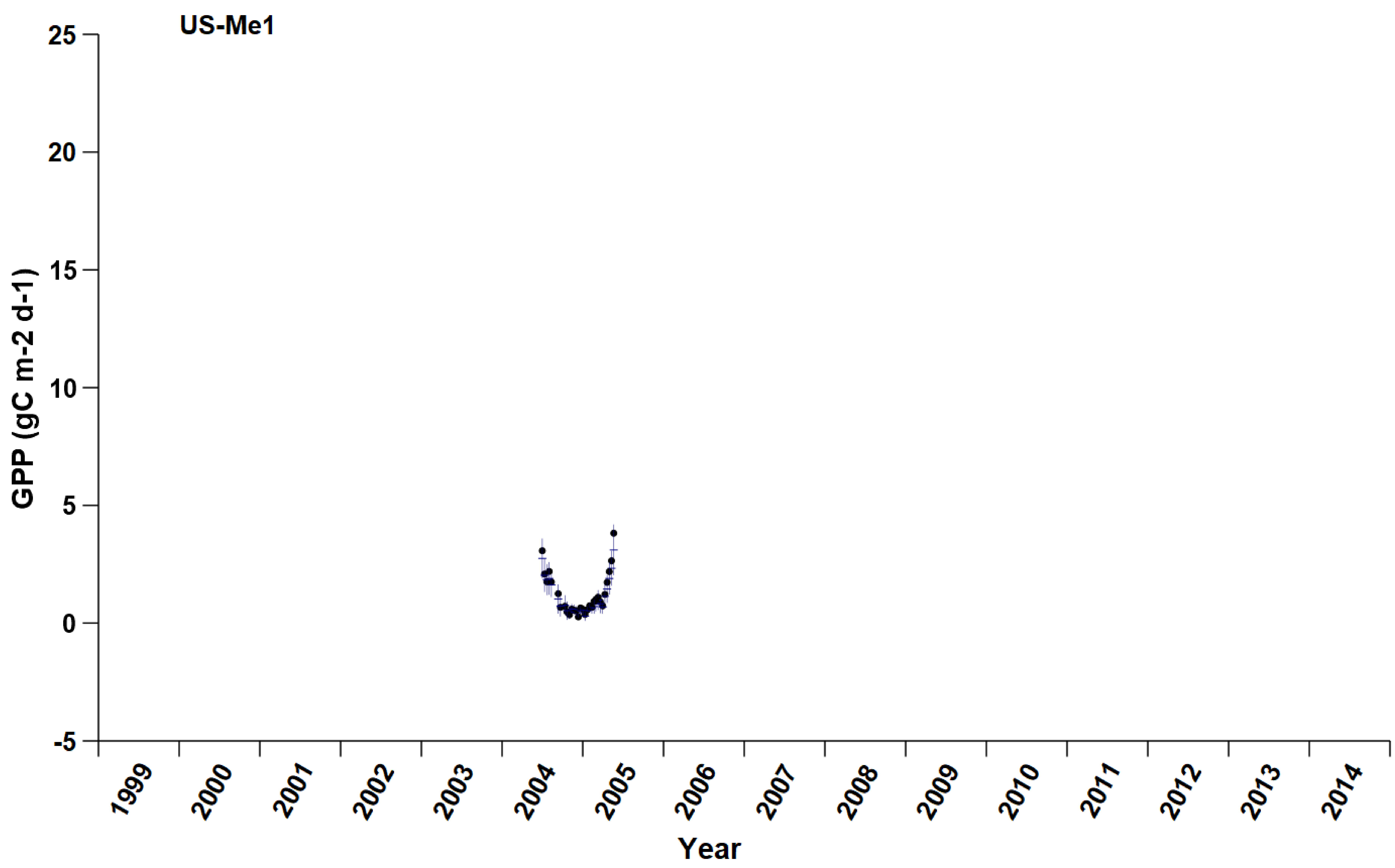
US-Me1
GPP (gC m-2 d-1)
Year
25
20
15
10
5
0
-5
1999
2000
2001
2002
2003
2004
2005
2006
2007
2008
2009
2010
2011
2012
2013
2014

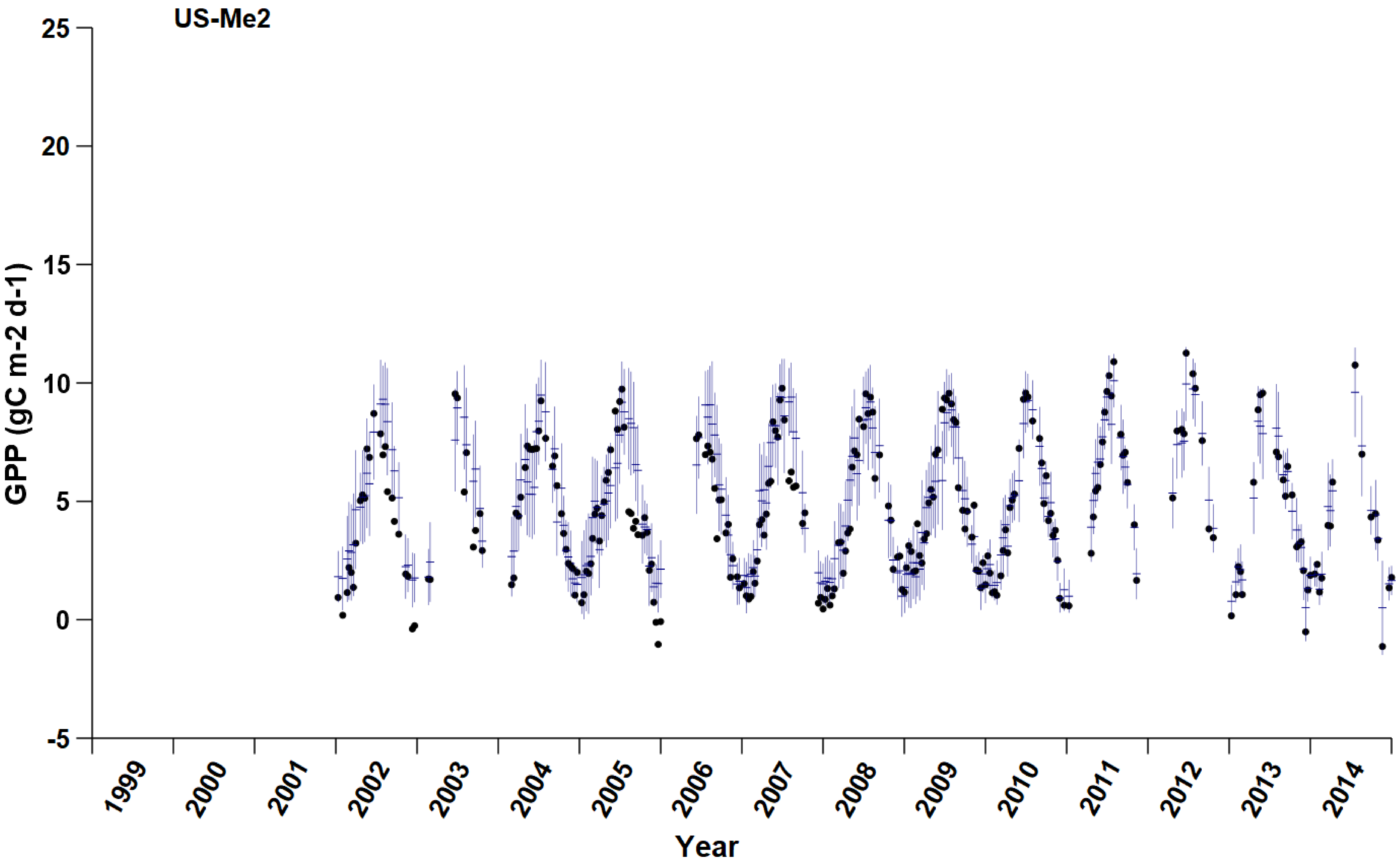

US-Me2
GPP (gC m-2 d-1)
25
20
15
10
5
0
-5
1999
2000
2001
2002
2003
2004
2005
2006
2007
2008
2009
2010
2011
2012
2013
2014
Year

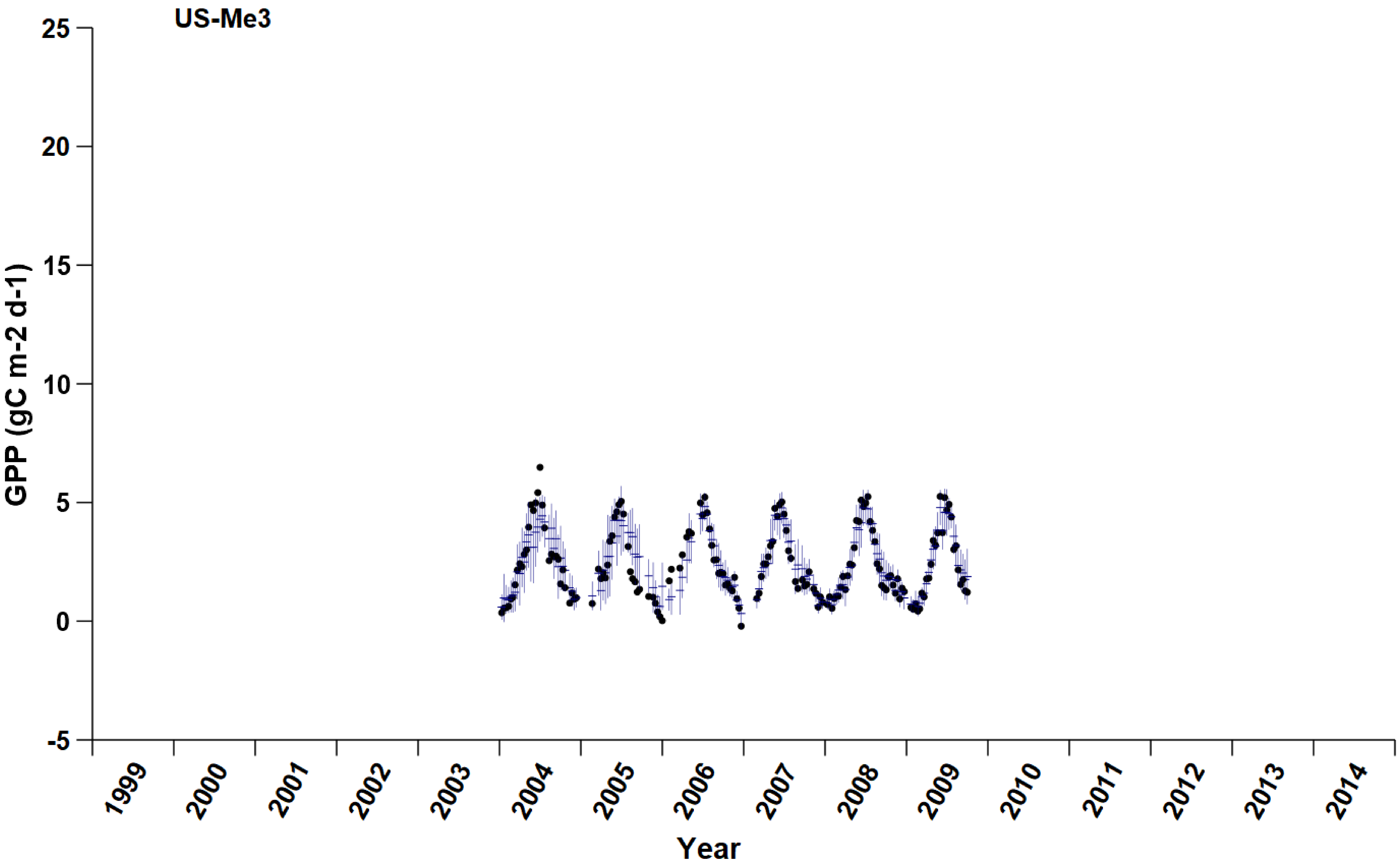

US-Me3
GPP (gC m-2 d-1)
25
20
15
10
5
0
-5
1999
2000
2001
2002
2003
2004
2005
2006
2007
2008
2009
2010
2011
2012
2013
2014
Year

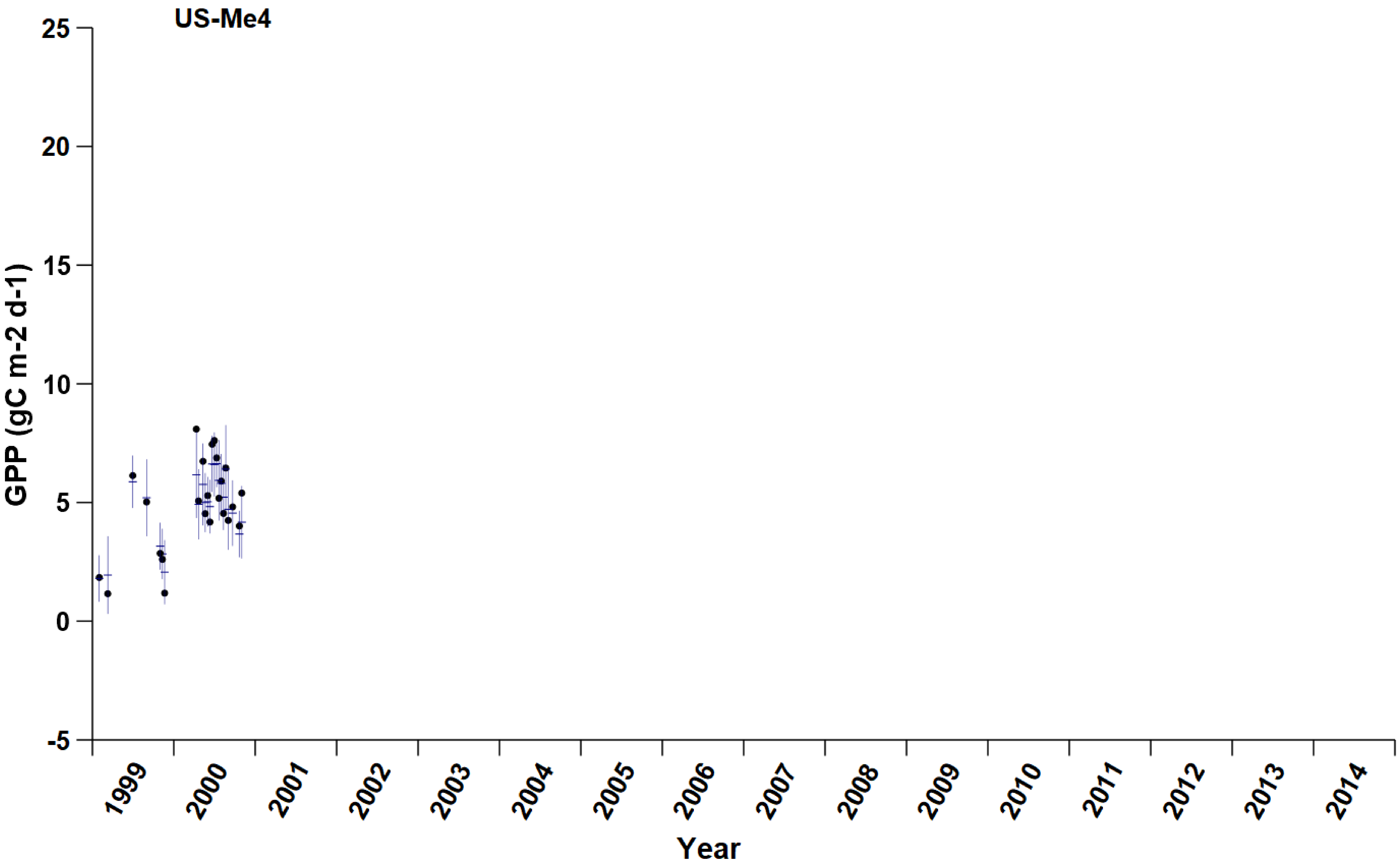
US-Me4
GPP (gC m-2 d-1)
25
20
15
10
5
0
-5
1999
2000
2001
2002
2003
2004
2005
2006
2007
2008
2009
2010
2011
2012
2013
2014
Year

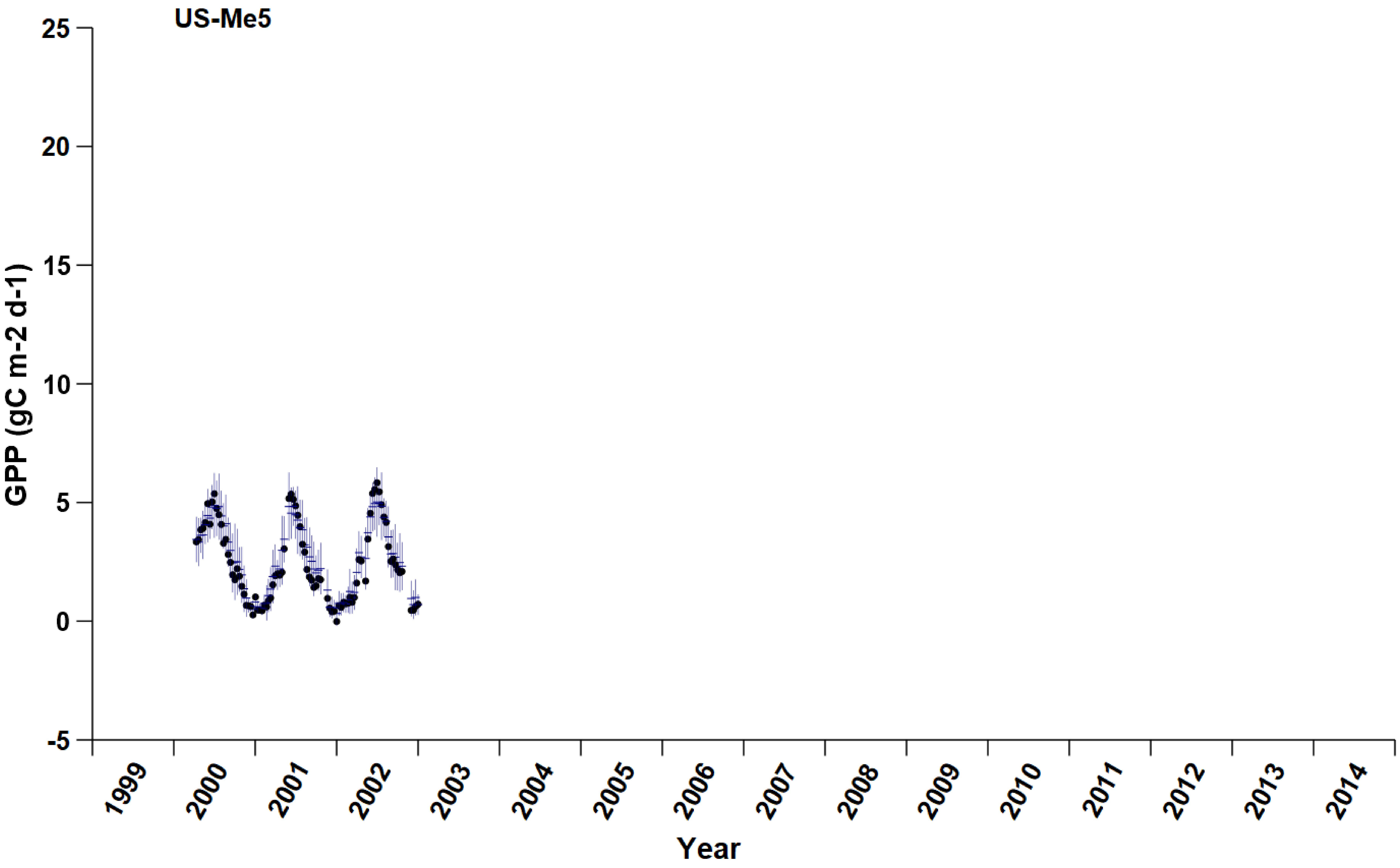

US-Me5
GPP (gC m-2 d-1)
25
20
15
10
5
0
-5
1999
2000
2001
2002
2003
2004
2005
2006
2007
2008
2009
2010
2011
2012
2013
2014
Year

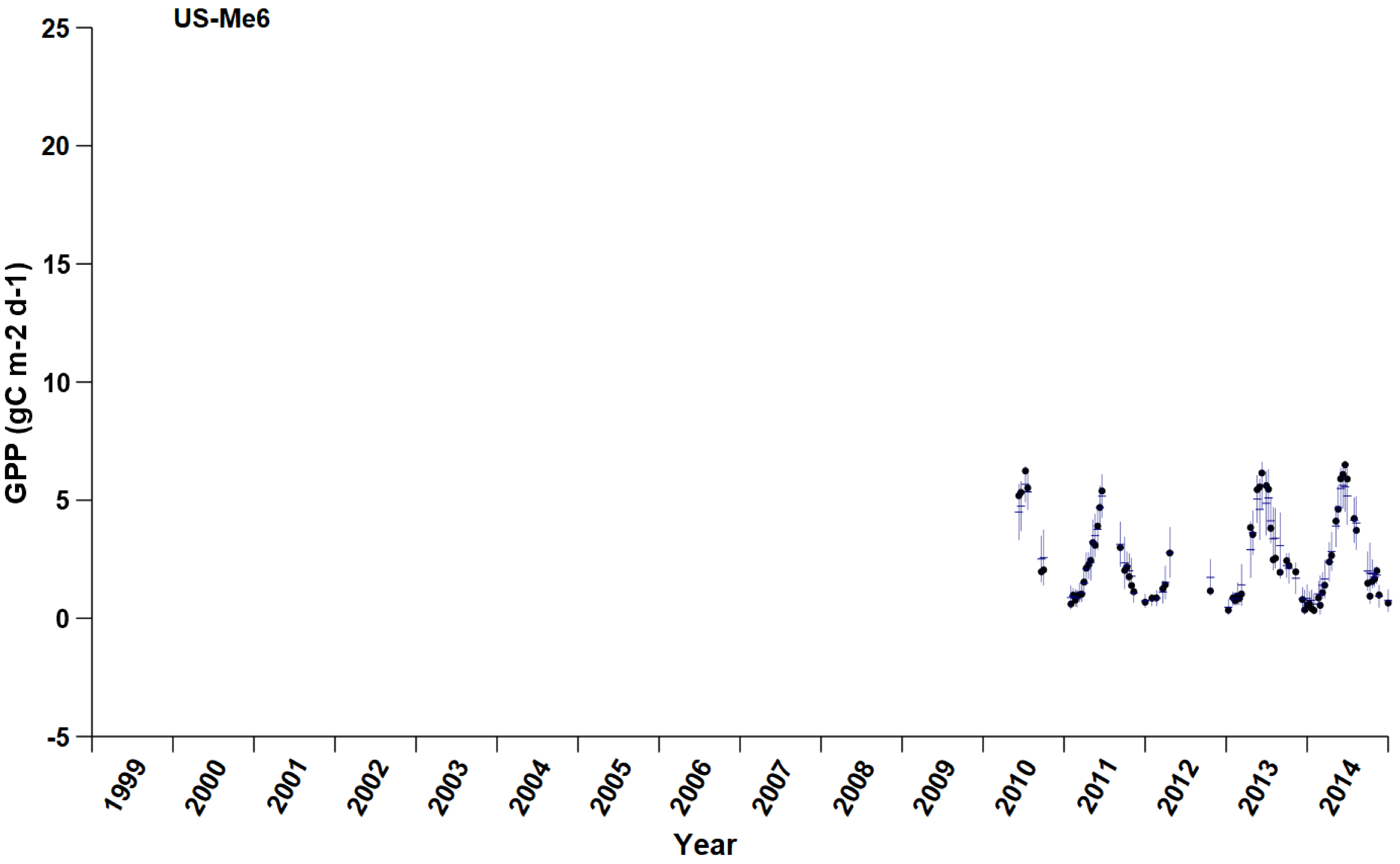

US-Me6
GPP (gC m-2 d-1)
25
20
15
10
5
0
-5
1999
2000
2001
2002
2003
2004
2005
2006
2007
2008
2009
2010
2011
2012
2013
2014
Year

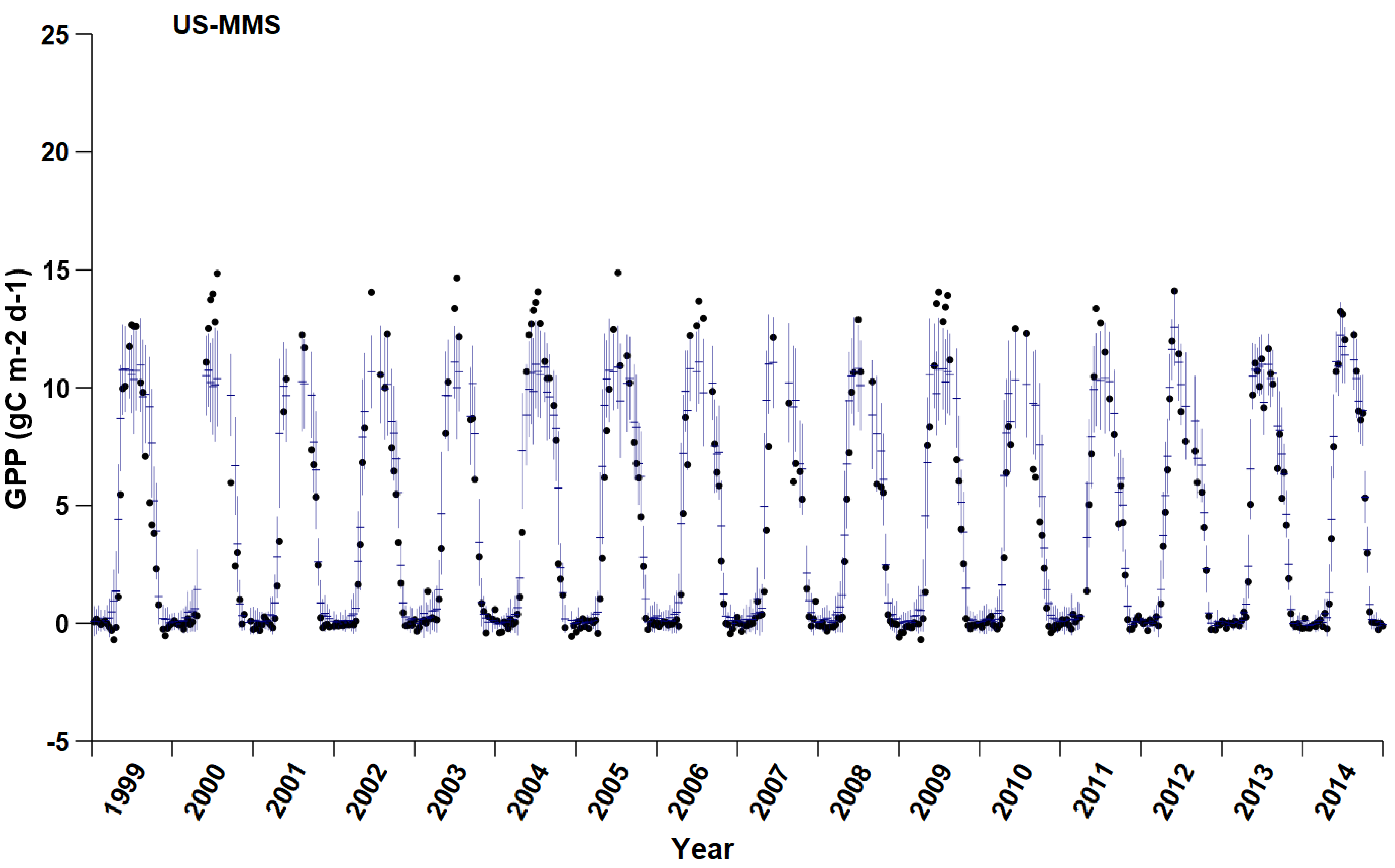
US-MMS
GPP (gC m-2 d-1)
25
20
15
10
5
0
-5
1999
2000
2001
2002
2003
2004
2005
2006
2007
2008
2009
2010
2011
2012
2013
2014
Year

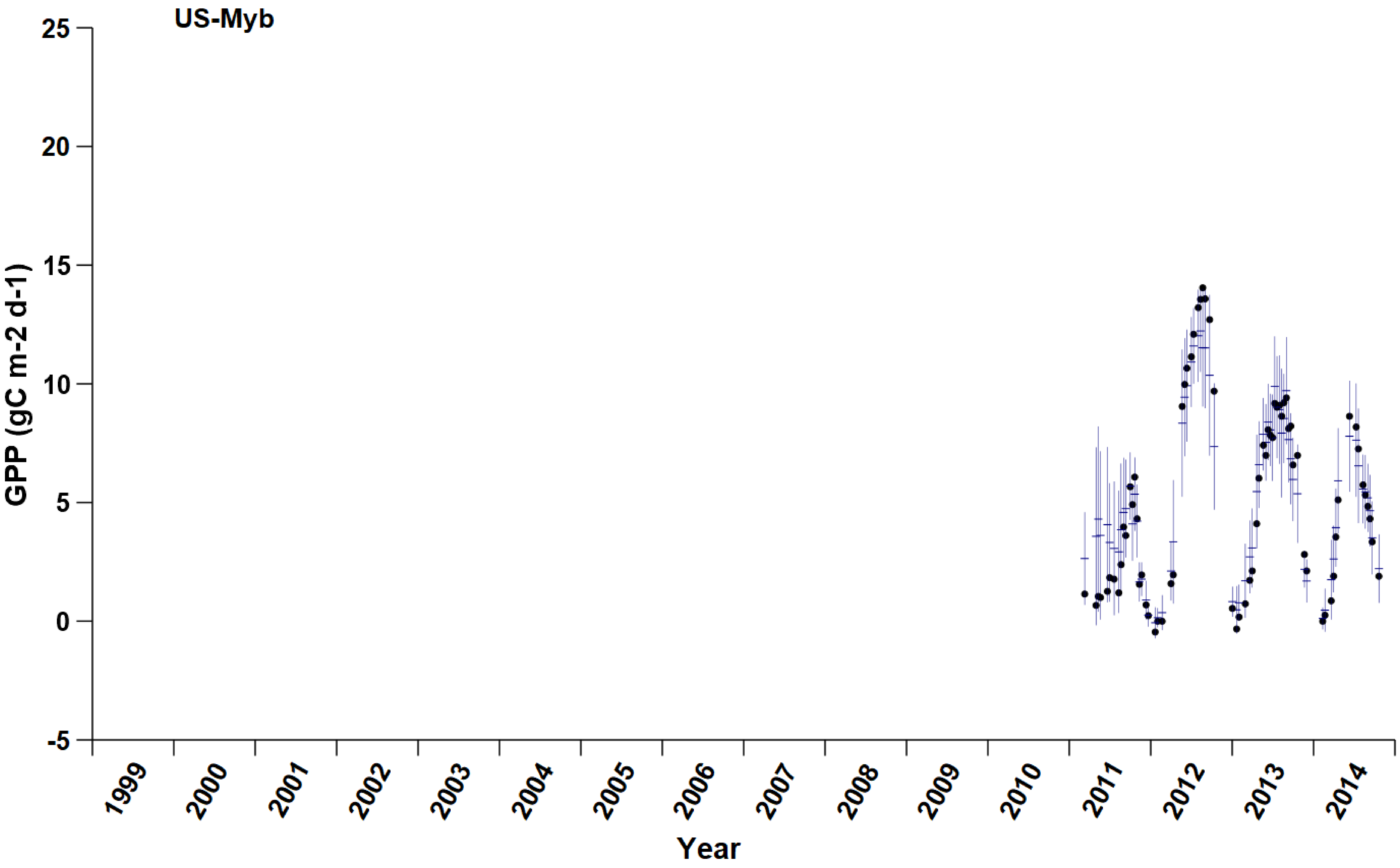

US-Myb
GPP (gC m-2 d-1)
25
20
15
10
5
0
-5
1999
2000
2001
2002
2003
2004
2005
2006
2007
2008
2009
2010
2011
2012
2013
2014
Year

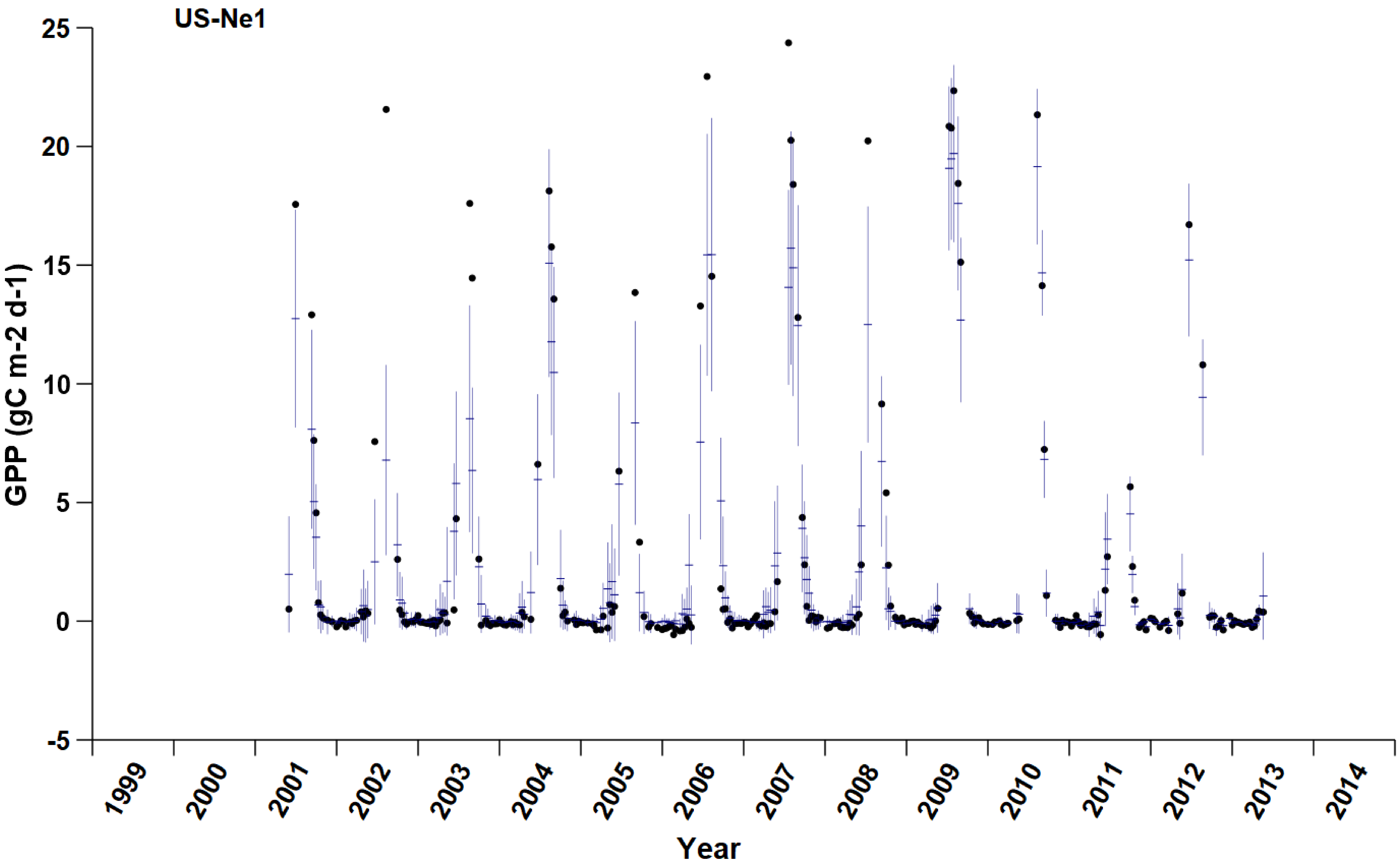

US-Ne1
GPP (gC m-2 d-1)
25
20
15
10
5
0
-5
1999
2000
2001
2002
2003
2004
2005
2006
2007
2008
2009
2010
2011
2012
2013
2014
Year

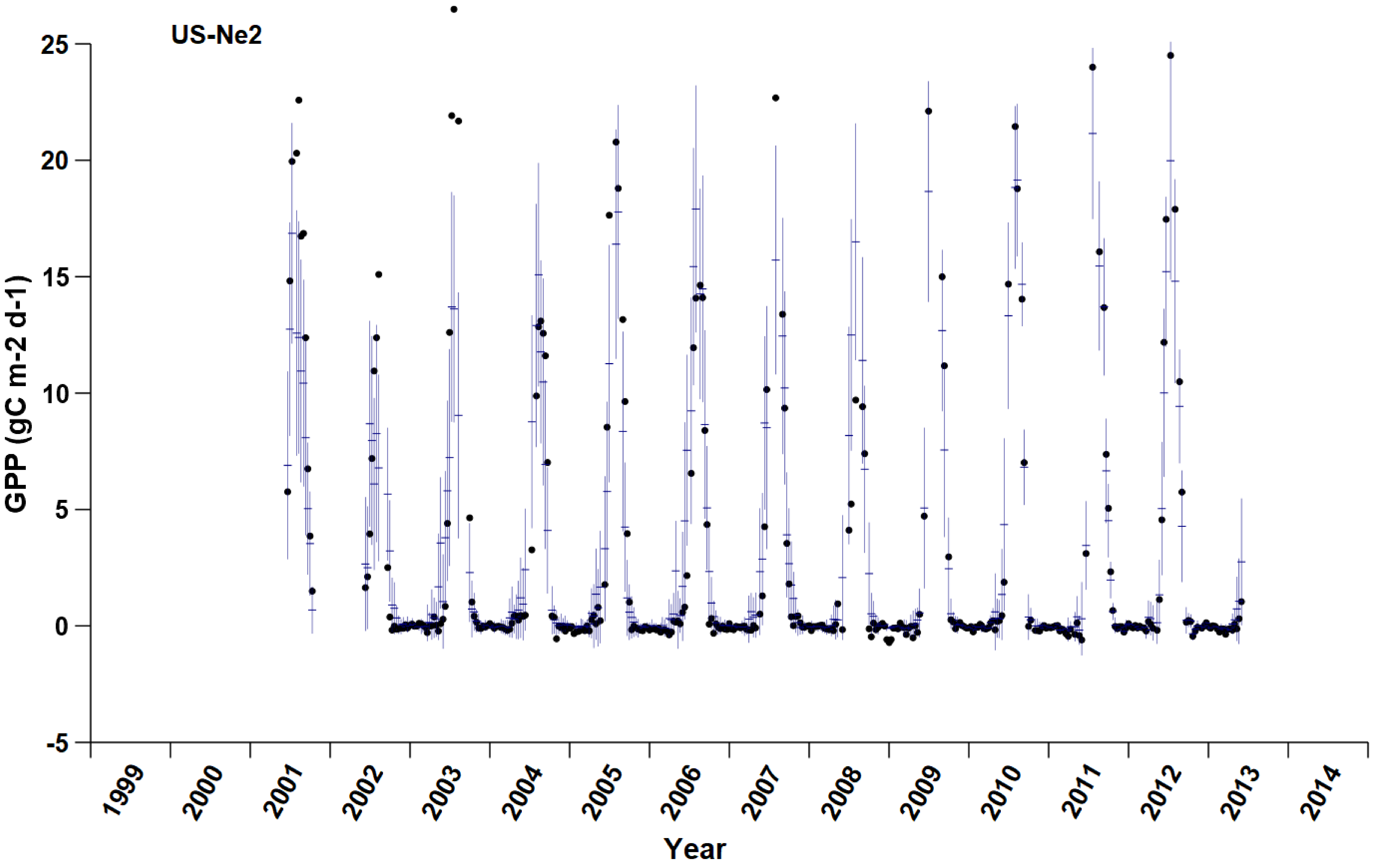
US-Ne2
GPP (gC m-2 d-1)
25
20
15
10
5
0
-5
1999
2000
2001
2002
2003
2004
2005
2006
2007
2008
2009
2010
2011
2012
2013
2014
Year

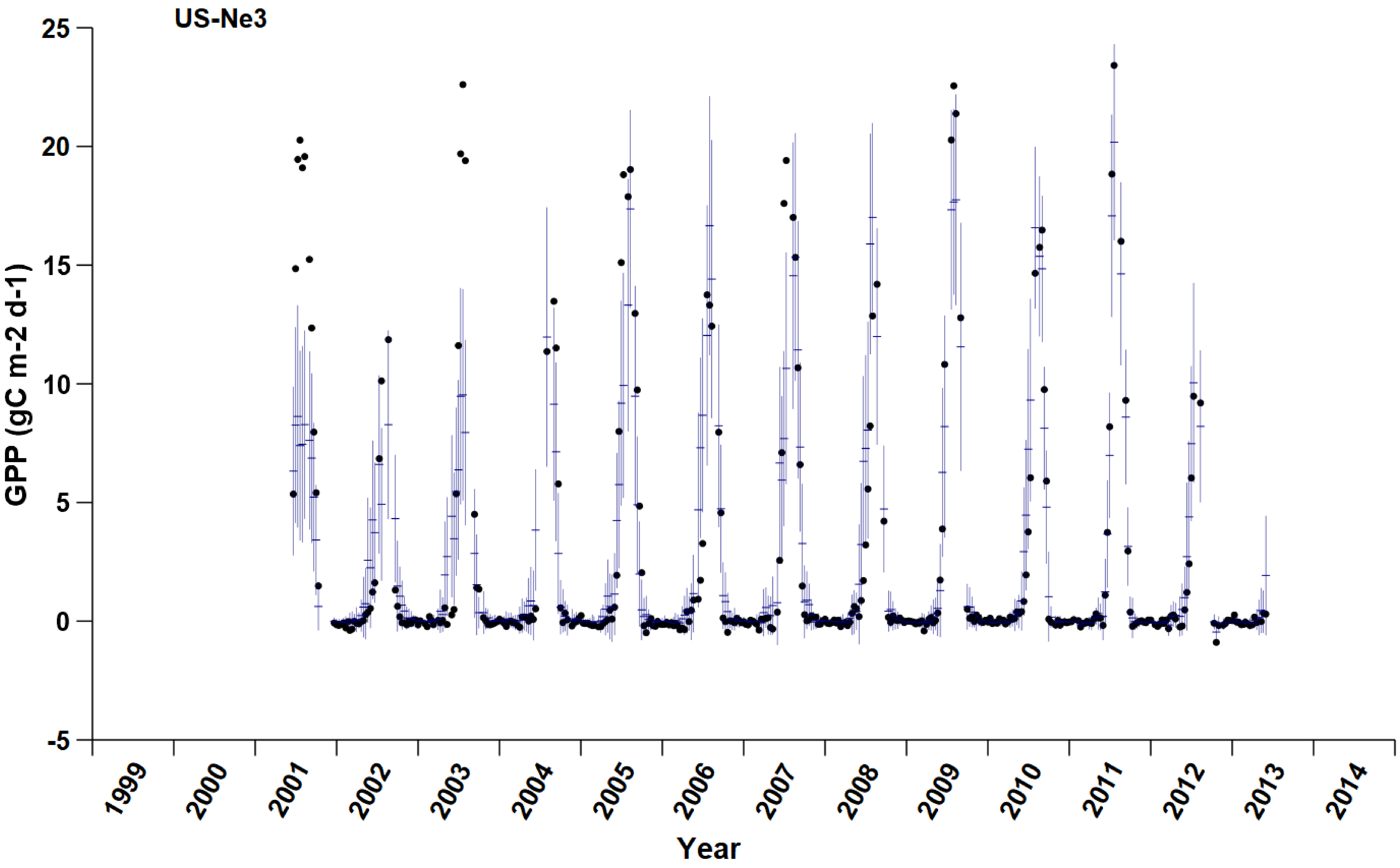

US-Ne3
GPP (gC m-2 d-1)
25
20
15
10
5
0
-5
1999
2000
2001
2002
2003
2004
2005
2006
2007
2008
2009
2010
2011
2012
2013
2014
Year

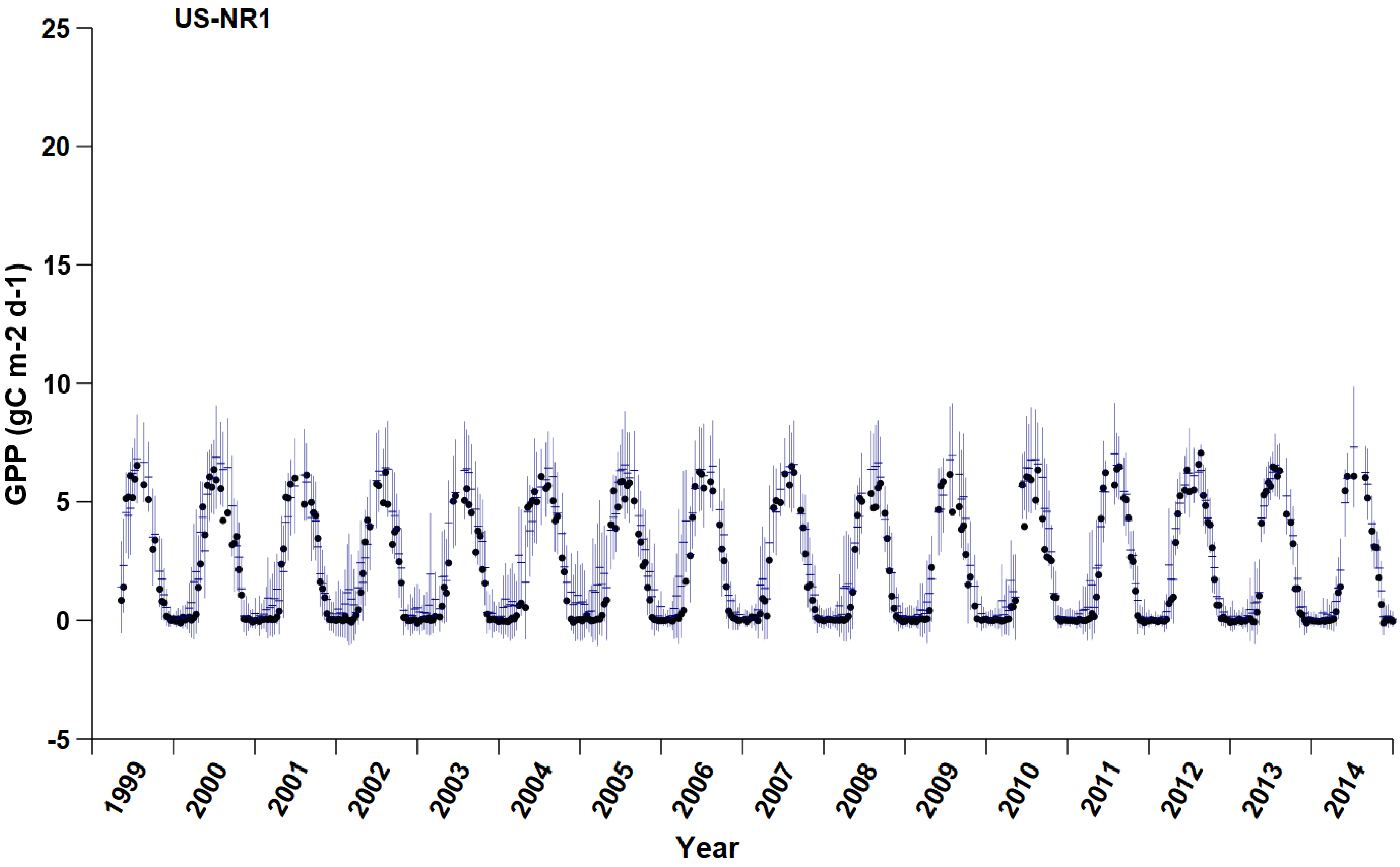

US-NR1
GPP (gC m-2 d-1)
25
20
15
10
5
0
-5
1999
2000
2001
2002
2003
2004
2005
2006
2007
2008
2009
2010
2011
2012
2013
2014
Year

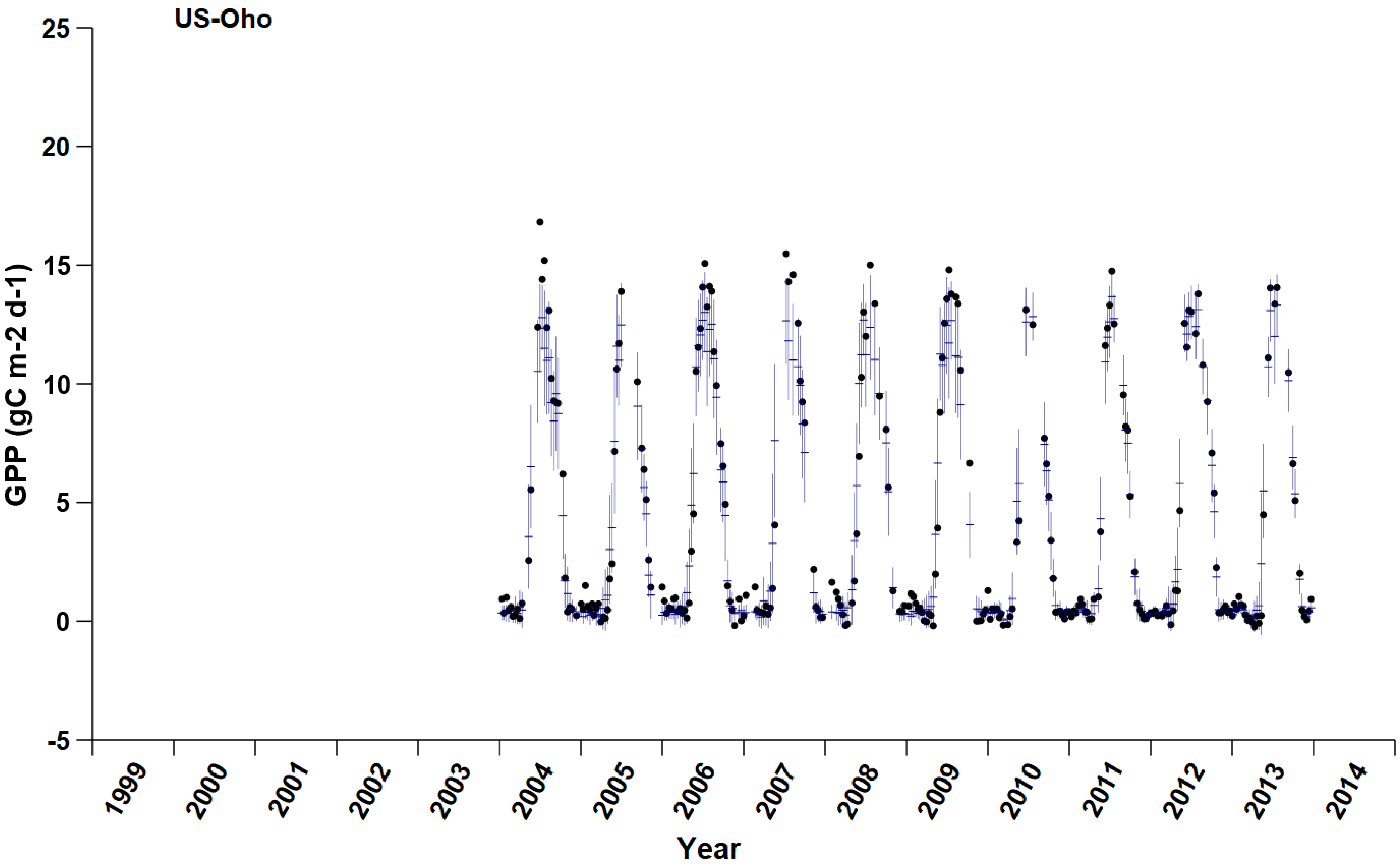
US-Oho
GPP (gC m-2 d-1)
25
20
15
10
5
0
-5
1999
2000
2001
2002
2003
2004
2005
2006
2007
2008
2009
2010
2011
2012
2013
2014
Year

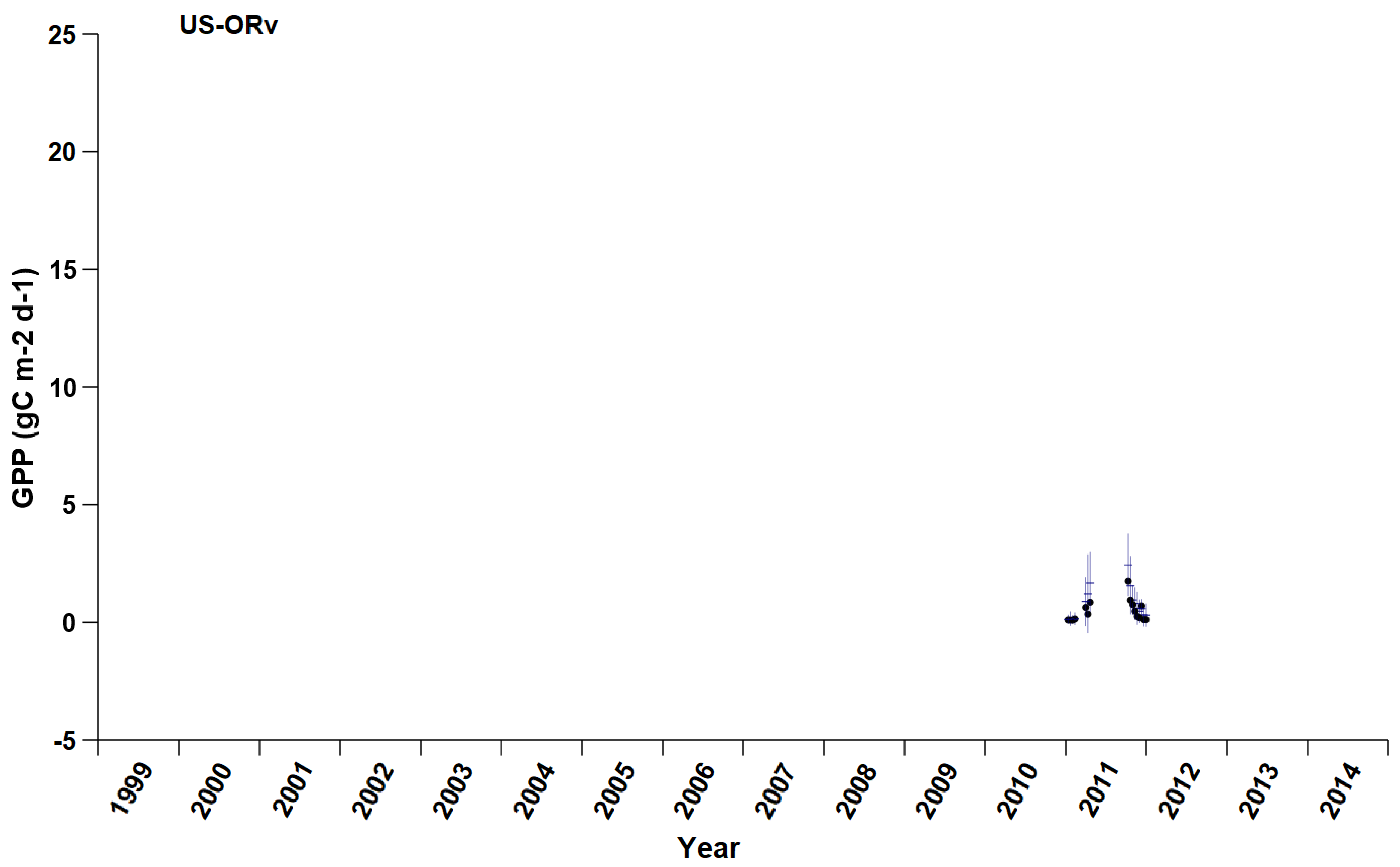
US-ORv
GPP (gC m-2 d-1)
25
20
15
10
5
0
-5
1999
2000
2001
2002
2003
2004
2005
2006
2007
2008
2009
2010
2011
2012
2013
2014
Year

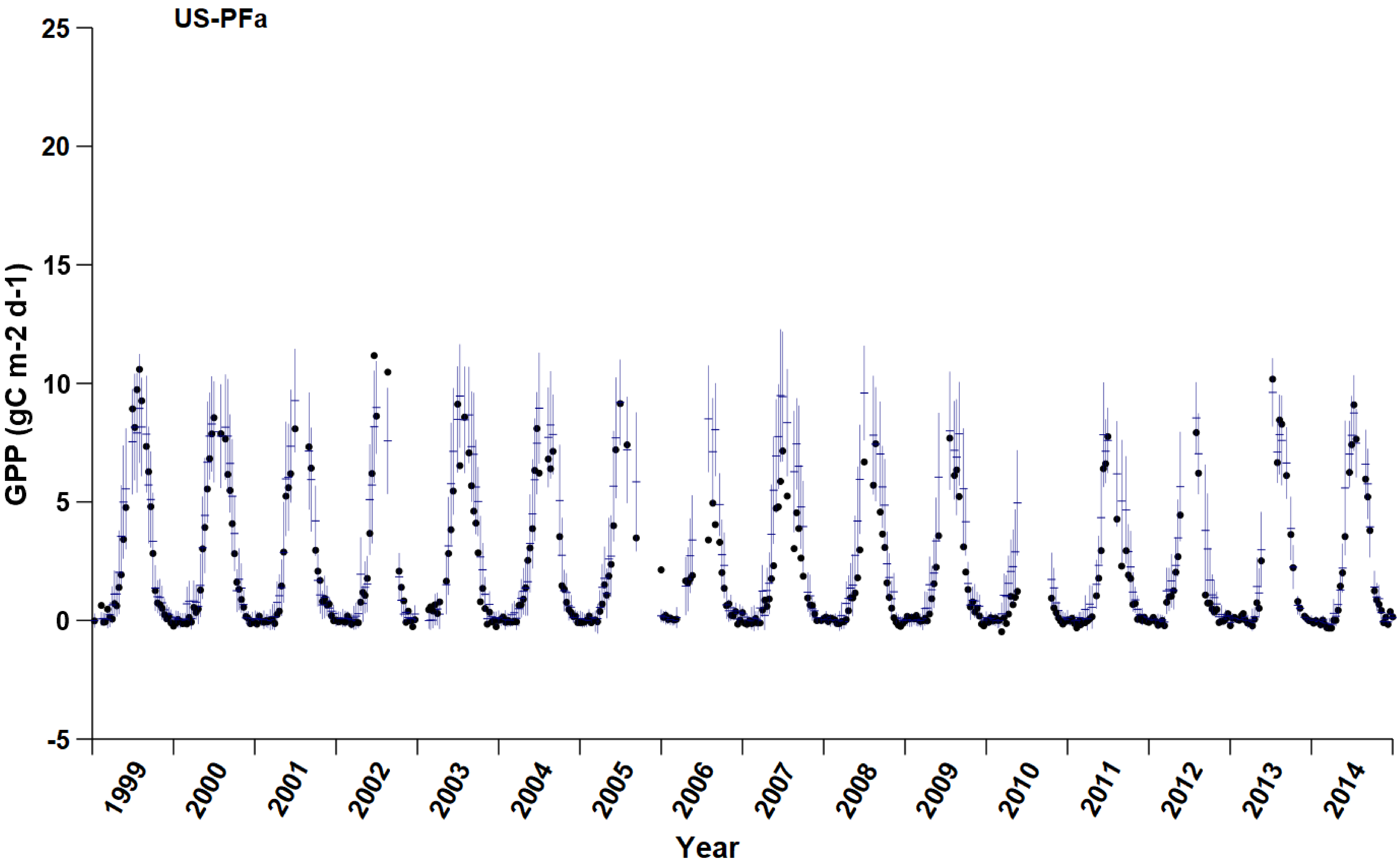
US-PFa
GPP (gC m-2 d-1)
25
20
15
10
5
0
-5
1999
2000
2001
2002
2003
2004
2005
2006
2007
2008
2009
2010
2011
2012
2013
2014
Year

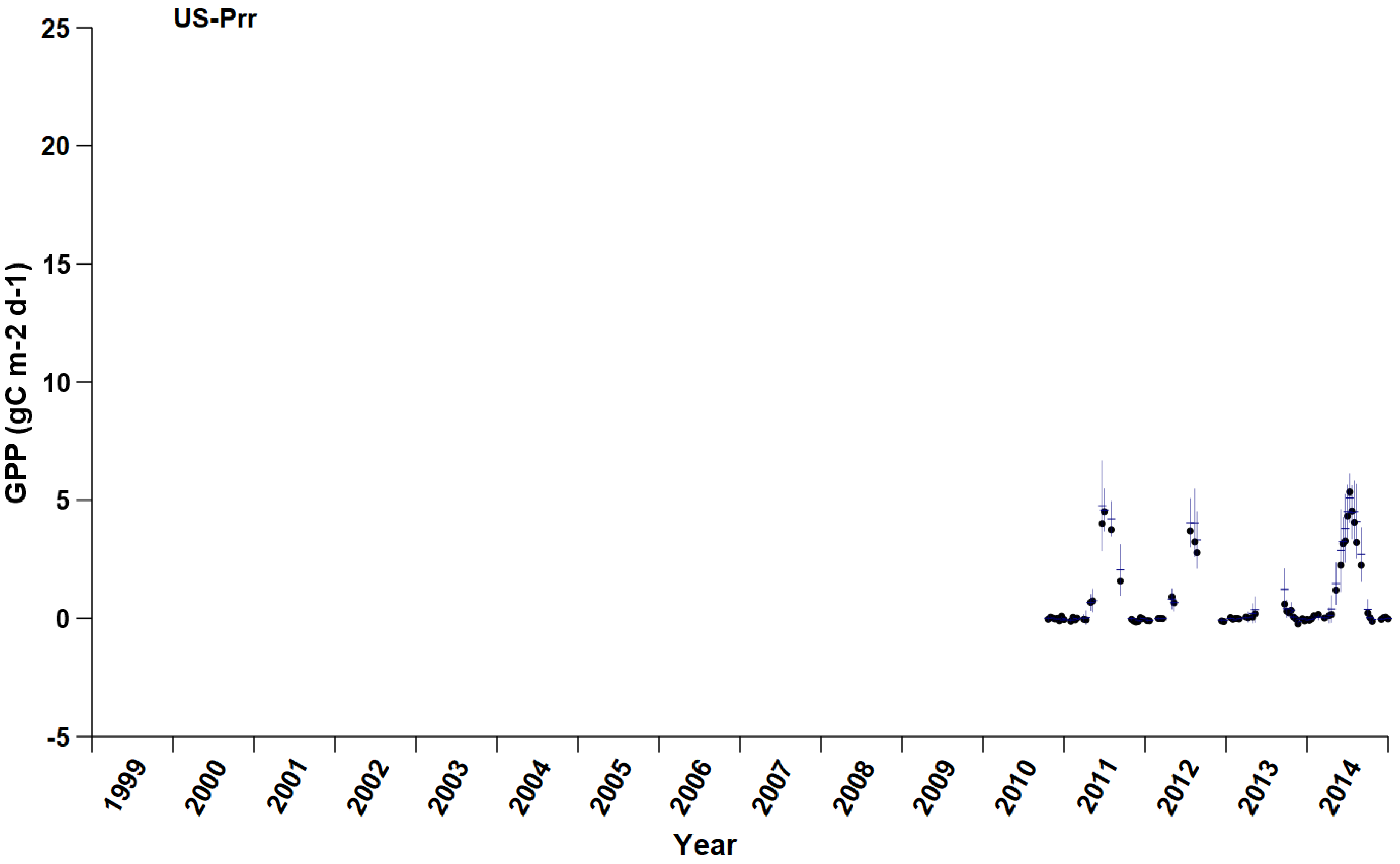
US-Prr
GPP (gC m-2 d-1)
25
20
15
10
5
0
-5
1999
2000
2001
2002
2003
2004
2005
2006
2007
2008
2009
2010
2011
2012
2013
2014
Year

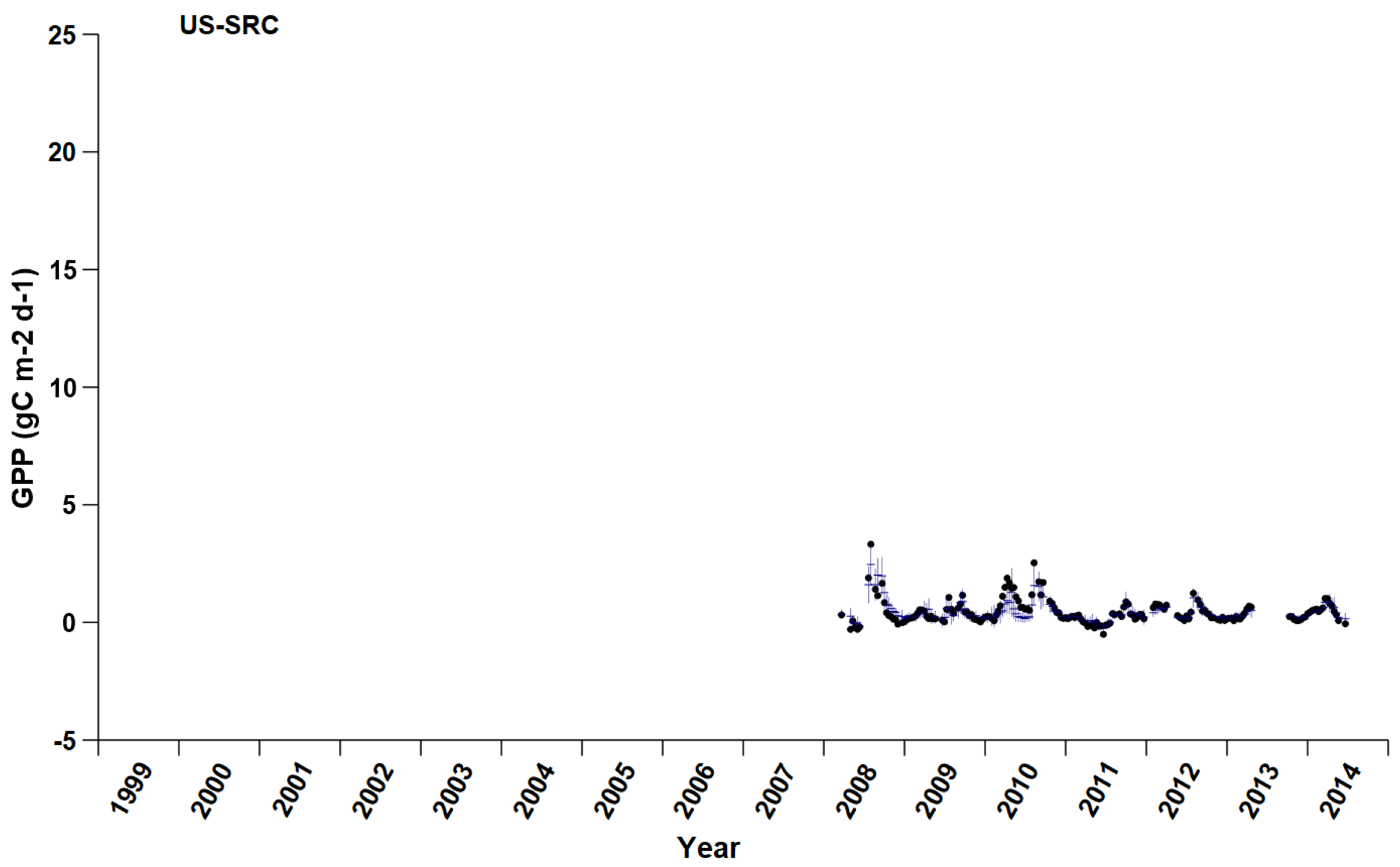

US-SRC
GPP (gC m-2 d-1)
25
20
15
10
5
0
-5
1999
2000
2001
2002
2003
2004
2005
2006
2007
2008
2009
2010
2011
2012
2013
2014
Year

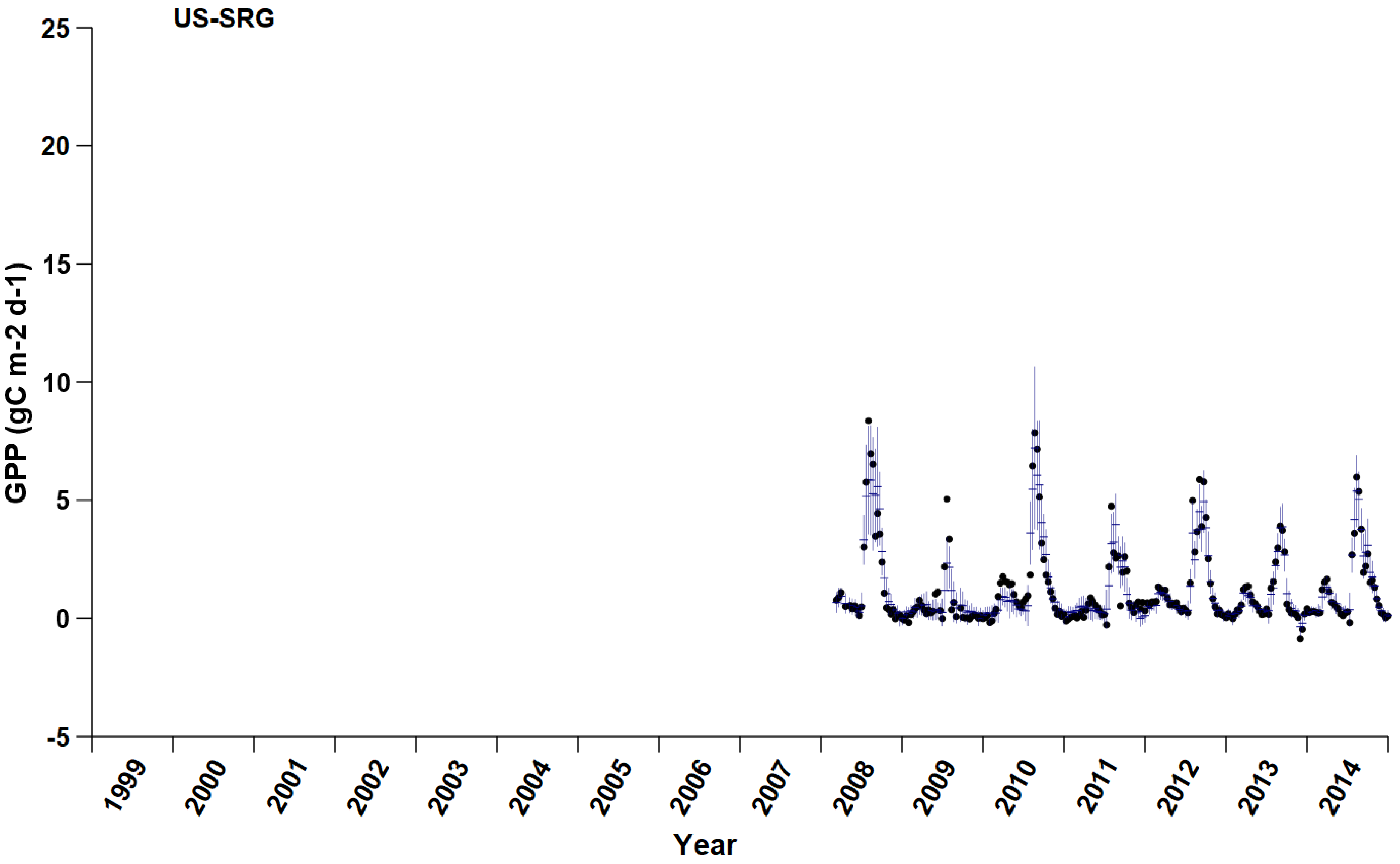
US-SRG
GPP (gC m-2 d-1)
25
20
15
10
5
0
-5
1999
2000
2001
2002
2003
2004
2005
2006
2007
2008
2009
2010
2011
2012
2013
2014
Year

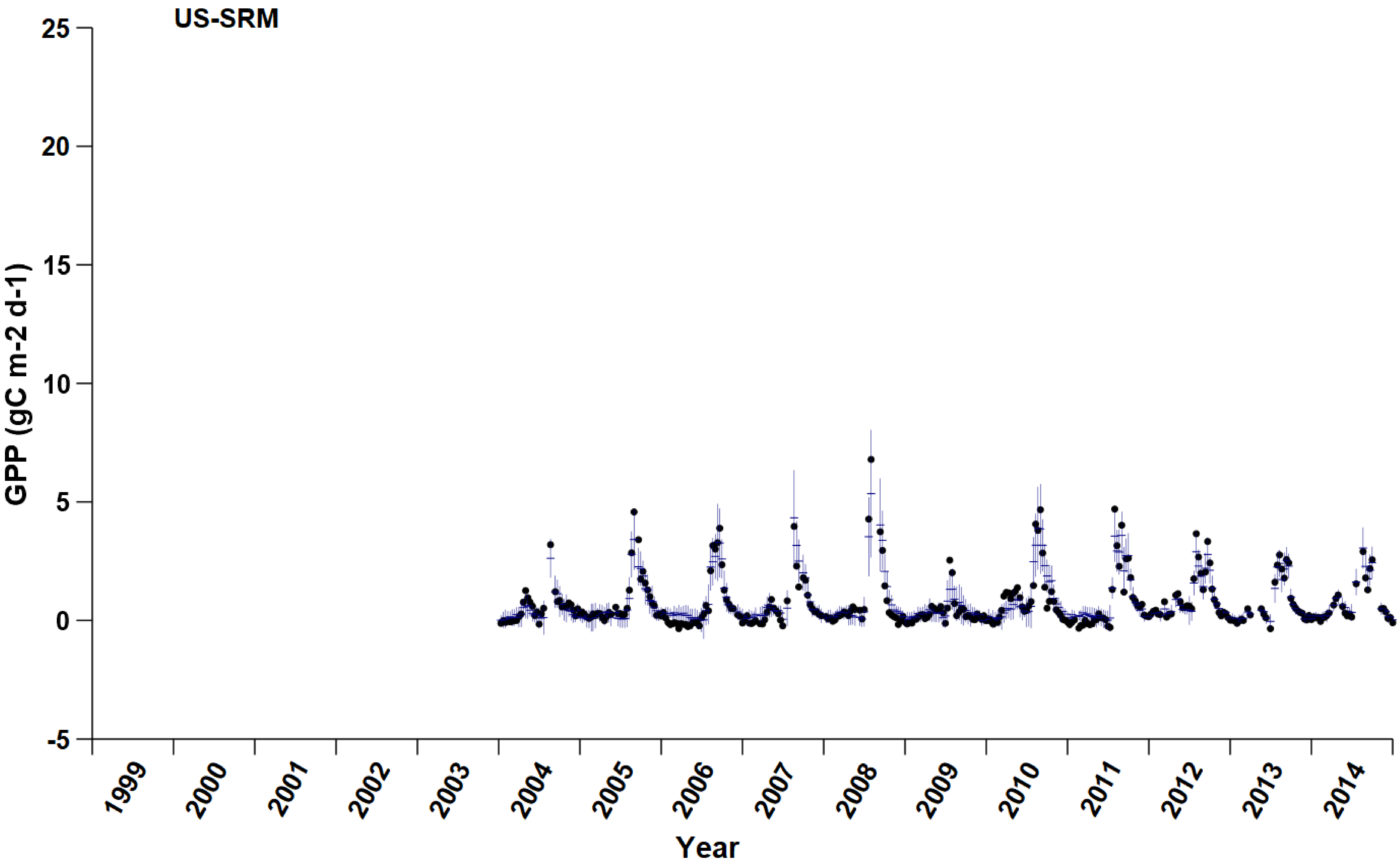

US-SRM
GPP (gC m-2 d-1)
25
20
15
10
5
0
-5
1999
2000
2001
2002
2003
2004
2005
2006
2007
2008
2009
2010
2011
2012
2013
2014
Year

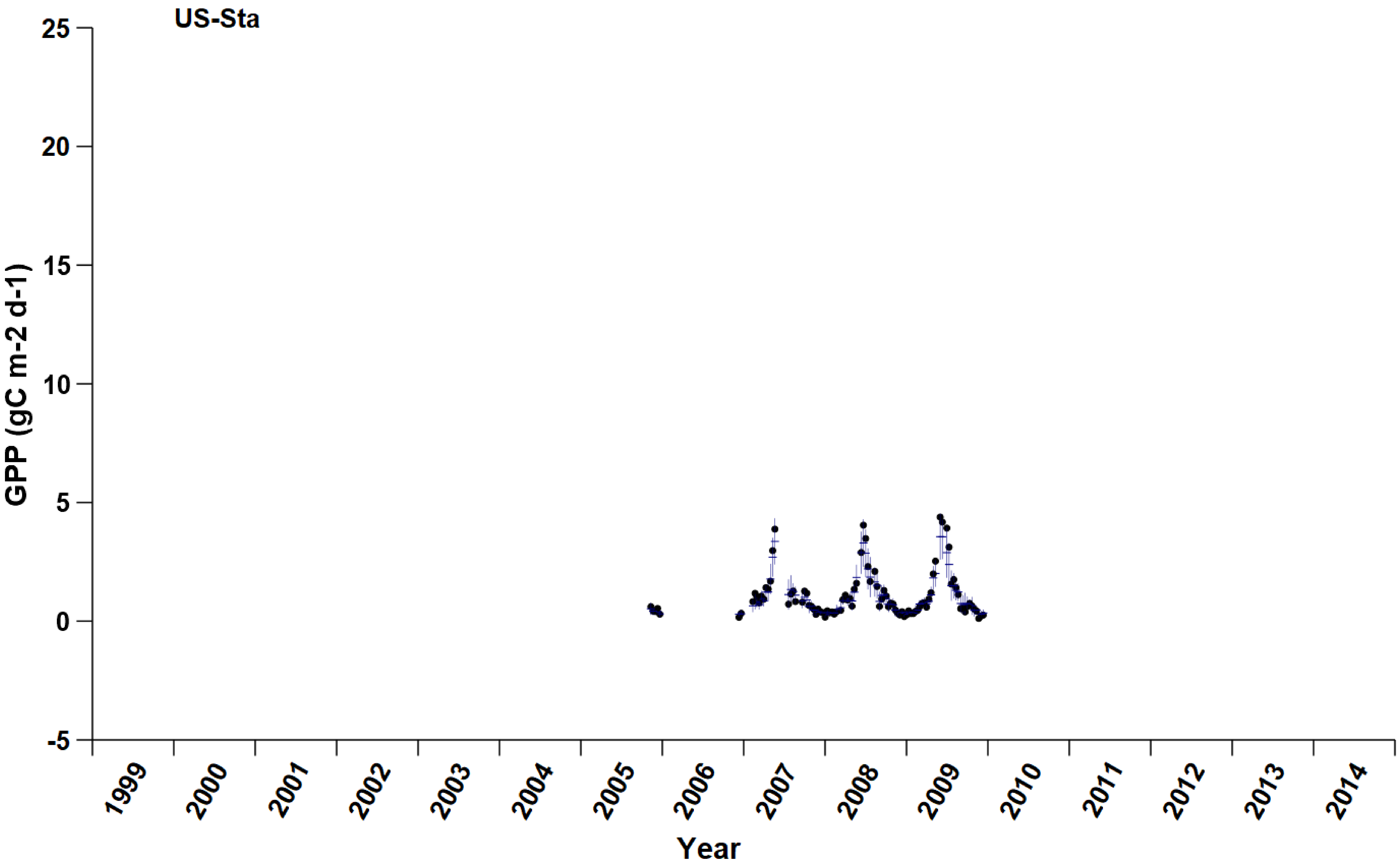
US-Sta
GPP (gC m-2 d-1)
25
20
15
10
5
0
-5
1999
2000
2001
2002
2003
2004
2005
2006
2007
2008
2009
2010
2011
2012
2013
2014
Year

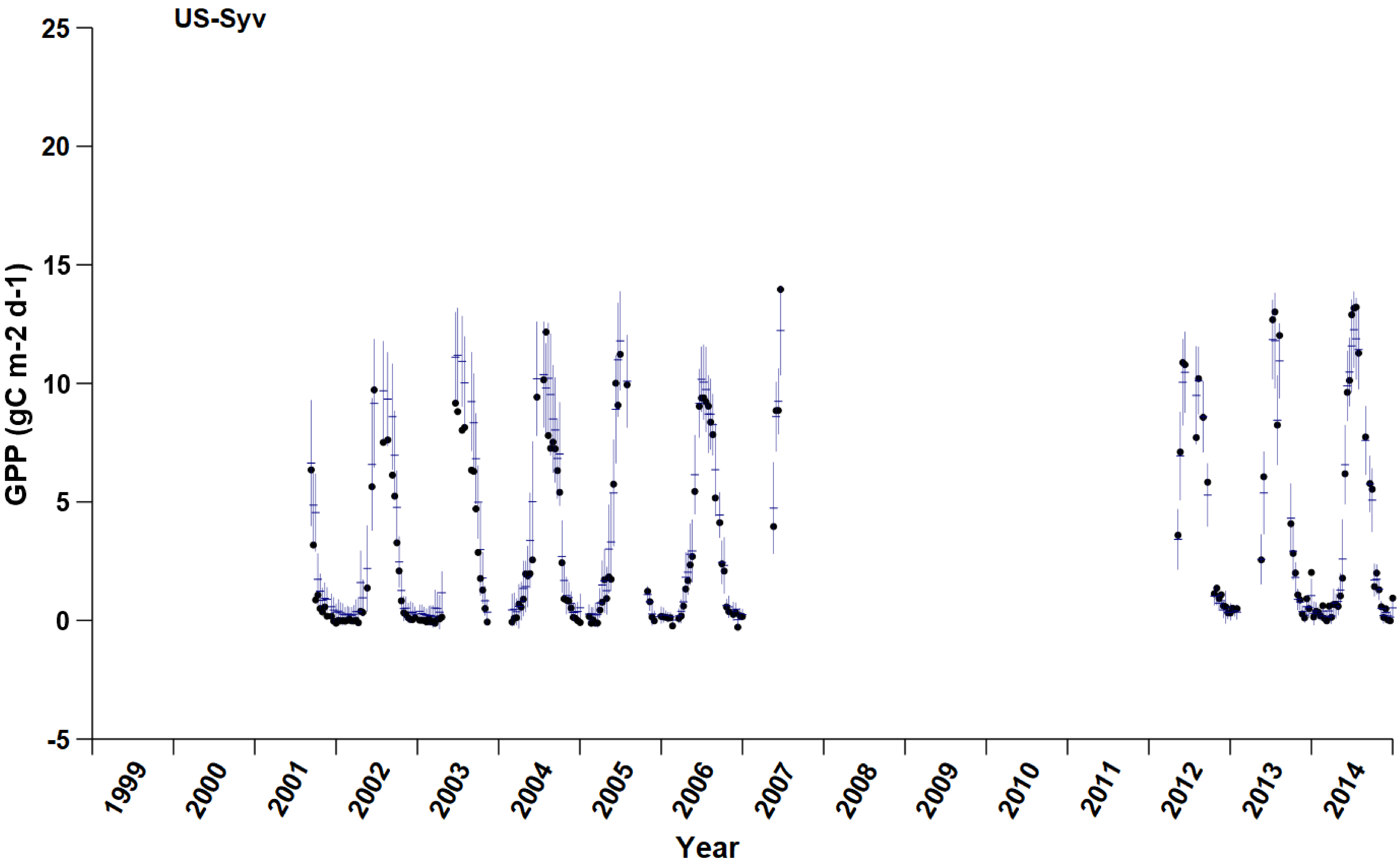

US-Syv
GPP (gC m-2 d-1)
25
20
15
10
5
0
-5
1999
2000
2001
2002
2003
2004
2005
2006
2007
2008
2009
2010
2011
2012
2013
2014
Year

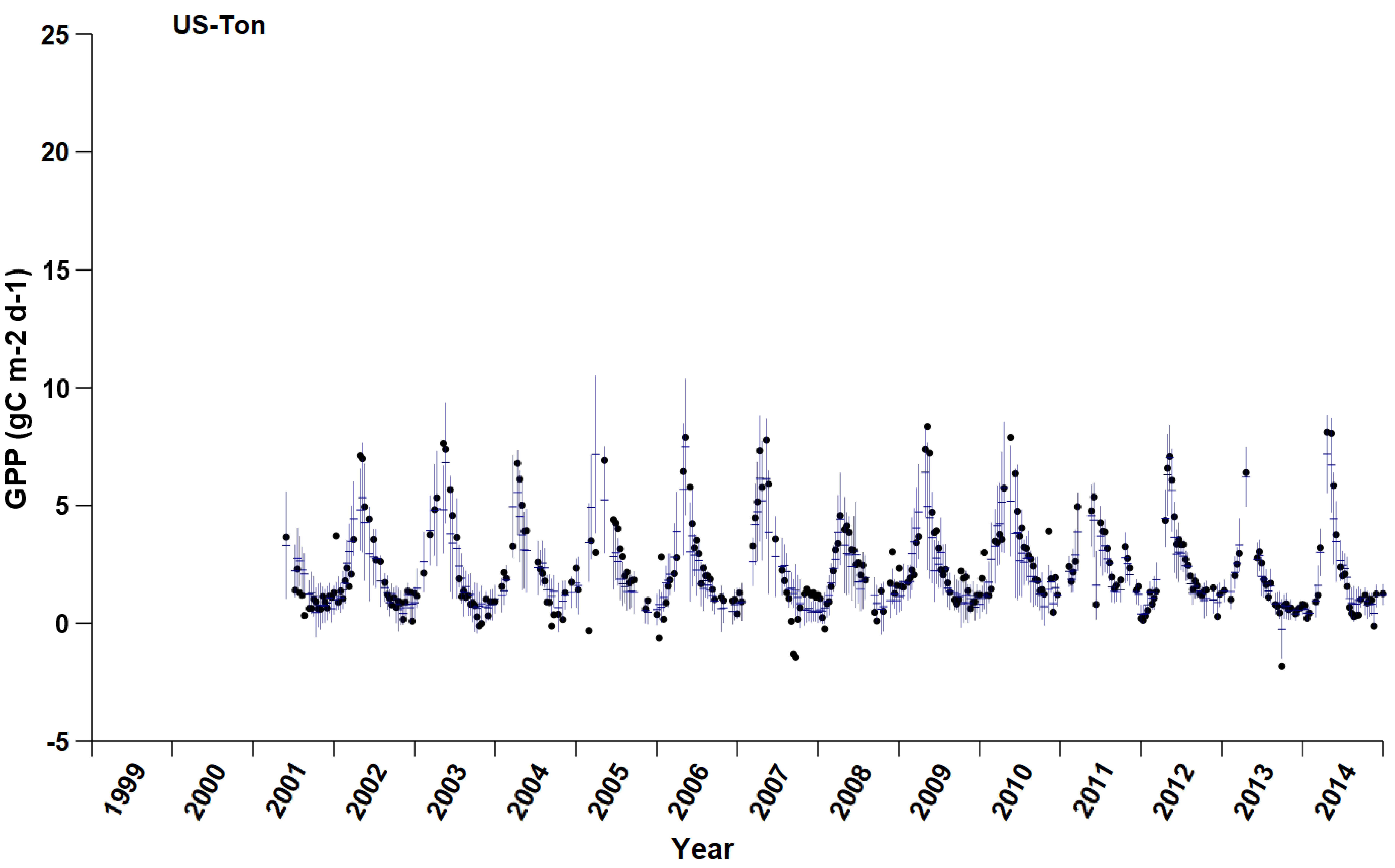

US-Ton
GPP (gC m-2 d-1)
25
20
15
10
5
0
-5
1999
2000
2001
2002
2003
2004
2005
2006
2007
2008
2009
2010
2011
2012
2013
2014
Year

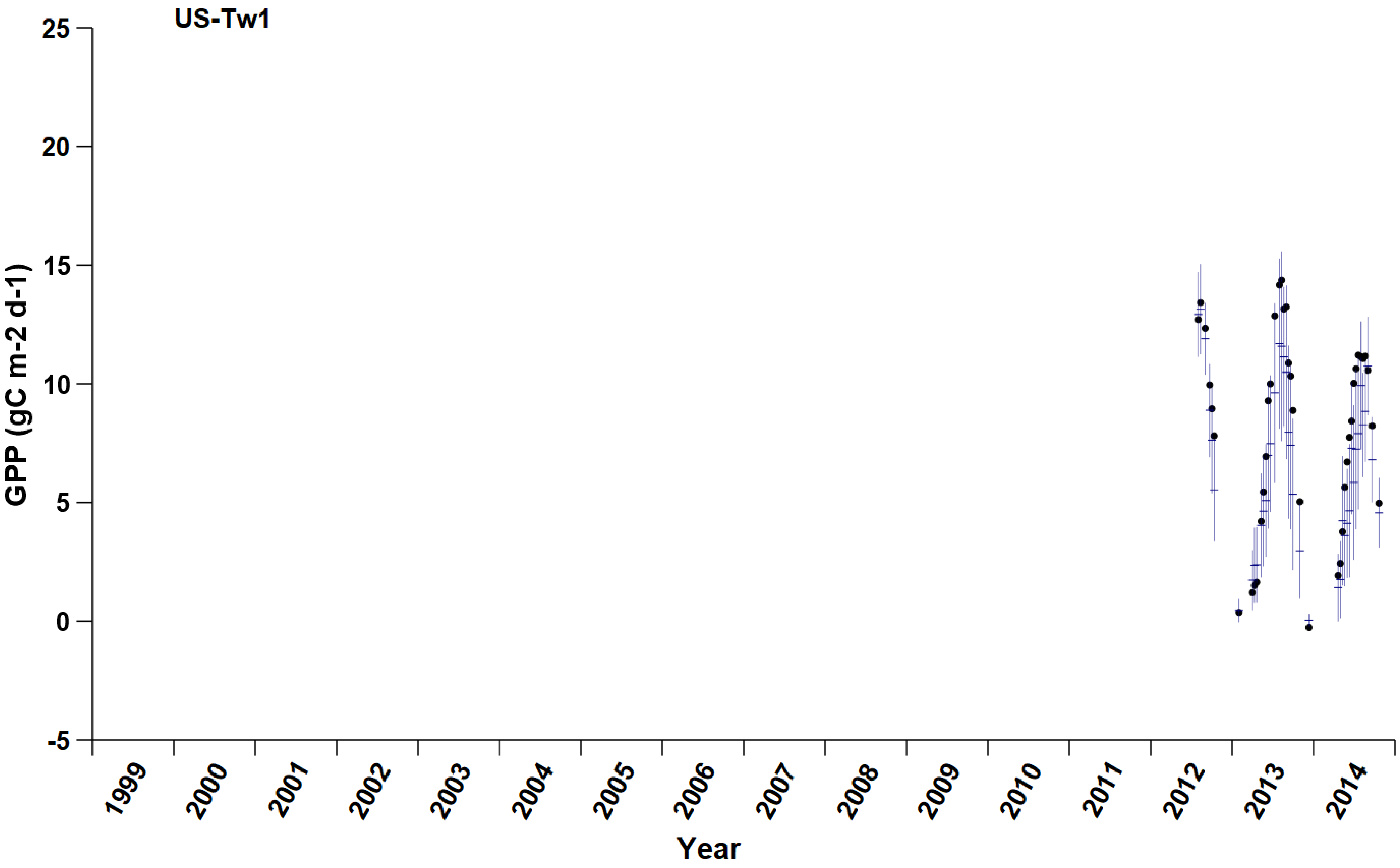

US-Tw1
GPP (gC m-2 d-1)
25
20
15
10
5
0
-5
1999
2000
2001
2002
2003
2004
2005
2006
2007
2008
2009
2010
2011
2012
2013
2014
Year

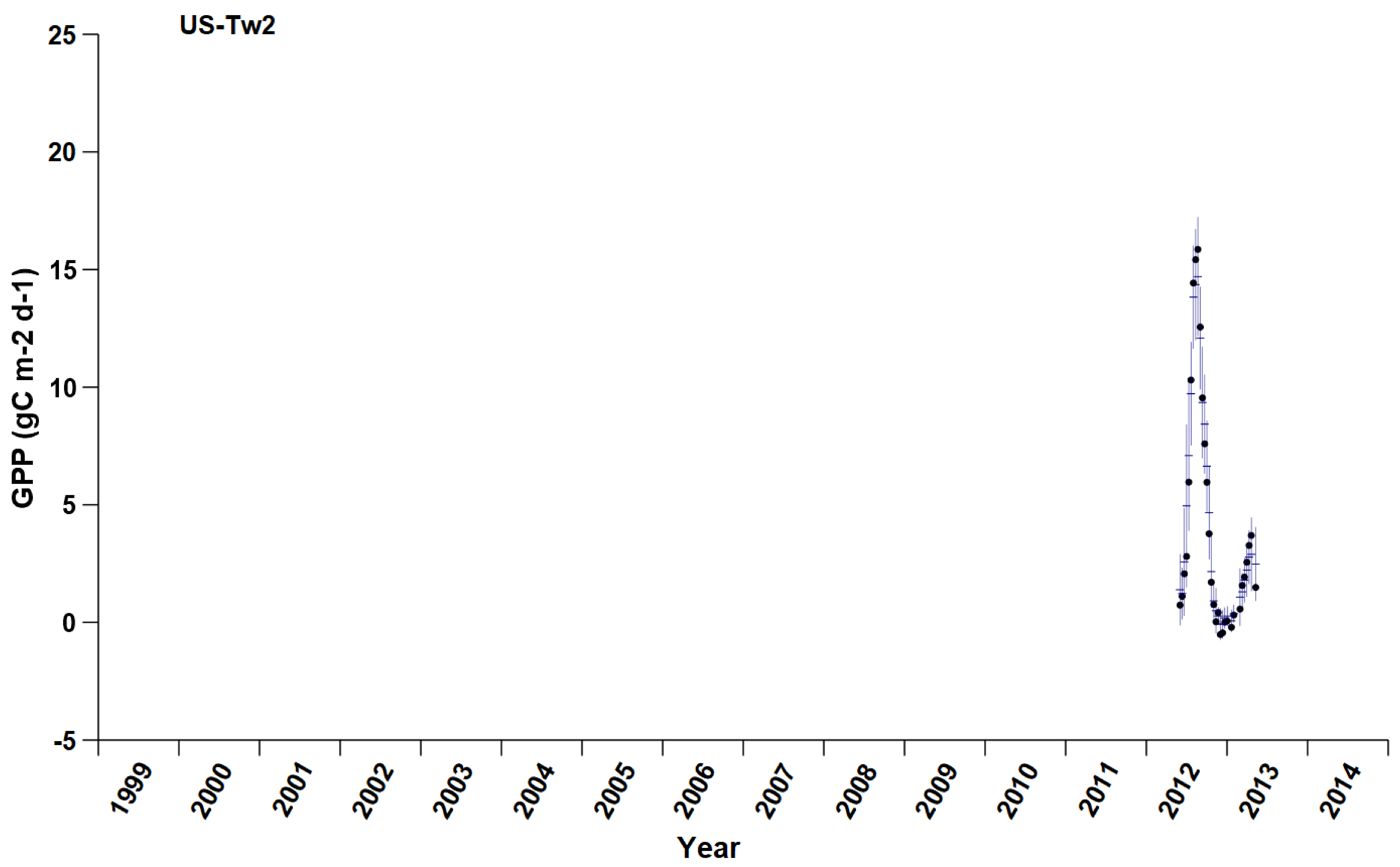
US-Tw2
GPP (gC m-2 d-1)
25
20
15
10
5
0
-5
1999
2000
2001
2002
2003
2004
2005
2006
2007
2008
2009
2010
2011
2012
2013
2014
Year

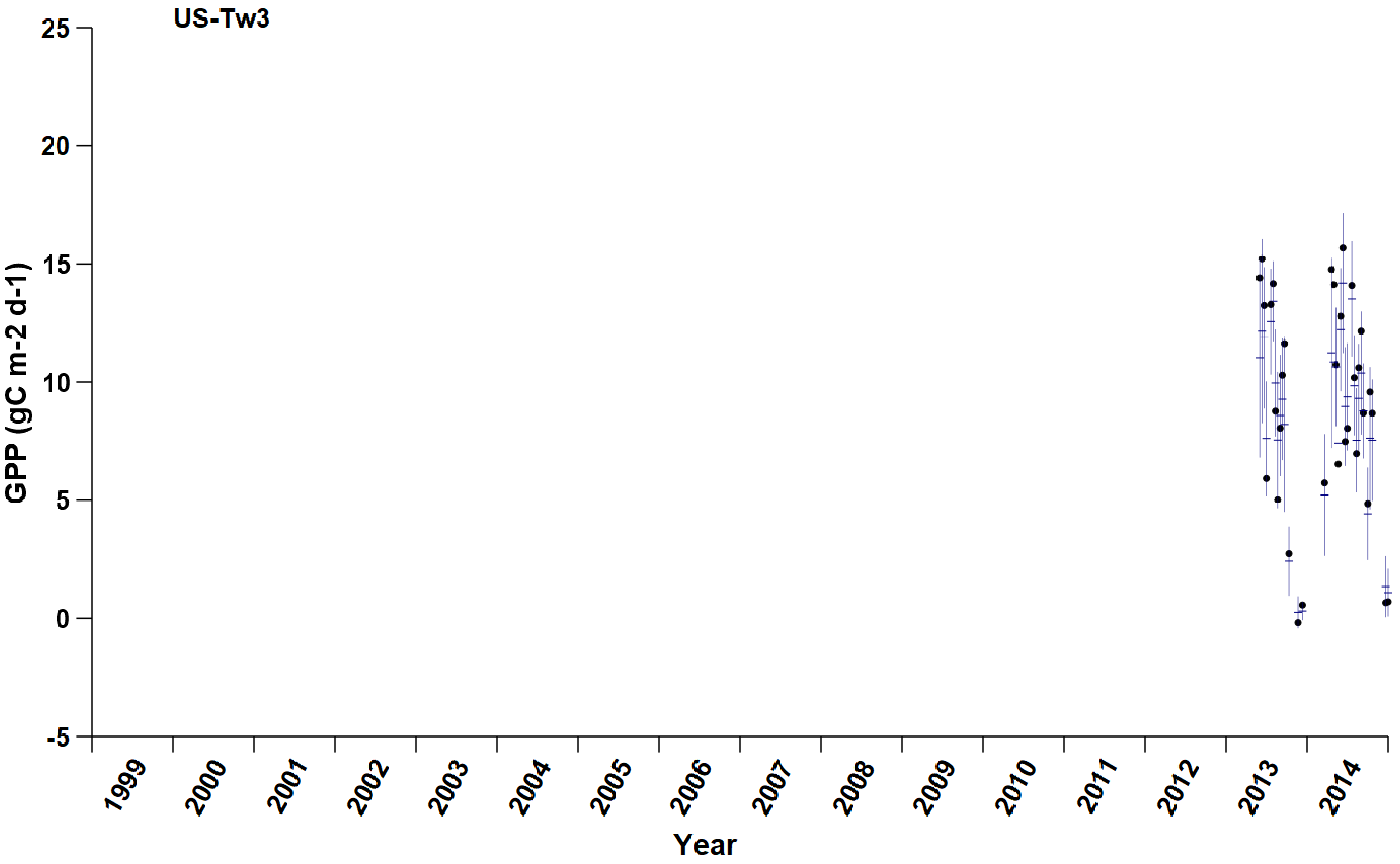
US-Tw3
GPP (gC m-2 d-1)
25
20
15
10
5
0
-5
1999
2000
2001
2002
2003
2004
2005
2006
2007
2008
2009
2010
2011
2012
2013
2014
Year

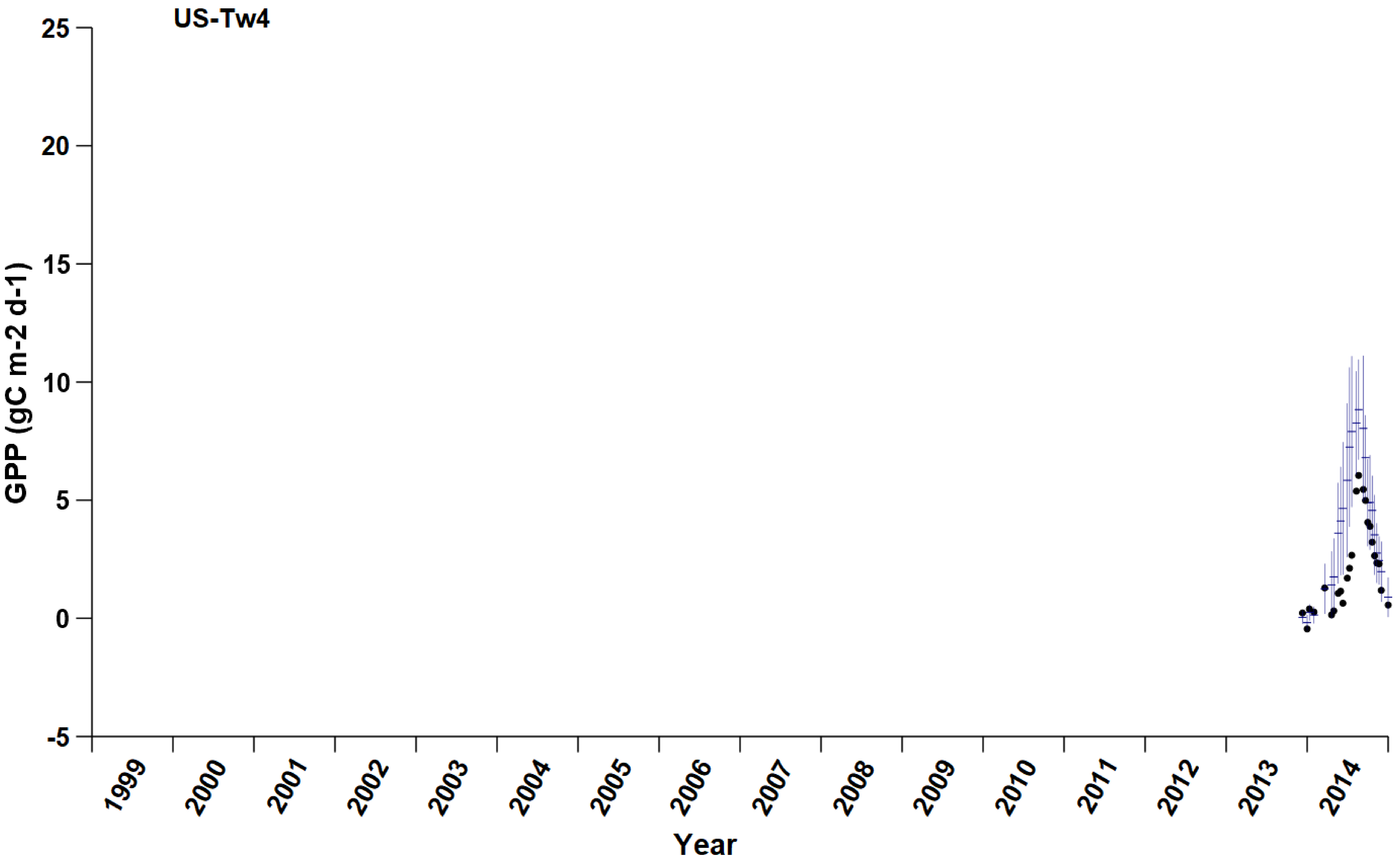
US-Tw4
GPP (gC m-2 d-1)
25
20
15
10
5
0
-5
1999
2000
2001
2002
2003
2004
2005
2006
2007
2008
2009
2010
2011
2012
2013
2014
Year

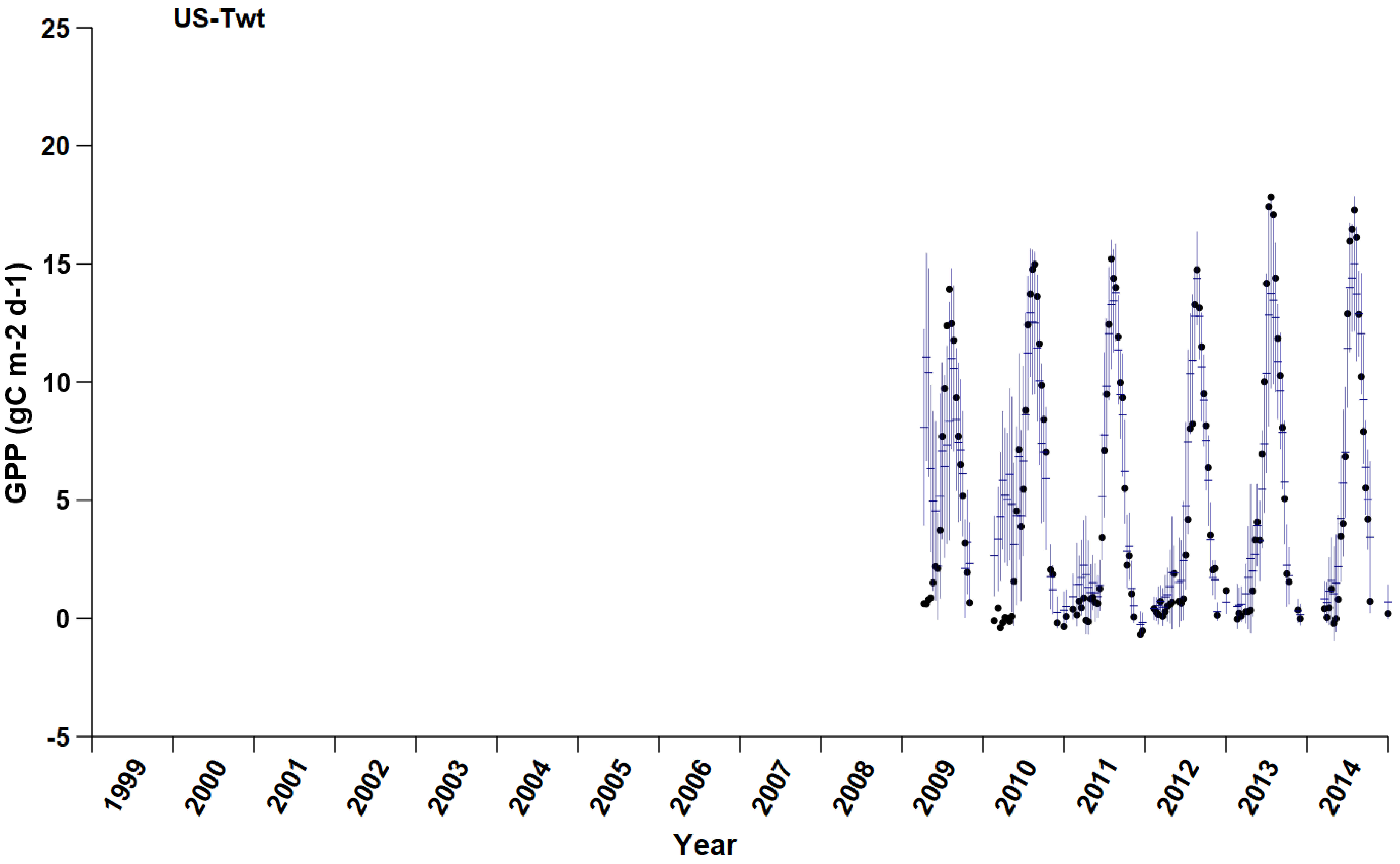

US-Twt
GPP (gC m-2 d-1)
Year
25
20
15
10
5
0
-5
1999
2000
2001
2002
2003
2004
2005
2006
2007
2008
2009
2010
2011
2012
2013
2014

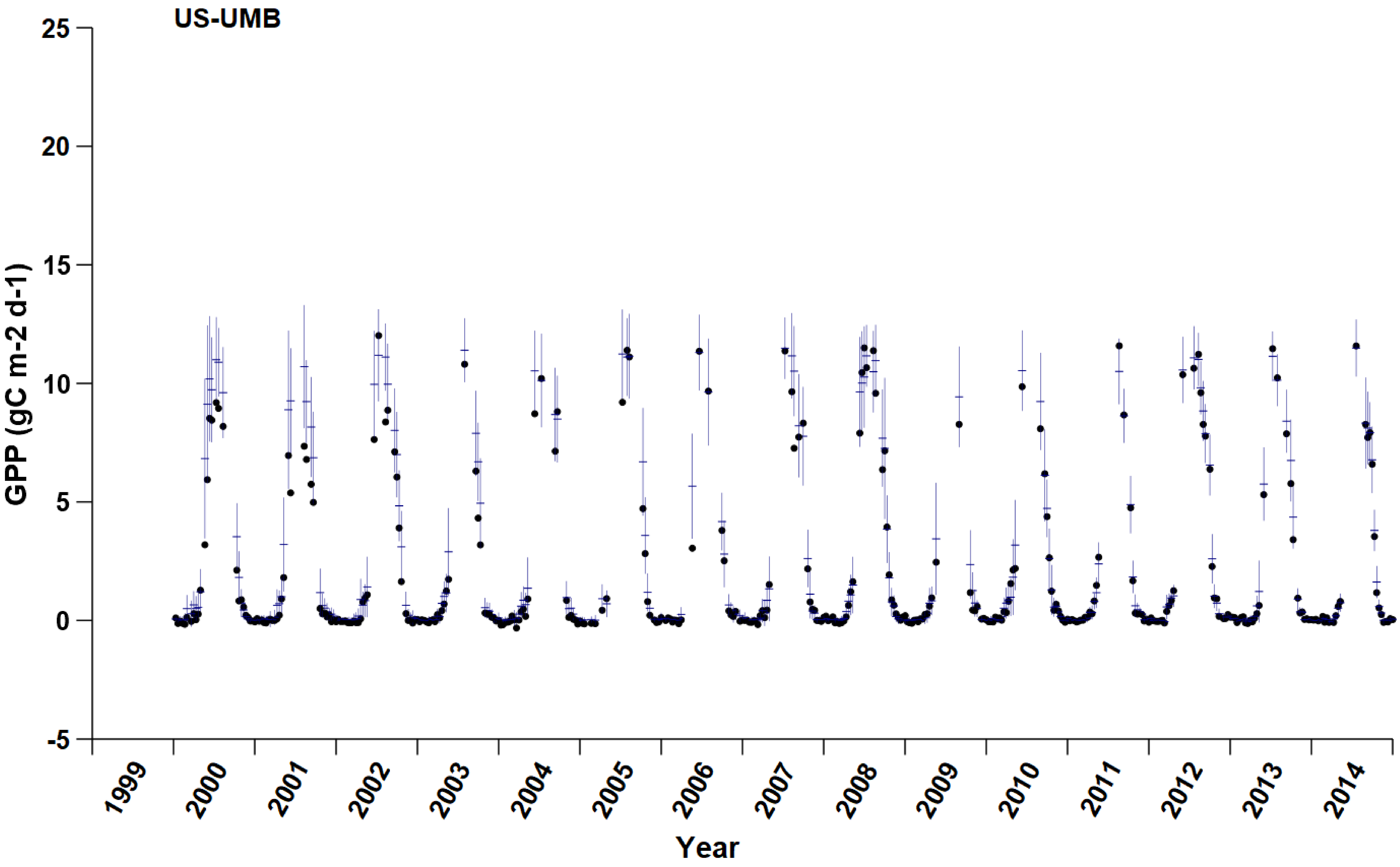
US-UMB
GPP (gC m-2 d-1)
Year
25
20
15
10
5
0
-5
1999
2000
2001
2002
2003
2004
2005
2006
2007
2008
2009
2010
2011
2012
2013
2014

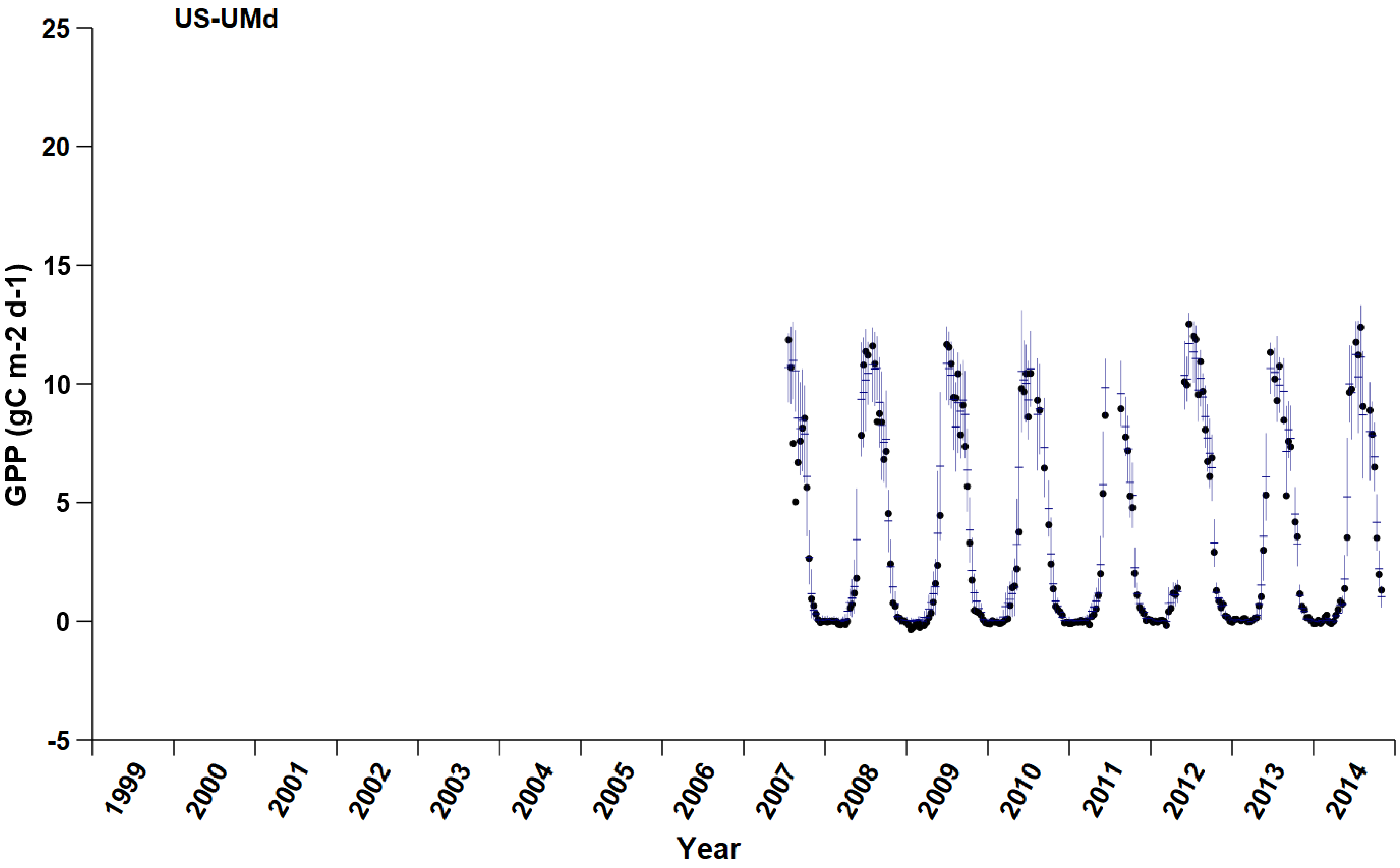

US-UMd
GPP (gC m-2 d-1)
25
20
15
10
5
0
-5
1999
2000
2001
2002
2003
2004
2005
2006
2007
2008
2009
2010
2011
2012
2013
2014
Year

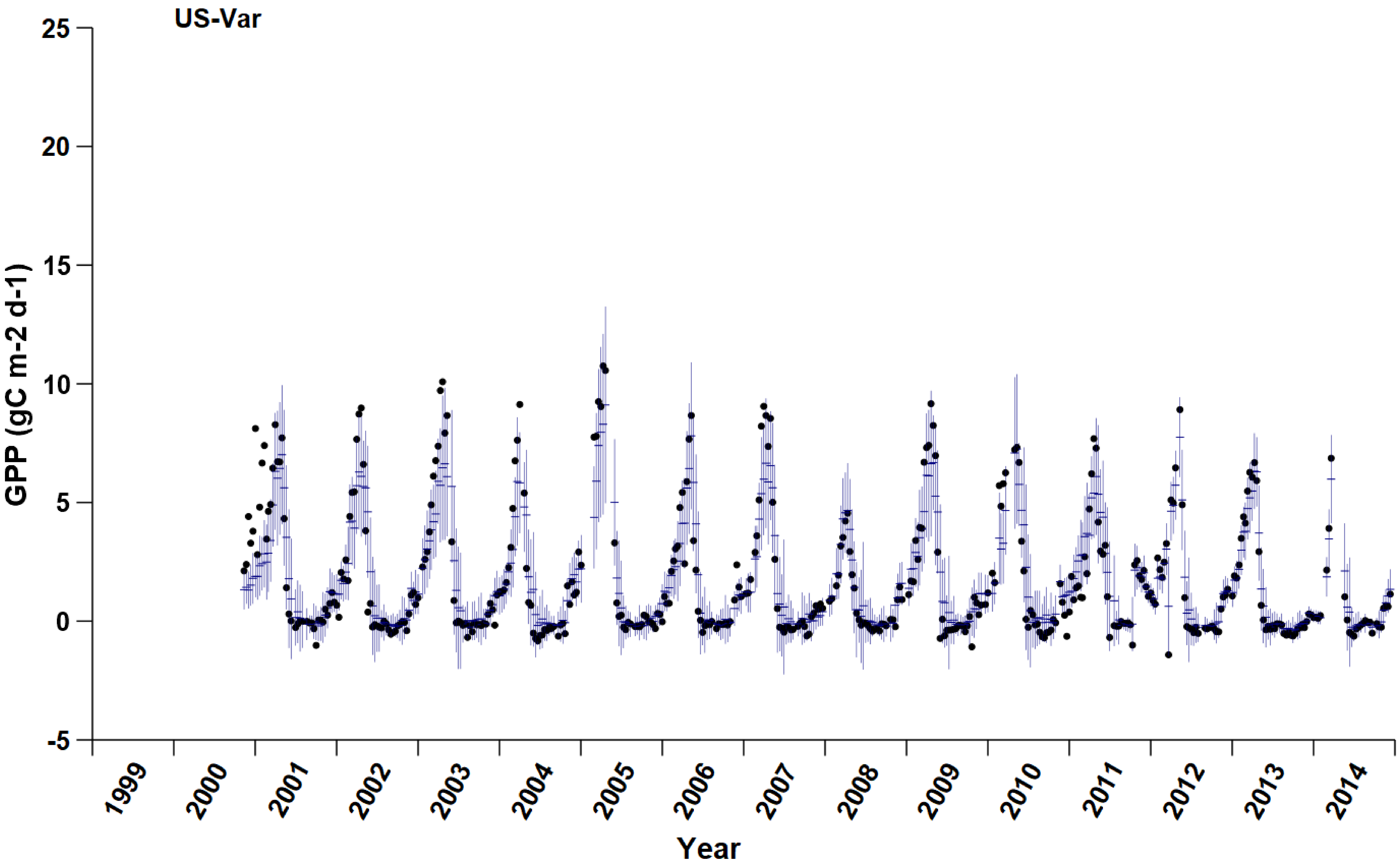

US-Var
GPP (gC m-2 d-1)
25
20
15
10
5
0
-5
1999
2000
2001
2002
2003
2004
2005
2006
2007
2008
2009
2010
2011
2012
2013
2014
Year

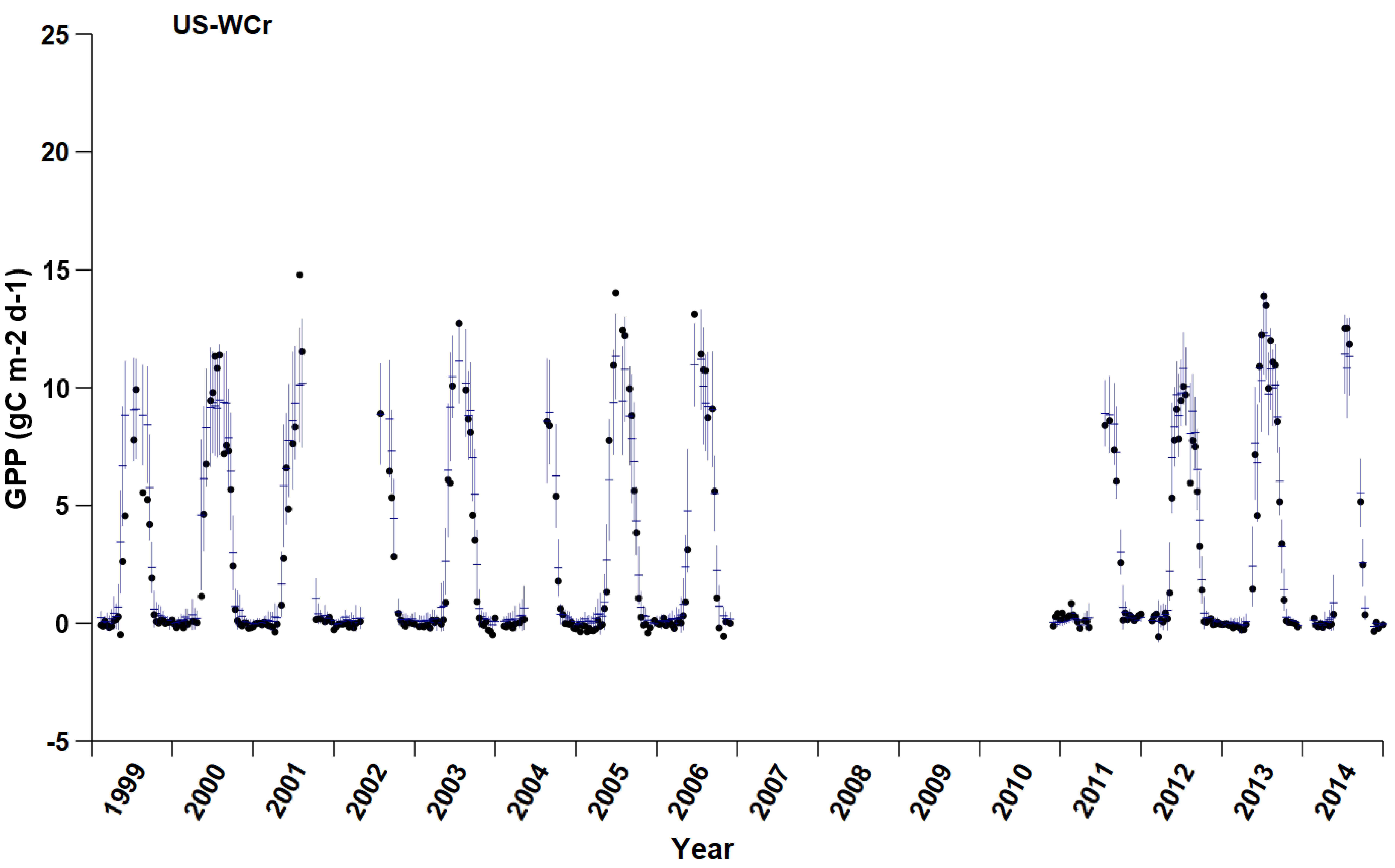

US-WCr
GPP (gC m-2 d-1)
25
20
15
10
5
0
-5
1999
2000
2001
2002
2003
2004
2005
2006
2007
2008
2009
2010
2011
2012
2013
2014
Year

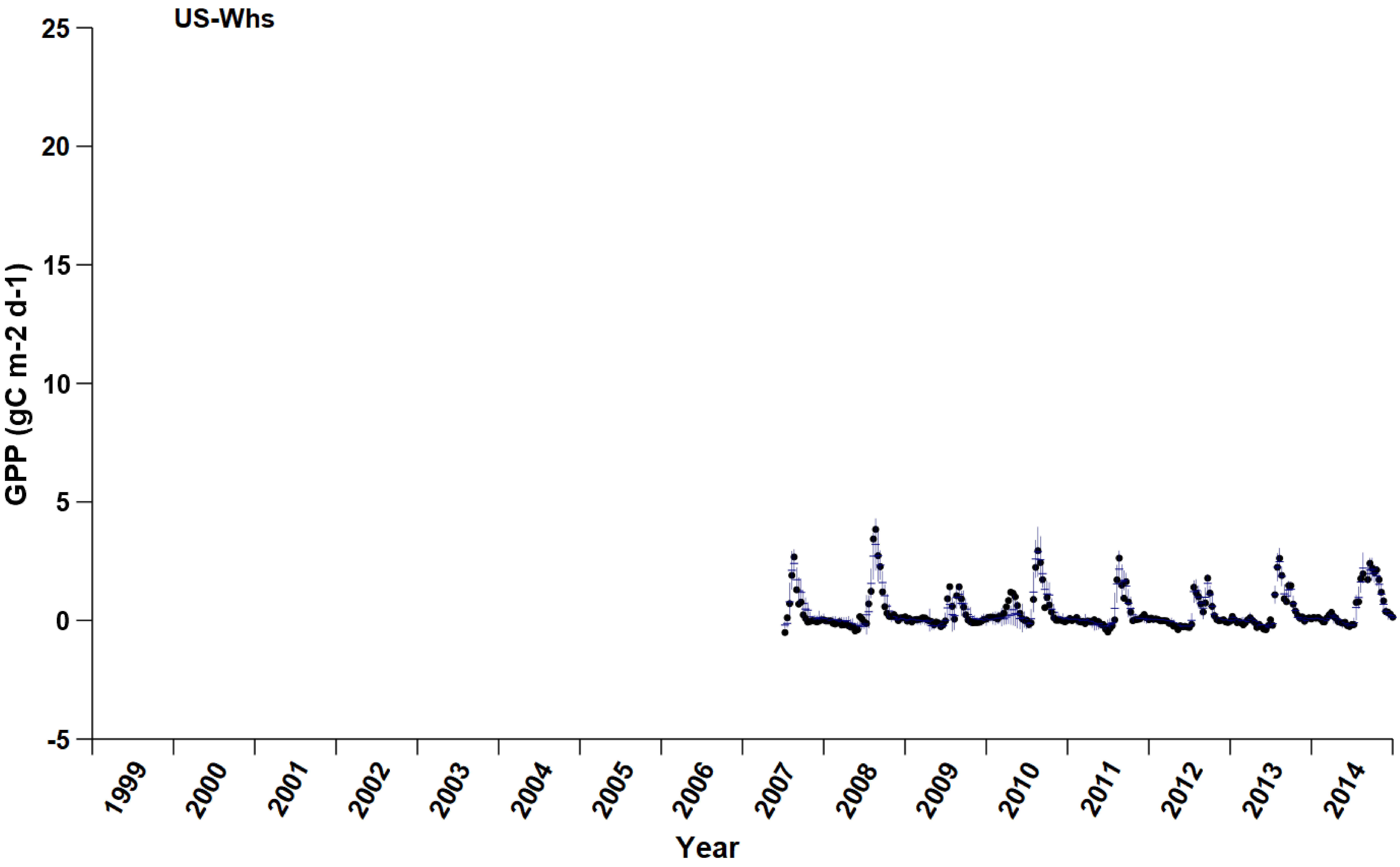

US-Whs
GPP (gC m-2 d-1)
25
20
15
10
5
0
-5
1999
2000
2001
2002
2003
2004
2005
2006
2007
2008
2009
2010
2011
2012
2013
2014
Year

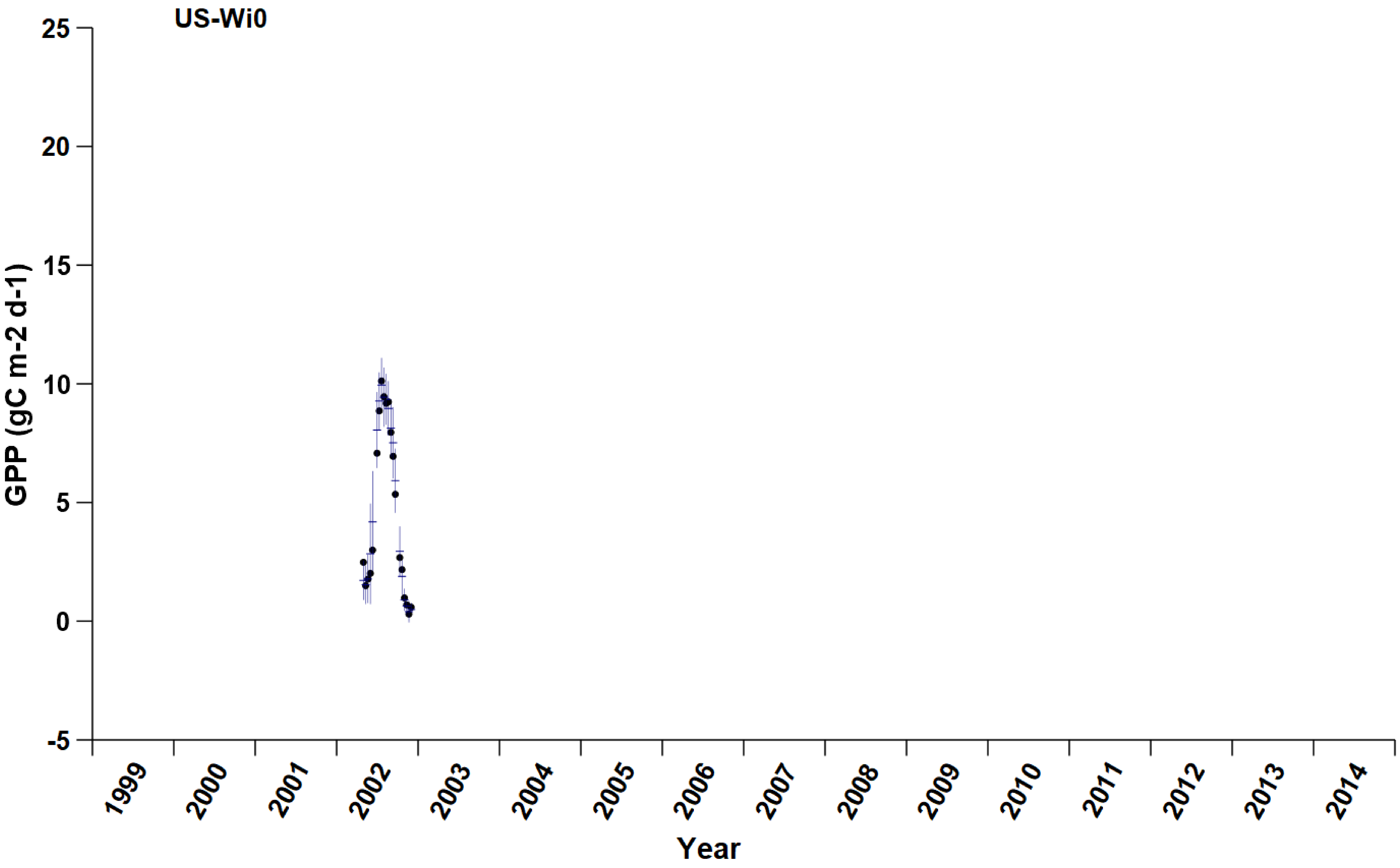
US-Wi0
GPP (gC m-2 d-1)
25
20
15
10
5
0
-5
1999
2000
2001
2002
2003
2004
2005
2006
2007
2008
2009
2010
2011
2012
2013
2014
Year

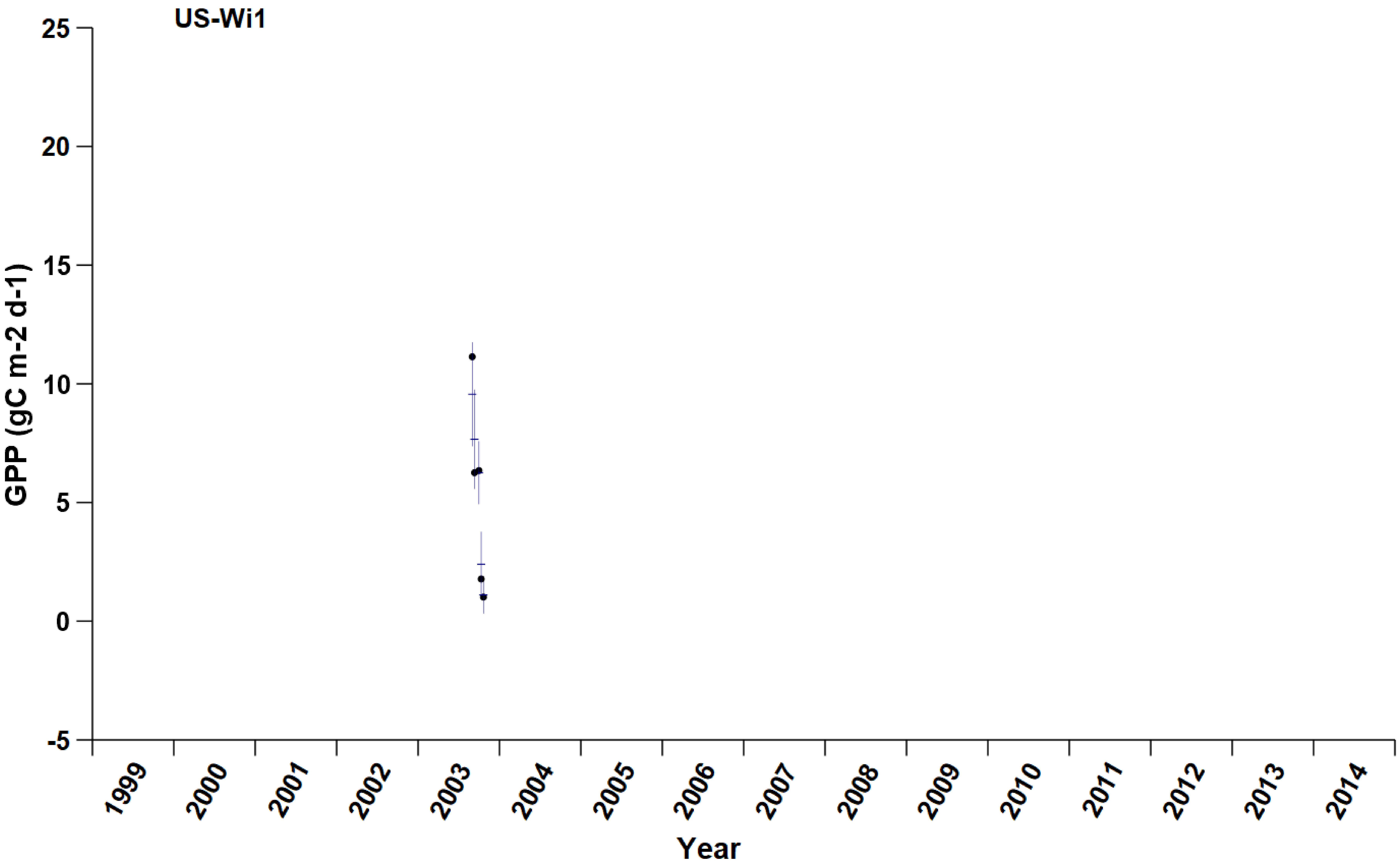
US-Wi1
25
20
15
10
5
0
-5
GPP (gC m-2 d-1)
1999
2000
2001
2002
2003
2004
2005
2006
2007
2008
2009
2010
2011
2012
2013
2014
Year

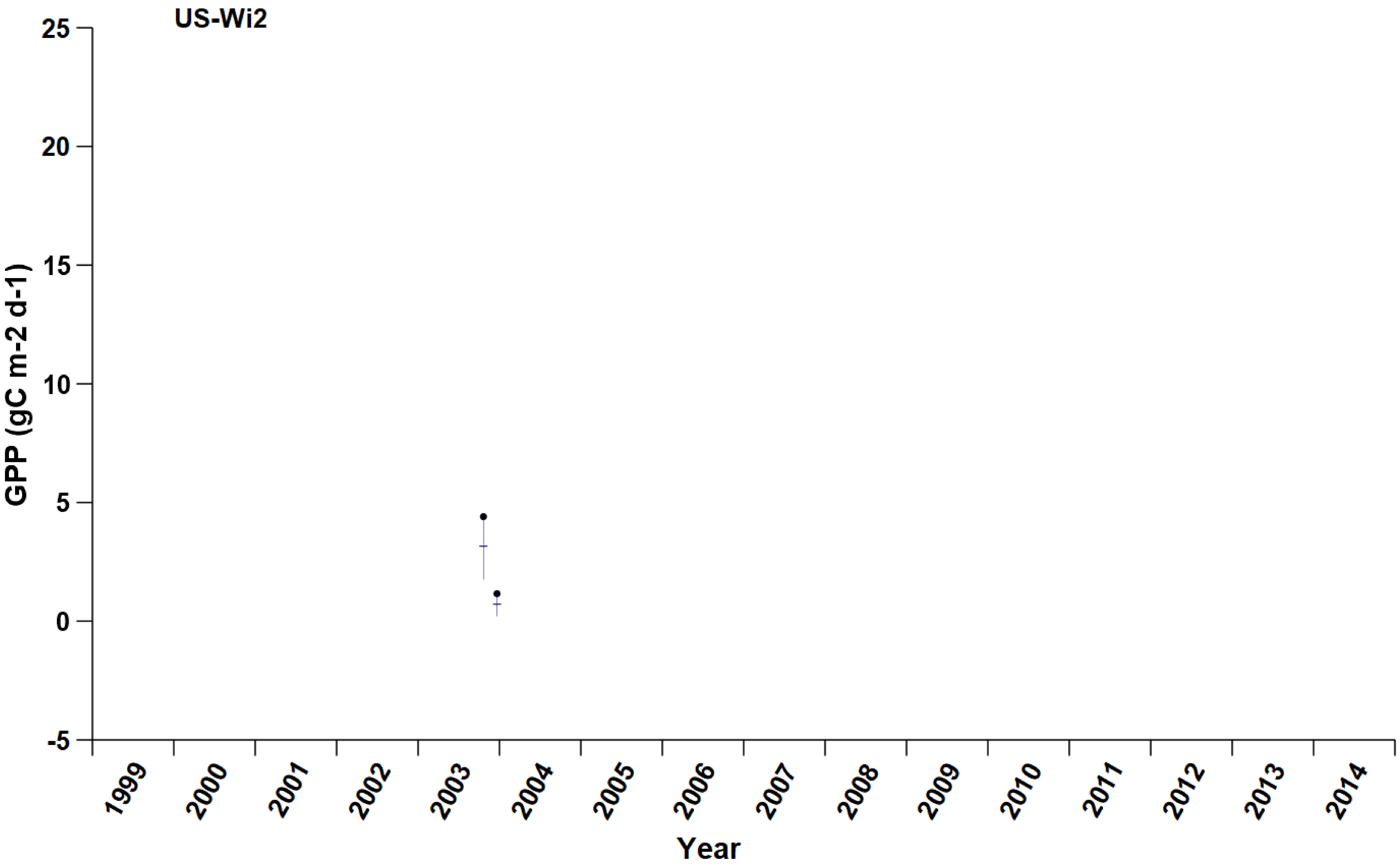
US-Wi2
GPP (gC m-2 d-1)
25
20
15
10
5
0
-5
1999
2000
2001
2002
2003
2004
2005
2006
2007
2008
2009
2010
2011
2012
2013
2014
Year

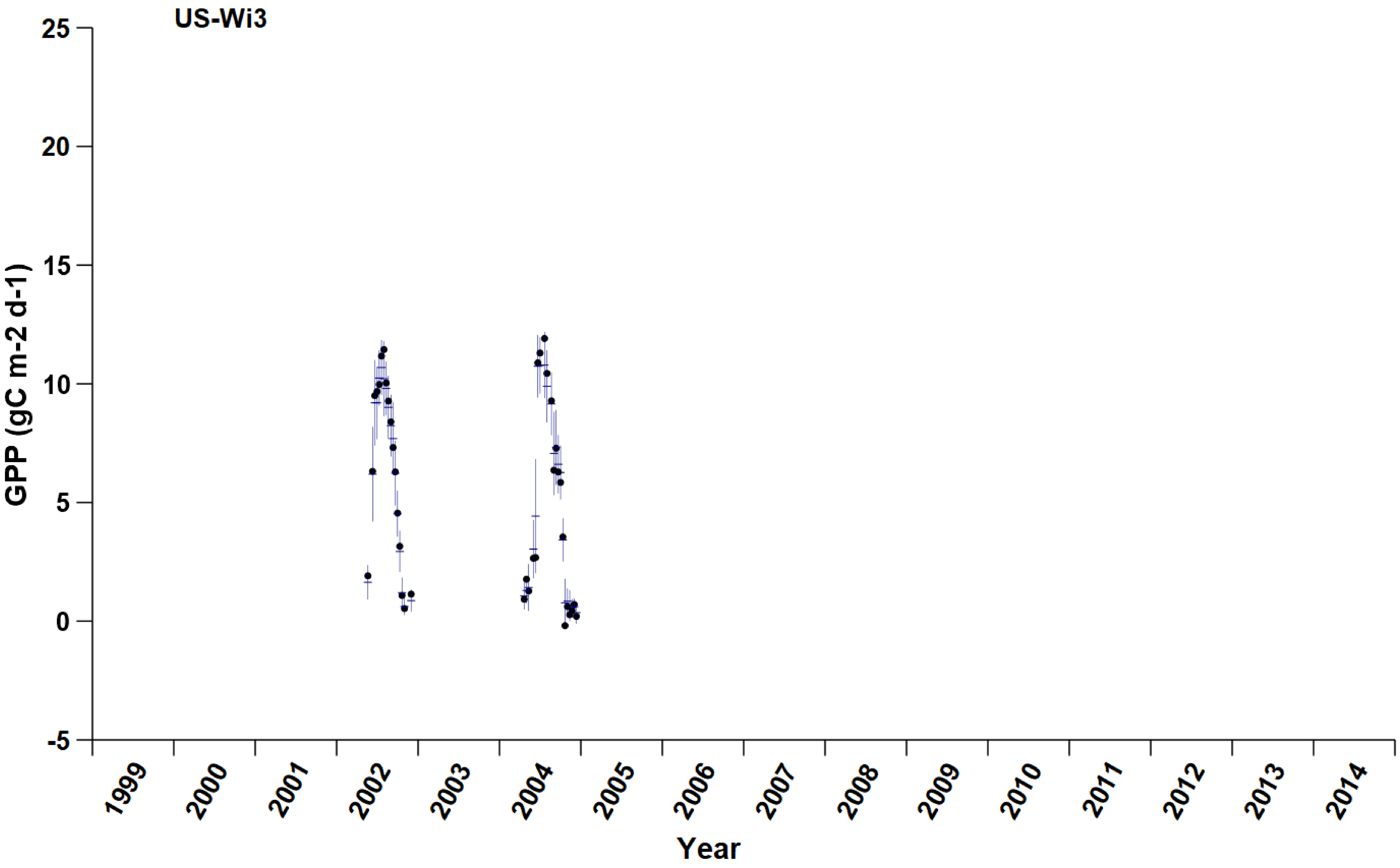
US-Wi3
GPP (gC m-2 d-1)
25
20
15
10
5
0
-5
1999
2000
2001
2002
2003
2004
2005
2006
2007
2008
2009
2010
2011
2012
2013
2014
Year

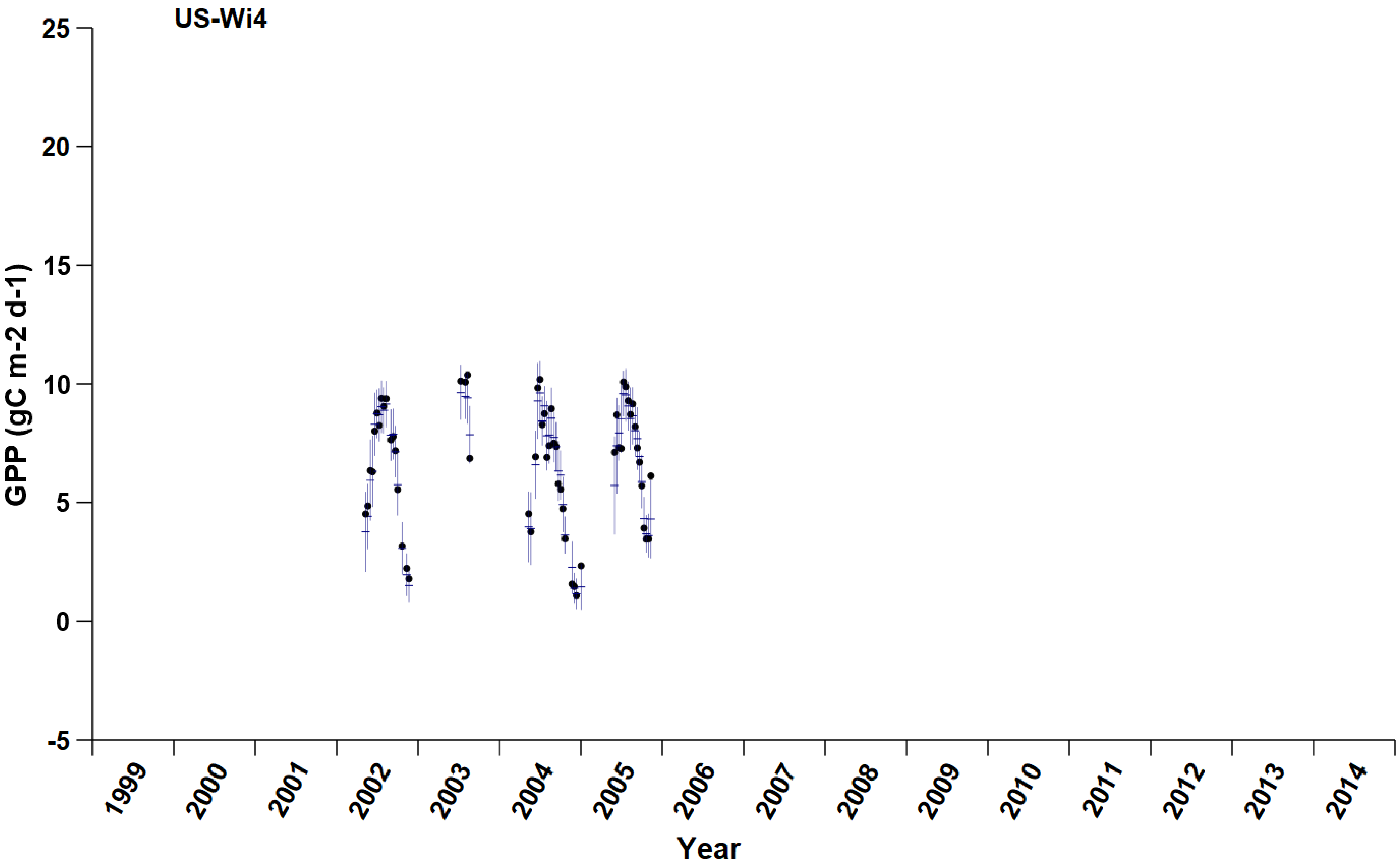

US-Wi4
GPP (gC m-2 d-1)
25
20
15
10
5
0
-5
1999
2000
2001
2002
2003
2004
2005
2006
2007
2008
2009
2010
2011
2012
2013
2014
Year

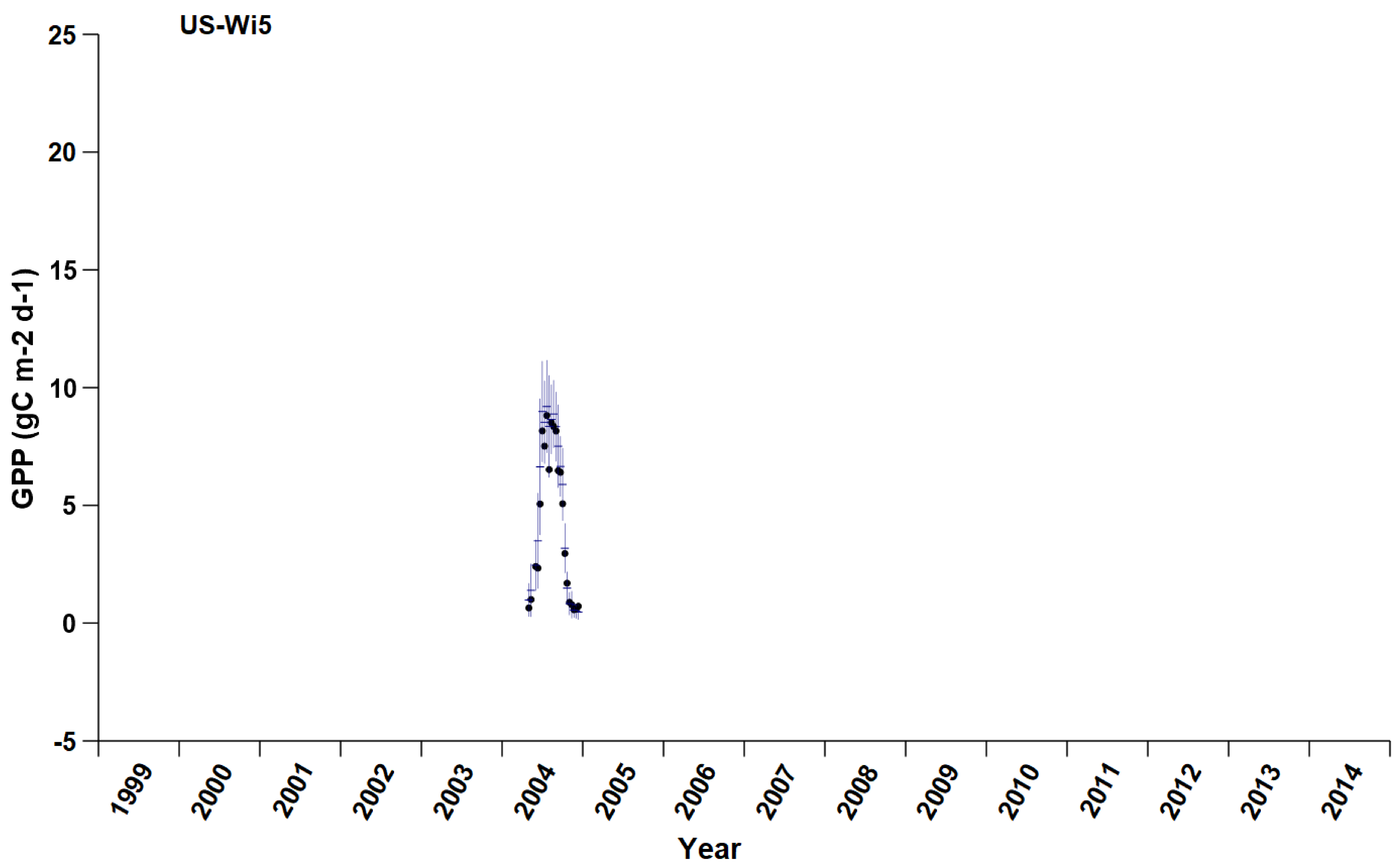

US-Wi5
GPP (gC m-2 d-1)
25
20
15
10
5
0
-5
1999
2000
2001
2002
2003
2004
2005
2006
2007
2008
2009
2010
2011
2012
2013
2014
Year

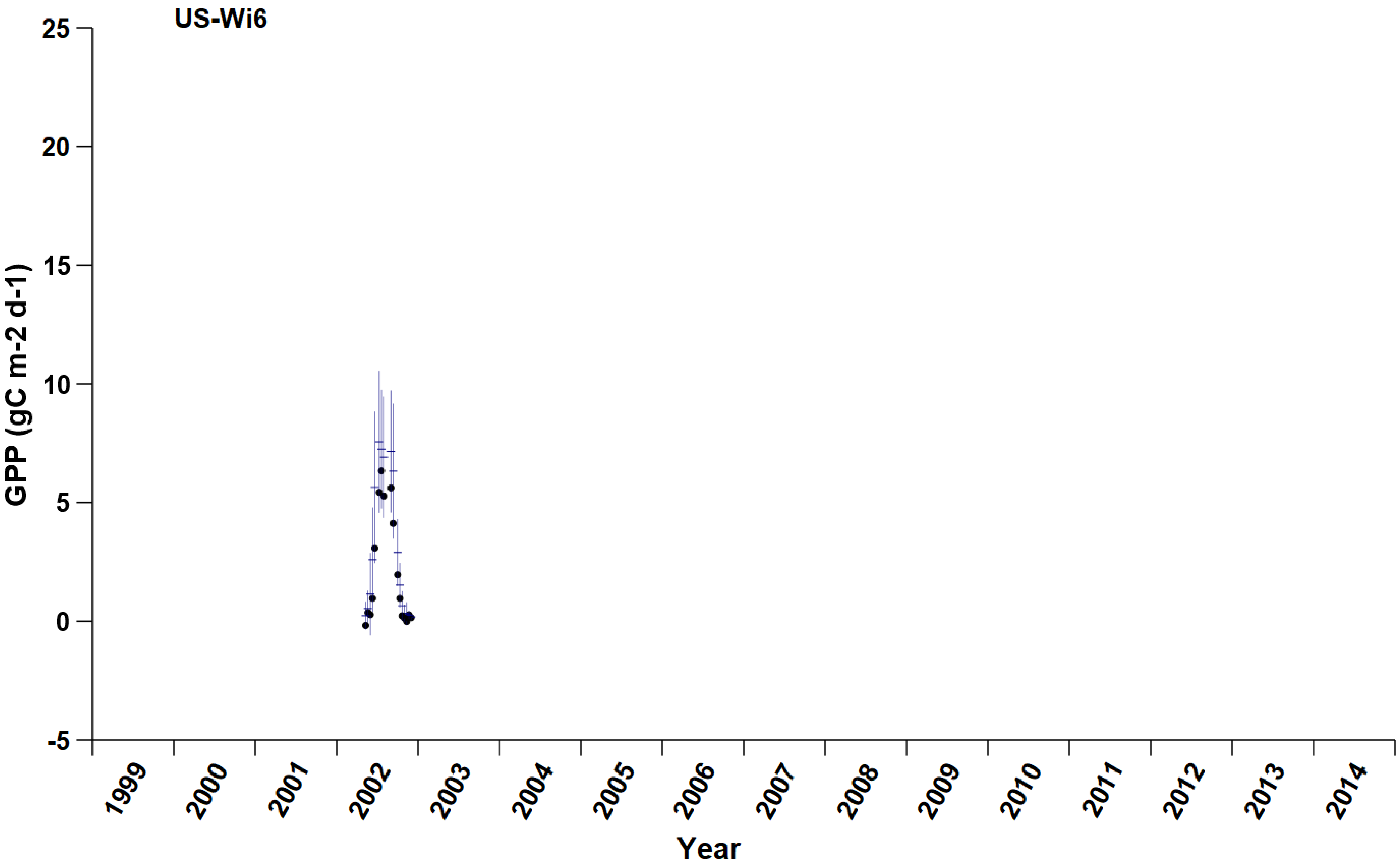
US-Wi6
25
20
15
10
5
0
-5
GPP (gC m-2 d-1)
1999
2000
2001
2002
2003
2004
2005
2006
2007
2008
2009
2010
2011
2012
2013
2014
Year

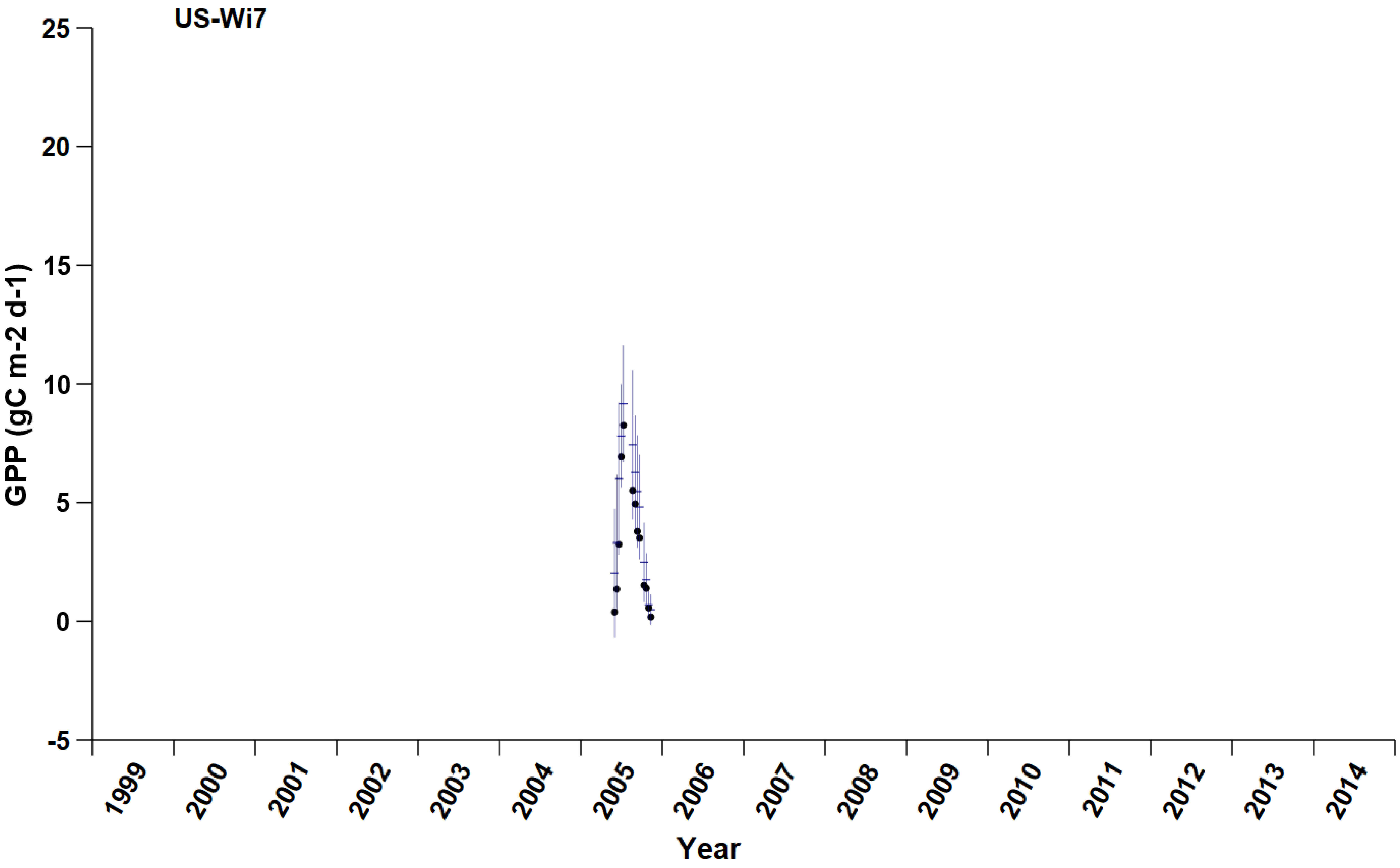
US-Wi7
GPP (gC m-2 d-1)
25
20
15
10
5
0
-5
1999
2000
2001
2002
2003
2004
2005
2006
2007
2008
2009
2010
2011
2012
2013
2014
Year

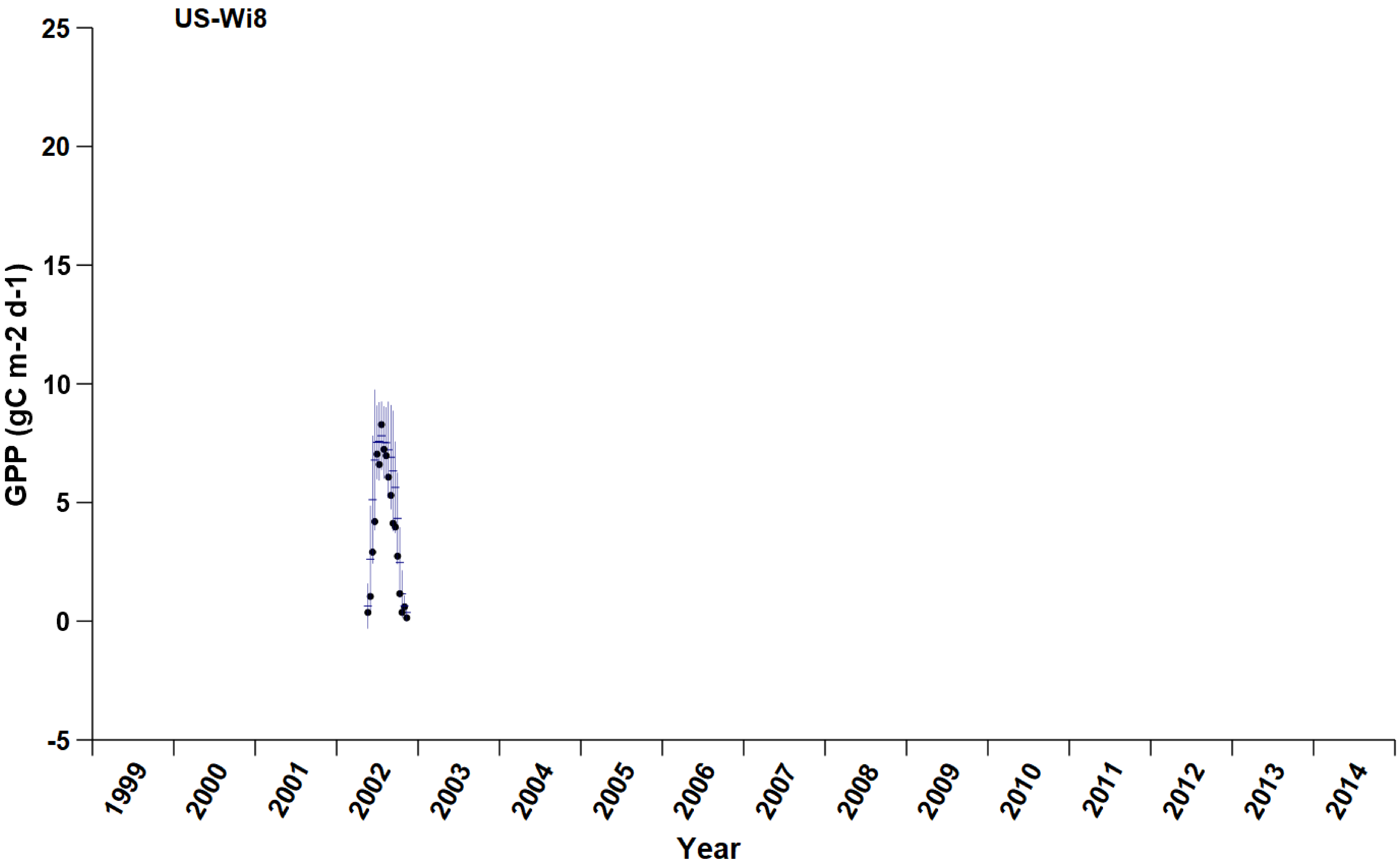

US-Wi8
GPP (gC m-2 d-1)
25
20
15
10
5
0
-5
1999
2000
2001
2002
2003
2004
2005
2006
2007
2008
2009
2010
2011
2012
2013
2014
Year

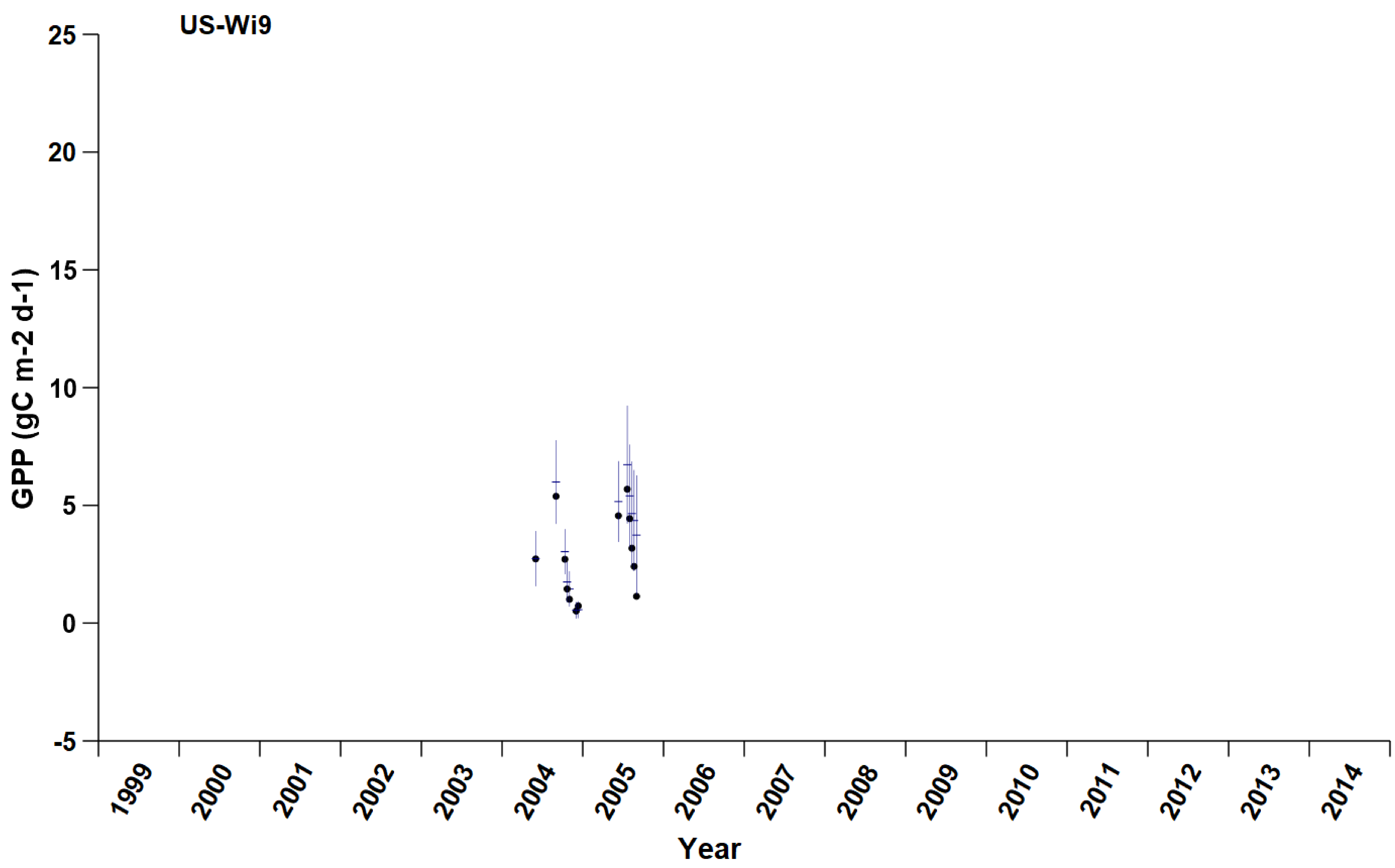
US-Wi9
GPP (gC m-2 d-1)
25
20
15
10
5
0
-5
1999
2000
2001
2002
2003
2004
2005
2006
2007
2008
2009
2010
2011
2012
2013
2014
Year

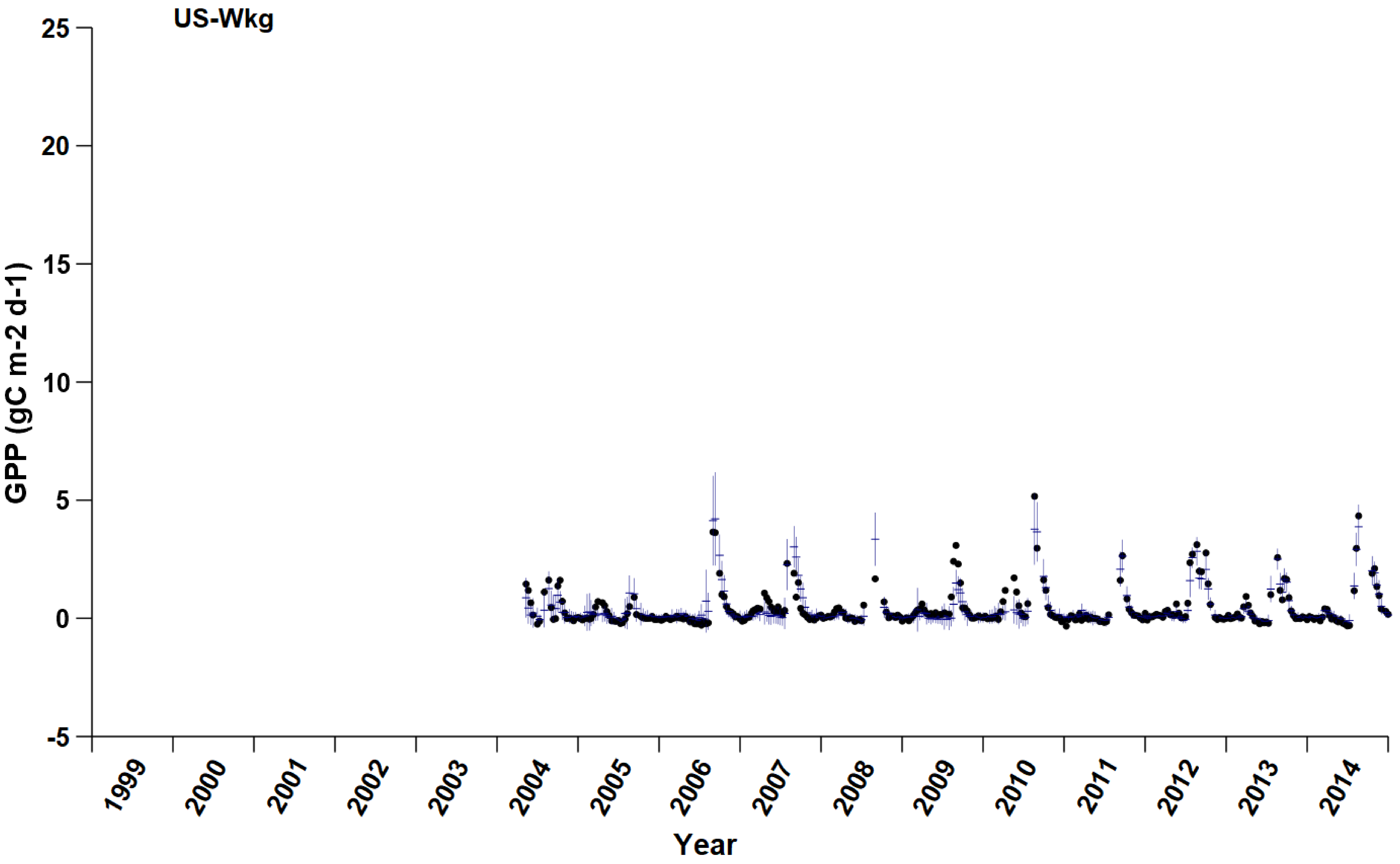
US-Wkg
GPP (gC m-2 d-1)
25
20
15
10
5
0
-5
1999
2000
2001
2002
2003
2004
2005
2006
2007
2008
2009
2010
2011
2012
2013
2014
Year

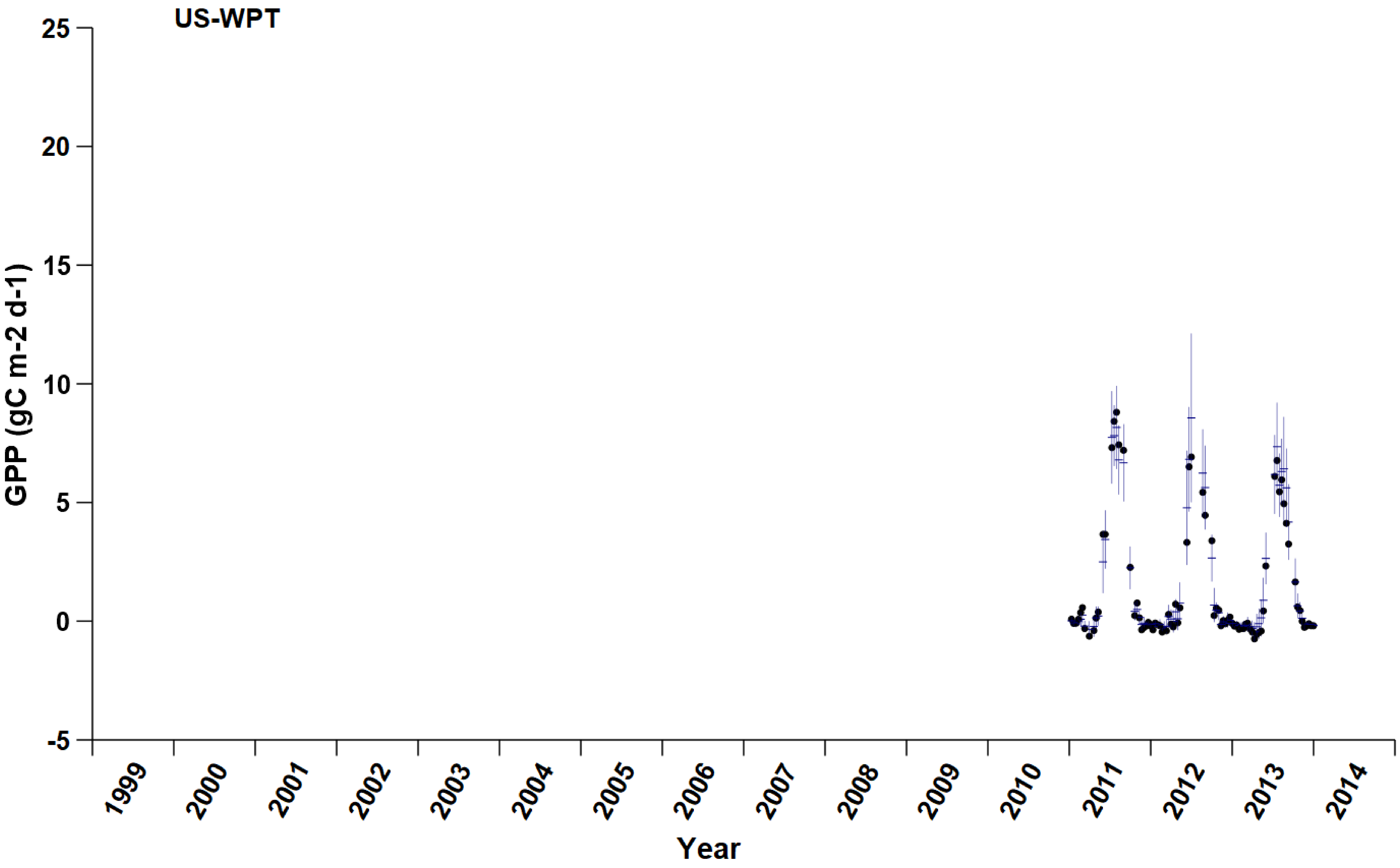

US-WPT
GPP (gC m-2 d-1)
25
20
15
10
5
0
-5
1999
2000
2001
2002
2003
2004
2005
2006
2007
2008
2009
2010
2011
2012
2013
2014
Year

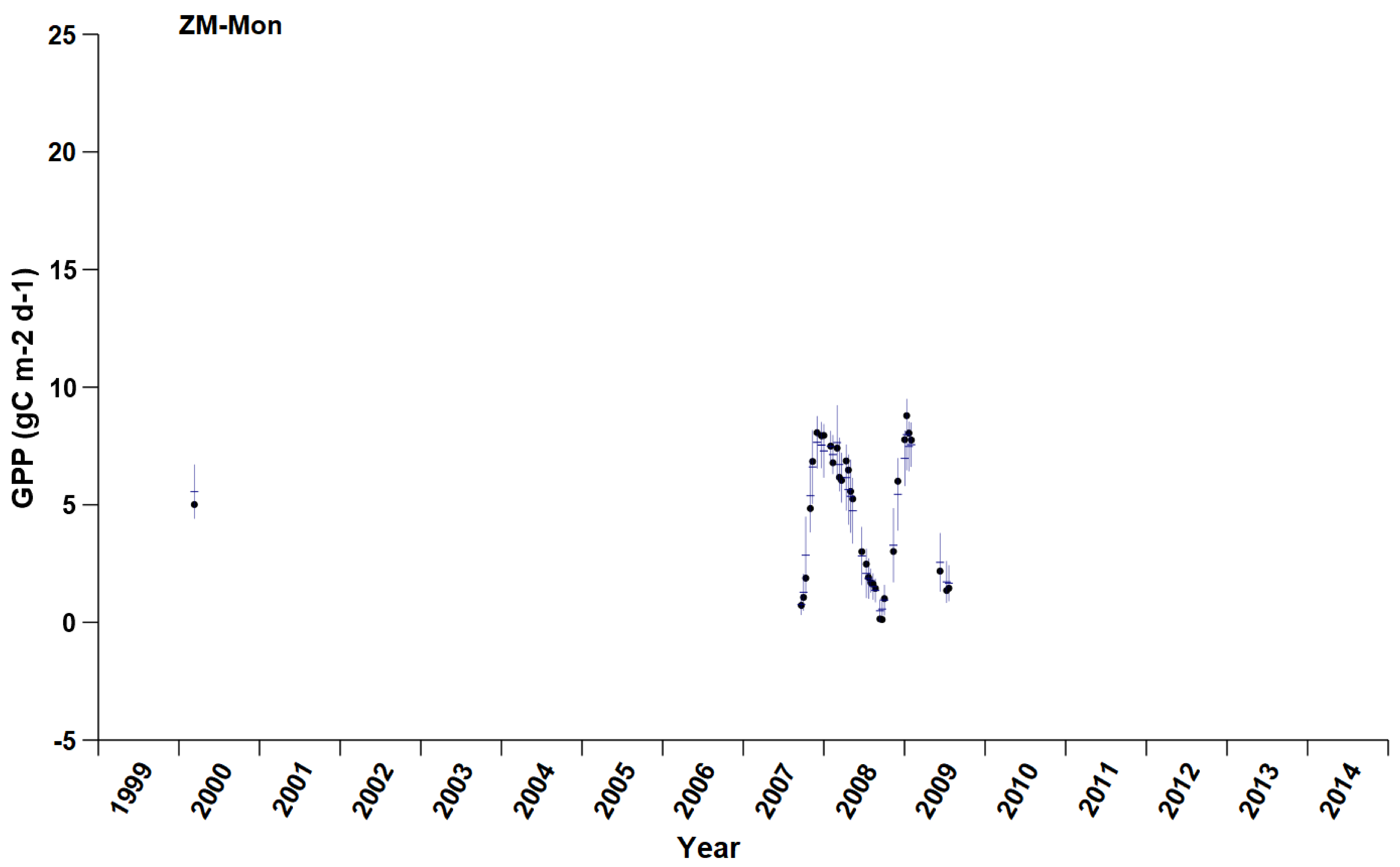
ZM-Mon
GPP (gC m-2 d-1)
25
20
15
10
5
0
-5
1999
2000
2001
2002
2003
2004
2005
2006
2007
2008
2009
2010
2011
2012
2013
2014
Year
