# Supplementary material for: Global terrestrial carbon fluxes of 1999–2019 estimated by upscaling eddy covariance data with a random forest
Source: Sci Data. 2020 Sep 24;7:313. doi: 10.1038/s41597-020-00653-5 (PMC7518252; doi:10.1038/s41597-020-00653-5)
Supplement: Supplementary file 1 — Supplementary information [file 41597_2020_653_MOESM1_ESM.zip › scidata/Supplementary File 4 (2).pdf]

RECO Time Series

This document contains plots of the time series variations of observed RECO and model predictions. Dark dots are observations. Horizontal blue bars are the means (predictions) and vertical blue lines are the standard deviations of flux values in the terminal nodes of 500 trees.

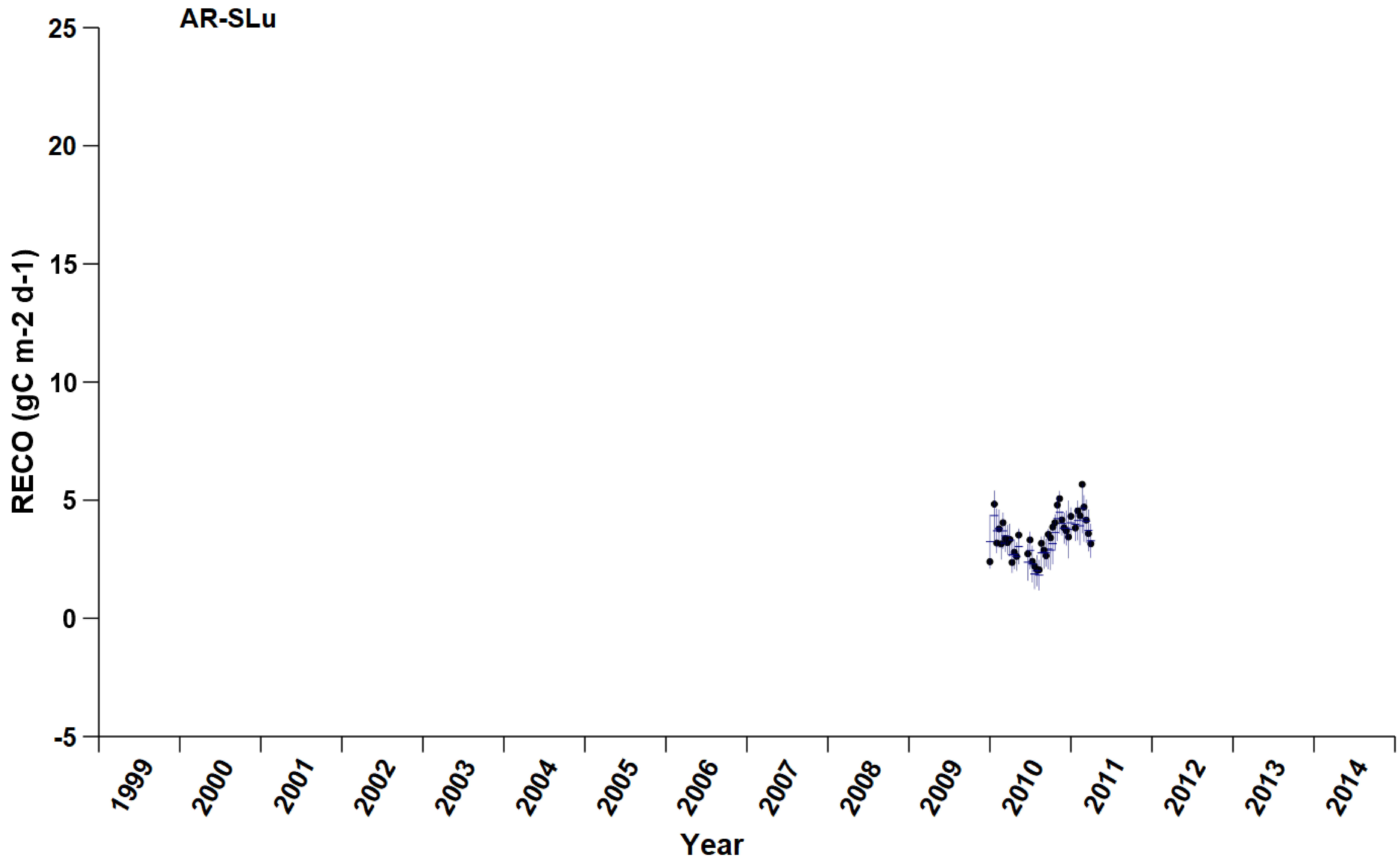
AR-SLu
RECO (gC m-2 d-1)
25
20
15
10
5
0
-5
1999
2000
2001
2002
2003
2004
2005
2006
2007
2008
2009
2010
2011
2012
2013
2014
Year

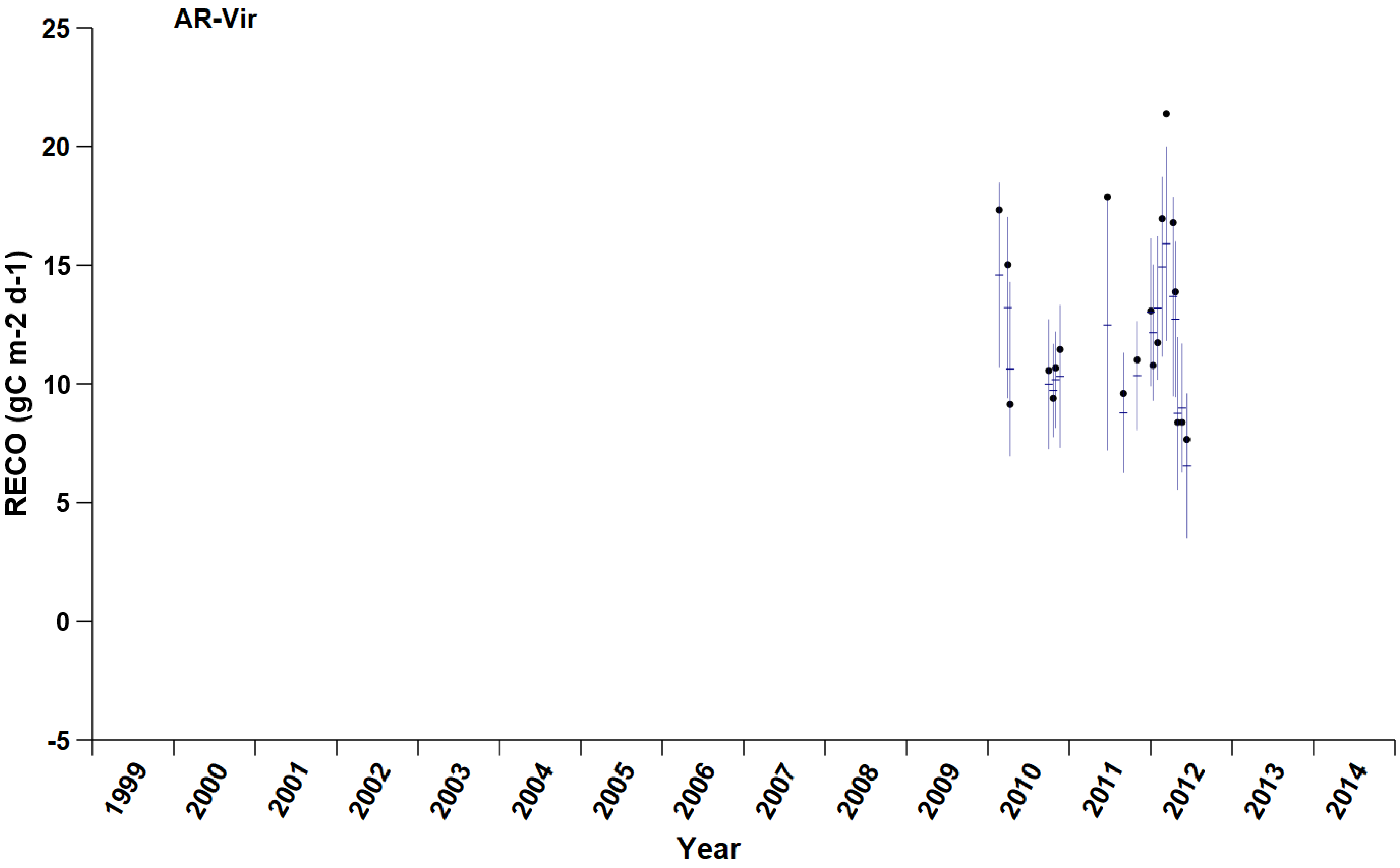
AR-Vir
RECO (gC m-2 d-1)
25
20
15
10
5
0
-5
1999
2000
2001
2002
2003
2004
2005
2006
2007
2008
2009
2010
2011
2012
2013
2014
Year

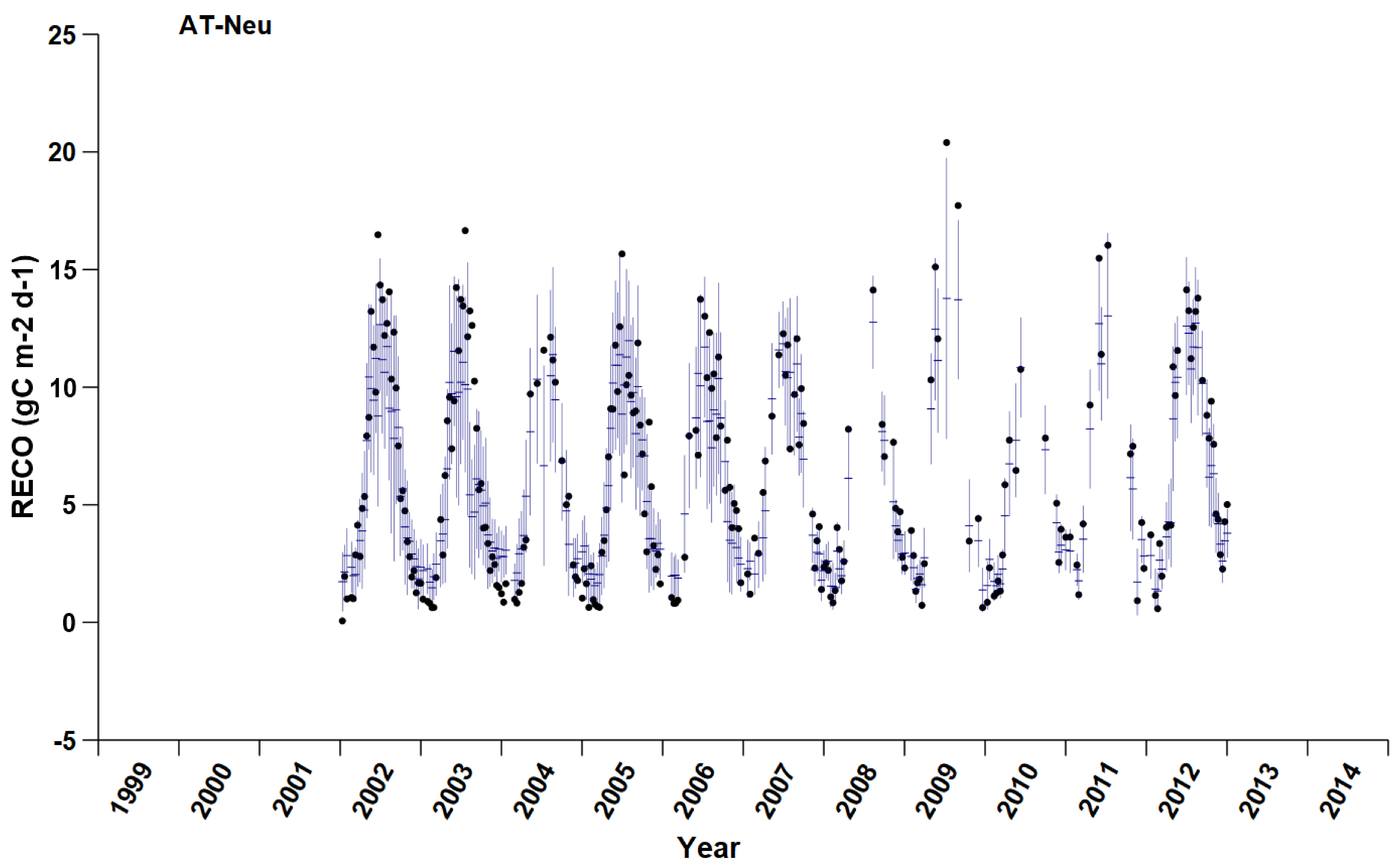
AT-Neu
RECO (gC m-2 d-1)
25
20
15
10
5
0
-5
1999
2000
2001
2002
2003
2004
2005
2006
2007
2008
2009
2010
2011
2012
2013
2014
Year

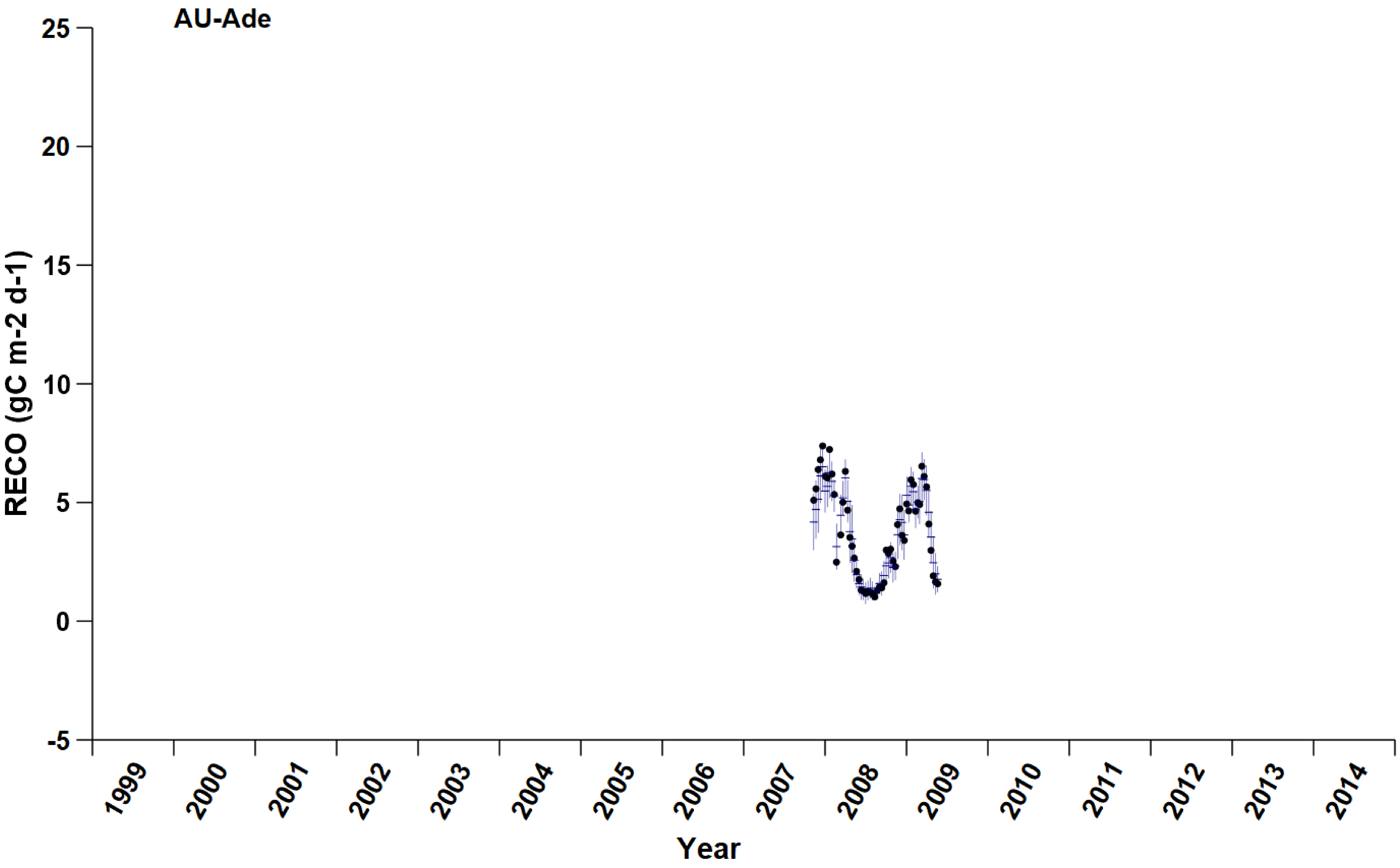
AU-Ade
RECO (gC m-2 d-1)
25
20
15
10
5
0
-5
1999
2000
2001
2002
2003
2004
2005
2006
2007
2008
2009
2010
2011
2012
2013
2014
Year

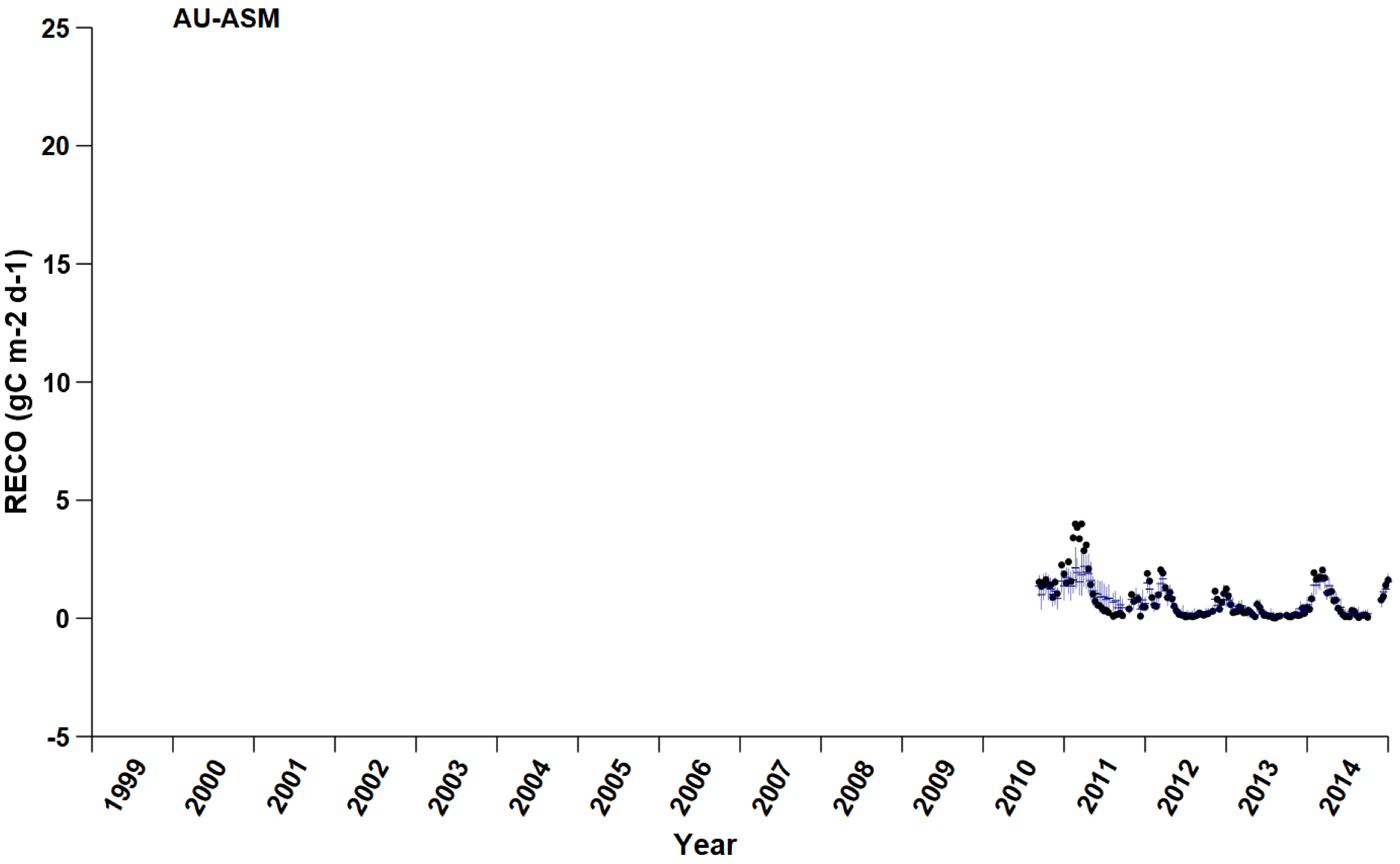

AU-ASM
RECO (gC m-2 d-1)
25
20
15
10
5
0
-5
1999
2000
2001
2002
2003
2004
2005
2006
2007
2008
2009
2010
2011
2012
2013
2014
Year

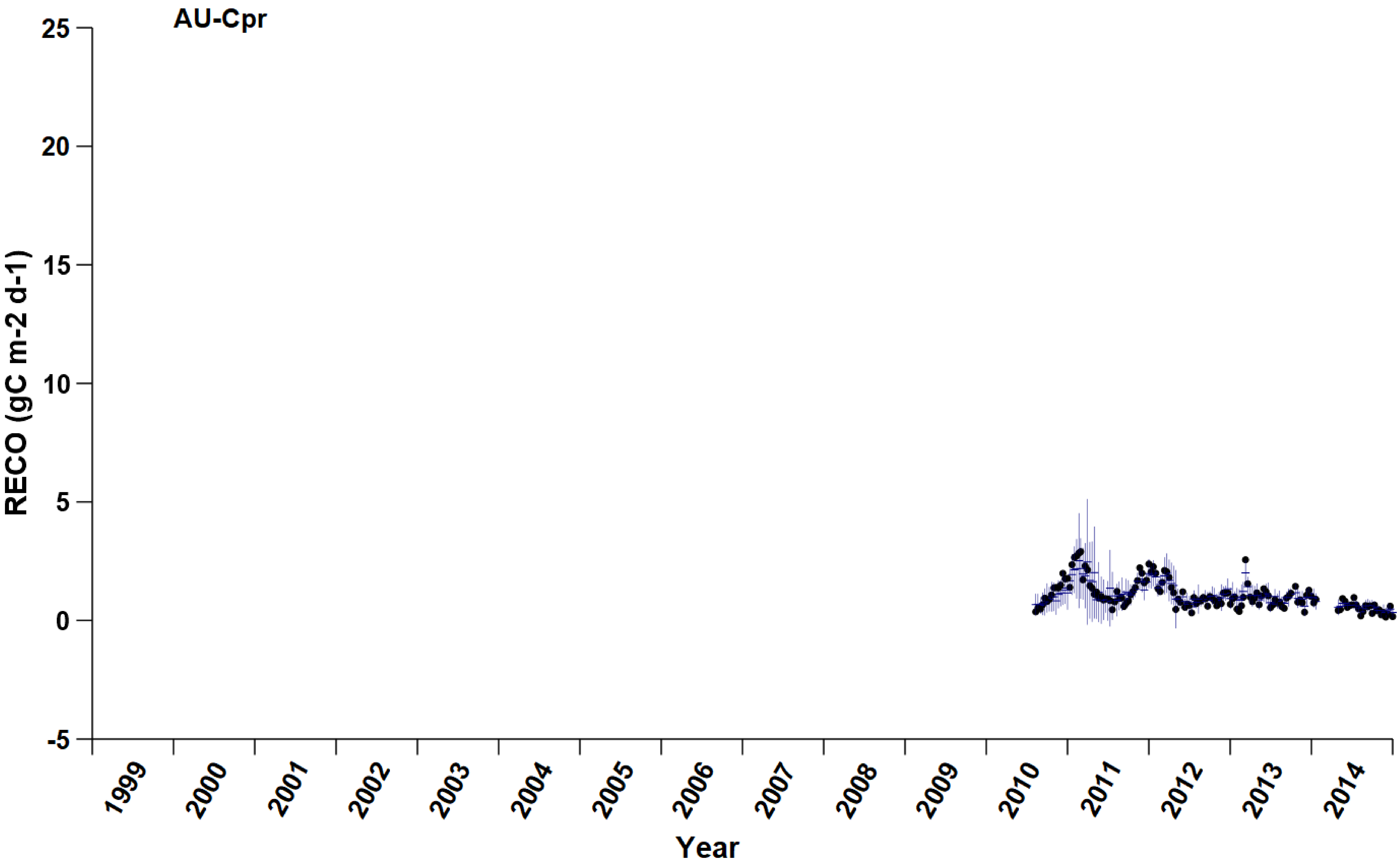

AU-Cpr
RECO (gC m-2 d-1)
25
20
15
10
5
0
-5
1999
2000
2001
2002
2003
2004
2005
2006
2007
2008
2009
2010
2011
2012
2013
2014
Year

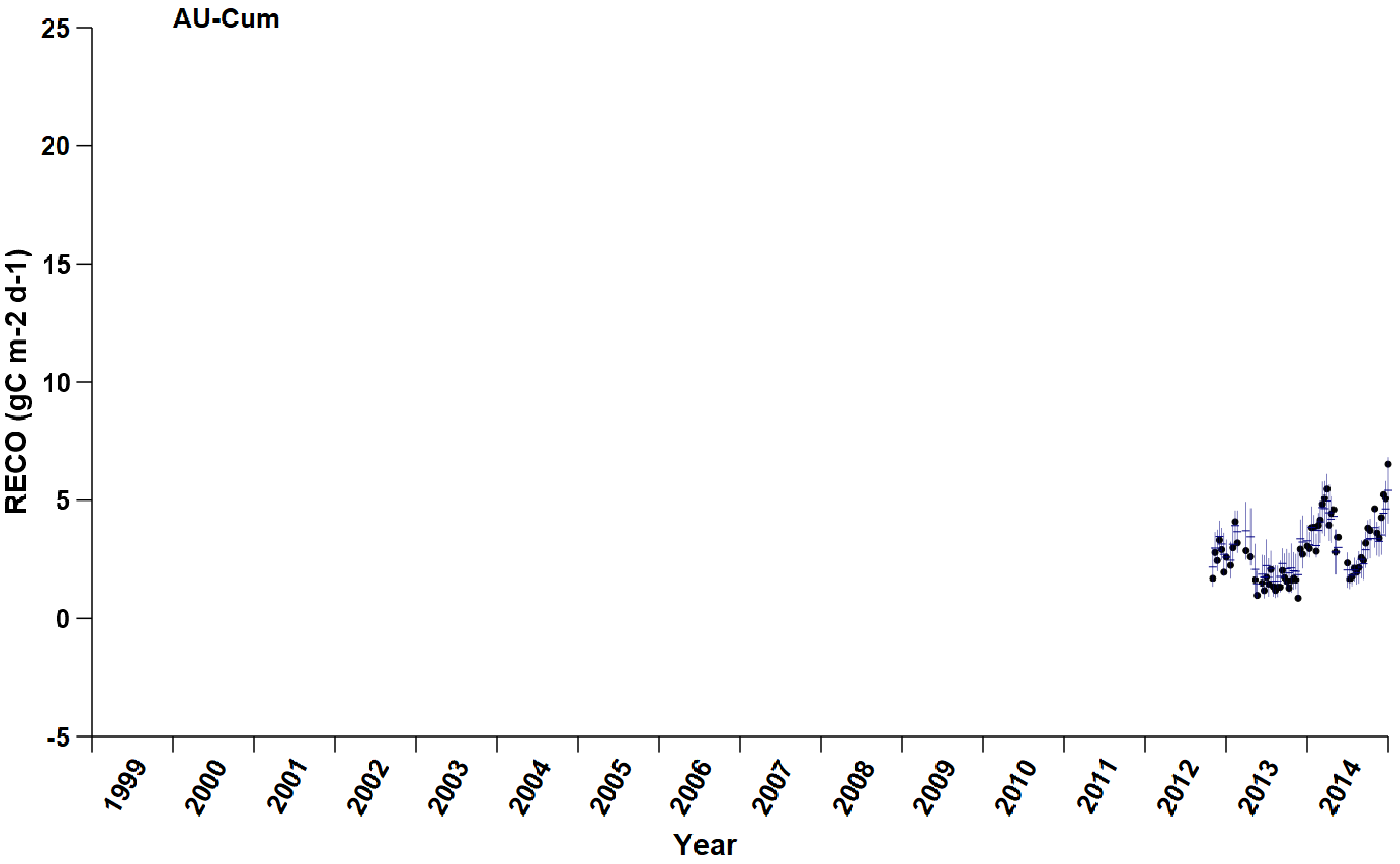

AU-Cum
RECO (gC m-2 d-1)
25
20
15
10
5
0
-5
1999
2000
2001
2002
2003
2004
2005
2006
2007
2008
2009
2010
2011
2012
2013
2014
Year

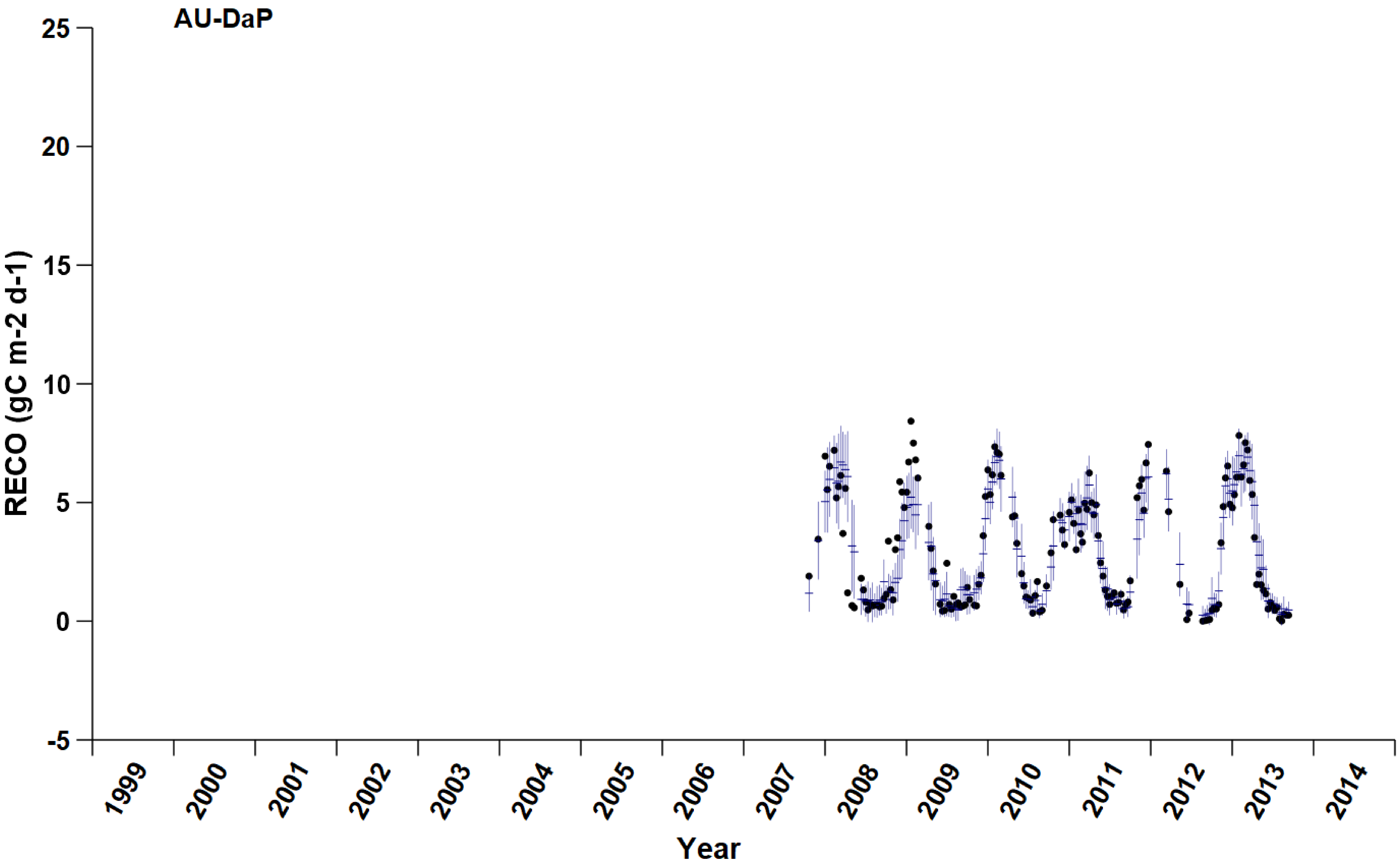

AU-DaP
RECO (gC m-2 d-1)
25
20
15
10
5
0
-5
1999
2000
2001
2002
2003
2004
2005
2006
2007
2008
2009
2010
2011
2012
2013
2014
Year

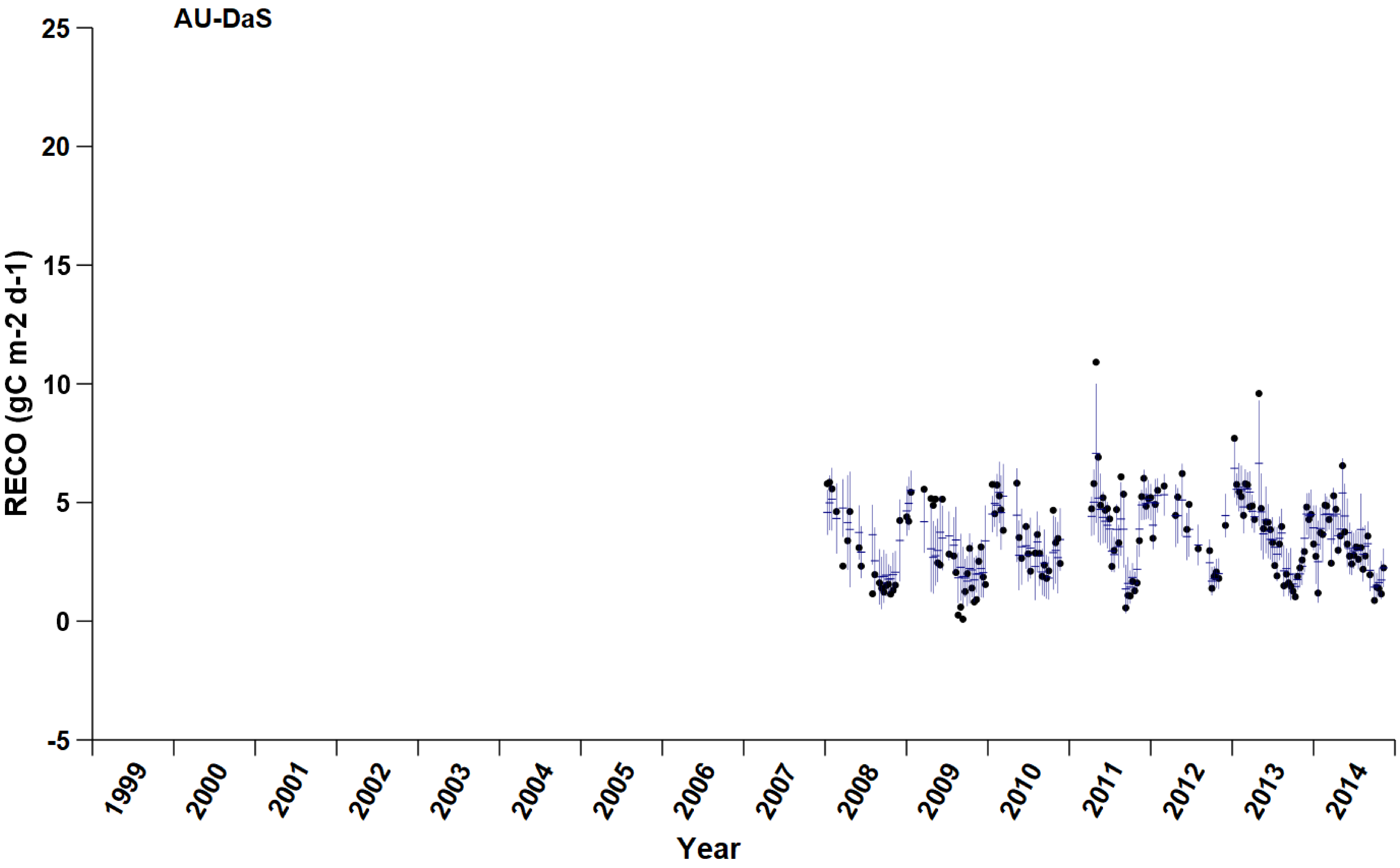
AU-DaS
RECO (gC m-2 d-1)
25
20
15
10
5
0
-5
1999
2000
2001
2002
2003
2004
2005
2006
2007
2008
2009
2010
2011
2012
2013
2014
Year

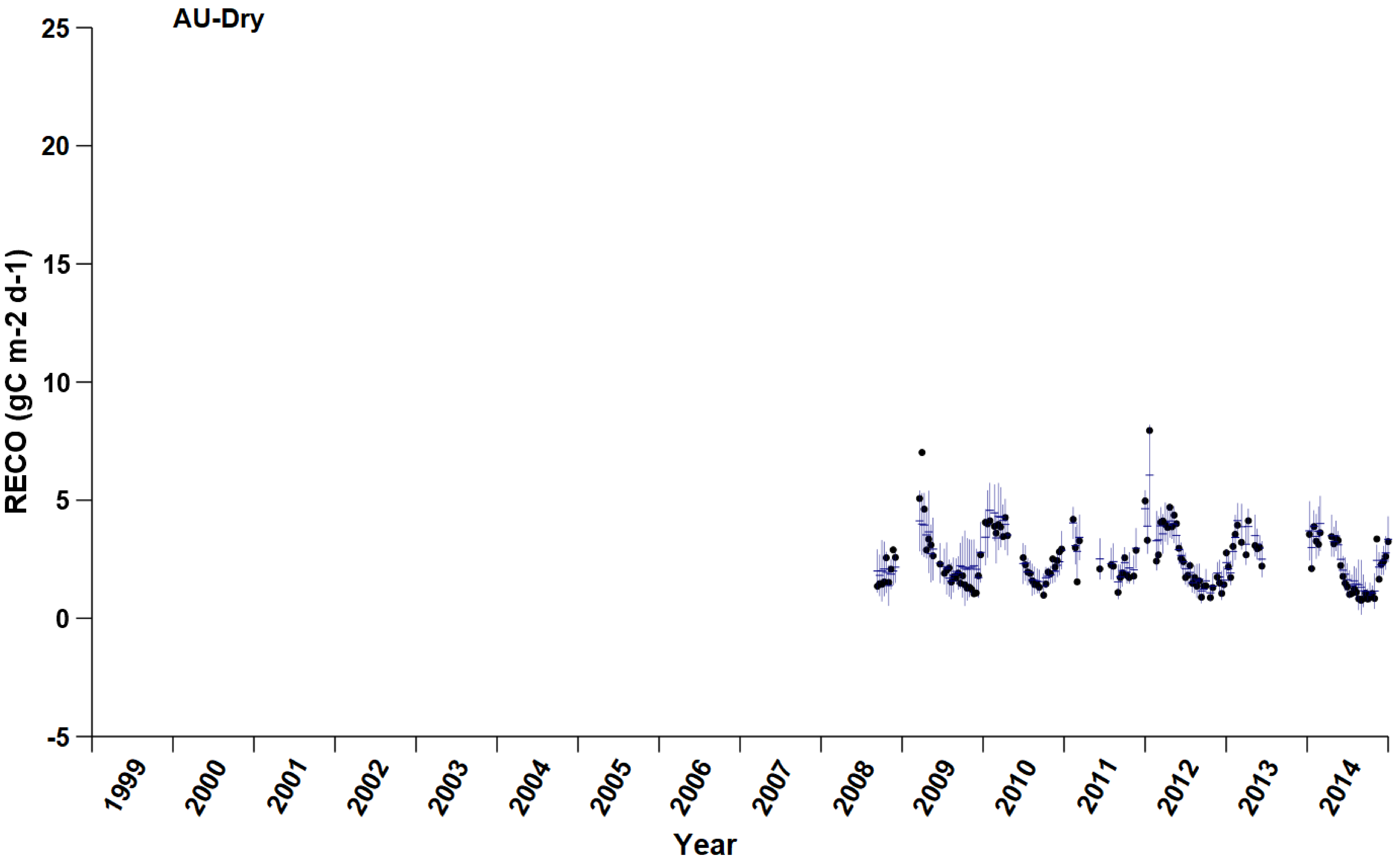

AU-Dry
RECO (gC m-2 d-1)
25
20
15
10
5
0
-5
1999
2000
2001
2002
2003
2004
2005
2006
2007
2008
2009
2010
2011
2012
2013
2014
Year

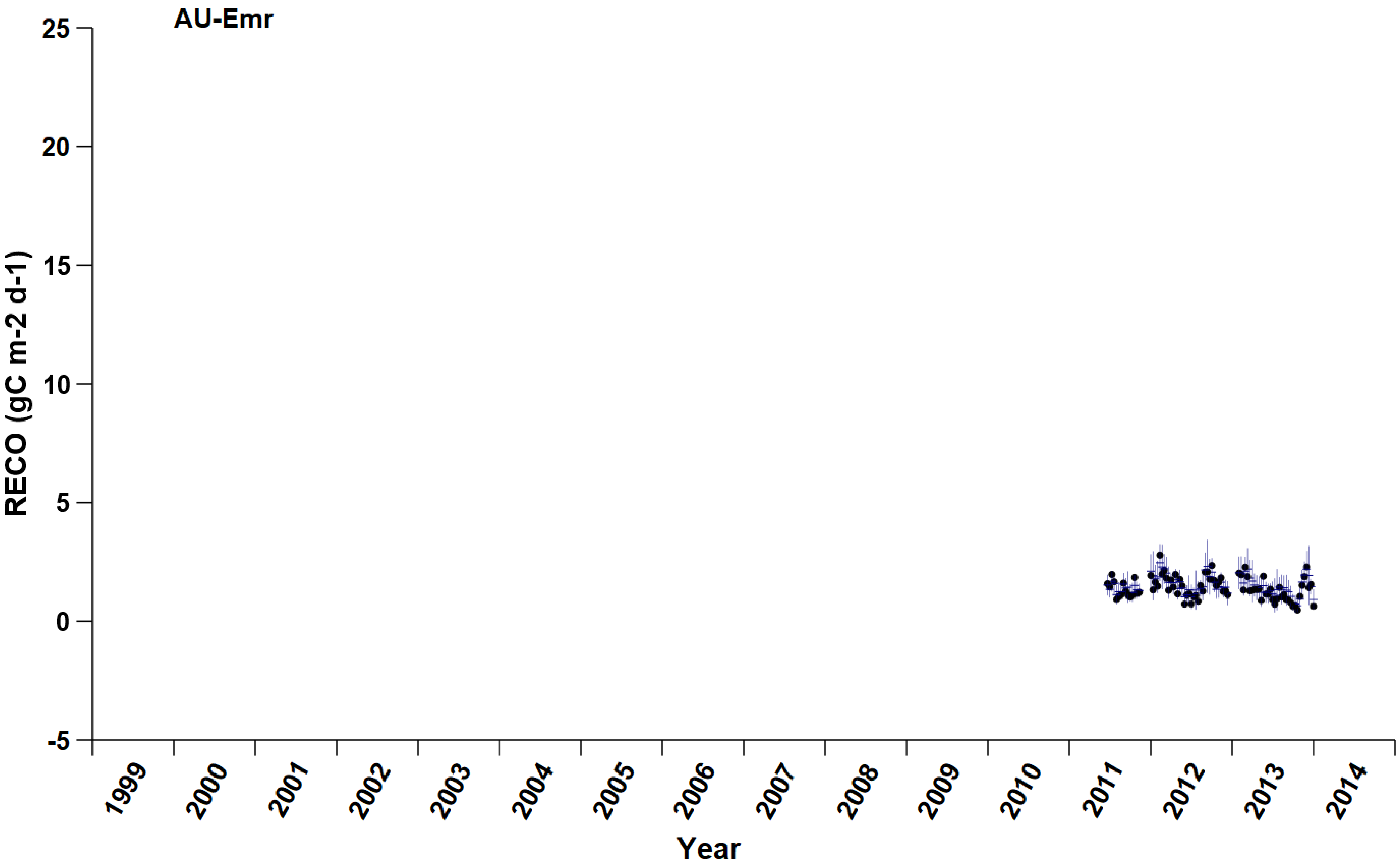
AU-Emr
RECO (gC m-2 d-1)
25
20
15
10
5
0
-5
1999
2000
2001
2002
2003
2004
2005
2006
2007
2008
2009
2010
2011
2012
2013
2014
Year

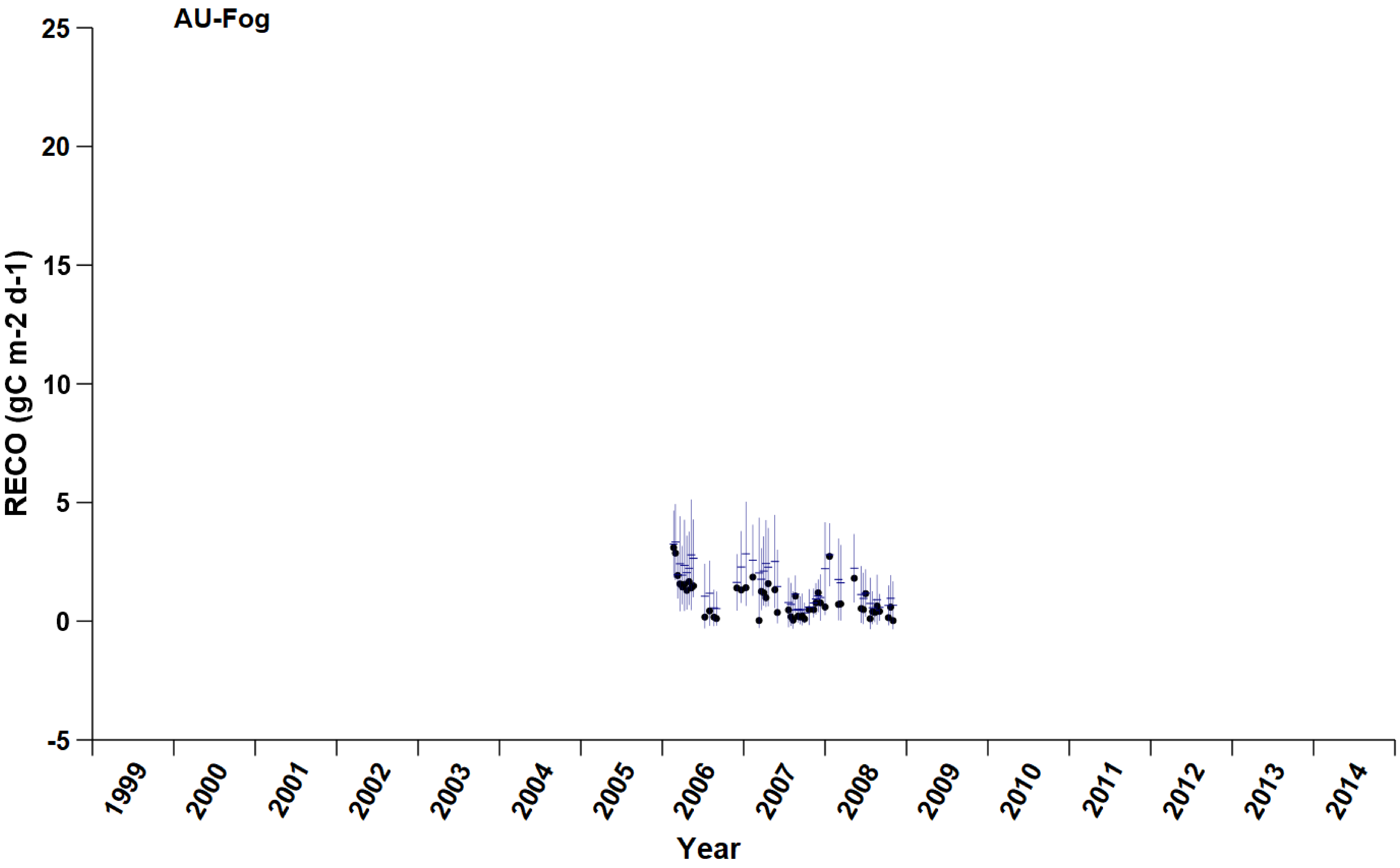
AU-Fog
RECO (gC m-2 d-1)
25
20
15
10
5
0
-5
1999
2000
2001
2002
2003
2004
2005
2006
2007
2008
2009
2010
2011
2012
2013
2014
Year

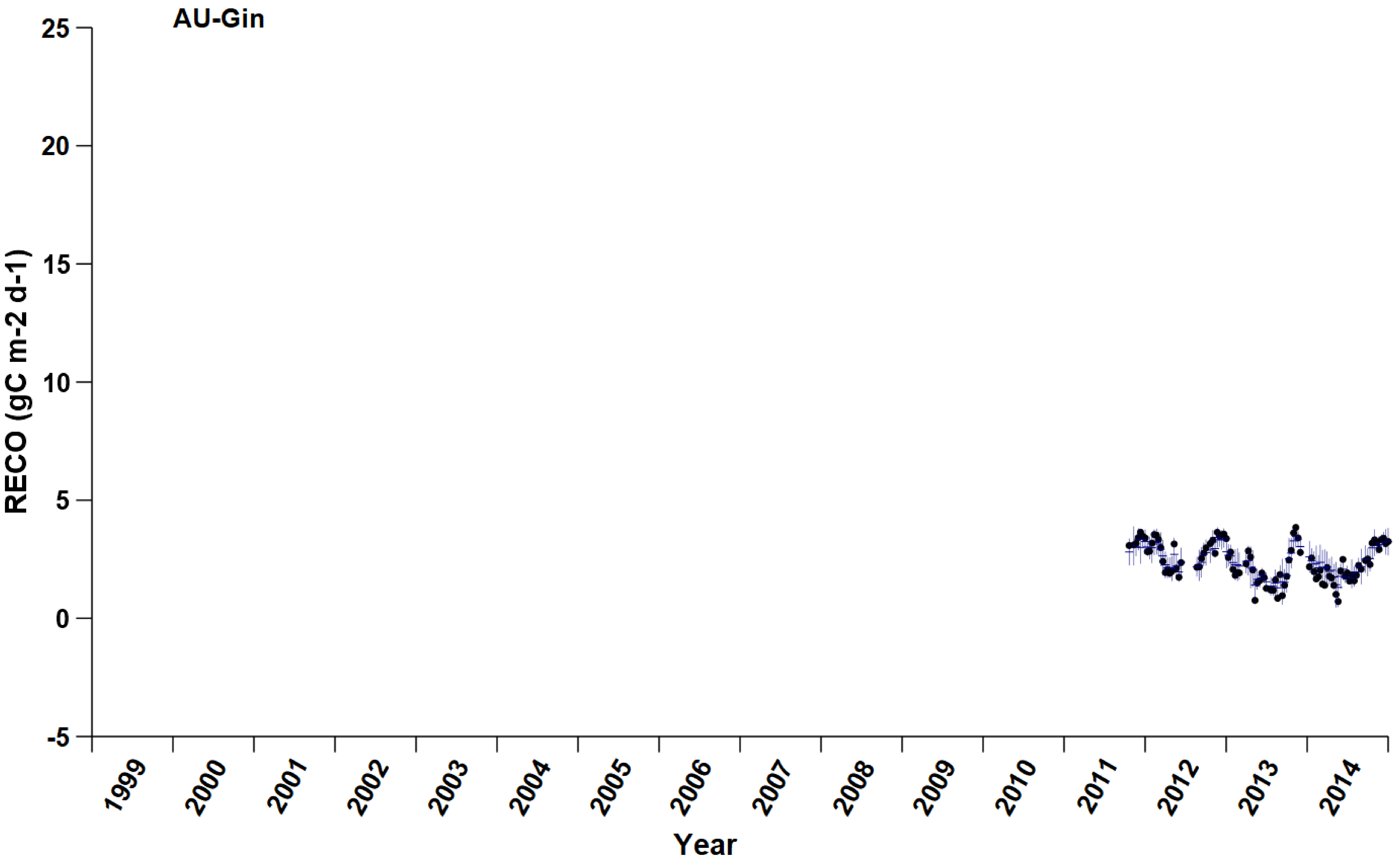
AU-Gin
RECO (gC m-2 d-1)
25
20
15
10
5
0
-5
1999
2000
2001
2002
2003
2004
2005
2006
2007
2008
2009
2010
2011
2012
2013
2014
Year

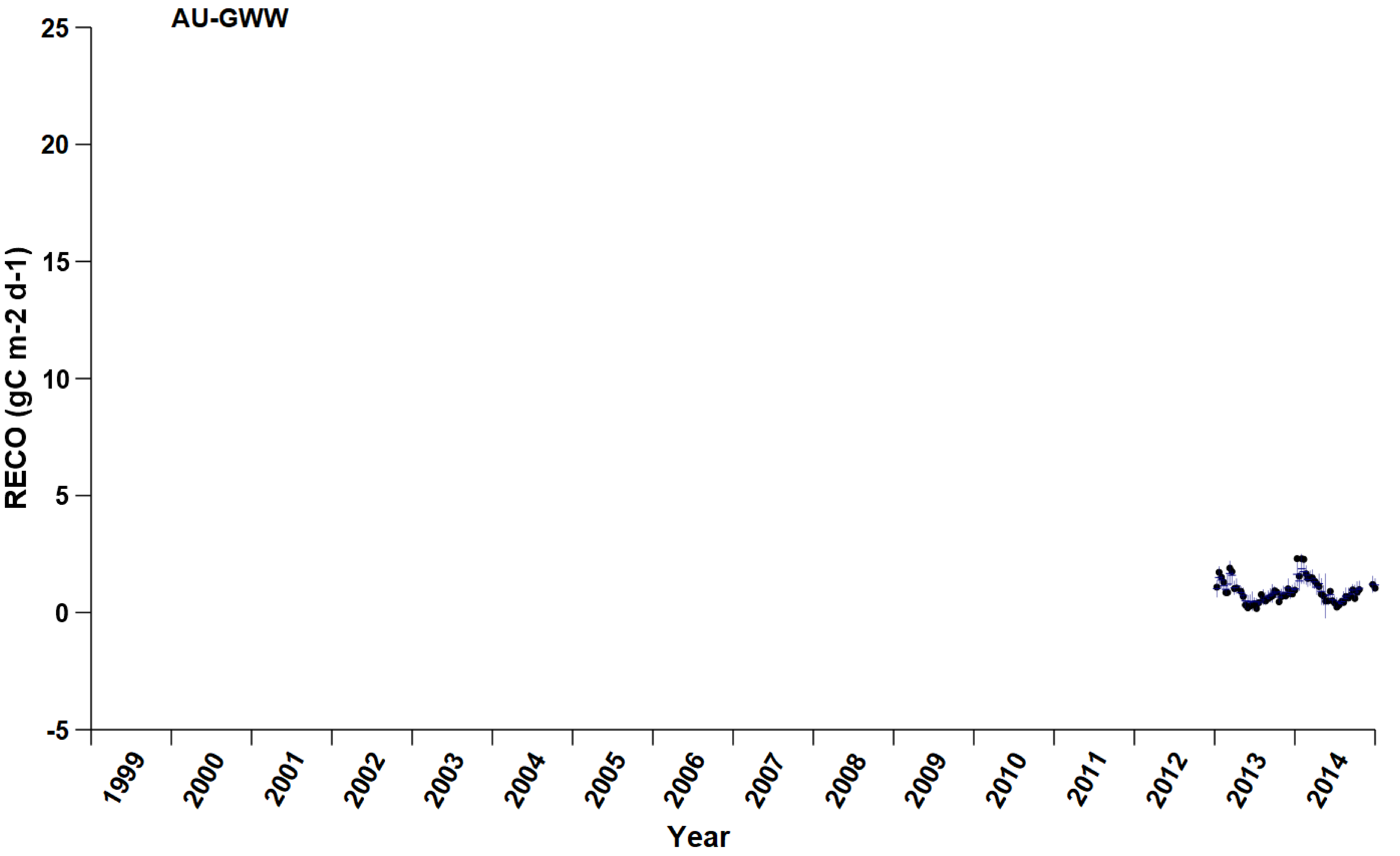
AU-GWW
RECO (gC m-2 d-1)
25
20
15
10
5
0
-5
1999
2000
2001
2002
2003
2004
2005
2006
2007
2008
2009
2010
2011
2012
2013
2014
Year

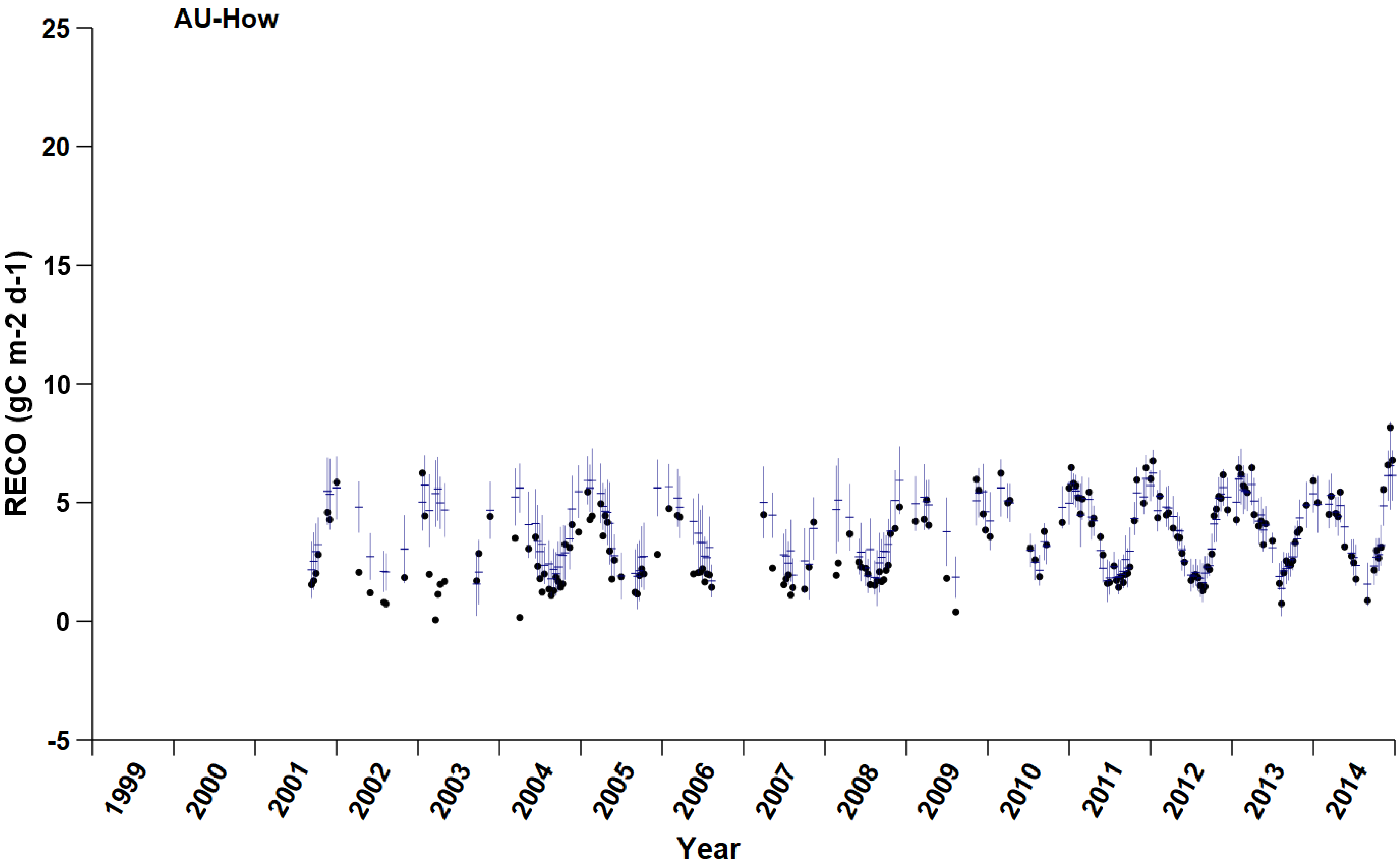

AU-How
RECO (gC m-2 d-1)
25
20
15
10
5
0
-5
1999
2000
2001
2002
2003
2004
2005
2006
2007
2008
2009
2010
2011
2012
2013
2014
Year

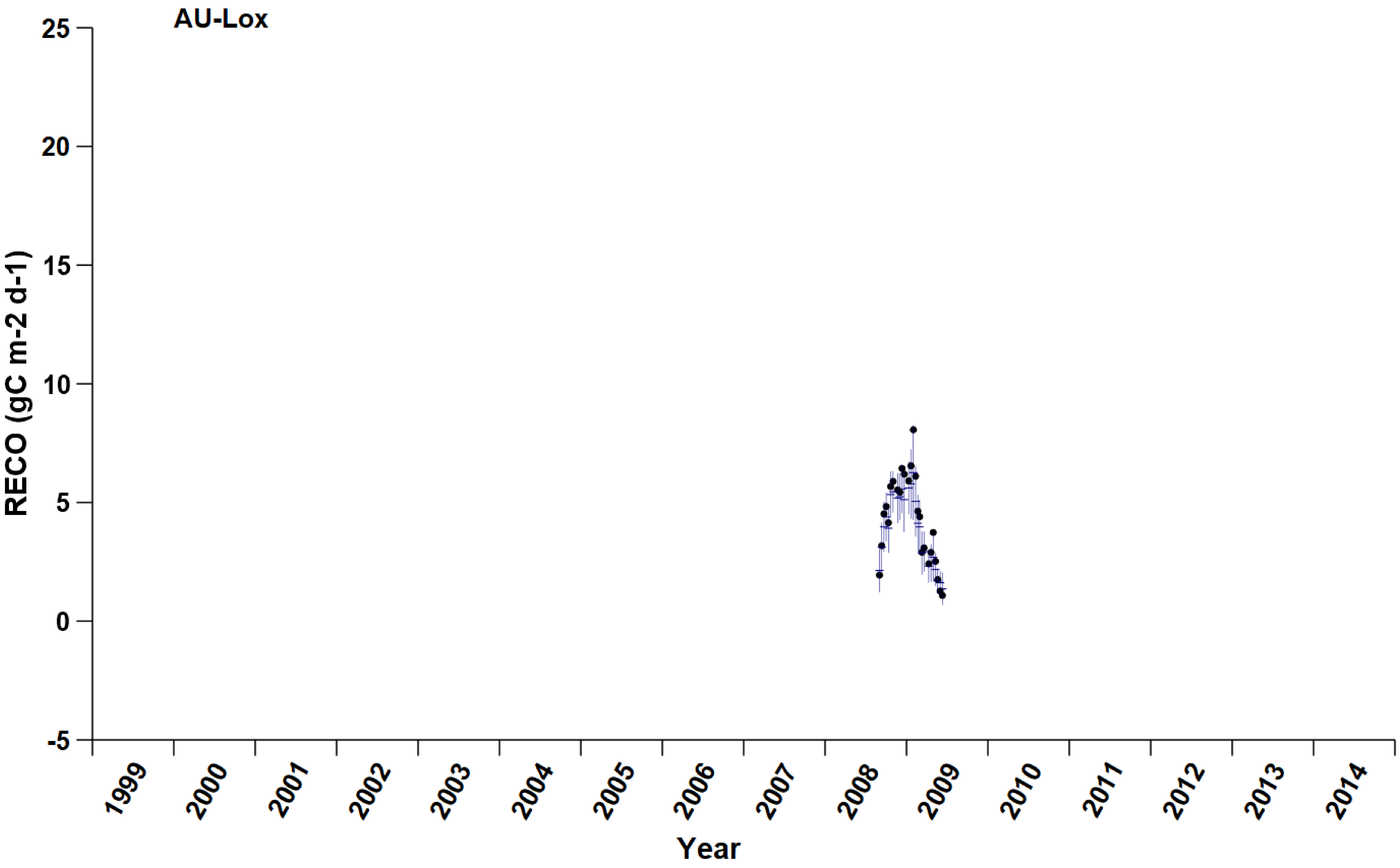

AU-Lox
RECO (gC m-2 d-1)
25
20
15
10
5
0
-5
1999
2000
2001
2002
2003
2004
2005
2006
2007
2008
2009
2010
2011
2012
2013
2014
Year

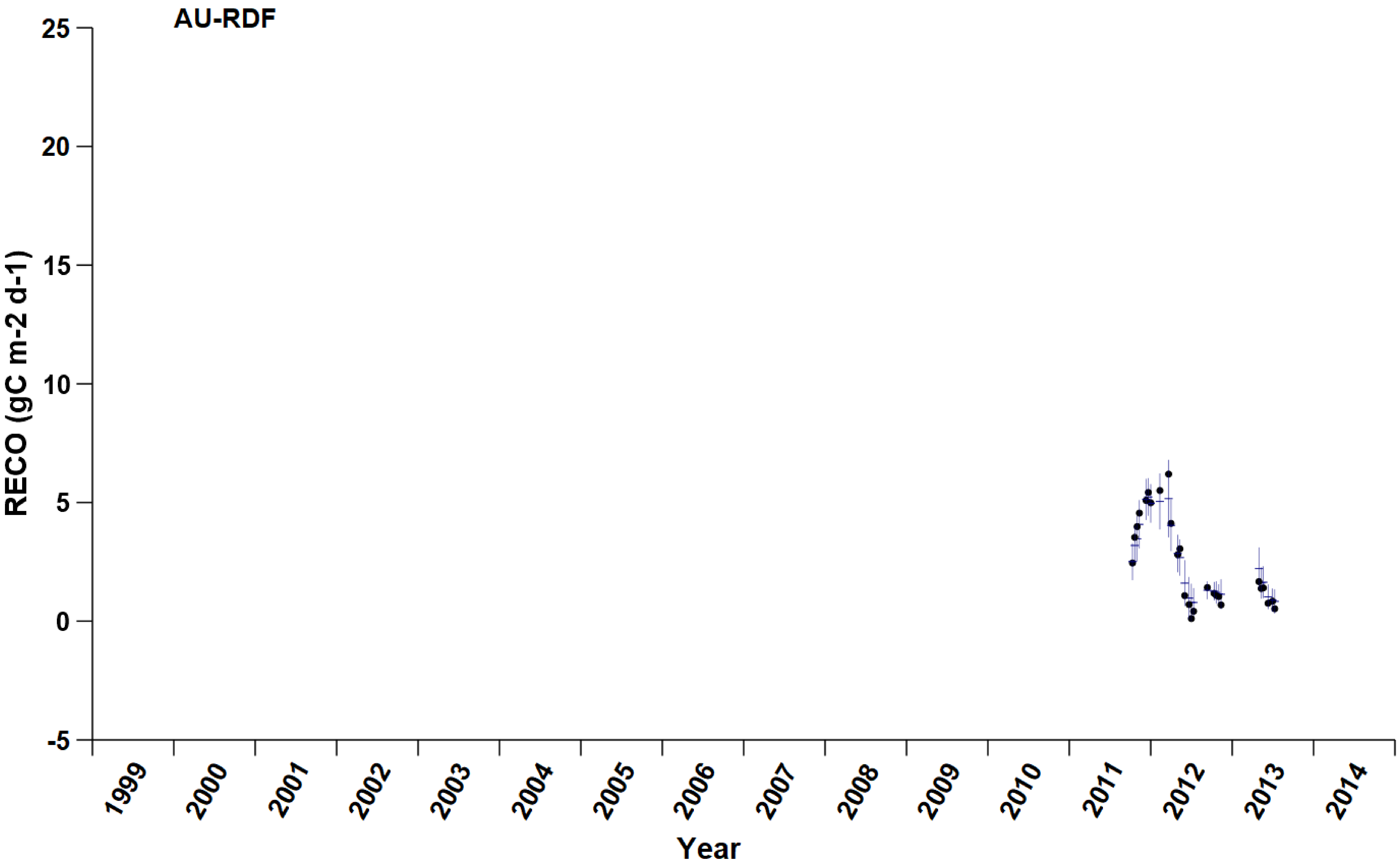

AU-RDF
RECO (gC m-2 d-1)
25
20
15
10
5
0
-5
1999
2000
2001
2002
2003
2004
2005
2006
2007
2008
2009
2010
2011
2012
2013
2014
Year

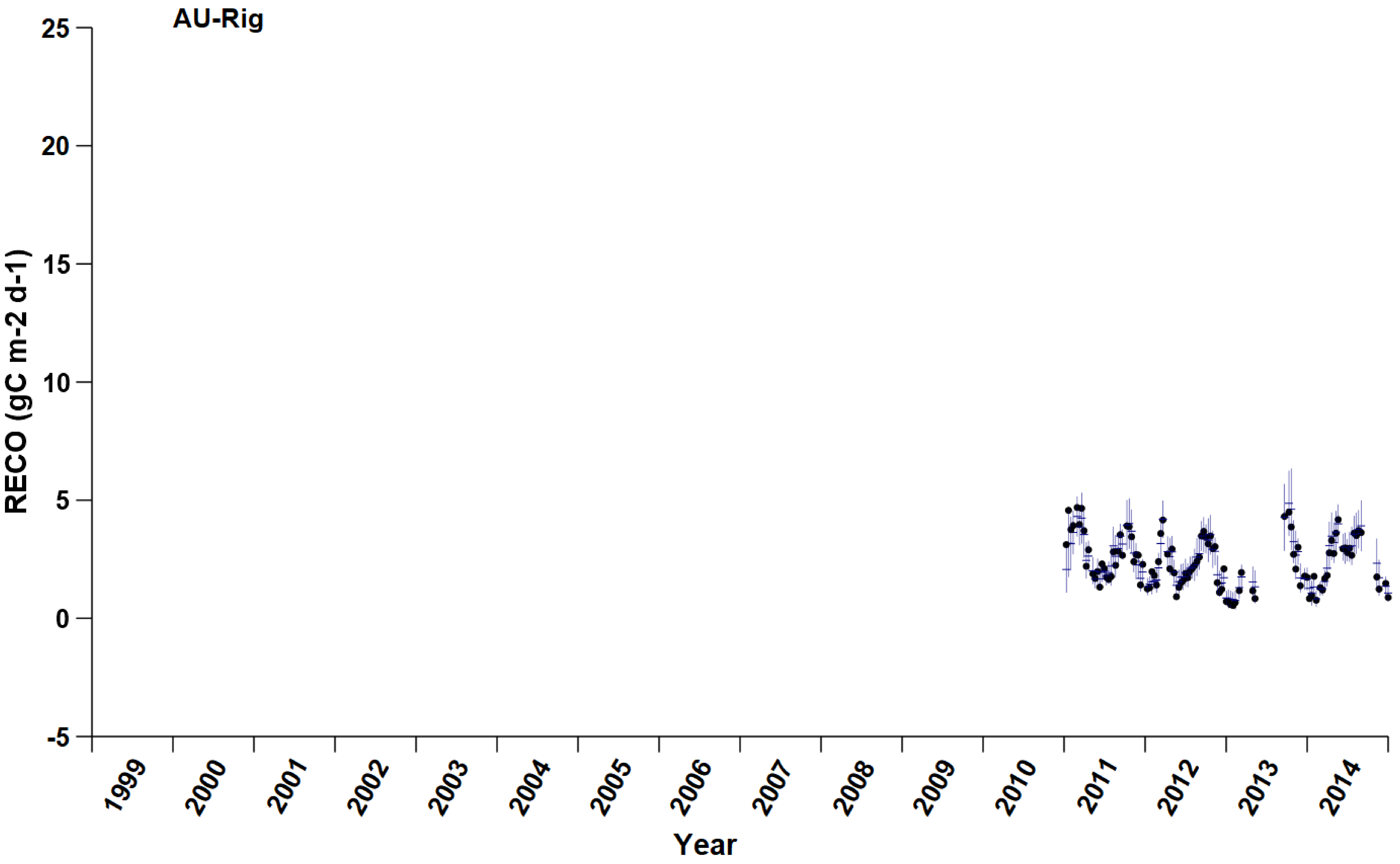
AU-Rig
RECO (gC m-2 d-1)
25
20
15
10
5
0
-5
1999
2000
2001
2002
2003
2004
2005
2006
2007
2008
2009
2010
2011
2012
2013
2014
Year

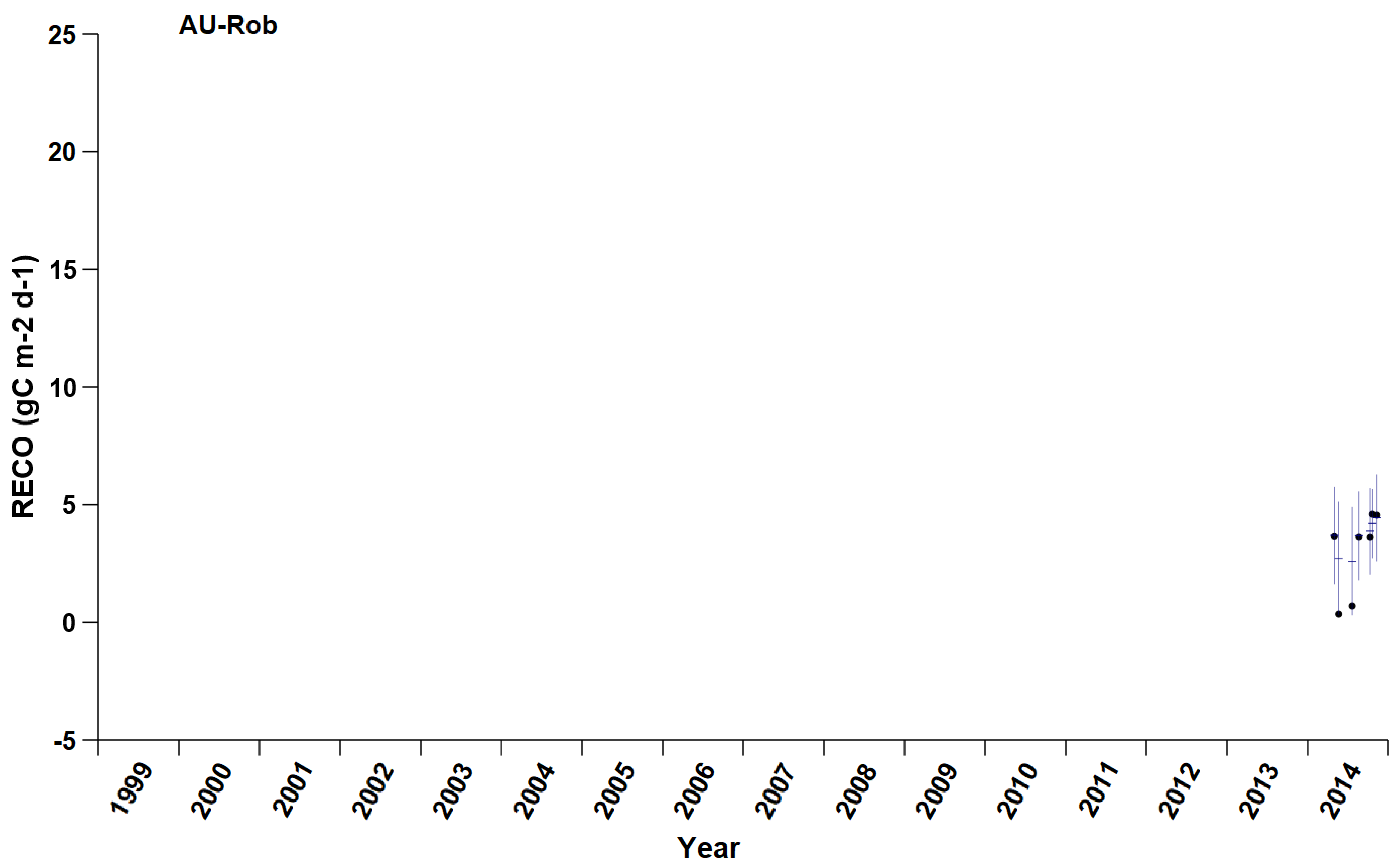
AU-Rob
RECO (gC m-2 d-1)
25
20
15
10
5
0
-5
1999
2000
2001
2002
2003
2004
2005
2006
2007
2008
2009
2010
2011
2012
2013
2014
Year

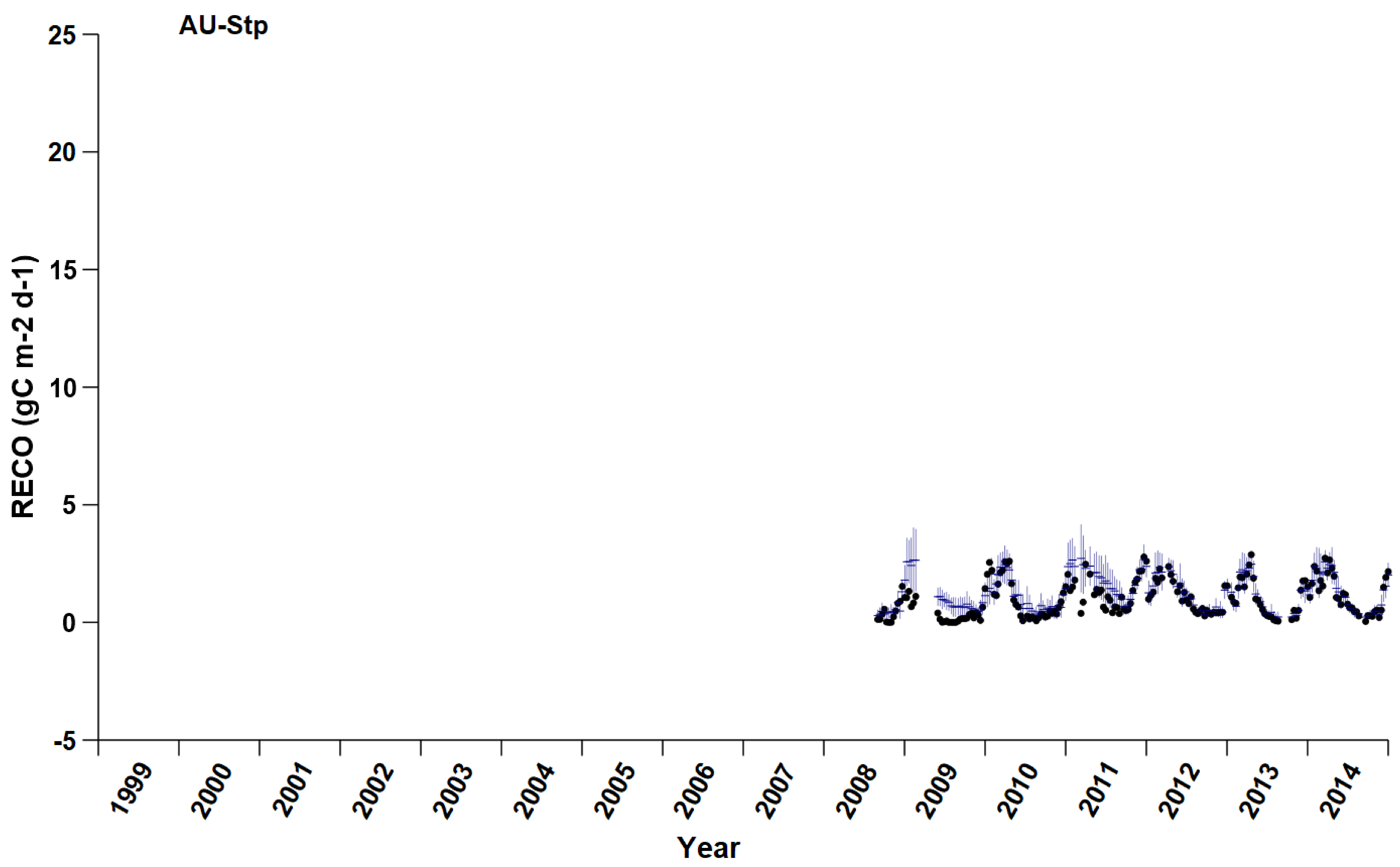

AU-Stp
RECO (gC m-2 d-1)
25
20
15
10
5
0
-5
1999
2000
2001
2002
2003
2004
2005
2006
2007
2008
2009
2010
2011
2012
2013
2014
Year

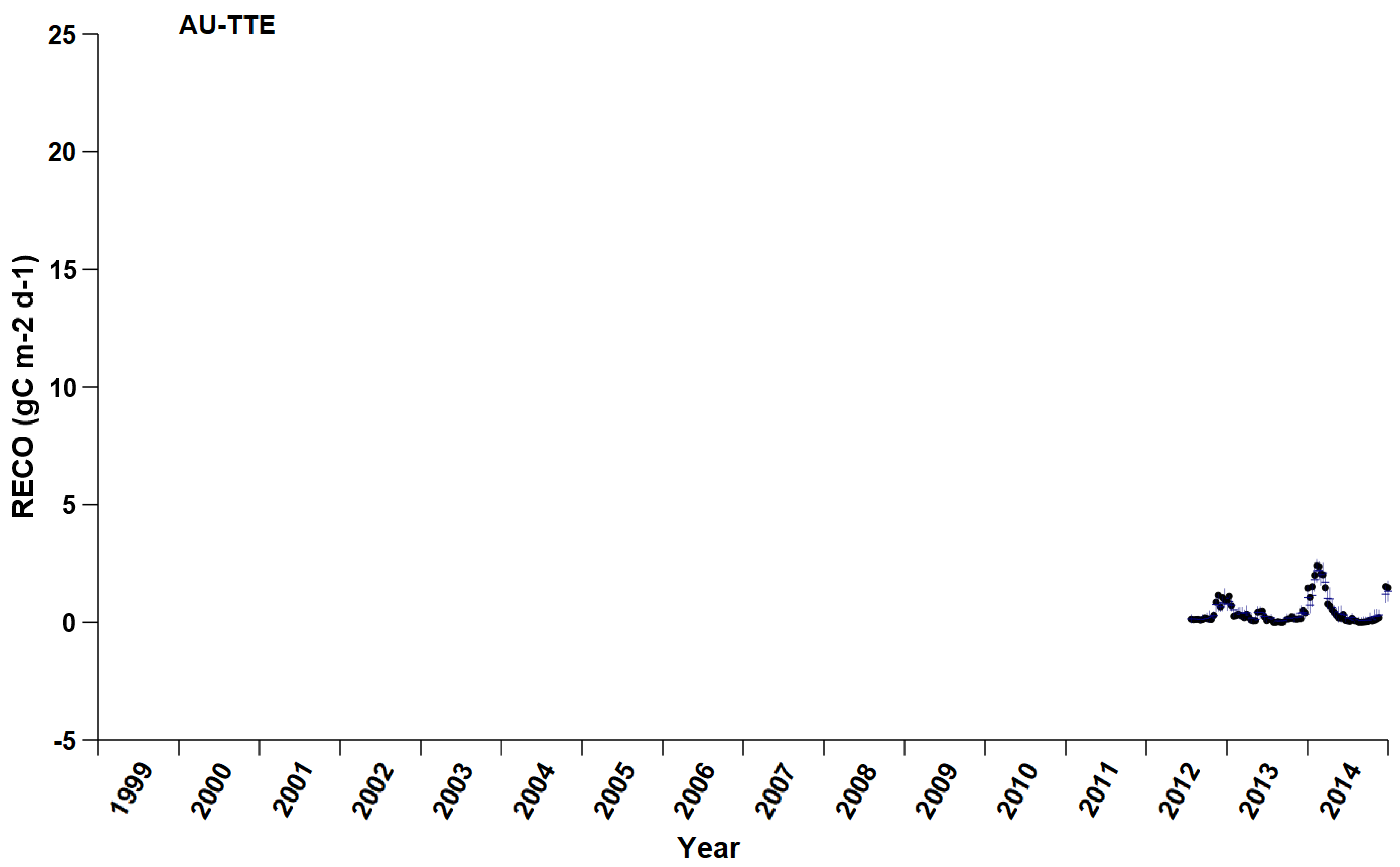
AU-TTE
RECO (gC m-2 d-1)
25
20
15
10
5
0
-5
1999
2000
2001
2002
2003
2004
2005
2006
2007
2008
2009
2010
2011
2012
2013
2014
Year

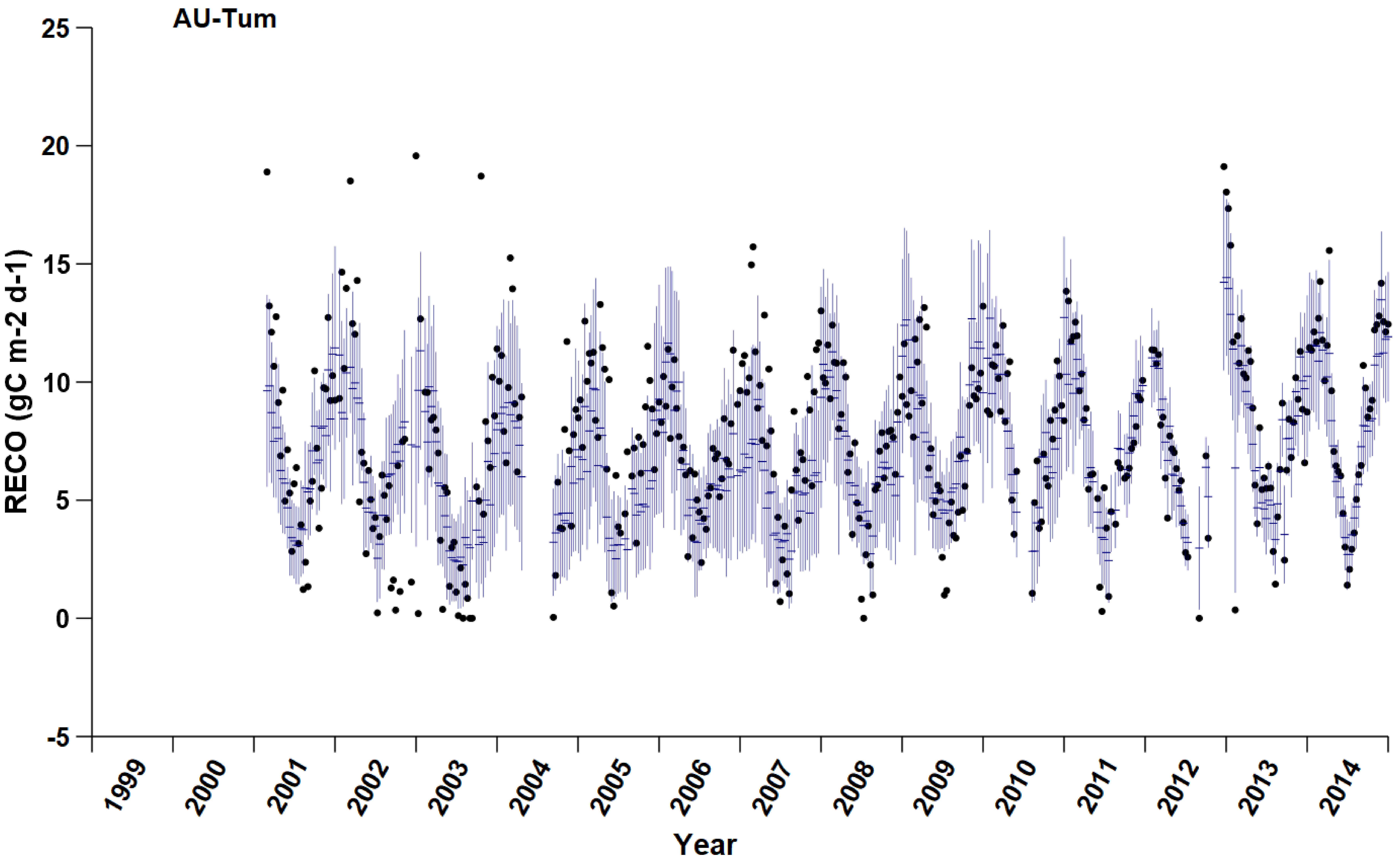

AU-Tum
RECO (gC m-2 d-1)
25
20
15
10
5
0
-5
1999
2000
2001
2002
2003
2004
2005
2006
2007
2008
2009
2010
2011
2012
2013
2014
Year

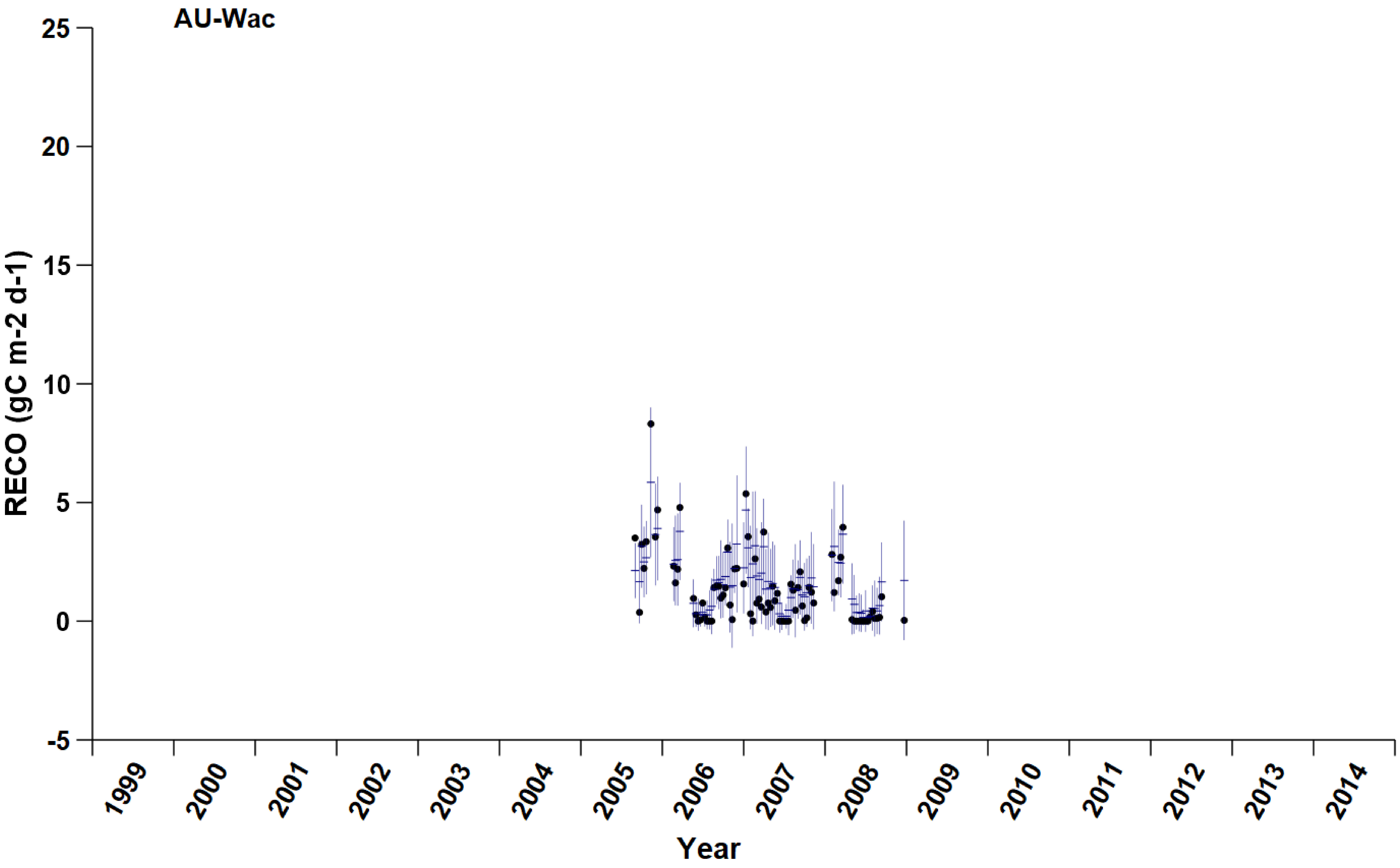
AU-Wac
RECO (gC m-2 d-1)
25
20
15
10
5
0
-5
1999
2000
2001
2002
2003
2004
2005
2006
2007
2008
2009
2010
2011
2012
2013
2014
Year

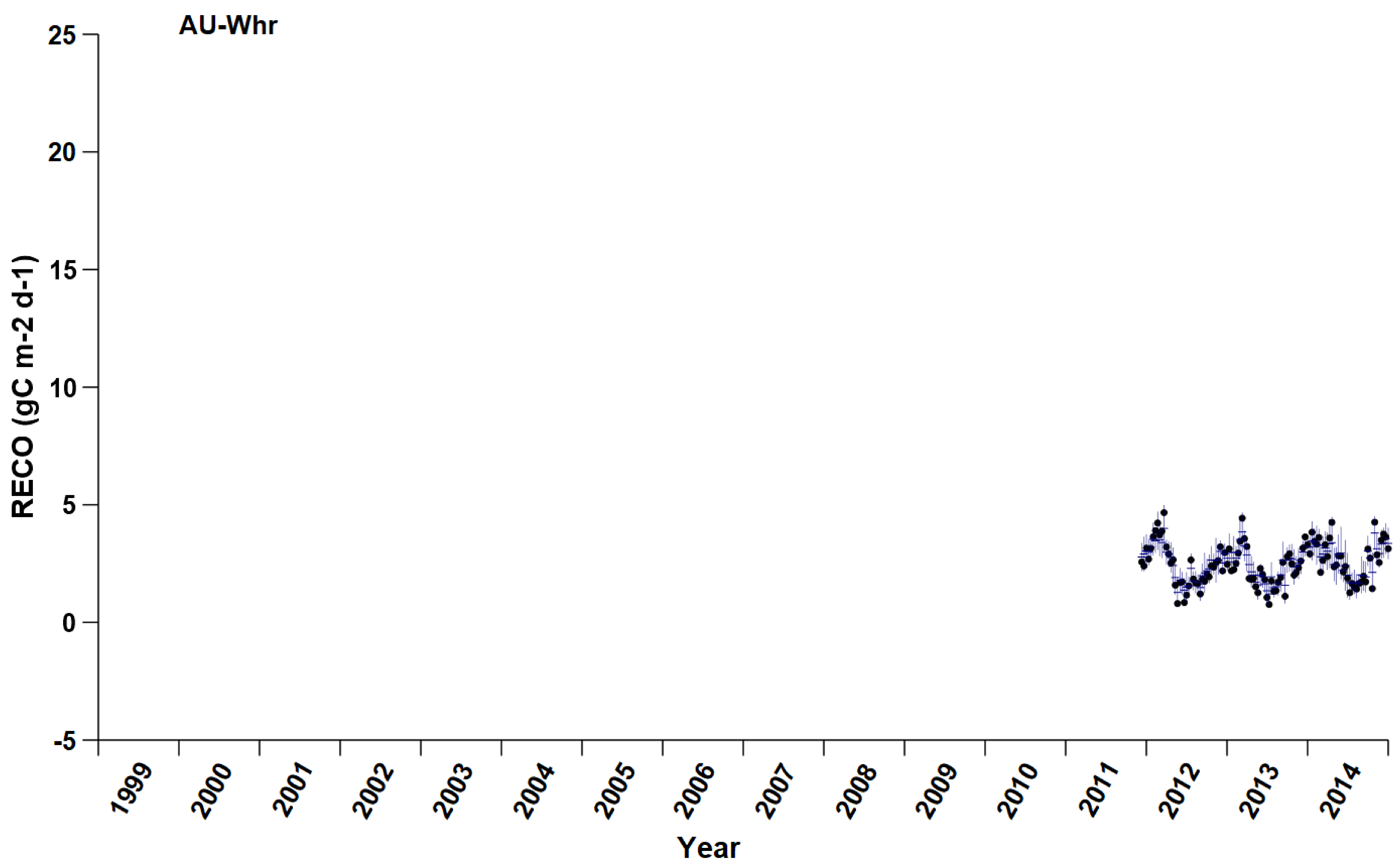

AU-Whr
RECO (gC m-2 d-1)
25
20
15
10
5
0
-5
1999
2000
2001
2002
2003
2004
2005
2006
2007
2008
2009
2010
2011
2012
2013
2014
Year

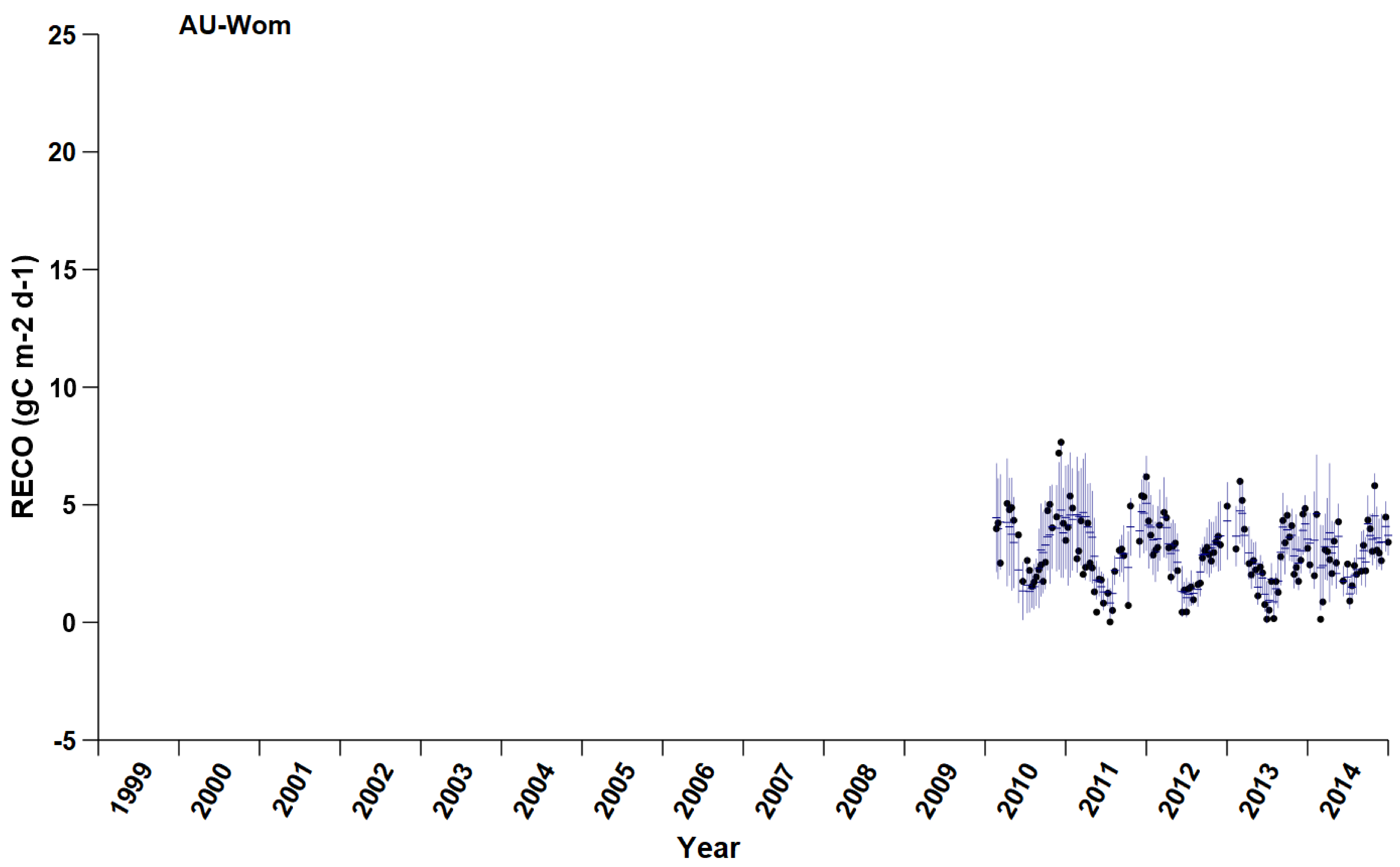
AU-Wom
RECO (gC m-2 d-1)
25
20
15
10
5
0
-5
1999
2000
2001
2002
2003
2004
2005
2006
2007
2008
2009
2010
2011
2012
2013
2014
Year

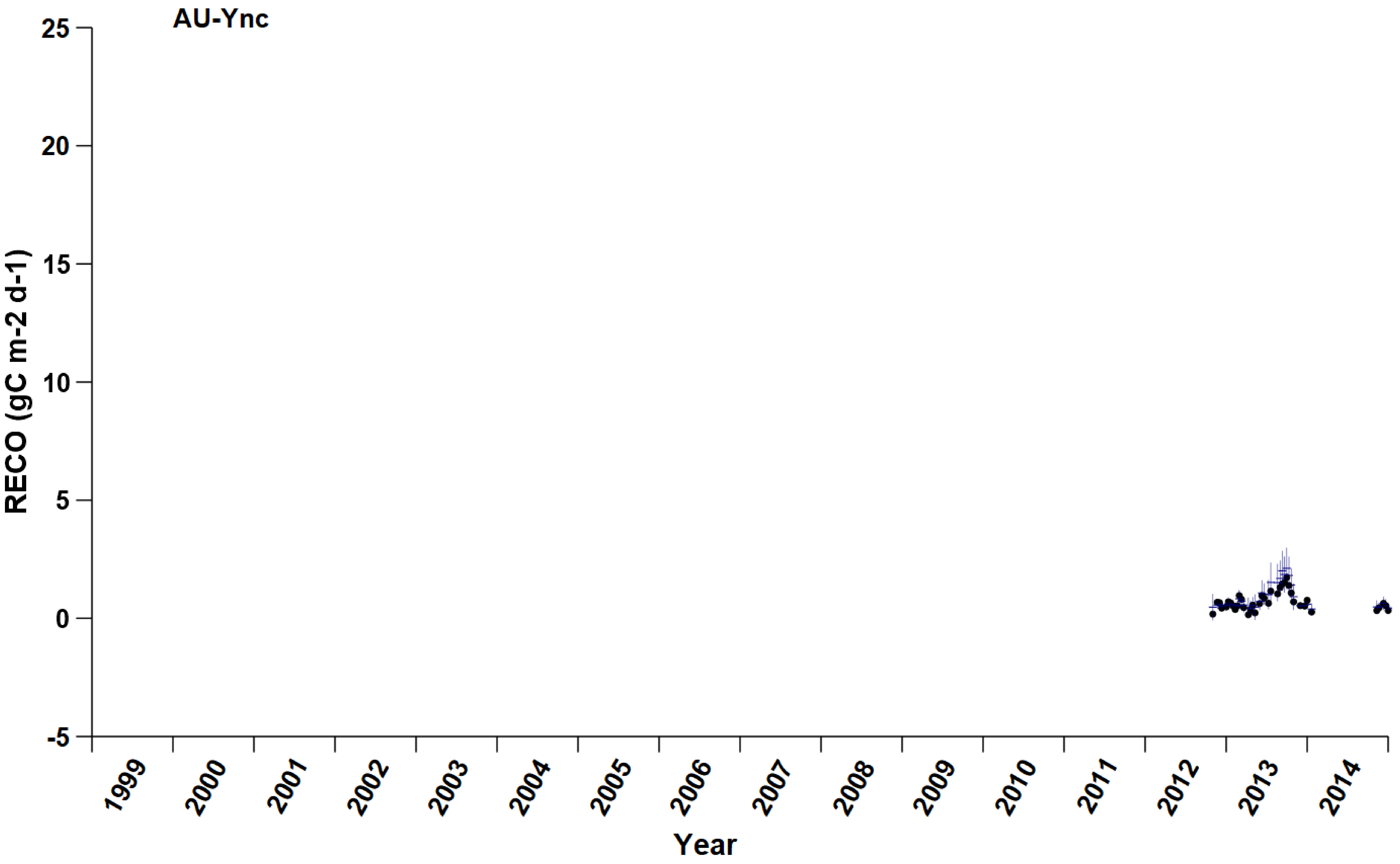

AU-Ync
25
20
15
10
5
0
-5
RECO (gC m-2 d-1)
1999
2000
2001
2002
2003
2004
2005
2006
2007
2008
2009
2010
2011
2012
2013
2014
Year

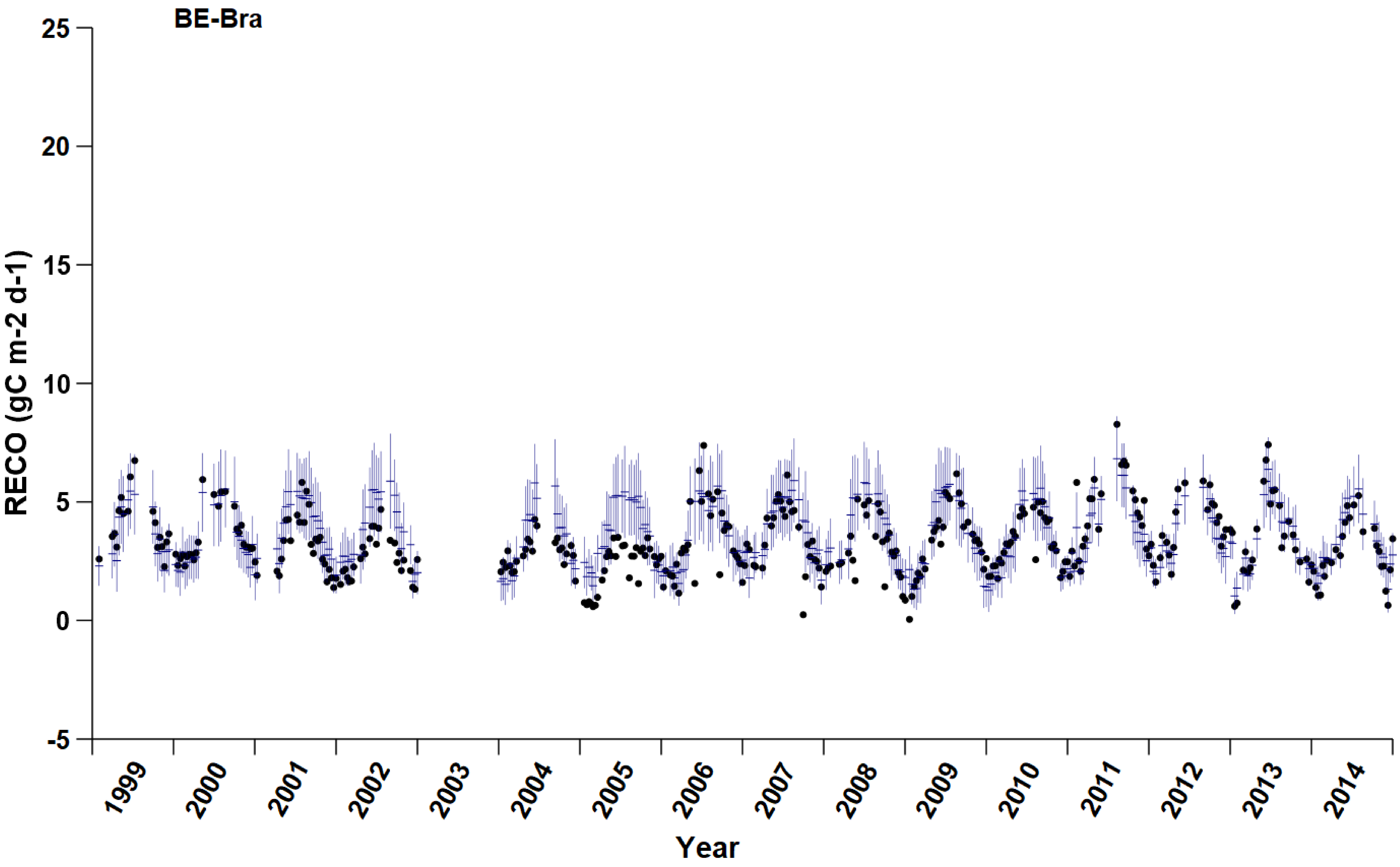
BE-Bra
RECO (gC m-2 d-1)
25
20
15
10
5
0
-5
1999
2000
2001
2002
2003
2004
2005
2006
2007
2008
2009
2010
2011
2012
2013
2014
Year

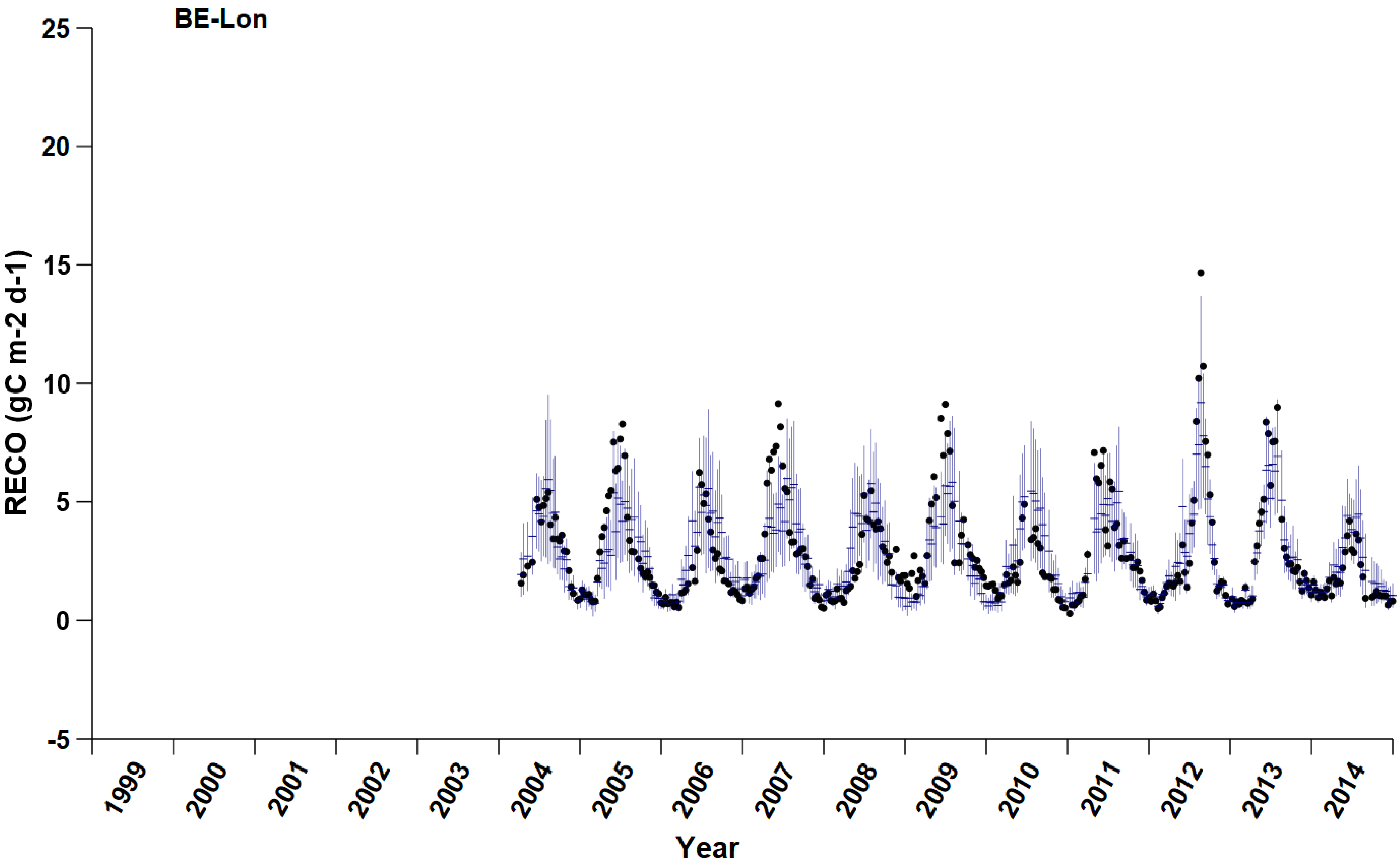

BE-Lon
RECO (gC m-2 d-1)
25
20
15
10
5
0
-5
1999
2000
2001
2002
2003
2004
2005
2006
2007
2008
2009
2010
2011
2012
2013
2014
Year

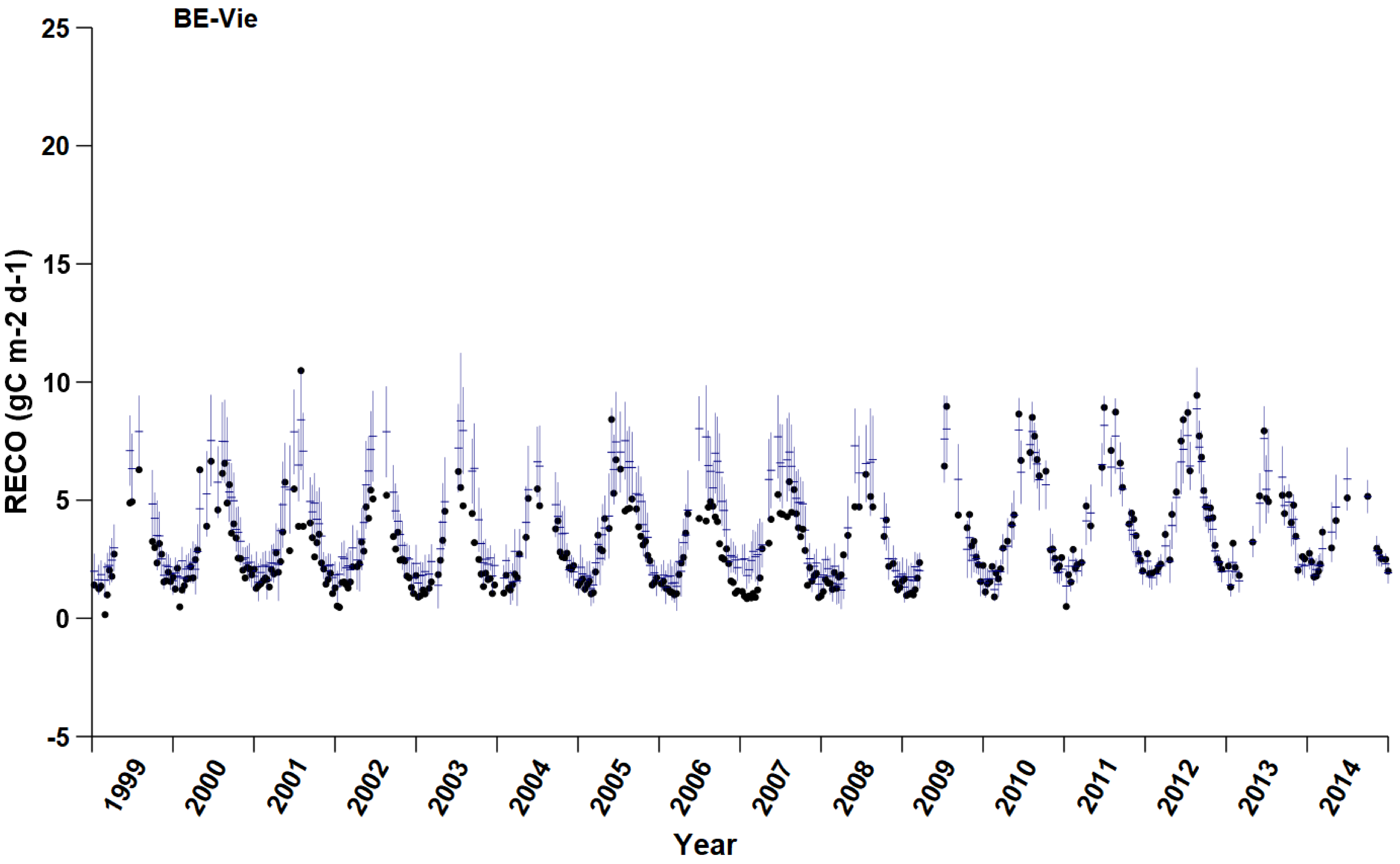

BE-Vie
RECO (gC m-2 d-1)
25
20
15
10
5
0
-5
1999
2000
2001
2002
2003
2004
2005
2006
2007
2008
2009
2010
2011
2012
2013
2014
Year

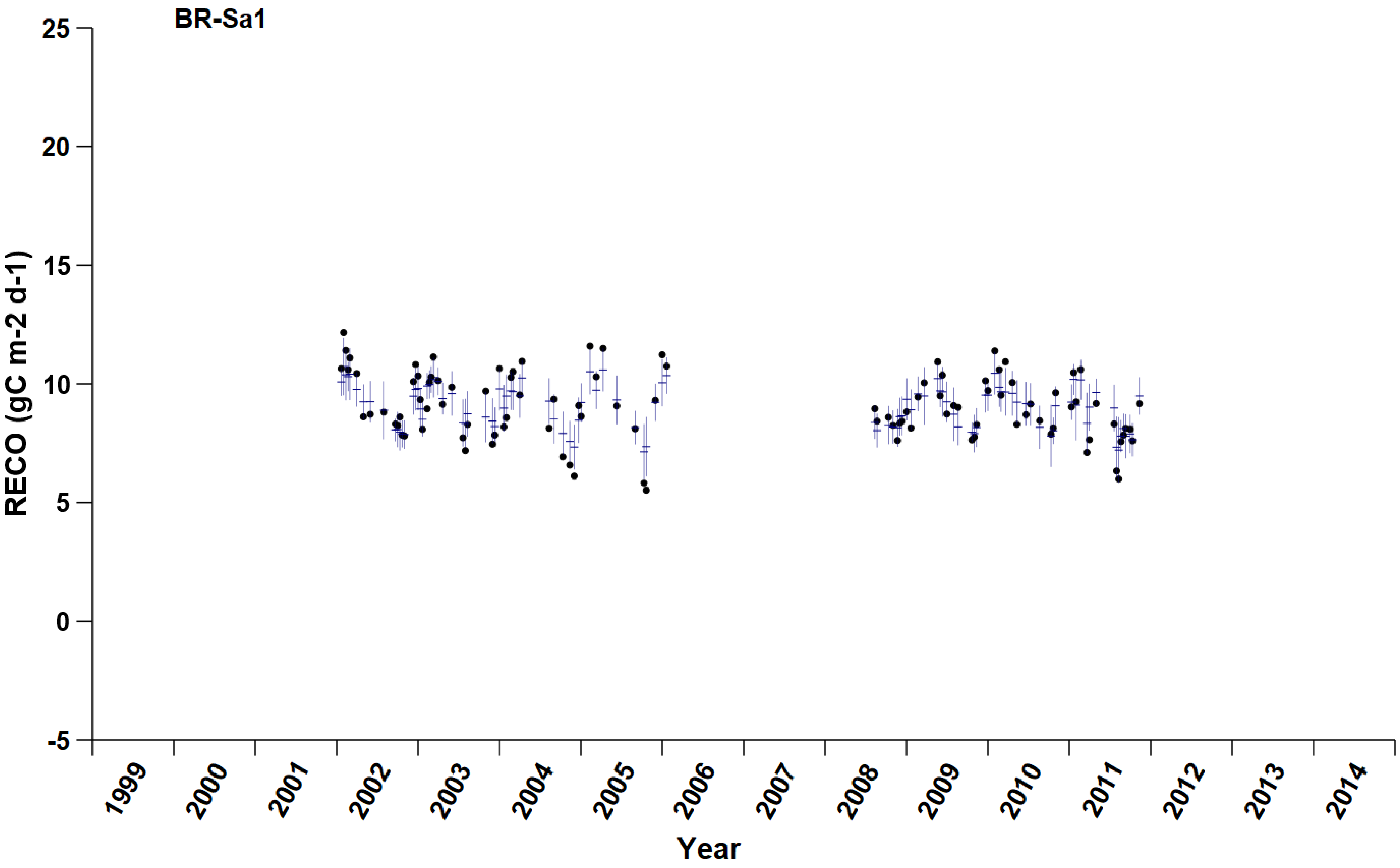
BR-Sa1
RECO (gC m-2 d-1)
25
20
15
10
5
0
-5
1999
2000
2001
2002
2003
2004
2005
2006
2007
2008
2009
2010
2011
2012
2013
2014
Year

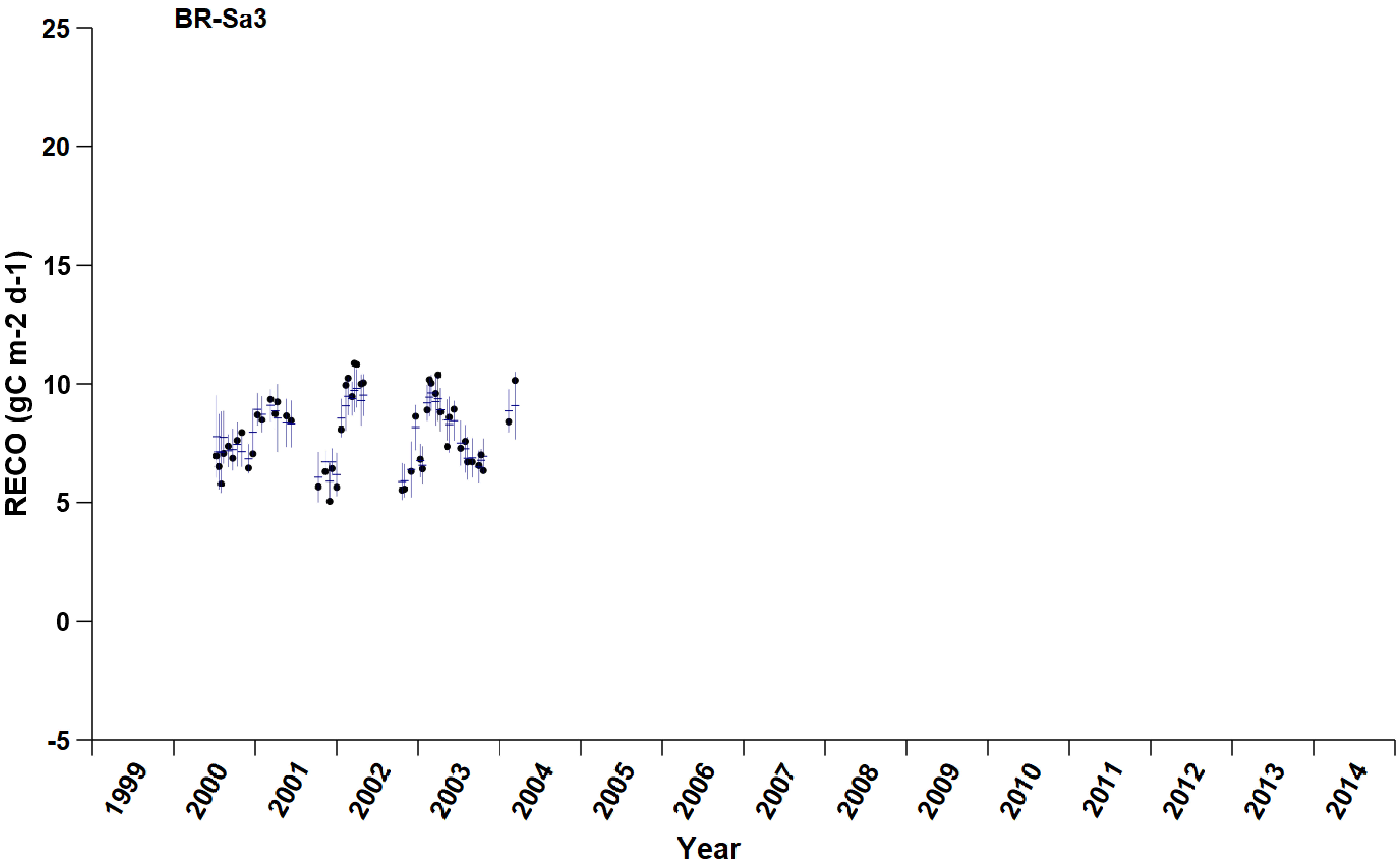
BR-Sa3
RECO (gC m-2 d-1)
25
20
15
10
5
0
-5
1999
2000
2001
2002
2003
2004
2005
2006
2007
2008
2009
2010
2011
2012
2013
2014
Year

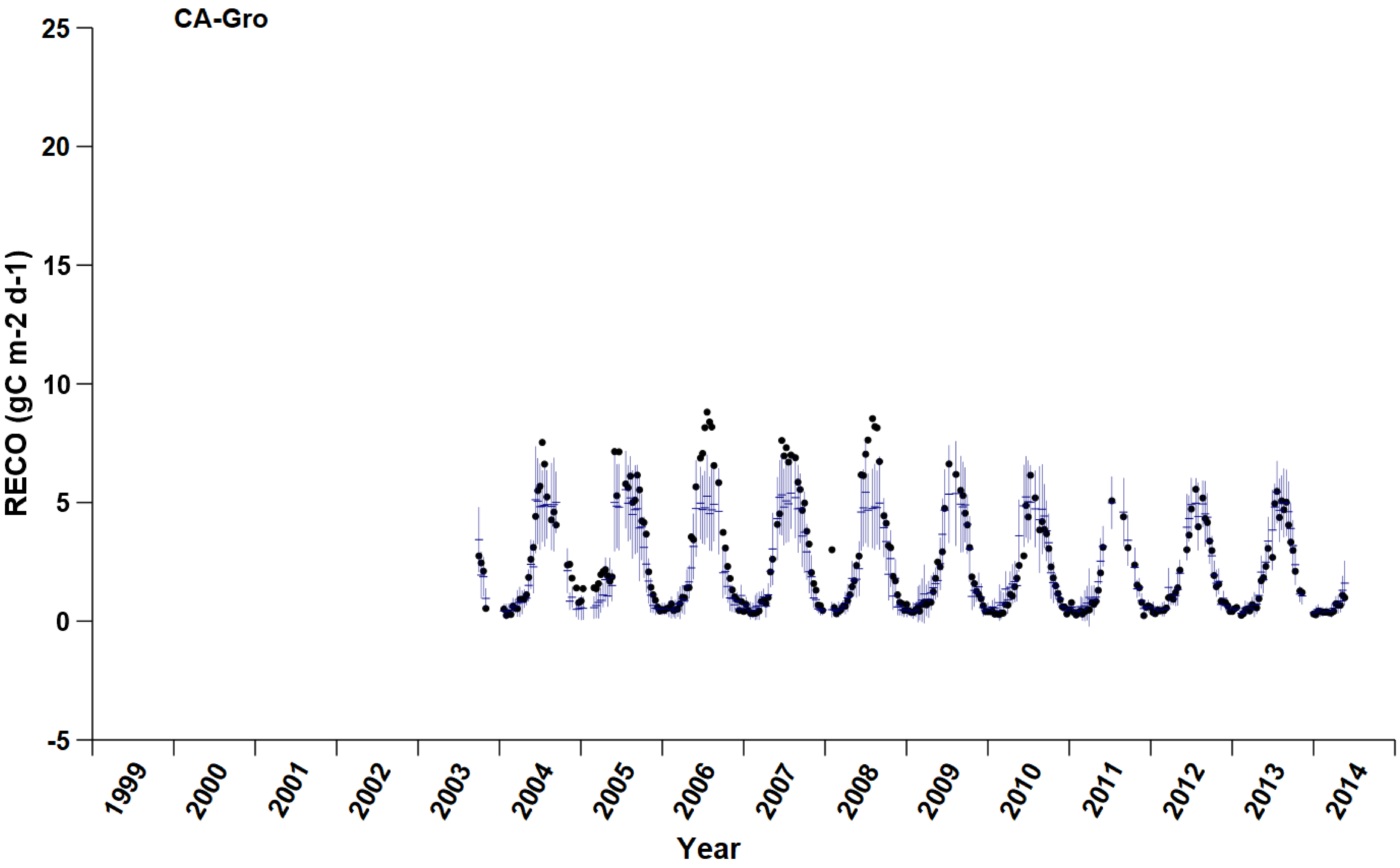
CA-Gro
RECO (gC m-2 d-1)
25
20
15
10
5
0
-5
1999
2000
2001
2002
2003
2004
2005
2006
2007
2008
2009
2010
2011
2012
2013
2014
Year

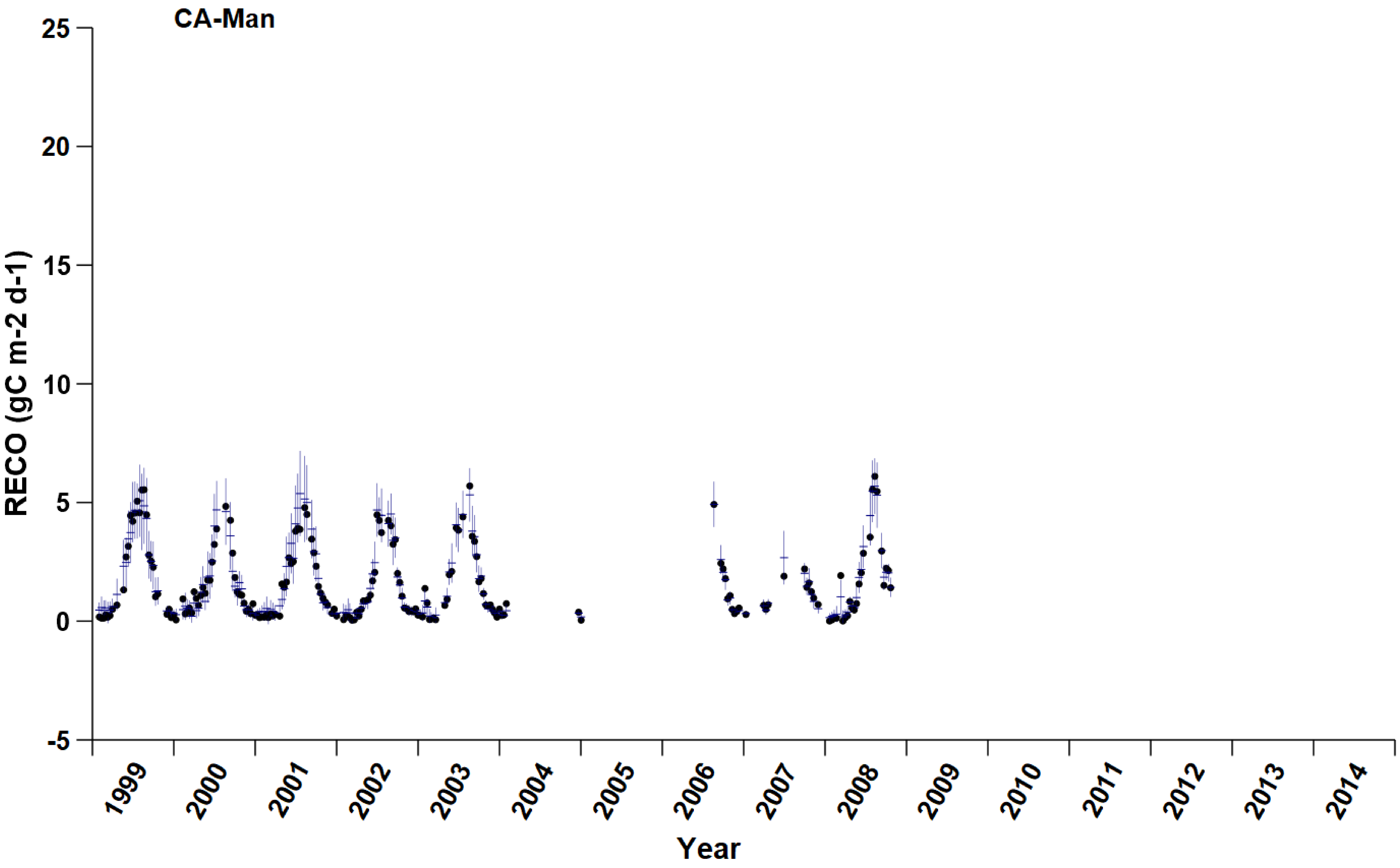
CA-Man
RECO (gC m-2 d-1)
25
20
15
10
5
0
-5
1999
2000
2001
2002
2003
2004
2005
2006
2007
2008
2009
2010
2011
2012
2013
2014
Year

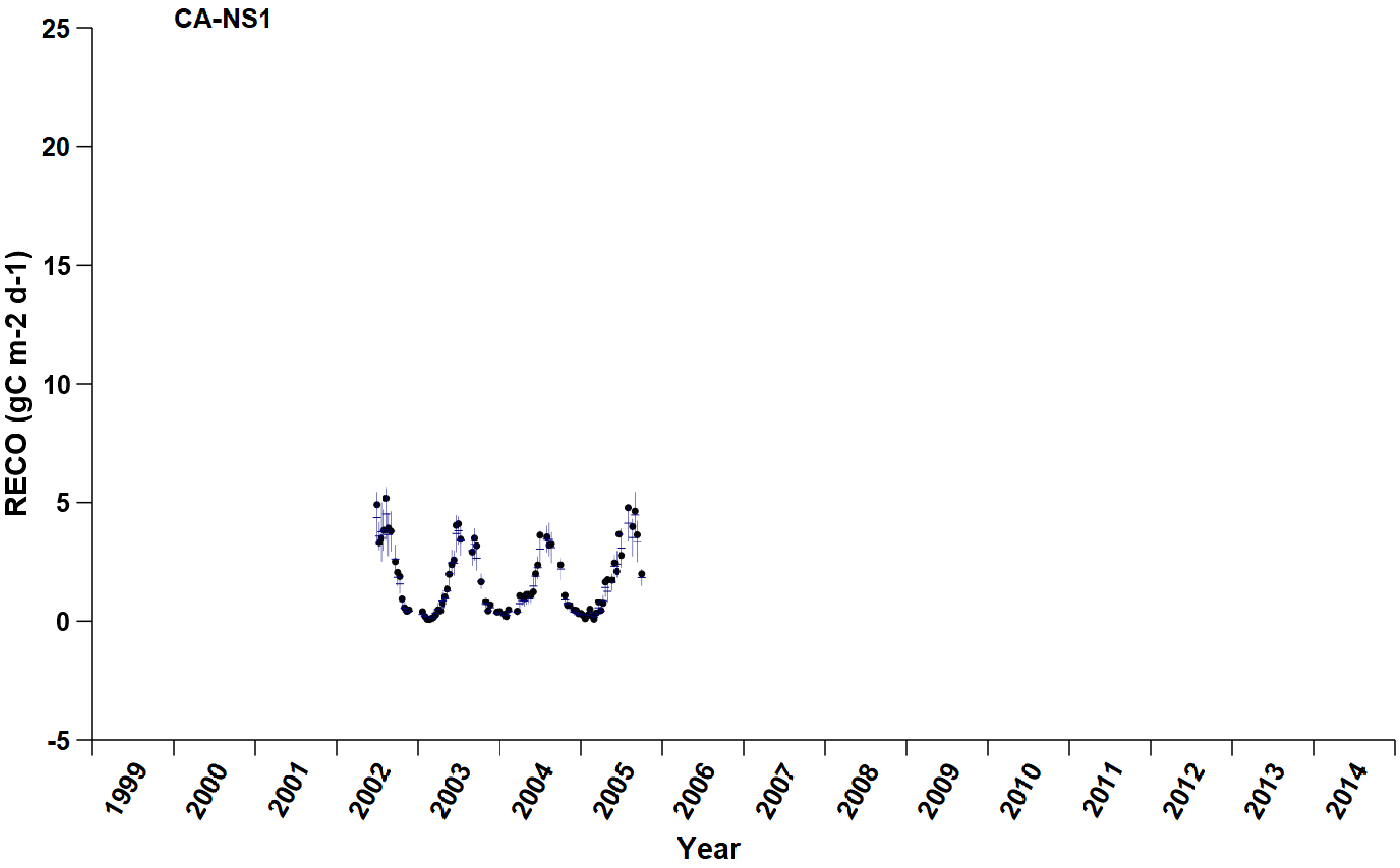
CA-NS1
RECO (gC m-2 d-1)
25
20
15
10
5
0
-5
1999
2000
2001
2002
2003
2004
2005
2006
2007
2008
2009
2010
2011
2012
2013
2014
Year

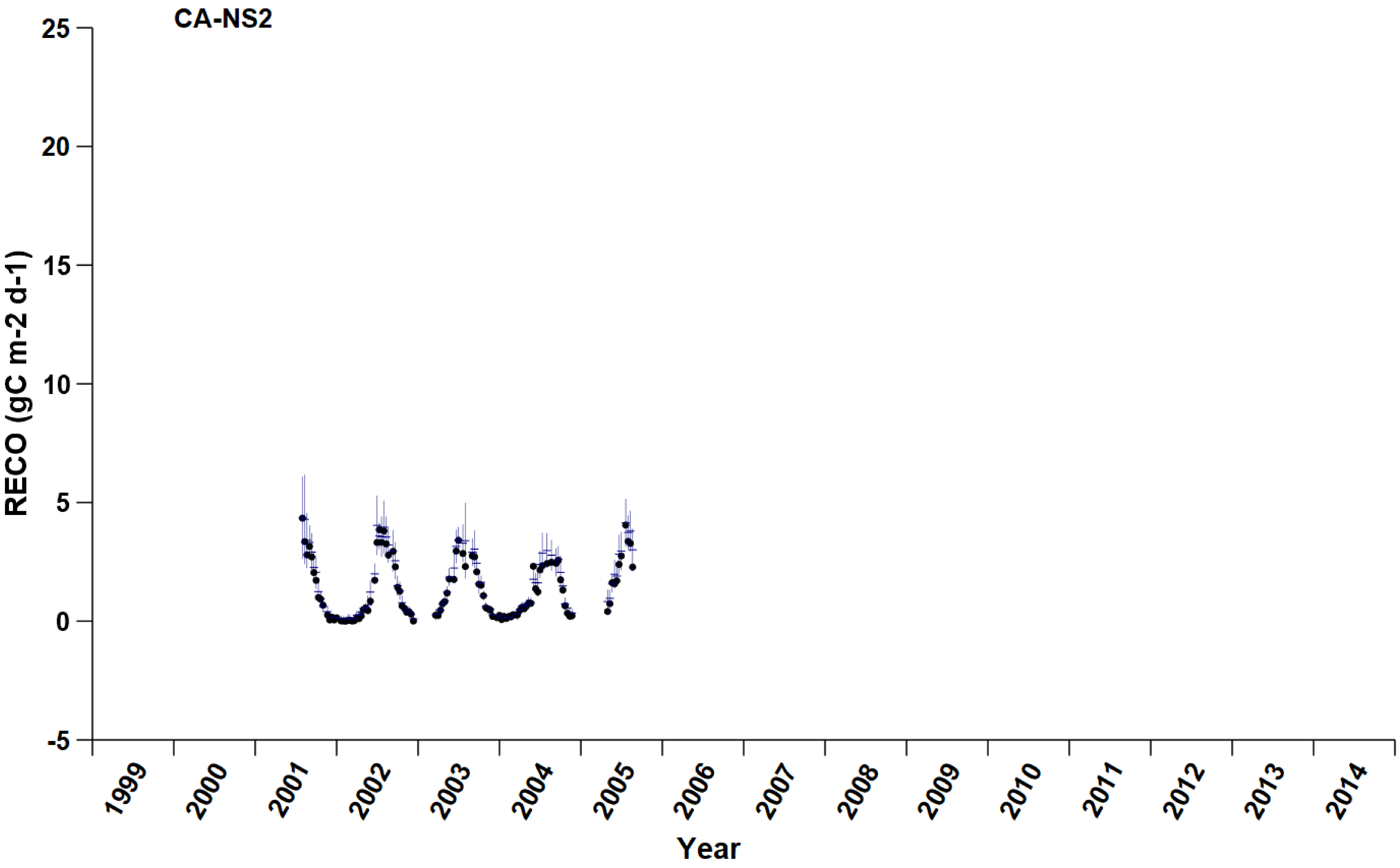
CA-NS2
RECO (gC m-2 d-1)
25
20
15
10
5
0
-5
1999
2000
2001
2002
2003
2004
2005
2006
2007
2008
2009
2010
2011
2012
2013
2014
Year

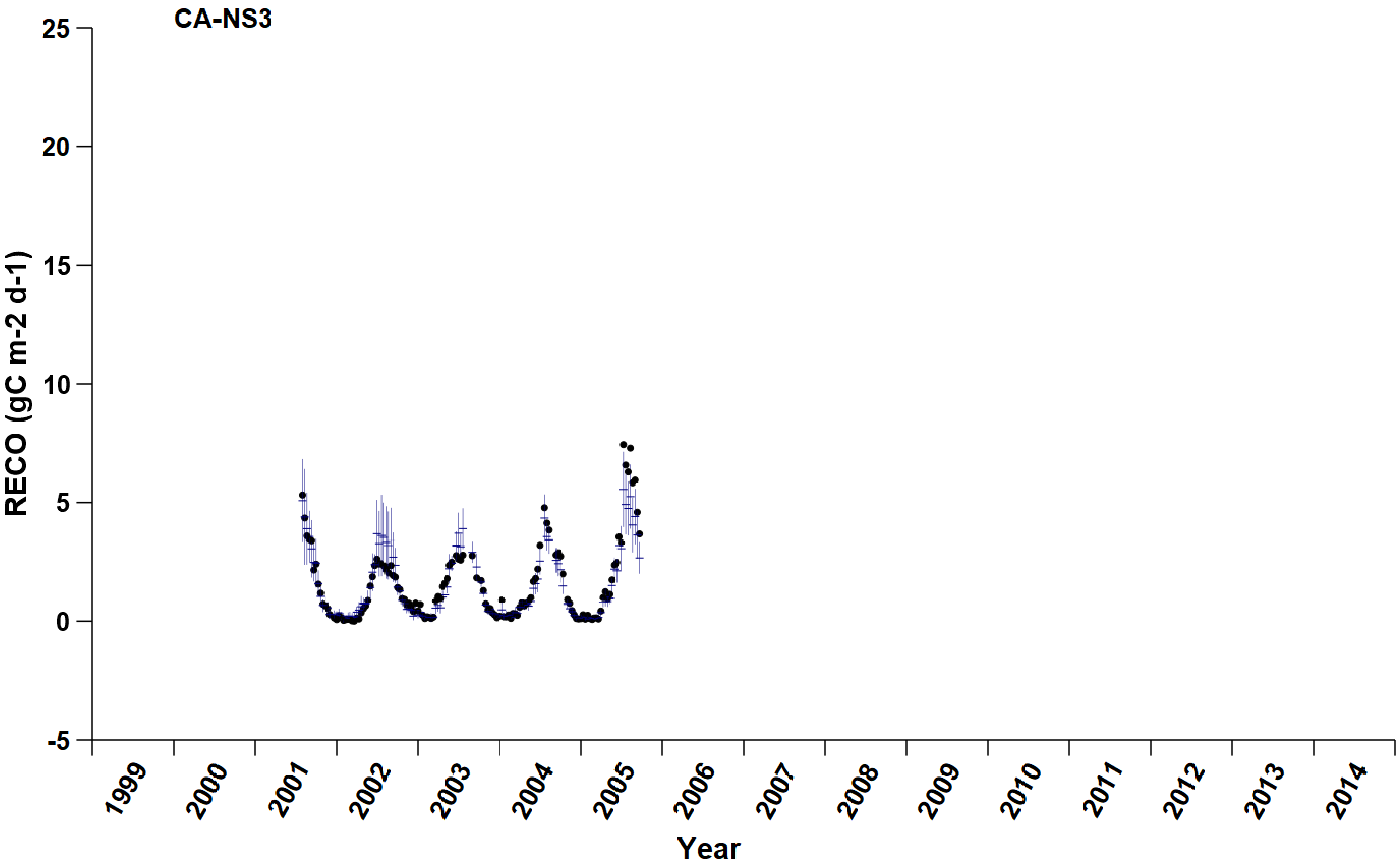
CA-NS3
RECO (gC m-2 d-1)
25
20
15
10
5
0
-5
1999
2000
2001
2002
2003
2004
2005
2006
2007
2008
2009
2010
2011
2012
2013
2014
Year

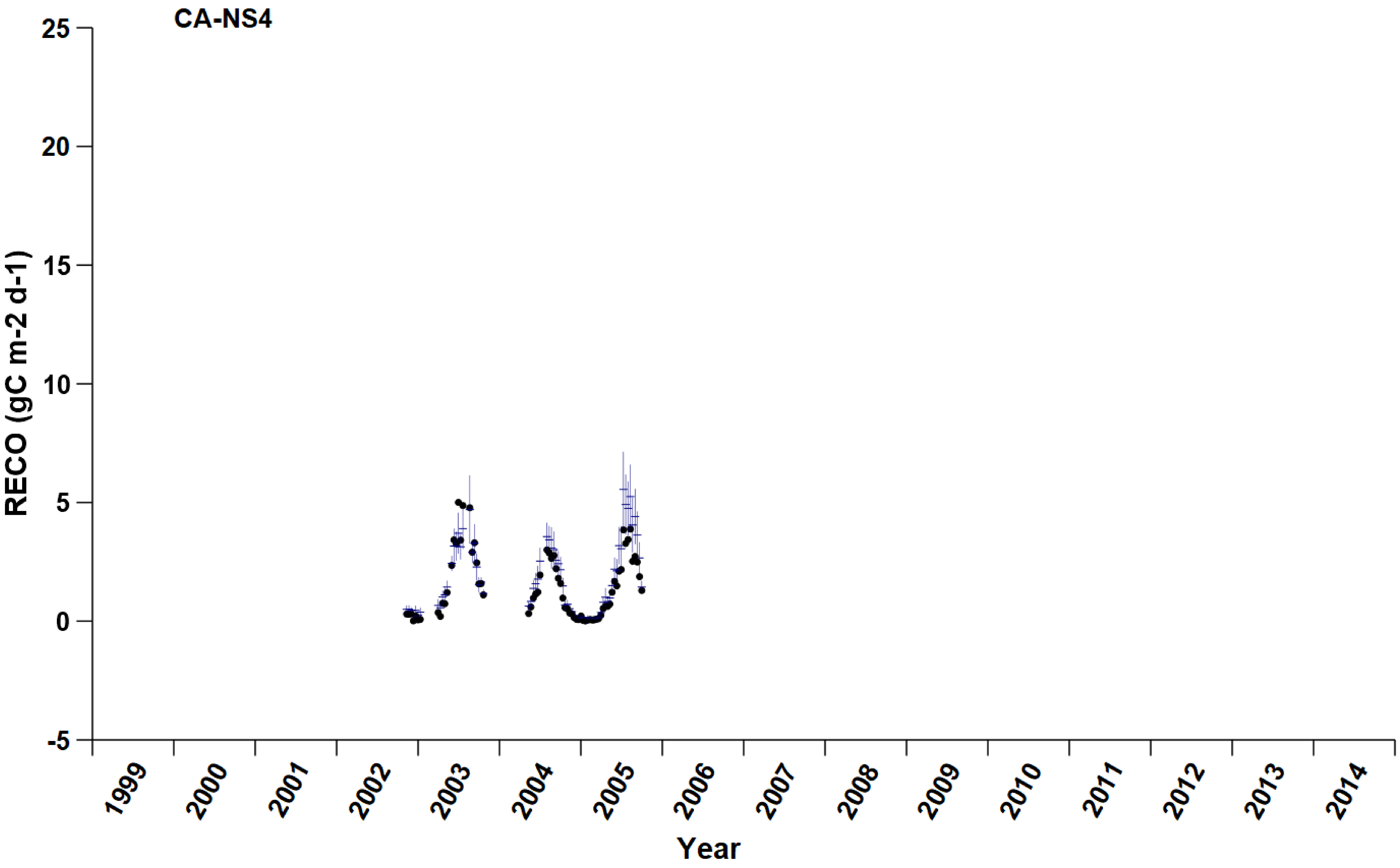
CA-NS4
RECO (gC m-2 d-1)
25
20
15
10
5
0
-5
1999
2000
2001
2002
2003
2004
2005
2006
2007
2008
2009
2010
2011
2012
2013
2014
Year

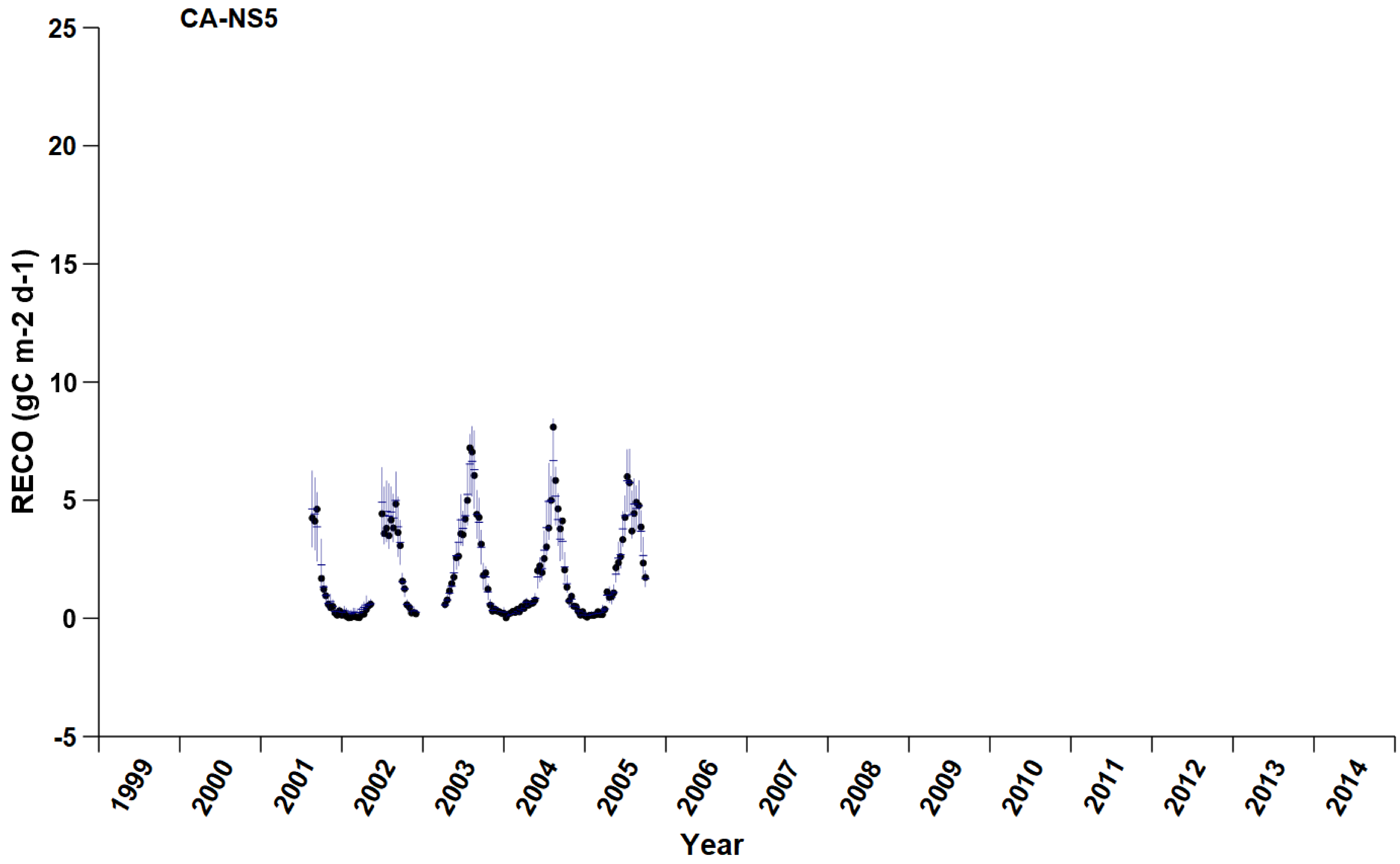
CA-NS5
RECO (gC m-2 d-1)
25
20
15
10
5
0
-5
1999
2000
2001
2002
2003
2004
2005
2006
2007
2008
2009
2010
2011
2012
2013
2014
Year

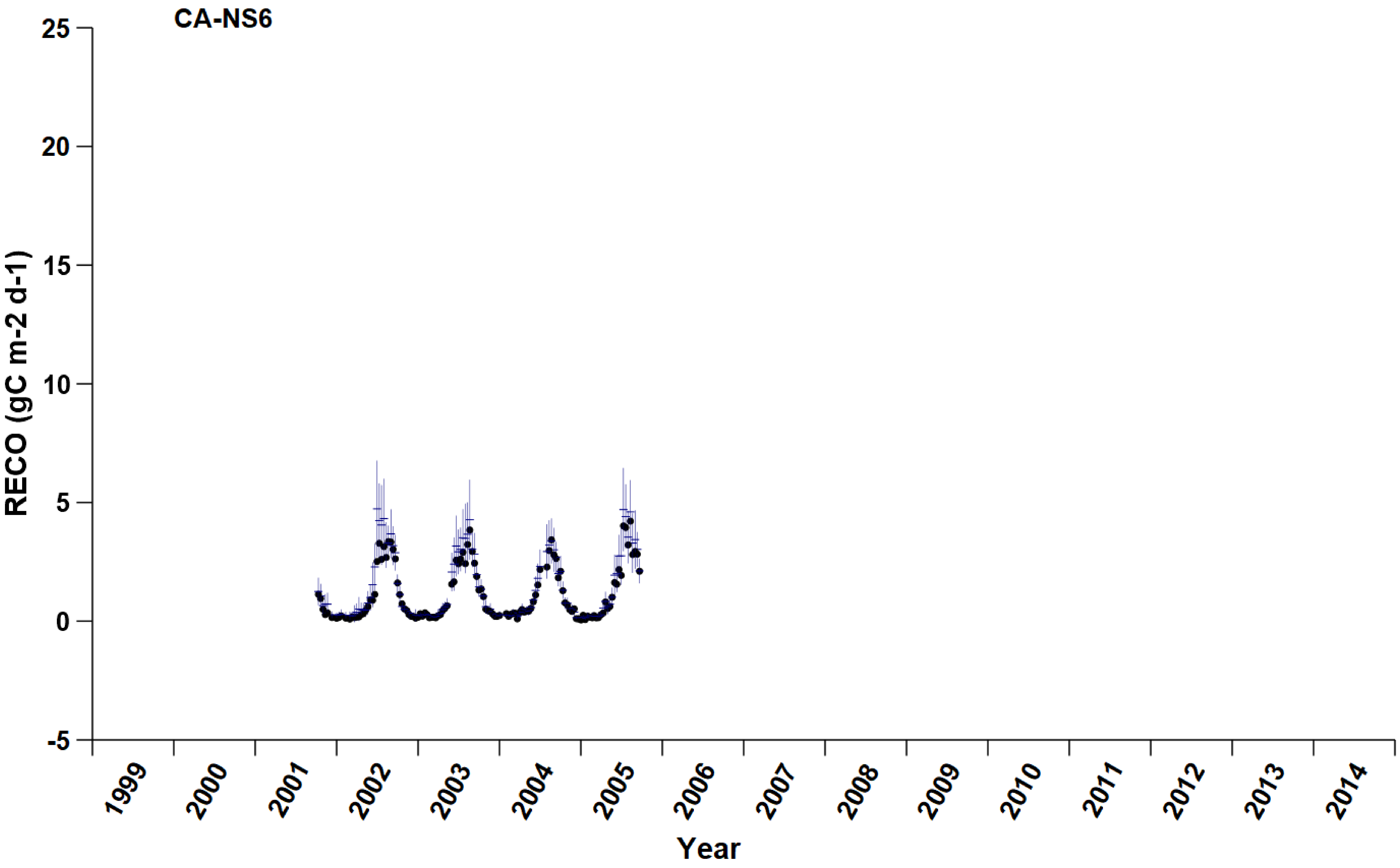
CA-NS6
RECO (gC m-2 d-1)
25
20
15
10
5
0
-5
1999
2000
2001
2002
2003
2004
2005
2006
2007
2008
2009
2010
2011
2012
2013
2014
Year

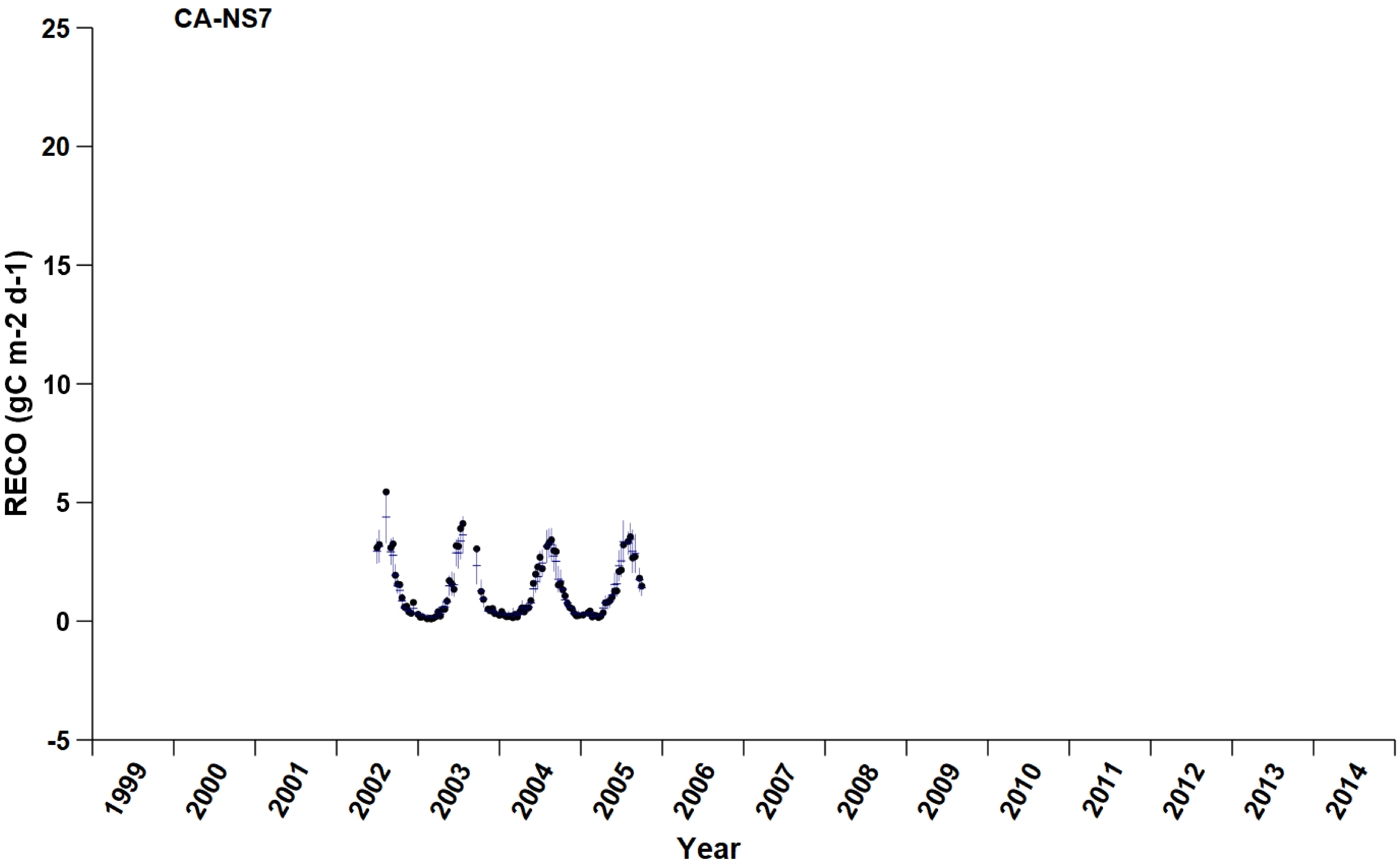
CA-NS7
RECO (gC m-2 d-1)
25
20
15
10
5
0
-5
1999
2000
2001
2002
2003
2004
2005
2006
2007
2008
2009
2010
2011
2012
2013
2014
Year

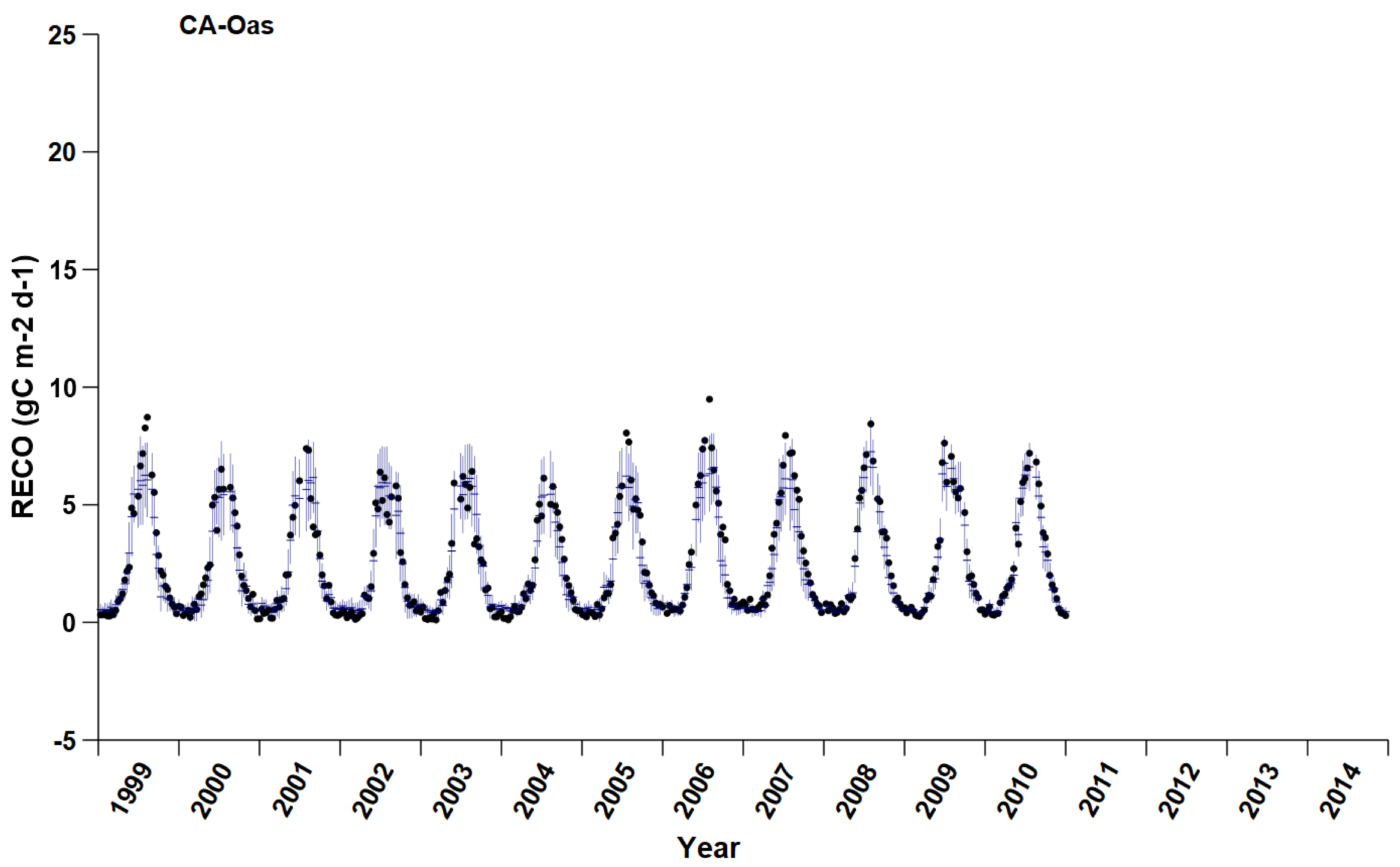
CA-Oas
RECO (gC m-2 d-1)
25
20
15
10
5
0
-5
1999
2000
2001
2002
2003
2004
2005
2006
2007
2008
2009
2010
2011
2012
2013
2014
Year

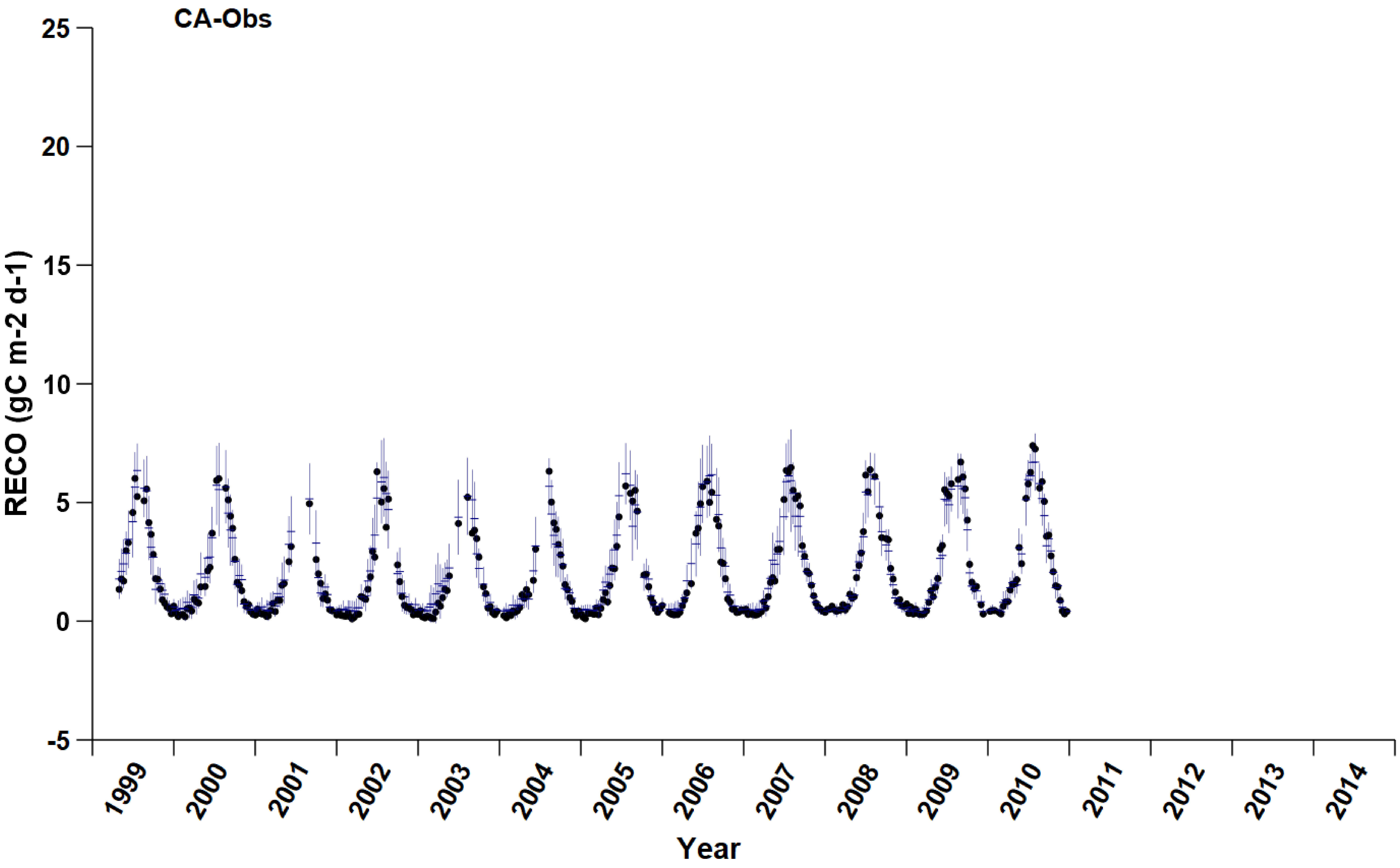
CA-Obs
RECO (gC m-2 d-1)
25
20
15
10
5
0
-5
1999
2000
2001
2002
2003
2004
2005
2006
2007
2008
2009
2010
2011
2012
2013
2014
Year

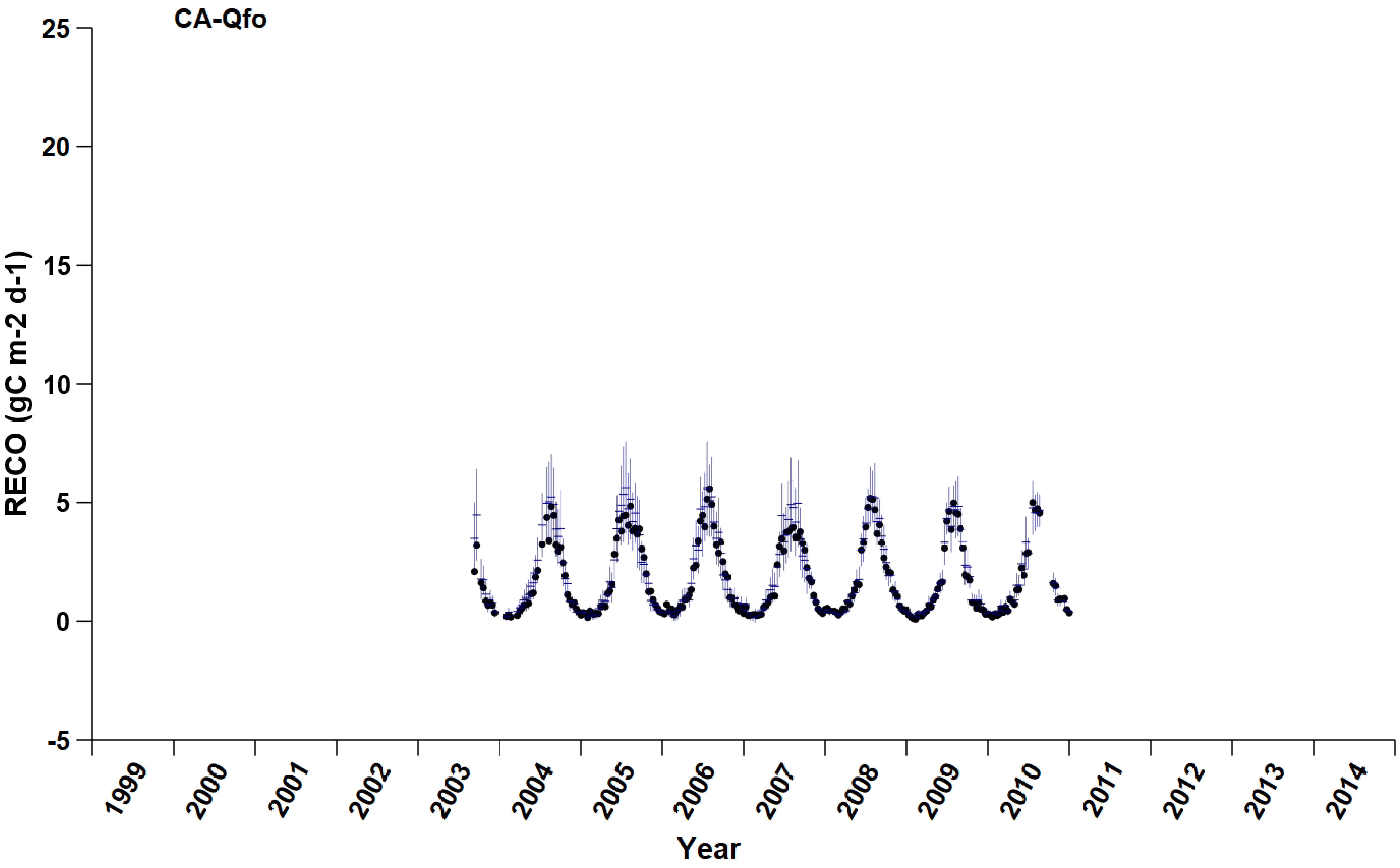
CA-Qfo
RECO (gC m-2 d-1)
25
20
15
10
5
0
-5
1999
2000
2001
2002
2003
2004
2005
2006
2007
2008
2009
2010
2011
2012
2013
2014
Year

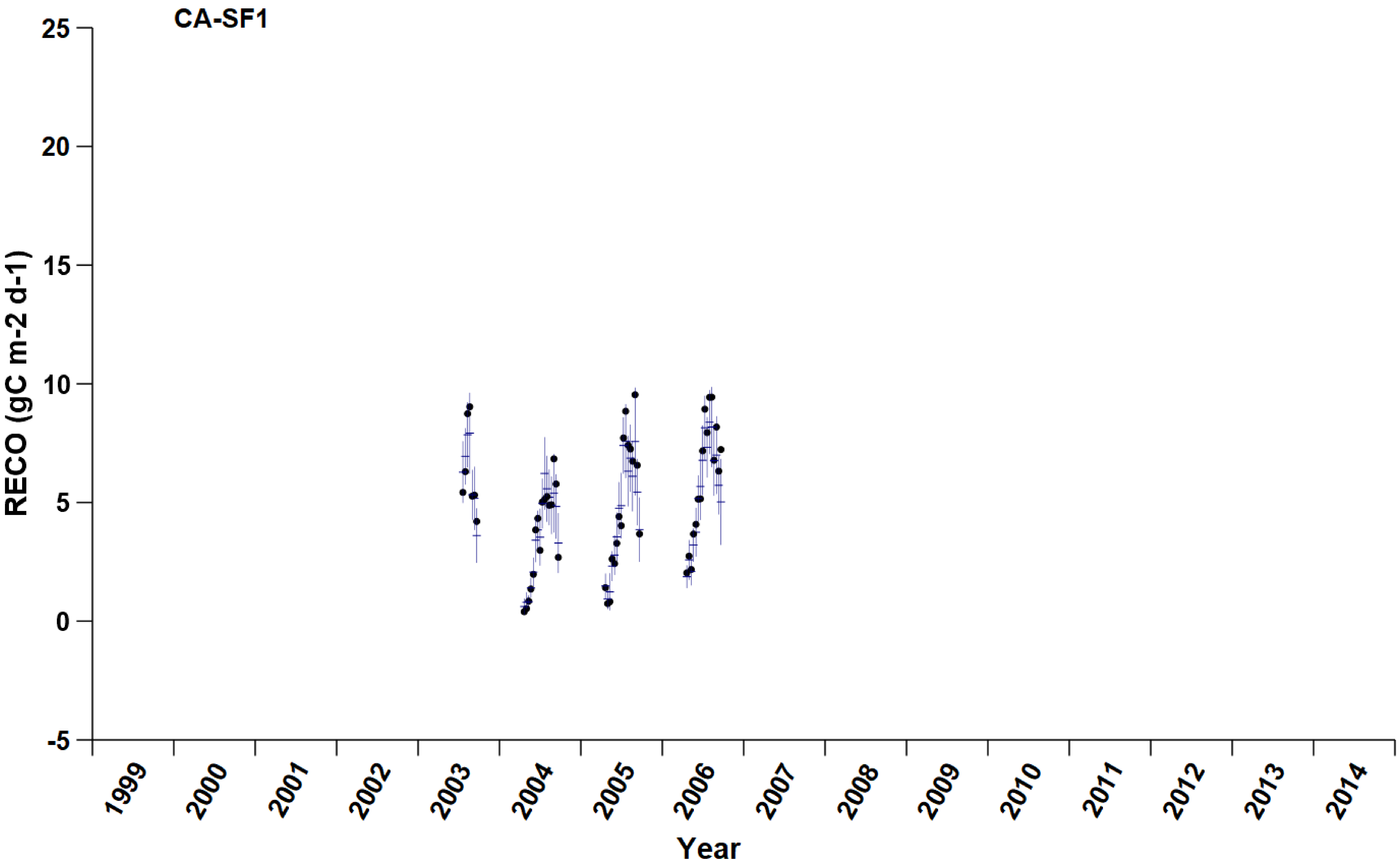
CA-SF1
RECO (gC m-2 d-1)
25
20
15
10
5
0
-5
1999
2000
2001
2002
2003
2004
2005
2006
2007
2008
2009
2010
2011
2012
2013
2014
Year

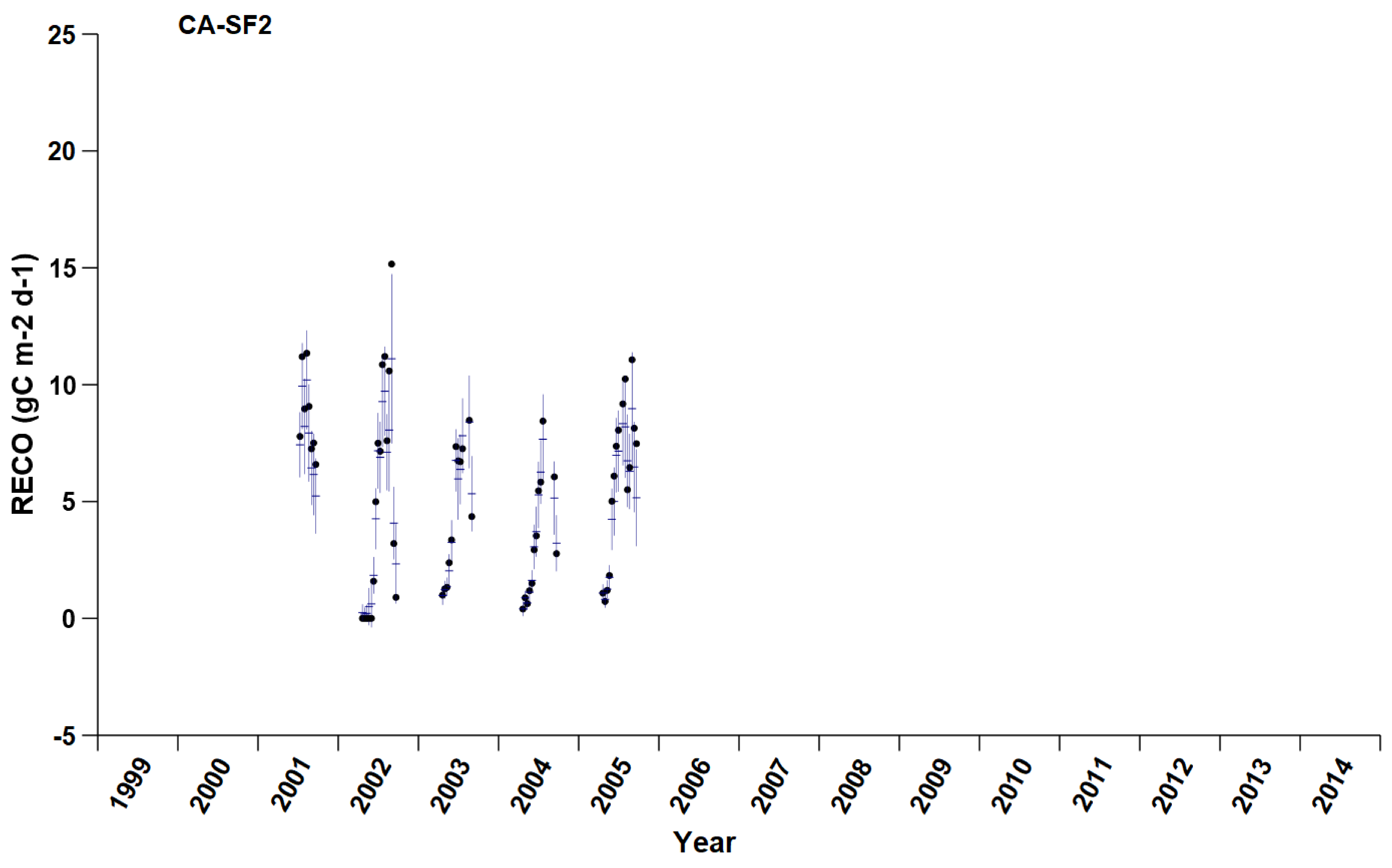
CA-SF2
RECO (gC m-2 d-1)
25
20
15
10
5
0
-5
1999
2000
2001
2002
2003
2004
2005
2006
2007
2008
2009
2010
2011
2012
2013
2014
Year

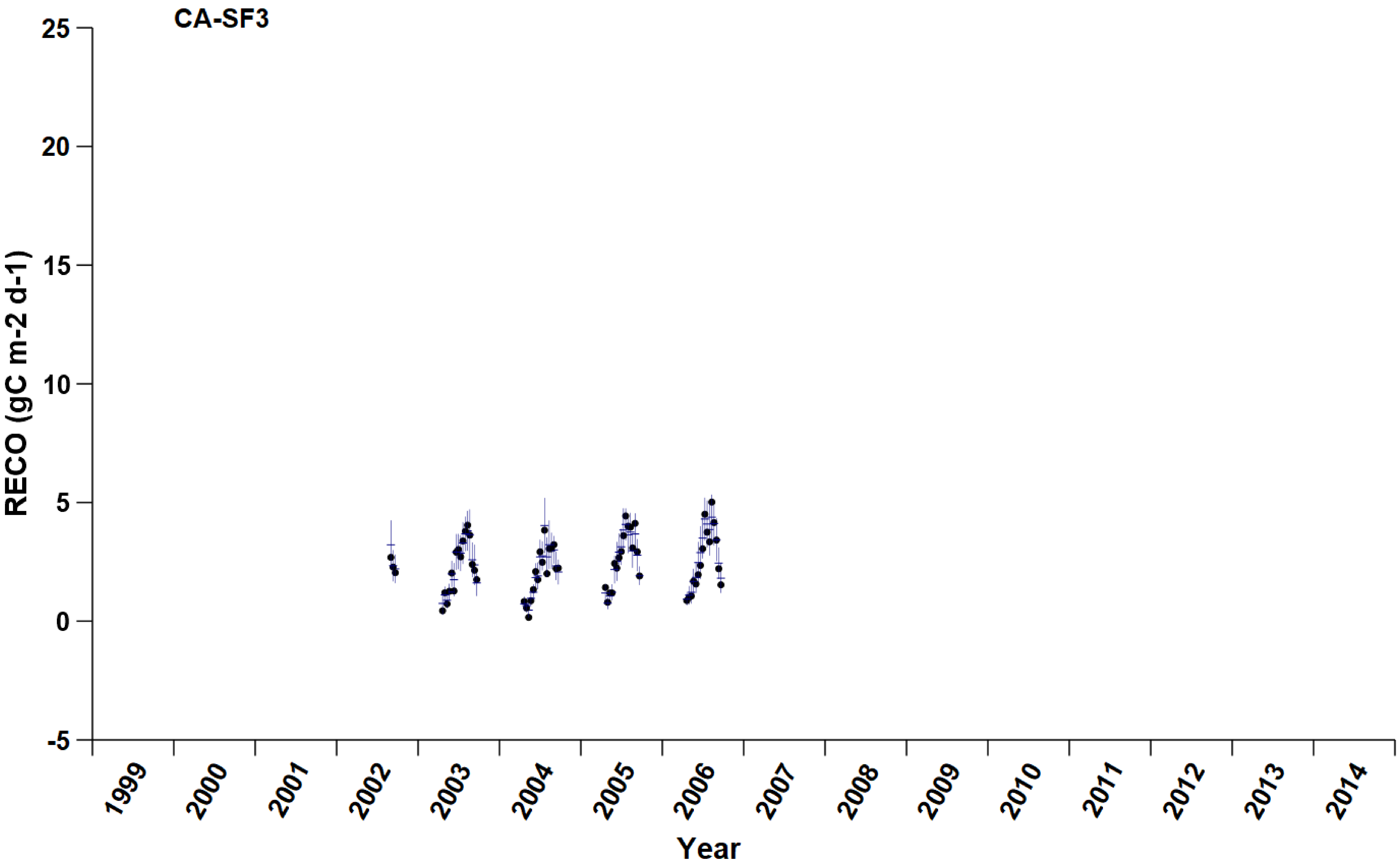
CA-SF3
RECO (gC m-2 d-1)
25
20
15
10
5
0
-5
1999
2000
2001
2002
2003
2004
2005
2006
2007
2008
2009
2010
2011
2012
2013
2014
Year

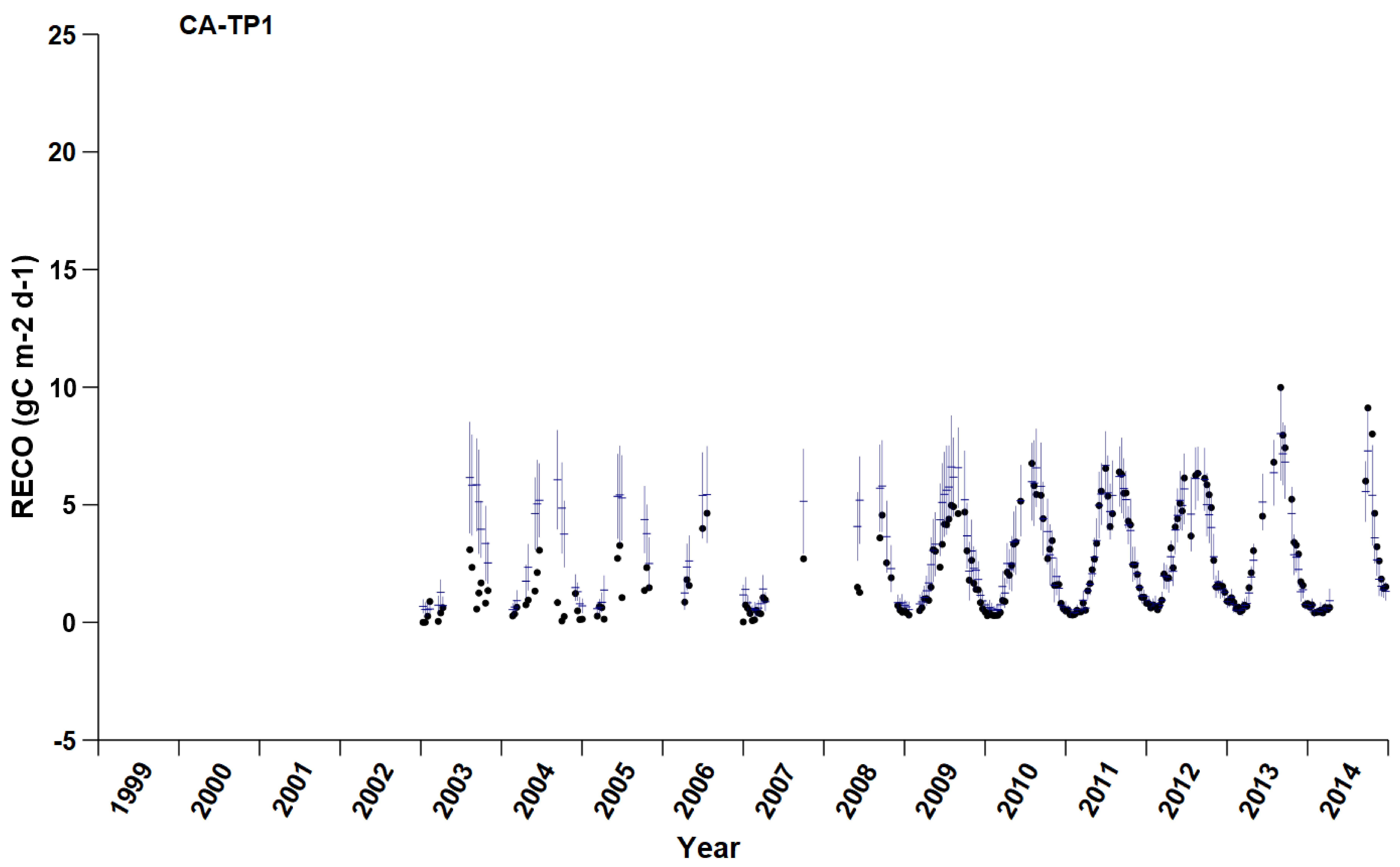
CA-TP1
RECO (gC m-2 d-1)
25
20
15
10
5
0
-5
Year
1999
2000
2001
2002
2003
2004
2005
2006
2007
2008
2009
2010
2011
2012
2013
2014

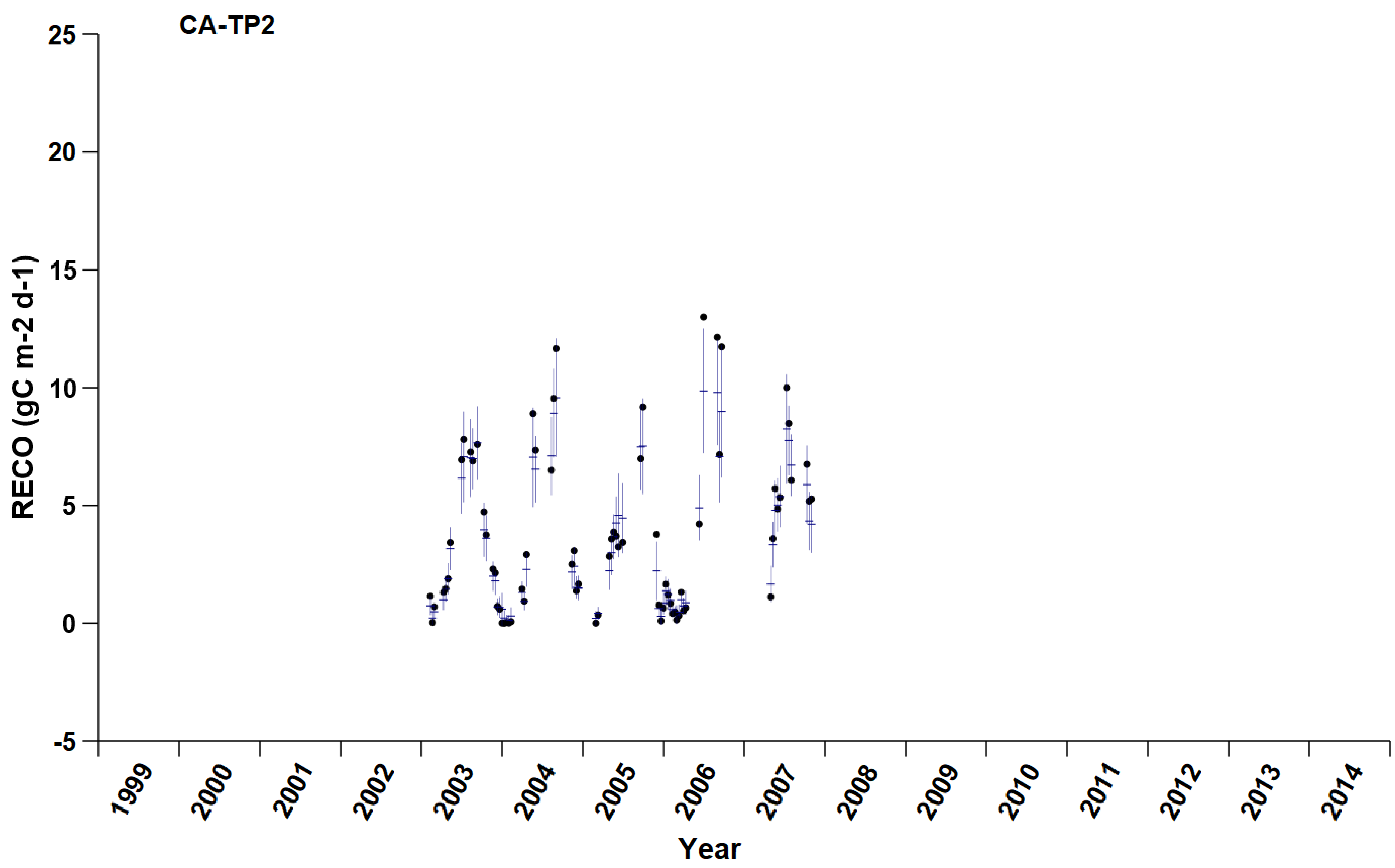
CA-TP2
RECO (gC m-2 d-1)
25
20
15
10
5
0
-5
1999
2000
2001
2002
2003
2004
2005
2006
2007
2008
2009
2010
2011
2012
2013
2014
Year

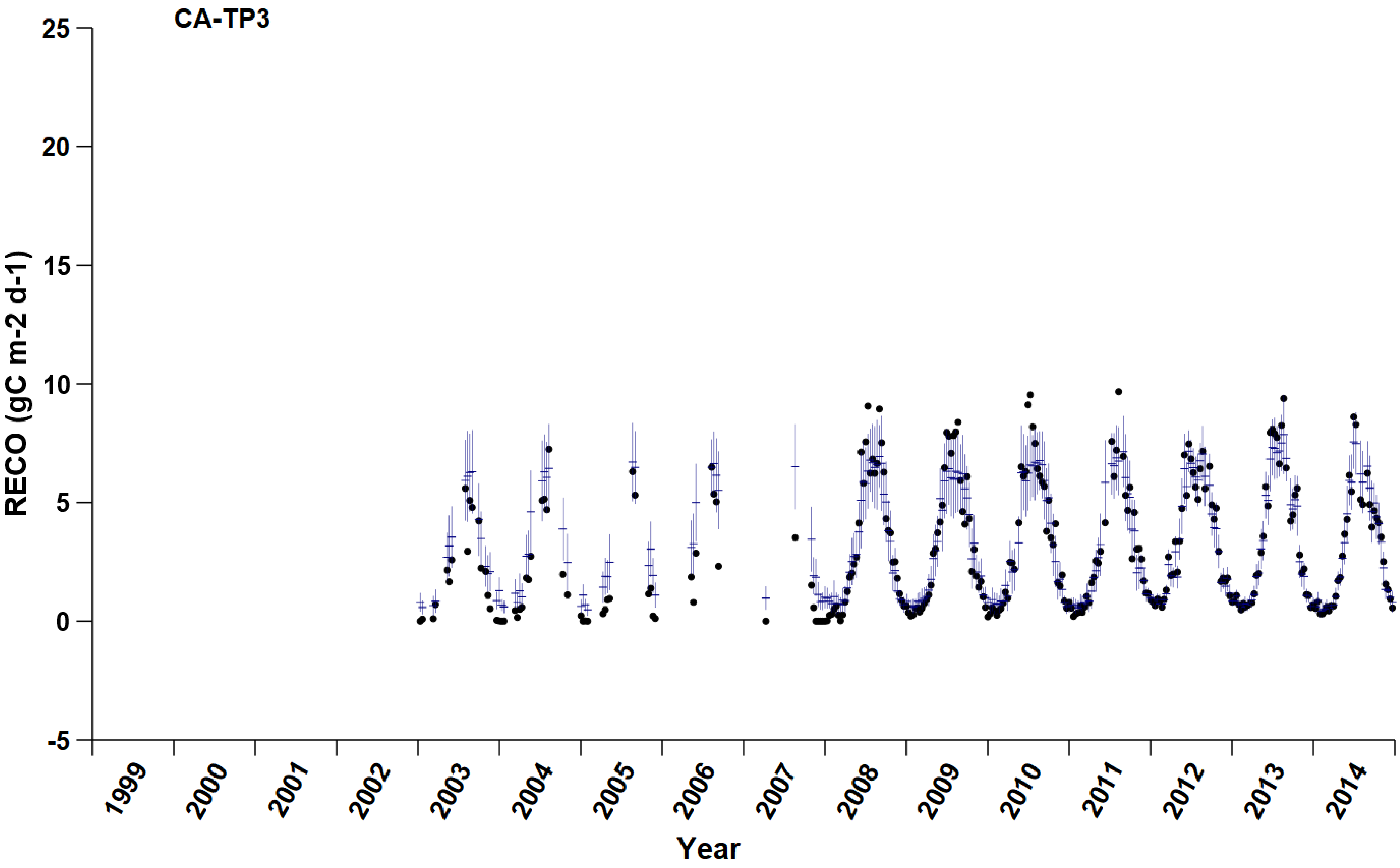

CA-TP3
RECO (gC m-2 d-1)
25
20
15
10
5
0
-5
1999
2000
2001
2002
2003
2004
2005
2006
2007
2008
2009
2010
2011
2012
2013
2014
Year

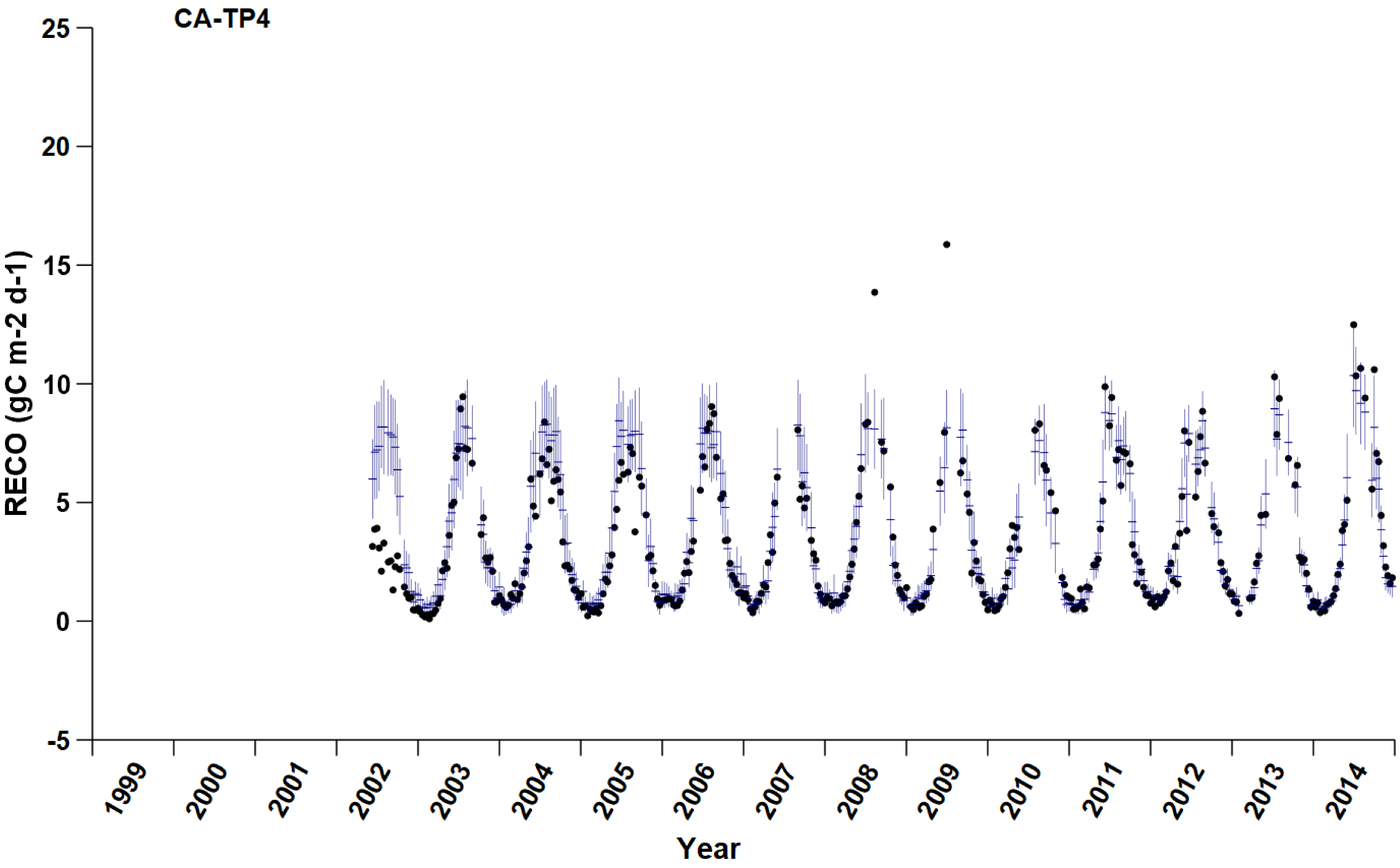
CA-TP4
RECO (gC m-2 d-1)
25
20
15
10
5
0
-5
1999
2000
2001
2002
2003
2004
2005
2006
2007
2008
2009
2010
2011
2012
2013
2014
Year

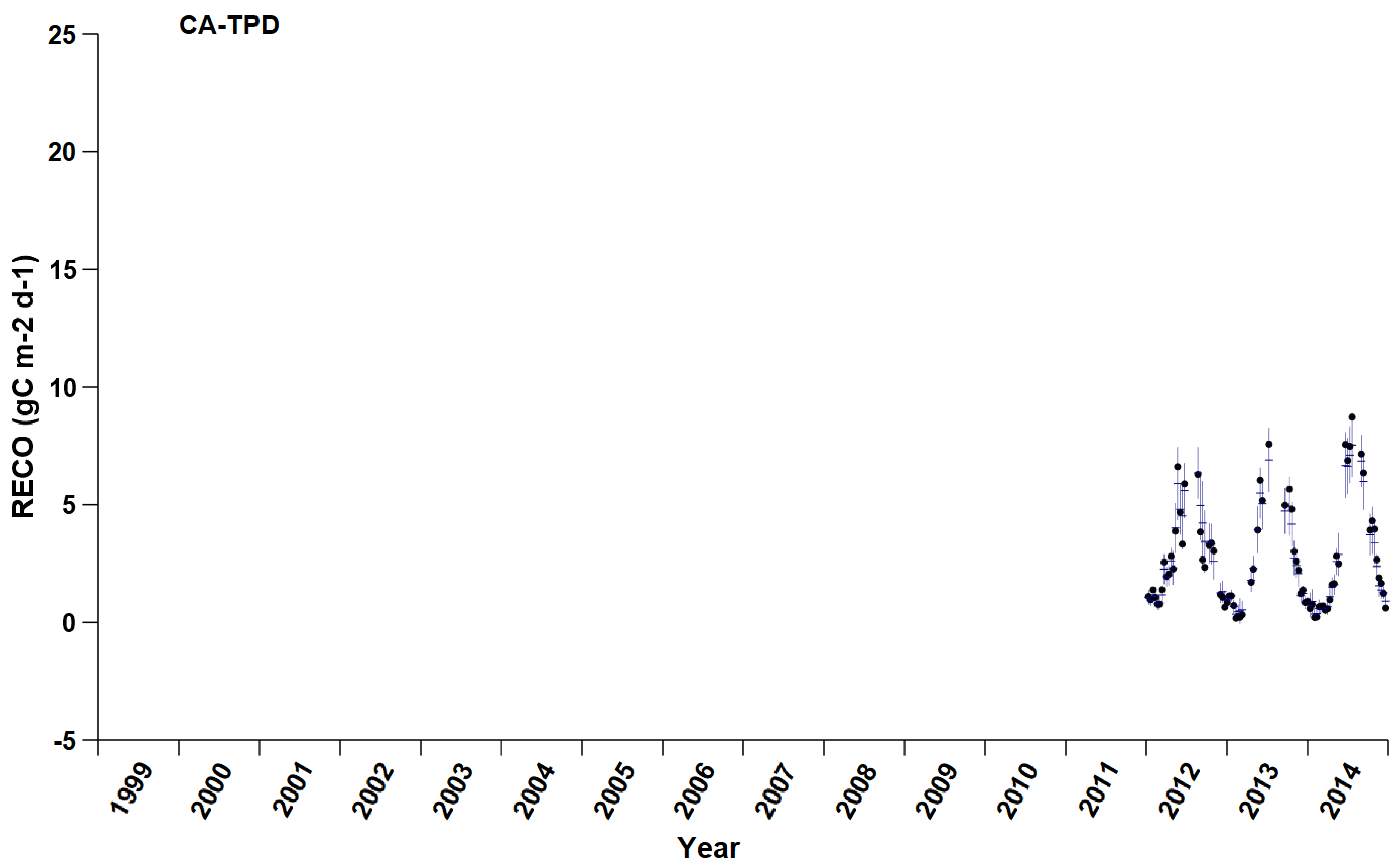

CA-TPD
RECO (gC m-2 d-1)
25
20
15
10
5
0
-5
1999
2000
2001
2002
2003
2004
2005
2006
2007
2008
2009
2010
2011
2012
2013
2014
Year

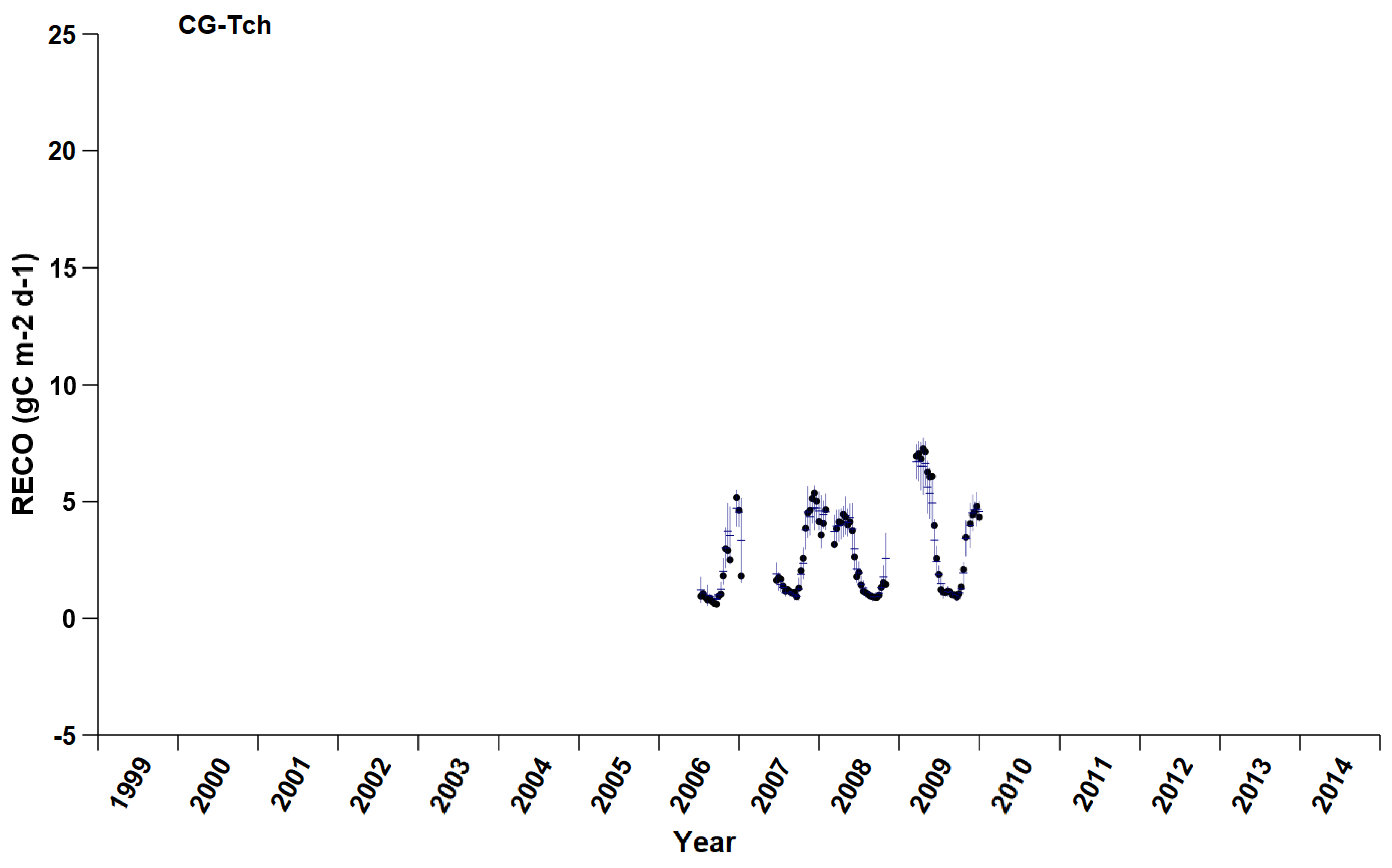

CG-Tch
RECO (gC m-2 d-1)
25
20
15
10
5
0
-5
1999
2000
2001
2002
2003
2004
2005
2006
2007
2008
2009
2010
2011
2012
2013
2014
Year

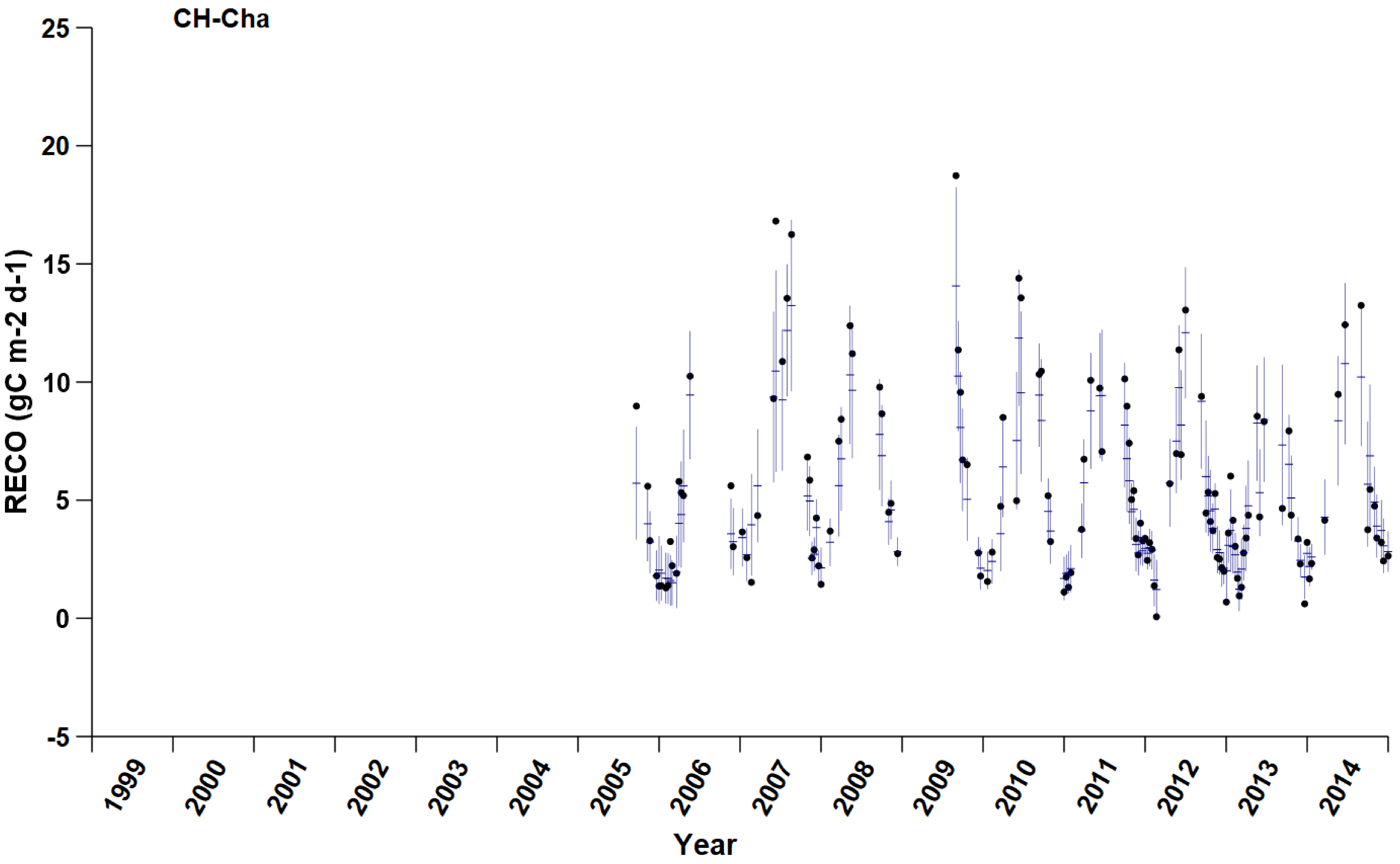
CH-Cha
RECO (gC m-2 d-1)
25
20
15
10
5
0
-5
1999
2000
2001
2002
2003
2004
2005
2006
2007
2008
2009
2010
2011
2012
2013
2014
Year

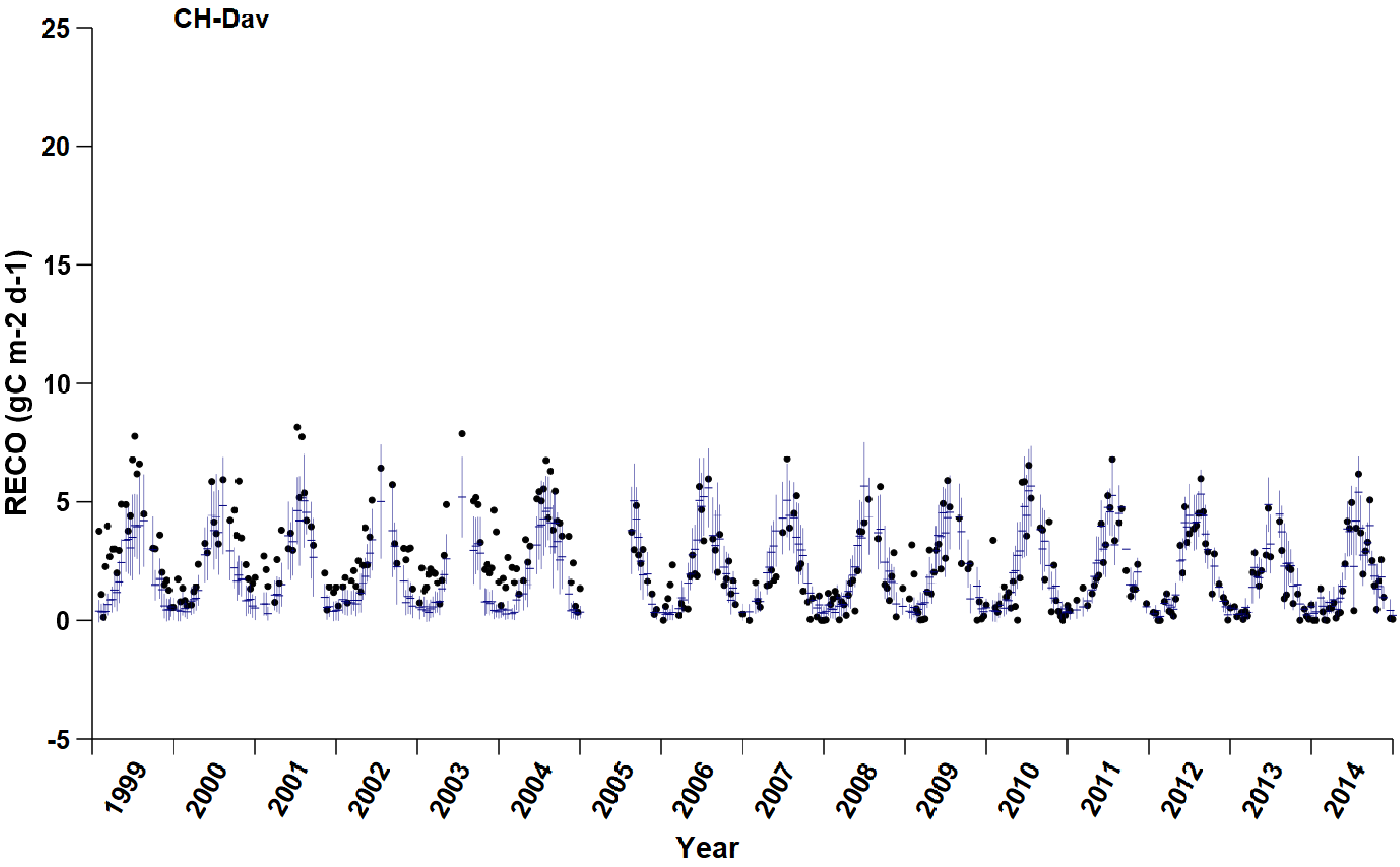
CH-Dav
RECO (gC m-2 d-1)
25
20
15
10
5
0
-5
1999
2000
2001
2002
2003
2004
2005
2006
2007
2008
2009
2010
2011
2012
2013
2014
Year

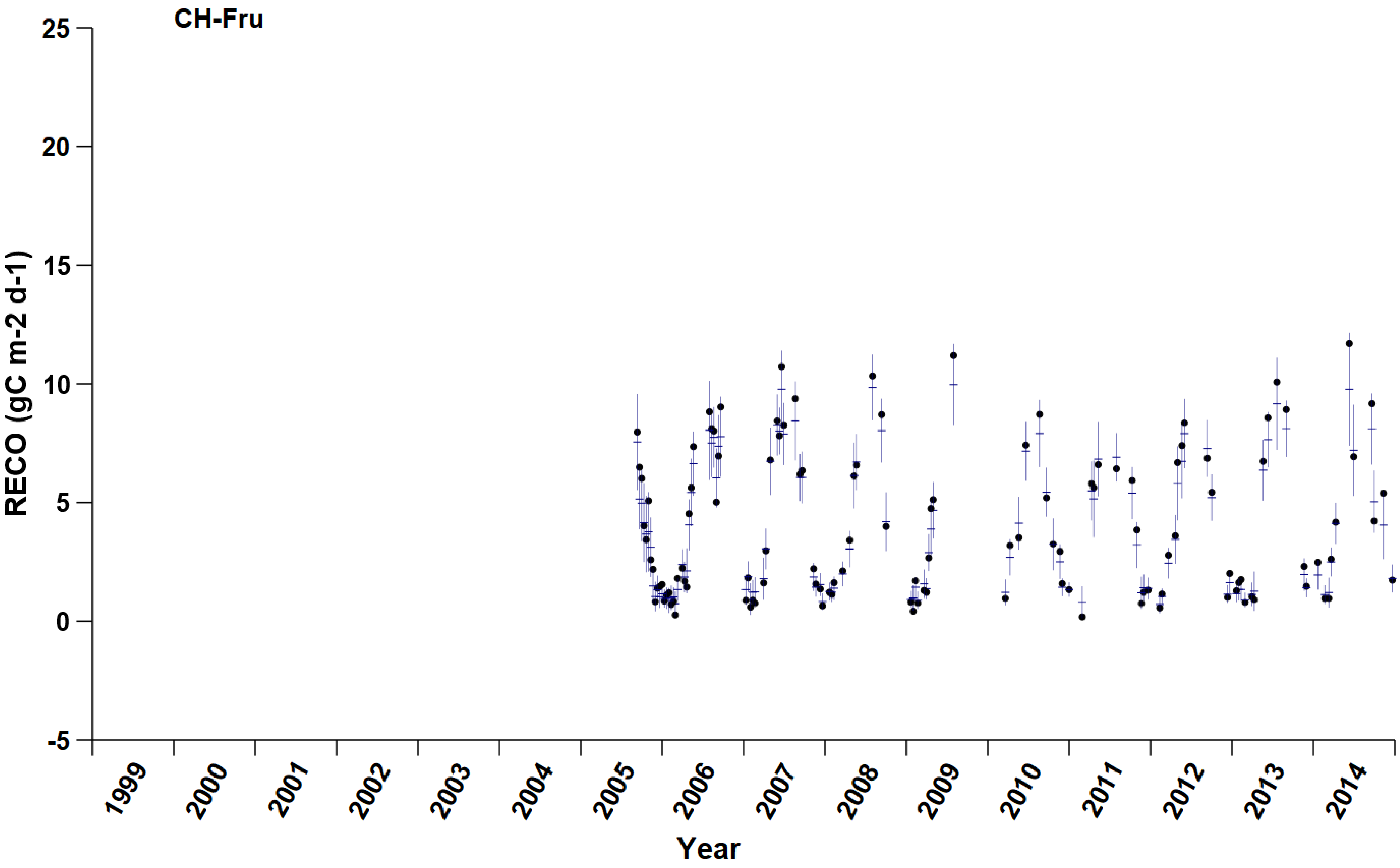

CH-Fru
RECO (gC m-2 d-1)
25
20
15
10
5
0
-5
1999
2000
2001
2002
2003
2004
2005
2006
2007
2008
2009
2010
2011
2012
2013
2014
Year

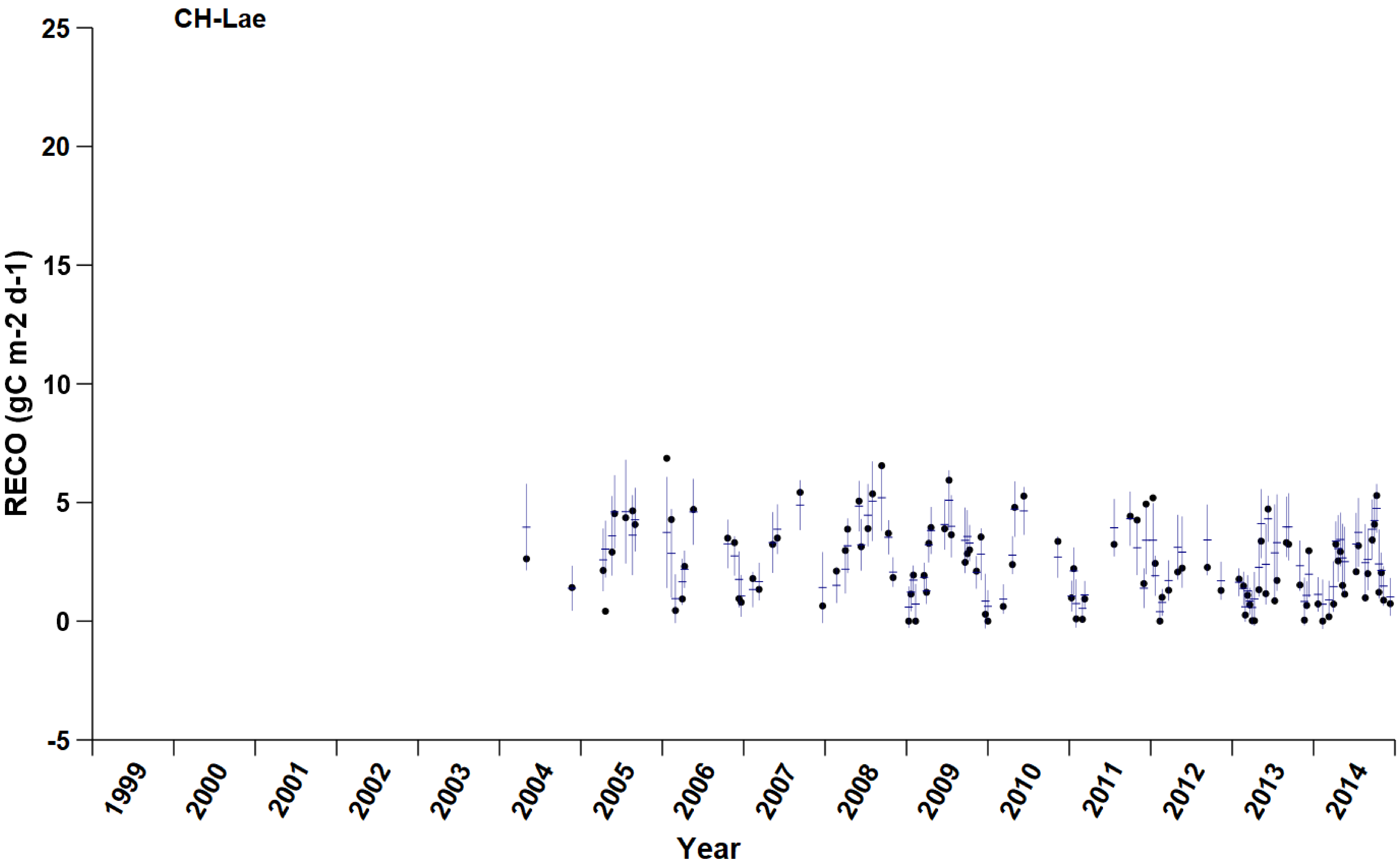
CH-Lae
RECO (gC m-2 d-1)
25
20
15
10
5
0
-5
1999
2000
2001
2002
2003
2004
2005
2006
2007
2008
2009
2010
2011
2012
2013
2014
Year

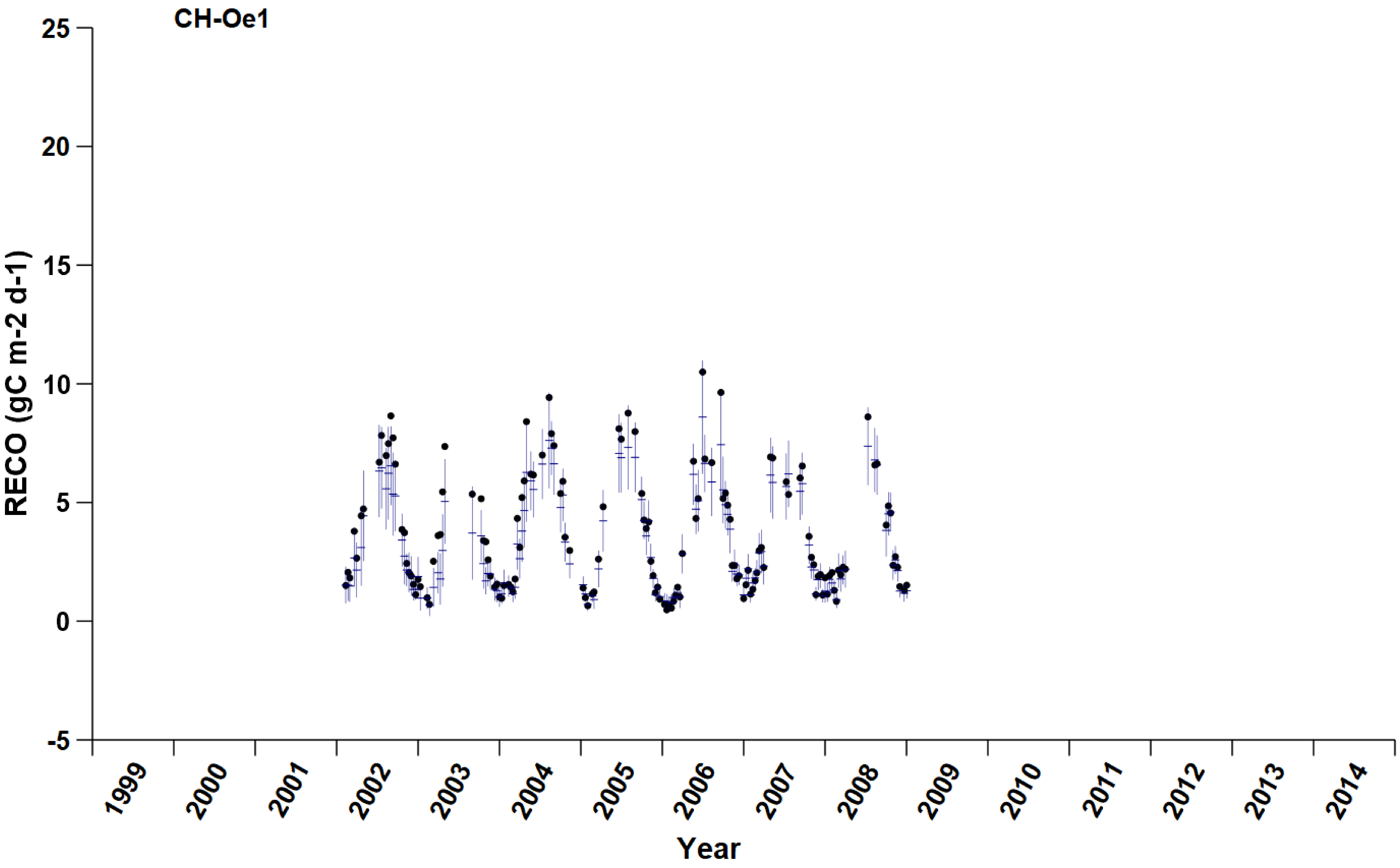
CH-Oe1
RECO (gC m-2 d-1)
25
20
15
10
5
0
-5
1999
2000
2001
2002
2003
2004
2005
2006
2007
2008
2009
2010
2011
2012
2013
2014
Year

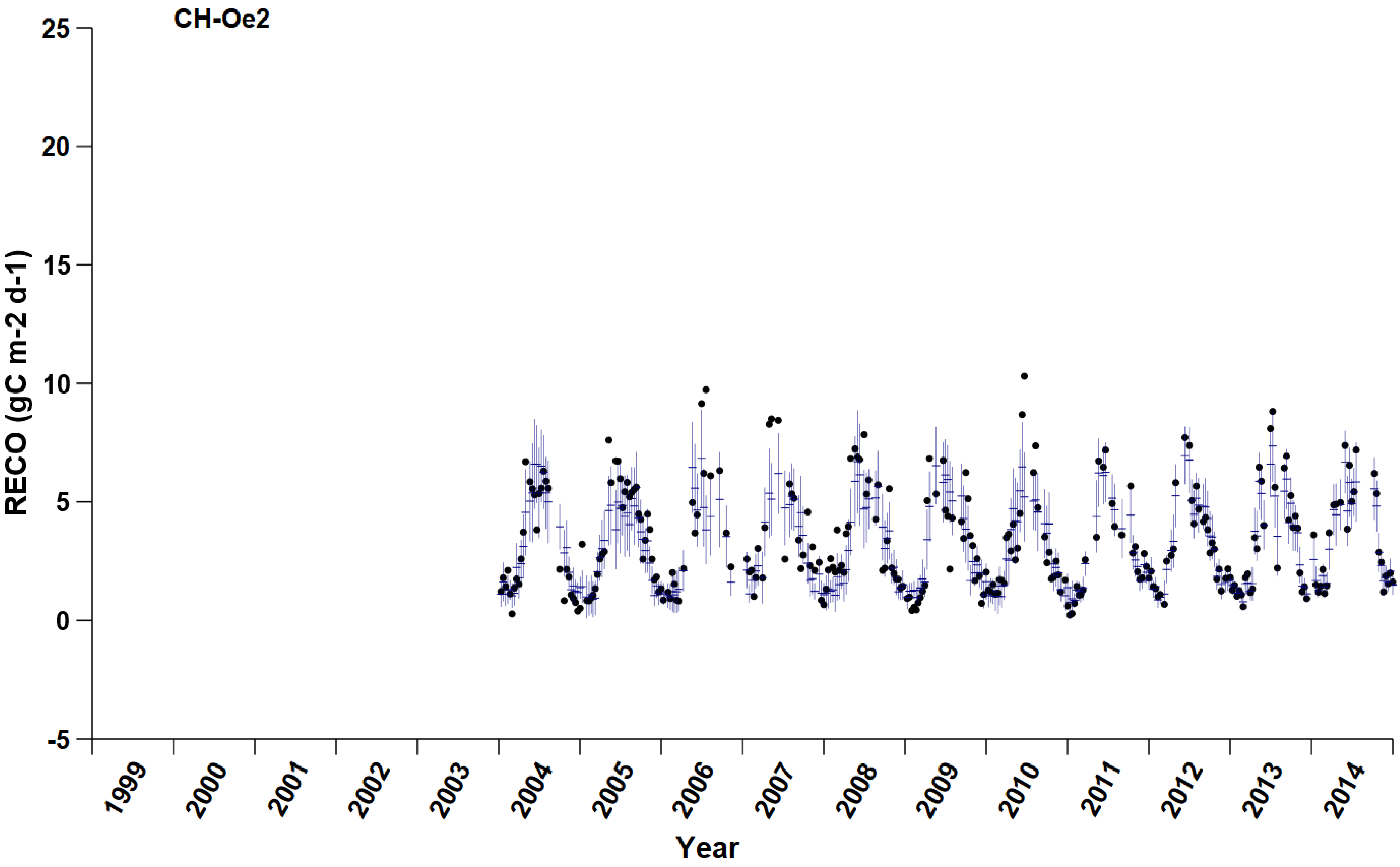
CH-Oe2
RECO (gC m-2 d-1)
25
20
15
10
5
0
-5
1999
2000
2001
2002
2003
2004
2005
2006
2007
2008
2009
2010
2011
2012
2013
2014
Year

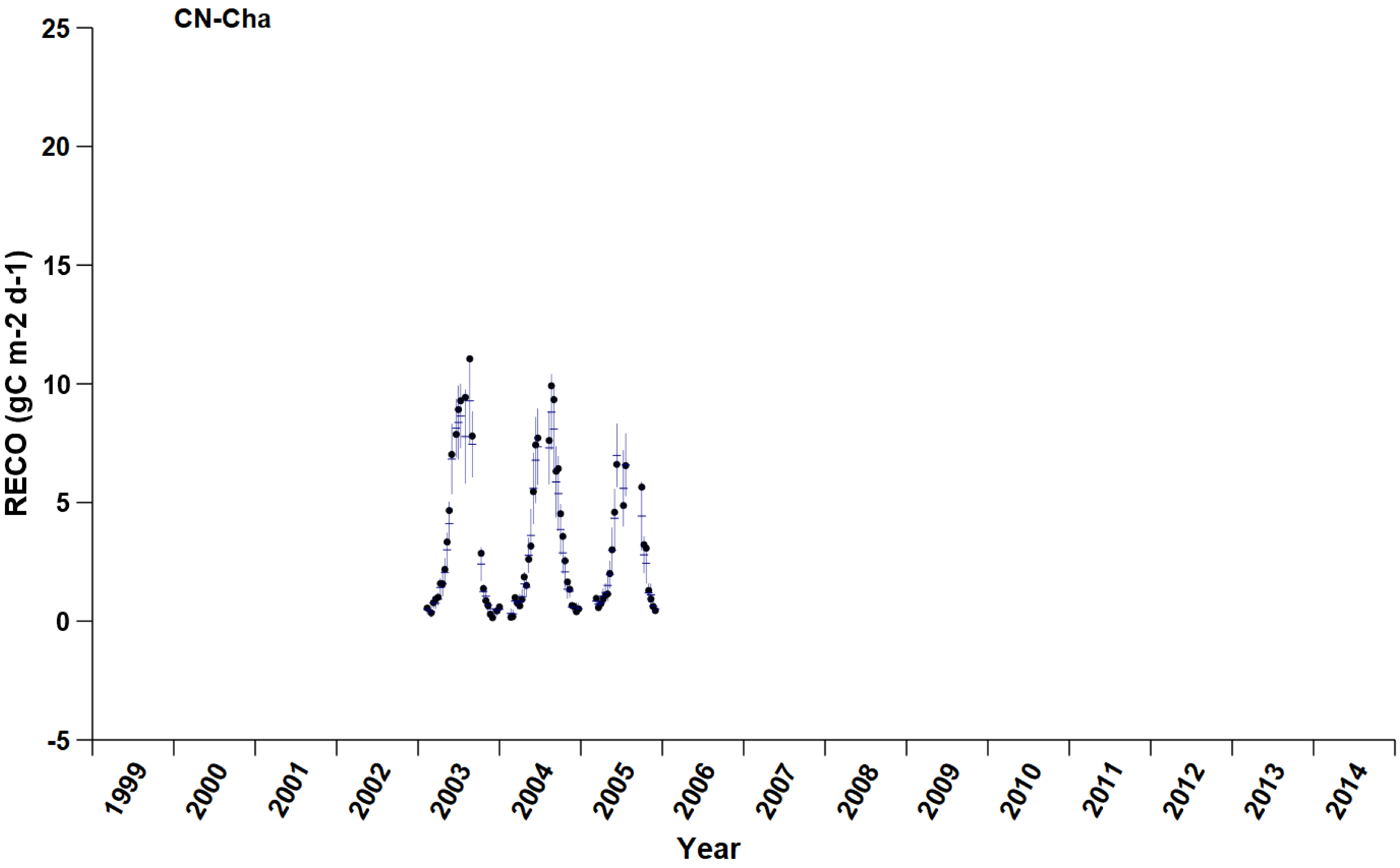

CN-Cha
RECO (gC m-2 d-1)
25
20
15
10
5
0
-5
1999
2000
2001
2002
2003
2004
2005
2006
2007
2008
2009
2010
2011
2012
2013
2014
Year

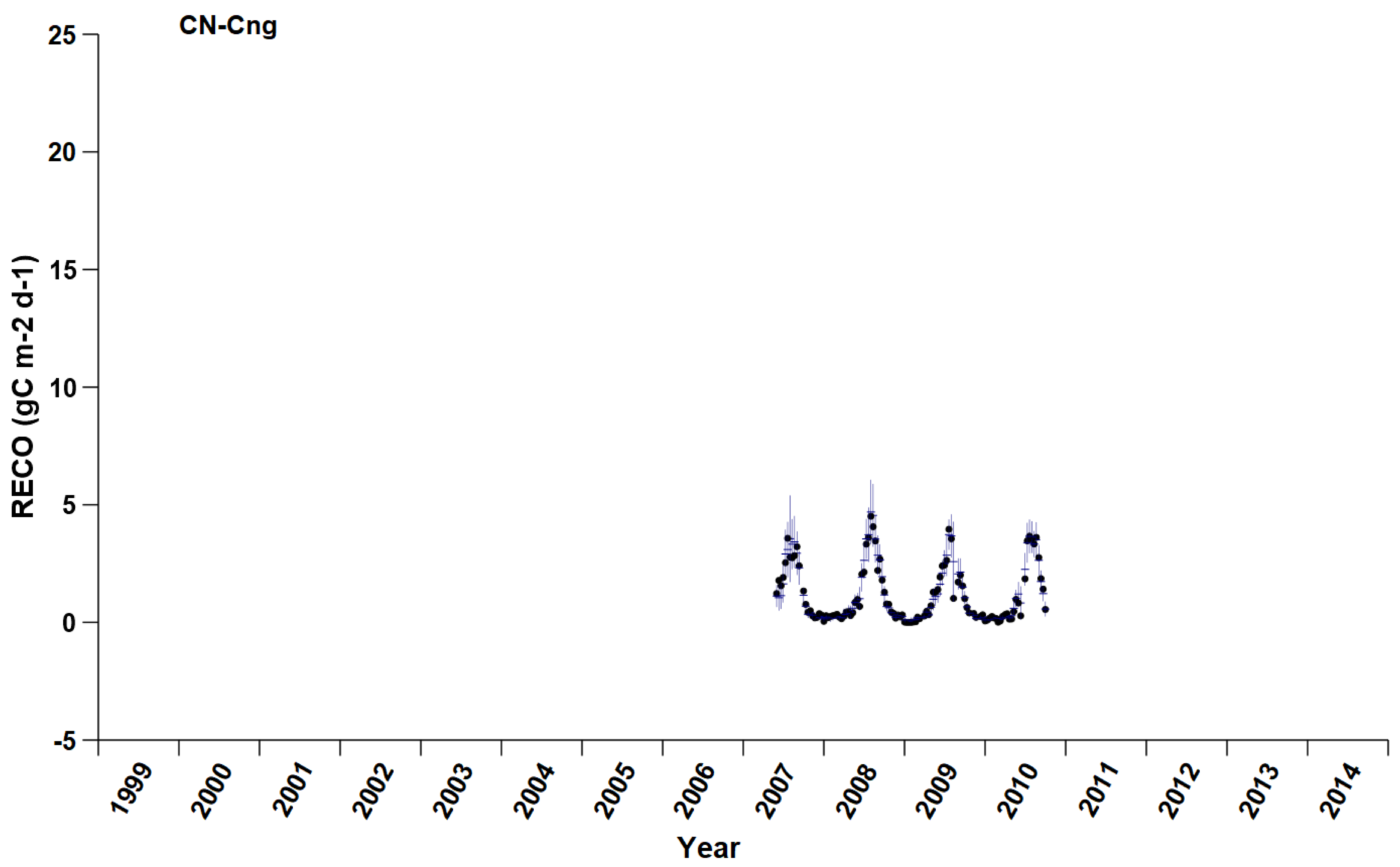
CN-Cng
RECO (gC m-2 d-1)
25
20
15
10
5
0
-5
1999
2000
2001
2002
2003
2004
2005
2006
2007
2008
2009
2010
2011
2012
2013
2014
Year

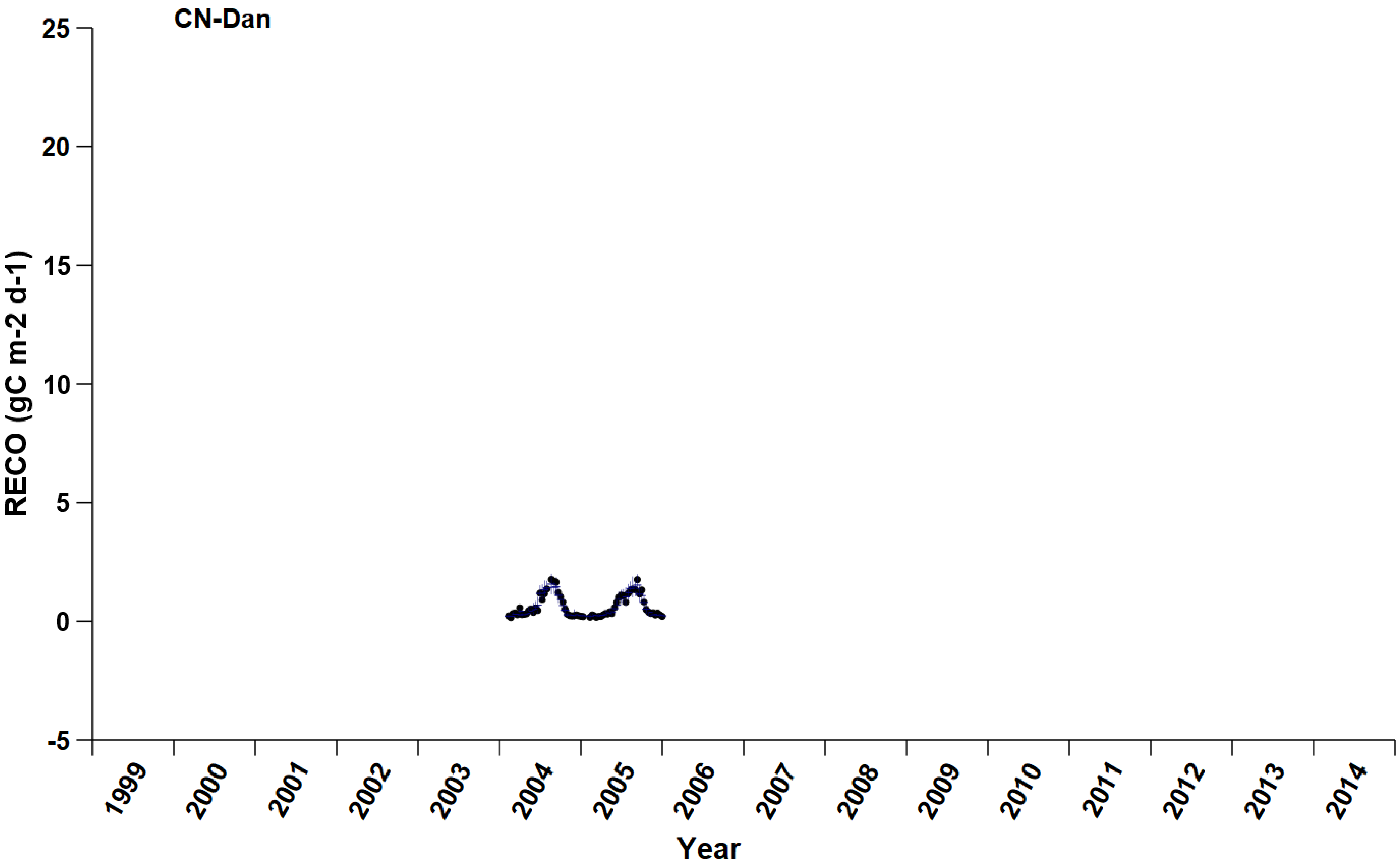
CN-Dan
25
20
15
10
5
0
-5
RECO (gC m-2 d-1)
1999
2000
2001
2002
2003
2004
2005
2006
2007
2008
2009
2010
2011
2012
2013
2014
Year

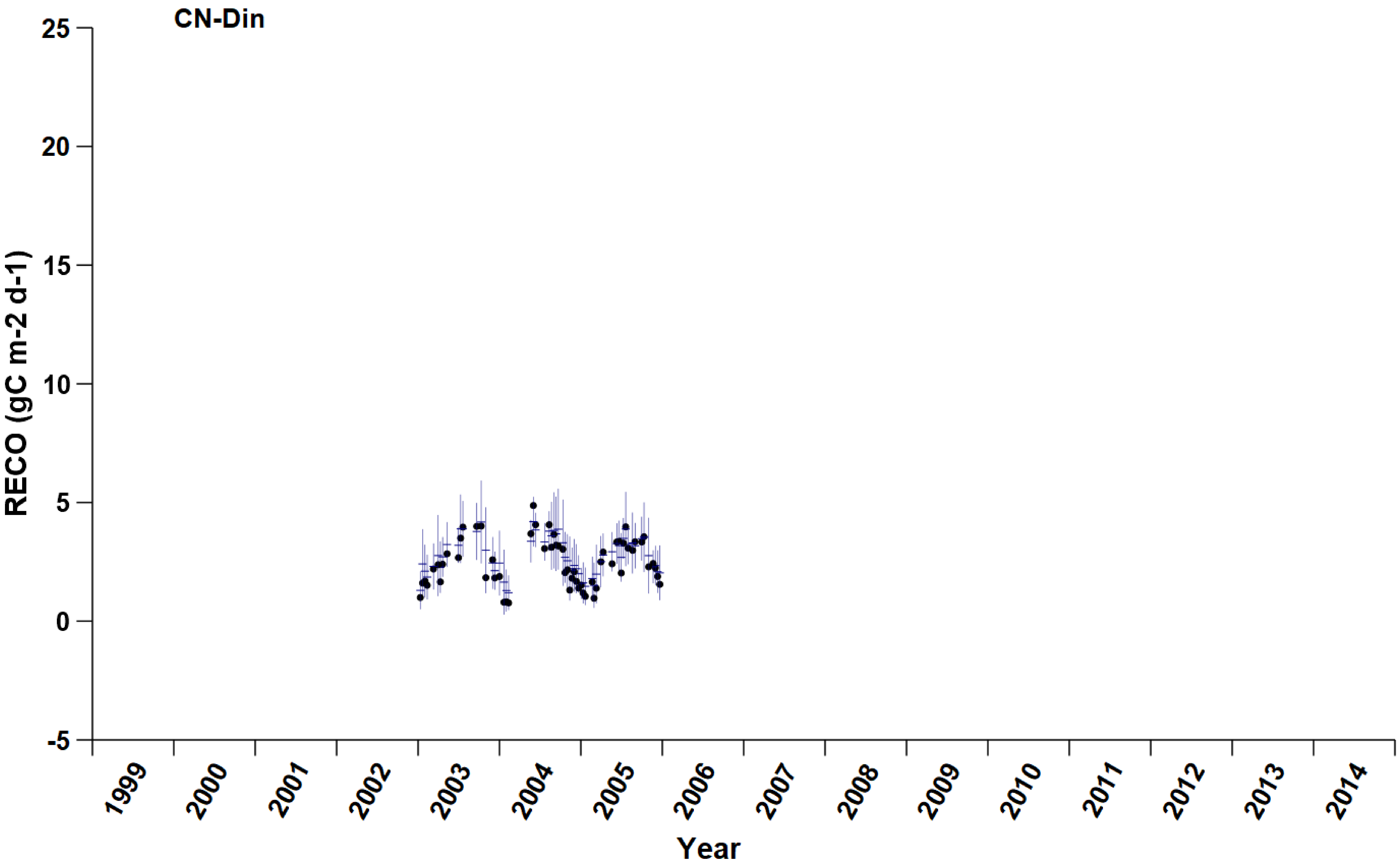
CN-Din
RECO (gC m-2 d-1)
25
20
15
10
5
0
-5
1999
2000
2001
2002
2003
2004
2005
2006
2007
2008
2009
2010
2011
2012
2013
2014
Year

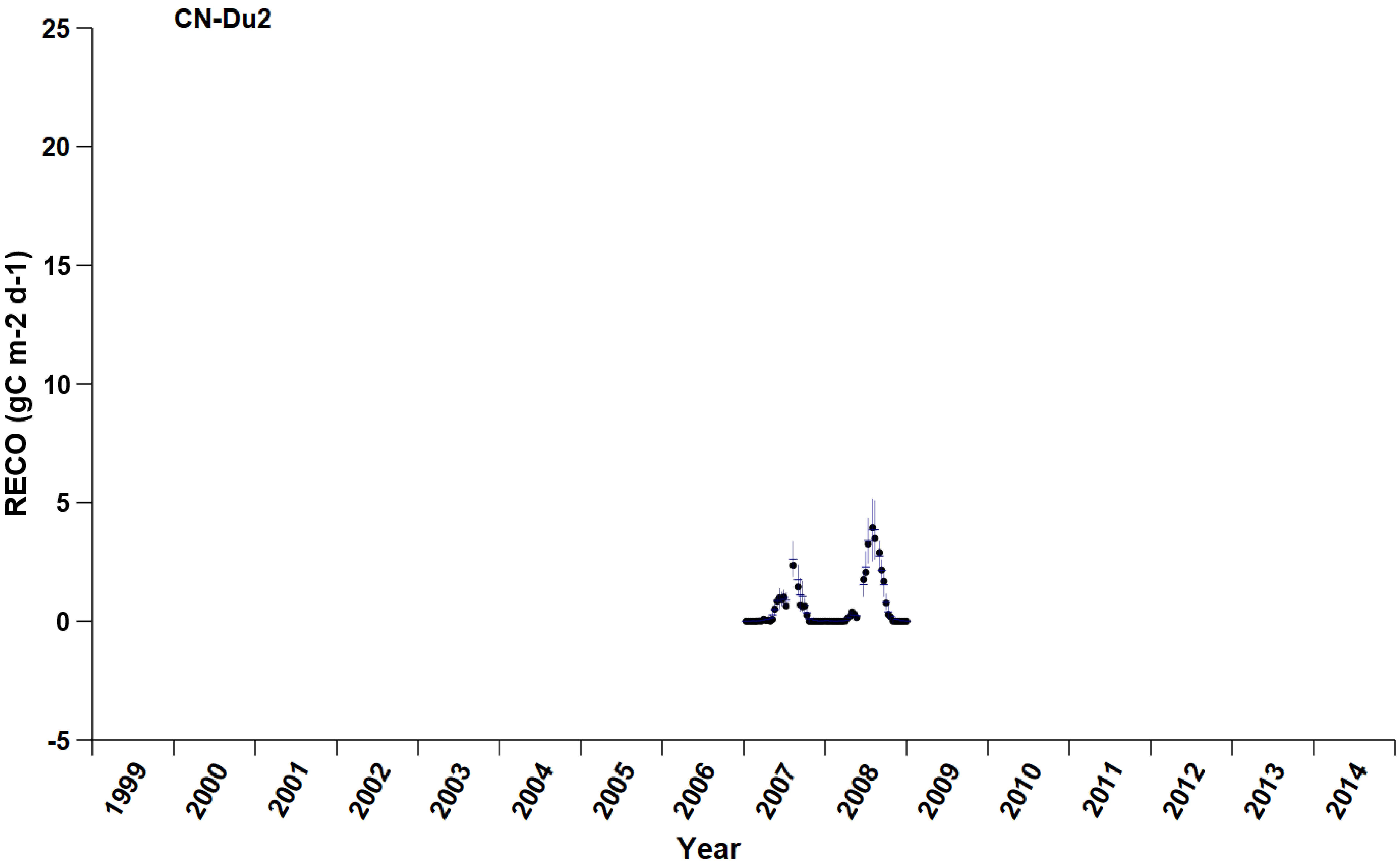
CN-Du2
RECO (gC m-2 d-1)
25
20
15
10
5
0
-5
1999
2000
2001
2002
2003
2004
2005
2006
2007
2008
2009
2010
2011
2012
2013
2014
Year

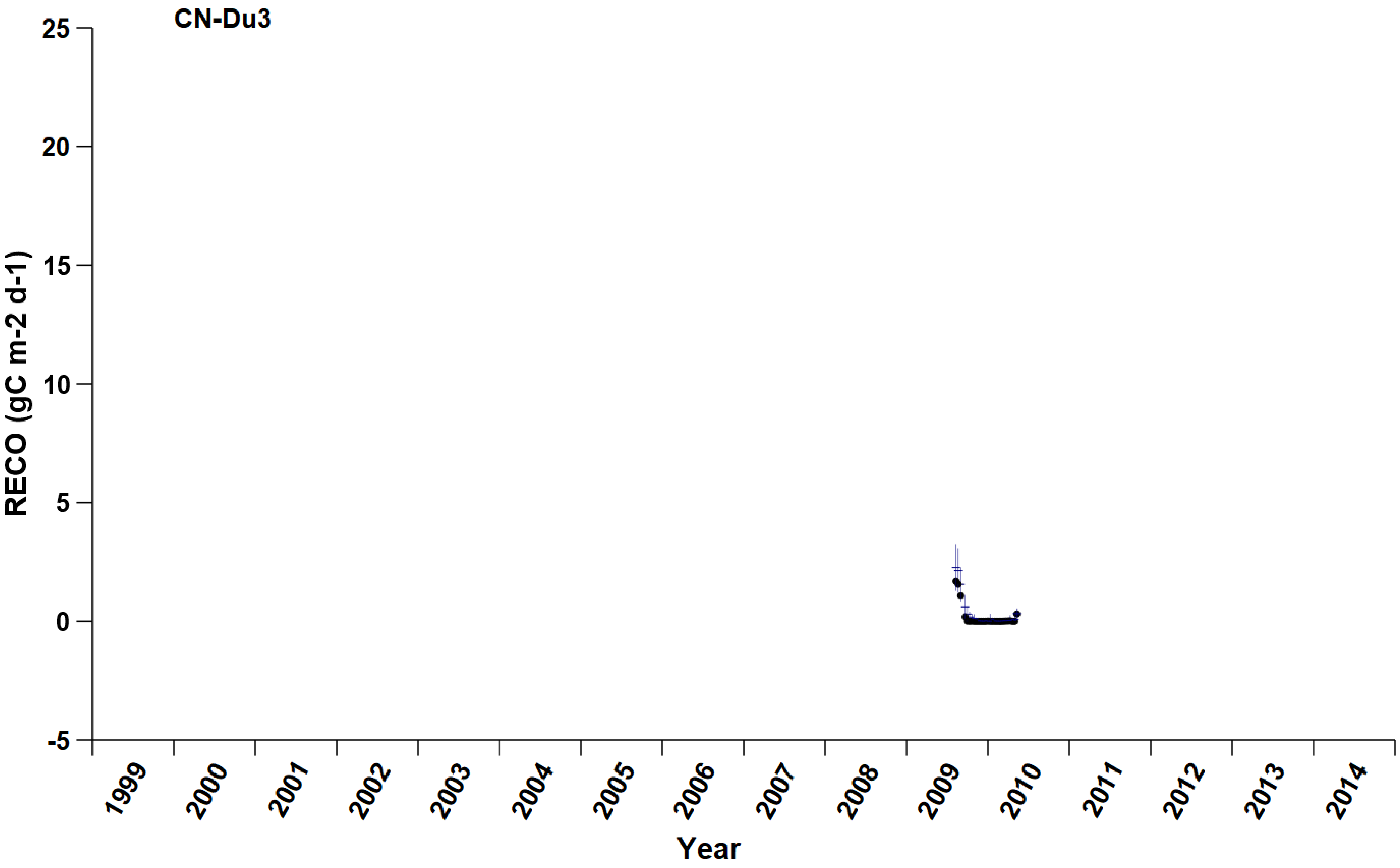
CN-Du3
RECO (gC m-2 d-1)
25
20
15
10
5
0
-5
1999
2000
2001
2002
2003
2004
2005
2006
2007
2008
2009
2010
2011
2012
2013
2014
Year

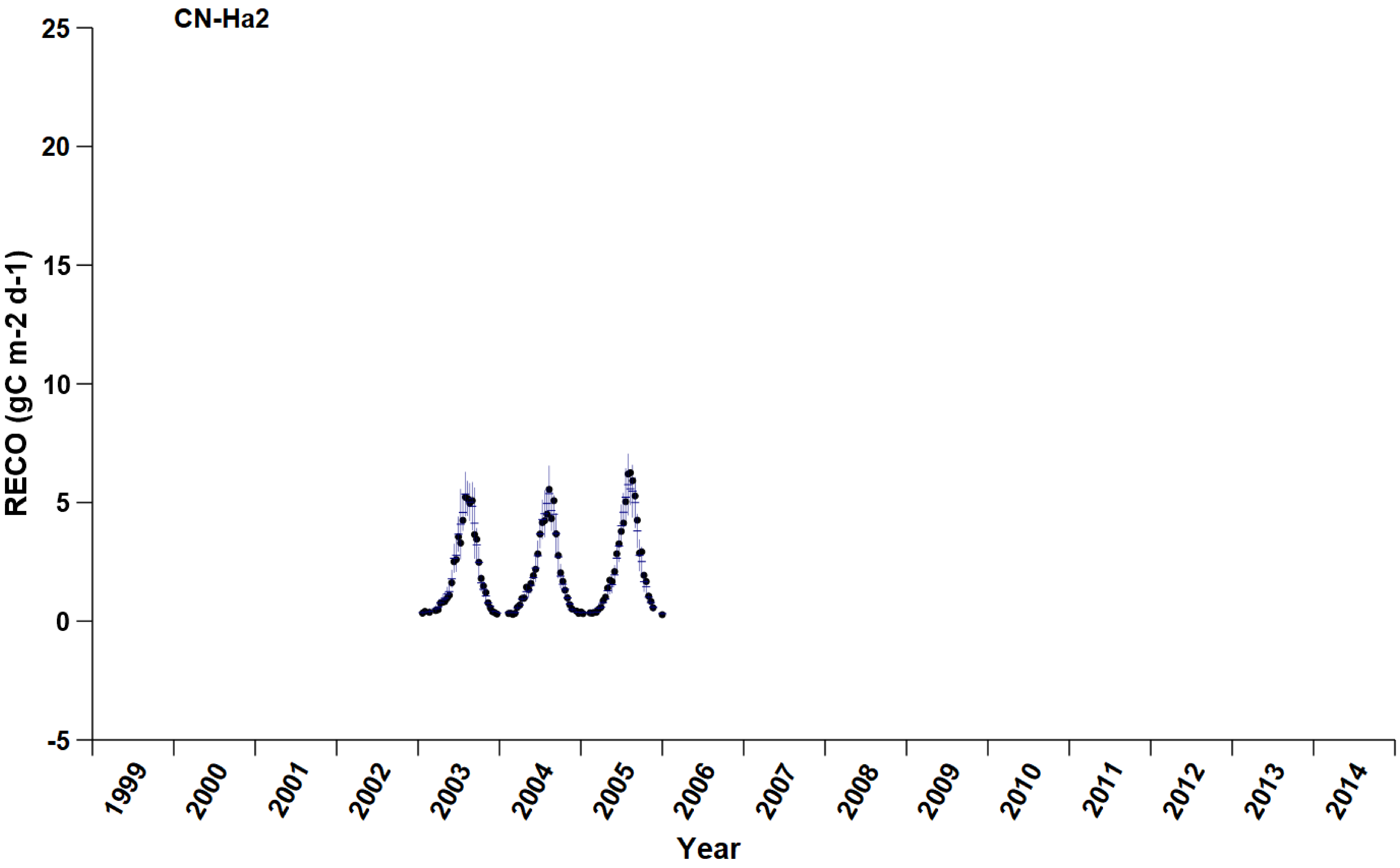

CN-Ha2
RECO (gC m-2 d-1)
25
20
15
10
5
0
-5
1999
2000
2001
2002
2003
2004
2005
2006
2007
2008
2009
2010
2011
2012
2013
2014
Year

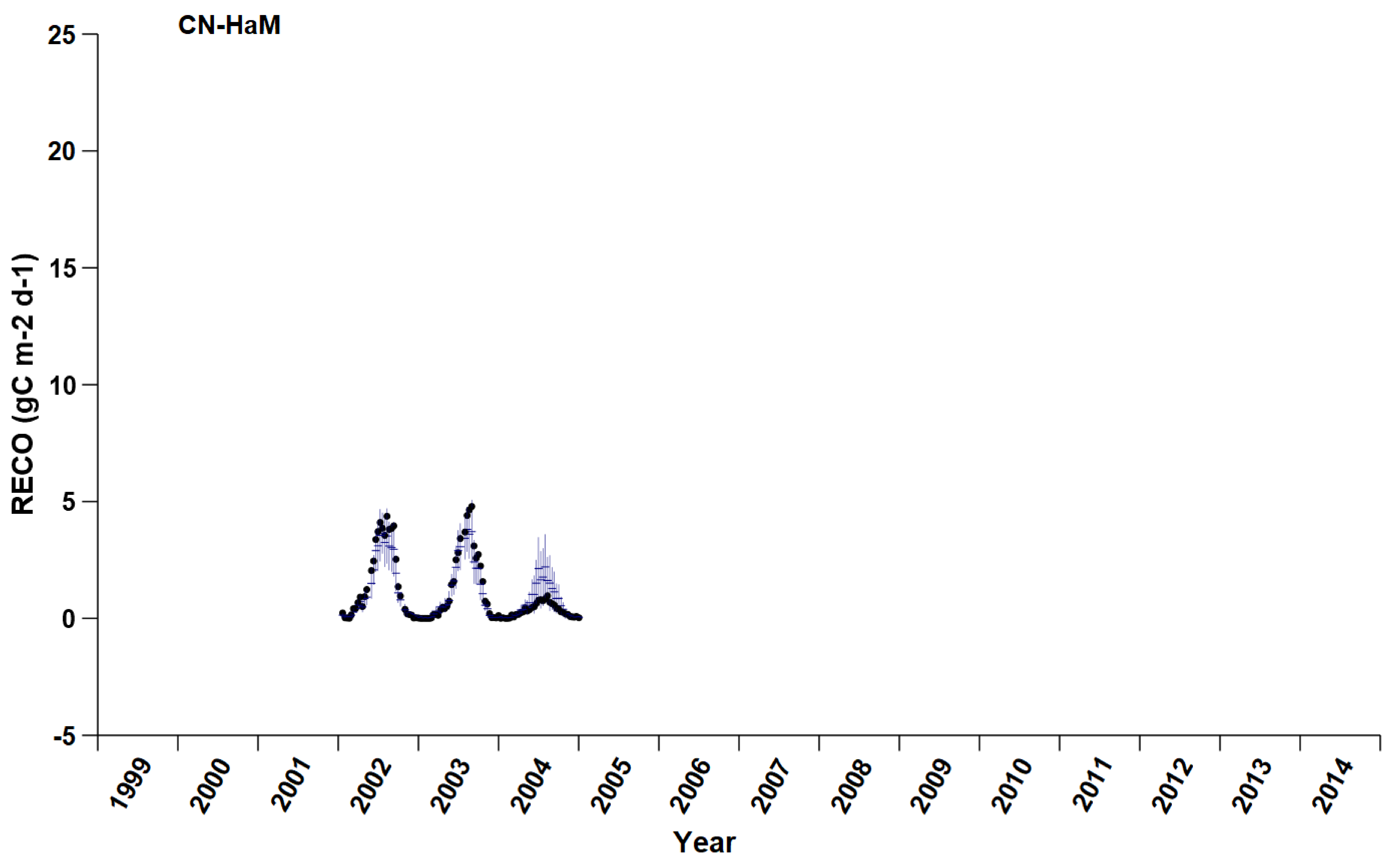

CN-HaM
RECO (gC m-2 d-1)
25
20
15
10
5
0
-5
1999
2000
2001
2002
2003
2004
2005
2006
2007
2008
2009
2010
2011
2012
2013
2014
Year

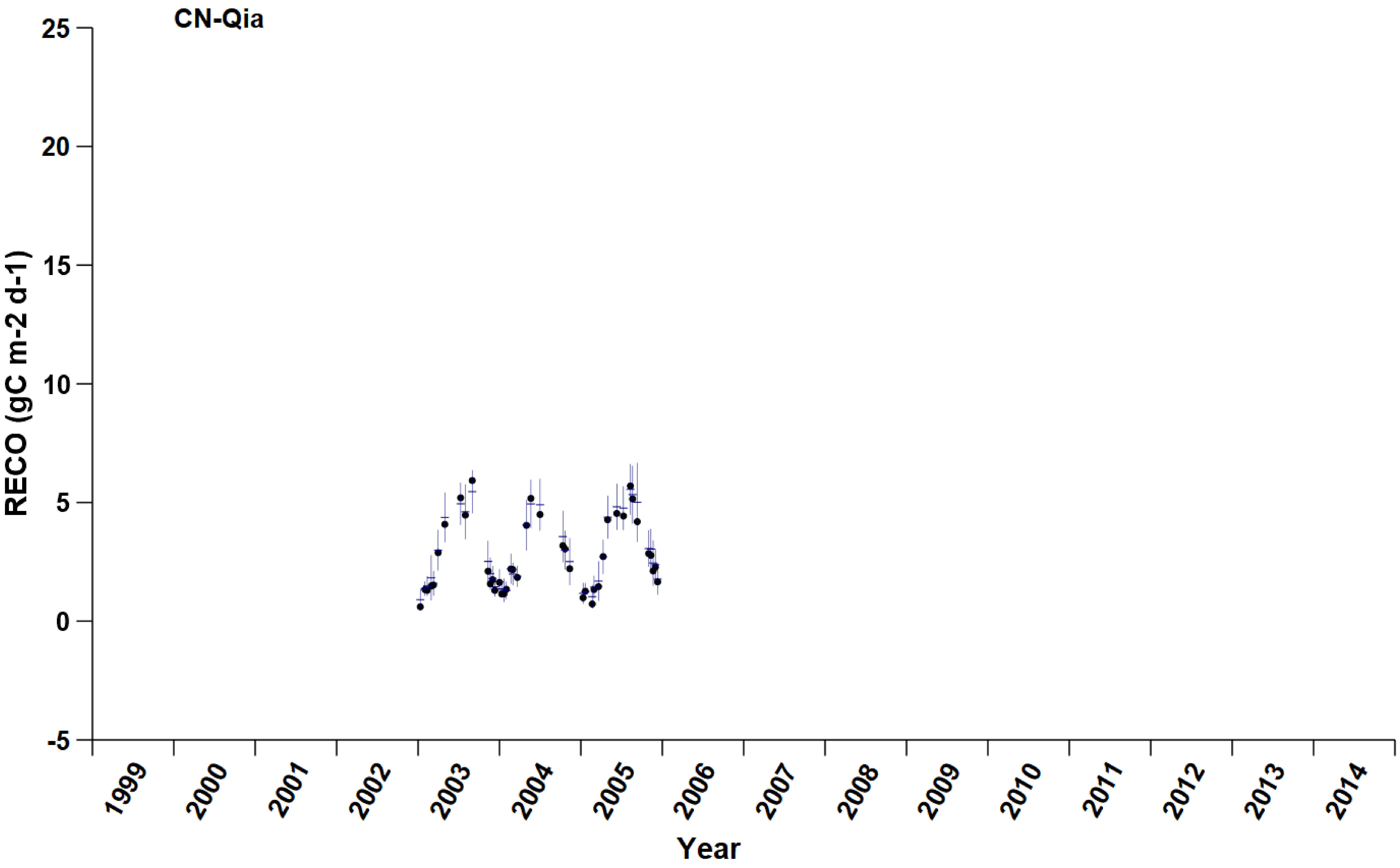

CN-Qia
25
20
15
10
5
0
-5
RECO (gC m-2 d-1)
1999
2000
2001
2002
2003
2004
2005
2006
2007
2008
2009
2010
2011
2012
2013
2014
Year

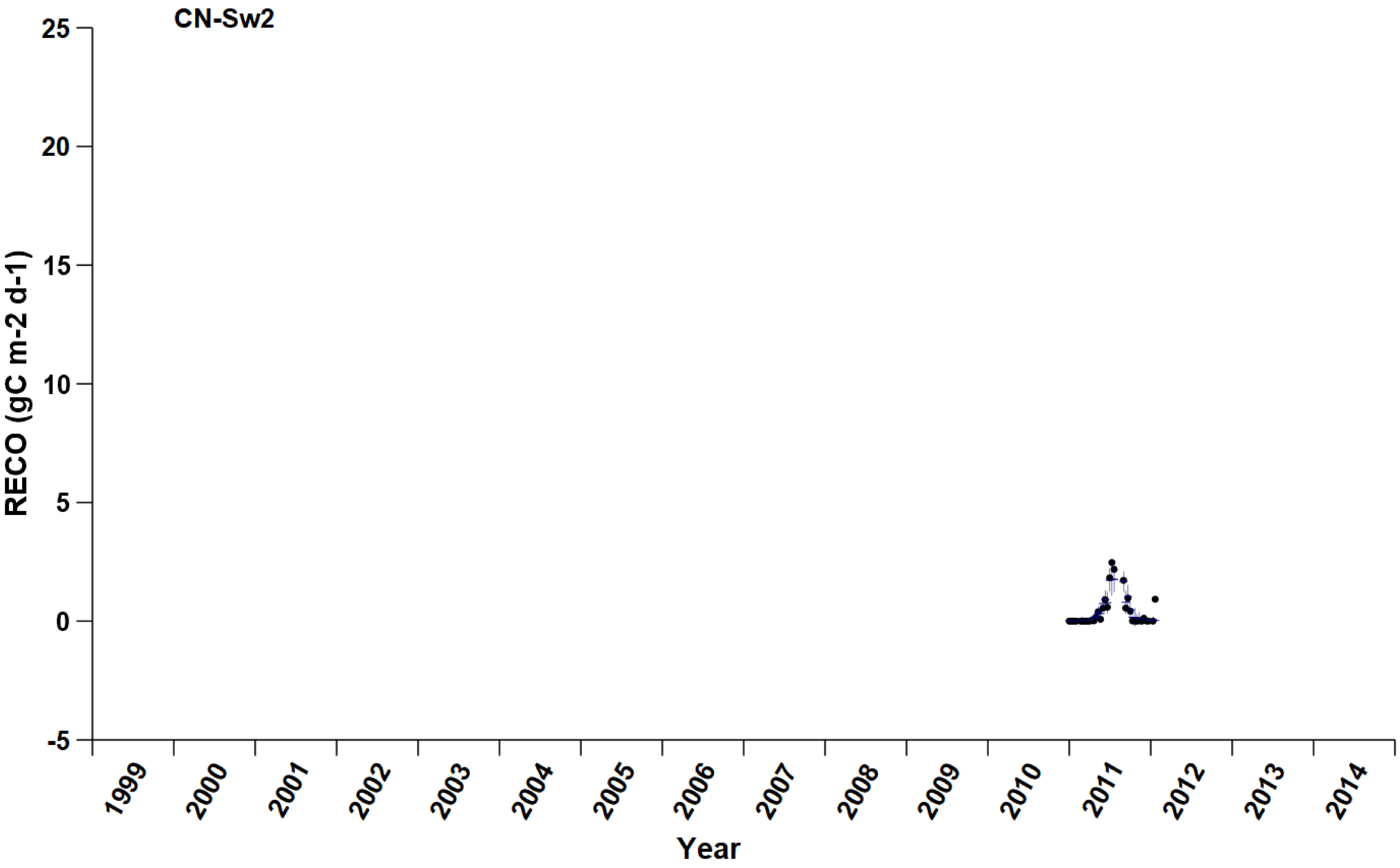
CN-Sw2
RECO (gC m-2 d-1)
25
20
15
10
5
0
-5
1999
2000
2001
2002
2003
2004
2005
2006
2007
2008
2009
2010
2011
2012
2013
2014
Year

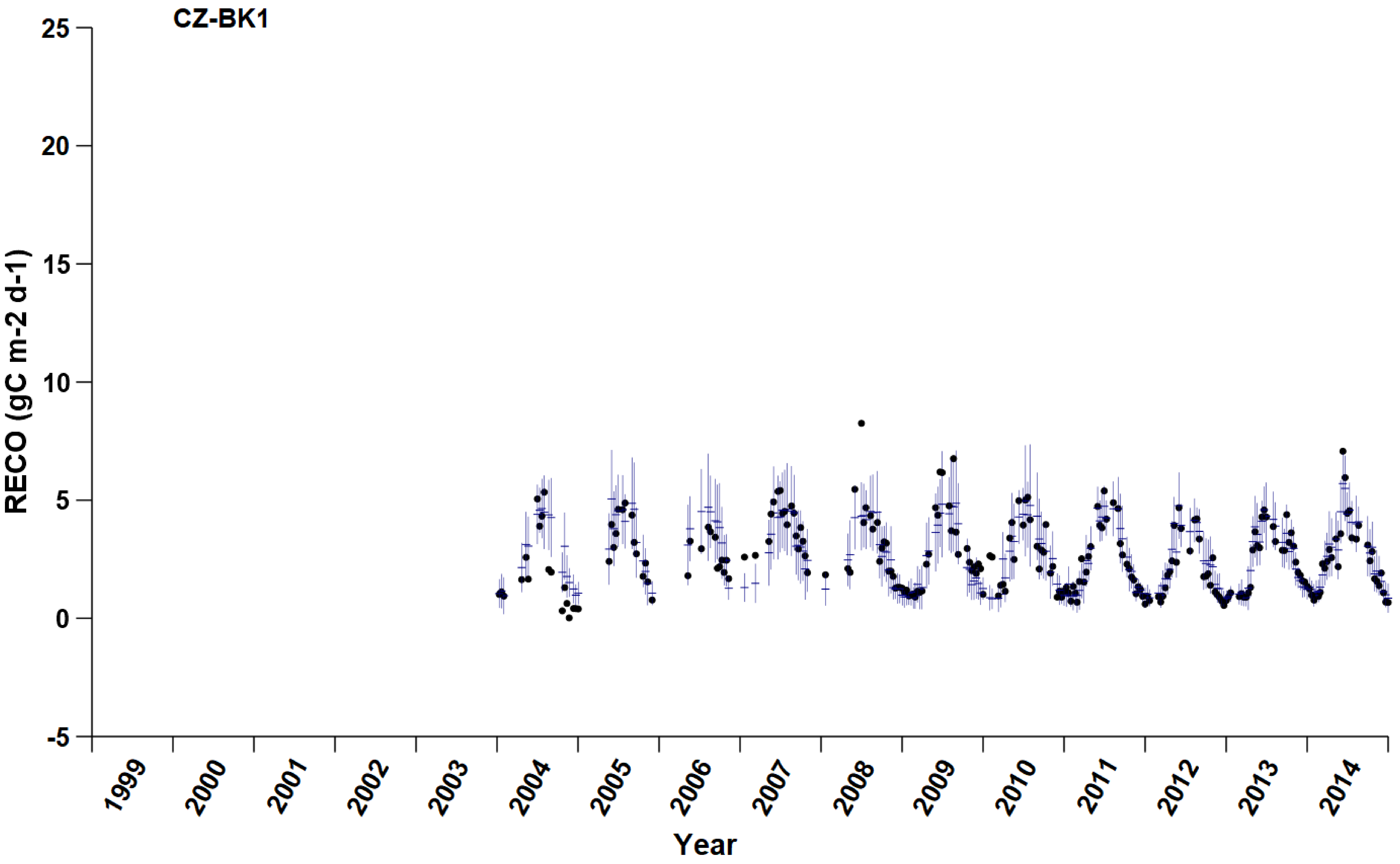
CZ-BK1
RECO (gC m-2 d-1)
25
20
15
10
5
0
-5
1999
2000
2001
2002
2003
2004
2005
2006
2007
2008
2009
2010
2011
2012
2013
2014
Year

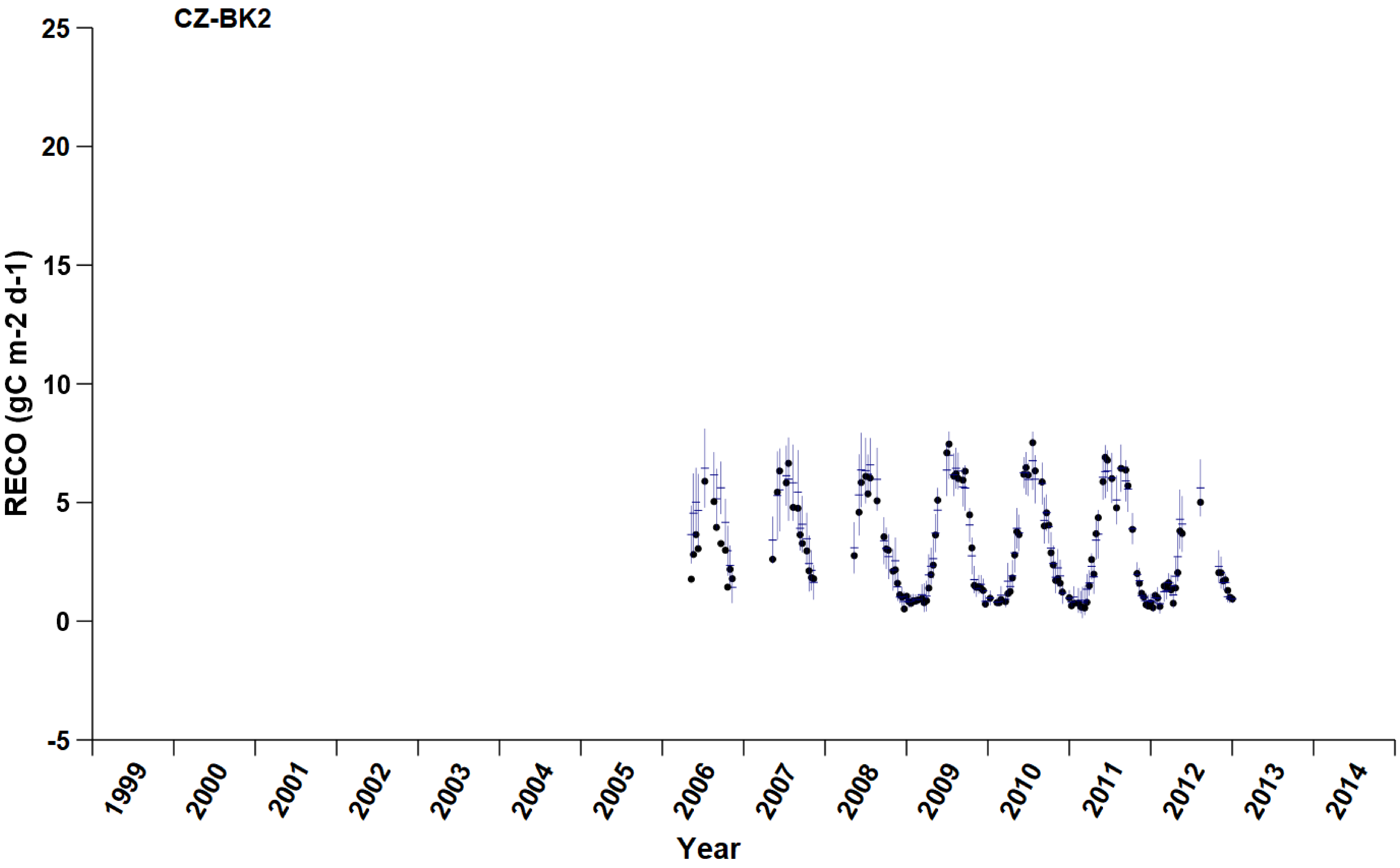
CZ-BK2
RECO (gC m-2 d-1)
25
20
15
10
5
0
-5
1999
2000
2001
2002
2003
2004
2005
2006
2007
2008
2009
2010
2011
2012
2013
2014
Year

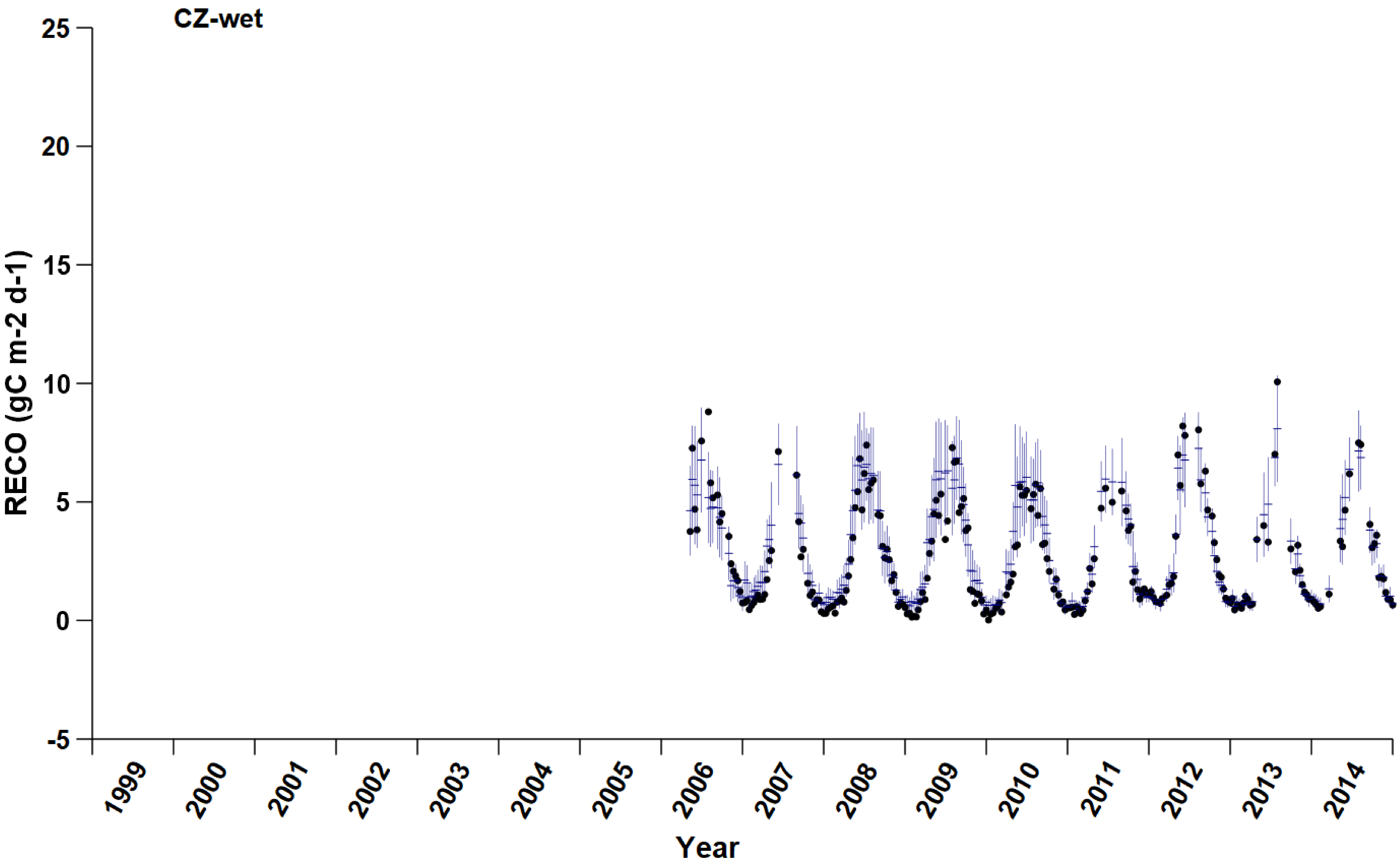
CZ-wet
RECO (gC m-2 d-1)
25
20
15
10
5
0
-5
1999
2000
2001
2002
2003
2004
2005
2006
2007
2008
2009
2010
2011
2012
2013
2014
Year

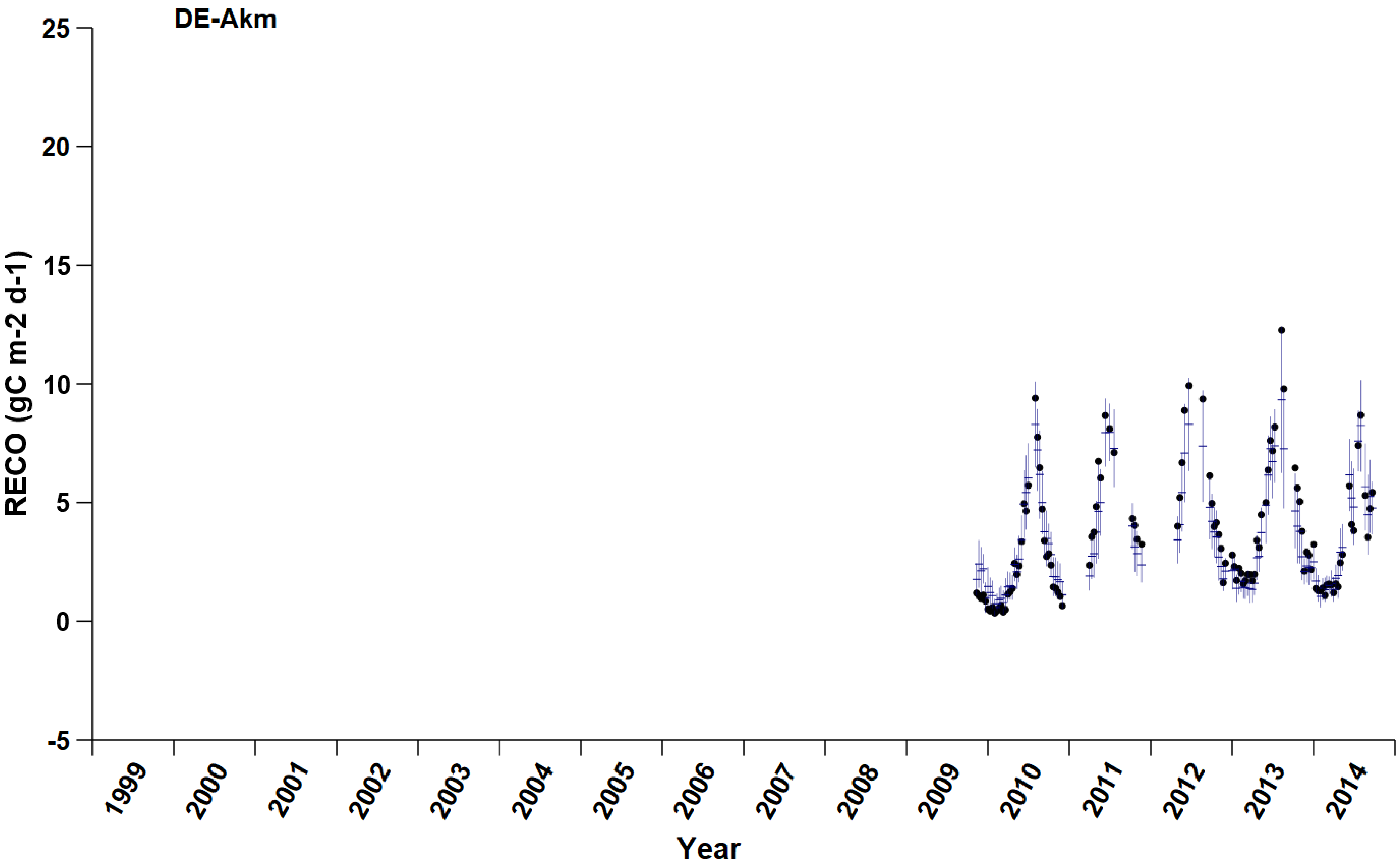
DE-Akm
RECO (gC m-2 d-1)
25
20
15
10
5
0
-5
1999
2000
2001
2002
2003
2004
2005
2006
2007
2008
2009
2010
2011
2012
2013
2014
Year

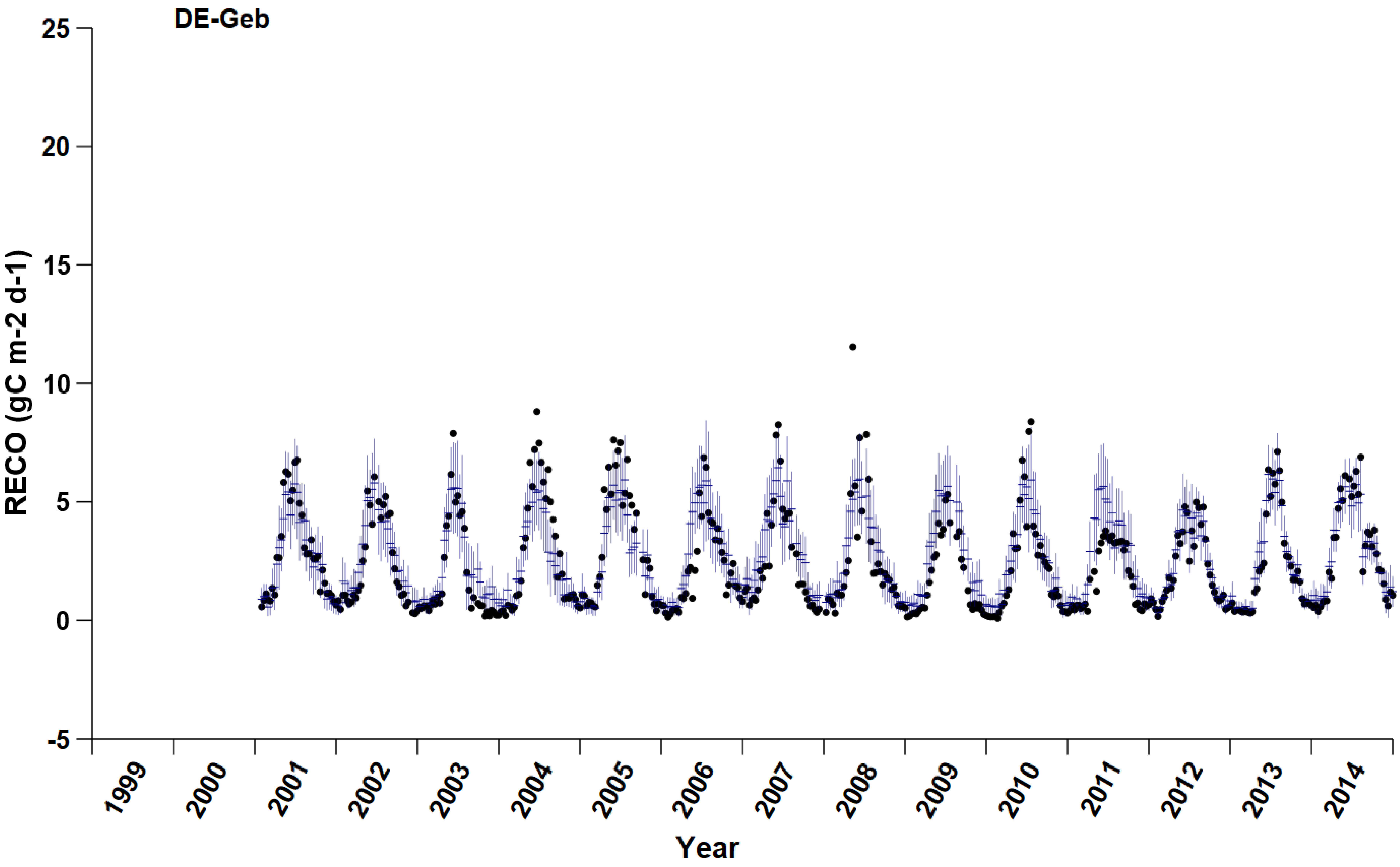

DE-Geb
25
20
15
10
5
0
-5
RECO (gC m-2 d-1)
1999
2000
2001
2002
2003
2004
2005
2006
2007
2008
2009
2010
2011
2012
2013
2014
Year

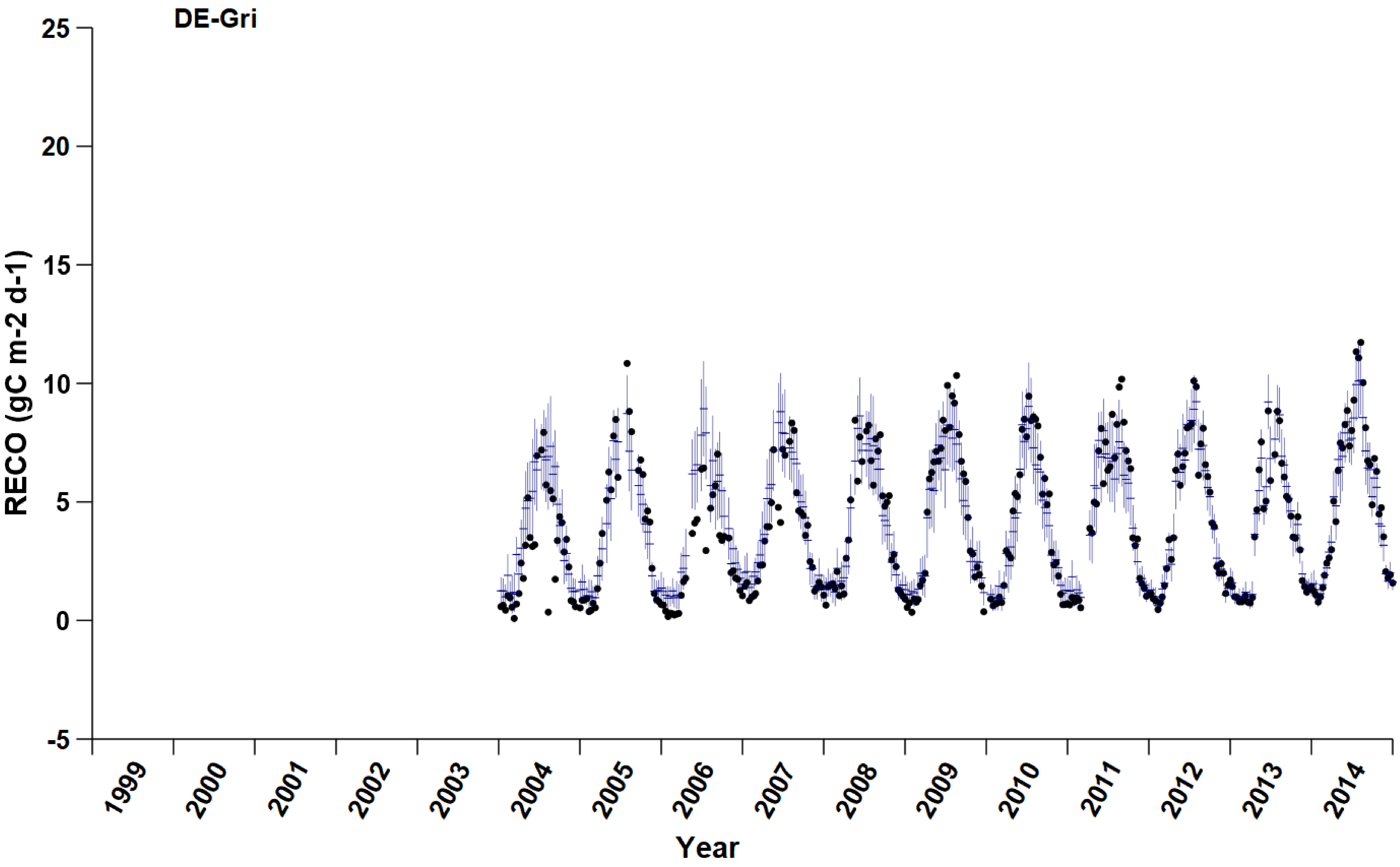
DE-Gri
RECO (gC m-2 d-1)
25
20
15
10
5
0
-5
1999
2000
2001
2002
2003
2004
2005
2006
2007
2008
2009
2010
2011
2012
2013
2014
Year

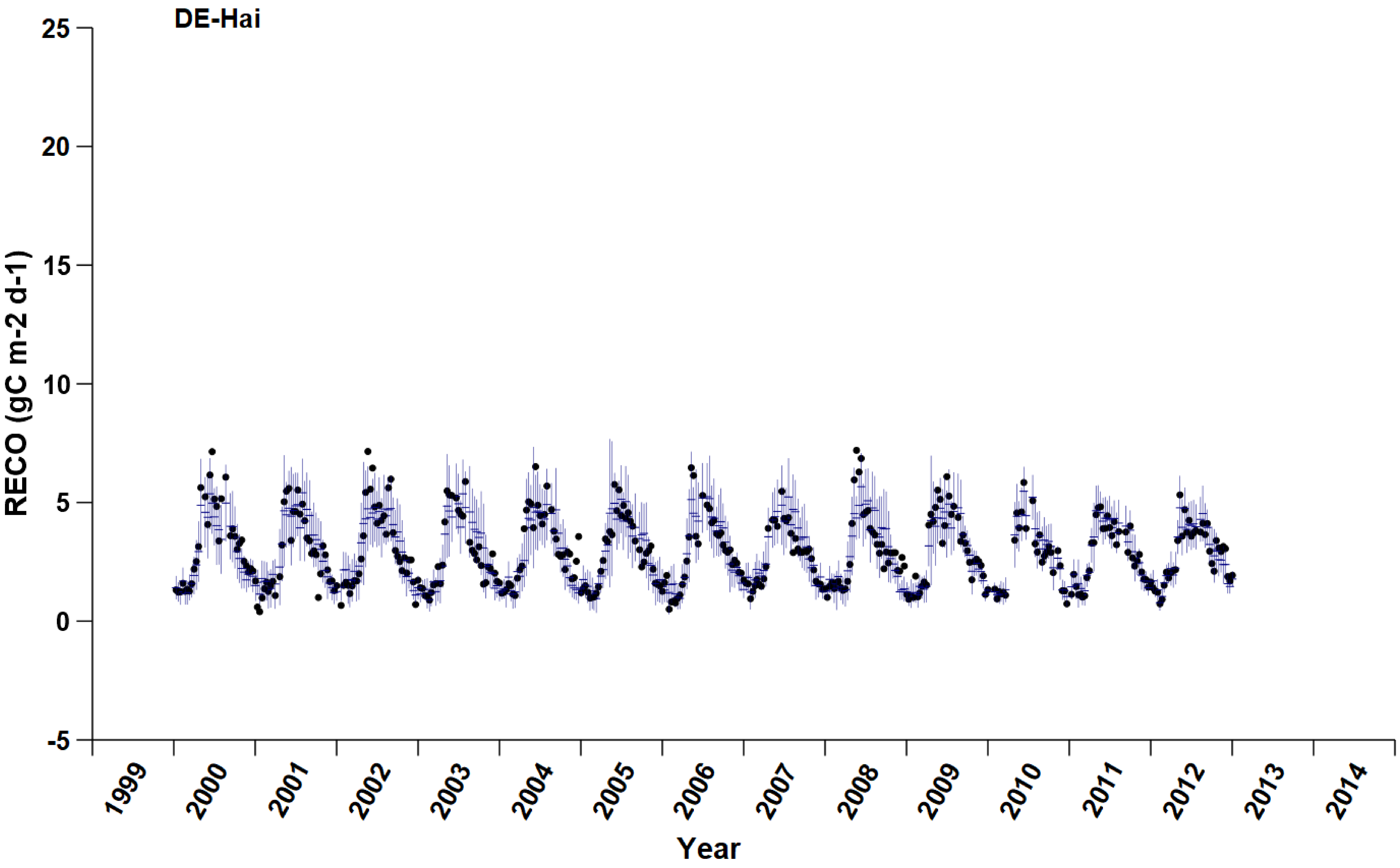
DE-Hai
RECO (gC m-2 d-1)
25
20
15
10
5
0
-5
1999
2000
2001
2002
2003
2004
2005
2006
2007
2008
2009
2010
2011
2012
2013
2014
Year

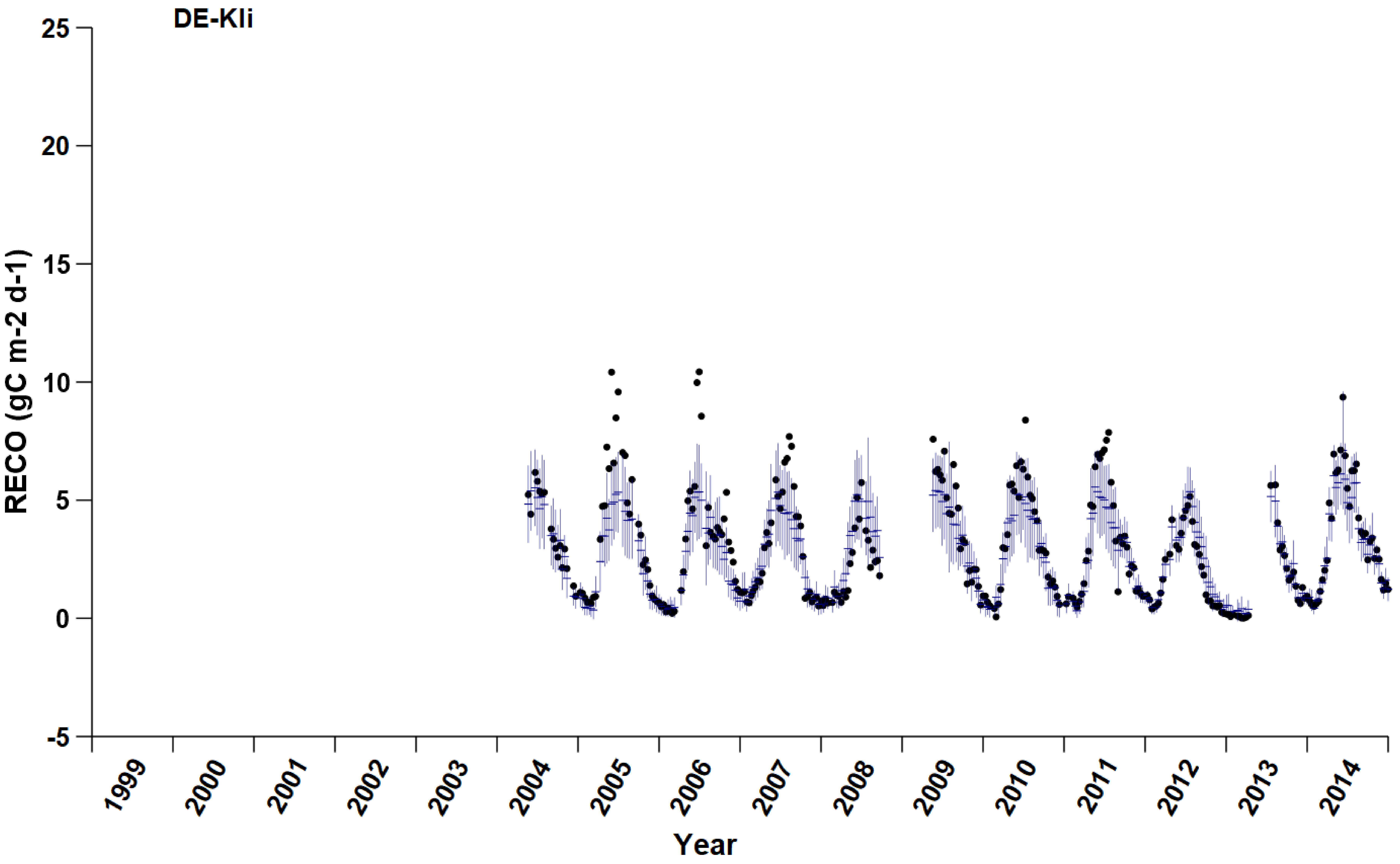
DE-Kli
RECO (gC m-2 d-1)
25
20
15
10
5
0
-5
1999
2000
2001
2002
2003
2004
2005
2006
2007
2008
2009
2010
2011
2012
2013
2014
Year

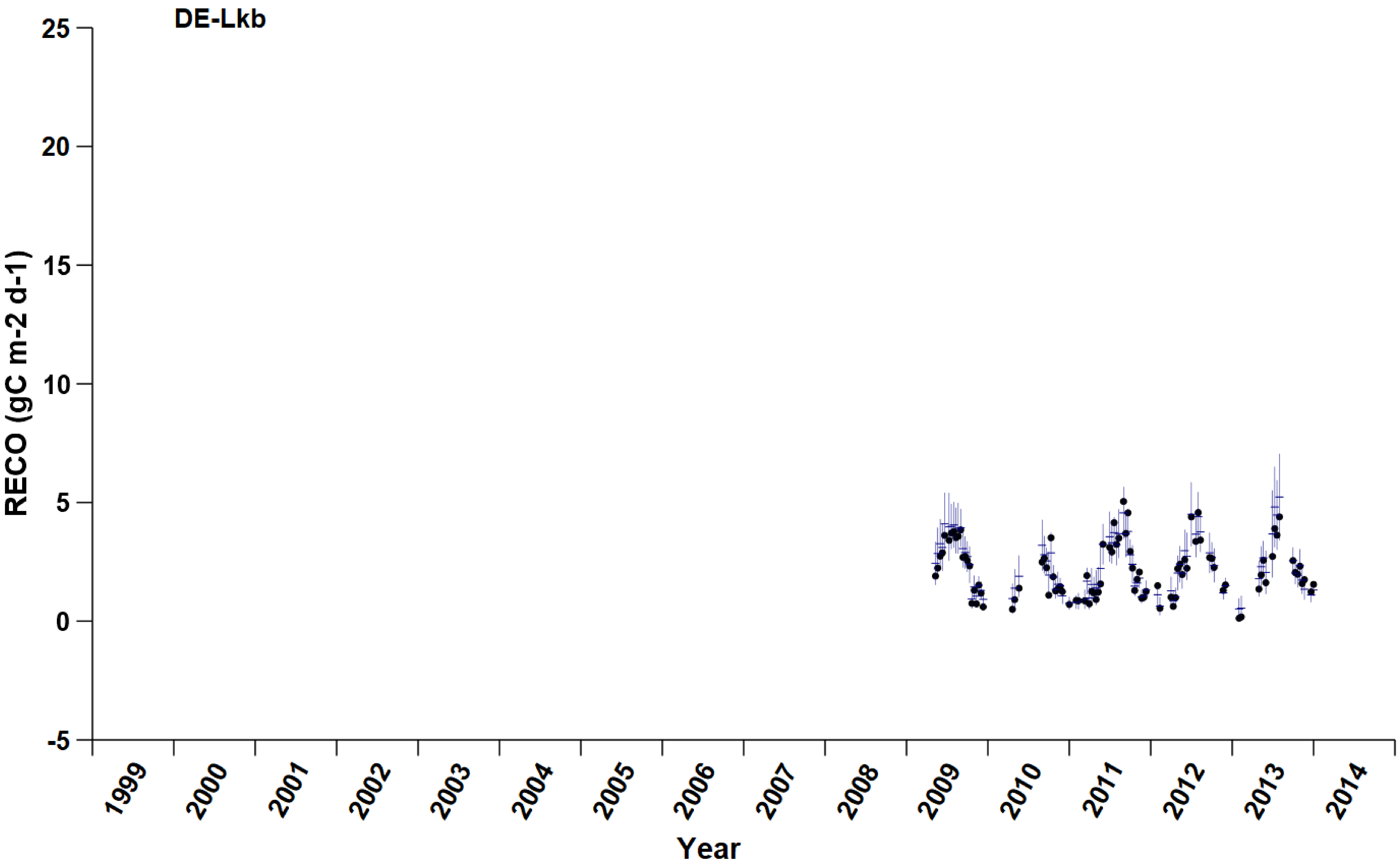
DE-Lkb
RECO (gC m-2 d-1)
25
20
15
10
5
0
-5
1999
2000
2001
2002
2003
2004
2005
2006
2007
2008
2009
2010
2011
2012
2013
2014
Year

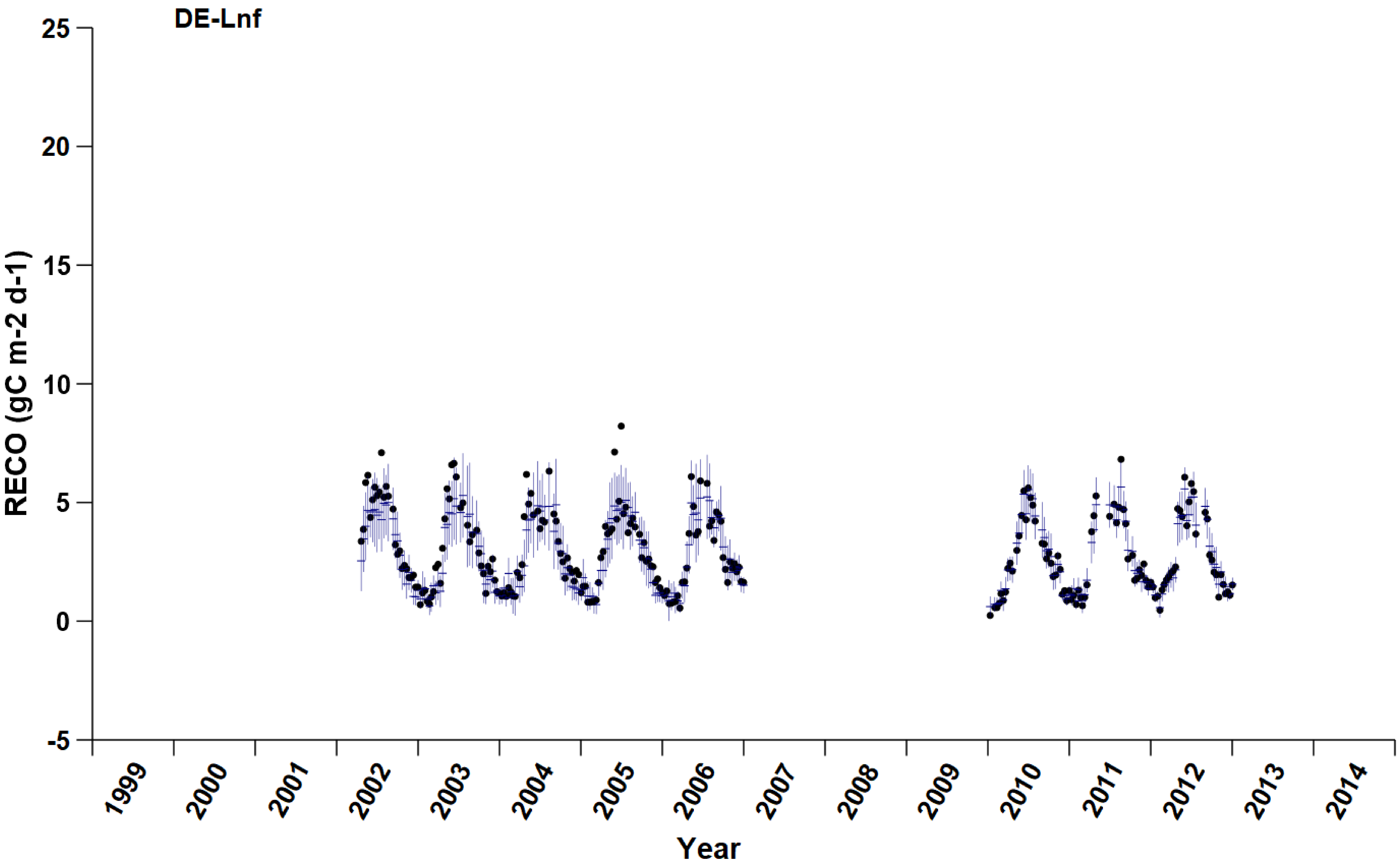
DE-Lnf
RECO (gC m-2 d-1)
Year
25
20
15
10
5
0
-5
1999
2000
2001
2002
2003
2004
2005
2006
2007
2008
2009
2010
2011
2012
2013
2014

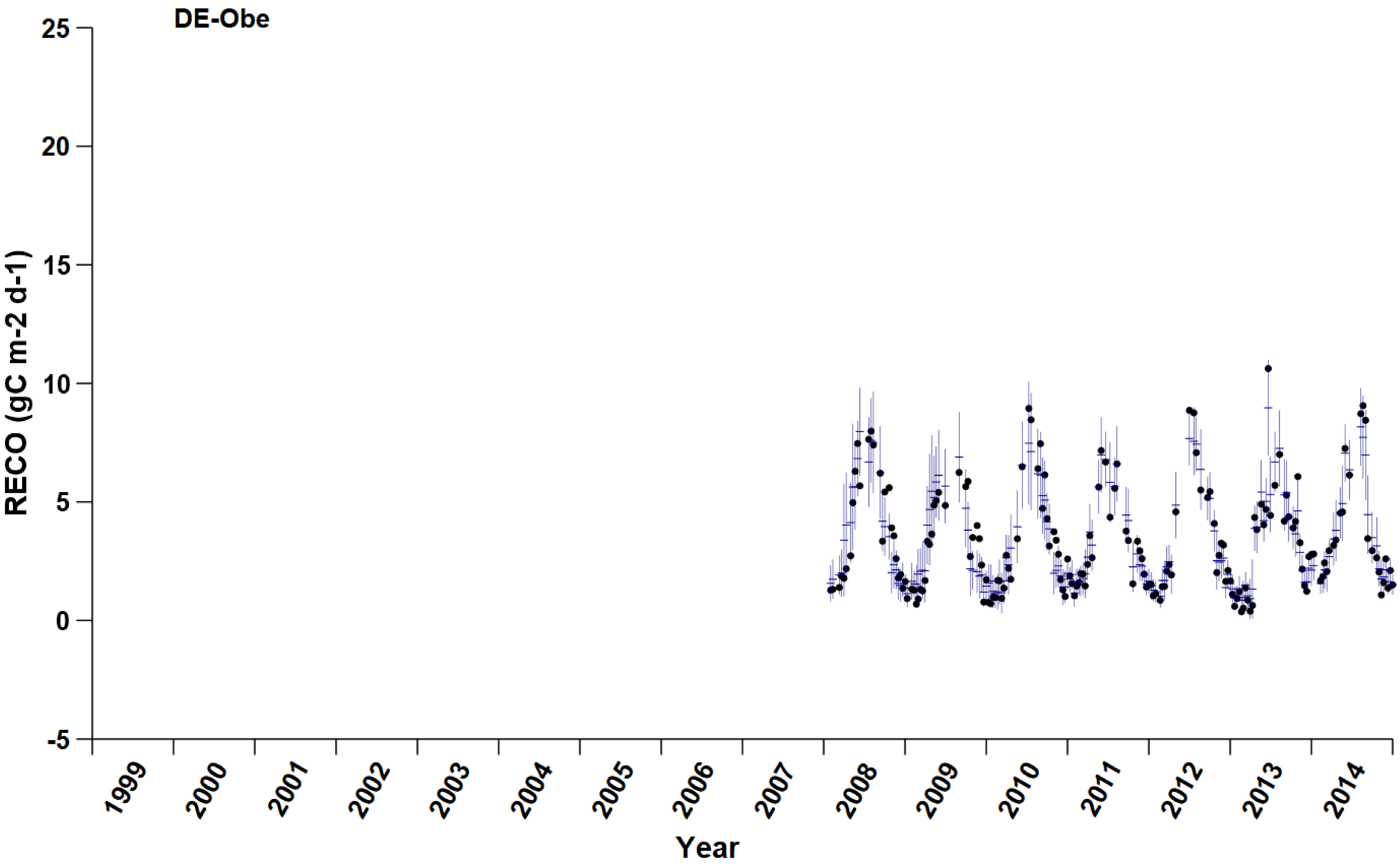
DE-Obe
RECO (gC m-2 d-1)
25
20
15
10
5
0
-5
1999
2000
2001
2002
2003
2004
2005
2006
2007
2008
2009
2010
2011
2012
2013
2014
Year

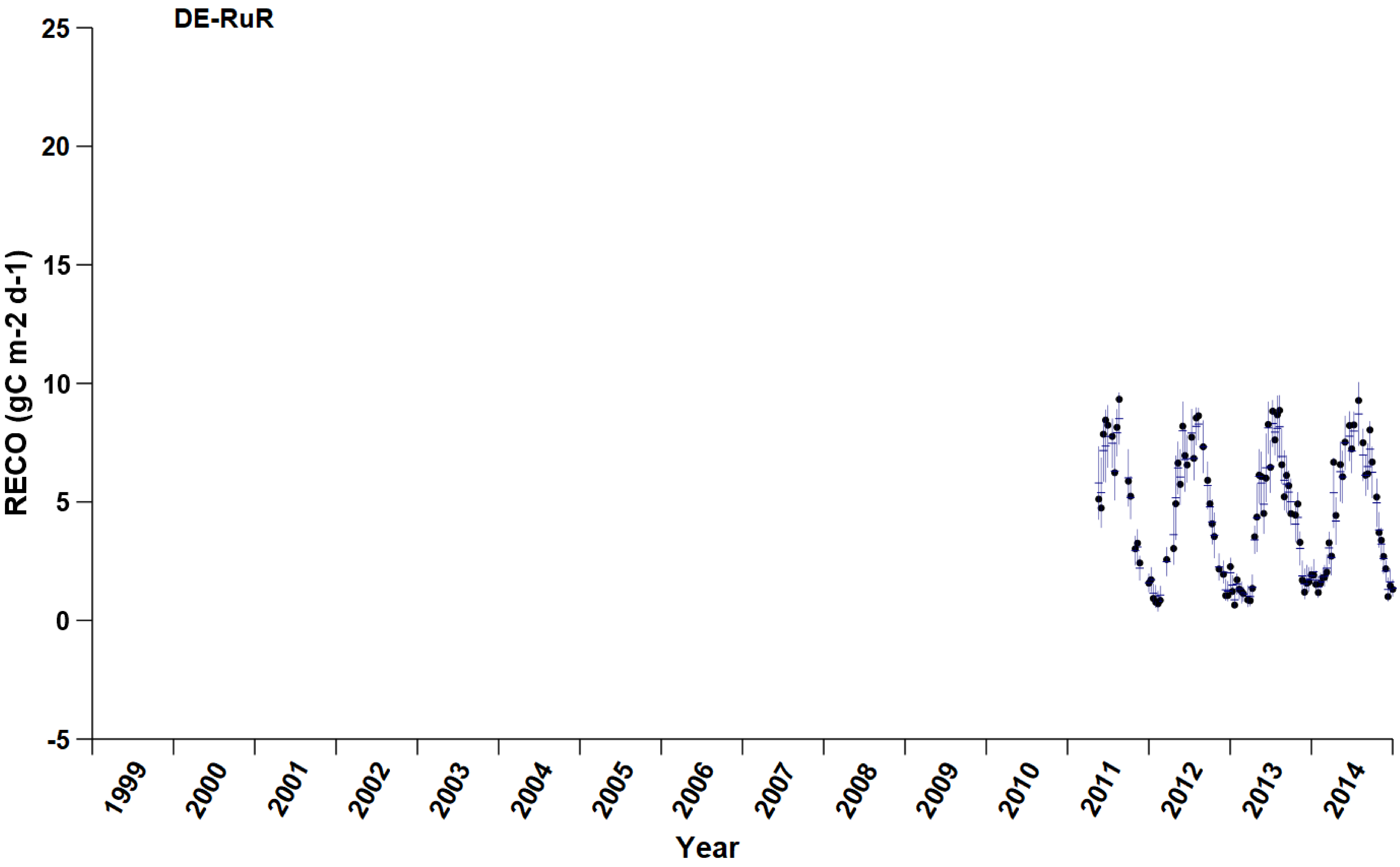

DE-RuR
RECO (gC m-2 d-1)
25
20
15
10
5
0
-5
1999
2000
2001
2002
2003
2004
2005
2006
2007
2008
2009
2010
2011
2012
2013
2014
Year

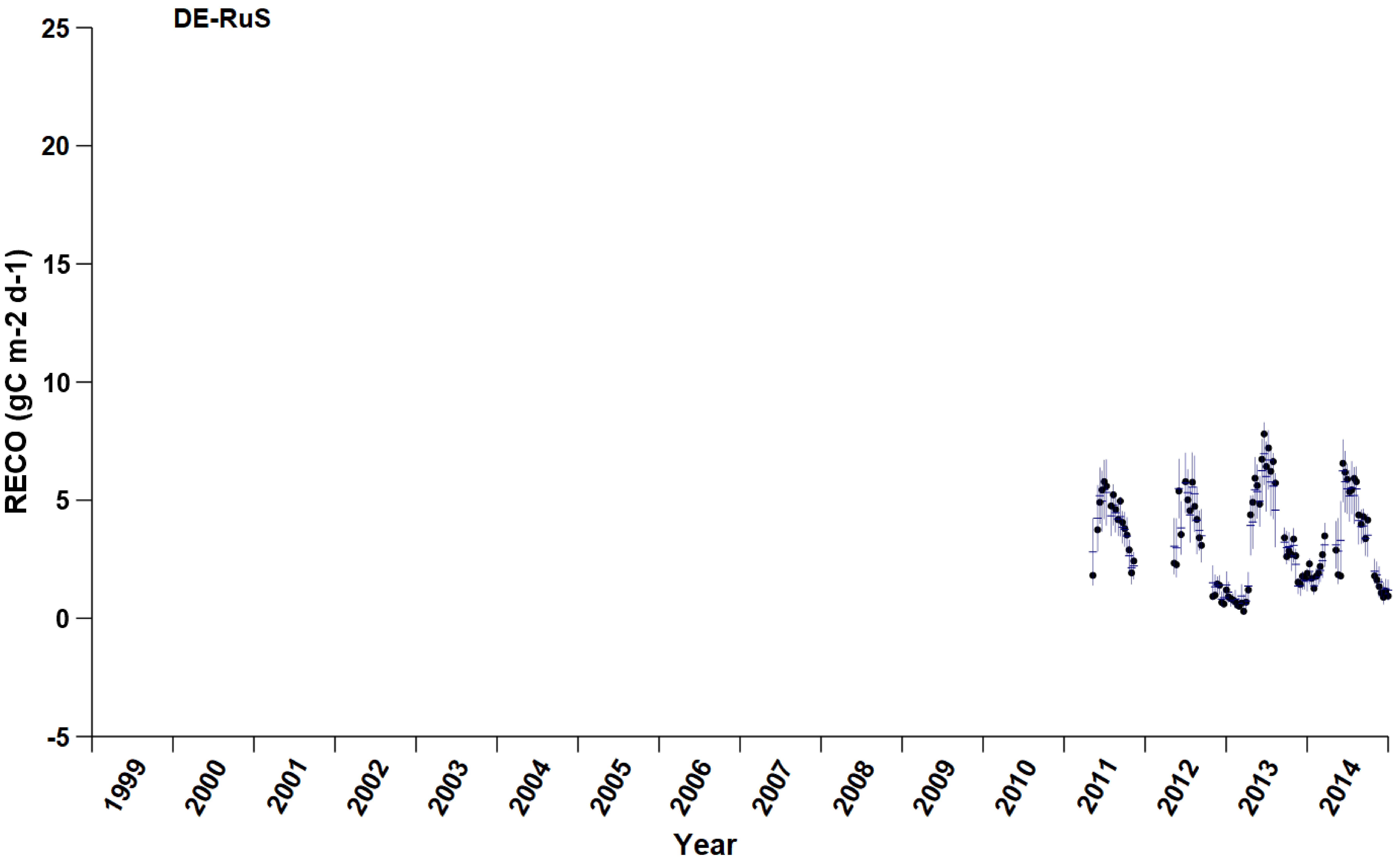
DE-RuS
RECO (gC m-2 d-1)
25
20
15
10
5
0
-5
1999
2000
2001
2002
2003
2004
2005
2006
2007
2008
2009
2010
2011
2012
2013
2014
Year

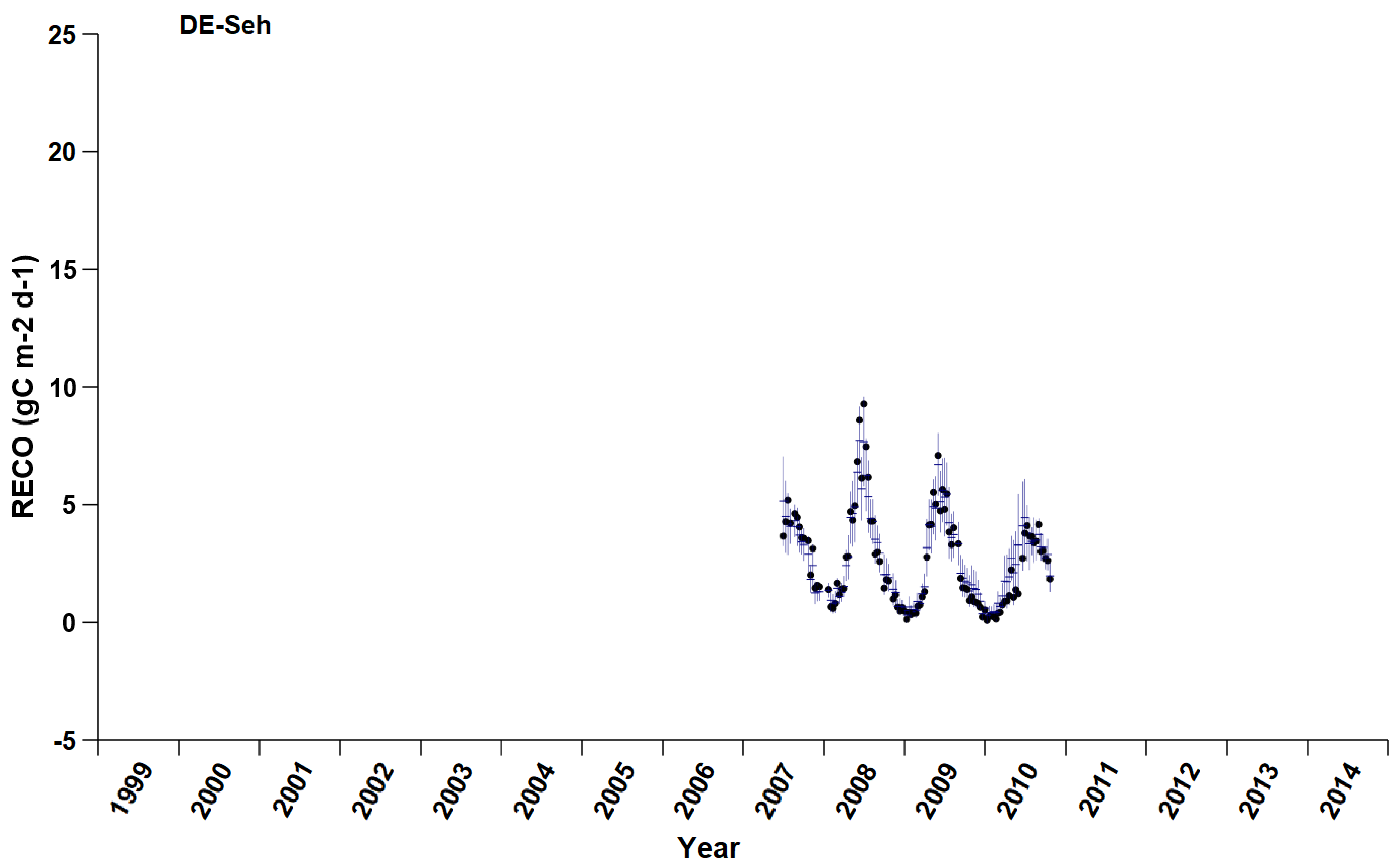
DE-Seh
RECO (gC m-2 d-1)
25
20
15
10
5
0
-5
1999
2000
2001
2002
2003
2004
2005
2006
2007
2008
2009
2010
2011
2012
2013
2014
Year

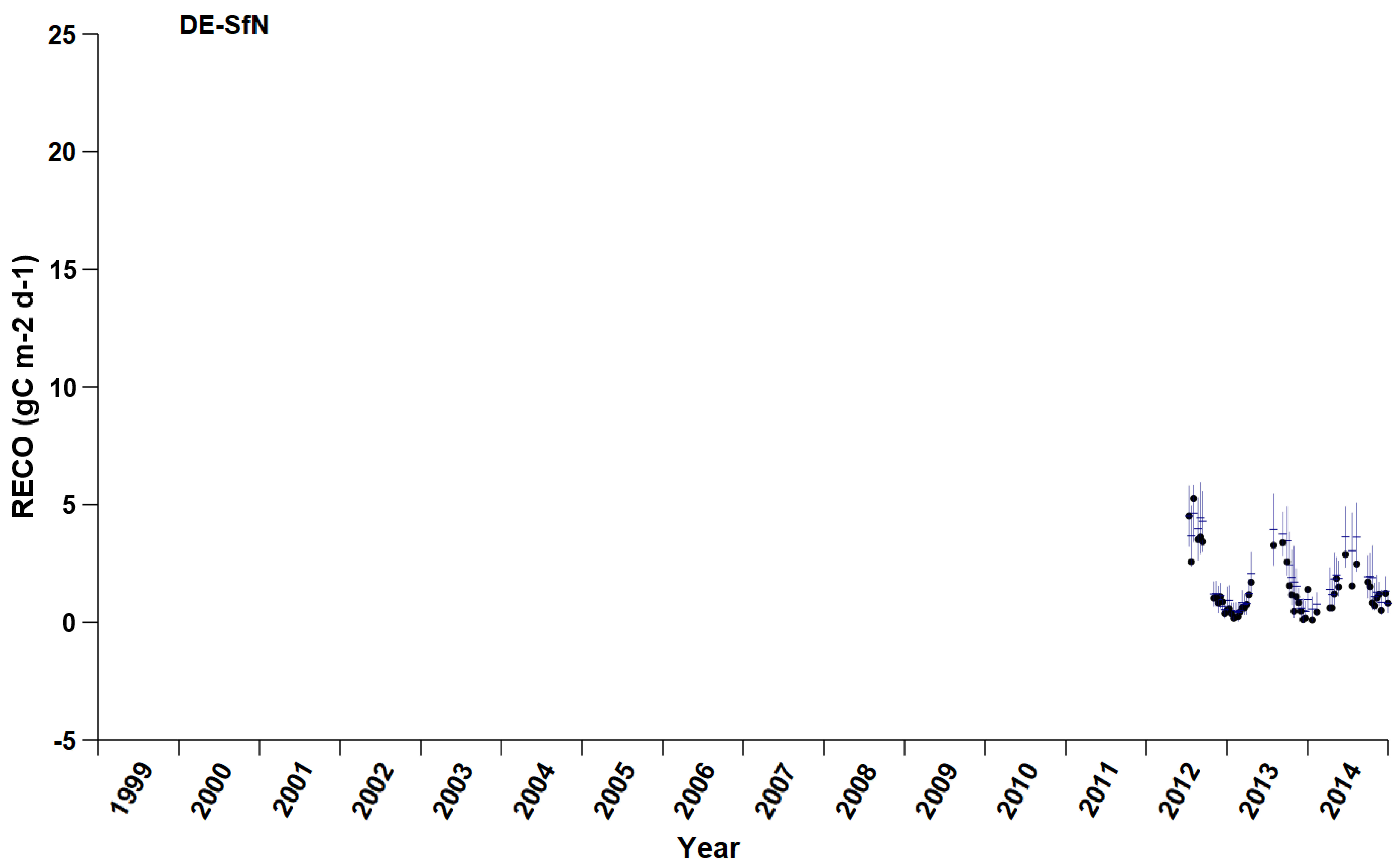
DE-SfN
RECO (gC m-2 d-1)
25
20
15
10
5
0
-5
1999
2000
2001
2002
2003
2004
2005
2006
2007
2008
2009
2010
2011
2012
2013
2014
Year

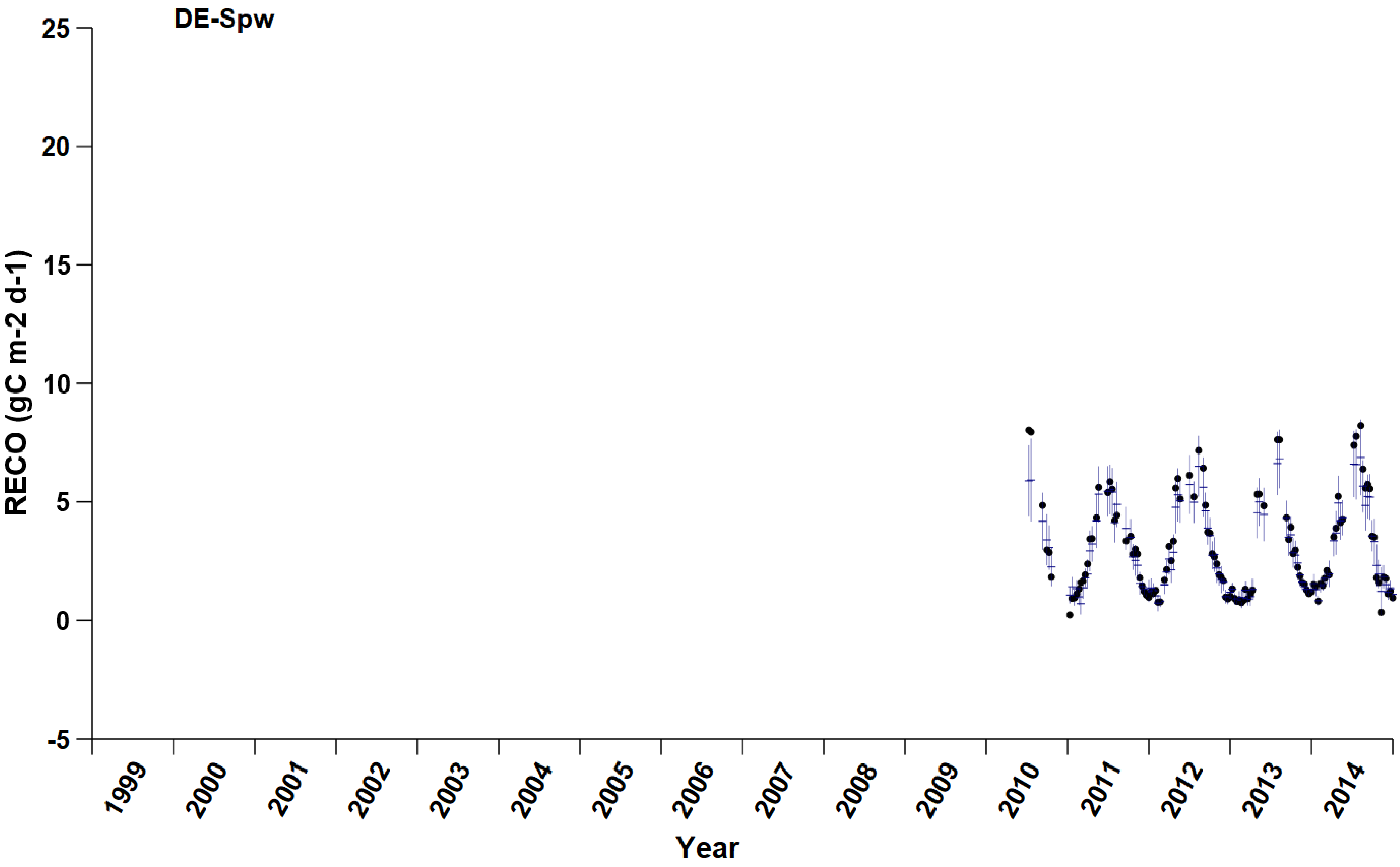
DE-Spw
RECO (gC m-2 d-1)
25
20
15
10
5
0
-5
1999
2000
2001
2002
2003
2004
2005
2006
2007
2008
2009
2010
2011
2012
2013
2014
Year

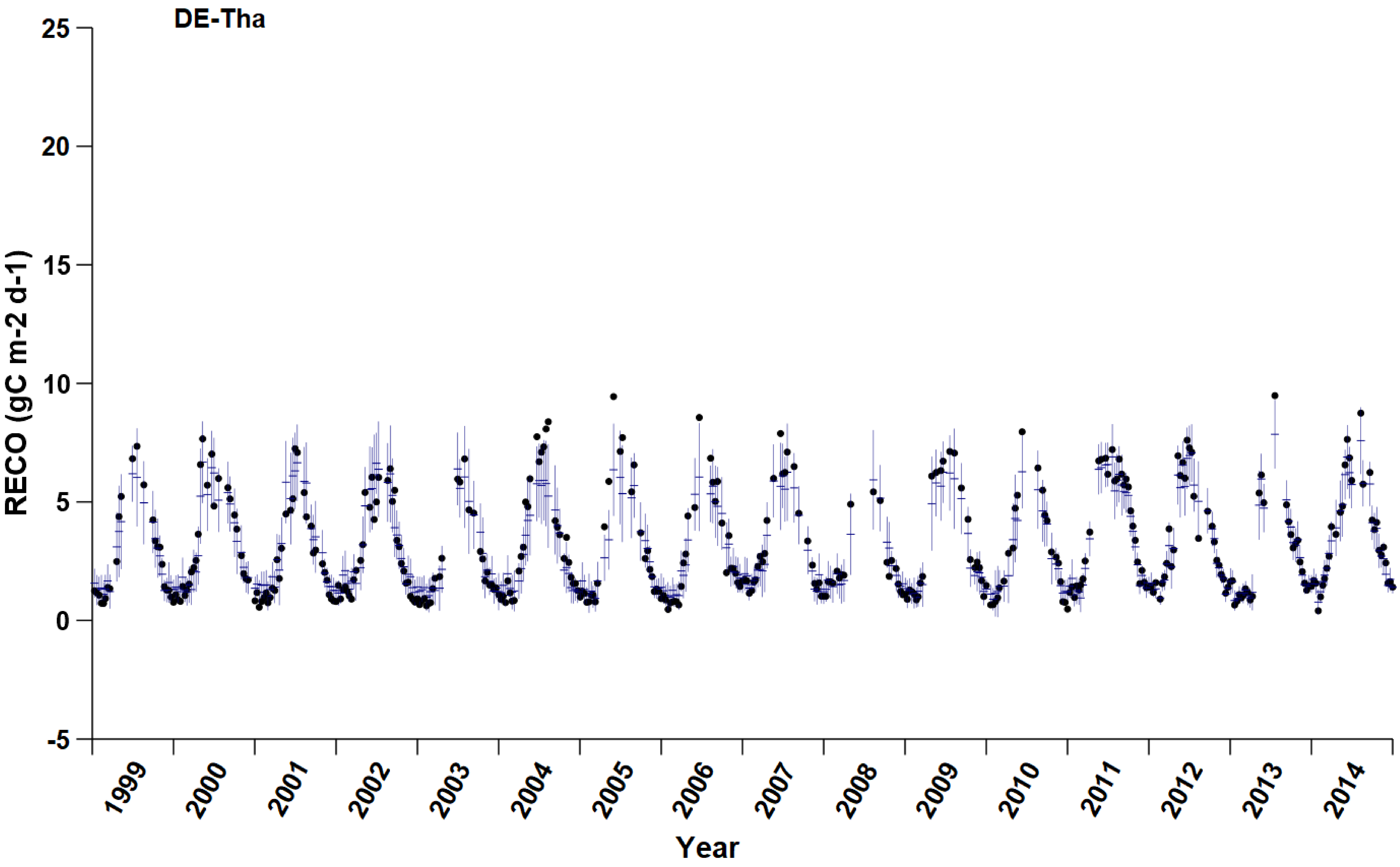
DE-Tha
RECO (gC m-2 d-1)
25
20
15
10
5
0
-5
1999
2000
2001
2002
2003
2004
2005
2006
2007
2008
2009
2010
2011
2012
2013
2014
Year

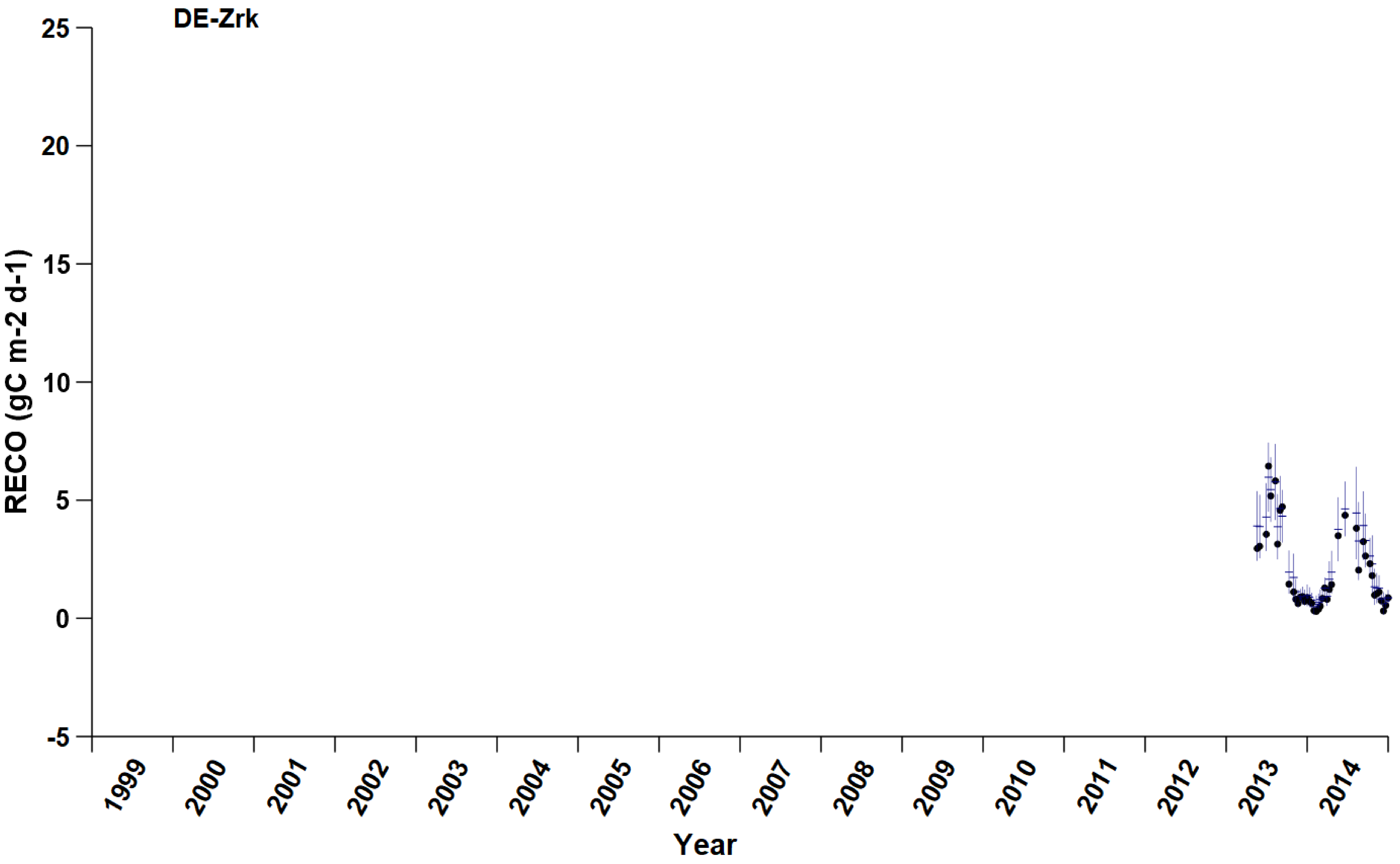

DE-Zrk
RECO (gC m-2 d-1)
25
20
15
10
5
0
-5
1999
2000
2001
2002
2003
2004
2005
2006
2007
2008
2009
2010
2011
2012
2013
2014
Year

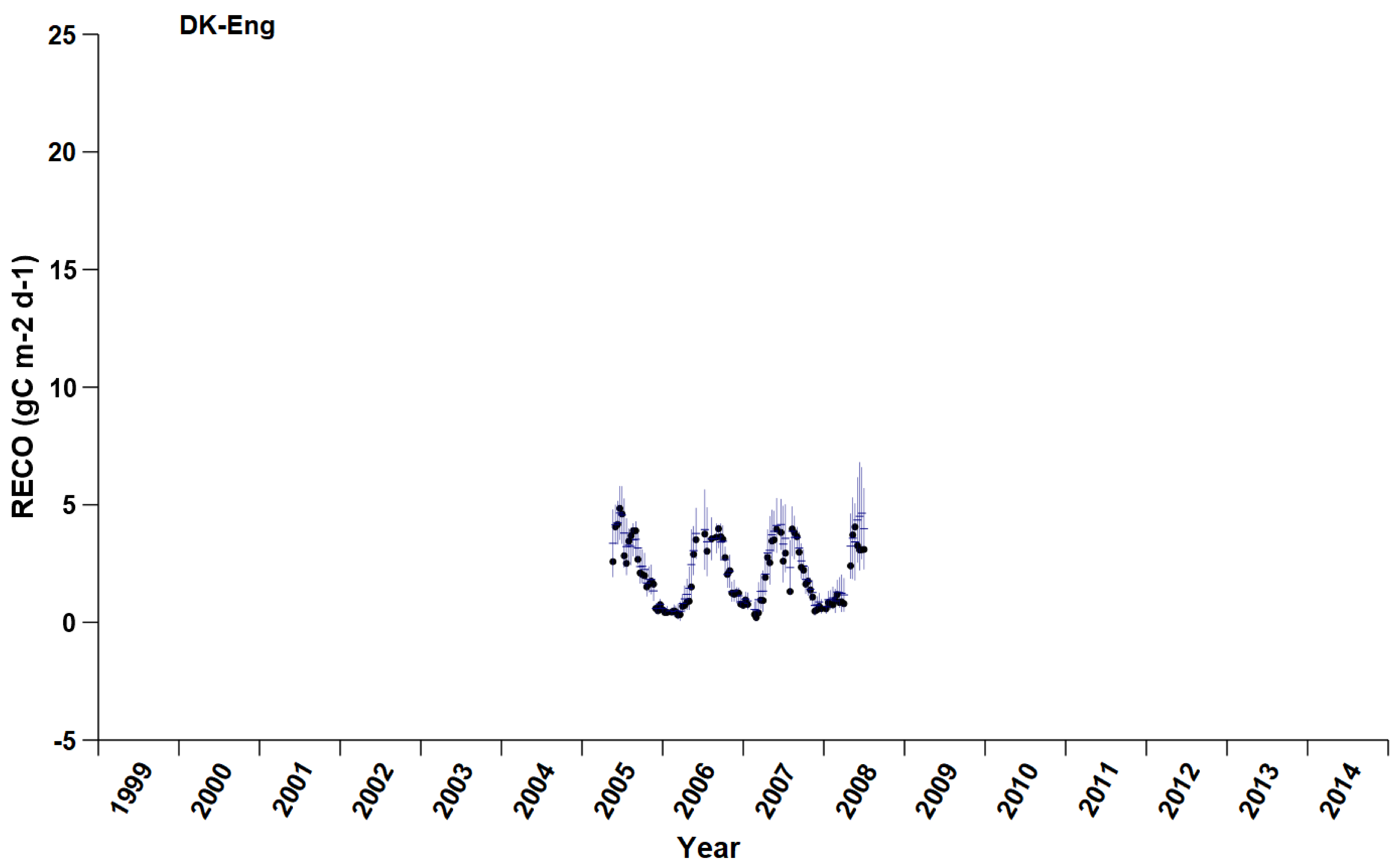
DK-Eng
RECO (gC m-2 d-1)
25
20
15
10
5
0
-5
1999
2000
2001
2002
2003
2004
2005
2006
2007
2008
2009
2010
2011
2012
2013
2014
Year

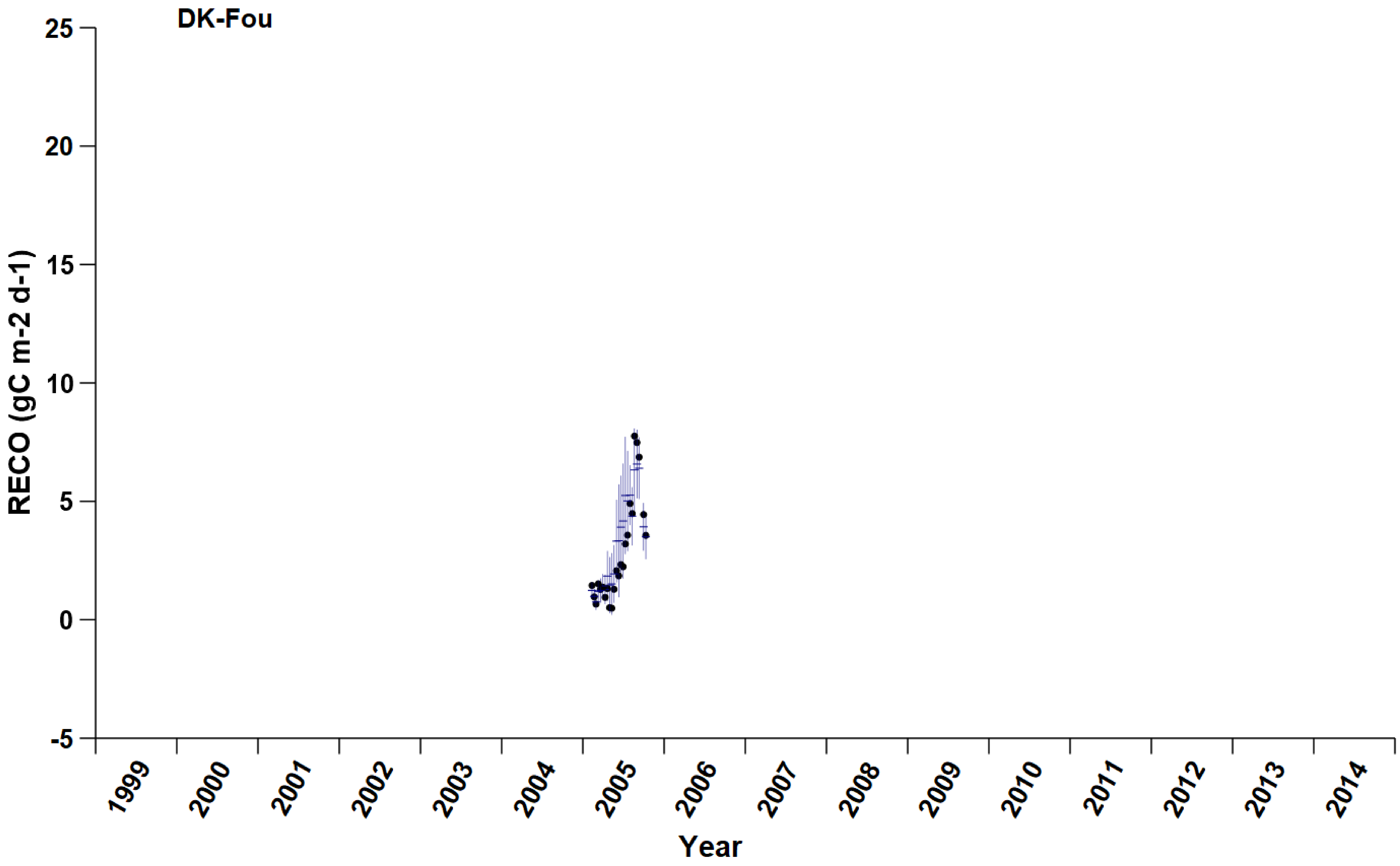
DK-Fou
RECO (gC m-2 d-1)
25
20
15
10
5
0
-5
1999
2000
2001
2002
2003
2004
2005
2006
2007
2008
2009
2010
2011
2012
2013
2014
Year

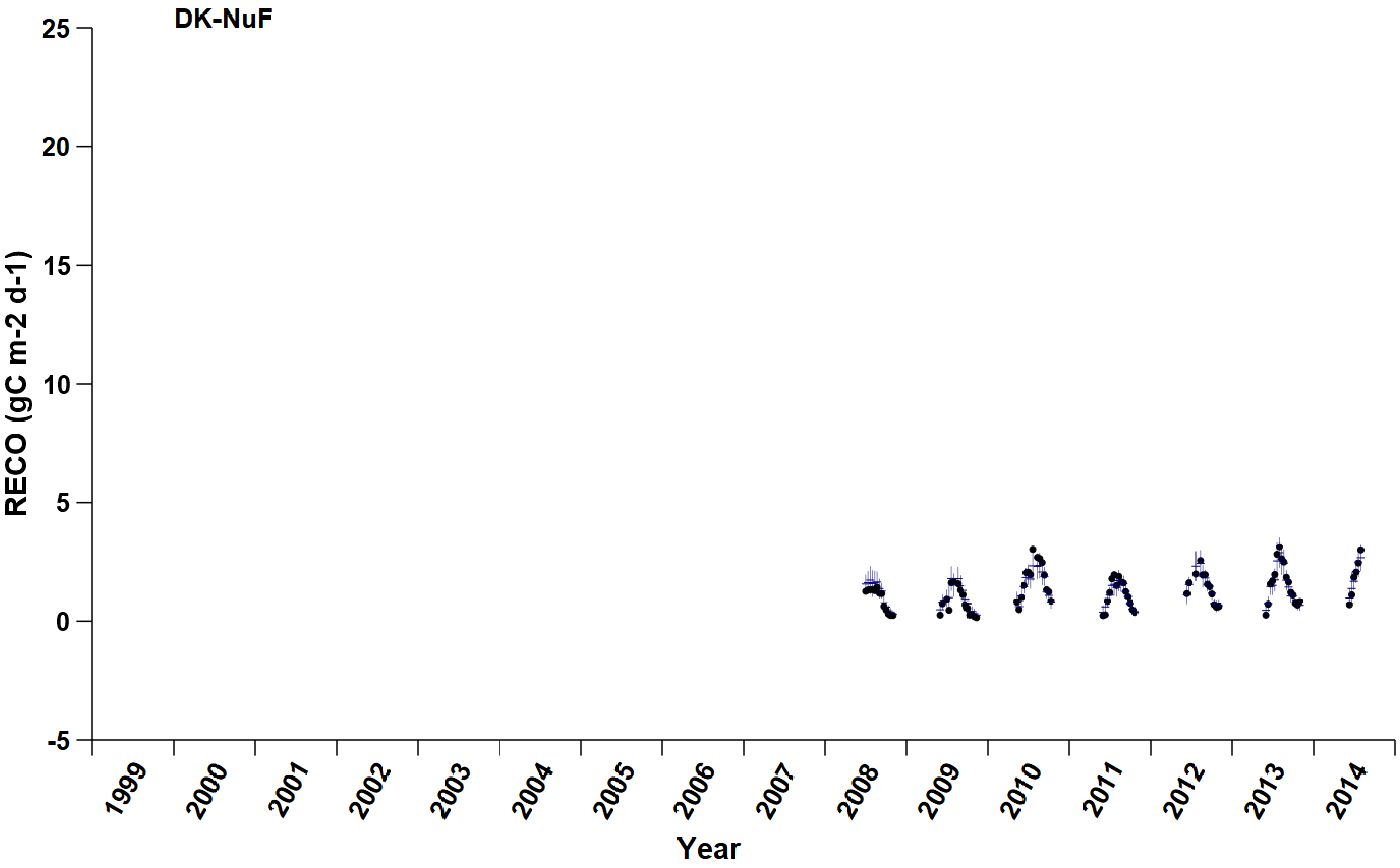
DK-NuF
RECO (gC m-2 d-1)
25
20
15
10
5
0
-5
1999
2000
2001
2002
2003
2004
2005
2006
2007
2008
2009
2010
2011
2012
2013
2014
Year

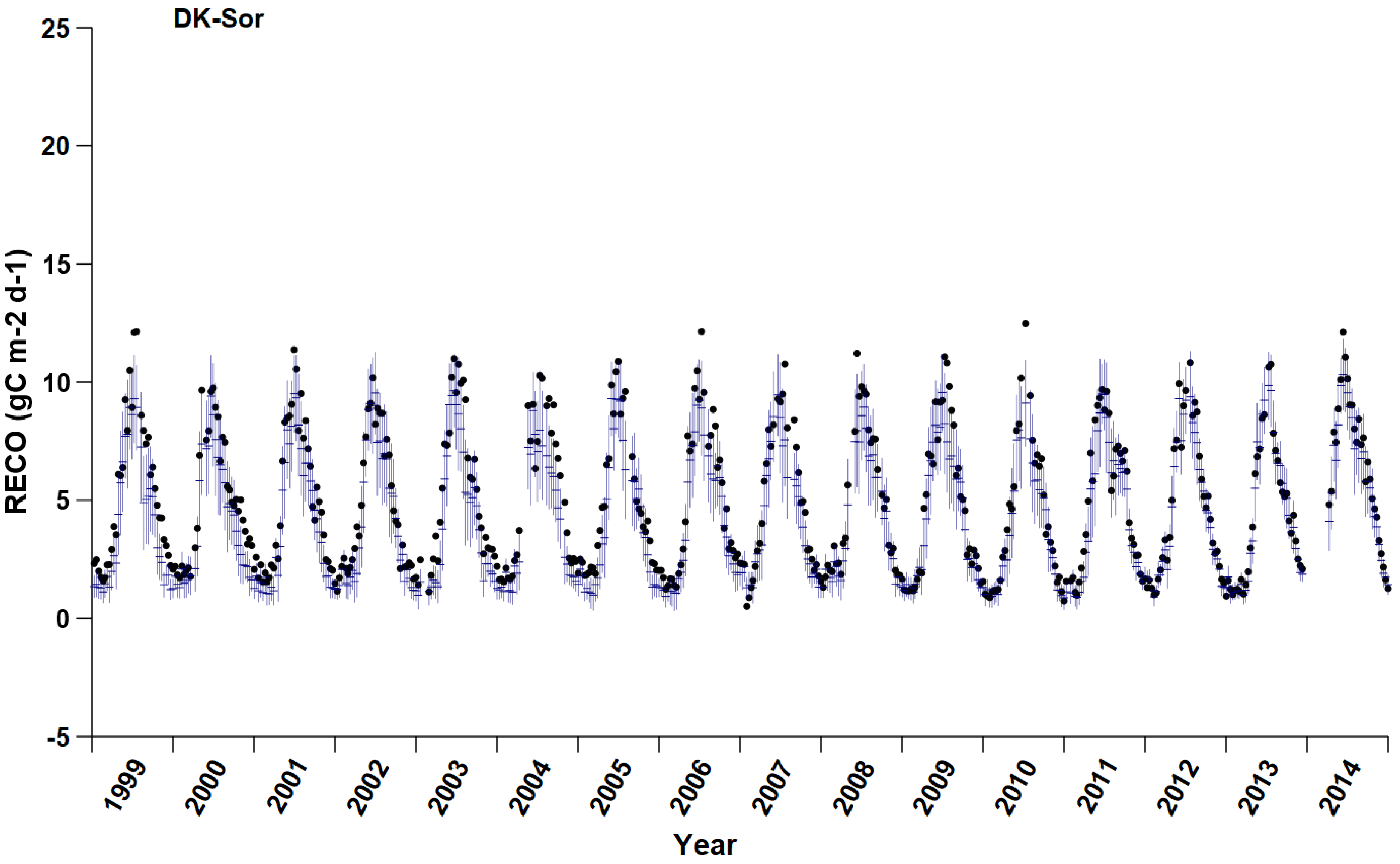

DK-Sor
RECO (gC m-2 d-1)
25
20
15
10
5
0
-5
1999
2000
2001
2002
2003
2004
2005
2006
2007
2008
2009
2010
2011
2012
2013
2014
Year

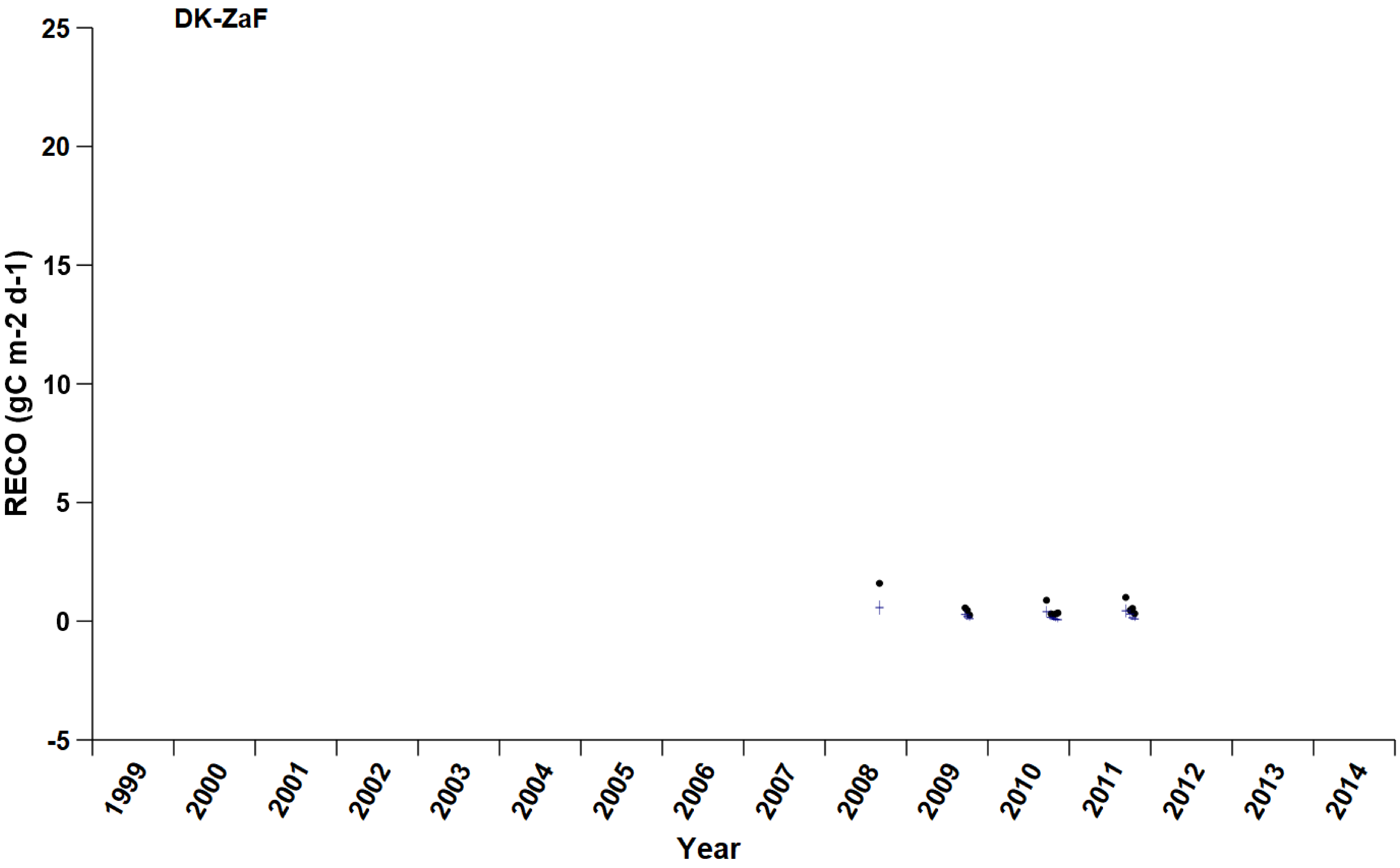

DK-ZaF
RECO (gC m-2 d-1)
25
20
15
10
5
0
-5
1999
2000
2001
2002
2003
2004
2005
2006
2007
2008
2009
2010
2011
2012
2013
2014
Year

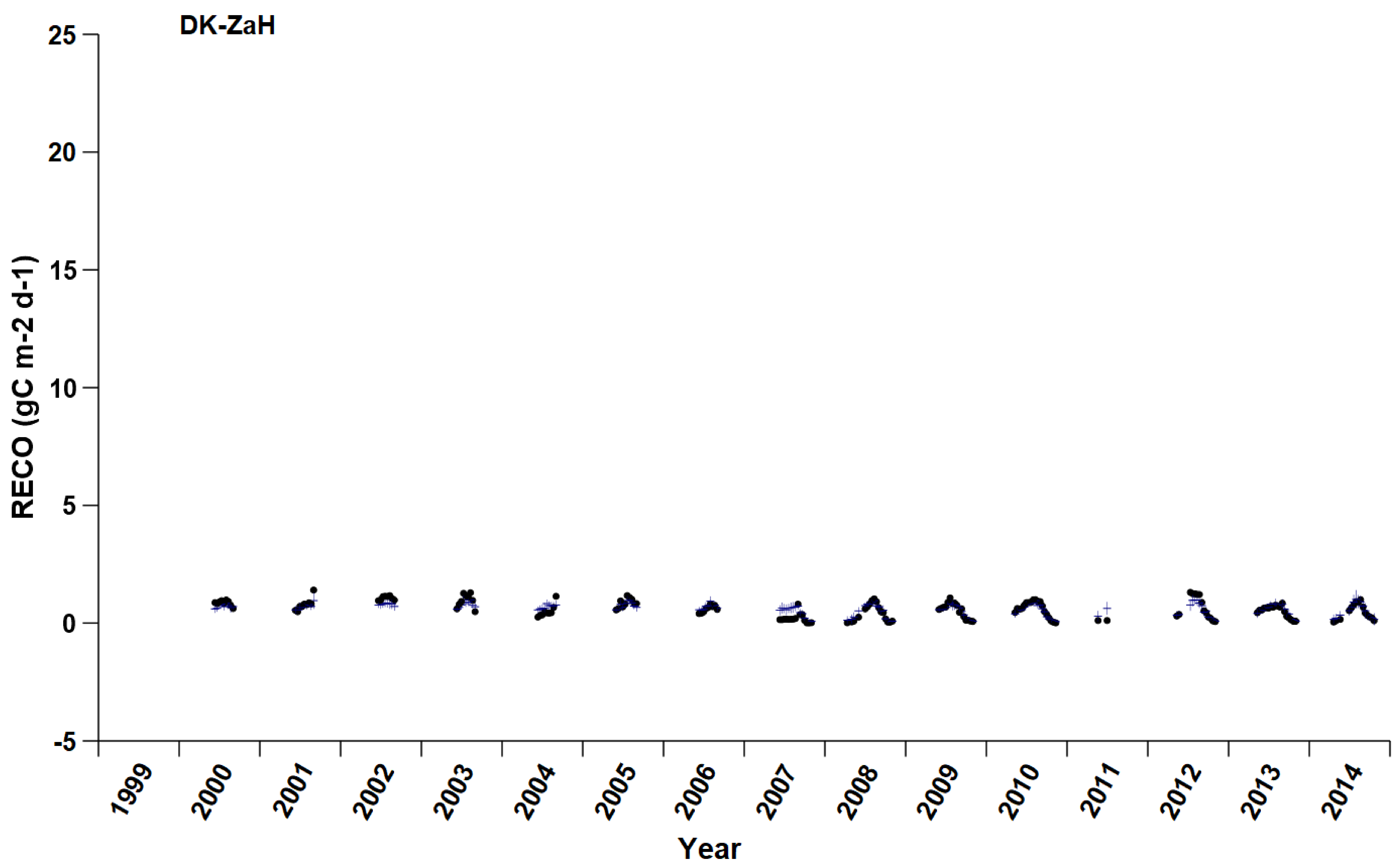

DK-ZaH
RECO (gC m-2 d-1)
25
20
15
10
5
0
-5
1999
2000
2001
2002
2003
2004
2005
2006
2007
2008
2009
2010
2011
2012
2013
2014
Year

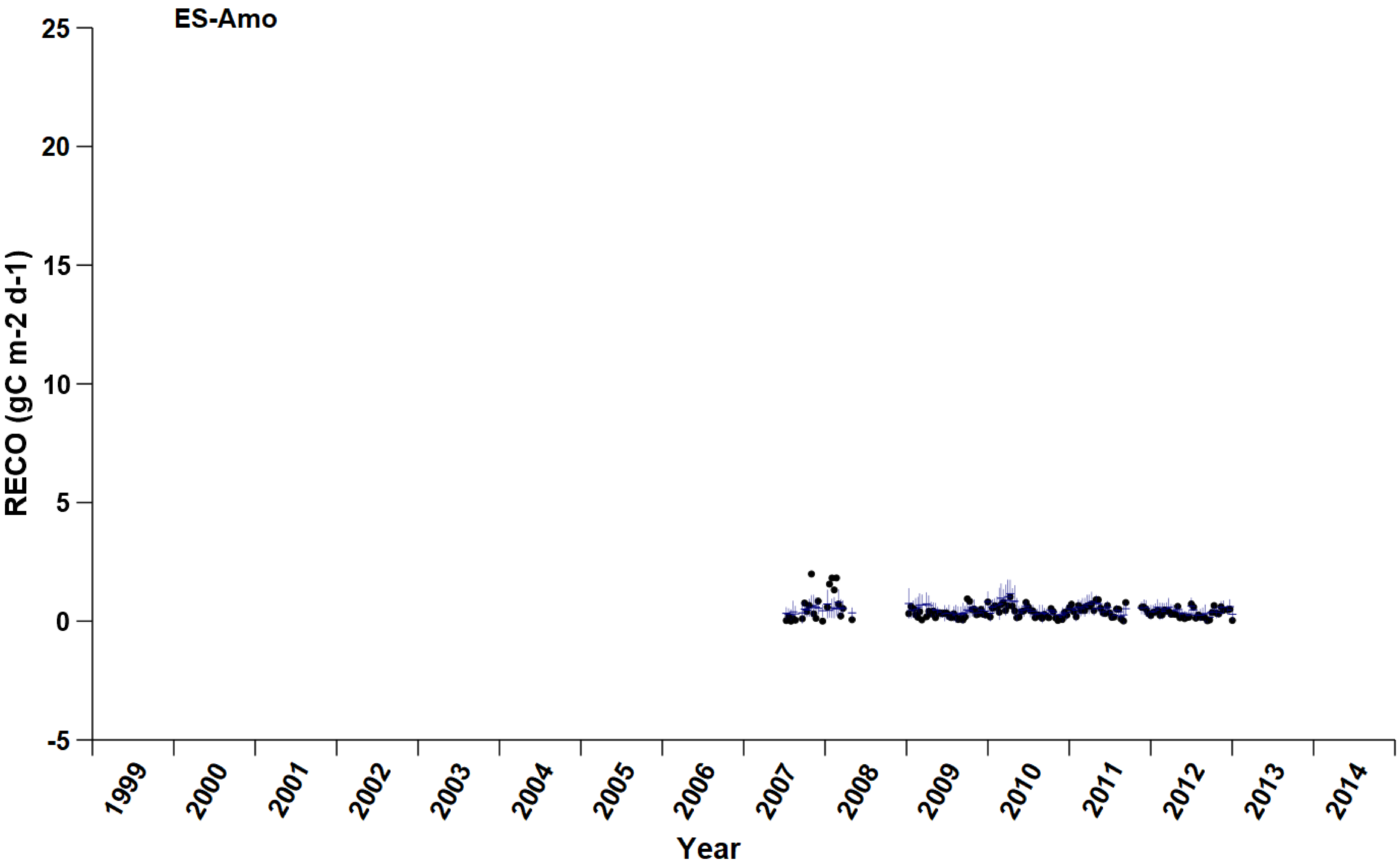
ES-Amo
RECO (gC m-2 d-1)
25
20
15
10
5
0
-5
1999
2000
2001
2002
2003
2004
2005
2006
2007
2008
2009
2010
2011
2012
2013
2014
Year

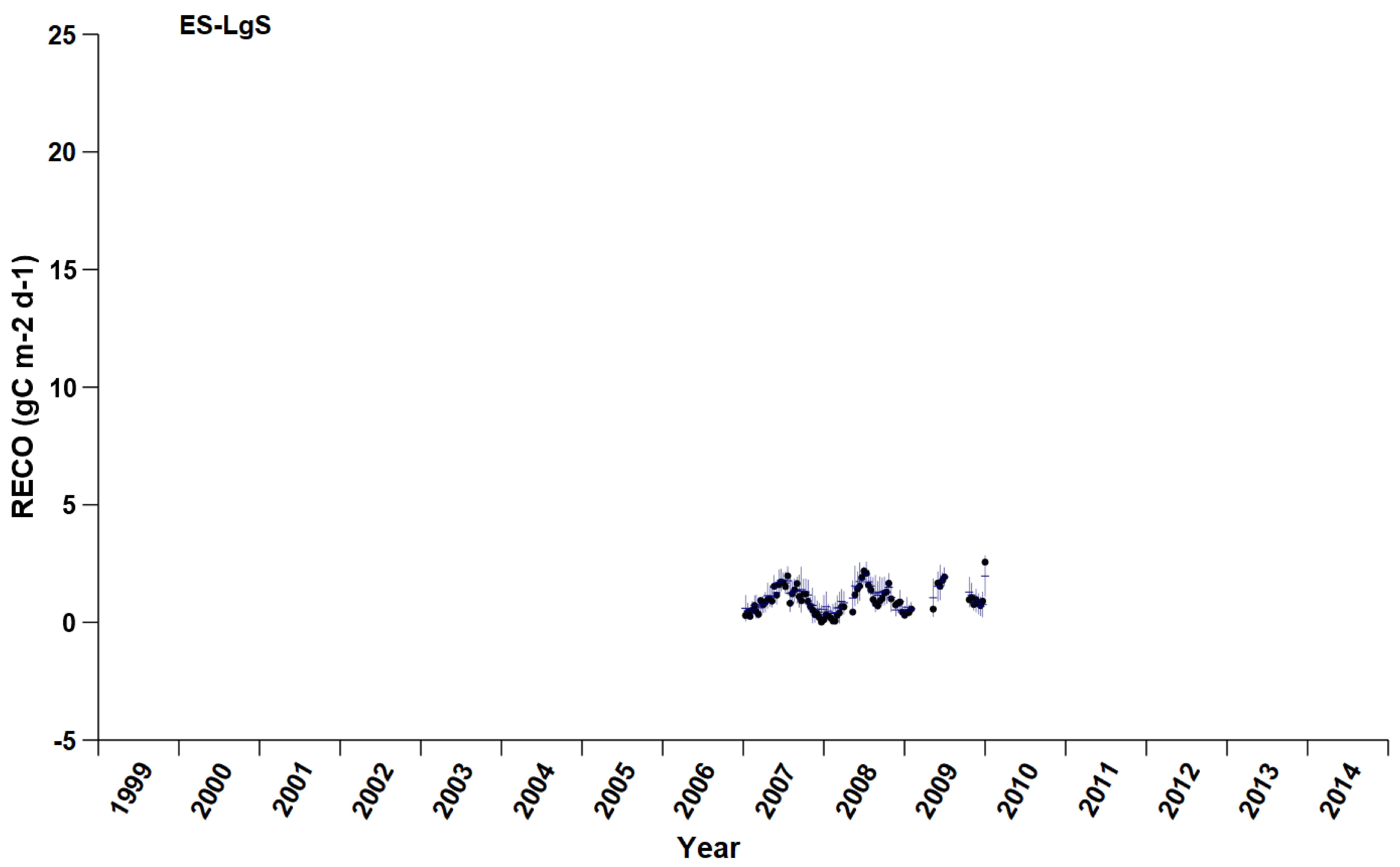

ES-LgS
RECO (gC m-2 d-1)
25
20
15
10
5
0
-5
1999
2000
2001
2002
2003
2004
2005
2006
2007
2008
2009
2010
2011
2012
2013
2014
Year

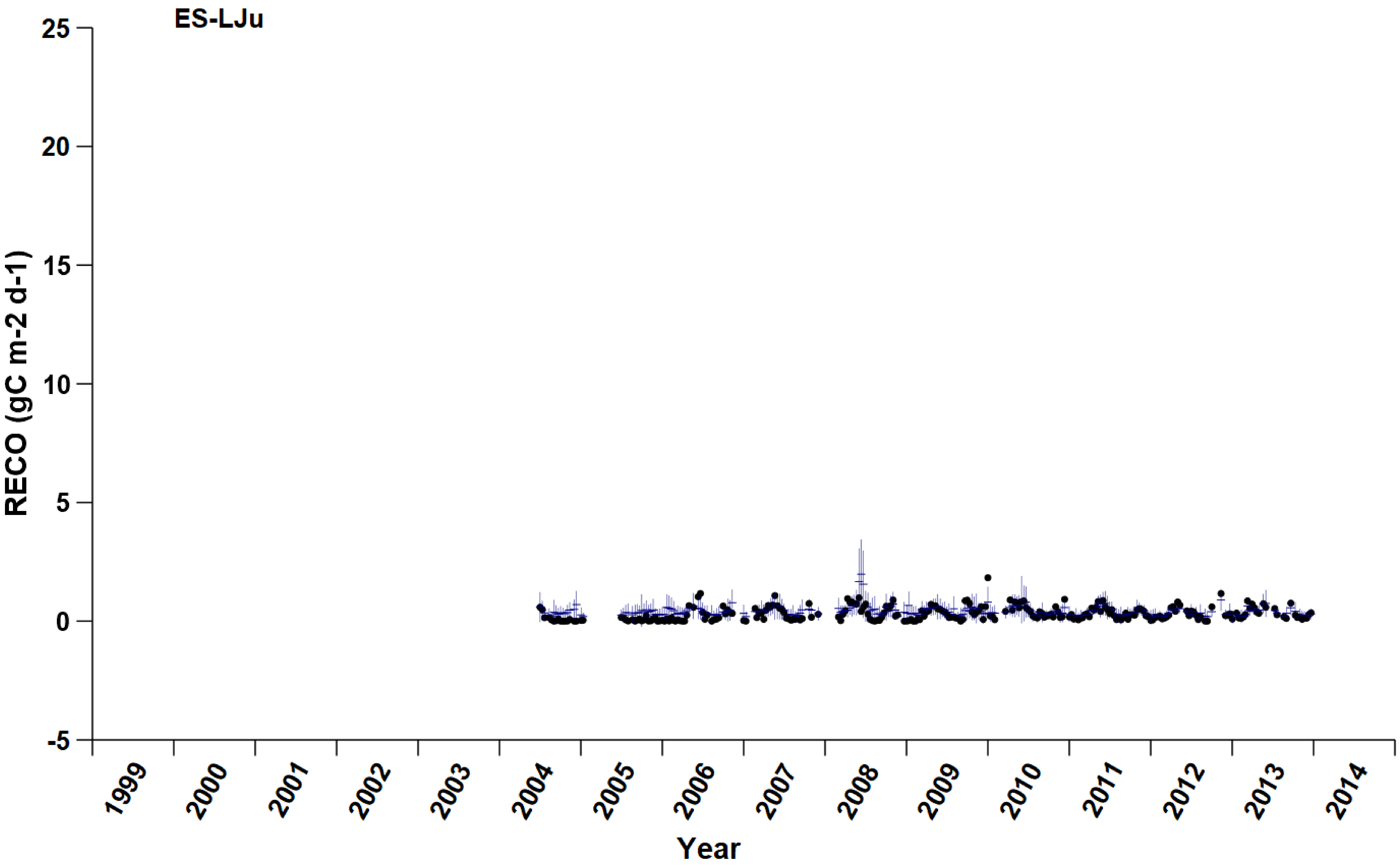
ES-LJu
25
20
15
10
5
0
-5
RECO (gC m-2 d-1)
1999
2000
2001
2002
2003
2004
2005
2006
2007
2008
2009
2010
2011
2012
2013
2014
Year

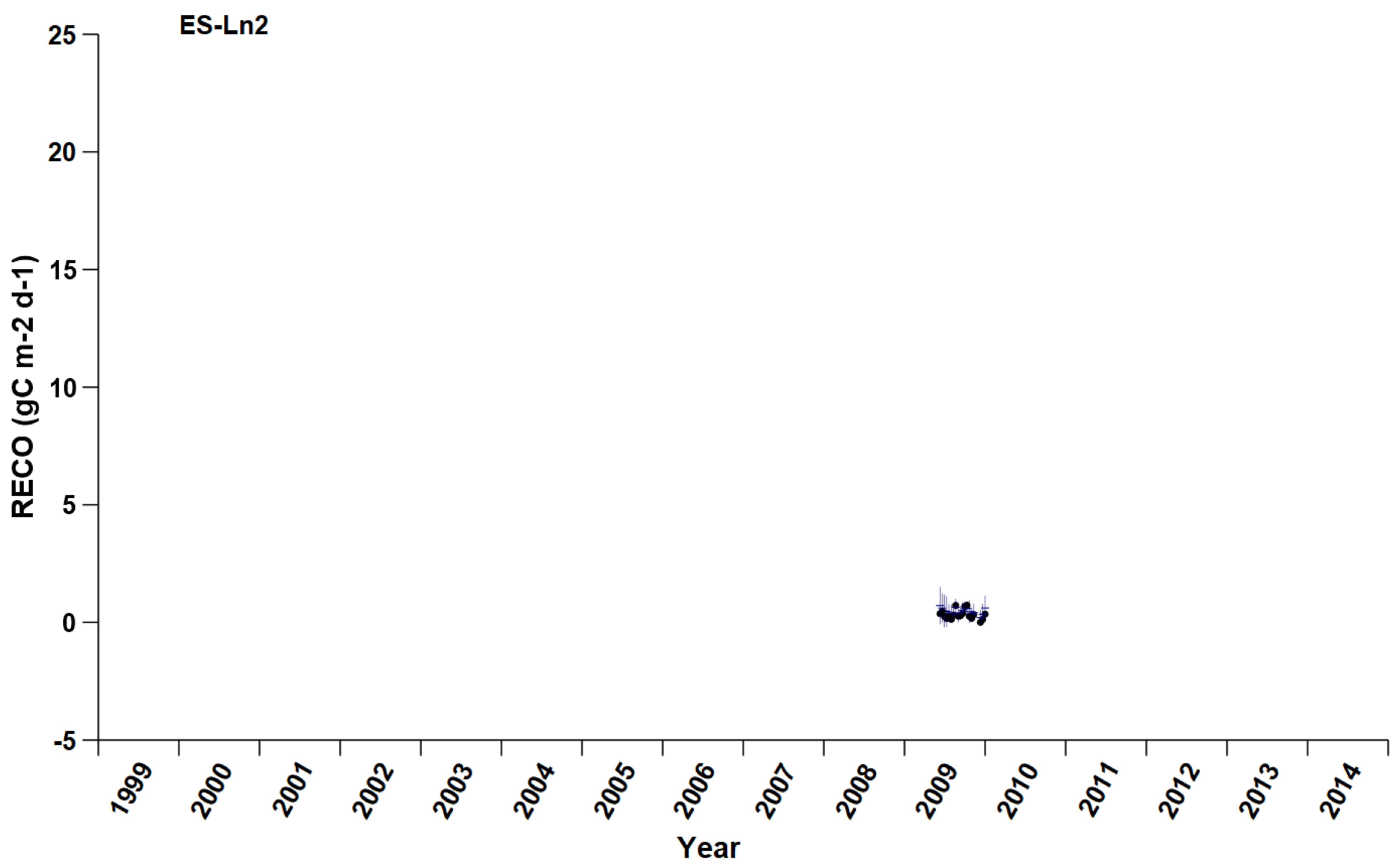

ES-Ln2
RECO (gC m-2 d-1)
25
20
15
10
5
0
-5
1999
2000
2001
2002
2003
2004
2005
2006
2007
2008
2009
2010
2011
2012
2013
2014
Year

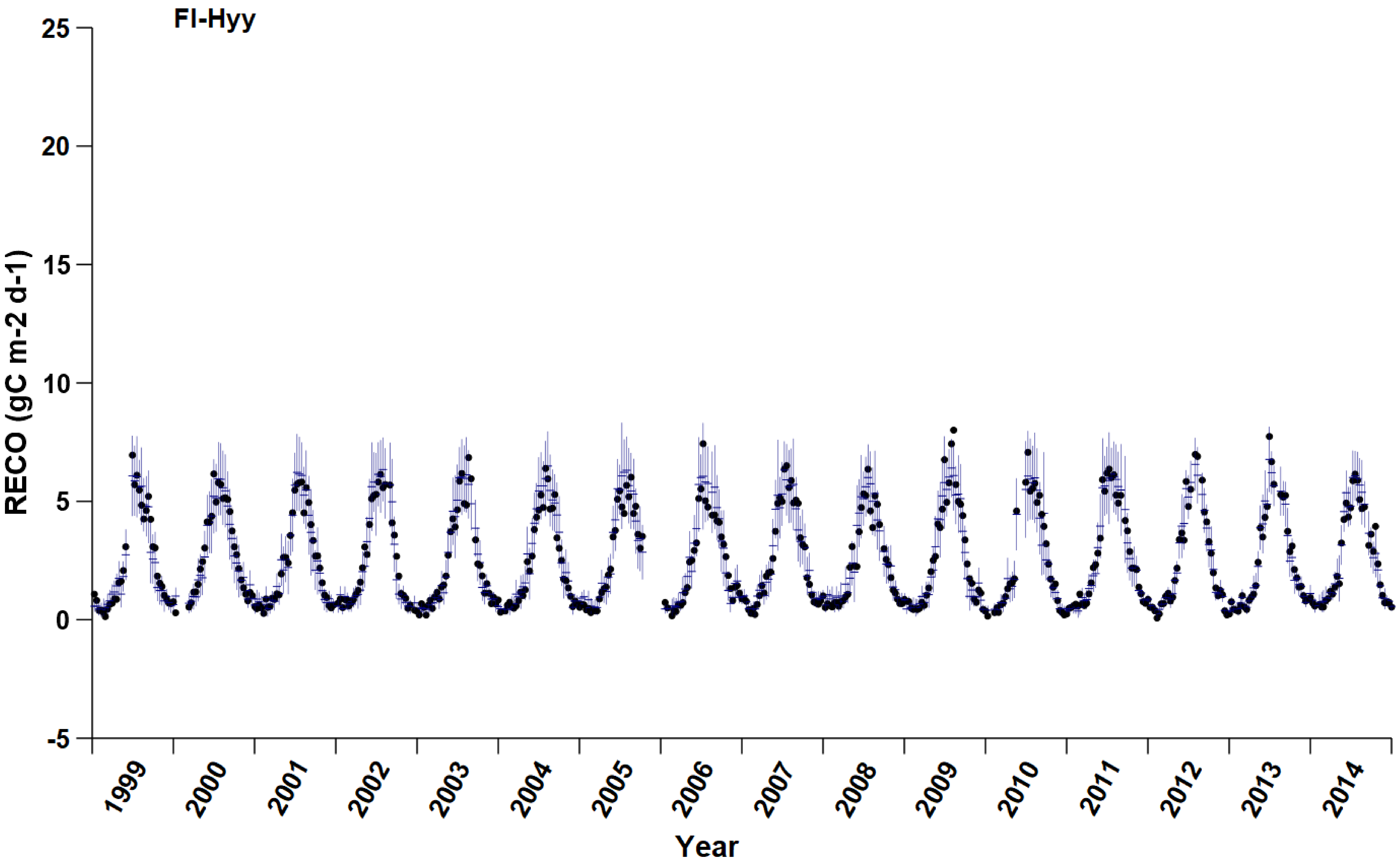
FI-Hyy
RECO (gC m-2 d-1)
25
20
15
10
5
0
-5
1999
2000
2001
2002
2003
2004
2005
2006
2007
2008
2009
2010
2011
2012
2013
2014
Year

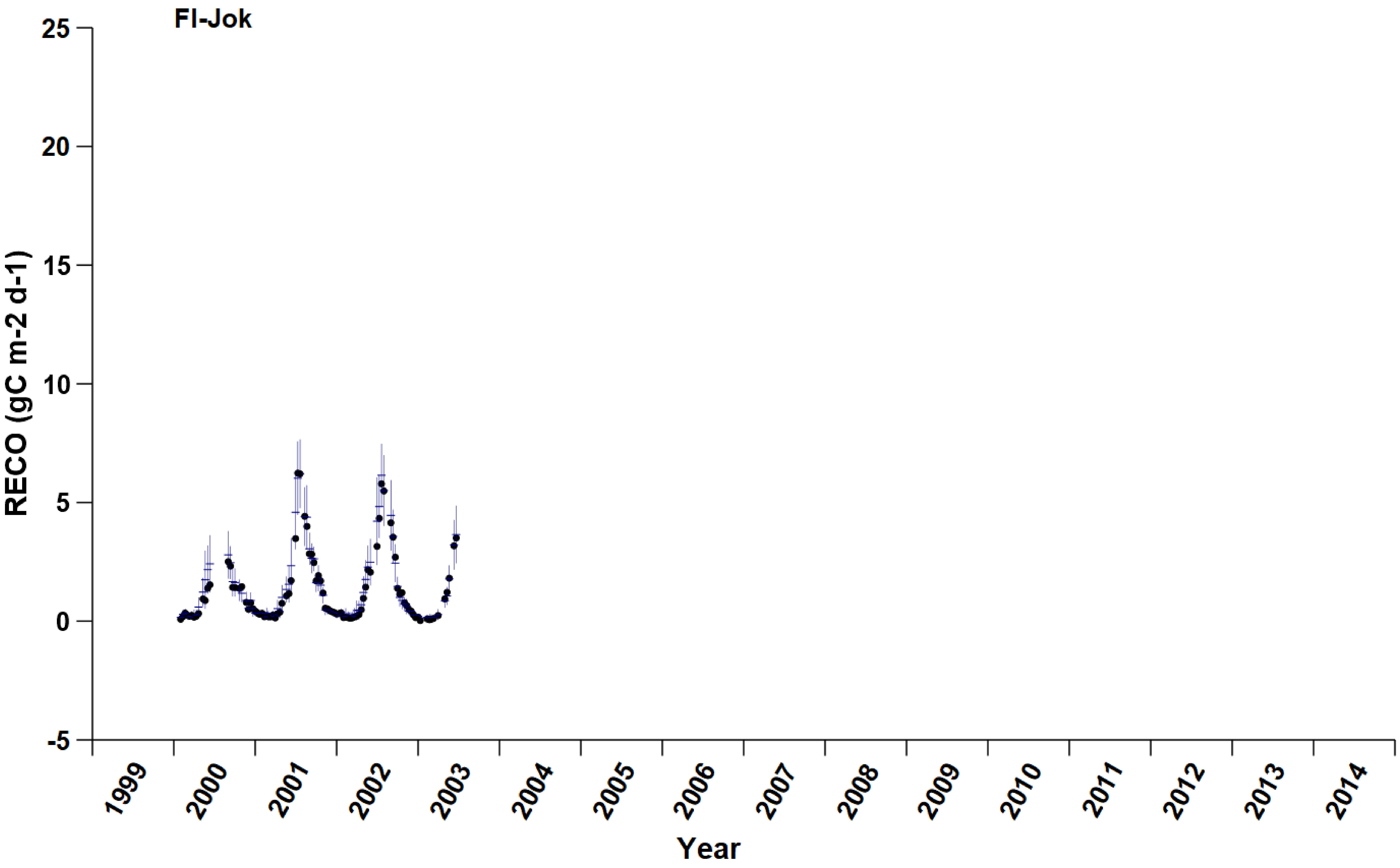

FI-Jok
RECO (gC m-2 d-1)
25
20
15
10
5
0
-5
1999
2000
2001
2002
2003
2004
2005
2006
2007
2008
2009
2010
2011
2012
2013
2014
Year

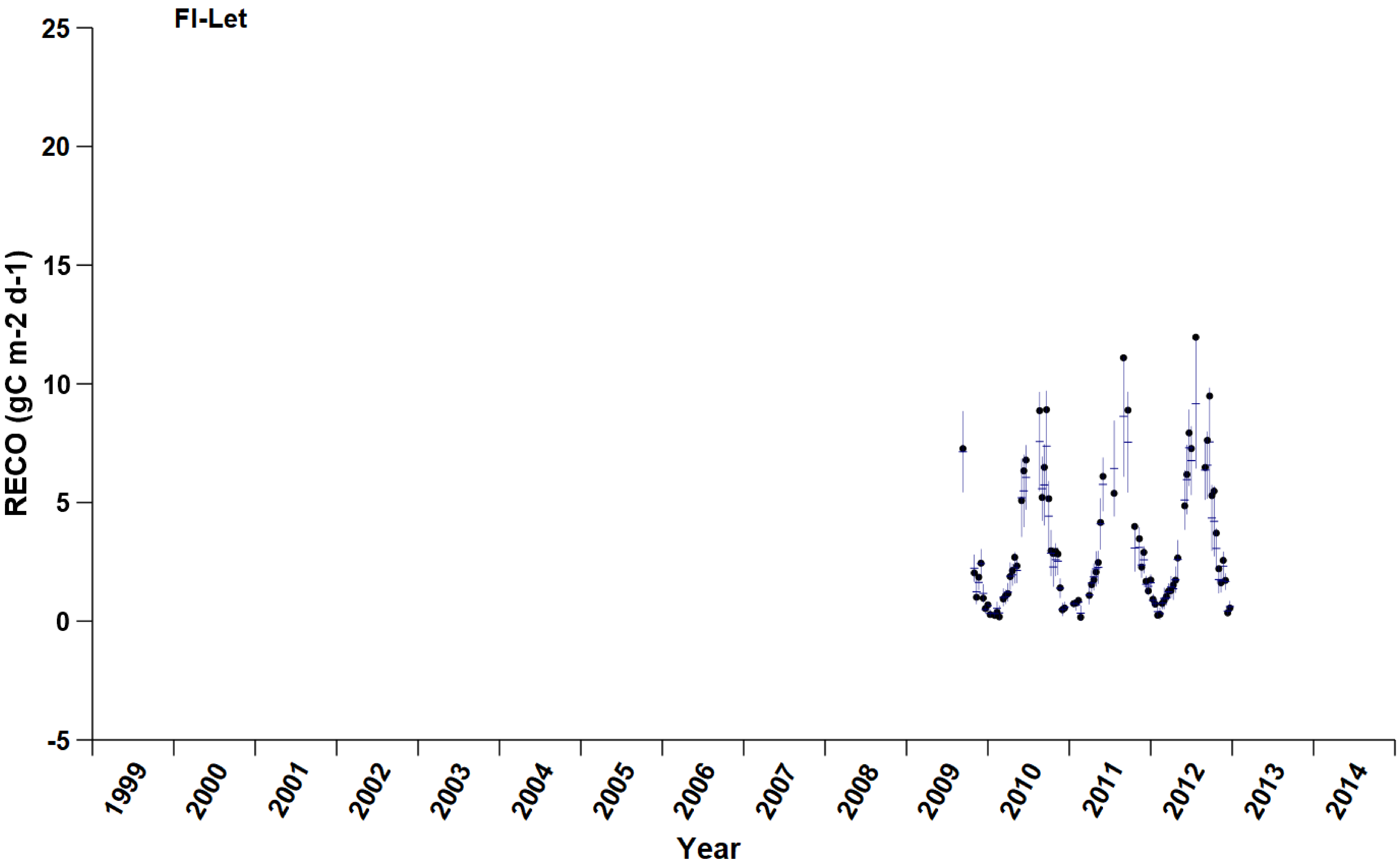

FI-Let
RECO (gC m-2 d-1)
25
20
15
10
5
0
-5
1999
2000
2001
2002
2003
2004
2005
2006
2007
2008
2009
2010
2011
2012
2013
2014
Year

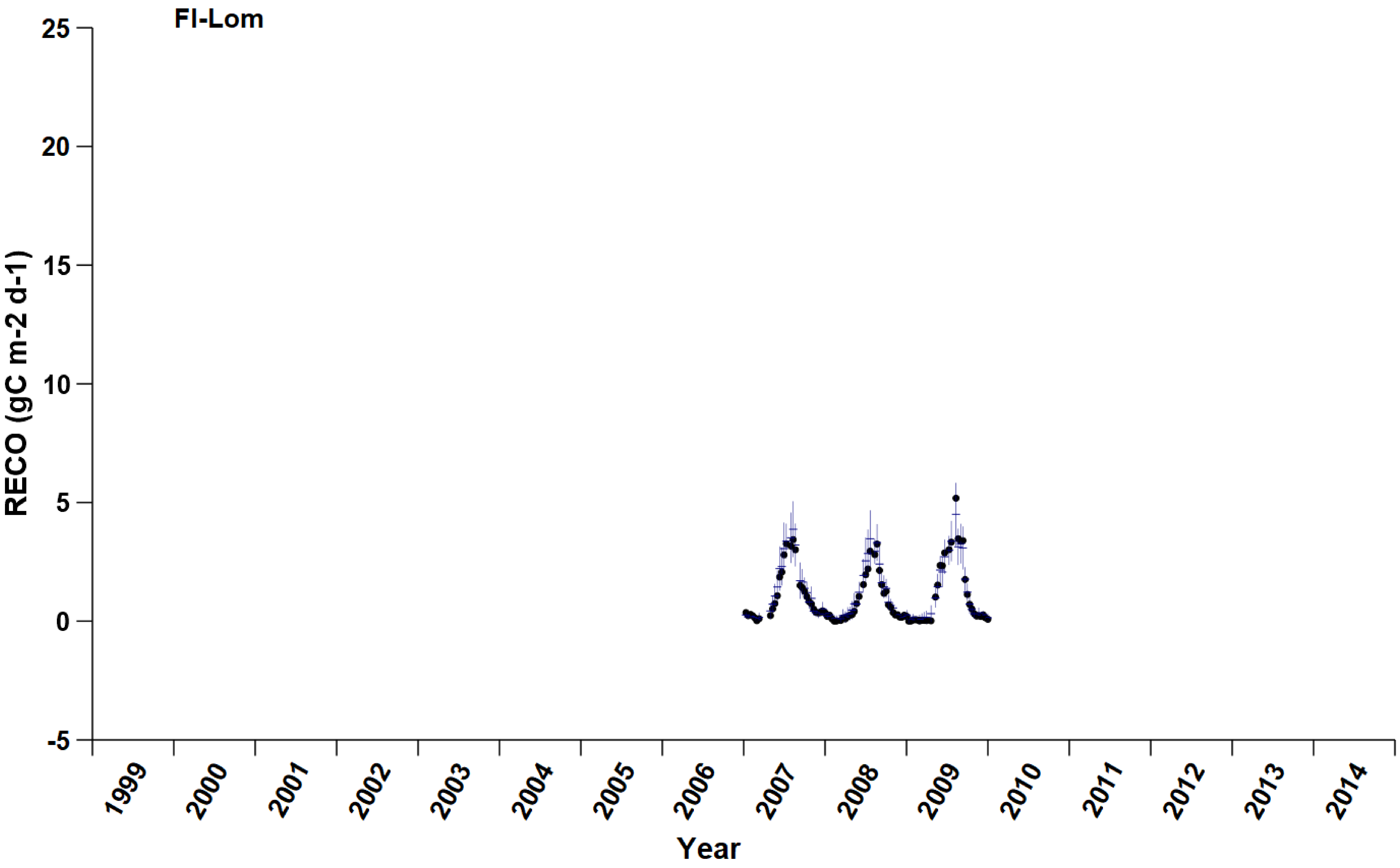

FI-Lom
25
20
15
10
5
0
-5
RECO (gC m-2 d-1)
1999
2000
2001
2002
2003
2004
2005
2006
2007
2008
2009
2010
2011
2012
2013
2014
Year

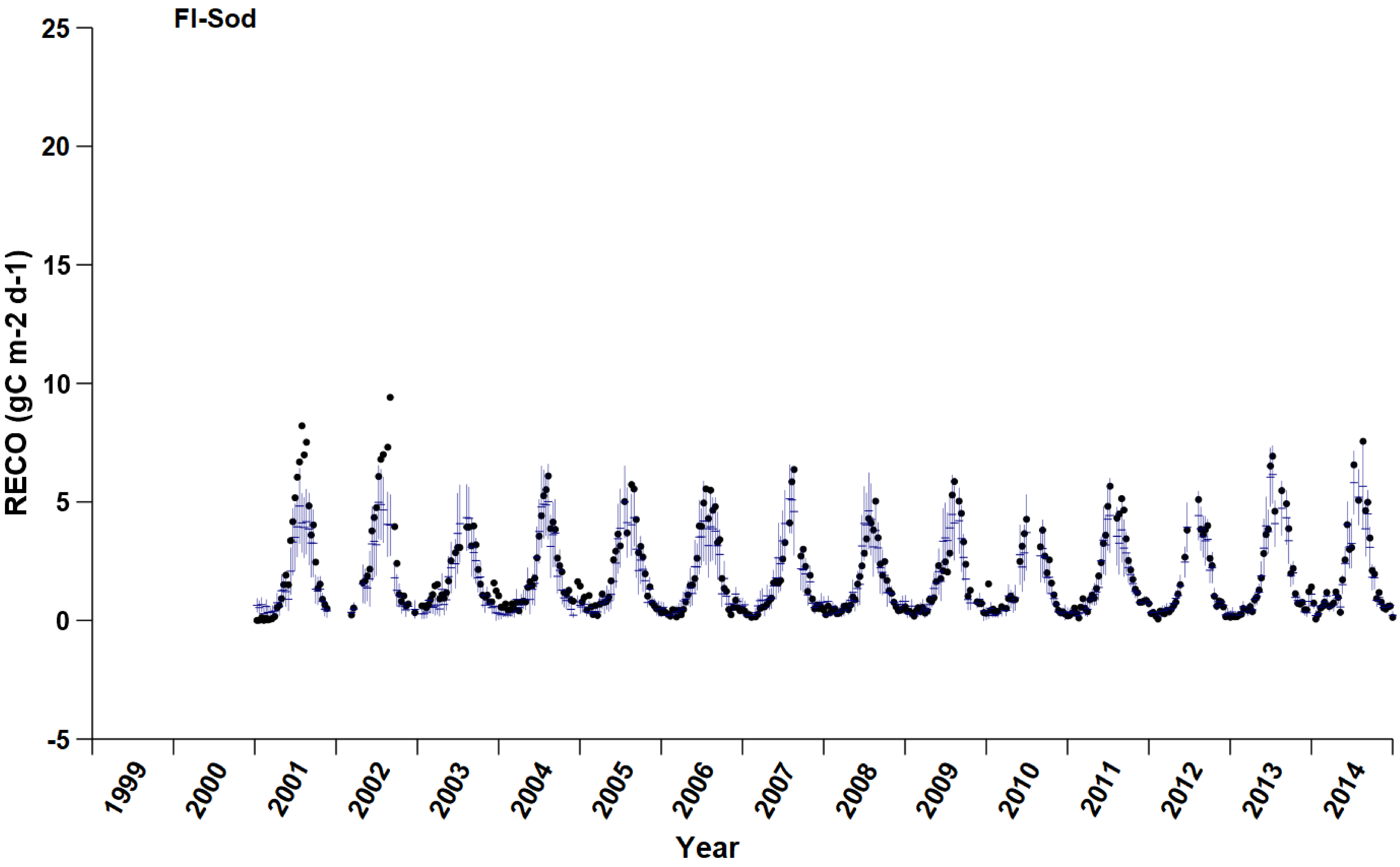
FI-Sod
RECO (gC m-2 d-1)
25
20
15
10
5
0
-5
1999
2000
2001
2002
2003
2004
2005
2006
2007
2008
2009
2010
2011
2012
2013
2014
Year

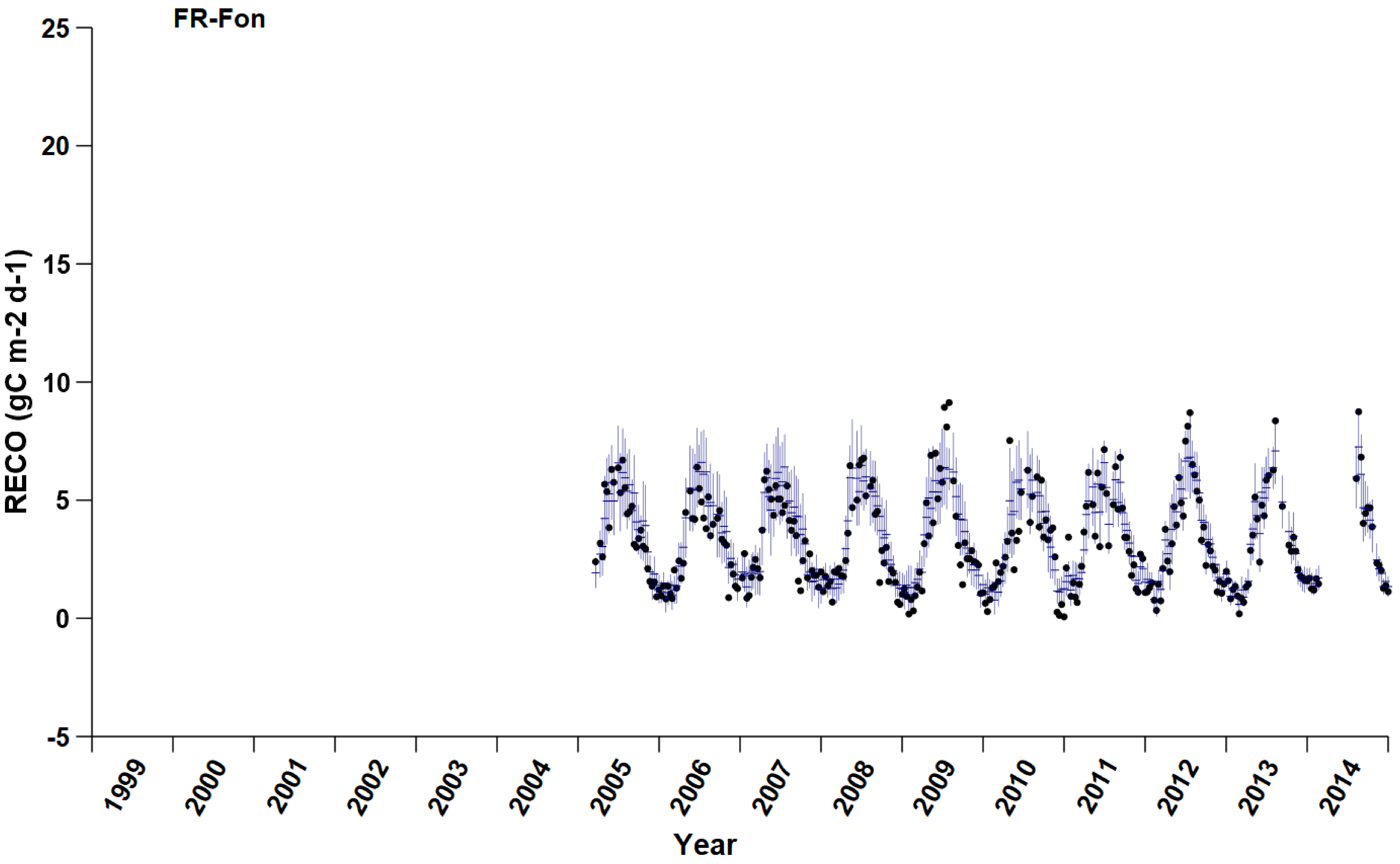
FR-Fon
25
20
15
10
5
0
-5
RECO (gC m-2 d-1)
1999
2000
2001
2002
2003
2004
2005
2006
2007
2008
2009
2010
2011
2012
2013
2014
Year

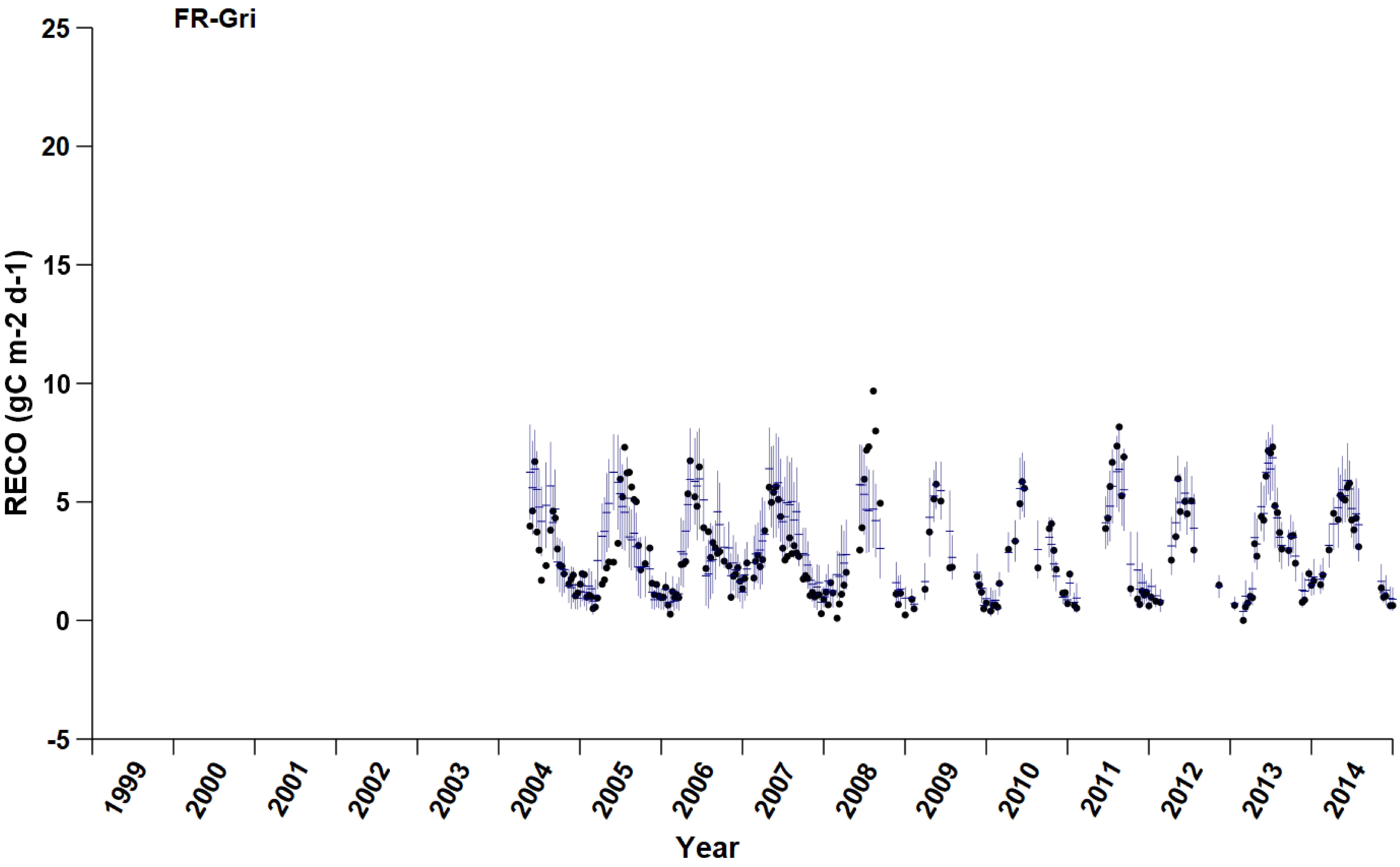
FR-Gri
RECO (gC m-2 d-1)
25
20
15
10
5
0
-5
1999
2000
2001
2002
2003
2004
2005
2006
2007
2008
2009
2010
2011
2012
2013
2014
Year

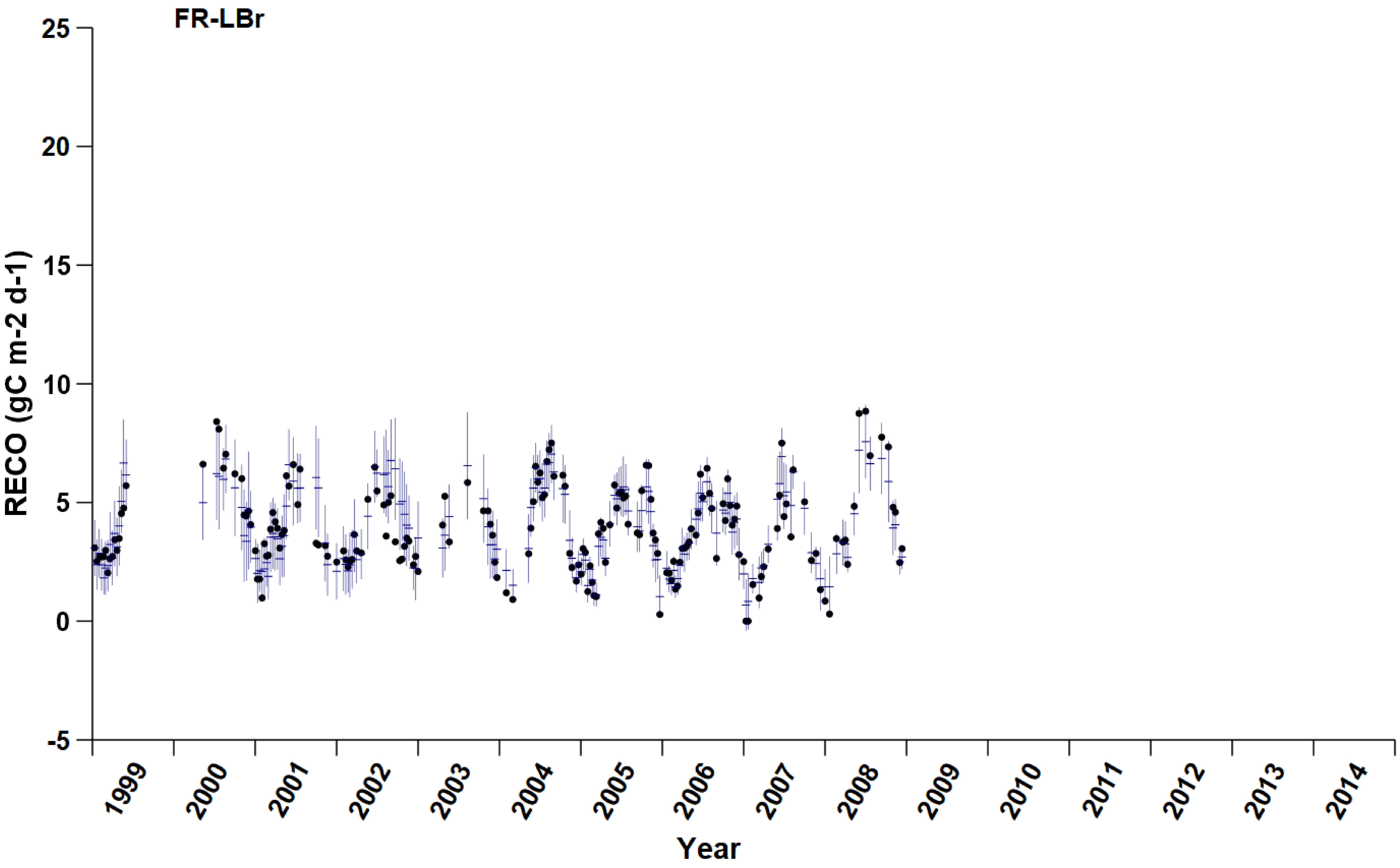

FR-LBr
RECO (gC m-2 d-1)
25
20
15
10
5
0
-5
1999
2000
2001
2002
2003
2004
2005
2006
2007
2008
2009
2010
2011
2012
2013
2014
Year

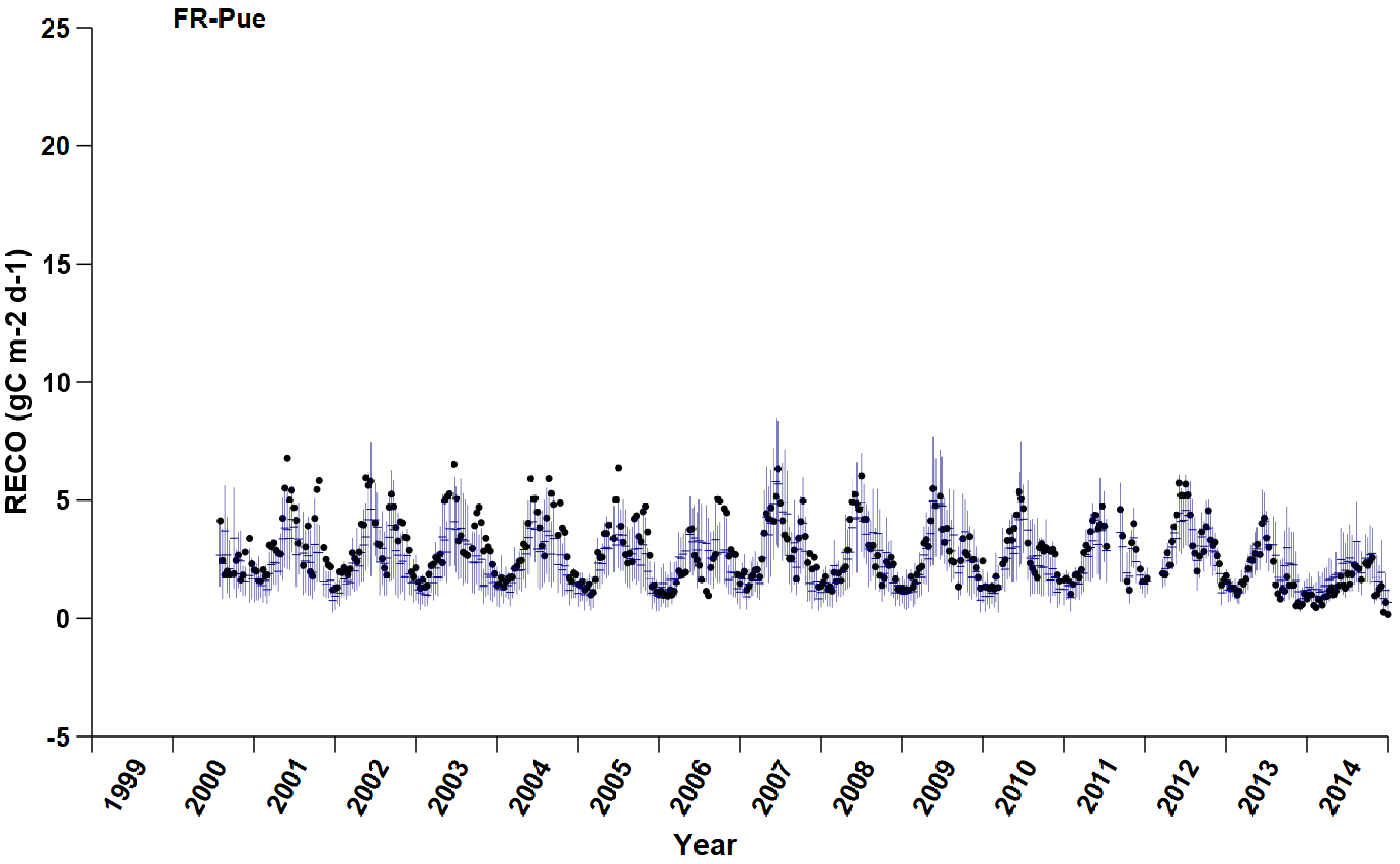
FR-Pue
RECO (gC m-2 d-1)
25
20
15
10
5
0
-5
1999
2000
2001
2002
2003
2004
2005
2006
2007
2008
2009
2010
2011
2012
2013
2014
Year

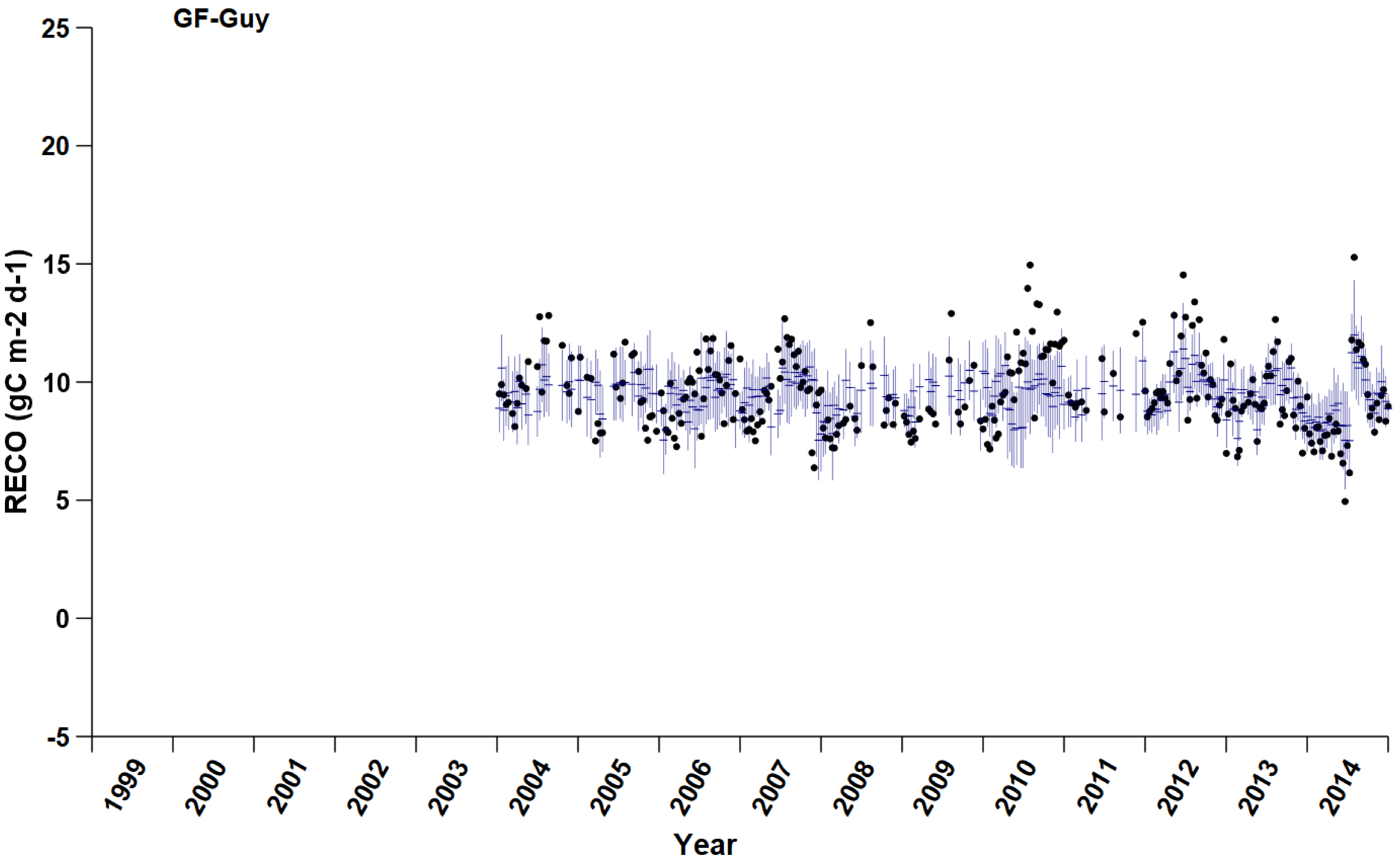

GF-Guy
RECO (gC m-2 d-1)
25
20
15
10
5
0
-5
1999
2000
2001
2002
2003
2004
2005
2006
2007
2008
2009
2010
2011
2012
2013
2014
Year

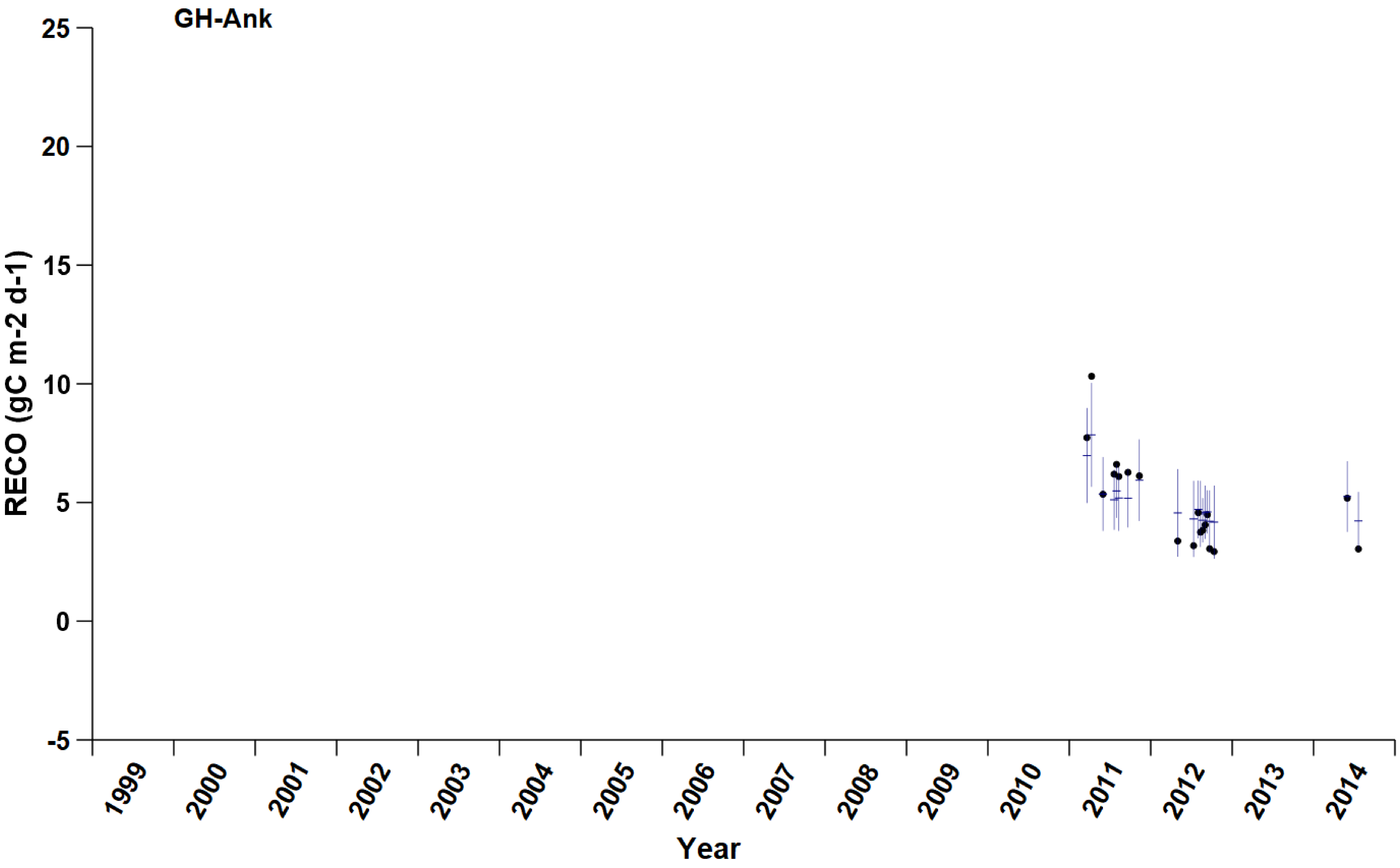

GH-Ank
RECO (gC m-2 d-1)
25
20
15
10
5
0
-5
1999
2000
2001
2002
2003
2004
2005
2006
2007
2008
2009
2010
2011
2012
2013
2014
Year

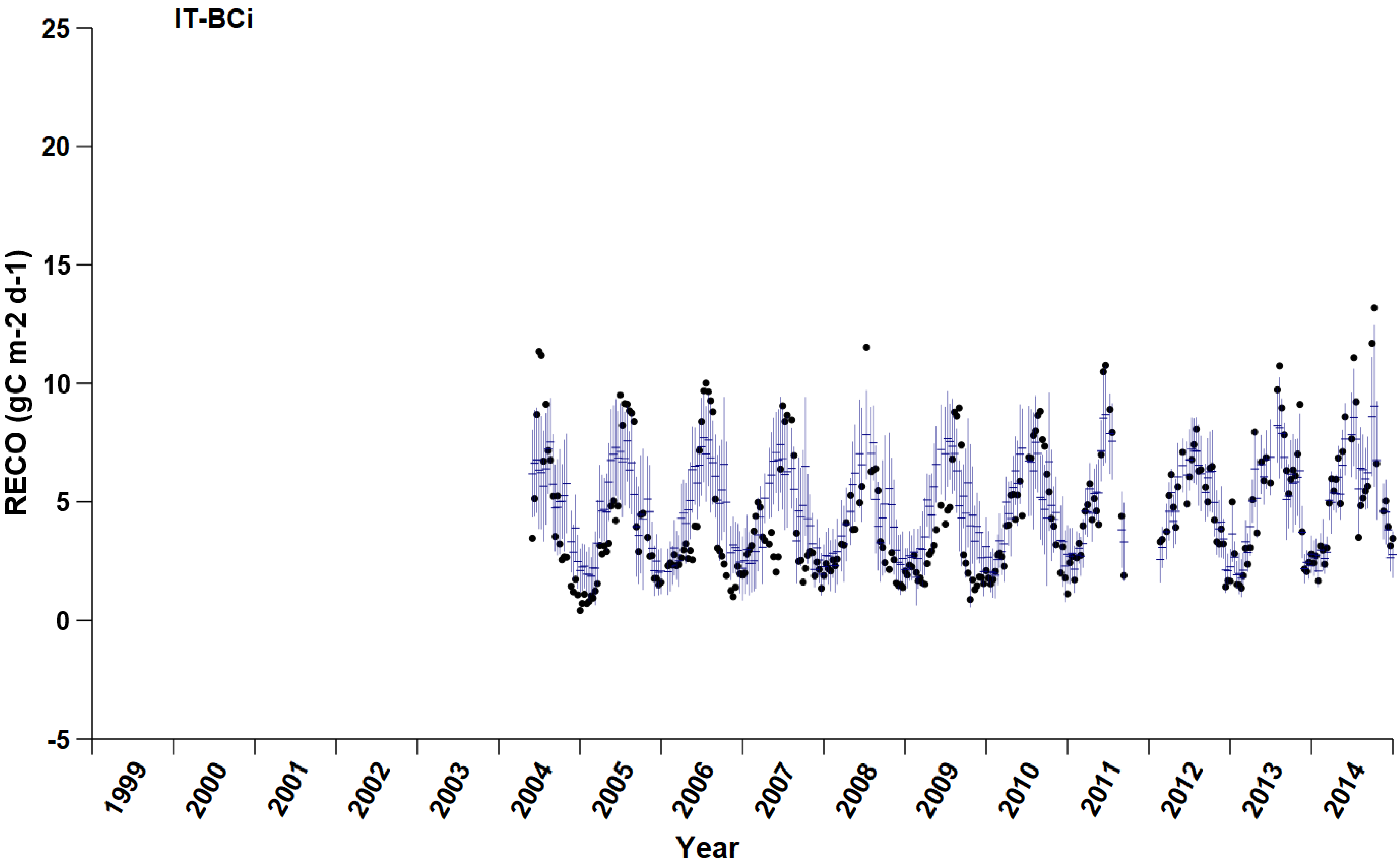
IT-BCi
RECO (gC m-2 d-1)
25
20
15
10
5
0
-5
1999
2000
2001
2002
2003
2004
2005
2006
2007
2008
2009
2010
2011
2012
2013
2014
Year

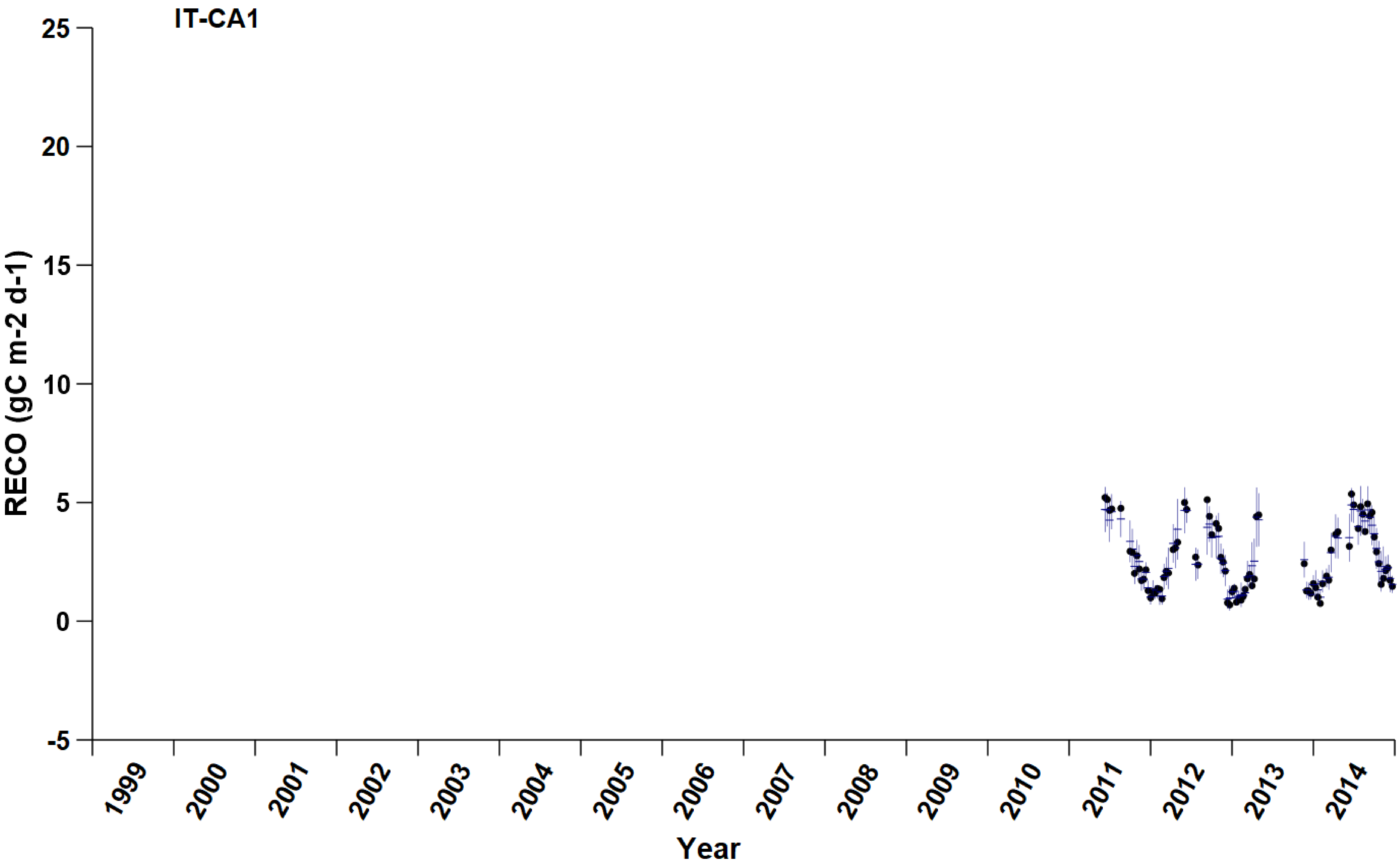

IT-CA1
RECO (gC m-2 d-1)
25
20
15
10
5
0
-5
1999
2000
2001
2002
2003
2004
2005
2006
2007
2008
2009
2010
2011
2012
2013
2014
Year

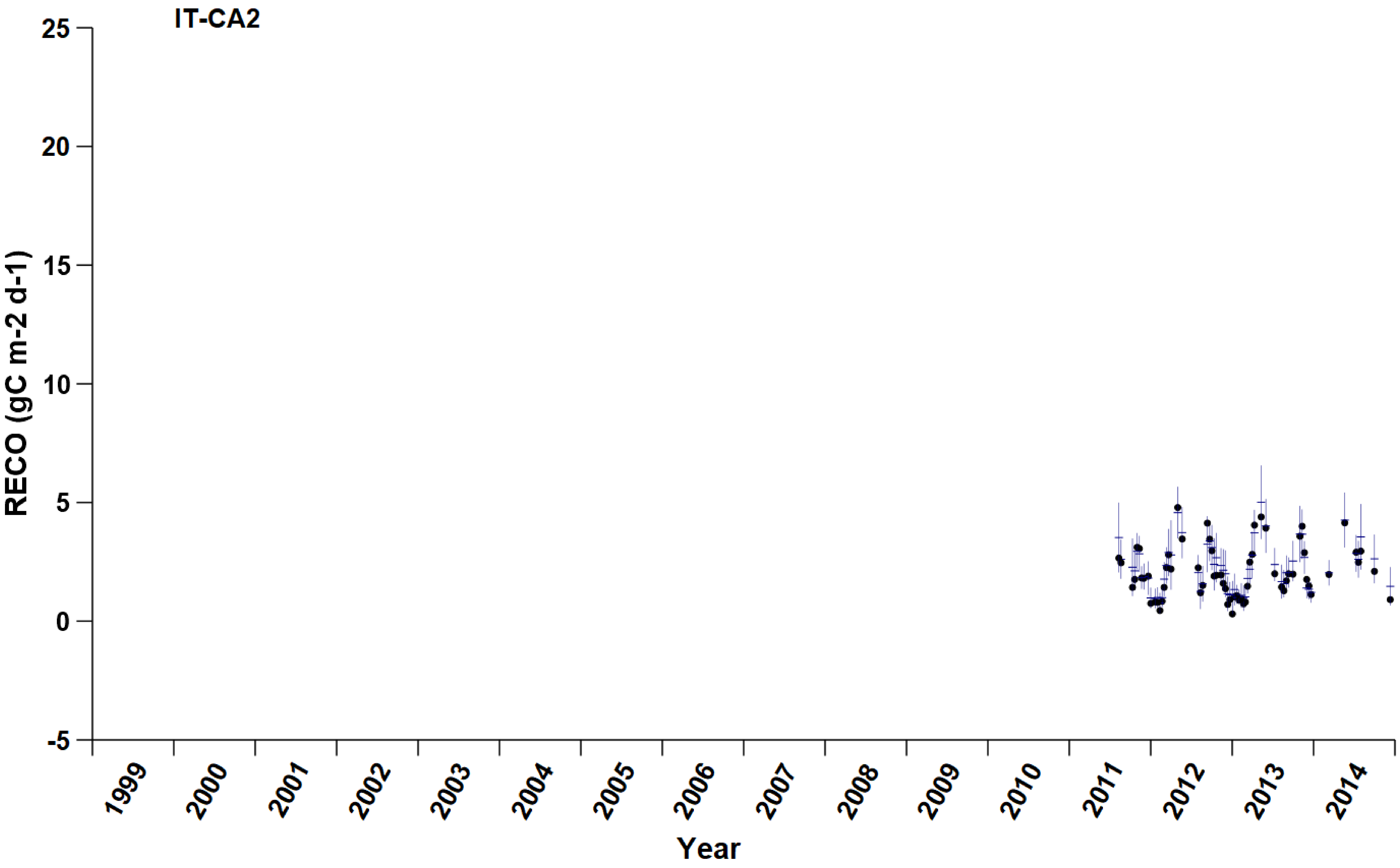
IT-CA2
RECO (gC m-2 d-1)
25
20
15
10
5
0
-5
1999
2000
2001
2002
2003
2004
2005
2006
2007
2008
2009
2010
2011
2012
2013
2014
Year

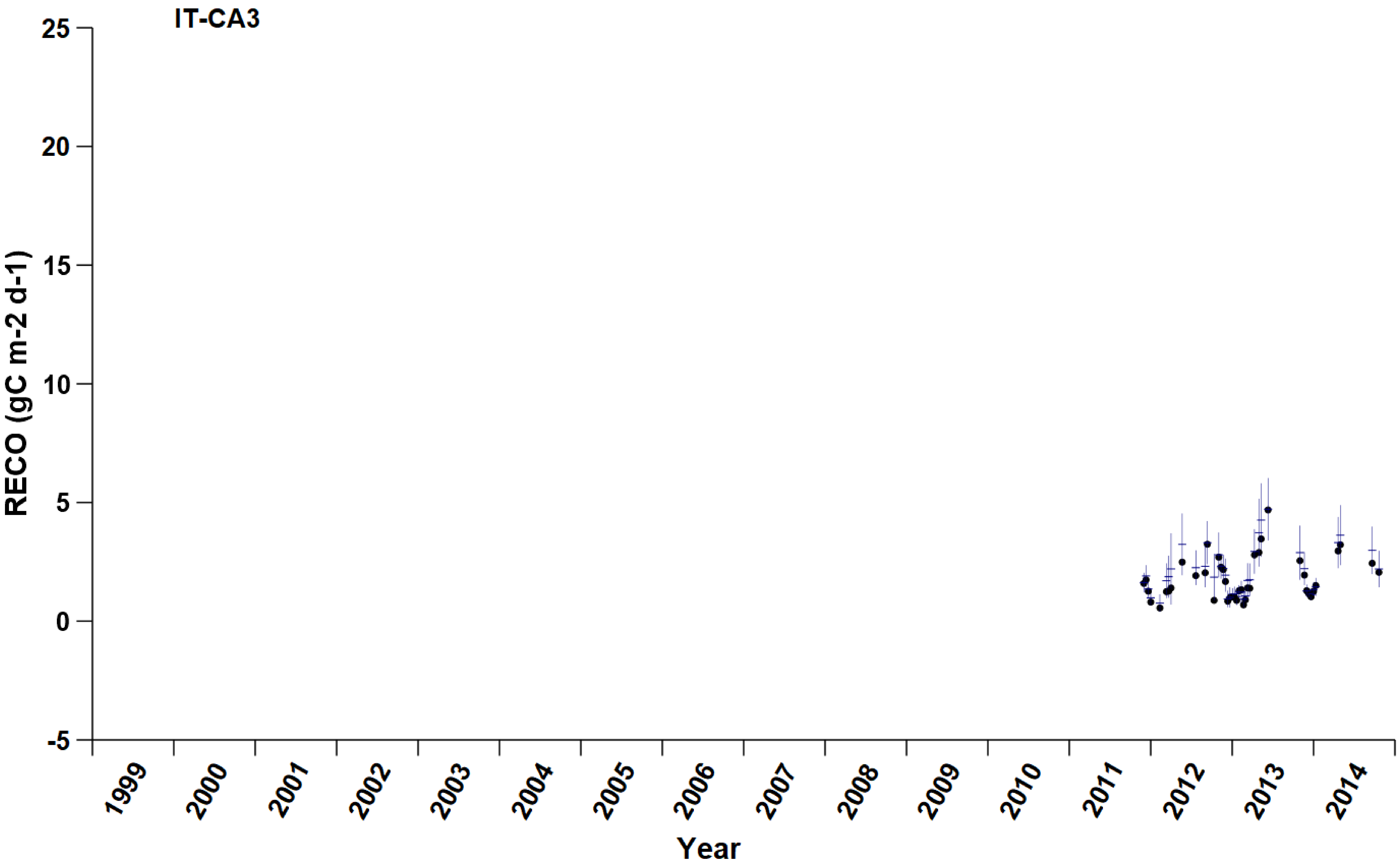

IT-CA3
25
20
15
10
5
0
-5
RECO (gC m-2 d-1)
1999
2000
2001
2002
2003
2004
2005
2006
2007
2008
2009
2010
2011
2012
2013
2014
Year

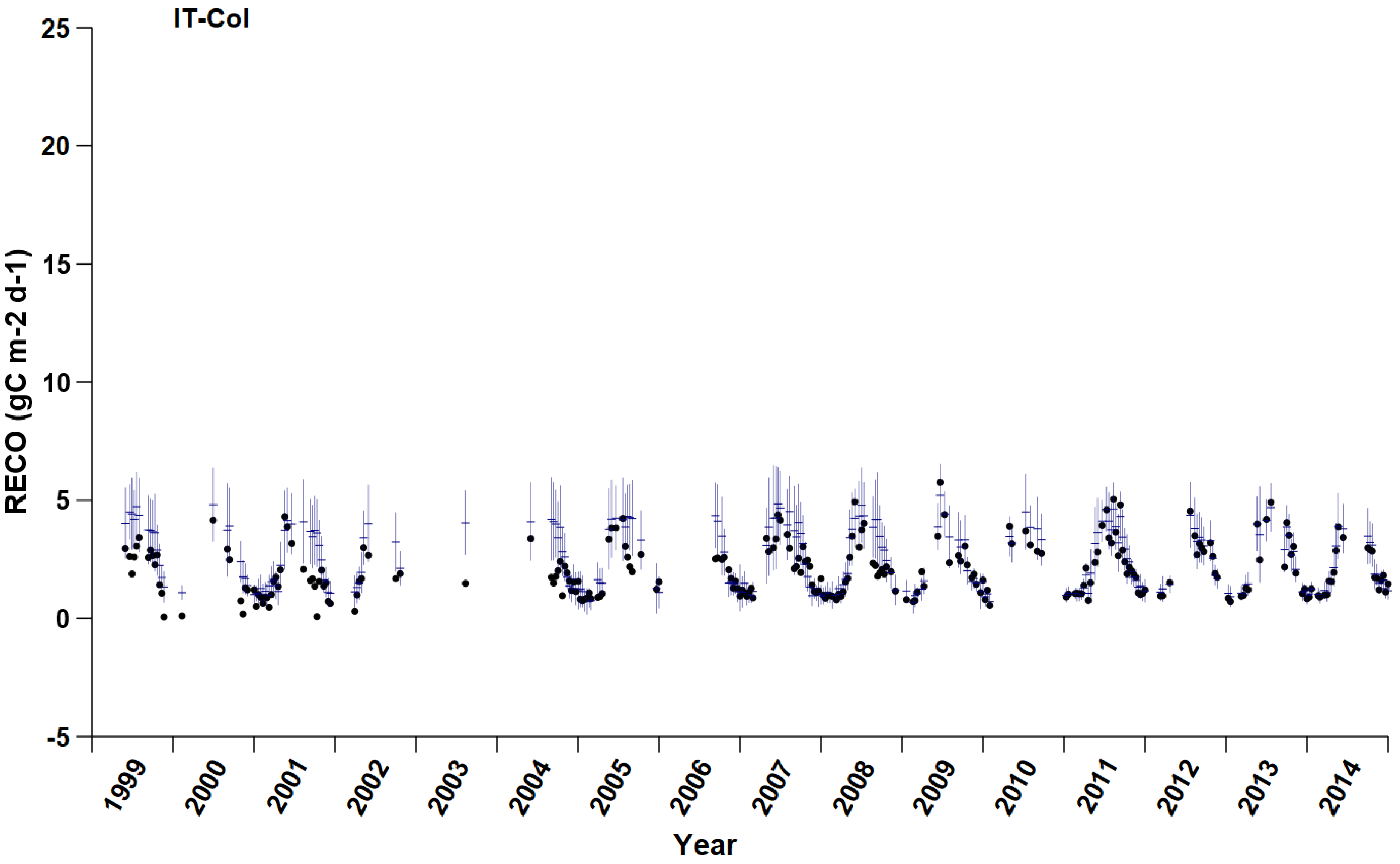
IT-Col
RECO (gC m-2 d-1)
25
20
15
10
5
0
-5
1999
2000
2001
2002
2003
2004
2005
2006
2007
2008
2009
2010
2011
2012
2013
2014
Year

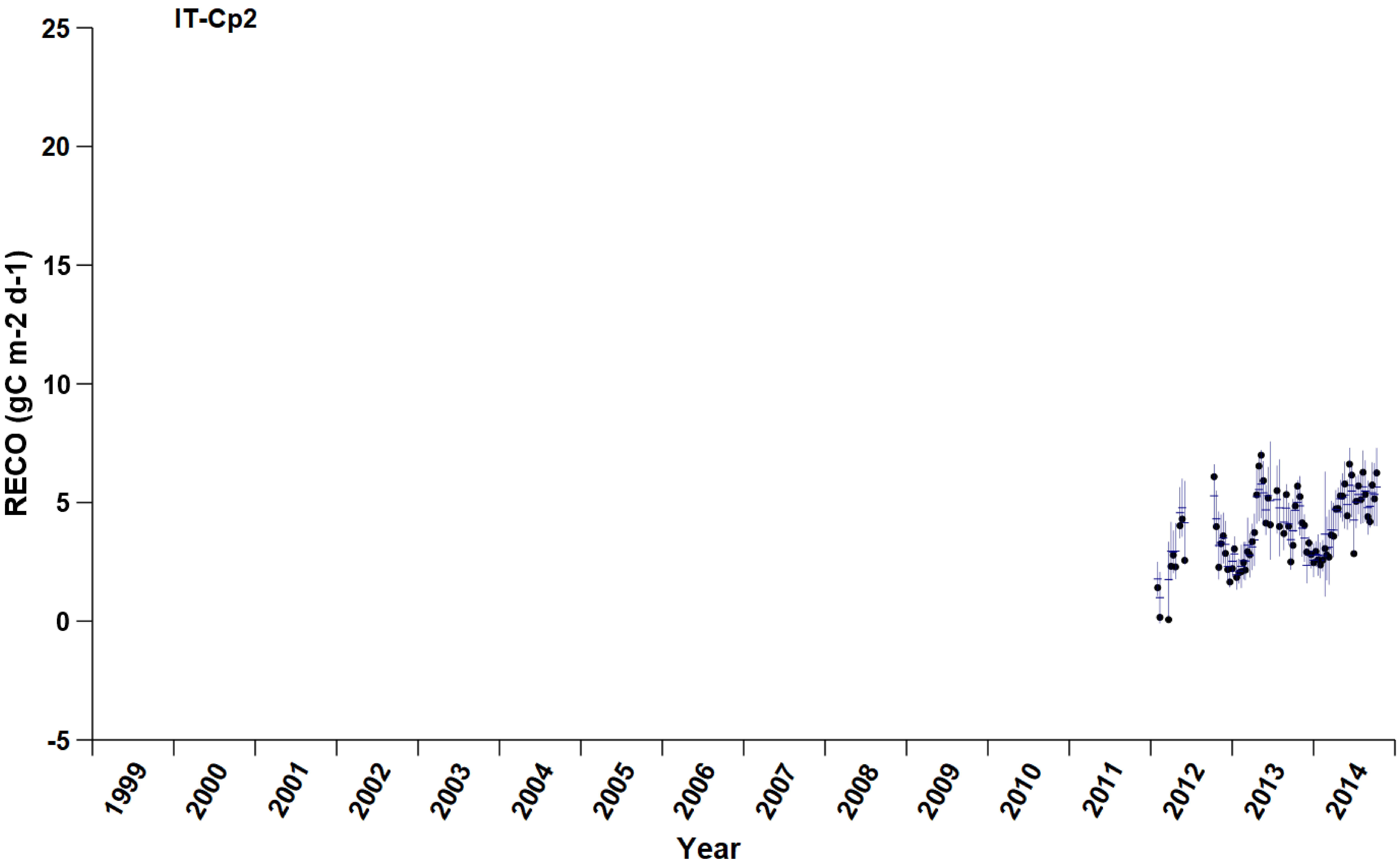
IT-Cp2
RECO (gC m-2 d-1)
25
20
15
10
5
0
-5
1999
2000
2001
2002
2003
2004
2005
2006
2007
2008
2009
2010
2011
2012
2013
2014
Year

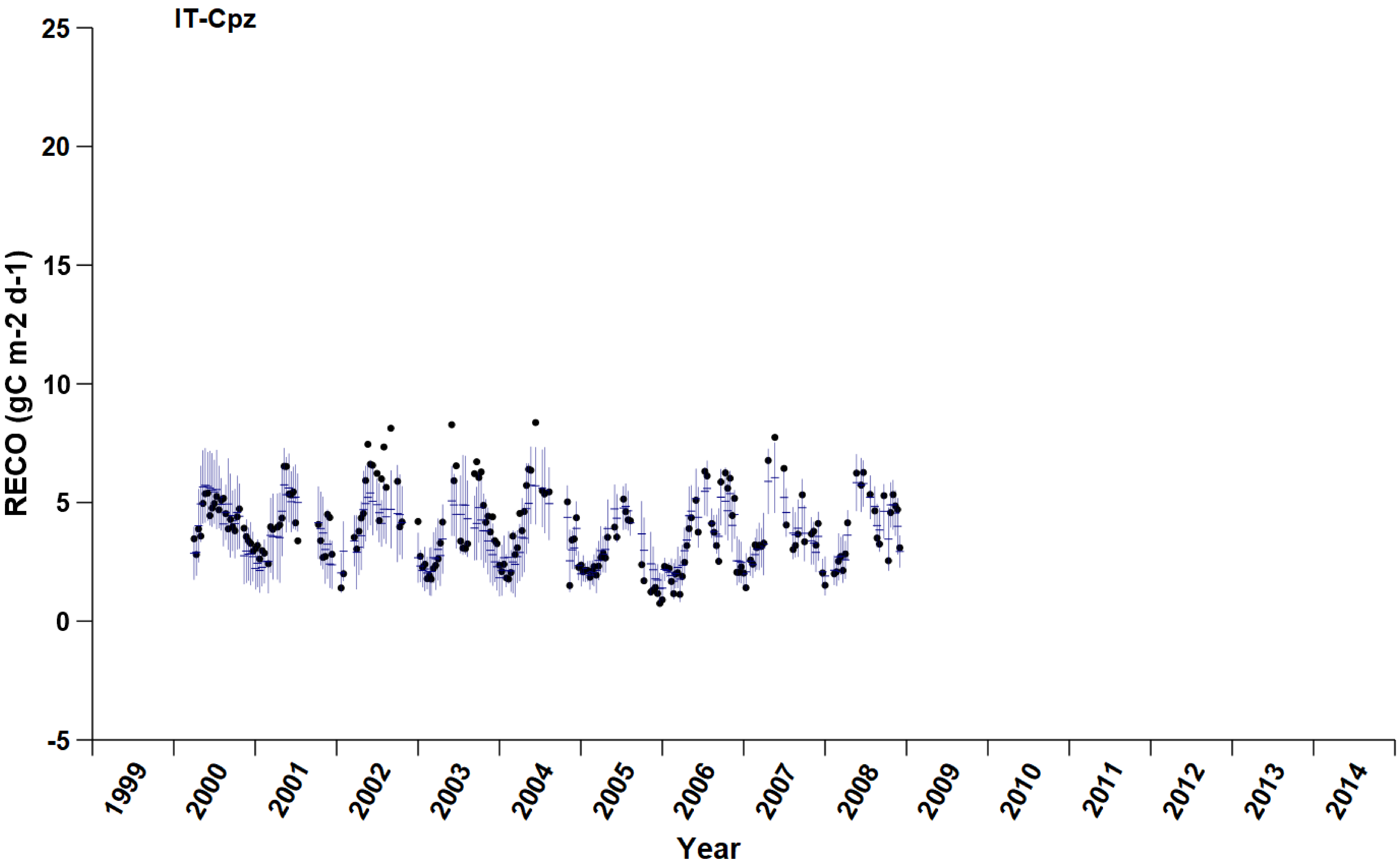

IT-Cpz
RECO (gC m-2 d-1)
25
20
15
10
5
0
-5
1999
2000
2001
2002
2003
2004
2005
2006
2007
2008
2009
2010
2011
2012
2013
2014
Year

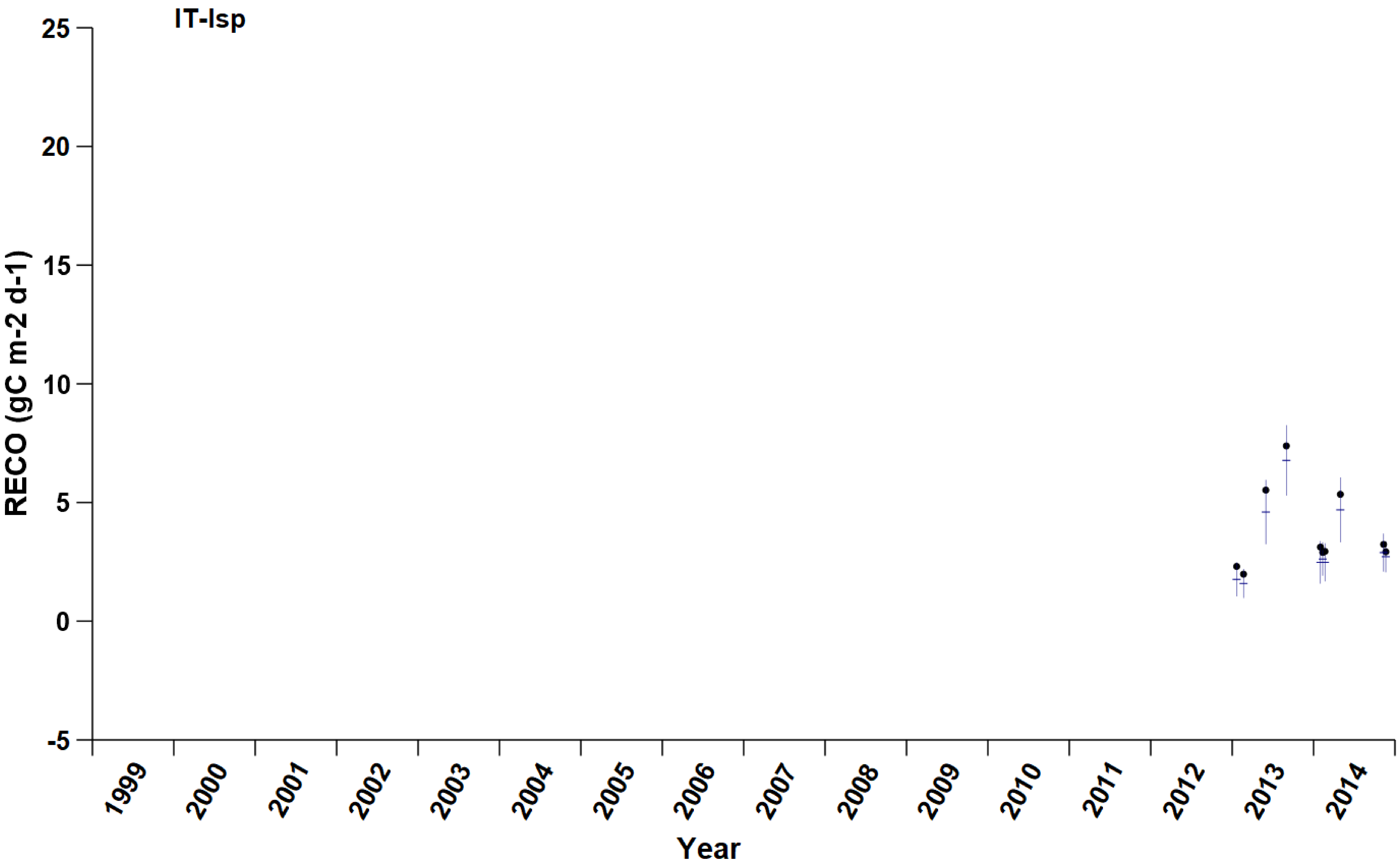
IT-Isp
RECO (gC m-2 d-1)
25
20
15
10
5
0
-5
1999
2000
2001
2002
2003
2004
2005
2006
2007
2008
2009
2010
2011
2012
2013
2014
Year

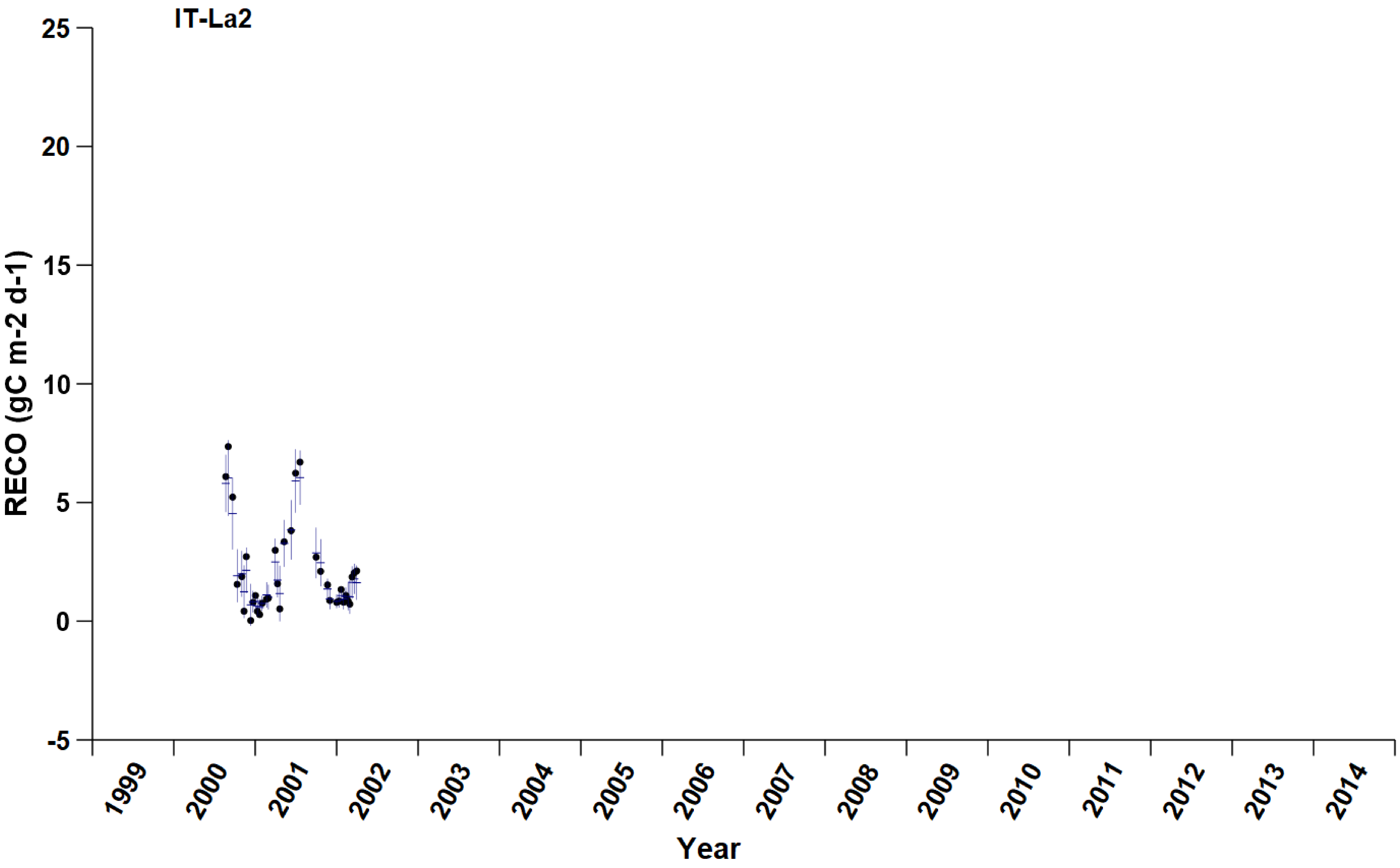
IT-La2
RECO (gC m-2 d-1)
25
20
15
10
5
0
-5
1999
2000
2001
2002
2003
2004
2005
2006
2007
2008
2009
2010
2011
2012
2013
2014
Year

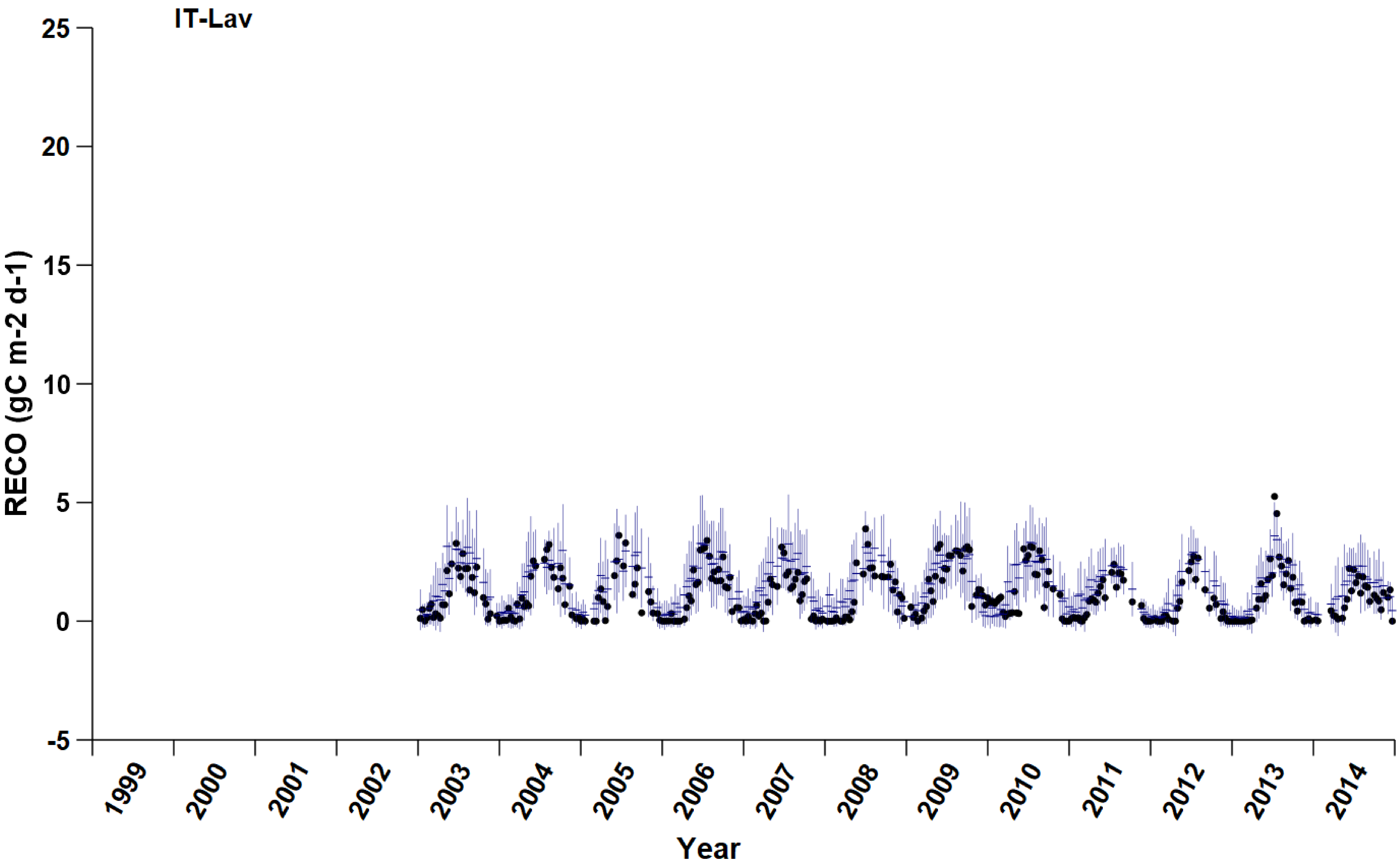

IT-Lav
RECO (gC m-2 d-1)
25
20
15
10
5
0
-5
1999
2000
2001
2002
2003
2004
2005
2006
2007
2008
2009
2010
2011
2012
2013
2014
Year

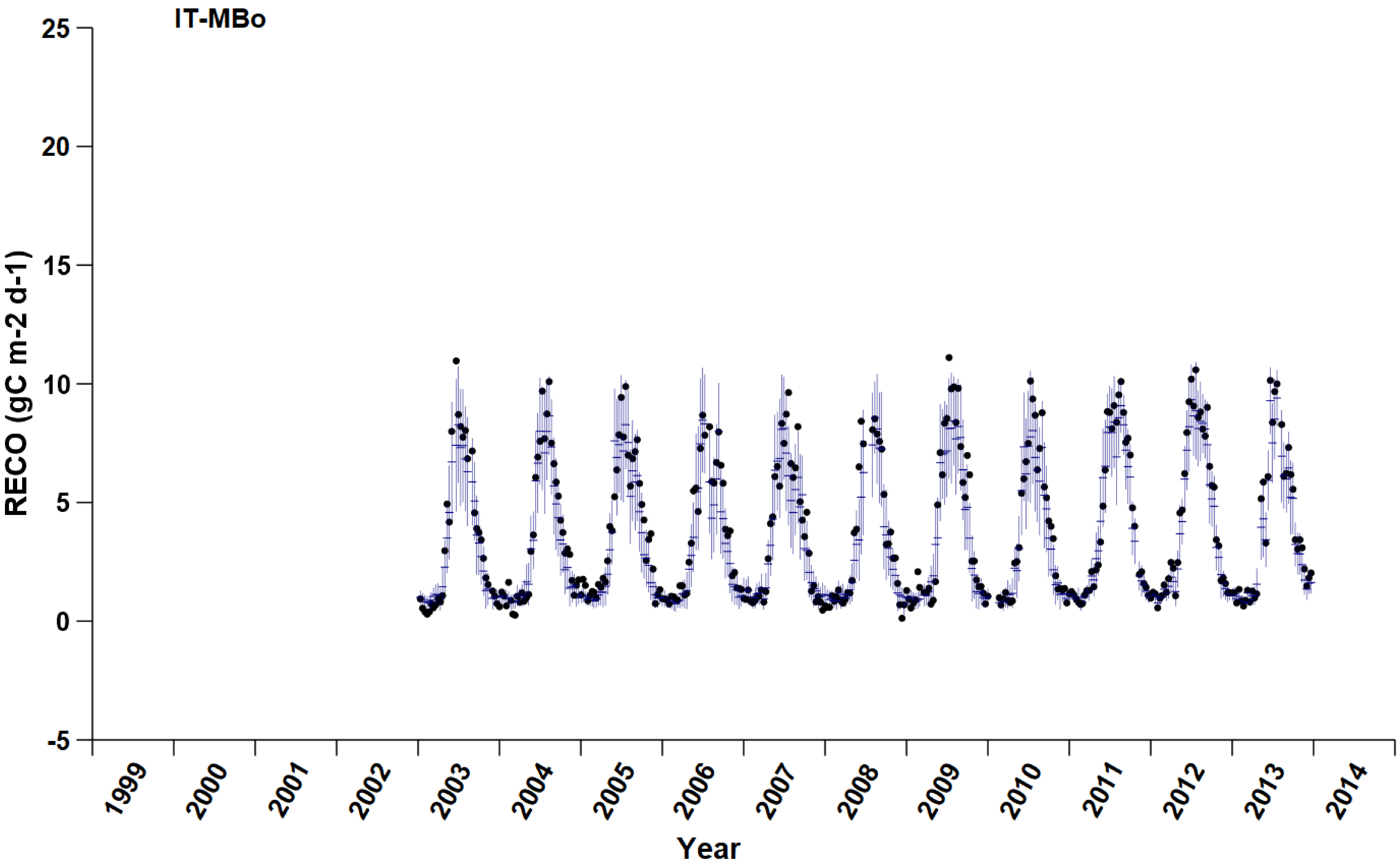
IT-MBo
RECO (gC m-2 d-1)
25
20
15
10
5
0
-5
1999
2000
2001
2002
2003
2004
2005
2006
2007
2008
2009
2010
2011
2012
2013
2014
Year

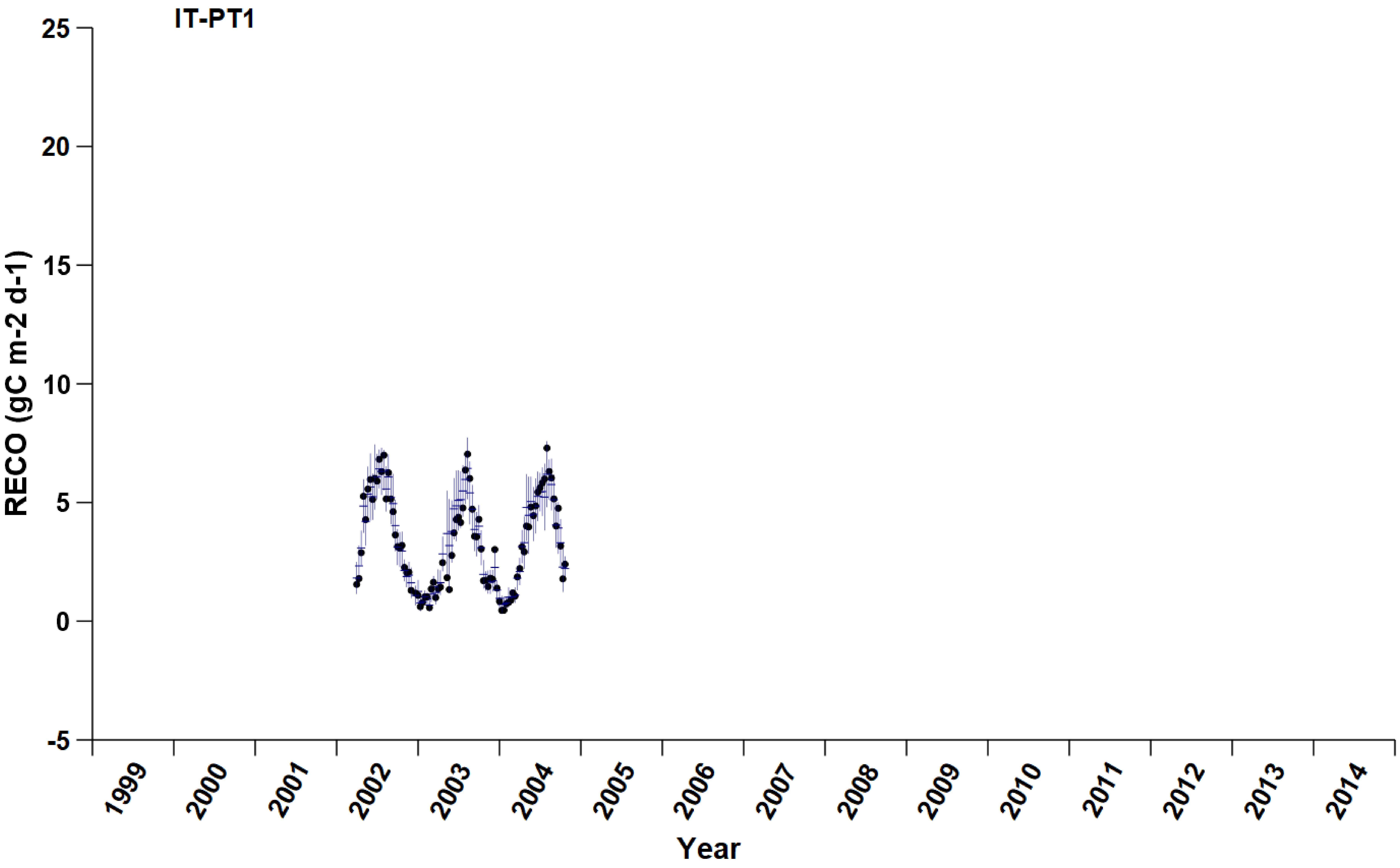

IT-PT1
RECO (gC m-2 d-1)
25
20
15
10
5
0
-5
1999
2000
2001
2002
2003
2004
2005
2006
2007
2008
2009
2010
2011
2012
2013
2014
Year

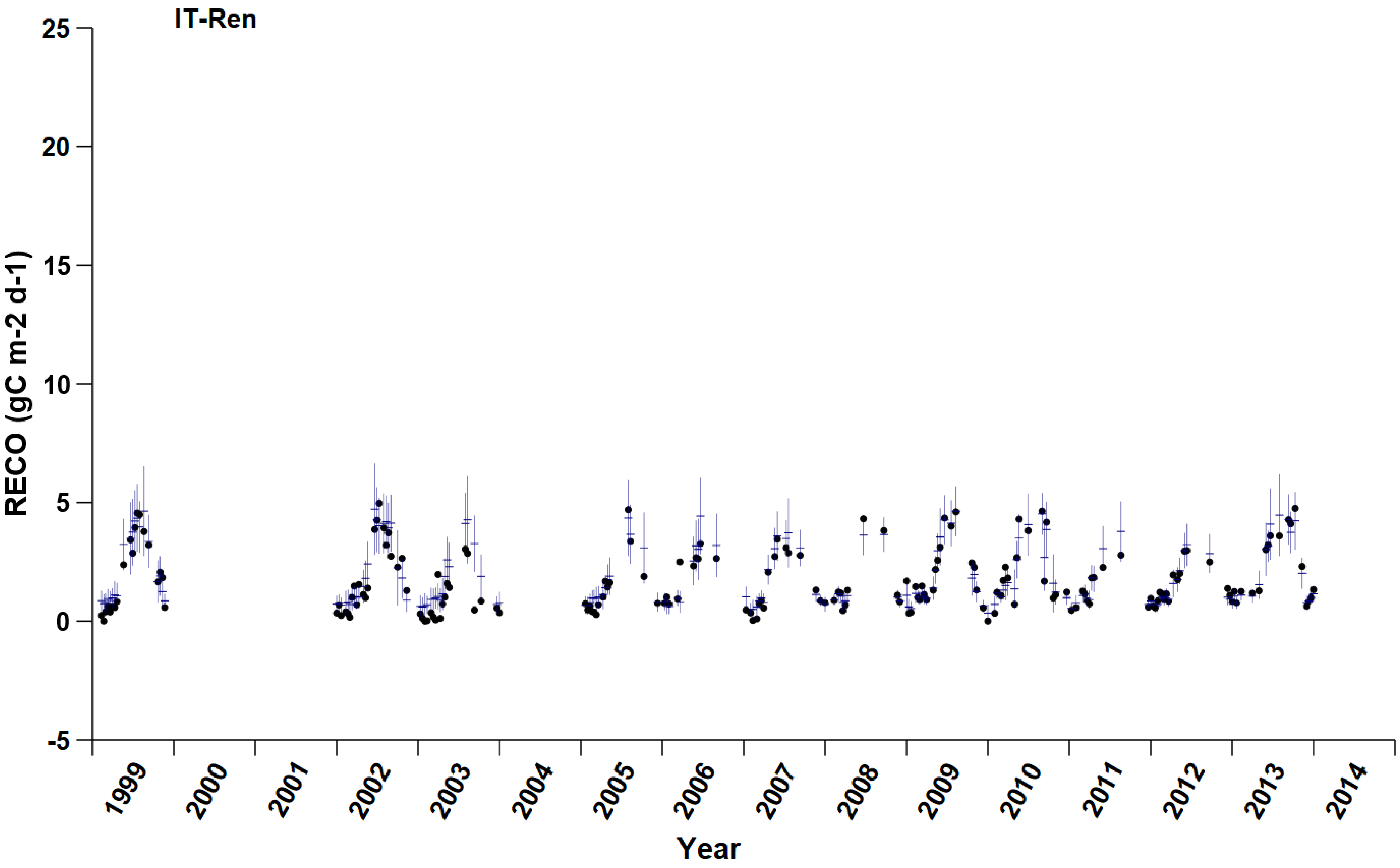
IT-Ren
RECO (gC m-2 d-1)
25
20
15
10
5
0
-5
1999
2000
2001
2002
2003
2004
2005
2006
2007
2008
2009
2010
2011
2012
2013
2014
Year

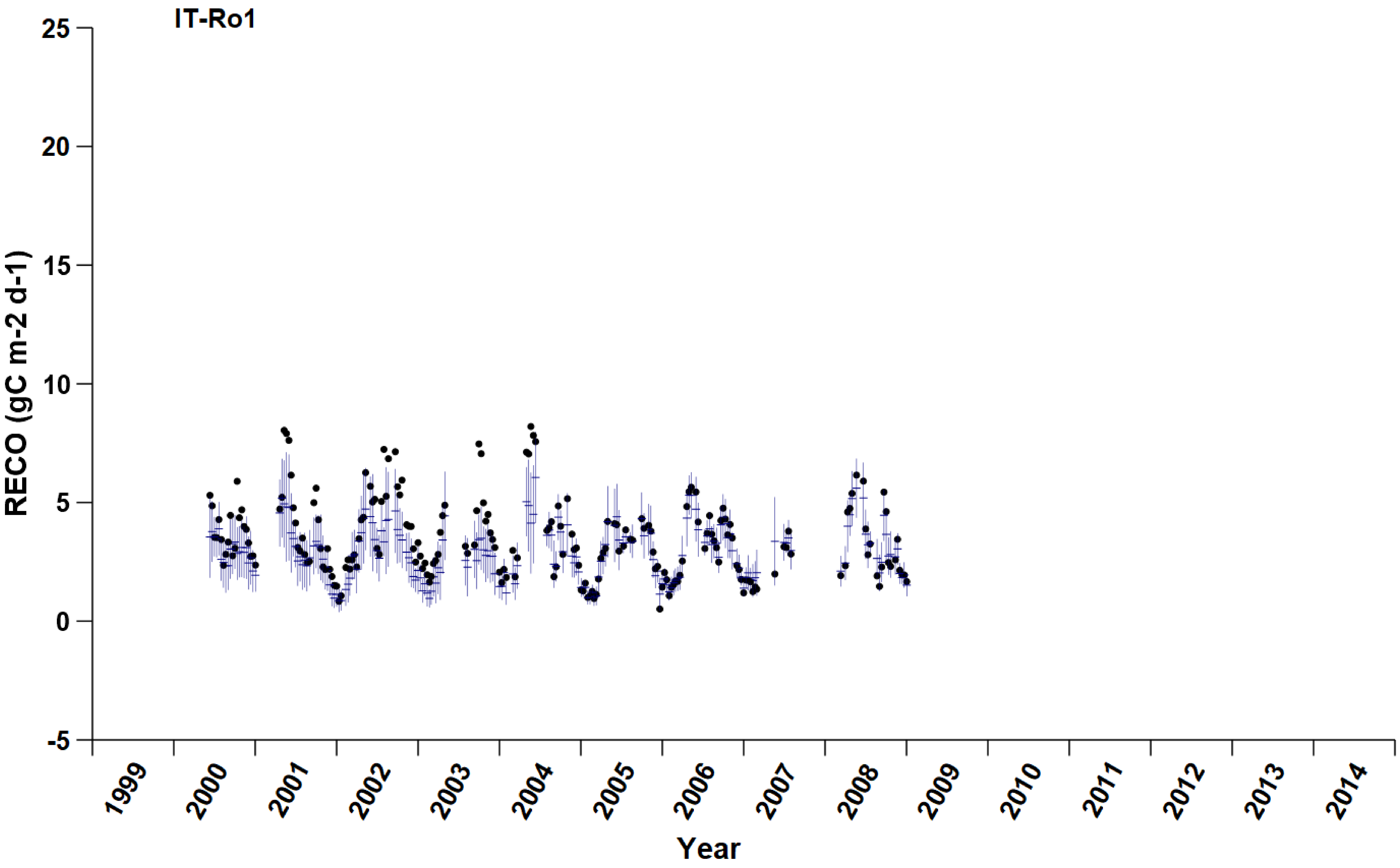

IT-Ro1
RECO (gC m-2 d-1)
25
20
15
10
5
0
-5
1999
2000
2001
2002
2003
2004
2005
2006
2007
2008
2009
2010
2011
2012
2013
2014
Year

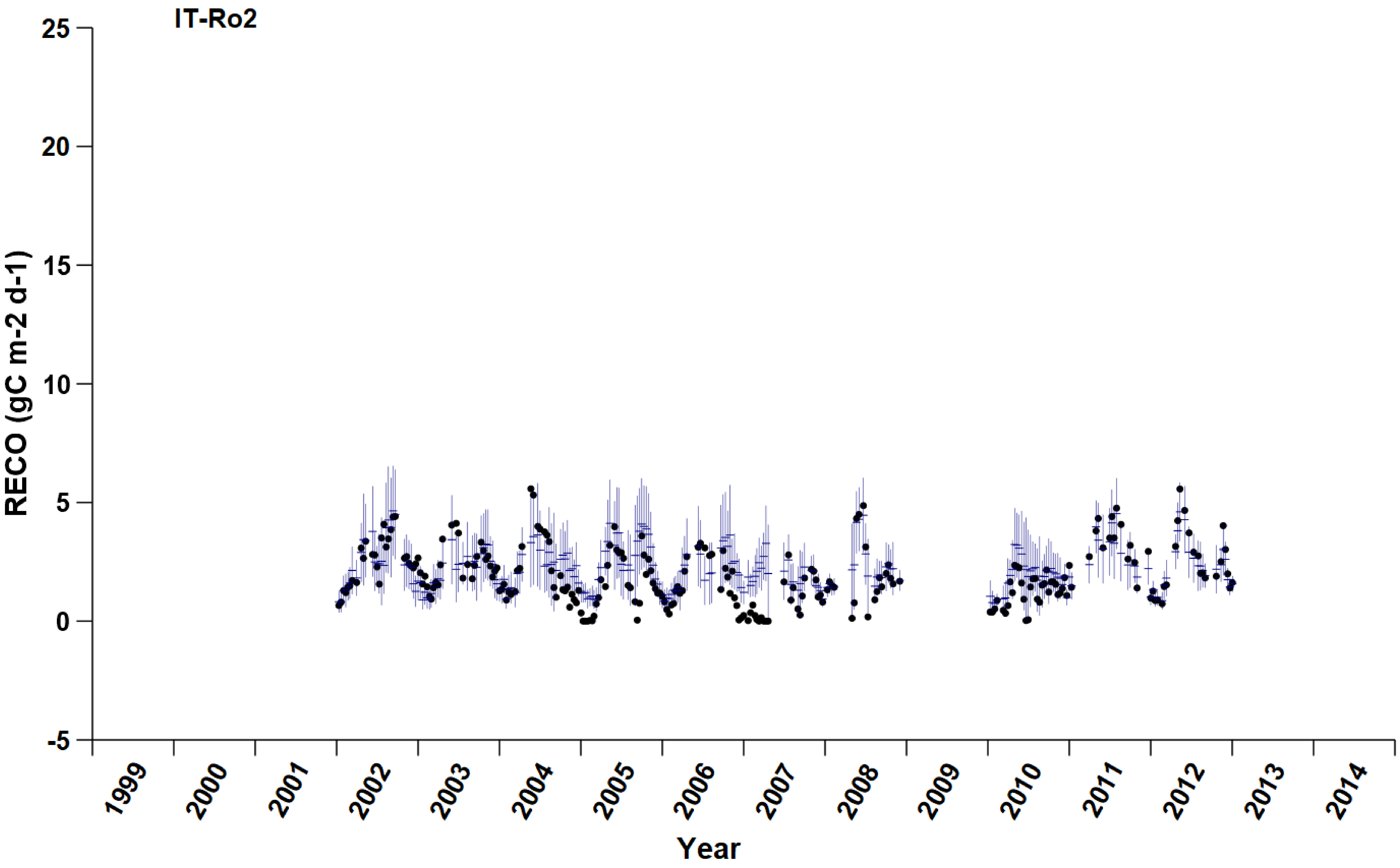
IT-Ro2
RECO (gC m-2 d-1)
25
20
15
10
5
0
-5
1999
2000
2001
2002
2003
2004
2005
2006
2007
2008
2009
2010
2011
2012
2013
2014
Year

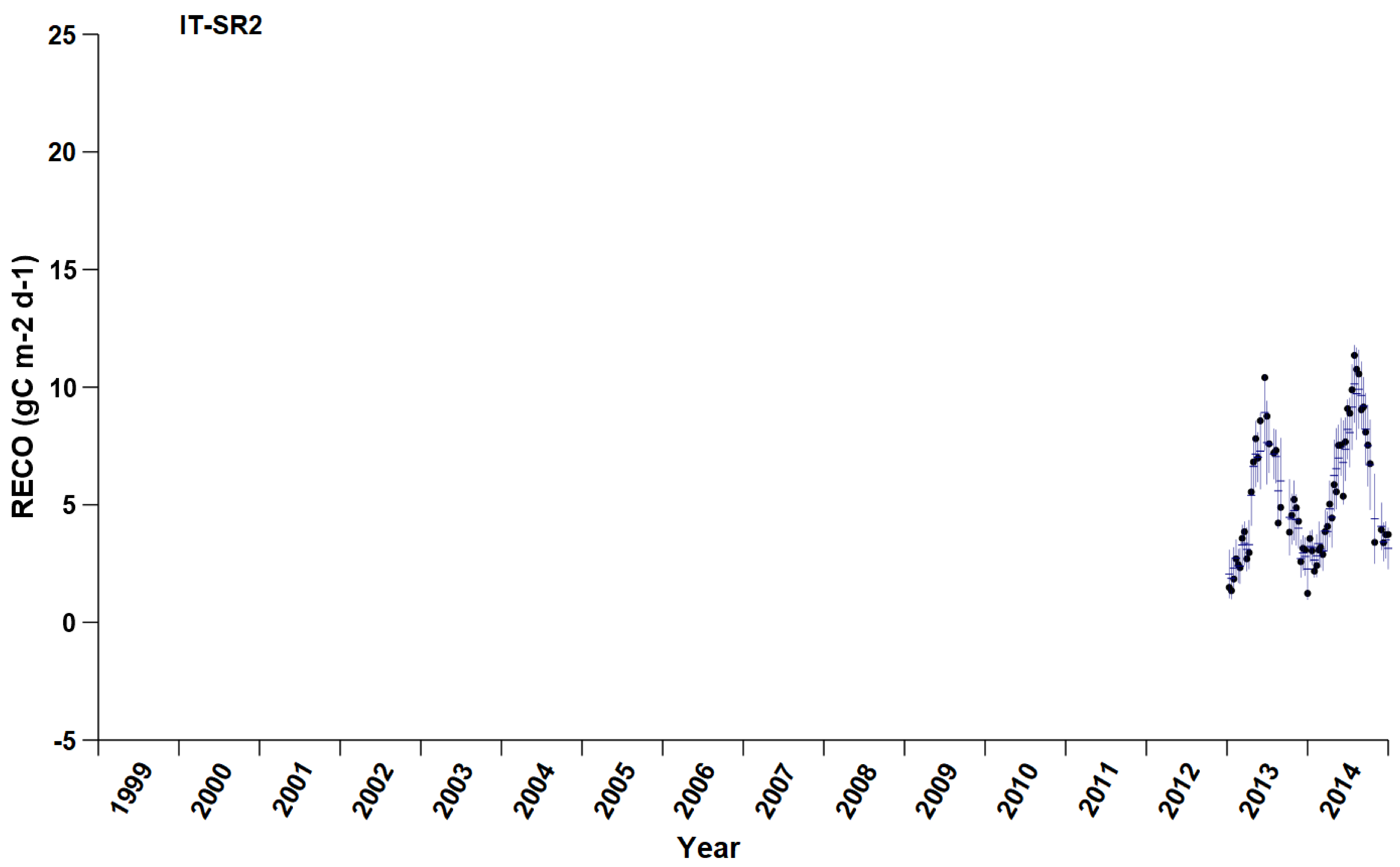

IT-SR2
RECO (gC m-2 d-1)
25
20
15
10
5
0
-5
1999
2000
2001
2002
2003
2004
2005
2006
2007
2008
2009
2010
2011
2012
2013
2014
Year

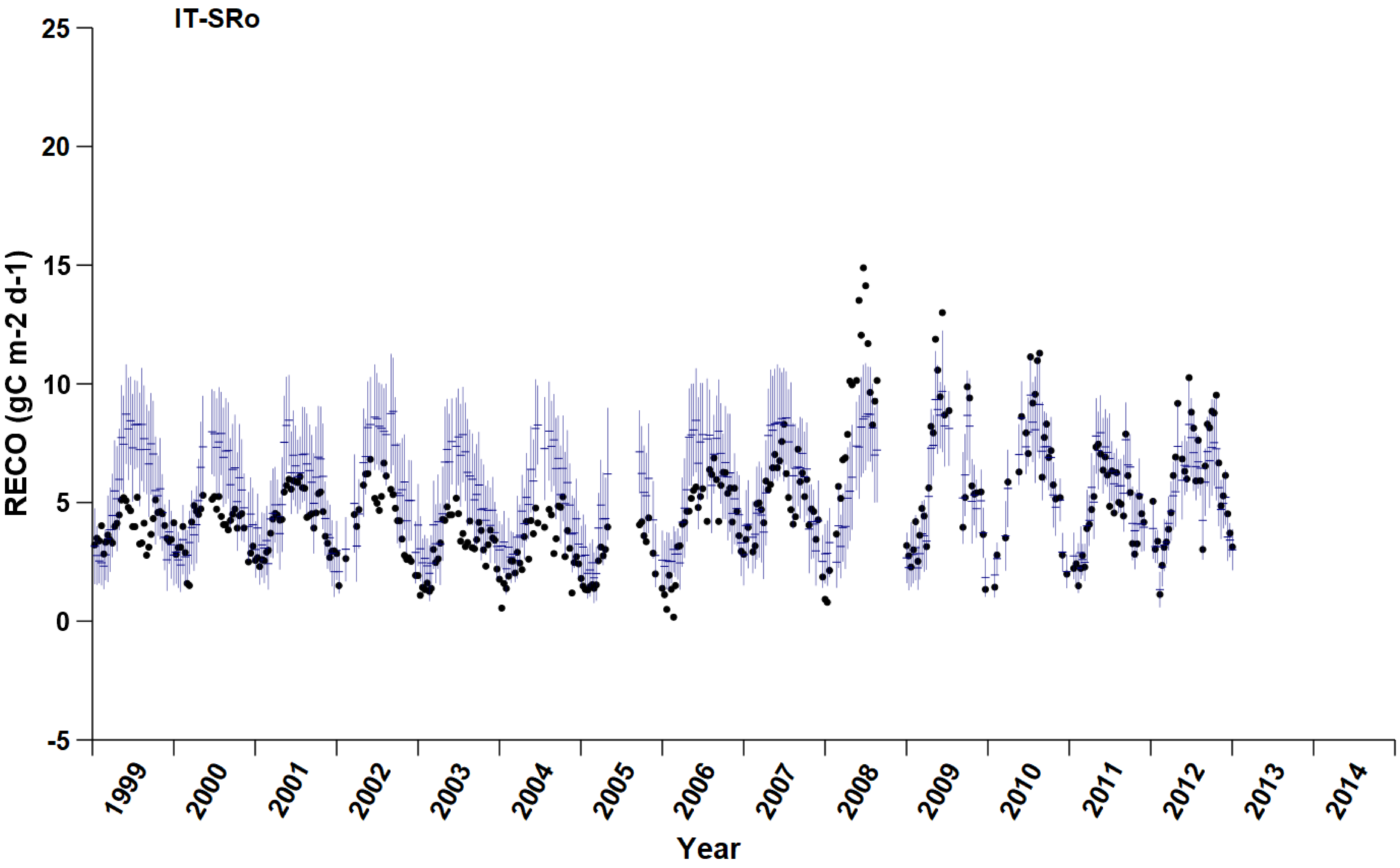

IT-SRo
RECO (gC m-2 d-1)
25
20
15
10
5
0
-5
1999
2000
2001
2002
2003
2004
2005
2006
2007
2008
2009
2010
2011
2012
2013
2014
Year

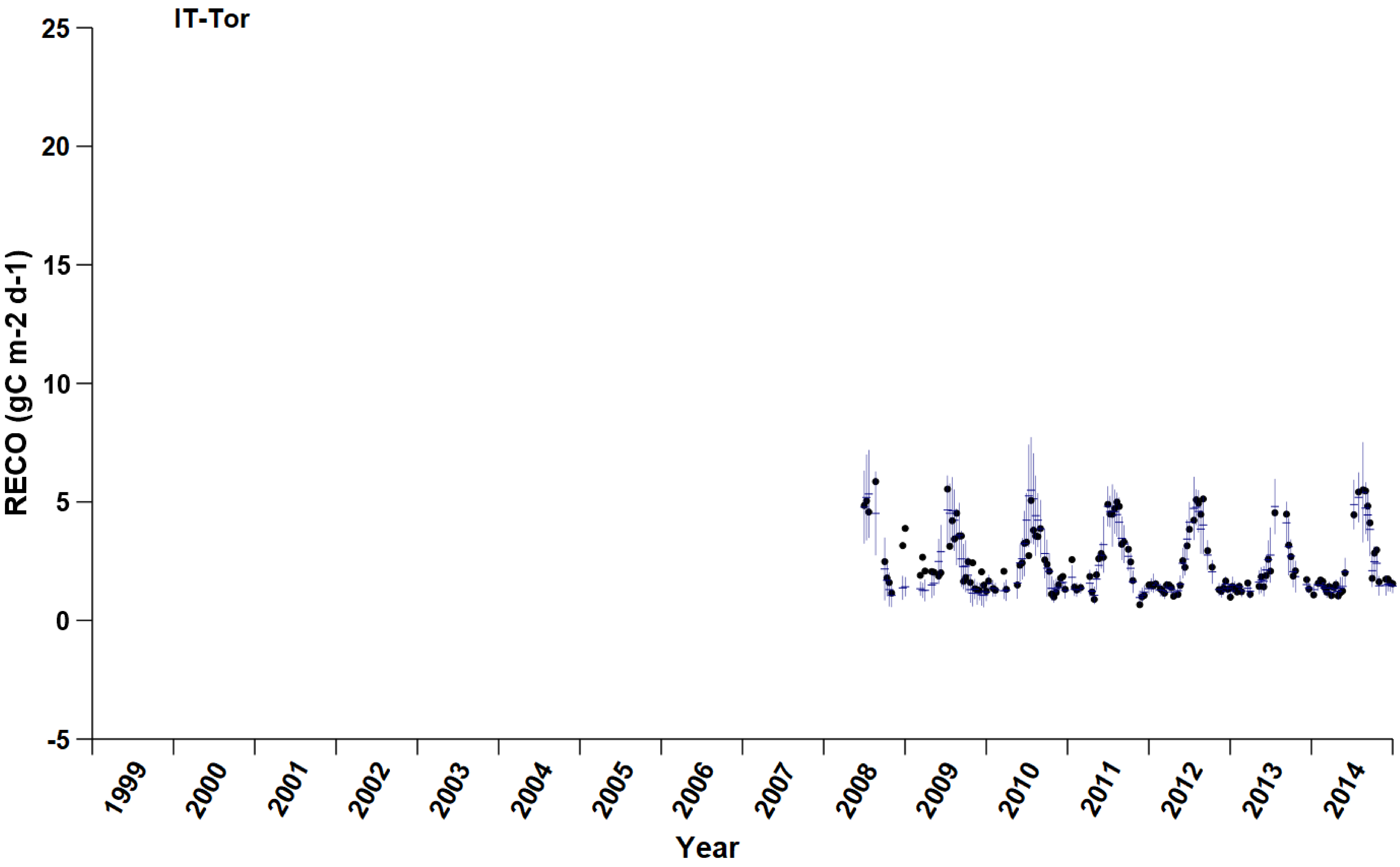
IT-Tor
RECO (gC m-2 d-1)
25
20
15
10
5
0
-5
1999
2000
2001
2002
2003
2004
2005
2006
2007
2008
2009
2010
2011
2012
2013
2014
Year

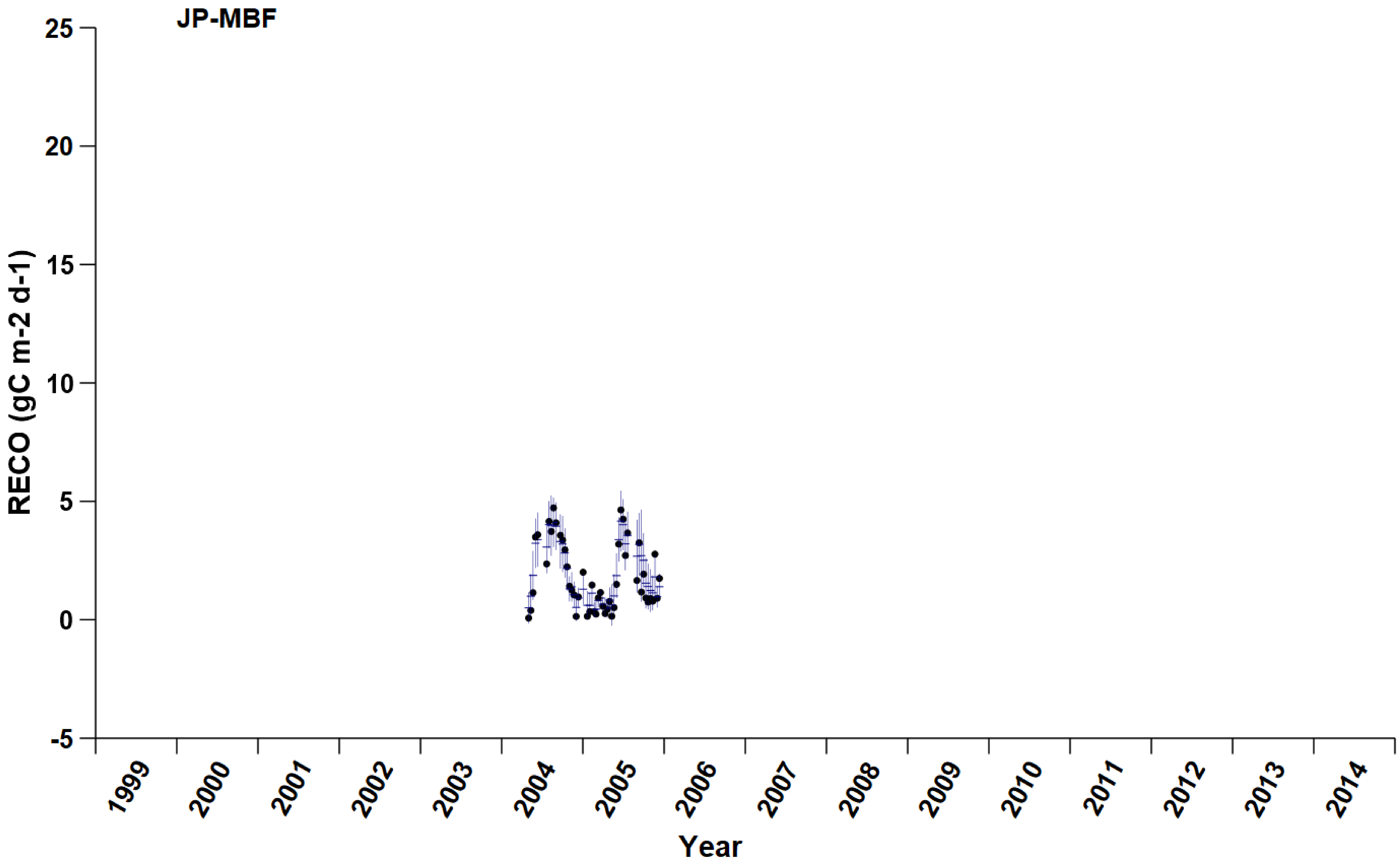

JP-MBF
RECO (gC m-2 d-1)
25
20
15
10
5
0
-5
1999
2000
2001
2002
2003
2004
2005
2006
2007
2008
2009
2010
2011
2012
2013
2014
Year

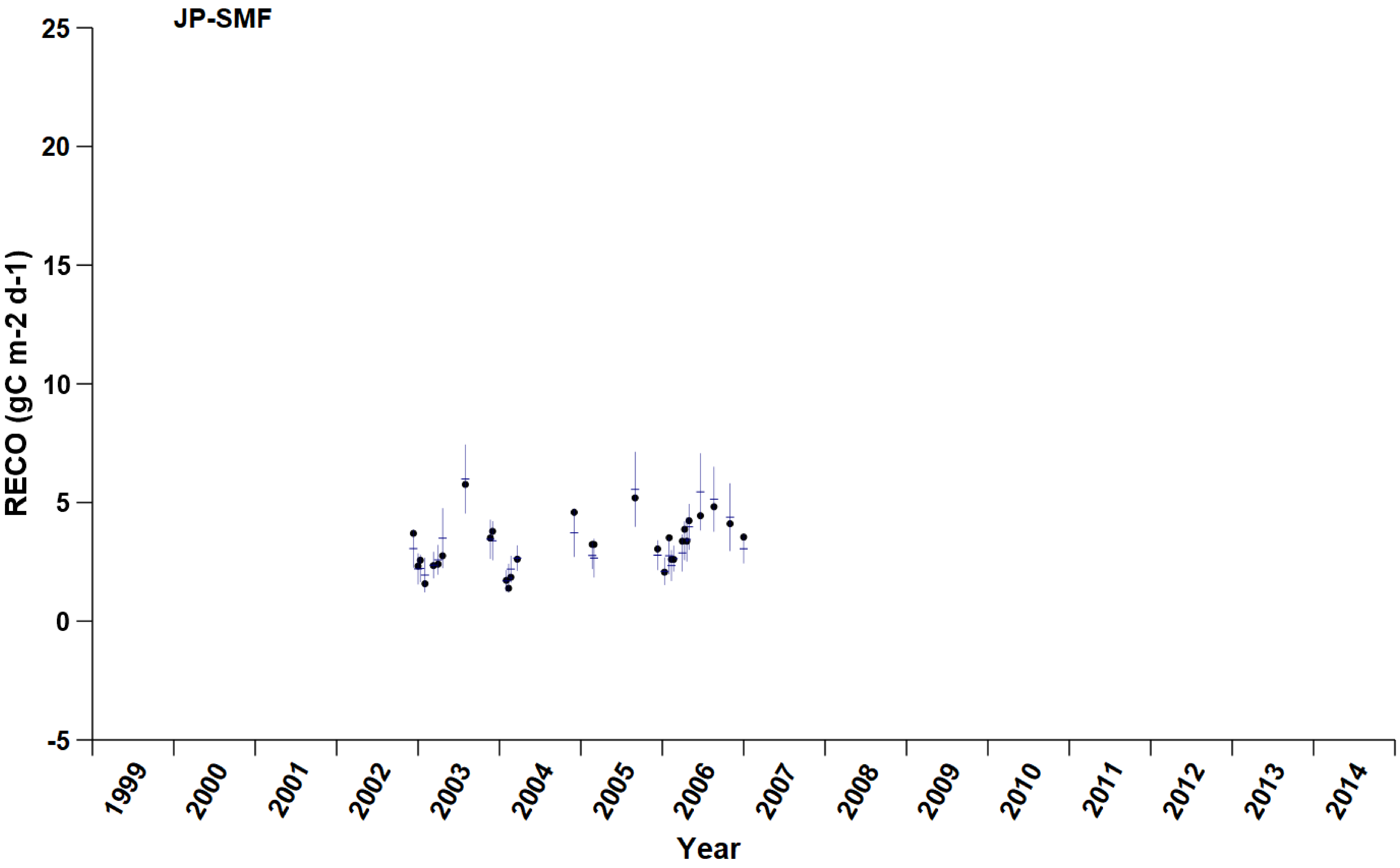

JP-SMF
RECO (gC m-2 d-1)
25
20
15
10
5
0
-5
1999
2000
2001
2002
2003
2004
2005
2006
2007
2008
2009
2010
2011
2012
2013
2014
Year

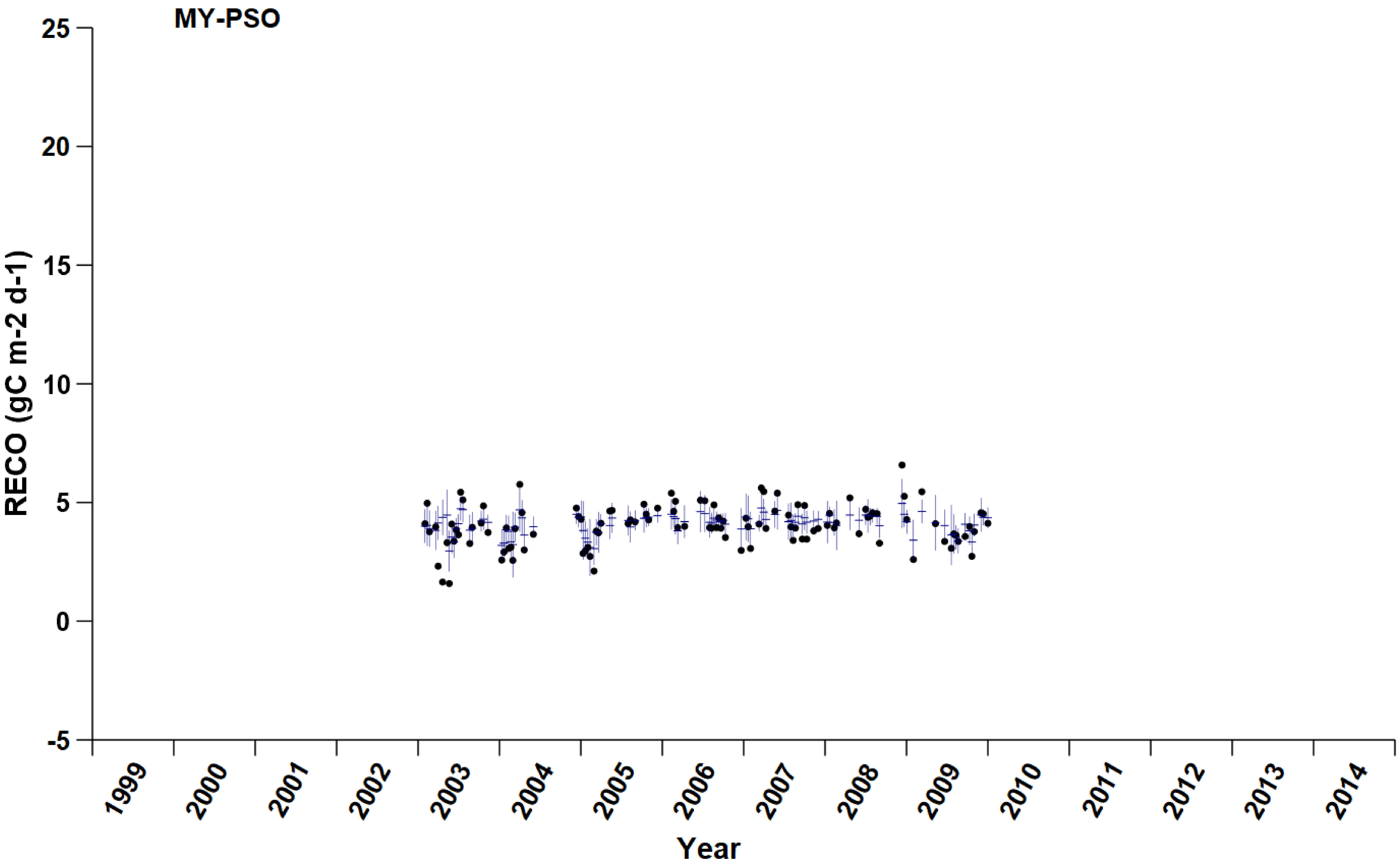

MY-PSO
RECO (gC m-2 d-1)
25
20
15
10
5
0
-5
1999
2000
2001
2002
2003
2004
2005
2006
2007
2008
2009
2010
2011
2012
2013
2014
Year

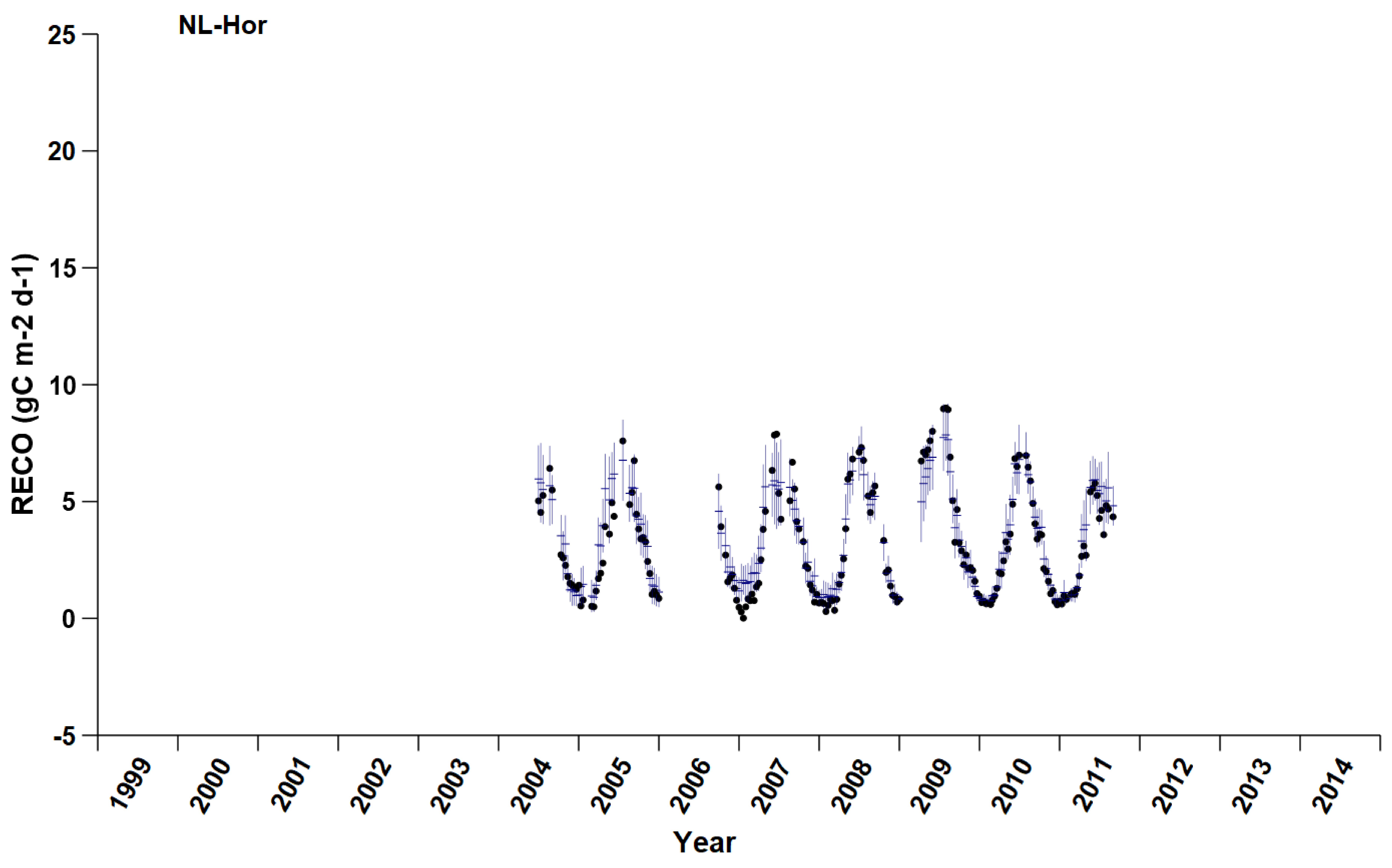
NL-Hor
RECO (gC m-2 d-1)
25
20
15
10
5
0
-5
1999
2000
2001
2002
2003
2004
2005
2006
2007
2008
2009
2010
2011
2012
2013
2014
Year

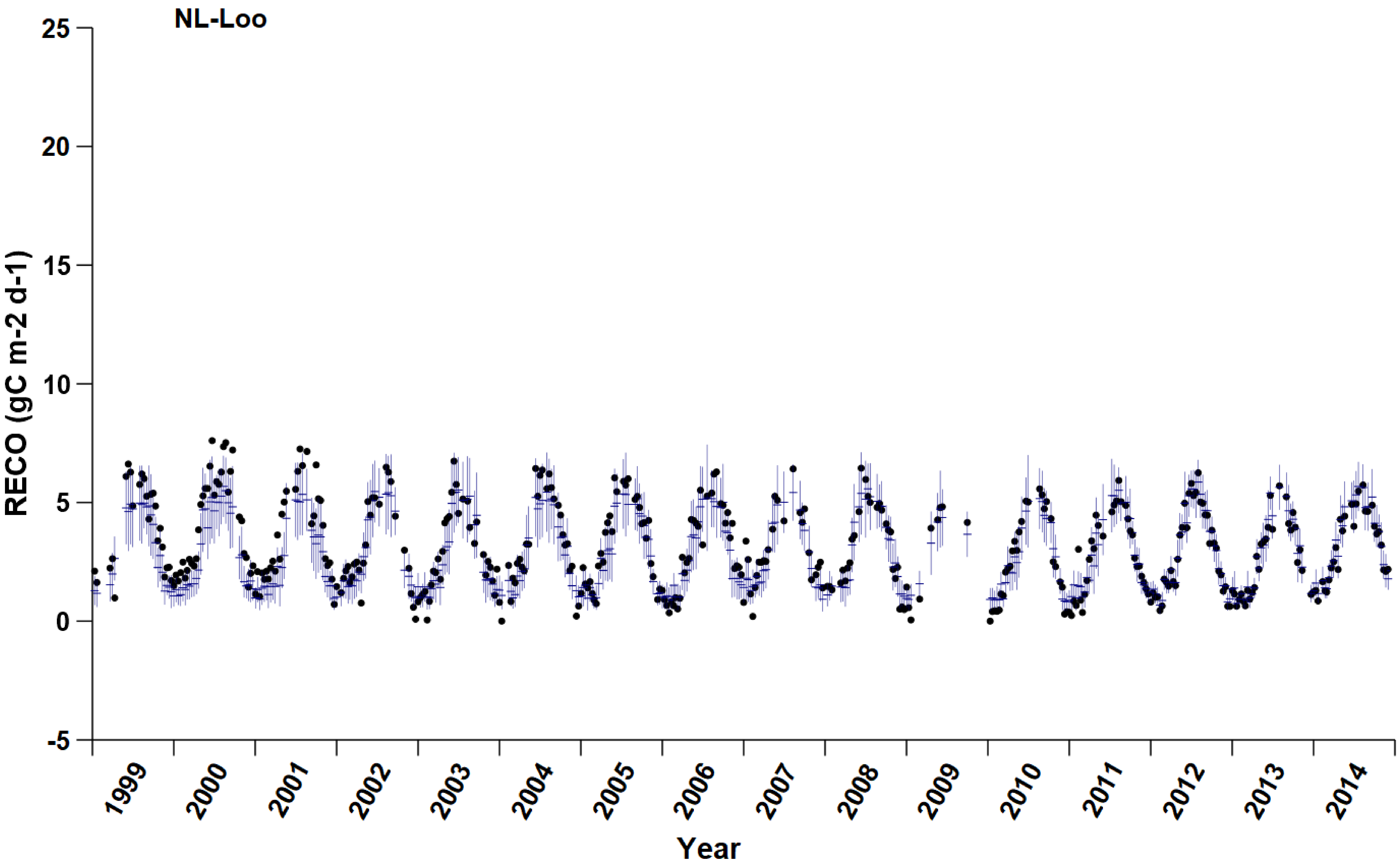

NL-Loo
RECO (gC m-2 d-1)
25
20
15
10
5
0
-5
1999
2000
2001
2002
2003
2004
2005
2006
2007
2008
2009
2010
2011
2012
2013
2014
Year

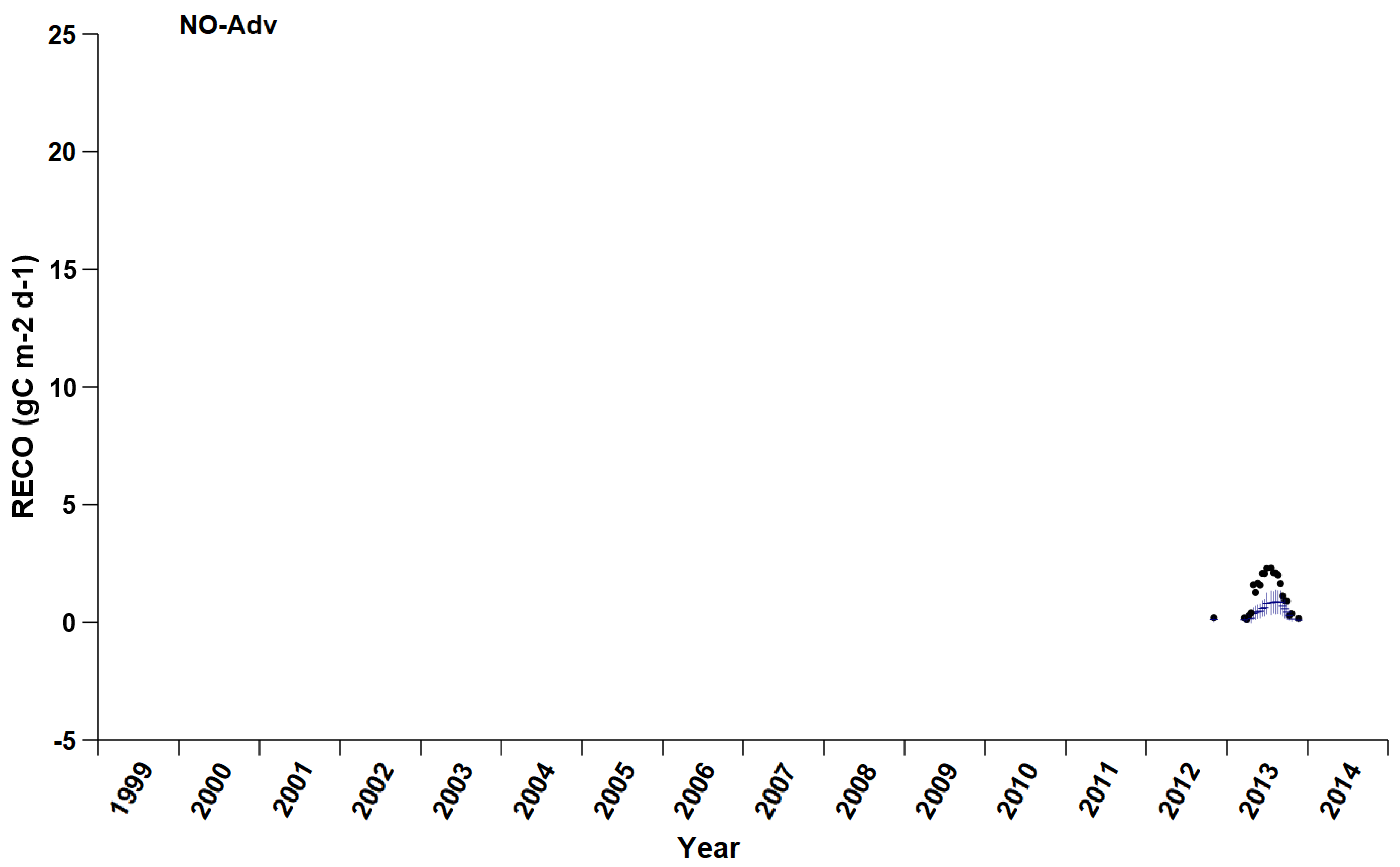
NO-Adv
RECO (gC m-2 d-1)
25
20
15
10
5
0
-5
1999
2000
2001
2002
2003
2004
2005
2006
2007
2008
2009
2010
2011
2012
2013
2014
Year

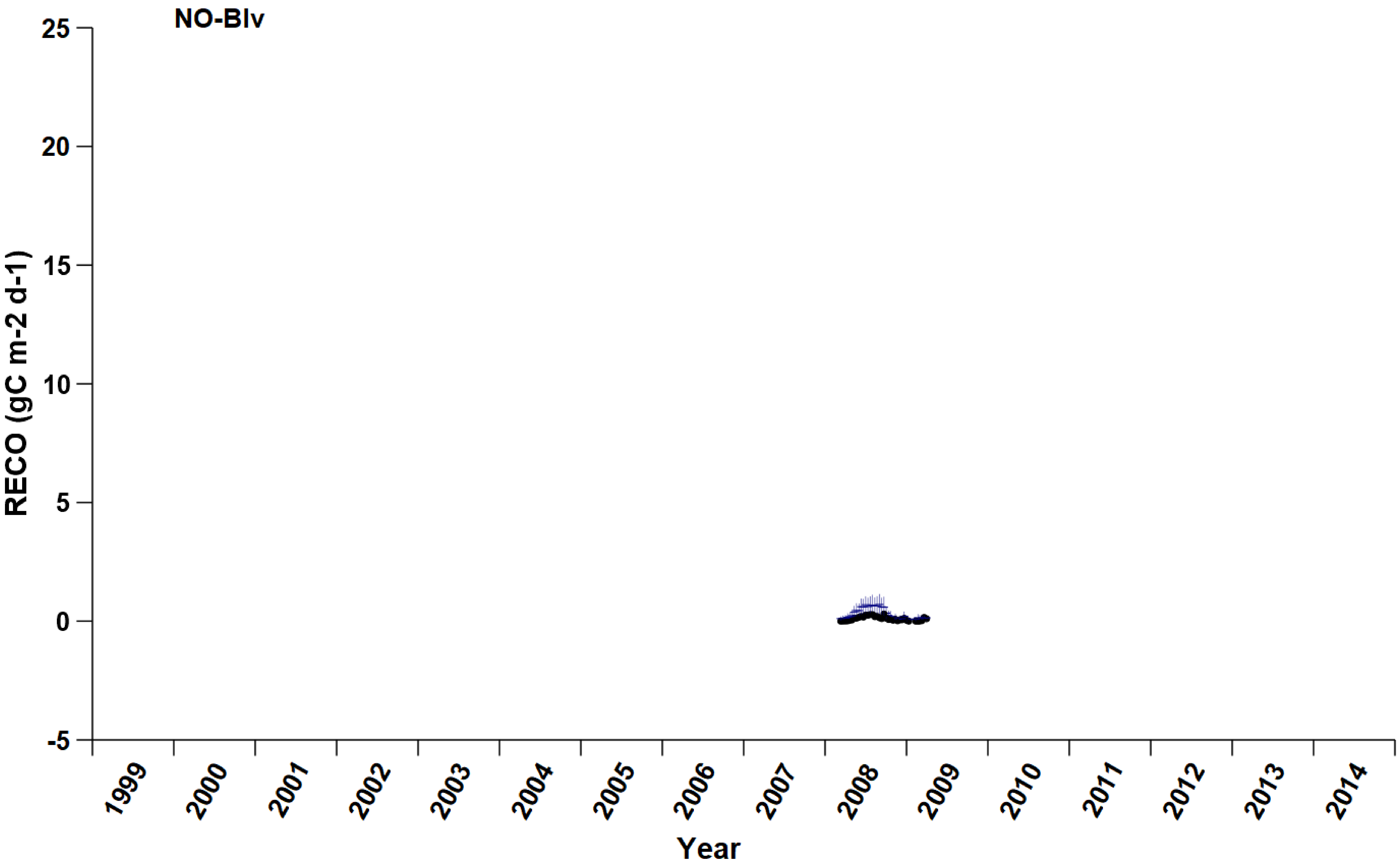

NO-Blv
RECO (gC m-2 d-1)
25
20
15
10
5
0
-5
1999
2000
2001
2002
2003
2004
2005
2006
2007
2008
2009
2010
2011
2012
2013
2014
Year

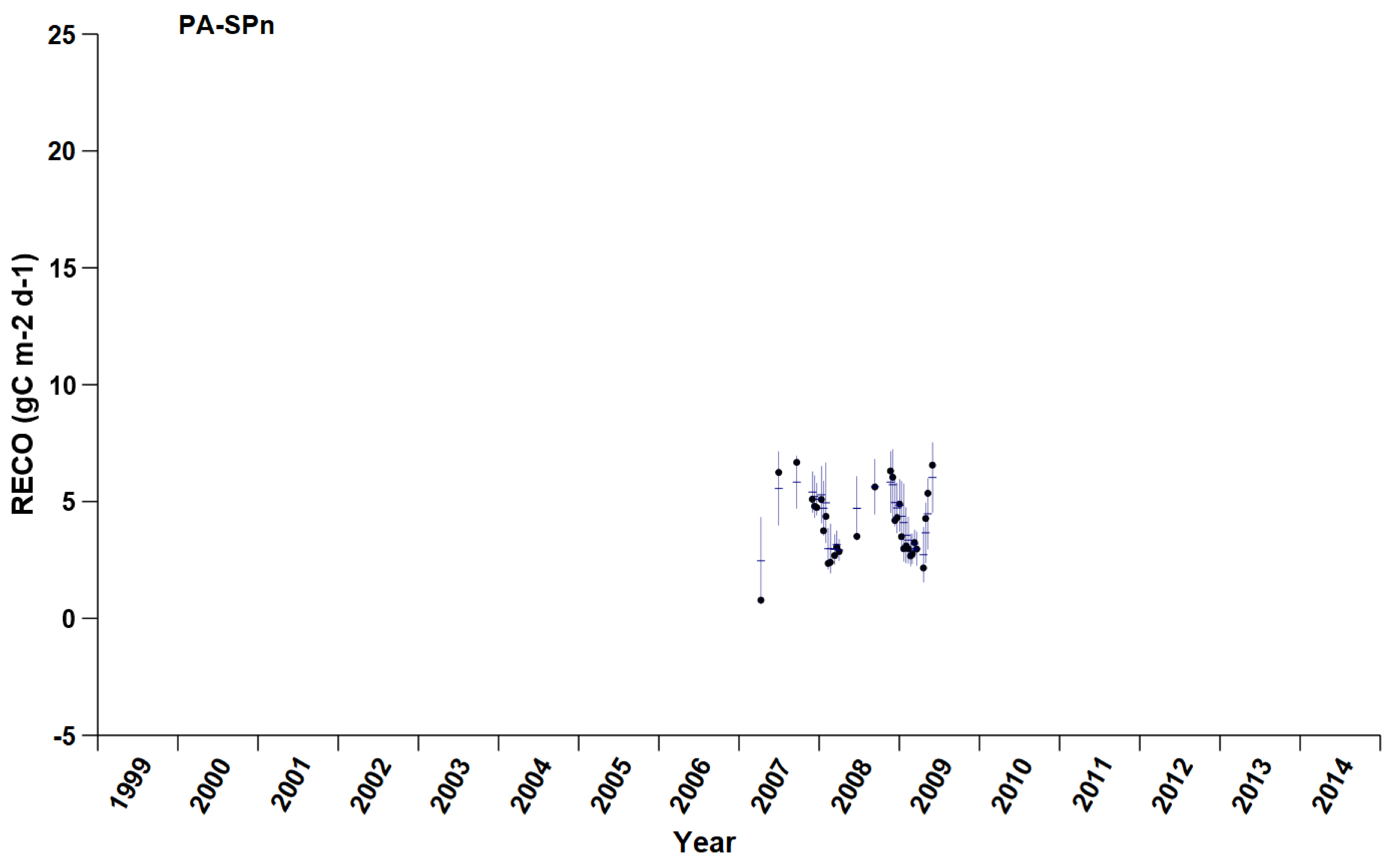
PA-SPn
RECO (gC m-2 d-1)
25
20
15
10
5
0
-5
1999
2000
2001
2002
2003
2004
2005
2006
2007
2008
2009
2010
2011
2012
2013
2014
Year

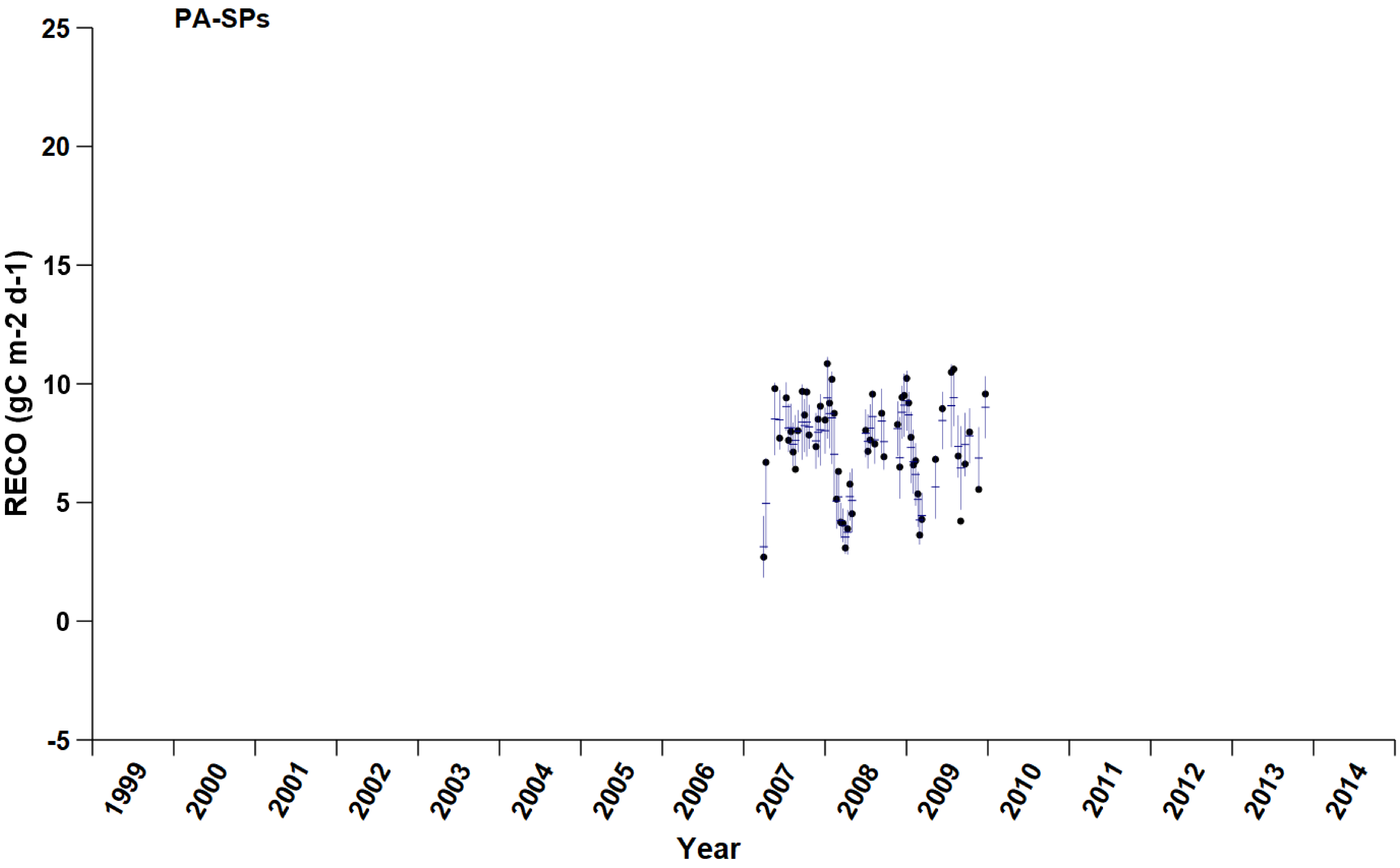
PA-SPs
25
20
15
10
5
0
-5
RECO (gC m-2 d-1)
1999
2000
2001
2002
2003
2004
2005
2006
2007
2008
2009
2010
2011
2012
2013
2014
Year

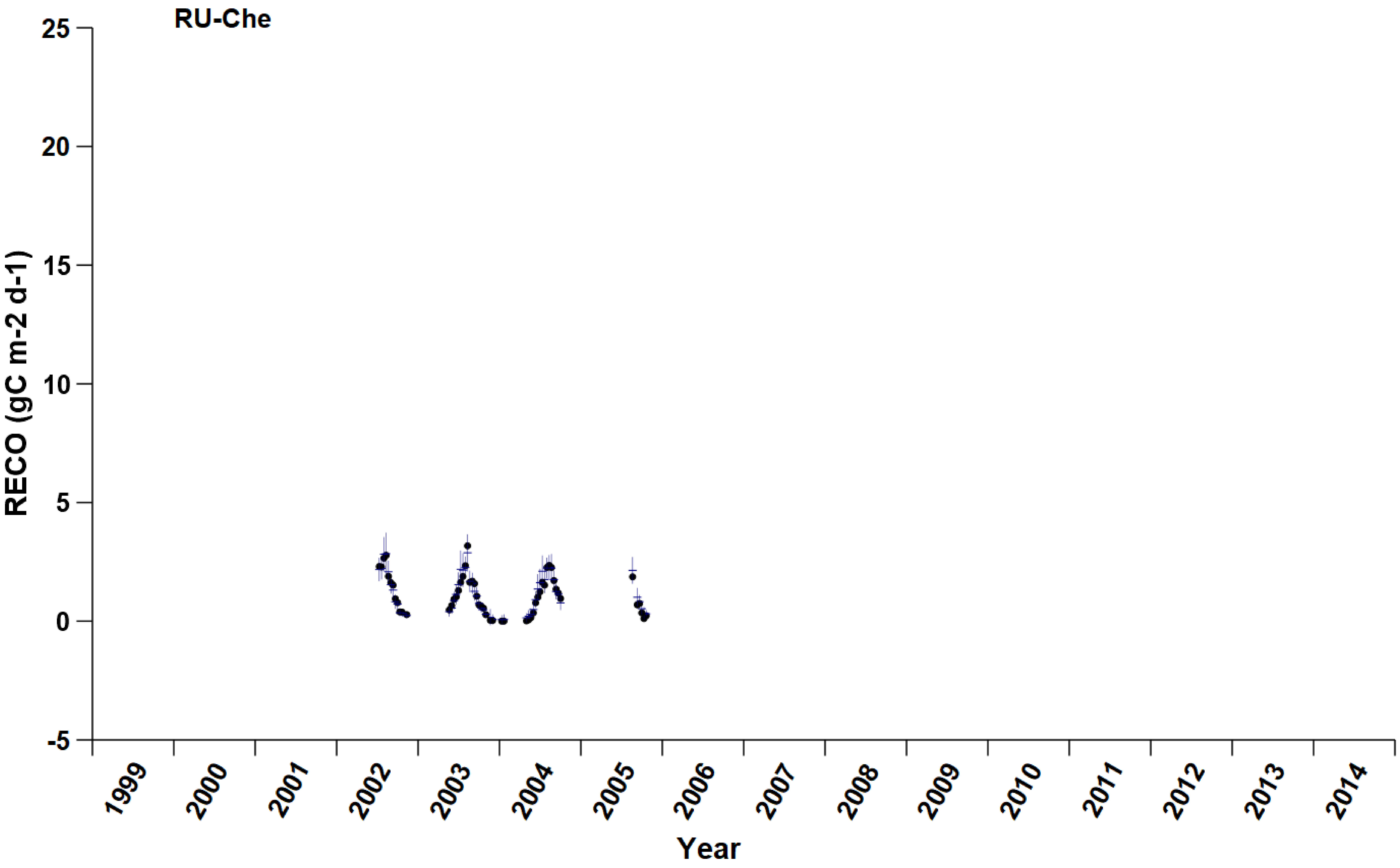
RU-Che
RECO (gC m-2 d-1)
25
20
15
10
5
0
-5
1999
2000
2001
2002
2003
2004
2005
2006
2007
2008
2009
2010
2011
2012
2013
2014
Year

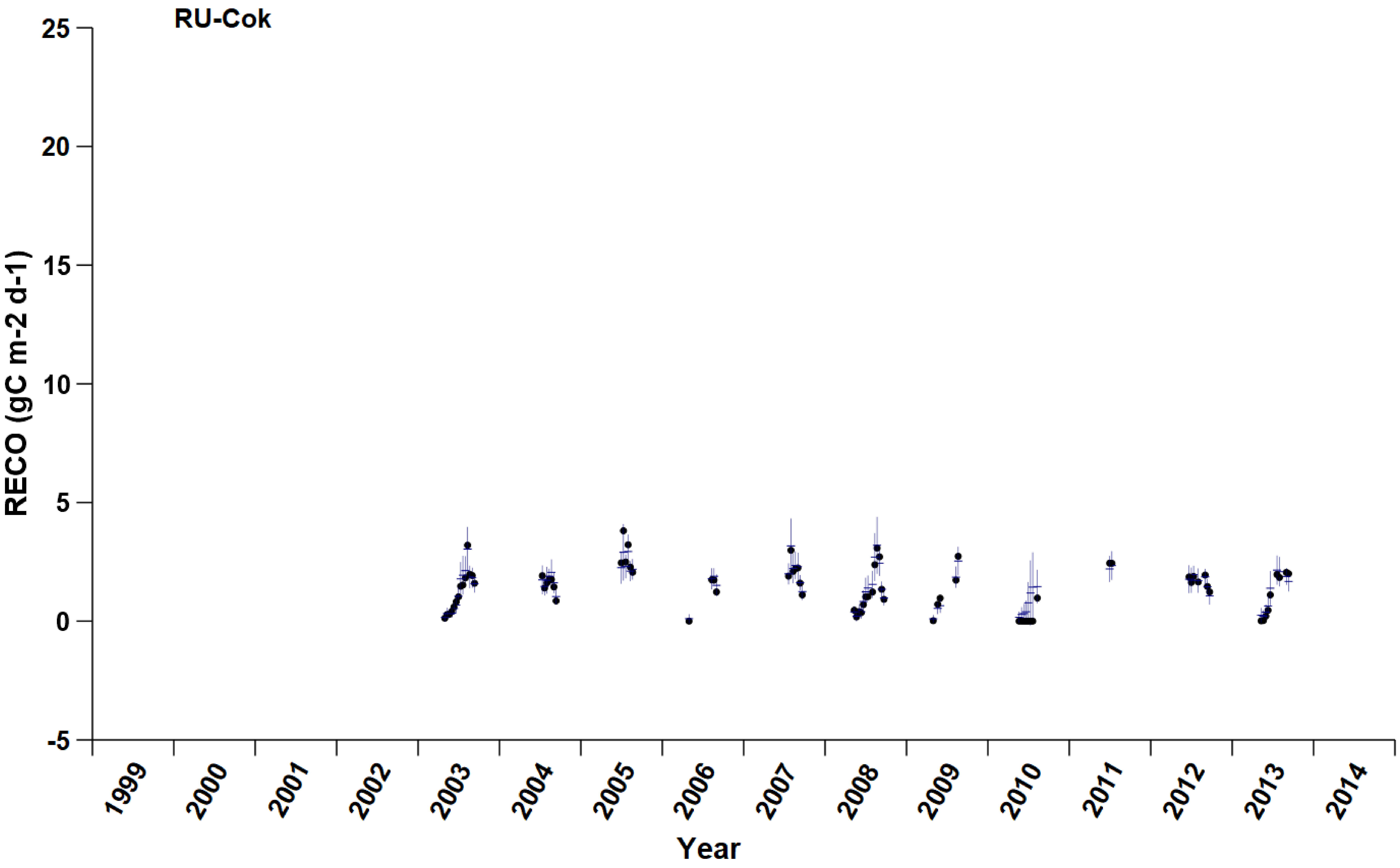
RU-Cok
RECO (gC m-2 d-1)
25
20
15
10
5
0
-5
1999
2000
2001
2002
2003
2004
2005
2006
2007
2008
2009
2010
2011
2012
2013
2014
Year

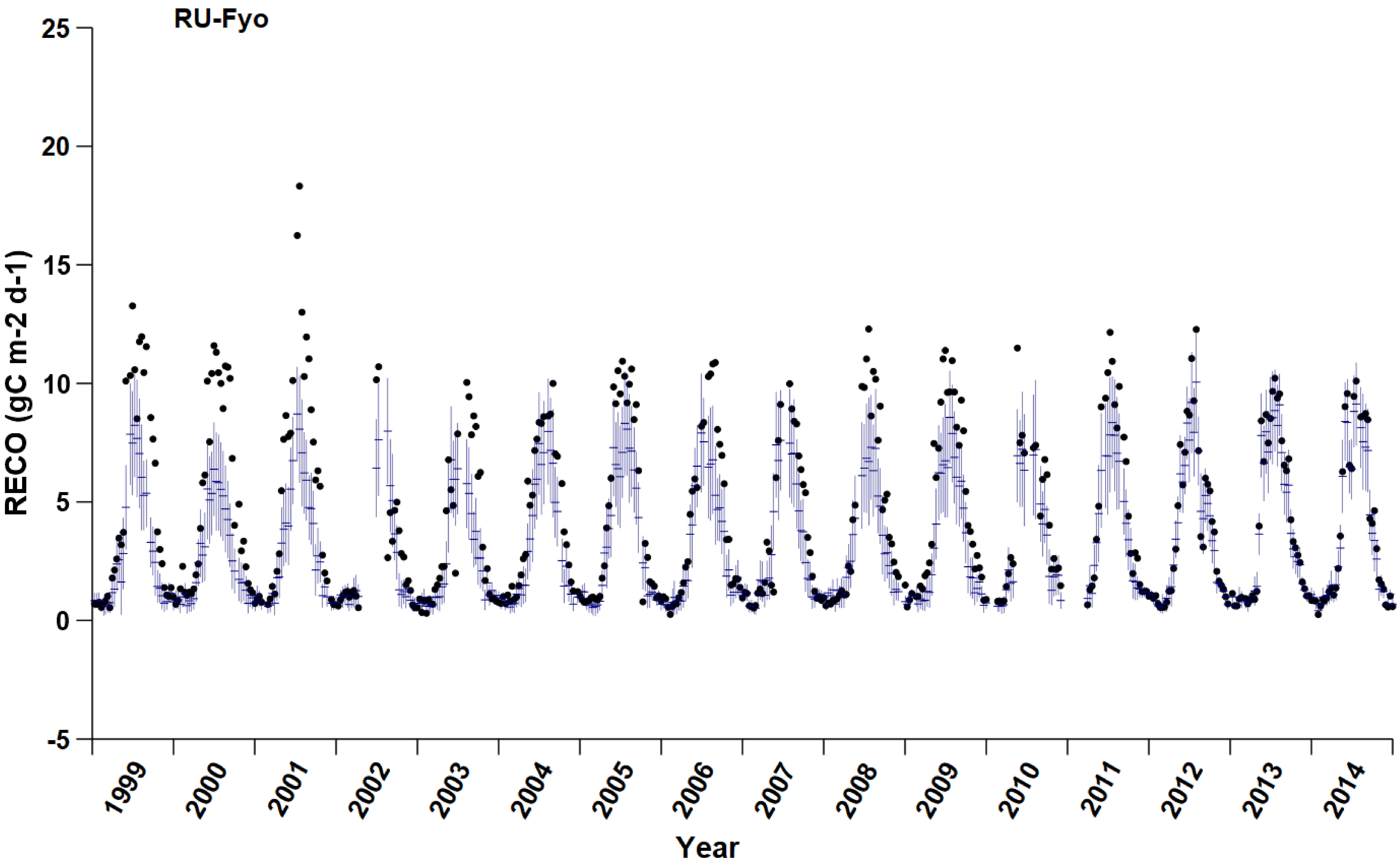

RU-Fyo
RECO (gC m-2 d-1)
25
20
15
10
5
0
-5
1999
2000
2001
2002
2003
2004
2005
2006
2007
2008
2009
2010
2011
2012
2013
2014
Year

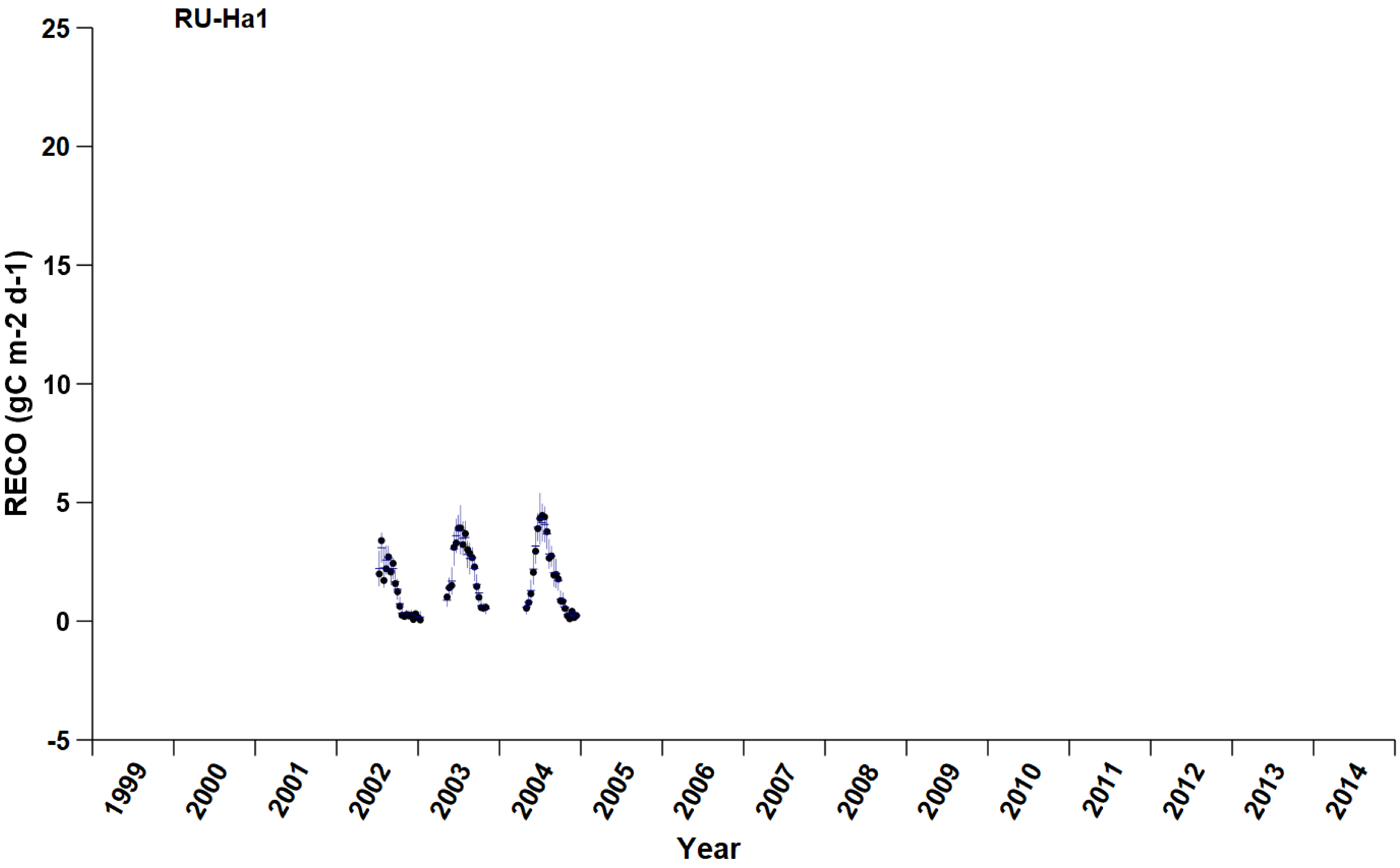
RU-Ha1
RECO (gC m-2 d-1)
25
20
15
10
5
0
-5
1999
2000
2001
2002
2003
2004
2005
2006
2007
2008
2009
2010
2011
2012
2013
2014
Year

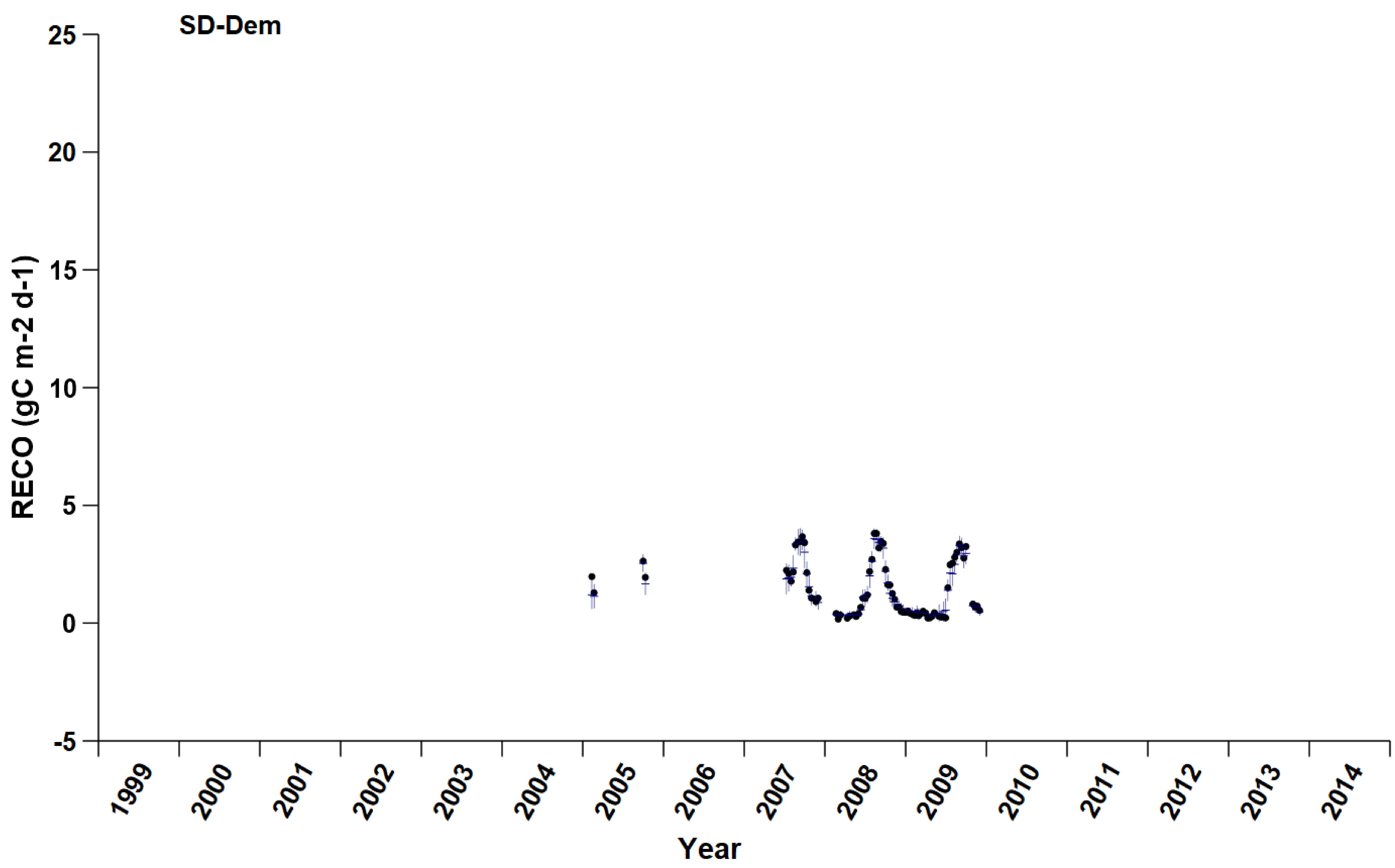
SD-Dem
RECO (gC m-2 d-1)
25
20
15
10
5
0
-5
1999
2000
2001
2002
2003
2004
2005
2006
2007
2008
2009
2010
2011
2012
2013
2014
Year

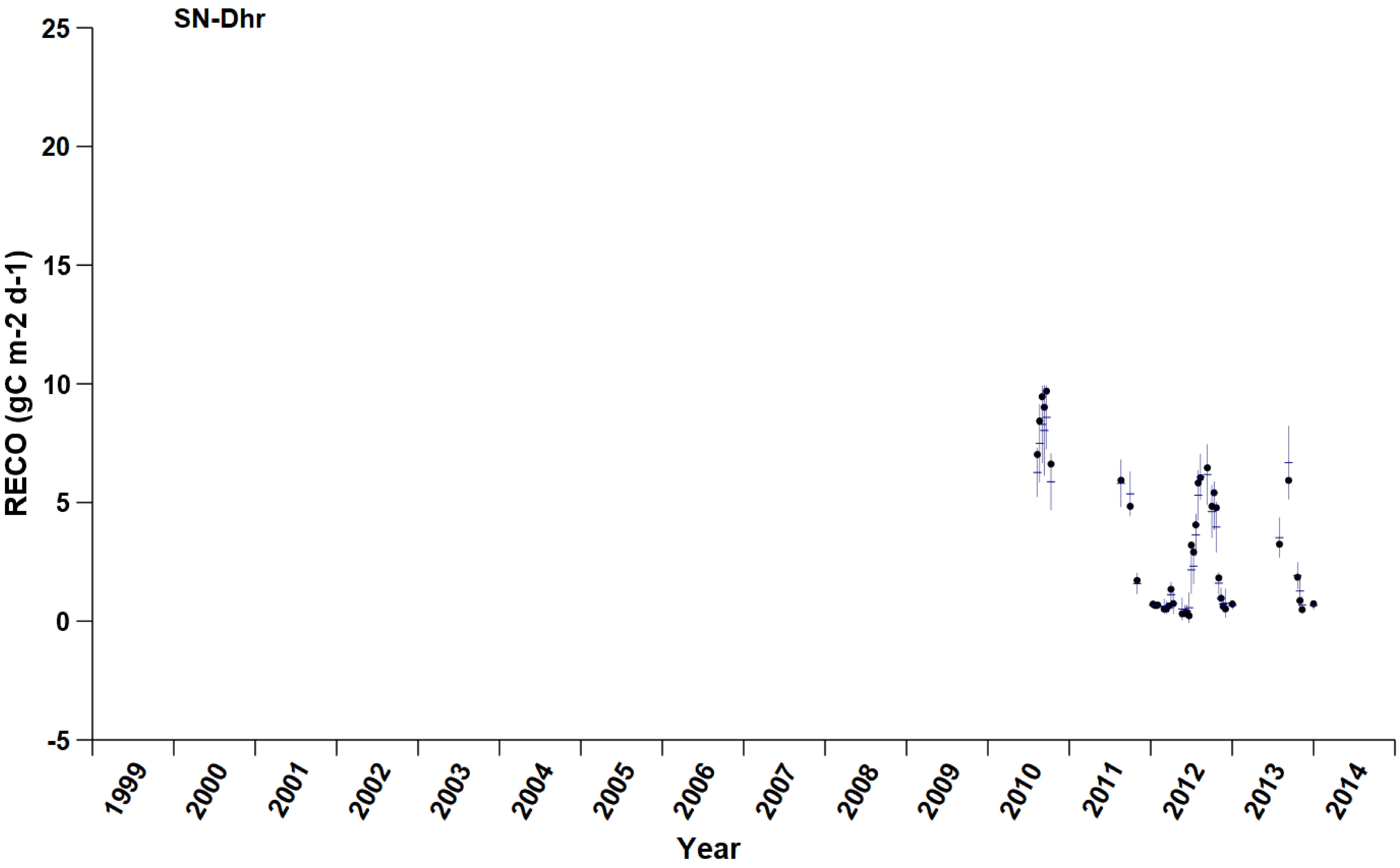

SN-Dhr
RECO (gC m-2 d-1)
25
20
15
10
5
0
-5
1999
2000
2001
2002
2003
2004
2005
2006
2007
2008
2009
2010
2011
2012
2013
2014
Year

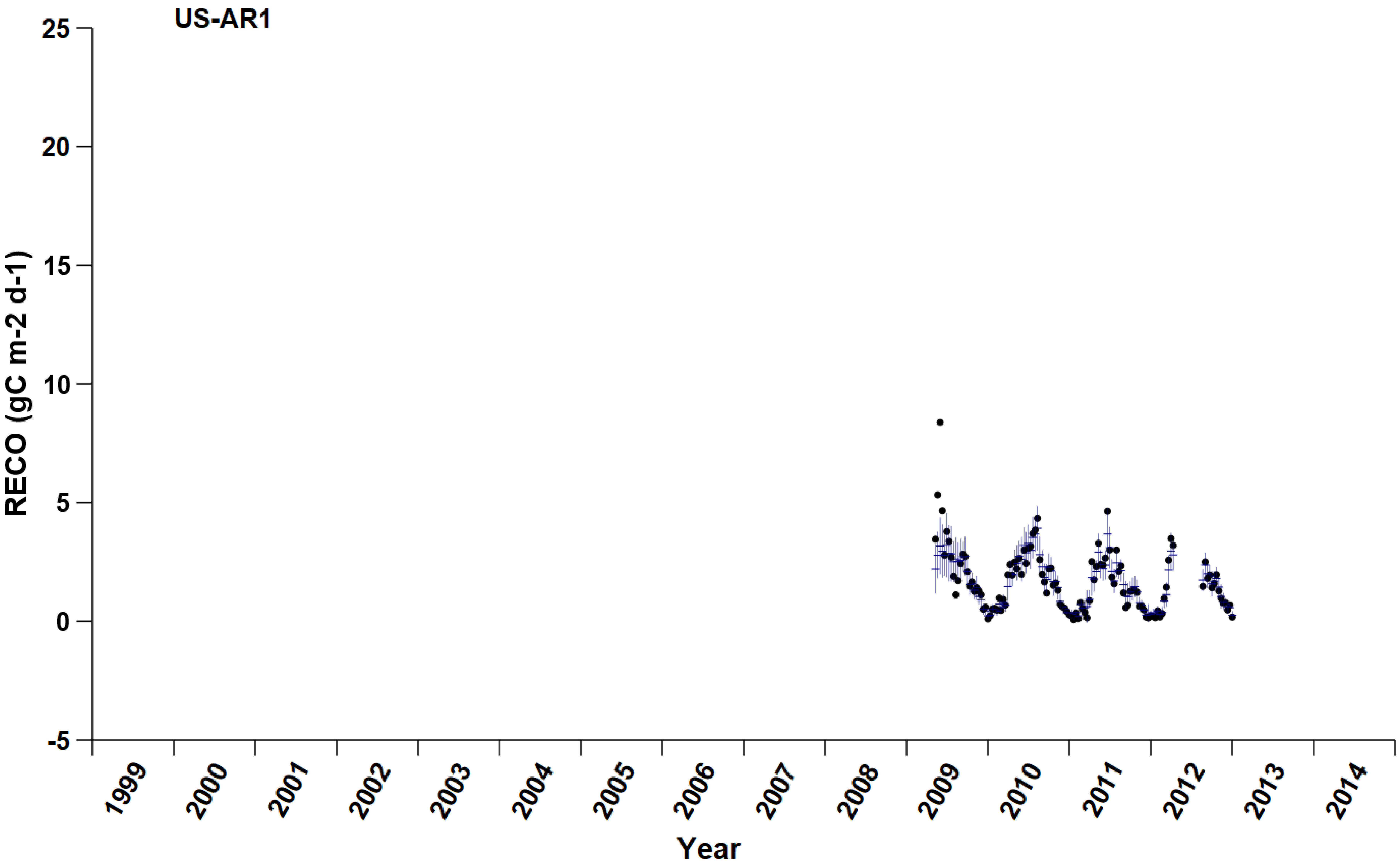
US-AR1
25
20
15
10
5
0
-5
RECO (gC m-2 d-1)
1999
2000
2001
2002
2003
2004
2005
2006
2007
2008
2009
2010
2011
2012
2013
2014
Year

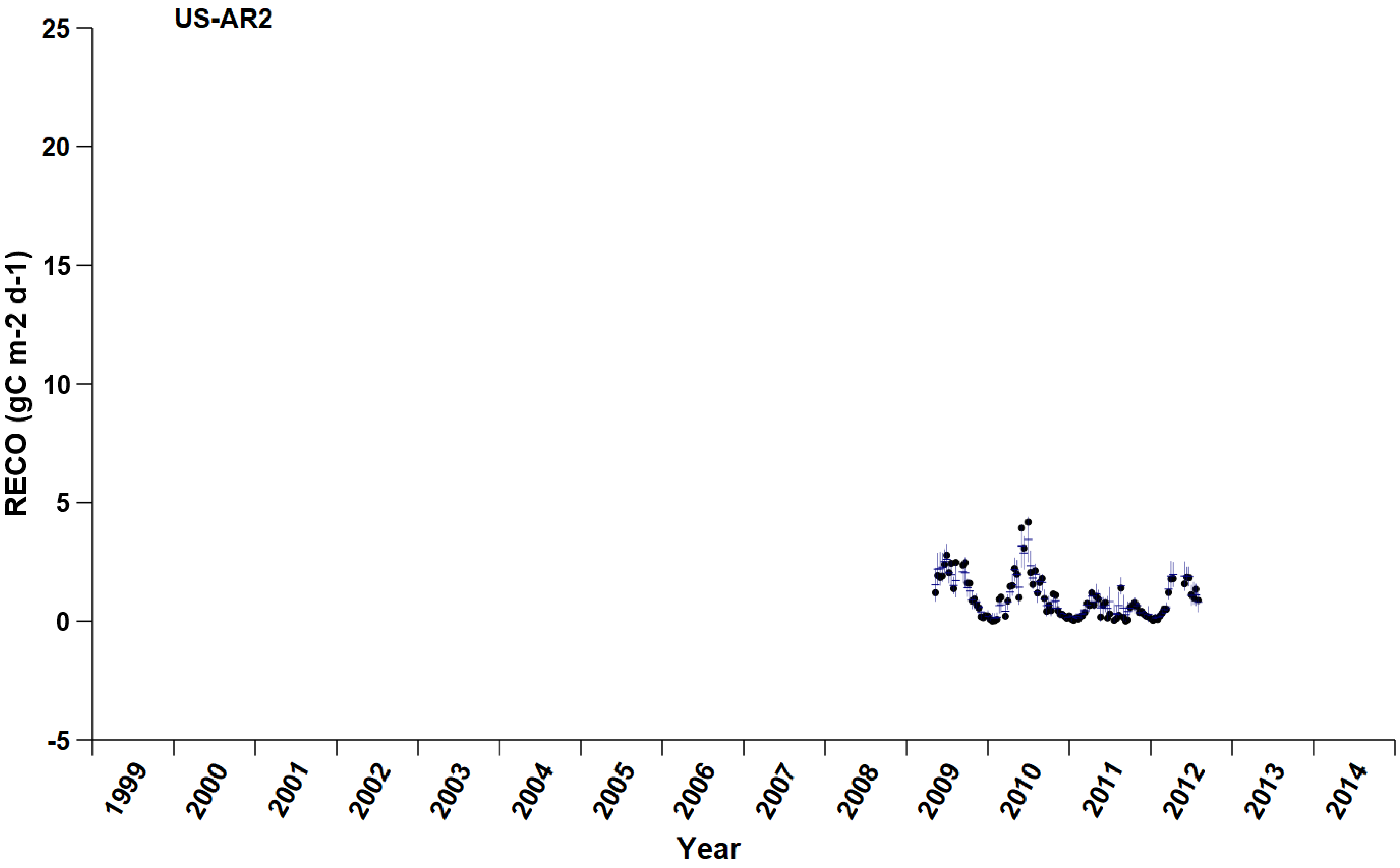

US-AR2
RECO (gC m-2 d-1)
25
20
15
10
5
0
-5
1999
2000
2001
2002
2003
2004
2005
2006
2007
2008
2009
2010
2011
2012
2013
2014
Year

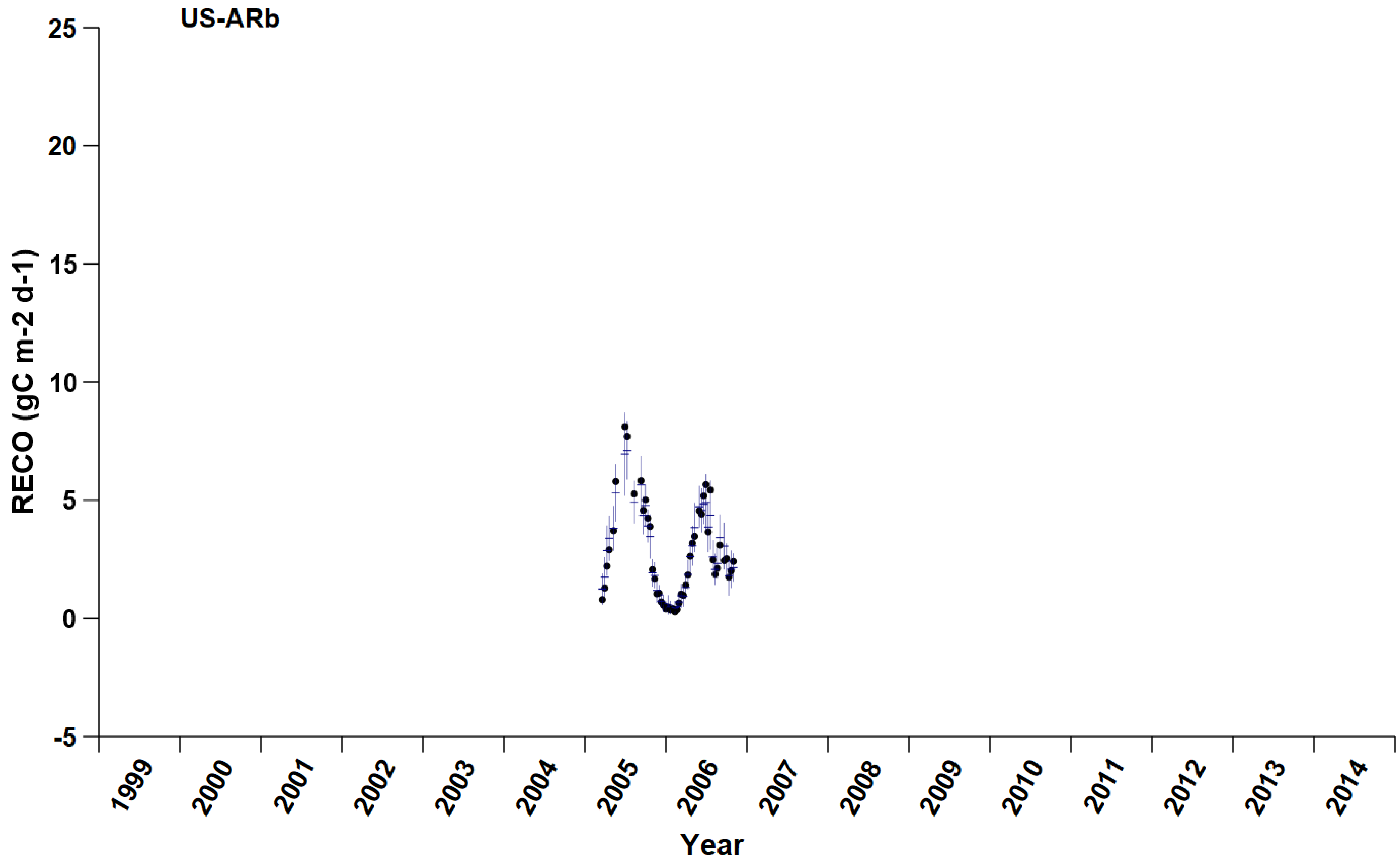
US-ARb
RECO (gC m-2 d-1)
25
20
15
10
5
0
-5
1999
2000
2001
2002
2003
2004
2005
2006
2007
2008
2009
2010
2011
2012
2013
2014
Year

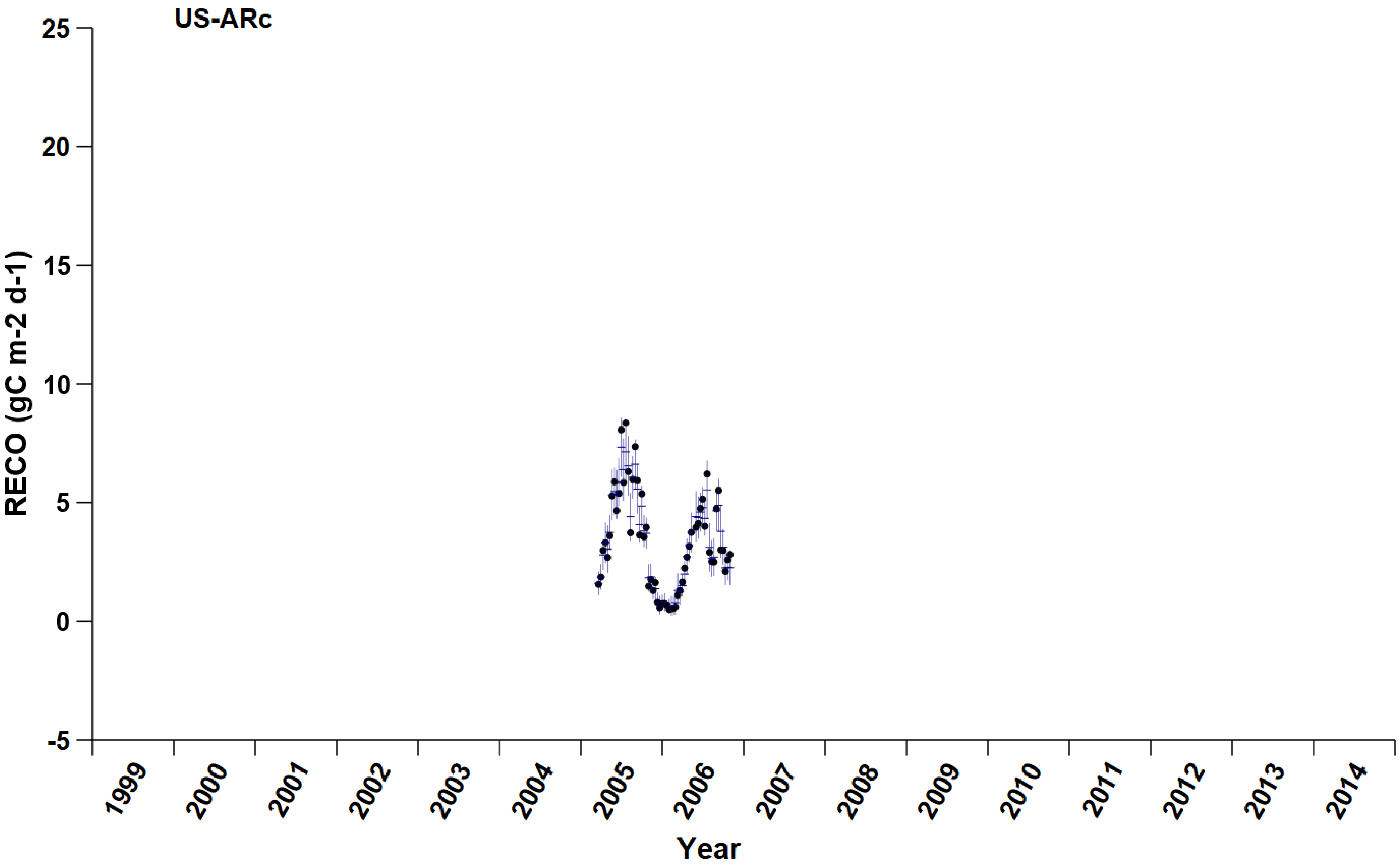
US-ARc
25
20
15
10
5
0
-5
RECO (gC m-2 d-1)
1999
2000
2001
2002
2003
2004
2005
2006
2007
2008
2009
2010
2011
2012
2013
2014
Year

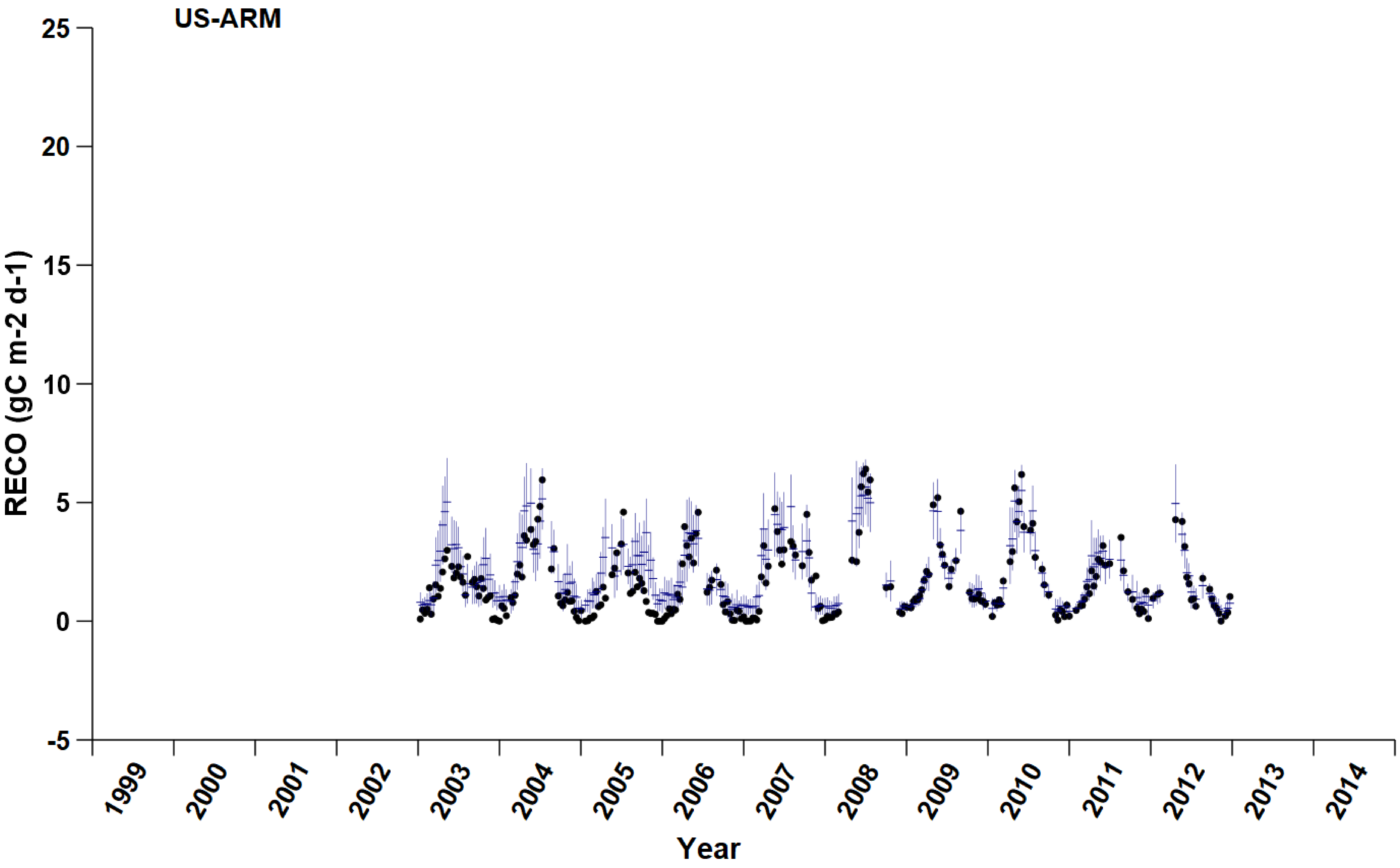
US-ARM
RECO (gC m-2 d-1)
25
20
15
10
5
0
-5
1999
2000
2001
2002
2003
2004
2005
2006
2007
2008
2009
2010
2011
2012
2013
2014
Year

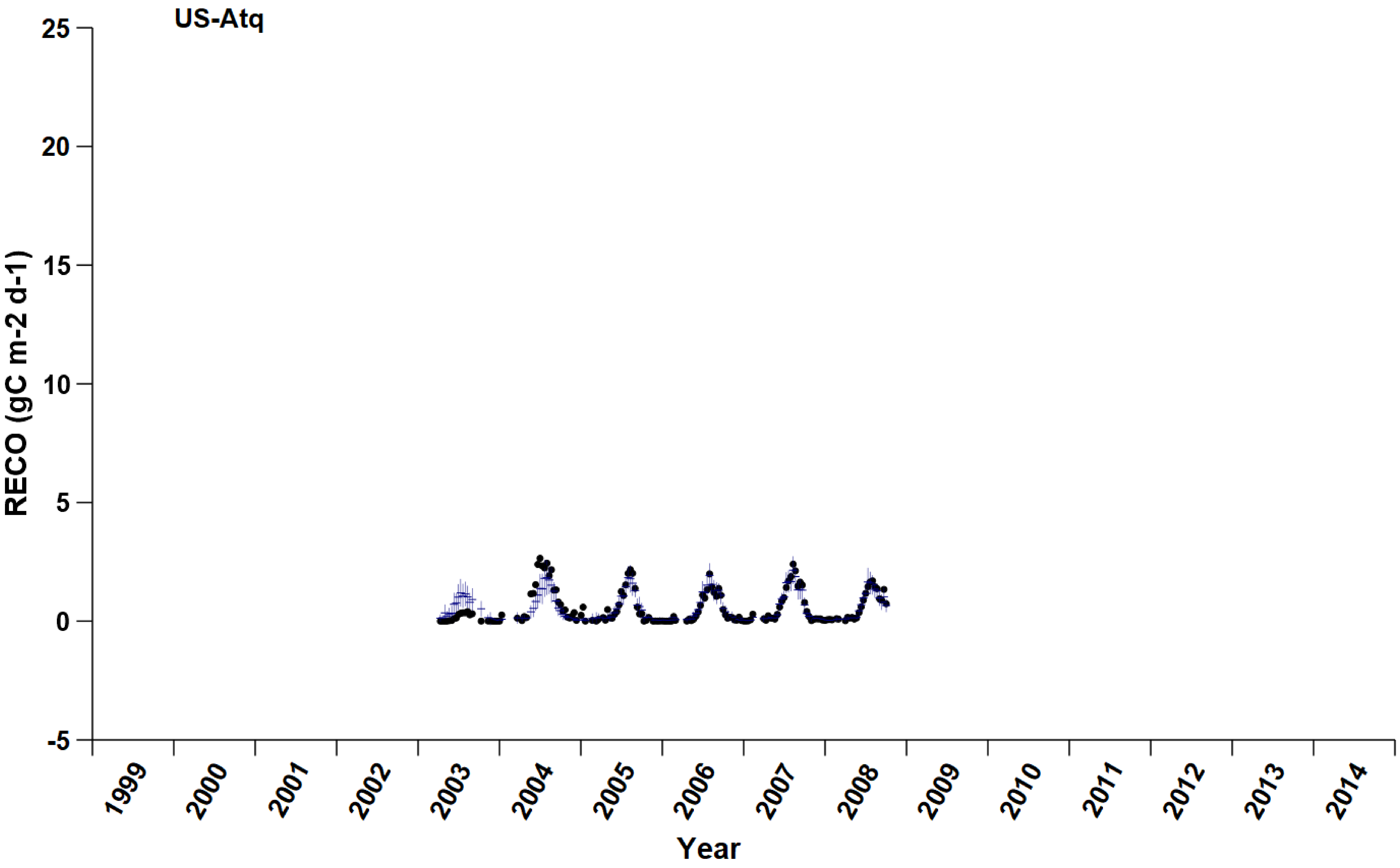

US-Atq
RECO (gC m-2 d-1)
25
20
15
10
5
0
-5
1999
2000
2001
2002
2003
2004
2005
2006
2007
2008
2009
2010
2011
2012
2013
2014
Year

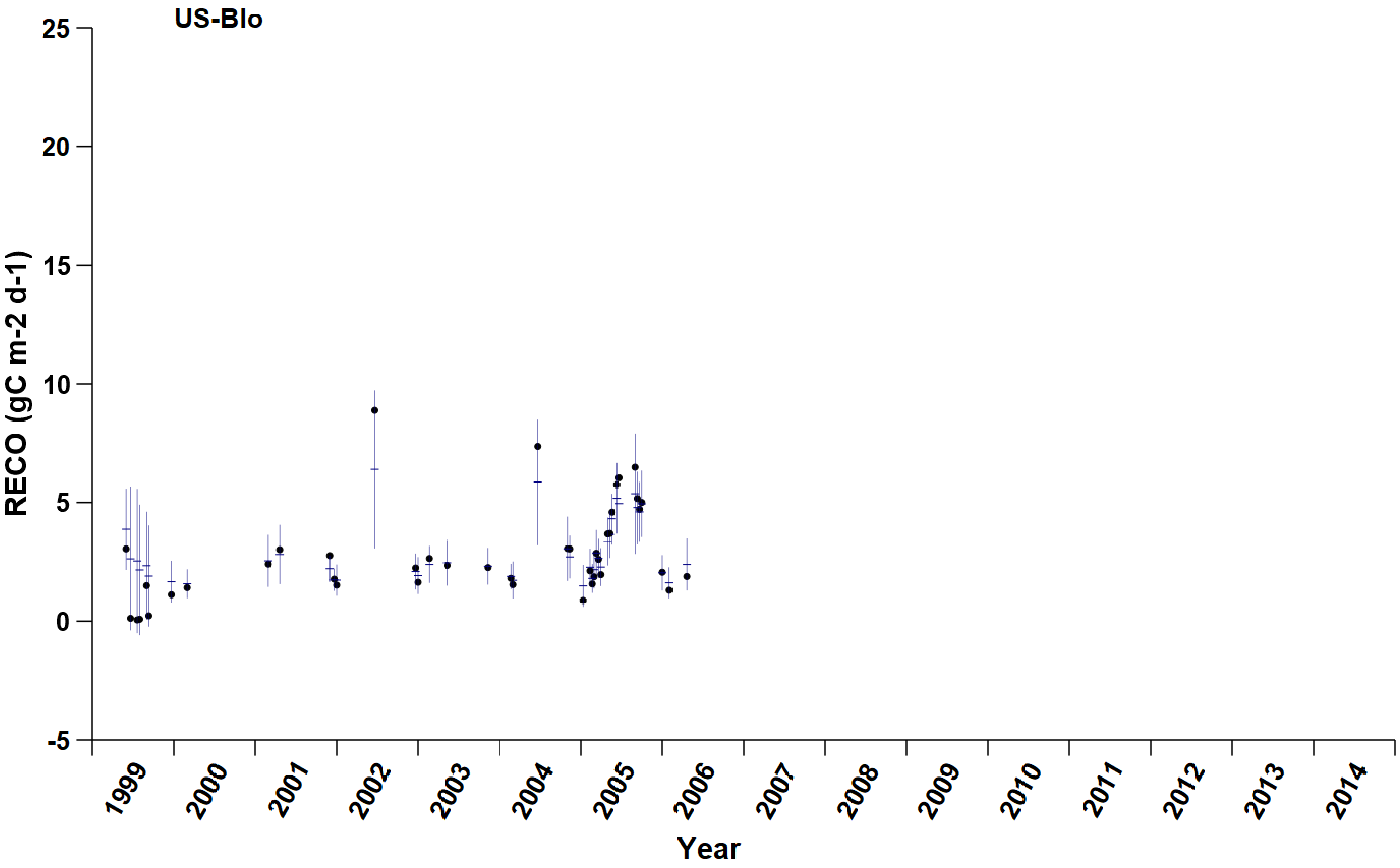

US-Blo
RECO (gC m-2 d-1)
25
20
15
10
5
0
-5
1999
2000
2001
2002
2003
2004
2005
2006
2007
2008
2009
2010
2011
2012
2013
2014
Year

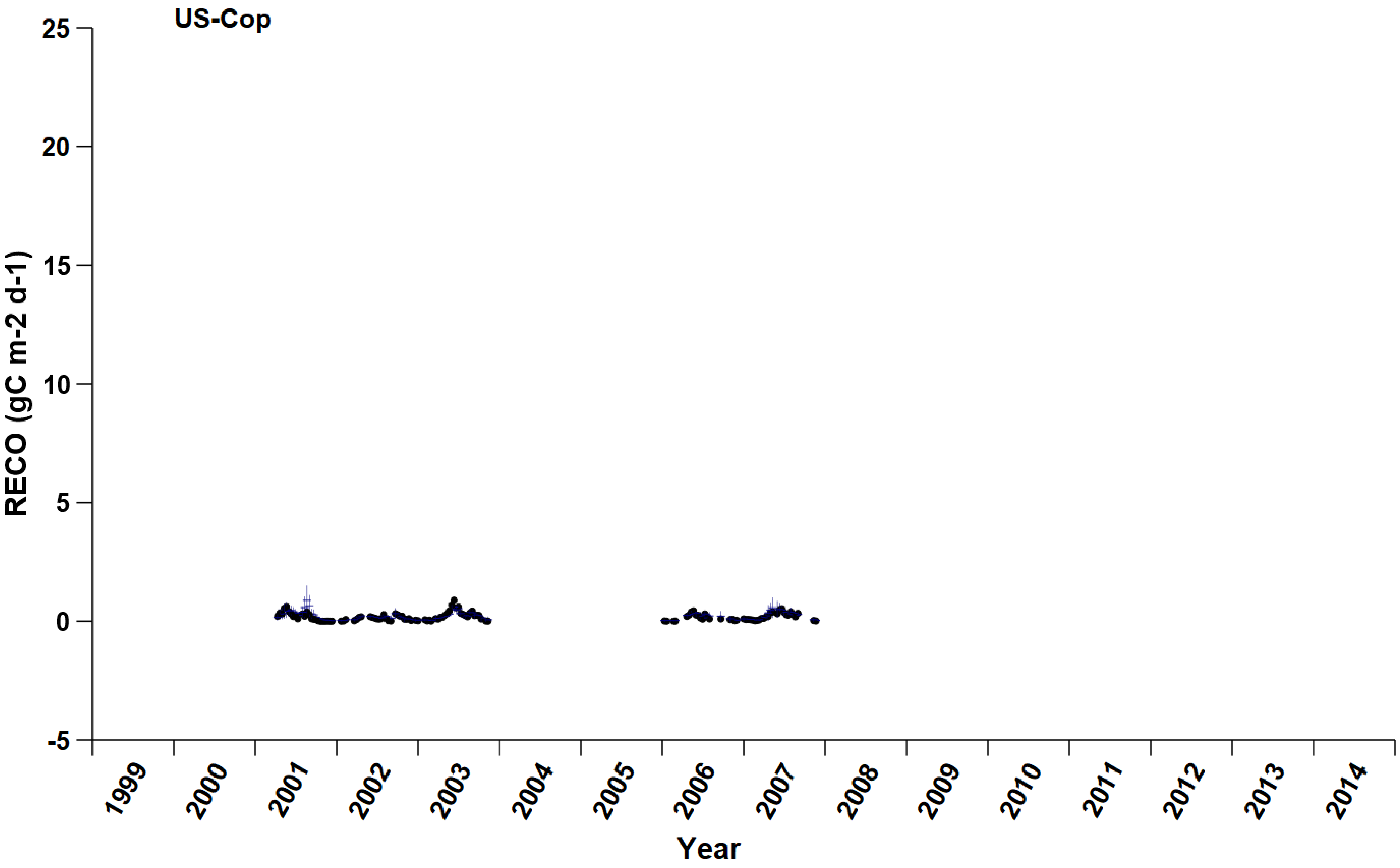
US-Cop
RECO (gC m-2 d-1)
25
20
15
10
5
0
-5
1999
2000
2001
2002
2003
2004
2005
2006
2007
2008
2009
2010
2011
2012
2013
2014
Year

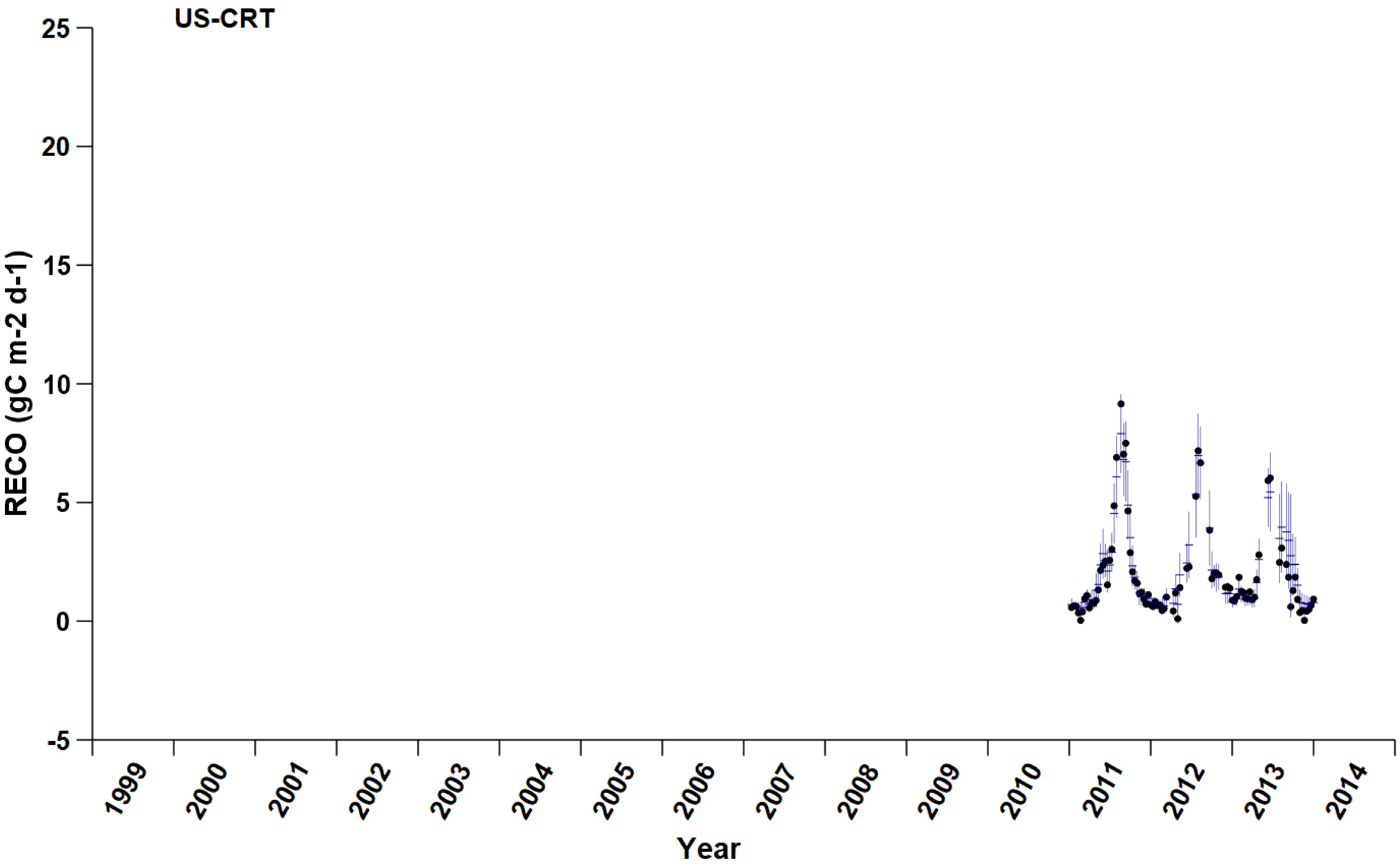
US-CRT
25
20
15
10
5
0
-5
RECO (gC m-2 d-1)
1999
2000
2001
2002
2003
2004
2005
2006
2007
2008
2009
2010
2011
2012
2013
2014
Year

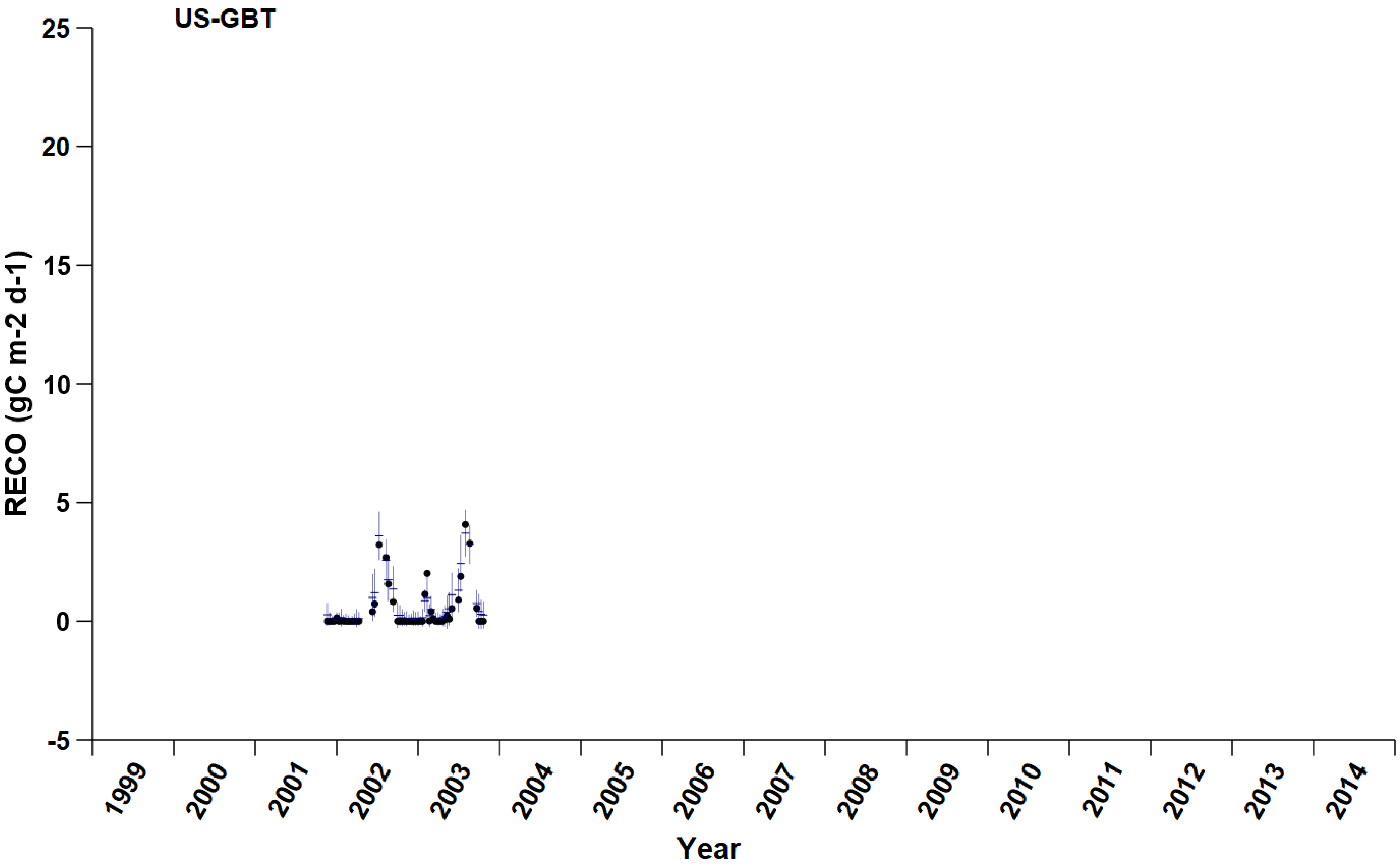
US-GBT
RECO (gC m-2 d-1)
25
20
15
10
5
0
-5
1999
2000
2001
2002
2003
2004
2005
2006
2007
2008
2009
2010
2011
2012
2013
2014
Year

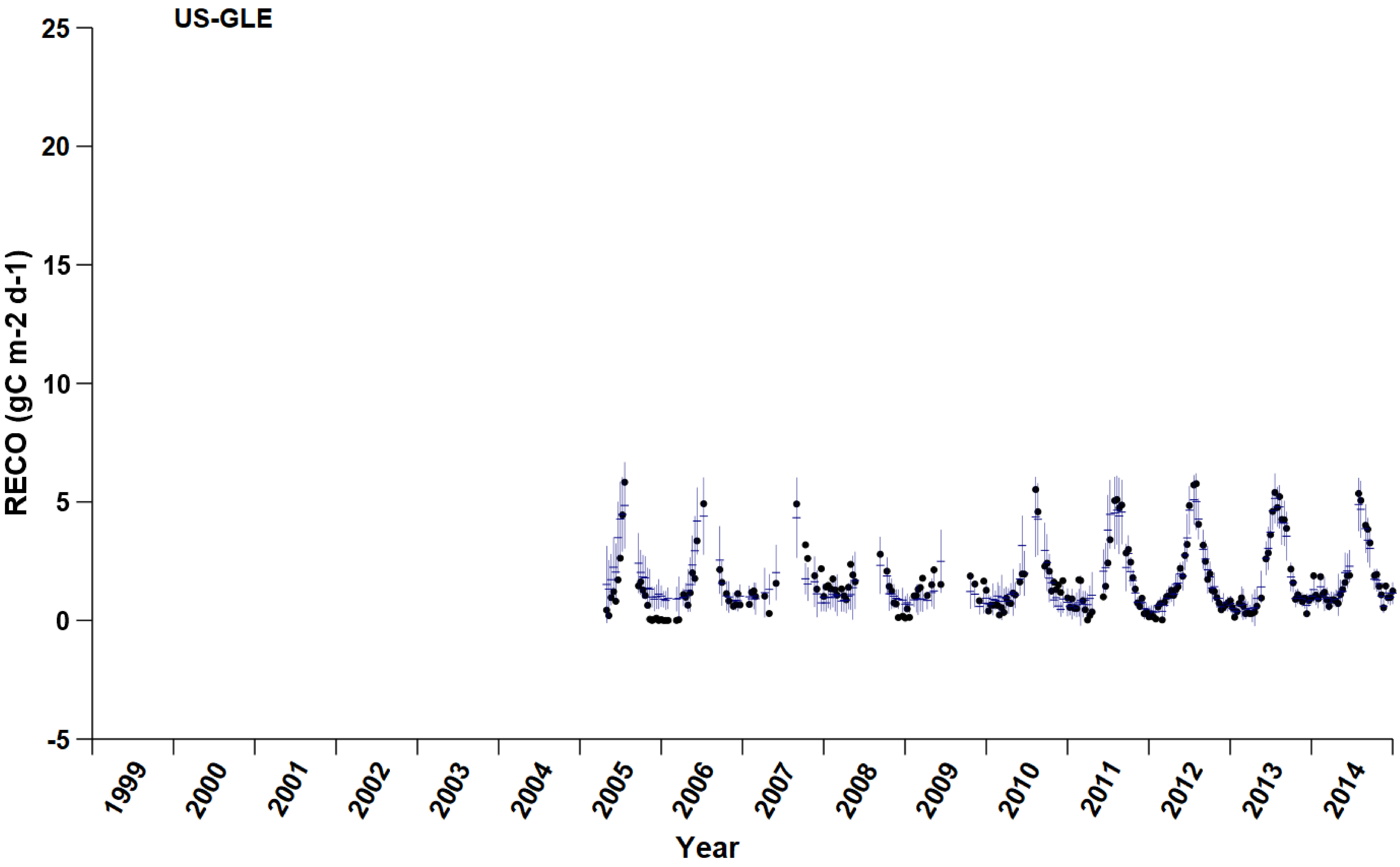
US-GLE
RECO (gC m-2 d-1)
25
20
15
10
5
0
-5
1999
2000
2001
2002
2003
2004
2005
2006
2007
2008
2009
2010
2011
2012
2013
2014
Year

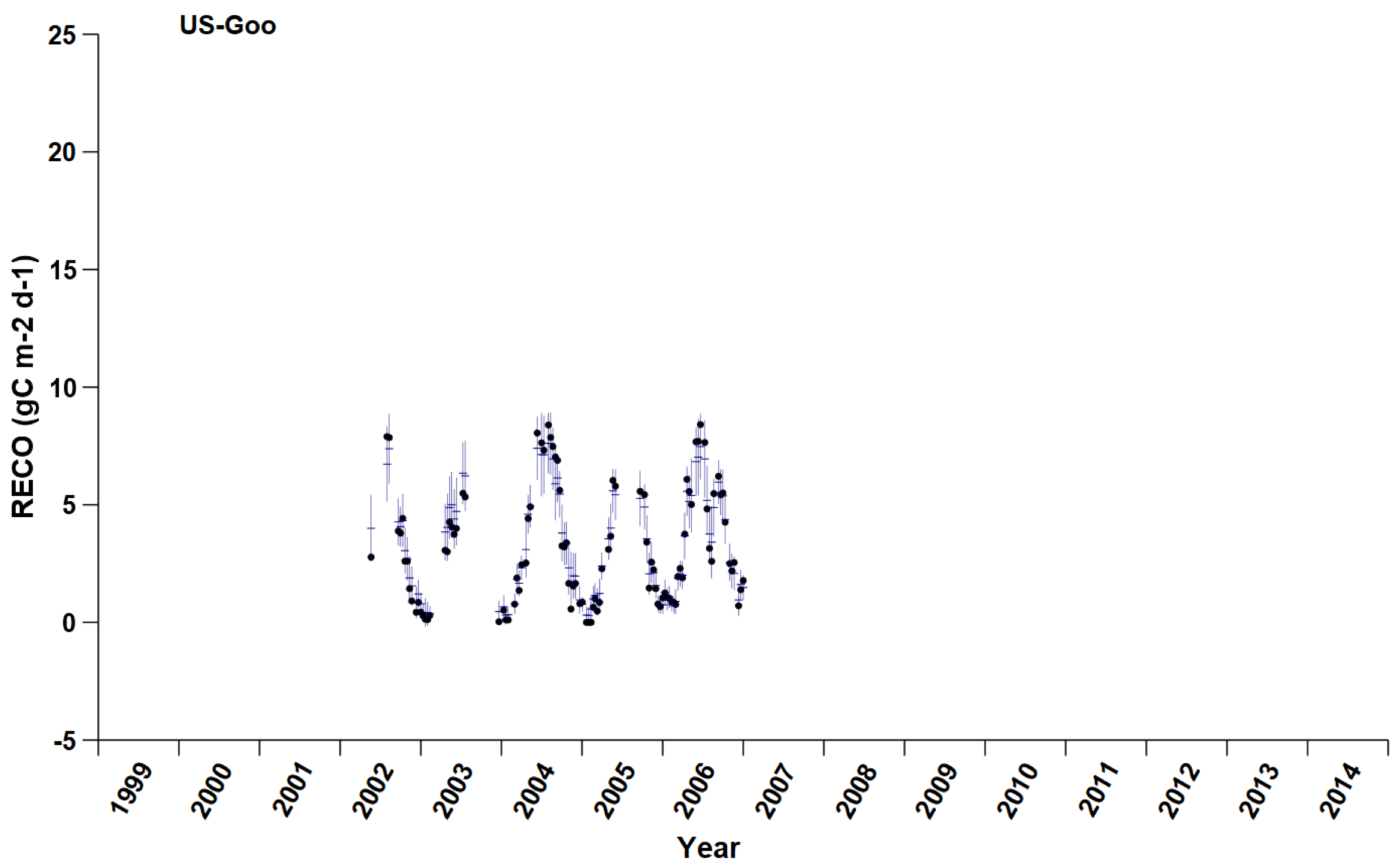
US-Goo
RECO (gC m-2 d-1)
25
20
15
10
5
0
-5
1999
2000
2001
2002
2003
2004
2005
2006
2007
2008
2009
2010
2011
2012
2013
2014
Year

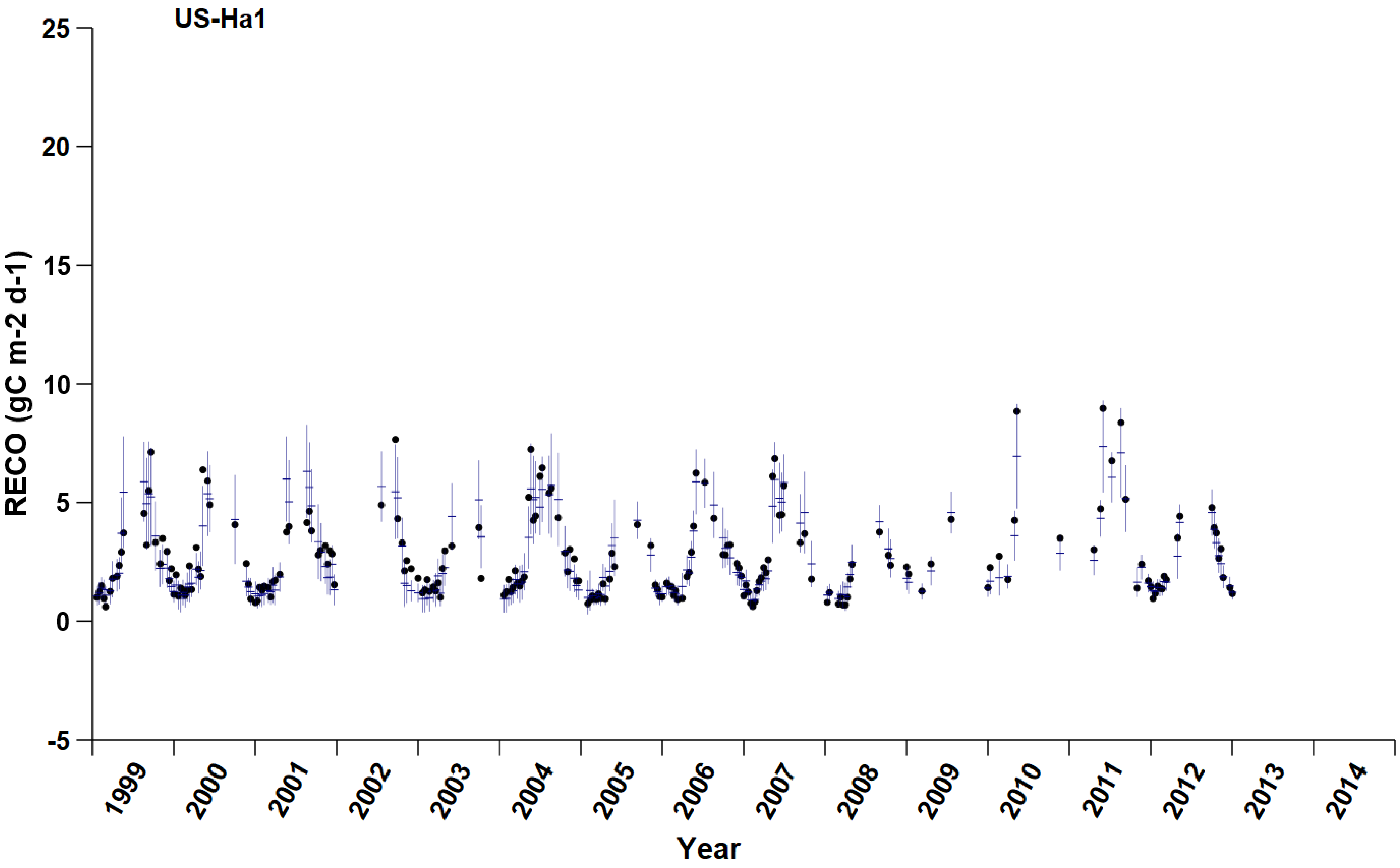
US-Ha1
RECO (gC m-2 d-1)
25
20
15
10
5
0
-5
1999
2000
2001
2002
2003
2004
2005
2006
2007
2008
2009
2010
2011
2012
2013
2014
Year

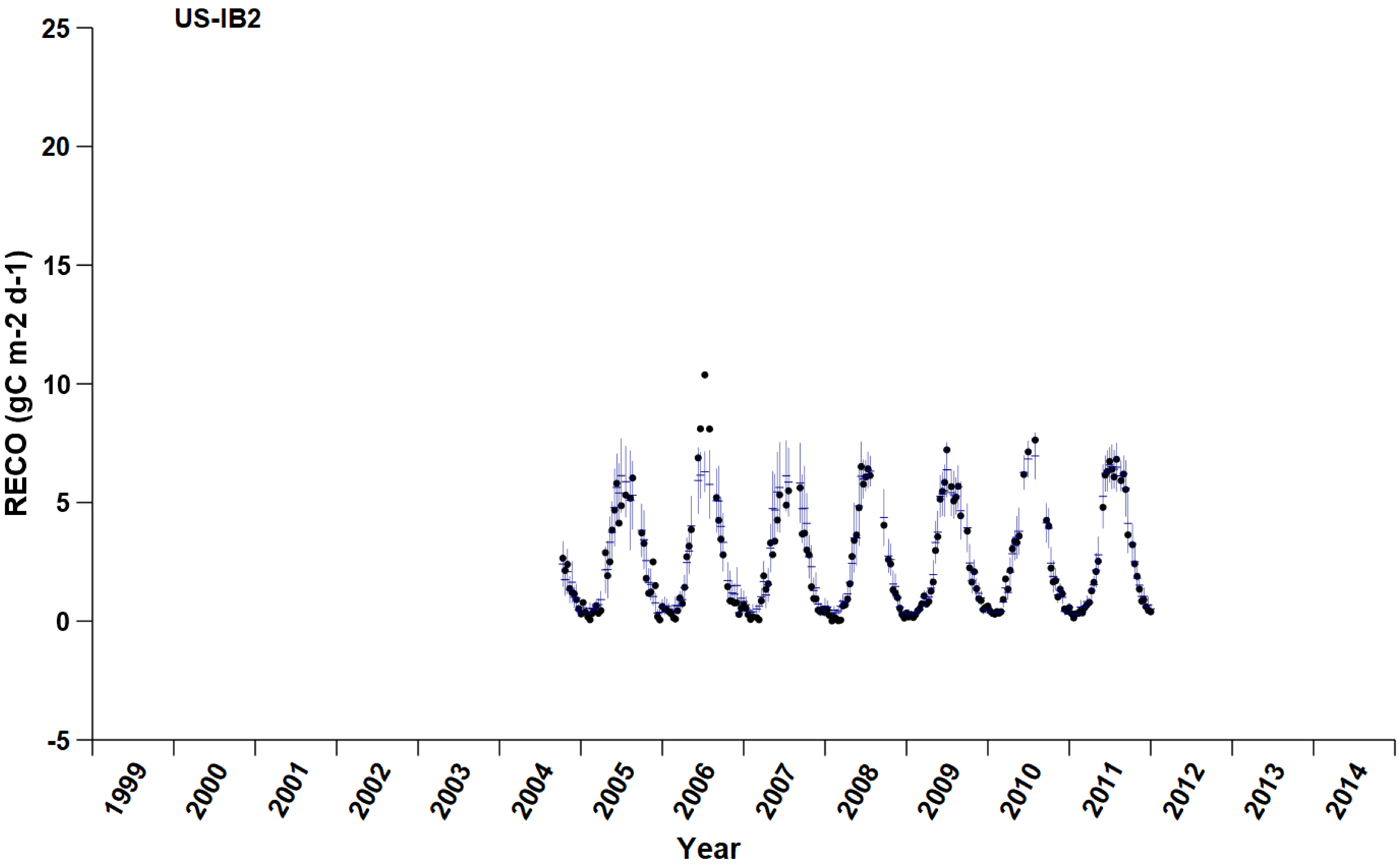

US-IB2
RECO (gC m-2 d-1)
25
20
15
10
5
0
-5
1999
2000
2001
2002
2003
2004
2005
2006
2007
2008
2009
2010
2011
2012
2013
2014
Year

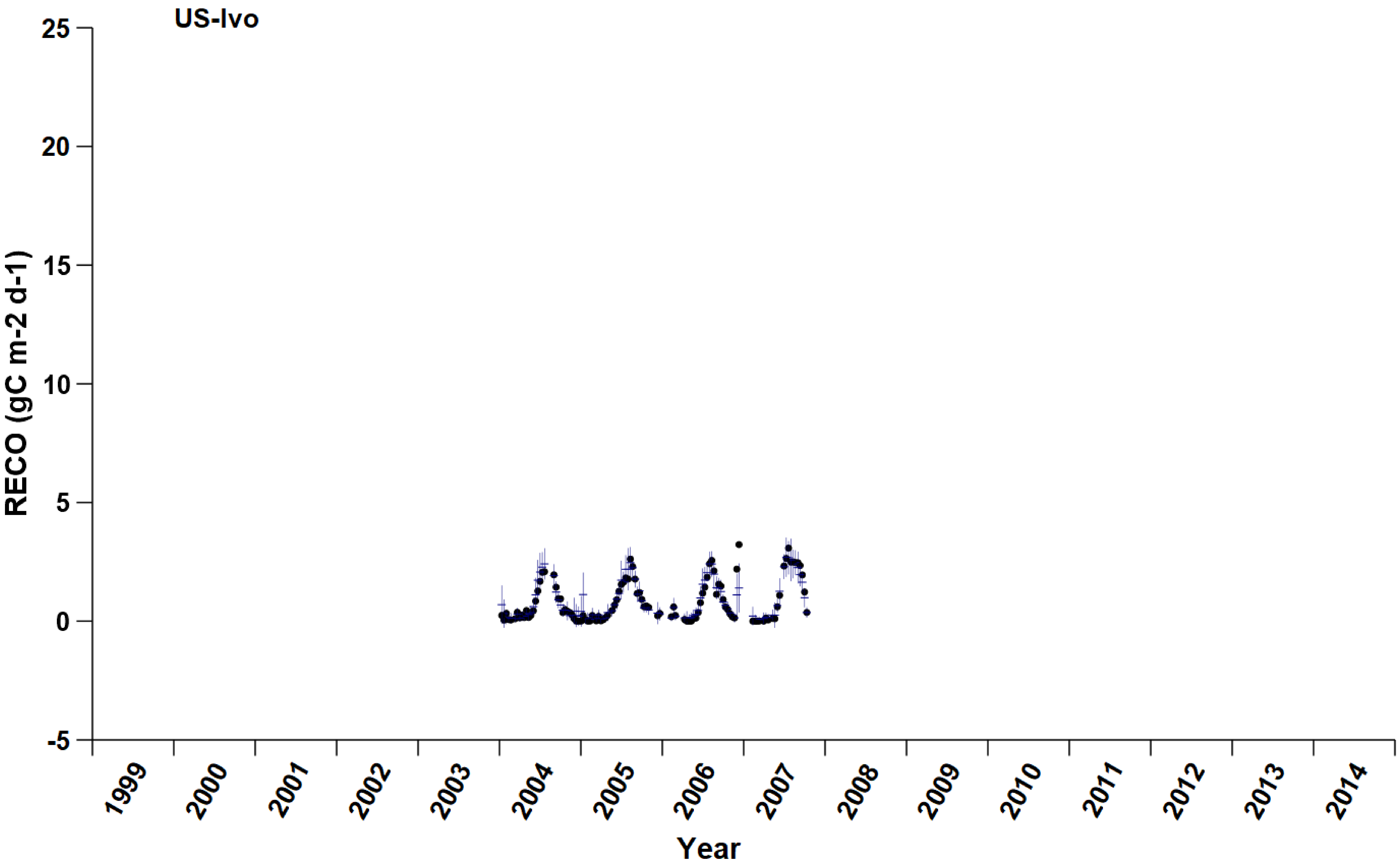
US-Ivo
RECO (gC m-2 d-1)
25
20
15
10
5
0
-5
1999
2000
2001
2002
2003
2004
2005
2006
2007
2008
2009
2010
2011
2012
2013
2014
Year

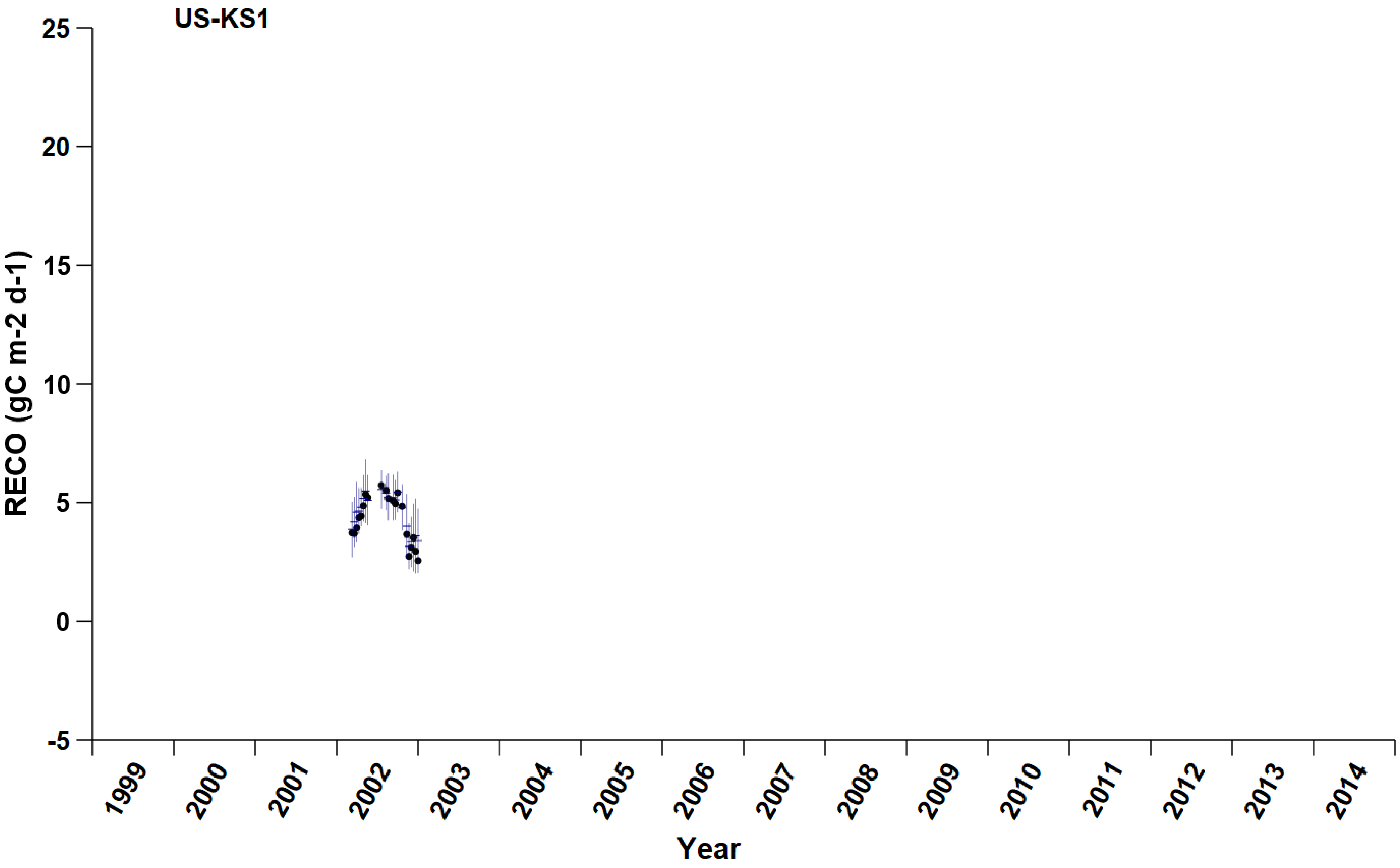
US-KS1
RECO (gC m-2 d-1)
25
20
15
10
5
0
-5
1999
2000
2001
2002
2003
2004
2005
2006
2007
2008
2009
2010
2011
2012
2013
2014
Year

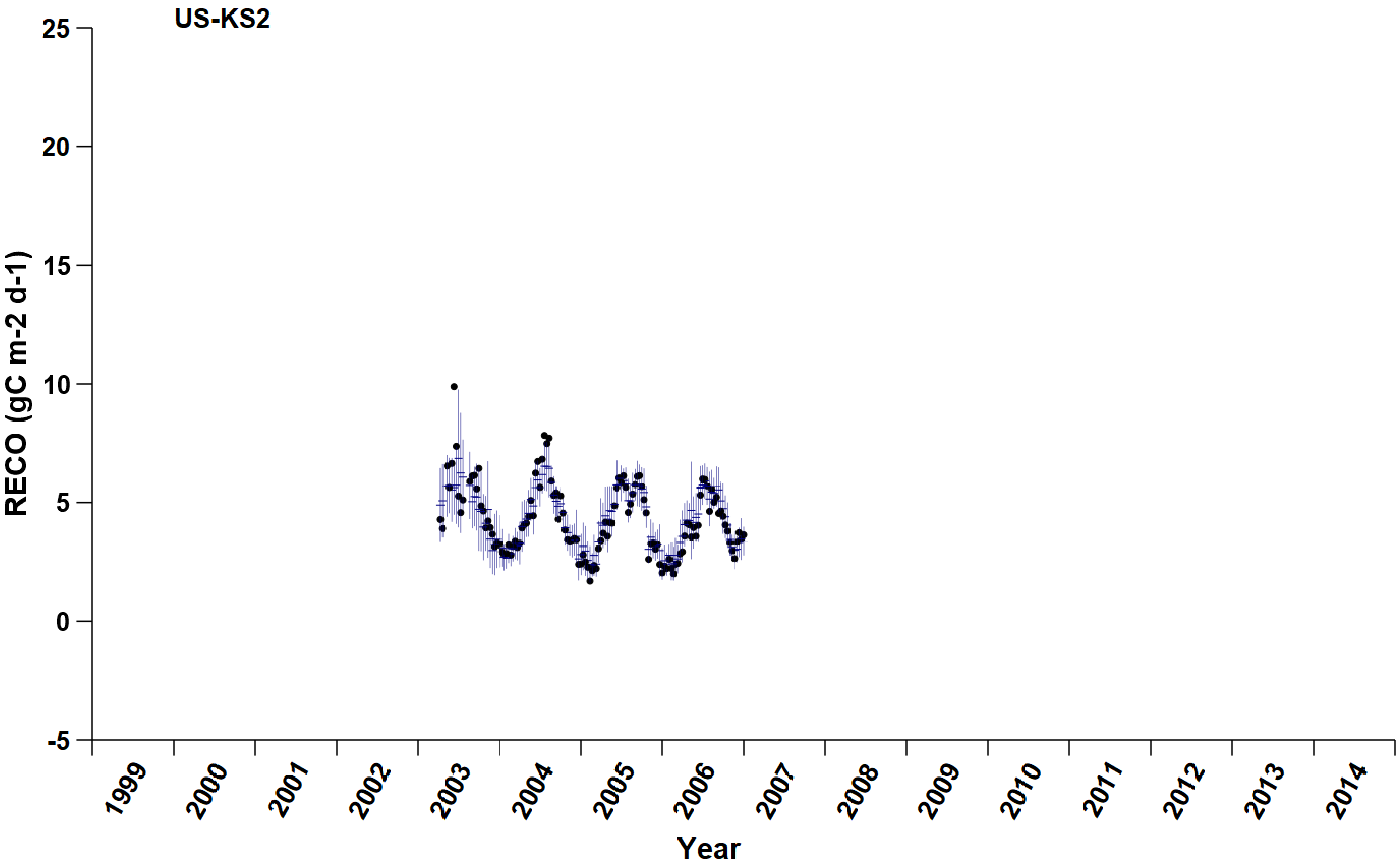
US-KS2
RECO (gC m-2 d-1)
25
20
15
10
5
0
-5
1999
2000
2001
2002
2003
2004
2005
2006
2007
2008
2009
2010
2011
2012
2013
2014
Year

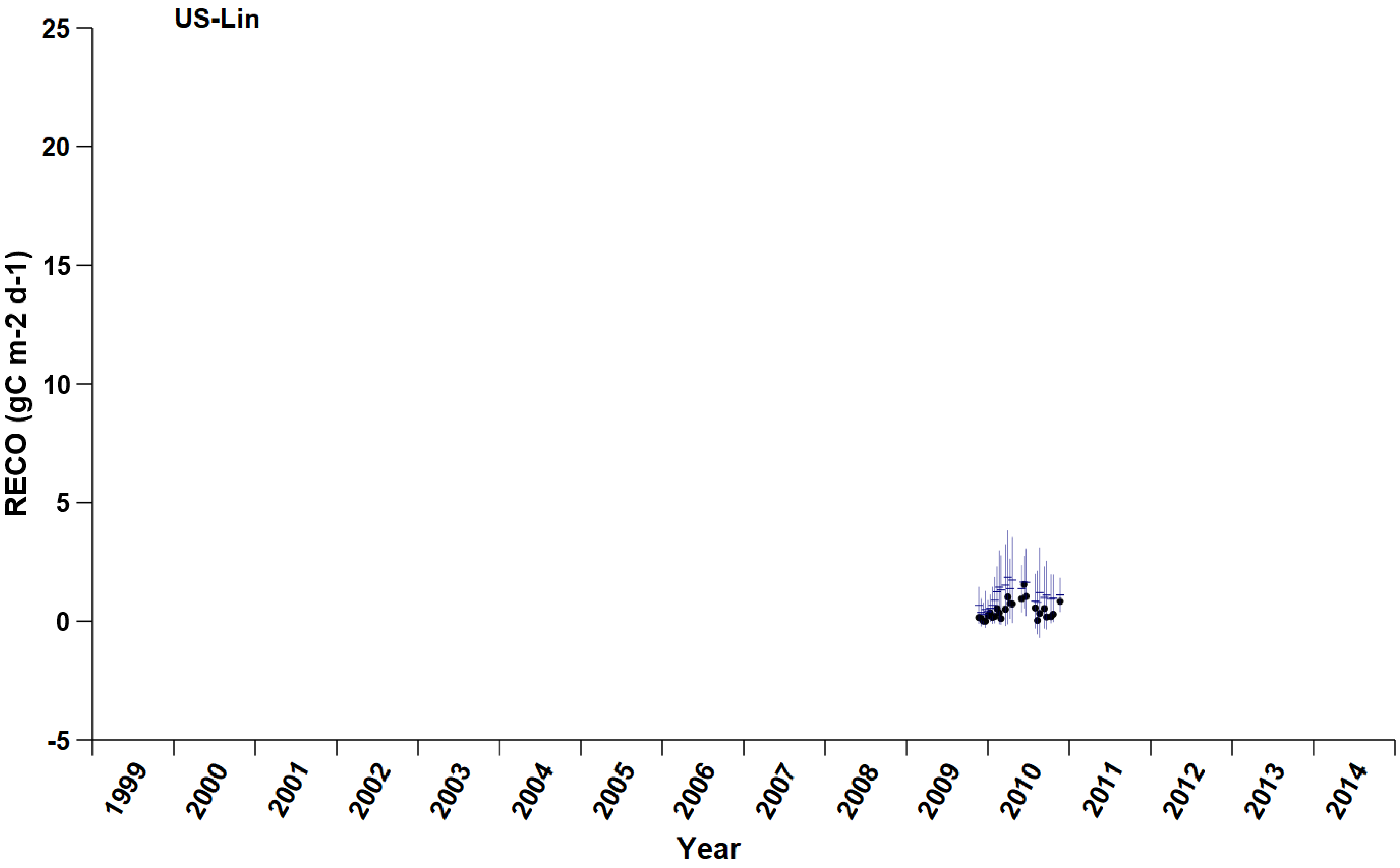

US-Lin
RECO (gC m-2 d-1)
25
20
15
10
5
0
-5
1999
2000
2001
2002
2003
2004
2005
2006
2007
2008
2009
2010
2011
2012
2013
2014
Year

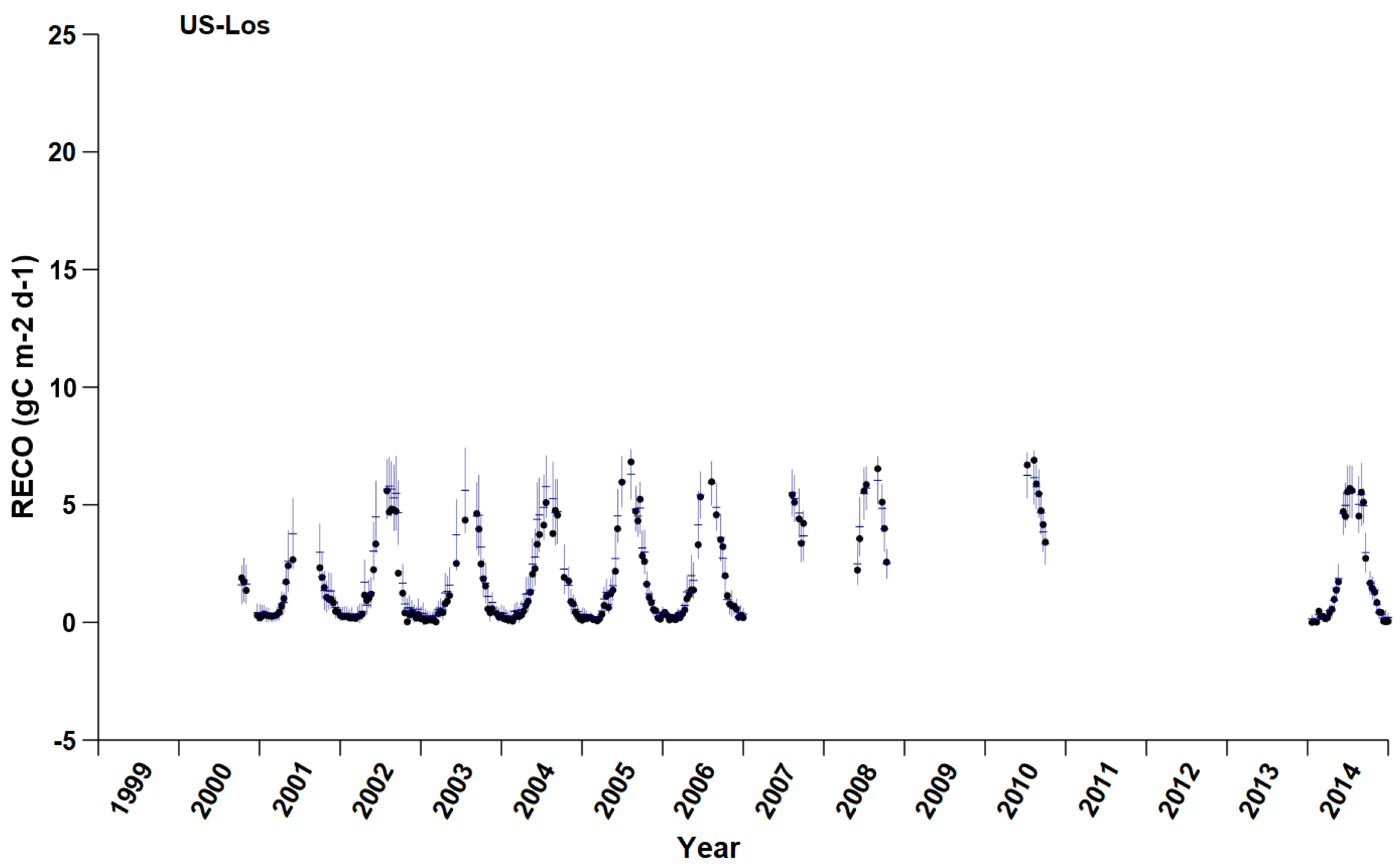

US-Los
RECO (gC m-2 d-1)
25
20
15
10
5
0
-5
1999
2000
2001
2002
2003
2004
2005
2006
2007
2008
2009
2010
2011
2012
2013
2014
Year

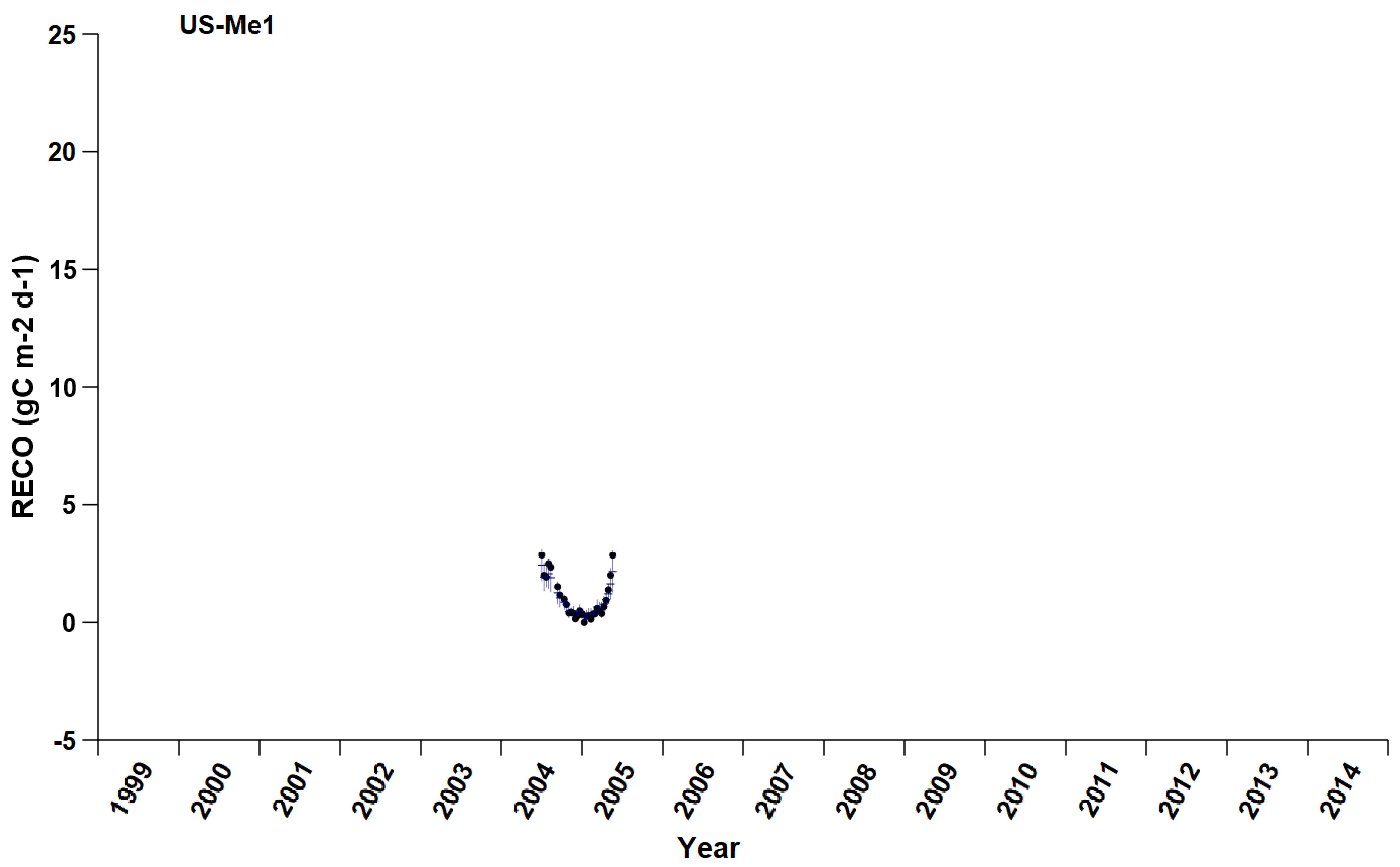
US-Me1
RECO (gC m-2 d-1)
25
20
15
10
5
0
-5
1999
2000
2001
2002
2003
2004
2005
2006
2007
2008
2009
2010
2011
2012
2013
2014
Year

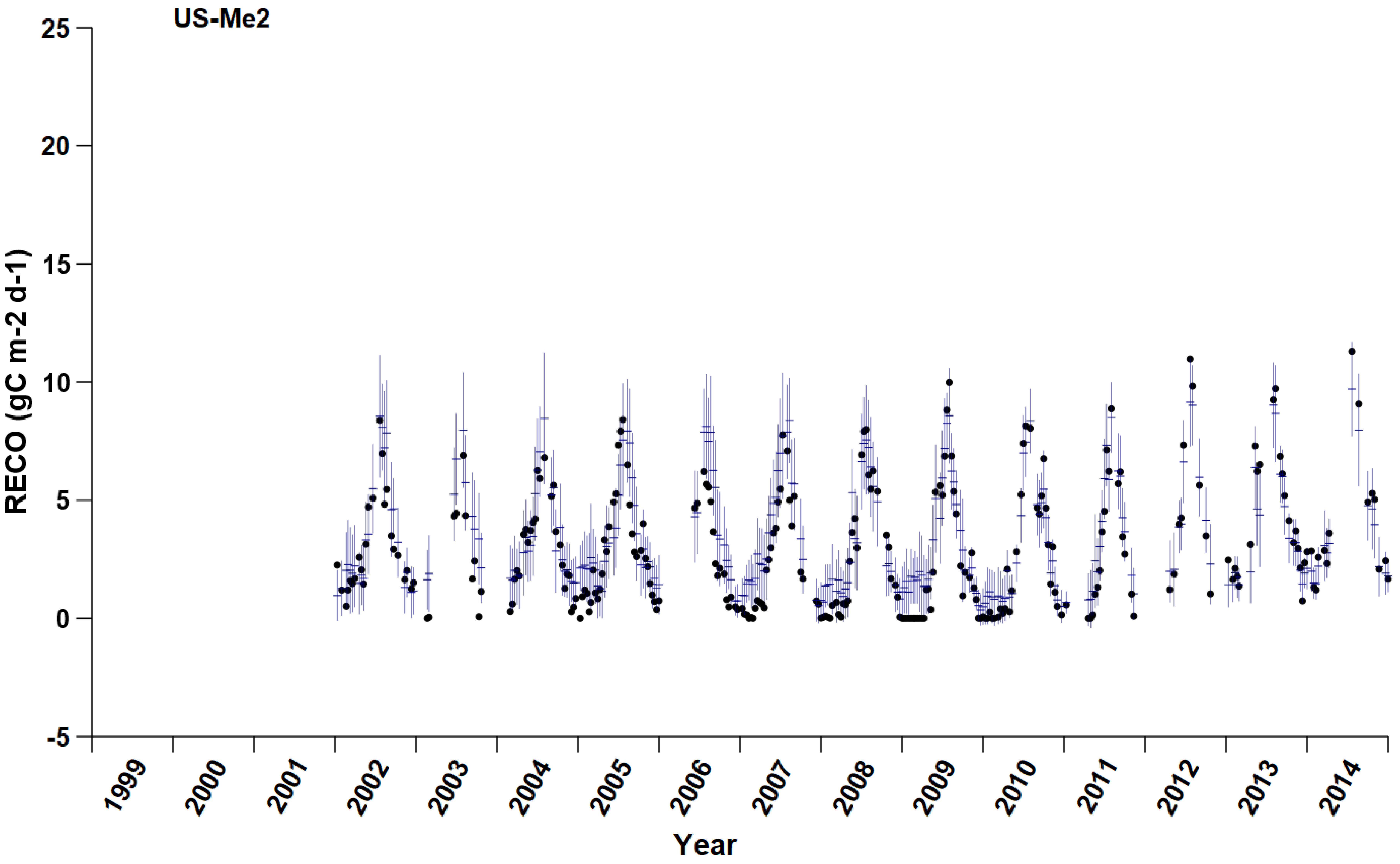
US-Me2
RECO (gC m-2 d-1)
25
20
15
10
5
0
-5
1999
2000
2001
2002
2003
2004
2005
2006
2007
2008
2009
2010
2011
2012
2013
2014
Year

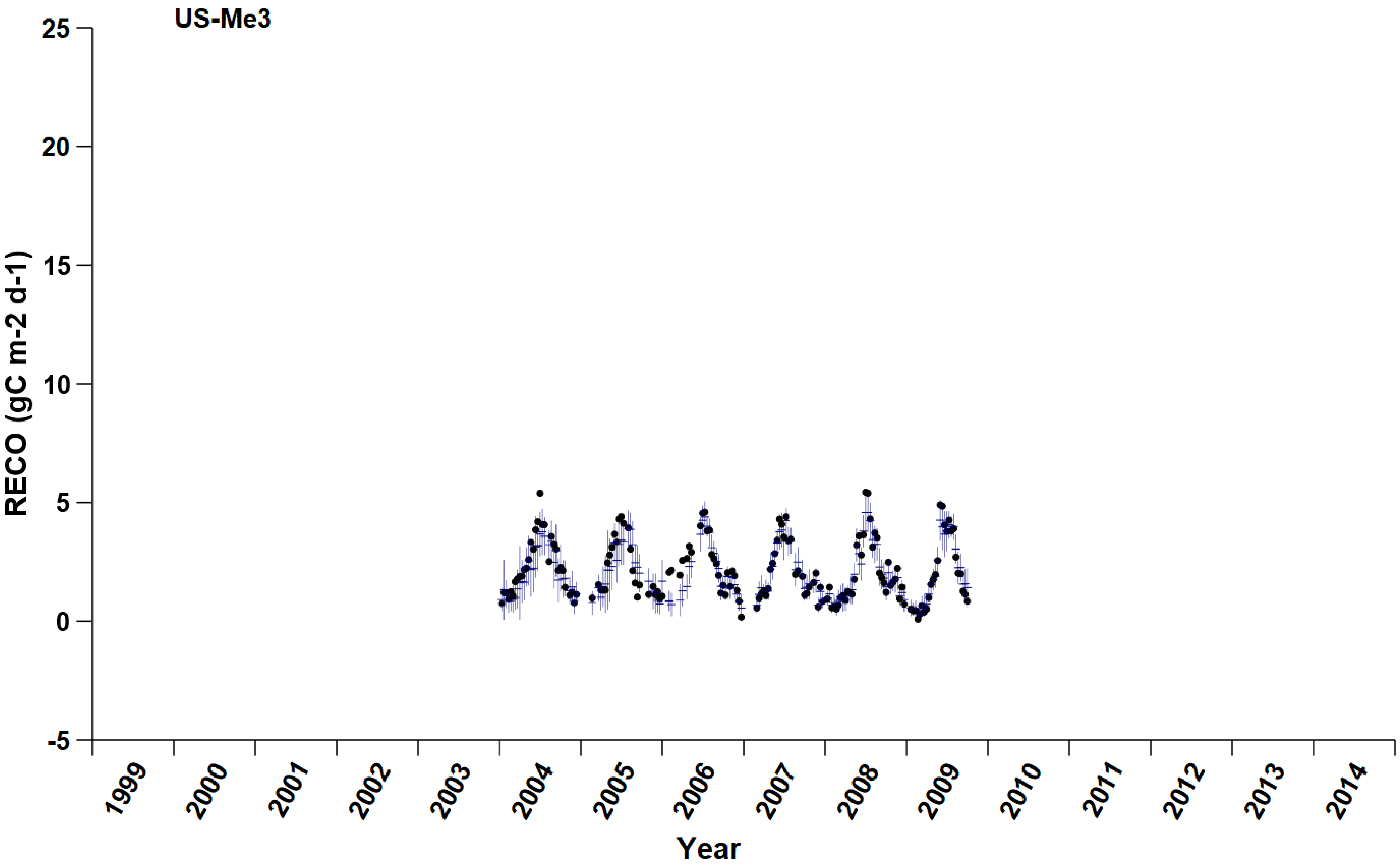
US-Me3
RECO (gC m-2 d-1)
25
20
15
10
5
0
-5
1999
2000
2001
2002
2003
2004
2005
2006
2007
2008
2009
2010
2011
2012
2013
2014
Year

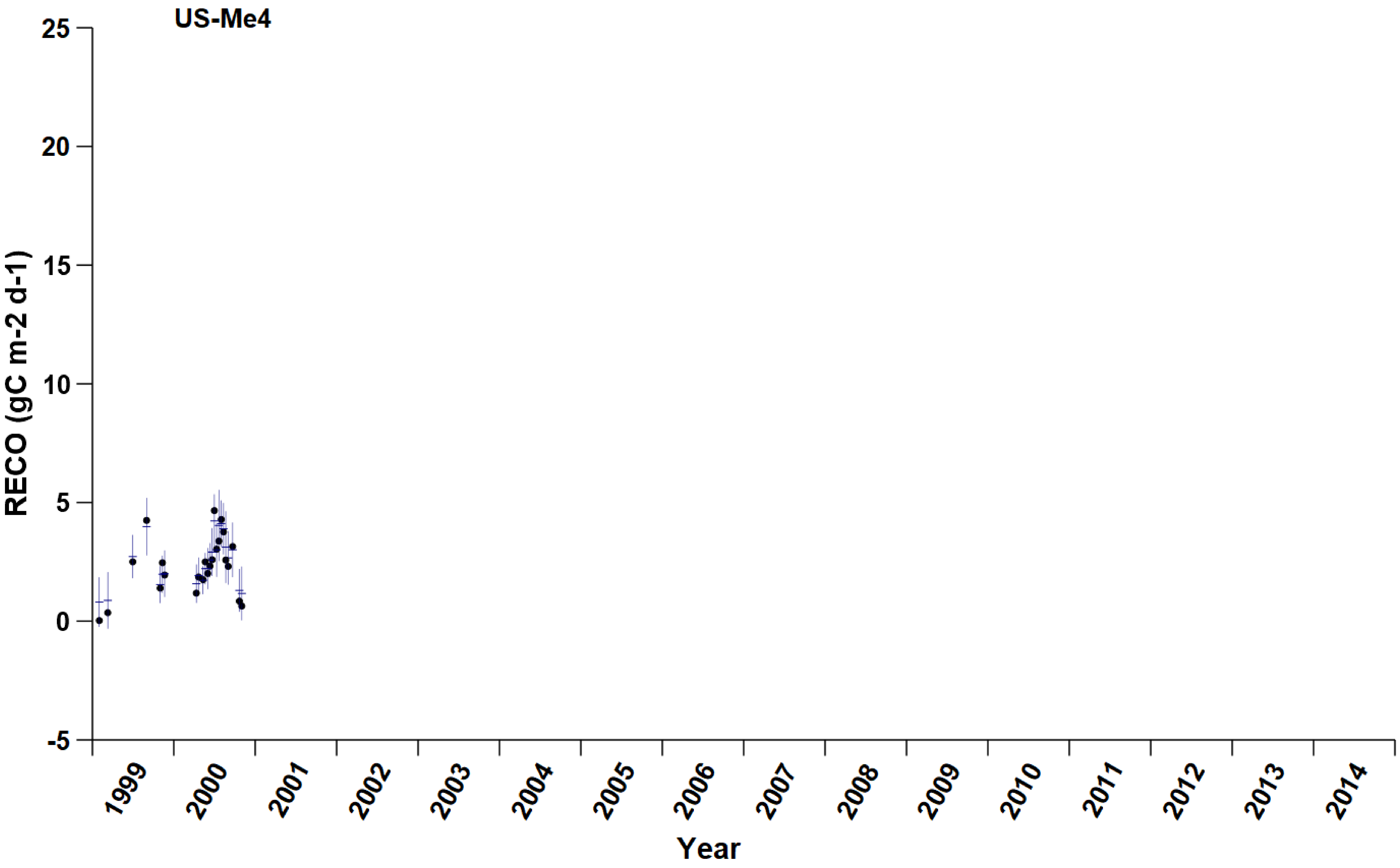

US-Me4
RECO (gC m-2 d-1)
25
20
15
10
5
0
-5
1999
2000
2001
2002
2003
2004
2005
2006
2007
2008
2009
2010
2011
2012
2013
2014
Year

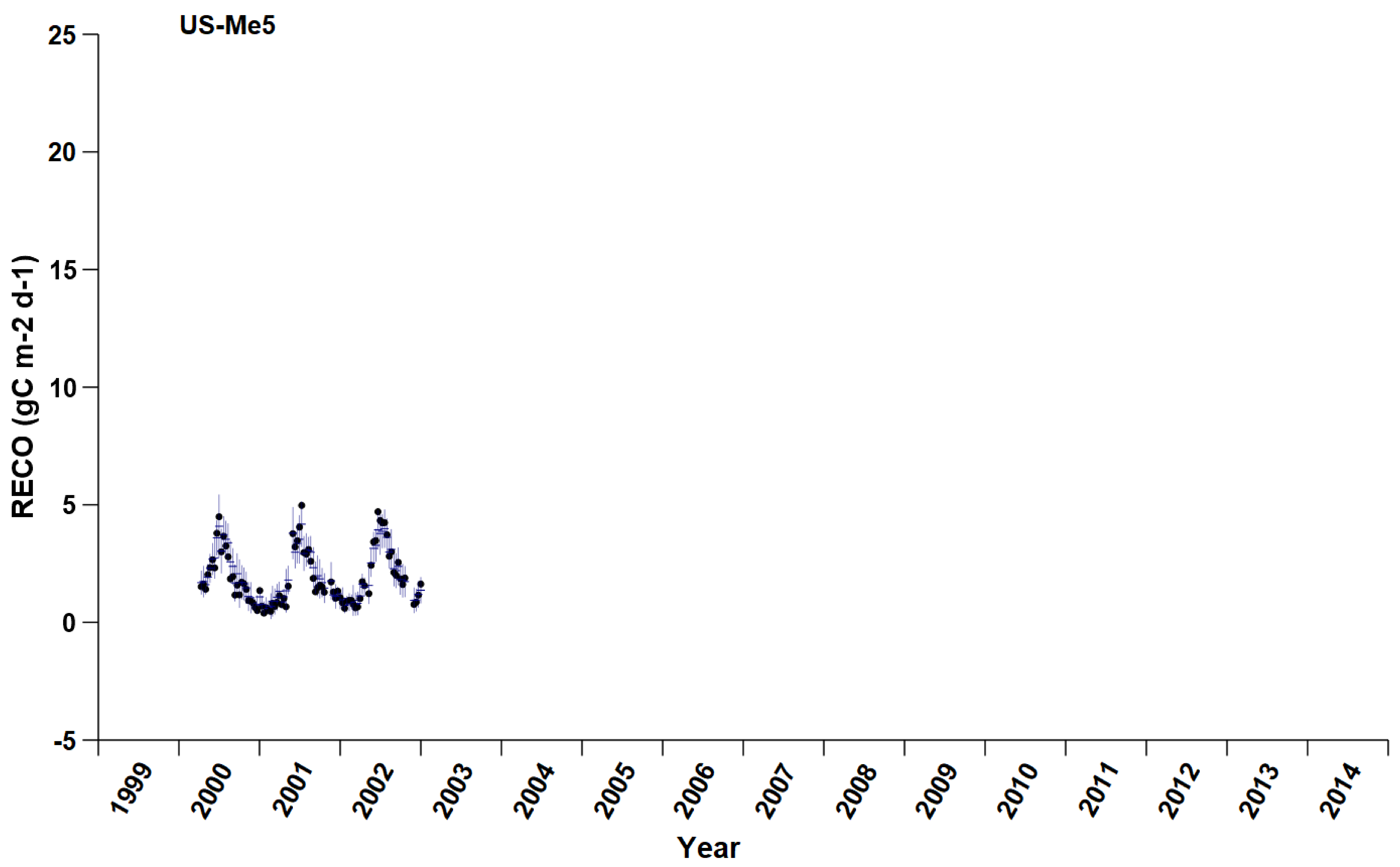

US-Me5
RECO (gC m-2 d-1)
25
20
15
10
5
0
-5
1999
2000
2001
2002
2003
2004
2005
2006
2007
2008
2009
2010
2011
2012
2013
2014
Year

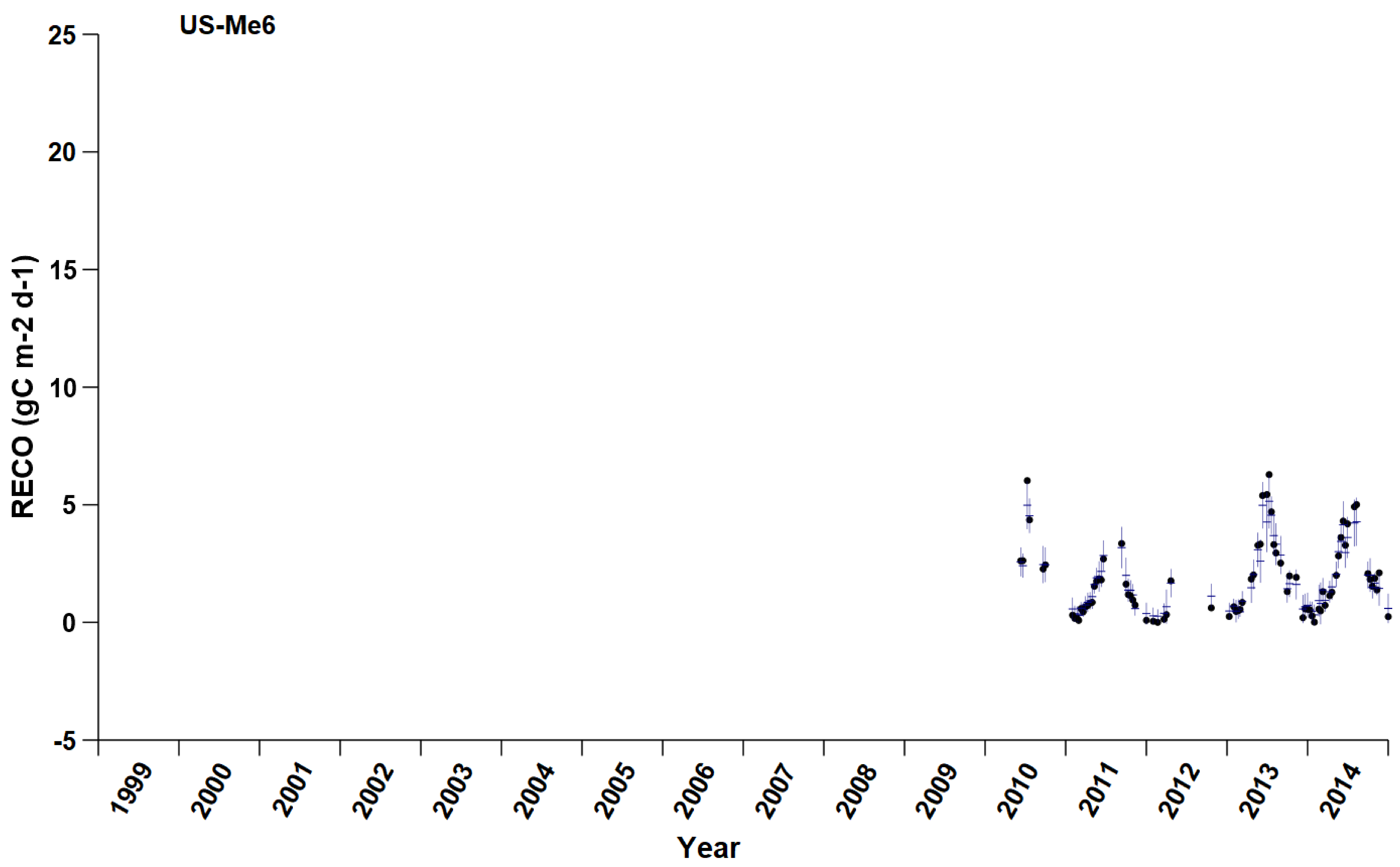
US-Me6
RECO (gC m-2 d-1)
25
20
15
10
5
0
-5
1999
2000
2001
2002
2003
2004
2005
2006
2007
2008
2009
2010
2011
2012
2013
2014
Year

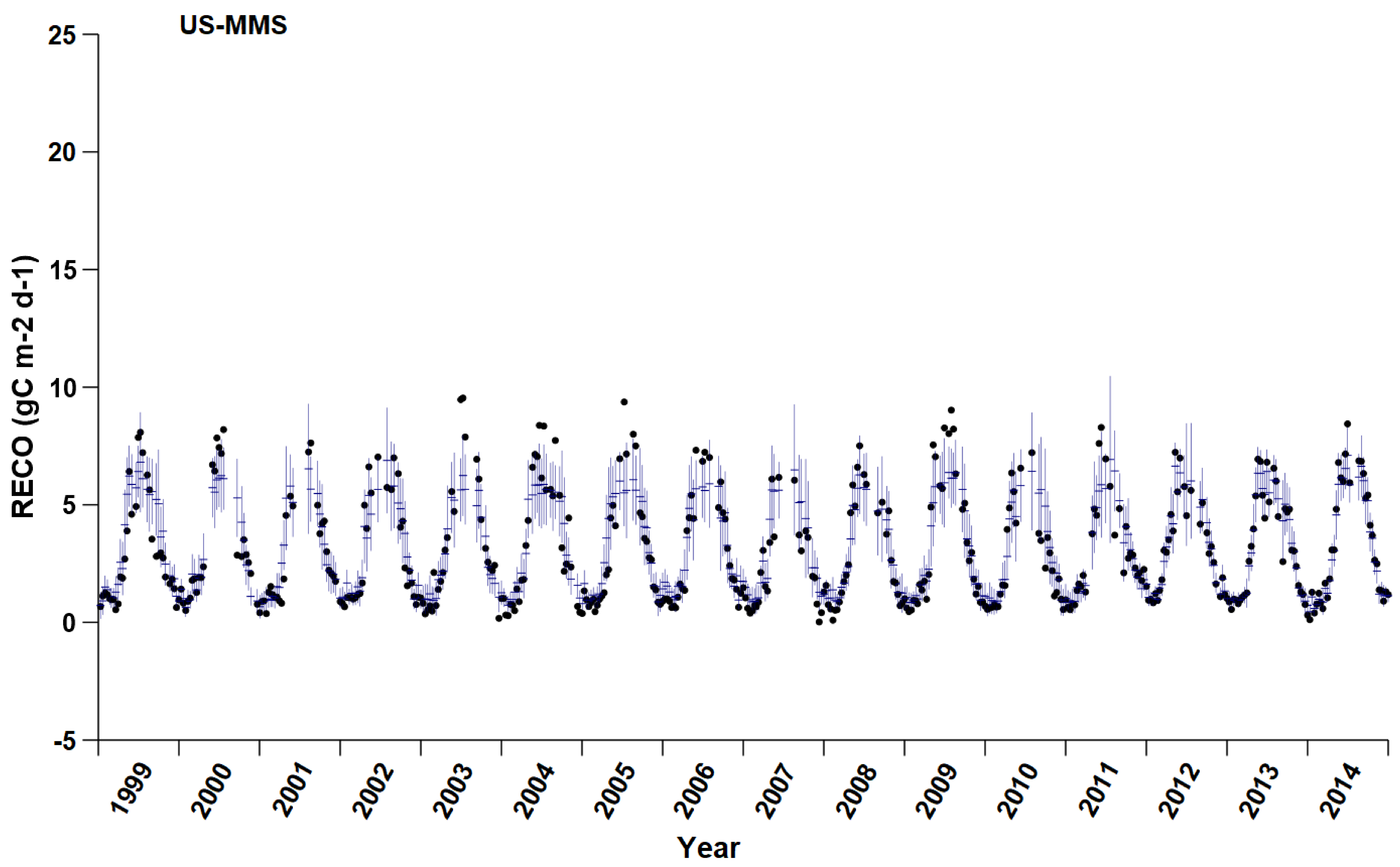
US-MMS
RECO (gC m-2 d-1)
Year
25
20
15
10
5
0
-5
1999
2000
2001
2002
2003
2004
2005
2006
2007
2008
2009
2010
2011
2012
2013
2014

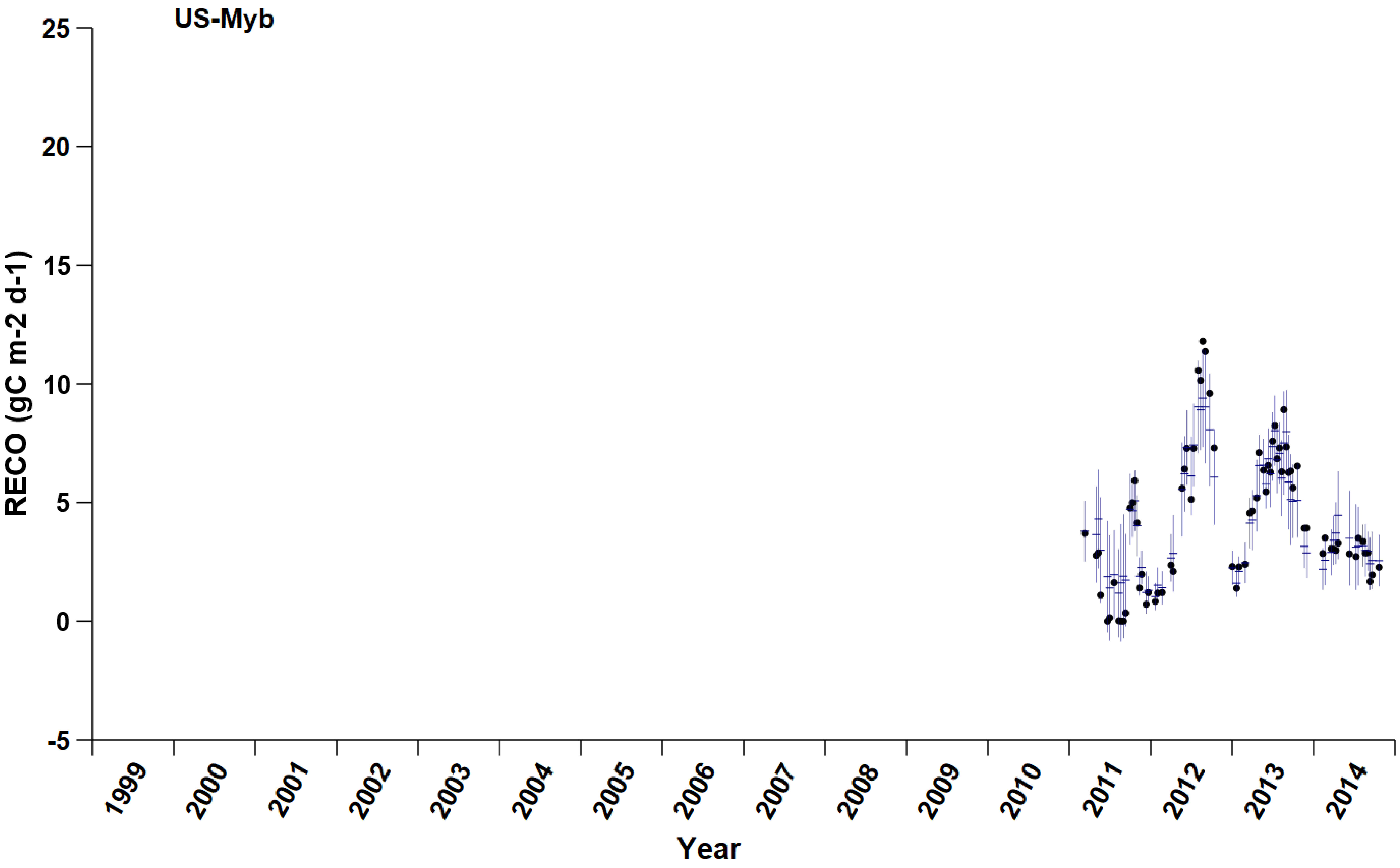
US-Myb
RECO (gC m-2 d-1)
25
20
15
10
5
0
-5
1999
2000
2001
2002
2003
2004
2005
2006
2007
2008
2009
2010
2011
2012
2013
2014
Year

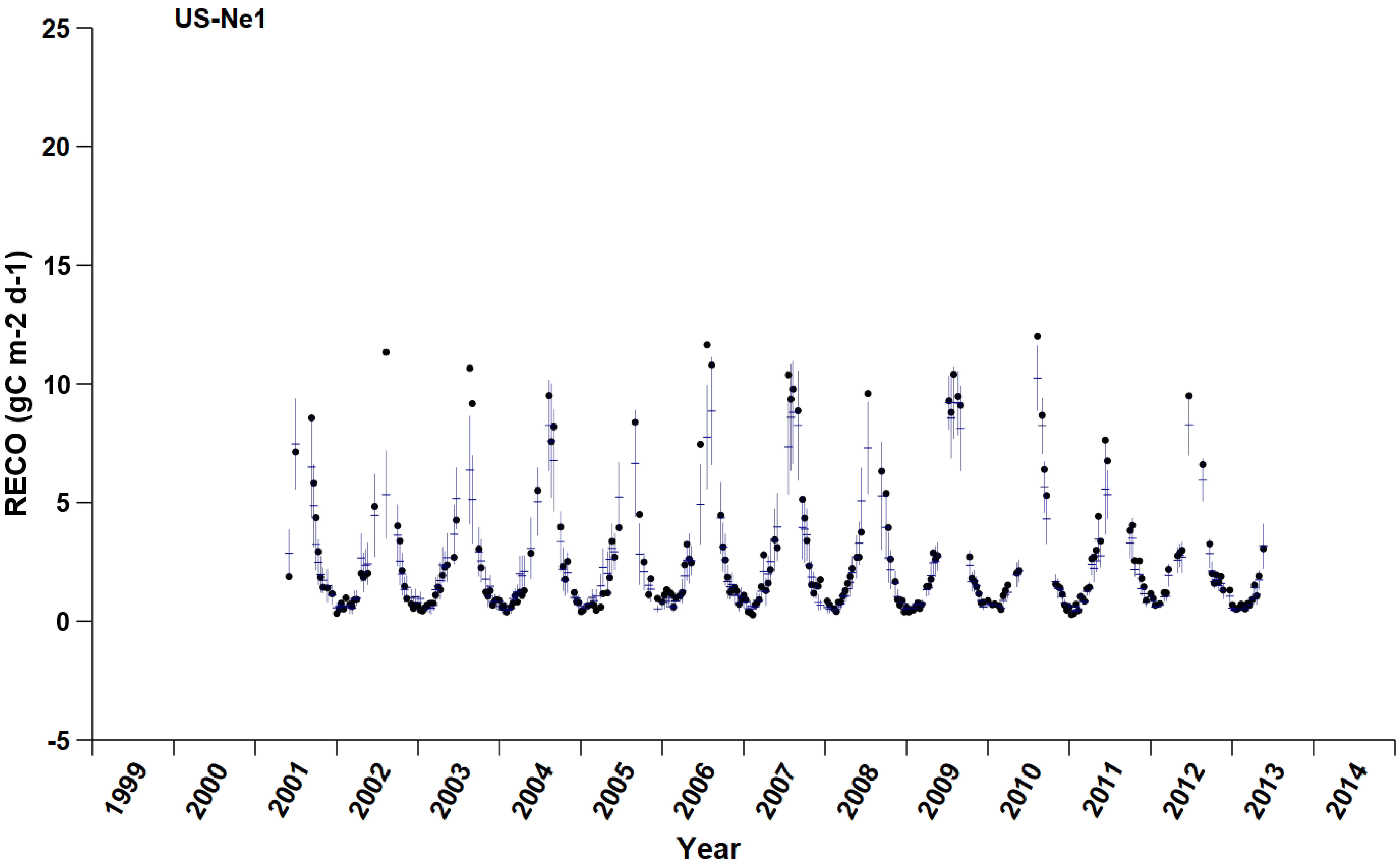
US-Ne1
RECO (gC m-2 d-1)
25
20
15
10
5
0
-5
1999
2000
2001
2002
2003
2004
2005
2006
2007
2008
2009
2010
2011
2012
2013
2014
Year

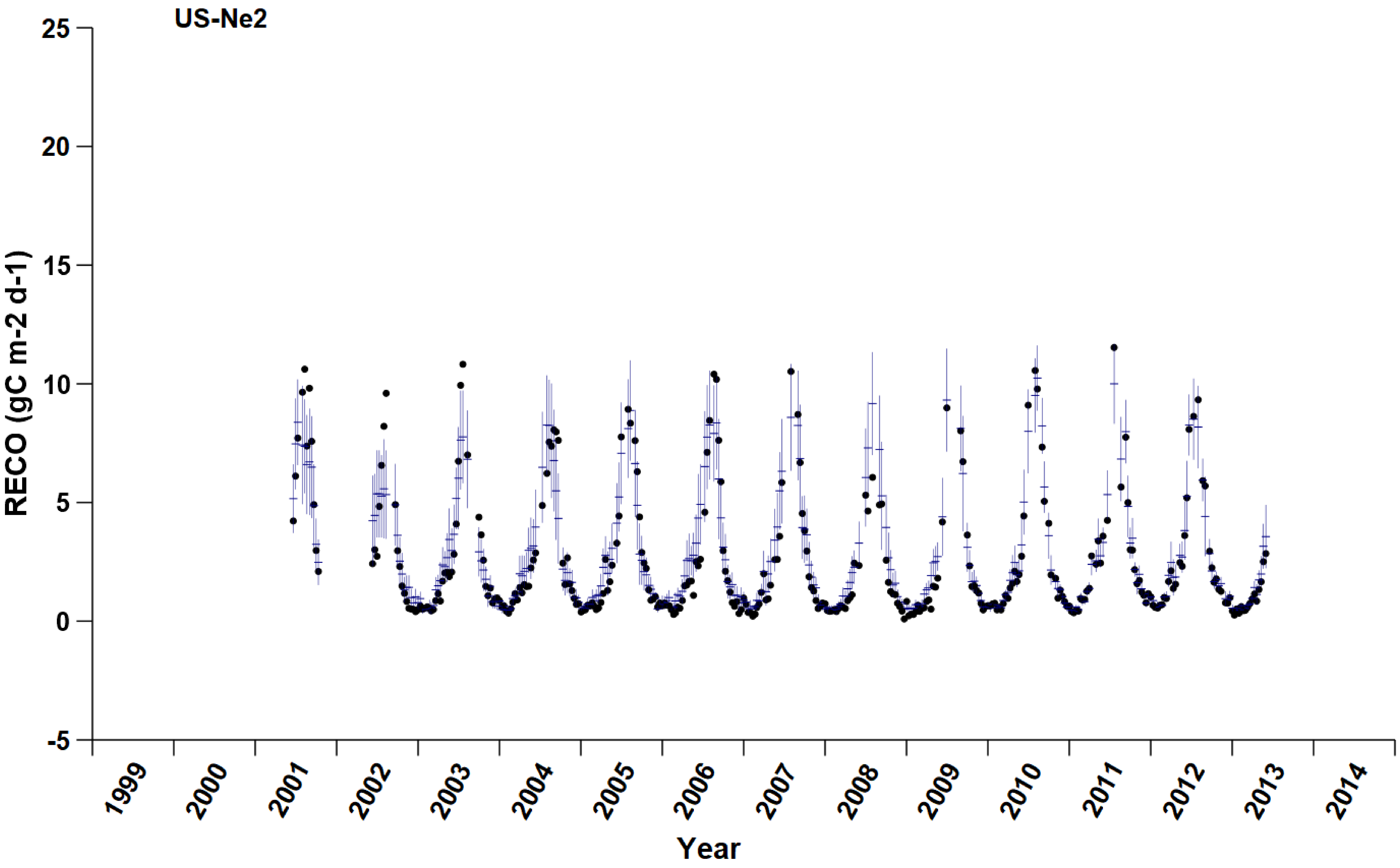
US-Ne2
RECO (gC m-2 d-1)
25
20
15
10
5
0
-5
1999
2000
2001
2002
2003
2004
2005
2006
2007
2008
2009
2010
2011
2012
2013
2014
Year

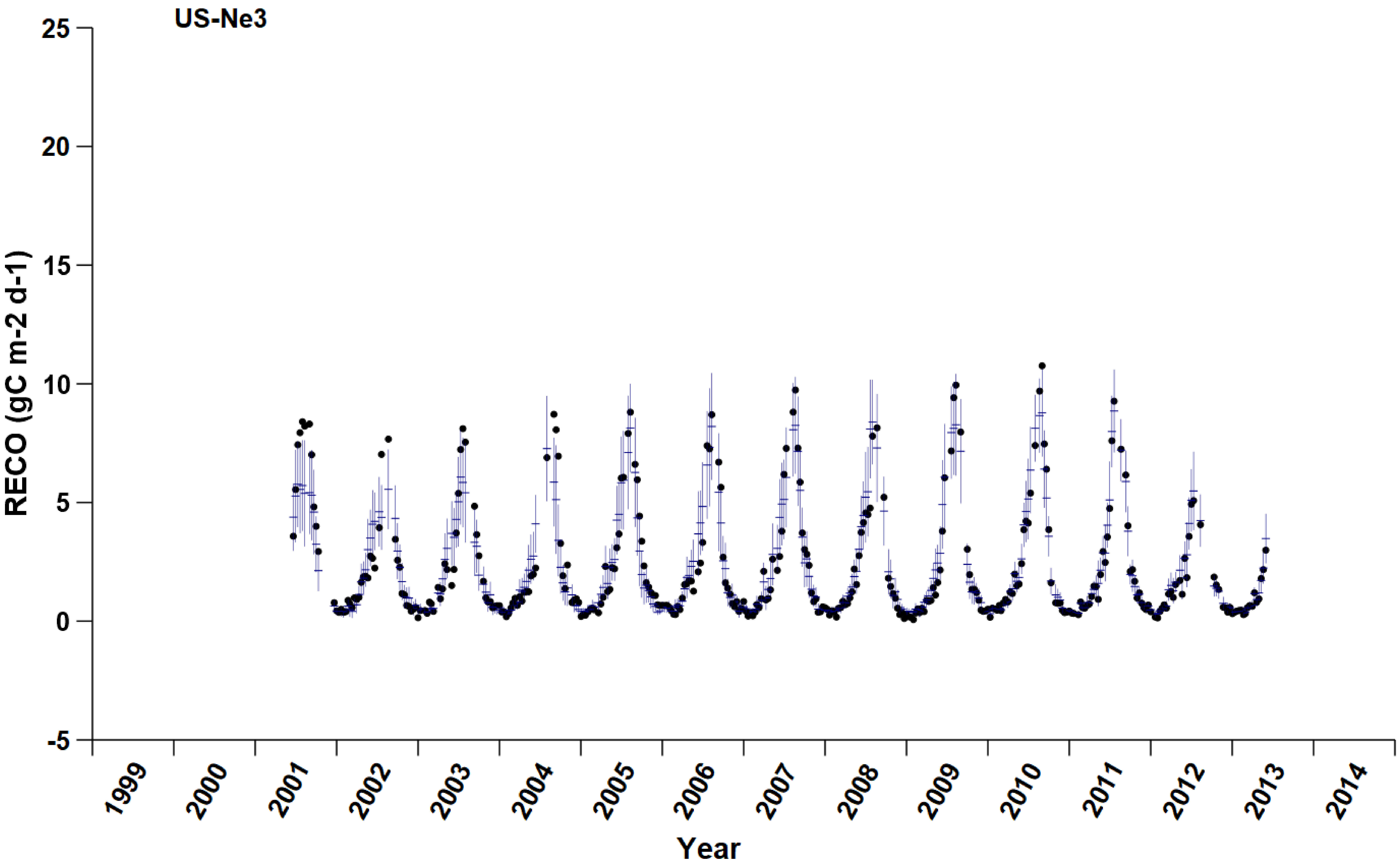
US-Ne3
RECO (gC m-2 d-1)
25
20
15
10
5
0
-5
1999
2000
2001
2002
2003
2004
2005
2006
2007
2008
2009
2010
2011
2012
2013
2014
Year

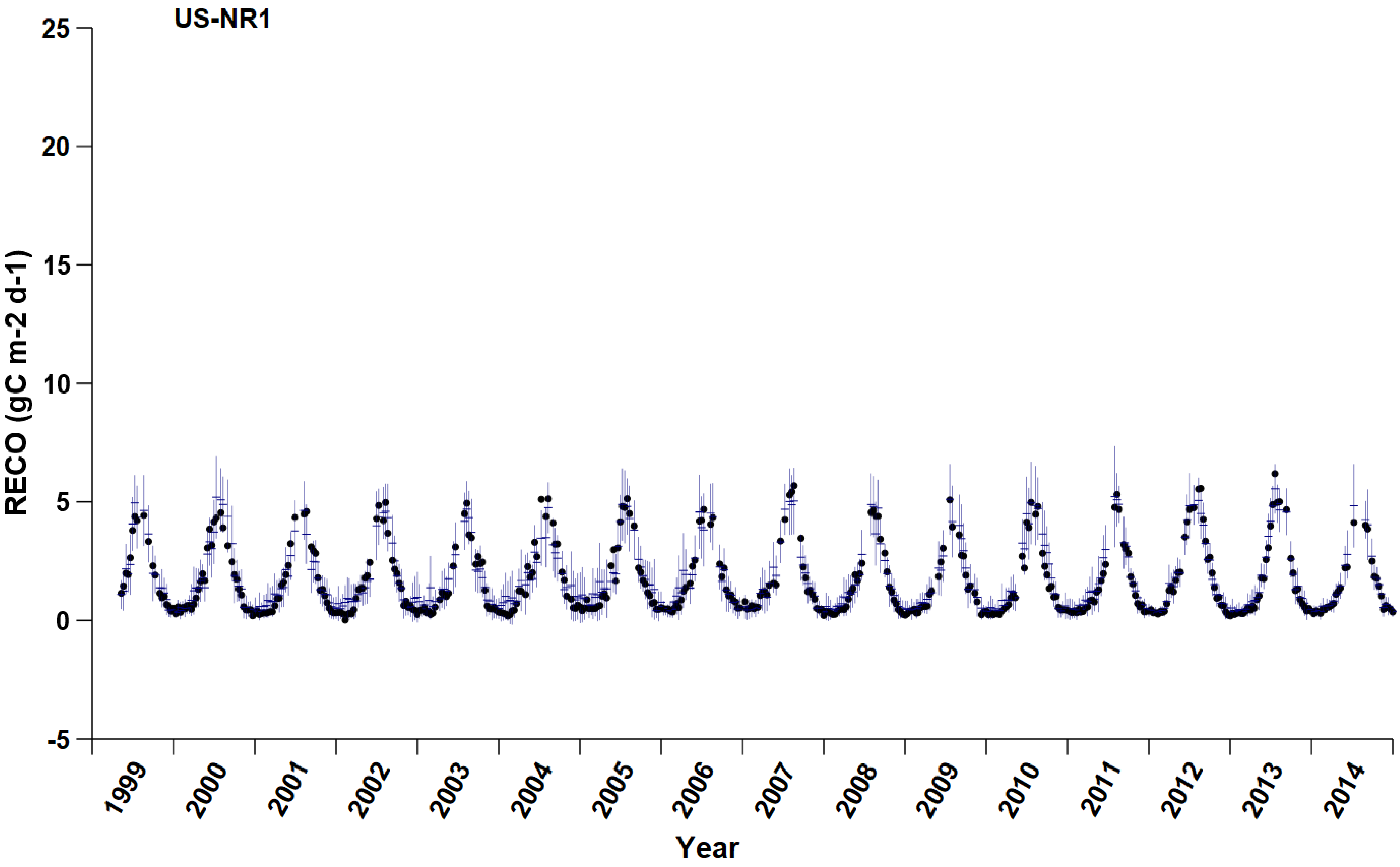
US-NR1
RECO (gC m-2 d-1)
25
20
15
10
5
0
-5
1999
2000
2001
2002
2003
2004
2005
2006
2007
2008
2009
2010
2011
2012
2013
2014
Year

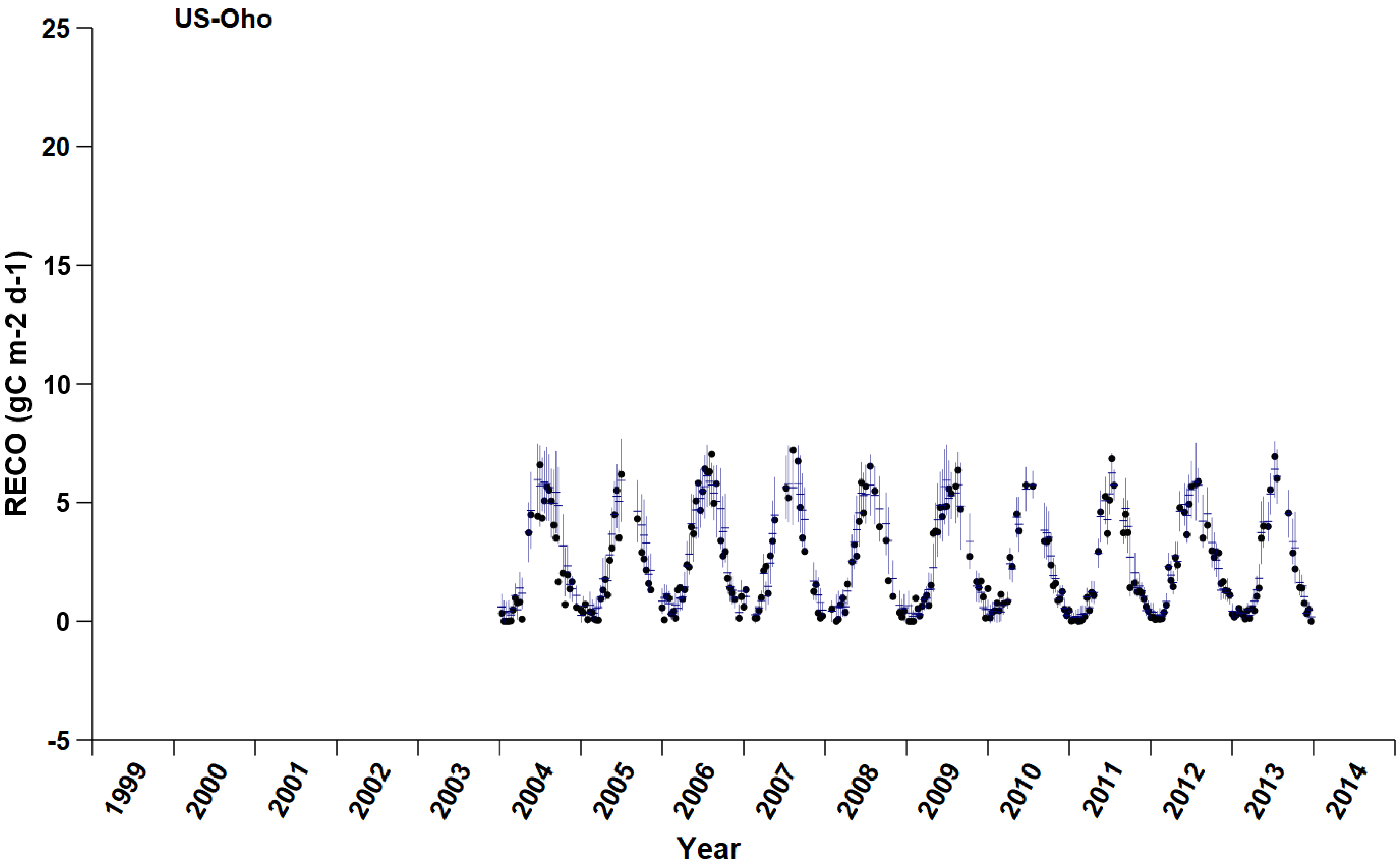

US-Oho
RECO (gC m-2 d-1)
25
20
15
10
5
0
-5
1999
2000
2001
2002
2003
2004
2005
2006
2007
2008
2009
2010
2011
2012
2013
2014
Year

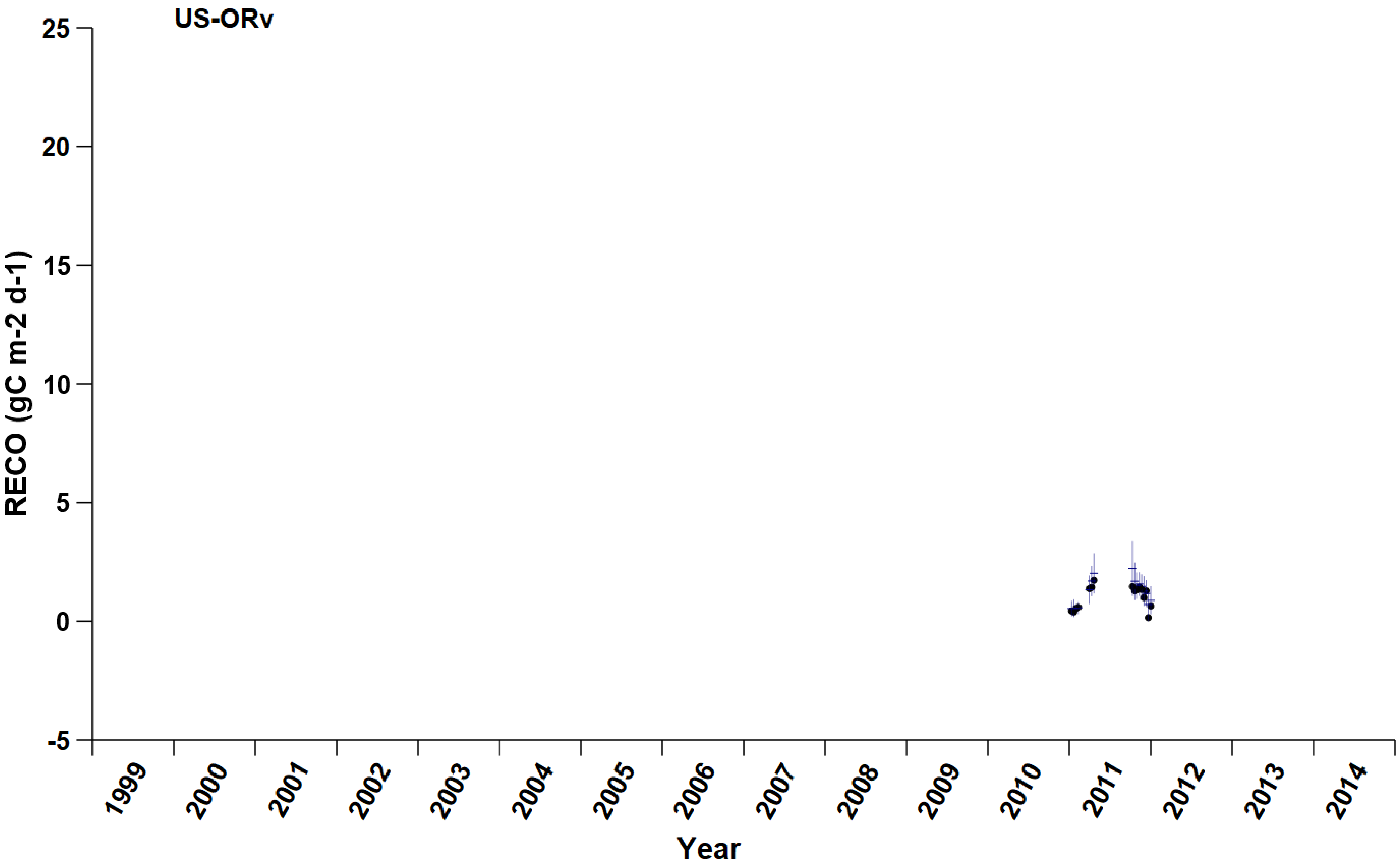
US-ORv
RECO (gC m-2 d-1)
25
20
15
10
5
0
-5
1999
2000
2001
2002
2003
2004
2005
2006
2007
2008
2009
2010
2011
2012
2013
2014
Year

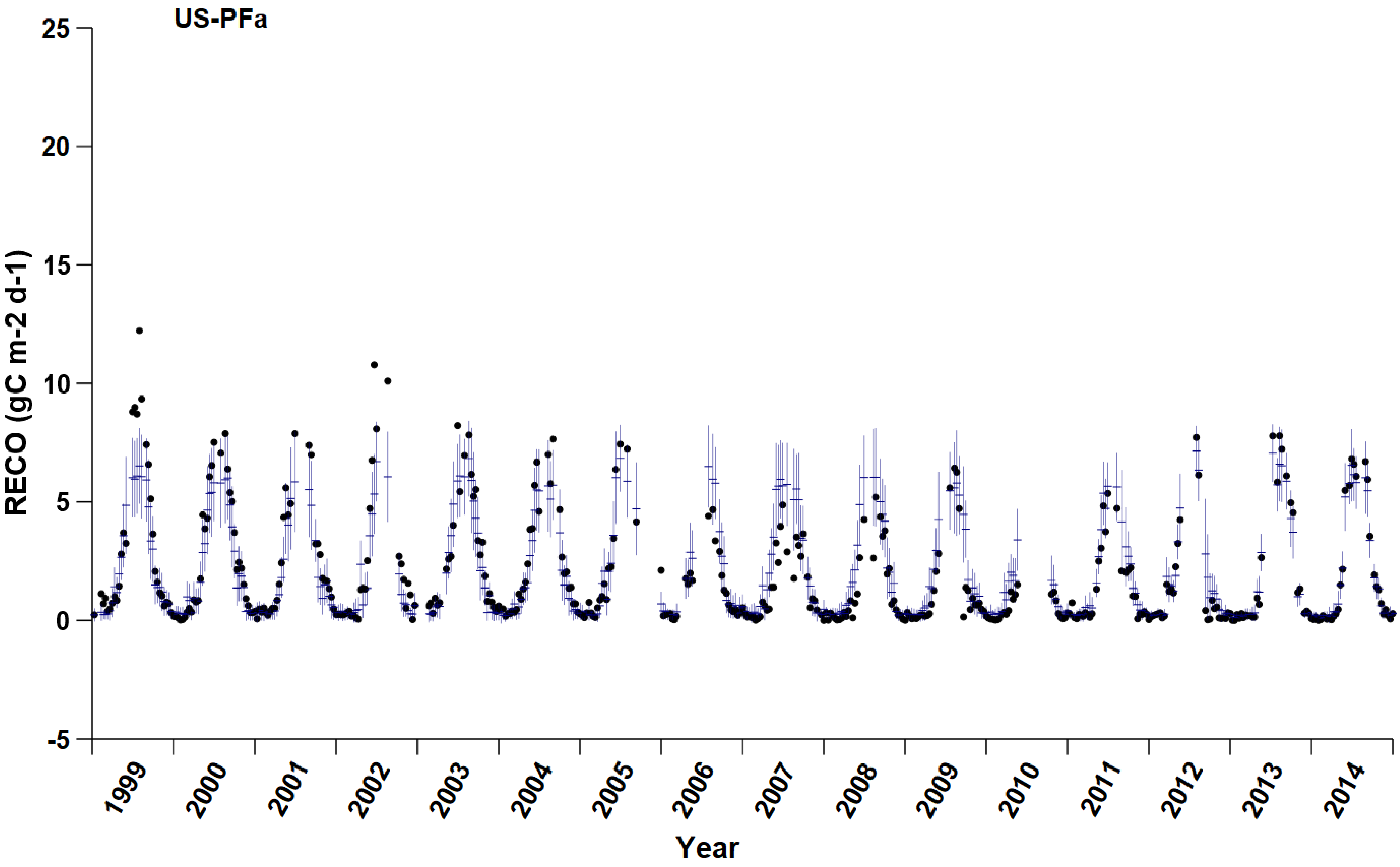
US-PFa
RECO (gC m-2 d-1)
25
20
15
10
5
0
-5
1999
2000
2001
2002
2003
2004
2005
2006
2007
2008
2009
2010
2011
2012
2013
2014
Year

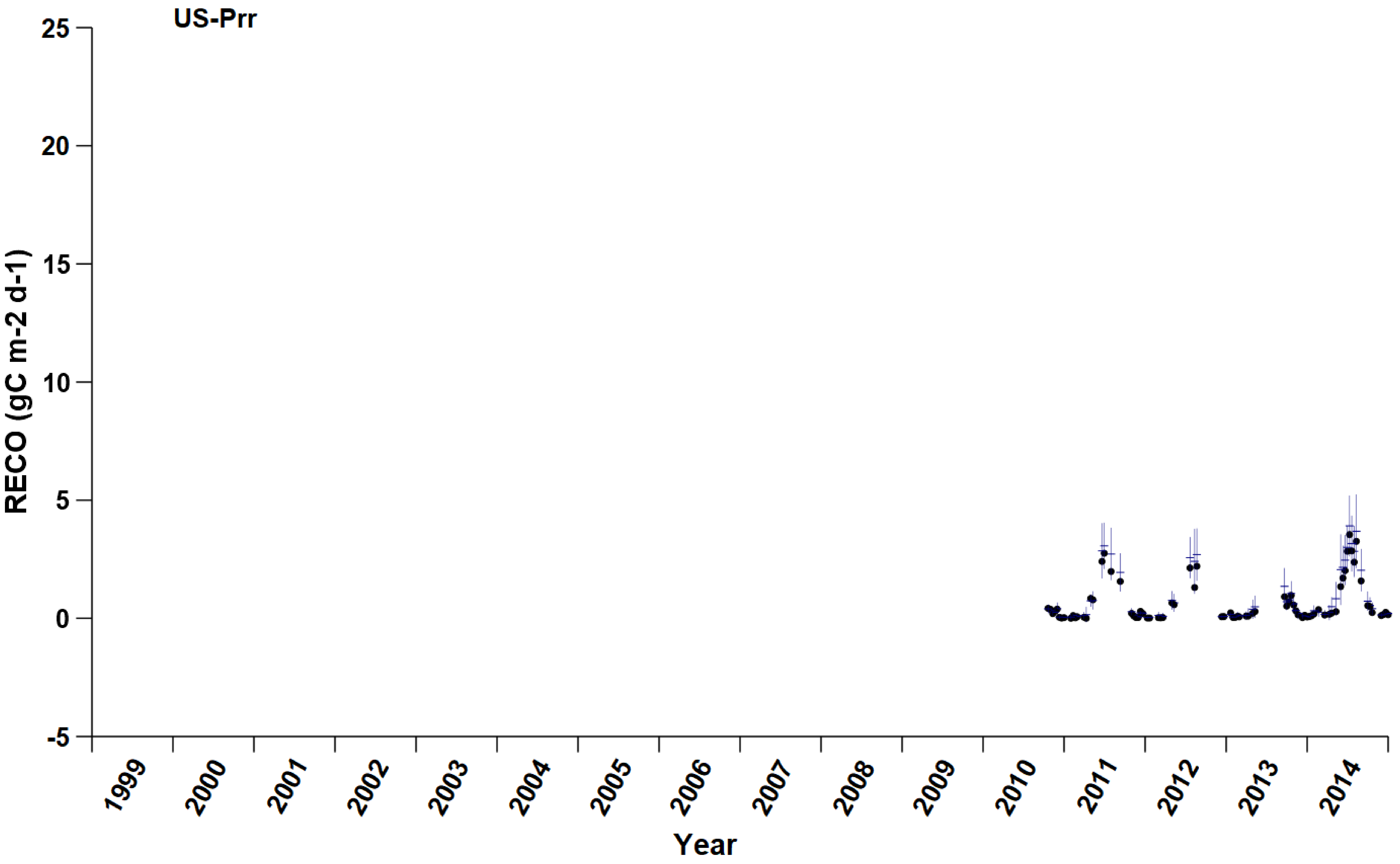
US-Prr
RECO (gC m-2 d-1)
25
20
15
10
5
0
-5
1999
2000
2001
2002
2003
2004
2005
2006
2007
2008
2009
2010
2011
2012
2013
2014
Year

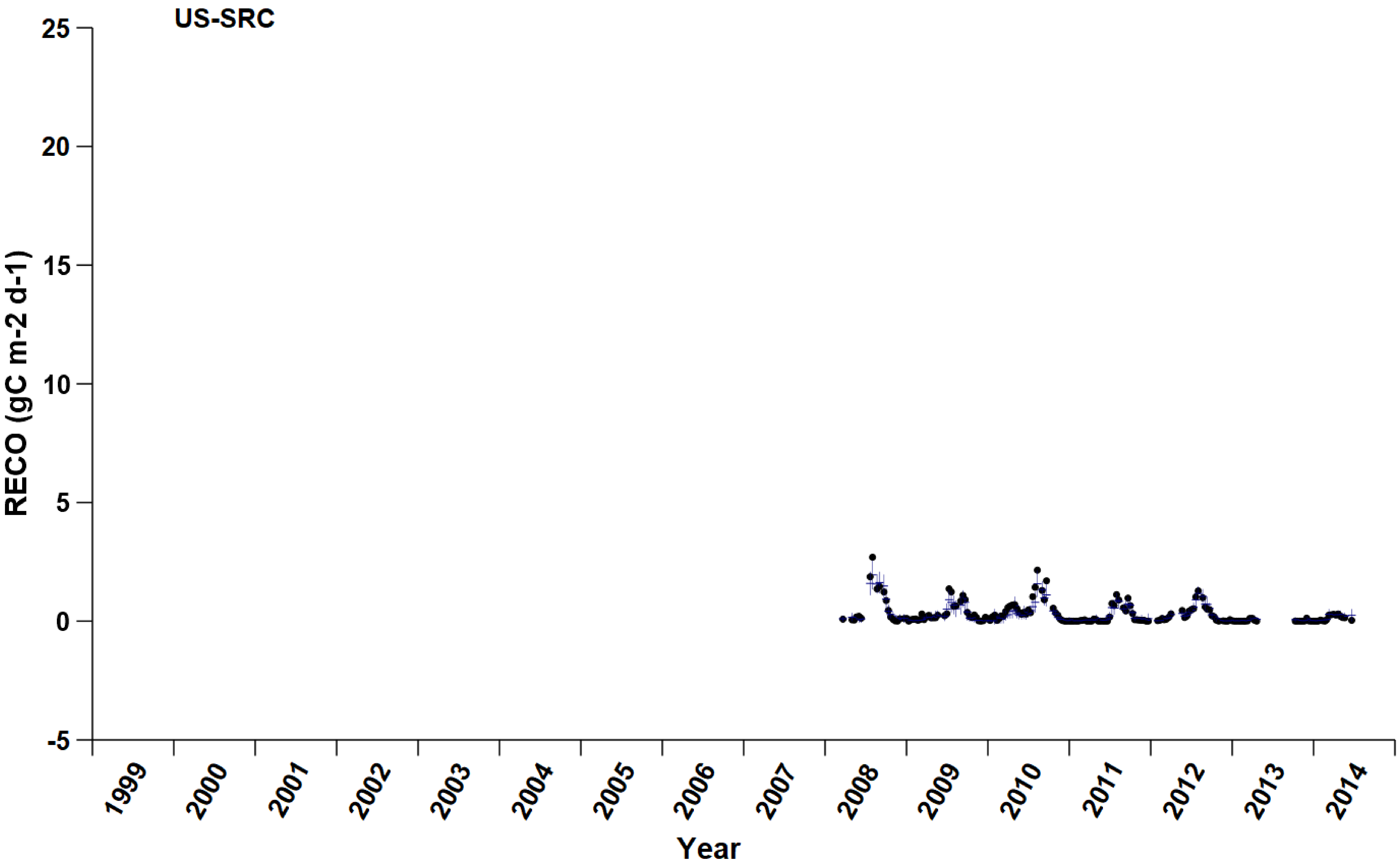
US-SRC
RECO (gC m-2 d-1)
25
20
15
10
5
0
-5
1999
2000
2001
2002
2003
2004
2005
2006
2007
2008
2009
2010
2011
2012
2013
2014
Year

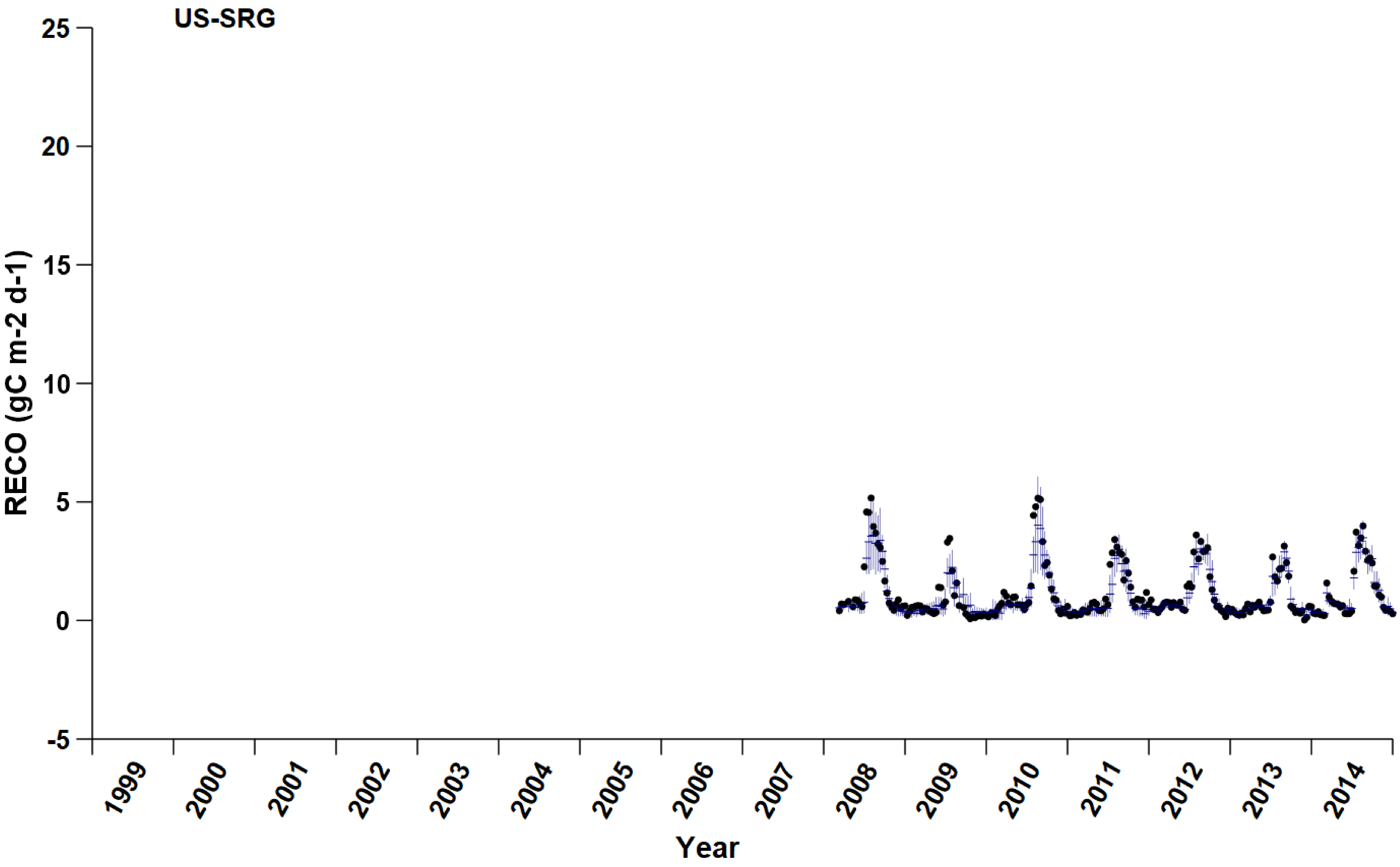

US-SRG
RECO (gC m-2 d-1)
25
20
15
10
5
0
-5
1999
2000
2001
2002
2003
2004
2005
2006
2007
2008
2009
2010
2011
2012
2013
2014
Year

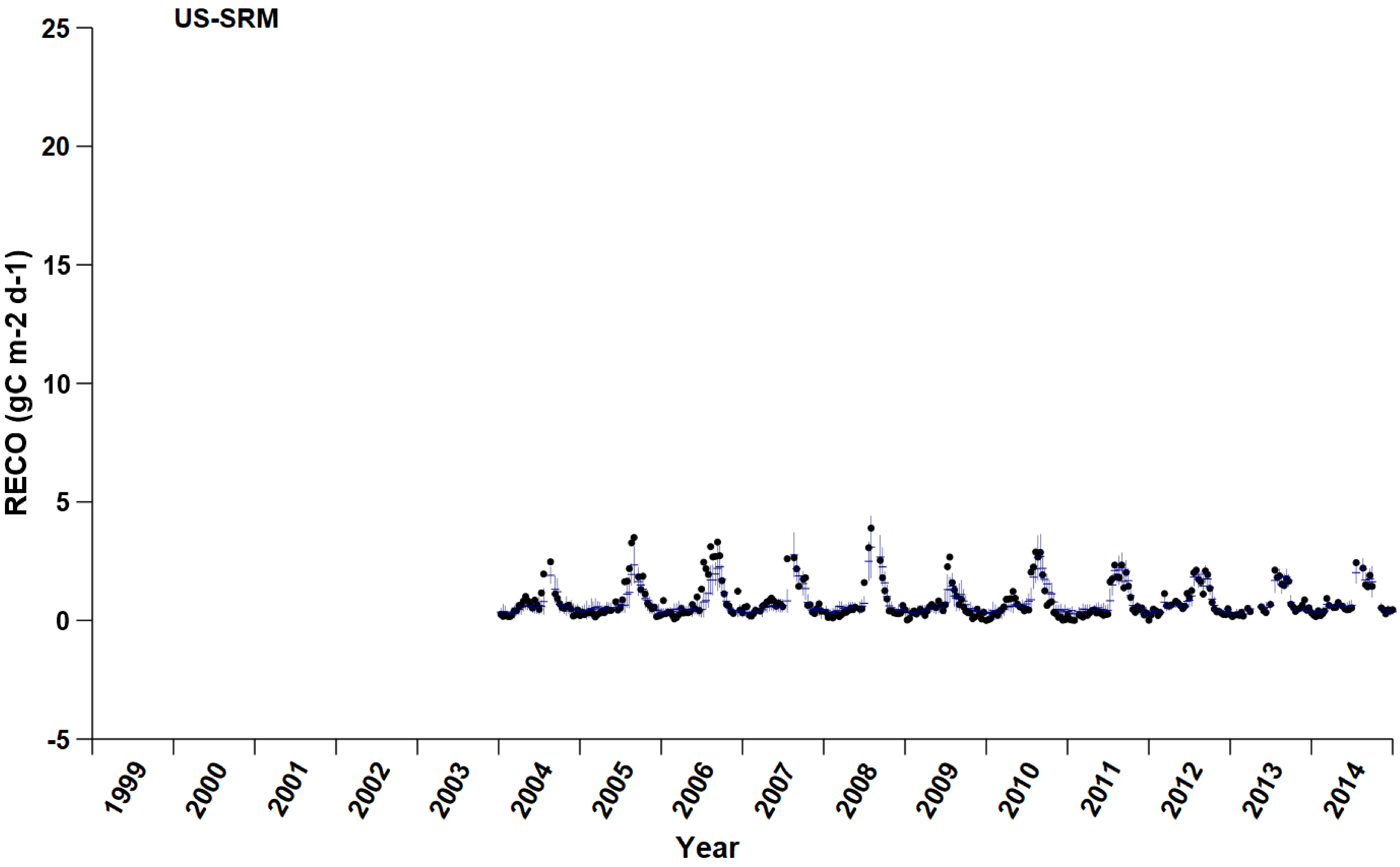
US-SRM
25
20
15
10
5
0
-5
RECO (gC m-2 d-1)
1999
2000
2001
2002
2003
2004
2005
2006
2007
2008
2009
2010
2011
2012
2013
2014
Year

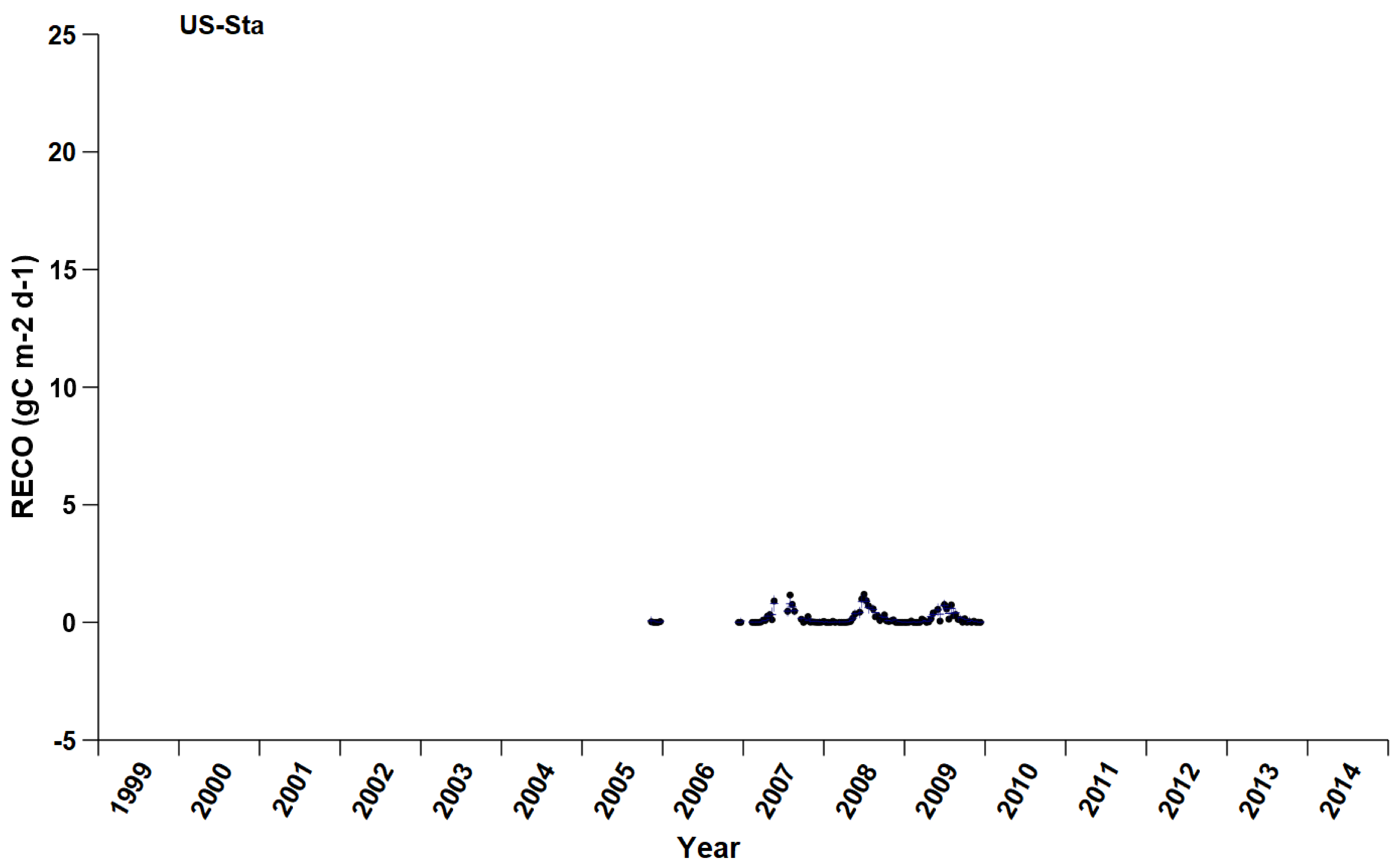

US-Sta
RECO (gC m-2 d-1)
25
20
15
10
5
0
-5
1999
2000
2001
2002
2003
2004
2005
2006
2007
2008
2009
2010
2011
2012
2013
2014
Year

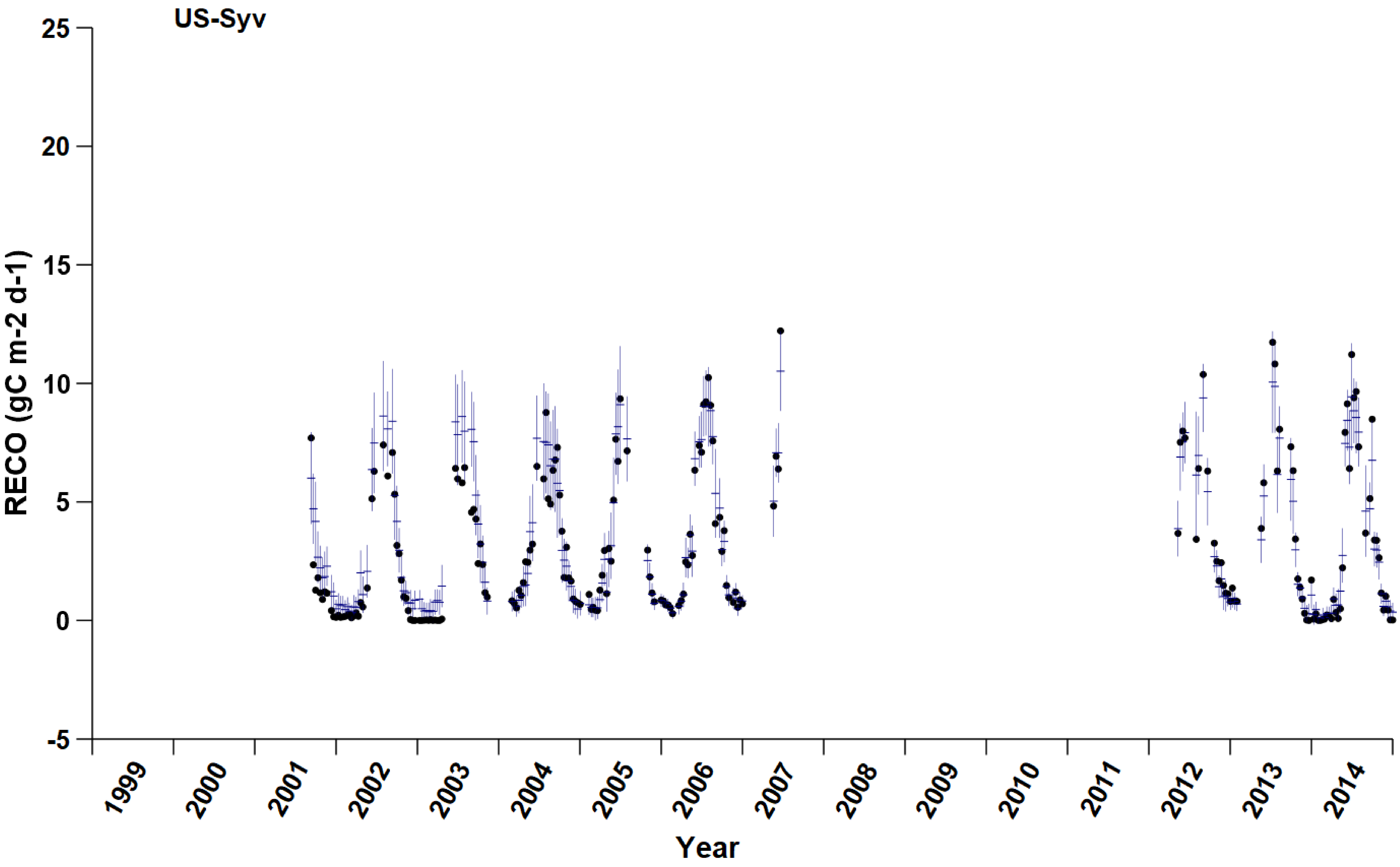

US-Syv
RECO (gC m-2 d-1)
25
20
15
10
5
0
-5
1999
2000
2001
2002
2003
2004
2005
2006
2007
2008
2009
2010
2011
2012
2013
2014
Year

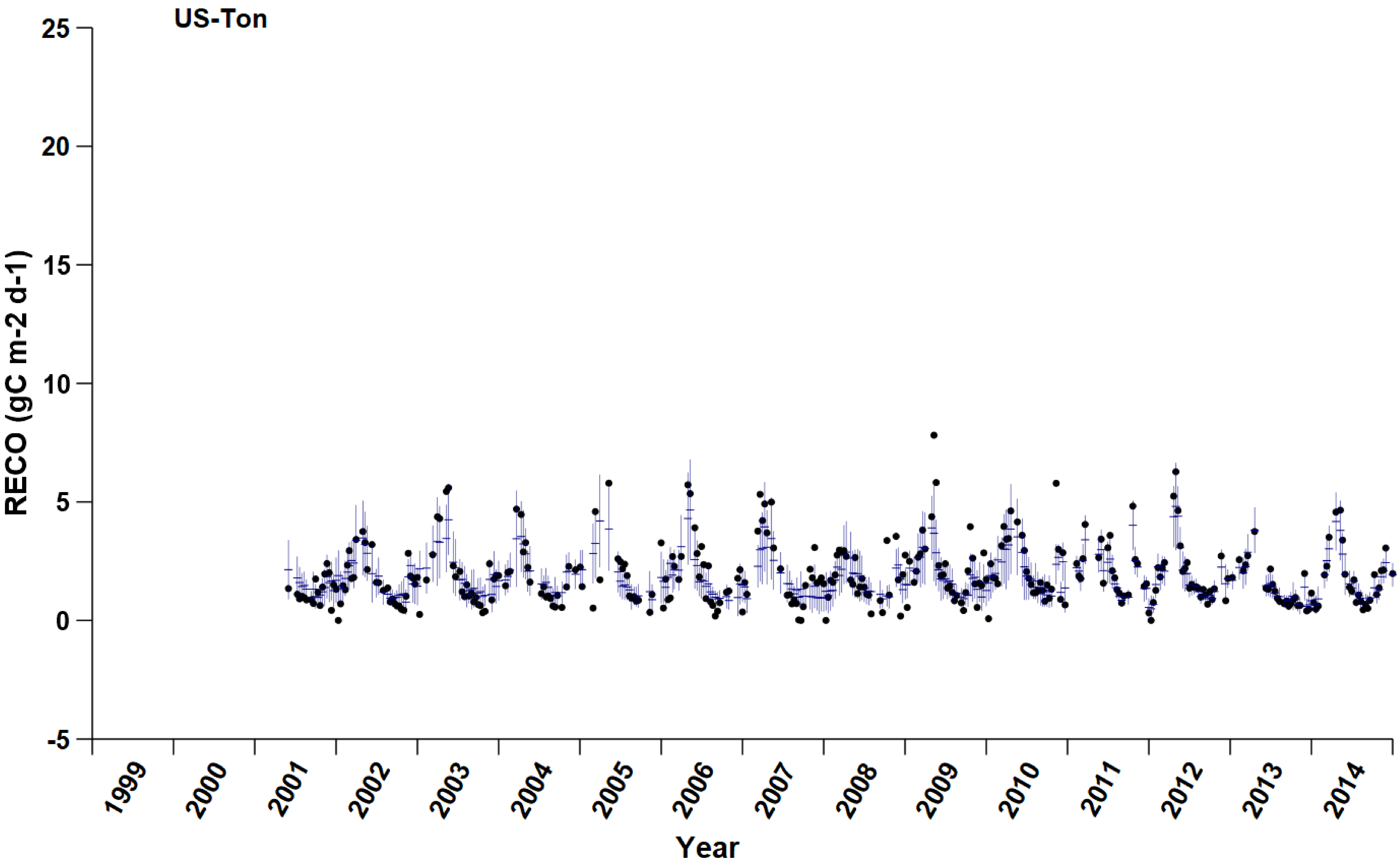

US-Ton
RECO (gC m-2 d-1)
25
20
15
10
5
0
-5
1999
2000
2001
2002
2003
2004
2005
2006
2007
2008
2009
2010
2011
2012
2013
2014
Year

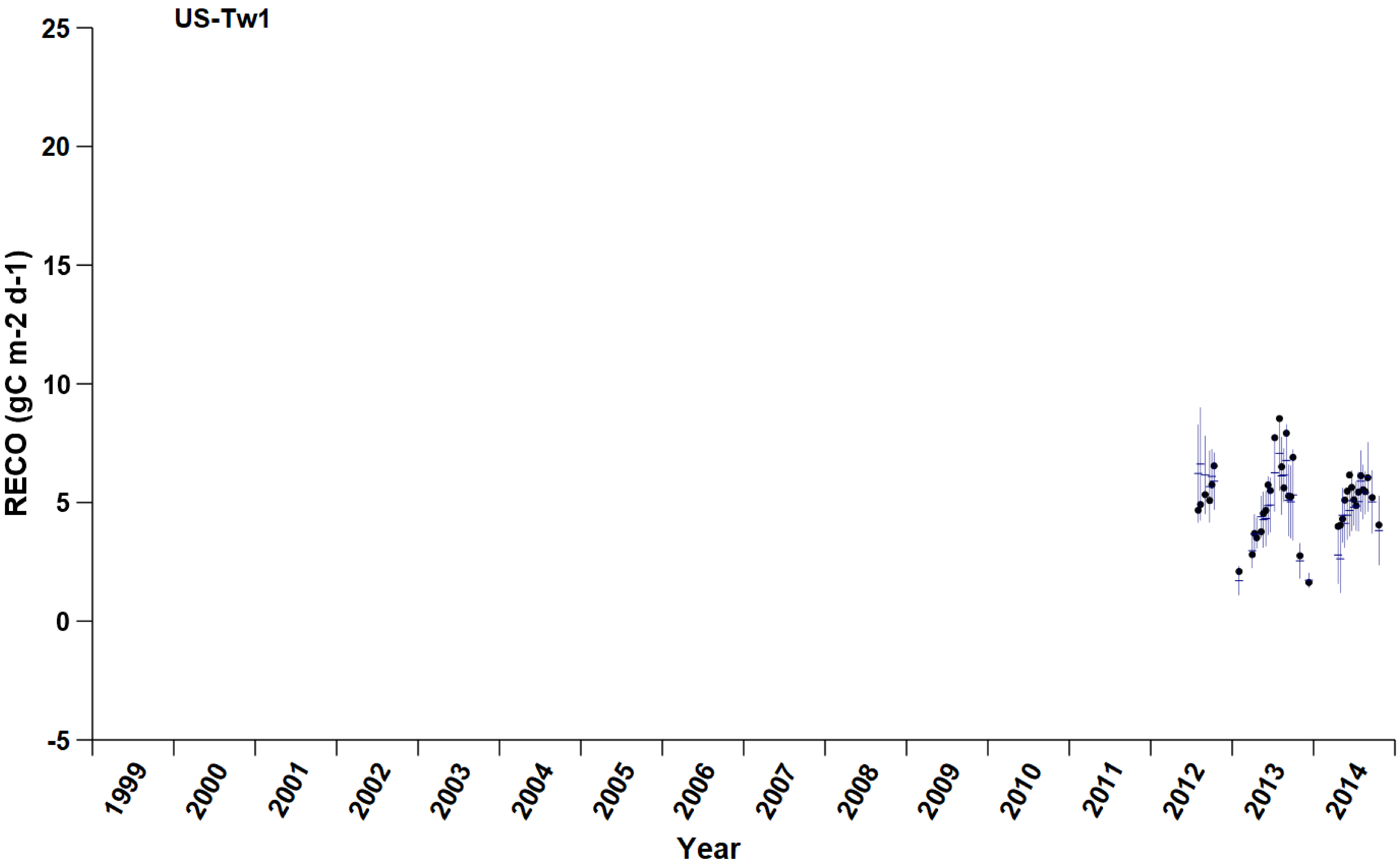

US-Tw1
RECO (gC m-2 d-1)
25
20
15
10
5
0
-5
1999
2000
2001
2002
2003
2004
2005
2006
2007
2008
2009
2010
2011
2012
2013
2014
Year

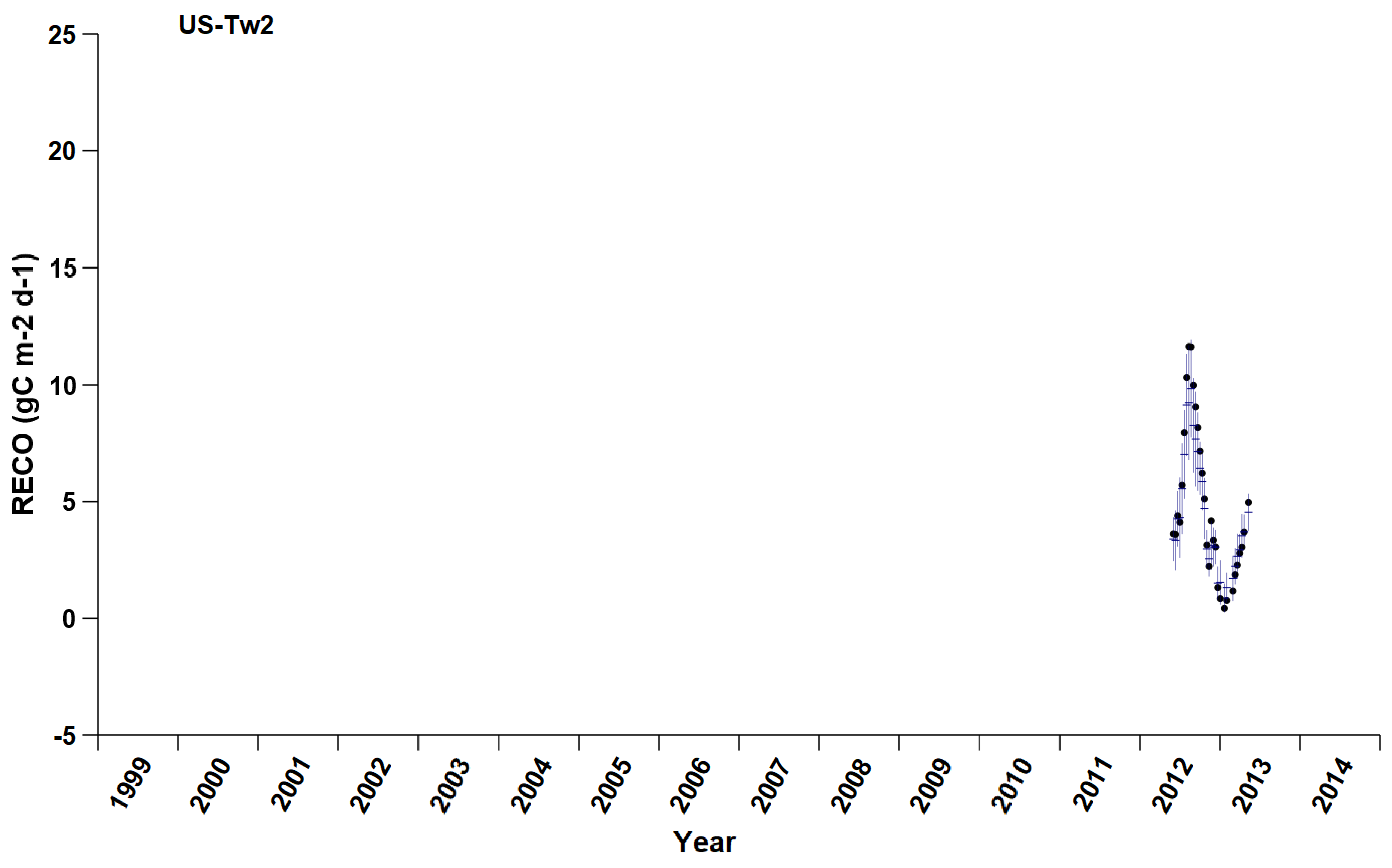
US-Tw2
RECO (gC m-2 d-1)
25
20
15
10
5
0
-5
1999
2000
2001
2002
2003
2004
2005
2006
2007
2008
2009
2010
2011
2012
2013
2014
Year

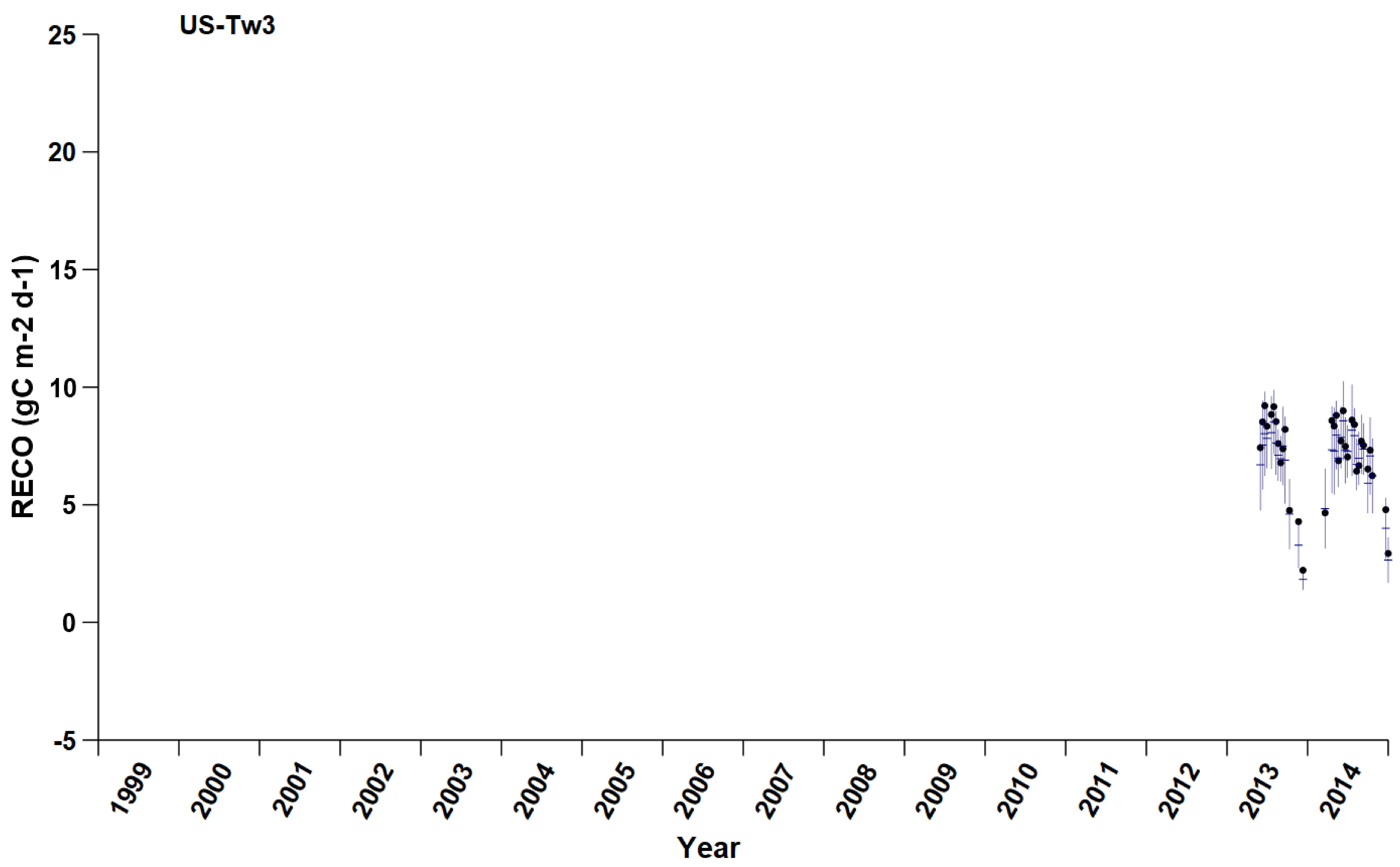

US-Tw3
RECO (gC m-2 d-1)
25
20
15
10
5
0
-5
1999
2000
2001
2002
2003
2004
2005
2006
2007
2008
2009
2010
2011
2012
2013
2014
Year

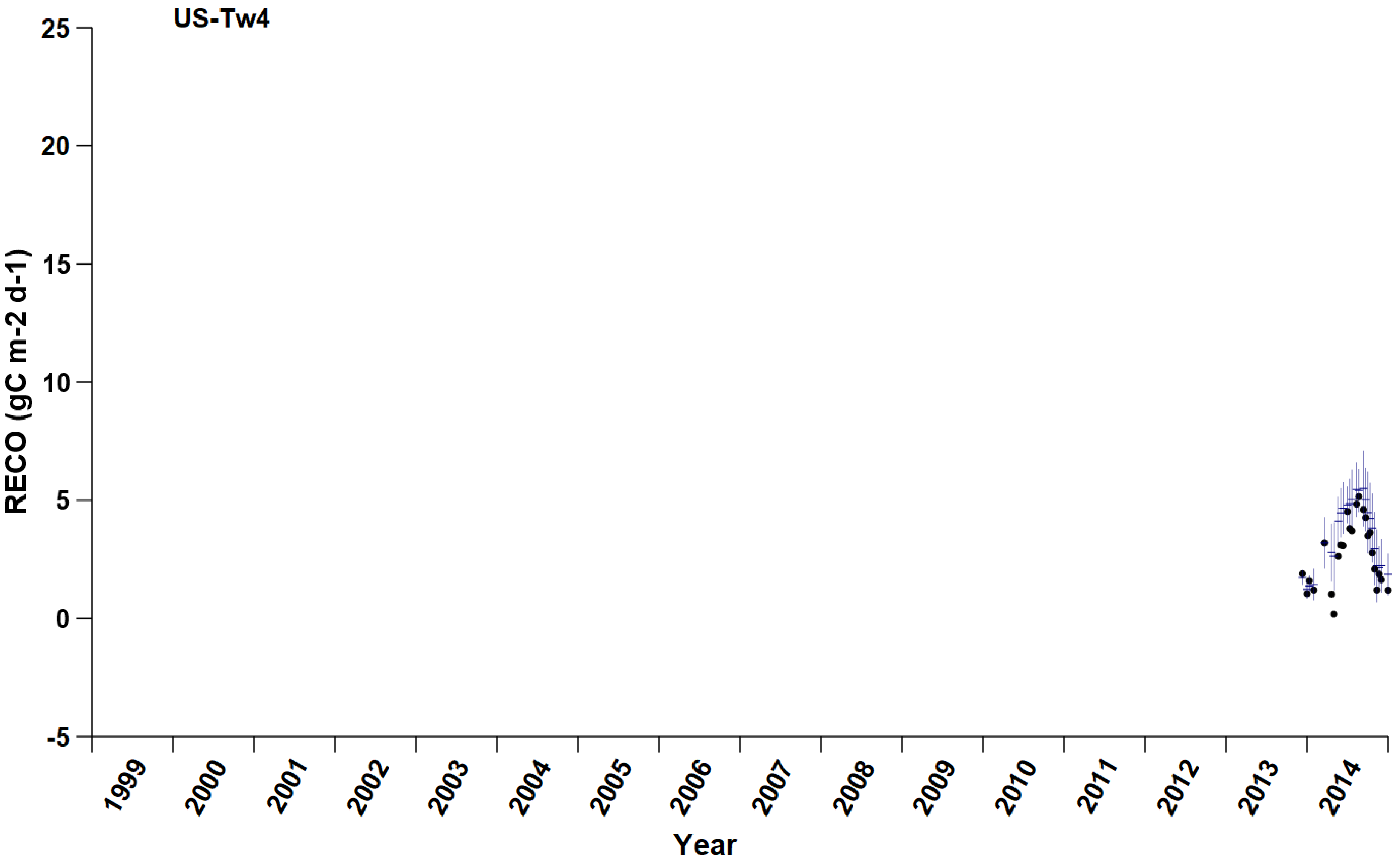
US-Tw4
RECO (gC m-2 d-1)
25
20
15
10
5
0
-5
1999
2000
2001
2002
2003
2004
2005
2006
2007
2008
2009
2010
2011
2012
2013
2014
Year

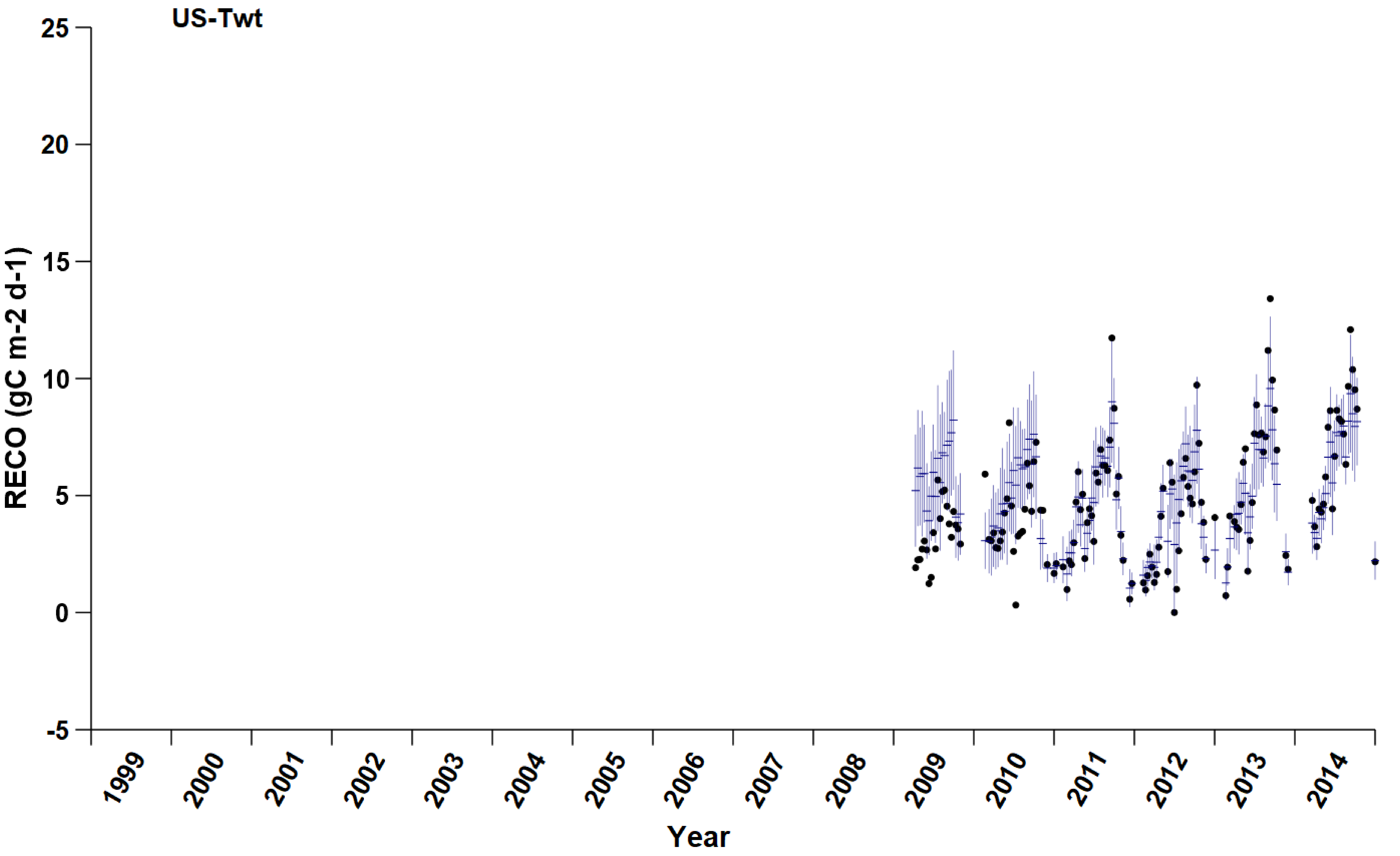

US-Twt
RECO (gC m-2 d-1)
25
20
15
10
5
0
-5
1999
2000
2001
2002
2003
2004
2005
2006
2007
2008
2009
2010
2011
2012
2013
2014
Year

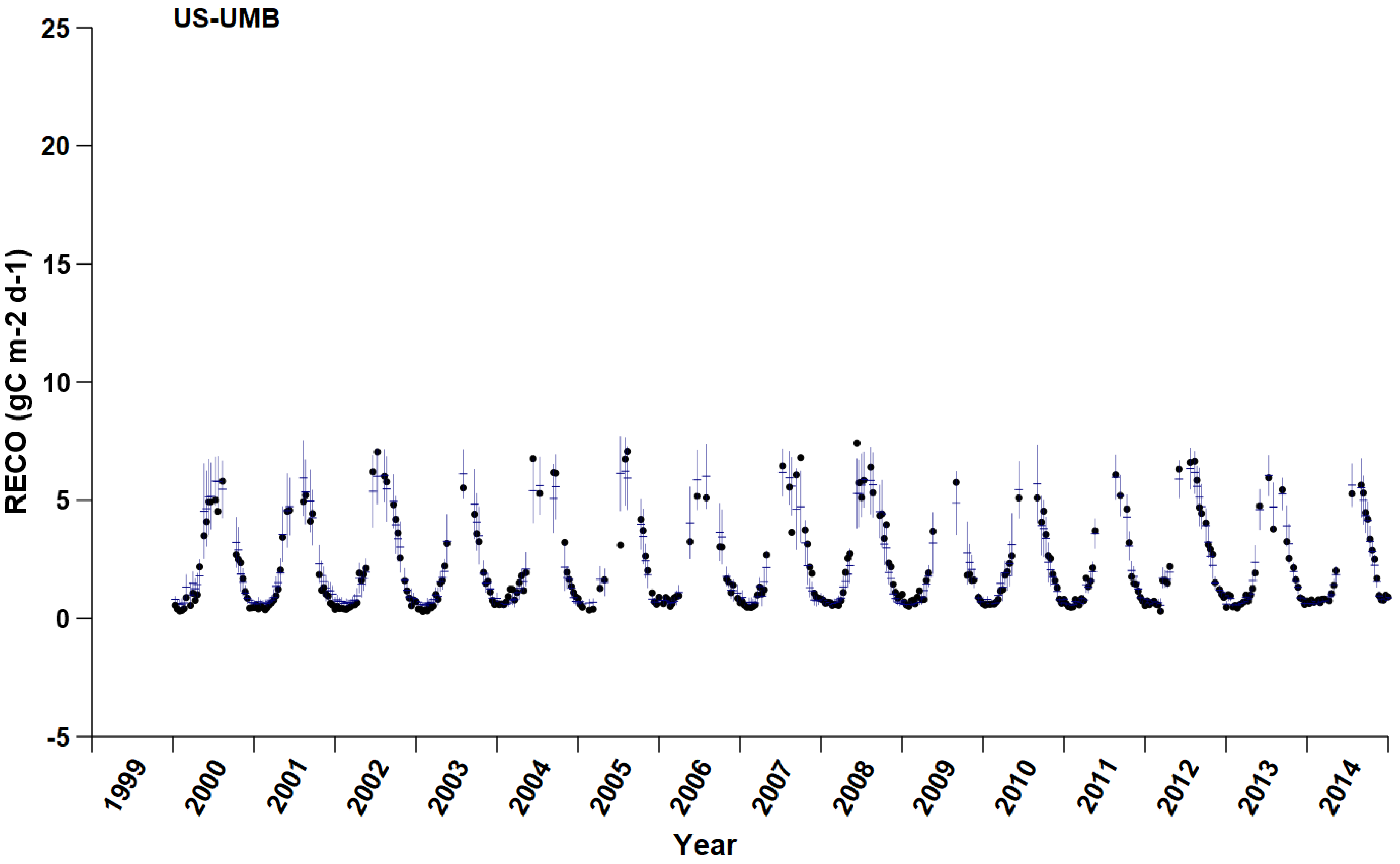
US-UMB
RECO (gC m-2 d-1)
25
20
15
10
5
0
-5
1999
2000
2001
2002
2003
2004
2005
2006
2007
2008
2009
2010
2011
2012
2013
2014
Year

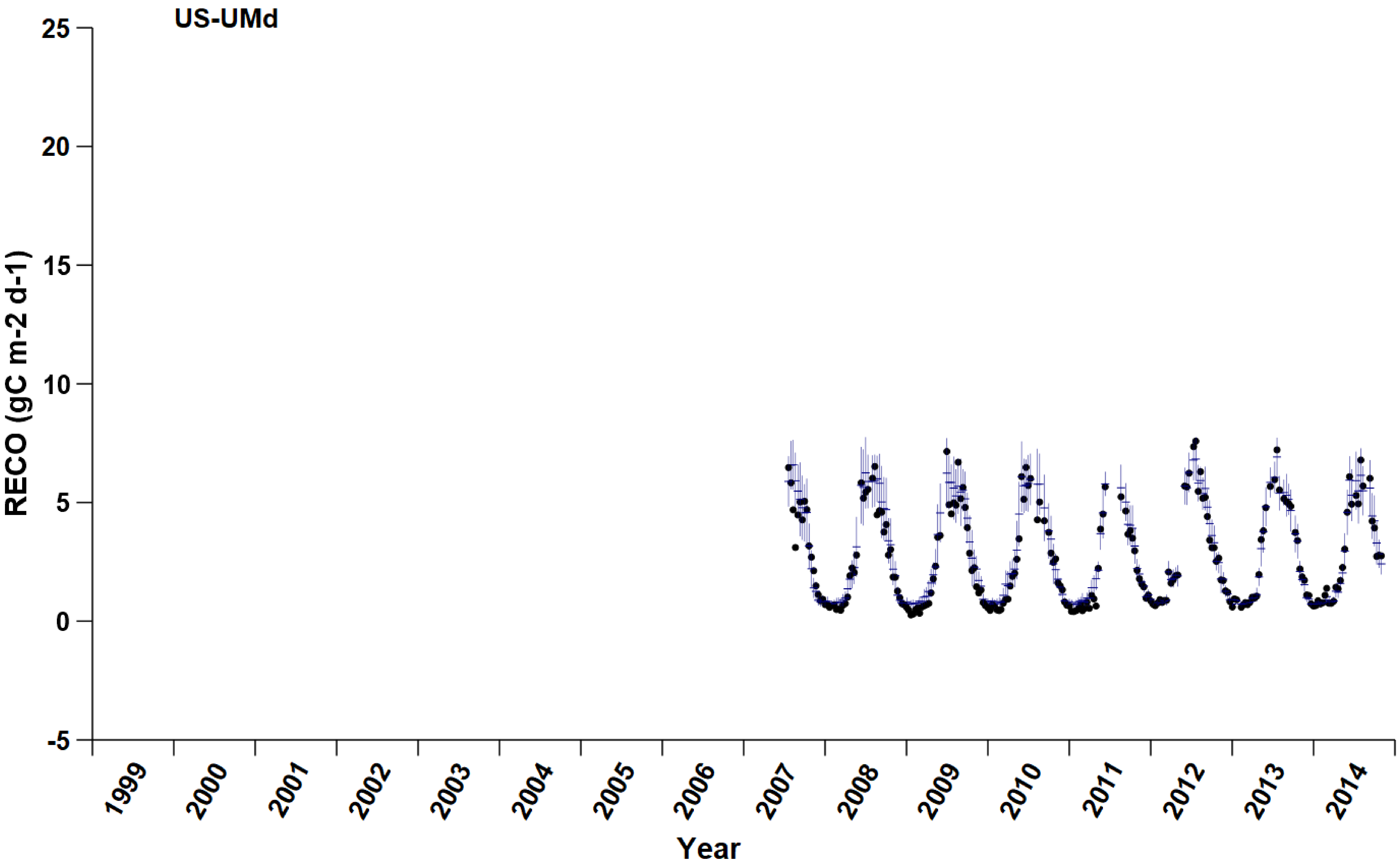

US-UMd
RECO (gC m-2 d-1)
25
20
15
10
5
0
-5
1999
2000
2001
2002
2003
2004
2005
2006
2007
2008
2009
2010
2011
2012
2013
2014
Year

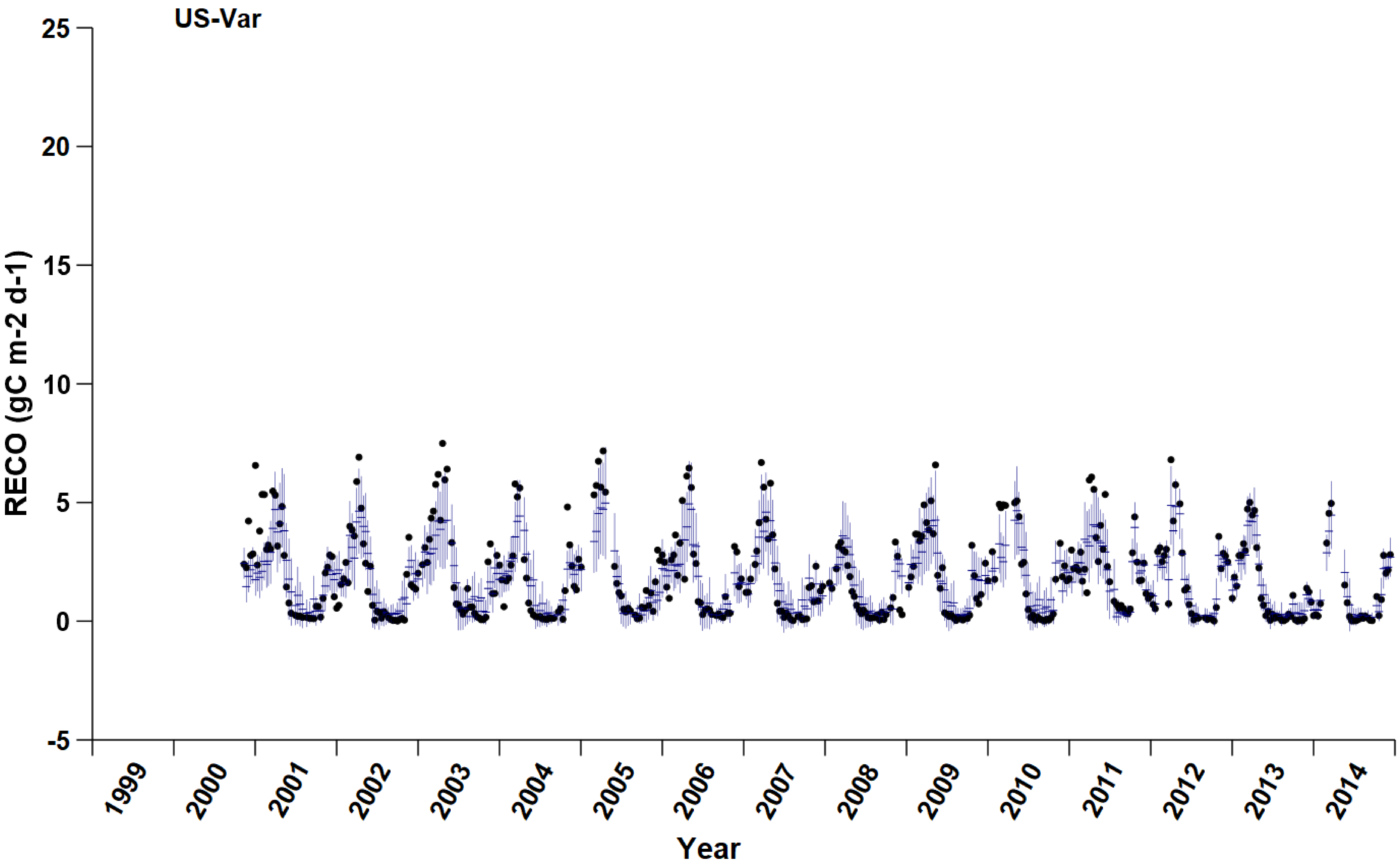
US-Var
RECO (gC m-2 d-1)
25
20
15
10
5
0
-5
1999
2000
2001
2002
2003
2004
2005
2006
2007
2008
2009
2010
2011
2012
2013
2014
Year

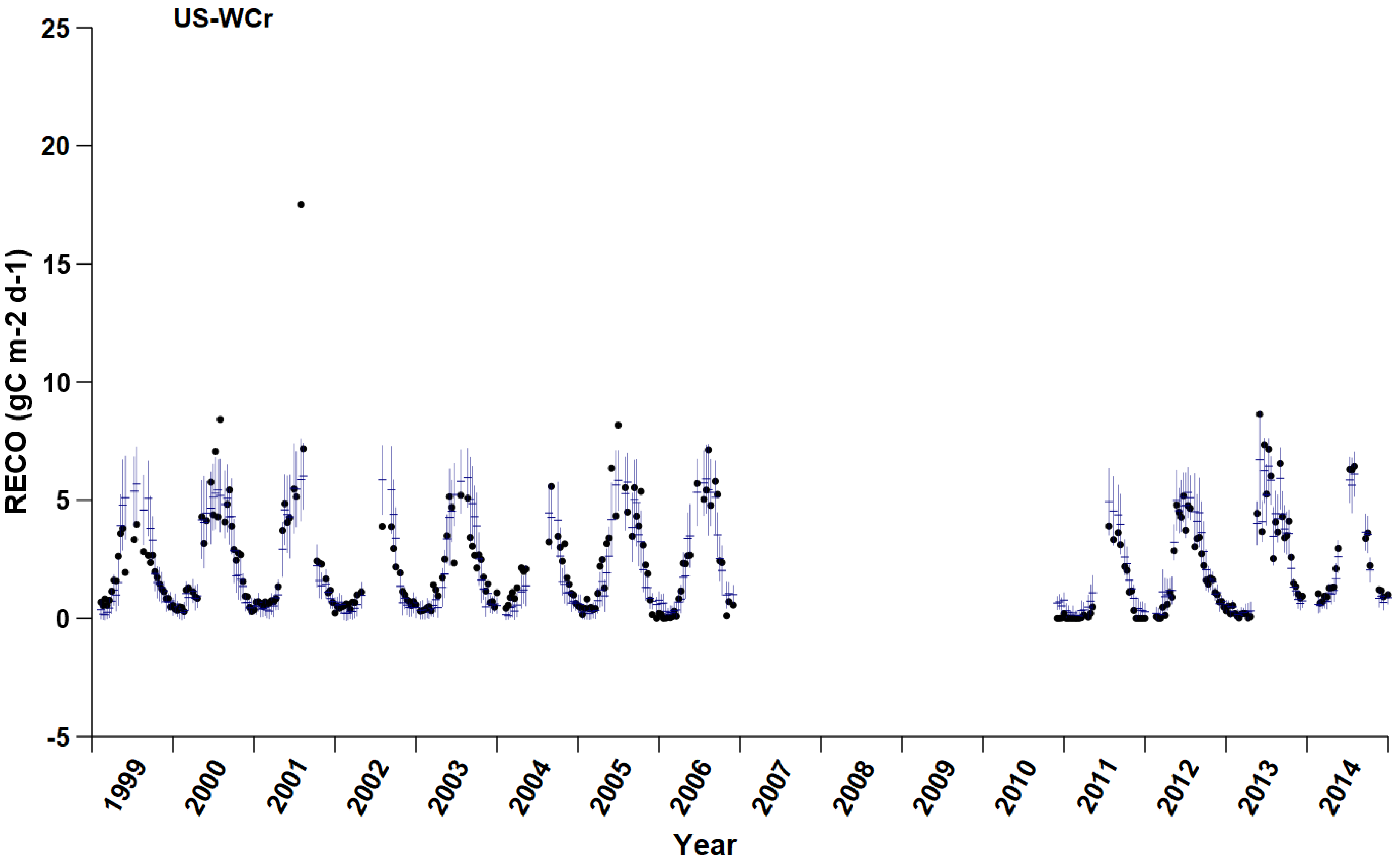
US-WCr
RECO (gC m-2 d-1)
25
20
15
10
5
0
-5
1999
2000
2001
2002
2003
2004
2005
2006
2007
2008
2009
2010
2011
2012
2013
2014
Year

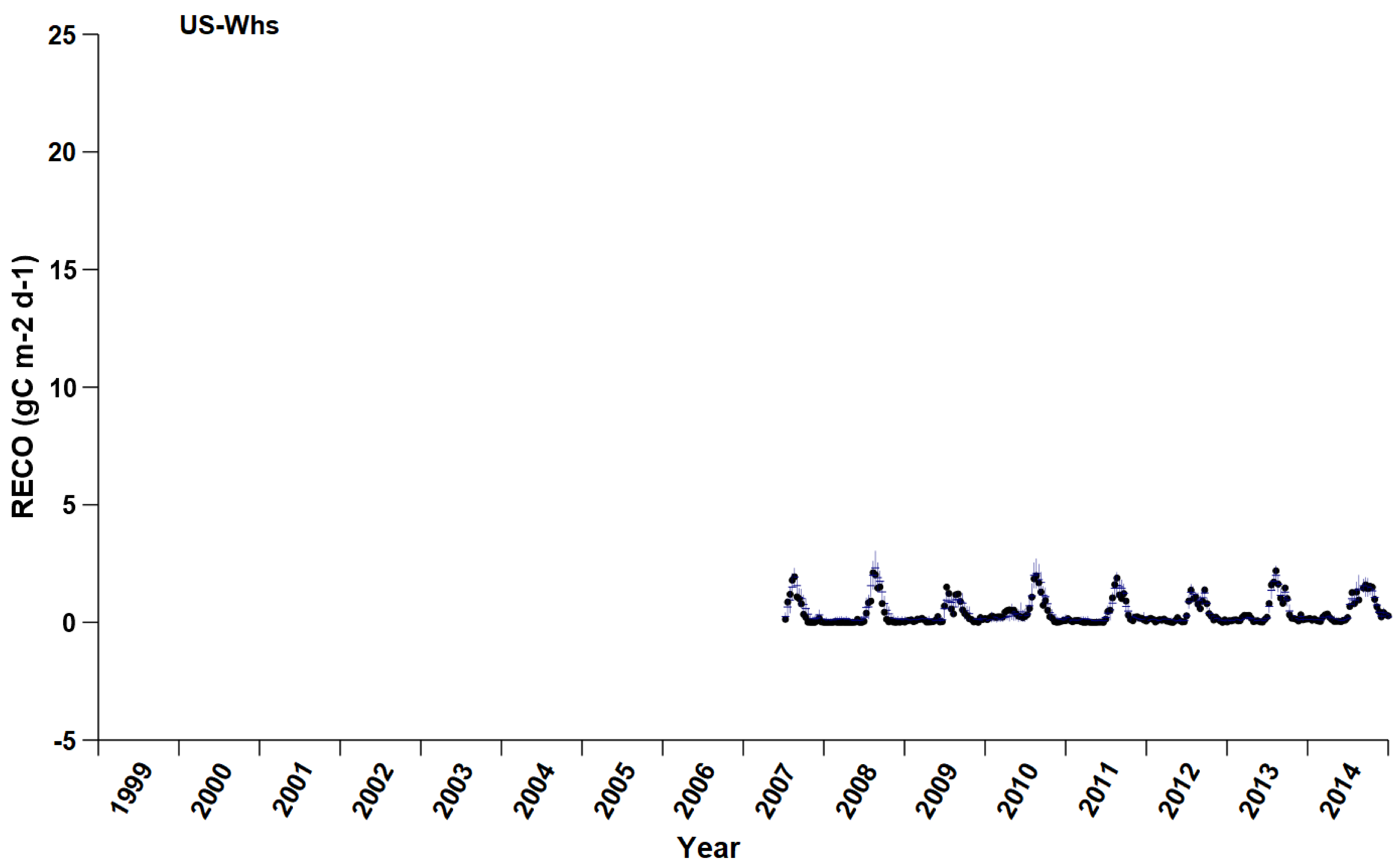
US-Whs
RECO (gC m-2 d-1)
25
20
15
10
5
0
-5
1999
2000
2001
2002
2003
2004
2005
2006
2007
2008
2009
2010
2011
2012
2013
2014
Year

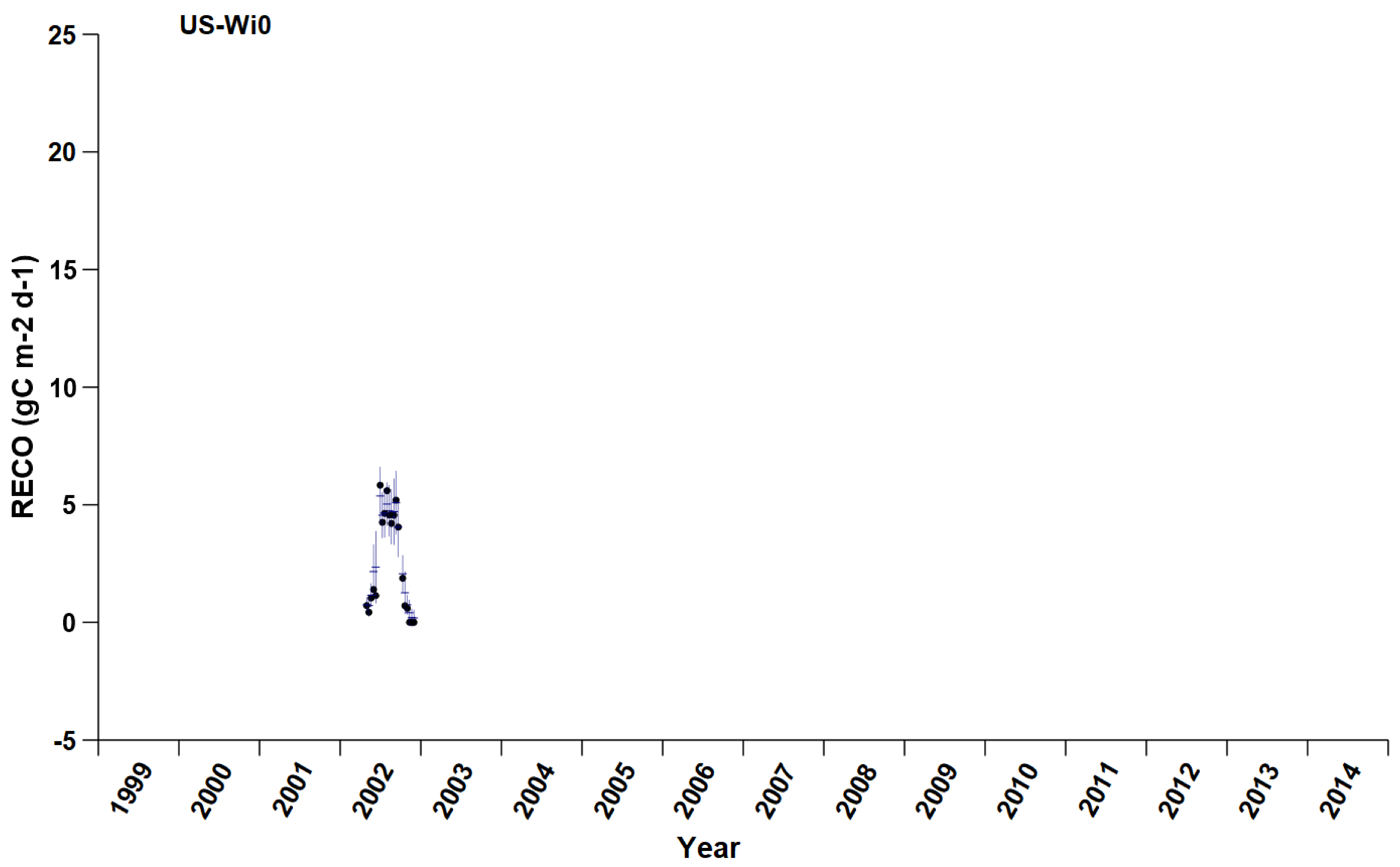

US-Wi0
RECO (gC m-2 d-1)
25
20
15
10
5
0
-5
1999
2000
2001
2002
2003
2004
2005
2006
2007
2008
2009
2010
2011
2012
2013
2014
Year

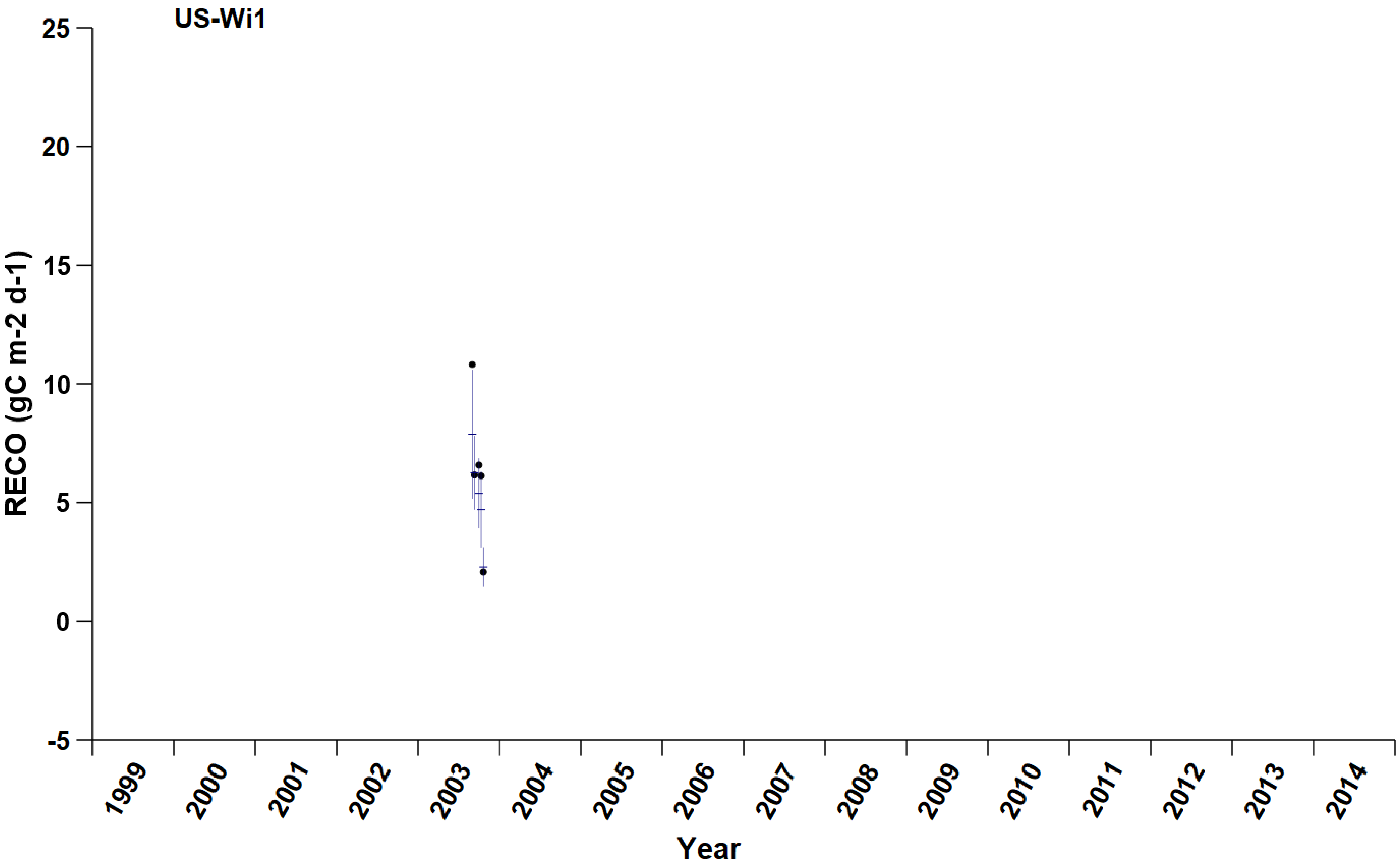

US-Wi1
RECO (gC m-2 d-1)
25
20
15
10
5
0
-5
1999
2000
2001
2002
2003
2004
2005
2006
2007
2008
2009
2010
2011
2012
2013
2014
Year

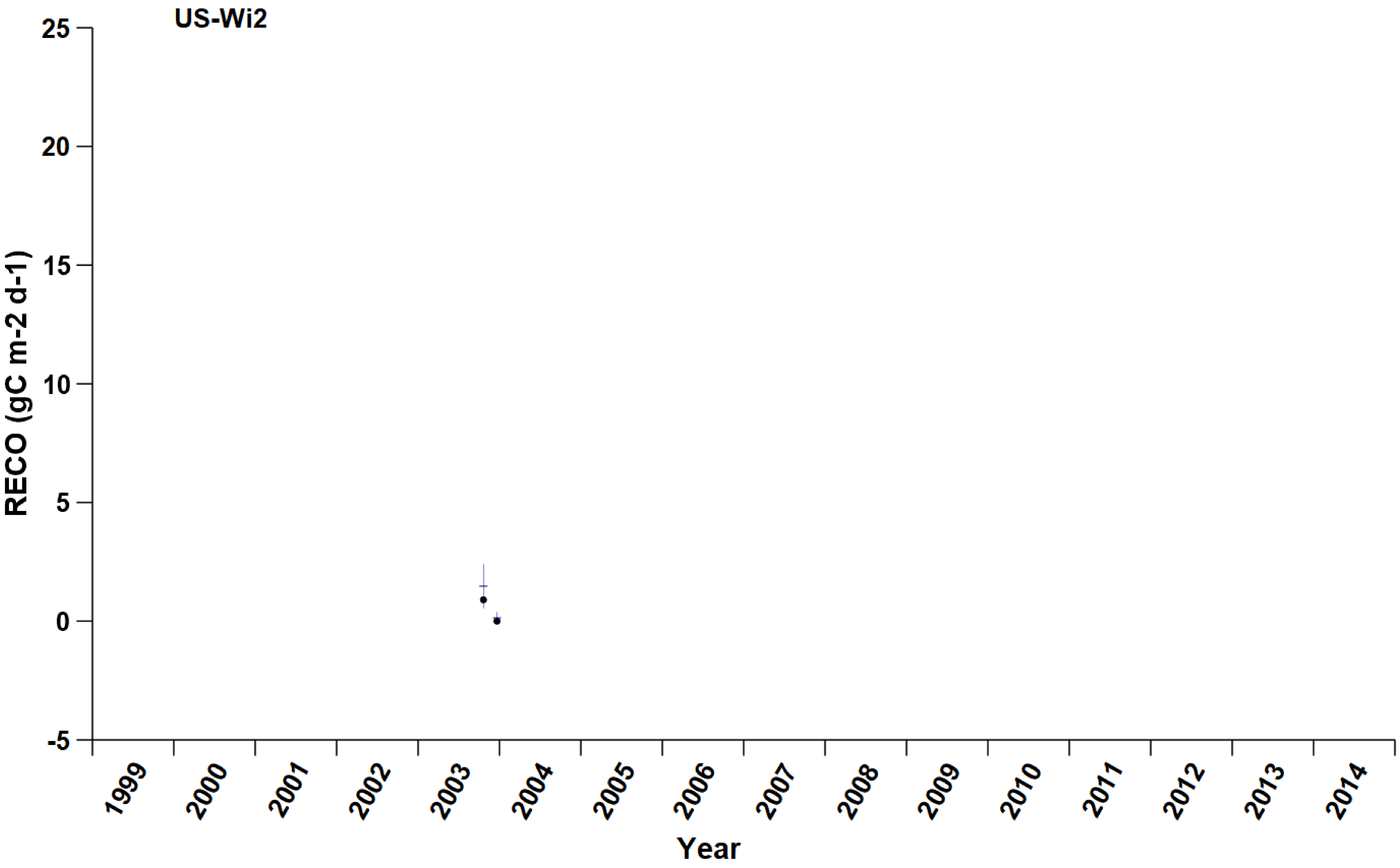
US-Wi2
25
20
15
10
5
0
-5
RECO (gC m-2 d-1)
1999
2000
2001
2002
2003
2004
2005
2006
2007
2008
2009
2010
2011
2012
2013
2014
Year

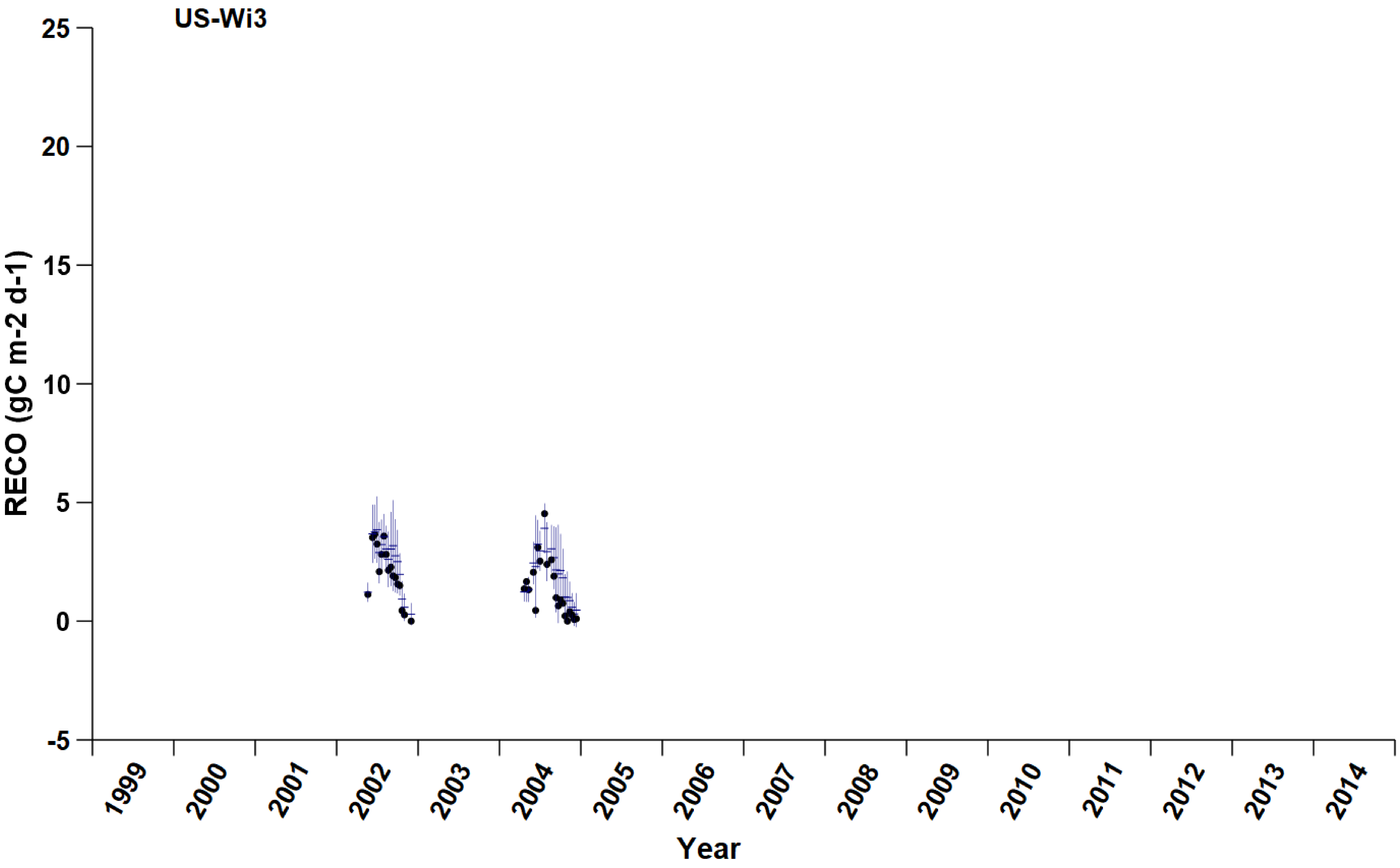

US-Wi3
RECO (gC m-2 d-1)
25
20
15
10
5
0
-5
1999
2000
2001
2002
2003
2004
2005
2006
2007
2008
2009
2010
2011
2012
2013
2014
Year

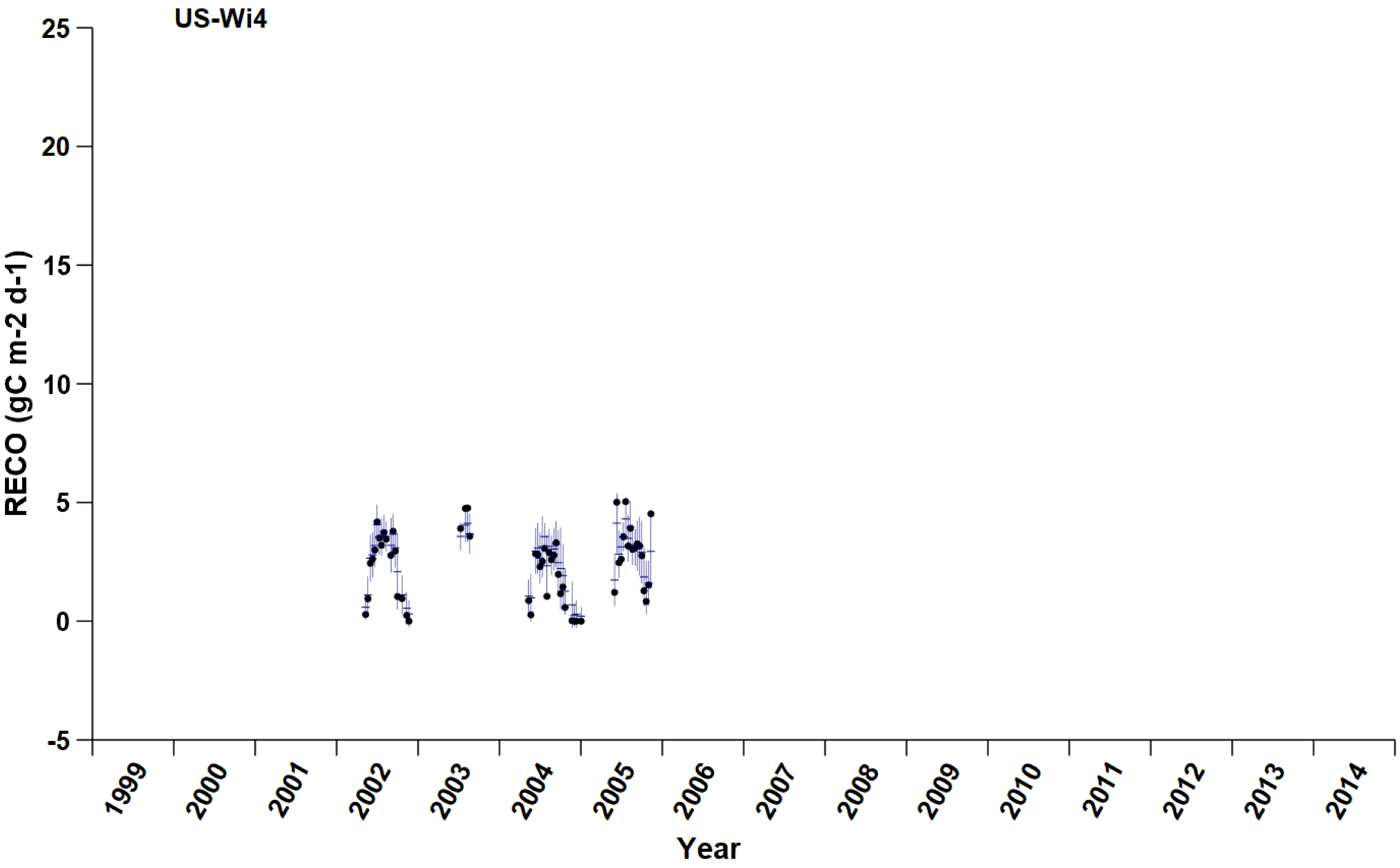
US-Wi4
RECO (gC m-2 d-1)
25
20
15
10
5
0
-5
1999
2000
2001
2002
2003
2004
2005
2006
2007
2008
2009
2010
2011
2012
2013
2014
Year

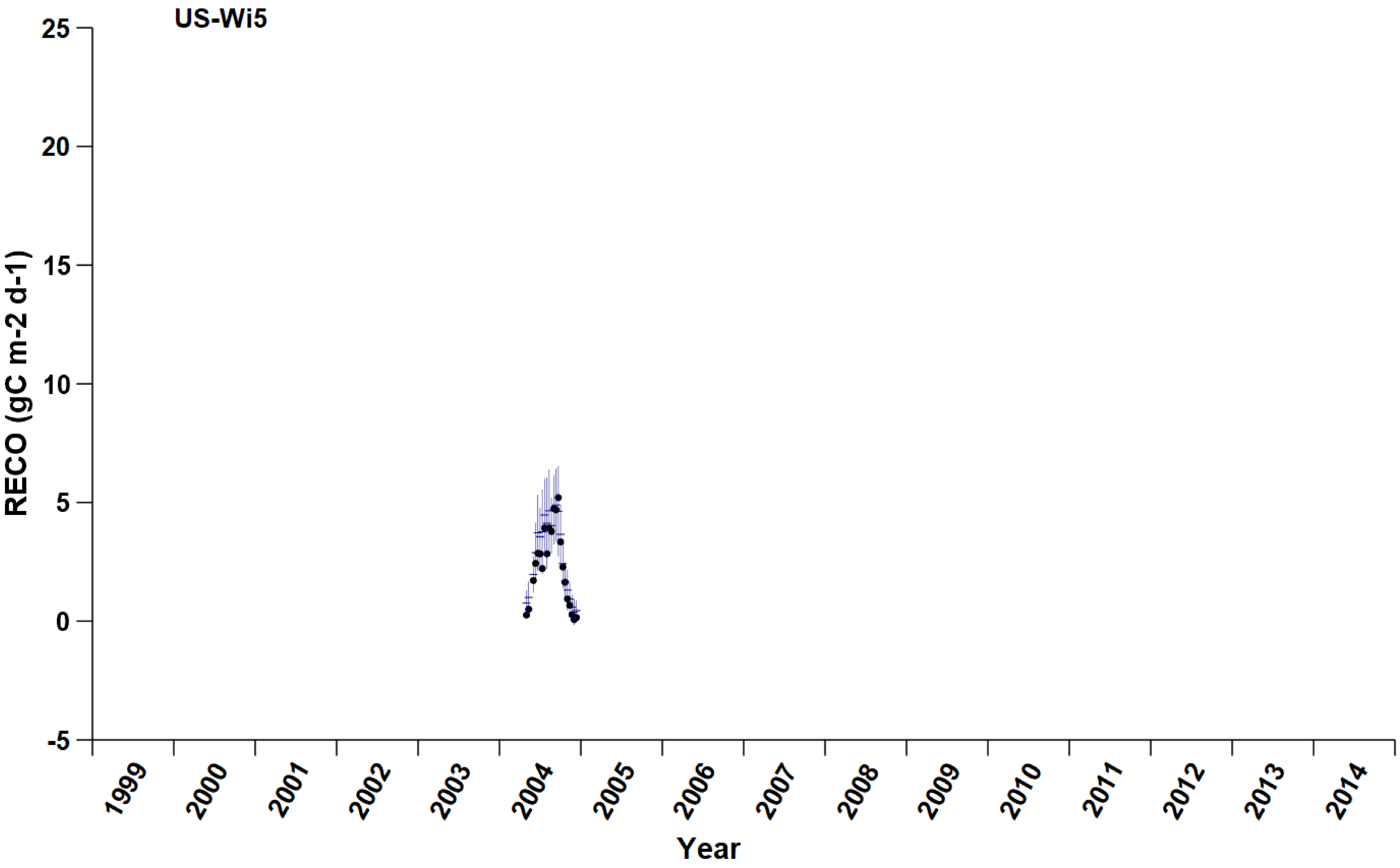
US-Wi5
RECO (gC m-2 d-1)
25
20
15
10
5
0
-5
1999
2000
2001
2002
2003
2004
2005
2006
2007
2008
2009
2010
2011
2012
2013
2014
Year

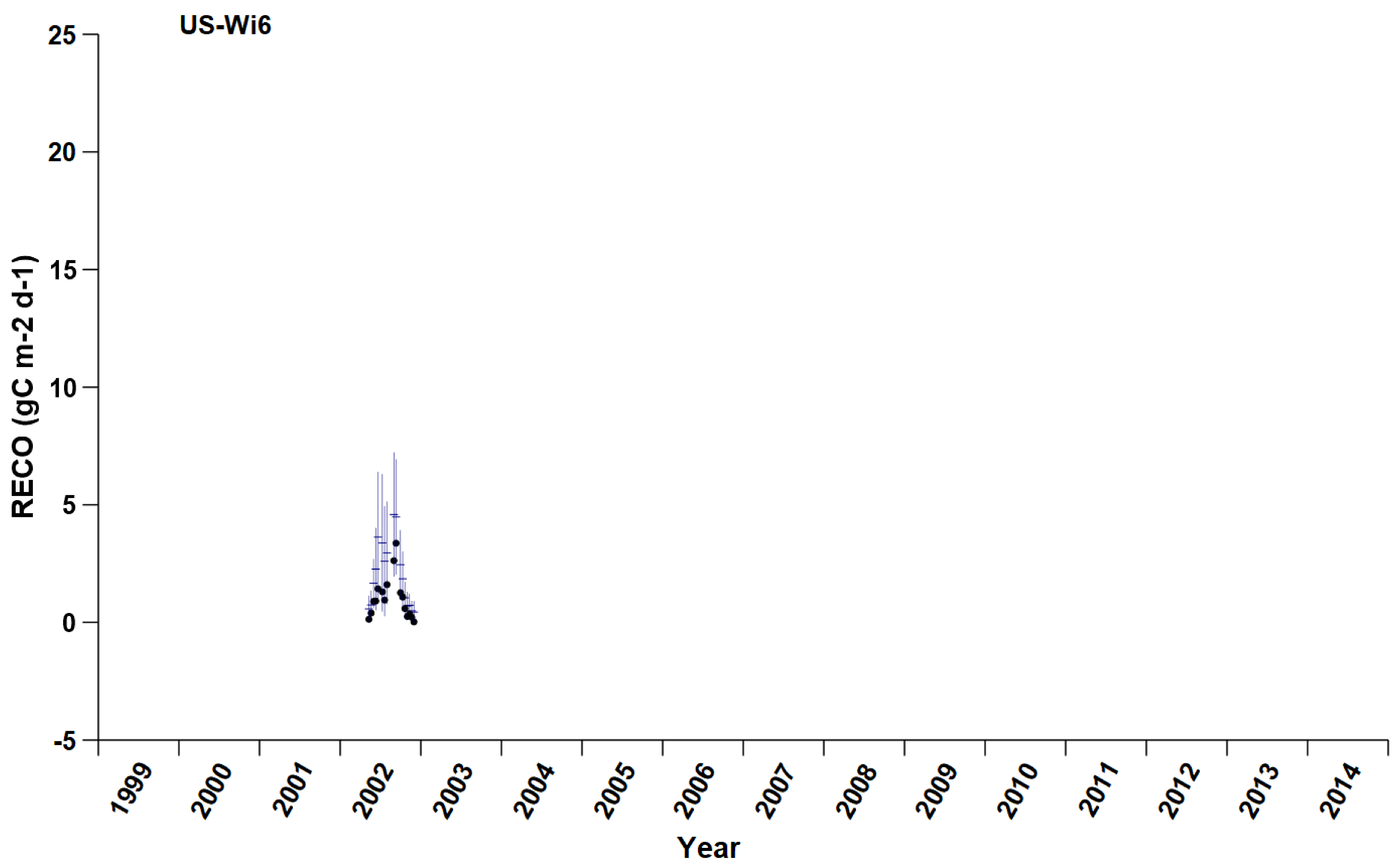

US-Wi6
RECO (gC m-2 d-1)
25
20
15
10
5
0
-5
1999
2000
2001
2002
2003
2004
2005
2006
2007
2008
2009
2010
2011
2012
2013
2014
Year

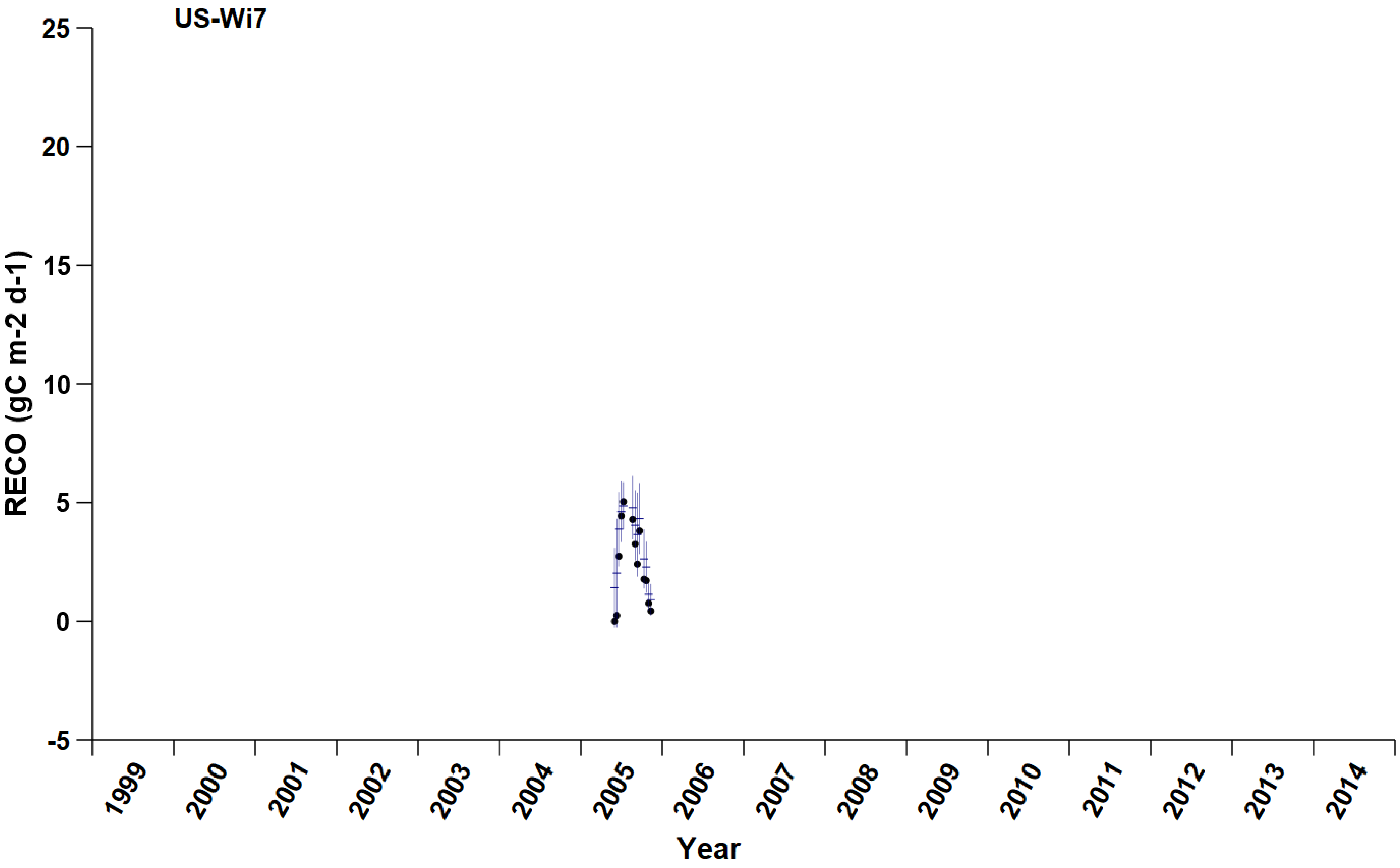
US-Wi7
25
20
15
10
5
0
-5
RECO (gC m-2 d-1)
1999
2000
2001
2002
2003
2004
2005
2006
2007
2008
2009
2010
2011
2012
2013
2014
Year

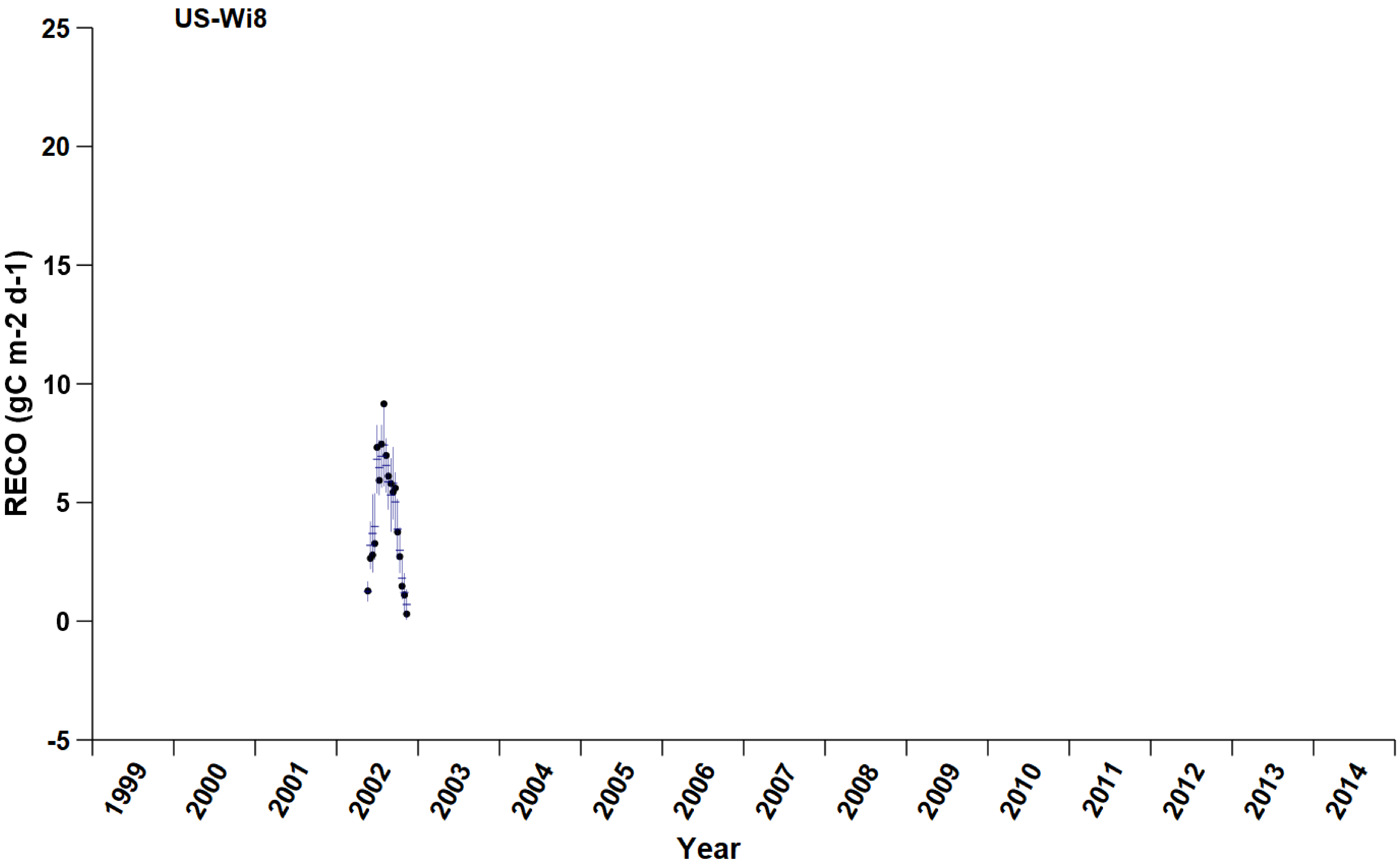

US-Wi8
25
20
15
10
5
0
-5
RECO (gC m-2 d-1)
1999
2000
2001
2002
2003
2004
2005
2006
2007
2008
2009
2010
2011
2012
2013
2014
Year

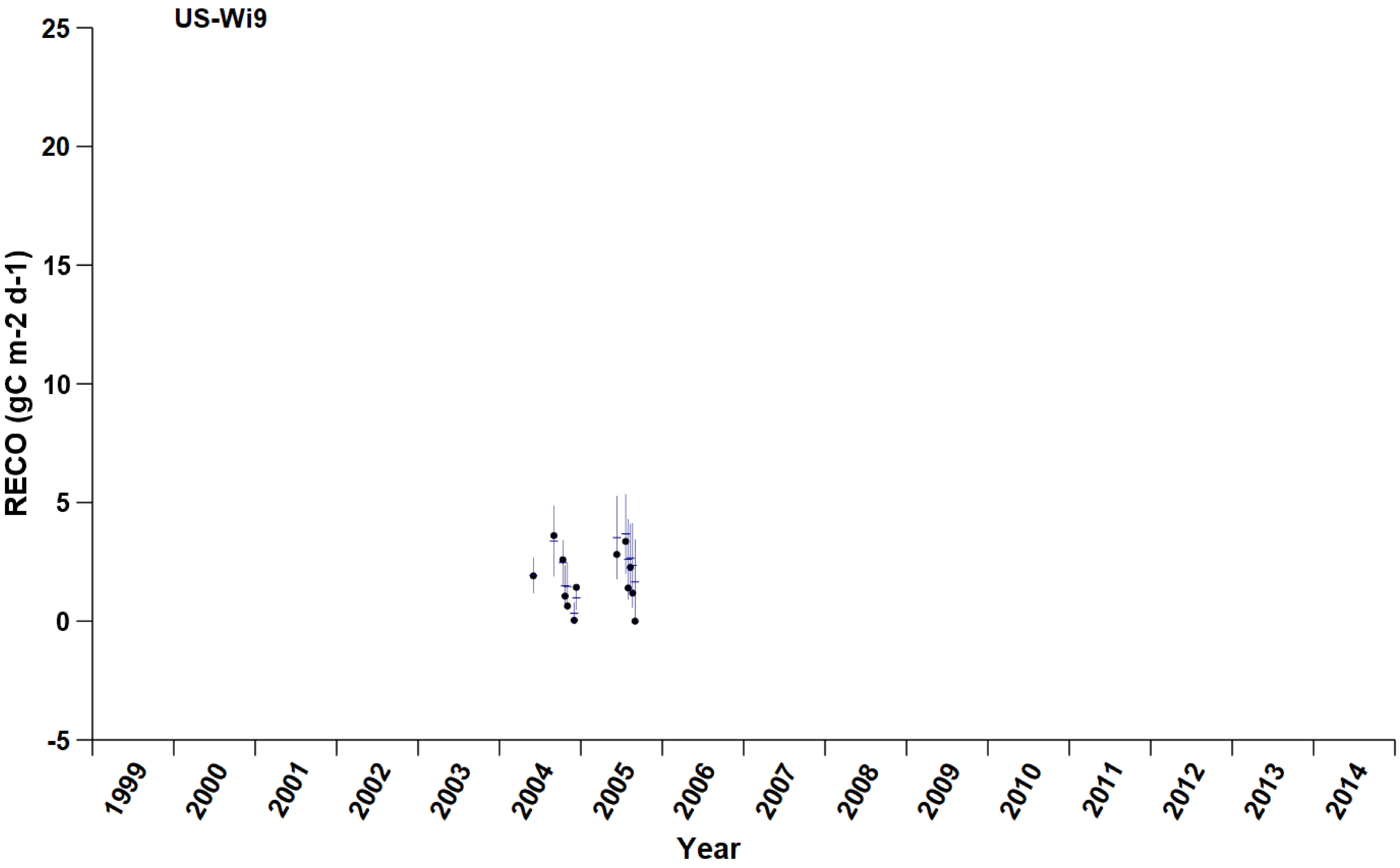

US-Wi9
RECO (gC m-2 d-1)
25
20
15
10
5
0
-5
1999
2000
2001
2002
2003
2004
2005
2006
2007
2008
2009
2010
2011
2012
2013
2014
Year

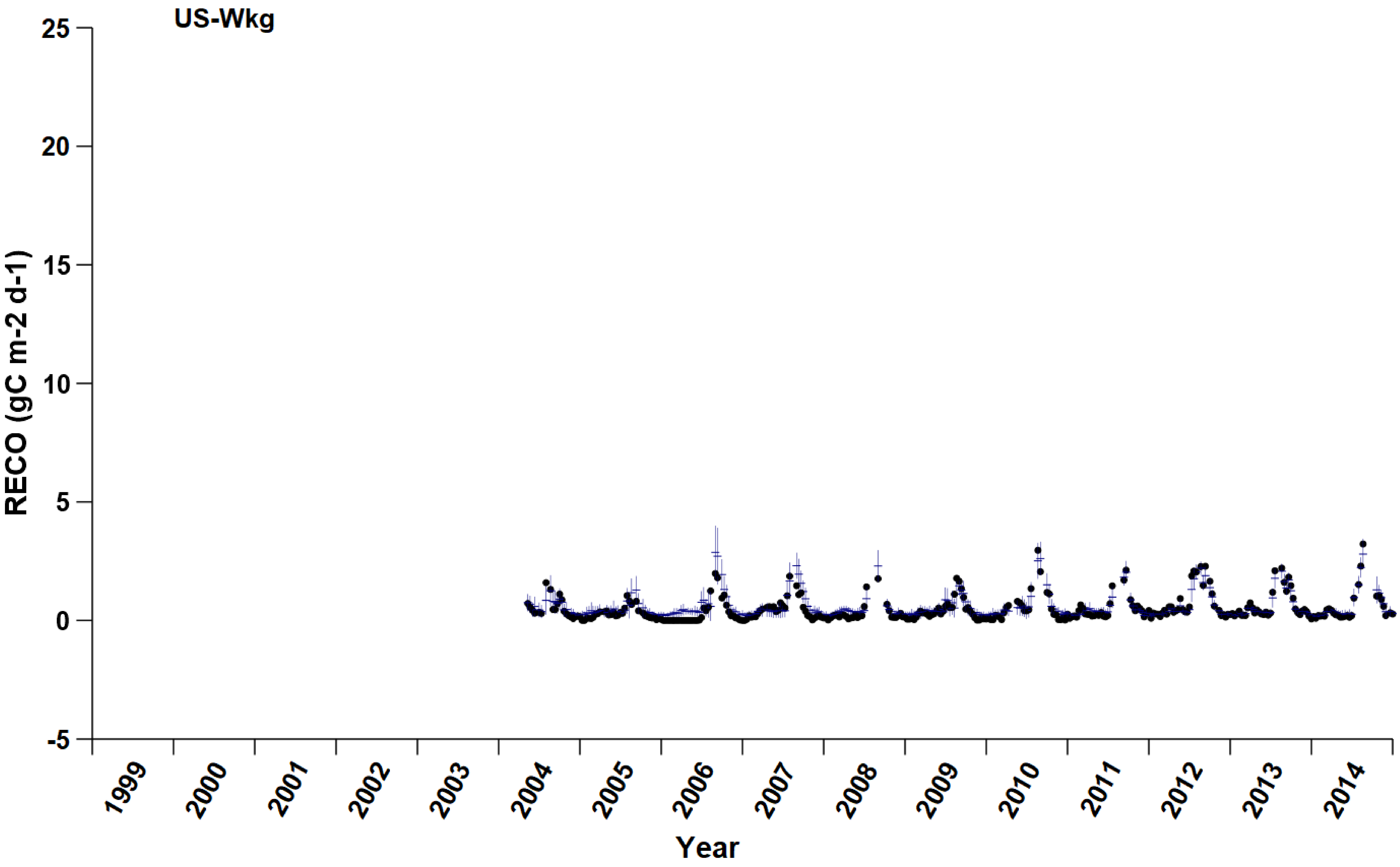
US-Wkg
25
20
15
10
5
0
-5
RECO (gC m-2 d-1)
1999
2000
2001
2002
2003
2004
2005
2006
2007
2008
2009
2010
2011
2012
2013
2014
Year

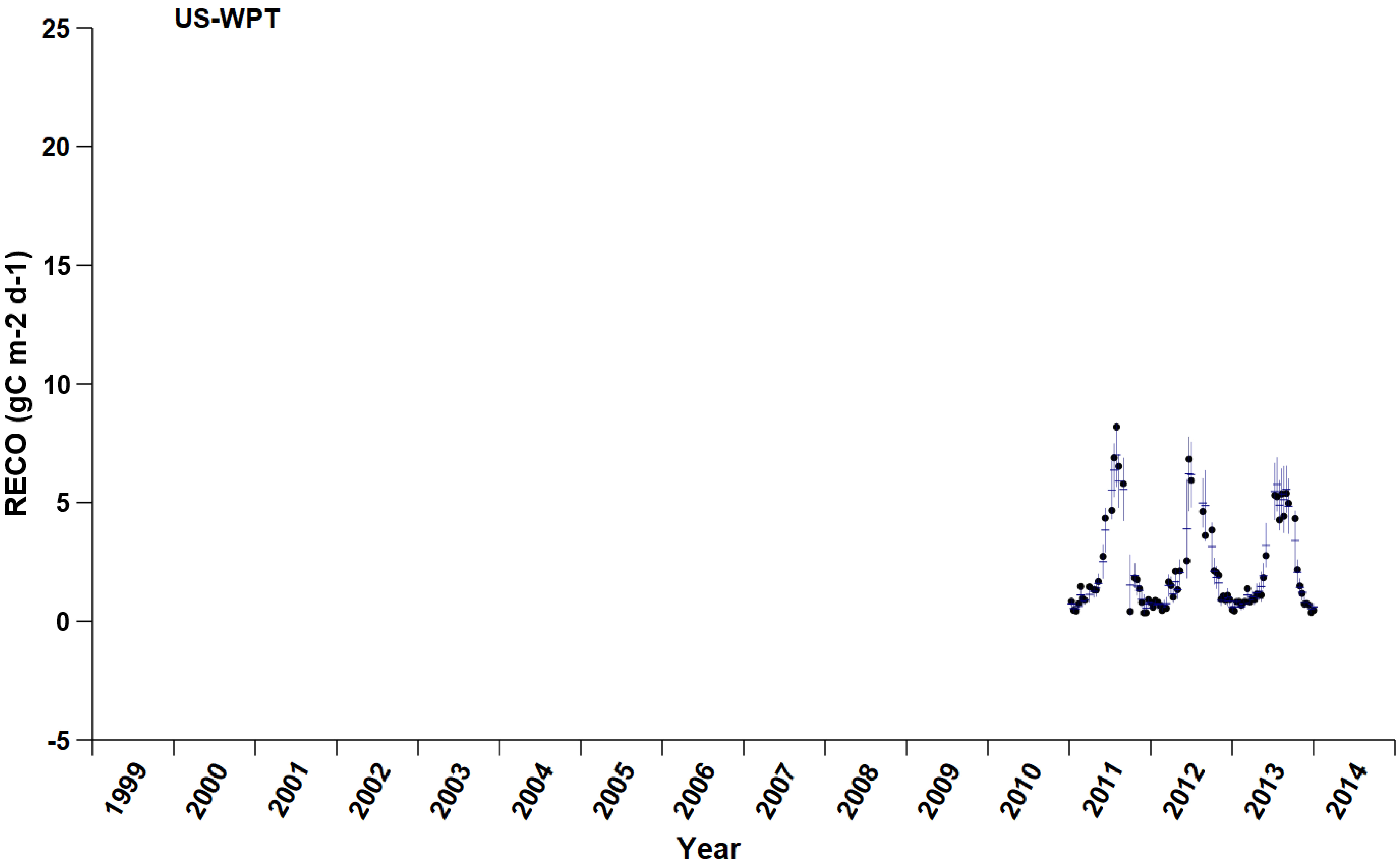
US-WPT
RECO (gC m-2 d-1)
25
20
15
10
5
0
-5
1999
2000
2001
2002
2003
2004
2005
2006
2007
2008
2009
2010
2011
2012
2013
2014
Year

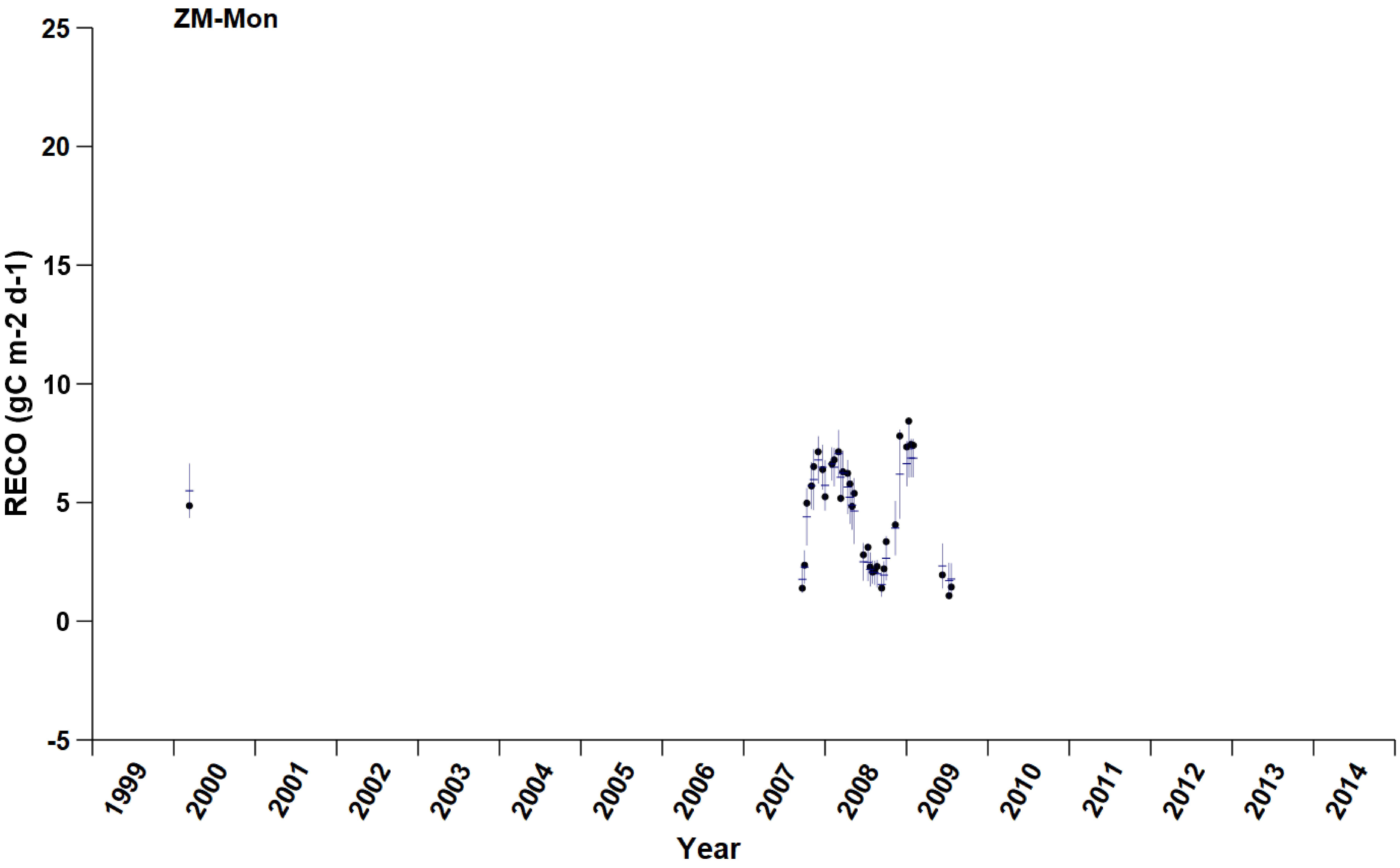
ZM-Mon
RECO (gC m-2 d-1)
25
20
15
10
5
0
-5
1999
2000
2001
2002
2003
2004
2005
2006
2007
2008
2009
2010
2011
2012
2013
2014
Year
